# Participatory and multi-disciplinary science dataset and surveys for the assessment of the microbiological and behavioural factors influencing fresh fruits and vegetables' waste at home

**DOI:** 10.1016/j.dib.2025.112434

**Published:** 2026-01-07

**Authors:** Camille Marchal, Damien Ballan, Sarra Azib, Morgane Innocent, Bertrand Urien, Annick Tamaro, Marine Le Gall-Ely, Emmanuel Coton, Adeline Picot, Jérôme Mounier, Louis Coroller, Patrick Gabriel

**Affiliations:** a Univ Brest, INRAE, Laboratoire Universitaire de Biodiversité et Ecologie Microbienne, F-29280 Plouzané, France; b Univ Brest, Laboratoire d’Economie et de Gestion de l’Ouest, 20 *Av*. Victor le Gorgeu, Brest, 29200, France

**Keywords:** Food waste, Food spoilage, Storage, Consumer behaviour, Microbiology, Citizen science

## Abstract

Fresh fruits and vegetables (FFV) represent the largest part of food waste at the consumer level. This waste directly results from FFV physiological and microbiological spoilage, itself intricately linked to behavioural factors such as consumer practices, including purchase, storage and hygiene practices, but also consumers’ perceptions towards spoilage. Based on a dual approach combining microbiological and behavioural sciences, we examined the link between FFV waste produced by 49 volunteering French households, measured using connected bins, the microbial ecology of their storage compartments, using culture-dependent and -independent approaches, and their consumer behaviour, cleaning and storage practices, through in-depth interviews and a dedicated survey. An exploratory qualitative survey carried out on 17 individuals followed by two quantitative data collections on 1048 and 815 representative French consumers enabled us to identify anti-FFV waste practices and to cluster consumers according to their anti-FFV waste behaviours. Spoilage dynamics of commonly consumed FFV, according to storage temperature, microbial contamination level and the presence or absence of surface wounds, were also performed in controlled conditions. This citizen-science-based dataset covers a wide array of microbiological and behavioural factors related to domestic FFV waste, as well as real measurements of waste volumes thanks to the innovative use of connected bins. Altogether, this data could provide interesting insights into more effective and accessible guidelines for FFV waste reduction at the consumer level, and thus to a potential reduction of global food waste and its related costs.

Specifications Table

**Table:** 

Subject	Biology & Social Sciences
Specific subject area	Microbiology and Behavioural Sciences *Impact of consumer behaviour, storage and cleaning practices toward fresh fruit and vegetables (FFV), and microbial contamination of their storage areas on FFV waste at the household level.* *Identification of anti-FFV waste practices and clustering of consumers according to anti-FFV waste behaviours.* *Spoilage dynamics of commonly consumed FFV.*
Type of data	Tables, Interview guides, Interviews transcriptions, Questionnaires, Filtered Sequencing Data
Data collection	49 volunteer households were recruited through advertisements in local newspapers and social media (Facebook and LinkedIn) and were provided connected waste bins (https://wikifactory.com/+uboopenfactory/foodrest) to weigh their FFV waste. The of FFV storage compartments’ microbial surface contamination was sampled by wet swabbing and assessed by culture-dependent and culture-independent approaches (DNA extraction using a NucleoSpin Kit for soil; ITS2 and 16S rRNA metabarcoding through Illumina MiSeq PE300 sequencing performed at Génome Québec Innovation Centre (MacGill University, Montreal, Canada)). ASVs were taxonomically assigned to the SILVA V138.1 and Unite (Full UNITE+INSD dataset) databases for the 16S and ITS tables, respectively. Sensor devices (Thermo Hygro probes -Proges Plus, Lille, France-) were used to monitor both the temperature and relative humidity of refrigerators. Household’s practices towards spoilage was established through in-depth semi-structured interviews (interview guides based on Gavard-Perret et al., 2018) and a dedicated consumer habits survey, allowing to identify 43 common anti-waste practices. These items were then integrated into two online questionnaires auto-administrated by 1048 and 815 French representative consumers belonging to a Panel firm in June 2023 and August 2024. In the second survey, respondent’s FFV waste was estimated through three scales (Ananda et al., 2023; Vischers et al., 2016; Stefan et al., 2013) FFV spoilage of dynamics was studied in controlled conditions on a selection of four commonly consumed FFV over 28 days.
Data source location	Households participating in qualitative study were selected in the Brest metropolitan area (France, latitude: 48.400002, longitude: −4.48333) Consumers participating in quantitative study (survey 1 & 2) were located in the French metropolitan area. Laboratoire Universitaire de Biodiversité et d'Écologie Microbienne & Laboratoire d’Économie et de Gestion de l’Ouest, Université de Bretagne Occidentale, Brest, France
Data accessibility	Repository name: Recherche Data Gouv - Espace Générique Data identification number: https://doi.org/10.57745/WRDJ3N Direct URL to data: https://entrepot.recherche.data.gouv.fr/dataset.xhtml?persistentId=doi:10.57745/WRDJ3N Instructions for accessing these data: All files described in this paper are downloadable freely from the repository.
Related research article	[1] Ballan D., Picot A., Rolland N., Bovo C., Prévost C., Coton E., Mounier J., 2025. Diversity of spoilage microorganisms associated with fresh fruits and vegetables in French households. International Journal of Food Microbiology, 437, 111204. https://doi.org/10.1016/j.ijfoodmicro.2025.111204

## 1 Value of the Data

• Half of food waste in Europe occurs at the consumer level, the majority of which consists of FFV. Our citizen-science-based dataset constitutes the base for a global analysis of the impact of both microbiological and behavioural factors on FFV waste at the household level. It focuses on the volume of fresh fruits and vegetables of all types that French households would have otherwise consumed but decided to discard for any reason.
• The innovative use of connected bins provides real measurements of household FFV waste, contrary to most studies based on self-reported measurements.
• Culture-dependent and -independent analysis of surface microbial contamination provide a detailed description of the microbial ecology of different storage types in participating households.
• The in-depth interviews and dedicated survey cover household behaviour throughout all the steps related to FFV consumption : purchasing habits, storage and hygiene practices for both refrigerated and unrefrigerated storage types, cooking and reusing altered FFV, and stance towards waste and spoiled products. These items were surveyed for i) the 49 selected households, allowing for the establishment of links between FFV waste and in situ storage surface contamination, and ii) in two large scale (*n* = 1 048 and *n* = 815) quantitative studies covering the whole French population.
• The role of supposedly key factors such as storage temperature, initial contamination level, product type and initial physiological damage, on spoilage was obtained in controlled conditions to experimentally measure their actual impact on FFV spoilage.
• This dataset is a foundation towards proposing key recommendations to fight food waste at the consumer level, an often overlooked link of the food production chain. It explores the many potential factors impacting FFV waste at home while offering specific protocols and insights for the implementation of sampling campaigns at the household level, allowing for a deeper understanding of household FFV waste both in France and across different countries. The quantitative measurement of FFV waste volumes allowed for the development of an exploratory PLS model, with the potential for predictive spoilage models based on several campaigns.

## 2 Background

Adopted in September 2015 by the EU, the Sustainable Development Goal “12.3″ targets to halve the per capita food waste at the retail and consumer level by 2030. Households account for 53 % of food waste in Europe, more than any other actor in the food chain. Fresh fruit and vegetables (FFV) are known to constitute the higher volumes of wasted food mainly associated with microbial contamination and spoilage. The FOODREST project was financed by the French National Research Agency (ANR-20-CE21–0006). Its goal was to better understand the microbiological and behavioural aspects of FFV waste at the household level, and to propose efficient recommendations acceptable by households to reduce FFV waste. The FOODREST project aimed at i) studying microbiota associated with FFV storage facilities at home as well as identifying the predominant microorganisms and factors responsible for FFV spoilage, ii) studying consumer perceptions and practices towards spoiled products and food waste, and iii) producing a set of efficient recommendations appropriate to consumer practices by evaluating the efficacy and acceptability of hygiene recommendations aiming at reducing food waste while guaranteeing food safety and by providing a best practices handbook and risk model for better food waste management at the consumer level.

## 3 Data Description

The files associated with this data-in-brief article are divided among three main areas of study: (i) the study of FFV waste volumes through two sampling campaigns among 49 volunteering French households, (ii) a quantitative study of the consumer anti-waste practices towards FFV based on two online questionnaires (1048 and 815 people respectively) among consumers belonging to panels representative of the French population and (iii) spoilage dynamic assays performed at the laboratory scale to determine the impact of the temperature, the presence of surface wounds and level of contamination of the storage area on FFV spoilage over a 28 days period.

Tables 1 through 8 as well as Doc. 1a & 1b and Doc. 2 encompass the data obtained through the FFV waste sampling campaigns among the 49 selected households from Brittany, France :

*Table 1 : Nomenclature (.xlsx table)*

Household number and nomenclature for each storage compartment, period (summer = P1 and autumn = P2) and visit (V1 and V3) among each period. The resulting “Sample_ID” details, in the following order, the household number, visit, period and storage compartment (unrefrigerated : fruit basket = *C*; unrefrigerated : others (e.g. shelf) = *A*; refrigerated = *R*; others (e.g. garage, cellar) = *M*) of each sample and was used in all tables related to these sampling campaigns. In cases where a single household used two different storage facilities at either room or refrigerated temperature (e. g. two different fruit baskets or refrigerators), a number was added at the end of the sample ID to distinguish them.

*Table 2 : Waste in gram per day per household (.xlsx table)*

Raw values for FFV waste measured for each household using the connected bins. The value is displayed in grams per day per household for the entire period of each campaign (i.e. in summer and autumn). A single bin was provided to each household for each period, resulting in a single waste value per household for all visits regardless of the storage type(s) used for FFV. Missing values are due to households leaving the study between the first and second campaign (*n* = 49 in summer and *n* = 37 in autumn).

*Table 3a & 3b : (a) Households socio-demographic descriptors (.xlsx table) (b) Socio-demographic descriptors of the subsample of households used for the exploratory qualitative study (.xlsx table)*

(a) Raw data detailing the socio-demographic parameters of each household, including household composition, family structure, number of children, area of residence and type of accommodation, as well as an auto-estimation of their overall FFV consumption. Variables concerning habitants age range, gender, working status and socioprofessional category are specific to the person in each household that was in direct contact with the research team during the study. (b) Details of the socio-demographic parameters of the subsample of 17 households used for the exploratory qualitative study analysis.

*Doc. 1a & 1b : (a) Details of interview guide 1 content (.docx guide) (b) Details of interview guide 2 content (.docx guide)*

(a) First interview guide, composed of open-ended questions asked to stimulate the discussion with participants concerning four themes : (1) “Value of fruits and vegetables” : a guessing game, encouraging participants to describe a particular FFV consumption experience, and thus the perceived value of FFV (2) “Fruits and vegetable purchasing, storage and consumption practices”, (3) “From edible to inedible” : FFV spoilage perceptions and emotional reactions to spoilage, and (4) “Food waste representations”. (b) Second interview guide, explores consumer reactions and behaviors towards product deterioration, through open-ended questions representing three themes : (1) “Representations of supply locations” (e.g. supermarket, organic food shops), (2) “The edibility continuum” : a discussion about spoilage perceptions and (3) “The genesis of fruits & vegetables”, including questions about reasons behind throwing away some products using pictures of the participants own spoiled FFV.

*Doc. 2 : Transcripts of household interviews 1 & 2 (.docx transcripts)*

Exhaustive anonymised transcripts of the 34 interviews performed with households using the interviews guides (*Doc 1a & 1b*) detailing their habits and practices towards FFV storing and preservation. Two interviews following interview guides 1 & 2 were performed for each of the 17 households listed in the subsample table (Table 3b).

*Table 4a & 4b : (a) Household survey questions (.docx questionnaire) and (b) answers (.xlsx table)*

(a) Overview of survey questions and answer options concerning household’s FFV storage and hygiene practices, translated to English (the original questionnaire was in French). (b) Curated table detailing the answers of each household, when applicable. For questions “Ref 13″, “Ref 15″, “Unref 5″ and “Unref 7″, the original answers corresponded to lists of items, and have been coded as binary “yes/no” variables, with one column per item. Empty cells for these variables correspond to “no”. Concerning questions requiring households to provide percentage estimations, answers were removed if aberrant values were provided (i.e. the total percentage for one question item was either higher than 130 % or lower than 70 %). Answers to the “Storage practices” section of the questionnaire can be found in the eponymous tab. A key tab detailing the variables can be found in the table and their modalities are provided.

*Table 5 : Refrigerator descriptors (.xlsx table)*

Raw values of minimum, maximum, mean and median temperatures and relative humidity measured inside each participating households’ refrigerator throughout each period. The data from sensors was gathered at the end of each sampling campaign, leading to a single value for all visits. Missing values are due to households leaving the study between the first and second campaign (*n* = 49 in summer and *n* = 37 in autumn).

*Table 6 : Clean scores (.xlsx table)*

Raw clean score values for each storage compartment established through visual inspection based on various factors. These scores were estimated on a scale of 1 to 3, 1 being the cleanest and 3 the dirtiest, during visits 1 & 3 of each campaign. The “cleanliness” variable aggregates clean scores using a matrix system (Clean = 1 to 3; Medium = 4 or 6; Dirty = 9). A tab detailing the matrix system is provided.

*Table 7 : Microbial counts (.xlsx table)*

Raw surface microbial counts in Log_10_ CFU or TFU (colony or thallus forming units) per cm² resulting from the culture-dependent analysis of household FFV storage spaces with various culture media. The suffix “_P” refers to counts after growth at 8 °C allowing to retrieve psychrotrotropic/psychrophilic microorganisms, while suffix “_M” refers to counts after growth at 25 to 37 °C, allowing to retrieve mesophilic microorganisms. Measurement of airborne fungal spores performed at the second visit of each sampling campaign using a bio-impactor placed near the fruit basket, and incubated at 25 °C before enumeration.

*Table 8a & 8b : (a) ITS and (b) 16S Metabar contingency table (.biom tables)*

Filtered amplicon sequence variant (ASV) tables obtained by metabarcoding on DNA extracted from the surface microbial communities of FFV storage compartments. ASVs were taxonomically assigned to the SILVA V138.1 and Unite (Full UNITE+INSD dataset, version 21/04/2024) databases for the 16S and ITS tables, respectively.

Tables 9a, 9b, 10a and 10b correspond to online questionnaires and answers of samples of 1048 (Table 9) and 815 people (Table 10), drawn from consumer panels representative of the French population (in terms of age, gender, socio-professional category, location and education level) using the quotas method to items concerning consumer FFV anti-waste practices at home. Tables 9c and 10c details the socio-demographics descriptors for each consumer panel. The anti-waste practices items were selected through an exploratory qualitative study as well as a review of both the academic literature and institutional reports.

Survey 1 aimed to quantify these practices to select the most frequently used as well as the most recommended by ADEME [2] and ANSES (French agency for food environmental and occupational health & safety). After their selection, Survey 2 aimed to cluster consumers according to their anti-waste practices. Auxiliary variables were also included, namely individual and global concern to food waste, environmental concern, access to a composter or vegetable garden, number of pets, and finally socio-demographic characteristics : gender, age, education level, professional status, socio-professional category, geographical location, family structure, household size and number of children.

*Table 9a, 9b & 9c : (a) Survey 1 questions (.docx questionnaire), (b) answers (.xlsx table) and (c) respondent’s socio-demographics descriptors (.xlsx table).*

(a) Overview of survey 1 questions and their corresponding answer options concerning household’s FFV storing and cleaning practices as well as their perceived mastery of good practices concerning FFV preservation, storage and storage cleaning. This questionnaire was translated to English as the original questionnaire was in French. (b) Table detailing the raw answers of each respondent, when applicable. (c) Table detailing the overall composition of the consumer panel’s socio-demographics descriptors (gender, age range, geographical location, Socio-professional category)

Items were numbered from 1 to 156 and given an abbreviated name based on the original question. Items were evaluated by respondents through various scales. The 5-point Likert scale according to the degree of agreement (``Strongly disagree'' to ``Strongly agree''), was used concerning refrigerated and unrefrigerated storage cleaning routines, mastery of FFV storage and cleaning practices, general knowledge, perception and practices regarding fresh or damaged FFV health benefits, cooking and consumption and finally perception of food waste. Scales of frequency were used, with either a “Never” to “Always” scale, with a possible “Not concerned” option, regarding FFV packaging and storing practices depending on FFV type and current condition, FFV preparation and cooking habits, and practices toward damaged FFV valorisation, or a scale ranging from “About once a week” to “About once or twice a year”, concerning refrigerated and unrefrigerated storage cleaning frequency. Some items were “yes/no” questions, notably regarding disinfection and drying of cleaning FFV storage, or lists of most used cleaning or disinfecting products. A small quiz was included to test respondents' general knowledge concerning FFV preservation in the form of seven multiple-choice questions. All answers were mandatory, although a “Not applicable” option was provided when relevant.

*Table 10a, 10b & 10c : (a) Survey 2 questions (.docx questionnaire), (b) answers (.xlsx table) and (c) respondent’s socio-demographics descriptors (.xlsx table).*

(a) Overview of survey 2 questions and their corresponding answer options concerning the 19 items selected after survey 1 regarding household’s FFV consumption (purchase, storage and cooking practices), hygiene practices, and sharing of FFV surplus. FFV waste was estimated through three scales measuring self-reported waste quantities and/or frequencies [[3], [4], [5]]. This questionnaire was translated to English as the original questionnaire was in French. (b) Curated table detailing the re-coded answers of each respondent, when applicable. (c) Table detailing the overall composition of the consumer panel’s socio-demographics descriptors (gender, age range, geographical location, Socio-professional category)

Question themes were numbered from Q1 to Q45, each divided into several numbered questions. The answer table contains the answers to the questions from themes Q1 to Q5 regarding FFV purchase, storing and conservation practices, cleaning of storage areas, consumption practices and waste prevention practices, with names re-coded as abbreviations based on the original question. Q6 to Q9 concerned the evaluation of FFV waste amount through the first scale [4], Q18 and Q19 evaluated FFV waste amounts through the second scales [3], and Q21 to Q25 allowed to evaluate FFV waste amounts through the third scales [5] and to obtain examples of FFV purchased and discarded by consumers. Q21 filtered consumers that had consumed FFV in the last week for Q18 to Q25. A qualifying question (Q1, re-coded as “Qfilter/disqualifier”) was integrated at the beginning of the questionnaire to filter out respondents that did not consume FFV and thus were not relevant to our study. Q2 (re-coded as Qfilter2) was used to evaluate the interest of consumers for FFV.

Answers to themes Q10 to Q17 (perception and valorisation of damaged FFV, perception of damaged FFV impact on health) and Q26 to Q32 (environmental concerns, opinions and habits concerning food consumption and waste, feeling of disgust regarding consumption of damaged FFV) were not included in the answer table. Q33 to Q45 provided details on the socio-demographic profile of respondents.

Items were evaluated by respondents through various scales. The 5-point Likert scales according to the degree of agreement (``Strongly disagree'' to ``Strongly agree'' or “Not at all” to “Extremely”) was used, notably for question Q2. A scale of frequency (“Never” to “Always”) was used for themes Q3 to Q5. A scale of level of disgust (“Not disgusting at all” to “Extremely disgusting”), “yes/no” questions and lists of features to pick from or to orders from most to least important to the respondent were also used. A qualifying question was integrated at the beginning of the questionnaire to filter non consumers of FFV who were not relevant to our study. All responses were mandatory, although a “Not applicable” option was provided when relevant. A key tab detailing the studied variables is provided.

Finally, Table 11 concerns the study of FFV spoilage dynamics in controlled conditions :

*Table 11 : Spoilage dynamic assays (.xlsx table)*

Table providing raw data for the initial weight and progress of weight loss due to spoilage of four FFV (apple, tomato, lemon and carrot) in controlled environments during a four weeks period. Contamination level, storage temperature and presence/absence of surface wounds on the FFV is specified for each of the 24 boxes (A to L, two replicates each). For a given condition, each line corresponds to an observation, with its sampling day. For each observation, the counts of altered or spoiled FFV, and the associated total and relative weight loss in grams were recorded, separated or grouped by box replicate. Area Under Disease Progression Curves (AUDPC) and their square roots are provided for the number of altered or spoiled FFV, and for relative weight loss, separated or grouped by box replicate. A key tab is provided detailing the variables.

All tables and files are available in the linked repository. The name and file type of the corresponding repository file for each of the tables and documents described above are detailed in Table 1.

**Table 1:** Overview of the tables provided for each area of study with their file type and content.

Area of study	Table / document name	File type	Corresponding respository file name	Content description
**FFV waste sampling campaigns**	Table 1 : Nomenclature	xlsx	Table1_Nomenclature	Household sample ID and detailed nomenclature for each period and visit.
	Table 2 : Waste in gram per day per household	xlsx	Table2_Waste_in_g_day_household	Average FFV waste in gram per day per household for each period.
	Table 3a : Households socio-demographic descriptors	xlsx	Table3a_Households_Sociodemographic_ descriptors	Household composition, living conditions and respondent's main socio-demographic descriptors.
	Table 3b : Socio-demographic descriptors of the subsample of households used for the exploratory qualitative study	xlsx	Table3b_Households_Sociodemographic_ descriptors_subsample	Household sub-sample composition, living conditions and respondent's main socio-demographic descriptors.
	Doc. 1a : Details of interview guide 1 content	docx	Doc1a_Interview_Guide_1	Open-ended interview guide exploring household respondent's perceived value of fruits and vegetables, their FFV purchasing, storage and consumption practices, their FFV spoilage perceptions and their food waste representations.
	Doc. 1b : Details of interview guide 2 content	docx	Doc1b_Interview_Guide_2	Open-ended interview guide exploring household respondent's representations of supply locations, their spoilage perceptions and their reasoning behind the FFV they discarded.
	Doc. 2 : Transcripts of household interviews 1 & 2	docx	Doc2_Households_anonymised_interview_ transcripts	Exhaustive anonymised transcripts of the 34 interviews performed on the sub-sample of 17 household respondent using the interviews guides 1 & 2.
	Table 4a : Household survey questions	docx	Table4a_Household_survey_questions	Translation of the questionnaire presented to households concerning their FFV purchasing, storage and hygiene practices.
	Table 4b : Household survey answers	xlsx	Table4b_Household_Survey_answers	Households questionnaire answers concerning : refrigerator usage and cleanliness; storage areas cleaning frequency, cleaning rinsing and drying practices and products used; FFV packaging practices; percentages of FFV purchased from different store type, sourcing and production type.
	Table 5 : Refrigerator descriptors	xlsx	Table5_Refrigerator_descriptors	Mean, median, minimum and maximum sensor temperature and relative humidity readings for each period.
	Table 6 : Clean scores	xlsx	Table6_Clean_scores	Visual clean score for each period and clean score calculation matrix.
	Table 7 : Microbial counts	xlsx	Table7_Microbial_counts	Culture-dependent storage surface microbial counts for mesophylic and psychrophylic TVC, *Pseudomonas* spp., LAB and enterobacteria, and mesophylic spore forming bacteria and fungi
	Table 8a : ITS Metabar contingency table	biom	Table8a_ASV_ITS_filtered	Filtered ASV tables from the ITS primer metabarcoding amplification of DNA extracted from storage surface microbial samples, taxonomically assigned to the Unite dataset
	Table 8b : 16S Metabar contingency table	biom	Table8b_ASV_16S_filtered	Filtered ASV tables from the 16S primer metabarcoding amplification of DNA extracted from storage surface microbial samples, taxonomically assigned to the SILVA dataset
**Quantitative study on consumer anti-waste practices towards FFV**	Tables 9a : Survey 1 questions	docx	Table9a_Survey1_Questionnaire	Detailed questions and answers for survey 1 regarding FFV preservation and packaging strategies, refrigerated and unrefrigerated storage practices, FFV storage cleaning and disinfection frequency, products and practices, FFV preparation and cooking skills, perception of fresh and damaged FFV and strategies to avoid FFV waste
	Tables 9b : Survey 1 answers	xlsx	Table9b_Survey1_answers	
	Tables 9c : Survey 1 respondent’s socio-demographics descriptors	xlsx	Table9c_Survey1_Sociodemographics_ descriptors	Percentage of the consumer panel of survey 1 belonging to each gender, age range, geographical location and socio-professional category.
	Tables 10a : Survey 2 questions	docx	Table10a_Survey2 _Questionnaire	Detailed questions and answers for survey 2 regarding FFV purchase planning and frequency, FFV storing and conservation practices, cleaning of refrigerated storage areas, FFV consumption and sharing practices and FFV waste prevention practices.
	Tables 10b : Survey 2 answers	xlsx	Table10b_Survey2_answers	
	Tables 10c : Survey 2 respondent’s socio-demographics descriptors	xlsx	Table10c_Survey2_Sociodemographics_ descriptors	Percentage of the consumer panel of survey 2 belonging to each gender, age range, geographical location and socio-professional category.
**Spoilage dynamic** **assays**	Table 11 : Spoilage dynamic assays	xlsx	Table11_Spoilage_dynamic_assays	Initial weights and progress of weight losses due to spoilage as well as the Area Under Disease Progression Curves and the number of altered and spoiled FFV lost regarding four FFV (apple, tomato, lemon and carrot) in controlled environments over 28 days.

## 4 Experimental Design, Materials and Methods

### 4.1 Fresh fruit and vegetable waste sampling campaigns

#### 4.1.1 Consumer households and sampling campaigns

Participation of households living in the Brest metropolitan area (Brittany, France) was solicited through advertisements in local newspapers and social media groups (Facebook and LinkedIn) to recruit households willing to commit to an eight-month study, with interested households completing an online registration form. Forty-nine volunteer households that regularly consumed FFV were then selected to qualitatively represent the French population as closely as possible. The final selected panel was fairly heterogeneous in terms of socio-demographic profiles, including age, household composition, number of children, working status, socio-occupational category, and residential area (urban, suburban or rural). All Participants were required to provide full informed consent to take part in the study and to allow the use of their data for academic research purposes.

Two sampling campaigns were performed during the year 2021, spanning from May to July 2021 and from September to December 2021 (referred to as “Summer” and “Autumn” campaigns). During each campaign, each household was visited four times, about two weeks apart. The first visit, at the start of each campaign, allowed to install the connected bins and provide instructions on their use and on the campaign’s protocols. Microbiological swabbing and a visual inspection of FFV storage compartments were performed on the second and fourth visit, and indoor air fungal spore concentrations were sampled during the fourth visit. Temperature and relative humidity (RH) sensors (Thermo Hygro probes -Proges Plus, Lille, France-) were also installed in refrigerator drawers on the second visit, and removed on the fourth. A semi-structured qualitative interview was conducted with each household representative during the second visit to explore their perceptions and behaviours regarding FFV food waste. Finally, consumers were asked to answer a dedicated survey on their habits on the fourth visit of the second campaign.

#### 4.1.2 Spoilage measure

Households were provided with a connected bin device specifically designed for this study and calibrated using standard weights (a detailed description of the device is available at [6]) which was used to specifically weigh any “normally edible” FFV that were discarded. Households were given the instruction to only use it to throw away “FFV, whole or partial, that they would have usually eaten but decided not to” for any reason, to make sure that people who got rid of consumable parts of the products (apple peels for instance) did not interfere in the measures. Connected bins were retrieved at the end of the experiment, allowing to monitor, with automatic data recording, the weight of FFV wasted by each household during a 2 month period during both seasons.

#### 4.1.3 Qualitative data collection and subsample selection

Individual interviews are a relevant method of data collection for exploring complex processes such as decision-making or evaluation mechanisms, but also for understanding intimate or even taboo subjects such as religion or sexuality. They also make it possible to detect any individual differences between participants [7]. This method therefore aims to gain an in-depth understanding of the interviewees' experiences and behaviours. Given that this qualitative study, in both its phases, seeks to explore and understand behaviours related to food waste, semi-structured interviews were chosen for the data collection. They are based on an interview guide that facilitates the exchange and puts the respondent at ease without restricting their freedom or imposing a specific order of questions [7]. They are particularly well suited to behavioural studies and are widely used in management sciences [8].

Thus, semi-structured interviews were conducted with each household representative during the second visit of each campaign. Distinct interview guides were used for each phase. The first phase aimed to explore FFV management behaviours, from purchase to disposal and their link with household food waste. It also aimed to identify factors that could influence these behaviours. The second phase, which built on the first, focused more specifically on the final phase of the food life cycle: disposal. Its objective was to better understand consumers' decisions when FFV showed signs of physical deterioration. This phase sheds light on the perceptions and emotional or rational justifications associated with the decision to throw away a spoiled product or not. It also served to clarify what the end of life of fruit and vegetables means for the interviewees.

All interviews were conducted in the respondents' homes, face-to-face. This in situ approach is particularly relevant in the context of a study on domestic behaviours related to food waste, as it allows practices to be placed in their real context and provides access to details that are often missing in interviews conducted in a neutral location. In addition, the interviews were conducted in a calm setting conducive to discussion, which facilitated fluid exchanges between the researcher and the respondent. The interviews lasted an average of 65 min in the summer campaign and 60 min in autumn campaign. All sessions were recorded and subsequently transcribed for analysis.

From the 49 participants, a subsample of seventeen individuals was selected for the analysis, resulting in a corpus of 34 interviews (for 17 participants and one interview per phase). The selection of the subsample was based on semantic saturation criteria [9] which consists in stopping qualitative data analysis when no new information emerges. In our case, this criteria was reached at the fifteenth pair of interviews, suggesting that additional data would not further enrich the findings. We nevertheless added two additional pairs of interviews to make sure semantic criteria was effectively reached.

#### 4.1.4 Consumer habit survey

In the autumn phase, the 49 participating households were asked to answer a survey consisting of a total of 40 questions grouped into five items : use of domestic refrigerator for FFV storage and associated hygiene practices, use of fruit baskets for FFV storage and associated hygiene practices, proportion of FFV originated from organic versus conventional agriculture, purchase habits (purchase frequency and locations) and preferred storage compartment according to different types of FFV. Before the survey, the questions measuring household practices were submitted to five marketing researchers, who were asked to evaluate their suitability. When an item was judged unclear or inappropriate, experts were invited to suggest alternative formulations. This step enabled the refinement of the items and ensured that the targeted practices were accurately measured. After this step, a pre-test of the questionnaire was conducted with 10 individuals from our network to ensure that all questions were clearly understood and interpreted as intended.

#### 4.1.5 *Temperature and* RH *monitoring in FFV storage compartment of household refrigerators*

Volunteers were also provided a sensor device (Thermo Hygro probes -Proges Plus, Lille, France-), calibrated by the manufacturer, to monitor both the temperature and RH of the dedicated FFV compartment in their refrigerator. Sensors were set to record temperature and RH once every 2 h, and were retrieved at the end of each sampling campaign.

#### 4.1.6 Visual inspection of FFV storage compartments

FFV storage compartments mainly consisted of both fruit basket and refrigerator compartments (*n* = 35) although a few households did not use either a refrigerator (*n* = 11) or a fruit basket (*n* = 3) to store FFV. Prior to microbiological sampling, a visual inspection of storage compartments was performed to evaluate their cleanliness. They were graded as either “clean” (absence of any visible FFV debris, dirt, liquid or stains), “moderately dirty” (presence of visible FFV debris and dirt but to a limited extent) and “dirty” (high presence of visible FFV debris, dirt, liquid residues and/or organic stains) and converted into numerical values (“clean” =1, “moderately dirty” =2; “dirty” =3). Based on these observations, a “clean score” variable ranging from 1 to 9 was calculated, after multiplying the 2 successive visual evaluations for a given storage compartment and sampling campaign.

#### 4.1.7 Microbiological sampling of FFV storage compartments

Microbiological sampling of FFV storage surfaces was performed by wet swabbing. A sterile cotton swab was moistened by immersion in 4 ml of sterile neutralizing buffer (Liofilchem, Roseto degli Abruzzi, Italy). Excess moisture was removed by compression of the swab against the inner wall of the container and the swab was rubbed across the surface of three predefined zones of 25 cm², based on a template created for this study, to collect microorganisms associated with storage surfaces. Collected samples were kept at 4 °C and analysed within <24 h. A total of 361 swabbings were performed throughout this study.

#### 4.1.8 Culture-dependent microbiological analysis of storage compartment surfaces

After vortexing swab tubes for about 10 s, 1 mL of the suspension was collected and underwent serial dilutions from 10^−1^ to 10^−4^. Then, using a Spiral automatic plating device (Interscience, France), 100 µL of each dilution were directly plated on 5 different solid culture media : Plate Count Agar (PCA, Difco, France) to enumerate total viable bacteria communities, M2Lev (20 g/L malt extract, 3 g/L yeast extract, 15 g/L agar, 5 mg/L penicillin G, 5 mg/L streptomycin (Sigma-Aldrich, Germany)) medium to enumerate total fungal communities, Violet Red Bile Glucose agar (VRBG) to enumerate enterobacteria, Cetrimide Fucidin Cephaloridine (CFC + supplement) medium to enumerate *Pseudomonas* spp., Man Rogosa & Sharpe (MRS) medium to enumerate LAB communities. Additionally, 1 mL of the first dilution was collected before being heated in a 95 °C water bath for 5 min. This suspension was then diluted as previously described and plated on solid Brain Heart Infusion (BHI) medium in order to retrieve spore-forming bacteria. PCA, MRS and BHI cultures were incubated at 30 °C for 72 h, CFC at 25 °C for 48 h, VRBG at 37 °C for 24 h and M2LEV at 25 °C for 3 days, before enumeration. Additionally, for total bacterial count, enterobacteria, LAB and *Pseudomonas* cultures, incubation was also performed at 8 °C for 7 days to enumerate psychrophilic/psychrotolerant microorganisms. Each culture was performed in two replicates.

#### 4.1.9 Metagenetic community analysis of storage compartment surfaces

The rest of the suspension from the swab tubes was collected and centrifuged at 9000 g at 4 °C for 15 min before discarding the supernatant.

##### 4.1.9.1 DNA extraction

Obtained pellets underwent DNA extraction using the following protocol. Cells were resuspended using 400 µL of a lysis buffer (TrisHCl pH8 20 mM, EDTA 2 mM, Triton X-100 1.2 %, 20 mg/mL lysozyme, 5 U/µL mutanolysine) and put in a beaded tube, in which 10 µL of 20kU/mL lyticase and 10 µL of 1 mg/mL RNAse were added. Tubes were then incubated at 37 °C for 2 h for enzymatic reactions. Tubes were then placed in a bead mill for 90 Sectionx 3, with 1 min rest on ice between each cycle. The rest of the extraction was then performed using a NucleoSpin Kit for soil following the manufacturer’s instructions. DNA quality was controlled using a Nanodrop spectrophotometer and was stored at −20 °C.

##### 4.1.9.2 Illumina MiSeq sequencing

The 341F (5′-CCTACGGGNGGCWGCAG-3′) and 805R (5′-GACTACHVGGGTATCTAATCC-3′) primers were used to amplify the V3-V4 region of 16S rRNA for bacteria, while the ITS3 (5′-GCATCGATGAAGAACGCAGC-3′) and ITS4_KYO1R (5′-TCCTCCGCTTWTTGWTWTGC-3′) primers were used to amplify the internal transcribed spacer 2 (ITS2) region for fungi [[10], [11]]. Both amplicon library preparation and Illumina MiSeq PE300 sequencing were performed using FLD_ill adapter (forward sequence : 5′-ACACTCTTTCCCTACACGACGCTCTTCCGATCT-3′ and reverse sequence : 5′-GTGACTGGAGTTCAGACGTGTGCTCTTCCGATCT-3′) at the Génome Québec Innovation Centre (MacGill University, Montreal, Canada). PCR mix was prepared in a 8 µl final volume (1 x Qiagen buffer, 1.5 mM MgCl_2_, 5 % DMSO, 0.2 mM dNTPs mix, 0.6 µM of each primer, 0.01 U/µl Qiagen HotStarTaq, 8 pM of sample DNA).

##### 4.1.9.3 Metabarcoding data processing

The Illumina sequencing generated a total of 2.1GB of raw data (2 × 300 bp reads) for each studied taxonomic target. The resulting raw sequencing data is available on the NCBI Sequence Read Archive under BioProject accession number PRJNA1374171. Trimming parameters were selected using the FIGARO tool [12]. For 16S analysis, trimming was performed on the 289 and 219 positions (forward/reverse) with a max expected error of 3 and 2, respectively and a read retention percentage of 82.81 %. For ITS analysis, trimming was performed on the 296 and 214 positions (forward/reverse) with a max expected error of 6 and 6, respectively and a read retention percentage of 80.33 %. For 16S analysis, chimeras removal was done using the VSEARCH tool [13] by challenging the datasets with the gold_v20110519 database which led to the removal of 7268 sequences while 35,229 sequences were kept. For ITS analysis, VSEARCH was used by challenging the UCHIME reference dataset which led to the removal of 537 chimera sequences while 8221 sequences were kept. Taxonomic assignment was performed by using the SILVA V138.1 and the Unite (Full UNITE+INSD dataset) databases for the 16S for ITS analysis, respectively.

The obtained ASV tables were converted to .biom files and a filtration step was performed by using the “*filter_taxa_from_otu_table*” command from the Qiime package in order to remove assignments of chloroplasts, mitochondria and missing assignation from the datasets. ASVs with counts < 10 were removed, as well as ASVs present in only one sample. This filtration step led to the obtention of 5906 and 2226 ASVs for the 16S and ITS analysis, respectively. Microbial community analysis were performed using the Microbiome Analyst tools [14], filtering was done by removing constant features and singleton that occurred once in total, low count filter was set at a value of 0 and low variance filter was set at a 10 % value, based on the inter-quartile range. Rarefaction was set to library sizes of 697 and 948 reads for 16S and ITS respectively and data transformation was performed based on the centered-log ratio.

#### 4.1.10 Indoor air fungal spore concentrations in households

Measurement of airborne fungal spores was performed at the second visit of each sampling campaign using a bio-impactor (Sampl’air, BioMérieux, Marcy-l'Étoile, France) placed near the fruit basket. An airflow rate of 100 L/min was applied for 1 min onto M2Lev agar medium supplemented with 5 mg/L penicillin G and 5 mg/L streptomycin (Sigma-Aldrich, Darmstadt, Germany) which was then incubated at 25 °C for 2–3 days before enumeration.

#### 4.1.11 Data curation

FFV waste for each household was expressed in grams per day per household. Mean values from both visits were calculated for each season for refrigerator temperature and RH. Two additional binary variables (“Ref 13. Disinfect” and “Unref 5. Disinfect”) were created to highlight the distinction between households that declared cleaning their refrigerated or unrefrigerated storage areas using cleaning products proved to provide acceptable disinfecting properties over a 5 min application period, (vinegar, baking soda, dish soap, multi-purpose cleaner, wet wipes and bleach) [15] and those using only water or lemon juice. Concerning question items regarding the percentage of FFV purchased from various types of stores or from different sourcing (“Cons 1″ & “Cons 2”), the answers to each question were complementary for a given item. Answers were considered aberrant and removed if their total was lower than 70 % or exceeding 130 % for each item respectively.

### 4.2 Quantitative study on consumer anti-waste practices towards FFV

#### 4.2.1 Survey 1

The qualitative study, along with a review of the academic literature and of institutional reports, such as those published by the French Ministry of Agriculture or the ADEME [2] allowed us to generate a list of 43 items reflecting different anti-waste practices as evoked by participants. These practices ranged from storage to disposal behaviours.

Survey 1 aimed to quantify these anti-waste practices in order to determine which behaviours were both the most frequently adopted and the most commonly recommended. It included the 43 items transformed into questions as well as questions about the respondent’s socio-demographics such as gender, age and socio-professional category. Anti-waste practices were judged on a 5-points Likert scale based on frequency (From “Never = 1″ to “Always = 5″, with an option “Not Concerned = 0″). The questionnaire was filled anonymously and autonomously by 1048 French representative consumers belonging to “*Panelabs*”, a panel firm, in June 2023.

Consumers were recruited by the panel firm using a quota-based sampling method. Quotas were set on four key criteria, age, gender, socio-professional category (SPC), and region of residence, with an additional requirement to maintain balance in education level. As the survey was administered through an online panel, the panel firm did not disclose the number of invitations sent which would be necessary for the computation of a traditional response rate or the conduct of a non-response analysis. However, all questionnaire items were mandatory, resulting in no item-level missing data. The final sample fully satisfies the predefined quota structure, which mitigates potential selection bias.

The “Not concerned” responses were treated as missing data and were not included in the calculation of mean scores. Instead, their proportion was examined to assess consumers’ level of concern and the practical relevance of each item. Items showing a high share of “Not concerned” answers were primarily those related to composting and feeding leftovers to animals. To better interpret these patterns, two additional variables were introduced in survey 2 : whether respondents had access to a compost bin and whether they owned a pet. This approach allowed applicability to be cross-checked and genuine non-concern to be distinguished from situations where the practice was simply not feasible.

#### 4.2.2 Survey 2

After selecting the 19 items referring to the most frequently used (statistically) and the most highly recommended anti-waste practices (by referring to ADEME [2] and ANSES experts), a second survey (Survey 2) was conducted in August 2024 among 815 French consumers, representative of the population and recruited from the same panel firm. This second survey was collected under the same conditions as Survey 1. It was administered online using the same panel infrastructure and quota-based sampling method. The quotas were defined according to age, gender, socio-professional category (SPC), and region of residence, with the same requirement to ensure balance in education level. All questionnaire items were mandatory. The aim of survey 2 was to identify consumer clusters according to these anti-waste practices and to describe them with relevant variables.

The questionnaire included the 19 items formulated as questions with 5-points Likert scale options ranging from “Never = 1″ to “Always = 5″. In this second survey, the option “Not concerned” was not presented to respondents, as it was reported at particularly high rates in survey 1 for items related to composting and feeding leftovers to animals. In order to provide a distinction between a genuine lack of concern for the practice from the respondent and a situation where the practice was not feasible, two additional variables were introduced : “Do you have access to a compost bin?” and “Do you own a pet?”. Moreover, the questionnaire was restricted to the 19 practices that had already been validated in Survey 1 as both highly frequent and broadly relevant across consumers, which removed the need for this response category.

Additionally, three reported food waste scales as well as “auxiliary variables” scales such as environmental concern scale, concern towards food waste scale, past education towards food waste scale and FFV consumer involvement scale were included. Respondents had to answer on a 5-point Likert scale ranging from “Strongly Disagree = 1″ to “Strongly Agree = 5”. These variables were shown to be strongly associated with consumer food waste anti-waste behaviours and thus, serve as descriptors for consumer clusters. Socio-demographic related questions such as gender, age and socio-professional category were also included.

### 4.3 Spoilage Dynamic Assays at the Laboratory Scale

#### 4.3.1 Microbial strains

Three fungal strains (including *Penicillium expansum* UBOCC-A-125,030, *Penicillium italicum* UBOCC-A-125,031 *and Botrytis cinerea* UBOCC-A-125,032*)* and two bacterial strains (namely *Serratia marcescens* UBOCC-A-325,001 and *Pseudomonas fluorescens* group UBOCC-A-325,002 group) were included in this experiment. All strains originated from isolates obtained from spoiled fruits and vegetables collected in the 49 participating households [1].

#### 4.3.2 Experimental design

Four different fruits and vegetables were included in the experiment, namely tomatoes, lemons, carrots and apples. These products were selected as they are commonly consumed by French people, are available in retail outlets during most of the year, and are susceptible to colonization by the selected organisms. Moreover, these FFV were identified as frequently wasted by households over the year, as observed in a previous study focusing on the identification of microorganisms responsible for FFV waste at home [1]. The selected products were organically grown to minimize the impact of phytosanitary product residues on experimental outcomes.

Experiments were conducted at either 7 °C (to mimic refrigerator conditions) or 19 °C (corresponding to room temperature). Refrigerator temperature was defined based on previous findings indicating that the median temperature measured in the FFV compartments of refrigerators in the 49 French households was 7 °C, while the room temperature was set at 19 °C following French government’s recommendation for optimal household comfort temperatures (LegiFrance, 2022). Likewise, RH was set at 80 % using a saturated solution of ammonium sulphate prepared at 762 g/l as reported in the same results. For each assay, both temperature and RH were continuously recorded using Thermo Hygro probes (Proges Plus, Lille, France).

Three levels of microbial load contamination applied to storage surfaces were evaluated. They were defined as follows: i) a disinfected surface, ii) the median of storage surface contaminations observed in French households (10^2^ TFU/cm² for molds and 10^3^/cm^2^ CFU for bacteria) and iii) the third quartile (10^4^ TFU/cm² for molds and 10^5^ CFU/cm² for bacteria) and were hereafter designated as “none”, “moderate” and “strong” contamination levels.

Finally, the impact of the presence of wounds on spoilage was assessed using either intact or wounded products. Wounds were artificially created by cutting three scars (3-mm each) on the equator of the fruit or vegetable with a sterile scalpel, as previously described [16,17].

In total, the experimental plan covered 48 conditions (3 levels of microbial loads x 2 levels of storage temperatures x 2 levels of wounding x 4 types of FFV) tested on 10 replicates for each of the four different FFV. Due to practical constraints (considering the large number of FFV to monitor), the entire experiment was conducted over three different periods, each corresponding to one level of microbial load. Individual FFV for each of the temperature x contamination x wound combinations were observed and used as a statistical unit during the experiment. For each of these combinations, 2 boxes containing 5 units of each FFV were set up, corresponding to 2 replicates of each condition. The spoilage variables (``Damaged'' and ``Spoiled'' FFV, and ``Relative weight loss'') for each FFV type were then summed per condition for each replicate for further analysis.

Each experiment was conducted using a lidded hermetic plastic box in which the mix of selected FFV (five representatives per FFV type) was placed for storage. For each level of microbial load x storage temperature x wounding, FFV were placed into two different boxes. Prior to use, each plastic box was disinfected using 70 % ethanol followed by Duotex disinfectant (Laboratoires Rochex, Juvigny, France), and allowed to dry. Each FFV was rinsed with distilled water, immersed in a 70 % ethanol bath for 10 min and then allowed to dry on sterile filters. The “wounded” fruits and vegetables were subsequently incised using a sterile scalpel. Finally, fruits and vegetables from all experimental conditions were weighed, in order to compare their mass at the beginning and end of the experiment.

#### 4.3.3 Inoculation methods

Fungal spore suspensions for contaminated conditions were prepared from 5–7 day-old cultures grown on malt and yeast extract agar (M2Lev) medium (20 g/l malt extract, 3 g/l yeast extract, 15 g/l agar). Conidia were collected using 2 ml of sterile water supplemented with 0.01 % Tween 80 (Sigma Aldrich, Saint Quentin Fallavier, France) using a sterile l-shaped spreader. The obtained suspension was then collected and the conidia were enumerated using a Malassez hemocytometer. Dilutions were then carried out using the same solution to reach the targeted spore concentration (1.0 to- 5.0 × 10^7^ spores/ml).

For bacterial inoculation, fresh cultures grown on Plate Count Agar (PCA) medium (Difco, Saint-Ferréol, France) were used. One or two colonies were picked and transferred into a tryptone salt sterile solution (Biokar, Allonne, France) and then vigorously vortexed, before optical density measurement at 600 nm. Bacterial concentration was calculated and the suspension underwent a series of dilutions in tryptone salt to achieve the target concentration (1.0 to 5.0 × 10^8^ CFU/ml). Subsequently, 1 ml of each fungal and bacterial suspension was inoculated and spread evenly on the bottom of the plastic box using a sterile l-shaped spreader and allowed to dry for about 30 min under a laminar airflow. Control of inoculum was performed by inoculating a mock box with the suspension which was sampled by swabbing once dried. Swab underwent vortexing for about 30 s in tryptone salt solution (9 g/l) then the suspension was diluted and plated on M2Lev medium supplemented with 5 mg/l penicillin G and 5 mg/l streptomycin (Sigma-Aldrich, Darmstadt, Germany) and PCA medium supplemented with 11 mg/l amphotericin B (Sigma-Aldrich, Darmstadt, Germany) to count fungi and bacteria, respectively.

Once the inoculum had fully dried, five products per FFV type (tomato, lemon, carrot and apple) were placed in each box as mentioned above and incubated according to the various conditions.

#### 4.3.4 Monitoring of spoilage dynamics

**Fig. 1:**
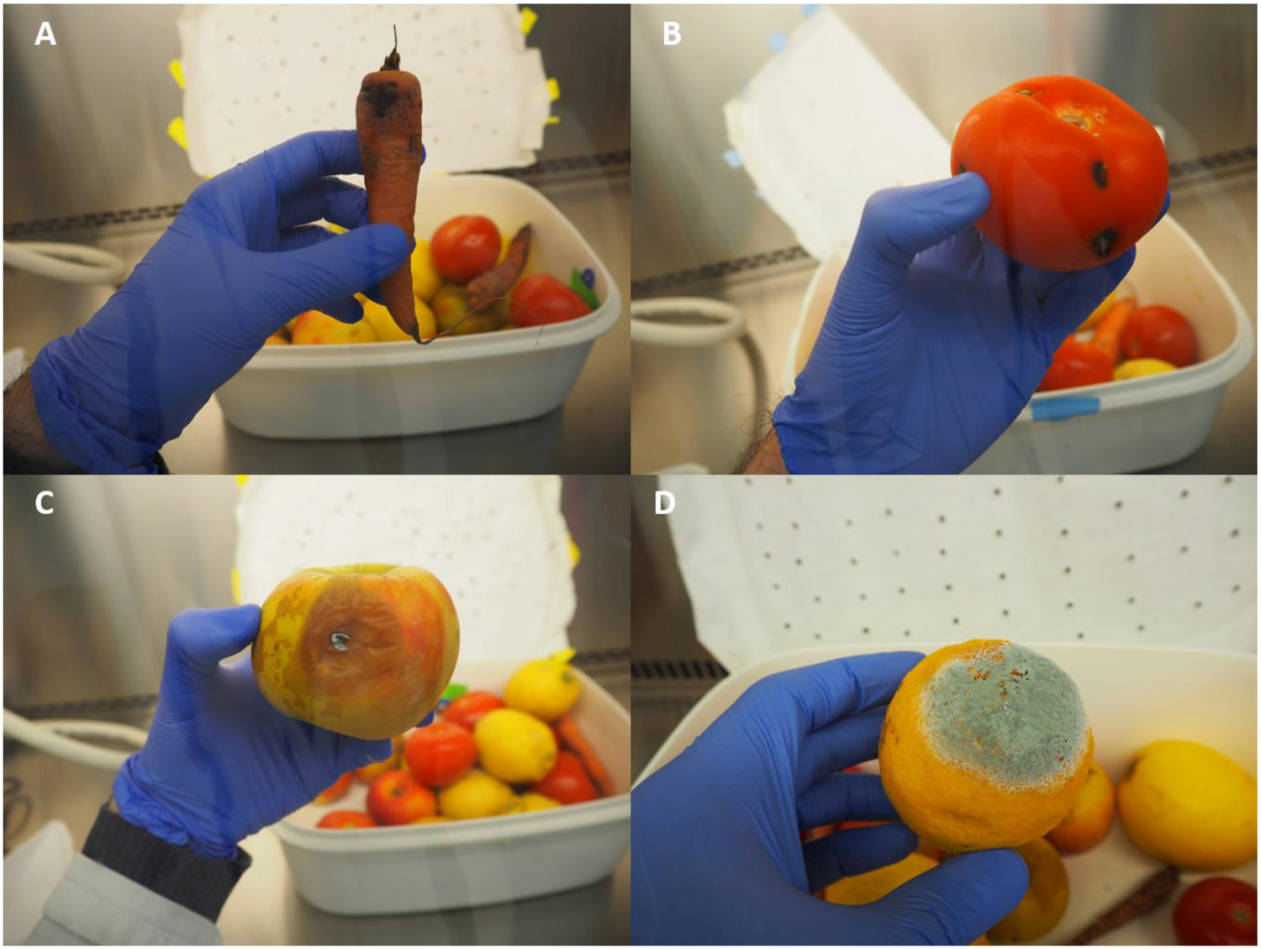
Photographs illustrating different types of FFV spoilage observed during the spoilage dynamic assay. Carrot browning (A), Dark lesions on tomato (B), Blue mold rot on apple (C) and lemon (D).

The experiment lasted 28 days. During the first 21 days, at intervals of 2–3 days, each plastic box was opened under sterile conditions to observe the products, take pictures and weigh the losses. After each observation, the products were randomly rearranged in order to vary which fruits and vegetables were in contact with the contaminated surface at the bottom of the box. For each observation, fruit or vegetable showing visible signs of spoilage were noted as “damaged”. Of them, those with spoilage affecting 50 % or more of the surface were noted as “spoiled”. Visible spoilage was defined as the presence of molds, browning, black spots, or rot on fruits and vegetables (Fig. 1). These spoiled products were then removed from the plastic box and weighed, as they were considered representative of items discarded by consumers in everyday life. Relative weight loss was then calculated following this formula:

$$Relative\;weight\;loss=\frac{Weight\;of\;discarded\;FFV}{Weight\;of\;discarded\;FFV+Weight\;of\;the\;remaining\;FFV}$$

On days 7, 14, 21 and 28, each of the three experiments was systematically observed, allowing comparisons between conditions to be performed at these predefined time points.

#### 4.3.5 Data curation

The obtained data comprised three main continuous dependent variables, namely number of “damaged” products, number of “spoiled” products and relative weight loss. The Area Under Disease Progression Curve (AUDPC) was computed at days 7, 14, 21 and 28 for each of the three variables [18] following the formula (R package *agricolae*):

$$AUDPC=\sum_{i=1}^{n-1}\;\frac{y_{i}+y_{i+1}}{2}\;\times \left(t_{i+1}-t_{i}\right)$$

where *y_i_* is a measurement of “symptom” levels (number of damaged products, number of spoiled products or relative weight loss of the considered product) at the *i*th observation, *t_i_* is the time (in days) at the *i*th observation and *n* is the total number of observations.

## Limitations

Concerning the FFV waste sampling campaigns, it would have been of interest to also measure the temperature and RH in the unrefrigerated storage areas to provide a more in depth view of the impact of these parameters on FFV waste. Similarly, the use of distinct connected bins for FFV waste stemming from either refrigerated or unrefrigerated storage types, as well as a metric of the total weight of FFV purchased and stored in each storage type would help decipher the actual contribution of each of the parameters when comparing storage types and household waste volumes. The choices made were done taking into account the acceptable level of commitment that could be requested from the participating households in order to obtain measures that matched reality while not being too constraining, and risking a slackening of efforts from households or higher numbers dropping off the campaigns.

Regarding the quantitative study on consumer anti-waste practices towards FFV, we used self-reported measures of practices and quantities of F&V discarded, which exposes our work to the risk of social desirability bias. We therefore emphasize the fact that these measures should be considered relative rather than absolute (Ananda et al., 2025).

## Ethics Statement

This research was conducted in full compliance with the General Data Protection Regulation (GDPR, Regulation (EU) 2016/679) and the French Data Protection Act (“Loi Informatique et Libertés”, amended Act No 78–17). All participants provided informed consent prior to data collection, as specified in the consent form approved by the research team.

The consent form clearly stated that the data collected would be used solely for academic research purposes and that all personal identifiers would be removed prior to analysis. Data were pseudonymised and stored securely on university servers, and a Data Management Plan (DMP) was declared to the funding agency, the French National Research Agency (ANR), in accordance with its open science and ethical data management requirements. This study does not involve medical or clinical research. No ethical approval protocol number was therefore required.

The research was carried out by the LEGO Research Laboratory (Laboratoire d’Économie et de Gestion de l’Ouest, Université de Bretagne Occidentale). A copy of the English version of the consent form is available in the *Consent form* section of the submission system.

## CRediT Author Statement

**Camille Marchal:** Formal analysis, Visualization, Writing - Original Draft; **Damien Ballan:** Conceptualization, Methodology, Investigation, Formal analysis, Visualization, Writing - Original Draft, Validation; **Sarra Azib:** Conceptualization, Methodology, Investigation, Formal analysis, Visualization, Writing - Original Draft, Validation; **Bertrand Urien:** Conceptualization, Methodology, Writing - Review & Editing, Validation; **Annick Tamaro:** Conceptualization, Methodology, Writing - Review & Editing, Validation; **Marine Le Gall-Ely:** Conceptualization, Methodology, Writing - Review & Editing, Validation; **Morgane Innocent:** Supervision, Funding acquisition, Resources, Investigation, Conceptualization, Methodology, Writing - Review & Editing, Validation; **Emmanuel Coton:** Supervision, Funding acquisition, Resources, Investigation, Conceptualization, Methodology, Writing - Review & Editing, Validation; **Adeline Picot:** Supervision, Funding acquisition, Resources, Investigation, Conceptualization, Methodology, Writing - Review & Editing, Validation; **Jérôme Mounier:** Supervision, Funding acquisition, Resources, Investigation, Conceptualization, Methodology, Writing - Review & Editing, Validation; **Patrick Gabriel:** Project administration, Supervision, Funding acquisition, Resources, Conceptualization, Methodology, Writing - Review & Editing, Validation; **Louis Coroller:** Supervision, Funding acquisition, Resources, Investigation, Conceptualization, Methodology, Writing - Review & Editing, Validation.

## Data Availability

Recherche Data GouvParticipatory and multi-disciplinary science dataset and surveys for the assessment of the microbiological and behavioural factors influencing fresh fruits and vegetables' waste at home. (Original data).

## Appendix Supplementary materials

Image, application 1
