# Supplementary material for: Participatory and multi-disciplinary science dataset and surveys for the assessment of the microbiological and behavioural factors influencing fresh fruits and vegetables' waste at home
Source: Data Brief. 2026 Jan 7;65:112434. doi: 10.1016/j.dib.2025.112434 (PMC12856149; doi:10.1016/j.dib.2025.112434)
Supplement: Supplementary file 1 [file mmc1.zip › Part1_FFV_waste_sampling_campaigns/Interviews/Doc1a_Interview_Guide_1.docx]

### **Interview Guide 1**

#### **Introductory Text**

We are now ready to begin this interview, during which I will ask you several questions about your consumption of fresh fruits and vegetables.
There are no right or wrong answers — what matters most is **your personal feelings and experiences**.

To make transcription easier and to ensure that your statements are not misinterpreted, I will record our conversation.
However, the data collected about you will remain **strictly anonymous**, as stated in the consent form you signed during our previous meeting.

Does that sound okay to you?

### **– VALUES OF FRUITS AND VEGETABLES**

*Alternate between “a fruit or a vegetable” and “a vegetable or a fruit” to avoid a halo-type bias.*

1. **First, I suggest a little guessing game.**
    I will ask you to think of a fruit or vegetable that you particularly like.
    Keep this fruit or vegetable in mind **without ever revealing its identity**!
    The idea is that I will try to guess what it is based solely on your description.

Do you have one in mind?
 Then go ahead — tell me everything about this fruit or vegetable.

**Follow-up questions:** • What is it like?
 • How can it be recognized, in your opinion?
 • At what time of day do you usually eat it (morning, noon, afternoon, evening)? Why?
 • How do you eat it — raw, cooked? Tell me what you do before consuming it.

- What are the different steps between the moment you decide to eat it and the moment you actually do?
   *(pre-consumption preparation: washing, peeling, cutting, cooking…)* • How often do you eat it approximately?
- Several times a day, once a day, several times a week, once a week...?
   • Where do you usually buy it?
   • What do you particularly like about this fruit or vegetable?
   • What do you feel when you eat it?
- In your opinion, what does it bring you? What are its advantages?
- Conversely, does it have any drawbacks (taste, preparation, shelf life, cost, accessibility)?

**End of the guessing game:** • I think your food was a fruit/vegetable.
 • And it was: ___________

### **(2)– PURCHASING, STORAGE, AND CONSUMPTION PRACTICES**

#### **Purchasing practices: “what, where, when, how, why, how much?”**

1. Now I’d like to talk about your shopping habits and your overall organization.
    For people living with others:
    Within your household, who is responsible for grocery shopping?
2. Tell me how your grocery shopping is organized.
    First, do you usually know what you’re going to buy before leaving for the store, or not really?

**Follow-up questions:** • Do you check your cupboards beforehand, or not?
 • Do you plan recipes in advance, or not?
 • Do you make a shopping list, or not?
 • Why?

1. Regarding fruits and vegetables, how do you decide on the quantities you buy?
2. You mentioned that you usually buy your ***F&V (from the guessing game)*** at ***such a place***.
    Is that true for all your fruits and vegetables, or not?

**Follow-up questions:** • Could you describe the different places where you buy or harvest your fruits and vegetables?
 • Why do you choose these places?
 • How often, approximately, do you go there?
 • Is it planned — for example, every Monday morning — or more spontaneous depending on your needs?

1. Could you list the different fruits and vegetables you usually buy?
    6.1 To start, which ones do you consume very regularly?
   - *Follow-up:* Why those ones in particular?
      6.2 Conversely, are there any fruits and vegetables you consume rarely or never?
   - *Follow-up:* Why rarely or never?

#### **Fruit and vegetable storage strategies (and knowledge of preservation)**

Now, let’s discuss your various fruit and vegetable storage places.

1. When you return from shopping, what do you do with your fruits?
    **Follow-up questions:** • Where and how do you store them?
    • Why?
    7.1 Do you consider this storage method ideal or not? Why?
2. When you return from shopping, what do you do with your vegetables?
    **Follow-up questions:** • Where and how do you store them?
    • Why?
    8.1 Do you consider this storage method ideal or not? Why?
3. Do you ever clean these storage areas or not?
    9.1 If yes, when and how do you do it?

#### **Preparation and consumption practices (and recipe knowledge = skills)**

Now, I’d like to discuss how you consume fruits and vegetables.

1. Let’s take a fruit you said you consume very regularly: __________
    In what form(s) do you eat it? (raw, cooked, recipes?)
2. Now let’s take a vegetable you said you consume very regularly: __________
    In what form(s) do you eat it? (raw, cooked, recipes?)
3. Are there any fruits or vegetables that you eat without peeling them? If yes, which ones?

### **(3) – FROM EDIBLE TO INEDIBLE**

Now, I’d like to discuss the moment when you decide to throw away a fruit or vegetable, or part of it.

1. When that happens, why do you decide to throw it away rather than consume it?
2. Generally speaking, how do you determine that a fruit or vegetable is no longer edible?
    • By sight, smell, touch, or taste?
3. When you are faced with a spoiled, rotten, or moldy fruit or vegetable, how do you feel?

### **(4)– FOOD WASTE: REPRESENTATIONS**

#### **4.1 Food waste: perceptions and representations**

To finish, I’d like to talk about food waste.

1. When I say “food waste,” what words or expressions spontaneously come to your mind?

16.1 Could you explain what “word 1” means to you?
 16.2 Could you explain what “word 2” means to you?
 16.3 Could you explain what “word 3” means to you?

1. Among all the words you mentioned, which one seems the most important to describe food waste?
    If you could choose only one, which would it be? Why?
2. It would be interesting to define what “food waste” means.
    Using your own words, could you give me your definition of food waste?
3. When you realize that you’re throwing away a fruit or vegetable, how do you feel?

#### **4.2 Use of the connected bin**

You have now been using the connected bin for a few days.

1. Tell me how it’s going. Do you feel like you use it frequently or rather rarely?
2. If you remember, could you tell me a few examples of what you’ve thrown away, and why?
3. In your opinion, what are the reasons why you sometimes end up throwing away or losing fruits and vegetables?
    22.1 Are there certain fruits or vegetables you throw away more often than others? Why do you think that is?

Regarding the practical use of this bin:

1. Have you ever hesitated before or after throwing away a fruit or vegetable — wondering, for example, “should this go in here or not”?
2. Have you ever forgotten to use the bin?
    If yes, that’s okay — we won’t hold it against you, but it’s important to know so we don’t report inaccurate results.
3. Have you changed the garbage bag or not? If yes, how many times?
