# Supplementary material for: Participatory and multi-disciplinary science dataset and surveys for the assessment of the microbiological and behavioural factors influencing fresh fruits and vegetables' waste at home
Source: Data Brief. 2026 Jan 7;65:112434. doi: 10.1016/j.dib.2025.112434 (PMC12856149; doi:10.1016/j.dib.2025.112434)
Supplement: Supplementary file 1 [file mmc1.zip › Part1_FFV_waste_sampling_campaigns/Interviews/Doc1b_Interview_Guide_2.docx]

### **Interview Guide 2**

### **(1)- Representations of Supply Locations**

4 cards : each one represents a supply location (supermarket, market, vegetable garden, greengrocer)


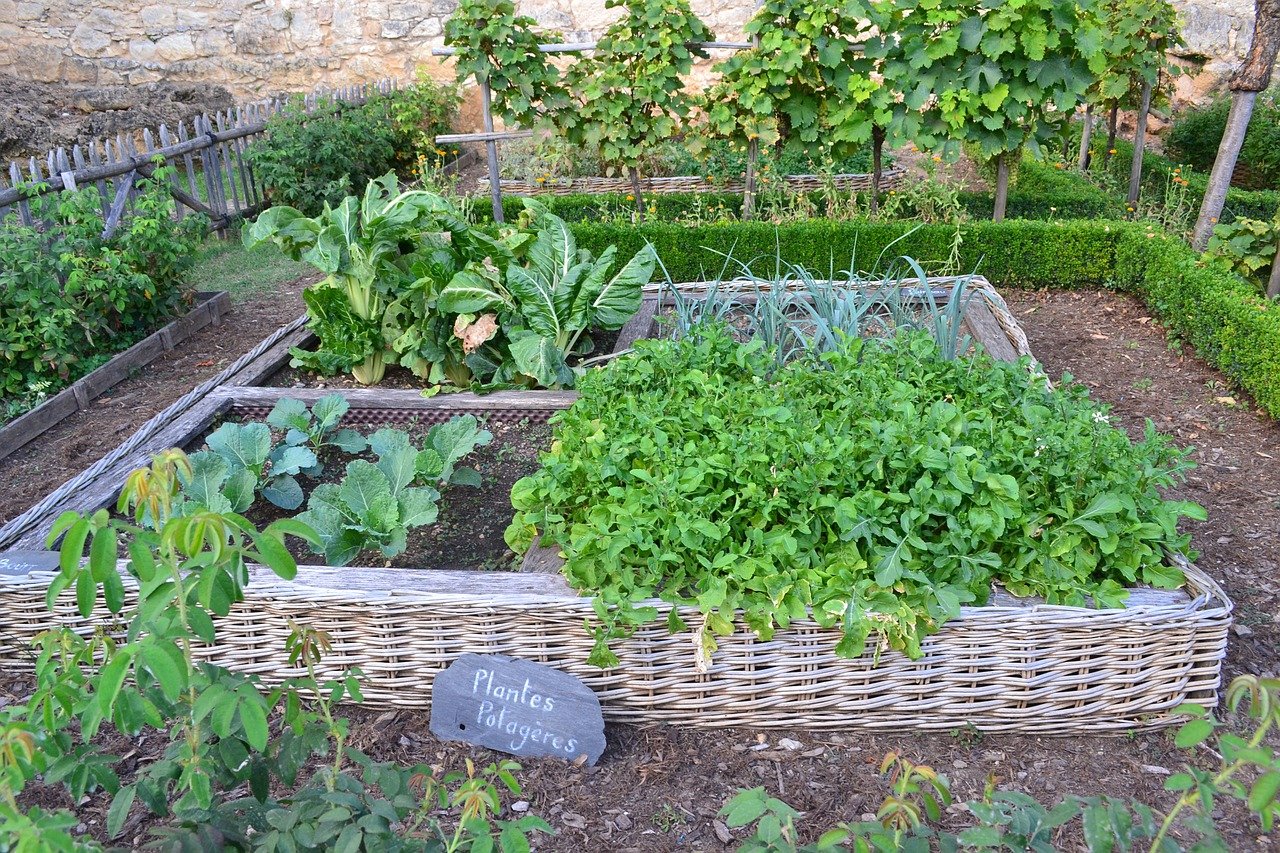

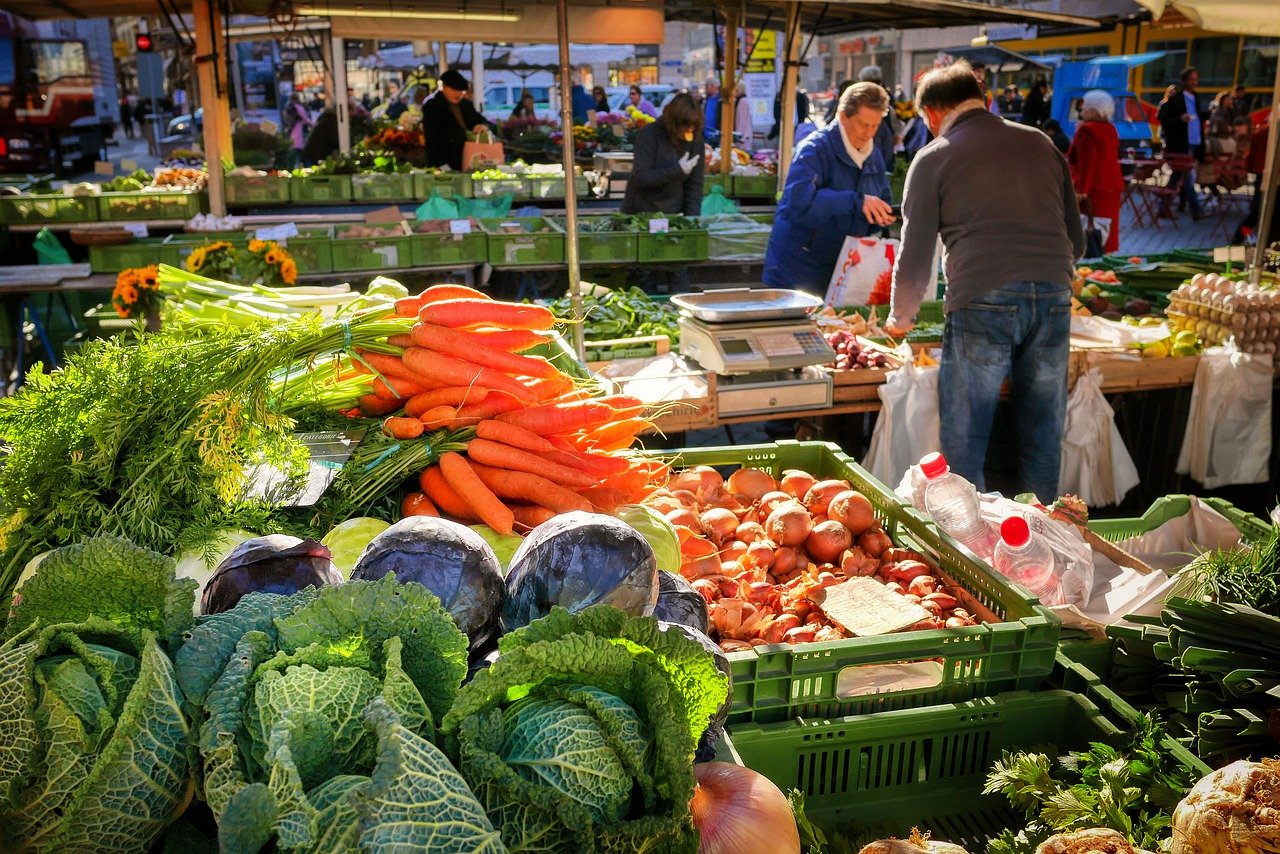

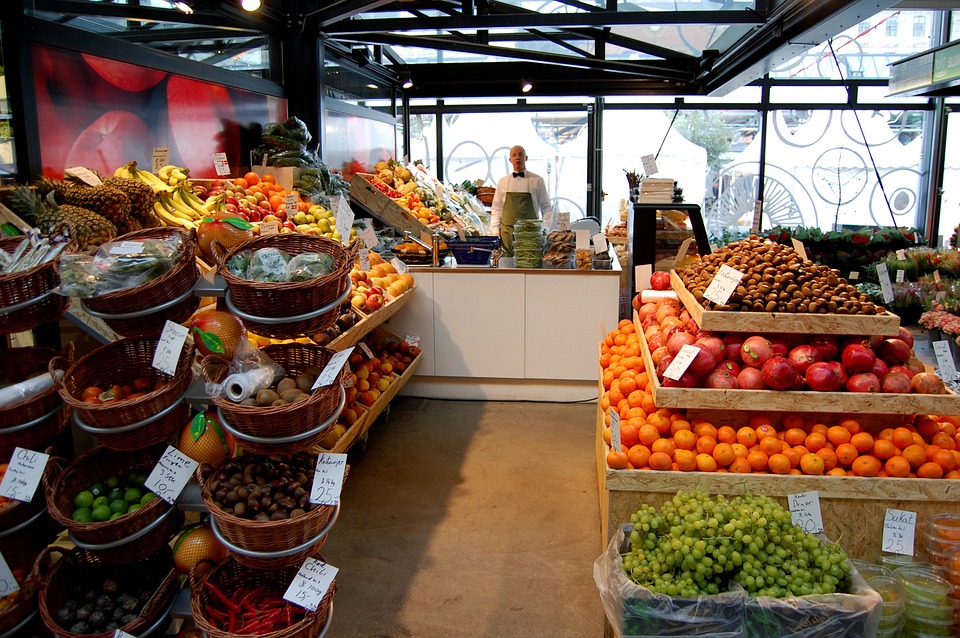

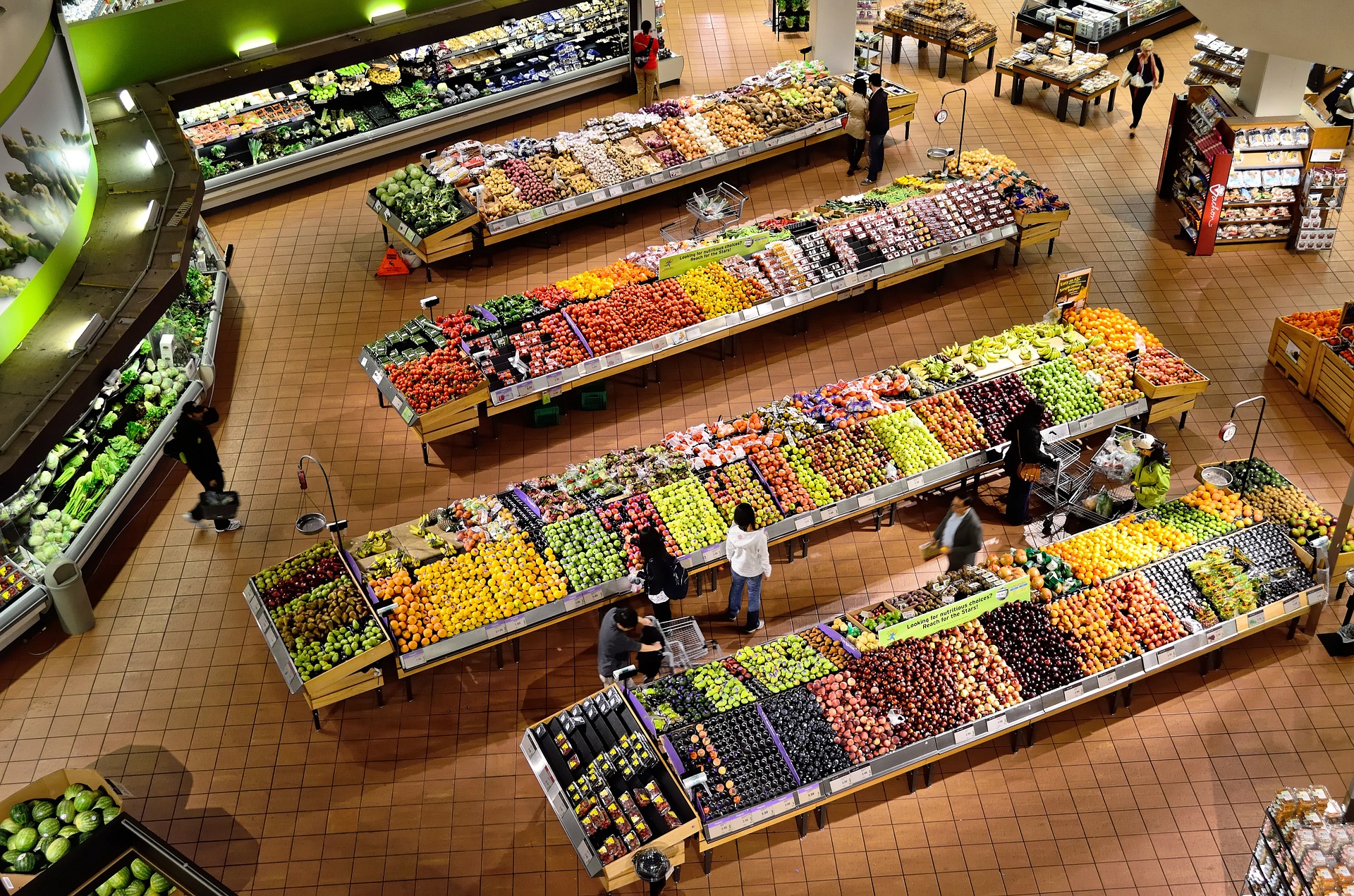


1. I’m going to ask you to choose one of these photos, turn it over, and tell me spontaneously the **first three words or expressions** that come to mind.
2. You mentioned “Word 1.” Could you tell me what it means to you? Why did you think of this word?
   *Then continue with Word 2; Word 3.*
3. Now, could you describe everything you see in this photo and tell me what it evokes for you?
4. In general, what do you think of this place?
5. Have you ever had any experience(s) with this type of place?
   - *If yes:* Could you tell me about it/them?
   - *If no:* Can you think of any particular reason why you have not experienced this type of place?
6. Is this a place where you would feel comfortable? Why?
7. Have you ever consumed products coming from this type of place? What do you think of them?
8. Do you ever throw away some of these products? Where do you dispose of them? What do you feel when doing so?

### **(2)- The Edibility Continuum**

10 images of bananas ranging from the least ripe to the most ripe.

1. Here are ten images of bananas. Could you arrange these bananas from the one that makes you **most want to eat it** to the one that makes you **least want to eat it**?
   - I will take a photo of your arrangement.
2. Now, I will ask you to talk about these bananas as if they were real and placed in front of you.
    Let’s start with the first one (describe its shape/color).
   - Could you describe it using your five senses?
   - What would you do with this banana at this stage, and why?
   - How do you feel about the idea of eating this banana?
3. Continue with the next banana images.
4. Now, I’ll ask you to summarize what you’ve said through the following three questions:
   - In your opinion, is there a point at which **eating the banana raw** would no longer be pleasurable? If yes, from which banana (shape/color)?
   - In your opinion, is there a point at which **eating the banana cooked** would no longer be pleasurable? If yes, from which banana (shape/color)?
   - In your opinion, is there a point at which the banana **loses its nutritional value**? If yes, from which banana (shape/color)?
   - In your opinion, is there a point at which the banana **could pose a potential health risk**? If yes, from which banana (shape/color)?

Depending on the interviewee’s answers, note from which point it remains acceptable to eat it raw/cooked — and why.
 Is there a point when it can **no longer be given to animals**? Why?

*(Keep in mind the different components of “value.”)*

### **(3)-: The Genesis of Fruits and Vegetables**

1. Here are photographs of fruits and vegetables (or parts of them) that you sent us just before throwing them away.
    Please pick the photo you wish to comment on.
   - Could you describe this photo?
   - Could you tell me why you threw away this fruit or vegetable?
      • Because of the taste?
      • Because it seemed unsafe?
2. Now, could you tell me the story of this fruit or vegetable?
    • Do you remember when you bought it, or not?
    • Where did you buy it?
    • Why did you buy it?
    • Where and how did you store it?
    • Why did you ultimately not consume it?
    • Do you remember what you felt at the moment you threw it away — or not?
