# Supplementary material for: Participatory and multi-disciplinary science dataset and surveys for the assessment of the microbiological and behavioural factors influencing fresh fruits and vegetables' waste at home
Source: Data Brief. 2026 Jan 7;65:112434. doi: 10.1016/j.dib.2025.112434 (PMC12856149; doi:10.1016/j.dib.2025.112434)
Supplement: Supplementary file 1 [file mmc1.zip › Part2_Quantitative_study_consumer_FFV_antiwaste_practices/Table09_Survey1/Table9a_Survey1_Questionnaire.docx]


 **[1-6]. Storage_spaces**
*Please indicate the frequencies associated with each of the following statements.*

*The ‘not applicable’ box is reserved for any statement that does not apply to your situation or is not relevant to your case.*

1.Where to store your fresh fruit and vegetables (FFV)

**1. fragile_fridge**
You store fragile FFV in the refrigerator.

**2. fragile_kitchen**
Store fragile FFV in the kitchen at room temperature (in a basket, container, etc.).

**3. fragile_cellar**
You store fragile FFV items in a separate place (cellar, cupboard, storeroom, separate room, etc.).

**4. conserv_fridge**
You store long-life FFV in the refrigerator.

**5. conserv_cuisine**
You store long-life fruit and vegetables in the kitchen at room temperature (in a basket, container, etc.).

**6. conserv_cellar**
You store long-life FFV products in a separate place (cellar, cupboard, storeroom, separate room, etc.).
Not applicable; Never; Seldom; Sometimes; Often; Always
This answer is mandatory. Scale : 0 ; 1 ; 2 ; 3 ; 4 ; 5



 **[7-13]. Storage_Tech**
*Please indicate the frequencies associated with each of the following statements.*

*The ‘not applicable’ box is reserved for any statement that does not apply to your situation or is not relevant to your case.*

2. Your fresh fruit and vegetables (FFV) storage routines

On the way back from shopping...

**7. pre_clean**
You wash the FFV before storing them

**8. pre_dry**
You dry the washed FFV before putting them away

**9. packaging_fragile1**
You store fragile FFV in the packaging in which they were sold to you (e.g. punnets, paper bags, plastic bags, etc.).

**10. packaging_fragile2**
You store fragile FFV in special storage containers (e.g. perforated bags, crates, boxes or refrigeration bags).

**11. packaging_conserv1**
You store long-life FFV in the packaging in which they were sold to you (e.g. punnets, paper bags, plastic bags, etc.).

**12. packaging_conserv2**
You store long-life FFV in special storage containers (e.g. perforated bags, crates, boxes or refrigeration bags).

**13. kitchenpaper**
You store you FFV on kitchen paper.
Not applicable; Never; Seldom; Sometimes; Often; Always
This answer is mandatory. Scale : 0 ; 1 ; 2 ; 3 ; 4 ; 5



 **[14-21]. Storage_Tech2**
2. Your fresh fruit and vegetables (FFV) storage routines (continued)

On the way back from shopping...

**14. heat_shelter**
You store FFV away from any source of heat.

**15. light_shelter**
You store FFV away from any source of light.

**16. fragile_visible**
You store fragile FFV in a visible and accessible place so that they can be consumed first.

**17. ripe_above**
You store the ripest FFV on top of the less ripe ones to prevent them from being crushed.

**18. not_touching**
You arrange the FFV so that they do not touch each other.

**19. isolate_ripe**
You store FFV separately if their presence accelerates the ripening of other FFV.

**20. sep_legume**
You store the different types of vegetables separated from each other.

**21. sep_fruits**
You store the different types of fruits separated from each other.
Not applicable; Never; Seldom; Sometimes; Often; Always
This answer is mandatory. Scale : 0 ; 1 ; 2 ; 3 ; 4 ; 5

**[22-23]. Unref_cleaning**
3. Your cleaning routines

**22. Unref_dirty**
You only clean your unrefrigerated FFV container when it is dirty.

**23. Unref_often**
You regularly clean your unrefrigerated FFV container.
Not applicable; Strongly disagree; Disagree; Neither agree nor disagree; Agree; Strongly agree
This answer is mandatory. Scale : 0 ; 1 ; 2 ; 3 ; 4 ; 5



 **24. Unref_freq**
How often do you clean your unrefrigerated FFV container?
About once a week; About once or twice a month; About once or twice per quarter; About once or twice a year; Don't know
This answer is mandatory. The question is only relevant if unref_often is among ‘Neither agree nor disagree;Agree;Strongly agree’.



 **[25-26]. Ref_cleaning**
Please indicate your level of agreement with each of the following statements.

**25. Ref_dirty**
You only clean your refrigerated FFV compartments when it is dirty.

**26. Ref_often**
You regularly clean your refrigerated FFV compartments.
Not applicable; Strongly disagree; Disagree; Neither agree nor disagree; Agree; Strongly agree
This answer is mandatory. Scale : 0 ; 1 ; 2 ; 3 ; 4 ; 5



 **27. Ref_freq**
How often do you clean your refrigerated FFV compartments ?
About once a week; About once or twice a month; About once or twice a year; Don't know
This answer is mandatory. The question is only relevant if Ref_often is among ‘Neither agree nor disagree;Agree;Strongly agree’.



 **28-29. Cleaning_product**
What product do you mainly use to clean the refrigerator?

Water only; Dishsoap; Baking soda; Multi-purpose cleaner (products or wipes); Lemon juice; Vinegar; Bleach; Don't know; Other
This answer is mandatory. A question asking ‘If Other, please specify’ is associated with this question. You may select multiple boxes. This question is only relevant if Ref_often is among ‘Neither agree nor disagree; Agree; Strongly agree’.

**29. Cleaning_product_other**
If “Other”, please specify:



 **30. Let_sit**

Do you leave the product on before rinsing?
Yes ; No
This answer is mandatory. The question is only relevant if Ref_often is among ‘Neither agree nor disagree; Agree; Strongly agree’.



 **31. Desinfection**
After this cleaning stage, do you proceed to a disinfection stage?

Yes ; No
This answer is mandatory. The question is only relevant if Ref_often is among ‘Neither agree nor disagree; Agree; Strongly agree’.



 **32-33. Desinfecting_product**
What type of disinfectant do you use most often?
Bleach; Lemon juice; Vinegar; Cleaning disinfectant (product or wipe); Household alcohol; Don't know; Other
This answer is mandatory. A question ‘If Other, please specify’ is associated with this question. You may tick multiple boxes. The question is only relevant if Disinfection is set to ‘Yes’.

**33. Desinfecting_product_other**
If “Other”, please specify:



 **34. Drying**
And finally, after cleaning, do you dry the various storage compartments?
Yes ; No
This answer is mandatory. The question is only relevant if Ref_often is among ‘Neither agree nor disagree; Agree; Strongly agree’.



 **[35-36]. Satisfaction**
4. For you...

**35. satisf_storage**
Your FFV storage routines are satisfactory.

**36. satisf_cleaning**
The cleaning routines for your FFV storage areas are satisfactory.
Strongly disagree; Disagree; Neither agree nor disagree; Agree; Strongly agree
This answer is mandatory. Scale : 1 ; 2 ; 3 ; 4 ; 5



 **[37-39]. Intent**
5. Your willingness to change your routines

**37. Intent_1**
If you had the right equipment, you would change your FFV storage and cleaning routines.

**38. Intent_2**
If you had the necessary knowledge and information, you would change your FFV storage and cleaning routines.

**39. Intent_3**
If you had the time, you would change your FFV storage and cleaning routines.
Strongly disagree; Disagree; Neither agree nor disagree; Agree; Strongly agree
This answer is mandatory. Scale : 1 ; 2 ; 3 ; 4 ; 5. The question is only relevant if satisf_cleaning is among ‘Strongly disagree ; Disagree ; Neither agree nor disagree ; Agree ; Strongly agree’.



 **[40-43]. Storage_skills**
Please indicate your level of agreement with each of the following statements.

6. Your general knowledge of storage

**40. Storage_skills1**
You know the appropriate places to store each FFV.

**41. Storage_skills2**
You know the ideal storage temperature for preserving FFV.

**42. Storage_skills3**
You know which compartments of the refrigerator to store FFV in.

**43. Storage_skills4**
You know how to choose storage locations based on the type of FFV.
Strongly disagree; Disagree; Neither agree nor disagree; Agree; Strongly agree
This answer is mandatory. Scale : 1 ; 2 ; 3 ; 4 ; 5



 **[44-46]. Cleaning_skills**
Please indicate your level of agreement with each of the following statements.

7. Your general knowledge for cleaning

**44. Cleaning_skills1**
You know which specific products to use to clean storage areas.

**45. Cleaning_skills2**
You know how often you should clean the refrigerator.

**46. Cleaning_skills3**
You know how often you should clean your unrefrigerated FFV container.
Strongly disagree; Disagree; Neither agree nor disagree; Agree; Strongly agree
This answer is mandatory. Scale : 1 ; 2 ; 3 ; 4 ; 5



 **47. Quiz1**
When the crisper tray is dirty, you must
clean it; disinfect it; clean and disinfect it; I don't know
This answer is mandatory. Scale : 0 ; 0 ; 1 ; 0



 **48. Quiz2**
When in contact with other fruits, bananas release a substance that:
accelerates the ripening of other fruits; slows down the ripening of other fruits; I don't know
This answer is mandatory. Scale : 1 ; 0 ; 0



 **49. Quiz3**
To extend the shelf life of bananas, I can store them in the crisper drawer of the refrigerator.
true; false; I don't know
This answer is mandatory. Scale : 0 ; 1 ; 0



 **50. Quiz4**
The presence of water condensation in the refrigerator's vegetable compartment:
increases the shelf life of fruit and vegetables; decreases the shelf life of fruit and vegetables; neither; I do not know
This answer is mandatory.



 **51. Quiz5**
Before storing strawberries, it is best to:
wash them to improve their shelf life; wash and dry them to improve their shelf life; do not wash them; I do not know
This answer is mandatory. Scale : 0 ; 0 ; 1 ; 0



 **52. Quiz6**
Consuming damaged FFV (wilted, dried out, bruised) is hazardous to your health.
true; false; false if the damaged part is removed; I don't know
This answer is mandatory. Scale : 0 ; 1 ; 0 ; 0



 **53. Quiz7**
Eating FFV that are mouldy or show signs of mould is dangerous to your health:
true; false; false if the mouldy part is removed; I don't know
This answer is mandatory. Scale : 0 ; 0 ; 1 ; 0



 **[54-61]. Preparation**
*Please indicate the frequencies associated with each of the following statements.*
*The ‘not applicable’ box is reserved for any statement that does not apply to your situation or is not relevant to your case.*

1.Vos routines de préparation des fruits et légumes (F&L) :

**54. prep_skin**
You cook or consume certain FFV with their skins on.

**55. prep_seeds**
You cook or consume certain FFV with their seeds.

**56. prep_stems**
You cook or consume the leaves or stems of certain FFV.

**57. prep_priority**
You prepare or consume the ripest or most fragile FFV first.

**58. prep_qty**
You cook FFV in advance for the coming days.

**59. prep_freeze**
You prepare frozen FFV.

**60. prep_preserve**
You make your own preserves from FFV.

**61. prep_impro**
You improvise meals from the FFV that are left in your storage areas.

Not applicable; Never; Seldom; Sometimes; Often; Always
This answer is mandatory. Scale : 0 ; 1 ; 2 ; 3 ; 4 ; 5



 **[62-63]. Donation**
Other practices series

**62. Donation_1**
You give some of the FFV you receive, purchase or harvest in large quantities to relatives or neighbours.

**63. Donation_2**
You give any surplus FFV you have left over when you go on holiday to neighbours or relatives
Never; Seldom; Sometimes; Often; Always
This answer is mandatory. Scale : 1 ; 2 ; 3 ; 4 ; 5



 **[64-71]. Val_util**
*Please indicate the frequencies associated with each of the following statements.*

2. Your consumption of fresh fruit and vegetables (FFV)

**64. vutil1**
Your consumption of fruit and vegetables provides you with vitamins and fibre.

**65. vutil2**
Your fruit and vegetable intake provides you with energy.

**66. vutil3**
Your fruit and vegetable intake keeps you hydrated.

**67. vutil9**
Your consumption of fruit and vegetables makes you feel full.

**68. vutil5**
Your fruit and vegetable intake helps you stay in shape.

**69. vutil6**
Your fruit and vegetable intake helps boost your immunity.

**70. vutil7**
Your fruit and vegetable intake helps you eat healthily.

**71. vutil8**
Your fruit and vegetable consumption helps improve your health.
Strongly disagree; Disagree; Neither agree nor disagree; Agree; Strongly agree
This answer is mandatory. Scale : 1 ; 2 ; 3 ; 4 ; 5



 **[72-80]. Val_knowledge**
2. Your consumption of FFV (continued)

**72. vknow1**
You enjoy learning about the different varieties and families of fruit and vegetables.

**73. vknow3**
you like to know the geographical origin of each fruit and vegetables.

**74. vknow4**
You like to know more about the seasonality of each fruit and vegetable.

**75. vknow5**
You like to know how each fruit and vegetable has been grown.

**76. vknow6**
You enjoy trying new fruit and vegetable.

**77. vknow7**
You enjoy discovering new flavour combinations between fruit and vegetables.

**78. vknow9**
You enjoy learning about fruit and vegetables (their storage, preparation, etc.).

**79. vknow10**
You enjoy devising recipes or preparations with fruit and vegetables.

**80. vknow11**
You enjoy sharing your knowledge about fruit and vegetables.
Strongly disagree; Disagree; Neither agree nor disagree; Agree; Strongly agree
This answer is mandatory. Scale : 1 ; 2 ; 3 ; 4 ; 5



 **[81-87]. Val_hedo**
2. Your consumption of FFV (continued)

**81. vhedo1**
You enjoy the texture (crunchy, juicy or melt-in-the-mouth) of certain fruit and vegetables.

**82. vhedo3**
You enjoy the taste of F&L.

**83. vhedo5**
You like the specific smell of each F&L.

**84. vhedo6**
You consumption of fruit and vegetables brings you pleasure .

**85. vhedo8**
Eating certain fruits and vegetables reminds you of a period or a memory.

**86. vhedo9**
You like the colours of fruits and vegetables.

**87. vhedo10**
Your consumption of fruit and vegetables gives you a feeling of well-being.
Strongly disagree; Disagree; Neither agree nor disagree; Agree; Strongly agree
This answer is mandatory. Scale : 1 ; 2 ; 3 ; 4 ; 5



 **[88-91]. Val_share**
2. Your consumption of FFV (continued)

**88. vshare1**
You enjoy discussing your FFV consumption with those around you.

**89. vshare2**
You enjoy eating certain FFV with family or friends (e.g. carrot sticks as an appetiser or raspberries as a snack or at a picnic)

**90. vshare4**
You enjoy preparing or cooking FFV when you have guests.

**91. vshare5**
You enjoy giving and receiving FFV in your circle of friends and family
Strongly disagree; Disagree; Neither agree nor disagree; Agree; Strongly agree
This answer is mandatory. Scale : 1 ; 2 ; 3 ; 4 ; 5



 **[92-97]. Val_ethics**
2. Your consumption of FFV (continued)

**92. vethics1**
You enjoy consuming FFV that have been produced in an environmentally friendly manner

**93. vethics3**
Your FFV consumption allows you to be environmentally responsible.

**94. vethics4**
Your FFV consumption enables you to support local producers/regions.

**95. vethics5**
Your FFV consumption allows you to respect the seasons.

**96. vethics6**
Your FFV consumption is consistent with your values.

**97. vethics7**
Your FFV consumption helps you to develop eating habits that are in line with your environmental concerns.

Strongly disagree; Disagree; Neither agree nor disagree; Agree; Strongly agree
This answer is mandatory. Scale : 1 ; 2 ; 3 ; 4 ; 5



 **[98-103]. Resid_util**
*Please indicate the frequencies associated with each of the following statements.*
*The ‘not applicable’ box is reserved for any statement that does not apply to your situation or is not relevant to your case.*

1. Your opinion on damaged fruit and vegetables

**98. resid_util1**
Damaged FFV still provide nutritional benefits.

**99. resid_util7**
Damaged FFV are still good for your health.

**100. resid_util3**
Damaged FFV can still be used in recipes (smoothies, baking, soups, compotes, dishes).

**101. resid_utill4**
Damaged FFV can still be used as animal feed.

**102. resid_util5**
Damaged FFV can be used to feed or enrich compost or soil.

**103. resid_util8**
Damaged FFV can make you feel full.
Not applicable; Strongly disagree; Disagree; Neither agree nor disagree; Agree; Strongly agree
This answer is mandatory. Scale : 0 ; 1 ; 2 ; 3 ; 4 ; 5



 **[104-107]. Resid_hedo**
Indicate your level of agreement with each of the following statements

**104. resid_hedo1**
Damaged FFV can still be enjoyable.

**105. resid_hedo3**
Damaged FFV can still taste good to you

**106. resid_hedo4**
Damaged FFV can still have a texture that you enjoy (soft, crunchy, juicy).

**107. resid_hedo5**
Damaged FFV can still smell pleasant to you.
Strongly disagree; Disagree; Neither agree nor disagree; Agree; Strongly agree
This answer is mandatory. Scale : 1 ; 2 ; 3 ; 4 ; 5



 **[108-109]. Resid_fin**
Indicate your level of agreement with each of the following statements

**108. resid_fin1**
Damaged FFV are still worth something to you financially.

**109. resid_fin2**
Damaged FFV still represents money that you have spent.
Strongly disagree; Disagree; Neither agree nor disagree; Agree; Strongly agree
This answer is mandatory. Scale : 1 ; 2 ; 3 ; 4 ; 5



 **[110-113]. Resid_symb**
Indicate your level of agreement with each of the following statements

**110. resid_symb1**
Damaged FFV still bears witness to the work of farmers.

**111. resid_symb2**
Damaged FFV still bears witness to a story, to the land where it grew.

**112. resid_symb3**
Damaged FFV is still evidence of the many resources mobilised upstream (water, fertiliser, soil, time, etc.).

**113. resid_symb4**
Damaged FFV is still food that should be respected and valued.
Strongly disagree; Disagree; Neither agree nor disagree; Agree; Strongly agree
This answer is mandatory. Scale : 1 ; 2 ; 3 ; 4 ; 5



 **[114-118]. Recuperation**
Indicate your level of agreement with each of the following statements

2. Your routines when faced with damaged FFV

**114. eat_as_is**

You eat a damaged FFV as it is, without processing or cutting it.

**115. cook_as_is**
You cook or process damaged FFV without cutting off the damaged part.

**116. cut_and_eat**

You cut off the damaged part of the FFV and eat what remains.

**117. cut_and_cook**
You cut off the damaged part of the FFV and cook or process what remains.

**118. cook_and_freeze**
You quickly cook any damaged FFV and store them in the refrigerator or freezer.
Never ; Seldom ; Sometimes ; Often ; Always
This answer is mandatory. Scale : 1 ; 2 ; 3 ; 4 ; 5



 **[119-121]. Good_discard**
*Indicate your level of agreement with each of the following statements*
*The ‘not applicable’ box is reserved for any statement that does not apply to your situation or is not relevant to your case.*

2. Your routines when faced with damaged FFV (continued)

**119. slow_ripening**
Place damaged FFV in the refrigerator or freezer to slow down the ripening process.

**120. compost**
You compost damaged FFV.

**121. animals**
You feed the damaged FFV to animals
Not applicable; Never; Seldom; Sometimes; Often; Always
This answer is mandatory. Scale : 0 ; 1 ; 2 ; 3 ; 4 ; 5



 **[122-127]. Cooking_skill**
*Indicate your level of agreement with each of the following statements*


3. You and cooking

**122. Cooking_skill1**
You know how to cook ‘home-cooked meals’ from raw ingredients.

**123. Cooking_skill2**
You know how to cook a meal on the spur of the moment using ingredients available at home.

**124. Cooking_skill3**
You know recipes that can be made using seasonal FFV.

**125. Cooking_skill4**
You know how to cook or prepare peelings, leaves, stalks or skins from FFV.

**126. Cooking_skill5**
You know how to use up damaged fruits.

**127. Cooking_skill6**
You know how to use up damaged vegetables.
Strongly disagree; Disagree; Neither agree nor disagree; Agree; Strongly agree
This answer is mandatory. Scale : 1 ; 2 ; 3 ; 4 ; 5



 **[128-133]. Waste_sensitivity**
*Indicate your level of agreement with each of the following statements*

**128. sensi_ind1**
You would be ashamed to waste food in front of someone

**129. sensi_ind2**
Managing food properly to minimise waste is a real concern to you.

**130. sensi_glob1**
Food waste poses problems for waste management in municipalities.

**131. sensi_glob2**
In France, food waste is now a major problem.

**132. sensi_glob3**
Food waste has truly harmful consequences for the planet.

**133. sensi_indiv3**
It affects you greatly to see someone throw away food that is still edible.
Strongly disagree; Disagree; Neither agree nor disagree; Agree; Strongly agree
This answer is mandatory. Scale : 1 ; 2 ; 3 ; 4 ; 5



 **[134-141]. Expectations**
*Indicate your level of agreement with each of the following statements*

*Consumers would waste less food if...*

**134. Expect2**
They were more aware of the environmental impact of food waste caused by households.

**135. Expect3**
They were more aware of the social impact of food waste caused by households.

**136. Expect4**
They were more aware of the economic impact of food waste caused by households.

**137.Expect1**
They were better informed about good food hygiene practices.

**138. Expect6**
They were better informed about the physical aspects that make food hazardous to health.

**139. Expect7**
They were better informed about anti-waste strategies to implement at home.

**140. Expect5**
They were encouraged by collective actions (e.g. community composting in the neighbourhood).

**141. Expect8**
They would have to pay money for what they waste.
Strongly disagree; Disagree; Neither agree nor disagree; Agree; Strongly agree
This answer is mandatory. Scale : 1 ; 2 ; 3 ; 4 ; 5



 **142. Gender**
You are :
Male; Female; Other
This answer is mandatory.



 **143. Age**
Which age group do you fall into?
18 to 24 years old; 25 to 34 years old; 35 to 44 years old; 45 to 54 years old; 55 to 64 years old; 65 years old or older.
This answer is mandatory.



 **144. educ_level**
What is your education level?
Without qualifications, Secondary school certificate; Vocational/professional qualifications; Baccalaureate; Two-year or three-year degree; Master's degree or higher
This answer is mandatory.



 **145. Pro_status**

You are :
In employment or seeking employment; Student; Retired; Not in employment
This answer is mandatory.



 **146. Sociopro_category**
What is your socio-professional category?
Agricultural worker ; Craftspeople, retailers and business owners ; Executives, higher intellectual professions or Liberal professions ; Technicians, supervisors and other intermediate professions ; Employees or Labourers

This answer is mandatory. The question is only relevant if Pro_status is among ‘In employment or seeking employment; Retired’.



 **147. Geo_location**
Where are you located?
Paris region ; North-West ; North-East ; South-West ; South-East
This answer is mandatory.



 **148. Fam_structure**
You live :

Single without child(ren); Single with child(ren); Couple without child(ren); Couple with child(ren) ; Other (with your parents, family members, friends, roommates, etc.)

This answer is mandatory. Tick a maximum of 2 boxes.



 **149. Nb_children**
Specify the number of children:

1 ; 2 ; 3 ; 4 ; 5 ; More than 5
This answer is mandatory. The question is only relevant if Fam_structure is among ‘Single with child(ren); Couple with child(ren)’.



 **150. Nb_inhabitants**
More specifically, how many people are in your household?
1 person; 2 persons; 3 persons; 4 persons; 5 persons or more
This answer is mandatory.



 **151-152. Accomodation**
Your accommodation:
Apartment ; House ; Other
This answer is mandatory. A question ‘If Other, please specify’ is associated with this question.

**152. Accomodation_other**
If “Other”, please specify:



 **153. Animal_household**
Do you have any pets?

Yes ; No
This answer is mandatory.



 **154. Compost_household**
Do you have an individual or communal compost bin?
Yes ; No
This answer is mandatory.



 **155. Garden_household**
Do you have a vegetable garden?
Yes ; No
This answer is mandatory.



 **156. Income**
To enable us to classify your answers according to living standards, please indicate the bracket in which your household's net monthly income (all income combined) falls:
Less than €1,500; €1,500 to €2,000; €2,000 to €2,500; €2,500 to €3,000; €3,000 to €3,500; €3,500 to €4,000; More than €4,000; Prefer not to say; Don't know
This answer is mandatory.

**Publication variables**

**

 157. KEY**
The response is automatic (primary key).



 **158. INPUT_DATE**
The reply is automatic. ‘dd/mm/yyyy hh:mm:ss’.



 **159. SAVE_DATE**
The reply is automatic. ‘dd/mm/yyyy hh:mm:ss’.



 **160. CHANGE_DATE**
The reply is automatic. ‘dd/mm/yyyy hh:mm:ss’.



 **161. FILL_DURATION**
The reply is automatic.



 **162. FILL_ORIGIN**

**

 163. FILL_LANGUAGE**
The reply is automatic.



 **164. FILL_DEVICE**
PC ; Tablet ; Smartphone
The reply is automatic.



 **165. PROGRES**
In progress; Completed; QuotaFull; ScreenOut
The reply is automatic.



 **166. LAST_QUESTION_ENTERED**
The reply is automatic.
