# Supplementary material for: Participatory and multi-disciplinary science dataset and surveys for the assessment of the microbiological and behavioural factors influencing fresh fruits and vegetables' waste at home
Source: Data Brief. 2026 Jan 7;65:112434. doi: 10.1016/j.dib.2025.112434 (PMC12856149; doi:10.1016/j.dib.2025.112434)
Supplement: Supplementary file 1 [file mmc1.zip › Part2_Quantitative_study_consumer_FFV_antiwaste_practices/Table10_Survey2/Table10a_Survey2 _Questionnaire.docx]

**Final questionnaire - cannot be changed**

**Introduction**

Hello,

The **LEGO** laboratory (Laboratoire d'économie et de gestion de l'Ouest) and the **LUBEM** laboratory (Laboratoire universitaire de biodiversité et d'écologie microbienne) are conducting a study on the **consumption and management of fruit and vegetables**, which will be used, among other things, to complete a doctoral thesis.

We would therefore like to invite you to take part in our survey and would be very grateful if you could do so.

Your answers will be treated anonymously and used for statistical purposes only, in accordance with the General Data Protection Regulation (GDPR)*.

There are no right or wrong answers. Please answer spontaneously.

Thank you in advance for your participation 🙂.

*<https://www.economie.gouv.fr/entreprises/reglement-general-protection-donnees-rgpd>


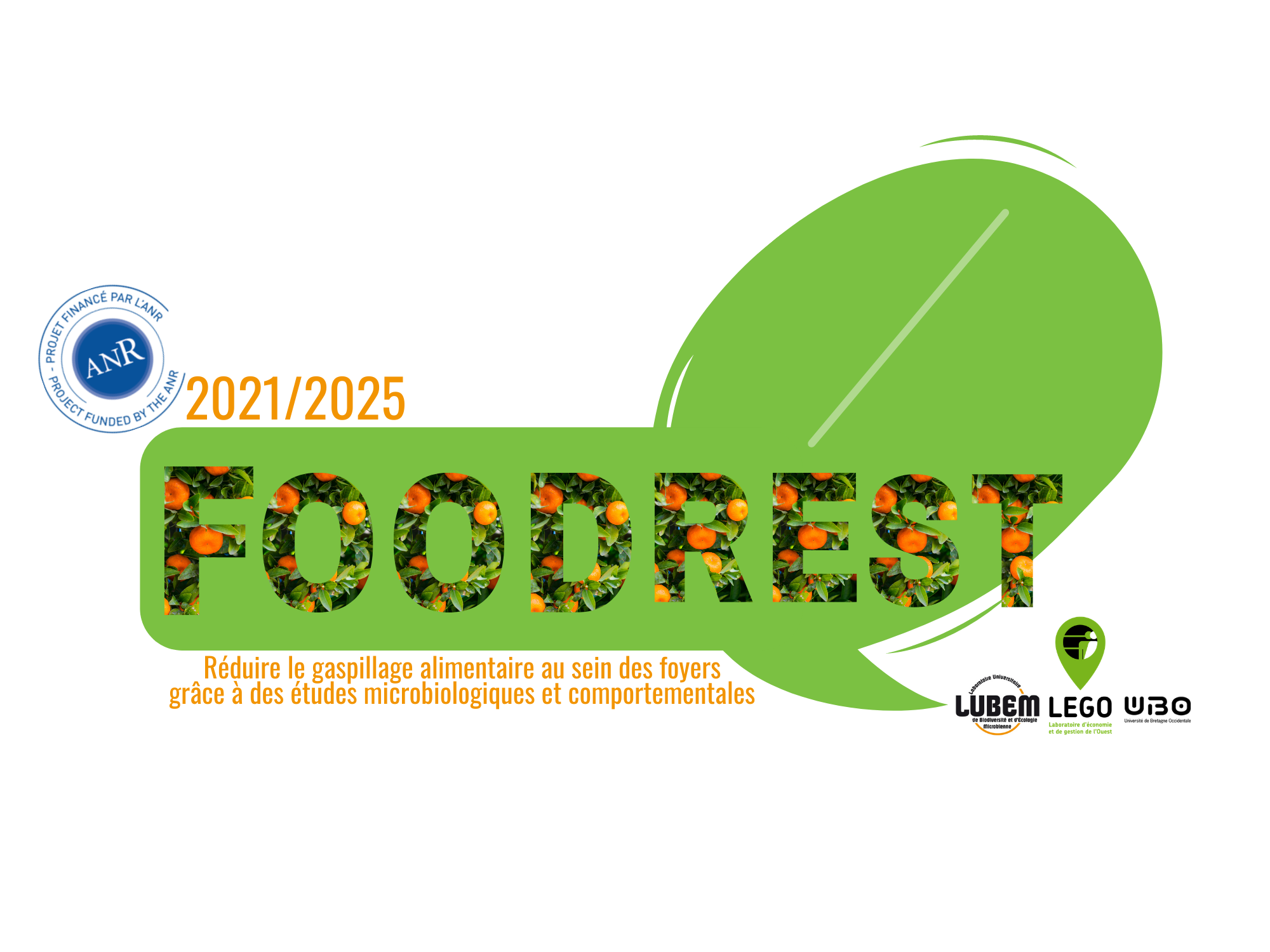


→ Next step for the participant : Q1

**S1 - Filter question/disqualifier**

**Page 1**

**Q1**

**At home, you consume fresh fruit and vegetables:**

*One answer only*

Single-choice question

| 1 | Never or almost never (less than once a month) |
| --- | --- |
| 2 | Occasionally (once or twice a month) |
| 3 | Regularly (3 to 4 times a month) |
| 4 | Very often (once a week or more) |

**Redirection case :**

**CASE 1**

If option “**1. Never or almost never (less than once a month)**” is ticked in Q1

→ The participant is then redirected towards Conclusion Profile disqualified.

**ELSE**

→ The participant is then redirected towards Q2

**S2 - Section 1**

**Page 2**

**Q2**

**Fruit and vegetables for you**

*Please indicate your level of agreement with the following statements*

Single-choice table

With lines in random order

|  |  | **Strongly disagree** | **Disagree** | **Neither agree nor disagree** | **Agree** | **Strongly agree** |
| --- | --- | --- | --- | --- | --- | --- |
| Q2_1 | Fruit and vegetables are something I attach particular importance to. | 1 | 2 | 3 | 4 | 5 |
| Q2_2 | You could say that fruit and vegetables are an area that interests me. | 1 | 2 | 3 | 4 | 5 |
| Q2_3 | I feel particularly drawn to everything related to fruit and vegetables in general. | 1 | 2 | 3 | 4 | 5 |

→ Next step for the participant : Q3

**Page 3**

**Q3**

**Your purchasing habits for fresh fruit and vegetables**

*Please indicate the frequency associated with each of the following practices.*

Single-choice table

With lines in random order

|  |  | **Never** | **Seldom** | **Sometimes** | **Often** | **Always** |
| --- | --- | --- | --- | --- | --- | --- |
| Q3_1 | You prepare a shopping list of fruit and vegetables before going shopping. | 1 | 2 | 3 | 4 | 5 |
| Q3_2 | You buy fruit and vegetables in small quantities, as and when you need them. | 1 | 2 | 3 | 4 | 5 |
| Q3_3 | You check your stock of fruit and vegetables before going shopping. | 1 | 2 | 3 | 4 | 5 |

→ Next step for the participant : T1

**Page 4 - Transition**

**T1**

Some types of fruit and vegetables are damaged much more quickly than others. They will be referred to as ‘fragile’ in this part of the questionnaire.e.

Example: strawberries, lettuce, raspberries, radishes

→ Next step for the participant : Q4

**Page 5**

**Q4**

**Your storing practices for fresh fruit and vegetables**

*Please indicate the frequency associated with each of the following practices.*

Single-choice table

With lines in random order

|  |  | **Never** | **Seldom** | **Sometimes** | **Often** | **Always** |
| --- | --- | --- | --- | --- | --- | --- |
| Q4_1 | You store fragile fruit and vegetables in the refrigerator to preserve them longer. | 1 | 2 | 3 | 4 | 5 |
| Q4_2 | You freeze fresh fruit and vegetables to preserve them longer. | 1 | 2 | 3 | 4 | 5 |
| Q4_3 | You store the most fragile or ripe fruit and vegetables in a visible location (e.g. on a shelf or in a visible compartment). | 1 | 2 | 3 | 4 | 5 |
| Q4_4 | You store heavy fruit and vegetables underneath lighter or more fragile fruit and vegetables. | 1 | 2 | 3 | 4 | 5 |
| Q4_5 | When storing fruit and vegetables, you isolate those that accelerate the ripening of others (e.g. bananas). | 1 | 2 | 3 | 4 | 5 |
| Q4_6 | You remove fruit and vegetables that are starting to spoil from the storage area or container so that they do not contaminate the others. | 1 | 2 | 3 | 4 | 5 |
| Q4_7 | In the refrigerator, you clean the areas used to store fruit and vegetables as soon as they become dirty.  (fixed line) | 1 | 2 | 3 | 4 | 5 |

→ Next step for the participant : Q5

**Page 6**

**Q5**

**Your practices regarding the consumption of fresh fruit and vegetables**

*Please indicate the frequency associated with each of the following practices.*

Single-choice table

With lines in random order

|  |  | **Never** | **Seldom** | **Sometimes** | **Often** | **Always** |
| --- | --- | --- | --- | --- | --- | --- |
| Q5_1 | You prioritise consuming or using the ripest or most fragile fruit and vegetables. | 1 | 2 | 3 | 4 | 5 |
| Q5_2 | You cook fruit and vegetables in advance for the next few days. | 1 | 2 | 3 | 4 | 5 |
| Q5_3 | You share surplus fresh fruit and vegetables with family, neighbours or other people (e.g. when you go on holiday). | 1 | 2 | 3 | 4 | 5 |
| Q5_4 | You share fruit and vegetables that are starting to spoil with family, neighbours or other people. | 1 | 2 | 3 | 4 | 5 |

→ Next step for the participant : Q6

**Page 7**

**Q6**

**Vegetables: In general, how often do you throw vegetables (excluding peelings) in the bin?**

*Please indicate the frequency*

Single-choice question

| 1 | 6 to 7 times **a week** |
| --- | --- |
| 2 | 3 to 5 times **a week** |
| 3 | 1 to 2 times **a week** |
| 4 | 2 to 3 times **a month** |
| 5 | Once **a month** |
| 6 | Less than once **a month** or never |

→ Next step for the participant : Q7

**Page 8**

**Q7**

**In general, how many portions of vegetables do you throw away each time (excluding peelings)?**

**(one portion = one handful of vegetables)**

*One answer only*

Single-choice question

| 1 | 1 portion, 1/2 portion or less |
| --- | --- |
| 2 | 1 to 2 portions |
| 3 | More than 3 portions |

→ Next step for the participant : Q8

**Page 9**

**Q8**

**Fruit: In general, how often do you throw fruit (excluding peelings) in the bin?**

*Please indicate the frequency*

Single-choice question

| 1 | 6 to 7 times **a week** |
| --- | --- |
| 2 | 3 to 5 times **a week** |
| 3 | 1 to 2 times **a week** |
| 4 | 2 to 3 times **a month** |
| 5 | Once **a month** |
| 6 | Less than once **a month** or never |

→ Next step for the participant : Q9

**Page 10**

**Q9**

**In general, how many portions of fruit do you throw away each time (excluding peel)?**

**(one portion = a handful of fruit)**

*One answer only*

Single-choice question

| 1 | 1 portion, 1/2 portion or less |
| --- | --- |
| 2 | 1 to 2 portions |
| 3 | More than 3 portions |

→ Next step for the participant : T2

**S3 - Section 3**

**Page 11 - Transition**

**T2**

In this section, we define damaged fruit or vegetables as: products whose initial physical condition has changed.

This change can be:

- **a change in shape or texture** (e.g. soft, wilted texture)

**AND/OR**

- **a change in colour (e.g. brown spots, blackheads)**

**AND/OR**

- **the appearance of an external agent** (e.g. small mould)

→ Next step for the participant : Q10

**Page 12**

**Q10**

**Your feelings: when you find damaged fruit or vegetables at home,**

*one answer per line*

Single-choice table

With lines in random order

|  |  | **Not at all** | **Slightly** | **Moderately** | **Very** | **Extremely** |
| --- | --- | --- | --- | --- | --- | --- |
| Q10_1 | You feel guilty | 1 | 2 | 3 | 4 | 5 |
| Q10_2 | You feel remorseful. | 1 | 2 | 3 | 4 | 5 |
| Q10_3 | You have a guilty conscience. | 1 | 2 | 3 | 4 | 5 |

→ Next step for the participant : Q11

**Page 13**

**Q11**

**For each of the following statements, please indicate your level of agreement.**

**NB: Damaged fruit or vegetables are products whose original condition has changed: change in shape and/or colour and/or appearance of small mould spots.**

Single-choice table

|  |  | **Strongly disagree** | **Disagree** | **Neither agree nor disagree** | **Agree** | **Strongly agree** |
| --- | --- | --- | --- | --- | --- | --- |
| Q11_1 | Damaged fruit/vegetables still provide nutritional benefits. | 1 | 2 | 3 | 4 | 5 |
| Q11_2 | Damaged fruit/vegetables are still good for your health. | 1 | 2 | 3 | 4 | 5 |
| Q11_3 | Damaged fruit/vegetables can still be used in recipes (smoothies, baking, soups, compotes, dishes). | 1 | 2 | 3 | 4 | 5 |
| Q11_4 | Damaged fruit or vegetables are still nutritious. | 1 | 2 | 3 | 4 | 5 |
| Q11_5 | Damaged fruit or vegetables can still be useful for cooking or processing. | 1 | 2 | 3 | 4 | 5 |

→ Next step for the participant : Q12

**Page 14**

**Q12**

**For the following questions, even if you do not have compost or pets, please answer according to your personal opinion, indicating your level of agreement.**

**NB: Damaged fruit or vegetables are products whose original condition has changed: change in shape and/or colour and/or appearance of small mould spots.**

*one answer per line*

Single-choice table

|  |  | **Strongly disagree** | **Disagree** | **Neither agree nor disagree** | **Agree** | **Strongly agree** |
| --- | --- | --- | --- | --- | --- | --- |
| Q12_1 | Damaged fruit/vegetables can still be used as animal feed. | 1 | 2 | 3 | 4 | 5 |
| Q12_2 | Damaged fruit/vegetables can be used to feed or enrich compost or soil. | 1 | 2 | 3 | 4 | 5 |
| Q12_3 | Damaged fruit and vegetables can still be used to enrich the soil and improve its quality. | 1 | 2 | 3 | 4 | 5 |
| Q12_4 | Damaged fruit and vegetables can still be used to make natural fertilizer. | 1 | 2 | 3 | 4 | 5 |
| Q12_5 | Damaged fruit and vegetables can still be used to contribute to a communal compost heap. | 1 | 2 | 3 | 4 | 5 |
| Q12_6 | Damaged fruit/vegetables can still return to nature and contribute to the life cycle of plants. | 1 | 2 | 3 | 4 | 5 |
| Q12_7 | Damaged fruit or vegetables can still be used to feed insects or earthworms. | 1 | 2 | 3 | 4 | 5 |

→ Next step for the participant : Q13

**Page 15**

**Q13**

**For the following questions, even if you do not have compost or pets, please answer according to your personal opinion, indicating your level of agreement.**

**NB: Damaged fruit or vegetables are products whose original condition has changed: change in shape and/or colour and/or appearance of small mould spots.**

*one answer per line*

Single-choice table

|  |  | **Strongly disagree** | **Disagree** | **Neither agree nor disagree** | **Agree** | **Strongly agree** |
| --- | --- | --- | --- | --- | --- | --- |
| Q13_1 | Damaged FFV can still be enjoyable. | 1 | 2 | 3 | 4 | 5 |
| Q13_2 | Damaged FFV can still taste good to you | 1 | 2 | 3 | 4 | 5 |
| Q13_3 | Damaged FFV can still have a texture that you enjoy (soft, crunchy, juicy) | 1 | 2 | 3 | 4 | 5 |
| Q13_4 | Damaged FFV can still smell pleasant to you | 1 | 2 | 3 | 4 | 5 |

→ Next step for the participant : Q14

**Page 16**

**Q14**

**For each of the following statements, please indicate your level of agreement.**

**NB: Damaged fruit or vegetables are products whose original condition has changed: change in shape and/or colour and/or appearance of small mould spots.**

*one answer per line*

Single-choice table

With lines in random order

|  |  | **Strongly disagree** | **Disagree** | **Neither agree nor disagree** | **Agree** | **Strongly agree** |
| --- | --- | --- | --- | --- | --- | --- |
| Q14_1 | Damaged FFV still bears witness to the work of farmers. | 1 | 2 | 3 | 4 | 5 |
| Q14_2 | Damaged FFV still bears witness to a story, to the land where it grew | 1 | 2 | 3 | 4 | 5 |
| Q14_3 | Damaged FFV is still evidence of the many resources mobilised upstream (water, fertiliser, soil, time, etc.) | 1 | 2 | 3 | 4 | 5 |
| Q14_4 | Damaged FFV is still food that should be respected and valued | 1 | 2 | 3 | 4 | 5 |

→ Next step for the participant : Q15

**Page 17**

**Q15**

**If someone eats a damaged fruit or vegetable tonight**

*one answer per line*

Single-choice table

|  |  | **Strongly disagree** | **Disagree** | **Neither agree nor disagree** | **Agree** | **Strongly agree** |
| --- | --- | --- | --- | --- | --- | --- |
| Q15_1 | They are likely to be ill in the coming days | 1 | 2 | 3 | 4 | 5 |
| Q15_2 | This illness will be dangerous to the person's health. | 1 | 2 | 3 | 4 | 5 |

→ Next step for the participant : Q16

**Page 18**

**Q16**

**You have just answered a series of questions about a “damaged” fruit or vegetable. Which key feature(s) would you choose to describe the product(s) you thought of?**

*Several answers are possible*

Multiple-choice question

| 1 | Change in shape (soft, wilted texture) |
| --- | --- |
| 2 | Colour change (brown spots or black dots) |
| 3 | Outside agent showing up (mould) |

→ Next step for the participant : Q17

**Page 19**

**Q17**

**Your practices for managing damaged fruit and vegetables.**

*Please indicate the frequency associated with each of the following practices..*

Single-choice table

|  |  | **Never** | **Seldom** | **Sometimes** | **Often** | **Always** |
| --- | --- | --- | --- | --- | --- | --- |
| Q17_1 | You eat damaged fruit or vegetables asap, without processing or cutting it. | 1 | 2 | 3 | 4 | 5 |
| Q17_2 | You transform, without cutting away the damaged part, damaged fruit or vegetables into a new dish, salad or dessert (or other). | 1 | 2 | 3 | 4 | 5 |
| Q17_3 | You discard the damaged part of a fruit or vegetable and eat or process the rest. | 1 | 2 | 3 | 4 | 5 |
| Q17_4 | You improvise a recipe specifically to save damaged fruit or vegetables. | 1 | 2 | 3 | 4 | 5 |
| Q17_5 | You put damaged fruit or vegetables in the refrigerator to slow down their ripening (and keep them longer). | 1 | 2 | 3 | 4 | 5 |
| Q17_6 | You throw damaged fruit or vegetables straight into the bin. | 1 | 2 | 3 | 4 | 5 |
| Q17_7 | You put damaged fruit or vegetables in the compost (individual or communal). | 1 | 2 | 3 | 4 | 5 |
| Q17_8 | You give damaged fruit or vegetables to animals (pets or others). | 1 | 2 | 3 | 4 | 5 |

→ Next step for the participant : T3

**S4 - Section 4**

**Page 20 - Transition**

**T3**

We will now look at your practices in your home over the last 7 days.

→ Next step for the participant : Q18

**Page 21**

**Q18**

**Vegetables: Over the past 7 days, how much vegetable waste (excluding peelings) did your household throw away?**

*One answer only*

Single-choice question

| 1 | Nothing |
| --- | --- |
| 2 | Almost nothing |
| 3 | A little |
| 4 | A lot |
| 5 | A great deal |
| 6 | I was not at home for the last 7 days |

**Redirection case :**

**CASE 1**

If option **“6. I was not at home during the last 7 days”** is ticked in Q18

→ The participant is then redirected to T4

**ELSE**

→ The participant is redirected to Q19

**Page 22**

**Q19**

**Fruit: Over the past 7 days, how much fruit waste (excluding peelings) did your household throw away?**

*One answer only*

Single-choice question

| 1 | Nothing |
| --- | --- |
| 2 | Almost nothing |
| 3 | A little |
| 4 | A lot |
| 5 | A great deal |
| 6 | I was not at home for the last 7 days |

**Redirection case :**

**CASE 1**

If option **“6. I was not at home during the last 7 days”** is ticked in Q19

→ The participant is then redirected to T4

**ELSE**

→ The participant is redirected to Q20

**Page 23**

**Q20**

**Have you bought or obtained fresh fruit and vegetables in the last 7 days? (excluding frozen and tinned produce)**

*One answer only*

Single-choice question

| 1 | Yes |
| --- | --- |
| 2 | No |

**Redirection case :**

**CASE 1**

If option **“1. Yes”** is ticked in Q20

→ The participant is then redirected to Q21

**CASE 2**

If option **“2. No”** is ticked in Q20

→ The participant is then redirected to T4

**ELSE**

→ The participant is then redirected to Q21

**Page 24**

**Q21**

**Please provide a list of fresh fruit and vegetables (excluding frozen and tinned produce) that you have purchased or obtained over the last 7 days.**

*Open-ended response*

Extensive text

_____________________
_____________________
_____________________

→ Next step for the participant : Q22

**Page 25**

**Q22**

**Vegetables (excluding frozen and tinned): From this list, what proportion of vegetables do you estimate you have thrown away in the last 7 days (excluding peelings)?**

*One answer only*

Single-choice question

| 1 | None |
| --- | --- |
| 2 | Less than one tenth (1/10) |
| 3 | Between one tenth (1/10) and a quarter (¼) |
| 4 | Between a quarter (¼) and half (½) |
| 5 | More than half (½) |

**Redirection case :**

**CASE 1**

If the option **“1. None”** is ticked in Q22

→ The participant is then redirected to Q24

**ESLE**

→ The participant is redirected toQ23

**Page 26**

**Q23**

**Give at most 3 examples of vegetables that you have thrown away, either whole or in part, in the last 7 days.**

*Open-ended response*

Extensive text

_____________________
_____________________
_____________________

→ Next step for the participant : Q24

**Page 27**

**Q24**

**Vegetables (excluding frozen and tinned): From this list, what proportion of fruit do you estimate you have thrown away in the last 7 days (excluding peelings)?**

*One answer only*

Single-choice question

| 1 | None |
| --- | --- |
| 2 | Less than one tenth (1/10) |
| 3 | Between one tenth (1/10) and a quarter (¼) |
| 4 | Between a quarter (¼) and half (½) |
| 5 | More than half (½) |

**Redirection case :**

**CASE 1**

If the option **“1. None”** is ticked in Q24

→ The participant is then redirected to T4

**ELSE**

→ The participant is redirected to Q25

**Page 28**

**Q25**

**Give at most 3 examples of fruit that you have thrown away, either whole or in part, in the last 7 days.**

*Open-ended response*

Extensive text

_____________________
_____________________
_____________________

→ Next step for the participant : T4

**S5 - Section 5**

**Page 29 - Transition**

**T4**

We would now like to hear your views on food and the environment.

→ Next step for the participant : Q26

**Page 30**

**Q26**

**Your attitude towards the environment in general**

*For each of the following statements, please indicate your level of agreement.*

Single-choice table

With lines in random order

|  |  | **Strongly disagree** | **Disagree** | **Neither agree nor disagree** | **Agree** | **Strongly agree** |
| --- | --- | --- | --- | --- | --- | --- |
| Q26_1 | I am very concerned about the environment | 1 | 2 | 3 | 4 | 5 |
| Q26_2 | Humans are severely damaging the environment | 1 | 2 | 3 | 4 | 5 |
| Q26_3 | I would be willing to reduce my consumption in general to help protect the environment | 1 | 2 | 3 | 4 | 5 |
| Q26_4 | Major policy changes are needed to protect the natural environment | 1 | 2 | 3 | 4 | 5 |
| Q26_5 | Major social changes are needed to protect the natural environment | 1 | 2 | 3 | 4 | 5 |
| Q26_6 | Anti-pollution laws should be enforced more strictly | 1 | 2 | 3 | 4 | 5 |

→ Next step for the participant : Q27

**Page 31**

**Q27**

**Your opinion on food waste**

*For each of the following statements, please indicate your level of agreement.*

Single-choice table

With lines in random order

|  |  | **Strongly disagree** | **Disagree** | **Neither agree nor disagree** | **Agree** | **Strongly agree** |
| --- | --- | --- | --- | --- | --- | --- |
| Q27_1 | I would be ashamed to waste food in front of someone | 1 | 2 | 3 | 4 | 5 |
| Q27_2 | It really upsets me to see someone throw away food that is still edible | 1 | 2 | 3 | 4 | 5 |
| Q27_3 | Managing food properly to minimise waste is – as far as I'm concerned – a priority | 1 | 2 | 3 | 4 | 5 |
| Q27_4 | In my circle of friends, wasting food is frowned upon | 1 | 2 | 3 | 4 | 5 |
| Q27_5 | Food waste poses an ethical problem for me in relation to those who are hungry | 1 | 2 | 3 | 4 | 5 |

→ Next step for the participant : Q28

**Page 32**

**Q28**

**Your opinion on food waste (continued)**

*For each of the following statements, please indicate your level of agreement.*

Single-choice table

With lines in random order

|  |  | **Strongly disagree** | **Disagree** | **Neither agree nor disagree** | **Agree** | **Strongly agree** |
| --- | --- | --- | --- | --- | --- | --- |
| Q28_1 | Food waste poses problems for waste management in municipalities | 1 | 2 | 3 | 4 | 5 |
| Q28_2 | In France, food waste is now a major problem | 1 | 2 | 3 | 4 | 5 |
| Q28_3 | Food waste has truly harmful consequences for the planet | 1 | 2 | 3 | 4 | 5 |

→ Next step for the participant : Q29

**Page 33**

**Q29**

**To what extent do you find the following products or situations disgusting?**

Single-choice table

With lines in random order

|  |  | **Not disgusting at all** | **Slightly disgusting** | **Moderately disgusting** | **Very disgusting** | **Extremely disgusting** |
| --- | --- | --- | --- | --- | --- | --- |
| Q29_1 | Eating overripe fruit | 1 | 2 | 3 | 4 | 5 |
| Q29_2 | Eating a banana with black spots | 1 | 2 | 3 | 4 | 5 |
| Q29_3 | Eating fruit (e.g., apples and peaches) with pressure marks or bruises | 1 | 2 | 3 | 4 | 5 |
| Q29_4 | Eating apple slices that have turned brown when exposed to air | 1 | 2 | 3 | 4 | 5 |
| Q29_5 | Eating brown avocado flesh | 1 | 2 | 3 | 4 | 5 |
| Q29_6 | Eating an overripe cucumber that may already be soft | 1 | 2 | 3 | 4 | 5 |
| Q29_7 | Eating shriveled/dried radishes | 1 | 2 | 3 | 4 | 5 |
| Q29_8 | Eating salad that is no longer crisp | 1 | 2 | 3 | 4 | 5 |

→ Next step for the participant : Q30

**Page 34**

**Q30**

**To what extent do you find the following products or situations disgusting?**

Single-choice table

With lines in random order

|  |  | **Not disgusting at all** | **Slightly disgusting** | **Moderately disgusting** | **Very disgusting** | **Extremely disgusting** |
| --- | --- | --- | --- | --- | --- | --- |
| Q30_1 | Eat the non-mouldy part of a mouldy tomato | 1 | 2 | 3 | 4 | 5 |
| Q30_2 | Eating bread from which the mould has been removed | 1 | 2 | 3 | 4 | 5 |
| Q30_3 | Eating hard cheese from which the mould has been removed | 1 | 2 | 3 | 4 | 5 |
| Q30_4 | Eating marmalade from which the mould has been removed from the surface | 1 | 2 | 3 | 4 | 5 |

→ Next step for the participant : Q31

**Page 35**

**Q31**

**How often did you engage in these activities during your childhood? (before the age of 11)**

*one answer per line*

Single-choice table

With lines in random order

|  |  | **Never** | **Seldom** | **Sometimes** | **Often** | **Always** |
| --- | --- | --- | --- | --- | --- | --- |
| Q31_1 | picking flowers, fruit or vegetables from a garden (or vegetable garden) | 1 | 2 | 3 | 4 | 5 |
| Q31_2 | having planted trees, seeds or plants | 1 | 2 | 3 | 4 | 5 |
| Q31_3 | having taken care of indoor or outdoor plants | 1 | 2 | 3 | 4 | 5 |

→ Next step for the participant : Q32

**Page 36**

**Q32**

**Your attitude towards food**

*For each of the following statements, please indicate your level of agreement.*

Single-choice table

With lines in random order

|  |  | **Strongly disagree** | **Disagree** | **Neither agree nor disagree** | **Agree** | **Strongly agree** |
| --- | --- | --- | --- | --- | --- | --- |
| Q32_1 | When I was young, my parents always asked me to finish everything on my plate | 1 | 2 | 3 | 4 | 5 |
| Q32_2 | When I lived with my parents, we kept and ate all the leftovers | 1 | 2 | 3 | 4 | 5 |
| Q32_3 | When I lived with my parents, playing with food was not tolerated | 1 | 2 | 3 | 4 | 5 |

→ Next step for the participant : Q33

**Page 37**

**Q33**

**In order to obtain recommendations on preventing food waste (hygiene and storage), which means of communication do you use most?**

*Several answers are possible.*

Multiple-choice question

| 1 | Posters (in public places, supermarkets, etc.) |
| --- | --- |
| 2 | Website and blogs |
| 3 | Mobile applications (example: Too Good to Go) |
| 4 | Radio |
| 5 | Television |
| 6 | Reports from public bodies (e.g. ADEME, ANSES) |
| 7 | Social media (Facebook, Instagram, TikTok, etc.) |
| 8 | YouTube channels |
| 9 | None |
| 10 | Others  Q33_10 - Précision |

→ Next step for the participant : Q34

**S6 - Sociodemographic variables**

**Page 38**

**Q34**

**Gender: You are**

Single-choice question

| 1 | Male |
| --- | --- |
| 2 | Female |
| 3 | Other |

→ Next step for the participant : Q35

**Page 39**

**Q35**

**Age group: Which age group do you belong to?**

*One answer only*

Single-choice question

| 1 | Under 24 years old |
| --- | --- |
| 2 | Between 25 and 34 years old |
| 3 | Between 35 and 44 years old |
| 4 | Between 45 and 54 years old |
| 5 | Between 55 and 64 years old |
| 6 | 65 years old and over |

→ Next step for the participant : Q36

**Page 40**


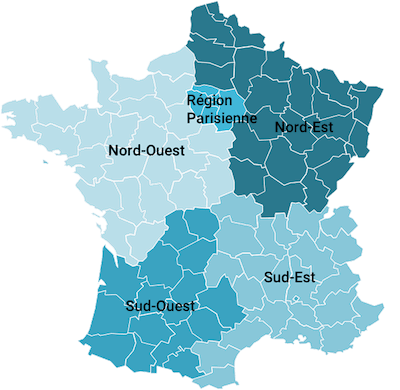


**Q36**

**Area of residence: In which area do you live?**

*One answer only*

Single-choice question

| 1 | Paris region |
| --- | --- |
| 2 | North-West |
| 3 | North-East |
| 4 | South-West |
| 5 | South-East |

→ Next step for the participant : Q37

**Page 41**

**Q37**

**Education Level: What is your education Level?**

*One answer only*

Single-choice question

| 1 | No diploma |
| --- | --- |
| 2 | Secondary School Certificate |
| 3 | Vocational qualification |
| 4 | Baccalaureate or Professional Certificate |
| 5 | Two-year or three-year degree |
| 6 | Master's degree or higher |

→ Next step for the participant : Q38

**Page 42**

**Q38**

**Your socio-professional category**

*One answer only*

Single-choice question

| 1 | Agricultural worker |
| --- | --- |
| 2 | Craftspeople, retailers and business owners |
| 3 | Executives, higher intellectual professions or Liberal professions |
| 4 | Technicians, supervisors and other intermediate professions |
| 5 | Employees |
| 6 | Labourers |
| 7 | Retired |
| 8 | Student |
| 9 | Unemployed |

→ Next step for the participant : Q39

**Page 43**

**Q39**

**Your accommodation: you live in:**

*One answer only*

Single-choice question

| 1 | Apartment |
| --- | --- |
| 2 | House |
| 3 | Other  Q39_3 - Précision |

→ Next step for the participant : Q40

**Page 44**

**Q40**

**Household structure: you live:**

*Plusieurs réponses possibles*

Single-choice question

| 1 | Single without child(ren) |
| --- | --- |
| 2 | Single with child(ren) |
| 3 | Couple without child(ren) |
| 4 | Couple with child(ren) |
| 5 | Other (with your parents, family members, friends, roommates, etc.) |

**Redirection case :**

**CAS 1**

If option **‘1. Single without child(ren)’** OR **‘3. Couple without child(ren)’** OR **‘5. Other (with parents, family members, friends, flatmates, etc.)’** is ticked in Q40

→ The participant is then redirected to Q42

**ESLE**

→ The participant is redirected to Q41

**Page 45**

**Q41**

**Number of children: specify the number of children you have**

*One answer only*

Single-choice question

| 1 | 1 |
| --- | --- |
| 2 | 2 |
| 3 | 3 |
| 4 | 4 |
| 5 | 5 |
| 6 | More than 5 |

→ Next step for the participant : Q42

**Page 46**

**Q42**

**Do you have any pets?**

*One answer only*

Single-choice question

| 1 | Yes |
| --- | --- |
| 2 | No |

→ Next step for the participant : Q43

**Page 47**

**Q43**

**Do you have access to compost at home (individual or communal)?**

*One answer only*

Single-choice question

| 1 | Yes |
| --- | --- |
| 2 | No |

→ Next step for the participant : Q44

**Page 48**

**Q44**

**Do you have a vegetable garden?**

*One answer only*

Single-choice question

| 1 | Yes |
| --- | --- |
| 2 | No |

→ Next step for the participant : Q45

**Page 49**

**Q45**

**Income: To enable us to classify your answers according to living standards, please indicate the net monthly income bracket of your household (all income combined).**

*One answer only*

Single-choice question

| 1 | Less than 1500 € |
| --- | --- |
| 2 | 1500 to 2000 € |
| 3 | 2000 to 2500 € |
| 4 | 2500 to 3000 € |
| 5 | 3000 to 3500 € |
| 6 | 3500 to 4000 € |
| 7 | More than 4000 € |
| 8 | Does not wish to respond |

→ Next step for the participant : Conclusion Profil validé 1

**Conclusion Profil validé 1**

**Thank you for taking the time to complete this questionnaire.**

Your answers have been recorded and will now be analysed.

We look forward to seeing you again in future surveys!

**Conclusion Disqualified profile 1**

**Thank you for answering these few questions.**

Unfortunately, we cannot invite you to continue this survey for the following reason:

- Your profile does not match what we are looking for in this survey.

We look forward to seeing you again in future surveys!
