# Supplementary material for: Participatory and multi-disciplinary science dataset and surveys for the assessment of the microbiological and behavioural factors influencing fresh fruits and vegetables' waste at home
Source: Data Brief. 2026 Jan 7;65:112434. doi: 10.1016/j.dib.2025.112434 (PMC12856149; doi:10.1016/j.dib.2025.112434)
Supplement: Supplementary file 1 [file mmc1.zip › Part1_FFV_waste_sampling_campaigns/Interviews/Doc2_Households_anonymised_interview_transcripts.docx]

**Anonymised transcripts of household interviews**

**Household F03, Interview 1**

00:00:00
*Représentant F03:* Donc il y a pas de raison, [euh], de pas répondre. Je veux dire-- voilà.

00:00:03
*Mme.2:* En tout cas un grand merci à vous. Donc, [euh], on va commencer dans un premier temps à- à jouer à une devinette. Donc--

00:00:15
*Représentant F03:* Est-ce que j'ai la bonne réponse ?

00:00:17
*Mme.2:* Bah, c'est moi qui doit avoir la bonne réponse [rires]. Donc en fin de compte, il va falloir que vous pensiez à un- à un lég-- à un fruit ou à un légume que vous aimez bien, [euh], que vous aimez vraiment bien-- enfin tout particulièrement, et vous devez pas me révéler son identité.

00:00:40
*Représentant F03:* D'accord.

00:00:40
*Mme.2:* Mais il va falloir que moi je devine ce que c'est, en fonction de votre description.

00:00:49
*Représentant F03:* D'accord.

00:00:49
*Mme.2:* Donc, [euh], ben quand vous avez pensé ou quand vous avez choisi votre légume ou votre fruit ben, on y va.

00:01:02
*Représentant F03:* Ok. Petit fruit.

00:01:02
*Mme.2:* C'est un petit fruit.

00:01:06
*Représentant F03:* Qui pousse à partir du mois de mai jusqu'à à peu près septembre.

00:01:10
*Mme.2:* Ouais.

00:01:10
*Représentant F03:* Il a la particularité d'être rouge. Il est-- il peut être acidulé ou très sucré. C'est-- il pousse en pleine terre. [Euh], une ville proche d'ici en est sa capitale. Et, [euh], on a fait des marmelade, des sirops, des liqueurs.

00:01:42
*Mme.2:* Des tartes ?

00:01:42
*Représentant F03:* Des tartes voilà, des glaces.

00:01:43
*Mme.2:* Ou rien du tout ?

00:01:43
*Représentant F03:* Ou rien du tout. Mangé comme ça nature, avec du sucre, du citron, de la chantilly.

00:01:43
*Mme.2:* Ou rien du tout ?

00:01:43
*Représentant F03:* Ou rien du tout. Voilà.

00:01:55
*Mme.2:* Ne serait-ce pas la fraise par exemple ?

00:01:57
*Représentant F03:* Mais si bien sûr. Mon fruit préféré.

00:01:57
*Mme.2:* C'est vrai ?

00:02:00
*Représentant F03:* J'en mange pratiquement deux kilos tous les trois jours.

00:02:03
*Mme.2:* Oui, c'est vrai que là je vois une barquette de- de fraises, et il me semble, effectivement, c'est- c'est des fraises qui viennent de pas très, très loin là.

00:02:10
*Représentant F03:* Voilà, hein. Pour ne pas citer le- la ville, hein.

00:02:11
*Mme.2:* Pour pas citer la ville, alors on citera pas, ça s'appelle Plougastel-Daoulas.

00:02:17
*Représentant F03:* Voilà, des fraises de pleine terre bien sûr.

00:02:18
*Mme.2:* Bio ?

00:02:18
*Représentant F03:* Bio, bien sûr.

00:02:23
*Mme.2:* Alors, vous avez dit, [euh], deux bien sûr pour pleine terre et bio.

00:02:28
*Représentant F03:* Oui.

00:02:29
*Mme.2:* C'est-à-dire?

00:02:30
*Représentant F03:* C'est-à-dire-- enfin après moi je suis maître crêpier donc, [euh], je travaille avec des tous petits producteurs, principalement bio bien sûr. Parce que ben pas de pesticides, c'est meilleur pour la terre et meilleur pour moi aussi donc-- mais, pour plein de monde. Donc pas de pesticides, quoi. Pas de produits étrangers dans mon assiette, on va dire ça comme ça.

00:02:53
*Mme.2:* D'accord. Donc, [euh], au-delà des- des- des fruits et des légumes, [euh], vous achetez principalement ou totalement local ?

00:03:02
*Représentant F03:* Oui, local.

00:03:02
*Mme.2:* Local.

00:03:02
*Représentant F03:* Local. Très local, moins de 50 kilomètres.

00:03:05
*Mme.2:* Moins de 50 kilomètres.

00:03:06
*Représentant F03:* Ça restreint, bien sûr. Ça restreint parce que bon, on pourrait se dire ben que je peux pas prendre certains fruits puisque ben, on trouve pas en local.

00:03:18
*Mme.2:* Ouais.

00:03:18
*Représentant F03:* Bon, il y a une erreur bien sûr. Les pamplemousses qui traînent dans la-- dans la corbeille de fruits, bien sûr.

00:03:23
*Mme.2:* Ah oui, j'ai pas vu.

00:03:24
*Représentant F03:* Ouais, mais ils sont dedans. Mais voilà, parce que ben-- parce que là j'ai acheté avec un autre verger et puis ben, elle avait des pamplemousses devant le nez donc j'ai été tenté. Je les ai pris quoi. Voilà. Mais autrement, c'est du très local quoi.

00:03:36
*Mme.2:* C'est du très local et toujours chez les petits producteurs, etcetera ?

00:03:39
*Représentant F03:* Toujours. Ouais, toujours.

00:03:40
*Mme.2:* Même pas de Coop Bio, etcetera quoi.

00:03:42
*Représentant F03:* Non. Parce qu'on est pas certain que tous les Coop Bio soit bien-- vraiment bio.

00:03:47
*Mme.2:* Ah ouais ?

00:03:48
*Représentant F03:* Ouais.

00:03:49
*Mme.2:* D'accord.

00:03:49
*Représentant F03:* Ouais. Voilà, donc du coup petits producteurs et même de fois des particuliers. Pour être sûr que bah, eux, pareil ils n'utilisent pas de produits pesticides, quoi. Souvent, quand ben je fais de la marmelade de mirabelle, la mirabelle c'est Lorraine, on est bien d'accord. Sauf que à Ploudalmézeau, il y a une dame qui a un mirabellier dans son jardin et qui, tous les trois ans, m'appelle [Prénom de l'interviewé] tu peux-- j'ai des mirabelles, la saison elle est finie, est-ce que tu les veux ? Bah je viens les chercher. Voilà.

00:04:24
*Mme.2:* Ça fait des confitures.

00:04:26
*Représentant F03:* Ah bah, extra quoi voilà. Nickel derrière, quoi. Parce que ben, justement, les fruits sont pas gorgés d'eau, sont gorgés de- de sucre, voilà. Donc moins de produits à rajouter aussi, [euh], dans la préparation, donc-- et puis bah des petits prix aussi parce que ben elle, elle me les vend mais vraiment au prix cadeau, quoi.

00:04:43
*Mme.2:* Ouais. Ouais, ouais.

00:04:48
*Représentant F03:* Mais ça lui fait plaisir.

00:04:48
*Mme.2:* Ouais, ouais, ouais, ouais.

00:04:49
*Représentant F03:* Bon bah on continue comme ça quoi. Pourquoi je vais aller les faire venir de Lorraine alors qu'à Ploudalmézeau, j'ai des-- j'ai des mirabelles, quoi ? Donc, du coup après pour faire plaisir, bah je marque sur mon bo-- sur le bocal, mirabelles de Ploudalmézeau. Donc c'est encore plus local aussi après, pour les vendre derrières aussi. Et, on peut expliquer aussi que ben, pourquoi on a été les chercher là, quoi ? Et donc, du coup la relation clientèle eh ben, elle est plus de confiance parce que bah on- on achète aussi auprès de petits quoi. Voilà. Moi mon but c'est pas de- de m'aggrandir et de faire le travail des grands. Mon but c'est de- de faire plaisir aux gens. C'est pas pareil.

00:05:35
*Mme.2:* Ouais, mais c'est un sacré enjeu.

00:05:37
*Représentant F03:* Ouais. Alors, j'en vis pas. Mais je m'en fous, ça reste du plaisir. Faire plaisir aux autres, c'est mon but.

00:05:52
*Mme.2:* Vos fraises, on va revenir sur vos fraises. [Euh], à quel moment vous les mangez ? Vous me dites que vous mangez beaucoup de fraises.

00:05:59
*Représentant F03:* Oui, elle va être-- là elles sont là mais pour ça il y en a plus hein, parce que je picore.

00:06:03
*Mme.2:* D'accord, donc la barquette, c'est à peu près 500 grammes.

00:06:05
*Représentant F03:* Par jour.

00:06:05
*Mme.2:* Par jour ?

00:06:07
*Représentant F03:* Par jour.

00:06:07
*Mme.2:* Ouais, c'est raisonnable hein.

00:06:10
*Représentant F03:* C'est raisonnable hein. Il n'y a que 32 kilocalories aux 100 grammes, c'est bon-- c'est rien. Et c'est picoré toute la journée quoi. J'ai déjà piqué dedans ce matin au petit dej. Mais j'avais un-- j'en ai d'autres au frigo. J'en ai-- j'ai-- certaines étaient un peu limite en consommation, donc je les ai mises avec du citron et du sucre. Elles sont au frigo, elles attendent. Elles seront mangées à mi-- ce soir.

00:06:33
*Mme.2:* Et donc, en fin de compte, dès le matin, au petit déjeuner, hop.

00:06:38
*Représentant F03:* Mais dans la nuit-- moi je travaille la nuit donc dans la nuit ma barquette elle passe.

00:06:42
*Mme.2:* Ouais, donc c'est-- à défaut de bonbons, c'est les fraises.

00:06:46
*Représentant F03:* Ouais. Mais comme ça peut être des kiwis, comme ça peut être n'importe quel fruit. Je veux dire, si le kiwi je le nettoie, je le dépiaute, je le mets en petits morceaux ben il va être picoré comme la fraise. C'est vraiment un-- ça remplace le bonbon, voilà. C'est- c'est vraiment-- j'ai besoin de ça. J'ai besoin de sucre et, [euh], ben plutôt que de manger du sucre, ben je mange du sucre naturel.

00:07:11
*Mme.2:* Et donc, c'est plutôt en guise de bonbon. Mais ça veut dire-- là ce midi vous allez pas manger a priori c'est- c'est--

00:07:15
*Représentant F03:* Ça y est j'ai mangé ma brioche c'est bon. Le midi, c'est qu'une brioche. Tout le temps.

00:07:15
*Mme.2:* Ouais enfin c'était même pas-- c'était un quart de brioche.

00:07:15
*Représentant F03:* Non j'avais déjà commencé avant que vous arriviez.

00:07:24
*Mme.2:* Ah d'accord.

00:07:24
*Représentant F03:* Non, non. Si, si. Je me suis dit je mange ma brioche, vite fait, avant qu'elle arrive.

00:07:31
*Mme.2:* D'accord.

00:07:31
*Représentant F03:* Et vous avez sonné, j'étais à la moitié, j'ai dit sac à main [rires]. Discretos, sac à main.

00:07:38
*Mme.2:* Ben vous voyez j'ai eu raison. J'ai eu raison d'arriver avec-- d'arriver à l'heure. Autrement vous auriez même pas pu manger votre brioche.

00:07:41
*Représentant F03:* Après si, je l'aurai mangé pendant quand même. Ça m'aurait pas gêné hein. Vous voyez, je l'ai fini devant vous donc ça m'aurait pas gêné de la commencer.

00:07:50
*Mme.2:* Donc, en fin de compte, [euh], ça fait-- le fruit ne fait pas partie du-- d'un repas.

00:07:56
*Représentant F03:* Ah si, si, si. Si parce qu'après-- si, si parce qu'après moi je vais-- quand je vais manger ce soir, mon repas de ce soir c'est chou fleur en vinai-- enfin en vinaigrette ou machin, [euh], il va y avoir de la viande de grison, il va y avoir-- comment, de la salade, il va y avoir du maïs, machin. Et en dessert, il y a mes fraises au sirop qui m'attendent dans le frigo, quoi.

00:08:16
*Mme.2:* Ah oui d'accord.

00:08:15
*Représentant F03:* Voilà. Donc c'est vraiment prévu dans le repas. Les- les fruits, c'est toute la journée, [en fait]. Je-- j'ai pas de base, je vais le manger le matin, s'il me faut une compote le matin, c'est pas à midi parce qu'il me faut un dessert. Non, je vais grignoter des fraises toute la journée et en plus, je vais en manger au repas. Ça fait vraiment partie de mon alimentation. Fruits et légumes, c'est allez 80 pourcent de mon alimentation, quoi. C'est pas juste au cas où, comme ça, machin. Ce matin j'étais en train de préparer des abricots pour les mettre en marmelade donc j'ai commencé à les nettoyer. Bah j'ai pas pu m'empêcher d'en manger quelques uns.

00:08:53
*Mme.2:* Ah bah de toute façon il faut vérifier si c'est bon.

00:08:56
*Représentant F03:* Voilà, donc là ils sont dans le sucre. Ils seront cuits ce soir. Certains sont partis là-dedans parce que ben ils étaient un peu limite. Vraiment limite-- ils étaient vraiment-- voilà.

00:08:59
*Mme.2:* Là-dedans c'était la poubelle.

00:08:59
*Représentant F03:* Oui, là-dedans c'était la poubelle. Parce que quand je les ai ouvert, ils étaient moisi à l'intérieur. Poubelle. Donc il y a eu trois fois ou quatre fois ouverture de boîte-- pour mettre dedans. Et puis bah, le reste est parti dans le seau hein, mais il y en a eu dans la bouche aussi.

00:09:13
*Mme.2:* Ouais, d'accord. Et donc, [euh], là on a parlé de-- des fruits. Les fruits c'est surtout, [euh], vous m'avez parlé de compote, donc c'est cuit.

00:09:28
*Représentant F03:* Ouais, mais c'est rare.

00:09:29
*Mme.2:* C'est rare ?

00:09:29
*Représentant F03:* Ouais, c'est rare. C'est rare, c'est souvent-- c'est à l'état brut qu'ils sont mangés.

00:09:37
*Mme.2:* Ouais, ouais, ouais ouais. Ouais, ouais, ouais, ouais. Oui, oui.

00:09:39
*Représentant F03:* Alors, ils vont être en compotes quand- quand ils sont bien mûrs, que c'est pas prévu de marmelade et je vais pas me faire une compote de kiwi, vous voyez-- de- de marmelade de kiwi. [Euh], du coup je ne-- soit je vais le manger comme ça parce que il est bon. Et si il est un peu trop mûr, il va partir en compote. Je vais rajouter un morceau de pomme dedans et compote mixée et puis c'est bon, quoi. Puisque la salade de fruits, s'il est un peu trop mûr ça sera un peu délicat à manger aussi. Mais compoté cuit, ça va aller vite. J'ai des gamelles qui font-- qui font en sorte que ça cuit très vite. Donc je peux vous dire que ça va vite.

00:10:19
*Mme.2:* Je veux bien vous croire. Je veux bien vous croire. [Euh], c'est vous qui faites tous vos achats en fruits et légumes ?

00:10:22
*Représentant F03:* Oui.

00:10:22
*Mme.2:* Et, [euh], comment ça se passe ? C'est au fur et à mesure de vos tournées, ou alors il y a un jour particulier pour acheter vos- vos fruits et légumes ?

00:10:37
*Représentant F03:* Oui, il y a des jours particuliers. Il y a le samedi matin, parce que je travaillais avec un-- une-- un- un petit magasin où elle fait principalement de la vente de fruits et de légumes. Et elle fait des tournées. Et donc comme je suis partenaire, moi je lui vends les crêpes du coup, pour ses clients eh bah, du coup je réserve mes fruits. Elle me les apporte sur le lieu de livraison et moi je récupère mes fruits et mes légumes.

00:10:57
*Mme.2:* D'accord.

00:10:57
*Représentant F03:* Voilà, après bah si j'ai pas-- si j'ai pas eu ma dose de fruits et légumes parce que ben X raison, j'ai oublié de les commander, je vais directement à son magasin les chercher.

00:11:08
*Mme.2:* D'accord. Donc ça, c'est plutôt le samedi.

00:11:11
*Représentant F03:* Ouais, le samedi et éventuellement le mercredi matin si j'ai pas eu mes-- si j'ai zappé sa commande le samedi matin, par exemple. Mais du coup, je vais les chercher à Saint-Vougay le mercredi matin.

00:11:21
*Mme.2:* D'accord et c'est constamment, [euh]--

00:11:24
*Représentant F03:* C'est régulier, c'est toutes les semaines.

00:11:25
*Mme.2:* C'est toutes les semaines. Et c'est que là.

00:11:26
*Représentant F03:* Non. Non, après bah comme j'ai dit j'ai été dans un autre verger, [euh], dans la semaine dernière, c'était jeudi der-- mercredi après-midi, j'étais du côté de Kérambléau, c'est du côté de Plouguin-- alors là c'est Saint-Vougay pour le mercredi et le samedi et autrement c'est du côté de Guipronvel, Coat-Méal, donc ça reste local, dans un verger où elle fait aussi de la vente en direct de fruits et de légumes que là j'avais vu sur internet. Donc du coup, j'ai commandé. J'ai demandé le mardi après-midi, mercredi après-midi j'ai été chercher les légumes et les fruits.

00:12:10
*Mme.2:* D'accord. Et c'est- c'est-- alors la- la personne du samedi ou du mercredi matin--

00:12:15
*Représentant F03:* C'est la même.

00:12:15
*Mme.2:* C'est toujours la même et autrement, c'est--

00:12:18
*Représentant F03:* C'est aléatoire quoi. Je veux dire quand je vois un truc qui est sur internet, j'appelle quoi.

00:12:22
*Mme.2:* D'accord. Ouais, ouais. Et quand vous faites vos approvisionnements, en fin de compte, vous avez pas des choix de fruits et légumes particuliers. Ce sera en fonction des--

00:12:32
*Représentant F03:* Des saisons.

00:12:33
*Mme.2:* Ouais, ça sera en fonction des saisons.

00:12:35
*Représentant F03:* C'est-à-dire que ben là si je veux de la fraise bon ben-- là si je veux faire des-- par exemple de la-- de la marmelade de fraise, ben ce sera que entre la période de mai et septembre. Si on me demande de la fraise fraîche à préparer au mois d'octobre, je vais leur dire non. Voilà, vous irez vous la manger-- vous prendrez ailleurs dans d'autres pays, mais moi je vais vous-- je peux vous fabriquer que pendant cette saison-là. Alors, si, après elle va être mise en confiture, entre guillemets. Donc j'ai un stock, entre guillemets, d'une année sur l'autre. Mais je la vends que entre mai et septembre. Je veux pas la vendre en dehors de cette période.

00:13:12
*Mme.2:* [Euh], en fraîche ?

00:13:13
*Représentant F03:* Non en-- quand je l'ai mise en confiture, je la mets-- je la vends plus après.

00:13:15
*Mme.2:* Vous la vendez qu'entre mai et septembre. C'est-à-dire durant la période de--

00:13:16
*Représentant F03:* Des fraises. La chieuse.

00:13:16
*Mme.2:* La période des fraises.

00:13:16
*Représentant F03:* Je suis une vraie chieuse.

00:13:16
*Mme.2:* D'accord, donc là les abricots-- comme la période des abricots, c'est beaucoup moins longtemps que les fraises, vous allez les vendre vos confitures de frais-- de-- d'abricots combien de temps ?

00:13:33
*Représentant F03:* Un mois. Pas plus. Un mois, deux mois, pas plus. Mais généralement c'est parti. J'achète que 10 kilos, hein. Je fais pas non plus la- la casse-pied. J'achète pas 100 kilos d'abricots. Là, j'ai acheté deux caisses de une kilos. Une fois nettoyé, ça va faire sept kilos. Ça fait à peu près 70 pots de 100 grammes. Et 70 pots en deux mois c'est vendu, hein. Mais j'en fais pas après. Je recommence pas une deuxième tournée. Je fais juste une tournée d'abricots. Les gens sont prévenus. Ils savent que ben, chez BREIZH Douceurs, il y a intérêt à se dire oui tout de suite. Parce qu'il y a pas après.

00:14:07
*Mme.2:* Et durant la saison ?

00:14:08
*Représentant F03:* Et durant la saison parce qu'après la marmelade d'abricots, je la vends pas en hiver. La mirabelle que j'ai eu l'année dernière, il y en a plus.

00:14:16
*Mme.2:* Bah de toute façon, vous pouvez plus la vendre parce que c'est plus la saison de la mirabelle.

00:14:20
*Représentant F03:* Oui, non mais j'aurais pu-- on- on-- je veux dire-- avoir fait, [euh], 100- 100 pots de mirabelles, ne pas avoir tout vendu l'année dernière et il me serait-- il aurait pu rester un stock de mirabelles en bocaux. Mais-- et envisager de la vendre. Mais comme je suis partie dans l'optique que moi je vends les produits que quand c'est la saison, ben je n'en vends plus. Voilà.

00:14:46
*Mme.2:* Oui, oui. Tout à fait.

00:14:48
*Représentant F03:* Même s'il m'en restait, je la vendrais pas. La chiante.

00:14:48
*Mme.2:* Non. Mais non, j'oserais pas dire ça.

00:14:52
*Représentant F03:* Parce que je veux-- je veux-- je veux qu'on-- que les gens comprennent qu'on peut pas avoir les fruits à n'importe quel moment du jour et de la nuit, ni dans l'année, parce que il y a des moments où le fruit arrive à maturité. Je m'en sers. Et je vends à ce moment-là. Je m'en sers et je les fabrique-- ce que je veux et je ne vends que pendant cette période-là.

00:15:13
*Mme.2:* Oui, mais vous pourriez très bien dire la confiture, ça se conserve. Et donc, je vendrai de la mirabelle en février ou en mars.

00:15:20
*Représentant F03:* Oui, mais moi je veux pas. Je veux que les gens comprennent que le produit il est meilleur quand on achète à maturité et donc qu'on le-- je le fabrique à maturité. En même temps, j'ai la particularité de ne mettre que très peu de sucre donc mes- mes gelées et marmelades ne se conservent pas longtemps.

00:15:36
*Mme.2:* D'accord.

00:15:37
*Représentant F03:* Aussi, parce que je veux pas dénaturer le produit. Voilà, je veux qu'on-- quand on mange une marmelade d'abricots ou quand on mange une marmelade de fraises, on ait le morceau de fraise et le goût de la fraise et non pas un goût de sucre aromatisé à la fraise ou aromatisé à la marmelade d'orange-- enfin ou à l'orange ou-- etcetera, etcetera quoi. Je veux qu'on mange un fruit. Que à l'ouverture du pot, on sente le produit et que quand on a mis la-- le pro-- le produit à la bouche, eh bah on ait l'impression d'avoir croqué dans un fruit frais. Donc, ça oblige à ne pas mettre d'agar-agar-- bah déjà je suis allergique, [euh], de pas mettre trop de sucre. Parce que bon, le sucre est un conservateur de longue durée. Mais moi, en mettant que 30 pour cent de sucre, je diminue la- la conservation de ma marmelade et donc, du coup bah je m'oblige à ne la vendre que pour-- pendant cette période-là.

00:16:31
*Représentant F03:* Maintenant, la personne qui me l'achète, par exemple, là en juin, les abricots et qui veut la manger qu'en décembre, c'est à ses risques et périls. Moi, je vais mettre une DLC de allez, maximum six mois. En décembre, ça va le faire. Il va la manger en janvier, il va me dire ouais, mais madame [nom de la Représentante], votre marmelade était périmée au mois de décembre-- au mois de janvier. Normal, je vous avais dit six mois.

00:16:53
*Représentant F03:* Voilà. Il peut la manger plus tard, il peut la conserver et la manger plus tard. Il n'y a aucun souci. Mais s'il la mange en février et qu'elle est moisie, ma DLC n'était que de six mois. Je me lave les papattes. Vous voyez ? C'est aussi ça. Il faut que les gens comprennent que on-- moi je suis pas un industriel. Je suis un petit producteur. Donc forcément, j'ai des contraintes, [euh], restrictives au niveau de-- les-- la direction sanitaire qui m'oblige à ne mettre que six mois sur mes marmelades parce que j'ai pris moi, l'initiative de descendre le taux de sucre dans mes marmelades pour justement que les gens, quand ils ouvrent, eh bah il mangent un fruit. Voilà, ils mangent pas un morceau de sucre. Donc forcément, il y a des contraintes, [euh], temporaires.

00:17:45
*Mme.2:* Oui, tout à fait. Et pour votre usage personnel, on va parler des légumes. D'accord ? Vous faites pas de marmelade de légumes ?

00:17:51
*Représentant F03:* Non pas encore. Je fais du confit d'oignon de temps en temps mais c'est juste à usage-- quand j'ai des repas de-- quand j'ai des repas de-- à domicile. Voilà.

00:18:02
*Mme.2:* D'accord. [Euh], donc, [euh], vous faites les courses-- vous devez faire les courses. Comment vous faites les courses ? Vous avez une liste de courses et vous--

00:18:09
*Représentant F03:* J'ai ma liste de courses, ouais. D'abord je prépare ma recette, c'est-à-dire je sais exactement combien de kilo de produits, de légumes j'ai besoin. Là pour-- bah tout simple, dimanche j'ai un repas. J'ai un repas, j'ai un retour de noces. Donc il y a 10 personnes. Je sais-- donc j'ai préparé un devis. La personne a accepté le devis et, [euh], ben me dit bah voilà il y a deux a-- il y a deux enfants et huit adultes, il y a cinq forestières et trois provençales. Donc du coup provençales, ça oblige donc tomates, courgettes, oignons, poivrons. Que des légumes de saison qu'on trouve à ce moment-ci. Les champignons, pas de souci pour la forestière. De la crème, poulet, point. Les légumes, du coup je sais combien ils sont. Huit. Huit adultes, mais il y a que trois provençales. Ben je vais faire en sorte d'acheter que là-- ce que j'ai besoin.

00:19:00
*Représentant F03:* Après, si jamais je vais garnir la crêpe et que si jamais il y en reste de-- des-- de la marmela-- de la préparation, la préparation appartient à la personne. Elle a acheté le- le- le repas, elle a acheté mon travail, les ingrédients et mon déplacement. Tout ce qui reste lui appartient. Elle conserve comme elle veut. Voilà.

00:19:18
*Mme.2:* D'accord.

00:19:20
*Représentant F03:* Moi, j'achète la quantité nécessaire à-- à un bon mangeur pour mettre sur une galette. Donc, je vais acheter admettons 500 grammes de- de tomates, 500 grammes d'oignons, 500 grammes de- de courgettes et autant de poivrons. Je vais faire la préparation, mettre en-- au frigo à conserver samedi soir. Dimanche matin, je ramène, je fais chauffer, je mets sur la galette, terminé.

00:19:44
*Mme.2:* D'accord. Et pour votre usage personnel, co-- comment ça se passe ? Vous--

00:19:50
*Représentant F03:* Pareil. J'achète les fruits et les légumes au fur et à mesure que j'ai besoin sauf les pommes de terre, les oignons que j'achète toujours-- que j'ai toujours un peu d'avance au cas où.

00:19:57
*Mme.2:* D'accord. Et donc, vous achetez en fonction de ce que vous avez besoin, mais vous-- ça veut dire que vous ouvrez les placards, vous ouvrez le frigo, vous-- voilà, vous faites votre liste et vous vous cantonnez à cette liste-là ?

00:20:10
*Représentant F03:* Oui.

00:20:11
*Mme.2:* Et si, par exemple, d'aventure-- complètement question idiote, d'aventure vous n'aviez pas mis sur la liste les fameuses fraises, que vous voyez des fraises, comme elle est pas sur la liste, vous allez pas acheter ?

00:20:23
*Représentant F03:* Si j'achèterai quand même.

00:20:25
*Mme.2:* Ah quand même. Donc, il y a un petit peu d'achats aussi envie.

00:20:28
*Représentant F03:* Il y a de l'achat-- il y a de l'achat de-- d'impulsion, bien sûr. Ouais.

00:20:32
*Mme.2:* Ouais d'accord.

00:20:32
*Représentant F03:* C'est clair. La fraise, c'est mon fruit préféré. Je boude quand il y en a pas quoi. Mais après j'irais pas acheter espagnol et compagnie quoi. J'attendrais qu'elles soient-- que la saison--

00:20:42
*Mme.2:* Que ça soit local. Et puis de pleine terre.

00:20:45
*Représentant F03:* Et de pleine terre, c'est important. Pas sous tunnel, de pleine terre. Je veux pas de- de- de substrat qui- qui fait pousser la plante. Voilà.

00:20:52
*Mme.2:* C'est vraiment du naturel. C'est ça ?

00:20:53
*Représentant F03:* Il y a pas mieux. Comme [inaudible 00:20:58], il faut attendre que le soleil soit là, que elle soit-- que ce soit-- que le soleil colore la fraise, donne son sucre et tout, donc--

00:21:06
*Mme.2:* Et, [euh], même pour vous, c'est que des petits producteurs. C'est pas des, [euh]--

00:21:13
*Représentant F03:* Je vais pas à Lidl, je vais pas à Leclerc. Je-- je vais au Leclerc juste pour le papier toilette et éventuellement produit vaisselle.

00:21:19
*Mme.2:* C'est pas du fruits et légumes ça.

00:21:20
*Représentant F03:* Non mais voilà, je veux dire-- hein-- mais--

00:21:21
*Mme.2:* Et c'est, [euh]-- à- à titre perso, c'est aussi dans le samedi ou le mercredi ou alors c'est plutôt-- ça dépend des--

00:21:34
*Représentant F03:* C'est souvent le samedi, mercredi parce que c'est les deux matinées où je suis à peu près disponible pour faire des courses.

00:21:37
*Mme.2:* D'accord. Donc vous allez faire au niveau professionnel et au niveau personnel.

00:21:42
*Représentant F03:* Oui, oui.

00:21:43
*Mme.2:* D'accord, ok.

00:21:45
*Représentant F03:* Ouais parce qu'après le lundi, c'est une journée blindée généralement, mardi matin je suis en déplacement-- je suis en livraison, l'après-midi je dors.

00:21:52
*Mme.2:* Ça vous arrive ?

00:21:52
*Représentant F03:* Mm. Mercredi je suis en production, mais mercredi matin je suis en livré-- je suis dans mes courses. Mercredi après-midi c'est- c'est production, jeudi c'est livraison. Jeudi après-midi, je dors à nouveau ou je prépare d'autres préparation, voilà. Et vendredi ben c'est-- c'est blindé de préparation.

00:22:17
*Mme.2:* Bon, [euh], alors on a parlé des fruits et-- on a parlé des fraises. On a parlé des abricots. [Euh], au fait, les pamplemousses, vous les avez acheté où ?

00:22:27
*Représentant F03:* Bah du coup à Kérambléau à--

00:22:28
*Mme.2:* Ah oui d'accord. Ok. Chez votre partenaire? On va dire ça comme ça.

00:22:33
*Représentant F03:* Oui, partenaire.

00:22:36
*Mme.2:* Est-ce qu'il y a des-- les kiwis ?

00:22:38
*Représentant F03:* Avec elle aussi.

00:22:40
*Mme.2:* Avec elle ?

00:22:40
*Représentant F03:* Ouais, il y a eu donc les-- bon après j'avais acheté le melon avec elle.

00:22:44
*Mme.2:* Melon, quand ?

00:22:45
*Représentant F03:* Melon là que j'ai mangé.

00:22:45
*Mme.2:* Ah ouais ?

00:22:45
*Représentant F03:* Ouais, mais bon on n'a pas aimé. Ils étaient pas suffisamment sucrés, donc j'en reprendrai pas. J'attendrai encore un peu de temps. Voilà. Donc j'ai acheté les abricots avec elle parce que justement elle avait mis qu'elle vendait les abricots à confiture. Justement, voilà. Donc je les ai acheté le jeudi de la semaine-- mercredi après-midi de la semaine dernière, mais je les ai fait que là, ce matin. Et encore, j'ai pas fini de les nettoyer. Mais ils sont bien restés. [Euh], du coup j'avais acheté les kiwis, les pamplemousses, les abricots, une boîte de radis. À deux, c'est vite fait une boîte de radis. Il en reste un tout petit peu au frigo. [Euh], et une salade que j'avais acheté avec elle aussi qui est encore au frigo. Voilà, qui sont conservés au frigo.

00:23:29
*Mme.2:* C'est vous qui faites toutes- toutes les courses et c'est vous qui décidez de ce qu'on va manger ?

00:23:35
*Représentant F03:* Ah non.

00:23:35
*Mme.2:* Ah.

00:23:35
*Représentant F03:* Alors, c'est pas moi qui décide. Enfin, en grande-- on va dire 80 pourcent c'est- c'est moi qui décide. Mais j'ai obligé mon mari à faire des menus.

00:23:48
*Mme.2:* Oh là.

00:23:50
*Représentant F03:* Et à me donner ses idées pour manger.

00:23:52
*Mme.2:* Je pourrais faire une photo des- des fameux menus tout à l'heure ?

00:23:54
*Représentant F03:* Ouais, ouais bien sûr. Pas de souci.

00:23:55
*Mme.2:* Ah ouais, il y a une liste.

00:23:55
*Représentant F03:* Non, non il y a pas de souci hein. Voilà. Parce que ça m'énerve, tous les jours depuis un an-- on est marié depuis quatre ans et quoi on va dire jusqu'à il y a trois mois, on mange quoi ce soir ? Fameuse question qui m'énerve bien. Moi qui aime bien tout pré-- planifier. Mes semaines sont planifiées presque des fois un mois à l'avance hein. Presque à une demi-heure près. Et lui, c'est toujours à l'arrache, oh bah on a qu'à manger ce qui traîne dans le frigo. Mais des fois il reste rien dans le frigo donc-- tout est au congèle. Et du coup on doit se servir du micro-ondes que j'évite hein. Je- je m'en sers, mais j'évite de m'en servir. Donc du coup, bah je râle parce que ben on mange à pas d'heure, quoi. Du coup, ma planification des repas. Donc planification des repas, il faut que je montre que-- parce que deuxième liste. J'ai donc pris l'habitude de noter ce que j'ai dans mes placards et mon congèle-- et mes congélateurs.

00:25:02
*Mme.2:* Ah oui, et là encore il y a des listes.

00:25:05
*Représentant F03:* Voilà, pareil des listes. Et donc quand on fait les menus, on-- d'abord on se sert de ça.

00:25:09
*Mme.2:* D'accord, on se sert de ce qu'il y a déjà en stock.

00:25:12
*Représentant F03:* Et si tenté qu'on voulait quelque chose de différent, à ce moment-là on fait une liste de courses et on achète que ce qui a besoin.

00:25:21
*Mme.2:* D'accord.

00:25:21
*Représentant F03:* Ce qui permet aussi de gérer le budget courses, de gérer les stocks dans la maison, de pas en acheter 36 000 machins, on est que deux, hein. Donc-- et puis, bah voilà-- et puis ben donc congélateur, placards, tout est noté.

00:25:34
*Mme.2:* D'accord.

00:25:35
*Représentant F03:* Et après, on fait les menus. Et après on s'y tient. Et si on s'y tient pas, bah c'est reporté sur la semaine suivante.

00:25:44
*Mme.2:* D'accord.

00:25:44
*Représentant F03:* Au cas où.

00:25:45
*Mme.2:* Bah c'est sérieux hein. Et ça, c'est valable pour les fruits et pour les légumes ?

00:25:51
*Représentant F03:* Mm.

00:25:51
*Mme.2:* D'accord.

00:25:52
*Représentant F03:* Mais aussi vous voyez, tous les placards donc ça veut dire que c'est caché les machins, tout est-- tout est-- tout est planifié quoi.

00:25:53
*Mme.2:* Tout est effectivement écrit, etcetera, etcetera.

00:25:53
*Représentant F03:* Ouais. Et c'est barré. Si je décide de faire par exemple, [euh], ce soir c'est pas noté mais-- parce que là il est daté du 24 parce que voilà il m'a pris la tête, il m'a dit oh tu fais chier avec tes menus, donc on a stoppé. Mais dès que je note dessus, je barre ici.

00:26:17
*Mme.2:* Oui, pour avoir les listes.

00:26:17
*Représentant F03:* Il est planifié.

00:26:17
*Mme.2:* Pour avoir les listes à jour quoi. Ouais, ouais, ouais. Ouais, ouais. [Euh], est-ce qu'il y a des-- alors on a beaucoup parlé de fruits, mais en légumes est-ce qu'il y a des--beaucoup des-- enfin des légumes que vous consommez beaucoup ?

00:26:32
*Représentant F03:* Oui.

00:26:33
*Mme.2:* Et très régulièrement.

00:26:34
*Représentant F03:* Oui, le chou fleur.

00:26:34
*Mme.2:* Chou fleur.

00:26:34
*Représentant F03:* Brocolis. Des artichauts, [euh], ben des oignons, des pommes de terre, [euh], des pois chiches, haricots rouges, des haricots en-- tous les haricots, [en fait], haricots verts, les beurres, [euh], les petits pois enfin, les flageolets aussi. [En fait], il y a juste que les topinambours que j'aime pas. Ça sera plus simple à dire. Topinambours, [euh], la poire de terre que j'aime pas non plus.

00:27:00
*Mme.2:* La poire de terre ?

00:27:03
*Représentant F03:* Oui, poire de terre. J'aime pas.

00:27:05
*Mme.2:* C'est quoi une poire de terre ?

00:27:06
*Représentant F03:* [Euh], c'est un légume en forme de poire mais qui- qui pousse vraiment dans la terre. C'est vraiment un légume-- c'est un peu, [euh], croquant et on a beau la cuire, la cuire, la cuire, ça reste croquant, ça m'énerve. Donc du coup j'aime pas. Ça a un goût légèrement sucré.

00:27:18
*Mme.2:* Donc il faut que ce soit mou un peu ?

00:27:23
*Représentant F03:* Ben disons que il faut que ça ait du goût. La poire de terre n'en a pas forcément.

00:27:23
*Mme.2:* D'accord, donc il faut que les- les- les fr-- les légumes aient du goût.

00:27:24
*Représentant F03:* Oui. Moi si une poire de terre elle a pas de goût, [euh], c'est pas la peine quoi. Si c'est un légume qui-- vous avez l'impression de manger de la flotte, [euh], bah autant boire un verre d'eau quoi, je veux dire.

00:27:26
*Mme.2:* Oui.

00:27:37
*Représentant F03:* Ça a beau être une-- bio et machin, si ça a pas de goût, ça a pas de goût, quoi. Du coup, il dégage de la liste quoi. Donc topinambour j'aime pas. [Euh], les scorsonères j'aime pas non plus, [euh], bah la poire de terre j'aime pas. Et autrement tout le reste j'aime, quoi.

00:27:38
*Mme.2:* D'accord. Et là, quand vous dites actuellement chou fleur, brocolis, oignons, haricots verts, petits pois. Mais les haricots verts et les petits pois c'est pas encore la saison.

00:28:00
*Représentant F03:* Ils sont pas encore là. Mais c'est des-- vous m'avez demandé qu'est-ce que j'aimais comme légumes.

00:28:02
*Mme.2:* D'accord. Ok.

00:28:02
*Représentant F03:* Sans préciser la saison donc pour l'instant, c'est pas de saison. Chou fleur c'est encore-- ça commence. Donc du coup chou fleur, il y en a déjà. J'en ai mangé hier soir, il en reste un peu au frigo donc voilà.

00:28:06
*Mme.2:* D'accord. Donc actuellement, c'est plutôt chou fleur, brocolis.

00:28:15
*Représentant F03:* Tomates cerises, tomates voilà, aussi. Il y a un concombre qui traîne là-bas, les courgettes dans la cuisi-- dans la chambre aussi. Parce que j'ai un-- vous avez vu les fruits-- les fruits sont là. Et il y a un stockage là-bas dans la-- dans mon économat puisque ma-- j'ai une pièce, une chambre, [en fait], qui a été aménagée en économat et c'est aussi ma réserve de fruits et de légumes là-bas.

00:28:21
*Mme.2:* D'accord.

00:28:37
*Représentant F03:* Vous pourrez voir tout à l'heure.

00:28:38
*Mme.2:* Cool, merci. Et est-ce qu'il y a des-- à part la poire de terre que vous ne mangez que très rarement, voire jamais, [euh], en-- vous m'avez cité quelques légumes--

00:28:51
*Représentant F03:* Patate douce que j'aime pas non plus, voilà.

00:28:54
*Mme.2:* C'est pas très local patate douce en plus.

00:28:56
*Représentant F03:* Non, puis j'aime pas. Il y en a hein. Si, si, il y en a.

00:28:56
*Mme.2:* Ah, il y en a ?

00:28:58
*Représentant F03:* Il y en a. Il y en a, à Poulesquet il y en a. Il y a un producteur de patates douces.

00:29:05
*Mme.2:* D'accord mais vous aimez pas.

00:29:05
*Représentant F03:* Non, j'aime pas.

00:29:05
*Mme.2:* D'accord. Et, [euh], et autrement il faut que ça soit le goût.

00:29:10
*Représentant F03:* Il faut que ça ait du goût. Si on vous fait à-- on vous met les yeux-- un bandeau sur les yeux et qu'on vous fait goûter un produit et que vous êtes incapable de repérer le produit, bah quel intérêt de l'acheter, quoi ? Parce que du coup, vous allez pas savoir avec quoi l'associer derrière, ni apprécier ce que vous allez manger dans votre assiette si vraiment ce produit n'a pas de goût.

00:29:35
*Mme.2:* Ouais, ouais.

00:29:36
*Représentant F03:* Maintenant, si on moi-- me ferme les yeux et puis que tiens, on a dit tiens on te donne ça, goûte et que là je retrouve par exemple le goût d'un abricot ou que je regoûte-- j'ai un bout de- de chou fleur dans la bouche et je suis capable de récupérer le goût du chou fleur. Ok, j'achète, je prends, je mange. Et je mangerai avec d'autant plus de plaisir parce que je retrouverai le goût du produit que-- et ça sera pas qu'un nom sur un bout de papier, quoi.

00:29:59
*Mme.2:* Ouais, ouais. Ouais, ouais, ouais. Ouais, ouais.

00:29:59
*Représentant F03:* Moi mes papilles elles ont-- elles sont-- exacerbées tous les jours. Dans la-- elles sont-- tous les jours elles sont en travail, donc à un moment donné ou à un autre si moi on me donne un produit qui a pas de goût, ben non. Ben non, je travaille pas. Je peux pas.

00:30:16
*Mme.2:* Ouais, ouais. Ouais. [Euh], quand vous faites, [euh], les courses, vous revenez et vos- vos fruits, qu'est ce que vous en faites ? Est-ce que-- où est ce que vous les rangez ? [Euh]--

00:30:30
*Représentant F03:* Bah, les fraises, à disposition.

00:30:30
*Mme.2:* Ouais. Ça j'ai bien compris, ouais. Mais, en fin de compte, question très bête, vos fraises, c'est 500 grammes par jour minimum. Hein, c'est ça hein ?

00:30:38
*Représentant F03:* Mm.

00:30:38
*Mme.2:* Vous allez vous fournir, le lundi et le mercredi. Ça veut dire que vous prenez votre stock. D'accord, ok.

00:30:42
*Représentant F03:* Je prends mon stock avec la dame. Avec [Prénom du fournisseur].

00:30:42
*Mme.2:* Et monsieur est aussi un gourmand de fraise, aussi ?

00:30:42
*Représentant F03:* Moins. Moins. Il est beaucoup moins fruits et moins légumes que moi. Lui, c'est les 20 pourcent qui restent. Moi, je mange 80 pourcent de la production-- enfin de-- des fruits et des légumes qui rentrent au domicile. Lui, il va manger les 20 pourcent qui restent.

00:31:14
*Mme.2:* D'accord.

00:31:14
*Représentant F03:* À nous deux ça fait 100 pourcent mais-- voilà. Mais, [euh], parce que je le force à manger des fruits et des légumes. Parce que c'est très bon pour la santé.

00:31:15
*Mme.2:* D'accord.

00:31:23
*Représentant F03:* Mais lui ça serait riz, pâtes, pommes de terre quoi voilà. Et on tourne à ça quoi [inaudible 00:31:31].

00:31:26
*Mme.2:* Donc on revient-- on revient aux- aux fruits, vous arrivez avec vos fruits. Les fraises on les laisse sur la table là--

00:31:26
*Représentant F03:* Alors une barquette là et le reste dans l'économat parce que c'est la pièce la plus froide de l'appartement. Voilà pourquoi. Elle est stockage au noir, hein, et, [euh], donc souvent je vérifie ce que j'ai d'abord acheté. Les fruits, la plupart sont mis dans la corbeille de fruits. Si vraiment j'ai pris, admettons trois kilos de pommes, bah le-- je mets ce que j'ai envie dans la sem-- qui sera mangé dans la semaine, le reste va aller dans l'économat.

00:32:06
*Mme.2:* D'accord.

00:32:06
*Représentant F03:* Et après, bah les fruits-- les légumes, c'est pareil. C'est placé dans l'économat, [euh], en attendant l'utilisation.

00:32:15
*Mme.2:* Et dans le frigo ?

00:32:15
*Représentant F03:* C'est rare.

00:32:15
*Mme.2:* C'est rare.

00:32:16
*Représentant F03:* La salade va être préparée de suite, c'est-à-dire qu'elle va être déposée dans le bac, [euh], dans le bac à plonge. Je vais fermer, je vais mettre un peu de vinaigre, je vais la nett-- la nettoyer de suite. Je vais pas la couper au couteau, je vais la nettoyer à la main pour éviter qu'elle toxide. Et, [euh], du coup, je vais les-- la lai-- la laisser aller, cinq minutes, le temps que-- de tuer le maximum de microbes avec le- le vinaigre et après elle est essorée et ré-- réfrigérée. Les radis vont être nettoyés, ils vont être équeutés et enlevé la petite racine, pareil. Et puis, les feuilles de radis si elles sont très bonnes, si elles sont de bonne qualité, elles vont être lavées et stockées au congélateur, elles serviront à faire des soupes ou un pesto. L'élastique va être gardé aussi [rires]. Et après ben, du coup les radis vont être pareil en même temps que la salade à nettoyer. Et puis, bah ils vont être stockés au frais et dans un bac rempli d'eau pour pouvoir les conserver et garder, [euh], leur croquant.

00:33:17
*Mme.2:* D'accord, [euh]--

00:33:17
*Représentant F03:* Tout ce qui a besoin d'a-- d'avoir un peu de manutention va être fait d'abord et tous les con-- les- les- les- les fru-- les légumes et les fruits qui peuvent se stocker sans être, [euh], périssables dans- dans la semaine vont rester dans l'économat.

00:33:31
*Mme.2:* D'accord. Mais la salade, si vous la préparez tout de suite, vous allez pas pouvoir la conserver pendant une semaine.

00:33:37
*Représentant F03:* Si. Il faut hyper bien l'essorer, vous la conservez au frigo.

00:33:46
*Mme.2:* Est-ce que, [euh], est-ce que selon vous, c'est un stockage, un or-- une organisation qui est idéale ?

00:33:52
*Représentant F03:* Bah pour moi ça me-- pour moi, ça me convient.

00:33:54
*Mme.2:* Ouais, ok.

00:33:56
*Représentant F03:* Après peut-être que pour d'autres, ça serait pas pareil mais pour moi, oui, ça me convient. Comme ça ma salade est à disposition, elle est lavée, elle me sert toute la semaine. Mes radis, c'est pareil.

00:34:07
*Mme.2:* Et est-ce qu'il vous arrive de nettoyer ces- ces lieux de rangement ?

00:34:11
*Représentant F03:* Oui, bien sûr.

00:34:12
*Mme.2:* Et quelle est la fréquence ?

00:34:15
*Représentant F03:* Tous les jours.

00:34:15
*Mme.2:* Tous les jours ?

00:34:16
*Représentant F03:* Bah, après moi je travaille dans ma cuisine donc, voilà. Le bac, il sert aussi bien à nettoyer les légumes qu'à faire la vaisselle. Je suis coincée, je suis obligée de les laver. Même si je veux pas, je suis obligée de le faire. Vous voyez il y a mes casseroles de caramel qui sont là. J'ai fait du caramel ce matin, j'ai pas fait ma plonge. Donc là si j'ai décidé de laver des légumes, bah faut d'abord faire la plonge. Après, je désinfecte mon évier. Et après je mets mes légumes.

00:34:40
*Mme.2:* D'accord. Et la corbeille de fruits ?

00:34:42
*Représentant F03:* Elle est généralement sur la table. Voilà. Elle est nettoyée à chaque fois qu'on remet des fruits et des légumes dedans.

00:34:47
*Mme.2:* D'accord. Et donc, c'est au minimum une fois par semaine ?

00:34:50
*Représentant F03:* Une fois par semaine, ouais.

00:34:51
*Mme.2:* Et le lieu ?

00:34:53
*Représentant F03:* L'économat, c'est pareil. Vous pouvez venir voir.

00:34:53
*Mme.2:* [Euh], bah on fera-- ouais, ouais, ouais et puis je ferai les photos après à ce moment-là, hein.

00:34:54
*Représentant F03:* L'économat, c'est pareil c'est à chaque fois que-- je mets mes légumes et mes fruits et puis--

00:35:00
*Mme.2:* Ah oui, en fin de compte c'est- c'est une chambre.

00:35:00
*Représentant F03:* Ah oui c'est une chambre transformée oui, oui.

00:35:01
*Mme.2:* Qui a été transformée en- en étagère.

00:35:00
*Représentant F03:* Voilà. Donc voilà. Donc l'économat. Donc là il y a mes abricots qui sont encore à nettoyer, certains sont abîmés dedans, on s'en fout. Et ceux que j'ai fait ce matin qui sont mis en sucre, en conservation pour pouvoir partir en cuisson tout à l'heure. Donc après ben les tomates cerises sont là. Mes oignons sont derrière. Tomates et mes pommes. En dessous j'ai des noix et des pommes de terre. Et le reste c'est un économat.

00:35:37
*Mme.2:* Oui, tout à fait. Tout à fait.

00:35:38
*Représentant F03:* Ici ce sont les œufs. Pas de frigo pour les œufs.

00:35:42
*Mme.2:* Eh oui.

00:35:43
*Représentant F03:* Contrairement à ce qu'on pourrait penser, hein. Après ça c'est des couvercles de bocaux. Ça c'est pour-- parce que je fais des coffrets donc du coup, c'est de la paille qu'on met dans-- à l'intérieur des coffrets. Et puis bah après ici c'est tout ce qui est, [euh], vide hormis la levure parce que j'ai plus de place de l'autre côté. Donc-- et là c'est réservé aux framboises ou des mirabelles, ou des fruits-- ou des- des gelées que je ne veux pas utiliser, ne pas vendre justement. Et après bah le reste est là.

00:36:13
*Mme.2:* Oui après, c'est effectivement du- du matériel pour--

00:36:16
*Représentant F03:* Travailler.

00:36:16
*Mme.2:* Pour travailler quoi.

00:36:17
*Représentant F03:* Et ici, c'est la réserve de farine. Donc farine de froment, farine de blé noir en-dessous. Donc bien séparées. Et après tout ce qui va être travaillé cette a-- dans- dans l'ann-- dans l'année, [euh], c'est de la-- je prépare donc c'est de la purée de noisette bio ou de la purée d'amande bio pour pouvoir préparer tout ce qui est pâte à tartiner maison. Voilà. Donc après, ben le lait. J'ai pas le droit d'utiliser du lait cru. Je suis obligée de le pasteuriser, mais sauf que ça me prendrait trop de temps pour utiliser-- de pasteuriser du lait. Je préfère l'acheter en-- tout fait quoi.

00:36:52
*Mme.2:* Et c'est du lait Laitik. C'est très, très bien.

00:36:56
*Représentant F03:* Bah oui. Tant qu'à faire. Et la farine--

00:37:02
*Mme.2:* Des 3 Abers.

00:37:03
*Représentant F03:* Les 3 Abers, bio. Pareil pour la baie noire.

00:37:05
*Mme.2:* Ouais, ouais.

00:37:06
*Représentant F03:* Alors là, j'ai juste testé, j'ai acheté une j'étais en-- dans le Morbihan la semaine dernière, [euh], alors il s'est trompé. Je voulais de froment, il m'a mis petit épeautre mais c'est pas grave c'est- c'est du blé aussi. Pareil, bio mais du Morbihan. Comme je vais être dans les parages du Morbihan, ne serait-ce qu'une fois par semaine au mois de juillet, il pourra peut-être devenir mon deuxième fournisseur si ça me convient. On essaye d'avoir-- de travailler avec du bon, quoi.

00:37:29
*Mme.2:* Oui, oui. Du bon et du local.

00:37:29
*Représentant F03:* Et du local.

00:37:40
*Mme.2:* D'accord, et donc bah oui votre-- vos fruits et légumes-- enfin vos légumes qui sont dans la-- dans la réserve, ils sont sur des clayettes.

00:37:49
*Représentant F03:* Oui.

00:37:49
*Mme.2:* Donc ces clayettes sont--

00:37:52
*Représentant F03:* Nettoyées.

00:37:52
*Mme.2:* Sont nettoyées ?

00:37:53
*Représentant F03:* Ouais. À chaque fois que, ben, j'ai fini le produit, ben, je nettoie la clayette. Puis, bah il sert-- après elle est la remise en hauteur, et puis si besoin bah, j'en prends une, je nettoie.

00:38:01
*Mme.2:* Ouais. Ouais, ouais, ouais. Oui, oui.

00:38:08
*Représentant F03:* Souvent, les produits qu'on achète sont pas forcément sales non plus.

00:38:19
*Mme.2:* Ouais. [Euh], bon alors là on devait parler des préparations et de la consommation, hein. [Euh], un fruit que vous consommez très régulièrement, on a parlé de la fraise. Vous la mangez principalement crue.

00:38:31
*Représentant F03:* Crue, ouais.

00:38:32
*Mme.2:* Hein, pas souvent crue-- cuite.

00:38:35
*Représentant F03:* Non, c'est rare.

00:38:35
*Mme.2:* Hein, c'est ça hein ?

00:38:36
*Représentant F03:* Ou cuite avec du citron, quoi. Mais bon, voilà.

00:38:39
*Mme.2:* Oui, oui, oui. Mais en général, c'est mangé--

00:38:41
*Représentant F03:* Généralement, c'est mangé comme ça à l'arrache.

00:38:46
*Mme.2:* Ouais, ouais. Et en légumes que vous mangez très régulièrement, j'ai entendu parler de chou fleur, là cette fois-ci.

00:38:52
*Représentant F03:* Chou fleur, tomate mais c'est plus chou fleur.

00:38:53
*Mme.2:* Plus chou fleur ?

00:38:53
*Représentant F03:* Bah ça y est, ça- ça a démarré donc--

00:38:53
*Mme.2:* Et donc, c'est plutôt cru ? C'est plutôt cuit ?

00:38:53
*Représentant F03:* Les deux.

00:38:53
*Mme.2:* Les deux ?

00:38:53
*Représentant F03:* Les deux. J'ai-- certaines fois où je vais l'émietter mais vraiment en faire une purée, enfin une poudre de chou fleur pour en faire une pizza. Et-- [rires].

00:38:53
*Mme.2:* Avec la pâte à pi-- vous faites la pâte à pizza avec le- le chou fleur ?

00:38:55
*Représentant F03:* Mm. [rires] Et je me sers du chou fleur pour faire une pâte à pizza, oui. Et, [euh], je rajoute juste un œuf et du coup, ça me fait un amalgame qui me permet de mettre ce que je veux dessus et pas-- je suis pas intolérante au gluten ni allergique au gluten, mais de temps en temps j'aime bien une pizza au chou fleur. Et je mets ce que je veux dessus après derrière.

00:39:35
*Mme.2:* Et les tomates ?

00:39:36
*Représentant F03:* Et les tomates, c'est pareil. C'est à l'arrache aussi.

00:39:38
*Mme.2:* C'est, [euh], plutôt--

00:39:38
*Représentant F03:* Tomates cerises, tomates entières hein. Ça peut être une salade de tomates avec bah du chou fleur ou des-- du maïs ou--

00:39:46
*Mme.2:* Donc là c'est plutôt cru ?

00:39:46
*Représentant F03:* Ouais, c'est toujours-- c'est- c'est rare que ça soit cuit.

00:39:49
*Mme.2:* D'accord, vous préférez--

00:39:49
*Représentant F03:* Sauf sur la pizza quoi.

00:39:49
*Mme.2:* Ouais, d'accord, ok. Et quand c'est cuit, il faut que ce soit bien, bien cuit ou ?

00:39:57
*Représentant F03:* Très peu cuit, quoi. [En fait], je-- il faut que ça reste avec de la mâche quand même à l'intérieur de la bouche.

00:40:03
*Mme.2:* Et, [euh], est-ce qu'il y a des fruits ou des légumes-- alors, tout à l'heure, vous m'avez dit que vous- vous prépariez la salade, les radis. Est-ce que vous-- il y a des produits-- enfin des légumes ou des fruits que vous n'épluchez pas ?

00:40:16
*Représentant F03:* Oui, la pomme, la poire. [Euh], la pomme, la poire, l'abricot parce que bon, [en fait], il y a rien à enlever. La pêche, j'enlève pas non plus. Le bru-- enfin parce qu'on dit nectarine maintenant mais moi j'appelle ça le brugnon. Dans le temps, c'était brugnon. Bah, la banane, si, je l'épluche, parce que bah voilà on peut pas faire autrement. Mais autrement--

00:40:37
*Mme.2:* Vous achetez des bananes ?

00:40:39
*Représentant F03:* C'est rare. Vous voyez, il y en a pas là.

00:40:41
*Mme.2:* C'est plutôt en hiver, quoi.

00:40:42
*Représentant F03:* Ouais, c'est vraiment-- et puis ça- ça reste du local-- vraiment par-- avec parcimonie. Parce que bon, j'aime. Mais bon, sans plus. Mais, [euh], en tant qu'entreprise, [euh], certains clients demandent à avoir un cake banane. Du coup, on est coincé, on est obligé d'acheter de la banane. Ils demandent aussi pancake bananes poires ou bananes chocolat. Du coup, bah coincé, on peut pas tout le temps leur dire, bah non. Je l'ai mise sur la carte, je suis coincée. Je suis obligée de faire de temps en temps, mais j'achète si j'ai par exemple un p-- un paquet de pancake ou deux pancakes, je vais acheter juste une banane. Voilà.

00:41:17
*Mme.2:* D'accord, ok.

00:41:17
*Représentant F03:* Juste ce que j'ai besoin. Après, j'achète pas pour stocker. J'achète-- je- je stocke juste pour ma consommation personnelle, ça, ça fait la semaine, quoi. Après bah, je rachèterai. Samedi, je rachèterai des légumes et puis voilà. Des fruits, des légumes.

00:41:37
*Mme.2:* Donc, [euh], les poireaux.

00:41:40
*Représentant F03:* Oui.

00:41:40
*Mme.2:* Vous les épluchez ou pas ?

00:41:41
*Représentant F03:* J'enlève juste le foin.

00:41:45
*Mme.2:* Ouais, c'est-à-dire la racine ?

00:41:46
*Représentant F03:* Oui.

00:41:47
*Mme.2:* D'accord.

00:41:47
*Représentant F03:* Ce qu'on appelle le foin.

00:41:48
*Mme.2:* D'accord. [Euh]--

00:41:48
*Représentant F03:* Et si vraiment le haut de la feuille est abîmé, je vais l'enlever.

00:41:56
*Mme.2:* Mais c'est tout ?

00:41:57
*Représentant F03:* Mais c'est tout. J'en-- bah la première feuille est aussi bonne que la dernière. L'endive, c'est pareil. Je n'épluche pas. J'enlève rien. Je mange le crou-- [euh], je mange--ce qu'on aime pas, ce que beaucoup n'aiment pas, moi je le mange. Le chou fleur, j'enlève juste les feuilles. Entre guillemets, le trognon de chou fleur moi je le mange. Le chou, c'est pareil. J'enlève pas les feuilles non plus. Et le trognon, je le mange. Je le mange cru.

00:42:21
*Mme.2:* Vous le mangez cru ?

00:42:21
*Représentant F03:* Oui, parce que c'est-- le maximum de vitamines est là.

00:42:25
*Mme.2:* D'accord.

00:42:27
*Représentant F03:* Voilà. Donc je mange le trognon de chou fleur, le trognon de chou-- de chou, je mange le trognon de brocoli, [euh], voilà. Je mange les trognons. Parce que c'est là qu'on a le maximum de vitamines. Je vais juste enlever-- comme le fond de la salade, c'est pareil, je vais juste enlever-- si elle a noircie, je vais juste enlever la pellicule noire. Le reste, je mange.

00:42:51
*Mme.2:* Et donc, [euh], moi j'appelle ça les côtes. Mais c'est peut-être pas les côtes--

00:42:52
*Représentant F03:* Les côtes de blette ?

00:42:52
*Mme.2:* Non les côtes de--

00:42:52
*Représentant F03:* De-- d'épinards ?

00:42:53
*Mme.2:* Non. Les côtes de la salade.

00:42:52
*Représentant F03:* Oui, bah je la mange.

00:42:52
*Mme.2:* D'accord.

00:42:52
*Représentant F03:* Pour moi ça fait parti du trognon. Je la mange.

00:43:06
*Mme.2:* Ok. D'accord. Bon, alors j'ai bien compris que vous faisiez vraiment attention à tout utiliser dans- dans le légume ou le fruit. [Euh], mais parfois ça vous arrive de jeter.

00:43:18
*Représentant F03:* Oui. Oui, oui.

00:43:18
*Mme.2:* D'accord. Quand est-ce que vous-- quand est-ce que ça- ça arrive ça ?

00:43:24
*Représentant F03:* Quand c'est moisi. Quand ça-- j'ai pas vu, et que c'est moisi. Par exemple là j'ai acheté, bah, mes abricots la semaine dernière parce que bah, voilà, j'avais que mercredi pour aller les chercher. Ils sont ouverts que le mercredi après-midi hein, ils font du drive. Donc j'ai été. Sauf que ben, jeudi je travaillais, j'étais sur la route. Jeudi après-midi j'ai fait des prépa mais pas ça. J'avais pas le temps de mettre les-- d'a-- de m'occuper des abricots. Vendredi, j'étais en cuisson toute la journée. Samedi matin, j'étais en cuisson et en préparation de marché. Samedi après-midi, j'étais en marché. Hier, j'ai fait des préparations de caramel et compagnie. Et aujourd'hui, ben il fallait que je m'attaque aux abricots. Donc j'ai- j'ai nettoyé les abricots. J'ai nettoyé une caissette de cinq kilos et donc ben, certains n'ont pas supporté.

00:44:08
*Mme.2:* Oui c'était stocké-- ils étaient stockés mais pas au frigo quoi.

00:44:09
*Représentant F03:* Ils étaient stockés et malgré le froid-- ben je les mets pas au frigo parce que ça va s'abîmer encore plus. Du coup, je les ai stocké quand même là-bas, dans le noir. Mais bon, pour autant, ils sont arrivés plus qu'à maturité et en les ouvrant c'était moisi à l'intérieur. Bon bah tout le monde est parti dans la bouteille-- dans la poubelle. Voilà.

00:44:27
*Mme.2:* D'accord. Donc quand c'est moisi.

00:44:27
*Représentant F03:* Quand c'est moisi.

00:44:28
*Mme.2:* Et dans ce cas-là c'est tout le- le fruit ou le légume ?

00:44:28
*Représentant F03:* Bah là j'ai mis tout le fruit parce que tout était moisi.

00:44:35
*Mme.2:* Tout était abîmé, ouais.

00:44:35
*Représentant F03:* Voilà, tout était abîmé. Si il avait eu que quelque-- qu'un seul endroit, j'aurais coupé la partie qui était abîmée, j'aurais gardé l'autre morceau. Soit dans ma bouche, soit dans la préparation.

00:44:48
*Mme.2:* Et comment vous déterminez que un fruit ou un légume n'est plus mangeable ?

00:44:54
*Représentant F03:* Bah quand vraiment il est vraiment moisi, quoi. Je veux dire--

00:44:54
*Mme.2:* Donc c'est quand on le voit quoi.

00:44:54
*Représentant F03:* Oui, voilà.

00:44:54
*Mme.2:* C'est pas l'odeur, c'est pas le toucher ?

00:44:54
*Représentant F03:* Non. C'est soit il s'est éclaté dans le-- dans le lieu de stockage parce qu'il était arrivé à-- trop à maturité. Les pommes de terre, si, c'est quand même à l'odeur parce que quand il y a des pommes de terre pourries dans le quartier, on sait que ouh là, il est temps de faire le nécessaire. [Euh], donc, voilà. Et autrement, ouais c'est vraiment au visuel quoi. Au visuel.

00:45:20
*Mme.2:* C'est plutôt visuel.

00:45:25
*Représentant F03:* Ouais.

00:45:25
*Mme.2:* D'accord. Et quand vous voyez ou que vous fait-- soyez-- oh là là, quand vous voyez ou que vous sentez un- un- un fruit ou un légume qui est abîmé, voire très abîmé, pourri, moisi, enfin bon voilà, qu'est-ce que vous ressentez ?

00:45:39
*Représentant F03:* De la colère contre moi.

00:45:39
*Mme.2:* D'accord, colère.

00:45:47
*Représentant F03:* De la colère contre moi parce que ben, voilà [Prénom de l'interviewé], t'avais qu'à t'y coller un peu plus tôt, t'aurais pas perdu ton produit. Voilà. Tout simplement. C'est que de la colère contre moi. C'est-- voilà, t'es- t'es pas-- t'as pas fait le nécessaire à ce moment-là. T'aurais dû le faire plus tôt. Pourquoi t'as pas bougé ton cul ? Enfin, voilà des-- c'est pas de la colère contre quelqu'un d'autre. C'est-- ça peut arriver que ça soit contre ma-- mon mari qui des fois m'achète des pommes et qui se rend pas compte que dans le lot qu'il m'a acheté, il y a une pourrie. Et alors là la colère est contre lui. Tout de suite. Mais généralement, c'est contre moi.

00:46:22
*Mme.2:* D'accord. [Euh], donc ça c'est quand vous voyez, quand vous sentez. Et quand vous le toucher ? Non, vous l'avez senti, vous l'avez vu avant.

00:46:22
*Représentant F03:* Ah bah touché, ça y est il part-- il part à la poubelle. À peine je le touche, il part à la poubelle.

00:46:36
*Mme.2:* Ouais. Ouais, ouais, ouais.

00:46:36
*Représentant F03:* Vous avez un abricot ou une fraise qui vous éclate dans les mains, pourquoi je l'ai pas mangé avant, quoi ?

00:46:41
*Mme.2:* Et là, c'est pas de la colère, c'est du regret de pas l'avoir mangé avant.

00:46:44
*Représentant F03:* Oui, puis en même temps c'est aussi-- quelque part c'est un peu de la colère parce que ben, elles sont à disposition. Et puis, ben je me dis bah non, arrête de manger, ça suffit. Voilà. Ne remplit pas ton estomac que de fraises, mange autre chose aussi à côté et puis bah je-- bon bah tant pis je la remets. À regret, je vais la remettre dans le truc. Puis je vais l'oublier. Donc après bah je vais me gronder parce que bah j'aurais dû la manger.

00:47:13
*Mme.2:* Bon, alors on va imaginer que vous l'ayez pas vu, que vous l'avez pas senti, que au toucher c'était pas si, [euh], catastrophique que ça. Et vous l'avez mis en bouche.

00:47:25
*Représentant F03:* Ouais, bah là c'est le-- alors c'est soit elle est pas encore très moisie et elle va descendre dans le gosier quand même.

00:47:32
*Mme.2:* Ouais ?

00:47:36
*Représentant F03:* Soit je vais la recracher.

00:47:39
*Mme.2:* D'accord, ok.

00:47:40
*Représentant F03:* Si vraiment elle était juste abîmée mais qu'il y avait pas de la moisissure dessus et que-- ou la moisissure était de l'autre côté, j'ai pas fait gaffe et j'ai croqué dedans, c'est soit je recrache de suite parce que je suis tombée sur le bout de moisi, soit bah j'avale parce que bah, tout compte fait, elle est partie trop loin quoi.

00:47:55
*Mme.2:* Ouais et est-ce que votre conjoint réagit de la même façon par rapport--

00:48:09
*Représentant F03:* Non, lui dès qu'il voit un truc qui est un peu-- entre guillemets, périme, lui c'est poubelle tout de suite.

00:48:09
*Mme.2:* Il se pose pas trop de questions, quoi ?

00:48:11
*Représentant F03:* Mm.

00:48:12
*Mme.2:* Et c'est tout le fruit ou tout le légume quoi.

00:48:12
*Représentant F03:* Mm. Même s'il y a une partie récupérable. Je l'entends déjà, oui mais bon c'était moisi quoi.

00:48:24
*Mme.2:* D'accord. Donc on ne prend pas de risque ou alors je veux pas m'enquiquiner ?

00:48:26
*Représentant F03:* Ah c'est je veux pas m'enquiquiner, je veux surtout pas m'empoisonner, ça va me tâcher les mains. J'ai un mari délicat. Du coup, s'il a le malheur par exemple de tomber sur une pomme de terre qui est un peu pourrie, [rires], au secours.

00:48:40
*Mme.2:* Bon. Alors, j'aimerais bien qu'on parle de gaspillage alimentaire. [Euh], quels sont les mots qui vous viennent à l'esprit lorsque vous entendez le terme gaspillage alimentaire ?

00:48:59
*Représentant F03:* Perte de temps, perte d'argent, [euh], non respect de-- bah du produit, non respect de la terre, non respect de travail. C'est déjà pas mal.

00:49:14
*Mme.2:* D'accord. Alors on va prendre-- on- on- on va prendre, [euh], perte de temps. Est-ce que vous pourriez m'expliquer le terme, perte de temps ?

00:49:23
*Représentant F03:* Perte de temps, je pen-- j'ai pensé à l'agriculteur qui a dû préparer sa terre, qui a fait-- qui l'a mis en semence, qui a donc tout géré, la partie entre le premier labour et la récolte. C'est une perte de temps de savoir que tout- tout le temps qu'il a utilisé pour qu'ensuite on mette le produit à la poubelle. Voilà, c'est toute cette partie-là qui est une perte de temps. Parce que si il avait su que son produit n'aurait pas été pris-- acheté par un magasin, ben pendant cette période de temps, il aurait pu faire autre chose et vendre un produit qui aurait peut-être pu être-- enfin préparer un produit qui aurait pu être-- voilà. Pour moi, c'est ça. C'est une perte de temps. Toute cette partie-là.

00:50:12
*Mme.2:* D'accord.

00:50:13
*Représentant F03:* La perte d'argent, pour l'agriculteur aussi. Déjà ça. Après gaspillage alimentaire, perte de temps pour moi aussi, [euh], de pas avoir utilisé le produit tout de suite au moment où je l'ai acheté ou dans la semaine qui a suivi. Perte d'argent parce que j'ai aussi perdu de l'argent entre la-- l'acheter, j'ai mis à la la poubelle. C'est comme si j'avais mis mon billet. Poubelle. Voilà, les deux sont- sont liés.

00:50:36
*Mme.2:* [Euh], non-respect du produit ?

00:50:40
*Représentant F03:* Ben non-respect du produit bah, on l'a quand même mis en terre, on l'a fait pousser. Pour rien. Pareil, c'est comme si on l'achetait là, maintenant, et qu'on le mettait directement à la poubelle. Ça sert à rien. Je veux dire, c'est- c'est à nous de prendre conscience que ben si on l'achète, c'est pour le manger. C'est pas pour laisser pourrir et attendre qu'il aille dans la poubelle, quoi. Voilà, ça c'est-- je-- j'ai du mal avec ça parce que-- voilà pourquoi moi je suis assez stricte sur-- entre le moment où le-- un produit rentre chez moi et au moment où les épluchures ou les détritus sont dech-- les- les pépins et tout ce que je peux pas récupérer, sont mis à la poubelle parce que ben il y a quand même du travail derrière. Il y a de l'argent derrière. On peut pas se permettre de mettre des choses à la poubelle comme ça, quoi. Et il y a un non-respect du-- ben du produit, du travail-- du travailleur qui a-- qui a-- qui a bossé quand même pour nous [inaudible 00:51:36] quoi. Donc tout ça, j'ai du mal quoi. Et je suis en colère contre tout ça.

00:51:40
*Mme.2:* Et là, vous m'avez donné perte de temps, perte d'argent ou non-respect. Quel est le mot le plus important pour vous, des trois ?

00:51:49
*Représentant F03:* Le non-respect.

00:51:51
*Mme.2:* Non-respect, ouais.

00:51:51
*Représentant F03:* Le non-respect. Ça englobe tout, [en fait]. C'est le non-respect. Le non-respect du travailleur, le non-respect du-- bah de- de-- bah de- de son travail, de l'argent qu'il a mis et, [euh], bah de tout ce qui-- tout ce qui va derrière, quoi. De tout-- bah du- du produit fini et de son-- de son usage. C'est vraiment le non-respect.

00:52:13
*Mme.2:* [Euh], et si je devais expliquer à mes enfants que je suis en train de travailler sur le gaspillage alimentaire donc il faudrait que je leur explique, que je leur définisse la notion de gaspillage alimentaire. Comment je pourrais leur expliquer ça ?

00:52:33
*Représentant F03:* Ça va être compliqué ça.

00:52:33
*Mme.2:* Comment je pourrais définir ce terme gaspillage alimentaire ?

00:52:44
*Représentant F03:* Ben on pourrait partir sur l'idée que-- je vais partir sur un bonbon. Votre enfant, il veut un bonbon. Sauf que pour avoir le bonbon fini, il faut d'abord, [euh], planter-- préparer une terre sur laquelle on va mettre de la canne à sucre qu'on va récolter, qu'on va transformer pour récupérer le sucre et que ensuite on va demander à une autre personne de cuisiner le sucre, de rajouter des produits colorants ou autres. Ensuite, on va demander à une autre personne de transformer ce sucre en petits morceaux, en petits bonbons. Ensuite, on va donner à une autre personne ces petits bonbons pour les emballer. Et ensuite on va les offrir à votre enfant. Et donc, le gaspillage alimentaire c'est de n'ouvrir-- c'est d'ouvrir le paquet de-- le- le bonbon, de le sucer un petit peu et de le mettre à la poubelle ensuite parce qu'on n'a pas aimé. Et donc, c'est un non-respect du- du travail de toutes les personnes qui ont fabriqué le bonbon.

00:53:50
*Mme.2:* Et en fin de compte, par ricochet, si on devait parler du gaspillage alimentaire en général, ce serait le non-respect du travail de l'ensemble de la chaîne.

00:54:00
*Représentant F03:* De l'ensemble de la chaîne du travail-- du travail pour avoir le bonbon fini. Pour qu'il soit dans la bouche--

00:54:04
*Mme.2:* Ou du fruit ou du légume, quoi.

00:54:04
*Représentant F03:* Ou du fruit ou du légume voilà mais, [euh], voilà. C'est, [euh], ça serait de cette logique-là que j'a-- moi je partirais pour expliquer à un enfant. Et lui montrer des dessins, avec des dessins pour expliquer.

00:54:17
*Mme.2:* Et pour expliquer à un adulte, ça serait aussi la même chose ?

00:54:20
*Représentant F03:* Ça serait la même chose, ouais.

00:54:20
*Mme.2:* Ouais, ok. [Euh]--

00:54:21
*Représentant F03:* [En fait], c'est vraiment ça. C'est vraiment le manque de respect du travail d'autrui.

00:54:30
*Mme.2:* Ouais. Et quand vous jetez un fru-- un fruit ou un légume, [euh], vous ressentez de la colère.

00:54:40
*Représentant F03:* Oui.

00:54:43
*Mme.2:* [Euh], vous ressentez d'autres choses aussi ou pas ?

00:54:46
*Représentant F03:* Bah de la colère. C'est surtout de la colère contre moi. Parce que ben comme je l'ai dit tout à l'heure, ben c'était à moi, ben, de trouver du temps dans mon planning de telle manière à pouvoir travailler le produit, sans-- sans qu'il se dénature.

00:55:01
*Mme.2:* Ouais, je vois.

00:55:02
*Représentant F03:* C'est à moi d'organiser mes journées en fonction de ce que je fais rentrer comme produit avec-- l'anticipe-- j'anticipe toujours, [euh], mes semaines en me disant, bah tiens cette semaine, tiens voilà, les abricots sont tombés, voilà ils sont-- et je les rentre. [Euh], du coup, [Prénom de l'interviewé], trouve du temps dans la semaine. Voilà, prépare ton planning de telle manière à ce que tu te donnes deux heures ou trois heures pour nettoyer tes abricots, pour pouvoir les mettre déjà en sucre après, bah ils attendent 12 heures avant d'être mis en cuisson. Donc, c'est à moi d'anticiper tout ça. Sauf que quand-- si je décidais par exemple, pendant ces trois heures, de me la couler douce sur mon canapé, mais pendant ce temps-là mes fruits s'abîment. Mais je perd du temps parce que après ces trois heures de temps, si je n'ai rien fait, j'ai d'autres choses à faire que je suis obligée de mettre en place. Du coup, c'est du temps perdu. Donc c'est de la colère que je ressens contre moi parce que j'ai pas bougé mes fesses.

00:55:50
*Mme.2:* D'accord. Bon. Alors je vois la- la- la poubelle qui- qui trône à côté de l'évier.

00:55:59
*Représentant F03:* Normalement, j'ai pas le droit d'avoir de- de poubelle dans ma cuisine.

00:56:02
*Mme.2:* Ah.

00:56:03
*Représentant F03:* Bah oui mais le co-- le do-- le sale ne doit pas côtoyer le propre. La mienne, elle est dehors. Mais bon, parce qu'elle est électronique, j'ai accepté qu'elle soit dans la cuisine. Voilà. Mais elle est-- elle est quand même pratique parce qu'elle est pas loin de là où je nettoie mes légumes et mes fruits.

00:56:18
*Mme.2:* Bon. Donc ça va.

00:56:18
*Représentant F03:* Donc ça va.

00:56:21
*Mme.2:* [Euh], est-ce que vous l'utilisez régulièrement ? A priori, oui hein.

00:56:25
*Représentant F03:* Oui. Oui, oui.

00:56:25
*Mme.2:* [Euh], vous m'avez parlé des- des abricots qui ont été jetés il y a pas très longtemps. Vous les avez jetés parce qu'ils étaient vraiment très, très, très, très abîmés. Il y a eu d'autres exemples ?

00:56:38
*Représentant F03:* Une fraise ou deux qui ont dû partir aussi, qui étaient moisies donc elles sont dedans. C'est tout.

00:56:39
*Mme.2:* Ouais, ok.

00:56:39
*Représentant F03:* Mais, il y a peu hein. Là aujourd'hui, si, elle a été utilisée un peu plus, Il y a des-- il y a des-- il y a eu des semaines où elle a pas du tout été ouverte.

00:56:51
*Mme.2:* Il y a des semaines où elle a pas été ouverte ?

00:56:52
*Représentant F03:* Ouais.

00:56:54
*Mme.2:* Malgré votre activité ?

00:56:57
*Représentant F03:* Ouais. Ouais.

00:56:58
*Mme.2:* Et est-ce que vous trouvez que c'est pratique ou pas ? Bon, elle est à côté de l'évier donc ça c'est pratique.

00:57:00
*Représentant F03:* Oui. Oui, oui, c'est quand même pratique. Après il manque une pédale.

00:57:05
*Mme.2:* Oui, il manque-- c'est vrai. C'est vrai, c'est vrai.

00:57:09
*Représentant F03:* Il manque la pédale. Parce que, [euh], on n'a pas tout le temps, [euh], la possibilité, [euh], ben d'avoir les mains propres pour soulever le couvercle, pour déposer ou jeter et refermer le couvercle. Une- une pédale serait indis-- presque indispensable parce que du coup, ben on a les fruits, les légumes dans la main.

00:57:30
*Mme.2:* Oui, ça aurait aurait été plus pratique.

00:57:32
*Représentant F03:* Ça aurait été plus pratique, voilà.

00:57:33
*Mme.2:* Ça aurait été plus pratique ouais. Ouais, c'est vrai.

00:57:34
*Représentant F03:* Après, c'est une idée, hein. Mais, à étudier.

00:57:35
*Mme.2:* Ouais, non mais c'est vrai que c'est, [euh], c'est une idée. Ouais, ouais. Ouais, ouais, ouais, ouais.

00:57:41
*Représentant F03:* Sinon oui, après- après, [euh], j'ai un deuxième souci, c'est le changement de sac.

00:57:43
*Mme.2:* Oui ?

00:57:45
*Représentant F03:* [Euh], il faut tenir le couvercle pour changer le sac et donc une seule main pour mettre un sac autour, c'est pas évident non plus.

00:57:50
*Mme.2:* C'est pas pratique non plus.

00:57:51
*Représentant F03:* Voilà, c'est les deux seules choses qui sont pas pratiques.

00:57:53
*Mme.2:* Bon. Est-ce que il y a eu des moments où vous vous êtes posée des questions, est-ce que je dois mettre ça dedans ? Ou est-ce que je dois pas mettre ça dedans ?

00:58:01
*Représentant F03:* Non, c'est-- Charlotte m'avait bien expliqué. Et puis en même temps je l'ai bien lu le bouquin. Il est dans le tiroir, toujours au cas où donc, voilà.

00:58:07
*Mme.2:* Non, Charlotte est bien.

00:58:08
*Représentant F03:* Oui, franchement là-dessus--

00:58:08
*Mme.2:* Ouais. Charlotte est très bien.

00:58:11
*Représentant F03:* Alors après j'avais-- bon j'avais le bouquin et donc quand j'avais des doutes, je reprenais le bouquin. Et en même temps, c'est bien expliqué dessus aussi.

00:58:17
*Mme.2:* Comme c'est Charlotte qui va écouter les enregistrements, Charlotte est très, très, très, très bien.

00:58:22
*Représentant F03:* Elle va se dire [Prénom de l'interviewé] elle est toujours aussi bavarde.

00:58:26
*Mme.2:* Et est-ce que ça vous arrive de-- d'oublier cette poubelle ?

00:58:29
*Représentant F03:* Oui, ça m'est arrivé de-- une fois ou deux d'oublier de mettre dedans.

00:58:33
*Mme.2:* D'accord. Ok. Non c'est simplement pour savoir.

00:58:34
*Représentant F03:* Oui, oui. Non mais je dis la- la vérité, oui ça m'est arrivé parce que avant, [euh]-- moi j'ai ma poubelle qui est dehors, on la voit bien mais je-- j'ouvre pas toutes les cinq minutes ma- ma porte-fenêtre. Parce que bah, pareil, j'ai pas les mains tout le temps propre pour ouvrir. Donc je fais au le petit seau blanc, et le petit seau blanc est ma poubelle, entre guillemets, de cuisine aussi.

00:58:49
*Mme.2:* D'accord.

00:58:55
*Représentant F03:* Et donc du coup quand elle est pleine, poubelle. Mais il y a des fois je dis, eh merde j'aurais pas dû mettre dedans. J'ai oublié la poubelle en-dessous. Voilà.

00:59:00
*Mme.2:* D'accord. Bon.

00:59:04
*Représentant F03:* Mais bon, c'est arrivé une fois ou deux et puis voilà. Après, j'ai grondé monsieur en disant, mais il y a la poubelle là hein.

00:59:13
*Mme.2:* Elle est trop petite c'est pour ça on l'a voyait pas. [Euh], et--

00:59:17
*Représentant F03:* Non mais il voulait pas s'en approcher parce qu'il avait peur parce qu'il voyait-- il y avait des voyants rouges, il me disait c'est quoi ces voyants, voilà.

00:59:17
*Mme.2:* Une poubelle intelligente.

00:59:25
*Représentant F03:* Et hier soir il m'a demandé, il m'a dit, on la garde après ? J'ai dit ah non, elle fait partie d'un projet, il faut la rendre parce qu'elle va servir dans d'autres foyers. Ah bon ? Parce que moi je commençais à la trouver pratique. Vous voyez ?

00:59:34
*Mme.2:* Mais vous allez de nouveau en avoir une autre après, hein.

00:59:38
*Représentant F03:* Oui, oui, c'est ce que je lui ait dit. Bah voilà. Mais j'ai dit, bah en attendant-- voilà.

00:59:39
*Mme.2:* [Euh], et est-ce que vous avez changé la-- le- le sac poubelle ? Oui ?

00:59:39
*Représentant F03:* Oui. Deux fois.

00:59:39
*Mme.2:* [Euh], deux fois déjà ?

00:59:39
*Représentant F03:* Oui, deux fois.

00:59:49
*Mme.2:* D'accord. Ok. Est-ce que il y a des- des points qu'on n'a pas abordés et que vous souhaiteriez qu'on aborde ?

00:59:57
*Représentant F03:* Non, je pense qu'on a fait le tour.

00:59:57
*Mme.2:* Ben, je sais pas. C'est à vous de me le dire.

01:00:02
*Représentant F03:* Je pense ouais, qu'on a fait le tour. Si parce que-- si parce que j'ai parlé des- des-- la non-praticité de certaines choses de la poubelle.

01:00:08
*Mme.2:* La pédale et le fait que, [euh]--

01:00:09
*Représentant F03:* Ça soit compliqué. [En fait], il faudrait qu'on puisse trouver le moyen de tenir le couvercle. Il faut être deux. C'est soit quelqu'un se colle à tenir le couvercle pendant que je change le sac, soit il tient tout seul. Sauf que là, il tient pas tout seul. Donc, on est obligé de le tenir et enlever le sac. L'enlever c'est facile. Mais le remettre, c'est plus délicat.

01:00:28
*Mme.2:* Oui. Oui, oui.

01:00:29
*Représentant F03:* Bon après, les sacs sont peut-être un petit peu serré pour la poubelle.

01:00:32
*Mme.2:* Ouais.

01:00:34
*Représentant F03:* Mais bon.

01:00:34
*Mme.2:* Mais d'un tout autre côté au-- c'est pour ça aussi que ça tient bien. Donc, voilà.

01:00:36
*Représentant F03:* Voilà. Mais autrement tout va bien.

01:00:39
*Mme.2:* Ok. Bah merci à vous.

01:00:41
*Représentant F03:* Bah de rien. J'étais pas si bavarde que ça. J'ai raconté des bêtises.

01:00:42
*Mme.2:* Mais non.

01:00:52
*Représentant F03:* Parfois, j'aime bien expliquer, voilà.

01:00:54
*Mme.2:* Mais c'est très sympa de-- franchement, merci.

**Household F03, Interview 2**

Speaker 0: Alors, pour commencer, je vais vous proposer quatre photographies. Je vais les mettre de dos et je vais vous inviter... Vous avez les miennes aussi, du coup ? Vous prenez celle que vous voulez vous la retournez et vous me dites spontanément les trois premiers mots ou expressions qui vous viennent à l'esprit

D'accord Vert. Vert. Jardin. Aromate. C'est joli. C'est plaisant. C'est bien agencé. C'est bien entretenu. Et ça donne envie.

Ok. Alors, pourquoi ça vous donne... Envie ? Envie, oui.

Parce que j'ai envie de les travailler, j'ai envie de les prendre, les goûter, les sentir. Oui, de les travailler, les manger, les jouer avec. Quand vous dites envie de les travailler, c'est... J'ai envie de les travailler, c'est-à-dire les associer soit avec autre chose, mais d'abord les jouer avec, c'est-à-dire les triturer, les sentir, les prendre dans la main, les « débioter », les mettre en bouche pour goûter, pour voir avec qu'est-ce que je ressens, ce que ça me donne envie, avec quoi, comment les travailler après, est-ce que je les travaille… seule ou en association, voilà quoi.

Ok, donc ça vous donne envie de mettre la main,

la main dedans, le nez dedans, enfin voilà, là j'ai envie d'ouvrir avec mes mains, mettre le nez à l'intérieur, voilà quoi, vraiment sentir, ressentir, toucher, malaxer, vraiment... Ben, pas me plonger dedans, mais bon, parce que c'est pas assez grand, mais pourquoi pas, quoi, mettre la tête dedans, voilà, quoi. C'est... Ouais. Ok.

Et pourquoi ça vous donne envie, comme ça, de faire plein de choses ?

Après, je sais, bon, voilà, c'est... Je pense que c'est mon côté cuisinier qui ressort à la surface. Après, c'est joli, c'est bien agencé. C'est pareil, si on a envie d'aller faire un tour, regarder, sentir, c'est vraiment plaisant. J'aurais été enfouie, j'aurais peut-être moins été explicite, quoique j'aurais peut-être été fouillée dedans quand même. (Silence) Pourtant, j'aime pas trop le vert. La couleur verte, j'aime pas. Pour en habit. Mais dans la cuisine, c'est joli, c'est plaisant, ça donne de l'espoir. Plein de couleurs.

Pour vous, voir du vert comme ça, ça a un côté agréable, avoir tout ce vert.

Oui, c'est plaisant. C'est la nature, c'est l'extérieur, c'est ce qui pousse naturellement. N'importe quelle terre battue, vous laissez... Il y a toujours une petite plante verte qui ressort, que ce soit une mauvaise herbe ou une jolie plante. Il n'y a pas de mauvaise herbe. Il y a toujours quelque chose qui pousse et généralement, ce qui pousse, c'est vert.

Ça représente la nature.

Tout ça, c'est nature. C'est la vie. C'est la vie. N'importe quelle terre battue, n'importe quel tremplin que vous voyez partout, vous laissez terre battue. Si vous ne recouvrez pas, il y a toujours quelque chose. Il y a toujours une plante. Et généralement, elles sont vertes. Oui, tout à fait.

Donc, globalement, c'est un lieu dans lequel vous vous sentirez bien.

Oui. J'en passerai des heures.

Est-ce que vous avez déjà eu une ou des expériences avec ce type de lieu ?

J'ai déjà eu un jardin potager, oui. J'ai mis la mer dans la terre, j'aime bien triturer, j'aime bien... Moi, ce que je n'aime pas trop, c'est les habitants de la terre. Alors, tomber sur la musique sur les mers... Non. Mais autrement, oui, jouer à la terre, j'ai toujours aimé ça. Oui.

Oui. On dit de jouer avec la terre, donc il y a un aspect un peu ludique de s'amuser.

Oui, tu peux tuer la terre, voir comment elle est faite, est-ce qu'il n'y a que des petits cailloux, est-ce qu'il y a de la vie, est-ce que ce n'est que des particules de terre, ou est-ce qu'il y a des morceaux de feuilles, vraiment triturer. Ce n'est pas jouer, juste gratter avec un râteau, prendre un peu de terre dans les mains et puis... On va passer d'abord comme ça et puis après on va passer sous l'eau pour voir qu'est-ce qu'il reste. Comment ça se désagrège, qu'est-ce qu'il me reste dans les mains. Sauf les busucs quoi. On les termine tout ça, ça ne me gêne pas. Mais les busucs et tout ce qui est serpent, enfin tout ce qui est un peu rampant comme ça, je n'aime pas trop. Je ne comprends pas.

Je ne suis pas fan non plus.

Les masses, tout ça, ce n'est pas la peine non plus. Mais bon, après le reste, moi j'aime bien. Ouais. D'accord.

Et du coup, vous avez déjà consommé des produits qui venaient de ce type de... Oui. Oui. Oui. Oui. Oui.

Frambosier peut-être ? Non, pas frambosier. Je le trouve épais. Ici, il y a les cassis dans le coin, il y a les frambosans cartés. Ici, il y a une plante de terre qui a encore poussé à l'extérieur. Frambosier. Ici, il mange tout ce qu'il y a dedans. Il y a des poireaux qui traînent aussi derrière. Frambosier.

Et du coup, quand vous aviez un potager, vous consommiez, vous faisiez des plantes, des légumes ?

Oui. Oui.

Et globalement, vous pensez quoi de ces produits, souvent, qui sont issus de potagers comme ça, de jardins ?

Bonne qualité, généralement.

Oui, généralement, vous les trouvez bons ?

Oui. (silence) Pas forcément meilleurs que dans le commerce, mais bons.

D'accord. Je crois que vous avez remarqué qu'il n'y avait pas non plus une grande différence.

Non, pas spécialement. Ça dépend dans quel commerce on va. Que ce soit du commerce ou les miens, on n'a pas forcément de saveur. différentes on va dire, la coriandre ça reste de la coriandre, poireaux, graisses poireaux, enfin je veux dire il ne sera pas meilleur parce qu'il sera cultivé par un maraîcher bio ou moi dans mon jardin, je veux dire c'est le même goût, le même aspect, lui il aura peut-être plus de blanc comme on dit, de fût (le poireau), parce qu'il aura peut-être plus profond que moi, mais ça reste la même plante à la base.

Et du coup, est-ce qu'il y a d'autres choses qui sont de bonne qualité, qui ont la saveur que vous trouvez quand même sensiblement la même ? Est-ce qu'il y a d'autres avantages ou pas avec des fruits et légumes qui sont issus du potager ?

La culture raisonnée, sûrement, parce que moins de produits de pesticides et compagnie, parce qu'on n'en met pas, du coup, les... Comment on appelle ça ? Les... Oh, paysans ! Non, pas paysans, mais...

Producteurs,

producteurs, voilà. Les petits producteurs à l'échelle... réduite. il est bien rare qu'on mette des produits phytosanitaires dans la terre pour aider à pousser. c'est juste par plaisir qu'on fait pousser c'est pas pour du rendement. Donc si ça pousse c'est bien c'est plus valorisant de dire j'ai réussi à faire mes poireaux c'est le plaisir de faire pousser de découvrir comment ça pousse même pour les enfants. je veux dire C'est ludique, en fait. À la base, quand j'avais fait mon potager, c'était plus pour montrer aux enfants comment pousser les légumes. On ne les trouvait pas en sachet dans le magasin.

C'est ça.

C'était plus ça. Après, ça pousse, c'est bien. Ça ne pousse pas. Ce n'est pas grave quand on recommence. Quand ça pousse et que l'enfant ne veut pas manger, c'est mieux de la planter. Oui, c'est vrai. Du coup, ça l'incite à manger ce qu'il a produit. Donc, plus facile à lui faire manger parce que justement, il a pris du temps pour le faire pousser. Donc, c'était une bonne expérience.

Vous pensez qu'il y a une différence quand c'est notre produit qu'on a produit ? Dans la manière dont on va le manger, dont on va le cuisiner ?

Non, pas forcément. Mais pour mes enfants, oui, c'était plus facile de les faire accepter de manger des légumes puisqu'ils les avaient fait pousser.

D'accord.

Mais après, non. Moi, non, non. Moi, je peux très bien acheter chez le maraîcher. Je ne vais pas aller vérifier comment il a fait sa culture pour manger soit du rutabaga ou de l'escorçonnerre. Ouais. Je m'en fous. Moi, je fais confiance, on va aller dire. J'ai la cuisine. Après, bon, si le plat ne donne pas un bel aspect, bon, je vais râler. Mais bon, c'est peut-être ma connerie aussi, quoi. Ouais. Pas forcément la sienne. Oui. Voilà. Peut-être un soupçon. Comment il a travaillé son produit ? J'irai lui poser la question la prochaine fois.

Aux maraîchers ? vos fruits et légumes, ils sont essentiellement issus de... Vous les achetez... Directement au marché. Effectivement, pour vous, il n'y a pas de différence entre son jardin et celui que vous pourriez avoir. C'est le même principe.

Oui

Ok. Est-ce que ça vous est déjà arrivé de jeter des produits que vous aviez un jour récoltés vous-même ? Et ben, pas d'affaires.

Oui, oui, ça m'est déjà arrivé, oui, bien sûr. Comme les pommes de terre terre, par exemple, qui est remplie de doriforts, ou de trous de doriforts. Et on se dit, mais pourquoi ils sont venus chez moi, quoi ? Et le voisin, il n'en a pas, quoi.

Donc c'est des produits que vous avez perdus surtout parce que... Mais pas parce qu'ils étaient stockés chez vous ?

Non. Après, il n'y en a pratiquement plus. Ils sont mangés régulièrement. C'est vraiment parce que c'est une bêtise. Je ne peux pas faire attention à ce que j'ai acheté. Ça m'arrive d'acheter des pommes. Il ne faut pas faire gaffe que dans le lot, il y a une pourrie. Forcément, c'est là. Je vais la garder. Je vais la dépioter. Je vais garder les côtés sains. le manger, je vais récupérer les pépins, les mettre au congèle et la peau au congèle aussi.

Qu'est-ce que vous faites avec les pépins et la peau ?

Ça me donne de la pectine pour faire mes confitures derrière.

Ah, vous utilisez ça pour faire les confitures ?

Oui, c'est une pectine naturelle. Oui, oui. Après, ce qui est pourri, c'est la poubelle bien sûr, mais c'est des piottés avant. Oui, d'accord.

Ok. Bon, ça marche, on a fait le tour du potager. Alors, est-ce que vous pouvez prendre notre photo et dire ce qui vous vient à l'esprit ?

Petit commerce de proximité, étalage, fruits, produits de saison, mais pas que. Produits étrangers, exotiques. Est-ce que je vois banane ? L'ananas, c'est pas produit en France, ça ? Des abîmations. Pourquoi ? Pourquoi pour ce petit engin, là ? Petit fruit mis dans du plastique, par exemple ? Ah oui. Alors qu'ils ont déjà... Souvent, les fruits et les légumes sont déjà protégés par la nature, on va dire. Donc, pourquoi éplucher et remettre en plastique ? Donc, une aberration pour moi. Même si le feu se gâte. Il y a d'autres moyens d'utiliser. Mon fruit, apparemment, plus de saison maintenant en automne. Parce qu'il y a des noix, il y a des clémentines, il y a du raisin. les citrons verts, tout ça c'est comme j'ai dit, c'est étranger, donc un peu plus de mal. Des légumes et des fruits qui ont fait beaucoup de kilomètres. Chili. C'est un plastique.

Est-ce que c'est des lieux dans lesquels ça vous arrive d'aller ou pas du tout ?

Alors moyennement.

Moyennement.

Si vraiment je ne trouve pas sur mon marché, je suis contrainte d'aller dans ce genre de magasin.

D'accord.

Voilà, c'est une contrainte.

D'accord.

Parce que je n'aime pas faire les courses déjà à la base. Non, je suis tenue d'en faire. par mon métier, mais si je peux éviter d'en faire dans les grandes surfaces ou dans des grands magasins, au secours, je n'y vais pas. Je préfère, je privilégie des petits producteurs, des petits producteurs. Je sais que tel jour je vais au marché, voilà, se récupérer mes fruits, mes légumes, ça fait la semaine et on part la semaine prochaine. J'ai horreur d'aller faire tous les jours des courses. (silence) Après, noeud pap, ça paraît surréaliste d'avoir un vendeur avec un noeud papillon. Après, l'espace entre les... Pour moi, il y a trop de fruits et des légumes au mètre carré. Rentabilité, d'espace.

Pour vous, il y a... C'est un magasin dans lequel vous pouvez vous sentir bien ou non, pas vraiment ?

Non, c'est juste en cas de panne. En dépannage, on va dire. En dépannage. Vraiment, il y passe 5 minutes. Pour ce que j'ai besoin, je ne regarde pas ce qu'il y a autour. Non, pas pour être tentée, mais justement pour ne pas passer 3 heures dans le magasin. à devoir patienter, discuter, je prends ce que j'ai besoin et puis je m'en vais. Vraiment du dépannage. Je vais passer vite fait pour pas dire.

Et les produits que vous pouvez trouver là-bas, quand vous vous y rendez pour un dépannage, qu'est-ce que vous en pensez ? Des fruits et légumes que vous pouvez avoir dans ce genre de proximité...

Après, je me poserai la question de savoir d'où ils viennent. Déjà, comme je l'ai dit, les limes, tout ce qui est sur le côté gauche, ça ne m'intéresse pas trop. Ce qui va m'intéresser, ce sont les fruits de saison. Et après, je vais poser la question quand même aux petits vendeurs. Ils ne sont pas pleins. Je vais demander d'où ils viennent, si je n'ai pas la provenance. Et je vais demander de quelle manière ça a été produit.

D'accord.

Si c'est de la culture raisonnée, je vais dire oui. Si c'est de la culture intensive, je ressors.

Ok.

Voilà. Et si les pommes, par exemple, que j'ai besoin, que je ne trouve pas sur le marché pour X raisons, parce qu'il n'y en a plus ou parce que je voulais une variété précise et que mon fournisseur ne l'avait pas, si les pommes que le petit vendeur à son noeud pap me dit qu'elles ont passé quelques mois dans les frigos, c'est non aussi. Je veux des fruits à la saison.

Donc ce genre de magasin, oui, mais tout dépend de ce qu'ils vendent.

Voilà. Et à combien il les vend aussi ? Et à combien il les vend ? Après, je comprends qu'il a besoin d'aller faire un tour à Rungis ou d'aller chercher ses bananes. Bien sûr, ce n'est pas lui qui va les chercher. Donc, ça ne vient pas de transporteur. Mais du coup, tout ça, c'est un coût. Je comprends. Mais on n'a pas spécialement besoin de pomplemousse asiatiques. Alors que ça peut être produit en Italie, beaucoup moins loin. Oui. Pourquoi faire venir en bateau des produits qu'on peut trouver par camion à juste une frontière de chez nous ? On nous parle d'écologie, on nous parle de machin, mais ça en fait partie. (silence) Même s'il y a des fleurs derrière, ça me donne moins envie. Du tout. C'est vraiment mon dépannage.

Ouais. Et vous, c'est quoi le magasin comme ça de dépannage dans lequel vous allez ?

Leclerc ? Ouais. Leclerc, Intermarché. ça va être dans ces deux-là, Leclerc, Intermarché, Lidl éventuellement. J'irais d'abord plus facilement à Lidl, bizarrement, qu'à Leclerc. Ouais. Et pourtant, bon, Leclerc, on pourrait penser que c'est un magasin de proximité. Il fait travailler certains producteurs locaux. Ouais. Mais bon, bizarrement, j'ai l'impression que ça m'est déjà arrivé de prendre des produits chez les deux ou les trois enseignes et ils tiennent moins longtemps, quoi.

Lesquels tiennent moins longtemps ?

Par exemple, des pommes, des bananes, des trucs que j'ai vraiment... Bon, j'ai besoin... Parce que, par exemple, demain, il y a des pancakes à la banane, il y a une banane sur le... Il y a une banane, quoi. Ouais.

Et, ouais, donc, les fruits et légumes que vous trouvez en grande surface, vous trouvez qu'ils tiennent moins bien que ceux que vous trouvez habituellement sur le marché.

Ouais.

En termes de conservation...

Oui, en termes de conservation.

Et selon vous, c'est comment ça se fait ? Bah, ils passent par un frigo, ça, non, non, non. Ils sont choqués en frigo avant d'être mis en sac et puis après ils sont déjà mis en sac et ils passent par la case frigo et après il n'y en a plus sur l'étage, on les ressort tout simplement. Du coup ils sont trop choqués, ils sont mal entreposés. Et du coup, manipuler rapidement aussi, il faut vite, vite, vite se rentabiliser, entre guillemets. Mais ça, beau, joli, pour donner envie aux consommateurs d'acheter. Donc, ils sont beaucoup plus manipulés que des fruits de Mon vendeur de pommes, [Nom du fournisseur], ici, à côté, va prendre soin de les enlever du pommier, mettre dans une cagette. Et quand moi, je vais aller chercher ma cagette, il n'y a que lui qui les aura manipulées. Vous voyez, donc, c'est plus délicat, on va dire.

Ce qui fait que ça me plaît, ce serait le frigo et en plus le fait qu'il soit trimballé, manipulé.

Oui, entrechoqué. Entrechoqué. Parce que bon, c'est le monde sur palette. Et puis après, c'est pris. J'imagine. Parce que bon, c'est pareil, c'est bourbourg. Tant bourbourg. Moi, je suis bourbourg, mais bon. Je prends soin de mes fruits et des légumes quand même. Quand je vais les chercher, je téléphone à... Je ne sais plus comment il s'appelle. [prénom] au début. J'ai besoin de 10 kilos de pommes. Je vais les prendre, je vais les mettre en cagette. La cagette, elle m'attend. Moi, je prends la cagette, je la mets dans la voiture. J'arrive ici, je la mets dans la chambre. Pas de manipulation de fruits. Manipulation de cagette, oui. Je ne veux pas dire que je roule gentiment. Je vais voir un lit de poules et une pomme se tosse avec l'autre. Forcément, celles-là vont s'abîmer plus vite. Elles vont aller travailler plus vite aussi. Je ne roule pas avec des coussins d'air sur la voiture. Et bon, non. Non, non. C'est bien que, bon, le transport va abîmer certains fruits. Ils seront travaillés en premier, puis c'est tout. Une fois qu'ils commencent à être abîmés, viens ici, toi, j'ai besoin.

OK.

Je ne vais pas les laisser pourrir dans la caisse avec les autres. Oui. OK. Mais elles n'ont pas le temps. Moi, quand j'ai besoin de pommes, c'est que j'en ai besoin, c'est... Généralement, quand je les prends, ça arrive qu'elles restent 2-3 jours dans la chambre froide, entre guillemets. Enfin, l'économa, on ne va pas dire chambre froide, parce que je n'ai pas de chambre froide. Mais il n'y a pas de chauffage dans cette pièce. C'est rare qu'elles restent plus d'une semaine dedans sans avoir été entre temps travailler. Parce que si j'ai besoin de pommes, c'est que j'ai des pancakes, j'ai de la compote, toute la gelée à faire. Généralement, j'appelle, j'ai besoin, elle me dit « Ok, pour quand ? » « Pour hier. ». C'est urgent ? Ouais, j'arrive. Voilà. Il me dit, je t'en ai préparé. J'ai dit, ok. Il m'a rien préparé. J'ai fait positif. Et puis, je vais travailler.

Donc, c'est vrai que ça, une semaine, c'est...

C'est parce que soit je suis pas là ou c'est parce que j'avais prévu de faire. Et puis, tout comme ça, il y a eu une urgence entre les deux.

Ouais. Voilà. Donc, c'est le max presque une semaine. Et puis, ça, ça laisse pas vraiment le temps pour ouvrir une pomme.

Non. Sauf si, comme je dis, ça peut être... Gentil avec les transports. Oui, mais voilà. C'est que je suis passée dans les nids de poules ou machin.

Et du coup, donc une conservation différente. Est-ce qu'il y a d'autres différences dans les produits que vous pouvez trouver ? Alors là, ici, c'est le commerce de proximité, mais c'est plus en grande surface après que votre dépannage. Est-ce que vous trouvez des différences dans le produit, autre que la qualité de conservation ?

Le goût, des fois, quand c'est produit intensivement, ça n'a pas de goût. Oui, il y a certains produits, vous trouvez, qui sont moins goûtés que les salades, par exemple.

Oui. Vous avez constaté une différence par rapport à...

Oui. Après, c'est pareil, ça tient moins longtemps aussi. Une salade qui a été coupée, vous avez, entre guillemets, sa tige, elle est blanche. Celle que vous prenez dans le commerce, parce que votre mari a décidé de manger une salade le soir, souvent, le cul est noir. Ça sous-entend qu'elle a au moins deux jours ou trois jours de stockage au frigo ou qu'elle a été coupée il y a trois jours.

Oui.

Après, vous devez la laisser dans l'eau pendant deux heures pour qu'elle récupère un peu.

Oui, vous faites ça, vous trempez la salade dans l'eau.

Oui, dans l'eau. Je la nettoie, je la mets dans l'eau avec un tout petit peu de vinaigre. Oui. Histoire de lui redonner un peu de vie, quoi. Qu'elle ne soit pas trop raplapla. Vous mettez la cuillère, c'est encore plus simple. Oui. Du coup, vous prenez la feuille et... Fiu, fiu, fiu. Bon. Mais si, je voulais de la salade ce soir. Tu as vu la tronche de ta salade ? Ça ne mange pas à moi.

C'est là le bon exemple pour montrer qu'il y a une différence par rapport à celle que vous achetez, tant qu'elle est plus fraîche.

Elle a été coupée le matin ou la veille et puis le cul est encore blanc. Parce que moi je n'ai pas le cul. Je vois, je vois. On vous les vend encore noirs. Les feuilles sont noires ou marrons foncés, on va dire, sur les étapes et puis ils vous les vendent encore. Alors que quand il est coupé, c'est deux jours. Et puis, il doit être cuit dans les deux jours. Sinon, c'est poubelle. Mais bon, les Parisiens, ils le savent. C'est ça.

Et du coup, la salade que vous avez achetée en dépannage parce qu'il fallait une salade, admettons, du coup, elle est finie à la poubelle. Qu'est-ce que vous ressentez à ce moment-là ?

Je suis en colère. Oui, parce qu'il m'a fait acheter une salade. Mon mari n'est pas fruit et légume. D'accord. S'il demande une salade, c'est qu'il en a envie. Donc je la lui prends, je l'y prépare. Et puis tout compte fait, je m'aperçois qu'elle n'est pas bonne à manger. Disons qu'elle est trop flétrie pour être mangée. Je vais râler.

Pourquoi vous êtes en colère ?

Parce que pour moi, il y a une perte d'argent, une perte de temps. Il a fallu aller l'acheter. Il a fallu la payer et qu'il s'est de l'argent mis à la poubelle d'une certaine manière. C'est comme si j'avais pris ma pièce et puis... Ouais. Des fois, je lui dis... "Ouais, mais j'en avais envie".

Ouais. Et alors, je vais demander une autre photo. Celle que vous voulez.

Ouh là ! C'est un endroit encore pire que le premier. Là, c'est vraiment... Je fuis. C'est encore pire que le dépannage. C'est vraiment l'extrême urgence. Voilà. Ça pourrait paraître joli. On pourrait dire qu'ils ont fait un effort au niveau d'harmonisation des couleurs. Oui. Mais ils acceptent.

À quoi vous pensez quand vous voyez ?

Grande distribution, tout de suite. Oui, grande distribution. Un magasin que je proscris. Ça peut donner l'illusion que c'est bien agencé, bien achalandé. Moi, je fuis ça. Ça me fait presque peur. Ça veut dire culture intensive ? Pareil, c'est des choses que... Pareil, aberration, sous plastique, qui ne respire pas, qui fermente, donc mauvaise conservation. Après, certains embouts marqués bio, c'est pareil, ça donne pas envie du tout.

Du coup, si là, vous vous projetez dans ce rayon-là, comment vous vous sentez au milieu de ces rayons ? Vous vous sentez mal ?

Oui. Je ne vais pas passer. Je vais passer à côté. Je ne vais même pas venir voir qu'est-ce qu'ils ont comme produit, qu'est-ce qui pourrait m'intéresser. Il n'y a rien qui m'intéresse, en fait. Oui. Donc, Donc il y a zéro plaisir à aller... Ah non, on a aucun plaisir. Aucun plaisir. Franchement, non. Là, c'est un magasin, je fuis. Je fuis.

Et donc, vous pensez quoi des produits, des fruits et légumes qui sont vendus dans ces endroits ?

Après, comme vous le disiez tout à l'heure, admettons que ce soit un Leclerc ou un intermarché, je sais que comme certains producteurs livrent pour les grandes surfaces parce qu'ils ont besoin du rendement et qu'il faut bien qu'ils en vivent, ça peut très bien être de bons produits et de bonne qualité. Pour autant, ça ne donne pas du tout envie d'aller acheter là.

Oui.

j'ai même pas du tout envie d'aller voir si les courgettes ou les aubergines elles sont bien de saison. je vois qu'il y a un peu tout mélangé. il y a des pommes et il y a encore des courgettes et des poivrons. et ben non quoi c'est plus de saison? c'est soit les pommes soit les courgettes. quoi? non? mais voilà c'est Bon, il y a une multitude de produits, mais il y a plein de variétés de pommes apparemment. Mais de l'autre côté, vous avez des salades, vous avez des oranges. Je suis désolée, mais les oranges et les pommes, ce n'est pas en même temps. Voilà, il y a un mode décalage, donc ça sous-entend que ça vient de loin. Et les courgettes et les poivrons, normalement, si les pommes sont sorties, les poivrons et les courgettes ne sont plus là.

Oui.

Donc moi, ça me choque. Je suis choquée qu'on puisse encore avoir des courgettes qui paraissent être au fond, des artichauts qui pointent leur nez. Des poivrons qui sont là, des artichauts, et des courgettes, poivrons qui sont là. Donc ça me choque à la base parce que... L'artichaut, il a sa place, le poireau a sa place, mais la courgette et le poivron ne sont plus. Donc, ça sous-entend que ce magasin achète des produits étrangers. Donc, culture intensive, sans référencement par rapport à nous. Nous, on a... Par exemple, le bio français n'est pas le bio espagnol, n'est pas le bio turc. Les normes sont différentes. Ça peut venir de Turquie, on va dire c'est du bio, mais oui, mais non. Parce que eux, ils ont encore le droit d'utiliser certains produits que nous n'avons plus. Pour moi, c'est pas correct vis-à-vis des producteurs français que ces produits-là soient là.

Oui, parce que... La législation, par exemple pour le faire du bio en France, des fois elle est très dure avec les producteurs qui essayent de le faire. Et à côté de ça, ça vous agace de voir qu'il y a des produits qui ne sont plus laxistes là-bas, qui ont le droit d'être vendus sous l'appellation bio quand même ? Et qui vont bénéficier d'être appelés bio.

Oui, et que les gens vont se jeter dessus parce que c'est bio. Alors que, ça se trouve, si on regarde bien la liste des produits phytosanitaires qu'ils ont utilisés, nous, en France, il n'y aurait même pas l'appellation culture raisonnée.

Oui. C'est ça.

Du coup, oui, là, ça me choque. Oui. Voilà. Donc voilà pourquoi pour moi c'est important de travailler par rapport aux saisons, de faire travailler au maximum les petits producteurs dans un rayon réduit quand même, et puis prendre au maximum des produits soit en culture raisonnée, soit en bio. Alors pas bio. bio comme la Grande Surface l'entend, parce que eux c'est plus un lobby C'est plus devenu une norme, entre guillemets, pour faire acheter les acheteurs. Mais privilégier les petits qui travaillent dur. Moi, je connais un maraîcher. Bon, là, c'est dans l'ambiance. Parce que dans quelques temps, je déménage. Oui, dans l'ambiance. Du coup, je commence déjà à prospecter au niveau maraîcher. Et celui avec qui j'ai envie de travailler, qui est d'accord de travailler avec moi, il est tout seul dans son hectare de terrain et travaille en bio et en culture raisonnée. Et il n'arrête pas de l'année. Il est tout seul. Donc j'ai plus envie de le faire travailler lui et de travailler ses produits que d'aller trois kilomètres plus loin. et... On a un paysan qui a des gros tracteurs et qui utilise des produits et qui a les jolies petites rédelles sur son tracteur et qui arrose plein de choses. Et pourtant, il dit qu'il est en culture raisonnée aussi. Alors je peux comprendre. Je ne vais pas aller fouiller et tout vérifier dans ses produits qu'il utilise. Mais je ne suis plus tentée d'aller avec celui qui travaille la terre de ses mains ou avec très peu d'outils que d'aller travailler avec celui qui a des tracteurs et compagnie.

Pour vous, c'est important de récompenser le producteur, mais qui travaille tout seul, qui travaille dur.

Oui, voilà.

Pour vous, c'est important d'aller du coup privilégier ce type de personne.

Parce que je ne suis pas seule acheteuse, encore heureuse, mais... Je lui donne une autre possibilité de manger peut-être correctement à sa faim tous les mois. Oui, ça c'est le faire vivre aussi d'une certaine manière. Oui, le faire vivre aussi, oui. Et ça, pour moi, c'est important. Comme mon producteur de farine, c'est pareil, je ne vais pas changer. J'en ai un autre dans le Morbihan qui me propose. Mais bon... Oui. C'est pareil, c'est la grosse usine. Oui. Ben non. Non.

Au-delà des... Du coup, le produit qui est produit par le producteur est sain par la manière dont il est cultivé. Pour vous, ça va au-delà du produit, c'est la personne qui est derrière.

Oui, c'est sa façon de travailler qui va m'aider à prendre la décision de savoir si je travaille avec lui ou pas. Oui. Alors, je ne prouve pas le tout bio, attention, mais je suis plus, je m'oriente plus vers ça et vers les cultures raisonnées. Oui. Moi, Une personne qui va mettre ses fraisiers en pleine terre et qui va me dire, [prénom de l’interviewée], je ne peux pas vous me donner de la fraise avant mai et juin, je vais dire oui, je l'attends. Et l'autre à côté qui va me dire, moi je peux vous produire de la fraise sous tunnel ou machin à partir du mois d'avril, je vais dire non. J'attendrai. parce que même si mes clients sont pressés d'avoir de la confiture de fraise, je vais leur dire que non, moi j'ai mon producteur, il ne peut me les proposer que fermé début juin. Après, c'est aussi une logistique, parce que du coup, toutes les fraises vont arriver au mois de mai, mai, juin, juillet, et bon, ça va être beaucoup de travail, mais en même temps, c'est à moi d'expliquer aussi aux gens que la fraise, ça se mange quand mai, juin, pas avant. Celui qui veut manger de la fraise en décembre, il l'achète, mais bon, je n'irai même pas lui piquer un manteau, même pas une chance. Non, parce que moi... comme elle est produite en pleine terre, elle récupère le terroir, elle récupère plein de choses, plein de vitamines, plein de choses. Et puis, elle a travaillé avec le soleil. Voilà, donc, tout ça, c'est important pour moi. Parce que, je prends le bon produit, le bon, le goût, le bon, le bon produit au bon moment.

Ouais.

Voilà. pas produire, pas acheter, même si mon entreprise, on me demande plein de choses, on me met plein le boum, ben non, non, non, c'est aussi nous, les producteurs, enfin, je deviens producteur, mais parce que je travaille, il y a plutôt transformateur de matière, c'est à moi d'éduquer aussi mes clients, et leur dire, ben non, moi je ne peux pas vous faire de la fraise au mois de décembre, alors ce que je peux vous faire, c'est, elle va être produite, je vais récupérer mes fraises au mois de juin, juillet, je vais en produire un certain nombre de pots, et éventuellement vous aurez la fin de mon stock, parce que je ne les aurais pas vendues, mais je ne vais pas vous faire de la fraise, parce que je l'aurai conservée au congélateur non plus, ça ne se fait pas, et je ne vais pas vous acheter de la fraise à 50 euros le kilo, pour vous faire joliment plaisir, pour avoir un pot de fraises à mettre sur votre table en mois de décembre, non ? Non, non, non.

Donc, vous êtes finalement intermédiaire entre le client et le producteur. Oui. Et du coup, vous vous donnez un peu quand même le rôle de... L'éducateur.

Oui, c'est ça. Oui, l'éducateur. Oui. Une chante, comme on dit. Non, non, non. C'est pas grave. J'assume parfaitement. J'assume parfaitement mes propos et mes actes. Il faut. Il faut.

alors dernière photo je pense ah ben ça c'est bien ça c'est solide c'est l'endroit où je préfère. j'aime bien entre le potager et ça c'est trop bien ça c'est un endroit où je kiffe j'aime autant toujours pas faire les courses. je reste pas très longtemps sur le marché non plus mais là j'ai beaucoup plus de plaisir à y aller parce que je peux voir les couleurs voir les produits, je peux les toucher, je peux parler avec le producteur, même si c'est pas forcément, ou le vendeur, parce que c'est pas forcément le producteur qui est sur place, mais au moins je peux poser des questions, je peux avoir des réponses aussi, c'est important, et puis je peux toucher, sentir, éventuellement goûter, parce que des fois je demande, vos oignons là ils sont comment ? Bah ils piquent, ou ils piquent pas, ou ils piquent pas, donnez-moi un goût ! Ça m'arrive de le dire ! Et puis, là, ça me donne plein d'envie, quoi. Envie de tout toucher, tout cuisiner, vraiment. Même si on voit que les feuilles de carottes, elles ont un peu jaune. Elles sont belles. Ça donne envie. C'est un lieu plaisant, c'est accueillant. Les gens, généralement, ont le sourire. C'est rare que sur un marché, vous ayez quelqu'un qui fasse la tête pour vendre ses produits, quoi. C'est rare. Généralement, même s'il n'est pas content parce qu'il n'a pas vendu grand-chose ou qu'il n'a pas produit grand-chose, généralement, il est content quand un client s'approche, lui dit bonjour. On ne fait pas fuir les gens. Du coup, ça donne envie de parler avec lui, de toucher ses produits. Tout de suite, il y a l'imagination qui se met en place. Même si j'y vais, par exemple, que pour une botte de carottes, Là, je vais peut-être être tapé par ces choux ou ces choux rouges. Je me dis, tiens, je vais peut-être faire un romanesco ou un truc à côté. Tout de suite, il y a l'imagination qui se met en place et mon travail. Tout de suite, il y a le cerveau qui dit, tiens, regarde [prénom de l’interviewée], il y a des pissenlits ou il y a des pensées. Tu peux peut-être mettre pour décorer ton assiette. Tu peux peut-être t'en servir aussi. Tout de suite, l'imagination se remet en place. L'envie de créer se remet en place. Là, autant je fuis (supermarché), parce que je n'ai pas d'envie, que dans ces deux-là, c'est plaisir. Franchement, c'est plaisir.

C'est plaisir de déambuler, en plus, côté inspirant.

Oui, voilà, tout de suite. Tout de suite, là, on pense aux carottes, on pense soit à des petites carottes tournées, comme des pommes de terre, mais on peut aussi penser à une potée, parce qu'il y a les choux, il y a les oignons, il y a les carottes. Après, il y a les petits oeufs dans le quartier, là. Il y a plein de choses, plein d'envie tout de suite. Là, mon imagination, elle revient au galop. Même si je n'avais pas prévu de rester peut-être trois heures au marché, je suis capable de le faire. Oui. Bon, je vais râler parce que mon planning, après, il est chourboulé, mais pas possible. Mais je ne regretterai pas mon expérience. marché, quoi.

Ouais.

Voilà. Là, je vais fuir. Je vais prendre juste ce que j'ai besoin. Bon, celui-là, je fuis. Là, je prends juste ce que j'ai besoin. Ouais. Donc, on va éviter. Donc là, mon planning, il est respecté. Non, c'est de l'or, mais c'est le cata, quoi. C'est le cata parce que je vais aller fouiller, je vais aller retrouver le... Je retrouve mon plaisir de... de toucher, de goûter, de sentir, de ressentir. Et puis tout de suite, comme je dis, l'imagination, le cerveau de mon cerveau de créateur va tout de suite se mettre en exergue. et du coup, qu'est-ce que je peux faire avec tel produit ? Lui va me donner. aussi, je lui ai demandé des conseils, lui va m'en donner, il va me dire... tel produit est bien, on ne va pas se marier avec tel produit, c'est un beau mariage, et du coup, pareil, l'imagination va se remettre en place.

Oui, le fait qu'on puisse discuter avec le vendeur ou le producteur.

Oui, il y a au moins une personne, là c'est un personnel, il n'y a que des gens qui ne connaissent pas leurs produits, qui mettent en rayon, Bon, certains connaissent leurs produits, on est d'accord. Mais ils sont plus là pour remplir les rayons et pour vendre plutôt que de conseiller. Là, si je demande qu'est-ce que je peux mettre, par exemple, avec une pomme Canada, il va déjà me demander c'est laquelle. On oublie. Bon, on oublie la question. Parce que là, si moi je lui pose avec quel fromage, par exemple, je peux associer une Canada, il est capable de me répondre. Parce qu'il aime ce qu'il fait. Là, c'est son métier, il l'a choisi. Là, c'est un boulot alimentaire pour ceux qui y travaillent. Alors, c'est un boulot alimentaire, c'est pour qu'il puisse payer en facture. Ici, c'est lui. D'abord, s'il fait ça, c'est qu'il aime ce qu'il fait. Sinon, il y a longtemps qu'il n'aura arrêté et puis il partirait dans une autre voie. Généralement, les producteurs de fruits et légumes, s'ils continuent, c'est qu'ils aiment ça. Et ça, ça fait plaisir parce que du coup, il a plaisir à m'expliquer comment il a produit, comment je peux l'associer, comment je peux le cuisiner, quel goût je vais avoir, etc. Et ça, c'est ça qui m'intéresse aussi.

C'est aussi parce que lui prend du plaisir que vous allez aussi en prendre après.

Oui.

Et puis après, moi, je vais pouvoir dire aussi, par exemple, j'ai besoin de carottes pour un repas. Je vais dire, ben voilà, elles viennent du champ de Monsieur [nom de l’exploitant]. Et puis, du coup, je vais faire sa promotion, entre guillemets, et puis lui ramener des clients peut-être ou pas. Les gens n'iront peut-être pas, mais ils ont reçu l'information. Je cuisine en local. Et c'est important. Les gens, ils reviennent sur la plupart des personnes qui mangent des légumes, cuisinés soit par un crépier, soit par un traiteur, soit par un boucher, peu importe. Ils aiment savoir d'où viennent les produits et ils ont plaisir à savoir qu'on cuisine avec des petits producteurs locaux. Et c'est important. C'est ce maillage-là qu'il faut refaire, qu'on a perdu. Mais qu'il faut refaire. Il faut remettre en place la relation petit producteur et traiteur ou cuisinier. C'est important de la conserver. De la retrouver et de la conserver. (silence) C'est de l'envie, d'accord. Mais c'est de l'envie. J'ai plaisir à les regarder. Ça me donne vraiment beaucoup d'envie. Beaucoup d'aller fouiller dedans. parce que là je fais les carottes mais je me dis qu'avec ça je peux faire un joli potage je peux faire une déco ça y est il y a l'esprit tout de suite

ici tous vos sens sont déjà en éveil alors que là pas du tout et du coup donc il y a le plaisir pour s'approvisionner en fruits et légumes. le plaisir est déjà là. est-ce que selon vous le plaisir il perdure après même dans la façon dont vous allez cuisiner.

je vais faire gaffe d'avoir le moins de déchets possible Là aussi, d'une certaine manière, parce que je les ai payés, je les aurais peut-être payés moins cher ou plus cher que chez le producteur, mais j'aurais moins de... Par exemple, si une feuille de salade est abîmée là, je dirais que c'est normal parce qu'elle a été au conserveur frigo. Puisque là, la feuille de salade, je vais essayer d'aller grignoter le maximum pour en perdre le moins possible.

Et pourquoi, du coup, cette salade-là, vous allez faire plus attention ?

Parce qu'elle a été produite avec amour. Il a pris du temps pour la mettre en place. Je l'imagine bien prendre des petites graines de salade, prendre du temps pour les mettre en petits pots, ensuite les arroser, les vérifier, les retransplanter dans son rang, mettre les bâcher, etc. Je me vois bien, je l'imagine bien le faire. Et puis, je l'imagine bien la veille de son marché, passer du temps dans son champ pour aller avec son couteau récupérer sa salade, en prendre soin, la tourner, la couper, la mettre dans ses cachettes vertes et puis les mettre dans son véhicule. Pas dans une unité de stockage avec des produits en pulvérisation pour les maintenir à bonne conservation pour le lendemain parce qu'elles vont être mises sur l'étal. Voilà. Il y a tout un processus que j'imagine bien en local et pas en grande surface. J'imagine qu'en grande surface, il va y avoir autant de plaisir. Mais ces salades-là qu'il va transporter et mettre dans la... parce qu'ils n'arrivent pas dans le rayon tout de suite elles sont d'abord mises en réserve. dans réserve elles sont mises en chambre froide et quand il n'y en a plus dans les rayons elles sortent de la chambre froide et elles arrivent dans le rayon. elles ont passé deux jours parce que sur le marché elles sont vendues le lendemain. du coup j'ai plus de respect pour ce travail là que pour la chambre froide parce que là il y a un respect de la nourriture qui est mise en place.

Oui, il y a un respect du produit en lui-même. Et ce qui, du coup, vous, va vous impacter aussi sur le respect qui en découle. Oui, qui en découle aussi. Même si, effectivement, personne n'aime jeter.

Oui, oui.

Mais là, d'autant moins.

Encore moins. Encore moins. Alors, pas forcément par rapport au prix, parce que généralement, quand on fait le psychose régulièrement, on s'aperçoit qu'en allant sur un marché, on ne paye pas plus cher qu'une grande surpresse. Oui. Généralement, les produits sont plus beaux, sont plus gros aussi. Parce qu'ils sont calibrés dans la grande surface. C'est-à-dire qu'ici, sur un marché, ils sont juste pesés. C'est au poids. Un chou, s'il pèse 1,5 kg, j'ai payé, admettons, 1,50 €. C'est 1 € le kilo. Dans la grande surface, j'ai payé 1,50 €, mais il est tout riquiqui. Il est calibré. Et l'un dans l'autre, avec un chou d'1,5 kg, je vais faire un chou farci, je vais garder des feuilles pour faire un chou farci. Je peux penser à faire un chou... Une beauté. Je vais trouver déjà plusieurs idées. Avec le chou de grande surface, je fais un repas. En plus, en même temps, le cul, je ne peux pas le manger. Moi, j'ai tendance à manger les tronions de chou. Il n'y a pas de perte. Il y a juste la petite rondelle que j'enlève. C'est tout. Tout le reste, c'est mangé. A la différence de la grande surface où le tronion est pratiquement noir, donc vous ne le mangez plus, il a perdu ses vitamines depuis longtemps. Souvent, certaines feuilles sont abîmées. Des fois, elles sont même sous-plastiques. C'est une aberration. Du coup, vous en perdez beaucoup plus que vous en conservez. Et vous ne faites qu'un seul repas. Vous avez payé le même prix, mais.. Alors quand vous faites vos courses sur le marché, vous vous apercevez qu'avec un beau chou, vous faites plusieurs jours. Je parle du chou, mais comme je parle des carottes, les carottes dans le commerce de proximité ou dans le commerce de surface, c'est très rare que vous ayez encore... Les fanes, elles sont souvent lavées. Il n'y a plus de fanes. Et en plus, elles sont mises en sachet. Elles produisent de la buée. La condensation de la buée. Et ce n'est pas rare qu'à l'intérieur, vous n'avez pas une petite brie. Oui. Ce n'est pas rare. Parce que là, sur le marché, il y a une belle potte de carottes avec ses fanes. Ensuite, ça fait penser à potage derrière. Potage de carottes ou potage de fanes. Donc, vous ne perdez rien en fait. Oui. Il y a peut-être juste cette feuille là qui est un peu jaune. Celle-ci. Mais vous ne perdez quoi ? Juste une feuille. Parce que tout se mange après dans la carotte. Même la petite racine.

Vous mettez tout quand vous faites un...

C'est pareil pour les radis. On a dit je mange la petite tige, la petite racine, tu la manges. Est-ce que s'il avait poussé, il aurait tout petit petit. Il n'y aurait plus rien. C'est parce qu'il y a un caillou qui l'a empêché de pousser plus loin.

Ah oui, ce serait...

Oui, il y aurait pratiquement rien. Moi, je mange. Si ça poussait, c'est que ça se mène. C'est ça. Non, mais sans plaisanter, je veux dire.

Donc là, toujours embêtant de perdre le produit. Mais là, c'est limite le fait de l'avoir payé. Donc mince, encore un produit que j'ai payé et qu'au final, je perds. Et ici, même s'il est payé, c'est pas vraiment ça qui va vous chagriner, entre guillemets. c'est tout ce que représente le produit derrière.

Le plaisir de le travailler derrière, après de le goûter, de le manger, de le travailler. Là je rémunère une seule personne aussi en achetant sur le marché, voire deux si jamais la personne a un vendeur. Je rémunère peut-être et je fais vivre une seule famille ou deux. Ici (supermarché), Je rémunère des grands... Oui. Multinationaux. Oui. Voilà.

Oui, donc en dehors même du plaisir, du produit qui va être de qualité, de ce qu'il représente même pour le travail, du coup, pour vous, c'est plus plaisant et en plus, une manière...

Une manière détournée de rémunérer le producteur et de le faire vivre tout simplement, quoi. Mais que lui, quoi. Parce que du coup, si on le paye aussi cher... Il y a une partie pour le magasin, il est beaucoup moins payé. Il y a une partie pour le magasin, il y a une partie pour le personnel, il y a les taxes, le restant, c'est les dividendes. C'est plus les multinationales qui vont bénéficier parce que la part magasin, la part salariale, elle est très peu dans les grandes surfaces. C'est plus les dividendes pour les actionnaires qui sont importantes. Ici (marché), il va aussi effectivement payer... Sa marchandise, ses semences, son travail. Mais il va surtout vivre avec une plus grande partie de l'argent récolté. C'est plus valorisant pour moi de lui donner 1,50€ pour sa botte de carottes ou son chou que de donner 1,50€ au Leclerc ou au intermarché, aux géants à côté.

Lui, pour le même chou, il aura 1,50€.

Il sera peut-être payé 10 centimes à côté. Et il devra s'estimer heureux. Oui. Si sa marchandise n'est pas refusée le matin quand il débarque parce qu'il y a une feuille de salade qui est peut-être un peu abîmée, la palette est refusée. Donc du coup, il ne sera pas payé. Donc j'ai plus de respect pour son travail là et plus envie de le rémunérer en achetant vraiment ses produits que d'aller dans ce type de magasin.

Et en plus, vous disiez qu'il y avait le plaisir d'aller au marché.

Oui.

C'est un lieu dans lequel vous vous sentez...

À l'aise. Oui. Vraiment à l'aise. Vraiment à l'aise. Hormis la période Covid qu'on passe. Oui. Mais c'est même plaisant. C'est plaisant parce que les gens, vous entendez tout le temps quelqu'un rigoler sur le marché. Là, vous entendez, c'est impersonnel. Il y a de la musique. Oui. Il n'y a personne qui rit. Vous riez dans un magasin, mais vous passez pour une folle. Là, vous riez sur un marché, vous allez entendre un rire en cascade derrière. Là, il y a de la vie. Là, c'est impersonnel, c'est... Pour moi, c'est un lieu de sauvetage à bannir. Même le gars avec son ne pas, il donne l'illusion d'être peut-être serviable et compagnie, mais pour moi, c'est trop pompeux, son ne pas. C'est trop standing, c'est trop... Son noeud papillon, il est de trop. C'est... Il est vraiment trop. Il donne l'impression d'être impersonnel et d'être froid et de ne pas avoir du tout envie de partager quelque chose avec nous. Et le lieu de grande surface, c'est la même chose. Alors, il y a plein de produits, mais on ne partage rien. Les gens, ils passent, ils ont leur caddie, ils regardent le produit, ils le mettent dans le caddie, et puis ils passent. Si vous avez le malheur de poser une question, vous regardez bizarrement, tu ne connais pas la réponse. Là, vous posez une question, on va vous répondre. On va vous rigoler. Même si c'est une question con, on s'en fout. Le paysan ou moi, je vais rigoler, et puis il y a un échange. C'est vraiment, là, l'impression de vie.

Donc ça, ça influencera après tout le travail que vous allez faire avec le produit.

Oui. Et puis le vendu. Et puis même dire, tiens, ce matin, j'étais au marché. Voilà, vos pommes de terre ou vos carottes, elles viennent d'un tel. J'étais sur le marché d'Avisi ou d'Artempion. Et j'ai eu plaisir à vous les choisir. Parce que j'ai choisi celles-là parce que j'étais bien conseillée. Et puis après, j'ai fait gaffe pour la cuisson, comme il m'avait expliqué. Voilà. Je vais retenir les consignes. Là, vous posez une question à celui qui a mis les carottes en machin, il ne va pas vous dire, attention, carottes nouvelles ou machin, il ne va pas vous dire, attention, cuisson délicate, en fait, à la vapeur. Là, il est capable de me répondre, vapeur ou plutôt gros bouillon. Et donc, du coup, je vais respecter le produit plus facilement ici. pour retrouver le goût pas trop cuit, pour trouver un petit peu de croquant. Et puis, je vais pouvoir le transcrire après aussi aux personnes qui vont recuire ou réchauffer ou manger. Derrière, la transmission de son savoir.

En plus, il y a peut-être un plaisir supplémentaire à manger un produit dont on sait d'où il vient.

Oui.

Et en plus, le fait que vous ayez des conseils pour le préparer, il peut aussi être meilleur grâce à ça.

Oui, aussi. Le fait que vous ayez tous les éléments pour le travailler derrière, pour le cuire, pour le travailler, pour le sublimer derrière. C'est ça. C'est un produit que vous achetez en grande surface. Combien de personnes vous conseillent à la caisse ou même dans le rayon ? Il n'y a pas une personne dans le rayon fruits et légumes qui est capable de vous expliquer quelle est la cuisson d'une pomme de terre. Maintenant, c'est marqué sur le sachet, comme on va dire pomme de terre frite, pomme de terre vapeur, machin, mais lui, il va me dire, non, plutôt celle-là, parce que c'est chair ferme, il vaut mieux prendre celle-là peut-être pour la cuisson, celle-là peut-être pour la cuisson des pommes de terre, par exemple, à l'eau, il vaut mieux prendre des pommes de terre pour faire des pommes de viande, peut-être prendre une pomme à chair ferme plutôt qu'une pomme qui est un peu plus farineuse. Lui, il est capable de me dire. Moi, je vais aller dans le crayon et je dis, vous avez des patates ? Il y a celle-là, il y a celle-là. Et puis, vous êtes là avec votre paquet. Je prends celle-là. Parce que c'est marqué sur le paquet, mais il n'y a plus personne.

Ce n'est pas que l'expérience d'achat.

on. Là, je fruis, là, je prends. C'est ça. Pas sectaire, [prénom de l’interviewée]. Je comprends tout à fait. Je ne suis pas du tout sectaire.

Pas du tout le même plaisir de s'y rendre qu'après de préparer et consommer. Est-ce que vous consommez de la banane ?

Oui. Je ne la consomme pas, je la travaille.

Vous la travaillez ? Vous ne la mangez pas ?

Si ça arrive, mais vraiment... Alors, ça va arriver quand... Voilà, pour demain, pour mercredi, pardon, j'ai... J'ai... J'ai... J'ai... Un pancake banane choc. C'est un paquet, mais par quatre. Donc, ça me fait l'équivalent de... 4 pancakes gros. comme ça. admettons toute la banane ne va pas être utilisée. on va dire un quart de banane ou la moitié d'une banane maximum la moitié d'une banane pour 4 pancakes. je fais quoi de la restante de la banane ? soit je la conserve dans l'hypothèse d'une prochaine commande mais bon je sais que je vais la laisser là. elle va pourrir, elle va s'abîmer. donc je vais manger le restant. voilà mais bon c'est pas un produit que j'achète pour consommer. C'est un produit que je mange pour ne pas perdre. La différence est là. Vous n'avez pas le choix d'en acheter pour... Pour les clients. Parce que pour eux, si je ne prépare pas ce qu'ils commandent, ils ne vont pas revenir. On est d'accord. Et puis, eux, dans leur tête, ces bananes, c'est en hiver. C'est pour eux un fruit de saison. C'est un fruit de saison exotique.

Oui.

Ce n'est pas un produit français. Alors, c'est français parce que Martinique, mais ça prend le bateau ou ça prend l'avion pour venir. Donc ça, ils n'ont pas pris encore conscience que j'évite de travailler sur ce type de produit.

À la base, c'est parce que vous privilégiez.

Voilà. Moi, je leur propose plutôt pancake citron et pancake orange parce que c'est français, ça vient de Menton. Là, d'accord. Mais il faut qu'ils aiment. Ce n'est pas le tout. Il faut leur proposer. Il faut qu'ils aiment. Il y a des gens qui aiment la banane. Du coup, je suis coincée. En fait, j'en ai qu'un.

Alors, là, il y a 10 bananes. C'est le même, mais à des stades différents.

D'accord.

Je vais vous demander de la ranger. Celle qui vous donne le plus envie, celle qui vous donne le moins envie.

Ah. D'accord. Pas la plus mûre ou la moins mûre, quoi.

En disant que, admettons que vous deviez la consommer d'abord comme la crue, quoi.

D'accord. Pas cuite, quoi. Non.

Après, on regardera justement à partir de quand vous avez envie de la cuire plutôt que de la manger crue.

C'est pas de la plante, ça. Non, c'est pas de la plante. C'est vrai. Déjà, ça, c'est bon. C'est bon. Ça, c'est limite. On se trompe. C'est bon. Moi, j'exagère déjà. Qu'est-ce que j'ai ? Voilà. Ça, c'est l'avant-dernière. Ça, c'est l'avant-dernière. Ça, c'est l'avant-dernière. Ça, c'est l'avant-dernière. Ça, c'est l'avant-dernière. Ça, c'est l'avant-dernière. Ça, c'est l'avant-dernière. Ça, c'est l'avant-dernière. Ça, c'est l'avant-dernière. Ça, c'est l'avant-dernière. Ça, c'est l'avant-dernière. Ça, c'est l'avant-dernière. Ça, c'est l'avant-dernière. Ça, c'est l'avant-dernière. Ça, c'est l'avant-dernière. Ça, c. Voilà. C'est vite fait.

Si on essaie de les mettre. Je vais prendre en photo votre rangement. Ça, c'est votre préféré ? Oui. À l'inverse, c'est bon. Oui, voilà. Je trouve que vous allez en mettre un petit peu. J'ai fait ça... Oui, oui, d'accord. On va... J'ai peut-être dû échanger. Non, c'est bon. Ça va ? Oui, c'est bon. Je vais prendre ma photo. Je vais mettre que c'est... C'est la meilleure. Alors... Je vais vous demander, du coup, pour chaque banane.

Alors, celle-là. Celle-là, elle est... Donc, le rond jaune. Ouais, jaune orange. Jaune orangé. Mûre. Elle est mûre. Elle est mûre. Elle est pleine saveur. Ouais. C'est le top au niveau gustatif. Alors, l'aspect visuel, on pourrait se dire, ah, t'es bon pour mettre à la poubelle. Mais non, mais non, c'est... Elle est au stade de sa maturité bonne à consommer. Là où, quand je vais l'ouvrir, elle va être un peu moelleuse, elle va se détacher, elle va être un peu collée aux doigts. Et c'est là qu'elle est la meilleure. Pour moi. On est bien d'accord que c'est pour moi. Mais c'est comme ça que j'adore. Là, j'ai juste besoin de... Je n'ai pas besoin d'une fourchette pour l'écraser. Je n'ai pas besoin de rajouter de sucre. Si j'avais besoin de faire une petite compote de banane, là, je la prends, je l'accroche et j'ai les notes de vanille qui reviennent, les petits grains qui sortent. Vraiment, j'ai un plaisir immense à la consommer.

OK. C'est la banane parfaite. La banane parfaite.

Celle-là, c'est la deuxième, sa copine, le carré bleu. Elle est en cours. Elle sera mangée dans la semaine. Vous attendez encore un peu. J'attends encore un peu pour la manger, oui. Elle n'est pas suffisamment mûre pour moi. Elle est en cours de maturité.

Ok.

Elle est jolie. La numéro, la ronde blanche, j'attends encore une semaine avant de la manger.

D'accord.

Même si sa tige, elle commence déjà à être moisie, le bout du haut, pour moi, ce n'est pas suffisant. La peau n'est pas suffisamment... Les marbreuses ne sont pas... Elle est que marbrée. Elle commence à revenir en maturité. Sa copine, le carré orange, elle est en cours de maturation. C'est vraiment le premier stade. La queue est encore jaune. Elle n'est pas du tout mûre.

Pour vous, quand la queue est jaune, c'est que ce n'est pas mûr.

Il faut que la queue soit noire.

Donc là, vous n'avez pas forcément le plaisir à manger une banane ?

Non.

Vous voyez la copine qui est là. La queue est jaune. Celle-là, elle ressemble à celle-là. La queue est jaune. Elle a quelques traces. Là, les jambes commencent à les toucher. Ici, c'est mûr. Parce que c'est mûr. C'est les extrémités qui sont... Oui, mais c'est surtout parce que quelqu'un a joué avec. Quelqu'un a tiré sur la queue. Du coup, ici... Ici, on voit bien que c'est que là parce que la personne a touché comme ça pour rattraper. Et ici, c'est parce que souvent, les gens, ils appuient pour voir si c'est mûr.

D'accord.

Sauf que ce n'est pas là que c'est mûr, c'est là où ça mûrit.

D'accord.

Là, c'est sec. Donc là, la banane, elle garde tout son jus, sa maturité. Tous ses vitamines sont à l'intérieur. Ici, c'est sec parce que ça fait déjà plusieurs jours qu'elle a été découpée. Mais ici, c'est abîmé parce que la personne a foutu son pouce pile poil dessus.

D'accord.

ça correspond bien c'est pas moi mais c'est presque. c'est parce qu'on a tendance à les prendre par la queue. donc normalement on doit prendre la tige et on doit la découper. donc là elle a été arrachée. c'est une personne qui a pris. moi j'en ai pris qu'une une banane parce que j'ai que un produit à travailler. elle sera travaillée mercredi parce que j'ai pas le choix sinon je l'aurai pas mangée. Celle-là me correspond à celle-là. Au carré orange. Et franchement, pour moi, ce n'est pas mûr.

Et du coup, tu as quel goût quand ce n'est pas mûr comme ça ?

Je la trouve farineuse. Pour moi, elle n'est pas... Ça n'a pas un goût de banane. Autant celle-là est à l'exact... Orange, exactement palé, que le carré orange, c'est banane quand c'est... C'est une banane. On me mettrait un bandeau sur les yeux. Je vous dirais oui, c'est une banane. Mais elle n'a pas de saveur. On ne s'explique même pas pour les verts qui arrivent après. Donc, le triangle vert, c'est une banane qui vient juste d'être sortie de sa main, on va dire. Mais bon, qui n'est pas mûre. Pareil, je ne mange pas. je vais acheter. c'est peut-être à ce moment là que je vais l'acheter ouais mais je la mange pas ouais. par contre celle là bon celle là c'est limite poubelle. quoi c'est vraiment dernier lieu avant dernier lieu le triangle blanc et le rang rose c'est limite poubelle. quoi c'est trop tard là je ne les mange pas. alors j'enlèverai la peau peut-être sur le triangle blanc pour voir quand même l'aspect intérieur et je goûterai quand même. mais sur le rond rose je n'essaye même pas parce que elle est bien flétrie et puis elle est bien moisie. donc du coup j'essaye même pas. le rond rose va à la poubelle directe mais le triangle j'aurais quand même un doute. triangle blanc j'essaierai quand même d'ouvrir.

Là, vous la mangez crue. Vous pouvez la cuisiner ou la manger crue. Ici, dans ce cas-là, vous la cuisinez ou vous la mangez crue aussi.

Après, ça va dépendre de comment est son aspect à l'intérieur. Si j'ai besoin de faire une compote de banane et que quand j'ai goûté crue, c'est bon, je la cuisinerai. Celle-là, c'est jeté. J'essaie même pas de voir comment est-ce qu'elle est à l'intérieur.

C'est l'aspect...

Oui, et puis... Les moisissures, le bout est moisi. Ici, c'est moisi. Là, c'est moisi. Il y a beaucoup de moisissures sur le produit. J'imagine qu'elle a été bien malaxée, bien pilétrée. Et donc, il y a du jus qui est sorti. Et donc, du coup, ça moisit. Ça ne donne pas du tout envie de la manger. Après, je me trompe peut-être, mais je n'ai vraiment pas d'ouvrir. Bon, celle-là, le triangle bleu, pas du tout mûr non plus. donne pas envie. et du coup les deux autres carré blanc et l'autre celle là c'est la sorte de bananier ça. elle vient dans le bateau le triangle rose elle est cueillie pour aller dans le bateau et les autres elles mûrissent pour le. elles commencent à mûrir pour le dans le bateau elles arrivent sur nos étals et c'est comme ça qu'on les prend la plupart du temps. mais c'est pas comme ça qu'on les consomme quand même.

Donc pour vous c'est à partir déjà du rond blanc que le goût est déjà moins bon ? Là c'est ok ?

Oui. Disons que pour moi elles ne sont pas mûres à partir du blanc. Le goût commence à arriver dans les deux dernières stades.

Et du coup c'est assez rapide en fait.

Oui ça peut être en une journée. Oui. On peut voir que ça peut aller... D'une journée à une autre, ça peut vite passer. Oui, c'est de là, même dans la journée. Et celle-là, le lendemain, elle est morte. C'est clair. Ça peut vite aller. Là, c'est trois jours. Maxi, entre ces quatre-là, c'est trois jours.

Une fois que ça a mûri, ça...

Oui, vous voyez, celle-là, pour demain ou après-demain, elle est souple. Mais pour moi, elle n'est pas encore suffisamment mûre. Disons qu'elle n'aura pas de goût. Elle aura un goût de banane comme la plupart des gens aiment. Mais avec mon palais exacerbé tous les jours, pour moi, elle n'a pas encore le goût de banane, vraiment banane. Vraiment, qui va me sublimer le palais. Oui, c'est l'intensité. C'est une question d'intensité aussi. C'est une question d'intensité parce qu'après, il va être cuit dans une pâte où il y a là aussi du chocolat. Du coup, il faut que tout le monde retrouve le goût de la pâte, le goût du chocolat et le goût de la banane. Pour moi, une banane que j'achèterais par exemple verte comme ça, qui va être obligée d'être coupée au couteau, dans deux jours, je la coupe à la main. Je peux l'écraser avec mes doigts. Celle-là, il faut un couteau et une fourchette pour la manger.

Oui, tout à fait.

Donc celle-là, elle n'a aucune saveur. Oui. La triangle rose, elle n'a aucune saveur. Elle doit être coupée au couteau. Et puis, il n'y a pas de goût, il n'y a pas de sensation, il n'y a rien. Il n'y a rien qui se passe. Du coup, il achète comme ça. Il dit, oh, une banane. Maman, tu prends la banane. Non, je n'achète pas, elles ne sont pas mûres. Je vais commencer à les acheter à partir de là. Donc, à partir du triangle rose.

Et est-ce que sur lui à un moment donné où elle perd ses apports nutritifs ou alors elle les a pas encore ?

Tout ça des apports, ça y a pas, ça y a pas, ça y a pas, ça commence à venir, ça et ces quatre là sont pas, ça commence à les avoir et là ils sont au maximum, et là ils sont perdus.

Ah d'accord, donc à partir du carré orange ? Là, les apports nutritifs arrivent là. Oui. Et par contre, après, ils disparaissent aussi, vous pensez ?

Ils sont peut-être encore là, mais en disant que moi, je ne les mange pas, du coup, je ne les profite pas. Donc, on est bien d'accord. Mais maintenant, je ne veux pas dire qu'ils n'y seront plus. Une pomme qui est pourrie, je pense qu'il n'y a pas beaucoup de personnes qui mangent pour récupérer les apports nutritifs d'une pomme pourrie. Et une banane, ça va être pareil. Je veux dire, les apports nutritifs sont peut-être là, mais je ne ferai pas l'effort de manger, quoi. Donc, à partir du triangle blanc et du rond rose, je suis réticente à manger la banane, on va dire.

Et qu'est-ce qui fait que vous ne voulez pas l'effort de la manger ? Comme j'ai dit sur le triangle blanc, si son aspect extérieur est moche, on va dire, mais qu'à l'intérieur, après que j'ai enlevé la peau, parce que j'ai quand même pris le temps de vérifier que si je peux encore l'utiliser, si elle est bonne gustativement, je vais la manger, je vais la cuisiner. La carré rose, je ne vais pas chercher à savoir. Là, je ne vais pas chercher du tout, même si elle est en pleine maturité, parce que pour moi, elle est limite pourrie. Je ne vais pas tenter de la manger, même si on me dit, mais si [prénom de l’interviewée], c'est là qu'elle est la meilleure, je vais avoir beaucoup de réticences. À la manger. Maintenant, si une personne me bande les yeux, pareil, et enlève la peau, la cuisine, et me la donne à manger, peut-être que j'aurais quand même plaisir à la manger. Mais parce que je n'aurais pas vu son aspect extérieur non plus avant.

Vous pensez que l'avoir vue un peu selon vous, comment ça te moisi, ça peut jouer sur après le déplaisir à la manger.

Oui, oui, oui. Oui, si on vous dit après, mais en fait, c'était celle-là. Je ne refuserai pas d'avaler ou d'avoir la satiété quand même. Mais je n'ai pas dégonflé quand même de m'avoir donné celle-là à remanger. Voilà. Même si j'ai pris plaisir à la manger.

Je vois.

Je pense que si l'expérience en fait tous, on aura quelques reproches à faire aux cuisiniers. Oui, c'est vrai. Et Même si, effectivement, c'est peut-être à ce stade-là qu'elle est la meilleure au niveau satiété. Peut-être. Mais je ne tenterai pas l'expérience. Ils m'ont plein gré. Oui. Il faudra me bander les yeux et peut-être m'attacher les mains. Et ouvre la bouche, je te donne quelque chose à manger.

Et vos réticences, elles sont gustatives ?

Non, non, juste visuelles. Là, c'est juste visuel. Oui.

Mais c'est après, pourquoi vous la mangerez ? Vous n'avez même pas envie de la manger.

Là, ça ne me donne pas envie. Oui. On est d'accord. Maintenant, si quelqu'un arrive à la cuisiner et que je ne vois pas le produit et que je trouve bon, et tu me dis que c'est celle-là que j'ai donné à manger, j'aurais été gonflée. Moi, j'aurais mis à la poubelle. Moi, je n'aurais pas pris le temps de la cuisiner. Et j'ai peut-être tort.

Parce que selon vous, en termes de goût, elle est trop... Elle est trop mûre pour moi. Oui. Puis elle risque d'être trop forte en goût et peut-être pas avoir qu'un goût de banane. Peut-être un goût de moisissure derrière aussi. C'est plus ça que son aspect ne me donne pas du tout envie de la consommer.

Oui, ce que vous pensez derrière, c'est...

C'est vraiment la dernière limite gustative.

Je vois. Est-ce que, selon vous, il y a un moment donné où la banane peut, là, être potentiellement, dangereuse pour la santé, ou pas ?

J'aurais dit celle-là, oui. La banane... Oui, la banane verte. C'est le triangle rose. Oui, celle-là, oui.

Selon vous, quand elles sont pas mûres, elles peuvent...

C'est peut-être indigeste. C'est au niveau de la digestion qui risque peut-être d'être compliqué... Notre corps a peut-être faculté à magasiner plein de nourriture et à transformer la nourriture. Je pense qu'elle va être indigeste à être consommée verte comme ça. Après, pareil, toujours juste mon avis perso. Après, je pense que nos enzymes dans l'estomac sont plus capables de modifier, enfin pas modifier, mais de...

Oui, de travailler.

J'étais en train de dire modéliser, mais non, ce n'est pas modéliser. Il y a des moments où j'ai perdu cette mémoire des mots. Nos enzymes transformaient la matière qu'on leur donne en potentielle énergie. Je pense qu'à partir de ce stade-là, carré orange, et éventuellement peut-être même encore ici, carrément, on va dire... C'est faisable. Triangle vert, oui, commence triangle vert. Là, ça commence à nourrir. Mais les trois premières, triangle rose, triangle bleu et carré blanc, ça commence déjà... Ces trois-là, c'est plus indigeste pour moi.

C'est mangeable, mais... C'est mangeable, mais il va plus de mal à... À digérer.

À digérer, quoi, voilà. À travailler... À faire travailler les enzymes. C'est bon.

Alors, j'ai fini. Si vous êtes attendus, il ne faut pas nous terminer.

Il y a une heure et demie, c'est bon.

Il est quelle heure ? Il est 4.

Ah ouais. Tiens. Je les reconnais. ah oui les jolies photos vous avez vu quand je fais des produits à la poubelle c'est vraiment pas tout le morceau en entier mini rhubarbe c'est vraiment tout petit bout.

est-ce que vous pouvez me raconter l'histoire de ces morceaux de rhubarbe?

j'étais en train de préparer de la compote de rhubarbe Ouais. Et j'étais en train de la nettoyer, puis on a enlevé, sur les tiges, on a enlevé des morceaux pour que ça soit digeste. Et du coup, moi, je me suis aperçue qu'il y avait des morceaux qui étaient abîmés. Donc, du coup, je les ai pris en photo avant de les mettre à la poubelle.

OK.

Enfin, ils n'ont pas tous été à la poubelle. Je crois que ce morceau-là a été mis au congélateur pour être à l'aise. Mais je crois que les autres ont été à la poubelle direct. OK. Et après, les citrons, pareil, ça s'abîme souvent que d'un côté. Hum. Du coup, moi, j'enlève que la partie qui est abîmée. Je me sers du reste. Donc là, en l'occurrence, c'est deux citrons. Le premier, je n'ai pas eu le temps de vous faire la photo entière avant de vous montrer où j'avais coupé. Donc après, j'ai montré que sur le deuxième, c'était abîmé. Oui.

Donc les citrons, là, vous coupez au... Je coupe vraiment au ras, ras, ras. Et j'utilise que même si la partie... pour laquelle j'ai coupé au rat vraiment, même si l'autre côté paraît potable et est un peu abîmé, j'utilise quand même. Ok. Oui. Voilà.

Ok. Et ils venaient d'où ces produits ?

Alors oui, c'est des citrons que j'avais achetés par le biais d'une association qui s'appelle [Nom de l’association], avec qui je travaille et qui font venir des produits. Là, c'était une opération sauvetage d'un petit producteur italien. Donc, ils avaient fait revenir des citrons et des oranges d'Italie.

C'est quoi le nom de l'association qui fait ?

C'est [Nom de l’association] à Brest.

Les [Nom de l’association]?

Les [Nom de l’association]. C'est une association d'usagers de quartier qui achètent des produits de bonne qualité auprès de petits producteurs comme moi. Tous les mois, une fois par mois. Il y a une vente. Là, vous ne le voyez pas. Donc là, il y a Kerango et Kaba Keroréen, mercredi 8, c'est livré. Donc voilà. Ok. C'est par les centres sociaux de Brest. D'accord. Donc du coup, là, ils avaient fait une opération chez Kerango, donc l'opération citron et orange. Donc j'avais acheté mes citrons chez eux.

D'accord. Vous les avez perdues ?

J'en ai perdues de demi. De demi. Le reste a été utilisé. Je peux montrer ce qu'il reste des citrons. Il devait servir... Les zestes. Sans restants. Et le jus. C'est le jus de citron qui a servi à cuire les zestes.

C'est un jus de citron, ça ? C'est un jus de citron. Ah, ouais.

Et ça, ce sont les zestes. OK. Donc, voilà. C'est un jus de citron qui est servi... Voilà. Jus de citron.

Chouette. C'est marrant, la couleur du jus de citron.

Parce qu'il y a un peu de sucre. Oui, c'est un sirop de citron. Et qui me sert, moi, à fabriquer mes pancakes derrière. Ah, oui. Voilà. Donc, ils sont confits, quoi. OK. Donc, ça a servi à ça. Ouais. Voilà. Et donc, c'est ça qui sert pour faire... Des claques au citron ou des peines claques au citron.

C'est réutilisé.

Il n'y a pas de perte. Je n'utilise pas tout de suite pour faire ça. Généralement, le jus est récupéré pour faire le sirop. Et le zeste, le ziste, le ziste est enlevé parce que c'est trop amer. Mais la pulpe est récupérée. Elle part en même temps que les zestes. Il n'y a pas que les zestes. Il y a aussi la pulpe et le citron que je mets dedans. J'estime que tout est à manger dans le citron.

Oui, du coup, ça a l'air normal.

Après, je peux vous le faire goûter. Vous verrez que c'est bien acide. J'imagine. C'est bien sucré ? Oui. Je peux vous le faire goûter. Vous verrez la différence. Il y a un caca à manger, pas trop l'autre.

Ça, c'est le sirop. Ça, c'est le sirop. Ça, ce sont les zestes. Faites-vous plaisir.

Oui, bien sûr. C'est gentil. Merci.

Comme ça, vous verrez ce qu'on peut faire. Bon, t'as aimé ? Je me suis bien amusée sur l'acidité du citron. Ah oui. Je le laisse bien. Il est confit dans le sucre, mais...

C'est bon quand même. C'est bon quand même, mais d'accord. Mais... C'est vrai qu'on connaît. Là, le citron, on crampe le citron, quoi. On crampe le citron. Ça m'est bien. Et là, c'est un sirop.

Et là, c'est un sirop. C'est bon aussi.

C'est bon aussi, c'est agréable. Mais c'est différent. C'est très bon.

Et vous voyez à quoi je m'amuse. C'est à partir de ces citrons-là. C'est à partir de ces citrons-là. Et on est d'accord, c'est bien du citron. Même si la couleur fait penser à une pluie de miel. Oui, mais ça donne un... Donc c'est ça qui me sert après pour les préparations derrière. Donc après je ne le passe pas au mixeur, je le redécoupe à la main. Parce que moi c'est quand même grand, ça ne cuit pas tant que ça. Le sabot est cuit mais il n'est pas quand même très moelleux. Il y a quand même de la mâche donc je suis obligée de le redécouper en petits morceaux pour l'incorporer dans les pancakes ou dans les cakes. Après je m'amuse quoi.

Oui, donc vous, il vous réveille bientôt. le produit quoi.

Rien n'est perdu quoi. Rien ne se fait. Sauf la partie moisie quoi. Parce que même si dans mon économa là-bas, c'est bien frais, il a pu être tossé. Certains vérifiaient aussi dans le transport à l'Italie. Ici forcément, ça n'a pas été mal non plus. Ça a dû être un peu choqué. Moi, je peux l'avoir très mal entreposé aussi. Je peux l'avoir piqué ou je peux l'avoir aussi... Ce n'est pas que la faute des autres. Je reconnais que j'ai peut-être attendu un peu trop longtemps avant d'utiliser aussi ou parce que... Occuper, faire autre chose. Voilà. Mais le reste a été utilisé. Voilà, il est là.

Parfait.

Du coup... Il n'y a pas trop de pertes, on va dire. Oui, ça. Il y a pas trop de pertes, là. Là, c'était un sauvetage. Je ne refuse pas non plus systématiquement des produits étrangers. La preuve, la banane, elle est bien là. Qu'est-ce que marquais-tu ? Guadeloupe et Martinique. Forcément, elle est venue par avion ou par bateau.

C'est sûr. Oui, c'est ça. C'est un produit en France. Oui, un produit en France quand même. C'est l'économie française. Oui, mais voilà. Ça voyage.

Oui, mais voilà. On va dire que c'est un sauvetage de bananes. Alors, d'un autre si je veux, après je vous laisse reprendre toutes vos activités. Disons que cet après-midi, c'est complet. Comme je vous ai dit, je ne vous cache pas que cet après-midi... Oui, c'est complet, quoi. C'est complet. C'est vous, d'abord. Après, c'est [prénom 1]. Après, c'est [prénom 3]. Après, c'est les gens. Et après, c'est [prénom 1]. C'est [prénom 2] à l'analyse. Et c'est jusqu'à 19h, ce soir.

Oui. Ça fait pas mal.

18h45, dernier délai. 19h45.

C'est pas mal.

Quand je lui ai dit ce matin que c'était un peu plus calme, c'est un peu plus calme. Il y a eu que deux cuissons de caramel à faire ce matin.

C'est bien quand c'est un peu calme quand même le matin.

Surtout lundi matin. Oui. On veut jeter le petit ronfle.

Alors là, l'idée, c'est qu'il y a des photos, soit de préparation, de moyens de conservation et de moyens de jeter aussi.

D'accord.

Et l'idée, c'est que vous mettez de côté celles qui vous parlent, ce que vous serez capable de faire, qui ressemblent à ce que vous faites. D'accord. Et celles qui ne vous parlent pas, vous les mettez de côté.

Oui, ça me plaît. Oui, alors ça c'est plus délicat. Mais oui, pourquoi pas. Ça, il y a encore des choses à manger. Alors ça, c'est un compost. Oui, mais il y a des choses qui auraient dû être mangées, utilisées avant d'être là. C'est possible.

C'est ça.

Parce que vous faites beaucoup de chips de fruits, de légumes avec ce qu'il y a là. V

ous en faites, vous, des chips de légumes ?

Oui. Voilà pourquoi je garde. Je regardais avant de jeter. Vous auriez jeté tout ça. Ce qui aurait été jeté dedans. Alors, les coquilles d'oeufs. Vous voyez le citron, là ? Le citron, je ne suis pas d'accord parce qu'à la différence de ce que j'ai fait, moi, je n'aurais coupé que la partie endommagée. Et c'est que la partie endommagée qui aurait été dans le compost. Oui. Mais la partie, elle est là, elle est dans les bocaux. Donc là, pour moi, c'est une aberration. Il y a encore de la matière à récupérer, même si c'est un compost. Ce que beaucoup de personnes jettent aussi, c'est feuilles de poireaux. C'est ce qu'on fait le bouquet garni avec. Et moi, j'en fais du potage aussi. Voilà pourquoi moi, je n'ai pas vu le compost tout de suite. Je n'ai pas vu l'emballage, l'entourage tout de suite. J'ai vu tout un amoncellement de choses qui sont consommables. Et les œufs, les coquilles d'œufs, alors moi, je ne le fais pas systématiquement, mais je sais qu'il y a des personnes qui le font, qui concassent et qui pulvérisent les coquilles d'œufs et qui les réintègrent à leur nourriture. Ah ouais ? Pour des problèmes de calcium, oui.

Ah ouais ?

Voilà. Donc, les œufs ne vont pas systématiquement au compost non plus. La coquille ne va pas nécessairement au compost. Voilà, ne va pas nécessairement au compost. On peut la manger. Voilà, on peut la manger. On peut, pour se recalcifier nos œufs.

Ah ouais ?

Voilà, donc... Voilà pourquoi elle est là. Complètement. Voilà l'explication. C'est dangereux. Oui, bien sûr. Là, c'est pareil. L'épluche, les oignons. C'est dommage parce que là, il n'y a pas de tri. Oui. Mais c'est pareil. Les oignons et une partie des épluchures peuvent faire les roux, les bouillons de légumes.

OK.

Dans les oignons, on peut récupérer la peau des oignons.

OK.

Donc, ça, oui. Alors, celle-là, je vais la mettre de côté parce qu'il y a des emballages à l'intérieur aussi, au carton. Là, c'est vraiment une poubelle. Mais à la base, non.

OK. Donc, on l'enregistre très bien. Service. Service. OK.

Alors. Bon, ça, j'ai donné l'explication. Ça, ça peut être votre poubelle, admettons, votre poubelle. Elle ne sera pas comme ça, mais...

Oui, ça ne sera pas vraiment ça dedans. Non. Mais oui, ça...

Là, oui. Non. Excusez-moi. Ce sont les oeufs qui arrivent. Vous voyez, je vous blâme. ..... Bon, ma conserve, elles sont là. La conserve aussi, là, en même temps. Vas-y, rentre, c'est bon. Bonjour. Là, j'ai une série. Vas-y, rentre. Tu peux rentrer, tu peux venir. Ça ne dérange pas. Bonjour. Charlotte, je t'ai dit que pour la poubelle connectée, je faisais partie d'un programme FOODRest. Du coup, le dernier entretien pour expliquer comment je travaille et pourquoi je jette certains produits, pas d'autres, et pourquoi je garde plus d'autres. Voilà. C'est ça. bon alors ça je mange, je mange pas pareil.

en fait l'idée c'est que ça ce sont des photos que les participants ont jetées et que moi j'aurais peut-être pu faire quelque chose avec l'idée c'est de dire soit ici là c'est poubelle ou soit non moi je le fais différemment. dans ce cas là qu'est-ce que je fais?

d'accord bah ça c'est la valeur. ok ça l'est encore belle. la tomate là ouais. oh bah oui ils l'ont pas jetée.

bah apparemment j'ai pas encore fait à personne.

Bah pourquoi ? B

ah je sais pas encore. Je sais pas.

Oh bah disons. Bah disons. Du coup, la tomate, vous serez capable de la manger en... Ah bah oui, en salade. Ah bah oui, elle a rien. Celle-là aussi. Les carottes aussi. Ah oui, attends. Je vais en potage. Potage éventuellement. Bah la banane, c'est comme ça que j'y mange. Ouais. celle-là c'est limite mais il y a encore un bouquet consommable. donc du coup celle-là elle est plus en soupe en soupe. ouais c'est en soupe aussi. par contre ça c'est plus en conserve. là parce que c'est ce que je fais celle-là c'est pour une score pas mal celle-là elle est pas mal celle-là. non franchement non pas là compost plutôt et celle-là il y a encore du consommable. celui-là c'est sous.

Au final, il n'y a que la tomate qui est moisie.

Sinon, le reste est consommable encore. Vous en avez fait quelque chose avec. Tu penses qu'il n'y a pas eu grand-chose dans la boîte ? Oui. Il y a eu que les citrons.

Les bouts de citron. Parfait.

C'est sérieux. Je dis que je fais des études, je rentre dans des études, je ne vends pas. On pourrait penser, [prénom de l’interviewée], du bague, des conneries, du... Des expériences. Je rentre dans des expériences scientifiques.

Parfait.

Là-dedans, il y a encore pas mal de conneries qui sont faites. Vous auriez pu faire autre chose. Moi, j'aurais fait différemment.

Alors, du coup, on a terminé. Normalement, l'idée, c'est que je repars avec la poubelle, vu que l'étude est terminée.

**Household F04, Interview 1**

00:00:03
*Mme. 1:* C'est parti. Alors [euh] [tilali ?] donc, dans un premier temps, je vous propose un petit jeu de devinettes.

00:00:10
*Mme. 2:* D'accord.

00:00:11
*Mme. 1:* Donc je vais vous demander de penser à [euh] un légume ou à un fruit--

00:00:14
*Mme. 2:* Ouais.

00:00:15
*Mme. 1:* --que vous aimez tout particulièrement.

00:00:17
*Mme. 2:* D'accord.

00:00:17
*Mme. 1:* Vous allez penser à ce légume ou à ce fruit sans jamais me révéler son identité.

00:00:21
*Mme. 2:* D'accord.

00:00:22
*Mme. 1:* L'idée est que je le devine, uniquement grâce à votre description.

00:00:26
*Mme. 2:* D'accord.

00:00:26
*Mme. 1:* Est-ce que vous en avez un en tête ?

00:00:28
*Mme. 2:* Ah oui.

00:00:30
*Mme. 1:* Ouais ? Alors c'est parti, dites-moi tout ce qui vous vient à l'esprit sur ce légume ou ce fruit.

00:00:34
*Mme. 2:* [Euh] Sucré.

00:00:35
*Mme. 1:* Ouais.

00:00:36
*Mme. 2:* Soleil.

00:00:37
*Mme. 1:* Ok.

00:00:42
*Mme. 2:* [Euh] Comment expliquer, c-c'est juteux.

00:00:50
*Mme. 1:* Mm-hmm.

00:00:50
*Mme. 2:* Voilà. Juteux. [euh] Qui a esprit [euh] été.

00:01:07
*Mme. 1:* Ouais.

00:01:12
*Mme. 2:* C'est l'été. [rires]

00:01:33
*Mme. 1:* Ouais.

00:01:33
*Mme. 2:* [Euh] Elle- douceur aussi. [silence 00:01:00-00:01:04] [euh] Acidité, un peu.

00:01:43
*Mme. 1:* Mm-hmm.

00:01:43
*Mme. 2:* Et couleur. [silence 00:01:14-00:01:18]

00:01:43
*Mme. 1:* D'accord.

00:01:43
*Mme. 2:* Et, [euh] comment vous expliquer ? Ouais, sans dire le fruit c'est compliqué. [euh] Fleur.

00:01:43
*Mme. 1:* Ouais.

00:01:43
*Mme. 2:* Ah ouais, ça, ça va être fleur. Et arbre, pour finir.

00:01:58
*Mme. 1:* Et ?

00:01:58
*Mme. 2:* Arbre.

00:01:58
*Mme. 1:* Arbre.

00:01:58
*Mme. 2:* Pour finir.

00:01:58
*Mme. 1:* D'accord. [euh] Qu'est-ce que vous aimez tout particulièrement [euh] dans ce fruit ?

00:01:58
*Mme. 2:* Dans ce fruit, [euh] le plus, le plus c'est le, [euh] ben c'est le goût évidemment, mais le goût sucré, le goût su-suave, sucré.

00:01:58
*Mme. 1:* D'accord, et à quel moment [euh] de la journée, le mangez-vous le plus souvent ? Le matin, le midi, le soir ?

00:02:05
*Mme. 2:* Le midi, après-midi.

00:02:06
*Mme. 1:* Midi, après-midi.

00:02:06
*Mme. 2:* Ouais, ouais.

00:02:06
*Mme. 1:* Oui. Est-ce que vous pouvez me raconter comment vous le manger ? C'est-à-dire [euh] toutes les étapes qu'il y a, avant le f-, le fait de--, quand vous décidez de le manger et le moment où vous le mettez dans votre bouche ? Qu'est-ce qui se p-, qu'est-ce que vous faites ?

00:02:23
*Mme. 2:* Alors, je le découpe en quartiers.

00:02:25
*Mme. 1:* Ouais. [silence 00:02:26-00:02:31]

00:02:33
*Mme. 2:* En quartiers. Je croque dans un quartier, évidemment.

00:02:35
*Mme. 1:* Ouais.

00:02:37
*Mme. 2:* Et [euh] ben c'est ce goût-là, ce goût sucré, ce goût qui fond en fait. Ça fond dans la bouche. Ça fond dans la dou- bouche, mais il y a quand même un petit arrière, un petit peu, un petit peu acide, un tout petit peu que j'aime bien. J'aime bien, voilà, cette [euh], cette [euh], c-. Donc pareil, deuxième quartier, troisième quartier, quatrième quartier, toujours. Je coupe toujours en quatre-

00:03:01
*Mme. 1:* En quatre.

00:03:02
*Mme. 2:* Toujours. [rire] C'est marrant. Parce que c'est juteux.

00:03:06
*Mme. 1:* Mm-hmm.

00:03:08
*Mme. 2:* Donc [euh], c'est plus facile à manger. Et, et donc, pour l'étape final, et ben j'ai un noyau que je jette-

00:03:17
*Mme. 1:* D'accord.

00:03:17
*Mme. 2:* À la poubelle.

00:03:21
*Mme. 1:* Ouais.

00:03:21
*Mme. 2:* Et que je--, souvent avec le couteau, je mange aussi ce qu'il y a dans le noyau. J'aime bien. [rires] Jusqu'au bout, jusqu'au bout. Voilà.

00:03:29
*Mme. 1:* Vous grattez dans le noyau ?

00:03:30
*Mme. 2:* Oui, avec le couteau. J'aime bien ce-, j'aime bien en fait avoir ce petit-, ce petit filament qu'on trouve dans le noyau. Voilà. Puis le noyau s'en va à la poubelle ou, voilà, au petit bonheur.

00:03:45
*Mme. 1:* Ok.

00:03:45
*Mme. 2:* C'est quand même plus facile à découvrir ce que c'est [rire]. Voilà.

00:03:52
*Mme. 1:* [Euh] Où l'achetez-vous ? Le plus souvent ?

00:03:54
*Mme. 2:* Sur les marchés.

00:03:56
*Mme. 1:* Sur les marchés.

00:04:02
*Mme. 2:* Et dans le jardin.

00:04:03
*Mme. 1:* Et dans le jardin.

00:04:03
*Mme. 2:* Ouais. Ensuite, c'est un peu en arrière-saison.

00:04:10
*Mme. 1:* D'accord.

00:04:10
*Mme. 2:* Ouais, ça complique ? [rire]

00:04:14
*Mme. 1:* [Euh] Et, selon vous, à quel rythme approximatif le mangez-vous ?

00:04:17
*Mme. 2:* Tous les jours.

00:04:18
*Mme. 1:* Tous les jours ?

00:04:18
*Mme. 2:* Quand c'est la saison, tous les jours. Ouais, ouais. Ah oui, tous les jours, deux fois par jour. Ou trois fois. Enfin, moi je mange beaucoup quand c'est la saison, je mange beaucoup, beaucoup.

00:04:29
*Mme. 1:* Mm-hmm.

00:04:29
*Mme. 2:* Ouais, ce fruit-là, entre autres [hein], puisque j'en ai d'autres aussi, mais celui-là spécifiquement. Ouais, ouais.

00:04:36
*Mme. 1:* D'accord.

00:04:36
*Mme. 2:* Je le mange, ouais. Et je fais plein de choses avec aussi.

00:04:40
*Mme. 1:* D'accord.

00:04:42
*Mme. 2:* Je fais des salades de fruits, des compotes, des confitures.

00:04:45
*Mme. 1:* D'accord.

00:04:47
*Mme. 2:* Et-et-et je les poche et je les congèle.

00:04:49
*Mme. 1:* Ok.

00:04:52
*Mme. 2:* Voilà, pour les avoir l'hiver après.

00:04:52
*Mme. 1:* D'accord.

00:04:53
*Mme. 2:* Voilà, en plus.

00:04:53
*Mme. 1:* Ok.

00:04:56
*Mme. 2:* En plus.

00:05:00
*Mme. 1:* [euh] Et qu'est-ce que vous ressentez quand vous le mangez, alors ? Vous m'avez déjà dit [euh] cette sensation de sucré [euh], juteux. [inaudible 00:05:06 - 00:05:07]

00:05:08
*Mme. 2:* Ouais, douceur qui se mange facilement, quoi. Qui, qui glisse en fait, qui se mange [euh]. Sensation, [ben] oui une sensation de plaisir [hein].

00:05:15
*Mme. 1:* Mm-hmm.

00:05:15
*Mme. 2:* Évidemment [hein]. De plaisir, de gourmandise.

00:05:18
*Mme. 1:* Ouais.

00:05:19
*Mme. 2:* Comme un bonbon. J'aime pas les bonbons, mais alors les fruits, qu'est-ce que j'aime ça. [Ah] Cette sensation d'un bonbon, d'un plaisir [quoi] qu'on- qu'on a. [euh], qu'on a, voilà c'est, c'est plaisir [euh] suave, plaisir du fruit, [euh] de manger [euh]. Je me pose, je mange jamais ça vite fait, je me pose toujours pour manger mon fruit. [rire]

00:05:39
*Mme. 1:* D'accord.

00:05:39
*Mme. 2:* Voilà.

00:05:39
*Mme. 1:* Et est-ce que vous avez [euh] une idée sur ce qu'il peut vous apporter, en plus du plaisir ?

00:05:47
*Mme. 2:* Des vitamines.

00:05:48
*Mme. 1:* Ouais.

00:05:48
*Mme. 2:* Et assurément des fibres. Des vitamines, des fibres [euh] ça c'est certain.

00:05:56
*Mme. 1:* Mm-hmm.

00:05:56
*Mme. 2:* Des fibres, [euh] [ben] [pff] des minéraux [hein], puisque y a des minéraux dans le fruit aussi--

00:06:04
*Mme. 1:* Ouais.

00:06:05
*Mme. 2:* --toutes ces choses-là qui m'apportent le bien-être pour la santé évidemment [hein].

00:06:08
*Mme. 1:* Mm-hmm.

00:06:09
*Mme. 2:* Pour tout [hein].

00:06:12
*Mme. 1:* Et à l'inverse, est-ce que vous lui trouvez quelques inconvénients ? Très large.

00:06:17
*Mme. 2:* Ça peut, ça peut, s'il est pas-, si c'est pas assez mûr-

00:06:20
*Mme. 1:* D'accord.

00:06:22
*Mme. 2:* Il faut vraiment qu'il soit parfait, parce que s'il est pas assez mûr, c'est dur. Ça a pas de goût.

00:06:28
*Mme. 1:* Mm-hmm.

00:06:28
*Mme. 2:* Voilà, comme beaucoup de fruits qui sont pas assez mûrs. Ou alors, ils sont mûrs et ils ont pas de goût. Ça arrive aussi.

00:06:33
*Mme. 1:* D'accord.

00:06:33
*Mme. 2:* Ça dépend d'où ils viennent. Ils sont mûrs, ils ont pas de goût, donc [euh] ça a pas de plaisir.

00:06:38
*Mme. 1:* Ouais.

00:06:38
*Mme. 2:* [Euh] J'le mange quand même. Je jetterais pas, mais je suis déçue [quoi]. Donc [euh], donc c'est pour ça que j'essaie d'en manger [euh] plus sur les marchés, en fait.

00:06:49
*Mme. 1:* D'accord.

00:06:49
*Mme. 2:* Plus [euh], plus [euh] sur les conseils du [euh] souvent du maraîcher ou du primeur pour [euh] avoir des fruits à [euh] à point [quoi].

00:06:55
*Mme. 1:* Ouais.

00:06:55
*Mme. 2:* J'préfère en acheter moins. Et puis en manger, [euh] plus de plaisir [quoi], avec plus de plaisir. [aboiements] Arrête.

00:07:01
*Mme. 1:* Ok, alors faut que j'essaie de [euh] trouver c'est lequel.

00:07:32
*Mme. 2:* Oui.

00:07:39
*Mme. 1:* C'est un fruit d'été, alors j'hésite. Je vais essayer, j-j-je dirais la pêche.

00:08:35
*Mme. 2:* Ouais.

00:08:35
*Mme. 1:* C'est la pêche ? [rire]

00:08:37
*Mme. 2:* Ouais, c'est la pêche.

00:08:37
*Mme. 1:* Chouette.

00:08:37
*Mme. 2:* Oui, le noyau [hein] c'est--

00:08:47
*Mme. 1:* Ouais, j'ai pensé à l'abricot aussi mais--

00:08:47
*Mme. 2:* Ouais, l'abricot non, et puis ici il y a pas d'abricots, que des pêches.

00:09:07
*Mme. 1:* Ouais.

00:09:07
*Mme. 2:* Voilà, moi mon pêcher énorme [hein], il est énorme, énorme. Il [inaudible 00:07:42- 00:07:42] ici, donc il est là le pêcher, vous le voyez, je pense que derrière. Je sais pas si vous voyez le grand arc qu'il y a là sur le côté ?

00:09:07
*Mme. 1:* Ouais.

00:09:07
*Mme. 2:* C'est un pêcher.

00:09:07
*Mme. 1:* D'accord.

00:09:07
*Mme. 2:* Et il y a des petites pêches, des toutes petites là, je vais en avoir [euh] des cageots, des cageots, et là c'est en arrière-saison, c'est plus [euh] en septembre.

00:09:19
*Mme. 1:* D'accord.

00:09:19
*Mme. 2:* Voilà, mais je commence-- [euh], là j'achète pas encore les pêches pour l'instant parce que je sais qu'elles sont pas bonnes.

00:09:19
*Mme. 1:* Ouais.

00:09:19
*Mme. 2:* Et je redescends dans le sud au mois de juillet, donc là, [pfff] c'est des, [ah] et puis c'est des pêches comme ça [quoi], c'est [ah] c'est le rêve, voilà, c'est le rêve. On en mange, on en mange, on en mange jusqu'à plus, plus [euh]-- Puis souvent quand je reviens ici, j'ai du mal à en trouver des bonnes.

00:09:23
*Mme. 1:* Ouais.

00:09:23
*Mme. 2:* Donc j'ai les miennes après qui sont excellentes, en fait. Très sucrées. Et c'est surprenant ici d'avoir des pêches comme ça. Donc, on a des pêches sucrées que je garde tout l'hiver.

00:09:24
*Mme. 1:* D'accord.

00:09:24
*Mme. 2:* Pour faire mes salades de fruit et des-des pêches Melba. Mes desserts.

00:09:24
*Mme. 1:* Ouais.

00:09:24
*Mme. 2:* Je les poche et je les congèle.

00:09:24
*Mme. 1:* D'accord.

00:09:24
*Mme. 2:* Ça se conserve bien.

00:09:24
*Mme. 1:* Ouais.

00:09:24
*Mme. 2:* Donc, voilà. Voilà, voilà. [rires]

00:09:24
*Mme. 1:* Chouette. Cette histoire de la pêche.

00:09:24
*Mme. 2:* C'est la pêche. Voilà. Entre autres [hein], parce que [ah] j'hésitais avec la mandarine qui vient de saison. La mandarine, je suis folle de mandarine. [rires] [Ah] Là, c'est- [pouh] c'est pareil, quoi, pareil. La même chose.

00:09:24
*Mme. 1:* Ouais, la même chose, c'est sûr.

00:09:24
*Mme. 2:* Ouais, ouais. Voilà.

00:09:24
*Mme. 1:* [Euh] Super. Alors maintenant, j'aimerais qu'on discute de vos pratiques d'achat et de votre organisation générale. Donc, là c'est vrai que vous avez déjà évoqué pas mal de choses. Au sein de votre foyer, qui est-ce qui est amené à faire les courses ?

00:09:24
*Mme. 1:* Moi, souvent. Moi.

00:09:24
*Mme. 1:* Souvent vous ?

00:09:24
*Mme. 1:* Souvent moi, mais parfois accompagnée de mon mari. Mais souvent, moi.

00:09:24
*Mme. 1:* Mmh-mm.

00:09:24
*Mme. 1:* Sur les marchés, c'est toujours avec mon mari. Nous, on fait [euh]--

00:09:24
*Mme. 1:* Mm.

00:09:24
*Mme. 1:* Mais autrement c'est moi.

00:09:24
*Mme. 1:* Ok. Et du coup, est-ce que vous pouvez me raconter comment ça s'organise justement vos-vos courses alimentaires ?

00:09:24
*Mme. 2:* [Bah] en géné-- [pff] Alimentaires ou vraiment fruits et légumes ?

00:09:27
*Mme. 1:* Comme-- Ça peut être [euh] à partir de l'alimentaire et après voir vos différentes--

00:09:31
*Mme. 2:* Parce que c'est vraiment dif-dis-dissocié de l'alimentaire.

00:09:34
*Mme. 1:* Oui ?

00:09:34
*Mme. 2:* J'achète toujours chez le primeur.

00:09:37
*Mme. 1:* D'accord.

00:09:37
*Mme. 2:* C'est une autre démarche. J'achète jamais de fruits et légumes [euh] au supermarché.

00:09:40
*Mme. 1:* Ok.

00:09:41
*Mme. 2:* Jamais. J'achète toujours ou chez le primeur ou au marché.

00:09:44
*Mme. 1:* D'accord.

00:09:44
*Mme. 2:* Donc, une fois par semaine, je vais chez le primeur.

00:09:47
*Mme. 1:* Ok.

00:09:49
*Mme. 2:* Toujours une fois par semaine. Ou au marché, ça dépend des saisons. L'été, on va plus au marché. Mais [euh] vu le contexte aussi, c'est plutôt primeur.

00:09:57
*Mme. 1:* D'accord.

00:09:58
*Mme. 2:* Voilà. Sur le primeur, je prends, [euh] je prends--, j'ai une petite liste en général, des petites choses que j'ai besoin. Après, il y a une autre, il y a une autre étape aussi que je fais [euh].

00:10:10
*Mme. 1:* Ouais.

00:10:10
*Mme. 2:* Y a une liste. Donc j'arrive là bas et je prends au bonheur de ce qui me [euh] ce qui me-- des envies, en fait et de ma petite liste, évidemment. Si je fais un pot-au-feu, je sais que j'ai mes légumes qui seront définis dans le pot-au-feu.

00:10:22
*Mme. 1:* Ouais.

00:10:22
*Mme. 2:* Et je sais que j'ai mes oranges pour les jus de fruits dans la semaine. Enfin, j'ai toujours un petit peu les mêmes choses.

00:10:27
*Mme. 1:* Oui.

00:10:27
*Mme. 2:* Mais j'ai aussi, [euh] j'ai aussi le plaisir de choisir ce qui me [euh] vient par-- voilà-- ce qui me-- je me dis : tiens qu'est-ce qu'ils sont beaux ceux-là, ils donnent envie. [Hop], je prends. Voilà. Donc, j'achète plus comme ça, [euh] sauf les-- tout ce qui est [euh] conservation : les pommes de terre, les échalotes, les oignons, les carottes,--

00:10:46
*Mme. 1:* Ok.

00:10:47
*Mme. 2:* --que j'achète chez un agriculteur.

00:10:50
*Mme. 1:* D'accord.

00:10:50
*Mme. 2:* Directement. Depuis des années. Toujours de la même manière.

00:10:55
*Mme. 1:* Alors, c'est-à-dire ? Comment ça se passe ?

00:10:57
*Mme. 2:* Alors, elle [euh], c'est une amie à moi, son père est-- son père est agriculteur, et je lui commande [euh] les pommes de terre pour toute la famille, en fait. D'ailleurs.

00:11:06
*Mme. 1:* D'accord.

00:11:06
*Mme. 2:* Mes enfants aussi. On commande cinq sacs de pommes de terre.

00:11:08
*Mme. 1:* D'accord.

00:11:09
*Mme. 2:* --que je conserve ici [euh] à la maison. Les enfants, comme ils habitent à côté, au besoin, ils viennent chercher. Mais moi, je les conserve ici. Pour qu'ils soient bien conservés, en fait, tout simplement.

00:11:19
*Mme. 1:* Ok.

00:11:19
*Mme. 2:* [Euh] Donc, ça, les carottes, l'ail, l'ail aussi. On me les donne en gou-- enfin on me les donne en-- j'avais oublié l'ail, ouais, l'ail aussi. Et donc du coup, je vais [euh] je vais entreposer tout ça au garage. Et je vais prendre mes oignons, mes échalotes en général sur-- à Roscoff, en fait.

00:11:39
*Mme. 1:* D'accord.

00:11:40
*Mme. 2:* Il y a des camions au bord de la route qui vendent des oignons de Roscoff et des-- Mon mari est là-bas aussi, donc [euh]. Des oignons de Roscoff, toujours. Des échalotes de la Botte, toujours.

00:11:51
*Mme. 1:* D'accord.

00:11:51
*Mme. 2:* Et je prends et j'entrepose des grosses quantités--

00:11:53
*Mme. 1:* D'accord.

00:11:54
*Mme. 2:* De- d'oignons, c'est des grands sacs [hein]. Ouais, de l'oignon, on en mange beaucoup, beaucoup [hein]. Pommes de terre. Voilà, c'est-- je vais chercher chez mon amie qui les entrepose en attendant. Elle va les chercher chez son père, elle me les donne, on les met dans la voiture. J'arrive ici et je les entrepose [euh] tous [euh] d'une manière différente.

00:12:12
*Mme. 1:* D'accord.

00:12:13
*Mme. 2:* Donc chez le primeur, je vous expliquerai après. Chez le primeur, bon voilà c'est ça, c'est vraiment au-au coup de coeur et beaucoup de fruits de saison [hein]. Beaucoup de fruits de saison. Là, en ce moment, on mange beaucoup de fraises.

00:12:24
*Mme. 1:* Ouais.

00:12:24
*Mme. 2:* Parce que la fraise a-- la fraise a-- est en saison [hein]. Mais c'est vrai que [euh] beaucoup, beaucoup, ouais quand même, de-- Mais bon, si j'ai envie d'une banane, je mangerai une banane, quoi. Mais voilà, c'est- c'est- après c'est pas des-- on aime bien les fruits de saison.

00:12:37
*Mme. 1:* Ouais.

00:12:37
*Mme. 2:* On va dire ça [euh]. Parce que sachant que j'ai quand même deux mandarines dans la-- dans le panier quoi.

00:12:43
*Mme. 1:* Ouais.

00:12:43
*Mme. 2:* Donc je me suis dit, je vais jusqu'au bout de la saison, cette année elle est tard la saison. [rires] Voilà. Donc ça, et puis voilà, quoi. Bon, donc après, [euh] [bah] quand je suis rentré à la maison avec mes-mes achats, je sais pas si vous avez besoin de compléments. Parce que--

00:12:56
*Mme. 1:* Si, si, dites-moi tout ce qui vous vient--

00:12:57
*Mme. 2:* Quand je rentre à la maison, je les entrepose dans mes paniers, ma caisse à champignons.

00:13:00
*Mme. 1:* D'accord.

00:13:01
*Mme. 2:* Chaque étage, chaque fruit. Chaque-- [oh], j'ai oublié les pommes, j'ai oublié de dire aussi-- Bon, on va passer par les pommes avant. Je vais chercher des pommes chez-- [euh], dans un verger. Directement.

00:13:11
*Mme. 1:* D'accord.

00:13:11
*Mme. 2:* Je prends des cageots de pommes. En général, on prend quatre cageots et on continue [euh] dans l'année, si on en n'a plus, on prend déjà quatre cageots.

00:13:20
*Mme. 1:* D'accord.

00:13:20
*Mme. 2:* Toujours. Chez le-, chez-, au verger, à Carlahon. Ici, ils ont des super pommes.

00:13:24
*Mme. 1:* Ok.

00:13:24
*Mme. 2:* Et j'achète, voilà, plusieurs variétés de pommes. On arrive ici. De toute façon, c'est toute la même manière. Je les mets dans mes caisses. Y comp- les pommes je les mets à part quand même, parce que j'ai pas envie que-- les pommes ça fait mûrir tout le reste. Donc, mes pommes sont ailleurs. À droite. [rires] Après, je range mes-, mes fruits et légumes dans des paniers.

00:13:41
*Mme. 1:* Ouais.

00:13:41
*Mme. 2:* Distincts. Comme ça, moi je sais-, je sais qu'au dessus c'est les tomates et les fruits qui s'abîment le moins. Ensuite, les oignons, les échalotes. Si j'achète des asperges, je sais que je les mets à l'ombre, en-dessous dans du papier journal. Des choses comme ça.

00:13:54
*Mme. 1:* Ouais.

00:13:55
*Mme. 2:* Et, et puis, les pommes de terre, quand je les ai, je les mets dans--, j'ai des grandes caisses. Je les enveloppe de papier journal, en fait, toujours.

00:14:02
*Mme. 1:* D'accord.

00:14:02
*Mme. 2:* Et je les enferme dans du papier journal, en fait, pour la-- pour l'hiver.

00:14:07
*Mme. 1:* D'accord.

00:14:07
*Mme. 2:* Et ça fait pas longtemps qu'on vient de les finir. On les a au mois de septembre--

00:14:09
*Mme. 1:* Ok.

00:14:10
*Mme. 2:* Début octobre. Donc ça se conserve très, très bien.

00:14:13
*Mme. 1:* D'accord.

00:14:13
*Mme. 2:* Donc je les garde comme ça, [bon][hein] dans le garage, c'est toujours frais. Donc, c'est pour ça que je les mets jamais au frigo puisque le garage suffit.

00:14:20
*Mme. 1:* D'accord.

00:14:20
*Mme. 2:* Donc, y a pas besoin de faire. Si j'entame un fruit, ou là j'ai entamé le concombre, le concombre je l'ai mis [euh], je l'ai mis au frigo avec un-, avec un film puisque je l'ai entamé.

00:14:29
*Mme. 1:* Ouais.

00:14:29
*Mme. 2:* Mais bon, ça reste deux jours. J'suis même pas sûr. Ce midi, je pense que je vais le finir. Et voilà, c'est comme ça qu'on-on entrepose nos légumes. Jamais au frigo.

00:14:37
*Mme. 1:* D'accord.

00:14:37
*Mme. 2:* C'est très rare. Ou l'autre jour, les betteraves rouges fraîches, ça évidemment, [ben] je les mets au frigo.

00:14:41
*Mme. 1:* Ouais.

00:14:41
*Mme. 2:* Et [euh] autrement, si jamais je vois que j'ai un fruit qui est un petit peu abîmé--

00:14:44
*Mme. 1:* Ouais.

00:14:45
*Mme. 2:* --ce qui arrive--, peut arriver avec les champignons, des choses comme ça qui sont un-- qui s'abîment même si on les achète frais, je vais couper, je les congèle tout de suite, immédiatement.

00:14:51
*Mme. 1:* D'accord.

00:14:53
*Mme. 2:* Après, j'ai un petit potager.

00:14:55
*Mme. 1:* D'accord.

00:14:55
*Mme. 2:* J'ai un petit potager. Où j'ai mes salades. Alors, j'ai des salades, j'ai des-- toutes mes herbes fraîches.

00:15:04
*Mme. 1:* Ouais.

00:15:04
*Mme. 2:* [Ah] ça, j'aime beaucoup. J'ai ciboulette, [euh] j'ai [euh] qu'est-ce que j'ai, [euh] romarin, verveine, thym.

00:15:10
*Mme. 1:* D'accord.

00:15:11
*Mme. 2:* Persil. Tout ça, j'aime beaucoup avoir dans le-- avoir dans le jardin. J'ai des plants de tomates, j'ai-- j'ai mis des courgettes. En fait, je mets toujours la même chose. Des radis, parce que j'adore les radis. Et [euh] j'ai mis un poivron cette année, voilà. J'ai un basilic qui est là, qui survit. Mais j'ai un basilic aussi. Voilà, j'ai un petit peu tout dans le jardin qui me [euh] permet, surtout pour le plaisir [hein]. Des fraisiers.

00:15:35
*Mme. 1:* Ouais.

00:15:35
*Mme. 2:* Des fraises, on va voir des fraises aussi, mais pas beaucoup [quoi]. Voilà un petit plaisir pour moi [euh], je me fais--. Des tomates cerises, si j'ai trois plants. Ils donnent beaucoup, beaucoup donc [euh]--. Voilà, donc [euh] j'ai un petit potager qui m'alimente. Surtout les salades, j'aime bien les salades fraîches.

00:15:48
*Mme. 1:* Ouais.

00:15:49
*Mme. 2:* Je trouve que ça permet-- [euh] C'est ce qui se-- le plus de difficultés à se conserver. La salade quand je la prends, donc que ce soit dans le jardin, ou parfois chez le primeur, ça peut arriver. Je la coupe, je la lave et je la mets dans une [euh] boîte spéciale.

00:16:01
*Mme. 1:* D'accord.

00:16:03
*Mme. 2:* Avec du sopalin dessus. En fait, ça absorbe l'humidité.

00:16:05
*Mme. 1:* D'accord.

00:16:06
*Mme. 2:* Et je mets au frigo. [euh] Surtout pour la salade [hein].

00:16:11
*Mme. 1:* Ok.

00:16:11
*Mme. 2:* Voilà.

00:16:13
*Mme. 1:* D'accord. Donc là, vous avez [euh], vous m'avez dit pas mal de stratégies de [euh] de conservation.

00:16:24
*Mme. 2:* Ouais.

00:16:24
*Mme. 1:* [euh] Alors, je vais revenir après. [euh] Si on--, quand on va, par exemple là vous m'avez dit chez le primeur, [euh] vous avez une liste, mais--

00:16:33
*Mme. 2:* Oui, j'ai une liste.

00:16:34
*Mme. 1:* --vous fonctionnez aussi [euh] à l'envie.

00:16:36
*Mme. 2:* Oui.

00:16:37
*Mme. 1:* Du coup, qu'est-ce que vous faites avant de partir [euh] faire vos courses ? Vous faites votre liste [euh] ?

00:16:42
*Mme. 2:* Je fais au fur et à mesure. Je dis : tiens, j'ai p--, j'ai un oignon, [oh] faudra que j'en prenne--. Parce que ça peut arriver aussi : par exemple, [euh] les oignons, c'est fini [hein], ma--

00:16:50
*Mme. 1:* Ouais.

00:16:50
*Mme. 2:* Ma, ma quantité. Je vais pas retourner à Roscoff, chercher des oignons.

00:16:53
*Mme. 1:* Ouais.

00:16:53
*Mme. 2:* Je vais mettre : [bah] tiens, faut pas que j'oublie de prendre un oignon, enfin deux ou trois oignons la prochaine fois. [Euh] voilà, on a envie de carottes râpées : faut pas oublier de prendre des carottes, quoi.

00:17:00
*Mme. 1:* Ouais.

00:17:00
*Mme. 2:* Des choses comme ça. Mais [euh] bon, souvent, puisque j'ai établi--, j'essaie d'établir des menus. Bon, maintenant un peu moins, parce que j'avais trois enfants et je travaillais à temps plein.

00:17:08
*Mme. 1:* D'accord.

00:17:08
*Mme. 2:* Et les trois enfants, ils mangeaient avec nous midi et soir, donc il fallait gérer les menus de la semaine, donc j'étais plus ordonné. On va dire. Là, y a plus que mon mari, moi, puis mon, mon dernier. Bon, il est interne, en plus. Donc, il est pas là tout le temps. Donc, on est un peu plus désordonné.

00:17:22
*Mme. 1:* Ouais.

00:17:22
*Mme. 2:* On, on marche un peu au coup de cœur. Quand mon mari me dit : Tiens, j'ai en--, je mangerais bien des en--, tu vas chez le primeur ? Je mangerais bien des endives au jambon, [bah] écoute [euh] tu peux prendre des endives ? Je lui dis : [bah] oui, on va prendre. Et puis demain, on mangera ça, ou après-demain. Ça fait un repas.

00:17:33
*Mme. 1:* D'accord.

00:17:34
*Mme. 2:* On est un peu plus, [euh] un peu plus désordonné. Mais bon, après, on-- Voilà, c'est vraiment au-, au-, par rapport aux menus de la semaine. Souvent j'établi quand même des menus. Enfin, des menus [euh] : je décide de certains plats différents. Mais c'est pas toujours fait [euh]-- Mais comme je cuisine tout, je n'achète--, j'achète. Alors, j'ai toujours des conserves, des petites conserves, par exemple de champignons.

00:17:54
*Mme. 1:* Ouais.

00:17:54
*Mme. 2:* Ça, [euh] je crois que j'ai. J'ai-- [ah] mon fils est parti camper, m'a emmené une boîte de lentilles. Ça, on n'en mange jamais d'habitude, mais j'ai une boîte de lentilles. Enfin, des petites-- Du maïs, par exemple, aussi. J'ai [euh] j'ai des petites choses qui ont--, mais qui me servent très, très occasionnellement.

00:18:10
*Mme. 1:* Ouais.

00:18:10
*Mme. 2:* C'est en dépannage. Parce que je me dis : les champignons, c'est vrai qu'on n'en a pas toujours sous le coude. Et puis, bon, même si c'est surgelé, j'en ai souvent aussi. Ça m'arrive d'aller parfois [euh] [euh] plus les hivers chez le [euh], chez [euh], [bah] chez écomiam en bas.

00:18:25
*Mme. 1:* Ouais.

00:18:25
*Mme. 2:* Chercher [euh], par exemple, des-- on va dire [euh] une-, un sachet de champignons, un sachet de petits pois, surgelés. Mais c'est très rare. Parce que je congèle quand même beaucoup. J'achète-, du coup, j'achète des potirons, les gros potirons, là. J'achète. On a- on a avec des collègues, on les coupe en carrés, on les congèle.

00:18:41
*Mme. 1:* D'accord.

00:18:41
*Mme. 2:* Voilà, des choses comme ça. Les haricots blancs, les haricots coco. Je prends des grands sacs et je les [euh] on les épluche tous et puis on les met au congèle aussi.

00:18:48
*Mme. 1:* D'accord.

00:18:48
*Mme. 2:* Voilà. On a quand même un rayon au con- où on congèle nos fruits et lég-, quelques fruits et légumes, quoi. Voilà.

00:18:53
*Mme. 1:* Ok.

00:18:54
*Mme. 2:* Ça, c'est sûr que on en a un petit peu, toujours un petit peu, quoi. Pour dépanner. Les petits pois, je vais pas les acheter en conserve, je les achèterais plutôt surgelés, si c'est pas la saison, et frais quand c'est la saison, qui arrive là, qui-- Voilà, donc [euh]. C'est plus-, plus ça quoi. Donc [euh] Et je-, je me fais vraiment une petite liste, sur mon petit panier habituel. Puis, j'arrive [euh]. Voilà.

00:19:13
*Mme. 1:* D'accord.

00:19:13
*Mme. 2:* Et vraiment toujours. Il y a des choses qu'on mange habitue-, qu'on est habitué. Après, j'achète toujours un concombre. Je-, j'adore les concombres. Y a que moi qui mange, mais j'adore. Donc, j'achète toujours. Il y a des choses que j'achète toujours, en fait.

00:19:24
*Mme. 1:* C'est ça.

00:19:24
*Mme. 2:* C'est récurrent, quoi. Donc, [euh] voilà. Après [euh], c'est, c'est-- le primeur est pas loin. Donc, on va toutes les semaines et en général je retourne--, je vais qu'une fois, j'ai pas besoin de retourner plusieurs fois.

00:19:36
*Mme. 1:* Ouais.

00:19:36
*Mme. 2:* J'arrive à m'organiser. Pour [euh], pour faire--, pour faire les achats dont j'ai besoin. Je fais avec ce que j'ai, quoi, en fait. De toute façon, je vais pas acheter [euh]--, s'il en reste, je vais pas aller racheter des légumes.

00:19:44
*Mme. 1:* Ouais.

00:19:46
*Mme. 2:* C'est pas la peine. S'il me reste deux tomates, je vais pas aller acheter d'autres. Je finis d'abord mes tomates. Et puis, puis voilà. Puis, les tomates [ben] c'est pas trop la période, donc-- J'ai acheté des petites là pour [euh], pour agrémenter un peu les plats, mais bon, après c'est quand même-- Tomates de serre, c'est moins bon. [rires] Voilà.

00:20:01
*Mme. 1:* Ok. Et du coup, quand vous achetez vos fruits et légumes, [euh] comment vous décidez des quantités que vous--, est-ce que c'est instinctif ? Est-ce qu'avant, c'était très-, très-, très délimité dans votre liste de courses ?

00:20:17
*Mme. 2:* Si je fais des grands plats, oui.

00:20:19
*Mme. 1:* D'accord.

00:20:20
*Mme. 2:* Je sais que je vais prendre-- Je fais un pot-au-feu, je vais prendre cinq navets je vais pas en prendre six. Je vais prendre cinq pour mettre dans le machin, dans le-, dans le pot-au-feu. Je vais pas aller en prendre six vu qu'il m'en restera un là, quoi. Les poivrons [euh], les poivrons, j'en ai aussi-- Par exemple, tiens, il y a une promo. J'ai besoin de trois poivrons : à la limite je vais peut-être plutôt en prendre six, je vais les couper.

00:20:38
*Mme. 1:* Ouais.

00:20:38
*Mme. 2:* Et mettre au congèle.

00:20:39
*Mme. 1:* D'accord.

00:20:40
*Mme. 2:* Plus facilement. Mais c'est vraiment [euh], ouais c'est pas, c'est pas préci- Enfin c'est pas précisé, c'est au petit bonheur, quoi. Après [euh], voilà.

00:20:51
*Mme. 1:* D'accord ? Et [euh] dans vos-, vous nous avez dit que vous n'achetez pas en supermarché vos--, vos fruits et légumes frais.

00:20:59
*Mme. 2:* Nan.

00:21:00
*Mme. 1:* Que chez le primeur ou à Roscoff, dans votre jardin, chez l'agriculteur--

00:21:05
*Mme. 2:* Ou au marché.

00:21:06
*Mme. 1:* Ou au marché. Et pourquoi vous choisissez ces lieux ? Pourquoi vous privilégiez ces lieux?

00:21:11
*Mme. 2:* [euh] Parce que je trouve qu'il y a une qualité. Déjà les-- les [euh]-- ils-- c'est pas en fri-- c'est pas mis en frigo.

00:21:18
*Mme. 1:* Oui.

00:21:19
*Mme. 2:* Donc déjà, c'est un fruit [euh], fruits et légumes, ils sont pas en frigo. Chez le primeur. Si c'est un frig--, un primeur qui met au frig-- dans les frigos. J'en veux pas.

00:21:26
*Mme. 1:* D'accord.

00:21:27
*Mme. 2:* Je veux pas de légumes qui sont mis au frigo, parce qu'ils s'abîment, en fait.

00:21:29
*Mme. 1:* D'accord.

00:21:29
*Mme. 2:* Je trouve que [euh] il s'abîment trop vite. Ils durent pas trop longtemps. Ils s'abîment trop vite.

00:21:33
*Mme. 1:* Ouais.

00:21:34
*Mme. 2:* Donc c'est pour ça que je préfère avoir des, des légumes qui tiennent une semaine [hein]. Je demande pas grand chose [hein]. Juste une semaine, mais je veux pas-- [euh]. Je me suis aperçue qu'au supermarché, on achète [euh] je sais pas, enfin de [euh], on va acheter des radis, au bout de trois jours, les pauvres radis-- [euh]. Ah, y a quelqu'un qu'a frappé, je crois. [Aboiements] Je vais aller voir

00:21:53
*Mme. 1:* Allez-y.

00:22:00
*Mme. 2:* Désolé.

00:22:00
*Mme. 1:* Il y a pas de souci. Allez-y. [Mme. 2 sort de la maison] [aboiements] [silence 00:22:01-00:22:21] [Mme. 2 revient dans la salle]

00:22:17
*Mme. 2:* Ah, c'est un Monsieur qui vendait des fraises ?

00:22:23
*Mme. 1:* [rires] Vous aviez ce qu'il faut.

00:22:28
*Mme. 2:* J'ai ce qu'il faut. Donc voilà, j'achèterais pas. Enfin, le gars il vient acheter des fraises--, enfin vendre des fraises, j'achèterais pas.

00:22:34
*Mme. 1:* Oui.

00:22:34
*Mme. 2:* Je fais confiance à mon primeur. Donc voilà, après. Je sais que la qualité est là. Mais au supermarché, je suis très déçue. Et le fait aussi que [euh] les gens touchent--

00:22:43
*Mme. 1:* Ouais.

00:22:44
*Mme. 2:* --les légumes, pour voir si c'est mûr, c'est machin. Alors que chez le primeur, c'est différent parce que-- quand on va chez le primeur, on dit tiens je voudrais un melon [euh] Est-ce que vous pourriez me le prendre le melon du jour, pour aujourd'hui, pour demain, pour [euh]-- Voilà. Est-ce que les fraises sont frai-, sucrées ? Est-ce qu'elles sont-- ? Et voilà, on n'a plus ces conseils derrière aussi.

00:23:01
*Mme. 1:* Ouais.

00:23:02
*Mme. 2:* Au supermarché, on n'a pas. Mais c'est vrai aussi, que le prix est plus élevé aussi chez le primeur.

00:23:06
*Mme. 1:* Ouais.

00:23:06
*Mme. 2:* Quand, [euh] quand j'avais tous mes enfants--, enfin--, tous mes enfants, j'avais-- j'avais pas--, j'allais au supermarché. Déjà dans un gain de temps, les courses, tout au même endroit. Puis, voilà. Là, bon, maintenant, j'ai le temps, donc je prends le temps plus de--, de--. [bah] je pense qu'un peu tout--, comme tout le monde, on--, on prend des choses, des meilleurs aliments, en fait. Moi, je joue sur ça et j'achète déjà beaucoup de produits français.

00:23:30
*Mme. 1:* D'accord.

00:23:30
*Mme. 2:* C'est en priorité les--, les--, les produits français. J'ai du mal un petit peu, si on veut un melon, on va pas acheter un marocain, on va attendre les français arrivent.

00:23:38
*Mme. 1:* OK.

00:23:40
*Mme. 2:* Voilà. Beaucoup privilégier, on va dire privilégier. Y a des moments-- Et pareil un couscous, je vais pas le faire en plein hiver, même si c'est la période--, on aime bien le couscous en plein hiver. Moi, je vais faire le couscous plutôt l'été, où y a les fruits du couscous-- qui sont de la période-- de la bonne période, quoi.

00:23:56
*Mme. 1:* Ouais

00:23:57
*Mme. 2:* Voilà donc plus ça quoi. Voilà.

00:24:00
*Mme. 1:* D'accord. Donc si je résume, c'est par-- [euh], si vous choisissez ces [euh], ces différents lieux pour vos achats, c'est en priorité la qualité, avec cette idée aussi que vous aimez pas que ce soit réfrigéré, et aussi pour privilégier les conseils directs avec-- [euh]

00:24:20
*Mme. 2:* Tout à fait, c'est important.

00:24:22
*Mme. 1:* Et en plus, [euh] de pays français, si c'est--

00:24:25
*Mme. 2:* Essentiellement, ouais. Ouais, prioritairement. Pas forcément local.

00:24:30
*Mme. 1:* Ouais.

00:24:30
*Mme. 2:* [Bah] si, pour les pommes de terre, tout ça, c'est du local.

00:24:33
*Mme. 1:* Oui.

00:24:33
*Mme. 2:* Mais [euh] en fait, non : français déjà. Moi, je trouve que français déjà c'est une--, on descend même dans le sud de la France [euh] au mois de juillet, si on n'a pas de melons, on adore les melons d'eau. Si on n'a pas du français, on n'achètera pas de l'espagnol.

00:24:46
*Mme. 1:* Ouais.

00:24:46
*Mme. 2:* On attend que le français soit là.

00:24:48
*Mme. 1:* D'accord.

00:24:48
*Mme. 2:* Et l'année dernière, il est arrivé trop tard, on n'en a pas mangé.

00:24:50
*Mme. 1:* D'accord.

00:24:51
*Mme. 2:* En fait, parce qu'ici [euh] ici, on a du--, du français, mais c'est très rare [hein]. Parce qu'on est en bout de chaîne, en général. On n'en a pas mangé.

00:24:59
*Mme. 1:* D'accord.

00:24:59
*Mme. 2:* Voilà donc [euh]. Voilà, voilà.

00:25:04
*Mme. 1:* Ok. Alors, c'est vrai que--, là, vous m'en avez déjà dit [euh] pas mal. Est-ce que vous pouvez me dresser une liste des différents fruits et légumes que vous consommez très régulièrement ? C'est du concombre, par exemple ? Ce genre de légumes que vous allez acheter tout le temps.

00:25:19
*Mme. 2:* Ouais. Concombres, salades tous les jours. Donc des carottes.

00:25:22
*Mme. 1:* Oui.

00:25:26
*Mme. 2:* [euh] Des carottes, des oignons, de l'ail. Alors là ! À toutes les sauces.

00:25:33
*Mme. 1:* Ok.

00:25:34
*Mme. 2:* Tout le temps. [euh] Beaucoup de courgettes aussi, on aime beaucoup la courgette.

00:25:39
*Mme. 1:* Ok.

00:25:41
*Mme. 2:* La courgette. Qu'est-ce qu'on aime ? Les poivrons. Je suis fan des poivrons. On aime les champignons. Beaucoup de champignons. Qu'est-ce qu'on aime ? les choux. Le chou, le chou-fleur. Mais alors les légumes, je crois que je les aime tous. Il aurait fallu prendre l'inverse : ce que j'aime pas. [rires]

00:26:00
*Mme. 1:* C'est ma suivante.

00:26:01
*Mme. 2:* Seulement ceux que j'aime pas. Parce que j'aime beaucoup, beaucoup, tous les--. Alors, qu'est-ce qu'on aime ? Les topinambours. J'adore les topinambours.

00:26:08
*Mme. 1:* Ouais.

00:26:08
*Mme. 2:* On mange la patate douce. Un petit peu particulièrement. On aime beaucoup. J'aime le panais. [euh] [aboiements] Ah non, c'est pas possible. Le fenouil, j'aime moins. Je suis moins fan du fenouil.

00:26:23
*Mme. 1:* OK.

00:26:23
*Mme. 2:* Donc ça c'est moins.

00:26:25
*Mme. 1:* OK.

00:26:26
*Mme. 2:* On va dire, mais je crois que c'est un des seuls moins parce qu'on--. Qu'est-ce que je mange ? Des tomates, évidemment.

00:26:31
*Mme. 1:* Oui.

00:26:33
*Mme. 2:* Tomates. [euh] C'est pas facile de se rappeler-- de voir-- enfin de voir tout, tout. Tous les légumes de saison, moi, j'arrive--, [euh] c'est presque-- [euh] le moment que je préfère c'est ça, c'est aller chez le primeur choisir les légumes. [ah] Qu'est-ce qu'on mange ? Les endives.

00:26:47
*Mme. 1:* Ouais.

00:26:47
*Mme. 2:* [Ohlala] qu'est-ce qu'on en mange quand c'est les-- [euh] l'hiver là. On mange plein d'endives.

00:26:51
*Mme. 1:* Ouais.

00:26:52
*Mme. 2:* Tous les jours, à la place de la salade, puisque c'est pas trop la salade en--. Alors, qu'est-ce qu'on mange d'autre ? Des poireaux. [Ah] oui ça aussi, en vinaigrette. On mange. [Euh] Du maïs. La journée du congé, donc je mets au barbecue par exemple.

00:27:07
*Mme. 1:* D'accord.

00:27:10
*Mme. 2:* Des trucs entiers. [Euh] Qu'est-ce que je mange ? Qu'est-ce que je mange comme légumes ? Des lent--, [bah] non lentilles c'est plus sec.

00:27:17
*Mme. 1:* Oui.

00:27:17
*Mme. 2:* Lentilles c'est les légumes secs. C'est vrai. Haricots blancs c'est légumes secs aussi. Haricots verts, quand c'est la période des haricots verts, petits pois.

00:27:26
*Mme. 1:* Oui.

00:27:26
*Mme. 2:* Haricots verts ou jaunes, peu importe la couleur. Des betteraves rouges, on mange beaucoup de betteraves rouges. Après [euh] des radis, des radis, [rires] je prends des radis. Radis noir.

00:27:43
*Mme. 1:* Ouais.

00:27:43
*Mme. 2:* Aussi le noir. On aime bien. On mange [euh], je cherche vraiment. Vraiment. Les--, les--, on a mangé, j'ai voulu faire une moussa--, une moussaka l'autre jour. Donc aubergines qu'on aime beaucoup. Les navets. Le rutabaga, j'adore. Tout. [rires] Tous les légumes mais moi je crois que je suis une grande fan de légumes, en fait. [euh] Ouais, plus plus, voilà, plus plus et. Alors, donc, je suis en train d'essayer de faire un petit peu le tour de ce que j'ai, ce que j'ai mangé ces derniers temps. Les asperges, c'est déjà les asperges. On en a mangé là parce que c'est la période des asperges. [euh] Je sais pas. J'en oublie sûrement. J'ai mon petit dossier là bas. [rires] Je vais le prendre, j'ai un-- j'ai un petit mémo, et puis je pense que--, j'arrive--. J'ai eu un petit mémo je trouvais que c'était sympa. Et là, [bah] voilà, j'en ai-- assez souvent des artichauts qu'on mange beaucoup. Les car--, j'avais dit les carottes déjà. Ah oui avec mon petit mémo je vais en retrouver plein. Le chou-fleur. [Ah] épinards : on n'en mange pas, parce que moi, j'aime bien, mais mon mari n'aime pas. Donc ça on mange pas. Il y a des choses qu'on mange pas. J'ai trouvé. La fève qu'on mange pas non plus.

00:29:07
*Mme. 1:* Ouais.

00:29:08
*Mme. 2:* Bon le reste, je vous avais dit tout le reste. Je vais faire comme ça. Comme ça, je les aurais tous. [marmonnements] Ça, c'est bon, c'est bon. [Ah] La mâche, non, j'aime bien. [Euh] Tout ce qui est salades différentes, de toute façon. La pomme de terre, évidemment.

00:29:20
*Mme. 1:* Ouais.

00:29:20
*Mme. 2:* Je vous l'ai pas dit mais c'est--, ça, c'est récurrent. Le brocoli, j'adore le brocoli ! Quand c'est la période. Le chou de Bruxelles. [Ah] J'adore ça aussi le chou de Bruxelles, on en mange quand c'est la saison, évidemment. L'hiver. Plus. [Marmonnements] Ça, on a fait le tour. Comme ça, j'ai tout-- je les ai tous fait, ça me donne des idées. Panais, j'ai dit. Topinambours. [Ah] salsifi. Salsifi j'en mange aussi, mais moins. Mais j'en mange quand même, parce que y a que moi qui mange.

00:29:48
*Mme. 1:* D'accord.

00:29:50
*Mme. 2:* [Euh] Du céleri. Céleri. Ça je mange beaucoup de céleri aussi. Potiron.

00:29:56
*Mme. 1:* Ok.

00:29:56
*Mme. 2:* On avait oublié potiron. Ça, c'est aussi souvent, souvent. Souvent, souvent. [Euh] [Toc, toc, toc] Ça, on est passé. Les petits pois, c'est fait. Après, je pense qu'on a fait le tour.

00:30:08
*Mme. 1:* Ouais.

00:30:09
*Mme. 2:* On en a pris pas mal. [rires] Je pense, ouais. Là, au niveau des--

00:30:14
*Mme. 1:* On en a... pas mal.

00:30:15
*Mme. 2:* On en a pas mal. Oui voilà. C'est surtout ceux que j'aime pas qu'il fallait prendre. [rires] Parce que tout le reste, on mange tout. Suivant la saison.

00:30:20
*Mme. 1:* Du coup. Sur ce que vous avez dit [euh] des-des légumes que vous mangez pas, vous avez dit : parce que mon mari n'en mange pas. Par exemple, vous avez dit les--

00:30:31
*Mme. 2:* Les épinards, j'achète pas.

00:30:32
*Mme. 1:* Ouais.

00:30:33
*Mme. 2:* Ouais. Mais je pense que aussi moi je suis pas-- je-j'aime bien, mais pas-- voilà, je suis pas une grosse fan, parce que j'aurais été fan, j'aurais mangé toute seule mon plat. Mais maintenant qu'on est à deux, en fait, on essaie de faire des plats pour manger à deux. Il y a que le concombre, mais c'est facile à faire.

00:30:48
*Mme. 1:* Ouais.

00:30:48
*Mme. 2:* Le concombre, il y a que moi qui mange ici, mais [euh] voilà. Le reste j'essaie de l'intégrer. Et mon mari [euh] il fait avec. Je mange souvent des légumes crus en hors-d'œuvre

00:30:59
*Mme. 1:* D'accord.

00:30:59
*Mme. 2:* On a souvent des crudités en hors-d'œuvre. Surtout en plus l'été, là. Beaucoup plus, quoi.

00:31:02
*Mme. 1:* Oui

00:31:03
*Mme. 2:* Des salades ou des crudités. Donc c'est pour ça. L'épinard. Ouais, je vois. Je pense que c'est ça, c'est parce que [euh] voilà, je pense que on--, je suis pas--, je cours pas après. Sinon, je pense que oui, voilà, j'en mangerais facilement.

00:31:14
*Mme. 1:* D'accord. Ce qui dirige votre consommation, du coup, de [euh] de légumes, là en l'occurence, c'est plutôt votre-votre attrait pour le--

00:31:27
*Mme. 2:* Pour tout ce qui est bon, j'aime bien tout ce qui est--, tout ce qui est légumes, tout ce qui est--. Moi, j'adore [hein]. J'en mange le matin, mais ça se ressent parce qu'en fait là, j'ai du-- j'ai un surpoids. Je le cache pas, j'ai arrêté de fumer, j'ai un surpoids.

00:31:39
*Mme. 1:* D'accord.

00:31:39
*Mme. 2:* Mais j'ai pas de problèmes de santé.

00:31:41
*Mme. 1:* D'accord.

00:31:42
*Mme. 2:* J'ai pas de cholestérol, j'ai pas de diabète, j'ai rien. Donc ça veut dire aussi que j'ai pas de tension.

00:31:45
*Mme. 1:* Oui.

00:31:45
*Mme. 2:* J'ai pas de médicaments, j'ai rien et je pense que l'alimentation, [euh] voilà, c'est essentiel pour la santé. Je pense que tous les légumes frais--, fruits et légumes frais dans l'alimentation, ça apporte tout ça. Je pense que, voilà, après ça, on a... on... Ouais, je pense que c'est essentiel toutes les herbes qu'on met dans l'alimentation, : la ciboulette, [euh] je mange du gingembre aussi. On a beaucoup de gingembre, des choses comme ça. Je pense que-- des noix. Tout ça, ça apporte, je pense des choses... pour la santé, déjà.

00:32:15
*Mme. 1:* Ouais.

00:32:16
*Mme. 2:* Essentiellement. Avant, je le voyais différemment, mais aujourd'hui, [bah] je pense que c'est plus ça.

00:32:20
*Mme. 1:* D'accord.

00:32:21
*Mme. 2:* Voilà.

00:32:21
*Mme. 1:* Et qu'est-ce qui... qu'est-ce qui fait que vous voyez ça différemment [euh] ?

00:32:25
*Mme. 2:* Parce que, avant, j'avais pas le temps.

00:32:28
*Mme. 1:* D'accord.

00:32:28
*Mme. 2:* Déjà. D'une, j'avais pas le temps. J'avais pas le temps de voir tout ça. J'avais pas le temps de réfléchir à ce que j'allais faire à manger, à ce que--

00:32:33
*Mme. 1:* Ouais.

00:32:33
*Mme. 2:* C'était des repas de famille et puis c'était-- Et l'alimentation a changé. Je pense que aujourd'hui, [euh] on voit beaucoup à la télé, tout ça, enfin-- On en parle plus et je pense que [euh] on est un peu plus alerté sur ces choses-là.

00:32:48
*Mme. 1:* Ouais.

00:32:49
*Mme. 2:* En se disant attention. Et moi, c'est un plaisir pour moi déjà, de manger des fruits et légumes, c'est un plaisir. Donc, comme c'est un plaisir, [bah] j'y vois pas, ouais j'y vois pas trop de contraintes, en fait. Je me dis c'est d'abord un plaisir de manger, [euh], de manger sain. Voilà. Je suis... je suis plus légumes que fruits. J'aime bien les fruits, certains fruits.

00:33:08
*Mme. 1:* Ouais.

00:33:09
*Mme. 2:* Mais je suis plus légumes. Mon mari aime plus les fruits. Voilà, l'inverse. Mais il mange quand même [hein]. Il suit [hein].

00:33:14
*Mme. 1:* Ouais.

00:33:16
*Mme. 2:* [rires] Il est comme moi. Il sait que c'est bon pour la santé, quand même. Voilà.

00:33:18
*Mme. 1:* D'accord et du coup, sur les fruits, [euh] pareil est-ce qu'il y en a que vous consommez-- la pêche--

00:33:28
*Mme. 2:* Et la mandarine.

00:33:29
*Mme. 1:* Et la mandarine. Dès que vous pouvez ?

00:33:31
*Mme. 2:* Ah ouais. L'orange.

00:33:32
*Mme. 1:* L'orange, aussi ?

00:33:33
*Mme. 2:* Jus d'orange. Ouais. Jus d'orange essentiellement.

00:33:35
*Mme. 1:* Vous le faites--, c'est vous qui le faites ?

00:33:38
*Mme. 2:* Ouais c'est moi. Pas toujours [hein]. Pas toujours. J'ai toujours [euh], là, [euh] j'ai toujours du jus de fruit au frigo, mais tout fait aussi.

00:33:45
*Mme. 1:* Oui.

00:33:45
*Mme. 2:* Mais autrement je fais-, je fais--, j'ai une--, j'ai-- comment-- pas une centrifugeuse. Un extracteur.

00:33:50
*Mme. 1:* D'accord.

00:33:50
*Mme. 2:* De jus. Donc je fais moi-même mes jus de pommes.

00:33:54
*Mme. 1:* D'accord.

00:33:54
*Mme. 2:* Déjà. [Ah] Des kiwis aussi j'en mange. J'aime bien les kiwis.

00:33:55
*Mme. 1:* OK.

00:33:58
*Mme. 2:* Je fais moi-même mes jus. Enfin. Beaucoup de jus. On change un peu, mais pas mélanger légumes et fruits. Que des jus de fruit.

00:34:04
*Mme. 1:* D'accord.

00:34:04
*Mme. 2:* J'aime pas les... mélanges. J'adore la concombre mais faut pas la mélanger avec autre chose. Je trouve ça dégueulasse. [rires] Et jus de betterave, merci. On a essayé. C'est dégueulasse. [rires] Donc, j'aime bien-- ouais, j'aime bien tous les fruits, les jus de fruit [euh] un peu particuliers. Mais bon, l'orange, la pomme. Alors, la pomme, moi j'en mange plutôt cuite.

00:34:25
*Mme. 1:* D'accord.

00:34:25
*Mme. 2:* J'ai tendance à manger de la pomme plutôt cuite que crue.

00:34:27
*Mme. 1:* D'accord.

00:34:28
*Mme. 2:* Je mange [euh] plutôt-- Voilà. Mais mon mari, c'est l'inverse. Il aime mieux cru, mon fils aussi, donc [euh]. On a tendance à manger ça. On mange un peu de banane. Moi, moins. Moi, c'est vraiment la mandarine, la pêche, [euh] le raisin, quand c'est la période du raisin, j'adore. [euh] J'aime bien l'abricot. Mais ici on a-- je trouve que les abricots--, j'en ai déjà acheté, là. J'ai acheté deux ou trois abricots pour--, histoire de les goûter. Ils étaient très chers. C'est pas bon. Mais, alors les fraises, là, je prends une barquette pour moi toute seule, juste pour un quatre heures. [rires] Je suis fan des fraises, mais pas autant que la pêche et pas autant que la mandarine. Mais j'aime la fraise. J'aime la fraise, quoi. Voilà. La fraise, la framboise.

00:35:13
*Mme. 1:* D'accord.

00:35:14
*Mme. 2:* Des fruits exotiques aussi, un peu. L'ananas, qui est bon aussi. J'aime beaucoup l'ananas. On achète- on achète de temps en temps. Bon, pas souvent, mais après, chez le primeur, ils le coupent en fait.

00:35:24
*Mme. 1:* D'accord.

00:35:25
*Mme. 2:* Ils le mettent dans un appareil. Ils le coupent. Donc il est frais, quoi. On rentre à la maison il est épluché, il est coupé. Ça, c'est quand même bien pratique. Ça, voilà. Après [euh], pamplemousse. Aussi on mange du pamplemousse.

00:35:36
*Mme. 1:* D'accord.

00:35:36
*Mme. 2:* [euh] En salade, ou avec [euh] de l'avocat, des crevettes-- Enfin, on les-- voilà. Pamplemousse, on en mange pas mal aussi.

00:35:43
*Mme. 1:* Ok.

00:35:44
*Mme. 2:* Et... puis voilà. Moi, je me dis que ça c'est--, ça a beaucoup d'apports de vitamine C. Donc ça évite de prendre des médicaments qui font des apports complémentaires, des compléments alimentaires. Je pense que moi, j'ai-- on n'a pas besoin de compléments alimentaires, parce ce qu'on trouve tout ça dans nos fruits et dans nos légumes. Je pense que c'est surtout ça : ça évite de prendre des médicaments. Des choses qu'on connaît pas trop. Voilà. Après, [euh] vitamines et-- Pour la santé, toujours [hein] toujours. Puis, plus on vieillit plus on doit faire attention à sa santé. [rires] Je crois que quand on est jeune, jeune, on le fait de moins en moins. Puis, plus on vieillit, plus on se dit : [ohlala] il serait peut-être bien de-- voilà. Pour la santé. Et on voit plus les choses, maintenant, aujourd'hui, de la même façon qu'avant, je pense.

00:36:26
*Mme. 1:* Ouais.

00:36:26
*Mme. 2:* Donc voilà. C'est ça essentiellement. Après, les mandarines, alors il y a tellement de variétés de mandarines. Chez le primeur, je me suis fait plaisir : des roses, des-des-des-- toutes les variétés de mandarines, de la clémenvilla, enfin toutes les-- tous les- tous les mélanges. Les lignées, l'audace, qui sont des mélanges pamplemousses, toutes les variétés, c'est plus des mandarines [Hein].

00:36:46
*Mme. 1:* Ouais.

00:36:46
*Mme. 2:* C'est plus des mandarines, c'est plus des clémentines. Mais toutes ces variétés d'agrumes, on va dire, d'agrumes. Parce que j'aime bien le citron aussi. La bergamote. J'aime bien.

00:36:54
*Mme. 1:* D'accord.

00:36:55
*Mme. 2:* J'aime beaucoup le citron vert, citron jaune. Voilà, des choses comme ça, des choses un petit peu différentes aussi. Voilà, et puis l'été, les fruits de s--, les fruits d'été qui sont très bons. Les cerises qui sont bonnes aussi, on mange des cerises en pleine période. Mais là, c'est pareil, faut qu'on prenne tout ça, quoi. Le kiwi, je mange. La mangue que j'aime beaucoup. Et là, c'est quand même très cher. Un truc qu'on mange pas beaucoup c'est la poire. Mais mon mari aime bien, mais comme c'est moi qui fais les courses, je lui en achète jamais, mais faudrait-- à chaque fois, je dis faut que je lui en achète parce que franchement... Mais non. Bon voilà. Mais les ananas on mange. Clémentines. Grenades, non. Grenades, non on n'en mange pas particulièrement. Ça arrive que je prenne à Noël des grenades et des petits... [euh] comment on appelle ça... des litchis.

00:37:41
*Mme. 1:* Ouais.

00:37:41
*Mme. 2:* Mais c'est toujours à Noël. Parce que je trouve, c'est des fruits exotiques, donc bon, réveillon tout ça, je sais pas trop. L'avocat qu'on considère comme un fruit aussi, qu'on mange beaucoup.

00:37:52
*Mme. 1:* Ok.

00:37:52
*Mme. 2:* Ça on mange beaucoup. On a quoi ? On a le kaki, tout ça on mange pas. En fait, ça fait pas partie--, tout ce qui est fruits exotiques, on un peu plus-- on aime, moi, j'aime beaucoup. On mange du melon, beaucoup de melon. La pastèque. ouais, melon, pastèque, melon d'eau. On aime beaucoup ça aussi. Les petits fruits : cassis, tout ça, un peu moins. La rhubarbe, on n'aime pas. Je crois que c'est le seul. Ouais, la rhubarbe on n'aime pas. Le reste, on le mange : cerises, fraises, mangues. Mangues, ouais un peu moins parce que ouais aussi--, mais on mange quand même, j'aime bien.

00:38:27
*Mme. 1:* Ouais.

00:38:27
*Mme. 2:* Franchement. [Bah] oui, je dirais que c'est juste ça, c'est tout. Le reste, on mange un tout petit peu plus. Après toutes les saisons et la poire. Moi, je devrais faire des efforts pour mon mari quand même, parce que le pauvre. Voilà. Mais la rhubarbe, non. J'aime pas et on fait beaucoup de confitures à la rhubarbe. Non.

00:38:44
*Mme. 1:* Même pas en confiture.

00:38:44
*Mme. 2:* Voilà. Ça c'est la seule. Y a des choses comme ça. La mûre. On va chercher les mûres. On va chercher les mûres. Ah, j'ai oublié de dire la châtaigne aussi, pour les légumes. On va chercher les châtaignes. Et on les cuit après ? On les met au congèle ? Voilà. Voilà, voilà.

00:39:02
*Mme. 1:* Ok.

00:39:02
*Mme. 2:* Je pense qu'on a fait le tour là. J'ai réussi à tous les trouver. [rires]

00:39:07
*Mme. 1:* Et du coup, à un moment donné vous avez parlé de l'ananas et vous avez raconté que c'était [euh] pratique, que-que votre primeur l'ait coupé. Est-ce que vous pensez qu'il y a parfois des fruits ou des légumes que vous allez consommer [euh] davantage ou au contraire moins, selon un côté pratique ? Ou non, c'est vraiment la question de goût qui va vous diriger ?

00:39:31
*Mme. 2:* [Bah] côté pratique, l'ananas certainement. Je crois que c'est-- Mais ce qui me dérange un peu aussi, c'est que... on a plus de mal à manger de fruits- de fruits exotiques en se disant qu'ils ne sont pas du froid, quoi.

00:39:42
*Mme. 1:* D'accord.

00:39:42
*Mme. 2:* Pour le reste, on en est-- Même les bananes [hein]. On en a [hein]. Moi, j'ai toujours--, parce que mon fils adore les bananes, mais c'est-c'est-c'est voilà. Après, c'est parce que aussi, on a moins- on a moins-- enfin, chez moi. Enfin, faut être- faut être logique aussi [hein]. Mais après, ici à la maison, ils sont pas souvent en train de me dire : tiens tu devrais prendre plus de ça, plus--. Non, non ils suivent le mouvement, donc ils prennent ce que je prends. Et c'est vrai qu'en fruits exotiques--, la mangue, j'adore, bon après [euh] et c'est cher, c'est très cher, bon ça vient pas d'à côté, alors j'trouve, voilà. Je veux dire, nous, c'est des fruits qu'on va manger pour Noël.

00:40:17
*Mme. 1:* D'accord.

00:40:18
*Mme. 2:* Même l'ananas [hein]. L'ananas, c'est plus souvent-- voilà c'est une période de plaisir. À un moment, à un moment vraiment on va faire des choses différentes avec, quoi. Mais [euh] non pas-- voilà. Après... L'avocat qui vient pas souvent d'ici non plus. Mais ça on en mange beaucoup, parce qu'on aime l'avocat. On aime beaucoup l'avocat.

00:40:34
*Mme. 1:* D'accord.

00:40:34
*Mme. 2:* Donc là, on ferme les yeux un peu. Ça peut--, c'est plus un peu au goût quoi. En fait voilà. Je pense que c'est-- Voilà.

00:40:41
*Mme. 1:* C'est le goût-- Enfin c'est le goût et le plaisir d'abord.

00:40:45
*Mme. 2:* C'est ça, d'abord.

00:40:46
*Mme. 1:* Et si c'est, du coup, un peu moyen. Et ben là, vous allez penser qu'en plus, il y a d'autres faits qui-- Voilà.

00:40:52
*Mme. 2:* Voilà. C'est ça. Je laisse de côté. On va dire que voilà. J'ai pas-- Ouais. Il y a des choses que je suis encore sceptique sur certains aliments, comme les bananes. Ça, ça me dérange, les bananes de savoir-- [bah] on sait pas si elles sont vraiment bio, si elles sont pas bio, si elles sont pas-- Parce que bio, je suis pas bio.

00:41:11
*Mme. 1:* Oui.

00:41:11
*Mme. 2:* Du tout. Tout ce qui est bio, c'est absolument pas une-un critère de choix pour moi.

00:41:17
*Mme. 1:* D'accord.

00:41:18
*Mme. 2:* Donc pour moi, je pense que ça suffit d'acheter-- Je-je pense que-- enfin même-- on a vu des émissions. On a fait des émissions, on s'aperçoit que bon, c'est sûr que le bio a des-des choses qui sont un peu plus positives. Mais on n'est pas des pro-bio du tout.

00:41:34
*Mme. 1:* D'accord.

00:41:34
*Mme. 2:* On va pas aller au rayon bio.

00:41:35
*Mme. 1:* D'accord.

00:41:36
*Mme. 2:* On n'y passe même pas, on s'arrête même pas. Voilà. Donc [euh]. Pourquoi ? Pour-- C'est comme ça. Après, je me dis que c'est trop-- c'est plus cher aussi. On va pas en rajouter une couche encore. Et je suis plus dans le local. Voilà. Je suis plus dans le local. Enfin, en priorité.

00:41:50
*Mme. 1:* D'accord.

00:41:52
*Mme. 2:* Plus que dans le bio. Voilà. Si on va dans le sud de la France, on va pas acheter [euh] des pêches qui viennent de l'autre bout de--. Alors qu'on a des pêchers partout, ils sont tellement nombreux les pêchers avec leurs grosses pêches. On n'a pas besoin d'aller plus loin. [rires] Voilà.

00:42:05
*Mme. 1:* Ok. Très bien. [euh] Alors est-ce qu'on pourrait revenir un peu à vos stratégies de stockage des fruits et légumes ? Vous en avez déjà parlé au tout début. [euh] Si on prend les fruits, du coup : est-ce que vous pouvez me dire un peu, toutes les [euh], voilà, toutes les stratégies que vous mettez en place, les choses que vous savez sur la conservation, pour-- [euh] voilà ?

00:42:31
*Mme. 2:* Alors, les fruits déjà, [bah] toujours au frais. Au frais, pas forcément au frigo. Au frais et avec le moins de lumière possible

00:42:37
*Mme. 1:* D'accord.

00:42:38
*Mme. 2:* Voilà. Et dans des caisses aérées, pas les uns sur les autres.

00:42:42
*Mme. 1:* D'accord.

00:42:43
*Mme. 2:* Dans des caisses, les caisses à champignons, c'est très bien pour ça. [inaudible 00:42:46-00:42:50] [rires] Les caisses à champignons. Voilà. C'est-- Donc [bah] des-des-- chaque étage, même s'il y a que deux tomates, chaque étage a aéré les fruits--, qu'ils aient de la place, en fait. Plus, ils sont tassés, puis si un fruit pourrit, évidemment b [bah] a va-, a va gêner tout le monde. Ça va pourrir tout le monde. Quoi. Donc [euh]

00:43:06
*Mme. 1:* Ouais.

00:43:06
*Mme. 2:* Et puis, je prends juste la petite quantité qu'on a besoin ici.

00:43:09
*Mme. 1:* D'accord.

00:43:09
*Mme. 2:* Je mets juste toujours--, j'ai un petit saladier.

00:43:12
*Mme. 1:* D'accord.

00:43:13
*Mme. 2:* Où j'ai un tout petit peu. J'ai un petit peu de-de fruits, pas-pas beaucoup. Un tout petit peu. Là, j'ai deux mandarines, deux oranges. Voilà. Pour-- [euh]. Les autres oranges sont là-bas, pour si quelqu'un a envie de manger une orange, parce que, c'est vrai, mon fils, il aime bien, donc [euh]. Voilà. Pour [euh], pour avoir, pour les avoir là. Il serait aussi bien d'y aller là-bas, mais c'est une question pratique. Surtout que quand la voiture est dans le garage c'est moins facile d'aller chercher les fruits. Après [euh] tout ce qui est [euh] un peu abîmé, tout ça on met de côté. On fait des choses avec.

00:43:44
*Mme. 1:* D'accord.

00:43:44
*Mme. 2:* On les cuisine donc [euh], ou on les congèle. Donc, si je vois, voilà, si j'arrive à la maison, je vois qu'il y a un fruit ou un légume, [euh], par exemple, une banane un peu trop mûre, je la coupe et je la congèle. Je vais faire des smoothies avec. Même si c'est pas prévu. Des fraises un peu abîmées, voilà pareil. Ou des pommes. Je vais faire une compote improvisée, je vais faire : tiens, j'ai deux pommes qui sont un peu abîmées. J'enlève le petit morceau qui est abîmé, et [hop] je vais le- je vais le cuire facilement. Ou le congeler. Ça dépend des fruits et des légumes, quoi. Voilà. Et toujours dans le garage, jamais au frigo, sauf quand ils sont entamés.

00:44:22
*Mme. 1:* D'accord.

00:44:24
*Mme. 2:* Voilà. Et du coup, le-les betteraves rouges cuites--, quand ils sont cuits aussi-- du coup, quand ils sont cuits, les légumes, [bah] je les- je les garde au frigo. Et la salade donc que je garde dans un saladier, dans un-un tupperware- un tupperware-- dans un-une caisse avec-- y a un petit système de-- c'est chez Ikea qu'on trouve ça-- un petit système d'aération.

00:44:46
*Mme. 1:* D'accord.

00:44:46
*Mme. 2:* Mais je mets du sopalin dessus en fait, ça absorbe l'humidité constamment. Ça, en fait, ça empêche qu'elle pourrisse. Tout en gardant [euh], en gardant sa fraîcheur. Après, autrement, voilà. Tout ce qui est fruit, c'est ça, en général. Ouais.

00:45:03
*Mme. 1:* D'accord. Et du coup, est-ce que pour vous, c'est le-- la manière dont vous disposez vos fruits, c'est le-le stockage idéal ou pas ?

00:45:10
*Mme. 2:* [Bah] y a toujours mieux [hein].

00:45:13
*Mme. 1:* Ouais.

00:45:13
*Mme. 2:* Mais pour moi, pour nous, ça nous convient bien, dans le sens où on a-- on jette très peu de fruits.

00:45:19
*Mme. 1:* D'accord.

00:45:19
*Mme. 2:* Et donc, ça veut dire que c'est entreposé d'une manière correcte. Puisqu'on avait essayé plusieurs systèmes. On a essayé des systèmes : on a mis au frigo, on mettait tout au frigo. Après, ça avait plus de goût. Ça enlève le goût, le frais, je trouve, ça enlève le goût. Et puis [ben] pour nous, c'est la conservation idéale. Pour nous.

00:45:36
*Mme. 1:* D'accord.

00:45:37
*Mme. 2:* Pour nous. Alors après, voilà, si c'est-- Nous, on s'aperçoit que c'est bien pour nous parce qu'on jette pas beaucoup. C'est surtout ça. C'est surtout de voir ça. Voilà, une salade, si j'ai besoin d'une salade je vais chercher dans le jardin et je la coupe, je l'entrepose. Mais je vais pas aller la prendre si j'en n'ai pas besoin. Dire : tiens je vais prendre une salade comme ça, non, voilà. Je vais prendre-- Et chez les légumes--, les fruits et les légumes, c'est pareil. Je vais pas prendre une salade si on n'est pas là deux jours. Je vais dire : [bah] je vais pas prendre une salade pour l'entreposer, puis pas la manger, quoi. Pour nous, ça nous convient.

00:46:07
*Mme. 1:* Oui.

00:46:08
*Mme. 2:* Voilà. Je pense qu'on a trouvé l'équilibre au niveau des fruits et légumes. Voilà.

00:46:12
*Mme. 1:* Très bien. Mais c'est en observant vos-vos déchets que vous vous êtes rendus compte petit à petit que-- ?

00:46:21
*Mme. 2:* Oui. Beaucoup de ça. De-de-- Mon mari disait : Oh c'est-- quel-quel dommage de jeter une pomme pourri. On trouve ça terrible. On s'est dit : faut qu'on fasse-- qu'on trouve une solution.

00:46:30
*Mme. 1:* Ouais.

00:46:31
*Mme. 2:* Qu'on aille--, qu'on mette dans des cageots. Les pommes séparément. Juste une rangée de pommes. Y en a dix rangées. C'est vrai qu'il y a beaucoup de caisses. Enfin, les pommes-- quand les pommes sont là, il y a une partie qui est prise. Et puis, on se dit : [bah] tiens, comme ça, ça per-- et puis, on peut les sortir et vérifier. On dit : tiens, de temps en temps, on soulève la caisse. Ah, il y en a pas une pourrie, ou un peu tâchée. On peut vérifier régulièrement le stock. C'est surtout ça, aussi. C'est de pouvoir voir quand on a des oignons qui sont étalés dans le-dans le-- On peut soulever, puis regarder, mais on-- voilà. Puis la qualité. On a trouvé aussi des choses qui font que-- moi, j'ai été dans un autre-- j'ai fait des primeurs. Je sais qu'il y a-- dans la rue il y a plusieurs. Il y a des magasins aussi de- de ferme [hein], on a un magasin pas loin. Et, ben, la qualité était pas là.

00:47:13
*Mme. 1:* OK.

00:47:13
*Mme. 2:* Donc, j'ai arrêté et je suis reparti. Je suis parti chez un autre primeur. Et j'ai trouvé un primeur qui me convient. Parce qu'il a des produits qui me conviennent aussi. Qui sont frais, parce que [euh] y a certaines personnes qui vont acheter des-des-des fruits et légumes pour le jour même, donc [bon] ça pose moins de problèmes. Mais moi, pour tenir une semaine dans notre façon de vivre, [bon], on a trouvé que c'était-- ça nous convenait, en fait, comme ça. Mais au fur et à mesure, c'est pareil. On a-- on s'est rendu compte, on s'est dit tiens on va aller-- Après, j'ai ma-ma soeur- ma soeur a une formation de fruits et légumes, elle a fait un BTS en fruits et légumes.

00:47:47
*Mme. 1:* D'accord.

00:47:47
*Mme. 2:* Elle a fait une formation initiale à Saint-Paul. Et donc, souvent elle est de bon conseil. Elle est diététicienne, en plus. Donc-- Elle est de bon conseil. Elle me dit : voilà. On en a discuté. Puis, toi, tiens tu fais comment ? Parce que moi j'ai des soucis avec ça. Puis elle me dit : [bah] tiens tu fais ça, c'est pas mal, c'est une solution. [Hop], alors, j'ai commencé à congeler, à pocher par exemple mes pêches. Au départ, je congelais pas, on faisait des confitures. Et puis, on s'est dit tiens, c'est vrai que faire des glaces et machin--, on va apprécier de les avoir après. [Bah] j'ai commencé à faire ça : les pocher et puis, voilà, au fur et à mesure, en étant attentive à ce que les gens me disent. Et puis, essayer ouais, de faire- de faire ça. Donc voilà. Pour nous, c'est la meilleure-- notre manière- cette manière-là. D'ailleurs, on le voit à la poubelle. Parce que la poubelle, on a ça dans la poubelle. J'ai mon mari qui dit : t'as mis quelque chose à la poubelle ? J'dis : Oh, j'ai mis de la salade, il y avait des petits bouts un peu abîmés. T'sais la salade. Et hier, y avait-- [euh] j'avais trois bouts de radis avec des vers. Mais je pense que j'en ai mangé avec des vers avant [rires] et je-je crois que dans la liste, il y avait des verts et je crois que j'en ai mangé avant. [rires] Bon bah, voilà, je-je vais pas les manger. Même avec tous les efforts du monde [inaudible 00:48:50-00:48:51] et la poubelle, voilà, j'ai pas vidé le sac. Y a rien dedans.

00:48:54
*Mme. 1:* Ouais.

00:48:54
*Mme. 2:* Y a un peu de feuilles de salade, de radis, un bout de concombre, je crois. Et une patate qui était-- je sais pas peut-être qu'elle était molle. Une patate qui était molle.

00:49:00
*Mme. 1:* Oui.

00:49:01
*Mme. 2:* Voilà. C'est tout, quoi.

00:49:02
*Mme. 1:* Ok, je vois. Et du coup, vos stratégies de stockage pour les légumes, ils s'apparentent aux-- à ceux que vous utilisez pour les fruits ?

00:49:14
*Mme. 2:* Ouais. C'est à peu près pareil.

00:49:14
*Mme. 1:* Ou y a des différences ?

00:49:15
*Mme. 2:* Non, c'est pareil aussi. C'est un stockage dans le garage aussi [euh]. À part les pommes de terre. Je filme à l'ombre les pommes de terre dans du papier journal à l'ancienne. Comme faisait ma grand-mère, en fait.

00:49:25
*Mme. 1:* Oui.

00:49:25
*Mme. 2:* Et elle les conservait bien comme ça. Donc [bah] j'ai gardé la- j'ai gardé la façon de faire et je les laisse là. Mes enfants. Ils les emmènent chez eux. Ils conservent pas de la même manière. Donc elles germent, elles sont vertes. Donc, finalement, il les laissent là, ils viennent les chercher comme il viennent là au moins trois ou quatre fois par semaine. Ils prennent leur panier, ils ont un filet, puis ils prennent leurs pommes de terre. Et on entrepose comme ça, parce qu'on trouve que c'est quand même mieux. Ça-ça se tient bien. Parce que, c'est vrai, ça aurait pas bien tenu. On aurait arrêté cette manière-là de-d'acheter des légumes, mais ça fait longtemps. Ça fait-- ouais ma collègue ça fait même 12-13 ans que je prends avec elle, quoi. Donc voilà. Et puis la qualité est là, sans doute [hein]. Parce que la qualité n'aurait pas été là, j'aurais arrêté.

00:50:05
*Mme. 1:* Oui.

00:50:06
*Mme. 2:* À prendre des pommes de terre qui sont pas bonnes. Donc voilà, la stratégie, elle est à peu près la même. Ouais, voilà. Sauf que les quantités sont plus élevées. Puisque pommes de terre, oignons, tout ça, c'est des grosses quantités.

00:50:18
*Mme. 1:* D'accord.

00:50:18
*Mme. 2:* Mais c'est pareil. C'est dans des casiers, puis on-on met pas un casier tassé d'oignons. Puis là, c'est plusieurs casiers. Là, c'est un peu maigre. C'est un peu vide là maintenant, puisqu'on arrive au bout de tout. Donc voilà.

00:50:31
*Mme. 1:* D'accord. Et est-ce qu'il vous arrive de nettoyer [euh] ces endroits de stockage ou pas ?

00:50:38
*Mme. 2:* [euh] Les casiers à champignons ? Parfois, je passe un coup de balayette, la petite balayette--

00:50:42
*Mme. 1:* D'accord.

00:50:43
*Mme. 2:* Pour enlever les pouloutes, mais jamais-- Je nettoie jamais, non. Je suis pas pour une hygiène stricte, en fait. Je pourrais [hein] mettre de l'eau de Javel, désinfecter, machin. Je n'y touche pas. Juste un coup de balayette, pour enlever les épluchures, machin, la poussière qu'il y a derrière je les détale, mais non, non, non : pas de- pas de spécificité au niveau de--

00:51:05
*Mme. 1:* Du nettoyage des cagettes.

00:51:05
*Mme. 2:* Non.

00:51:08
*Mme. 1:* Ok. Alors [euh] donc là, c'est plus sur la partie de préparation et de consommation des fruits et légumes. C'est pareil, on a déjà-- vous avez déjà abordé pas mal de choses. Donc, prenons un truc que vous avez dit vous consommez très régulièrement. Donc vous avez dit la pêche, mais vous avez déjà sorti pas mal de choses sur la pêche. Donc on va peut-être en trouver un autre.

00:51:29
*Mme. 2:* La pomme, alors.

00:51:30
*Mme. 1:* La pomme. Et est-ce que vous pouvez me dire justement, un peu toutes les formes que vous pouvez la manger ? Votre mari la mange crue, vous-vous la mangez--, vous la préférez cuite. Est-ce que vous pouvez me raconter un peu-- ?

00:51:42
*Mme. 2:* [Bah] la pomme, en vrai... On fait des tartes aux pommes, des-des-des tartes, des gâteaux aux pommes. Quand c'est la période des-- on fait beaucoup de beaucoup de-- des muffins aux pommes. Je fais tout plein d'autres choses aux pommes. Je suis fan de pommes, alors [euh]. Je fais des pommes avec du boudin.

00:51:59
*Mme. 1:* Oui.

00:51:59
*Mme. 2:* Je fais des pommes [euh] au caramel avec un dessert. [euh] Je fais des pommes [euh] [pff]-- Mon-mon mari fait des compotes puisque lui, c'est compotes, confitures. Lui c'est plus ça. Moi, j'ai pas la patience, en fait, de faire. J'aime bien cuisiner, mais là-- Alors, le fait de stériliser les pots, ça moi, ça me gave. Lui, il fait ça. On fait des compotes. On fait-- Qu'est-ce qu'on fait d'autres [euh] ? Un bout de gâteau. Mais on peut faire un rôti de porc aux pommes, par exemple, des choses comme ça. Pommes-pruneaux, pommes-noix. Les salades. Salades-salades composées.

00:52:34
*Mme. 1:* Ouais.

00:52:34
*Mme. 2:* Avec des pommes. Les pommes Granny, j'aime assez [euh] j'aime assez associer avec du saumon, des choses comme ça. Voilà. Les-- toutes les sauces pour la pomme voilà, la pomme c'est ça, mais des autres fruits aussi qui sont-- qui peuvent s'associer, que ce soit l'orange avec du canard, ou quelque chose comme ça. Tous les- tous les fruits les accompagnent- les accompagnent différemment. Après, c'est vrai, moi, j'aime bien- j'aime bien [euh] plus de cette manière-là. À part les oranges, les mandarines et... les prunes aussi. On avait dit les prunes? Je mange des prunes aussi. [rires] Mais j'aime mieux--, voilà, j'aime bien accompagner. J'aime le sucré-salé, tout simplement dans la cuisine. Donc ça aussi, ça-ça aussi, c'est mon homme. Voilà. Donc [euh]. Et on lui fait à lui, la pomme c'est ça. Et puis- et puis on a du-- des kiwis un petit peu abimé, on va faire aussi des compotes avec de la pomme. Comme on a tout sous le coude, on va faire une petite compote. Allez, une petite compote flash. Dans les yaourts aussi. On aime bien mettre un petit peu de- un petit peu de fruits. Je fais mes yaourts et puis je mets des-- un petit peu de confiture de fraises, un petit peu de fruits coupés aussi. Souvent. Voilà.

00:53:50
*Mme. 1:* Ok. Et les légumes ? Prenons un légume que vous avez dit manger beaucoup. Alors, y a--

00:53:59
*Mme. 2:* On mange beaucoup de légumes.

00:54:01
*Mme. 1:* [rire] C'est pour ça.

00:54:02
*Mme. 2:* Les poireaux, on mange beaucoup aussi.

00:54:03
*Mme. 1:* [Bah] prenons le poireau par exemple.

00:54:04
*Mme. 2:* [Ben] le poireau--

00:54:04
*Mme. 1:* À quoi vous le mangez ? Comment vous le mangez ?

00:54:07
*Mme. 2:* Ben je vais le manger en pot-au-feu, en kit à farce, en cuit. Et alors, cuits à vapeur. J'adore. On adore avec un œuf dessus.

00:54:15
*Mme. 1:* D'accord.

00:54:16
*Mme. 2:* Un œuf cuit dessus, un peu. Mimosa, là. On aime bien [euh] le poireau que-que-- en soupe. On mange beaucoup de soupe. Tous les jours, on mange de la soupe l'hiver et tous les jours, on change de soupe. Tous les jours on change de soupe. Je fais une soupe pour cinq jours. Je congèle ma soupe dans mes casiers.

00:54:34
*Mme. 1:* D'accord.

00:54:34
*Mme. 2:* Et comme ça, ça nous permet, comme on n'est qu'à deux, de changer de soupe. Mais j'en ai au congèle, toujours. Plein. Là, c'est vide. Pareil, c'est pas la période. Et j'ai des boîtes, en fait, qui font une soupe pour deux jours.

00:54:45
*Mme. 1:* D'accord.

00:54:46
*Mme. 2:* Donc, je fais aussi beaucoup de soupes de poireaux. C'est--, ça c'est très bon.

00:54:49
*Mme. 1:* Ouais.

00:54:49
*Mme. 2:* On fait [inaudible 00:54:49] avec du poireau. Quand j'achète-- quand c'est-c'est-- bon là j'ai du [inaudible 00:54:54] au congèle encore, mais avec une poêlé de-de poireaux, c'est excellent. Qu'est-ce qu'on... [pfff]. Oui, le poireau, y a une tarte, une tarte aux poireaux. C'est très, très bon. Les tartes [euh], les tartes, les tourtes, la béchamel, des choses comme ça. Voilà. Plein, plein, plein de choses. Comme moi, j'aime beaucoup cuisiner et que j'ai le temps, je cuisine beaucoup beaucoup. Ouais, ouais.

00:55:25
*Mme. 1:* Ok. Chouette. Alors, donc c'est vrai que vous les cuisinez beaucoup.

00:55:30
*Mme. 2:* Ouais.

00:55:30
*Mme. 1:* Et d'ailleurs vous avez dit que vous préfériez souvent ce qui est cuit, plutôt que ce qui est cru. Et, du coup, ça veut dire qu'il y a une préparation faite avec le fruit ou le légume. Est-ce que vous pouvez me raconter, essayer de trouver peut-être des fruits et des légumes que vous n'épluchez pas, par exemple ? Avant de les-les consommer.

00:55:47
*Mme. 2:* Les carottes nouvelles.

00:55:49
*Mme. 1:* D'accord.

00:55:49
*Mme. 2:* Les pommes de terre nouvelles. J'épluche jamais.

00:55:52
*Mme. 1:* OK.

00:55:54
*Mme. 2:* [Euh] J'épluche pas les pommes de terre pour la raclette.

00:55:57
*Mme. 1:* D'accord.

00:55:58
*Mme. 2:* Par exemple. Je mange la peau avec.

00:56:03
*Mme. 1:* OK.

00:56:04
*Mme. 2:* On mange la peau avec. [euh] Même une salade. Quand je les cuit. Je cuis beaucoup à la vapeur mes légumes.

00:56:09
*Mme. 1:* D'accord.

00:56:10
*Mme. 2:* Alors, qu'est-ce que j'épluche pas ? Qu'est-ce que j'épluche pas ? Jamais le--, jamais la courgette.

00:56:16
*Mme. 1:* D'accord.

00:56:18
*Mme. 2:* Jamais le concombre non plus.

00:56:21
*Mme. 1:* D'accord.

00:56:21
*Mme. 2:* Je mange tout avec la peau. J'enlève pas beaucoup la peau [hein] des-des-- Qu'est-ce que-- dans quoi on enlève la peau ? Dans quoi on enlève la peau ? Ben, alors là ? Même l'ail, je mets en chemise dans les rotis. Avec la- avec la viande.

00:56:39
*Mme. 1:* D'accord.

00:56:40
*Mme. 2:* On mange pas la peau après. Je les épluche pas, mais on mange pas la--

00:56:42
*Mme. 1:* Oui.

00:56:43
*Mme. 2:* On les laisse en chemise, en chemise. J'épluche jamais les tomates. Y en a qui font j'épluche jamais les tomates, les poivrons. J'épluche jamais. Qu'est-ce qu'il y a comme légumes que j'épluche pas ? [euh] Le radis, je mange aussi la fanne de radis quand c'est du jardin.

00:57:02
*Mme. 1:* D'accord.

00:57:03
*Mme. 2:* Pas en supermarché ou-- en supermarché ou en primeur. Parce que je trouve que le-le-la fanne est trop montée. Quand je mange mes radis du jardin, je fais des salades avec les radis, avec les radis entiers. Alors, qu'est-ce que j'épluche pas ? Alors là ? Alors là ? Alors là ? Mystère. Je pense que c'est pas mal, déjà. [rires] Je cherche, mais bon je pense que voilà, c'est-c'est à peu près ça. J'enlève--, les autres j'épluche [hein]. Le poireau j'enlève toujours la première peau--

00:57:32
*Mme. 1:* Ouais.

00:57:32
*Mme. 2:* --autour. Les oignons, [bah] j'achète beaucoup d'oignons nouveaux, là, avec les oignons puisqu'on a fini les vieux oignons. Donc on prend des nouveaux. J'enlève toujours la première petite peau qu'il y a dessus.

00:57:45
*Mme. 1:* Ouais.

00:57:45
*Mme. 2:* Et c'est tout. Autrement, non.

00:57:46
*Mme. 1:* Et quand vous n'épluchez pas un fruit ou un légume. Est-ce que vous- du coup, vous êtes-- vous le coupez directement ?

00:57:58
*Mme. 2:* Je lave toujours.

00:57:59
*Mme. 1:* Vous lavez avant ?

00:58:01
*Mme. 2:* Toujours. Ouais. Je lave toujours mes-- Les fruits un peu moins, je suis-- les fruits, je lave un peu moins.

00:58:10
*Mme. 1:* D'accord.

00:58:11
*Mme. 2:* La pomme, je la lave même pas, je la mange comme ça. Mais les légumes, ouais. Je lave toujours les légumes.

00:58:17
*Mme. 1:* D'accord.

00:58:19
*Mme. 2:* Toujours, toujours. Donc [euh]. Je les passe sous l'eau, j'y mets-- ouais, parce qu'ils ont été touchés, parce qu'ils ont--, mais la pêche aussi [hein]-- enfin la pêche--, le fruit aussi, mais je trouve que ça dénature plus le fruit, de le passer sous l'eau. La pêche sous l'eau. Enfin tout sous l'eau, je trouve que non-- ça me--, je ne laverais jamais mes fraises. Jamais. Je trouve que ça les abîme. Et, par contre, les-les fruits ah oui je lave tout. Les pommes de terre, heureusement, parce que si on les mangeait avec la terre dessus. Le pommes de terre nouvelles, c'est pas terrible. [rires]

00:58:47
*Mme. 1:* Ok, ça marche. Alors, j'aimerais qu'on échange sur le moment où vous jetez un fruit ou un légume, ou une partie seulement. Vous m'avez dit que ça n'arrive pas souvent.

00:59:01
*Mme. 2:* Non.

00:59:01
*Mme. 1:* Mais quand cela arrive, [euh] pourquoi vous décidez de le jeter, plutôt que de le consommer ?

00:59:07
*Mme. 2:* Parce que souvent, c'est pourri, c'est de la pourriture. Moi, j'achète-- l'aspect, je m'en fous. L'aspect, ça m'est égal. C'est pas parce que ça paraît moche-- souvent on va couper le morceau qui paraît moche, parce qu'on voit qu'il est pourri. C'est vrai qu'au goût franchement le pourri c'est pas terrible. On essaie parfois sur la pomme, on sait pas, elle est un peu molle. On mange un peu et on dit : ah non c'est pas bon, c'est pourri. On a-- ce qui nous est arrivé avec une pomme aussi, donc on a dû couper juste le morceau qui était abîmé. C'est vrai.

00:59:34
*Mme. 1:* Ouais.

00:59:34
*Mme. 2:* Juste la pourriture. L'aspect, ça me- ça me fait rien. Pourriture. Un ver. On va dire : [bah] [beurk]. Le ver, il sort du radis comme ça, j'ai pas eu envie de manger. Oui, faut avouer. Sachant qu'il y a des fruits où il y a des vers, comme les cerises, tout ça, on ferme les yeux [hein].

00:59:48
*Mme. 1:* Ouais.

00:59:49
*Mme. 2:* Mais non là, ça m'a rien dit trop. Voilà. Après, c'est ça. C'est surtout-surtout la pourriture.

00:59:56
*Mme. 1:* D'accord.

00:59:58
*Mme. 2:* Un œil, un machin, je m'en fous, ça c'est-c'est pas--

01:00:01
*Mme. 1:* Ouais.

01:00:01
*Mme. 2:* Du moment que c'est mangeable, en fait.

01:00:02
*Mme. 1:* Ok. Et généralement, comment vous déterminez, justement, qu'un fruit ou un légume n'est plus [euh] consommable parce que vous le trouvez trop-- ?

01:00:15
*Mme. 2:* En le coupant. En le coupant. Je vais toujours, je vais pas regarder, en disant : non, c'est pas bon, il sera pas bon, [pouf] je jette. Si je vois qu'il est un petit peu abîmé, je coupe.

01:00:21
*Mme. 1:* D'accord.

01:00:21
*Mme. 2:* Et je regarde la partie qui est abîmée. Je coupe avec le couteau. Je dis : tiens, c'est abîmé là. Parfois, ça va être un tout petit peu plus abîmé en dessous, mais je mange quand même, peu importe. Mais je jette- je jette avec le couteau. J'enlève la partie qui est-- Je vais pas jeter un fruit parce qu'il y a un petit bout-- ou la moitié -- ou là, le concombre, il y avait un tout petit bout qui était pourri au bout, j'ai juste coupé le bout et c'est tout. J'ai regardé. J'ai dit : [bah] oui c'est un petit peu abîmé, c'est tout, quoi. Et j'ai jeté le petit bout. Puis, c'est tout. Donc voilà. Après-- Donc, voilà. En coupant.

01:00:54
*Mme. 1:* Ok. Donc, finalement, il n'y a pas de moment où vous allez voir le fruit ou le légume ou le sentir abîmé, et directement le jeter ?

01:01:05
*Mme. 2:* Non, même une tomate si je la prends et qu'elle est molle, je vais pas la jeter en disant tiens, elle est molle. Si je vois qu'il y a un petit bout de moisi dessus, je vais la couper pour voir jusqu'où ça va. Je peux couper des tranches jusqu'à ce que voilà--. Parfois, je peux aussi, voilà, j'ai une tomate, je la coupe en deux. Je vois il y a une partie qui est pourrie. J'enlève juste la partie pourrie avec le couteau. Je vais pas enlever-- jeter la partie entière. Je veux pas. Juste les petits bouts, quoi, qui sont abîmés. Autrement ça arrive souvent. Suffit qu'il ait un petit choc ou un truc comme ça. Donc, voilà.

01:01:34
*Mme. 1:* Et donc, là, [bah] donc vous aviez-- enfin, vous avez parlé du ver. Imaginons, que vous ne voyez pas du tout qu'un fruit ou un légume est abîmé, un peu pourri, mais que vous vous en apercevez une fois que vous l'avez mis dans votre bouche. Qu'est-ce que-- Comment vous vous sentez ? Qu'est-ce que vous ressentez à ce moment-là ?

01:01:51
*Mme. 2:* [Bah] [euh] je vais le recracher, je vais cracher le morceau que j'ai mangé, mais moi, ça va pas m'empêcher de manger le-- Je vais regarder, quand même si-- voilà-- comme j'aurais croqué dans le fruit, le machin, je dirai : b [bah] iens [bof]. B [Bah] e goût est un peu--, bon ça arrive. Les tomates, ça peut arriver : une tomate un peu aigre, souvent quand c'est-- Bon, je vais pas cracher [hein], sauf si vraiment-- Non c'est pas vraiment un gros dégoût, mais je vais quand même cracher le morceau, puis je vais vérifier [euh] si-si tout le truc, l'ensemble du fruit, ou du légume est pas pourri en entier. Et puis, voilà quoi. Après. C'est tout. Je vais pas jeter non. Il va pas être puni pour autant. Je vais le manger, quand même. [rires]

01:02:32
*Mme. 1:* Ok, super. Alors, [euh] pour finir, je voudrais qu'on discute du gaspillage alimentaire.

01:02:36
*Mme. 2:* Ouais.

01:02:36
*Mme. 1:* Donc là, quand je vous dis gaspillage alimentaire : quels sont les mots ou les expressions qui vous viennent spontanément à l'esprit ?

01:02:45
*Mme. 2:* [Euh] Qu'est-ce qui me vient spontanément à l'esprit ? Ben, ben, ben, pas grand chose. Mais, en fait, [euh] je me dis qu'il y a quand même des gens qui ne mangent pas à leur faim, par exemple, des fruits et légumes. Des gens qui ne mangent pas à leur faim et c'est vraiment dommage de jeter des trucs, alors qu'on peut le maîtriser. On peut maîtriser un minimum ce gaspillage. Il y a des gens qui n'ont pas-- des gens, des jeunes, qui ont pas la possibilité de manger comme nous, [euh] aussi bien que nous, alors jeter des choses ça me-- Mais on a toujours été comme ça. Là, c'est pas en vieillissant. C'est toujours été comme ça, de façon. On a beaucoup aussi, de reconditionné beaucoup de choses parce qu'on trouve que le gaspillage c'est-- on peut pas gaspiller. Un plat, on finit un plat, il reste ça de pâtes on jettera jamais. Donc, il y a toujours un moyen de reconditionner les gens. Et il y a des gens qui font pas d'effort. Qui se disent : bon allez, [hop], c'est pas grave. Ça, moi, ça me met un petit peu hors de moi. Je trouve que, voilà. Le gaspillage, c'est ça. Puis bon si après on peut être un peu plus disciplinés, sur ces choses-là.

01:03:51
*Mme. 1:* Ouais.

01:03:51
*Mme. 2:* Déjà la poubelle on devrait moins la remplir. Si on gaspille moins. Il y aura moins de-de-- moins de déchets, mais aussi moins de déchets alimentaires dans-- C'est bon pour tout le monde, pour la planète, pour les enfants, tout le monde, c'est très bon- c'est mieux. Et je trouve que-- ouais, je trouve que le gaspillage c'est vraiment-- les gens-les gens ne sont pas assez attentifs, je crois. Ils n'ont pas assez d'idées de ce que tu-- on peut faire avec un fruit et un légume. Ils se posent moins de question que-que de se dire : tiens, on va gaspiller, on va jeter. C'est facile de jeter. C'est la facilité de jeter. C'est trop facile de dire : tiens, bof, allez, un petit peu trop, on va couper un bout, on va jeter tout. Et puis- et puis, financièrement, ça a un coût.

01:04:41
*Mme. 1:* Oui.

01:04:41
*Mme. 2:* Je pense que ça a un coût financier aussi. Dont on n'a pas-- si on peut faire des économies, qu'elles soient là ou ailleurs. C'est-c'est pas mal aussi. Moi, je trouve que voilà. Nous on est-on est pour tout, on jette rien. On jette rien. On aime bien les produits primaires comme ça. On aime bien-- Moi, j'aime bien cuisiner. J'achète très peu de conserves.

01:05:01
*Mme. 1:* D'accord.

01:05:01
*Mme. 2:* Sauf les tomates.

01:05:03
*Mme. 1:* D'accord.

01:05:03
*Mme. 2:* J'achète des tomates pelées tout-- pour tout l'hiver, parce que y a pas de tomates l'hiver. On va pas cuisiner avec des tomates de chez-- des-- des coulis de tomates, des choses comme ça, parce que ça je fais pas. Ça-- je fais en période, je fais beaucoup de ketchup, des choses comme ça, en période de tomates, mais quand c'est pas la période. Et je trouve qu'aujourd'hui, il y a quand même des possibilités de faire--, même si on ne veut pas acheter chez le primeur, il y a toujours des fruits et des légumes surgelés qu'on peut acheter. Ça, je trouve qu'il y a moins de gaspillage. Et je trouve que c'est pas-- c'est une bonne alternative, en fait, au gaspillage. C'est d'avoir-- de prendre la quantité, en fait, qu'on veut dans le congélateur. Je trouve que c'est pas mal, ça. Voilà.

01:05:39
*Mme. 1:* Tout à fait. D'accord. [Euh] Et du coup, si on-- parce que ça pourrait être intéressant de créer des-des définitions du gaspillage alimentaire. Et vous, avec vos propres mots, quelle serait votre définition à ce-- du gaspillage alimentaire ? Si on devait écrire un Petit Robert : le gaspillage alimentaire, qu'est-ce que vous y mettriez ?

01:06:00
*Mme. 2:* [Bah] moi je mets-- je mettrais [euh] le gaspillage alimentaire [ohlala] c'est pas facile de trouver une définition.

01:06:12
*Mme. 1:* Avec vos mots.

01:06:12
*Mme. 2:* C'est le-- c'est la non-réflexion. La non-réflexion de-de-- fin d'aliments quoi. Je sais pas comment expliquer ça. De-des personnes en gros, à l'utilisa-- la non-réflexion des personnes à jeter-- à savoir que faire de leurs aliments-- de leurs produits abîmés. De leurs produits ou de-de-de leur-- oui, de leurs produits abîmés, abîmés. Ou il y en a qui sont même pas abîmés, mais il y en a qui jettent parce qu'ils en ont plus envie. Moi, ça me... encore pire. De produits abîmés. Voilà, je pense que les gens, ils ont pas ou ils respectent pas ça ou ils n'ont pas idée-- on ne leur explique pas assez peut-être. On a parlé des-de-- comment j'allais dire, de tout ce qui était fruits et légumes, c'est bon pour la santé, machin. Il faut peut-être aussi aller plus loin en disant : oui, ne gaspillez pas. Il y a eu les fruits moches, il paraît, au moment des fruits moches et machin, et je pense que c'est un peu où il y a un peu plus de public vers-vers savoir ne pas gaspiller. Comment ne pas gaspiller ? Pourquoi et pourquoi ne pas gaspiller ? Et je pense que le gaspillage, c'est ça, c'est que il y a une ignorance quelque part. Une ignorance de la-- Puis voilà, puis on produit des choses, si c'est pour les jeter après, c'est-c'est pas terrible. Voilà.

01:07:43
*Mme. 1:* [Euh] D'accord. Et quand vous réalisez que vous jetez, vous, un fruit ou un légume à la poubelle, qu'est-ce que vous ressentez ?

01:07:51
*Mme. 2:* [Bah] ça m'énerve à chaque fois, ça m'agace. Je me dis : si j'avais-- oh j'aurais dû regarder hier. Voilà, aujourd'hui, un petit peu de pourriture, peut-être qu'hier y a vait pas.

01:07:59
*Mme. 1:* Oui.

01:08:00
*Mme. 2:* Alors je suis-- ça-ça m'agace. Ça m'agace. Je n'aime pas jeter. Je n'aime pas jeter quoi que ce soit d'ailleurs, il y a des choses qu'on jette, je sais pas. Il y a un bouquin, on jette à la poubelle. Alors qu'il pourrait être reconditionné, mais il n'y a pas de reconditionnement prévu pour. Je trouve que ça me-- ça m'agace, ça m'agace. Ça me-- On a toujours été comme ça. On a toujours-- On aime--, on préférerait que ce soit recyclé. Mais bon, après, le gaspillage des fruits et légumes, ça va pas être recyclé. On peut mettre là, les petits morceaux qu'il y a, là tout ça, j'aurais pu mettre dans un seau, aller donner aux chevaux. Parce que ceux-là, ils ont pas droit, mais ceux-ci, ils ont droit. Donc voilà, je trouve que au moins d'avoir un intérêt à acheter quelque chose parce que là, c'est la fin du circuit. Quelque part, la poubelle, c'est la fin du circuit. Je fais pas de compost parce que je peux pas faire du compost à cause des blessures qu'on a partout, mais je que c'est une chose. Ça m'agace, voilà.

01:09:01
*Mme. 1:* Je comprends, je comprends.

01:09:03
*Mme. 2:* Voilà.

01:09:03
*Mme. 1:* Alors juste pour terminer, [euh] on pourrait discuter un peu de l'utilisation de la poubelle connectée. Vous vous l'avez fait.

01:09:08
*Mme. 2:* Oui.

01:09:09
*Mme. 1:* Vous l'avez un peu évoqué. Alors du coup voilà, ma question c'est justement : est-ce que vous avez l'impression de l'utiliser fréquemment ou très peu ? Du coup, je pense savoir votre réponse.

01:09:17
*Mme. 2:* On l'a pas utilisé pendant plus d'une semaine et mon mari dit : t'as mis quelque chose dedans ? Je dis : non. Et tout le monde est dans l'air. On sait qu'il faut mettre là, et tout, machin. Que ce soit mes-mes enfants, mes petits-enfants quand ils arrivent, je leur dis : tu laisses, si tu manges pas, tu laisses là. Mais non, on n'a pas eu-- en fait-- y a voilà, on a eu une banane qui était un petit peu mûre. J'ai dit à mon mari : Ah, y aura peut-être quelque chose à mettre dedans. Il me dit : Tu rigoles, ou quoi. Il vient là et il l'a mangé.

01:09:40
*Mme. 1:* Ouais.

01:09:42
*Mme. 2:* Donc finalement il l'a mangé. Donc, voilà. Je sais bien que voilà, c'est-c'est-- On a très peu. Et la salade encore. C'était même pas des feuilles entières. C'était des petits bouts qui avaient collé, un petit peu noirs. Un petit bout de concombre, un tout petit bout. Un petit peu de pommes. Mais vraiment, il y a vraiment- il y a rien dedans. On s'est dit-- mon mari m'a dit : ils vont se dire que t'as rien mis dedans, que tu t'es trompée [rires] ou elle marche pas. J'ai dit : si, si on a commencé à mettre des choses dedans. Donc ça marche.

01:10:10
*Mme. 1:* Je demande aux personnes, si jamais ils l'ont oubliée ou pas. Non, vous ne l'avez pas oubliée, mais-- D'accord.

01:10:15
*Mme. 2:* Et elle marche.

01:10:17
*Mme. 1:* Et donc il y avait quelques petites choses dedans. Vous m'avez dit quelques bouts de salade, le radis, tout ça, mais pas de choses entières. Uniquement des morceaux prélevés.

01:10:26
*Mme. 2:* Une patate ?

01:10:27
*Mme. 1:* Ah oui, et la patate. Et plus, une patate qui était ramollie.

01:10:28
*Mme. 2:* Ouais, qui était ramollie. qui était vraiment, la pauvre. On pouvait même pas l'éplucher. Parce que on fait des essais. On pouvait même plus l'éplucher. Voilà, c'est vrai que franchement, non, non, voilà donc très peu de choses.

01:10:41
*Mme. 1:* D'accord.

01:10:42
*Mme. 2:* Très peu.

01:10:43
*Mme. 1:* Et est-ce que- est-ce que vous avez des fois, une idée de-des fruits et légumes que vous jetez, quand même, peut être plus que les autres ? Est-ce que, selon vous, il y a des, voilà des fruits et légumes dans votre foyer qui auraient tendance à se perdre davantage ?

01:11:01
*Mme. 2:* Oui, je trouve que le--, [bah] le concombre j'en mange beaucoup, mais je trouve que c'est fragile.

01:11:05
*Mme. 1:* D'accord.

01:11:05
*Mme. 2:* Le concombre est hyper fragile. Et souvent, je pense que c'est une histoire de conditionnement. C'est souvent les bouts, en fait, qui sont abîmés. Le concombre est très fragile. On a... la carottes. Les carottes nouvelles : quand on les achète, on a intérêt à les cuire très vite, parce qu'elles sont--. Là, j'ai eu un souci avec une botte où elles étaient un peu juste. Je les ai congelées, j'ai coupé en deux, j'ai congelé. Mais voilà quoi. Je trouve que les carottes nouvelles. C'est essentiellement lié-- les-- tout ce qui est-- pour tout ce qui est légumes nouveaux. En fait.

01:11:37
*Mme. 1:* Ouais.

01:11:38
*Mme. 2:* Les petits pois, c'est pareil. Faut faire attention au conditionnement si on les prend. Il suffit qu'il y ait un petit-- Les haricots blancs, c'est pareil. Il suffit qu'il y ait un de pourri et voilà. Qui est ramassé sous la pluie, et puis qui est pas bien séché. Voilà, ça donne-- ça a des conséquences après. Les oignons : s'ils sont pas bien séchés. On les aime, mais souvent-- il y a des gens qui-- moi j'ai moins ce problème-là. Y a des oignons qui pourrissent, en fait, facilement. Je pense que essentiellement, mais de moi, des légumes que j'achète, c'est surtout ça. Et par exemple, les oranges aussi, les oranges. Mais je pense que voilà le transport. Elles sont un peu bousculées. Voilà. Ça, c'est sûr. Mais si j'ai une orange qui est un petit peu abîmée, je vais couper juste le petit bout de l'orange, et je vais faire un jus de fruit avec quand même. Je vais pas la jeter, quoi. Voilà.

01:12:25
*Mme. 1:* Ok. Donc, pour vous, la raison principale qui fait que vous êtes parfois amenée à jeter un fruit, un légume, c'est plutôt une histoire de conditionnement.

01:12:33
*Mme. 2:* Oui, pas d'entreposé chez nous. Ouais, chez nous, non. Quand ils arrivent, ils sont aux petits soins dans leurs petites caisses. Donc, ils sont pas manipulés, on soulève, on s'achète à tout va par leur machine à tuer. Je regarde, je tiens, je prends celle ci. Je vais quand même toucher, mais pas propres valeurs avec à la clé, un petit peu plus mûr pour entrer, plutôt que de la laisser. Donc voilà. Mais je pense que le conditionnement, il y est pour beaucoup. Le transport, je crois. Je pense.

01:13:07
*Mme. 1:* D'accord. Est-ce qu'il vous est arrivé de vous poser une question- une ou plusieurs- avant ou après avoir jeté dans la poubelle ? À savoir : est-ce que- est-ce que ça ça devait aller dedans ou pas ? Ou est-ce que non, vous n'avez pas eu de-- ?

01:13:23
*Mme. 2:* Non, puisque ça c'était bien tout ce qu'on mange habituellement. Ouais, on a pas mangé. Mon mari m'a demandé-- je sais plus ce que c'était. Il m'a dit : [bah] ça tu mets ? Je lui ai dit : non, non tu mets pas, puisque c'est ce qu'on, ce qu'on n'a pas l'habitude de manger, je sais plus ce que c'était.

01:13:41
*Mme. 1:* C'était de l'ordre de l'épluchure ?

01:13:42
*Mme. 2:* Ouais, je pense. C'était, c'était, c'était [bah] c'était peut-être les pommes de terre parce que les nouvelles, on les épluche pas. Les vieilles, on les épluche pas au début, après on les épluche parce que la peau elle durcit un peu. Et c'était ça, il m'a dit : tu mets les épluchures de pommes de terre ? J'ai dit : non, puisqu'à cette période là, on les mange pas. On va pas en perdre l'intérêt. Non, non, on a été-- Enfin, c'est moi qui faisait aussi. Je leur disais : vous me laissez de coté. Parce que je peux pas éplucher, donc ils épluchaient tout. Et j'ai dit : tu laisses et moi je ferai le tri après de ce qu'il y a-- S'il y a un petit bout de patates qui est abîmé, tu creuses, et puis voilà, C'est tout.

01:14:15
*Mme. 1:* Ok. Chouette. Donc vous ne l'avez pas oubliée. Et le sac, du coup, vous ne l'avez pas changé ?

01:14:20
*Mme. 2:* Non, non, ça j'ai pas besoin. Le pauvre. Puis, il y a pas d'odeurs. Donc [euh].

01:14:26
*Mme. 1:* Ouais. Ok. J'en ai d'autres, si besoin. Et [ben] voilà, j'ai posé l'ensemble de mes questions. Merci beaucoup pour cet échange riche. Est-ce que j'ai des choses sur lesquelles vous aimeriez revenir, des choses que j'aurais pu oublier ? Ou vous pensez qu'on a fait un bon petit tour ?

01:14:46
*Mme. 2:* Non, je pense qu'on a fait un bon tour. Non, non, je pense que ça va. Voilà.

01:14:56
*Mme. 1:* Super. [Bah] merci.

**Household F04, Interview 2**

*Mme. 1:* Pour commencer, quatre illustrations.

*Mme. 2:* D'accord.

*Mme. 1:* Je vais vous demander d'en choisir une, de la retourner et de me dire spontanément ce qui vous vient à l'esprit.

*Mme. 2:* [Ah] Ce qui me vient à l'esprit-là, c'est le primeur local. [Rire]

*Mme. 1:* Le primeur local ?

*Mme. 2:* Le primeur local, [voilà], produit locaux, [voilà].

*Mme. 1:* D'accord.

*Mme. 2:* C'est un peu ça. Un petit primeur avec des produits locaux.

*Mme. 1:* Pourquoi vous avez pensé ça ?

*Mme. 2:* [Inaudible 00:00:29-00:00:31]. Peu de produits, pas d'excès de produits.

*Mme. 1:* Ouais.

*Mme. 2:* Ça aussi, et puis ouais la présentation, je trouve que ça fait-- C'est un petit peu comme chez mon primeur. Voilà c'est pour ça. [Rire]

*Mme. 2:* Ouais, peu de produits, un peu de tout quand même, pas forcément bio. Il n'y a pas d'appellation, mais pour moi, de petits produits ouais. Des produits locaux, [euh], je dirais pas non plus puisque il y a autant d'aubergines que de carottes, donc, ouais. Non, non, [voilà]. Chez un petit primeur, donc un petit primeur.

*Mme. 1:* D'accord. Et [du coup], est-ce que vous avez déjà des expériences avec ce type de- de lieux ?

*Mme. 2:* Ouais, souvent dans le sud dans-- de la France-- de la France, en Ardèche, il y a beaucoup de petits-- il y a beaucoup de petits primeurs comme ça. Et ici- ici, moi, je fais mes courses que chez le primeur.

*Mme. 1:* D'accord.

*Mme. 2:* J'allais sur les marchés, je ne vais plus sur les marchés.

*Mme. 1:* D'accord.

*Mme. 2:* Puis [du coup], j'ai dit, je n'y vais plus.

*Mme. 1:* Ok.

*Mme. 2:* Donc, je vais plus chez le primeur, [voilà]. Mais je n'achète pas non plus mes fruits et légumes chez-- au supermarché, j'achète essentiellement chez le primeur. On a déjà essayé deux primeurs. On en a deux à Landerneau, on a le choix.

*Mme. 1:* D'accord.

*Mme. 2:* Et après ici, [bah], voilà. Je suis-- Je suis revenu chez un un primeur que j'avais eu il y a des années.

*Mme. 1:* Ouais.

*Mme. 2:* Et le monsieur était décédé. Ses enfants avaient repris l'affaire. C'était catastrophique.

*Mme. 1:* D'accord.

*Mme. 2:* Les enfants étaient très jeunes, très très jeunes et ils avaient des produits qui étaient minables.

*Mme. 1:* Ha !

*Mme. 2:* Donc ils ont-- Là, j'étais reparti, j'étais parti sur les marchés, j'avais essayé un autre primeur sans être-- Bon, les marchés j'suis satisfaite. Mais là bon, [voilà], je vais plus. Et je me suis dit allez je-- Pourquoi pas ? Je-- Ils ont une bonne réputation maintenant donc je vais retourner voir.

*Mme. 1:* D'accord.

*Mme. 2:* Et en effet. Alors, moi je suis pas déçu-là. Depuis quelques mois, j'y vais-- enfin, depuis quelques mois. [Bah], depuis le Covid [hein]. Depuis le covid-- depuis le début du confinement.

*Mme. 1:* Ouai.

*Mme. 2:* Donc ça fait un moment que j'y vais, et je suis pas déçu.

*Mme. 1:* OK

*Mme. 1:* Ouai, qu'est ce qui fait que vous avez privilégié donc [euh] depuis le covid ce n'est plus le marché. Mais qu'est-ce qui fait que vous-- [Du coup], vous choisissez plus tôt le primeur que-qu'un autre magasin ?

*Mme. 2:* En supermarché, j'ai eu à essayer pendant [bah], le confinement, j'étais déçu des fruits et légumes.

*Mme. 1:* D'accord.

*Mme. 2:* Et je trouve que oui, la- le fait, en fait que les produits soient touchés et excessivement.

*Mme. 1:* Ouai.

*Mme. 1:* Comme il y a beaucoup de produits, ils sont manipulés, ils sont mis en frigo, etc. etc. Que chez le primeur, il y a peut-être eu qu'une étape en moins. [On va dire].

*Mme. 1:* Ouai

*Mme. 2:* Celle de grossiste [euh] donc, [voilà]. Donc il n'est peut-être pas le grossiste mais il y a peut-être une étape en moins. Puisqu'ils font-- ils font-- Ils marchent beaucoup, avec des producteurs aussi, [euh], Pas forcément locaux mais [inaudible 00:02:58-00:03:58]

*Mme. 1:* [Rire].

*Mme. 2:* Assis, assis, j'ai dit. Assis.

*Mme. 1:* Et donc globalement-là, le-- chez le primeur vous êtes satisfaite des produits que vous-y trouvez ?

*Mme. 2:* Complètement.

*Mme. 1:* OK

*Mme. 2:* Complètement.

*Mme. 1:* Et en termes de- de plaisir d'aller [euh] au magasin.

*Mme. 2:* J'y vais beaucoup, ouai. Ça me plaît beaucoup parce que c'est-c'est bien achalandé.

*Mme. 1:* D'accord.

*Mme. 2:* Et, [euh] donc, [du coup], c'est des-- c'est donc deux files et deux gars qui tiennent ça et c'est jeune. Donc ça a été achalandé aussi de manière un peu jeune, avec des produits, des nouveaux produits, du vrac aussi.

*Mme. 1:* D'accord.

*Mme. 2:* De l'épicerie qu'ils ont commencé à faire, du fromage, des fromages.

*Mme. 1:* D'accord.

*Mme. 2:* Ils sont un petit peu étoffés sur les produits. Et ça, c'est plus des produits locaux. Mais [voilà], et je trouve, ouai, très agréable l'accueil,

*Mme. 1:* Oui

*Mme. 2:* [Enfin voilà], le magasin lui-même. Il me plait beaucoup.

*Mme. 1:* D'accord. Donc c'est--

*Mme. 2:* Pas de foule, pas de foule aussi non plus dans le magasin, parce que moi je n'y vais pas forcément aux mêmes heures que les gens-- je vais en journée ou il n'y a personne, donc c'est très très bien. Et le fait aussi de pouvoir commander des produits un peu typiques, par exemple. [Bah] là, j'ai fait--je fais fini, je fais mes cornichons.

*Mme. 1:* Ouai.

*Mme. 2:* Ça faisait déjà quelques années. Je faisais plus-- enfin quelques-- non, quoi-quoi trois ans que je ne faisais plus de cornichons. Là, j'ai recommandé, j'ai eu des cornichons. On a eu-- on a commandé fraise à confiture, on a eu des fraises à confiture. [Voilà] les commandes spécifiques ou il m'appelle carrément. Ou il marche sur-sur Messenger ou Facebook donc on fait--

*Mme. 1:* D'accord.

*Mme. 2:* Ils ont- ils ont- on met un petit message et puis c'est parti quoi donc. Je trouve que c'est très très bien cette méthode- cette méthode me convient très bien. Et puis ce n'est pas loin.

*Mme. 1:* Ouai.

*Mme. 1:* C'est à côté du supermarché, donc [voilà]. Et puis, vous savez comment, [bah] on peut faire plein de choses. On peut commander des paniers. [euh]Souvent, même moi ce que je fais, j'aime bien quand je vais chez quelqu'un, acheter un panier de fruits par exemple.

*Mme. 1:* Oui

*Mme. 2:* Et [Voilà] donc ils le feront à la demande et sûrement. Je suis toujours contente. Vous avez des paniers gagnés- des paniers garnis de fruits ou de légumes, [enfin] pourquoi pas ?

*Mme. 1:* D'accord. Et [euh] [du coup], vous y allez-- vous y allez régulièrement, c'est [euh]--

*Mme. 1:* Une fois par semaine à peu près.

*Mme. 1:* Ouai. [OK], Donc ce n'est pas forcément une corvée pour vous de vous y rendre ?

*Mme. 2:* Pas du tout. Au contraire, j'y vais que quand je rentre, ouai, quand je suis libre. J'ai du mal dans les magasins. Mais bon ça, c'était pendant le Covid. J'ai du mal un petit peu à aller au magasin où on drive formidable. Dans les grands magasins, mais alors là, là, j'ai beaucoup-- j'aime beaucoup parce que c'est bien achalandé. Puis on vous laisse tranquille, on est pas dessus, on n'est pas sur vous en train de vous demander, [voilà]. Et puis même les produits spécifiques. Je suis une fan aussi, J'aime-- bon j'aime bien parler pêches, moi j'aime beaucoup les agrumes à cette époque-ci.

*Mme. 1:* Oui.

*Mme. 2:* Je suis une fan d'agrumes et généreuse agrumes, un peu différente des autres. Donc je fais, je goûte. Et puis; bah elle me-elle me fait goûter certains produits aussi. Ça permet d'acheter des produits qu'on ne connaît pas forcément [euh]. Surtout l'agrume parce que moi, c'est-- je sais plein de choses sur l'agrume.

*Mme. 1:* Ouais. [rire]

*Mme. 2:* ouais, ouais. Là-là, c'est la saison.

*Mme. 1:* [OK], vous fait découvrir, on vous conseille aussi des fois sur--

*Mme. 2:* [Voilà], on conseille, on peut con-- sur tout. On peut être conseillé sur tout [euh].

*Mme. 1:* D'accord. Et est-ce que ça vous arrive de-de jeter des produits que vous avez achetés [euh] chez le primeur-chez le primeur ?

*Mme. 2:* Très peu, très peu. Parce que, comme j'achète fréquemment, je jette très très, très peu.

*Mme. 1:* D'accord. Et quand ça vous arrive, qu'est-ce que ça vous fait quand vous êtes amené à jeter ?

*Mme. 2:* [Eh bah[, je peux jeter [hen]. Je ne jette pas si je peux pas-- si je peux éviter de jeter [euh]. J'ai-- ils ont même un produit la-bas, enfin, pas un produit un rayon DLC en fait et j'achète des produits voilà de--

*Mme. 1:* Oui.

*Mme. 2:* Là j'ai acheté [euh]-- j'ai acheté il n'y a pas longtemps des asperges qui étaient en-en fin de vie, mais qui étaient bonnes et voilà après [euh], c'était [euh] puisqu'elles avaient plus de chair à bois, et la couleur un petit peu moins blanche que les autres. Enfin bon, je trouve que ce n'est pas mien-- c'est pas mal. Des-des-- [voilà] d'acheter des produits qu'on mange en famille.

*Mme. 1:* [Mm-hmm]

*Mme. 2:* Même des petites pommes abîmées, ou des- de- des fruits bizarres, et même des pommes-des produits. Après, je n'achète pas beaucoup de légumes, peu de légumes, maintenant beaucoup de fruits.

*Mme. 1:* D'accord.

*Mme. 2:* Peu de légumes mis à part les choux en fait. Tout ce qui est choux. Puisque les carottes je les ai du producteur, les oignons, les échalotes et les pommes de terre, j'ai du producteur. Donc les légumes de base. Je les ai [euh] pour tout l'hiver.

*Mme. 1:* D'accord.

*Mme. 2:* Même si j'arrive au bout, là maintenant, [voilà] donc j'achète peu, enfin beaucoup de choux, tout ce qui est choux, tout ce qui est choux en fait [euh].

*Mme. 1:* OK.

*Mme. 2:* Choux salade, comme ça, je mange les choux comme la salade. Voilà, on est dedans les endives. Mais voyez c'est plus [euh]-- Donc, [voilà].

*Mme. 1:* OK [euh] ça marche. Alors une autre photo, celle que vous voulez.

*Mme. 2:* Au choix. [Rire]

*Mme. 1:* [Rire]

*Mme. 2:* [Ah beh bah] là c'est les marchés [hen].

*Mme. 1:* Les marchés libres ?

*Mme. 2:* Ouai c'est les marchés.

*Mme. 1:* Qu'est-ce qui vous vient [euh] à l'esprit ?

*Mme. 2:* Le sud [rire]

*Mme. 1:* Le sud ?

*Mme. 2:* Ouai, les grands marchés dans le sud de la France. Plein légumes, mais beaucoup, Les choux, c'est pas trop vu là-bas oui, c'est pas trop l'époque. C'est plus les tomates ou des choses comme ça, oui, plus [euh], moi, ça m'évoque plus [euh] oui, là comme ça maintenant, puisque j'y vais plus du tout en d'autres périodes.

*Mme. 1:* Oui

*Mme. 2:* Pour des vacances, le plaisir de traîner, de manger des fruits dans le sud de la France et des légumes [euh]. Quand on arrive dans le Sud, dans l'Ardèche toujours, j'achète de quoi faire une ratatouille quand on arrive.

*Mme. 1:* D'accord.

*Mme. 2:* Dans les deux jours, je fais une grande ratatouille. Tout le temps, tout le temps, donc quand on va au marché, excessif-- exclusivement ça revient en fruits et légumes.

*Mme. 1:* [OK]

*Mme. 2:* [Voilà].

*Mme. 1:* Pour vous, ça a un air de vacances [euh] ?

*Mme. 2:* Ouais, ouais, ouais. Oui, un air de vacances ou plus. Avec mon mari, on court les marchés, on est capable de faire un petit-- tous les jours de la semaine, un marché différent tous les jours.

*Mme. 1:* OK

*Mme. 2:* Ah oui, on va en vacances [euh]. [Alors] on part en vacances au mois de-- si tout va bien au mois de mai et là c'est pareil, là tout de suite la première chose qu'on va regarder, c'est ou il y'a les marchés.

*Mme. 1:* Oui.

*Mme. 2:* Et on va faire les marchés.

*Mme. 1:* Donc c'est une activité régulière pour vous pour le moment ?

*Mme. 2:* Ouai, c'est ça. Oui, oui voilà ça fait un plaisir, une activité Je suis une sortie comme [euh]-- comme si [euh], voilà, il y'en a qui vont visiter des choses. Nous, on va visiter les marchés. [Rire]

*Mme. 1:* Vous vivez bien.

*Mme. 2:* Ha ouai.

*Mme. 1:* Et qu'est-qui-- est-ce que vous saurez dire qu'est-ce qui fait que vous prenez du plaisir à déambuler dans le marché ?

*Mme. 2:* J'aime bien les- la diversité des produits.

*Mme. 1:* D'accord.

*Mme. 2:* On peut avoir de toutes sortes de produits différents à des prix différents. Même le prix, je ne suis pas forcément à regarder les prix.

*Mme. 1:* Ouai

*Mme. 2:* Avant le prix. Je regarde surtout les-- la qualité des produits. En fait [euh], les tomates de pleine terre, des choses comme ça quand on voit ça dans le Sud [ha] c'est l'enfer [hen] ! [Voilà]. Chercher des produits [euh] qui n'est toujours pas forcément bio. Apparemment, je ne suis pas très bio, mais les produits producteurs locaux, quoi, vous faites, sauf certains produits, on ne peut pas [euh]-- on ne peut pas avoir puisque il n'y a pas ça des poignées qu'à même. Par exemple, le melon jaune. On a beaucoup de mal dans le sud à trouver des melons jaunes.

*Mme. 1:* D'accord.

*Mme. 2:* Français. Quand je cherche des melons français, on n'en trouve pas.

*Mme. 1:* Oui.

*Mme. 2:* Donc, [voilà] des choses comme ça. Par exemple, on aime bien chercher des produits un peu différents, qu'on mange ici aussi, quoi! Voilà bah c'est les pêches-les pêches, les-- on en mange ici, c'est des bonnes. [Rire]

*Mme. 1:* Ouai c'est des bonnes.

*Mme. 2:* Ouais, c'est ça. Oui, elles sont très bonnes.

*Mme. 1:* Oui.

*Mme. 2:* On en mange toujours puisqu'elles sont congelées, donc on en mange toujours. Donc, voilà-voilà donc le plaisir. Et puis le plaisir de traîner ne. On va au marché le mercredi, on va-- on va au marché presque plus de deux heures.

*Mme. 1:* Vous allez toujours dans le marché [euh]--

*Mme. 2:* Il y'en a de plus grands de la région, dans le village où on va en vacances.

*Mme. 1:* D'accord.

*Mme. 2:* En Ardèche, non, c'est-- [bah], on y va tout le temps, mais on-- c'est à Sentilli mercredi et c'est vraiment la sortie. Et pour les gens là-bas, c'est leur sortie.

*Mme. 2:* Ouai.

*Mme. 2:* C'est un très très bon marché, de deux ou trois heures. On est capable d'aller [euh]. On adore, on adore ça. On aime bien visiter, traîner, puis on a l'impression qu'il y une ambiance. Même si c'est la dernière. C'est un petit peu compliqué, mais une ambiance plus--ouai mais côté détente [euh], [Voilà].

*Mme. 1:* OK et est-ce que vous aviez un peu la même-- enfin, vous disiez que vous all- vous alliez au marché [euh] avant le Covid ici. Est-ce que c'est un peu la même idée [euh] ?

*Mme. 2:* Ouais, c'était un peu la même idée. C'était le dimanche et puis bon mon mari [euh], on-on va le dimanche ensemble ou on traine au marché. Mais on avait notre primeur, on aimait beaucoup le primeur, donc on allait toujours, au même primeur toujours [euh].

*Mme. 1:* [Mm-hmm]

*Mme. 2:* C'était, voilà c'était une sensation moi de : un peu de vacances, un peu de trainer traîner, d'acheter des-nos produits locaux. En fait, on aimait bien ça aussi. Et puis moi, avec le Covid, puis on a, on fait plus parce que moi je ne pouvais plus, moi. Ce que j'entends c'est qu'il y'en a qui laissent couler certains qui ne respectent rien, qui-- je ne peux plus. C'est rédhibitoire. Ça reviendra peut-être, mais pour l'instant c'est- c'est une page qui est tournée.

*Mme. 1:* D'accord.

*Mme. 2:* Mais pas en Vacances.

*Mme. 1:* [Mm]

*Mme. 2:* Je pense qu'en vacances, ce sera toujours avec le masque. Dans le Sud, c'était avec le masque et tout. Mais il y a-- mais pas ici ce n'est pas ici. Ici je ne le sen pas.

*Mme. 1:* Ouai. Donc il y a-- quand vous allez au marché, c'est et pour les produits, la diversité [euh] et en même temps le côté [euh] sympa. Je--

*Mme. 2:* Ouais sympa. Oui [voilà] de-des commerçants aussi qu'on connaissait, [voilà], qu'on voit habituellement comme notre primeur au moins. Des commerçants qu'on aime bien et puis qu'on apprécie de retrouver et de discuter avec eux.

*Mme. 1:* Ouai. [Rire] Ouai je comprends. Et [euh]- et vous pensiez quoi des produits en termes de [euh]- de qualité ? Est-ce que--

*Mme. 2:* La qualité est très très bonne, [ah] oui.

*Mme. 1:* [Mm]

*Mme. 2:* Il y'a une bonne qualité de produits. [Ahh] C'est un primeur, puis il m'a toujours conseillé-bien conseillé. [euh] Quand on était [euh] là-bas il nous a toujours bien conseillés, les bons produits. On n'a jamais eu à se plaindre de la nature des produits [euh].

*Mme. 1:* Ouais.

*Mme. 2:* Comme en règle géné-- j'ai le même-la même chose chez mon primeur ici quoi.

*Mme. 1:* D'accord.

*Mme. 2:* [voilà] donc [euh], sauf que c'est plus au marché [quoi] !

*Mme. 1:* Ouais. Mais vous avez quand même du plaisir à aller au primeur ?

*Mme. 2:* Ouais, ouais, j'aime bien.

*Mme. 1:* OK. Et est-ce que ça vous--vous avez le souvenir que ça vous est arrivé de perdre des produits que vous aviez achetés au marché ou même quand vous êtes dans le Sud des fois, ça vous arrive de perdre des produits que--

*Mme. 2:* Alors la première année, oui, mais pas après.

*Mme. 2:* D'accord.

*Mme. 2:* Donc ça fait quand même neuf ans qu'on y va. La première année, on a perdu des produits parce qu'on a acheté des cagettes de pêches, des cagettes de melons, mais on s'est vite rendu compte qu'on est incapable de manger ça puisque ça se conserve pas bien avec la chaleur. Donc on fait plus de marchés, on achète de pêche, de machin, enfin les fruits du jour en fait, [voilà]. Plus les fruits du jour. Des tomates pareilles, on les conserve deux jours, trois jours, et pas plus. On sait que la première année, on savait qu'on prenait beaucoup trop de choses. Ça donnait envie.

*Mme. 1:* [Rire]

*Mme. 2:* Des cageots, de belles-- je ne sais plus si c'est les choses d'ici. Les tomates du pays, des belles tomates qui ont un goût de tomate.

*Mme. 1:* Ouai.

*Mme. 2:* Mais un Cageot, ça ne va pas résoudre quelque chose. Quand on est-- on se retrouvait à trois à manger un cageot de tomates en trois jours. Et les melons, c'est pareil. On a-- nous, on a arrêté.

*Mme. 1:* D'accord.

*Mme. 2:* C'est la première année, la première année. Puis on a vite compris que non, non, c'était pas-- parce que là-bas on mange énormément de fruits et de légumes.

*Mme. 1:* Ouai.

*Mme. 2:* Beaucoup, beaucoup.

*Mme. 2:* Une bonne chose aussi, amener des fruits et légumes beaucoup là-bas.

*Mme. 1:* OK. Alors si on passe à une autre photo.

*Mme. 2:* [Mm-hmm] Alors l'Institut BOUTCHU je vois ça plus tôt commercial. En fait, plus tôt grande-grande surface qui se vend une grande surface.

*Mme. 1:* Ouai, tout ça, vous-- à quoi ça vous fait penser ?

*Mme. 2:* Grand rayon. Aux grands-grands rayons, bien rangés, avec beaucoup, beaucoup, beaucoup de produits.

*Mme. 1:* Ouai.

*Mme. 2:* Qu'on peut manipuler, et re-remanipuler, faire, tomber, remettre au port.

*Mme. 1:* Ouai.

*Mme. 2:* Ouai, trop grand. Pour moi ça, c'est-- ça, c'est pour moi-- ça, c'est--ça fait bien longtemps déjà que c'est rédhibitoire, mais voilà, ça me-- ça me convient pas. Je trouve que la produit-les produits, moi en cela, je vois des produits qui ont été en frigo longtemps et qui [bah[ quand on les prend, [bah] ils ne sont plus [euh]-- puis en frigo, donc ils n'apprécient pas, ils sont blettes au bout de deux jours. On achète un filet d'oranges, ce qui m'est déjà arrivé quand même. [euh] On va dire occasionnellement de me dire : tiens je ne vais pas aller chez le primeur, j'ai un truc à prendre, une carotte [hen], une bouse d'ail. Mais à chaque fois, je suis déçu quoi.

*Mme. 1:* Oui.

*Mme. 2:* Très déçu des produits. Même une gousse d'ail, vous pouvez en acheter et ne pas avoir d'ails. Il y a trois gousses qui sont pourries. On n'a même pas vu. Donc voilà. Non non. Les produits ça me-- ça me vas pas

*Mme. 1:* Et donc c'est-- est-ce, enfin, est-ce que c'est exclusivement sur le fait qu'ils s'abîment trop vite ? Ou [euh], ou--

*Mme. 2:* Ils s'abîment oui.

*Mme. 1:* Ouai

*Mme. 2:* Il n'y a pas de conservation qu'ils s'abiment, et la qualité des produits.

*Mme. 1:* [Mm]

*Mme. 2:* Par exemple, [bah] on tranche les oranges. Je prends une orange avec jus chez mon primeur, c'est une bonne orange avec du jus dedans. J'ai exemple [inaudible 00:16:04-00:16:08] une orange, une pomme, enfin bref, et je leur ai donné des oranges pour faire du jus de fruit. Mais ma fille m'a dit: " mais moi, j'achète chez Leclerc, j'ai pas autant de jus dans mes oranges que nous, on a deux oranges et le prix équivalent encore. "

*Mme. 1:* Ouai.

*Mme. 2:* Donc [voilà], je trouve que la qualité [euh], la qualité est là aussi quoi. Il le faut vraiment. Ces des produits ont peut-être été plus longtemps entreposés aussi. Moi je peux dire frais puisque même chez le primeur quand j'ai des courgettes, c'est pas forcément frais non plus.

*Mme. 1:* Ouai.

*Mme. 2:* Mais [euh] c'est des produits qui sont moins-moins moins promenés, moins travaillé donc qui sont purs et puis moins vieux. C'est ce que je vois. [euh]

*Mme. 1:* Mais oui. [Et du coup], il la quand vous êtes dans les surf-- grandes surfaces, est-ce que pareil, il y'a un certain plaisir à-a faire ses courses ou pas ? comment--

*Mme. 1:* Chez--au primeur ?

*Mme. 1:* Non, en grande surface.

*Mme. 2:* Oui, c'est moi, si je vais au drive, ça me va très bien.

*Mme. 1:* D'accord.

*Mme. 2:* Mon minimum syndical [inaudible 00:17: 05-00:17:09). Je vais encore plus au drive chez Leclerc et je vais chercher des petites courses, ce qu'on appelle des petites courses.

*Mme. 1:* Ouai.

*Mme. 2:* Toutes les semaines, quand il n'y a personne dans les magasins puisque j'ai le choix. Donc je ne vais pas aux heures de pointe.

*Mme. 1:* Oui.

*Mme. 2:* Ni vendredi ni samedi.

*Mme. 1:* Ouais

*Mme. 2:* Donc [voilà], je vais acheter. L'appoint que les produits sont achetés. [euh]-sont achetés par le drive, quoi essentiellement.

*Mme. 1:* C'est que vous préférez faire un propre effet drive [euh] room direct dans le cercle des gens qui--

*Mme. 2:* Non j'ai jamais aimé les room. J'aime les faire, mais j'aime pas faire les magasins.

*Mme. 1:* Oui.

*Mme. 2:* J'aime pas faire les magasins. J'aime pas faire les magasins, je suis pas une fan de magasins. C'est pas ma-- c'est moi qui aime faire des sorties funs. Les magasins, moi je-- si je vais aux magasins, c'est que j'ai besoin de quelque chose autrement.

*Mme. 1:* Ouais.

*Mme. 2:* Mais ils me font travailler, ce n'est pas une sortie pour moi, le magasin.

*Mme. 1:* Oui. Maintenant, on sait que une classe c'est pas un plaisir et par contre le primeur ou le marché [euh], vous avez du plaisir.

*Mme. 2:* Tout à fait, ouai. J'y mets- j'y vais même pas dans le rayon.

*Mme. 1:* Ouai.

*Mme. 2:* Je contourne un rayon, je ne m'approche-- je ne m'approche même pas des fruits. [Rire]

*Mme. 2:* Ouai.

*Mme. 2:* Je n'y vais pas. Je vais à la poissonnerie souvent, au bout.

*Mme. 1:* Ouai.

*Mme. 2:* [Papp], puis je traverse tout pour aller au liquide, pour finir les courses et c'est tout. Minimum. [euh]

*Mme. 1:* Ouai. Et quand ne vous disiez bah la dernière fois si exceptionnellement, il me manque de l'ail pour ça, on en prend ici.

*Mme. 2:* Oui on en prend au super marché si jamais vraiment il n'y en a pas.

*Mme. 1:* C'est ça et quand par exemple qu'il y a déjà des gousses qui sont abîmées, est-ce que vous-vous voulez jeter ? Qu'est-ce que ça vous fait de--

*Mme. 2:* [Ah bah] Je- je-- ça m'horripile un peu. Je trouve que c'est- c'est non seulement du gaspillage, mais [euh] c'est-c'est de vendre des produits qui sont pas-- qui sont pourris, enfin voilà.

*Mme. 1:* Oui.

*Mme. 2:* Moi j'achète des patates, j'achète des oignons, j'achète-- ils sont là tout l'hiver et ils ne sont pas pourris. Donc on a envie de savoir comment ils sont entreposés leur truc quoi. C'est l'humidité de l'eau ? Où ils sont ramassés [euh] c'est humide ? [Enfin], je sais pas. [bah] C'est eux aussi qui entreposent peut-être mal aussi leurs produits.

*Mme. 1:* [Mm-hmm]

*Mme. 2:* Pas forcément, le supermarché encore au-dessus.

*Mme. 1:* Oui. Avant [euh]--

*Mme. 2:* Avant [quoi] ! [Voilà], ouai. [Mm-hmm]

*Mme. 1:* D'accord. Si ça--vous allez être énervé d'avoir de jeter, mais--

*Mme. 2:* Ouai, [bah] oui.

*Mme. 1:* Enervé contre la--

*Mme. 2:* Tout à fait, ouai. Contre [bah] oui, contre le-le système.

*Mme. 1:* C'est ça.

*Mme. 2:* Voilà le système, de-de production de produits. Surtout que ça ne met pas en valeur nos produits [euh], [voilà]. Le pri- le- le-- enfin, le primeur [euh] on-- de plus en plus en plus c'est des produits qui sont apparemment c'est marqué dessus étiquettes, machins primeur du coin, mais ça ne met pas en valeur.

*Mme. 1:* Oui.

*Mme. 2:* On en a envie, ça donne que du boulot, quoi.

*Mme. 1:* Oui.

*Mme. 1:* Il y a du boulot, quoi.

*Mme. 1:* Oui.

*Mme. 2:* Il y a du coup là-dessus.

*Mme. 1:* Oui vous trouvez que [enfin], [en vrai], ça donne pas envie dans les supermarchés ?

*Mme. 2:* Non. Pas du tout. Moi, pour moi oui.

*Mme. 1:* Est-ce que vous-vous sauriez dire pourquoi justement, ils ont-- ils sont moins enviés ou ils ne sont pas valorisés ces produits ? Ce type de magasin ?

*Mme. 2:* [Bah], je ne sais pas c'est mal achalandé de toute façon, mais bon, je pense que ça dépend des magasins peut-être aussi, mais en fin de compte moi, ça ne m'a jamais--en fait, comme je- je- j'ai pas ces habitudes-là, je peux avoir du supermarché ou je vais aller. Ma fille travaille au relais, cours Leclerc, je suis allé voir, il est super joli, Leclerc Rolex, j'y vais, mais je-je ne vais jamais au march-- au rayon fruits et légumes. Peut-être que c'est bien achalandé. On vit-mais j'y vais jamais, j'ai pas envie.

*Mme. 1:* [Mm-hmm]

*Mme. 2:* [inaudible 00:20:27-00:20:33] J'ai fait une croix dessus, c'est sûr, Ouai. Non non, franchement [euh] la seule chose moi c'est une gousse d'ail, c'est un bouquet de persil.

*Mme. 1:* Ouai.

*Mme. 2:* Ou encore un peu de persil. [Mais bon], parfois je vais [euh], [voilà] je vais quand même [euh] si j'ai que un peu de persil à acheter, je vais chez le primeur chercher que ça. Mais si j'ai autre chose à acheter, Je me rends en ville, je suis pressé, j'y passe toujours-- souvent donc. Sinon, je prends le temps de faire les deux tranquillement.

*Mme. 1:* Oui.

*Mme. 2:* Quand j'ai le temps, [bah] je prends le temps [quoi] ! [Voilà]. C'est pas--

*Mme. 1:* Ouai.

*Mme. 2:* Par contre, quand je travaillais c'était différent. On a moins le temps, on se pose moins de questions. [Alors là], là c'était tout. Drèves Légumes, fruits, légumes et tout le tralala à l'époque [hen] ce qui était pas-- mais il y avait pas de primeur non plus.

*Mme. 1:* Ouai.

*Mme. 2:* donc, [voilà].

*Mme. 1:* OK, et [alors], ultime photo. [rire]

*Mme. 2:* [Ah bah] ça, c'est le potager.

*Mme. 1:* Oui.

*Mme. 2:* Le potager [euh]. On a des bons produits, on aime bien cultiver dans son jardin ses petites salades. Moi, j'aime ça, moi.

*Mme. 1:* Oui.

*Mme. 2:* Ce n'est pas grand-chose [hen], ça correspond à ça.

*Mme. 1:* Oui.

*Mme. 2:* Mais j'aime bien mes herbes. J'aime bien [euh]-- surtout les herbes, tout ce qui est herbes, persil, l'oignon, persil, ciboulette.

*Mme. 1:* Ouai.

*Mme. 2:* Lorsque je mets de la verveine, en fait toutes les aromatiques que j'aime beaucoup, mes salades.

*Mme. 1:* Ouai.

*Mme. 2:* J'ai mis courgettes, j'ai des poivrons. Petit petit potager. J'ai mis trois plants de tomates. Ça a donné beaucoup.

*Mme. 1:* D'accord.

*Mme. 2:* Un petit potager [euh] familial. On ne manquait pas de productions pour avoir du plaisir.

*Mme. 1:* Oui.

*Mme. 2:* Production ? Je ne peux pas. J'aurais pas de sol assez puisque je mange beaucoup de fruits et légumes.

*Mme. 1:* Oui

*Mme. 2:* Je n'aurai pas assez non plus de production pour me faire plaisir.

*Mme. 1:* D'accord, et il vient d'où selon vous, le plaisir de--pour le potager ?

*Mme. 2:* [Bah] Moi, je suis la tête dans la terre, c'est bien. J'ai la tête dans mon jardin, c'est bien.

*Mme. 1:* [Rire]

*Mme. 2:* C'est gâté, ça détend. [Oh là là] ! Oui, bon, moi, dès qu'il fait beau, je file à maison toute rouge, ça me coûte de rester dedans, je sors. Et puis le plaisir de se vider la tête. Et puis je me force à pas le toucher parce qu'on faut le laisser se reposer un peu, le jardin.

*Mme. 1:* D'accord.

*Mme. 2:* Je me force à ne pas y toucher, sinon ce serait--

*Mme. 1:* [Rire]

*Mme. 2:* Bon et tant mieux en plus déjà le mois de janvier.

*Mme. 1:* [Rire]

*Mme. 2:* Le potager c'est- c'est-- j'aime bien, c'est un plaisir.

*Mme. 1:* Oui.

*Mme. 2:* C'est un plaisir. Puis de manger des produits qu'on a-- qu'on a. Mais c'est ça que je fais les salades, moi j'aime bien les salades. Je me mets vraiment à la production de salade tranquille tout l'été [euh] qui se fait.

*Mme. 1:* Ouai.

*Mme. 2:* Puis, je trouve ça super sympa.

*Mme. 1:* D'ailleurs, vous-- c'est quoi des produits que vous arrivez à produire ?

*Mme. 2:* [Bah] L'ansol, et bon les radis [hen]. Vu que c'est la saison, c'est les radis jusqu'au- jusqu'au-- jusqu'au mois de juin [hen]. Après bon la salade, tout de suite, dès que je peux, je mets de la salade, j'ai mis [euh] des poivrons. Cette année, c'était des minis, donc j'ai eu un tout petit peu.

*Mme. 1:* D'accord.

*Mme. 2:* Très très peu. L'année prochaine, je vais mettre des piments, c'est plus intéressant parce que ça pousse aussi.

*Mme. 1:* D'accord.

*Mme. 2:* Mais quand on les coupe, on amène, on les garde tout l'hiver nos congèle [euh] J'ai des courgettes, j'ai mis des épis de courgettes qui envieillissaient partout puisque j'ai un petit potager.

*Mme. 1:* Oui.

*Mme. 2:* Donc c'est des pieds, et [bah], c'était su-- j'ai eu pas mal de courgettes, c'était sympa. C'étaient les jaunes.

*Mme. 1:* D'accord.

*Mme. 2:* C'est des surprises. Je ne savais pas qu'elles étaient jaunes. Qu'est-ce que j'ai foutu ? [Rire] [inaudible 00:23:52-00:23:54] Et [voilà] quoi. Un petit plaisir. De petites fraises, des tomates, [voilà] un petit pot-- un petit potager. Qui fait [euh]- qui fait, ouai, qui fait plaisir. Et puis, surtout les aromatiques [hen] j'aime bien.

*Mme. 1:* Oui.

*Mme. 2:* Le romarin, est-ce que j'ai d'autres encore ? Lauriers de la monte. Et que on a tous les hivers aussi parce que j'ai toujours [euh].

*Mme. 1:* Ouai.

*Mme. 2:* Je conserve tous mes aromatiques que fraîche, c'est sympa.

*Mme. 1:* Et [euh] duquel vous disiez qu'il y avait le plaisir de les manger ?

*Mme. 2:* Ouai.

*Mme. 1:* Et le plaisir est-- il est [euh]-- il est relatif au fait que c'est-c'est vos produits ou pas.

*Mme. 2:* [Bah] Déjà que c'est mes produits, mais je sais comment je les ai--

*Mme. 2:* Oui.

*Mme. 2:* Comment je les ai cultivés, je ne sais pas traiter. Et je les ai-- je sais que je peux les manger quand ils sortent du jardin, que les radis, les petits si on enlève la terre, il les mangent comme ça, et qu'ils vont pas une malade parce que--

*Mme. 2:* Oui.

*Mme. 2:* [Voilà], parce que il n'y a pas de-- il n'y a pas d'engrais et de pesticides, mais tout ce qu'ils ont ensuite suivent.

*Mme. 1:* Oui.

*Mme. 2:* [Voilà] essentiellement.

*Mme. 1:* Et [euh] est-ce que ça vous arrive des fois de [voilà]- d'être amené à jeter un fruit ou légumes dans le jardin ?

*Mme. 2:* Dans je jardin, c'est rare.

*Mme. 1:* Oui.

*Mme. 2:* Ou je ramasse avant-- ou je ramasse avant si peut-être en fin de saison on vraiment en fin-fin, une courgette est un peu abîmée. J'enlève juste le petit coin puis je- je la mets au congèle tout de suite.

*Mme. 1:* D'accord.

*Mme. 2:* Des choses comme ça, mais c'est-[Ah] c'est très rare. On les ramassait vraiment avant. [euh] Si j'ai des petites-des petites tomates ou comme les pêches que j'avais [inaudible00:25:23-00:25:27]

*Mme. 1:* Ouai.

*Mme. 2:* Mais ça ne va pas. Voilà ça va pas--rarement à la poubelle [ah non] ! C'est retraités [voilà]. De façon à ce que--les choux ils aiment bien. Tout ça, oui, ils aiment bien, |voilà].

*Mme. 1:* Mais des fois, c'est les choux qui-- quand les feuilles sont un peu abîmées. [euh]

*Mme. 2:* Ouais, oui, essentiellement un fruit. Oui bah si, ça peut arriver dans le jardin, au moins. Ici c'est rare on mange tout même pour une pomme. Là c'est-- on a acheté des pommes et ça commençait à être peut-être un peu flétri.

*Mme. 1:* Ouai

*Mme. 2:* On les mange quand même. [hen] Pas grave. [hen] Donc ça gêne pas. [Rire] On ne peut plus faire de jus puisqu'il n'y a plus assez de jus dedans, mais oui, on les mange quand même. [hen] Parce que les pommes c'est pareil. On achète pour tout l'hiver, donc [euh]-- chez un--dans un verger [hen]. Donc forcément là maintenant elles sont moins jolies. Mais ce n'est pas grave. Elles sont mangées qu'à même. Les pommes, les kiwis et les poires.

*Mme. 1:* Pommes, kiwi et poire, ça c'est vous allez les chercher dans un verger ?

*Mme. 2:* [AH] ouai, à Caron. Il y'a beaucoup de gens qui en vont chercher.

*Mme. 1:* Oui, donc il y'a ça, puis il y'a les légumes que vous disiez que vous allez chercher chez le producteur ?

*Mme. 2:* Oui, chez le producteur.

*Mme. 1:* [Et du coup], Vous pensez quoi de ces produits-là ?

*Mme. 2:* [Ah bah] Ils sont top. [Alors] Ce n'est même plus un producteur puisque c'est un monsieur en retraite.

*Mme. 1:* D'accord.

*Mme. 2:* Et c'est le père d'une amie à moi. [rire]

*Mme. 2:* D'accord.

*Mme. 2:* Qui était pech-marin pêcheur et [euh] agriculteur à l'époque. Donc aujourd'hui--

*Mme. 1:* Il faisait les deux ?

*Mme. 2:* Mais voilà, il faisait les deux. [Bah] oui, puisque à Clairemon il a la-- il a la mer, donc ils faisaient les deux.

*Mme. 1:* [Mm-hmm]

*Mme. 1:* Et puis, mais ce n'est pas une grosse production, mais il a continué en retraite pour arrondir ses fins de mois. En fait.

*Mme. 1:* Ouai

*Mme. 2:* Et nous, [bah] je continue à aller chercher là-bas. Mais il s'est mis en retraite [hen]. Et je trouve ça sympa parce que-- puis c'est marrant, on- on- on est-on arrive là-bas, on veut des fruits, mais il nous met plein de trucs en plus. Je commande beaucoup [hen], je commande 200 kilos [euh].

*Mme. 2:* [Ah Oui].

*Mme. 2:* De-de pommes de terre parce que c'est ça, c'est à peu près ça, ouai, c'est ça 200 kilos dans la saison.

*Mme. 1:* D'accord.

*Mme. 2:* Plus les oignons, plus [euh] plus l'ail, les carottes [eh bah], voilà toujours des potirons, plus des courgettes, des machins. Voilà, puis bon le service c'est plus agréable quand même. [Ah] ! Le seul inconvénient que je vais y trouver, c'est que la conservation est un peu compliquée.

*Mme. 2:* D'accord.

*Mme. 2:* Maintenant qu'il y a plus bon, après c'est plus, c'est clos. Donc il n'y a plus jamais d'antigènes dessus.

*Mme. 1:* Oui.

*Mme. 2:* C'est interdit. Donc [euh] j'ai été à un magasin vert. J'ai trouvé un produit à base de mantes écolo-écologique. Pour [euh] brantiger mes pommes de terre, mais ça marche pas. Je voulais lui dire que ça marche pas.

*Mme. 1:* [Ben oui]

*Mme. 2:* J'ai fait un essai. Ça marche pas. Ça coûte très cher, mais ça marche pas.

*Mme. 1:* Ouai. [Rire]

*Mme. 2:* Ouais ça m'est revenu à 20 euros. Enfin, c'est cher 20 euros [hein]

*Mme. 1:* Oui.

*Mme. 2:* Juste pour [euh]-- juste pour mes patates et ça pas marché. Donc inutile, [voilà].

*Mme. 1:* Mais elles ont quand même quand même germé ?

*Mme. 2:* Elles ont germée. Mais pourquoi pas, on les mange qu'à même.

*Mme. 1:* Oui.

*Mme. 2:* On les a toujours mangé quand même. Ça ne nous a jamais aimé-- gêné de manger des pommes de terre germées. Après, elles ne sont pas traitées. Elles sont engraissées goémon donc--.

*Mme. 1:* Oui.

*Mme. 2:* Pareil un autre traitement de-- agressif.

*Mme. 1:* Oui.

*Mme. 2:* [Bon voilà]. Ça ne changera pas. C'est plaisant depuis plus de dix ans, et plus que-que ça même, ça doit faire 12 ou 13 ans.

*Mme. 1:* [Et du coup], vous savez tout contrôler, comment vous y allez ?

*Mme. 2:* Le mois de septembre, enfin, fin septembre quand ma collègue m'appelle elle me dit :" [Ah] c'est bon, ça y est ". Donc vraiment mon amie-vraiment, je vais là-bas à la ferme directement.

*Mme. 1:* Une fois du coup par saison ça--

*Mme. 2:* Ouai. Une fois par saison. C'est tout. C'est arrivé l'année dernière quand on a eu besoin d'un sac supplémentaire. Ma collègue qui habite ici ou je passe par chez elle à Leslevent puis, [euh] elle habitait entre les deux, donc je prends un- un sac. Mais |voilà] en plus supplément. [Mais bon] ! [Voilà] c'est pas forcément l'or. En général, on arrive bien à gérer, parce qu'après les pommes de terre nouvelles arrivent.

*Mme. 1:* Oui.

*Mme. 2:* Donc on n'achète pas trop non plus de-- enfin, on va pas racheter maintenant parce que dans un moi, on arrive au bout-là.

*Mme. 1:* Oui.

*Mme. 2:* Puisque bon de toute façon des pommes de terre nouvelles arrivent donc [euh]. [Ah] pas tout de suite [hen] [mais bon]!

*Mme. 1:* [Mmhmm]

*Mme. 2:* Ouai ça va venir donc, voil-- on- on calme.

*Mme. 1:* Et vous- et vous- vous-vous faites saison d'hiver?

*Mme. 2:* Ouai, c'est ça, ouai, ouai, tout à fait ouai. Pour les soupes, les potages et tout le reste.

*Mme. 1:* Ouai.

*Mme. 2:* [Ah] C'est sûr.

*Mme. 1:* Est-ce que ça vous arrive des fois [du coup], de les perdre, ces produits quand vous les jetez, qu'est-ce que ça vous fait ?

*Mme. 2:* Je jette pas.

*Mme. 1:* Oui.

*Mme. 2:* C'est très [euh], alors la fin. Si on n'a par exemple une pomme de terre un peu abimée ou un peu-- comme ça va au chaud [hen], mais moi, je jette pas.

*Mme. 1:* Ouai.

*Mme. 2:* Je mets pas à la poubelle.

*Mme. 1:* D'accord.

*Mme. 2:* Pour moi, mettre dans la poubelle ça-- c'est nul, en fait ça--c'est une démarche que je fais pas. Je ne mets pas dans la poubelle. Je vais-je vais donner aux chevaux. Ou alors aux-- ou à côté s'il y avait des chèvres qui mangent ça de mon frère [euh]. Et on ne donne pas la patate entière. Si elle est abimée, je vais juste couper la part qui est abimée.

*Mme. 1:* Oui.

*Mme. 1:* Je ne veux pas jeter la patate entière. Ça c'est hors de question, [voilà]. Juste petit morceau abîmé, on enlèvera le morceau abîmé. Je pense que c'est une habitude de consommation. [euh]

*Mme. 1:* Oui.

*Mme. 2:* [inaudible 00:30:25-00:30:26].

*Mme. 1:* Donc pour vous c'est différent de donner au cheval que de le mettre à la poubelle ?

*Mme. 2:* [Ah] ouai. Pour moi, c'est inutile de le mettre à la poubelle, ouai. Je ne vois pas l'intérêt. Parfois je- je-- ça m'apporte pas satisfaction vous donner au cheval. Je suis content de le voir mon cheval, il est content, après il a l'air content. [Rire]

*Mme. 1:* [Rire]

*Mme. 2:* Ils sont échappés par-- qu'il y a pas longtemps, ils l'ont pris. Il m'a reconnu, ils m'ont suivi tous les deux. Ça c'est beau [inaudible00:30:51-00:30:52].

*Mme. 1:* [Rire] OK, [bah] ça marche. Alors une autre partie pour terminer. [Alors] est-ce que vous consommez des bananes ?

*Mme. 2:* Ouai. De temps en temps.

*Mme. 1:* De temps en temps.

*Mme. 2:* On n'a pas forcément toujours la banane, |non voilà]. Parfois il n'y en pas, mais parfois.

*Mme. 1:* Oui.

*Mme. 2:* C'est mon fils. Souvent il aime bien.

*Mme. 1:* Il y'en a qu'à même [euh] de temps en temps aussi [quoi].

*Mme. 2:* Ouais, ouai, ouai, ouai, pas excessivement, mais de temps en temps.

*Mme. 1:* Alors ?

*Mme. 1:* [inaudible 00:31:28-00:31:29] parce que [euh], [Rire] on en prend quatre ou cinq. On en prend toujours trop.

*Mme. 1:* Ouai.

*Mme. 1:* Et [euh] quand on voit qu'elle commence à être un peu abimée [inaudible 00:31:35-00:31:36]

*Mme. 1:* Et alors, mais c'est ça justement.

*Mme. 2:* C'est ça, allé [up] !

*Mme. 1:* Mais je vais vous demander.

*Mme. 2:* Oui.

*Mme. 1:* Donc il y a dix photos de Bananes.

*Mme. 2:* Ouai.

*Mme. 1:* De les ranger. De celle qui vous fait [euh] le plus envie à celles qui vous donnent le moins envie. Ouai à consommer comme ça.

*Mme. 2:* D'accord. Alors déjà celle-ci un peu moins, celle-ci, en fait-- un peu moins, moins, un peu moins on va dire. Mais ça non plus, parce que c'est ouvert, moi j'aime pas ça. [inaudible 00:31:59-00:31:60]. Ça, ça va. Ça, ça va. Ça, ça va, et celle-ci, ça va aussi. Ça, c'est encore bon. Après, à acheter, je sais pas. A acheter ou à en consommer ?

*Mme. 1:* Et ouai [euh], celles qui vous donnent envie d'être mangée et, celles qui est au contraire ne vous donnent pas envie.

*Mme. 2:* Ouai, je ne suis pas très alèse, mais bon, cela ne me gênerait pas. Il y'aurait juste un litige entre celles-ci. On va la mettre dans--voilà. C'est pour faire un michec et [voilà].

*Mme. 1:* Ouai ça--

*Mme. 2:* Ça ne me gêne pas.

*Mme. 1:* On va regarder justement ce qui va vous--

*Mme. 2:* Ça me gêne pas.

*Mme. 1:* Alors si on les range, si je fais une photo de classement.

*Mme. 2:* Alors si on les range, alors ça non, en dérhibitoire et trois autres aussi oui.

*Mme. 1:* Celle-là vous ferait le plus envie ?

*Mme. 2:* [euh] Pas forcément celle-là.

*Mme. 1:* C'est laquelle s'il fallait qu'on arrange [euh] ?

*Mme. 2:* Alors si on range, on va ranger en ordre chronologique. [Bon bah] celle-là ça va, celle-ci est un peu verte [hen], ça nous ramène à là. [euh] Là, ça va, celle-ci aussi on va mettre ça-- ça m'a plus en fait, mais non, si on va dire moins.

*Mme. 1:* Ouais,

*Mme. 2:* [Voilà], ça c'est, [voilà].

*Mme. 1:* Ça va vous--

*Mme. 2:* L'ordre, [voilà].

*Mme. 1:* OK, je prend une photo. Et celle-là vous la-vous-- ça vous fait quoi de ça?

*Mme. 2:* [Alors bah] Ça, j'ai envie de dire presque tout ça. Je vois ça [euh], ça je prends pas, ça je prends pas, ça [ppp] même pas [hen] et [euh] ça non, là j'en veux pas. Je sais pas ces bananes merde, ça va pas murir chez moi.

*Mme. 1:* Oui, donc vous les achetez même pas quand elles sont vertes ?

*Mme. 2:* [Ah non], non, non pas du tout.

*Mme. 1:* Parfait, [alors du coup], dans l'idée, ce serait de me dire ce que vous faites déjà quand elle est verte comme ça.

*Mme. 2:* [Mm-hmm]

*Mme. 1:* Je veux dire, des fois, je dis pour que les gens puissent [Tpuip] savoir de quelles bananes on parle.

*Mme. 2:* Ouai.

*Mme. 1:* Donc quand c'est triangle rose là, vous n'achetez même pas.

*Mme. 2:* Non.

*Mme. 2:* Elle sera jamais comme ça chez vous.

*Mme. 2:* Non, trop vert.

*Mme. 1:* OK, et donc quand il y a le carré [euh] blanc.

*Mme. 2:* C'est pas trop mal.

*Mme. 1:* Oui

*Mme. 1:* Ouai, c'est pas trop mal là, oui. Là ça va, je me dis bon, ça va.

*Mme. 1:* OK

*Mme. 2:* Mais après, franchement, je prendrais plus une gachet [hen]. Voilà celle-ci ou celle-là.

*Mme. 1:* Le carré sur blanc.

*Mme. 2:* Pourquoi pas ?

*Mme. 1:* Ouai.

*Mme. 2:* S'il y avait autre chose, ça pareil, pourquoi pas ?

*Mme. 1:* Le triangle, donc le triangle le bleu ?

*Mme. 2:* [Mm-hmm]

*Mme. 1:* OK.

*Mme. 2:* Ça, pas ouai, ouai, ouai. S'il y a à choisir entre les deux, je choisirais plutôt celle-là.

*Mme. 1:* Vous préférez le triangle vert ?

*Mme. 2:* Carrément celle-là. Puisque je trouve que je me dis, elle est mûre.

*Mme. 1:* Ah ouai.

*Mme. 2:* Elle est prête à manger.

*Mme. 1:* Celle qui vous fait finalement le plus envie à être mangée ce serait--

*Mme. 2:* C'est celle-là.

*Mme. 1:* Le carré orange, [voilà]. D'accord, et donc après, c'est le rond blanc.

*Mme. 2:* Après s'il y a ça par exemple, moi si je prends un panier par exemple chez le, voilà chez mon--le distoukeur. [Ah ouai ouai] Je suis abonné maintenant à tout coup. Tout ça finit vite.

*Mme. 1:* Ouai.

*Mme. 2:* [Eh bah] En fait, voilà ça, ça me gêne pas.

*Mme. 1:* Ouais.

*Mme. 1:* Je ferais mes jus, [voilà], consommation je ferais un michec avec.

*Mme. 1:* Donc là en blanc, vous ne la mangez pas crue ?

*Mme. 2:* Mon mari si, mon mari si, moi non. Mais moi j'ai du mal avec les bananes moi.

*Mme. 1:* Ouais.

*Mme. 2:* Déjà dans un premier temps. Mais là, si j'ai ça ou ça, ça me gêne pas de l'ouvrir enfin, mais je ne jetterais pas.

*Mme. 1:* D'accord.

*Mme. 2:* Ça, c'est certain que je jette pas ça. Et on achète une banane écrasée. Les compotes, mon mari, mais ça aussi dans les pommes. [Bah] Il aime bien faire les compotes avec des bananes.

*Mme. 1:* Oui.

*Mme. 2:* Aux bananes.

*Mme. 1:* Ou c'est en michec ou en compote.

*Mme. 2:* Voilà, ouai. Les crus [euh] Ouais, voilà cru, on va s'arrêter peut être là, quoi.

*Mme. 1:* Ouai.

*Mme. 2:* Mon fils en mange et c'est mon fils qui mange en cru, je veux dire, voilà. Mais il va pas manger vert non plus. Je sais qu'il y en a qui aime bien. Mais il ne mangera pas vert.

*Mme. 1:* Oui. D'accord. Et donc après celle-là.

*Mme. 2:* Celle-là j'achète pas du tout et celle-ci je le trouve un peu trop noire. Ce que je me dis c'est dedans, est-ce que c'est pareil ou pas?

*Mme. 1:* Ouais,

*Mme. 2:* C'est un risque en fait, donc ouai. Je ne prendrais même pas et peu importe l'ordre. J'ai peut-être on va dire que même-- bon là allez elles sont bonnes, mais je ne prendrais même pas, j'achèterais même pas.

*Mme. 1:* Ouai.

*Mme. 2:* Aucune des-aucune des-- aussi peu que la toute verte, moi, j'achèterais pas la toute noire non plus.

*Mme. 1:* Oui. OK. Est-ce que selon vous, il y a un moment ou la banane, elle peut être dangereuse pour la santé ou-ou pas ?

*Mme. 2:* [Ah bon] Dangereuse pour la santé ? Je ne suis pas sûr. Mais après, si elle est toute noire, si c'est peut-être pas chez toi, si c'est très mou, si c'est oxydé, je pense que s'il est tout de noir dedans, non c'est pas terrible. Tu manges et le gout n'est pas bon. Est-ce que naturel ce n'est pas nécessaire ? Donc le goût n'est pas bon, donc c'est pas bon pour le corps.

*Mme. 1:* Ouai.

*Mme. 2:* [Eh bah] ! C'est ce que je me dis. Moi je me dis que là, c'est bon-là, il n'y a pas de soucis [hen] tant qu'elle est un peu blanche, même un peu tachée [hen]. Pour moi, c'est pas mauvais pour la santé.

*Mme. 1:* Mais pour vous, c'est le signe naturellement que le corps est vous dire si c'est bon ou pas.

*Mme. 2:* Ouai, c'est ça ouai, ouai. Moi je pense ouai, ouai. Un avocat qui est très noir, qui vous verre, qui est très noir, il peut être bon, et moche.

*Mme. 2:* Oui.

*Mme. 2:* Bon et si vraiment il n'a pas bon goût, bah voilà, on rejette et puis on ne mange pas. Je pense qu'on peut faire facilement la part des choses.

*Mme. 1:* Ce n'est pas forcément l'aspect visuel qui compte ça va être le goût qui va déterminer.

*Mme. 2:* Là j'ai acheté il n'y a pas longtemps une mangue puisque on est dans l'époque et mangue n'était pas très jolie, [bah alors] qu'est-ce qu'elle était bonne.

*Mme. 1:* Oui.

*Mme. 2:* [Ah] c'était super pour bon. [Voilà] et puis pas très jolie et un peu abimée, enfin comme si elle était abimée et puis |bah] finalement.

*Mme. 2:* oui.

*Mme. 2:* [Oh là là] ! [Eh] bah elle était très bonne.

*Mme. 1:* OK. Est-ce que selon vous, il y a un moment donné ou [euh] la banane, elle perd ses apports nutritifs, ou alors les apports en calories ?

*Mme. 2:* Ouais, je pense quand même, ouai, je pense que là [euh], là ça-- c'est des fibres, alors je crois que les bananes il y'a beaucoup de fibres et de vitamines tout ça. Mais je pense que quand elles sont pourries, ça veut dire que ça a commencé à détruire. J'imagine la qualité nutritive de la banane.

*Mme. 1:* Oui.

*Mme. 1:* Je ne pense pas à l'or vraiment, mais quand ça devient noir, ça, si c'est noir dedans, je pense que oui. Pas noir- noir, menton [euh]. Une banane, elle peut-être noire d'un côté et blanche de l'autre, je vais la manger. Bon j'ai- je vais couper le côté noir je mangerais l'autre.

*Mme. 1:* Oui.

*Mme. 2:* Ça veut pas dire la banane en entier, la part qui est abimée, je pense que ça perd vraiment de nutritif.

*Mme. 1:* Oui.

*Mme. 2:* Nutriment. [rire]

*Mme. 1:* Mais par c'est-- [rire] et quand elle est verte comme ça, est-ce qu'elle a ses qualités ou pas? C'est-à-dire qu'est-ce que vous, vous pensez de cette--

*Mme. 2:* Moi je pense que c'est une histoire de goût. En fait, il semble qu'elle n'est pas mure et que bon j'aime pas les fruits, j'aime pas les bananes mûres. Verte, j'ai l'impression qu'elles ont maqué de soleil quoi.

*Mme. 1:* Oui.

*Mme. 2:* Quelle ont pas-- quelles ont été ramassées trop tôt. Donc elles n'ont pas fini de mûrir en fait.

*Mme. 1:* Oui.

*Mme. 2:* Je pense qu'une banane, comme tout fruit, si tu en récolte trop tôt, il peut ne pas aussi y avoir tous les- toutes les qualités [euh] nutritives.

*Mme. 1:* Oui.

*Mme. 2:* D'un fruit qui a été engorgé de tout ce qu'il fallait et cueilli à maturité. [euh] C'est pas pour rien que là maintenant, parce qu'il est mou à mon goût comme le raisin, pour faire les vins.

*Mme. 1:* Oui.

*Mme. 2:* Tout ça, il y a des seuils de maturité. En fait, je pense.

*Mme. 1:* Oui. OK, alors ça roule pour la banane. [rire]

*Mme. 2:* Ça roule pour la banane. [inaudible 00:39:27-00:39:28] [rire]

*Mme. 1:* Alors l'autre bout, alors là, ce sont [toux] des fruits et légumes qui ont été jetés par [euh] les participants, il faudrait. Et ici, [alors] j'aurais dû mettre ça, on a ce qu'on peut faire, en gros des fruits et légumes.

*Mme. 2:* Ouai, composte.

*Mme. 1:* Donc il y a les différents types de belles--

*Mme. 2:* Chez une amie, moi je regarde plus les coquillages, tous les coquillages pour ses poules.

*Mme. 1:* Pour les poules ?

*Mme. 1:* Ouai

*Mme. 1:* Donc pour vous les poules, on pourrait dire à la rigueur, ça peut être les chevaux ?

*Mme. 2:* Ouai, voilà, on peut dire ça.

*Mme. 1:* On pourrait dire ça. [diaphonie 00:40:13-00:40:17]

*Mme. 2:* Le composte, non jamais.

*Mme. 1:* Ouai.

*Mme. 2:* Ça, c'est pas, ça c'est pas, ça c'est pas--

*Mme. 1:* Il y avait des rats.

*Mme. 2:* J'avais des rats, je ne sais pas, [voilà].

*Mme. 2:* Et ça, [du coup], c'est le-- une poubelle de classique.

*Mme. 2:* [Bah] c'est une poubelle classique, [voilà] quoi. Ça ne va pas aux chevaux puisqu'il y a des choses qu'il ne faut pas donner forcément, [quoi].

*Mme. 1:* Exactement.

*Mme. 2:* On est obligé de mettre ça dans la poubelle. [hen]

*Mme. 1:* Et là, c'est différentes choses qu'on peut faire avec les fruits et légumes.

*Mme. 1:* Ouai.

*Mme. 1:* Et donc, l'idée c'est que vous gardez celles qui vous parlent, des choses que vous, vous-vous faites--

*Mme. 2:* D'accord.

*Mme. 1:* Avec vos fruits et légumes.

*Mme. 2:* Ouai, [voilà] savoir [euh], [bah] ça c'est-- ça c'est congèle.

*Mme. 1:* Oui.

*Mme. 2:* C'est congèle, bah oui, moi [euh]

*Mme. 1:* Ouai.

*Mme. 2:* je suis la reine de [euh]-- j'ai acheté deux kilos de--chez Bruxelles l'autre jour, je les ai blanchies, je les ai congelées.

*Mme. 1:* [Bah oui]. Mais vous congelez que ce soit préparé ou pas encore, vous congelez ?

*Mme. 2:* Ouai, je congèle pratiquement systématiquement pour éviter que ça s'abîme tout simplement. Pas toujours j'ai un brocoli, mais je sais que je vais le faire. Je l'ai acheté en entier, donc je sais que je vais le faire-là, [voilà].

*Mme. 1:* Oui.

*Mme. 2:* Après [euh] avant hier, on est qu'aujourd'hui, mardi, non c'était vendredi. Mon mari adore les compotes. Oui, il les fait [hen] la cuisine, [bah oui], cuisiner [euh]

*Mme. 1:* Ouai, des poêlées, enfin c'est cuire [euh]--

*Mme. 2:* [Oh là là]! Mais sûr, mais quand c'est frais en plus. Il me dis tien, j'ai acheté ça pour ça. Hier soir, on a mangé des-des asperges.

*Mme. 1:* Oui,

*Mme. 2:* Mes asperges je les aie achetés, je les ai cuisinés assez rapidement. Des salades de crudités à tous les- tous les repas.

*Mme. 2:* Oui

*Mme. 2:* Tous les midi, on mange ça de crudités donc--

*Mme. 1:* D'accord.

*Mme. 2:* Alors ça, c'est plus [euh] soupe ou pas ? Ça doit se prendre à chaud.

*Mme. 1:* Alors c'est un-c'est un gaspacho ouai.

*Mme. 2:* [inaudible 00:41:42-00:41:44]

*Mme. 1:* Ouais, vous en faites des fois, ou--

*Mme. 2:* Non j'aime pas.

*Mme. 2:* Ouai

*Mme. 2:* Non, j'aime pas.

*Mme. 1:* Ça, on va l'enlever.

*Mme. 2:* Ouai.

*Mme. 2:* J'aime bien les soupes.

*Mme. 1:* Oui elles arrivent. [Rire]

*Mme. 2:* Ouai ça c'est beau. [Ah bah oui], les tartes. [Bah oui], les desserts. [Ahhh]

*Mme. 1:* Voilà ça c'était--

*Mme. 2:* Ça, c'est bon, les michecs évidemment.

*Mme. 1:* Ouai

*Mme. 2:* Mais je ne vois pas forcément mes fraises parce que [euh] je trouve que c'est dommage--

*Mme. 1:* Ouai

*Mme. 2:* De broyer les bonnes fraises- les fraises.

*Mme. 1:* Ouais.

*Mme. 2:* Moi j'aime les fraises, c'est comme ça donc.

*Mme. 1:* [Bah oui].

*Mme. 2:* C'est mais-- bon.

*Mme. 1:* [inaudible 00:42:12-00:42:14]

*Mme. 2:* [Voilà] on va dire la banane, la- la- la banane puisque bon ça va être deux fruits évidemment.

*Mme. 2:* Ouais.

*Mme. 2:* Alors-là, ça c'est génial-- en plus avec de la grenade dedans c'est trop bon.

*Mme. 1:* Ouais.

*Mme. 2:* Souvent on fait ça pour les fêtes. C'est essentiellement de la grenade et [euh] [bah] des soupes,[quoi].

*Mme. 1:* Et des soupes [quoi].

*Mme. 2:* Tous les jours, tous les jours, tous les jours.

*Mme. 1:* Ouai l'hiver [euh]--

*Mme. 2:* Tout le temps, tout le temps, tout le temps.

*Mme. 1:* Même l'été, vous d'être assez soupe ?

*Mme. 2:* Non, je fais la Lettone aux crudités.

*Mme. 1:* Ouais.

*Mme. 2:* C'est bon les crudités, le soir c'est beaucoup de salades.

*Mme. 2:* Oui.

*Mme. 2:* Mais la soupe tout l'hiver, tout l'hiver, des soupes différentes régulièrement.

*Mme. 1:* Oui

*Mme. 2:* Et c'est vrai que j'arrive à faire. [euh] Je fais des soupes pour trois, quatres jours que je mets [euh] dans le frigo.

*Mme. 2:* Ouai.

*Mme. 2:* Je conserve et puis, [oh là là] ! Et puis on consomme et si il reste un peu le jeudi, [bah] je mets au congèle, le reste pour retrouver des carêmes entre deux soupes.

*Mme. 2:* Ouai.

*Mme. 2:* Si j'ai fait trop. Mais maintenant, avec l'appareil [inaudible 00:42:59-00:43:02] c'est plus [euh]-- j'arrive plus à modérer mes-mes repas et à les calibrer parce que [bah], j'ai quand même trois enfants et--

*Mme. 1:* Ouai

*Mme. 2:* Et on mangeait tous ensemble, tout le temps. Et les quantités sont complètement différentes.

*Mme. 1:* Ouai.

*Mme. 2:* Aujourd'hui [inaudible 00:43:13-00:43:15] [bah] c'est les mêmes quantités. J'avais tendance à faire les mêmes quantités qu'avant. Il fallait que je te réduise quand même à un petit peu tout ça.

*Mme. 1:* Ouai, il y a un temps d'adaptation. [euh]

*Mme. 2:* C'est dur, c'est pas facile, ouai, ouai. Puis-là, je deviens sage. Je commence un petit peu--je fais de la soupe pour la semaine.

*Mme. 1:* OK

*Mme. 2:* J'arrive à faire le repas pour quatre jours parce que vendredi on ne mange pas de soupe [inaudible 00:43:30:-00:43:32]

*Mme. 1:* Il y a des habitudes.

*Mme. 2:* Ouai, c'est ça, [voilà]. [rire] Et puis [voilà], donc on mange des soupes, [bah ouai], beaucoup de soupes avec tous les légumes qu'on a. Puisque-là, mon mari hier a coupé un peu de champignons qu'on avait.

*Mme. 1:* Ouai.

*Mme. 2:* Il a coupé. On s'est décidé à couper, à faire la soupe et congeler du coup, puisque pour--

*Mme. 1:* Oui pour le reste après [euh]--

*Mme. 2:* Voilà, pour le reste, on peut faire un poulet ou peu importe.

*Mme. 1:* [Mm-hmm]

*Mme. 2:* Ouai, donc tout ça, ça me parle.

*Mme. 1:* OK, alors l'idée c'est de prendre les fruits et légumes et de me dire ce que vous auriez fait ? Est-ce que ils sont plutôt-- ils vont aux chevaux ou à la poubelle ? Ou est-ce que vous les auriez cuisinés ?

*Mme. 1:* [Bah oui], oui, oui, [bah oui], oui, oui, oui, oui, [bah oui].

*Mme. 1:* Il va ou lui alors ?

*Mme. 2:* [Ah bah] il va dans une salade.

*Mme. 1:* Il va dans la salade ?

*Mme. 2:* Même si on ne peut forcément manger, si-- je ne sais pas si je verrais bien le bout abîmé ou pas. Mais moi, quand ils sont flétris, il n'y a plus rien dedans je les mange.

*Mme. 1:* Ouais.

*Mme. 2:* Je les coupe en rondelles, je vais les mettre dans une salade d'œufs ou de machins.

*Mme. 1:* [Alors up], on peut mettre--

*Mme. 2:* Ouai, en salade, ouai.

*Mme. 1:* En salade.

*Mme. 2:* [Bah oui bah] Et [euh] pourquoi on n'en mangerait pas ? Ça a aidé des gens qui n'ont pas à manger.

*Mme. 1:* Apparemment.

*Mme. 2:* [Bah] moi, c'est super tomate quoi.

*Mme. 1:* Oui.

*Mme. 2:* [Ahhh] Je trouve ça, voilà assez [euh]-- alors ça, ça peut être une salade de fruits ou une sauce ou--

*Mme. 1:* Ouai.

*Mme. 2:* Moi j'ai ma salade de fruits, je coupe le bout qui manque.

*Mme. 1:* Ouai, vous enlèverez vraiment.

*Mme. 1:* Ouais. Là, [bah], l'orange pareille.

*Mme. 1:* Oui.

*Mme. 2:* Machin, [eh bah] là c'est pareil Michec avec.

*Mme. 1:* Vous enlevez le noir.

*Mme. 2:* J'enlève juste le noir et encore pas tous et ça passe très bien dans le michec. Alors les poivrons, ça dépend. [Bah] là, c'est pareil si [inaudible 00:45:43-00:45:06]

*Mme. 1:* Oui.

*Mme. 2:* Le gros, moi je pense qu'il va aller aux chevaux plus tôt [hen], parce qu'il est bien flétri.

*Mme. 1:* Ouai.

*Mme. 2:* Je pense que pour moi, il aurait été au congèle avant. Je pense que je l'aurais mis au congèle avant qu'il soit trop flétri, juste avec le morceau voilà quoi. [Bah] là c'est pareil, c'est le bout, je pense, qui était abîmé de-de la courgette. Je pense que le reste a été mangé.

*Mme. 1:* Ouai, c'est possible.

*Mme. 1:* Pour vous ici si on voit que c'est pas beau, c'est pas-- bon c'est abimer quoi.

*Mme. 1:* Donc ça, les chevaux les consomment ?

*Mme. 2:* Les chevaux les consomment.

*Mme. 1:* Ouai.

*Mme. 2:* Ça, c'est mon mari. [Alors là] Tous même si c'est abîmé, [pup] il coupe les bouts et up elle s'est compote, il en fait--

*Mme. 1:* [Rire]

*Mme. 2:* Ah bah oui, moi je mange ça. [hen]

*Mme. 1:* Oui.

*Mme. 1:* Je coupe juste ça.

*Mme. 1:* Ouai.

*Mme. 1:* C'est prêt et je vais manger [euh], je vais nettoyer toute ma salade à chaque fois que je fais de la salade, même s'il y a des petits bouts abimés. [euh]

*Mme. 1:* Ouai.

*Mme. 2:* Ça, je mange. Alors là, la pauvre carotte il faut être honnête.

*Mme. 1:* [Rire]

*Mme. 2:* Je ne sais même pas si ça peut s'éplucher, ça.

*Mme. 1:* Ouai.

*Mme. 2:* Tellement c'est flétri donc [euh] [inaudible 00:45:54-00:45:55]. Là c'est pas flétri, donc je pense que en coupant bien les bouts, je pense qu'il y a toujours moyen de faire une soupe ou quelque choses avec.

*Mme. 1:* D'accord.

*Mme. 2:* Ça, c'est pareil. Les bouts verts, on peu faire de la soupe avec. pas-pas le reste, on va enlever le reste.

*Mme. 1:* Oui là pour vous, il y a des parties abimées sur le vert, mais-mais--

*Mme. 2:* Oui, mais le reste oui, quoi. Oui c'est toujours [euh], on met une soupe-- on fait une soupe avec ou même [euh] on se servirait de condiments pour faire une soupe comme un oignon, quoi, [voilà] ! Après [euh]. [Voilà] elles ont payé-- elles ont pris chère les anguilles, je pense.

*Mme. 2:* Ouai.

*Mme. 2:* Mais sachant que si on les nettoie cœur, ou peut-être que si elles sont comme ça partout, non, ça va aux chevaux.

*Mme. 2:* Ouais.

*Mme. 2:* On va être honnête que, allez aux chevaux. Et ça, c'est la mienne ?

*Mme. 1:* Oui. [rire]

*Mme. 2:* Ça, c'est la mienne, qu'elle est tout pourrit.

*Mme. 1:* Ouais [inaudible 00:46:35-0046:37].

*Mme. 2:* Et celle-ci, je l'avais [euh] achetée au supermarché en fait.

*Mme. 1:* D'accord.

*Mme. 2:* J'avais acheté au supermarché, j'avais dû vous l'indiquer, j'avais pas--

*Mme. 1:* C'est possible, j'avais pas noté le commentaire.

*Mme. 2:* J'avais pris ça dans une foire à la tomate ou un truc comme ça.

*Mme. 1:* D'accord.

*Mme. 2:* J'avais pris ça, mais à ceci, j'avais pas-- elles étaient toutes belles.

*Mme. 1:* Ouai.

*Mme. 2:* Et j'ai ouvert le sachet, c'était [euh] [bah] ça déole [hen], c'était machin, mais j'avais été arti- attiré. J'avais dit, tiens, ils font de la tomate, ça va être frais. Tiens, c'est tout sympa.

*Mme. 1:* [Rire]

*Mme. 2:* Tomates au machin, [bah voilà]. Cocktail, [bah voilà].

*Mme. 1:* Et au final [euh] ?

*Mme. 2:* [Et bah] elle a fini [euh]--

*Mme. 1:* Pour les cheveux.

*Mme. 2:* [Eh bah oui], elle a fini, [voilà]. [Voilà], j'avais mis un bout, au congèle un bout. Un bout [euh] comme on dit pour les chevaux. [Rire]

*Mme. 1:* Ça, ils se régalent pas mal. [Rire]

*Mme. 2:* [Ah bah] oui, ils mangent oui, oui. Mais pas beaucoup [hen] parce que j'arrive toujours à--

*Mme. 1:* Oui normalement, vous arrivez à sauver qu'à même--

*Mme. 2:* Oui, oui à sauver beaucoup de choses, ouais. Si j'attends-j'attends pas qu'ils soient abimés. Si ça commence à s'abimer, c'est au congèle [hen]

*Mme. 1:* Oui, oui, c'est ça, vous avez votre méthode, finalement [euh]--

*Mme. 2:* C'est ça, ouais, ouai.

*Mme. 1:* Tourne-- quand on vous écoute, qui-- c'est que vous congelez régulièrement quand vous voyez que vous allez pas le manger là ou frais [euh]

*Mme. 2:* Je trouve que ouais. Parce que même les poireaux, même si on n'y fait rien, on coupe ça. J'ai un petit- des petits sachets, on met dans le congèle et je m'en sers pour autre chose.

*Mme. 1:* Oui.

*Mme. 1:* Dans mon congèle, on trouve j'ai des petits sachets d'un peu de tout, mais je trouve toujours l'utilité de les mettre.

*Mme. 1:* Ouais.

*Mme. 2:* De les mettre après [euh] [bah] comme là où c'est différent. [euh] On mange moins différents. Là c'est-c'est joli, non ? Je trouve ça chouette comme concombre à manger.

*Mme. 1:* Oui ils ont-- oui là c'est--

*Mme. 2:* Ouais, voilà.

*Mme. 1:* Il y a pas mal de gens qui diraient, bah j'adorerais faire. [rire]

*Mme. 2:* Oui, c'est ça, ouais, ouais, ouais.

*Mme. 1:* [Rire]

*Mme. 2:* Moi, c'est moi- moi j'ai un congèle avec tiroirs [euh] et l'autre est là. J'ai cassé les tiroirs parce que ça m'énerve. J'ai mis des étagères c'est un peu bien. Ils sont vieux, mais ils marchent bien.

*Mme. 1:* Oui.

*Mme. 2:* Je le garde. Donc voilà, mais je fais plein de trucs [euh] ouais, différents. Avec une poêlée de légumes, j'aurais tendance à faire plus avec des produits très frais.

*Mme. 1:* Oui.

*Mme. 2:* Quand j'ai des poêlées de légumes, plutôt que de les congeler par exemple.

*Mme. 1:* D'accord.

*Mme. 2:* Ça me prend quand même beau- beaucoup d'eau. Donc si je prends une poêlée, ça, si on congèle ça- ça, ça va prendre de l'eau, [quoi].

*Mme. 1:* Oui.

*Mme. 2:* Évidemment, je vais privilégier les légumes frais quoi.

*Mme. 1:* D'accord.

*Mme. 2:* Je ne vais pas forcément tout le temps. [euh]

*Mme. 1:* Qu'est-ce que vous faites avec les--

*Mme. 2:* Les poêlées, on n'en mange pas beaucoup l'hiver.

*Mme. 1:* D'accord.

*Mme. 2:* On va manger beaucoup de poêlés plus au printemps.

*Mme. 2:* D'accord.

*Mme. 2:* Donc c'est les saisons des-- surtout [euh] pour [bah] les radis. On mange beaucoup de radis noirs en poêlées, des concombres, des choses comme ça.

*Mme. 1:* Ouais courgettes, carriers [euh].

*Mme. 2:* Les courgettes, voilà ne sont plus là, mais en hiver, moins.

*Mme. 1:* Ouais.

*Mme. 2:* On est plus casanier, plus sur des-- on mange beaucoup de choux, beaucoup, beaucoup de choux.

*Mme. 1:* Ouais.

*Mme. 2:* Choux rouge, choux de Bruxelles, chou blanc.

*Mme. 1:* Ouais.

*Mme. 2:* [euh] [Bah], Tous les choux [hen] moi je mange tous les choux. Et là, j'ai acheté moi le brocoli, le romanesco, tous ces choux-là, on mange beaucoup de choux. [inaudible 00:49:03-00:49:05] on mange des choux. [Rire] voilà. Donc c'est ça, surtout. Là, après l'automne, beaucoup de champignons on à manger. [euh]

*Mme. 1:* Ouais.

*Mme. 2:* Donc beaucoup de champignons, on a mangé. J'ét-- c'est des chara-- c'est des châtaignes. Je les ai nettoyés, je les ai congelés. Il m'en reste pour tout l'hiver.

*Mme. 1:* D'accord.

*Mme. 2:* [Bah] j'ai fait [euh] bah, j'ai fait beaucoup ça. J'ai congelé beaucoup [hen]. Le châtaignier, oui, je jette pas [hein] en fait, on n'achète pas. A Noël, on a farci avec les châtaignes [euh].

*Mme. 1:* Oui.

*Mme. 2:* Ils vont déjà épluché au congèle [hen]. Mais ouais, c'est vrai, nous, on adore. Là j'ai [henn] fait ça cette semaine bah là [euh] hier, on l'a mangé en croûte. J'ai mis une pâte feuilletée dessus.

*Mme. 1:* D'accord.

*Mme. 2:* Ça a fait le repas du soir [hen].

*Mme. 1:* Ouais.

*Mme. 2:* Un petit peu de lardons [euh], un truc comme ça et ce soir, c'est des châtaignes.

*Mme. 1:* [Mm-hmm]

*Mme. 2:* [inaudible 00:49:46-00:49:48]. Non non, on arrive, mais moi je sais ça, je-j'arriverai pas-- je me dis [voilà], il y a des choses-- même là, [hen] là, franchement, en coupant les bouts, on arrive toujours à manger. [euh]

*Mme. 1:* Oui.

*Mme. 2:* À manger les-les petits morceaux, [quoi]. [Mais bon], ça peut arriver, voilà qu'il y ait des produits, mais ça, ça, moi, je ne jette pas ça. Pour moi, c'est un bon produit ça.

*Mme. 1:* Oui.

*Mme. 2:* C'est un bon produit, une salade, là, ça s'est mis un peu, toujours une salade. [hen] Donc 'est fragile [hen] ! Donc, il faut couper. Celle du Jardin sont les mêmes-les mêmes des limaces.

*Mme. 1:* Oui.

*Mme. 2:* Donc je- j'épluche tous les trois limaces en plus, et encore pas toujours, souvent la salade quoi. Quand j'ai du monde, je fais attention.

*Mme. 1:* [Rire]

*Mme. 2:* Elle est lavée, elle est propre, mais il y a des trous de limaces dedans.

*Mme. 1:* [Bah oui], c'est-- on ne peut pas trop y échapper.

*Mme. 1:* C'est ça, donc [euh], [bah voilà].

*Mme. 1:* C'est une gage de-- naturel. [ire]

*Mme. 2:* Je sais que je sais que c'est-- [voilà] je sais que c'est les miennes, que-comment je-- ils ont été faites et [voilà]-- et élevées, puis [voilà].

*Mme. 1:* Oui.

*Mme. 2:* [Voilà, voilà]. [inaudible 00:50:39-00:50:49]

*Mme. 1:* Oui.

*Mme. 2:* [Bah] les fruits [euh] entre deux saisons on arrive à--

*Mme. 1:* Oui.

*Mme. 2:* On commence à mettre des fruits dedans, à Noel, on aime bien, on aime bien [euh]

*Mme. 1:* Faire des salades [euh]--

*Mme. 2:* Des salades, des jus. On fait beaucoup de jus de fruits aussi. [hen]

*Mme. 1:* Ouais.

*Mme. 2:* [Bah] là [voilà] les pommes ont certes flétrie, mais j'ai fait beaucoup de fruits-de jus de fruits, [bah] dans l'extracteur [hen], donc--

*Mme. 1:* D'accord. [Ah oui] et ça vous donne du jus ?

*Mme. 2:* [Bah ouai], c'est super bien.

*Mme. 1:* Ouais.

*Mme. 2:* Le jus de pomme, j'en ai fait beaucoup. [hen] J'ai fait pomme, j'ai fait poire, j'ai fait pomme. J'ai fait pommes, poires, j'ai fait les pommes, kiwi, j'ai fait pomme-paume, comment ça s'appelle ? Comment elle s'appelle, la papaye?

*Mme. 1:* Ouai.

*Mme. 2:* [Voilà] des choses comme ça que je fais, je change, on fait plein de jus de fruits quand-quand j'ai des-des repas des choses comme ça. [inaudible 00:51:28-00:51:33] Donc on leur fait les cocktails. [euh]

*Mme. 2:* [Bah oui].

*Mme. 1:* On fait des cocktails, ça plait toujours. [euh]

*Mme. 1:* [Bah oui], oui, j'imagine.

*Mme. 2:* On les fait frais. J'ai stérilisé un peu aussi pour faire un peu de jus de pomme, mais ça demande beaucoup de boulot en fait de stériliser. [euh]

*Mme. 1:* Ouais.

*Mme. 2:* J'ai pas d'appareils donc [euh], je stérilise quatre bouteilles et puis cinq bouteilles, et puis voilà, j'y arrive après [euh]--.

*Mme. 1:* Ouais.

*Mme. 2:* Je ne fais pas plus, quoi.

*Mme. 1:* C'est vrai, que c'est-- en fait, quand on a un peu-- on a les idées qu'on prend le- qu'on a le temps aussi, et les appareils aussi. [euh]

*Mme. 2:* Par exemple j'ai essayé fruits et légumes j'aime pas.

*Mme. 1:* D'accord.

*Mme. 1:* Non, j'ai essayé, mais--

*Mme. 1:* Ça ne marche pas avec [oh là là] !

*Mme. 2:* En fait j'ai essayé, ce qu'il a fait et j'adore le jus de carottes avec l'orange. J'ai essayé du jus de carotte. En fait, j'en extrais jamais assez de jus.

*Mme. 1:* [Ah] !

*Mme. 2:* Il faut trois kilos de carottes pour [euh]--

*Mme. 1:* Ça ne m'étonne pas trop. Je savais que c'est pas très juteux, en fin la carotte. Donc je me demande comment ils font.

*Mme. 2:* [Bah] je ne sais pas. [hein] Il faut des tonnes de carottes pour faire un jus de fruits, [quoi].

*Mme. 1:* Ouais.

*Mme. 2:* J'ai eu à essayer, ça fait beau-- on a essayé [euh].

*Mme. 1:* Le jus de tomate aussi je crois que ça fait--

*Mme. 2:* Moi, j'aime pas.

*Mme. 1:* [Ah Oui] ?

*Mme. 2:* Pour moi c'est comme les cageots de choux. [hen] J'aime pas les jus de tomate.

*Mme. 1:* Ouais.

*Mme. 2:* [Ah] c'est-- non, non ça va.

*Mme. 1:* [Rire]

*Mme. 2:* [Et voilà].

*Mme. 1:* [Rire]

*Mme. 2:* Donc [euh] je mélange pas trop de fruits. [euh] Ouais les concombres, j'aime assez. Je mange bon bon, donc ça va.

*Mme. 1:* Ouais, le concombre avec le gaspacho. [euh]

*Mme. 2:* Ouai ça, c'est--[voilà] plus tôt celui-ci, il est juteux. Mais-mais le fruit, je le fais.

*Mme. 1:* Ouais.

*Mme. 2:* Les pêches, [bah] je fais pas. [Oh] les pêches, on en avait marre à un moment.

*Mme. 1:* Les jus de pêches ? [Ah oui] ?

*Mme. 2:* [Bah] ouais, on en avait marre. Avec l'extracteur, c'est super bien.

*Mme. 1:* Oui.

*Mme. 1:* Ça va vite. [euh]

*Mme. 2:* Avec l'extracteur, c'est chouette. [hen] Bon, il faut le sortir, et puis il faut-il faut se dire, tien, je vais faire trois, quatre bouteilles aujourd'hui, mais c'est simple. J'aime bien ces mélanges puisque extracteurs, ça fait pas-- [bah] ça grogne.

*Mme. 1:* Ouai.

*Mme. 2:* Donc j'ai un pré, ça grogne pour faire les deux, pour faire des mélanges orange pomme qui est sympa. Si-si c'est banane, je fais-- puis banane, pomme. Donc plein de choses, plein de choses.

*Mme. 1:* [Alors] là c'est gentil vous-- il n'y avait pas que la--

*Mme. 2:* Non, il y avait une petite chose, ouais.

*Mme. 1:* Ouais je l'ai dit ça, ça--

*Mme. 1:* Je bricole par-ci-par-là.

*Mme. 1:* Est-ce que--non c'est pas celle-là. Ça aurait pu s'assembler avec des tomates ?

*Mme. 1:* Ouais, oui [bah oui], c'est les tomates les plus compliquées du lot, [voilà]. Quand c'est celle du jardin, c'est moins compliqué. Surtout celles du-- celle de-- ouais, de jardin, elles ont quand même tendance à être moins pourries quoi.

*Mme. 1:* Ouais.

*Mme. 2:* Mais après, je pense que c'est aussi ça la qualité des fruits et des-des légumes, donc.

*Mme. 1:* Donc pour vous le-- ça--

*Mme. 2:* La qualité et la-la-la qualité et la constance en achat--

*Mme. 1:* Ouai.

*Mme. 2:* Des fruits et légumes, c'est aussi les acheter [euh]-- c'est aussi ne pas acheter trop de fruits et légumes.

*Mme. 1:* Oui, il y a une.

*Mme. 2:* Ouais, mais voilà, il faut faire [euh]-- j'ai un petit monsieur qui est client de mon mari. Il lui emmène régulièrement des sachets et le-- chez Lidl, il va chercher les invendus en fait.

*Mme. 1:* D'accord.

*Mme. 2:* Et puis, [bah] voil-[euh]-- ça a l'air de lui faire plaisir, puisqu'il ramène toujours à mon mari [euh] les petits sachets de pistaches, d'épinards. Ça, bon on accepte, on n'est pas, [voilà].

*Mme. 1:* [Rire]

*Mme. 2:* Mais je le fais [euh], je fais vite, vite, vite. Je mets vite au congèle, vite, vite, vite.

*Mme. 1:* Oui.

*Mme. 2:* [Bah], il y a des choses que j'avoue que j'ai pas pu consommer. [hen]

*Mme. 2:* Oui.

*Mme. 2:* Tu tries les choses comme ça qui étaient blettes, [quoi].

*Mme. 1:* Oui, oui il y a des fois.

*Mme. 2:* Mais [voilà], c'était eux, c'était-- moi j'aurais jamais acheté ça, moi.

*Mme. 1:* Oui, mais des fois ça- ça fait partie qu'à même.

*Mme. 2:* Ça leur fait plaisir, ils ont l'air d'être contents, mais c'est juste comme ça, on accepte.

*Mme. 1:* [Bah oui].

*Mme. 1:* Pour leur faire plaisir.

*Mme. 1:* [Et du coup], ça peut entraîner des pertes chez vous après [quoi].

*Mme. 2:* C'est ça, voilà, chez moi, voilà. Mais bon, très peu, mais-- en fin, c'est pour ça que je dis à mon mari souvent : [Ah bah] tu-- donnes aux autres, [quoi]. Donne aux autres.

*Mme. 1:* [Rire] On transmet aux chevaux [quoi].

*Mme. 1:* Ouais, ouais, ouais, moi c'est bon [hein].

*Mme. 2:* [Voilà], un bout de tomate.

*Mme. 1:* Oui.

*Mme. 2:* Parce que l'autre bout, j'ai dû le manger. [Rire] Ça, c'est sûr, oui.

*Mme. 1:* Ça, oui.

*Mme. 2:* Il y'a un bout pourri de l'autre côté [bah] parce que--

*Mme. 1:* C'était bien. [Sonnerie du téléphone] Allez-y.

*Mme. 2:* Oui, excusez pardon, un instant.

*Mme. 2:* C'est mon fils.

*Mme. 2:* Oui, Corentin. Qu'est-ce qui t'arrive ? [Ah bon], pourquoi ? tu viens ? [Ah bah], d'accord. Ouai, je viens te chercher tout à l'heure ? Si tu-- Ouai, ouai, pas de soucis Patric. [Oh] pas de soucis. Ouai,ouai, nickel, tu es adorable. Ouai, ouai,ouai, [Hmm]. Ouai, ouai,ouai, Rendez-vous comme la dernière fois chez Patricia. [Hmm] Ouai, ouai, ouai. Non mais pas de soucis, tu m'appele quand t'a fini. OK. C'est toujours pareil, ça va ? Pas d'autres-pas d'autres contacts aujourd'hui ? [Bon, bon] c'est bien [Rire]. [bon bah], écoute, pas de soucis. Tout à l'heure [alors]. [hein].

*Mr 1:* Ouai, tout à l'heure.

*Mme. 2:* Bisou !

*Mr 1:* [Rire]

*Mme. 2:* Quand, il est sexy, alors il l'a reconnu, ouai.

*Mme. 2:* Ouai [rire]

*Mme. 2:* Son copain [hein] son copain, son frère qui avait conquis-- qui avait le Covid aussi, ils vont au sport, après le soir, il me dit : " moi, j'y vais pas ou j'y vais tout seul. " Un cauchemar ouai, ça va, ouai.

*Mme. 1:* [Rire]

*Mme. 2:* Ça s'est compliqué, ça change notre vie.

*Mme. 1:* Ouai, c'est clair.

*Mme. 2:* Oui, donc la tomate, ouai, c'était ça. Mais je pense que moi c'est des problèmes de tomates, que j'ai.

*Mme. 1:* Problème de tomate. [rire]

*Mme. 1:* Ouais, problème de tomates.

*Mme. 2:* Ouai, ouai.

*Mme. 1:* [En bah], Elle venait d'où, celle-là, vous disiez ?

*Mme. 2:* Celle-là, mais ça, je crois que je sais plus [hein]

*Mme. 1:* [Alors] ça, c'était--

*Mme. 2:* Je ne sais plus [hein], celle-ci c'était ça je me rappelle. L'autre, je sais plus. [Ah] c'est bizarre. [hen]

*Mme. 1:* Oui.

*Mme. 2:* Je me rappelle plus. J'avais dû le mettre, j'avais dû le rajouter pour-- en mémoire puisque je mettais. [euh]

*Mme. 1:* C'est possible que vous m'ayez mis des commentaires.

*Mme. 2:* Je le mettais en mémoire. En général pour-- d'où ça vient ?

*Mme. 1:* Chez le primeur Moisson, effectivement.

*Mme. 2:* Ouai, c'est ça, ouai. Alors j'avais cette tomate-là. Moisson, pourquoi Moisson ? Pourquoi j'ai mis Moisson ? Chez le primeur Moisson. Moisi. Et ça, oui je ne savais pas [rire].

*Mme. 1:* Si, si c'était le nom du primeur.

*Mme. 1:* Non, non, [bah voilà] c'était moisi. [Voilà, Voilà], acheté en grappes, moisissures apparentes. Alors celle-là, ouai la tomate, ouai, c'était [euh],-- je ne l'avais pas vue. C'est en la prenant dans le machin avec-- le sachet tête dessus, donc on voyait pas.

*Mme. 1:* Et les autres, elles étaient correctes ? [euh]

*Mme. 2:* Et ouais, les autres, tous les autres, ouai nickel. [Ahh] ouai, ouai, celle-ci elle était saoule, elle a dû prendre un peu d'eau, un petit peu de pourriture et puis [voilà, quoi].

*Mme. 1:* Ouais.

*Mme. 2:* Et puis après, je les mange pas tous ensemble.

*Mme. 1:* Ouai.

*Mme. 2:* J'en prenais une ou deux pour mettre dans la salade. La tomate, c'est pareil, c'est peut-être moi qui les gardais aussi un peu trop longtemps.

*Mme. 1:* Ouais.

*Mme. 2:* Donc [bah], après j'ai mangé tout ce qui n'était pourri, je sais- j'ai-- en fait, je coupe jusqu'à ce je vois le truc là, pourri, mais j'arrête.

*Mme. 1:* Oui.

*Mme. 2:* On a mangé. Et puis là, c'était un navet, pareil.

*Mme. 1:* [Alors] oui.

*Mme. 2:* [Alors], le navet--

*Mme. 1:* C'est quoi l'histoire de ce navet ? [rire]

*Mme. 2:* En fait, l'histoire du navet c'est votre collègue qui est venu.

*Mme. 1:* Oui.

*Mme. 2:* Faire des-cherchez, ses machins, tout ça.

*Mme. 1:* Des prélèvements.

*Mme. 2:* Il y avait un petit navet pourri qui m'avait échappé. Un tout petit, je sais pas d'où il sortait. [rire] Non, non j'avais acheté des navets pour faire un couscous. Et je pense que j'en ai oublié un petit dans le-- qui s'est glissé dans le panier et on ne l'a pas vu. Donc on l'a retrouvé le petit-le petit navet.

*Mme. 1:* Il était caché [quoi] ?

*Mme. 2:* Il était caché, mais en général ça c'est des produits, je n'achète pas en grosses quantités non plus. Je vais faire un couscous, j'achète juste le nécessaire pour faire le couscous. Je- je vais pas acheter en plus deux ou trois navets pour rien.

*Mme. 1:* Oui, il y a des fois, quand vous avez prévu un repas spécifique, vous avez acheté la quantité pour un repas [hein].

*Mme. 2:* Oui, des bons plats. Ouai, ouai, ouai, je-- essentiellement. Oui, oui, j'ai le produit de base, mais après le reste [voilà]. [Hmm], tout à fait.

*Mme. 1:* [Bon bah] ça marche. [Bah] merci beaucoup. J'ai passé toutes mes questions.

*Mme. 2:* [Rire] C'est essentiel.

**Household F07, Interview 1**

00:00:00
*Speaker 1:* Qui va m'aider à retranscrire ? Un, deux, 18. Alors est-ce que vous êtes prêts ?

00:00:29
*Speaker 2:* Oui, c'est bon.

00:00:31
*Speaker 1:* Pour commencer, je propose un petit jeu de devinette, je vais vous demandez de penser à un fruit ou à un légume que vous aimez tout particulièrement. L'idée, si vous avez pensé à ce fruit, ce légume sans me dire son identité, je vais devoir le faire uniquement grâce à la description.

00:00:49
*Speaker 2:* Grâce à ma description.

00:00:50
*Speaker 1:* Ouais vous allez me dire tout ce qui vous passe par la tête sur ce fruit ou ce légume. Et puis, au bout d'un moment, je vais tenter de deviner.

00:00:59
*Speaker 2:* [Euh] Rouge.

00:01:00
*Speaker 1:* Ok.

00:01:06
*Speaker 2:* [Euh] Fruits.

00:01:06
*Speaker 1:* Ouais.

00:01:12
*Speaker 2:* [Euh] Noyau.

00:01:13
*Speaker 1:* Oui.

00:01:17
*Speaker 2:* [Euh] Été.

00:01:18
*Speaker 1:* Hmm.

00:01:22
*Speaker 2:* [Euh] Charnu, [euh] sucré.

00:01:30
*Speaker 1:* Ouais.

00:01:39
*Speaker 2:* Queue.

00:01:41
*Speaker 1:* Ouais.

00:01:41
*Speaker 2:* [Rire]

00:01:44
*Speaker 1:* Ça se concrétise. [Rire]

00:01:47
*Speaker 2:* Il en faut d'autres ? [Rire] ?

00:01:49
*Speaker 1:* Qu'est-ce que vous aimez particulièrement dans ce fruit ?

00:01:55
*Speaker 2:* Hors la saisonnalité ?

00:02:00
*Speaker 1:* Hmm.

00:02:00
*Speaker 2:* Vue que c'est [inaudible 00:02:02-00:02:02] depuis que je viens d'apprendre que-- et qu'on est loin qu'on est en plein dedans.

00:02:11
*Speaker 1:* Ok. En général, vous le mangez quand ? C'est plutôt le matin, plutôt le midi, l'après-midi, n'importe quand, le soir ?

00:02:23
*Speaker 2:* Midi et soir.

00:02:24
*Speaker 1:* Ouais. Est-ce que vous pouvez me raconter les différentes étapes qu'il y a avant que vous puissiez le consommer, lié à votre fruit, qu'est-ce que vous faites avant de le mettre dans votre bouche ? S'il y a des étapes.

00:02:39
*Speaker 2:* Je vais l'acheter.

00:02:39
*Speaker 1:* Ouais.

00:02:40
*Speaker 2:* Ça enfin ouais, avant enfin du début de l'achat ou du producteur ?

00:02:47
*Speaker 1:* De l'achat jusqu'à ce que vous vous soyez avec.

00:02:52
*Speaker 2:* [euh] Acheter, le laver souvent avant, en rentrant et [euh] et- et le manger. Sans oui, on serait pour la cerise, on fait pas de préparation, [oh] pardon. [rire]

00:03:15
*Speaker 1:* Je l'avais, je l'avais. [rire] En général vous l'achetez où cette cerise, ces cerises ?

00:03:25
*Speaker 2:* [euh] Soit en grande surface, soit primeur, soit magasin de producteurs.

00:03:32
*Speaker 1:* D'accord. [inaudible 00:03:37-00:03:37] Alors [euh] vous avez dit que vous aimez bien la saisonnalité, puis certains fruits sucrés, est-ce que vous trouvez d'autres avantages à ce fruit ? Est-ce que vous arrivez à dire ce que vous ressentez quand vous mangez [rire] et boire ?

00:03:56
*Speaker 2:* Oui ça donne envie. [Euh] Bon le croquant.

00:04:09
*Speaker 1:* Ouais.

00:04:12
*Speaker 2:* Le croquant, la facilité de- de le manger aussi.

00:04:19
*Speaker 1:* Ouais.

00:04:19
*Speaker 2:* C'est ça s'embarque partout vous avez pas besoin de- de le découper, de le-- voilà, il se mange comme ça quoi.

00:04:28
*Speaker 1:* Ouais, ok. Et à l'inverse est-ce que vous lui trouvé certains inconvénients ou pas ce fruit ?

00:04:35
*Speaker 2:* [euh] [bah] Particulièrement cette année, son prix. [rire]

00:04:39
*Speaker 1:* [rire] Tout le monde m'en parle du prix des cerises.

00:04:43
*Speaker 2:* Voilà c'est pas cher, mais enfin pas trop.

00:04:45
*Speaker 1:* Ouais.

00:04:45
*Speaker 2:* Neuf euros [inaudible 00:04:45-00:04:52]

00:04:47
*Speaker 1:* Hmm

00:04:54
*Speaker 2:* Le prix [euh] la rapidité de péremption.

00:05:01
*Speaker 1:* Ouais.

00:05:05
*Speaker 2:* Je sais pas comment [inaudible 00:05:05-00:05:07]

00:05:08
*Speaker 1:* Oui, si ce que je veux dire c'est que si on parle de conservation, c'est dur.

00:05:12
*Speaker 2:* C'est dur de le conserver ouais.

00:05:16
*Speaker 1:* Ce qui implique de le manger [euh]

00:05:18
*Speaker 2:* quand on l'achète, un peu de temps après l'achat.

00:05:22
*Speaker 1:* Ouais. On le [inaudible 00:05:23-00:05:24] du coup. [rire] C'est bon, c'est la cerise. Ok, alors là, je voudrais qu'on discute de vos pratiques d'achat et de votre organisation générale au sein de votre foyer qui est amené à faire des courses alimentaires.

00:05:43
*Speaker 2:* Moi et ma femme.

00:05:46
*Speaker 1:* Ouais, et [euh] est-ce que vous pouvez me raconter comment vous vous organisez sur ces achats alimentaires ?

00:05:56
*Speaker 2:* On n'est pas très bien organisé.

00:05:57
*Speaker 1:* D'accord.

00:06:04
*Speaker 2:* [Euh] on n'a pas de jours fixes ou de voilà c'est quand il manque des choses et qu'on essaye d'anticiper nos repas.

00:06:11
*Speaker 1:* Ouais

00:06:13
*Speaker 2:* Au plutôt en fin de semaine. En semaine, s'il y a besoin, c'est juste de l'appoint ou du drive.

00:06:25
*Speaker 1:* D'accord.

00:06:27
*Speaker 2:* Et- et suivant les disponibilités, on a souvent nos dispo voilà.

00:06:40
*Speaker 1:* Et du coup, vous faites en fin de semaine, c'est les gros enfin c'est les grosses courses et en semaine, c'est plus des--

00:06:48
*Speaker 2:* Ouais.

00:06:49
*Speaker 1:* Et en termes de fréquence, même s'il n'y a pas de jours [euh] fixe, est-ce que vous sauriez dire à peu près combien de fois [euh].

00:06:57
*Speaker 2:* On va consommer on va aller acheter fruits et légumes ?

00:07:00
*Speaker 1:* Ouais ouais.

00:07:04
*Speaker 2:* Dans la semaine [euh] face voyage, je pense facilement trois fois.

00:07:10
*Speaker 1:* Trois fois de la semaine ?

00:07:11
*Speaker 2:* Ouais je pense. Oui, que ce soit juste un petit truc à un moment, mais [euh] sinon je pense qu'on n'est plus autour de trois que de deux.

00:07:26
*Speaker 1:* D'accord et [euh] donc c'est une anti vous avez parlé d'anticipation des repas, qu'il manquait quelque chose, ce que vous avez mis, c'était avant de partir ou pas ? Comment ouais ? [rire]

00:07:37
*Speaker 2:* Oui. [euh] Oui, oui oui on a une liste du coup.

00:07:41
*Speaker 1:* D'accord.

00:07:46
*Speaker 2:* [euh] Normalement, après j'ai toujours des achats en plus ou le truc en plus.

00:07:54
*Speaker 1:* Ouais, oui elle n'est pas fixe [euh].

00:07:55
*Speaker 2:* Non elle n'est pas.

00:07:56
*Speaker 1:* Elle n'est pas exhaustive donc [euh] et [euh] dans cette liste de courses, comment vous la comment vous l'organisé ? Est-ce que notamment sur les fruits et légumes on voit apparaître les fruits et légumes vraiment en détail ou-- ?

00:08:11
*Speaker 2:* Ah non.

00:08:11
*Speaker 1:* Non.

00:08:18
*Speaker 2:* Oui [rire] fruits et on voit ce qui ce qu'il y a suivant voilà. Mais si ça peut enfin ça peut arriver pour [euh] [silence 00:08:27-00:08:30] ça peut arriver si on a un dessert en particulier, des choses comme ça ou si [euh] ou si on voit qu'il y a une envie ou autre, notamment [euh] notamment pour les enfants, [euh] on a régulièrement des bananes ou ce genre de choses. Donc si on a pas on le peut noter, mais la plupart du temps, c'est plutôt fruit.

00:08:55
*Speaker 1:* Oui et les légumes c'est la même organisation ou s'est écrit légumes ? Sauf s'il y a des recettes--

00:09:02
*Speaker 2:* Sauf s'il y a des recettes oui.

00:09:06
*Speaker 1:* D'accord.

00:09:08
*Speaker 2:* D'ailleurs, je pense qu'il y a même pas écrit légumes. C'est on passe au rayon et on prend. [rire]

00:09:15
*Speaker 1:* D'accord.

00:09:17
*Speaker 2:* On prend ou [euh] on sait ce qu'il y a avant de partir on sait ce qu'il y a et [euh] et on prend ce qui manque.

00:09:27
*Speaker 1:* D'accord.

00:09:29
*Speaker 2:* Si ce n'est [euh] si ce n'est les légumes [euh] carottes, pommes de terre, mais sinon le reste, peut être qu'on peut noter. Carottes, carottes ça nous arrive souvent de noter mais ce n'est pas toujours [inaudible 00:09:41-00:09:41] [rire]

00:09:41
*Speaker 1:* Pas oublier les carottes.

00:09:41
*Speaker 2:* C'est ça pas oublier les carottes parce que on a des gros mangeur de carottes. [rire]

00:09:55
*Speaker 1:* Ok et comment vous gérez les quantités que [euh] vous achetez donc [euh] les fruits et légumes ? vous êtes face à vos fruits et légumes comme vous, vous savez les quantités que vous allez--

00:10:07
*Speaker 2:* Consommer ?

00:10:12
*Speaker 1:* Oui.

00:10:12
*Speaker 2:* Ça dépend des fruits si sont enfin des ouais alors [euh] en fonction de chaque fruits ou de chaque légumes et de sa péremption.

00:10:23
*Speaker 1:* D'accord.

00:10:24
*Speaker 2:* Souvent quand-même [ah], c'est plus facile de choisir [euh] un melon. C'est un entier que- que je sais pas moi [silence 00:10:33-00:10-40] bon nombre de pommes de terre ou de trucs, c'est plus forcement c'est plus compliqué.

00:10:44
*Speaker 1:* Hmm

00:10:45
*Speaker 2:* [silence 00:10:44-00:10:48] [euh] Et sur le nombre bon à peu près sur ce qu'on consomme, enfin je pense que c'est à la louche quoi !

00:10:55
*Speaker 1:* Oui.

00:10:55
*Speaker 2:* Mais par rapport à ce qu'on consomme ou aux recettes qu'on fait avec, on a à peu près je pense le ratio.

00:11:02
*Speaker 1:* D'accord.

00:11:05
*Speaker 2:* C'est pas pesé, c'est pas au gramme prêt ou [ah] c'est [euh] c'est plus au nombre de [euh] de légumes ou de fruits que de que du poids, que du poids total.

00:11:26
*Speaker 1:* Ouais, d'accord vous savez à peu près combien vont vendre sur les fruits, combien vont être mangés dans les prochains jours ?

00:11:32
*Speaker 2:* On essaye.

00:11:35
*Speaker 1:* Vous êtes combien dans votre foyer ?

00:11:41
*Speaker 2:* Cinq.

00:11:41
*Speaker 1:* Vous êtes 5 [silence 00:11:37-00:11:40] Alors si on essaie de faire une liste des fruits et légumes que vous consommez très régulièrement, donc en commençant par les fruits ou les légumes, comme vous voulez ceux que vraiment on va trouver très souvent chez vous quoi.

00:12:03
*Speaker 2:* [euh] Fruits [euh] [silence 00:11:57-00:12:02] banane forcément, pomme, orange, bon après c'est les fruits qui sont là toute l'année, presque aussi forcément et après c'est plus de saisons [euh] en fruits [euh] melons.

00:12:06
*Speaker 1:* Oui.

00:12:24
*Speaker 2:* [euh] [silence 00:12:20-00:12:35] après, ça dépend des saisons oui. Généralement oui vraiment en fonction des saisons après.

00:12:43
*Speaker 1:* Sais pas donc y a des cerises ?

00:12:45
*Speaker 2:* Fraises,

00:12:47
*Speaker 1:* Ouais

00:12:47
*Speaker 2:* pêches, abricots, [euh] mangues, ananas,

00:12:58
*Speaker 1:* Oui.

00:13:00
*Speaker 2:* citron, poires si poires il y a souvent aussi quand-même j'aurai dû mettre dans le--

00:13:09
*Speaker 1:* Avec les pommes et les bananes ?

00:13:11
*Speaker 2:* Ouais c'est ça.

00:13:12
*Speaker 1:* Est-ce que vous avez en tête des fruits que vous connaissiez que, par contre vous allez voilà vous allez consommer que très rarement, voire jamais.

00:13:20
*Speaker 2:* Voire jamais ? [euh] Le kaki, comme ça qui me vient, [euh] pamplemousse aussi qu'on prend régulièrement enfin qui est aussi toute l'année.

00:13:39
*Speaker 1:* Ouais.

00:13:39
*Speaker 2:* [euh] Qu'est-ce que je prends quasiment jamais ? [silence 00:13:41-00:13:49] La noix de coco.

00:13:52
*Speaker 1:* Hmm.

00:14:02
*Speaker 2:* [euh] [silence 00:14:56-00:14:01]

00:14:03
*Speaker 1:* C'est pas mal et la noix de coco, pardon, pourquoi vous la consommer [euh]--

00:14:08
*Speaker 2:* Parce qu'on la prend on [silence 00:14:10-00:14:12] comment confectionner, comme on dit, élaborés enfin en- en lait de coco,

00:14:20
*Speaker 1:* Oui.

00:14:22
*Speaker 2:* ou en crème de coco.

00:14:23
*Speaker 1:* D'accord et le kaki ?

00:14:31
*Speaker 2:* Et le kaki, [euh] parce que personne n'apprécie je pense. [rire]

00:14:39
*Speaker 1:* Simplement.

00:14:42
*Speaker 2:* Pas trop.

00:14:43
*Speaker 1:* À part cela, sinon [euh] sinon la saisonnalité, vous allez pouvoir prendre pas mal de tous les fruits qui-- Et sur les légumes,

00:14:51
*Speaker 2:* Légumes

00:14:53
*Speaker 1:* quels sont les légumes que un peu les basiques ?

00:14:55
*Speaker 2:* Basiques bah carottes, pommes de terre.

00:15:00
*Speaker 1:* Ouais.

00:15:01
*Speaker 2:* [euh] Courgettes.

00:15:02
*Speaker 1:* Oui.

00:15:06
*Speaker 2:* [euh] Aubergines,

00:15:08
*Speaker 1:* Hmm.

00:15:08
*Speaker 2:* tomates, avocats, [silence 00:15:11-00:15:15] [euh] oignons

00:15:22
*Speaker 1:* Ouais.

00:15:22
*Speaker 2:* [silence 00:15:23-00:15:27] On a droit de regarder ce qu'il y a ?

00:15:32
*Speaker 1:* Oui. [rire]

00:15:32
*Speaker 2:* Avec des noix toute l'année aussi.

00:15:34
*Speaker 1:* Des noix ouais.

00:15:35
*Speaker 2:* Des fruits, oui. Fruits à coque.

00:15:38
*Speaker 1:* Ouais.

00:15:45
*Speaker 2:* [silence 00:15:39-00:15:44] [euh] Ouais les principaux, c'est ceux là quand-même.

00:15:47
*Speaker 1:* D'accord.

00:15:47
*Speaker 2:* Je pense.

00:15:49
*Speaker 1:* Est-ce que vous en avez en tête que voilà vous connaissez, mais que chez vous [euh] vous allez que très rarement le- le consommer, voire- voire jamais ?

00:16:01
*Speaker 2:* Voire jamais, [silence 00:15:57-00:16:00] [euh] les blettes,

00:16:02
*Speaker 1:* Oui.

00:16:06
*Speaker 2:* le panais,

00:16:07
*Speaker 1:* Oui.

00:16:07
*Speaker 2:* [euh] [silence 00:16:07-00:16:17] le topinambour, on avait ça un moment cherche- cherche les [rire]

00:16:31
*Speaker 1:* C'est ça.

00:16:31
*Speaker 2:* Des- des légumes qu'on mange jamais il y en a sûrement.

00:16:35
*Speaker 1:* Par exemple, est-ce que vous mangez du chou ou pas ?

00:16:42
*Speaker 2:* [euh] Du chou-fleur, oui, régulièrement. Chou vert, ça nous arrive, mais pas trop préparés. Bah des souvent préparé ou pas- pas en entier pas souvent en entier.

00:16:56
*Speaker 1:* D'accord.

00:16:58
*Speaker 2:* Choux brocolis plein.

00:17:00
*Speaker 1:* D'accord.

00:17:00
*Speaker 2:* Pas de ah choux de Bruxelles, Choux de Bruxelles--

00:17:02
*Speaker 1:* Non.

00:17:03
*Speaker 2:* Ça-ça les enfants n'aiment pas trop. Ou on ne cuisine pas bien je pense que les mauvais souvenirs d'école. [rire]

00:17:15
*Speaker 1:* Cette odeur de la quantine. Ah je vois je l'ai. Donc les enfants [inaudible 00:17:20-00:17:20] Et parlant de blettes, pourquoi [euh] vous consommez pas de blettes ? Pour les mêmes raisons ou--

00:17:31
*Speaker 2:* Ouais puis [euh] je trouve ça plus fade.

00:17:35
*Speaker 1:* Ouais.

00:17:37
*Speaker 2:* [euh] et qu'il faut des recettes, et que j'en ai pas forcément et puis que ouais et les enfants n'aiment pas non plus

00:17:43
*Speaker 1:* Oui.

00:17:43
*Speaker 2:* Donc ça veut dire qu'il faut faire deux repas.

00:17:47
*Speaker 1:* Ouais.

00:17:48
*Speaker 2:* Et bon voilà.

00:17:52
*Speaker 1:* Et le panais ?

00:17:54
*Speaker 2:* Les mêmes raisons.

00:17:55
*Speaker 1:* Ouais pareil.

00:18:02
*Speaker 2:* Chou-rave c'est compliqué aussi.

00:18:03
*Speaker 1:* Oui.

00:18:05
*Speaker 2:* Pour les enfants, pareil.

00:18:10
*Speaker 1:* [silence 00:18:06-00:18:10] Le poireau vous en consommez ou pas beaucoup ?

00:18:13
*Speaker 2:* Si.

00:18:13
*Speaker 1:* Si aussi.

00:18:14
*Speaker 2:* Mais on en n'achète pas puisqu'on en a tout le temps souvent souvent au congél.

00:18:21
*Speaker 1:* Ok.

00:18:22
*Speaker 2:* Soit du jardin, donc oui, l'hiver quoi.

00:18:27
*Speaker 1:* Ouais, vous avez un pota vous avez un potager ?

00:18:30
*Speaker 2:* Ouais.

00:18:31
*Speaker 1:* Qu'est-ce que vous cultivez dans ce potager à part les poireaux ?

00:18:36
*Speaker 2:* Alors [euh] pommes de terre. Alors, la pomme de terre, j'ai mis patate douce, petit pois, fraises, haricots verts, tomates, poivrons, concombres. Je vais trop vite ?

00:19:01
*Speaker 1:* Non [rire]

00:19:02
*Speaker 2:* [euh] Artichauts.

00:19:08
*Speaker 1:* Oui.

00:19:08
*Speaker 2:* [euh] On a fait le tour ou quoi ! ce que j'ai là, je crois.

00:19:14
*Speaker 1:* Pas mal de choses [inaudible 00:19:15-00:19:15]

00:19:15
*Speaker 2:* Ouais, et plus j'ai un deuxième producteur enfin j'ai mon père qui a fait aussi son potager et qui nous amène aussi [euh].

00:19:25
*Speaker 1:* Ok.

00:19:26
*Speaker 2:* Toute sa production [euh] sous serre.

00:19:29
*Speaker 1:* Ok.

00:19:29
*Speaker 2:* Voilà donc à partir de on va dire de [euh] avril jusqu'à octobre, c'est quasiment [euh] on a quasiment que des fruits achetés. Tous les légumes,

00:19:44
*Speaker 1:* [ah].

00:19:45
*Speaker 2:* la plupart du temps viennent de l'un ou l'autre des potagers enfin plutôt celui de mon père, mais [euh] [rire].

00:19:50
*Speaker 1:* [rire].

00:19:54
*Speaker 2:* dès qu'il vient il nous ramène, donc voilà.

00:19:55
*Speaker 1:* Ok, sinon, c'est mais après l'hiver, vous avez quand même le peut-être le poireau et tout qui--

00:20:04
*Speaker 2:* Oui, c'est ça ouais poireau pommes pommes de terre de conservation. S'il y a un peu [euh] bon il y a épinards [inaudible 00:20:13-00:20:13] épinard radi aussi.

00:20:16
*Speaker 1:* Ok dans votre potager ?

00:20:20
*Speaker 2:* Ouais ouais j'ai mis pas mal, après faut voir si,

00:20:26
*Speaker 1:* [rire] Si ça pousse.

00:20:26
*Speaker 2:* Si ça a poussé.

00:20:26
*Speaker 1:* C'est ça et du coup, donc quand c'est pas dans votre potager, vous avez dit sur les cerises que vous pouvez les acheter en grande surface, chez primeur et chez mon magasin au magasin de producteurs. Est-ce que vous pouvez me raconter un peu les,

00:20:38
*Speaker 2:* [diaphonie 00:20:39-00:20:41]

00:20:43
*Speaker 1:* Pourquoi vous choisissez tel ou tel endroit pour tel ou tel produit ? comment vous-- ?

00:20:51
*Speaker 2:* [euh] C'est d'abord en fonction de notre temps et notre disponibilité, je pense. Et que quand on veut aller vite, on fait l'essentiel et on fait les grandes courses Leclerc et on fait pour toute la pour tous les besoins alimentaires ou non alimentaires.

00:21:10
*Speaker 1:* Oui. alimentaires

00:21:14
*Speaker 2:* Et après qu'on prend plus spécifiquement des fruits ou des légumes, ouais c'est soit le primeur [euh] sur Landerneau, parce que [euh] parce que parce qu'on aime bien les ces fruits là enfin ce qu'ils proposent en général.

00:21:34
*Speaker 2:* Aux magasins de producteurs [euh] parce que bah parce que c'est le plus souvent local et[euh] et ou directement du producteur, et ça, c'est plus quand on a le temps ou qu'on se donne le temps parce que souvent, ça oblige à aller d'abord au moins enfin de faire deux magasins parce que forcément il y a le non alimentaire [inaudible 00:21:59-00:21:59] donc c'est plus question de temps que de choix, de toute façon c'est ouvert que vendredi samedi donc ça limite aussi.

00:22:07
*Speaker 1:* Dans les magasins de producteurs ?

00:22:11
*Speaker 2:* Ouais.

00:22:11
*Speaker 1:* Il est où ce magasin ?

00:22:14
*Speaker 2:* Pencran Kerbalanec c'est les fermiers du net si vous connaissez, si vous avez déja entendu.

00:22:20
*Speaker 1:* [euh] Bah moi je ne connais pas, mais il y a plusieurs personnes qui participaient à l'étude qui sont de Pencran et --

00:22:27
*Speaker 2:* Et qui vont là.

00:22:27
*Speaker 2:* Ouais.

00:22:29
*Speaker 1:* D'accord [diaphonie 00:22:29-00:22:29]

00:22:29
*Speaker 2:* Bon après ils sont le fermier du Net, c'est à Brest.

00:22:31
*Speaker 1:* D'accord.

00:22:33
*Speaker 2:* C'est leur [euh] point de vente enfin c'est leur [euh] point de vente brestois, où ils vendent leur production de de la ferme de Pencran.

00:22:42
*Speaker 1:* D'accord, donc ils ont deux--

00:22:43
*Speaker 2:* Ils ont ouais deux deux magasins et [euh] un site de production [euh] de- de viande [euh] enfin ils font abattoir enfin pas abattoir mais découpe viande porcine et bovine et après y ramènent les fruits [euh], beaucoup de fruits de bah les cerises elles viennent de Toulouse.

00:23:10
*Speaker 1:* Oui, oui, ils centralisent.

00:23:13
*Speaker 2:* Ils centralisent ouais ce qui est local et après, ils ont un ou deux producteurs du Sud.

00:23:19
*Speaker 1:* Oui ça ils ont des partenariats avec des producteurs du Sud et ils peuvent,

00:23:23
*Speaker 2:* Oui c'est ça.

00:23:24
*Speaker 1:* Du coup proposer des choses,

00:23:25
*Speaker 2:* Proposer des choses.

00:23:25
*Speaker 1:* Qui sont locales [inaudible 00:23:28-00:23:30]

00:23:32
*Speaker 2:* Non, c'est ça [rire].

00:23:38
*Speaker 1:* Ok donc c'est surtout le le temps qui va faire qui va régir un peu vos,

00:23:42
*Speaker 2:* Ouais je pense.

00:23:44
*Speaker 1:* Vos choix et pourquoi du coup, mais quand vous avez le temps, vous choisissez le le primeur et le producteur. C'est [euh] vous avez dit que c'était local et en plus, vous aimez le [euh] la qualité chez le primeur. Est-ce qu'il y a autre chose ou c'est essentiellement [euh] ça ?

00:24:01
*Speaker 2:* C'est essentiellement ça.

00:24:02
*Speaker 1:* Ouais.

00:24:02
*Speaker 2:* Ouais.

00:24:04
*Speaker 1:* Donc, c'est bon et--

00:24:06
*Speaker 2:* La variété aussi enfin la quelle va oui oui la variété [euh] [silence 00:24:08-00:24:12] la plus grande variété de choix, je pense.

00:24:15
*Speaker 1:* Ouais [silence 00:24:16-00:24:19] ok je vais atchoumer alors là si on s'intéresse à vos lieux de stockage, [euh] quand vous rentrez de vos courses, qu'est-ce que vous faites de vos fruits et qu'est-ce que vous faites de légumes ?

00:24:39
*Speaker 2:* [euh] On les range soit là dans notre zone je sais pas comment dire soit dans nos rangements à légumes, soit dans le frigo, dans le bac à frites, dans le bac à légumes, soit [euh] dans le scellier pour [euh] pour essentiellement les pommes de terre, oignons,

00:25:04
*Speaker 1:* Ouais.

00:25:08
*Speaker 2:* enfin légumes racines, on va dire voilà.

00:25:14
*Speaker 1:* Et [euh] qu'est-ce que vous allez mettre dans le frigo et pourquoi vous mettez ces produits là dans le frigo et pas [euh] dans les temperatures ambiantes [inaudible 00:25:23-00:25-25]

00:25:26
*Speaker 2:* Alors c'est une bonne question [rire] [ah] Plutôt plutôt des légumes, très rarement des fruits [toux].

00:25:38
*Speaker 1:* Oui.

00:25:38
*Speaker 2:* Qu'est-ce qu'on y met ? [euh] Concombre,

00:25:43
*Speaker 1:* Oui.

00:25:48
*Speaker 2:* poivron [silence 00:25:44-00:25:48] aubergine souvent.

00:25:50
*Speaker 1:* Oui.

00:25:57
*Speaker 2:* Radis et c'est à peu près tout.

00:25:58
*Speaker 1:* Ok.

00:26:00
*Speaker 2:* Et pourquoi Je sais pas plus [rire].

00:26:05
*Speaker 1:* C'est [euh].

00:26:07
*Speaker 2:* Par habitude [euh] sûrement. [silence 00:26:09-00:26:13]

00:26:14
*Speaker 1:* C'est bien souvent le cas.

00:26:15
*Speaker 2:* Ouais.

00:26:18
*Speaker 1:* [euh] Et du coup, les fruits sont quasiment tous [euh] ici [euh].

00:26:22
*Speaker 2:* Ouais en corbeille ou les,

00:26:24
*Speaker 1:* Oui.

00:26:27
*Speaker 2:* Dans les compartiments.

00:26:28
*Speaker 1:* Ok, ok et est-ce que ça semble, est-ce que ça vous convient comme endroit de stockage en termes de de- de de réussite de conservation ? Ou est-ce que justement, il y a des fruits et des légumes où vous dites non cela on a du mal [euh] comment vous--

00:26:50
*Speaker 2:* C'est pas, c'est un peu mieux, mais ce n'est pas idéal puisque c'est quand même bien exposé à la lumière et que, du coup, ça développe assez vite et plus que là depuis [euh] deux semaine, on a des mouches de fruits et que c'est [euh],

00:27:03
*Speaker 1:* Oui.

00:27:03
*Speaker 2:* c'est vite agaçant.

00:27:04
*Speaker 1:* Ok, [rire] et [euh] donc elle s'est liée aussi.

00:27:12
*Speaker 2:* Ouais.

00:27:13
*Speaker 1:* Et [euh] est-ce que vous connaissez ou pas, justement, des des stratégies de conservation, parfois des rumeurs, parce qu'il y a beaucoup de on dit que ça, ça doit aller là où ça. Est-ce que vous en avez en tête ou pas ? Est-ce que [euh]--

00:27:35
*Speaker 2:* Bah de [euh] [silence 00:27:30-00:27:33] [euh] d'éviter de mélanger les fruits pour [euh] que ça développe ou pas la maturité.

00:27:40
*Speaker 1:* Oui.

00:27:41
*Speaker 2:* Ça dépend de ce qu'on veut, [euh] d'éviter de nous mettre à la lumière, c'est là où on est pas terrible,

00:27:49
*Speaker 1:* Oui.

00:27:52
*Speaker 2:* [euh] et donc de compart, de compartimenter [euh], alors ça on essaye, mais je pense que c'est pas suffisant.

00:28:00
*Speaker 1:* Oui.

00:28:07
*Speaker 2:* [euh] [silence 00:28:02-00:28:05]

00:28:10
*Speaker 1:* Hmm.

00:28:10
*Speaker 2:* Voilà.

00:28:10
*Speaker 1:* Et donc [euh].

00:28:10
*Speaker 2:* Et ou après source de chaleur, donc à l'abri de [euh] enfin au frais si possible, évidemment.

00:28:20
*Speaker 1:* Ok et donc si vous n'aviez enfin là, admettons, vous n'avez aucune contrainte matérielle, spatiale, financière, qu'est-ce que vous ferez de vos fruits et légumes ? Comment vous les--

00:28:32
*Speaker 2:* Comment je les stockerais ?

00:28:34
*Speaker 1:* Oui.

00:28:38
*Speaker 2:* [euh] Idéalement, un grand une grande étagère ou ouais une grande étagère ouais faut de la place, compartimenter avec les grilles anti anti-mouches ou anti, je sais pas quoi.

00:29:01
*Speaker 1:* Ouais.

00:29:02
*Speaker 2:* Mais qui sont aussi dans un endroit sec et frais et et avec [euh] plein de bacs à rangements voilà.

00:29:14
*Speaker 1:* Ok et est-ce que vous, quand vous avez dit qu'il fallait éviter de mélanger les fruits, est-ce que vous savez lesquels ? Parce qu'en soit, parce qu'il y en a qui font mûrir en dehors de ce que vous disiez est-ce que vous savez lesquels font autant ça fait mûrir les autres.

00:29:30
*Speaker 2:* Les bananes.

00:29:31
*Speaker 1:* Ouais.

00:29:32
*Speaker 2:* Font mûrir les autres.

00:29:33
*Speaker 1:* Ouais.

00:29:43
*Speaker 2:* [euh] [silence 00:29:38-00:29:41] Ouais beaucoup des bananes, je ne sais pas trop d'autres [euh].

00:29:44
*Speaker 1:* Ouais.

00:29:46
*Speaker 2:* [euh] L'ananas, peut-être aussi un moment, je sais plus.

00:29:49
*Speaker 1:* Ok les bananes aussi elle les mets séparément ou sur le papier [euh].

00:29:58
*Speaker 2:* Ouais, on essaye [euh] ou si, notamment pour notamment l'hiver je pense pour les poires, par exemple, qui n'arrivent toujours pas à maturité, qui sont toujours au contraire on va plutôt les mettre avec.

00:30:13
*Speaker 1:* Ok.

00:30:14
*Speaker 2:* Ça mûrissent un peu donc ça laisse ou (euh] l'été des pèches enfin elles arrivent,

00:30:19
*Speaker 1:* Ouais.

00:30:22
*Speaker 2:* hyper dur, par contre, tout ce qui est [euh] de courte [euh] durée ouais presque séparé là pareillement avec les cerises plutôt, je les laisse en barquettes, les fruits rouges, souvent ça reste,

00:30:32
*Speaker 1:* Ouais.

00:30:32
*Speaker 2:* En séparer barquette, les avocats qui ont aussi besoin de mûrir [euh] je vais, je vais mélanger.

00:30:42
*Speaker 1:* Ok donc vous appliquez un peu le--

00:30:44
*Speaker 2:* Ouais on essaye.

00:30:45
*Speaker 1:* Ouais ok en terme de nettoyage [euh] des différents lieux contenant donc le frigo, le cellier, puis là les différents compartiments. Comment vous organisez est-ce qu'il y a quelque chose de systématique ou [euh]

00:31:06
*Speaker 2:* Non.

00:31:06
*Speaker 1:* Non.

00:31:06
*Speaker 2:* [rire] On est pas bon.

00:31:08
*Speaker 1:* Oh comme.

00:31:09
*Speaker 2:* Ouais.

00:31:09
*Speaker 1:* Beaucoup là.

00:31:09
*Speaker 2:* Comme beaucoup ouais.

00:31:09
*Speaker 2:* C'est [euh].

00:31:13
*Speaker 2:* [euh] Bah déjà, c'est souvent plein ou on prend pas le temps de vider et relaver, la corbeille c'est ce qu'on fait le plus.

00:31:21
*Speaker 1:* Ouais.

00:31:21
*Speaker 1:* Et les autres [euh] bah se font vite penser, quand c'est un peu justement pourri ou qu'il y a des légumes abîmés ou légumes ou fruits abîmés, que du coup on on enlève, on fait un petit coup enfin sauf s'il y a sauf s'il y a beaucoup de légumes abîmés d'ailleurs, [inaudible 00:31:38-00:31:38] on les enlève et puis on remet [euh] par dessus [inaudible 00:31:42-00:31:42]

00:31:42
*Speaker 1:* Ok.

00:31:44
*Speaker 2:* C'est pas l'idéal, dans le cellier, on fait presque jamais enfin si on va dire deux fois par an. Et dans le frigo, bah quand on lave le frigo, c'est à dire tous les [ah] les [euh] trois mois. Ouais quatre fois par an c'est pas mal.

00:31:58
*Speaker 1:* Ouais.

00:32:03
*Speaker 2:* Je pense qu'on est on [inaudible 00:32:04-00:32:04]

00:32:11
*Speaker 1:* Ok, donc il y a quand même [euh] enfin il y a quelque chose de régulier, mais c'est pas enfin, c'est pas tous les jours, il n'y a pas un jour dans la semaine, mais il y a quand même une fréquence--

00:32:20
*Speaker 2:* Régulière sans doute pas suffisante mais il y a [inaudible 00:32:22-00:32:22]

00:32:23
*Speaker 1:* Mais je ne sais pas si--

00:32:25
*Speaker 2:* Et on met souvent [euh] on essaye de toujours mettre du du papier absorbant.

00:32:32
*Speaker 1:* D'accord.

00:32:34
*Speaker 2:* Sous chaque compartiment en ciment pour faciliter le lavage et plus pour éviter aussi le pourrissage pour que ça pourrisse plutôt sur le sur le sopalin plutôt que directement sur [euh] sur le compartiment notamment, ou même dans le [euh] dans le fait que ça puisse absorber le jus si s'il y a un jus qui s'écoule.

00:32:58
*Speaker 1:* OK, donc y a du sopalin [inaudible 00:33:01-00:33:01]

00:33:03
*Speaker 2:* Ouais.

00:33:03
*Speaker 1:* Ok.

00:33:03
*Speaker 1:* Est-ce que je peux vous prendre un sopalin ?

00:33:06
*Speaker 2:* Je dois avoir même des mouchoirs si vous voulez.

00:33:16
*Speaker 1:* Peu importe [rire] merci. Je suis allergique au pollen je crois.

00:33:19
*Speaker 2:* [inaudible 00:33:17-00:33:19]

00:33:20
*Speaker 1:* Ouais, c'est dur. Merci.

00:33:25
*Speaker 2:* Il y a de la pluie, c'est retombé et voilà.

00:33:27
*Speaker 1:* Ouais [inaudible 00:33:27-00:33:30] Merci c'est bien [rire]

00:33:38
*Speaker 2:* [inaudible 00:33:36-00:33:39]

00:33:40
*Speaker 1:* Alors, donc ok pour les [euh] les contenants. Est-ce que dans le bac à légumes, vous avez aussi [euh] un sopalin ?

00:33:49
*Speaker 2:* Ouais.

00:33:49
*Speaker 1:* Ouais, ok. C'est à dire que sur les écouvillons, [inaudible 00:33:55-00:33:58] Ok, si maintenant on discute sur vos pratiques de- de préparation et de consommation de ces fruits et légumes, si on prend un fruit que vous avez dit consommer régulièrement chez vous, qu'on a un peu toute l'année comme la banane. Qu'est-ce que comment elle est consommée au sein de votre famille cette banane ? Sous quelle forme--

00:34:20
*Speaker 2:* Crue.

00:34:25
*Speaker 1:* Crue ? Exclusivement crue ?

00:34:29
*Speaker 2:* [euh] De temps en temps quand c'est un peu abîmé en smoothie.

00:34:33
*Speaker 1:* Ok. [silence 00:34:33-00:34:39]

00:34:40
*Speaker 2:* C'est pas une partie ouais, c'est pas [euh] systématique du tout, mais [euh] ça, ça arrive.

00:34:46
*Speaker 1:* Ouais. Ok, [euh] si on prend la pomme.

00:34:54
*Speaker 2:* Crue.

00:34:54
*Speaker 1:* Crue aussi.

00:34:55
*Speaker 2:* Ouais, ouais, la plupart des fruits je pense que [euh] crus, oui.

00:35:08
*Speaker 1:* Ouais. La pomme elle est [euh] très rarement chez vous enfin ou jamais mise dans des préparations.

00:35:18
*Speaker 2:* Ça arrive si on, mais pas- pas hyper souvent, que ce soit en gâteau, ou même au four [euh] ça peut arriver, mais c'est pas c'est pas régulier,

00:35:31
*Speaker 1:* Oui.

00:35:31
*Speaker 2:* c'est pas, c'est pour ça que je dis plutôt crue la plupart du temps quand-même.

00:35:36
*Speaker 1:* Oui, [inaudible 00:35:36-00:33:38] Ok [euh] la pomme, est-ce que vous l'éplucher ou pas quand vous la mangez crue ?

00:35:48
*Speaker 2:* Oui.

00:35:49
*Speaker 1:* Ouais.

00:35:50
*Speaker 2:* On l'épluche la plupart du temps. [euh] Mais c'est surtout pour les enfants aussi.

00:35:59
*Speaker 1:* D'accord.

00:36:00
*Speaker 2:* Et puis après ça dépend [euh] [silence 00:36:04-00:36:07] Ouais parce qu'on prend pas toujours que du bio, donc, ça dépend aussi. Et [silence 00:36:16-00:36:20] ouais, donc et les enfants, souvent préfèrent--

00:36:23
*Speaker 1:* [rire]

00:36:27
*Speaker 2:* voilà [rire]

00:36:29
*Speaker 1:* Ok. [silence 00:36:30-00:36:33] Donc les enfants préfèrent la pomme épluchée ?

00:36:37
*Speaker 2:* Oui.

00:36:38
*Speaker 1:* Et des fois, vous avez des pommes bio ?

00:36:41
*Speaker 2:* Ouais.

00:36:42
*Speaker 1:* Et du coup, ça va changer vous, c'est pas même si c'est vous qui consommez la pomme, ça va changer votre--

00:36:47
*Speaker 2:* Non moi je coupe aussi.

00:36:49
*Speaker 1:* Oui.

00:36:50
*Speaker 2:* Même si c'est beau, mais je préfère aussi, [rire] en quartier

00:36:58
*Speaker 1:* Y a pas que les enfants qui préfèrent [inaudible 00:36:56-00:36:56] La poire c'est pareil ? en quartier

00:37:01
*Speaker 2:* Surtout si on est en fin de rep enfin c'est souvent que c'est en fin de repas que c'est pas [euh] sur un coup de pouce ou sur [euh]

00:37:07
*Speaker 1:* Oui.

00:37:09
*Speaker 2:* [euh] Entre deux repas ou au goûter, [inaudible 00:37:12-00:37:12] moins régulièrement.

00:37:16
*Speaker 1:* Donc c'est [euh] c'est un dessert vous la,

00:37:19
*Speaker 2:* Oui.

00:37:19
*Speaker 1:* vous la préparez c'est pas-- mais si un jour [euh] pour la ça peut vous arriver de la prendre

00:37:28
*Speaker 2:* Comme ça, oui oui oui oui.

00:37:33
*Speaker 1:* Est-ce que vous mangez le trognon ou pas ?

00:37:35
*Speaker 2:* Non.

00:37:35
*Speaker 1:* Non. Il n'y a personne au sein du foyer qui se, qui finit la pomme

00:37:39
*Speaker 2:* Non, non

00:37:45
*Speaker 1:* La poire, c'est le même

00:37:48
*Speaker 2:* La poire, on coupe le, on enlève le [euh] la pomme, ce qui différencie, c'est qu'on a aussi un comment ça s'appelle un tranche- une trancheuse, et les enfants aiment bien [euh]

00:37:58
*Speaker 1:* C'est un truc [euh]

00:37:59
*Speaker 2:* Pas mandoline, non c'est ça là, c'est pour le saucisson ou le pain [inaudible 00:38:05-00:38:05]

00:38:05
*Speaker 1:* Ok

00:38:06
*Speaker 2:* les tranches toutes fines et ça change la donne pour les petits.

00:38:10
*Speaker 1:* Oui.

00:38:10
*Speaker 2:* Voilà, [euh] la poire, c'est en quartier, oui tout le temps à éplucher et ça [inaudible 00:38:17-00:38:17]

00:38:17
*Speaker 1:* Ok.

00:38:20
*Speaker 2:* De temps en temps [euh] des tartes aux poires, mais c'est de temps en temps aussi.

00:38:25
*Speaker 1:* D'accord. [silence 00:38:25-00:38:29] Ok si on prend un légume que vous avez dit, consommer régulièrement la carotte [rire] qu'il ne fallait oublier sur la liste

00:38:41
*Speaker 2:* [rire] Crue, et en batonnet.

00:38:42
*Speaker 1:* D'accord. Ok.

00:38:47
*Speaker 2:* En apéro,

00:38:48
*Speaker 1:* Ouais.

00:38:51
*Speaker 2:* ou en début de repas.

00:38:52
*Speaker 1:* Ok

00:38:52
*Speaker 2:* Comme entrée quoi. Alors ou crue [euh] directement [euh] et toucher les enfants aussi voilà. Evidemment parfois râpée, mais [euh] et aussi après en consommation sur des sur des plats [euh] régulièrement.

00:39:11
*Speaker 1:* Ok, donc crue et cuite.

00:39:14
*Speaker 2:* Oui, mais c'est plus souvent pour que ce soit cru qu'on en est toujours de la cru, en tout cas.

00:39:20
*Speaker 1:* Et donc crue, ça peut être en batonnet, à l'apéro voilà. Râpée en entrée,

00:39:25
*Speaker 2:* Ouais.

00:39:26
*Speaker 1:* ou grignoter comme ça [euh] je vois. [rire] OK, et quand elles sont cuites, est-ce que vous avez en tête des [euh] des plats

00:39:36
*Speaker 2:* des plats

00:39:37
*Speaker 1:* ouais que dans laquelle vous allez mettre--

00:39:39
*Speaker 2:* le ragoût, le couscous ou- ou tajine, [euh] la blanquette.

00:39:51
*Speaker 1:* Ouais.

00:39:58
*Speaker 2:* [euh] [silence 00:39:53-00:39:57] Et les légumes au four [euh] on les mélange de légumes quoi ou on met carottes, pommes de terre, courgettes, oignons, puis je sais quoi on met tout ça au four.

00:40:09
*Speaker 1:* Ok.

00:40:12
*Speaker 2:* Et soupes.

00:40:13
*Speaker 1:* Soupes

00:40:14
*Speaker 2:* Soupes, évidemment.

00:40:18
*Speaker 1:* Et [euh] vous épluchez systématiquement la carotte ?

00:40:25
*Speaker 2:* Oui.

00:40:26
*Speaker 1:* Ok. Est-ce que ça vous arrive d'avoir les carottes avec les fans ou pas du tout ?

00:40:32
*Speaker 2:* Oui, venant du potager, forcément enfin--

00:40:36
*Speaker 1:* Bah ouais.

00:40:36
*Speaker 2:* Et j'utilise pas les fans.

00:40:38
*Speaker 2:* Non [rire]

00:40:41
*Speaker 1:* C'était ça ma question. Donc [euh] et les carottes, vous vous en avez dans votre potager non c'est--

00:40:48
*Speaker 2:* Non, ça vient de chez mon père.

00:40:49
*Speaker 1:* Oui, ok. Et vous les vous [inaudible 00:40:54-00:40:54] pas.

00:40:57
*Speaker 2:* Les fans, non.

00:40:57
*Speaker 1:* Oui.

00:40:58
*Speaker 2:* Non, [euh] non.

00:41:04
*Speaker 1:* [rire] Celle de Radis, c'est pareil ?

00:41:06
*Speaker 2:* Celle de Radi, c'est pareil.

00:41:09
*Speaker 1:* Oui.

00:41:09
*Speaker 2:* [euh] Ça j'en ai [euh] non, c'est pareil, je fais pas de pesto ou de [euh] non.

00:41:19
*Speaker 1:* Non.

00:41:19
*Speaker 2:* On fait de la salade, [inaudible 00:41:20-00:41:20] [rire]

00:41:24
*Speaker 1:* Mais je oui, j'ai appris en faisant cette étude qu'on pouvait faire du pesto avec.

00:41:27
*Speaker 2:* Oui.

00:41:28
*Speaker 1:* Oui, je [inaudible 00:41:29-00:41:30]

00:41:31
*Speaker 2:* Non moi non plus.

00:41:31
*Speaker 1:* Ouais.

00:41:31
*Speaker 2:* Mais [silence 00:41:31-00:41:34] [rire]

00:41:36
*Speaker 1:* C'est consommable mais-- [rire]

00:41:39
*Speaker 2:* Est-ce que c'est une plus value ? Je ne suis pas sûr.

00:41:41
*Speaker 1:* Ouais c'est ça oui. Ok et les radis, est-ce que vous, quand vous consommez des radis, est-ce que vous les épluchez ou pas ?

00:41:54
*Speaker 2:* On les non on les épluche pas, on coupe la queue et les feuilles,

00:41:58
*Speaker 1:* Oui.

00:41:59
*Speaker 2:* et on les lave.

00:42:03
*Speaker 1:* Ok.

00:42:06
*Speaker 2:* Crus à sel.

00:42:06
*Speaker 1:* Oui.

00:42:10
*Speaker 1:* Crus, sel, odeur. [rire]

00:42:16
*Speaker 2:* Odeur évidemment oui.

00:42:16
*Speaker 1:* Ok, [inaudible 00:42:17-00:42:17] La salade, du coup, vous en consommez ou pas ?

00:42:22
*Speaker 2:* Ouais si.

00:42:23
*Speaker 1:* Si, si ouais.

00:42:24
*Speaker 2:* Donc ça, ça va au frigo aussi [euh]

00:42:26
*Speaker 1:* Oui.

00:42:27
*Speaker 2:* parce que là il y a une bataille familiale. [rire]

00:42:29
*Speaker 1:* [ah] c'est-à-dire ?

00:42:30
*Speaker 2:* [inaudible 00:42:30-00:42:30] la salade achetée sous vide et la salade qu'on tire du jardin voilà.

00:42:38
*Speaker 1:* Et du coup qui gagne le [rire] le conflit? Toujours en cours.[inaudible 00:42:-42:00:42:44]

00:42:43
*Speaker 2:* [euh]Oui, c'est souvent ni l'un ni l'autre. C'est souvent- si moi je fais une salade du jardin [euh], c'est moi qui la mangerai.

00:42:52
*Speaker 1:* D'accord.

00:42:54
*Speaker 2:* [rire] Les petites bêtes sont sont interdites,

00:42:58
*Speaker 1:* Oui.

00:42:59
*Speaker 2:* dans l'assiette.

00:43:02
*Speaker 1:* Oui. Donc, c'est votre épouse qui se qui aurait tendance à acheter plutôt les salades en--

00:43:07
*Speaker 2:* Oui.

00:43:07
*Speaker 1:* Et [euh] quand c'est ensachées vous vous la mangez quand-même ou pas ?

00:43:11
*Speaker 2:* Oui, mais là aussi, c'est souvent [euh] en quantité c'est souvent plus mis à la poubelle

00:43:19
*Speaker 1:* Oui.

00:43:19
*Speaker 2:* que [euh] au fond de que dans l'assiette.

00:43:21
*Speaker 1:* D'accord. C'est [euh] est-ce que vous savez pourquoi elle finit [euh]

00:43:27
*Speaker 2:* Bah parce que la date de péremption ça,

00:43:33
*Speaker 1:* Hmm.

00:43:33
*Speaker 2:* et que ça ouais ça se dégrade très vite quand-même.

00:43:38
*Speaker 1:* Et celle du Jardin vous pensez qu'elle se que vous la perdez moins ?

00:43:42
*Speaker 2:* Ah oui !

00:43:42
*Speaker 1:* Ouais ?

00:43:44
*Speaker 2:* Ouais. Bah même justement, même préparé une fois qu'elle est [euh] se laver, découper, [inaudible 00:43:51-00:43:51] se faire plus.

00:43:53
*Speaker 1:* Ouais, mais c'est ça quand vous avez la- la salade, vous la vous la préparer--

00:44:01
*Speaker 2:* Ouais je la prépare, je l'essore ou [euh] ou j'ai du mesclun et je fais que je prends quelques feuilles et ça y est j'en ai plein donc c'est plus facile [euh]

00:44:16
*Speaker 1:* Ouais, ok. [euh] Si on prend le [ah] non, mais si le poivron si vous en avez, c'est ce que vous en achetez pas forcément très souvent.

00:44:26
*Speaker 2:* [oh] Je l'ai même je pense.

00:44:28
*Speaker 1:* Bah ouais ?

00:44:29
*Speaker 2:* Ouais.

00:44:29
*Speaker 1:* Et donc que vous avez dit vous des fois vous le congeler ?

00:44:32
*Speaker 2:* Ouais on congèle en congèle de la courgette pour les soupes l'hiver, du poireau, les petits pois du jardin,

00:44:43
*Speaker 1:* Ok.

00:44:47
*Speaker 2:* les épinards.

00:44:47
*Speaker 1:* Ok.

00:44:49
*Speaker 2:* Voilà.

00:44:50
*Speaker 1:* Ça marche ! Et le poireau vous le consommez comment ? Sous quelle forme vous le consommer vous ?

00:44:57
*Speaker 2:* Soupe.

00:44:57
*Speaker 1:* Ouais.

00:44:59
*Speaker 2:* Quiche.

00:44:59
*Speaker 2:* Ouais.

00:44:59
*Speaker 2:* [silence 00:45:00-00:45:03] Et et en mijoté je veux dire enfin avec le voilà.

00:45:12
*Speaker 1:* Ok et vous avez votre poireau, comment vous le préparer ?

00:45:19
*Speaker 2:* On coupe la racine et on coupe [euh] un tiers du verre.

00:45:25
*Speaker 1:* Ok. Vous consommez un tiers du verre et vous retirez deux tiers ?

00:45:34
*Speaker 2:* Ouais, je pense à peu près ouais [inaudible 00:45:38-00:45:41]

00:45:41
*Speaker 1:* Ok, [euh] ça marche ! [euh] Alors là, si on s'intéresse au moment où vous jetez un fruit ou un légume ou une partie seulement quand ça arrive, pourquoi décidez vous de le jeter plutôt que le consommer ?

00:46:07
*Speaker 2:* [euh] Parce qu'il est trop gâté.

00:46:08
*Speaker 1:* Ouais.

00:46:11
*Speaker 2:* La plupart du temps, c'est ça. Ou que enfin je pense à la carotte si elle est toute fripée, qui y a plus le qu'elle est toute molle, au niveau gustatif, ça aura plus d'intérêt ou [euh] gustatif je sais pas hum. [euh] Voilà !

00:46:32
*Speaker 1:* D'accord.

00:46:35
*Speaker 2:* C'est essentiellement ça.

00:46:39
*Speaker 1:* Donc, là, vous avez parlé de fripper, molle, est-ce que vous pouvez me dire ce qui vient de déterminer que là, le fruit ou le légume pour vous ne vaut plus la peine d'être plus d'intérêt--

00:46:53
*Speaker 2:* Ou noirci quoi ?

00:46:54
*Speaker 1:* Ouais.

00:46:56
*Speaker 2:* Noircie [euh] ouais noircie bah ouais tous les fruits, les avocats qui sont avocats, avocats, mangues qui sont ou c'est quand on ouvre qu'on voit que [rire] toutes les nervures sont noires et,

00:47:11
*Speaker 1:* Surprise !

00:47:11
*Speaker 2:* Voilà [rire] [euh] et ouais, voilà banane, oui, ça dépend un peu, mais là [euh] ça dépend, ça dépend qui le mangent.

00:47:26
*Speaker 1:* Ouais là, ça m'intéresse justement, est-ce qu'il y a des différences [euh] enfin dans votre foyer est-ce que vous avez remarqué des différences dans les préférences de maturité des fruits donc souvent la banane ? C'est un exemple oui, et c'est à dire,

00:47:37
*Speaker 2:* La banane, le melon.

00:47:37
*Speaker 1:* Le melon aussi. banane, le melon, le monsieur

00:47:40
*Speaker 2:* Sur le côté, musqué ou pas.

00:47:42
*Speaker 1:* Oui.

00:47:42
*Speaker 2:* Qu'il apprécie ou pas [inaudible 00:47:43-00:47:45] [rire]

00:47:44
*Speaker 1:* Oui.

00:47:51
*Speaker 2:* [euh] L'avocat tout le monde l'aime de la même manière, mais on ne sait jamais [euh] quand est ce qu'il est ou on est quand est-ce qu'il est à point.

00:47:59
*Speaker 1:* Oui.

00:48:05
*Speaker 2:* [euh] Quel autre fruit, c'est comme ça ? Le poire.

00:48:08
*Speaker 1:* Oui.

00:48:08
*Speaker 2:* [inaudible 00:48:09-00:48:10]

00:48:16
*Speaker 2:* Ouais et sur la banane du coup comment on qui aime quoi ? Qui la préfère comment ?

00:48:23
*Speaker 2:* [euh] Moi j'app- [euh] pour les enfants et ma femme, il faut que ce soit sucré, pas acide.

00:48:31
*Speaker 1:* Ok.

00:48:31
*Speaker 2:* [silence 00:48:31-00:48:35] Sucré, pas acide, sans amertume en gros.

00:48:38
*Speaker 1:* Oui.

00:48:39
*Speaker 2:* Et moi [euh] l'acidité me dérange pas du tout. Je pense ouais au pamplemousse ou [inaudible 00:48:47-00:48:47] ouais il y a que Matthis et moi qui aimons ça pratiquement parce que c'est trop acide pour eux.

00:48:52
*Speaker 1:* D'accord, donc, c'est vous la préférez enfin vous, peu importe quand est-ce que vous la mangez et le reste de la famille, ça va plutôt être quand elle est sucré, pas acide du coup elle est elle est mûre mûre.

00:49:09
*Speaker 2:* Mûre ou oui par contre pas oui pas musqué quoi ! il faut que ce soit parce que si ça devient musqué c'est plus bon non plus hein, pour moi, ça marche pas.

00:49:24
*Speaker 1:* Ok, il faut qu'elle soit il y a un temps [euh]

00:49:26
*Speaker 2:* Oui.

00:49:26
*Speaker 1:* [diaphonie 00:49:26-00:49:30]

00:49:28
*Speaker 2:* Ça va pas non plus.

00:49:32
*Speaker 1:* Ok [euh] et du coup donc, quand quand vous déterminez qu'un fruit et légume est n'est pas enfin que, vous voulez pas le consommer, c'est plutôt sur la vue du coup que vous [inaudible 00:49:48-00:49:48]

00:49:50
*Speaker 2:* Oui la vue et le toucher.

00:49:52
*Speaker 1:* Oui et quand vous êtes face à un fruit et un légume qui justement bah beaucoup moue ou trop gâté qu'est-ce que qu'est-ce que ça vous fait d'être face à ce--

00:50:11
*Speaker 2:* [euh] Bah, c'est embêtant, mais [euh] ça va au compost [rire].

00:50:18
*Speaker 1:* Oui.

00:50:18
*Speaker 2:* Oui, ça fait du déchet, ça fait des déchets voilà.

00:50:22
*Speaker 1:* Ok. [silence 00:50:23-00:50:29]

00:50:30
*Speaker 2:* Ça dépend des fruits ou ça dépend de oui les fruits ont une excellent engrais je crois

00:50:33
*Speaker 1:* Oui.

00:50:36
*Speaker 2:* Et est-ce que vous saurez dire là justement pour lesquelles fruits où là vous vous dites là c'est vraiment dommage plus que d'autres, parce qu'il y en a qui vont--

00:50:45
*Speaker 1:* Fraises, cerises, tous ces fruits faut les prendre rapidement parce que si ça commence à être un peu noirci ou trop gâté ouais c'est dommage.

00:50:59
*Speaker 1:* Et pourquoi cela c'est peut être des fois, peut être plus dommage que d'autres ? Qu'est-ce qui selon vous--

00:51:09
*Speaker 2:* On sait que c'est un fruit fragile, je pense.

00:51:10
*Speaker 1:* Oui.

00:51:10
*Speaker 2:* Le prix.

00:51:18
*Speaker 1:* Oui.

00:51:18
*Speaker 2:* [euh] voilà.

00:51:26
*Speaker 1:* Oui ok et donc est-ce que ça vous est arrivé ou pas un jour de [euh] ne pas avoir forcément remarqué que les fruits et légumes commençaient à être trop [euh] avancé du coup de le manger et d'avoir ce goût ? Est-ce que vous arriveriez à qualifier ce goût dans les fruits et légumes qui deviennent trop--

00:51:46
*Speaker 2:* Trop mûr ou trop [ah] ouais ça a un goût particulier, au moins un avis un avis du pourri d'oeuf

00:51:57
*Speaker 1:* [ah] Oui c'est ça.

00:52:06
*Speaker 2:* D'oeufs.

00:52:06
*Speaker 1:* Ok.

00:52:11
*Speaker 2:* [euh] Ouais qui dégoute quoi !

00:52:11
*Speaker 1:* Ouais [inaudible 00:52:10-00:52:12] Et même moi la première à décrire ce goût qu'on qu'on a tous connu et [euh] quand on avale bah ça a un goût de pourri quoi ! et quand on essaye d'aller plus loin on a pas de--

00:52:25
*Speaker 2:* Ouais [inaudible 00:52:26-00:52:30]

00:52:32
*Speaker 1:* Oui. Je pense aux

00:52:33
*Speaker 2:* Je pense aux aux senteurs olfactifs là, comme pour les [inaudible 00:52:35-00:52:36]

00:52:36
*Speaker 1:* Oui, [silence 00:52:37-00:52:41] ok et donc, du coup [euh], il y a le goût, il y a la texture, il y a les odeurs. Et du et pourquoi vous, c'est pas bon ? Est-ce que c'est la seule raison pour laquelle vous souhaitez pas consommer ? Ou est-ce que, selon vous, il y a d'autres raisons à ne pas consommer ?

00:53:02
*Speaker 2:* Ouais y a des raisons desanté ouais.

00:53:04
*Speaker 1:* Oui. c'est à dire selon selon vous il y a--

00:53:09
*Speaker 2:* Des maladies possible ou on peut parler de dysentrie ou de je sais pas quoi mais il y a le [euh] oui de maladies digestives,

00:53:12
*Speaker 1:* Ok.

00:53:22
*Speaker 2:* ou alors de troubles digestifs.

00:53:23
*Speaker 1:* donc c'est pas très sain de manger quelque chose qui est trop [euh]--

00:53:36
*Speaker 2:* Ouais.

00:53:36
*Speaker 1:* Ok ça marche alors pour [inaudible 00:53:39-00:53:40] Si on discute du gaspillage alimentaire, sa généralité, quand je vous dis gaspillage alimentaire, quels sont les mots qui vous viennent comme ça [euh] spontanément [euh] à l'esprit ?

00:53:57
*Speaker 2:* [euh] Enorme?

00:53:57
*Speaker 1:* Ouais. Ouais.

00:54:08
*Speaker 2:* [euh] Hmm pas suffisamment de mobilisation.

00:54:12
*Speaker 1:* Ouais.

00:54:24
*Speaker 2:* Ouais [euh] [silence 00:54:15-00:52:22] et manque de solutions de de recyclage ou de je sais pas comment dire mais de seconde main, presque. [rire]

00:54:34
*Speaker 1:* Oui.

00:54:37
*Speaker 2:* C'est pas du c'est pas pour trop pour l'alimentaire, c'est souvent pour autre chose, mais mais aussi quand même.

00:54:45
*Speaker 1:* Uh uh oui.

00:54:49
*Speaker 2:* Ouais.

00:54:50
*Speaker 1:* Quand vous parlez de vous avez dit pas suffisamment de mobilisation qu'est-ce que vous, qu'est-ce que vous voulez signifier ?

00:55:00
*Speaker 2:* En prenant compte des pouvoirs publics et et ou des collectivités sur [euh] ces questions là ?

00:55:05
*Speaker 1:* Ouais.

00:55:10
*Speaker 2:* On pense tous à la cantine où [euh] c'est là où les efforts sont faits, mais dans toutes les entreprises, dans tous les restaurants dans tous les-

00:55:18
*Speaker 1:* Ouais.

00:55:22
*Speaker 2:* les restaurations collectives, c'est quand même pas alors que beaucoup beaucoup de Français mangent mal [rire] dans la semaine et c'est pas forcément là où là où-

00:55:34
*Speaker 1:* Ouais.

00:55:34
*Speaker 2:* On pense, ouais ouais.

00:55:39
*Speaker 1:* Ouais.

00:55:39
*Speaker 2:* Ouais et puis après bon, on sait que voilà chaque ménage [inaudible 00:55:41-00:55:41] mais c'est énorme aussi, mais force ouais. Il n'y a pas que les ménages, mais c'est aussi.

00:55:50
*Speaker 1:* Oui ici, on tente une une définition du gaspillage alimentaire, donc si vous on vous appelait, on dit c'est vous qui devez donner la définition du gaspillage alimentaire ou le petit Robert donc avec vos, vos mots et votre propre façon de concevoir. Qu'est-ce que comment vous le définiriez ?

00:56:16
*Speaker 2:* Tout aliment ou [euh] plat préparé non consommé [euh] à date de de consommation non dangereuse pour la santé c'est comme ça.

00:56:51
*Speaker 1:* Ok ça marche. Alors [euh [euh] pour finir, finir juste sur la poubelle connectée des questions un peu pratico pratique. Vous l'avez depuis, ça fait presque un mois là maintenant, oui un mois passé.

00:57:05
*Speaker 2:* Ouais.

00:57:07
*Speaker 1:* [euh] Comment ça se passe? Racontez moi. Est ce que vous le avez gentillement beaucoup, pas beaucoup?

00:57:17
*Speaker 2:* Pas beaucoup [euh] j'ai surtout laissé pourrir trop dans [rire] la cuvette.

00:57:22
*Speaker 1:* [ah] Le petit jus.

00:57:23
*Speaker 2:* Le petit jus [euh] petit jus, pas mal de jus et pas mal de mouche.

00:57:26
*Speaker 1:* Oui oui.

00:57:29
*Speaker 2:* [euh] Je pense que c'est quand même [euh] un peu plus de chaleur d'un coup ça a monter [euh] d'un coup.

00:57:34
*Speaker 1:* Ouais.

00:57:36
*Speaker 2:* Sinon, non [euh] alors je ne sais pas si moi je l'utilise assez facilement. Les enfants ont pas trop fait je crois.

00:57:42
*Speaker 1:* Ouais.

00:57:42
*Speaker 2:* Mais parce que c'est plus c'est nous qui,

00:57:44
*Speaker 1:* Ouais.

00:57:46
*Speaker 2:* [rire] qui recyclons ou qui qui qui regardons ce qui est ajouté ou pas.

00:57:50
*Speaker 1:* D'accord.

00:57:51
*Speaker 2:* Voilà il y a pas eu d'incident technique.

00:57:55
*Speaker 1:* Ok.

00:57:59
*Speaker 2:* [euh] Et je me suis demandé justement quand j'ai enlevé le [euh] le sac, j'ai vu qu'il y avait justement un peu de quelques là je l'ai lavé.

00:58:11
*Speaker 1:* Oui, vous avez oui pas de souci. Donc c'est là [euh] les premiers rendez-vous que j'ai fait [euh] j'ai réalisé que je peux encore changer tout ça. Et là, les derniers, sûrement ça fait un mois des gens qui me disent J'ai toujours pas jugé, j'ai fait [ah] on va le faire tout seul [rire] parce que là vous allez voir et sinon, il y a un bon petit jus [euh].

00:58:33
*Speaker 2:* Ouais. [diaphonie 00:58:33-00:58:36]

00:58:37
*Speaker 1:* Les sacs [euh] je sais pas ils sont compostables et tout mais ils ne tiennent pas à mon avis trop le--

00:58:43
*Speaker 2:* Oui oui.

00:58:46
*Speaker 1:* Vu qu'ils sont compostable je pense qu'il y a une certaine fragilité quand-même,

00:58:48
*Speaker 2:* C'est ça.

00:58:48
*Speaker 1:* et du coup ça, le jus tombe après.

00:58:50
*Speaker 2:* Le jus et se dissout facilement à même le sac [en fait].

00:58:54
*Speaker 1:* Ouais, ouais c'est ça. Donc il faut pas hésiter ouais à changer de sac même si vous n'avez rien dedans, je peux vous en donner si vous avez besoin. [rire]

00:59:02
*Speaker 2:* Non, je pense pas.

00:59:03
*Speaker 1:* Il y a ce qu'il faut ?

00:59:07
*Speaker 1:* [euh] Alors est-ce que vous avez en tête [euh] quelques exemples de ce que [euh] vous avez jeté ?

00:59:16
*Speaker 2:* [euh] Oui [euh] des avocats comme d'hab [euh] des pommes pourries, je crois.

00:59:30
*Speaker 1:* Ouais.

00:59:30
*Speaker 2:* Un peu [euh] concombre,

00:59:38
*Speaker 1:* D'accord.

00:59:39
*Speaker 2:* je crois aussi quelques carottes toute fripées,

00:59:47
*Speaker 1:* Ouais.

00:59:57
*Speaker 2:* [euh] [silence 00:59:49-00:59:55] mangue, une entière.

00:59:59
*Speaker 1:* Ouais elle a dû peser lourd celle-là.

01:00:02
*Speaker 2:* Ouais c'est ça [rire] c'est ça c'est ce que je me suis dans-- [rire] [euh] ouais, c'est plus les fruits entiers ouais c'est là où tout le monde se dit mince c'est quand-même beaucoup.

01:00:12
*Speaker 1:* Ouais.

01:00:13
*Speaker 2:* C'est ça.

01:00:18
*Speaker 2:* [euh] Ouais, c'est ça surtout, je pense.

01:00:22
*Speaker 1:* Ok. Ça marche.

01:00:26
*Speaker 2:* Et après quelques petits fruits rouges oui.

01:00:28
*Speaker 1:* Oui, est-ce que ça vous est arrivé de vous poser des questions du style est-ce que ça ça doit aller dedans ou pas ? Ou non c'est plutôt [euh]

01:00:40
*Speaker 2:* Non, pas trop.

01:00:41
*Speaker 1:* Ou peut-être à part enfin les enfants qui ont pu peut-être des fois pas forcément [euh] y penser. [euh] Est-ce que vous, c'est possible si vous à un moment donné oublié en faisant ça, [inaudible 01:00:54-01:00:54] dedans ?

01:00:55
*Speaker 2:* Ouais, une- une fois ou deux je ne sais plus pourquoi, mais ça a dû m'arriver une fois ou--

01:01:01
*Speaker 1:* Oui, il y a pu avoir un ou deux oublis [euh] par ci par là.

01:01:03
*Speaker 2:* Ouais, ouais.

01:01:05
*Speaker 1:* Ok. [euh] Et l'avocat, vous avez dit comme d'hab si c'est- c'est un- un fait. Vous avez le sentiment de jeter [euh] beaucoup.

01:01:18
*Speaker 2:* Ouais je pense que c'est celui là qu'on jette le plus en entier, on,

01:01:23
*Speaker 1:* Ouais

01:01:23
*Speaker 2:* ou on l'achète, on se dit que c'est bien et [euh] ou il est dur. Ou alors faut prendre des murs, après à chaque fois. Mais [euh]

01:01:32
*Speaker 1:* Oui.

01:01:32
*Speaker 2:* mais ça veut dire qu'il faut le manger tout de suite aussi penser à [inaudible 01:01:35-01:01:35] Je pense que c'est l'un des fruits les plus compliqués.

01:01:39
*Speaker 1:* Ouais. Ok. [euh] [eh] ben, c'est tout bon. Merci beaucoup. Est-ce que il y a des choses sur lesquelles vous aimeriez revenir ou est-ce que vous avez en tête des points que j'aurais pu oublier [euh], qui sont fondamentaux [euh] dans votre conservation [rire] de légumes frais ?

01:02:04
*Speaker 2:* Je crois pas.

01:02:05
*Speaker 1:* Alors j'ai une petite question est-ce qu'il y a des fruits et légumes que vous allez consommer davantage en surgelés ou en conserve ou non c'est relatif-- [euh] ?

01:02:15
*Speaker 2:* En conserve c'est haricot vert.

01:02:18
*Speaker 1:* Ouais.

01:02:20
*Speaker 2:* Et en congelé [euh] épinards, petits pois, mais--

01:02:28
*Speaker 1:* Mais c'est vos frais qui sont congelés.

01:02:31
*Speaker 2:* Épinards c'est les deux on ne l'a pas suffisamment, et que c'est très facile,

01:02:36
*Speaker 1:* Ouais.

01:02:37
*Speaker 2:* tout aussi bon congelé que pas congelé.

01:02:40
*Speaker 2:* Ok, et les haricots, on conserve pareil, est-ce qu'il y a une raison pour laquelle les haricots, on conserve--

01:02:49
*Speaker 2:* Parce que les enfants préfèrent cela que les enfants [rire] et madame préfèrent ceux là à ceux du jardin.

01:02:55
*Speaker 1:* Ok.

01:02:56
*Speaker 2:* Qui peuvent avoir quelques fibres,

01:02:57
*Speaker 1:* Oui, oui.

01:02:59
*Speaker 2:* ou être trop croquants. Là, ils sont tous uniformes.

01:03:01
*Speaker 1:* Ouais. [rire]

01:03:04
*Speaker 2:* Voilà.

01:03:04
*Speaker 1:* Ok, ça marche. Merci.

01:03:08
*Speaker 2:* Mais les haricots, c'est tout [inaudible 01:03:10-01:03:10] Toutes les personnes avec qui on a discuté des conserves, et même moi d'ailleurs, c'est l'un des légumes qu'on consomme le plus en--

**Household F07, Interview 2**

*Speaker 1:* [Inaudible 00:00:00-00:00:05] Alors, pour commencer, je vous propose quand même encore un petit jeu. [rire] Si on peut appeler ça un jeu. Il va y avoir quatre illustrations ici. Voilà. Et je vais vous demander d'en choisir une, au hasard, de la retourner et de me dire spontanément ce qui vous vient à l'esprit, les mots ou les expressions qui vous viennent comme ça, spontanément à l'esprit.

*Speaker 2:* Potager--

*Speaker 1:* Oui.

*Speaker 2:* Carré, [euh] été.

*Speaker 1:* Oui.

*Speaker 2:* [Euh] moi, je ne sais pas [euh], poireaux, choux, salades, vignes, framboises, agencés.

*Speaker 1:* Ok. Donc, vous avez dit potager, en carré. Qu'est-ce qui vous fait [euh] penser [euh] ce mot là ?

*Speaker 2:* Bah les, comment on appelle ça ? Vu que c'est en surélevé.

*Speaker 1:* Oui.

*Speaker 2:* Et que- et que il y a des allées tout autour, que il y a du buis, que les framboisiers sont aussi limités par un parterre.

*Speaker 1:* Ok.

*Speaker 2:* Que la vigne est bien rangée derrière, entre le buis, que-- voilà.

*Speaker 1:* Ok.

*Speaker 2:* Qu'il y a quatre-- Que dans les... le carré, il y en a...il y a quatre carrés...il y a quatre- quatre légumes différents.

*Speaker 1:* Ouais. Et vous avez dit été ?

*Speaker 2:* Ouais. Comme les vignes... comme la vigne, elle est... fin d'été, comme la vigne est verte et que- et que les poireaux sont déjà assez hauts. Mais ce n'est pas encore-- Voilà.

*Speaker 1:* Ok. Donc pour vous, ça correspond à une fin d'été, cette photo ?

*Speaker 2:* Ouais. Ouais, je pense.

*Speaker 1:* Ok. Alors est-ce que vous pouvez me... maintenant me décrire ce que ça vous évoque, ce potager ? Qu'est-ce que- qu'est-ce que vous en pensez ?

*Speaker 2:* [Euh] Quoi ça m'évoque ? Un potager de retraité [euh] qui a le temps d'entretenir son--

*Speaker 1:* Ouais.

*Speaker 2:* [rire] son potager tous les jours ou à un potager de château ou de- ou de religieuses ou de choses comme ça, ou c'est [euh] aussi-- Enfin, c'est le buis qui me fait penser à ça. Voilà.

*Speaker 1:* Ok.

*Speaker 2:* Quelqu'un qui a le temps d'entretenir en tout cas, ouais.

*Speaker 1:* Ouais. Est-ce que vous avez déjà vécu une ou des expériences avec un lieu de ce type ou pas ?

*Speaker 2:* Vu, ouais. Vécu ? Oui, oui.

*Speaker 1:* Ouais. Est-ce que vous pouvez me raconter [euh] en gros vos... votre expérience ou vos expériences avec soit un potager comme ça ou un potager [euh] de manière générale ?

*Speaker 1:* Ben, je dirais le château de Villandry.

*Speaker 1:* Ok.

*Speaker 2:* Où c'est aussi dans des carrés comme ça, où c'est un peu carré, propre, avec du buis partout. Alors c'est encore-- C'est encore un cran au-dessus, mais ça me fait penser à ça.

*Speaker 1:* Ok. Est-ce que vous, vous avez un potager ou pas ?

*Speaker 2:* Oui. Moins bien entretenu, mais avec pas mal de choses aussi qui sont sur la photo.

*Speaker 1:* Ok. On retrouve quoi chez vous ?

*Speaker 2:* Eh ben, de la photo, on trouve la vigne, on trouve les framboisiers.

*Speaker 1:* Ok.

*Speaker 2:* La salade, les... je ne sais pas, je pensais [inaudible 00:04:21-00:04:23] un truc comme ça.

*Speaker 1:* [inaudible 00:04:26-00:04:29]

*Speaker 2:* Donc oui, mais pas-- Enfin, directement en terre, à la différence de là où ils ont mis en carré quoi.

*Speaker 1:* Ok. Est-ce que c'est un-- Le potager, est-ce que c'est un milieu dans lequel vous vous sentez bien ou pas ? Qu'est-ce que-- ?

*Speaker 2:* Oui, oui, oui, oui, oui puisque j'aime bien et puis que j'ai un grand potager, on va dire.

*Speaker 2:* Oui.

*Speaker 1:* Et que ouais, c'est chouette. C'est agréable de faire cette... ces- ces légumes et de préparer ces trucs. Après, ça demande un peu de temps et c'est un peu la difficulté.

*Speaker 2:* Ouais. Ok. Donc, vous avez déjà consommé des produits issus d'un potager, j'imagine ?

*Speaker 1:* Oui.

*Speaker 2:* Et qu'est-ce que vous en pensez de ces produits ?

*Speaker 1:* Ah bah, que c'est, que c'est meilleur et.... enfin, souvent meilleur. Souvent, pas toujours. Ce n'est pas vrai-- . [Euh] Souvent meilleur, qu'on a ça en abondance, que c'est le plaisir d'avoir de la terre à l'assiette.

*Speaker 1:* Ouais.

*Speaker 2:* [Euh] Après, ouais, on sait, on sait d'où ça vient, donc, c'est-- voilà. C'est des produits sains, on sait [euh] forcément- on sait forcément ce qu'on mange, quoi. Et qu'on a plus de plaisir du coup à cuisiner quand on sait que ça vient du jardin.

*Speaker 1:* Ouais. Est-ce que vous sauriez dire pourquoi vous éprouvez plus de plaisir quand ça vient de votre jardin ou pas ?

*Speaker 2:* Parce qu'on est allé du début de la plantation à la récolte et à trouver les recettes et à cuisiner, dans le processus.

*Speaker 1:* Ouais. D'accord. Du coup, il y a cette idée d'avoir du... oui, du début à la fin à l'assiette.

*Speaker 2:* Mm. Tout le cycle.

*Speaker 1:* Tous les cycles. Ok. Vous avez dit c'est souvent meilleur, pas toujours, mais c'est souvent meilleur. Meilleur que- que quoi, par exemple ?

*Speaker 2:* Que les légumes achetés en supermarché ou [euh] ou- ou même en primeur. Enfin, chez un primeur, c'est souvent encore meilleur, oui.

*Speaker 1:* Ok.

*Speaker 2:* Ça dépend des produits, mais c'est quand même souvent meilleur.

*Speaker 1:* Ouais. Je vois. Et est-ce que ça vous arrive-- Est-ce que ça vous est déjà arrivé de jeter certains des produits qui viennent de votre potager ?

*Speaker 2:* Très souvent. [rire]

*Speaker 1:* Très souvent.

*Speaker 2:* Parce que tout arrive en même temps, par moment.

*Speaker 1:* Ouais.

*Speaker 2:* Et que donc- et que donc, ben voilà. De ce qu'on a prévu, après, soit on donne, soit des fois c'est périssable et du coup- et du coup, ben voilà. Mais c'est-- Ou, enfin, c'est de notre jardin et des fois, ça vient aussi beaucoup du jardin de mon père et- et là aussi, il y a beaucoup, par moment, à jeter.

*Speaker 1:* Ok. Et [euh] qu'est-ce que vous ressentez quand vous êtes amené à jeter quelque chose que vous avez produit ?

*Speaker 2:* Ben, de la déception. C'est dommage.

*Speaker 1:* Ouais.

*Speaker 2:* On fait plus attention aux déchets alimentaires dans ces cas-là. Ouais, ça c'est sûr.

*Speaker 1:* Vous faites plus attention quand vous pensez quand ça vient de votre potager ?

*Speaker 2:* Ouais. Oh bah oui. On est plus-- ou en tout cas, on est plus réceptif.

*Speaker 1:* Ouais. Est-ce que vous arriveriez à expliquer pourquoi vous faites plus attention ou voilà, vous êtes plus réceptif à ces produits-là ?

*Speaker 2:* Ben, pour l'effort et [rire] l'effort produit avant. On sait que-- Voilà.

*Speaker 1:* Ok. Ça marche. Alors je vais vous inviter à prendre une deuxième et de faire le même exercice, d'ailleurs de me dire les trois premiers mots ou expressions qui vous viennent spontanément à l'esprit.

*Speaker 2:* Alors [euh] un magasin de primeur de fruits et de fleurs [rire] Je ne vois pas de légumes [rire]

*Speaker 1:* Je crois qu'il y en a au fond. [inaudible 00:09:41]

*Speaker 2:* Bah peut-être. Ah oui, c'est ça. De fruits et légumes. Et [euh] ça me fait penser à quoi ? [euh] Bah aux commerces de proximité.

*Speaker 1:* Ouais. Est-ce que vous avez déjà vécu des expériences avec ce genre de commerce ou pas ?

*Speaker 2:* Oui.

*Speaker 1:* Ouais. Est-ce que vous pouvez me raconter ces expériences ? Comment-- Qu'est-ce que vous pensez de ces magasins-là ? Dans quelles circonstances vous y allez ?

*Speaker 2:* J'y vais le plus souvent pour prendre des fruits.

*Speaker 1:* Mm.

*Speaker 2:* [euh] Qu'est-ce que j'en pense ? Que c'est plutôt bon. Après [euh] que c'est parfois les mêmes... à peu près les mêmes produits qu'en supermarché, au final.

*Speaker 1:* Mm-hmm.

*Speaker 2:* [euh] Mais que voilà, ça fait marcher le commerce de proximité et que ce n'est plutôt pas mal pour ça. Ce n'est pas forcément-- ils, ils tendent à chercher à faire plus bio et local, mais c'est pas--

*Speaker 1:* Ouais.

*Speaker 2:* Ce n'est pas forcé. Ça dépendra du magasin ou de--

*Speaker 1:* Ouais.

*Speaker 2:* Ce n'est pas comme c'est joliment emballé dans un... dans les [rire] dans des paniers, que ça, forcément, ça ne vient pas forcément de, de je ne sais pas, ouais, de l'autre bout... enfin de l'Amérique du Sud ou de-- Voilà [inaudible 00:11:34-00:11:35]

*Speaker 1:* Ok. C'est-- Donc, ce que vous dites, c'est, donc les produits sont globalement bons. Néanmoins, c'est davantage le fait de faire marcher le commerce de proximité qui vous encouragerait à y aller que- que le produit en soi.

*Speaker 2:* Ouais. Que le produit en lui-même, ouais.

*Speaker 1:* Ok. D'accord. Et [euh] dans le ou les commerces que vous avez de ce type-là [euh] dans lequel vous vous rendez. Est-ce que c'est un lieu où vous vous sentez bien ou pas vraiment ? C'est par nécessité que vous y allez ou non, vous avez quand même un peu de plaisir à vous y rendre ? Comment vous-- ?

*Speaker 2:* Si, c'est agréable. Après, [euh] ce qui est compliqué, c'est que ça multiplie les- les lieux. C'est toujours ça.

*Speaker 1:* Complètement.

*Speaker 2:* [euh]

*Speaker 1:* Bonjour.

*Speaker 3:* Bonjour.

*Speaker 2:* Que dans celui que je vais, ça peut parfois bien cailler parce que il n'y a pas [rire] [inaudible 00:12:32-00:12:35] on garde donc ça caille. Voilà, surtout à cette époque et qu'il serait là toute la journée.

*Speaker 2:* Ok

*Speaker 2:* Mais voilà, ça c'est du détail.

*Speaker 1:* Ouais.

*Speaker 2:* Est-ce que t'aimes bien aller dans les magasins comme ça ? Ça te fait penser à quel magasin ?

*Speaker 3:* Ah. C'est à Carrefour City.

*Speaker 2:* Ah Carrefour city. Ouais, c'est vrai que c'est un peu présenté comme ça--

*Speaker 4:* Bonjour.

*Speaker 1:* Bonjour [inaudible 00:13:01]

*Speaker 1:* Toi et Mathis, vous avez fait un bisou aux autres ?

*Speaker 5:* Non.

*Speaker 2:* [euh] Voilà.

*Speaker 4:* Pardon.

*Speaker 2:* Ça te fait penser à quel magasin et qu'est-ce que... comment tu ressens les choses quand tu vas dans ce magasin ? [euh] Et moi je disais à [inaudible 00:13:23-00:13:25]

*Speaker 1:* Bah, ça fait bizarre parce que vous avez-- que c'est un serveur au fond- au fond du resto.

*Speaker 2:* [inaudible 00:13:29-00:13:30] un papillon. [rire] Le style du magasin.

*Speaker 4:* Ça me trouble un peu. Moi, ça me fait penser à Leclerc tout simplement.

*Speaker 2:* Ah ouais.

*Speaker 2:* T'as une zone un peu à part avec des fruits et les légumes.

*Speaker 2:* Ouais. On voit que c'est un commerce de proximité quoi.

*Speaker 1:* Oui, oui. Je sais bien, mais c'est faire penser à quoi ?

*Speaker 2:* Oui, oui, oui. [euh] Qu'est-ce- qu'est-ce que je ressens après ? Ben voilà, ouais, d'aller- d'aller prendre des fruits quoi, surtout.

*Speaker 1:* Ok. Donc le plaisir serait plus dans le fait de--

*Speaker 2:* De récupérer des--

*Speaker 1:* D'être dans le commerce de proximité que- qu'autre chose quoi.

*Speaker 2:* Ah oui.

*Speaker 1:* Que le plaisir du lieu en lui-même, pas-- non.

*Speaker 2:* Non, non. Pas forcément.

*Speaker 4:* Moi, c'est plus on a l'impression dans un commerce de proximité, d'avoir la qualité [euh]

*Speaker 2:* Je dis que non moi.

*Speaker 4:* Hein ? Quoi ?

*Speaker 2:* Moi, je dis que non.

*Speaker 4:* Que non quoi ?

*Speaker 2:* Ben, il n'y a pas forcément de différence.

*Speaker 4:* Il n'y a pas forcément. Parce que, je pense que ils ont les autres grandes surfaces aussi se multiplient. Enfin, Leclerc, ils ont aussi fait une sorte de kiosque au milieu, où on peut aussi -- plus facilement parler avec des personnes qui nous aident à choisir. Service qu'on trouve aussi dans les.... chez les primeurs. Et finalement la qualité est là. Enfin, je suis presque moins déçue par les fruits de ce kiosque-là, chez Leclerc, que [diaphonie 00:14:57]

*Speaker 1:* Ouais. Donc ça, c'est un-- c'est aussi quelque chose qui [euh] qui peut influencer vos choix de, lieux, le fait qu'il y ait une personne qui soit là pour vous guider, des fois sur les- sur les produits.

*Speaker 4:* Non. Ça sera plus la qualité, la qualité des produits. Mais là, en plus justement, ça m'aide à moins me tromper d'avoir quelqu'un qui a l'air de s'y connaître un peu plus quoi.

*Speaker 1:* Ouais.

*Speaker 1:* Mais si je suis sûre qu'il y a la qualité, ce n'est pas forcément la personne qui va faire que--

*Speaker 2:* Qui est là pour que--

*Speaker 4:* Tu viens faire un bisou à la personne ?

*Speaker 5:* Ouais.

*Speaker 1:* Et du coup, est-ce que ça vous arrive de... une fois que vous avez acheté donc, notamment des fruits, de jeter des produits que vous aviez, à la base, achetés là-bas ou pas ? Oui. Et à ce moment-là, qu'est-ce que vous ressentez face au fait d'avoir perdu un de ces- un de ces fruits ou légumes ?

*Speaker 4:* Ben, l'autre jour, j'ai carrément jeté un radis alors que je venais de l'acheter. Enfin, je les avais achetés le matin. J'ai ouvert le truc, j'étais " ah non ". J'ai voulu les prendre pour être rapide et efficace et pas avoir à les nettoyer et ouais, et en fait, ça sentait extrêmement mauvais quand-- Donc quel gâchis !

*Speaker 1:* Ben ouais. Donc acheter déjà-- des fois ça arrive d'acheter déjà pourri quoi.

*Speaker 4:* Ça ou même en drive, tu sais, la dernière fois, les concombres, ils étaient [euh] tout, tout abîmés aussi. Alors je ne les ai pas jetés parce qu'il y avait quand même une bonne partie qui était [euh] mangeable. Mais- mais là, on se dit " bah mince quoi. "

*Speaker 1:* Ouais, c'est sûr.

*Speaker 2:* Oui. Et puis même quand on choisit des fois hein, ça reste traîner et puis on... ça a le temps de pourrir ou-- voilà.

*Speaker 1:* Ouais.

*Speaker 2:* Ouais. Moins, moins à cette époque-ci. C'est ce qu'on se disait sur les--

*Speaker 2:* Ouais.

*Speaker 4:* Qu'on jetait moins.

*Speaker 2:* Qu'on jetait moins. Enfin, on s'en rend compte [diaphonie 00:16:49] en fait, on n'emmène rien quasiment.

*Speaker 4:* Que ça se dégrade moins vite.

*Speaker 1:* Ouais.

*Speaker 2:* Parce que- parce que c'est des fruits oui, qui- qui se préservent et qui se gardent mieux l'hiver ou l'automne quoi pour--

*Speaker 4:* [Nom de l'enfant], tu es censée être couchée là.

*Speaker 2:* On voit une sacrée différence, oui.

*Speaker 1:* Par rapport à la première période de l'étude--

*Speaker 2:* Ah ouais.

*Speaker 1:* C'est intéressant.

*Speaker 4:* Ben, par exemple, cet été, on a eu beaucoup de petites mouches. Je pense que ça n'a pas aidé à conserver [euh] les fruits et légumes qu'on avait là.

*Speaker 2:* Oui, mais c'est beaucoup aussi les fruits les plus périssables quoi, donc de base. Une orange--

*Speaker 4:* Oui. Ça se tient mieux que--

*Speaker 2:* Une poire, il faut quelques jours avant-- Enfin, on les achète--

*Speaker 4:* Oui, que des fraises qui s'abîment tout de suite ou voilà, c'est sûr.

*Speaker 1:* Ouais, c'est des fruits et légumes qui tiennent un peu mieux, mais peut-être aussi des températures de l'extérieur qui font que c'est peut-être aussi plus facile. C'est intéressant que vous vous aperceviez même du coup à travers la poubelle, quoi. Le fait de se dire voilà--

*Speaker 2:* Ah Oui.

*Speaker 4:* Oui. C'est ce qu'on s'est dit hier. C'est ce que je lui ai dit. Il y a une banane achetée. Ah ouais, bien, comme ça, on a un truc acheté [rire].

*Speaker 1:* Allez [inaudible 00:17:51-00:17:54] dans la poubelle.

*Speaker 4:* Et c'est là qu'on s'est dit qu'on jetais moins du coup.

*Speaker 1:* Que-- Ok. C'est bien. C'était tout l'intérêt aussi de faire l'étude sur deux périodes différentes.

*Speaker 2:* Ouais, distinctes.

*Speaker 1:* Ben ouais. Ok. Donc ça vous arrive de jeter de ces produits-là ? Est-ce que, ouais, est-ce qu'il y a une différence entre jeter ces produits-là que par rapport à ceux du potager ou par rapport à d'autres lieux ? Ou globalement, c'est [euh] la même déception. Même si vous avez dit tout à l'heure que le potager, c'était, ça peut être une déception supplémentaire ?

*Speaker 2:* Ouais.

*Speaker 1:* Ouais ? Ok.

*Speaker 2:* Et qu'on ne va pas essayer de- de préserver ce qu'il y a à préserver ou [euh] d'en faire. Ça peut arriver, mais c'est à la marge quand même quoi. Pour les fruits, quelques soucis de temps en temps, ce genre de choses, mais pour les légumes--

*Speaker 4:* [diaphonie 00:18:51] elle a juste envie de pas... elle n'a pas envie de dormir, donc--

*Speaker 1:* Ouais.

*Speaker 2:* On jette, je crois. Ou alors en soupe [diaphonie 00:18:59-00:19:01] de toute façon, elles sont bonnes.

*Speaker 4:* Allez, fais dodo [Nom de l'enfant]. On verra tout à l'heure. Fais dodo.

*Speaker 2:* Les légumes sont bonnes souvent.

*Speaker 1:* Donc selon vous, vous allez peut-être faire moins d'efforts pour préserver un fruit ou un légume qui vient de ces lieux-là que dans le potager, vous allez peut être essayer de le garder plus longtemps ?

*Speaker 2:* Ouais, peut-être un peu. Ouais, ou d'en faire quelque chose-- Après, c'est souvent plus facile puisque dans le potager-- enfin, surtout là, maintenant, il suffit de laisser dans le potager et puis on prend quand on en a besoin donc [euh] À cette époque-ci aussi. Ben ou même, enfin--

*Speaker 4:* Ben oui, on va, on va récolter quand on en a besoin dans la limite de [inaudible 00:19:39-00:19:41]

*Speaker 2:* Oui, c'est ça. C'est ça.

*Speaker 4:* Ou alors, on va-- s'il faut récolter, enfin, je pense aux artichauts, récolter, c'est, ah ben de là, il y en a qui sont prêts, ben on va faire des artichauts ce soir.

*Speaker 1:* Ouais. Oui, ça guide aussi du coup votre votre--

*Speaker 2:* Le choix du menu.

*Speaker 1:* Oui, le choix du menu, oui. Et en même temps, ça fait moins de légumes à stocker. Enfin, s'ils ne sont pas stockés, ils risquent moins de se perdre.

*Speaker 2:* Oui. C'est ça. Sauf quand on fait des haricots verts ou des épinards et que là, faut tout ramasser en même temps.

*Speaker 4:* Oui, c'est ça.

*Speaker 1:* C'est là qu'on peut avoir de la perte des fois.

*Speaker 2:* Ouais.

*Speaker 4:* Là, par exemple, il y a les potirons, enfin, ce que je disais, je n'aime pas ça au point d'en manger autant que ce que je devrais pour liquider le stock quoi.

*Speaker 1:* Oui. C'est sûr. Ouais. Il y a des légumes comme ça qui sont un peu moins faciles à-- Il faut être ingénieux pour--

*Speaker 2:* Oui, [diaphonie 00:20:26-00:20:28] toujours un qui pourrit et qu'on- on le compost.

*Speaker 2:* Ben oui. Ok. [enfant qui pleure]

*Speaker 4:* C'est toujours comme ça ici.

*Speaker 2:* Oui. C'est habituel. [inaudible 00:20:39]

*Speaker 1:* Et ouais. Il n'y a que les enfants qui n'aient pas leur bouleau. [rire]

*Speaker 2:* C'est ça.

*Speaker 1:* Ok. Alors une autre. Même exercice. Qu'est-ce qui vous vient à l'idée, spontanément ?

*Speaker 2:* Supermarché.

*Speaker 3:* Supermarché.

*Speaker 2:* Rayonnage.

*Speaker 1:* Ouais.

*Speaker 4:* En tout cas, moi, ça me fait plus envie de me rendre là que- que là quoi.

*Speaker 1:* Plus envie d'aller chez le primeur que-- Et pourquoi en l'occurrence, ça vous-- Qu'est-ce qui fait que là, ça vous donne moins envie que là ?

*Speaker 4:* Parce que j'ai l'impression qu'il sera de meilleure qualité chez le primeur quoi. Parce que les couleurs ont l'air plus belles, je ne sais pas. Parce que les fruits ont l'air-- Les légumes ont l'air plus-- C'est ce qu'on vous disait tout à l'heure. Enfin, quand il y a un kiosque dans le supermarché, j'ai l'impression aussi que la qualité [euh]

*Speaker 1:* C'est l'agencement qui va vous faire penser-- ?

*Speaker 4:* Peut-être me conditionner sur la qualité du produit quoi. Mais en même temps, par expérience, en général, je suis pas déçue de ce que j'achète.

*Speaker 1:* Ouais.

*Speaker 2:* Et que ce n'est pas un grand supermarché, puisque tout est pesé plutôt et que ce n'est pas- que ce n'est pas des pesées automatiques, voilà. Et que ça ressemble à un supermarché--

*Speaker 1:* [inaudible 00:22:25-00:22:27]

*Speaker 2:* Oui mais tu vois les.. c'est des- c'est des--

*Speaker 4:* Oui, oui. Je comprends ce que tu dis.

*Speaker 1:* C'est des mesures [euh]

*Speaker 4:* Mais je crois que c'est [inaudible 00:22:34]

*Speaker 1:* C'est un super, mais pas un hyper, peut-être. Supermarché, pas un hypermarché.

*Speaker 4:* C'est plus pour te donner une indication [inaudible 00:22:44] et après tu vas...ce que t'as le droit d'aller regarder.

*Speaker 1:* Oui, il n'est pas si grand que ça celui-là, ce supermarché. Dans cette-- ce rayon de--

*Speaker 2:* Si, il est grand ce rayon. Mais je ne sais pas, je me demande si ce n'est pas bio ou-- enfin, je ne sais pas, du coup.

*Speaker 4:* Je suis occupée. Fais dodo.

*Speaker 1:* Ok. Alors est-ce que vous avez déjà eu des expériences avec ce type de lieu ?

*Speaker 2:* Oui. [rire] Fréquemment.

*Speaker 4:* Pas de panique.

*Speaker 1:* Je ne connais pas. Et du coup, est-ce que vous pouvez me raconter globalement votre... vos expériences dans ces rayons ? Est-ce que c'est des moments-- Comment vous vous sentez dans ces- dans ces rayons-là ?

*Speaker 4:* Ben, j'ai moins confiance, moi, sur la- sur la qualité du produit présenté.

*Speaker 1:* D'accord.

*Speaker 2:* Qu'on regarde plus le-- C'est sûrement qu'on est conditionnés, qu'on regarde plus, du coup, les- les offres ou les prix, et plutôt que le choix du- du ou des légumes ou du fruit qu'on vient prendre.

*Speaker 2:* Ok.

*Speaker 1:* Si on-- Si dans la liste des courses, on a noté fruits, on regardera qu'est-ce qu'on prend en fonction [inaudible 00:24:16] aux emplettes.

*Speaker 1:* Oui. Davantage que chez le primeur ou--

*Speaker 2:* [euh] là. Ouais- ouais. Sûrement.

*Speaker 4:* En ça, on n'a pas la même expérience. Moi, je fais moins bien attention aux prix que toi.

*Speaker 2:* Oui.

*Speaker 1:* Vous serez plus-- Là, ce serait plus chez le primeur plus a l'envie de prendre le fruit qui vous fait envie ? Et là, il y a le critère prix qui va--

*Speaker 2:* Ouais. Sans doute.

*Speaker 1:* Davantage que--

*Speaker 2:* Davantage que là, en tout cas.

*Speaker 1:* Ouais. Ok. Et vous madame, pas de différence là-dessus ?

*Speaker 4:* [diaphonie 00:24:50-00:24:54] C'est-à-dire que, ben je me dis [euh] un supermarché c'est censé être moins cher, donc je fais peut être en fonction de fruits ou légumes qui fait envie ou qui a l'air d'être de- d'être de bonne qualité, plutôt que, je ne sais pas aux primeurs, j'aurai des conseils, je ne sais pas. Au primeur-- Non, je crois que moi, à chaque fois, je, je fais en fonction de celui qui me fait envie, en fonction de ce que j'ai prévu de faire à manger. Donc, quel que soit... Et c'est vrai que des fois j'ai des surprises sur le prix. [rire]

*Speaker 1:* Oui. Sur le ticket. Je n'avais pas celui-là.

*Speaker 4:* Ah oui. Quand même.

*Speaker 1:* Ok. [euh] Du coup, vous avez déjà consommé des produits qui viennent de ce type de magasin. Et qu'est-ce que vous pensez de ces produits, de manière générale, même si là, vous pensez qu'il y a quand même une meilleure qualité, globalement. Vous avez des produits qui restent corrects ou c'est-- ?

*Speaker 4:* C'est très variable.

*Speaker 1:* Ouais.

*Speaker 2:* Ouais. C'est difficile de répondre à la question. C'est difficile.

*Speaker 1:* [diaphonie 00:25:54-00:25:56] très bonne qualité comme normalement--

*Speaker 1:* Ça dépend du fruit ou du légume ou ça dépend du moment ? Enfin--

*Speaker 4:* Du moment, je pense. Enfin, en ce qui me concerne. Mais maintenant [euh] ouais, - maintenant, je prends très rarement [euh] sur ce... enfin dans ce... dans cette configuration-là quoi.

*Speaker 1:* Ouais.

*Speaker 2:* Il y a quand même un côté loterie un peu plus important, des fois quoi.

*Speaker 4:* Oui, c'est vrai.

*Speaker 1:* Ouais. Qu'est-ce que-- Est-ce que vous pouvez développer ce côté loterie ?

*Speaker 2:* [rire] Ben, en drive, c'est encore plus important. Je trouve qu'effectivement, on ne peut pas le choisir. Ça, tu l'as dit tout à l'heure.

*Speaker 4:* Ouais, mais ça vient de là quand même.

*Speaker 2:* Mais ça vient de là quand même. Mais du coup, tu ne choisis pas suivant tes critères à toi. Et même après, selon nos critères à nous. [euh] Je veux dire une poire, tu peux la prendre, tu n'en sais rien de comment elle sera et si elle sera farineuse ou si il faudra trois ou quatre jours avant qu'elle soit bonne et si [euh]--

*Speaker 4:* Ou si elle ne sera jamais bonne.

*Speaker 2:* Ou si elle ne sera jamais bonne.

*Speaker 4:* Et ça, ça arrive quand même régulièrement.

*Speaker 2:* Ben oui, c'est pour ça que je prends cet exemple-là. Et que-- et quand tu le prends- quand tu le prends, tu ne sais pas, c'est la loterie. [euh] bah des clémentines, il y a un peu de tout aussi. Tu ne sais pas forcément au départ si l'on voit à la texture ou à la-- mais la sucrosité ou le--

*Speaker 4:* C'est ça. Alors que justement, dans ce fameux kiosque-là chez Leclerc hier, il nous a fait... enfin, il m'a fait goûter tous les cléme... bah, toutes les possibilités et toutes les sortes de clémentines. Et on a choisi en connaissance de cause là.

*Speaker 1:* Ok, ils font goûter dans- dans, dans le kiosque là du coup ou que vos-- Ok.

*Speaker 4:* Ouais, ouais, ouais. L'autre fois c'était le raisin. Ils peuvent effectivement vous faire goûter.

*Speaker 1:* Ok.

*Speaker 2:* Et la loterie, parce que ben, il y a plein de fruits qui sont [euh] abîmés ou [euh] ou ont reçu un coup et- et du coup [euh] bah pourrissent beaucoup plus vite. Et ça, on ne le voit pas non plus à l'achat.

*Speaker 1:* Ouais.

*Speaker 4:* Une question-- Alors, au drive, il y aussi une question de maturité, souvent. Vous vous retrouvez des- des bananes vertes, des tomates très loin d'être mûres, des poires, pareils, qui sont lourds comme des cailloux, ça je trouve. Oui, bah commence par essayer [Nom de l'enfant]. Commence par essayer [Nom de l'enfant].

*Speaker 1:* Donc, finalement, ce... la loterie fait que, enfin, si j'extrapole un peu, vous êtes peut-être plus...enfin, il y aura plus de déceptions avec ces produits-là que ceux dont on a parlé avant ?

*Speaker 2:* Même si--

*Speaker 1:* Même si des fois on est surpris quand même et c'est, c'est ce qui est encore plus surprenant du coup, d'être surpris.

*Speaker 2:* C'est ça. Ouais, ouais.

*Speaker 4:* Oui. Et que là, le prix est en général plus cher et que du coup, ça passe encore moins.

*Speaker 1:* Ouais, le fait que- le fait que vous ayez payé un produit plus cher, vous allez avoir une--

*Speaker 4:* Une attente plus importante.

*Speaker 1:* Ouais. [rire]

*Speaker 3:* [inaudible 00:28:52-00:28:58]

*Speaker 4:* Fais dodo, ma chérie. Bon allez, descends, tu vas parler des fruits et légumes. T'es la spécialiste. Descends t'es la spécialiste des fruits... dans les fruits. Descends.

*Speaker 1:* Et donc, quand vous jetez des produits qui viennent d'ici, est-ce que vous diriez que c'est le même ressenti que sur les, les, les autres environnements ou, ou pas ? Parce que justement le fait que ce soit, il y a un côté loterie, est-ce que ça change quelque chose ou non, la déception de jeter sera identique ? Même si on peut garder le potager en exception, vu que c'est.. vous nous avez dit un effort supplémentaire.

*Speaker 2:* Pour moi, ça change rien.

*Speaker 4:* Ouais, moi je suis plus fataliste avec ça. Enfin, on se dit, ouais, bon bah-- Je m'attends plus à jeter là, quoi.

*Speaker 1:* Ouais. Et du coup, vous êtes-- Est-ce que ça change quelque chose au moment de jeter si vous avez anticipé peut-être plus de jeter ici. Est-ce que--

*Speaker 4:* Moi, ça m'embête plus de jeter-là.

*Speaker 1:* Ça vous vous embête plus de jeter chez le primeur que- que en grande surface ?

*Speaker 4:* Mm. Tu peux descendre. Tu vas le chercher ?

*Speaker 1:* Ok. La dernière photo.

*Speaker 2:* Le marché.

*Speaker 4:* Le marché.

*Speaker 1:* Le marché. Alors, qu'est-ce qui vous vient spontanément à l'esprit?

*Speaker 2:* Marché, primeur.

*Speaker 4:* Moi, j'y vais très rarement, mais--

*Speaker 2:* Non, c'est d'abord dis ce que tu vois sur la photo ?

*Speaker 4:* Ah, pardon.

*Speaker 2:* [rire] Fruits et légumes?

*Speaker 3:* Moi, ça me fait penser--

*Speaker 4:* Aux vacances.

*Speaker 3:* Non.

*Speaker 4:* Je pourrais faire ça ?

*Speaker 2:* Ouais.

*Speaker 1:* Ouais, ouais.

*Speaker 3:* Aux--

*Speaker 2:* Aux quoi ?

*Speaker 3:* À un magasin de [inaudible 00:31:03-00:31:10]

*Speaker 1:* Est-ce que c'est un lieu qui... dans lequel vous avez déjà vécu une ou des expériences ?

*Speaker 2:* Oui.

*Speaker 1:* Ouais. Alors, il y a vacances, rarement ?

*Speaker 2:* Vacances. Rarement. Ouais.

*Speaker 1:* C'est quoi vos occasions de marcher ?

*Speaker 2:* Et ben, quand c'est quand on descend [euh] samedi matin, rarement à Landerneau.

*Speaker 1:* C'est rare ?

*Speaker 2:* Ouais. Ben oui, oui. Non, c'est pour ça que c'est souvent les vacances.

*Speaker 1:* Et les vacances, c'est à Landerneau, ou c'est quand vous êtes en vacances, même ailleurs ?

*Speaker 2:* Quand on est en vacances ailleurs. Quand on est ici--

*Speaker 4:* Quand on a le temps de flâner.

*Speaker 1:* D'accord, Ok. Donc, pour vous, c'est un... le marché c'est un, un lieu de flânerie ?

*Speaker 4:* Oui. Et du coup, cette fois-là, autant [diaphonie 00:32:11] j'achète mes fruits et légumes en fonction de- de ce que je pensais faire. Autant là, ça peut être aussi [euh] plus, comme on est dans la flânerie, c'est ah tiens, ça, ça a l'air bon, on achète quoi.

*Speaker 1:* Ouais. En fait, les achats sont plus spontanés au marché que vous y allez moins avec une liste de courses que aux autres--

*Speaker 2:* Mais ne comprendra pas forcément de fruits et légumes non plus. C'est pour ça qu'on n'y va pas.

*Speaker 4:* Ben, si, on y va quand même.

*Speaker 2:* Ouais.

*Speaker 1:* Ouais, c'est, c'est vrai qu'il y a pas que les fruits et légumes sur le marché. [rire] Moi, je suis très fruits et légumes, mais il y a d'autres [rire]

*Speaker 2:* Alors, non, la flânerie, ce n'est pas forcément ça quoi.

*Speaker 4:* On va prendre des olives, souvent.

*Speaker 2:* Oui, mais ce n'est pas là que tu le trouveras. Ce n'est pas chez le-- ces petits-là. C'est pour ça que--

*Speaker 1:* Oui. Il y aura d'autres producteurs de--

*Speaker 1:* Il y aura d'autres producteurs.

*Speaker 1:* Effectivement.

*Speaker 2:* Il y aura des fruits et des légumes peut être mais-- Et après, il y a des-- de chez le marché. Moi, je ferais la différence entre les, les- les marchés conventionnels où ils font effectivement comme là il y a un peu de tout ou le petit producteur local qui vient déposer... enfin, qui vient vendre ses, ses fruits et légumes, quoi.

*Speaker 1:* Ouais.

*Speaker 2:* Je fais un peu le distinguo. Où au final, les fruits et légumes qui vont là, on peut les retrouver exactement les mêmes ici, quoi.

*Speaker 1:* Ouais.

*Speaker 2:* Avec juste une différence de prix.

*Speaker 1:* Ouais. Ouais.

*Speaker 2:* [rire] Voilà.

*Speaker 4:* Ça t'inspire quoi ma chérie ?

*Speaker 1:* Du coup, si-- enfin, voilà la face qu'est-ce que vous préférez, vous préférez aller acheter avec le producteur ou acheter avec le--

*Speaker 2:* Ben, acheter avec le producteur du coup.

*Speaker 1:* Et pourquoi du coup [euh]-- pourquoi préférez-vous tant qu'à faire être face... enfin voilà, acheter directement depuis le producteur que depuis du coup lui revendre.

*Speaker 2:* Parce qu'il n'y a pas d'intermédiaire, parce qu'on a directement--

*Speaker 4:* Pour faire une meilleure, une bonne action [rire]

*Speaker 1:* Ouais.

*Speaker 2:* Ouais. De payer au juste prix aussi du coup.

*Speaker 1:* Ouais.

*Speaker 4:* Mais, c'est bon. Pour faire-- pour encourager ce genre de- d'initiative.

*Speaker 1:* Ouais.

*Speaker 2:* Mm-Hmm.

*Speaker 1:* Ouais. Donc [euh] si on revient sur le... la flânerie un peu du marché, vous direz que c'est un lieu, globalement, dans lequel vous vous sentez [euh] bien ?

*Speaker 2:* Oui, ce serait dire qu'on a le temps. C'est aussi la question de temps. Enfin, quand on va au marché, souvent, c'est qu'on a du temps. Ou même, même un samedi matin, c'est qu'on a du temps, quoi. Supermarché ou même le primeur, c'est... le temps, il peut compter.

*Speaker 4:* Ben, le primeur, moi, j'y vais si j'ai que ça à acheter et que il faut et que-- enfin, voilà, ça me fait gagner du temps.

*Speaker 1:* Ok.

*Speaker 4:* Et que les fruits et légumes que j'ai vus chez Leclerc ne me convenaient pas quoi.

*Speaker 1:* Oui.

*Speaker 2:* Dans ce cas là, je retourne chez le primeur à un autre moment.

*Speaker 1:* Ouais, ce qui dédouble après le--

*Speaker 2:* Le temps, voilà.

*Speaker 1:* Et ouais, c'est dingue. On a la proximité, mais spécialisé dans chaque-- Donc si on reprend votre expérience avec le marché, elle est plus dans la balade, dans la flânerie que- que essentiellement pour le produit, même si après il y a le plaisir de consommer certains produits, même autres que les fruits et légumes. Donc-- Et si on prend les fruits et légumes, [euh] globalement, ce sont des produits que vous trouvez de bonne qualité ou, ou pas, ou-- ?

*Speaker 2:* De façon globale ?

*Speaker 1:* Ouais, le problème, c'est que ce n'est pas quelque chose... ce n'est pas vous n'achetez pas régulièrement là-bas, donc ce n'est pas évident comme question hein.

*Speaker 4:* Je ne dirais pas que non plus il y a une différence folle quoi.

*Speaker 1:* Ouais.

*Speaker 1:* Non, c'est ça.

*Speaker 1:* Ouais. Ok. Donc, vous avez dit qu'au final--

*Speaker 2:* Ben oui, c'est les mêmes. Enfin, ils vont acheter chez le Saint et [rire] donc je n'ai pas [euh], je n'ai pas le... je n'ai pas cette image-là du coup.

*Speaker 4:* Autant il a certains produits, je ne sais pas si on allait acheter autre chose. Par exemple, tu vas en boucherie, t'achètes une viande et là ah ouais, elle est vraiment bonne par rapport à-- tu ne peux pas dire fruits et légumes.

*Speaker 2:* Non. Ou alors effectivement un producteur, ou là on voit un peu plus ou--

*Speaker 4:* Là, je ne sais pas. Quand on allait à--

*Speaker 2:* Si. Le--

*Speaker 4:* Dirinon.

*Speaker 2:* Ouais, mais--

*Speaker 4:* Ça, il y avait... c'est toujours pareil. Il y avait toujours [diaphonie 00:36:49-00:36:52]

*Speaker 2:* Quand tu achètes, quand t'as un producteur de fraises dans le marché, c'est plus agréable que de le vendre directement... de l'acheter directement [diaphonie 00:35:58]

*Speaker 4:* Ouais, mais est-ce que c'est vraiment meilleur ?

*Speaker 2:* Ah ouais. Si quand même. Pas que... ouais.

*Speaker 4:* Moi, je ne suis pas sûre |inaudible 00:37:07-00:37:10]

*Speaker 1:* Mm. Donc si-- Voilà, avec le producteur, il y a une petite idée que peut être ce serait quand même meilleur, mais ce n'est pas certain. Et sinon, l'intérêt, c'est plutôt le fait qu'il n'y ait pas, en tout cas, il n'y a pas d'intermédiaire.

*Speaker 2:* C'est ça.

*Speaker 1:* Parce qu'il y avait un commerce de proximité, un peu une dynamique de consommation direct, quoi.

*Speaker 2:* Ouais.

*Speaker 1:* Ok. Bon, je pense qu'on a fait le tour pour ce... cette première partie. Alors la deuxième. C'est la première fois que je le fais donc voilà. Vous allez tester. [rire]

*Speaker 4:* On veut bien.

*Speaker 1:* [diaphonie 00:37:51-00:37:52] Il y a dix images de banane, je peux les retourner là, cette fois. Je vais vous demander de les ranger. Alors, en plus, vous allez peut-être avoir des différences tous les deux. De celle qui vous donne--

*Speaker 4:* Vous voulez peut-être que--

*Speaker 1:* Non. Vous pouvez faire un par un et j'espère que vous allez peut-être débattre sur le... donc celle qui vous donne le plus envie à celle qui vous donne le moins envie. Allez-y, vous pouvez-- Il y en a dix en tout.

*Speaker 3:* Il y a que des bananes.

*Speaker 1:* C'est que c'est la même banane.

*Speaker 2:* C'est la même, mais stade de maturité différent.

*Speaker 1:* C'est ça.

*Speaker 5:* Je peux avoir un sucre ?

*Speaker 4:* Un sucre ? Quelle drôle d'idée.

*Speaker 3:* Ça c'est quoi ?

*Speaker 2:* C'est la même banane, mais qui est un peu pourri.

*Speaker 5:* Ça, je l'ai pris en dernier.

*Speaker 2:* Hop hop. Écoute, je ne sais pas trop si c'est-- On peut échanger là ?

*Speaker 4:* Ben, regarde--

*Speaker 2:* Oui. Ouais, mais c'est- c'est- c'est la même.

*Speaker 4:* Mais oui. C'est la même.

*Speaker 2:* Et ouais, t'as raison. T'as raison- t'as raison.

*Speaker 1:* Donc là, elles sont rangées [euh] comment ?

*Speaker 2:* Par stade de maturité.

*Speaker 1:* En stade de maturité. Prendre en photo vos rangements. Elles sont grosses mes photos. Parfait. [inaudible 00:39:21- 00:39:24] Ok. Et alors, maintenant si vous les ranger de celle qui vous donne le plus envie à la... celle qui vous donne le moins envie. Est-ce que c'est pareil. Est-ce en fonction-- ça reste celle qui vous donne le plus envie, elle sera--

*Speaker 2:* Là, non- non- non. Comment tu fais ?

*Speaker 4:* Ben, [inaudible 00:39:40]

*Speaker 2:* Du plus envie au moins envie, on a dit.

*Speaker 1:* Ouais.

*Speaker 2:* On les fait comme ça.

*Speaker 1:* Pas tout à fait.

*Speaker 2:* Je ne sais pas.

*Speaker 3:* Ça, c'est comment maman ?

*Speaker 1:* Alors, est-ce que vous pouvez me raconter pourquoi vous avez mis les bananes dans cet ordre ?

*Speaker 2:* Comme ça ?

*Speaker 1:* Ouais.

*Speaker 2:* [euh] J'aurais peut-être fait...ouais.

*Speaker 1:* On peut bouger hein.

*Speaker 2:* Non, non, c'est bon. Parce que c'est [euh] que ce soit encore, sur les premières, qu'elles soient suffisamment matures et- et pas vertes non plus, et pas complètement sans- sans goût ou sans sucre, comme- comme là quoi. Et après, elles se gâtent et donc du coup, alors il y a plus de- de sucre, mais- mais c'est tout visqueux.

*Speaker 1:* [rire]

*Speaker 2:* Je sais pas comment le dire. [rire] Et que du coup, c'est pas... c'est moins agréable.

*Speaker 1:* Alors si on reprend, vous aimez qu'elle soit sucrée, donc vous savez qu'elle soit suffisamment mature. Mais sauf qu'à la fin, elles sont toujours sucrées, mais c'est la texture qui va vous... qui va devenir désagréable.

*Speaker 2:* Qui va devenir désagréable ou moins- moins agréable, ouais.

*Speaker 1:* Oui. Alors si on prend la première, donc c'est la... c'est le rond blanc. Est-ce que vous pouvez essayer de vous projeter, de l'imaginer là devant vous et de me la décrire à travers vos cinq sens, pas les cinq, mais parties.

*Speaker 2:* Une fois qu'on enlève la peau ?

*Speaker 1:* Ouais. Comme vous voulez, là, vous l'avez devant vous. Qu'est-ce que-- ?

*Speaker 2:* Qu'est-ce que-- [euh] Ferme, sucré.

*Speaker 1:* [rire]

*Speaker 2:* [euh] Je ne suis pas très banane hein. [rire]

*Speaker 1:* Ah, vous n'êtes pas très banane ?

*Speaker 2:* Ouais. Ça n'aide pas. Ça n'aide pas.

*Speaker 1:* Ça peut être que ferme et sucré hein ?

*Speaker 2:* Ouais. Bah alors, ce sera ça.

*Speaker 1:* Ce sera que ça. Et donc là, vous avez cette banane. Et quand elle est comme ça, qu'est-ce que vous faites, du coup ?

*Speaker 2:* Ben, je la mange.

*Speaker 2:* Ouais, vous la manger [euh]-- ?

*Speaker 4:* Tu la manges comment ?

*Speaker 2:* Je la mange comment ? Ben, nature.

*Speaker 1:* Ok.

*Speaker 4:* [inaudible 00:43:23]

*Speaker 2:* C'est ça. Les... ouais. Bon, de toute façon, les quatre cinq premières, c'est nature.

*Speaker 1:* Ok. donc les quatre cinq premières, natures. Et après ? A partir de laquelle, là vous vous arrêtez ?

*Speaker 2:* J'arrête--

*Speaker 4:* Celle-là, on pourrait la faire en smoothie plutôt non ?

*Speaker 2:* Ouais. Celle-là en smoothie, peut être celle-là aussi. Et les autres, ben, on attend de les manger. Ces deux-là, on attend pour les manger et ces deux-là,

*Speaker 4:* On ne mange pas.

*Speaker 2:* On ne mange pas.

*Speaker 1:* Je note. C'était le carré- carré bleu, orange jaune en smoothie. Triangle bleu, triangle rose, on- on attend. [rire]

*Speaker 2:* On attend. [rire]

*Speaker 1:* Ok.

*Speaker 2:* Et [euh] Rond blanc ou carré orange, ça peut aussi être en salade de fruits, mais [euh] bah c'est comme ça qu'on, ouais.

*Speaker 4:* Moi, je ferais une banane [inaudible 00:44:36], banane écrasée aussi. J'écrase et je mets un peu d'eau dedans.

*Speaker 1:* Ok, avec pareil, les premières ou tout là pourrait se faire en-- ?

*Speaker 4:* Moi, ça serait ces deux-là et celle-là aussi. Et peut-être celle-là, la dernière.

*Speaker 1:* Ok. Et là, elle serait.. dans le carré bleu, elle serait trop- trop mature pour l'écraser dans.

*Speaker 4:* Ouais. Moi, je n'aurai pas... enfin ça vient... je ne la mettrai pas en troisième.

*Speaker 1:* Ok, pourquoi ça vient-- ?

*Speaker 4:* Parce que je la trouve trop mûre

*Speaker 1:* Ok.

*Speaker 2:* Et qu'elle en aurait enlevé la moitié.

*Speaker 1:* Ok. Et là vous n'avez pas le même goût et la même exigence sur la- la banane, la maturité de la banane ?

*Speaker 4:* Ouais.

*Speaker 1:* C'est souvent ça la banane. Je trouve que la banane, c'est la- c'est la plus conflictuelle. [rire] Ok, donc vous la cuisinez des fois en smoothie. Est-ce qu'il y a d'autres façons de la, de la cuisiner la banane ou écrasée comme vous faites ?

*Speaker 4:* [inaudible00:45:32-00:45:34] en purée, en salade de fruits et voilà. C'est tout.

*Speaker 2:* Ouais. Pas en gâteau.

*Speaker 4:* Non.

*Speaker 2:* Ouais. Vous ne faites pas de gâteau ?

*Speaker 2:* La dernière fois, ça n'a pas été uS grand succès.

*Speaker 4:* Pas en glace.

*Speaker 4:* En glace, ça serait bon, mais--

*Speaker 2:* Mais avec une glace, mais--

*Speaker 4:* Ben, une glace à la banane, c'est bon. [rire]

*Speaker 2:* Ouais. Pas flambée, ou un peu des fois [euh] dans les rolls.

*Speaker 4:* Dans les crêpes.

*Speaker 2:* Dans les crêpes.

*Speaker 4:* Moi, je prendrai plus à la crêperie quoi. Ici on fait souvent- on fait souvent des crêpes, mais--

*Speaker 1:* Donc si on reprend, vous, elle est plus consommable cru ? À partir de là, vous ne la mangerez plus ?

*Speaker 2:* Je pourrais la manger, mais je ne prendrai pas forcément plaisir [inaudible 00:46:28]

*Speaker 4:* Je pense qu'elle a moins de goût quoi.

*Speaker 2:* Oui. Je pense qu'elle a moins de goût, ouais.

*Speaker 1:* Et vous dès le carré bleu, cru, c'est--

*Speaker 4:* Ben, déjà, je n'aurai pas forcément classé comme ça. Donc, couci-couça.

*Speaker 1:* Allez-y. Classez.

*Speaker 4:* Hop. Je vais changer comme ça.

*Speaker 2:* Celle-là en première, c'est sûr. Celle-là, là comme ça. Tu vois, comme ça.

*Speaker 4:* Ça a l'air peut-être bien comme ça.

*Speaker 1:* Ok.

*Speaker 1:* Ben je vois plus que celle-là [diaphonie 00:46:56-00:46:58]

*Speaker 2:* Celle-là, tu la mangeras ?

*Speaker 4:* Non plus. Arrivé là, [rire]. Arrivé là, ça peut [inaudible 00:47:07]

*Speaker 1:* Ouais. Je vais prendre en photo aussi.

*Speaker 4:* Qu'est-ce que tu fais ?

*Speaker 1:* Ok. Elle est...commence à être un petit peu marron, mais juste à point quoi.

*Speaker 4:* Voilà. À point. Après ça... là, ça dépend. Si je l'ouvre et que il n'y a pas de trace marron, je pense qu'elle sera très bonne.

*Speaker 1:* Oui. Dès qu'il y a des traces marronnes à l'intérieur, c'est ce que--

*Speaker 4:* Ben, j'enlève. J'enlève ou je fais en banane écrasée. J'accepte en banane écrasée [inaudible 00:47:48] mais pas trop. Pas trop.

*Speaker 1:* Et alors que-- Admettons là pour les deux, celle-là à la base, elle n'est pas consommable cru. Admettons que vous êtes contraint de la manger. Qu'est-ce que ce- qu'est-ce que vous ressentez ? Disons, elle est devant votre- elle est devant votre assiette et on vous dit vous n'avez pas le choix, vous la mangez.

*Speaker 4:* Ben, ça peut même être un peu dégoûtant.

*Speaker 1:* Vous là, elle vous dégoûte déjà ?

*Speaker 4:* Ouais. Par la- la, la--

*Speaker 2:* La texture.

*Speaker 4:* Oui. La texture, oui.

*Speaker 1:* C'est la texture plus que le goût qui va venir vous dégouter plus que-- ?

*Speaker 1:* Ben, le goût aussi, mais peut-être de manière moins, moindre que la texture.

*Speaker 2:* Celle-ci, je pense aussi que tu le mangerais sans problème.

*Speaker 4:* Oui, oui, oui sans doute. Mais ça dépend hein. Là, moi, à mon avis, c'est quand même très marron quand je l'ouvre. [rire]

*Speaker 1:* C'est très marron, oui.

*Speaker 4:* Mais c'est pour ça que celle-là, j'ai... je pense que je ne la mangerais même pas en fait, au même titre que ces deux-là. Celle-là, il y a peut-être un espoir.

*Speaker 1:* Ça reste un, un peu [inaudible 00:49:12]

*Speaker 4:* Elle me fera presque un peu plus envie celle-là, puisque je me dis qu'il n'y a pas de pourriture. Mais au final, je pense que je ne la mangerais pas, parce qu'elle ne sera vraiment pas bonne quoi.

*Speaker 1:* Ouais., ouais. Alors si on les rerange en... si on les range en maturité.

*Speaker 2:* Ouais. Comme ça là.

*Speaker 1:* C'est facile ça. Oui, c'est simple. Hop. Ok. Selon vous, est-ce qu'il y a un moment où [euh] le goût de la banane se transforme ? Si oui, à partir de... à quelle vignette, la banane, le goût a changé ?

*Speaker 4:* Ben, pour moi, ça change à chaque fois.

*Speaker 1:* À chaque fois, le goût change ?

*Speaker 2:* Ah oui ?

*Speaker 4:* Ben si, entre là et là.

*Speaker 2:* Oui, oui, oui.

*Speaker 4:* Là ce n'est pas mangeable, là, c'est mangeable. Et à chaque fois, c'est un petit peu plus sucré, jusqu'à ce que ça ait un goût un peu-- que le sucré finalement soit remplacé par le goût du pourri, quoi. Ce n'est pas vraiment le pourri, je dirais, mais peut-être que ce soit trop fait quoi. Enfin, je ne sais pas.

*Speaker 2:* Moi, je dirais là.

*Speaker 4:* Celles-là sont pareilles quoi.

*Speaker 2:* Ah ouais, quasi. Enfin, pas celle-là. Là ou là quoi.

*Speaker 1:* Ok. [euh] alors, est-ce que selon vous, il y a un moment donné ou la banane, elle perd [euh] ses apports nutritifs ou pas ?

*Speaker 2:* Ouais. Je ne sais pas trop laquelle. Là, quand même.

*Speaker 1:* [rire]

*Speaker 4:* Moi, je n'en sais pas. Moi, je dirais presque à la première et après [euh] après--

*Speaker 1:* Ici, elle n'en a pas ?

*Speaker 4:* Oui. Enfin, pas- pas assez.

*Speaker 1:* Donc au triangle rose, elle n'aurait pas d'apport nutritif. Mais après, elle ne les perdrait pas une fois qu'elle les développe.

*Speaker 4:* Non. Je ne pense pas.

*Speaker 1:* Non. Ok. Et est-ce que, selon vous, il y a un moment donné ou la banane devient potentiellement un danger d'être consommée ou pas ? Danger pour votre santé ou non ?

*Speaker 4:* Moi, je pense que la première. J'imagine, maux de ventre, enfin, [diaphonie 00:52:11]

*Speaker 1:* Ok.

*Speaker 2:* Plus la première que la dernière d'ailleurs. Bon, dans la dernière, faudra regarder comment c'est dedans. [rire]

*Speaker 1:* Oui [rire]. Ok. Bon, ça marche. C'est tout bon sur la banane, on a fait le- on a fait le tour de la banane. [rire] Alors pour quasi terminer, je vais vous montrer d'autres photos que normalement, vous allez pouvoir [euh] reconnaître.

*Speaker 2:* [inaudible 00:52:59-00:53:02]

*Speaker 1:* C'est ça, donc des photos que vous avez envoyées ? Donc de fruits et légumes que vous avez jetés. Alors là, l'idée, c'est je vais vous demander d'en choisir un... une photo, et de me la décrire du coup ce que--. Donc on est sur des fruits là, on est sur des fruits. Celle-ci, est-ce que vous pouvez me la décrire, du coup. Qu'est-ce qu'on y voit sur cette photo ?

*Speaker 2:* Une clémentine moisie ayant le quart de la clémentine--

*Speaker 4:* T'avais coupé en morceaux ?

*Speaker 2:* Ah, non, non, non. C'est qu'elle était restée collée dans ce coin-là. [rire]

*Speaker 1:* D'accord. Le quart s'était désagrégé ?

*Speaker 2:* Ouais.

*Speaker 4:* Une partie s'était enlevé quand t'as--

*Speaker 2:* Oui. Ça s'est enlevé quand je l'ai- quand je l'ai enlevé du carton ou du.. ou [inaudible 00:54:01] Donc elle était presque asséchée.

*Speaker 1:* Ouais, elle est-- elle fait-- Donc, vous avez jeté tout le... toute la clémentine quand vous l'avez trouvé comme ça ?

*Speaker 2:* Ouais comme ça, c'était aussi, sûrement que c'était en plein air, plus protégée par la peau.

*Speaker 1:* Ouais. Donc, elle n'était plus protégée. Ok. Alors, est-ce que vous vous souvenez un peu de l'histoire de ce fruit ? De cette clémentine ? Est-ce que vous vous rappelez où est-ce que vous l'avez acheté ou pas ? Ou très probablement--

*Speaker 2:* Probablement en supermarché, en cagette au deux kilos cinq, ou deux kilos. Enfin voilà. Et que dans le lot, il devait rester celle-là.

*Speaker 1:* Ok. Et où est-ce que vous l'aviez stocké cette clémentine ?

*Speaker 2:* Dans, dans sa cagette.

*Speaker 1:* Elle est restée dans sa cagette ?

*Speaker 2:* Elle est restée dans sa cagette.

*Speaker 4:* Dans la cuisine.

*Speaker 2:* Dans la, dans la cuisine.

*Speaker 1:* Ouais. Ok.

*Speaker 2:* À l'air libre. Sans le- sans le sachet ni sans-- voilà.

*Speaker 1:* Ok. Et est-ce que vous arriveriez à connaître la raison pour laquelle, finalement, elle n'a pas été consommée, cette clémentine ?

*Speaker 1:* Moi, je sais pourquoi je ne l'ai pas consommé. C'est que je les ai trouvées trop acides.

*Speaker 1:* Le lot, tout le lot était-- ?

*Speaker 4:* Ouais.

*Speaker 2:* Celle-là, tu t'en souviens ?

*Speaker 4:* Ah ben, je sais que jusqu'à hier

*Speaker 2:* Jusqu'à peu, jusqu'à hier.

*Speaker 4:* Jusqu'à hier, les clémentines, j'ai trouvé trop acide. J'ai une très faible tolérance

*Speaker 1:* À l'acidité ?

*Speaker 4:* À l'acidité. Alors que lui, beaucoup plus.

*Speaker 1:* Ok.

*Speaker 2:* Et moi, pourquoi celle-là ?

*Speaker 1:* Oui. Pourquoi celle-ci, elle a fini comme ça, finalement ?

*Speaker 4:* Et pas les autres ?

*Speaker 1:* Et pas-- Elle n'a pas été consommée ?

*Speaker 2:* Parce qu'elle a sans doute reçu un choc, je pense.

*Speaker 1:* Ouais.

*Speaker 2:* Et avant-- Enfin, au moment du soit du transport ou même de la- de la pose de- de la clémentine.

*Speaker 1:* Vous pensez que le choc ne vient... enfin, il n'était pas forcément chez vous, quoi.

*Speaker 2:* Ah oui, non. Je pense oui. Vu qu'il est resté collé comme ça, au carton, au cageot. Je pense que--

*Speaker 1:* Ok. Alors, est-ce que vous vous souvenez de ce que vous avez éprouvé au moment de- de jeter cette clémentine ?

*Speaker 2:* Qu'est-ce que je me suis dit ? Que ça n'arrive pas souvent de la voir dans cet état-là. Autant on peut la voir comme ces deux photos-là, qui est fréquent. Mais là où ça s'est [euh] asséché et est ouvert juste en le sortant du cageot, ce n'est pas--

*Speaker 1:* Ok. Donc surprenant par rapport à son... à l'état dans lequel elle est.

*Speaker 2:* Oui, par ailleurs... enfin, de l'autre côté, elle paraissait presque bonne, en fait.

*Speaker 1:* Ouais. Ouais. Une surprise, quand on la prend--

*Speaker 2:* C'est ça. [rire]

*Speaker 1:* Ok. Et vous, vous aviez l'intention de la manger quand vous l'avez levée ou vous saviez déjà--

*Speaker 1:* Je ne sais plus.

*Speaker 2:* Oui. Vous ne savez plus sur le.

*Speaker 4:* Ça aurait pu.

*Speaker 2:* Ça aurait pu oui.

*Speaker 2:* Ce n'est pas de l'autre côté.

*Speaker 1:* [inaudible 00:58:01-00:58:05] c'est derrière que ça. [rire]

*Speaker 1:* C'est ça.

*Speaker 1:* Ok. Alors, si on en prend une autre photo.

*Speaker 4:* Ben, je ne sais pas, c'est toi qui l'as jeté.

*Speaker 2:* C'est moi qui l'ai jeté ?

*Speaker 4:* C'est une poire qui a l'air d'avoir pris un coup.

*Speaker 2:* Ouais, percée.

*Speaker 4:* Et qui du coup s'est gâtée.

*Speaker 1:* Ouais.

*Speaker 4:* Probablement, qu'elle n'a pas été mangée, parce que je me souviens, les dernières poires qu'on a achetées n'étaient pas bonnes à consommer en fait. Finalement, quand elles mûrissaient, elles n'étaient pas bonnes.

*Speaker 2:* Pas les dernières, l'avant-dernière.

*Speaker 4:* Quand on les coupait, elle pouvait sembler, extérieurement parlant, être bonnes et en les coupant, ben elles étaient en train de pourrir à l'intérieur quoi.

*Speaker 2:* Ouais.

*Speaker 4:* Alors celle-là, comme on voit une fois qu'elle est abîmée sur l'extérieur, sans doute qu'en plus elle avait pris un coup et qu'elle a pourri. Ouais, parce que pour l'instant les poires, elles n'ont pas été un franc succès.

*Speaker 4:* Je trouve.

*Speaker 1:* Mais les poires, c'est que....c'est curieux. Enfin, j'entends beaucoup de problèmes autour des poires. [rire]

*Speaker 4:* Et j'ai l'impression moi que c'est un peu, enfin, quel que soit le fruit, c'est un peu par... qu'il y a des saisons finalement, où les poires vont être systématiquement très bonnes, les oranges. Et puis, des années où il n'y aura pas une fois où quasiment où elles seront bonnes quoi. Je sais que pour les abricots où ça aussi, ça a cet effet-là.

*Speaker 2:* Ouais, c'est par période, enfin, par tournée de ... par année.

*Speaker 4:* Laisse, c'est parce qu'elle veut aller monter sur celui de Mathis.

*Speaker 1:* Ok. Et très probablement, elle vient d'où cette poire ? Est-ce que vous vous souvenez de son histoire ?

*Speaker 4:* Alors, celle-là précisément, non. Mais en ce moment, les poires on pouvait... moi, ça m'est arrivé d'en prendre en drive.

*Speaker 1:* Ouais.

*Speaker 4:* Les poires, moi, j'ai pris en drive. Est-ce que t'as pris autrement toi ?

*Speaker 2:* Chez primeur.

*Speaker 1:* Ah oui, oui si, j'ai pris chez le primeur une fois aussi.

*Speaker 2:* [inaudible 01:00:27-01:00:29] elles n'étaient pas bonnes.

*Speaker 4:* Non. Non, non. Moi, c'est pour ça que j'ai l'impression que pour l'instant, les poires, ce n'est pas--

*Speaker 2:* Et je les-- Je n'en trouve plus les poires de Rosnoën. Ben ce qu'ils ont, cette année, ils n'ont pas eu beaucoup, et du coup, ça arrive pendant les.. d'habitude, poires de proximité, enfin notamment, [inaudible 01:00:47] les poires de Rosnoën, et là, il y avait pas cette année.

*Speaker 4:* Tu penses qu'elles sont meilleures, [inaudible 01:00:50]

*Speaker 2:* Ah, t'es sûr que elles, enfin meilleures, en tout cas, elles tiennent mieux où elles ne sont pas farineuses comme celles qu'on peut avoir quoi.

*Speaker 1:* Quand vous dites elles tiennent mieux, c'est que selon le lieu de provenance, vous n'avez pas le même, même-- Donc la qualité du produit, même gustativement, mais il y a aussi sur la façon dont ils se conservent, vous voyez des différences dans la--?

*Speaker 2:* Ah, ouais, je trouve.

*Speaker 1:* Ah, oui.

*Speaker 2:* Poires, pommes.

*Speaker 1:* Mais après, si elles sont passées par Rungis, c'est sûr qu'elles ont déjà bien voyagé.

*Speaker 2:* Et puis qu'elles sont passées par les frigos partout de toute, de toute façon, quoi.

*Speaker 1:* Oui.

*Speaker 2:* C'est ça et que du coup, on ne sait pas. Enfin, oui.

*Speaker 1:* Selon vous, le frigo, ça a un impact sur- sur la qualité du fruit ?

*Speaker 2:* Ouais, quand même. Ça peut. Forcément.

*Speaker 4:* Je ne sais pas, mais- mais en tout cas, si... effectivement, ça peut- ça peut se dire que ça fait un moment qu'elles ont-

*Speaker 1:* Oui, oui, qu'elles ont été ramassées.

*Speaker 2:* Oui. Et puis, vu la- Enfin, oui. Les poires, on peut en avoir un toute l'année. Elles sont quand même sur l'étale toute l'année. Alors que a priori, c'est de septembre à janvier. A priori si on réussit à bien les- les conserver sans frigo.

*Speaker 4:* On a le calendrier si tu veux, pour te dire que tu ne risques pas de voir des poires.

*Speaker 2:* Il y a encore des poires en décembre.

*Speaker 4:* Là, t'es en juillet là.

*Speaker 2:* Elles sont encore en février et elles ne sont plus en mars. Si, elles sont encore en mars. D'ailleurs, les poires pommes, elles sont toute l'année, sauf en mai, alors, jusqu'au mois d'avril.

*Speaker 1:* Ok. C'est une- c'est une belle saison. [rire]

*Speaker 1:* Jusqu'au mois d'avril.

*Speaker 4:* Attention, c'est fragile. Et tu ne mets pas- tu ne mets pas tes ongles dedans.

*Speaker 2:* Ouais, et elles sont plus-- Elles reprennent en août. Donc, d'août à avril, on peut avoir poires- poires et pommes.

*Speaker 4:* Deux minutes. Tu fais attention.

*Speaker 1:* Ok. Mais bon, c'est possible qu'au début des saisons, elles ne soient pas-- ou à la fin, enfin, ou aux extrémités de la saison.

*Speaker 4:* C'est ça.

*Speaker 1:* Cette poire a été stockée où ? Est-ce que vous vous souvenez ?

*Speaker 4:* [euh] Dans le panier à fruits, dans la cuisine.

*Speaker 1:* Ok. Et est-ce que vous avez une idée de pourquoi celle-ci, elle n'a pas été consommée ? Est-ce que du coup c'est-- ?

*Speaker 4:* Alors, moi, je pense que c'est parce que l'expérience n'était pas concluante avec les autres, avec ses camarades que nous avions achetés en même temps.

*Speaker 1:* Ok. Du coup, ça a fait que--

*Speaker 4:* Du coup, celle-là, on se dit [euh] c'est pareil.

*Speaker 1:* Ok.

*Speaker 2:* Et puis, elle est ouverte.

*Speaker 4:* Oui, oui. Elle est, peut-être qu'en plus, elle s'est abîmée, comme elle était--

*Speaker 1:* Ouais. Donc là, il y a le choc qui fait qu'en plus elle s'est abîmée, même à l'extérieur. Et en plus de ça, même ses camarades qui, qui ne paraissaient pas abîmés de l'extérieur, n'étaient pas très--

*Speaker 4:* Et non. Je n'ai pas une bonne expérience des, des poires pour l'instant. Cette année, ce n'est pas ça.

*Speaker 1:* Il faut qu'elles fassent leur preuve encore.

*Speaker 4:* C'est ça.

*Speaker 1:* Ok. Et au moment de la jeter, est-ce que vous avez le souvenir d'avoir éprouvé un sentiment particulier ou pas ? Qu'est-ce que-- ? À part, il faut que j'envoie une photo à Charlotte. [rire]

*Speaker 4:* C'est ça. Ça transforme notre vécu avec--

*Speaker 1:* C'est ca, [inaudible 01:04:43] les poires. [rire]

*Speaker 2:* [euh] Non. Encore une fois.

*Speaker 4:* Vous voyez ce que je veux dire encore une fois.

*Speaker 1:* Ok. Ça marche. Alors, pour finir, juste sur la poubelle connectée, vous en avez déjà un peu parlé. L'idée, justement, que vous avez l'impression d'en jeter cette... en cette période-là. Là, l'idée de la poire, et donc est-ce que vous avez le sentiment de- de jeter quand même des produits plus que d'autres ou pas en cette période-là ?

*Speaker 2:* Ben, plus de fruits. Les légumes en fait, on les... on n'a pas jeté de légumes.

*Speaker 4:* On les consomme.

*Speaker 2:* On les consomme.

*Speaker 4:* Si on les achète, c'est que c'est réfléchi dans un menu. Alors que les fruits, c'est de toute façon, on- on va en manger, donc il faut qu'on en ait. Mais ce n'est pas pour un menu précis, quoi.

*Speaker 1:* Ouais. Donc, vous jetez... vous perdez plus de fruits que de légumes, on va dire. Largement ?

*Speaker 2:* Largement, ouais. Ouais, là, il n'y a que la salade qu'on jette, à la limite.

*Speaker 4:* Oui, oui. Sinon les autres, c'est acheté dans un but précis.

*Speaker 2:* Et ça péri moins aussi là, peut-être.

*Speaker 1:* Ouais [inaudible 01:06:07-01:06:08]

*Speaker 3:* Et ça peut être aussi parce que tes parents nous amènent des- des légumes. Et qu'on n'avait pas forcément choisi d'avoir ces légumes-là à ce moment-là ou qu'on n'a pas les mêmes habitudes de consommation qu'eux. Donc du coup, on ne les utilise pas forcément. Mais ce n'est pas arrivé beaucoup.

*Speaker 1:* Ouais. Parce ce que du coup, ce n'est pas choisi dans votre menu ? Ils arrivent sans--

*Speaker 2:* Ouais, ouais. C'est ça. Et ce qu'avec les enfants... Enfin, je sais que c'est... ben souvent le soir, c'est,... ben, il rentre tard, donc c'est moi qui vais faire à manger. Et avec les enfants, je n'ai pas forcément toujours le temps de- de cuisiner des légumes. Ça prend un peu de temps pour cuire ça.

*Speaker 3:* C'est la fraise à mamie ?

*Speaker 2:* C'est la fraise à Mamie. Non, c'est la myrtille à mamie

*Speaker 4:* Et donc c'est comme ça que je peux être amenée à finalement ne pas- ne pas les faire quoi. Mais en général--

*Speaker 2:* Et parce qu'on fait aussi plus souvent à cette époque-ci de soupe et que du coup l'état des légumes est aussi un peu moins important, parce que c'est mixé de toute façon.

*Speaker 4:* Non pas. En général, on les fait, ils sont abîmés.

*Speaker 2:* Non, en général, ils ne sont pas abîmé. Mais si il y en a abîmés, tu coupes le goût et c'est moins gênant.

*Speaker 4:* Mais c'est vrai que je pense que ce qu'il faut retenir, c'est que on les cuisine... enfin, c'était prévu quoi. Donc, il y a moins de perte parce que ça s'inscrit dans un... dans une prévision.

*Speaker 1:* Donc, sur les légumes, il y a d'une, le fait que ce soit... enfin, il y a une organisation qui fait que normalement ils sont prévus pour être consommés, donc il y a moins de perte. Et en plus s'ils sont abîmés, les soupes permettent de--

*Speaker 2:* Les soupes permettent de-- ouais.

*Speaker 1:* Et donc ça, même quand.. Si vous faites des smoothies quand même aussi avec les fruits, mais moins que--

*Speaker 4:* Non, pas souvent. Les soupes, on en fait beaucoup beaucoup, mais smoothie, c'est rare. Ça sera justement s'il y a une banane, pour revenir dessus, s'il y a une banane qui [rire]

*Speaker 2:* Et en y rajoutant un autre, une poire un peu abîmée ou un.. ou une pomme.

*Speaker 4:* Ouais, ça reste rare.

*Speaker 1:* C'est moins fréquent que--

*Speaker 4:* Est-ce que tu peux arrêter de te promener avec ce truc qui risque de casser ma chérie. J'aimerais bien que tu le reposes.

*Speaker 1:* Donc, finalement, est-ce que...enfin, la soupe, est-ce que c'est une stratégie ? Est-ce que des fois vous allez faire une soupe en disant ben là c'est abîmé, je vais faire une soupe ou non c'est je vais faire une soupe, oh lui, il est un peu abîmé, je le mets quand même dedans.

*Speaker 4:* Oui.

*Speaker 1:* C'est plus... ce n'est pas je vais faire une soupe parce que là je vois que c'est en train de se perdre.

*Speaker 4:* Non. Parce que de base on fait des soupes.

*Speaker 1:* Ouais, c'est on veut faire une soupe, mais là, la carotte n'est pas très belle, mais ce n'est pas grave, on la met quand même dedans ?

*Speaker 4:* Mm-Hmm. Parce que les enfants aiment bien les soupes. Que c'est aussi une manière pratique de leur faire manger des légumes, et que-- Donc on fait ça. Ça peut être fait pour plusieurs soirs. Enfin--.

*Speaker 2:* Ouais. Facilité.

*Speaker 1:* Ouais. La soupe c'est--

*Speaker 4:* C'est- c'est efficace.

*Speaker 1:* Ok. Ben, très bien. Et ben j'ai tout posé mes questions.

*Speaker 4:* Ben, c'est parfait.

*Speaker 2:* Parfait.

*Speaker 1:* Un grand merci. Alors, la suite de l'étude. Donc là, vous avez [euh].

**Household F08, Interview 1**

00:00:04
*Speaker 1:* C'est partit. Ok alors, dans un premier temps, je vous pose un petit jeu de devinettes. Je vais vous demander de penser à un fruit ou un légume [euh] que vous aimez tout particulièrement. Vous allez penser à ce que ces légumes sans jamais me révéler son identité.

00:00:21
*Speaker 2:* D'accord.

00:00:22
*Speaker 1:* L'idée, c'est que je devine uniquement grâce à votre description.

00:00:25
*Speaker 2:* OK.

00:00:27
*Speaker 1:* Est-ce que vous en avez un en tête?

00:00:32
*Speaker 2:* [Euh].

00:00:40
*Speaker 1:* Choisissez votre légume.

00:00:40
*Speaker 1:* [Euh] c'est bon.

00:00:40
*Speaker 1:* Oui, alors allez-y je vous écoute. Dites moi tout sur ce fruit ou ce légume.

00:00:47
*Speaker 2:* Qu'est-ce qu'il faut que je vous dise ? [euh] Déjà c'est un fruit d'été.

00:00:48
*Speaker 1:* Ouais.

00:00:50
*Speaker 2:* [Euh] granuleux, un peu d'apparence.

00:00:52
*Speaker 1:* [mhh]

00:00:55
*Speaker 2:* Dont j'aime toute la famille et de couleur rose foncé (euh] bordeaux.

00:01:05
*Speaker 1:* Ok, qu'est-ce que vous aimez tout particulièrement en ce fruit ?

00:01:10
*Speaker 2:* Son goût.

00:01:11
*Speaker 1:* Son goût?

00:01:15
*Speaker 2:* Le fait que souvent bah ça pousse dans les jardins que on le cueille et on le consomme immédiatement.

00:01:19
*Speaker 1:* Ok. A quel moment de la journée vous mangez le plus souvent, est-ce qu'il y a un moment précis plutôt le matin ?

00:01:36
*Speaker 2:* Quand je le trouve [rire].

00:01:36
*Speaker 1:* Ah

00:01:42
*Speaker 2:* [rire]

00:01:42
*Speaker 1:* Ok

00:01:45
*Speaker 2:* Donc quand.. Là je n'en ai pas dans mon jardin.

00:01:47
*Speaker 1:* Ouais.

00:01:47
*Speaker 2:* Ca fait partie des choses prévues

00:01:51
*Speaker 1:* Du coup, vous dites que vous mangez immédiatement et que finalement, quand ... racontez moi quand vous ... quand vous le voyez, ce que vous faites jusqu'à ce qu'il entre dans votre bouche est qu'il y'a des étapes ?

00:02:04
*Speaker 2:* Bahh, bah Je vais vérifier qu'il est mûr.

00:02:07
*Speaker 1:* Ok.

00:02:07
*Speaker 2:* Le cueillir, en fait, comme il ne se cueille que quand il est mûr. Je vais voir s'il vient facilement quand je dois le cueillir

00:02:13
*Speaker 1:* Ok.

00:02:14
*Speaker 2:* et directement des mains à la bouche.

00:02:17
*Speaker 1:* OK.

00:02:20
*Speaker 2:* sans transition.

00:02:23
*Speaker 1:* [rire] Ca marche. [Euh] alors du coup, la question c'est ou est-ce que vous vous le procurez le plus souvent ?

00:02:29
*Speaker 2:* [Euh] bah c'est plutôt dans, dans un jardin.

00:02:30
*Speaker 1:* dans un jardin ? ok.

00:02:32
*Speaker 2:* J'ai mal choisi c'est ca ? [rire]

00:02:39
*Speaker 1:* [rire] Non c'est très bien. [Euh] alors, est-ce que vous arrivez à.. à dire ce que vous ressentez quand vous le mangez ?

00:02:45
*Speaker 2:* Bah beaucoup de plaisir. C'est quelque chose, c'est un fruit sucré, juteux, qu'on n'a pas si souvent l'occasion de manger parce que c'est pas quelque chose qu'on trouve à prix raisonnable dans le commerce.

00:02:56
*Speaker 1:* D'accord.

00:02:59
*Speaker 2:* Voilà. Sinon, je le trouve parfois en pâtisserie.

00:03:02
*Speaker 1:* Ok.

00:03:04
*Speaker 2:* Il est aussi en pâtisserie, mais le vrai plaisir, c'est de le trouver dans un jardin et de pouvoir se faire une orgie.

00:03:12
*Speaker 1:* Je vois, et est-ce que vous avez le sentiment qu'il vous apporte quelque chose en plus du plaisir [euh] du plaisir de le manger ?

00:03:26
*Speaker 2:* Bah [euh] Je sais pas, j'aime bien l'idée de vraiment de le cueillir soi-même et de le mettre tout de suite, de tout de suite le consommer. c'est ça, c'est le plaisir de ... de le produire soi, de le trouver [euh] c'est le plaisir de la découverte en faite. Et puis de le partager aussi avec les enfants qui viennent se goinffrer à mes côtés parce qu'ils me voient.

00:03:50
*Speaker 1:* Ok.

00:03:50
*Speaker 2:* Mon mari moins.

00:04:01
*Speaker 1:* Ah il est moins ?

00:04:01
*Speaker 2:* C'est pas un gamin de, c'est un gamin de la ville donc les fruits en dehors des pommes [rire]

00:04:04
*Speaker 1:* Je vois. Et est-ce qu'à l'inverse vous lui trouvez quelques inconvénients ?

00:04:04
*Speaker 2:* Ben, c'est pas toute l'année, c'est même pas 2 mois par an.

00:04:08
*Speaker 1:* D'accord.

00:04:09
*Speaker 2:* Et puis, il y a des années où je n'en ai même pas du tout.

00:04:12
*Speaker 1:* D'accord.

00:04:13
*Speaker 2:* Donc c'est pas un fr... C'est un fruit qui est à la fois très simple et pas, pas si facile que ça à dégoter.

00:04:18
*Speaker 1:* Ouais.

00:04:24
*Speaker 1:* Ok et puis vous avez dit que ... aussi dans le commerce, il est pas des fois dans des prix aussi

00:04:29
*Speaker 2:* Oui et puis il est moins bons dans le commerce surtout.

00:04:33
*Speaker 1:* Ouais.

00:04:35
*Speaker 2:* Parce que ce qui est bon, c'est quand il est gorgé de soleil aussi.

00:04:37
*Speaker 1:* Ok. Alors, c'est le moment de vérité.

00:04:44
*Speaker 1:* [Inaudible]

00:04:47
*Speaker 1:* Je dirais la framboise.

00:04:49
*Speaker 2:* Oui.

00:04:50
*Speaker 1:* Oui ? [rire]

00:04:53
*Speaker 2:* C'était ça ou la fraise.

00:04:55
*Speaker 1:* Ah oui ? C'est un peu la même famille mais .. dans l'esprit

00:04:59
*Speaker 2:* Bah oui, es fraises j'en ai sur le balcon.

00:05:01
*Speaker 1:* Oui ? On retrouve un peu ..

00:05:04
*Speaker 2:* C'est même .. je me suis dit en avancant dans vos questions c'est amusant parce que c'était ça ou la fraise et la fraise c'est un peu pareil, sauf que les fraises, j'en ai [rire]

00:05:11
*Speaker 1:* Ouais, plus accessible

00:05:12
*Speaker 2:* J'en achète dans le commerce.

00:05:16
*Speaker 1:* Chouette. Alors maintenant j'aimerais qu'on discute de vos pratiques d'achat et de votre organisation générale et du coup, au sein de votre foyer, qui est-ce qui est amené à faire les courses alimentaires?

00:05:29
*Speaker 2:* Oh, c'est moi 95%

00:05:30
*Speaker 1:* D'accord. Et du coup, racontez moi comment vous vous organisez ?

00:05:37
*Speaker 2:* [Euh] bah en général, je m'en occupe le vendredi après midi avant de récupérer les enfants donc [euh) supermarché et voilà, j'ai envie de dire le moins mieux

00:05:47
*Speaker 1:* mhhmhh

00:05:48
*Speaker 2:* Et [euh] je complète par [euh] le primeur quand je peux. Pas trop à cette saison parce qu'il y a moins de choix

00:05:55
*Speaker 1:* Ok.

00:05:57
*Speaker 2:* Et [euh] et une ou deux fois par mois, par [euh] le supermarché, par le, les surgelés.

00:06:07
*Speaker 1:* Ok. Donc, c'est une organisation assez régulière, vous allez souvent le vendredi après midi ?

00:06:11
*Speaker 2:* [Euh] Ouais, il m'arrive dans la semaine de racheter un truc ou deux en particulier. En fait y'a un truc que j'y pense pas assez à vous. C'est y a un truc idiot. C'est que les bananes, par exemple, en ce moment, il n'y a pas beaucoup de fruits, donc je prends des bananes, mais je veux les acheter le jour même pour les manger le soir

00:06:26
*Speaker 1:* D'accord.

00:06:26
*Speaker 2:* Parce que chez moi, il les aime pas noircies, vraiment voilà Donc là, je viens d'acheter [euh] Je sais plus j'en ai acheté mardi et on les à consommer le soir même.

00:06:35
*Speaker 1:* D'accord.

00:06:36
*Speaker 2:* Donc, là, c'est vraiment presque à l'unité.

00:06:40
*Speaker 1:* Vous devez aller [euh]

00:06:42
*Speaker 2:* Mais bon, c'est en passant, en rentrant de l'école, on passe, on récupère, et puis ..

00:06:48
*Speaker 1:* D'accord. Et Est-ce que vous savez ce que vous allez acheter avant de, d'aller faire vos courses par exemple le vendredi après midi au supermarché? Est-ce que vous savez ce que vous allez acheter avant de partir ou bien ?

00:06:58
*Speaker 2:* Grosso modo, oui, puisque c'est relativement les mêmes choses.

00:07:02
*Speaker 1:* D'accord

00:07:02
*Speaker 2:* D'une semaine sur l'autre, il y a des choses qui reviennent, on a des habitudes.

00:07:06
*Speaker 1:* mhh

00:07:07
*Speaker 2:* Donc oui, grosso modo, oui, j'ai une, j'ai souvent ma liste.

00:07:09
*Speaker 1:* Ouais.

00:07:12
*Speaker 2:* Mais après ma liste, par exemple [euh] dans les choses, il va y avoir écrit un fruit et je vais regarder ce qui a à l'étale et en fonction j'achète j'achète pas, par exemple.

00:07:20
*Speaker 1:* D'accord

00:07:21
*Speaker 2:* Mais après, tout ce qui est de la charcuterie, je prends toujours grosso modo les mêmes choses.

00:07:29
*Speaker 1:* Ouais.

00:07:29
*Speaker 2:* [Euh] la, comment ca s'appel ? la crèmerie pareil.

00:07:30
*Speaker 1:* Ouais.

00:07:31
*Speaker 1:* Donc c'est assez régulier.

00:07:34
*Speaker 1:* Ok. Donc votre liste quand vous en faites une comment elle s'organise est-ce que ?

00:07:41
*Speaker 2:* Par rayon casiment

00:07:41
*Speaker 1:* Ouais ? Donc avant de partir, ça vous arrive

00:07:45
*Speaker 2:* Ouais donc soit je fais sur un papier [euh] volant [euh] et puis je repartie par groupe de .. d'amis de .. par groupe selon suivant par exemple je vais mettre je sais pas la crèmerie dans au même endroit, etc. Soit c'est dans mon téléphone j'ai en grosune liste ou je coche décoche suivant les jours. Mais c'est, je l'utilise quand j'ai pas le temps de faire papier. Je préfère papier.

00:08:06
*Speaker 1:* D'accord. Ok. Et du coup, quand vous êtes au moment d'acheter des fruits et des légumes,

00:08:11
*Speaker 2:* Mhmh

00:08:11
*Speaker 1:* Comment est-ce que vous décidez des quantités [euh] que vous allez acheter? Donc vous m'avez parlé des bananes presque à l'unité pour qu'elle soient..

00:08:17
*Speaker 2:* Ouais ca j'en prends les bananes, par exemple je sais, j'en prends. J'en prends cinq. Parce que si j'en prends parce qu'on est cinq. Mais [euh] si... j'ai remarqué que si j'en prends plus [euh], souvent, il y en a qui se perdent.

00:08:27
*Speaker 1:* D'accord.

00:08:27
*Speaker 2:* C'est ..voilà, les bananes, c'est un truc assez [inaudible]. Je ne sais pas pourquoi.

00:08:32
*Speaker 1:* Mh

00:08:32
*Speaker 2:* Après, ça va être à l'allure des fruits [euh] au choix qui a.

00:08:36
*Speaker 1:* Mh

00:08:40
*Speaker 2:* [euh] Finalement, j'achète pas tant que ça de légumes frais, je vais acheter des tomates, des concombres, des carottes.

00:08:44
*Speaker 1:* D'accord.

00:08:45
*Speaker 2:* Surtout à cette saison.

00:08:48
*Speaker 1:* Mh

00:08:48
*Speaker 2:* [euh] En légumes ca va être sur tomates, concombres, carottes sur ces mois là. Je vais acheter chez le primeur par ... Par contre, c'est quasiment que chez le primeur des, des betteraves que je cuit, et j'ai récemment découvert que je pouvais les mettre au congélateur donc j'en fais une quantité importante le congélateur et je décongèle quand j'ai envie.

00:09:08
*Speaker 1:* D'accord.

00:09:09
*Speaker 2:* [euh] Après, bah y a bien sûr oignon, ail, pommes de terre ce jour là, ça je vais acheter aussi pareil. Pas, pas chaque semaine parce que bah je vais acheter un gros paquet. et puis ça peut durer un certain temps.

00:09:18
*Speaker 1:* Mh

00:09:20
*Speaker 2:* Donc ça, c'est les trucs en légumes, c'est des trucs classiques que je prends quasiment toute l'année. Les tomates, je sais que c'est pas toute l'année [euh] à prendre, mais [euh] mais bon. Et puis sinon après, c'est suivant pour les légumes, ça vraiment être selon ce que je vois, ca peut être des champignons, je sais que les champignons, il y en a toute l'année, donc ça peut être des champignons, mais comme il y en a à chaque fois, il y en a toujours un qui n'aime pas chez moi, donc pareil pour les courgettes. [euh] pareil pour les aubergines. Vraiment, ça va être là. Si je prends des légumes frais, c'est plutôt rare pour les légumes parce que je préfère avec le surgelé, c'est plus fin, c'est plus pratique en fait. Les légumes, c'est surtout du surgelé.

00:10:01
*Speaker 1:* D'accord.

00:10:05
*Speaker 2:* Pour les légumes ca c'est fait, pour les fruits, ça va vraiment être en fonction de ce que je vois.

00:10:10
*Speaker 1:* D'accord.

00:10:11
*Speaker 2:* Il n'y a pas de règle, il n'y a pas de normes. Des fois, j'ai une demande, en ce moment, j'ai de la demande pour des pommes parce qu'ils savent qu'il n'y a plus grand chose d'autre et que les fraises de Plougastel à 15 euros du kilo, c'est hors de question. C'est devenu hors de question alors que pendant longtemps on était très fraises de Plougastel.

00:10:30
*Speaker 1:* Mh

00:10:32
*Speaker 2:* Donc [euh] en ce moment, il y a peu de choix donc [euh] il y a les abricots qui arrivent, pour l'instant, ils sont d'Espagne.

00:10:39
*Speaker 1:* Mh

00:10:39
*Speaker 2:* Donc j'en prends avec parcimonie, mais on aime tous les abricots, donc voilà. Donc en ce moment, ca fait deux fois de suite que je prends des abricots parce qu'il y en a, mais j'en prends que pour une fois.

00:10:50
*Speaker 1:* D'accord.

00:10:51
*Speaker 2:* Et je les laisse mûrir un peu parce qu'ils sont pas ... ils sont pas encore tops. [euh] Voilà donc j'essaye de voilà en ce moment c'est compliqué parce qu'on est en fin d'hiver.

00:11:02
*Speaker 2:* Ouais.

00:11:03
*Speaker 2:* Et c'est vraiment fin si y'a les tout ce qui arrive là, les fruits d'Espagne et... Les nectarines, les pêches, mais pour l'instant, je n'ai pas acheté, je regarde, mais j'achète pas, ça me paraît trop tôt. Pareil, le melon [euh] là il vient du Maroc ca m'attire pas Voilà donc j'attends la ... c'est pas la bonne saison pour les fruits et légumes, donc vous poser la question à un moment, mais [euh] je fais vraiment en fonction de ce que je trouve, ce qui a l'air intéressant. Mais j'essaye de m'atteler sur les saisons, être dans des trucs de saison. Ça, c'est important pour moi. C'est rare que... des fraises en décembre, ça ne m'intéresse pas . C'est la base, c'est un peu comme pour le poisson en fait.

00:11:48
*Speaker 1:* Mh

00:11:48
*Speaker 2:* Le poisson y a des saisons aussi. Et donc. Même si on se fait avoir hein des fois y a un truc qu'on réalise pas du tout que c'est pas de saison.

00:11:58
*Speaker 1:* Ouais. D'accord et du coup, vous avez [euh] comment dire, Vous avez différents lieux où vous procurer [euh] Et qu'est-ce qui fait que des fois, vous allez choisir par exemple les betteraves vous les acheter chez le primeur? Qu'est-ce qui fait que vous choisissez [euh] celui là, en particulier pour par exemple pour la betterave et pas pour autre chose ?

00:12:18
*Speaker 2:* [euh] Pour la betterave? C'est parce que .... bah déjà une histoire de coût, et puis moi, j'aime bien les cuire moi-même, etil y a que chez lui que je les trouve crues.

00:12:25
*Speaker 1:* D'accord.

00:12:26
*Speaker 2:* C'est aussi simple que ça.

00:12:27
*Speaker 1:* D'accord. Ok. Alors en termes de fréquence, vous m'avez dit que c'est, vous allez une ou deux fois par mois pour aller chercher du surgelé ?

00:12:44
*Speaker 2:* Ouais

00:12:45
*Speaker 1:* Presque toutes les semaines pour aller au supermarché...

00:12:47
*Speaker 2:* Ouais, moi, toutes les semaines, c'est pas presque c'est toutes les semaines.

00:12:49
*Speaker 1:* C'est toutes les semaines.

00:12:51
*Speaker 2:* Et le primeur, c'est selon.

00:12:52
*Speaker 1:* D'accord.

00:12:53
*Speaker 2:* Le primeur c'est ICCI moi je vais souvent chez ICCI

00:13:00
*Speaker 2:* Donc ouais ok, à côté

00:13:00
*Speaker 2:* Ouais qui est, Nan ICCI "I.C.C.I" hein

00:13:07
*Speaker 1:* I.C.C.I ?

00:13:07
*Speaker 2:* Vous connaissez pas ?

00:13:07
*Speaker 1:* Non [rire]

00:13:07
*Speaker 2:* C'est marrant. Vous êtes à Brest et vous connaissez pas? Nan c'est un magasin qui vend surtout du local

00:13:12
*Speaker 1:* D'accord.

00:13:13
*Speaker 2:* Et qui essaye de vendre fin c'est un ... En fait ils sont [euh] y a juste un ... ils vont chercher direct chez le producteur

00:13:19
*Speaker 1:* D'accord y'a pas d'intermédiaire a part...

00:13:21
*Speaker 2:* Chez eux, je sais que cette saison et que voilà alors après c'est pas toujours donné, mais en ce moment, c'est moins intéressant parce qu'il y a, il y a moins de choix quoi.

00:13:31
*Speaker 1:* Oui.

00:13:32
*Speaker 2:* C'est pas la bonne saison pour plein de choses.

00:13:34
*Speaker 1:* Oui.

00:13:35
*Speaker 2:* Mais sinon, je peux y aller tout en... quand j'ai le temps et que c'est la bonne saison. J'y vais toutes les semaines aussi.

00:13:40
*Speaker 1:* Oui. Daccord. [Euh] Alors vous avez déjà commencé à lister les fruits et légumes que vous achetez, que vous consommez régulièrement. Là l'idée, c'est du coup d'avoir tous les légumes que vous avez l'habitude de vous procurer. Donc vous avez dit les tomates, concombres, carottes.

00:14:03
*Speaker 2:* Mhmh

00:14:03
*Speaker 1:* Betteraves, oignons, ail, pommes de terre.

00:14:05
*Speaker 2:* Mhmh

00:14:07
*Speaker 1:* Champignons, courgettes, aubergines. Est-ce que y en a d'autres comme ca que vous avez, comme vous avez dit sur votre liste, c'est souvent les mêmes choses qui reviennent parce est-ce que y en a d'autres comme ça que vous voyez qui, qui reviennent régulièrement sur le..

00:14:20
*Speaker 2:* [euh] Eventuellement de la salade en sachet.

00:14:23
*Speaker 1:* Ouais.

00:14:24
*Speaker 2:* C'est le truc pas écolo du tout, mais...

00:14:30
*Speaker 1:* Pourquoi vous préférez l'acheter en sachet de votre salade?

00:14:33
*Speaker 2:* Parce que ça me saoule de la préparer [rire] Nan puis on en perd beaucoup si ... on en perd beaucoup de la salade, donc j'en prends peu.

00:14:42
*Speaker 1:* D'accord.

00:14:44
*Speaker 2:* Et [euh] et du coup, là, je sais qu'il n'y aura pas de perte.

00:14:46
*Speaker 1:* ok. C'est pour un côté à la fois pratique,

00:14:50
*Speaker 2:* Ouais je prends que quand je sais que je vais en avoir besoin.

00:14:53
*Speaker 1:* d'accord.

00:14:55
*Speaker 2:* Ca peut m'arriver de craquer pour une belle salade et puis après, je vais la mettre au fond du frigo, je vais l'oublier.

00:14:59
*Speaker 1:* Ouais.

00:14:59
*Speaker 2:* Ca peut m'arriver de prendre un chou fleur de temps en temps.

00:15:05
*Speaker 1:* D'accord.

00:15:05
*Speaker 2:* Mais chez moi, c'est la soupe à la grimace, ça ne marche pas toujours.

00:15:09
*Speaker 1:* Ouais.

00:15:10
*Speaker 2:* [Euh] si il y a des radis, ça, ça m'arrive de prendre à la saison. On sait que ça commence là, les radis depuis Pâques. [euh] En légumes on est là ?

00:15:20
*Speaker 1:* Mh

00:15:23
*Speaker 2:* Roh je vois pas. Après, ça peut m'a... ah si les [euh] comment ca s'appelle [euh] de temps en temps, mais pareil, ça n'a pas beaucoup de succès. C'est un tubercule aussi, mais c'est [euh] c'est blanc. Ah, ça va revenir!

00:15:39
*Speaker 2:* Taupinanbour ?

00:15:40
*Speaker 2:* [euh] Non, c'est plus petit. Ah zut. Ca m'arrive de prendre, fin en fait ça m'arrive des fois de prendre un truc juste parce que ça, ça m'intrigue

00:15:49
*Speaker 1:* Ouais. Ok.

00:15:50
*Speaker 2:* Donc ça peut m'arriver de prendre un truc juste parce que je le vois à létale et que ça me plaît.

00:15:54
*Speaker 1:* D'accord donc c'est pas forcément des choses régulières mais de temps en temps, à l'insctinct.

00:15:58
*Speaker 2:* Voilà tout à fait (rire]

00:16:01
*Speaker 1:* Je vois. [euh] Et en termes de fruits, le (inaudible] , est-ce qu'il y en a que vous achetez régulièrement ou pas ? vous m'avez dit que vous faites aussi en fonction des saisons ?

00:16:12
*Speaker 2:* ouais, bava dire les pommes, les bananes quasiment toute l'année.

00:16:15
*Speaker 1:* D'accord

00:16:16
*Speaker 2:* [euh] les pommes, quasiment pas l'été déjà, parce qu'il y a plein d'autres fruits. Et puis ensuite, parce que c'est comme c'est plutôt l'automne. Voilà.

00:16:24
*Speaker 1:* D'accord

00:16:26
*Speaker 2:* Les banane aussi. La la la bananes, c'est pareil. L'été quand y'a pleind'autres fruits je prends autre chose.

00:16:33
*Speaker 1:* Ok.

00:16:36
*Speaker 2:* [euh] Après [euh] bah c'est selon les saisons, donc là, il y a les fraises, les fraises qui sont déjà là. Mais là, je prends des fraises plutôt du sud ouest pour des questions de coûts, va y avoir... Derrière, il y aura les abricots, les pêches, les melons, tous les fruits d'été. Et puis l'hiver après beaucoup les agrumes.

00:16:55
*Speaker 1:* D'accord.

00:16:58
*Speaker 2:* Là, on a fini en mais là les clémentines, ça a beaucoup de succès chez nous, un peu d'orange, et puis moi j'aime bien le pamplemousse.

00:17:04
*Speaker 1:* D'accord.

00:17:07
*Speaker 2:* Dans les légumes j'ai oublié le, mais c'est un fruit en fait, j'ai oublié l'avocat.

00:17:10
*Speaker 1:* Ok.

00:17:12
*Speaker 2:* Mais pareil, c'est des histoires de saison sera plus l'été qu'on en prendra, ce qui est idiot parce que en fait ca pousse en hiver [rire]

00:17:20
*Speaker 1:* Ouais. D'ailleurs du coup, pourquoi ? [euh] Pourquoi vous achetez plus justement vous en été ?

00:17:28
*Speaker 2:* Pour les salades, les salades composées comme tout le monde?

00:17:31
*Speaker 1:* Ouais.

00:17:32
*Speaker 2:* C'est par préférence là.

00:17:33
*Speaker 1:* Biensûr.

00:17:36
*Speaker 2:* Et les olives aussi tiens, c'est pas frais par contre. Vous cherchez, vous intéressez au frais vous ?

00:17:42
*Speaker 1:* Ouais, oui les olives vous les achetez en ..

00:17:46
*Speaker 2:* En boîte

00:17:49
*Speaker 1:* En conserve. Et si maintenant on s'intéresse aux fruits et légumes que vous consommez, que rarement, voire jamais est-ce ce que vous en avez là, vous voyez quelques légumes, par exemple, que vous connaissez, mais que vous vous n'achetez pas, n'achetez pas?

00:18:05
*Speaker 2:* Bah les fraises de Plougastel.

00:18:07
*Speaker 2:* Les fraises de Plougastel comme vous m'avez dit, ca vous m'avez dit que ...

00:18:10
*Speaker 2:* Bah on consomme des fraises, mais les fraises, ce sont les fraises spécifiquement de Plougastel pour une histoire de coût, simplement. Et j'en achète d'autres. C'est juste pas celle là. [Euh] Je vais éviter tout ce qui vient de loin.

00:18:24
*Speaker 1:* D'accord.

00:18:27
*Speaker 2:* Je ne sais pas, je ne vais pas acheter. Alors, les bananes, par exemple, on n'en fabrique pas. Donc oui, oui, là, je je vais pas... Mais je ne vais pas prendre des pamplemousses qui viennent de, de Floride, si je peux l'éviter ou .. Alors est-ce qu'on en produit en Europe. [euh]C'est pas, il n'y a pas un truc qui me soit rédhibitoire, c'est juste, j'évite de prendre ce qui vient de loin, quel que soit, et j'essaie d'éviter aussi le hors saison ou ce qui a été visiblement traité pour les citrons, par exemple,j'essaie de prendre des citrons non traités. C'est plus. Donc, il n'y a pas de non, c'est vraiment suivant [euh] les modes de, de j'allais dire les modes de fabrication, donc. Après, il y a des trucs que j'achète peu, les mangues, par exemple, j'en achète très peu. Alors que j'adore ça parce que bah ça vient de loin. Souvent, ça a été collecté dans des conditions bofs, quoi voilà. Et un fruit que j'aime beaucoup mais que j'achète jamais c'est le litchi. Parce que comme j'ai de la famille créole, bah le litchi de magasin en France ca n'a rien à voir.

00:19:28
*Speaker 1:* C'est vrai, donc là c'est en termes de goût.

00:19:33
*Speaker 2:* Voilà, voilà le litchi c'est à part et voilà la mangue, l'ananas, j'en achète peu. Alors j'aime ça aussi pour la parce que souvent, ça vient de loin. D'ailleurs, j'en ai un d'ananas cette fois-ci. Mais (euh] c'est [euh] il n'y a pas de trucs où c'est vraiment non.

00:19:49
*Speaker 1:* Ouais.

00:19:49
*Speaker 2:* C'est vraiment, c'est par rapport aux circonstances. Ce qui vient de Papouasie. Nan les bananes de Papouasie, pourquoi pas, mais si on peut, c'est comme quand on vous propose un truc bio et que j'allais dire, on va me proposer des haricots verts bio qui ont été faits en Chine, quoi. Pour moi, c'est antinomique ... la facture énergétique [eu] Voilà.

00:20:13
*Speaker 1:* Ok. [euh] Alors Désormais, j'aimerais qu'on s'intéresse à vos lieux de stockage

00:20:17
*Speaker 2:* Mhmh

00:20:19
*Speaker 2:* Donc des fruits et légumes quand vous revenez de vos courses, que faites vous de vos fruits?

00:20:25
*Speaker 2:* [Euh] Ça dépend lesquels

00:20:26
*Speaker 1:* Ouais.

00:20:27
*Speaker 2:* Ceux qui sont pour consommer rapidement, je les mets sur le compotier ou ceux qui craignent pas le .. Ceux qui crai.. ceux qui ont besoin de ouais non ce pour dans le compotier ceux qui sont à cosommer rapidement après je les stock vous avez vu mon placard dernière fois, je ne sais pas si vous vous souvenez, j'ai un placard dans ma cuisine avec

00:20:47
*Speaker 1:* Ouais la grille.

00:20:48
*Speaker 2:* Des grilles et donc je vais les poser dessus quand ils sont en sac papier, je laisse tel que quasiment

00:20:55
*Speaker 1:* D'accord.

00:20:55
*Speaker 2:* Et [euh] et sinon, je les sort, je les pose. J'essaye de mettre les agrumes à part,

00:21:01
*Speaker 1:* d'accord.

00:21:02
*Speaker 2:* Mais je n'y arrive pas toujours.

00:21:05
*Speaker 1:* Pourquoi?

00:21:07
*Speaker 2:* Parce que faudrait que je leur trouve un endroit spécifique et j'ai pas encore trouvé comment.

00:21:12
*Speaker 1:* D'accord, c'est plus par contrainte de, d'endroit ?

00:21:17
*Speaker 1:* Non c'est parce que je, les agrumes, je pense, ont une influence sur les autres.

00:21:22
*Speaker 1:* D'accord. donc vous, quand vous pouvez vous essayer de les mettre à part, mais c'est pas toujours ...

00:21:27
*Speaker 2:* Non c'est pas toujours possible. C'est que j'ai l'impression que ça fait vieillir les autres fruits plus vite.

00:21:31
*Speaker 1:* D'accord.

00:21:31
*Speaker 2:* C'est, je l'ai lu plusieurs fois et j'ai l'impression que c'est vrai.

00:21:34
*Speaker 1:* D'accord.

00:21:34
*Speaker 2:* Après c'est peut être complètement du truc de bonne femme, mais voilà.

00:21:39
*Speaker 1:* Mais justement, l'idée, c'est justement de voir pour vous qu'est-ce que, quesqu''un stockage idéal par exemple sur les fruits, selon vous? Les agrumes devraient être à part.

00:21:47
*Speaker 2:* Mh

00:21:47
*Speaker 1:* Qu'est-ce que vous avez d'autre comme ça, d'idées de stratégies de stockage qui peuvent être?

00:21:53
*Speaker 2:* [pff] J'évite de mettre au frigo

00:21:55
*Speaker 1:* D'accord. Pourquoi vous ..?

00:21:59
*Speaker 2:* Parce que je trouve, ça casse les goûts, même si on les ressort après, et puis en plus jes les oublies moi au frigo.

00:22:03
*Speaker 1:* Ouais.

00:22:04
*Speaker 1:* C'est là où j'aurais de la perte parce que je vais oublier ça sera dans le tiroir en bas et..

00:22:08
*Speaker 1:* Oui. [rire]

00:22:12
*Speaker 1:* D'où le fait de les mettre, ceux qui se consomment rapidement ..

00:22:15
*Speaker 2:* Visibles.

00:22:15
*Speaker 1:* Vous les rendez visibles. OK. [euh] Et si on s'intéresse maintenant aux légumes de la même façon quand vous revenez de vos courses, que faites vous de vos légumes ?

00:22:26
*Speaker 2:* Alors bêtement je mets les tomates au frigo, là par contre [rire]

00:22:28
*Speaker 1:* D'accord.

00:22:29
*Speaker 2:* Et il m'arrive de les oublier .

00:22:31
*Speaker 1:* [rire] D'accord.

00:22:32
*Speaker 2:* Mais ca à peu de conséquences sur les tomates. Tomates, concombres, ça va au frigo en général.

00:22:35
*Speaker 1:* D'accord.

00:22:39
*Speaker 2:* [euh] Si je veux avoir, je sais pas moi, des courgettes. Pareil pour les courgettes c'est selon. Si pense les faire rapidement, je vais les laisser sorties, sinon je les mets dans le frigo.

00:22:48
*Speaker 1:* D'accord.

00:22:49
*Speaker 2:* [Euh] Et tout ce qui est tubercules, carottes, [euh] pommes de terre, oignons, ail, ça va dans mon placard, sur les grilles.

00:22:58
*Speaker 1:* D'accord. Vous mettez quelques légumes. qu'est-ce qui fait que les tomates concombres.

00:23:05
*Speaker 2:* Parce que c'est plus fragile.

00:23:06
*Speaker 1:* D'accord.

00:23:07
*Speaker 2:* Ce qui paraît plus fragile va au frigo. On va dire.

00:23:10
*Speaker 1:* Oui.

00:23:11
*Speaker 2:* Sauf si je sais que je fais rapidement

00:23:12
*Speaker 1:* D'accord.

00:23:15
*Speaker 2:* Et [euh] ce qui me paraît moins fragile et que je veux garder je vais les mettre les grilles. Du reste, je pense que quand j'achète des légumes, j'achète plutôt des, fin frais, j'achète plutôt des choses qui sont peu fragiles.

00:23:24
*Speaker 1:* D'accord. Et du coup, est-ce que pareil, ce que. Est-ce que, selon vous, c'est une, c'est un stockage idéal ou pas? Est-ce que ?

00:23:36
*Speaker 2:* Je trouve que ça se garde bien là dans vos placards.

00:23:39
*Speaker 1:* D'accord.

00:23:39
*Speaker 2:* C'est ça, ça, l'air circule et du coup, je pense que ça aide à ce que ça se garde.

00:23:45
*Speaker 1:* Ok.

00:23:45
*Speaker 2:* Parce qu'avant c'est dans un tiroir dans mon ancienne maison, c'était dans un tiroir et je pense ca vieillissait quand même plus vite et puis surtout, je voyais moins bien donc ...

00:23:53
*Speaker 1:* Oui.

00:23:53
*Speaker 2:* c'etait moins pratique.

00:23:58
*Speaker 1:* Ok. Et ces lieux de stockage, est-ce que ça vous arrive de les nettoyer. Donc il y'a le frigo? Vous avez une corbeille.

00:24:09
*Speaker 2:* Hm

00:24:10
*Speaker 1:* Et du coup le tiroir à grilles.

00:24:11
*Speaker 2:* Honnêtement ?

00:24:13
*Speaker 1:* honnêtement là y'a pas de ... c'est honnêtement

00:24:17
*Speaker 2:* Honnêtement [euh] quand je vois que c'est sale oui.

00:24:18
*Speaker 1:* Ouais.

00:24:19
*Speaker 2:* Sinon je n'y pense pas.

00:24:20
*Speaker 1:* D'accord.

00:24:21
*Speaker 2:* Et je peux pas vous dire un rythme, ça va dépendre... Voilà ca va être, si j'ai un accident, la corbeille, ça doit m'arriver de temps en temps quand elle est, quand elle est vide.

00:24:32
*Speaker 1:* Ouais.

00:24:33
*Speaker 2:* De lui donner un coup. Mais j'essaie de ne pas avoir de fruits qui pourrissent dessus, ce qui évite d'avoir à aller à la nettoyer en fait. Nan le plus [inaudible], c'est le tiroir du,du frigo, mais finalement, cette saison je l'utilise peu donc j'avais dit la dernière fois que j'allais le laver mais je ne l'ai toujours pas fait par exemple.

00:24:49
*Speaker 1:* D'accord.

00:24:49
*Speaker 2:* Parce que comme je l'utilise peu après bah..

00:24:52
*Speaker 1:* Ouais.

00:24:52
*Speaker 2:* Et sinon le, le placard où je le stocke. En fait [euh], pour l'instant, je n'ai pas vraiment eu besoin parce que comme c'est des, c'est un truc en métal, un petit coup, c'est très vite fait quand il y'a un soucis. Mais j'ai l'impression que ça, vraiment, ça .. comme c'est les tubercules tout ça de toute façon je les épluche derrière.

00:25:14
*Speaker 1:* Ouais.

00:25:15
*Speaker 2:* Après si je vais avoir de la terre, tout ça oui il faut que je nettoie.

00:25:17
*Speaker 1:* D'accord.

00:25:19
*Speaker 2:* Il n'y a pas un je suis pas, je suis pas en train de faire les poussières déjà, vous voyez mon séjour voilà. Et en plus c'est monsieur qui fait le ménage. [rire] Donc, lui, je sais que les placards, ça va pas. Je sais, même si c'est lui qui s'occupe de ça non, il fera pas donc... Déjà, il a du mal à faire le sol, donc [euh] voilà

00:25:38
*Speaker 1:* Donc juste quand vous voyez que c'est sale et que...

00:25:39
*Speaker 2:* Voilà.

00:25:41
*Speaker 1:* Et donc c'est, vous faites comment, vous faites [euh]? quel ? Vous mettez du produit ou c'est un coup d'eau ? comment ?

00:25:48
*Speaker 2:* Ca va dépendre de comment c'est sale [rire]

00:25:50
*Speaker 1:* Je vois.

00:25:52
*Speaker 2:* Le coup de chiffon, c'est rapide, c'est souvent fait.

00:25:54
*Speaker 1:* D'accord.

00:25:54
*Speaker 2:* Avec la poussière tout ça. Coup de chiffon, c'est souvent fait. [Euh] le produit bah [eu] en règle général, quand je vais mettre mes fruits et légumes dessus, j'évite d'en mettre. [Euh] je, j'ai eu ma période vinaigre et finalement, je pense que l'eau chaude, c'est ce qu'il y a de mieux.

00:26:10
*Speaker 1:* Ok.

00:26:11
*Speaker 2:* Et si j'utilise du produit, je vais rincer derrière et ça va être genre ce qu'on appelle monsieur propre et compagnie.

00:26:17
*Speaker 1:* Oui.

00:26:17
*Speaker 2:* Et dans les cas extrêmes, gel la javel, tous les trucs à la javel je mets aussi.

00:26:23
*Speaker 1:* Ok.

00:26:24
*Speaker 2:* Comme derrière, on mange,j'essaie de bien rincier dans ces cas là.

00:26:26
*Speaker 1:* D'accord Alors là, j'aimerais qu'on discute de votre façon de consommer [euh] vos fruits et légumes

00:26:36
*Speaker 2:* Mhmh

00:26:37
*Speaker 1:* Si on prend par exemple un fruit que vous avez dit consommer régulièrement. Donc, la pomme retrouvée toute l'année.

00:26:44
*Speaker 2:* Mhmh

00:26:45
*Speaker 1:* Sous quelle forme vous la consommez au sein de votre foyer?

00:26:47
*Speaker 2:* [Euh] La pomme fraiche au départ ?

00:26:51
*Speaker 1:* Ouais vous avez la pomme, voir un peu toutes les façons que vous avez [euh].

00:26:55
*Speaker 2:* On va faire, alors si c'est de la pomme de notre jardin, on va faire des tartes. Ca c'est le deuxième truc, mais le principal, on la consomme beaucoup, tout bêtement.

00:27:02
*Speaker 1:* Ouais.

00:27:03
*Speaker 2:* En partageant à la fin du dîner quoi.

00:27:07
*Speaker 1:* Ok.

00:27:07
*Speaker 2:* Il n'y a pas, [euh] parce que en fait le truc. C'est là c'est c'est. J'ai un de mes enfants qui a un problème de, avec tout ce qui est fruits et légumes cuits..

00:27:17
*Speaker 1:* D'accord.

00:27:17
*Speaker 2:* .. à la texture. Donc, du coup, avant, je faisais des compotes, des machins, des trucs et finalement, comme lui, il mange bien tout ce qui est cru, aussi bien dans les fruits et légumes que il ne mange pas quand c'est cuit parce qu'il y a des morceaux qu'il ne supporte pas la texture. Bah du coup, maintenant, on fait surtout en cru et au couteau et basta quoi. Parfois, on fait des fruits en salade.

00:27:37
*Speaker 1:* D'accord.

00:27:38
*Speaker 2:* Quand la saison le permet. Fin je dis ca j'en ai fait une, je sais plus, il n'y a pas longtemps

00:27:42
*Speaker 1:* D'accord.

00:27:43
*Speaker 2:* J'ai fait kiwi banane, tiens, je vais vous parler et vous mettre les kiwis dans les fruits que...

00:27:46
*Speaker 1:* Que vous consommez régulièrement

00:27:49
*Speaker 2:* Quand c'est la saison encore une fois.

00:27:51
*Speaker 1:* Quand c'est la saison.

00:27:51
*Speaker 2:* Là on arrive à la fin mais on en avait encore quelques un.

00:27:52
*Speaker 1:* Mh.D'accord donc, concernant là les fruits, davantage crues ?

00:28:01
*Speaker 2:* Oui.

00:28:01
*Speaker 1:* Que cuits ?

00:28:02
*Speaker 2:* Oui

00:28:02
*Speaker 1:* Mais de temps en temps, vous avez. Des fois, ça peut vous arriver de les préparer [euh]

00:28:08
*Speaker 2:* Oui faire une tarte aux pommes, une compote ou des pommes au four.

00:28:13
*Speaker 1:* Ouais. Et quand vous mangez crue [euh] la pomme? Vous faites comment elle est, admettons là vous avez votre pomme vous voulez la manger, qu'est-ce que vous faites comme étapes ?

00:28:22
*Speaker 2:* Alors, ça va dépendre de la taille.

00:28:24
*Speaker 1:* D'accord

00:28:25
*Speaker 2:* Si c'est cette petite pomme, j'enlève juste le milieu et je donne aux enfants.

00:28:29
*Speaker 1:* OK.

00:28:30
*Speaker 2:* Et sinon je vais la couper en quartiers. Puis je vais enlever le milieu et je donne aux enfants. On mange la peau chez nous, puisqu'on prend des pommes qui sont non traitées.

00:28:36
*Speaker 1:* D'accord, vous n'épluchez pas la pomme.

00:28:39
*Speaker 2:* Sauf si en fait, c'est oui ou non c'est à dire que quand c'est moi qui les achète et que je sais qu'elles sont pas traités on mange la peau. Si j'ai, si j'ai le moindre doute, on enlève la peau.

00:28:47
*Speaker 1:* D'accord. Ok.

00:28:54
*Speaker 1:* Et maintenant, si on prend un légume que vous consommez régulièrement donc on a dit, par exemple [euh] la tomate.

00:29:03
*Speaker 2:* Ce n'est pas ce qu'on consomme le plus nous c'est le concombre

00:29:05
*Speaker 1:* Concombre ? Et ben prenons le concombre.

00:29:09
*Speaker 2:* Le concombre ça va être soit. En fait, on fait notre petit apéro dînatoire le vendredi soir, donc là, on le débite en bâtonnets.

00:29:18
*Speaker 1:* Ouais.

00:29:18
*Speaker 2:* Et on le mange comme ça. Sinon, bah je vais les faire, j'allais dire bêtement coupés rondelles avec [euh] avec un petit peu de ciboulette qui est juste là.

00:29:29
*Speaker 1:* Ouais.

00:29:30
*Speaker 2:* Puis du sel et éventuellement un yaourt si j'en ai mais là ce week-end on a fait ca juste avec une petite vi... un petit peu de vinaigrette.

00:29:39
*Speaker 1:* D'accord et la même façon, est-ce qu'il y a des étapes quand vous avez le concombre ?

00:29:45
*Speaker 2:* Bah je vais l'éplucher, puis je vais le débiter selon la façon dont, dont, dont on va le manger derrière.

00:29:50
*Speaker 1:* D'accord.

00:29:53
*Speaker 2:* Mais par contre on l'épluche le concombre en général.

00:29:54
*Speaker 1:* Ouais. Ok. Et si on prend la ce que vous faites avec le concombre, il se mange [euh]?

00:30:01
*Speaker 2:* Que cru

00:30:02
*Speaker 1:* Ouais j'ai du mal à le voir cuit [rire] Et l'action pour la carotte ?

00:30:07
*Speaker 2:* Alors la carotte, quand c'est des carottes fraîches 95 pour cent du tempselles vont, je ne vais pas les cuire.

00:30:11
*Speaker 1:* D'accord.

00:30:14
*Speaker 2:* Si j'en cuit en général, je les prends déjà en surgelés en fait, donc [euh]. Mais ça peut m'arriver d'en mettre dans des ragoûts, des choses comme ça.

00:30:20
*Speaker 1:* D'accord.

00:30:21
*Speaker 2:* Mais sinon, j'allais dire c'est le même traitement que le concombre. On l'épluche, on la débite en bâtonnets pour l'apéro du vendredi soir.

00:30:28
*Speaker 1:* Ouais.

00:30:29
*Speaker 2:* Ou on la râpe pour [euh] pour faire une entrée.

00:30:33
*Speaker 1:* OK.

00:30:33
*Speaker 2:* Ca, c'est ce qu'on fait le plus courant. Et puis, occasionnellement, je m'en sers dans mes ragoûts.

00:30:37
*Speaker 1:* OK. Davantage cru que cuit ?

00:30:42
*Speaker 2:* Ouais pour la même raison que les fruits ouais c'est que...

00:30:45
*Speaker 1:* Avec vos enfants ?

00:30:45
*Speaker 2:* Ouais j'en ai une. Nan les deux autres ça va hein. J'en ai un qui est très, très compliqué pour ça.

00:30:48
*Speaker 1:* D'accord. OK. [euh] Donc, est-ce que vous avez des fruits ou des légumes que vous consommez sans rien éplucher ? Donc on a dit la pomme. Du coup, la pomme il reste le trognon on va dire.

00:31:04
*Speaker 2:* Ouais.

00:31:05
*Speaker 1:* Est-ce que vous avez en tête des fruits et des légumes, que vous, alors il y' avait la framboise [rire] que vous avez dit manger comme ça. Est-ce que vous en avez d'autres en tête que vous n'allez rien toucher.

00:31:14
*Speaker 2:* Bah je sais pas les queues de fraises ca compte ou pas ?

00:31:18
*Speaker 1:* Oui voilà, c'est un fruit ou un légume où en fait, on n'enlève rien dessus, on le mange

00:31:23
*Speaker 2:* Bah je sais pas même des cerises, on mange pas de noyau, donc [euh]. Qu'est-ce qui aurait ? Pas le Kiwi y'a de la peau..

00:31:31
*Speaker 1:* Ouais. ?

00:31:35
*Speaker 2:* [euh] Le fruit de la passion, ça fait partie des trucs que je prends, même si ça vient de loin pareil y'a de la peau.

00:31:40
*Speaker 1:* Mh

00:31:42
*Speaker 2:* Bah forcément, quasiment a un noyau ouquelque chose. Peut être la mûre quand on en cueille.

00:31:46
*Speaker 1:* Ouais.

00:31:49
*Speaker 2:* Mais des fruits ou il y a zéro déchet [euh] Non, je ne vois pas trop. Non. Je réfléchis là spontanément, il n'y a rien donc [euh] si je dois répondre à la question.

00:32:01
*Speaker 1:* C'est justement la question qu'on se demande est-ce qu'il y a des fruits ou légumes ?

00:32:07
*Speaker 2:* Ah j'avais trouvé la framboise.

00:32:08
*Speaker 1:* La framboise effectivement [rire]

00:32:16
*Speaker 2:* [inaudible la Framboise. Nan je réfléchis. [euh] ouais j'ai envie de dire la fraise des fois les cueille même sans les queues ici.

00:32:19
*Speaker 1:* Ouais.

00:32:20
*Speaker 2:* C'est comme la Franboise quand on les cueille les fraises

00:32:23
*Speaker 1:* C'est des choses qu'on vient cueillir du coup même

00:32:26
*Speaker 2:* Ouais, c'est des trucs comme ça ouais. La presse tourne avec des pommes dans... Bah les groseilles, mais pareil les groselles y'a toujours des trucs entre les dents. Ah le raisin pareil va y'avoir la tige.

00:32:38
*Speaker 2:* Mh. Ouais

00:32:40
*Speaker 2:* Non je vois pas trop.

00:32:43
*Speaker 1:* Très bien. Alors là, j'aimerais qu'on discute du moment où vous, vous jetez un fruit ou légumes ou une partie seulement.

00:32:53
*Speaker 2:* Je l'ai fait hier, je l'ai fait hier

00:32:54
*Speaker 1:* [rire]

00:32:57
*Speaker 2:* Si vous , vous devez vous dire que je vous oublie nan ?

00:33:00
*Speaker 1:* Je regarde juste avant les, j'ai vu, il y avait des choses [euh]. Mais non, on sait bien que ce n'est pas forcément, on va pas jeter tous les jours.

00:33:08
*Speaker 2:* [rire] ah hier j'ai quintuplé le poids de la poubelle parce que j'ai jeté des morceaux de pomme. J'étais furieux en plus.

00:33:15
*Speaker 1:* Ah voilà, et du coup, qu'est-ce qui fait que vous décidez de le jeter plutôt que de le consommer à ce moment là ?

00:33:20
*Speaker 2:* [euh] bah quand c'est bien pourri, clairement.

00:33:23
*Speaker 1:* D'accord. Ok. Et comment vous déterminez qu'un fruit ou un légume justement est pourri ?

00:33:34
*Speaker 2:* [euh] A la couleur.

00:33:35
*Speaker 1:* A la couleur ?

00:33:35
*Speaker 2:* A la couleur et puis au toucher aussi.

00:33:36
*Speaker 1:* Au toucher ?

00:33:37
*Speaker 2:* Plus rarement au goût.

00:33:41
*Speaker 1:* Plus rarement au goût.

00:33:41
*Speaker 2:* Ça arrive, mais ça, c'est rare qu'au goût on dise "oulah"

00:33:48
*Speaker 1:* Ouais. Et ça, quand ça vous arrive, admettons, vous avez pas remarqué avant que le fruit ou légume était pourri ? Et que vous consommez comment vous réagissez quand vous avez du [inaudible] dans la bouche ?

00:34:00
*Speaker 2:* Moi, j'avale stoïquement mes enfants recrachent sans aucune [rire] bienséance.

00:34:08
*Speaker 1:* Je vois.

00:34:11
*Speaker 2:* "Oh c'est dégoûtant maman"

00:34:13
*Speaker 1:* [rire]

00:34:13
*Speaker 2:* Nan mais c'est arrivé là, on avait des ... qu'est-ce que c'était, des [inaudible] , non ? des tangerines.

00:34:21
*Speaker 1:* Je ne sais aps ce que c'est.

00:34:21
*Speaker 2:* C'est un agrume encore.

00:34:23
*Speaker 1:* D'accord.

00:34:23
*Speaker 2:* Et l'un de mes enfants qui pourtant d'habitude il n'a pas de soucis à trouver que c'était pas bon donc il a tout craché, mais en fait, bah c'est moi qui ai mangé parce que pour moi, il n'y a pas de soucis.

00:34:33
*Speaker 1:* D'accord.

00:34:34
*Speaker 2:* Voilà, mais.

00:34:37
*Speaker 1:* Et quand vous êtes face à du, quand vous du coup, visuellement, quand vous voyez du pourri [euh] qu'est-ce que vous ressentez ?

00:34:48
*Speaker 2:* [Euh] Bah je suis pas contente [rire]

00:34:51
*Speaker 1:* Ouais.

00:34:51
*Speaker 2:* [euh] Non, je ne ressens pas grand chose, je me dis juste c'est con en fait.

00:34:54
*Speaker 1:* Ok.

00:34:54
*Speaker 2:* C'est con j'aurais dû le manger plus tôt ou je l'ai mal choisi.

00:34:58
*Speaker 1:* Ok.

00:35:01
*Speaker 2:* C'est plutôt ce genre de réflexion, mais il n'y a pas de dégoût si c'était ça la question.

00:35:04
*Speaker 1:* Mh

00:35:05
*Speaker 2:* C'est extrêmement rare qu'il y a du dégoût. Ça m'est arrivé une fois avec un choux qu'on avait oublié et où j'ai mis mon doigt et mon doigt s'est enfoncé et en fait, on a pensé... Voilà. Ca oui, parce que c'est c'est un dégoût par rapport au toucher, au fait qu'il y avait des bouchons partout et tout ça

00:35:20
*Speaker 1:* D'accord.

00:35:21
*Speaker 2:* Sinon non, il n'y a pas de dégoût.

00:35:24
*Speaker 1:* S'il y a du dégoût, ce serait davantage au toucher que vous en aurez...

00:35:28
*Speaker 2:* Ouais.

00:35:28
*Speaker 1:* .. Que visuellement ?

00:35:30
*Speaker 2:* Nan visuellement.

00:35:31
*Speaker 1:* Ouais. Ok.

00:35:34
*Speaker 2:* Mais c'est très rare, mais il en faut quand même.

00:35:37
*Speaker 2:* Ouais.

00:35:38
*Speaker 2:* Non mais c'est plus voilà. Parfois, ça peut être de l'exaspération si c'est un truc que j'ai que j'ai acheté récemment. Là, le coup d'hier, c'était de l'exaspération, clairement.

00:35:48
*Speaker 2:* Oui.

00:35:49
*Speaker 2:* C'était une pomme qui avait une bonne allure et puis je coupe. Et je vois qu'elle été noircie à l'intérieur. Ca veut dire déjà que un, contrairement à ce que je pensais, elle a probablement été traité et deux bah voilà quoi, il y a du gâchis quoi.

00:36:02
*Speaker 1:* Ouais.

00:36:06
*Speaker 1:* Alors, justement. On va parler du gaspillage alimentaire. [euh] Quand je vous dis gaspillage alimentaire, quels sont les mots qui vous viennent spontanément à l'esprit ?

00:36:18
*Speaker 2:* Stupidité.

00:36:20
*Speaker 1:* D'accord.

00:36:20
*Speaker 2:* Voilà [euh] manque de réflexion.

00:36:26
*Speaker 1:* Ok.

00:36:28
*Speaker 2:* Ce qui revient au même en fait aussi ?

00:36:32
*Speaker 2:* Et [euh] égoïsme

00:36:35
*Speaker 1:* Ok. Alors pourquoi avoir choisi stupidité ? selon vous ?

00:36:43
*Speaker 2:* Parce que je pense que on, on est une société qui est totalement idiote là dessus, c'est à dire que on surconsomme et le gaspillage alimentaire fait partie de cette surconsommation. [euh]C'est, c'est comme quand j'explique à mes enfants ils me disent oui, la viande, parce que c'est de la viande, c'est un exemple assez parlant. Oui on tue des animaux pour se nourrir.

00:37:07
*Speaker 2:* Bah oui.

00:37:08
*Speaker 2:* Et à partir du moment où l'animal on le mange, la mort de cet animal est justifiée puisque c'est une façon de nous nourrir, nous sommes omnivores. Et donc, par contre, si on jette [euh] de la viande, ça veut dire que l'animal est mort pour rien. Et donc y'a un énorme gâchis, c'est le gâchis d'une vie. C'est un peu la même chose avec les fruits et légumes. C'est moins parlant comme exemple, parce que c'est voilà mais y'a quand même ce fruit il a poussé, ça a pris du temps et il a grandi. Il y a quelqu'un, peut être qui l'a planté, etc. Et donc tout ce travail là et bah il va être perdu.

00:37:40
*Speaker 1:* Mh

00:37:41
*Speaker 2:* Et [euh] et ça, c'est sans compter de au fait que bah peut être que si nous, on l'avait pas acheté, que quelqu'un d'autre aurait pu être nourri avec. C'est pour ça que je dis c'est stupide et qu'il faudrait éviter tout plein de choses derrière auxquelles nous, bah comme on n'a qu'à ouvrir le frigo pour manger, on pense pas mais, mais qui restent vraies ?

00:38:01
*Speaker 1:* Ouais. Vous avez dit manque de réflexion?

00:38:06
*Speaker 2:* Bah c'est un peu ça, c'est, il faut respecter aussi [euh] le, respecter ce que c'est la nourriture, ce n'est pas un dû contrairement à ce qu'on a l'air de penser dans notre société. Je ne pense pas que ce soit dû.

00:38:19
*Speaker 1:* Ouais.

00:38:19
*Speaker 2:* Et [euh] donc bah faut respecter déjà le travail qu'il y a derrière, les investissements qu'il y a derrière, l'argent que nous-même on y met.

00:38:27
*Speaker 2:* Ouais.

00:38:27
*Speaker 2:* Et puis, et puis, être raisonnable dans sa consommation, peut être réfléchir à tout ce que ça induit à, notre consommation.

00:38:37
*Speaker 1:* Ouais.

00:38:38
*Speaker 2:* Là, il y a, il y a tous les ans, il y a des exemples et on continue à avancer très vite. Là le coup du porte conteneurs qui a bouché l'économie mondiale, c'est quand même quelque chose. Faut que ça rentre dans les cerveaux et ça ne marche pas quoi.

00:38:51
*Speaker 1:* Mh.

00:38:53
*Speaker 2:* On a plein d'avertissements donc violà. Et la nourriture, ça fait partie de ça. C'est à dire que quand on veut acheter un truc qui a transité par bateau pendant des semaines, on contribue à la pollution de la planète. Donc, si en plus on achète un truc, je veux dire on va reprendre le coup.. je ne sais pas moi va acheter des fraises qui ont poussées, qu'on a ammenées par avion depuis l'Amérique du Sud.

00:39:15
*Speaker 1:* Mh.

00:39:17
*Speaker 2:* [euh] On va les acheter, puis après les jeter, ça n'a aucun sens.

00:39:19
*Speaker 2:* Ouais.

00:39:21
*Speaker 2:* C'est on va, en fait on contribue à la pollution en faisant ça. Je sais pas si y'a des fraises en Amérique du Sud, c'est pas forcément un bon exemple parceque les fraises en hiver, elles viennent plutôt de, de Hollande, mais c'est pareil puisque ces fraises, elles auront poussées dans des serres chauffées. Donc ça veut dire qu'on a pollué,fin on a chauffé des serres pour avoir des fraises à un moment qui n'est pas le bon. Ces fraises là, en plus, n'auront pas le goût qu'elles ont si elles ont poussées grâce au soleil. Donc Un elles sont moins bonnes. Et Deux si en plus on les jette derrière, c'est un non-sens total.

00:39:46
*Speaker 1:* Mh.

00:39:48
*Speaker 2:* Donc voilà c'est vraiment pour ça, [euh] c'est vraiment pour ça que je fais attention fait.

00:39:52
*Speaker 1:* Mh.

00:39:52
*Speaker 2:* Et ce n'est pas que pour les fruits et légumes, c'est, c'est pour tout ce qu'on consomme. C'est [euh] voilà c'est parfois pouvoir, préférer le, le seconde main plutôt que d'aller demander à quelqu'un de reproduire un truc et de refaire.

00:40:05
*Speaker 1:* D'accord.

00:40:09
*Speaker 2:* C'est plutôt un truc général ca. Votre étude, c'est qu'un tout petit bout de, d'un cheminement global que j'ai.

00:40:15
*Speaker 1:* Complètement. Donc vous avez dit égoïsme aussi ?

00:40:21
*Speaker 2:* Parce qu'on en a rien à foutre que y'a des petits enfants en Chine qui travaillent pour qu'on... qui récoltent les petits haricots verts pour les mettre en surgelés dans un conteneur frigorifiés de Chine jusqu'ici. Très peu de gens sachent. Quand je dis que la Chine est le premier producteur de haricots verts, personne me croit. Personne ne me croit, en disant que ce n'est pas possible ils ont pas le climat. Bah vu la taille du pays si ils ont le climat.

00:40:46
*Speaker 1:* Mh.

00:40:48
*Speaker 2:* Et j'avais fait l'expérience il y a quelques années j'avais mis au défis de trouver la provenance des fruits et légumes de marque Leclerc. En surgelé hein.

00:40:55
*Speaker 1:* Mh.

00:40:56
*Speaker 2:* C'est impossible.

00:40:56
*Speaker 1:* Ah oui ?

00:40:57
*Speaker 2:* Bah essayez.

00:40:59
*Speaker 1:* Je me suis jamais prêté au jeu.

00:41:02
*Speaker 2:* Essayez.

00:41:07
*Speaker 1:* Pour l'égoisme c'est que les personnes ..

00:41:09
*Speaker 2:* Qui n'en ont rien à foutre de savoir d'où ça vient, du moment qu'ils l'ont

00:41:12
*Speaker 1:* D'accord.

00:41:14
*Speaker 2:* Voilà, pour parler clairement.

00:41:15
*Speaker 1:* Ouais.

00:41:16
*Speaker 2:* C'est que on pense pas à tout ce qu'il y a derrière et on ne cherche même pas à se poser la question.

00:41:22
*Speaker 1:* Oui.

00:41:24
*Speaker 2:* Et c'est peut être des questions qu'on devrait se poser un petit peu de temps . Et pas que pour les fruits et légumes.

00:41:30
*Speaker 1:* Oui.

00:41:32
*Speaker 2:* Et même pour Amazon, il faut se poser la question qu'est-ce qu'il y a derrière Amazon? C'est tout un cheminement global. c'est, oui, Amazon, c'est pratique. Moi-même, je suis cliente d'Amazon, mais. Peut être que, ca induit des choses. Voilà c'est comme les gens qui sont contents qu'il y ait des automates aux péages. Les gens, il y avait des gens qui travaillaient aux péages. Que font ils maintenant? Donc c'est un cheminement, c'est une réflexion globale, qu'on a au niveau de j'allais dire, mon mari et moi, c'est une réflexion globale qu'on a sur un peu tout c'est ... Ce n'est pas uniquement les fruits et légumes, les fruits légumes, ça me concerne, mon mari sera plus concerné sur trop acheté, je sais pas moins de DVD ou disques ou de livres, [euh] ou de meubles, ou d'accessoires.

00:42:16
*Speaker 1:* Ouais.

00:42:19
*Speaker 2:* Mais là dessus, c'est une réflexion vraiment globale, familiale on va dire.

00:42:31
*Speaker 1:* Ok. [euh] Si on, si vous deviez choisir qu'un mot entre ceux que vous avez dit entre la stupidité, manque de réflexion, égoïsme. Si, le plus important pour vous vous parlez de gaspillage alimentaire, ce serait lequel ?

00:42:45
*Speaker 2:* Egoïsme.

00:42:46
*Speaker 1:* Egoïsme ?

00:42:47
*Speaker 2:* Parce que ça, c'est ce qui implique ,ce qui a entraîne deux autres je pense.

00:42:54
*Speaker 1:* Ok. Ok. Et si on tentait de faire une définition du gaspillage alimentaire? Si vous vous deviez rédiger dans Le Petit Robert une définition, qu'est-ce qu'on y verrait après, Gaspillage alimentaire?

00:43:08
*Speaker 1:* [Euh] Bonne question.

00:43:09
*Speaker 1:* Ouais, avec vos propres mots [inaudible] ?

00:43:12
*Speaker 2:* Oui, je sais bien [euh] . Ça va être [rire] le fruit d'un manque de réflexion au moment de l'achat.

00:43:22
*Speaker 1:* Ok. Très bien.

00:43:26
*Speaker 2:* Ca pourrait être très court.

00:43:27
*Speaker 1:* Hm.

00:43:33
*Speaker 1:* Ok. J'ai uen question vous l'avez déjà évoquée quand vous réalisez que vous jetez un fruit et légumes à la poubelle qu'est-ce que vous ressentez ?

00:43:43
*Speaker 2:* Vous m'avez déjà posé la question.

00:43:44
*Speaker 1:* Ouais. Alors c'était, l'autre question elle était plus sur le fait que si y'a du moisis devant. Et là le fruit et légume vous le jetez.

00:43:50
*Speaker 2:* Ah oui non. Parce qu'en fait, effectivement, le fait que soit moisi, ça n'a aucun effet sur moi.

00:43:53
*Speaker 2:* C'est ça.

00:43:53
*Speaker 2:* C'est pour ça que, bah c'est vraiment de l'exaspération parce que je m'en veux d'avoir quelque part je m'en veux d'avoir mal géré. C'est vraiment ça. Je me sens stupide, je me sens stupide d'avoir mal géré. J'ai mal géré le truc voilà. Et parfois un peu de colère face au fournisseur qui a des produits de mauvaise qualité.

00:44:15
*Speaker 1:* Ouais.

00:44:17
*Speaker 2:* Et du reste, ICCI , la le primeur, il est très bien. Ca m'est arrivé une fois ou deux de lui dire, "Vous savez, vos pêches bah j'ai dû en jeter". Et en général, il tient compte de ça parce que, il demande d'avoir des retours clients.

00:44:29
*Speaker 1:* Oui.

00:44:29
*Speaker 2:* Parce que quand, lui il ne voit pas forcément quand le fruit n'est plus bien. Ça lui permet d'adapter aussi son offre. Donc, ça m'arrive de parler avec le fournisseur sans aucune agressivité attention.

00:44:43
*Speaker 1:* [rire] La scène [rire]

00:44:44
*Speaker 2:* Ça m'arrive de faire une réflexion en disant "vous savez vos pêches". Ça m'est arrivé là c'était la fin de saison des pêches, de dire "ouais les pêches là je vais pu en repredre parce que la dernière fois, il y a.."

00:44:52
*Speaker 1:* Ouais. Biensûr ouais.

00:44:58
*Speaker 1:* D'accord. Alors là, pour terminer [euh] on va pouvoir discuter de la poubelle connectée, son utilisation [euh] générale. Vous l'avez depuis quelques jours maintenant, et racontez moi comment ça se passe, est-ce que vous avez utilisé fréquemment ou au contraire que très peu ?

00:45:16
*Speaker 2:* [rire] Bah je suis content parce que j'utilise très peu.

00:45:18
*Speaker 1:* Ouais, Ok. Donc, vous avez, vous avez jeté vous m'avez dit des choses hier. Donc une pomme.

00:45:35
*Speaker 2:* Non pas une pomme.

00:45:35
*Speaker 1:* Des bouts de pomme ?

00:45:37
*Speaker 2:* Des bouts de pomme.

00:45:37
*Speaker 1:* Des bouts de pomme.

00:45:37
*Speaker 2:* Attention c'est important, des bouts de pommes.

00:45:37
*Speaker 1:* Tout à fait des bouts de pommes.D'ailleurs qu'avezz vous fait des autres bouts de pomme

00:45:38
*Speaker 2:* ? J'ai mang, j'ai mangé le reste de la pomme.

00:45:41
*Speaker 1:* D'accord. .

00:45:44
*Speaker 2:* J'allais pas nous jeter la pomme entière.

00:45:44
*Speaker 1:* Ouais ok.

00:45:46
*Speaker 2:* Je pense que vous en avez d'autres qui ne sont pas comme ça, mais moi, c'est il fallait pas jeter la pomme.

00:45:49
*Speaker 1:* Vous n'avez enlevé que ce que vous trouviez trop...

00:45:51
*Speaker 2:* Bah que la partie voilà. Et j'en ai peu plus d'ailleurs que ce que j'aurais peut être fait s'il n'y avait pas eu la poubelle connectée je me suis dit aller comme ca on leur fait plaisir. [rire] [inaudible] Parce qu'ils vont croire que je ne pense pas à eux.

00:46:07
*Speaker 1:* Dans mes questions et cette idée là aussi, est-ce que ca vous ait arrivé de l'oublier ou non et dans quel cas c'est pas grave, et qu'on sache s'il y a des personnes qui en fait ont...

00:46:17
*Speaker 2:* Non j'ai pas, j'ai pas oubliée.

00:46:17
*Speaker 1:* Vous ne l'avez pas oubliée ?

00:46:17
*Speaker 2:* Je ne l'ai pas oubliée.

00:46:17
*Speaker 1:* Super.

00:46:17
*Speaker 1:* Et donc vous avez jeté quelques petits bouts de pomme. Vous vous souvenez avoir jeté autre chose ou pas?

00:46:27
*Speaker 2:* [euh] J'ai du jeter, je sais que j'ai jeté un bout de fruit, mais je sais plus que c'était peut être un bout de kiwi une fois.

00:46:31
*Speaker 1:* Ouais.

00:46:33
*Speaker 2:* Et l'autre fois c'était un bout de tomate.

00:46:35
*Speaker 1:* Et pour quelle raison vous les avez [euh]

00:46:39
*Speaker 2:* Bah elles étaient plus très mangeables.

00:46:41
*Speaker 1:* D'accord.

00:46:41
*Speaker 2:* Et à chaque fois, le reste du fruit, par contre, a été consommé.

00:46:44
*Speaker 1:* OK, c'était que des parties à chaque fois..

00:46:46
*Speaker 2:* Ouais le reste aa été consommé, j'ai coupé la partie pas bonne et puis le reste on l'a mangée.

00:46:50
*Speaker 1:* Et selon vous, quelles sont les raisons pour lesquelles parfois vous êtes amenés à jeter, à perdre des fruits et légumes? Ou, est-ce qu'il y'a une raison ? Ou des raisons d'ailleurs ?

00:47:04
*Speaker 2:* (euh]Ben oui, un manque de réflexion à l'achat, ce que je faisais déjà plusieurs fois. Mais [euh] ça va être.. là par rapport à ce que jette, là clairement, c'est que le kiwi, on est en fin de saison, donc il va pas forcément tenir comme je l'aurais pensé.

00:47:18
*Speaker 1:* Oui.

00:47:19
*Speaker 2:* pour Kiwi. La tomate, j'ai pas fait gaffe. Au moment de l'acheter, elle était déjà marquée.

00:47:24
*Speaker 1:* Ok.

00:47:26
*Speaker 2:* Et la pomme [euh]? Je pensais que je l'avais achetée il y a 2 ou 3 jours, mais nan c'était peut être une semaine ceci dit. La pomme c'était une mauvaise surprise la pour le coup [euh] voilà.

00:47:41
*Speaker 1:* Ok. Est-ce que vous avez le sentiment ou pas qu'il y a des fruits et légumes qu'on, qu'on, qu'on jette plus facilement et qu'on perd plus vite que les autres? Est-ce que vous avez?

00:47:53
*Speaker 2:* Alors [euh] oui. En fait, je pense qu'il y a des fruits qu'il faut manger rapidement quand on les achète.

00:47:59
*Speaker 1:* D'accord.

00:48:01
*Speaker 2:* C'est vraiment il y a des fruits, il faut pas. Voilà, c'est quand je vous disais qu'il m'arrive d'en acheter dans la semaine c'est cause de ça. Et la banane en fait partie chez moi. Après moi, je peux manger de bananes anciennes, mais comme les autres, non, je ne vais pas manger tout le régime.

00:48:13
*Speaker 1:* Oui.

00:48:13
*Speaker 2:* En part... Donc la banane, la fraise aussi,il faut la ranger rapidement.

00:48:17
*Speaker 1:* Ouais.

00:48:20
*Speaker 2:* Il y a des fruits, vraiment, il faut les manger rapidement donc je les achète quand je sais que je vais les manger rapidement.

00:48:26
*Speaker 1:* C'est ça.

00:48:28
*Speaker 2:* C'est surtout ça, et puis des fois, il y a des mauvaises surprises quoi là, ça, on n'y peut rien. C'est ce que , c'est le coup du kiwi [euh] parce que, où du lot de kiwi,

00:48:39
*Speaker 2:* où j'ai pas vu qu'il y en avait un pourri. Ca m'est arrivé des clémentines aussi. On trouve un lot, on n'a pas vu, il y en a un qui est pas beau je me suis faite avoir, mais. Comme je jette peu, je ne suis pas forcément la mieux placée pour vous dire.

00:48:51
*Speaker 1:* Mh [rire] très bien.

00:48:55
*Speaker 1:* [Euh] Est-ce qu'il vous est déjà arrivé de vous poser des questions avant ou après avoir jeté dans la poubelle du style est-ce que ça, ça va dedans ou pas? Ou est-ce que non on sait...

00:49:04
*Speaker 2:* Dans votre poubelle ?

00:49:05
*Speaker 1:* Ouais dans la poubelle connectée ? Non tout était ?

00:49:08
*Speaker 2:* Oui c'était clair.

00:49:09
*Speaker 1:* Ok. Et du coup, vous avez changé de sac poubelle ou pas ?

00:49:13
*Speaker 2:* Non. Il y'a 4 morceux de trucs qui se battent au fond.

00:49:13
*Speaker 1:* [rire] OK. ca marche. J'ai posé toutes mes questions. Merci beaucoup, d'avoir répondu. Néanmoins, s'il y a des choses sur lesquelles vous aimeriez revenir, des choses que je n'aurais peut être pas pensé?

00:49:31
*Speaker 2:* Non [euh], juste je pense que ce n'est pas forcément la bonne. Ce n'est pas la saison. J'en le plus de gâchis.

00:49:37
*Speaker 1:* Oui, je sais ça

00:49:40
*Speaker 2:* Je pense que quand genre les pêche, les brugnon, tout ça quand je les fais en salade, c'est là où je peux avoir des choses à ajouter.

00:49:47
*Speaker 1:* Ouais.

00:49:48
*Speaker 2:* C'est plus les fruits d'été qui, ca sera intéressant de voir cet automne, puisqu'il y'aura encore quelques fruits d'été. Je crois que c'est plus les fruits d'été qui entraînent de la perte que les fruits d'hiver. Après, on verra si j'ai raison ou pas.

00:50:00
*Speaker 1:* Mh

00:50:00
*Speaker 2:* Ca sera intéressant pour nous deux.

00:50:03
*Speaker 1:* Ca on va voir, en septembre, on revient.

00:50:06
*Speaker 2:* Oui, donc ca sera intéressant pour nous deux de voir ça. Donc, à cette saison, ce sont des fruits qui sont assez assez, assez costauds, donc finalement peu. En achetant qu'une fois par semaine, j'ai pas trop de soucis.

00:50:15
*Speaker 1:* Ouais.

00:50:16
*Speaker 2:* Parce que ça peut m'arriver de prendre mes bananes le vendredi, on va aller manger le samedi, par exemple, après il n'y aura plus de banane dans la semaine. Là, cette semaine, j'en ai racheté à nouveau parce que je sais qu'ils aiment ça

00:50:25
*Speaker 1:* Ouais.

00:50:25
*Speaker 2:* Que je tiens à ce qu'ils aient un fruit ou un légumes frais tous les jours au dîner.

00:50:31
*Speaker 1:* Ok.

00:50:32
*Speaker 2:* Donc voilà, mais. C'était la question, c'est par rapport au gâchis, c'est ca ? une chose à redire ?

00:50:39
*Speaker 1:* Oui voilà c'était si vous pensez que la saison, du coup, n'est pas, c'est pas la plus propice c'est pas celle qui va déterminer le plus le gaspillage.

00:50:45
*Speaker 2:* Je ne pense pas, après je serais curieuse de voir justement par votre étude, de le voir, parce que c'est aussi pas la saisonou j'ai plus de frais.

00:50:52
*Speaker 1:* Oui, c'est ça qui est forcément [euh] effectivement..

00:50:56
*Speaker 2:* Et puis l'autre jour, je me faisais la réflexion. Je parlais du fait que quand je faisais une compote [euh] une confiture de fraises, j'avais du déchet. En fait, c'est du déchet type épluchage ca rentre pas dans votre gaspillage parce que si je fais une confiture de fraises, je récupère des fraises dont je sais qu'elles sont déjà en partie gâtée.

00:51:11
*Speaker 1:* Oui.

00:51:12
*Speaker 2:* donc. Du coup, c'est des choses que je n'aurais pas consommé de toute façon donc du coup, oui j'essaye d'avoir de toute façon très peu de déchets, donc j'espère que ça va continuer à pas remplir la poubelle.

00:51:22
*Speaker 1:* Oui, donc c'est ca quand vous faites, quand vous faites une, une confiture vous allez prendre des fruits du coup qui sont...

00:51:32
*Speaker 2:* déclassés.

00:51:35
*Speaker 1:* D'accord, et dela... Est-ce que du coup, est-ce que vous vous dites j'ai envie de faire une confiture, Je vais regarder celles qui sont abîmées, fin les plus, qui sont déjà un peu fatiguées. Ou bien c'est parce que vous voyez des fatigués que vous vous dites je vais faire une confiture du coup ?

00:51:50
*Speaker 1:* C'est plus le deuxième.

00:51:50
*Speaker 1:* D'accord c'est pas, c'est du coup une stratégie de... elles sont en train de se perdre, il faut en faire...

00:51:54
*Speaker 2:* [euh] oui sauf que c'est aussi, c'est par exemple chez les fraises, par exemple, on parlait des fraises de Plougastel.

00:51:59
*Speaker 1:* Ouais.

00:52:01
*Speaker 2:* [Euh] le primeur ou je les achète, ou je les achète pas d'ailleurs [rire] 15 euros le kilo [inaudible]

00:52:09
*Speaker 2:* Il va avoir sur côté des cartons de fraises à confiture.

00:52:13
*Speaker 1:* D'accord.

00:52:13
*Speaker 2:* Et c'est souvent par trois kilos et ça va être pas très cher. Fin ca va être un prix acceptable.

00:52:18
*Speaker 2:* Ok.

00:52:18
*Speaker 2:* Et pour tout vous dire, quand je vais faire ma confiture, je vais récupérer dedans les fraises qui sont en bon état, je vais les mettre de côté pour qu'on les consomme normalement.

00:52:26
*Speaker 2:* Oui.

00:52:27
*Speaker 2:* Et c'est celles qui sont abîmées que je vais mettre en confiture.

00:52:32
*Speaker 2:* Et c'est pareil pour les abricots, d'ailleurs.

00:52:33
*Speaker 1:* OK.

00:52:33
*Speaker 2:* C'est surtout ces deux là que je fais en confiture, c'est je vais pas acheter des fruits à confiture. Et puis je vais mettre. J'ai récupéré dedans

00:52:42
*Speaker 1:* C'est un peu l'inverse de...

00:52:44
*Speaker 2:* Je vais récupérer dedans ceux qui sont consommables normalement, c'est souvent pour les abricots plus des défauts d'aspect qui font qu'ils sont déclassés.

00:52:49
*Speaker 1:* D'accord.

00:52:49
*Speaker 2:* Donc moi un abricot qui a l'air d'avoir, qui a je sais pas moi qu'il y a un petit poc c'est pas, c'est pas de la pouriture. Si il a eu un petit coup de bec ou si il va avoir, je sais pas moi, des sortes de taches de rousseur est souvent mis de côté parce qu'ils sont pas beaux, mais ils sont très biens.

00:53:06
*Speaker 1:* Oui.

00:53:07
*Speaker 2:* C'est vraiment l'inverse en fait.

00:53:08
*Speaker 1:* Ok. C'est acheter des...

00:53:10
*Speaker 2:* Faire une bonne affaire. Acheter des abricôts à confiture et les manger normalement.

00:53:15
*Speaker 1:* C'est ca. C'est, OK.

00:53:16
*Speaker 2:* Je suis curieuse de savoir si vous en avez d'autres des comme ça [rire]

00:53:33
*Speaker 1:* Pour l'instant j'en avais pas enttendu. Mais [euh] moi je ne savais même pas qu'il y'avait des fruits déclassés.

00:53:33
*Speaker 2:* Si si ça peut s'acheter.

00:53:33
*Speaker 1:* Alors que si en effet ils sont juste moins beaux mais ils sont pas encore... i

00:53:35
*Speaker 2:* Les abricots typiquemen c'est un effet d'aspect. Et par contre les fraises c'est vraiment quand elles sont déclassées. Si sur trois kilos de fraises, je récupère 800 grammes, c'est déjà très, très bien.

00:53:45
*Speaker 1:* Ouais. Ouais je vois.

00:53:49
*Speaker 1:* Des histoires de taille pour les fraises aussi parce qu'elles sont trop petites ou trop grandes.

00:53:55
*Speaker 1:* C'est une stratégie, la stratégie, mais ça marche. Est-ce que vous d'autres

00:54:02
*Speaker 2:* Non mais je suis curieuse de savoir les autres, comment

00:54:05
*Speaker 1:* Comment ils gèrent, ca va être l'idée.

00:54:09
*Speaker 2:* Et je suis curieuse de voir ce que font les autres pour savoir si je suis vraiment dans un extrême ou s'il y a d'autres personnes qui ont le même type de cheminement?

00:54:16
*Speaker 1:* C'est ça. L'idée ca va être justement d'interroger tout le monde et de comprendre toutes les stratégies ou toutes les réflexions aussi, et toutes les pratiques différentes. Et puis vous situer après peut être, on pourra vous dire, une fois que ...

00:54:31
*Speaker 2:* Et ca m'amusera de savoir...

00:54:32
*Speaker 1:* Ouais voilà c'est ça, c'est ça on vous le dira.

**Household F08, Interview 2**

*Speaker 2:* [Inaudible] Alors, on a des petits supports. Ça, c'est à moi. Ça [inaudible]. Je vais vous demander de [euh] prendre une de ces photos, de la retourner.

*Speaker 1:* Les quatre ou une seule ?

*Speaker 2:* Une seule, on va faire une par une, et de me dire spontanément ce qui vous vient à l'esprit. Admettons les trois premiers mots ou expressions qui vous viennent...

*Speaker 1:* Je commence par celle-ci.

*Speaker 2:* En tête, ouais.

*Speaker 1:* [Euh] premier mot, c'est frais.

*Speaker 1:* Ouais.

*Speaker 1:* Mm-hmm je sais pas, le deuxième, c'est icci, i deux c i, le magasin.

*Speaker 2:* Oui.

*Speaker 1:* Après. [Euh] je sais pas. Ce qui est bizarre, c'est qu'il y a des légumes d'été, des légumes d'hiver sur la même photo. Donc je sais pas si ça rentre dans votre cadre. Je vois des concombres et je vois des, des carottes. Il me semble que c'est pas en même temps. Mais je me trompe peut-être.

*Speaker 2:* Ouais. Et qu'est-ce qui vous a fait penser à frais, spontanément ?

*Speaker 1:* Ben le côté primeur, quoi. C'est un côté plus artisanal que le rayon supermarché de Leclerc, quoi.

*Speaker 2:* Ouais.

*Speaker 1:* Les prix sont pas donnés, hein [rires].

*Speaker 2:* Et du coup, vous avez pensé à ici, c'est parce que c'est un magasin que vous... ?

*Speaker 1:* Que je fréquente, oui, qui est plutôt primeur.

*Speaker 2:* Et [euh] alors du coup, est-ce que c'est un magasin dans lequel vous vous sentez [euh] bien ? Ou alors c'est un magasin dans lequel vous allez par contrainte, nécessité de faire des courses ?

*Speaker 1:* [Euh] ça, par exemple, je vais quand j'ai le temps d'y aller. Donc j'aime bien y aller. Mais par exemple, en ce moment où j'ai un planning un peu complexe, j'y vais pas beaucoup.

*Speaker 2:* D'accord.

*Speaker 1:* Donc [euh] je sais pas si ça répond à la question. J'aime bien y aller, c'est juste que ben j'ai pas toujours le temps d'y aller. Et puis en hiver, c'est peut-être, il y a, il y a peut-être une idée reçue. Il y a peut-être moins de, c'est moins intéressant que, j'achète moins de fruits et légumes. Je me suis rendu compte avec votre étude, qu'en hiver, j'achetais beaucoup moins de fruits et légumes en dehors des clémentines.

*Speaker 2:* Ouais.

*Speaker 1:* Maintenant j'arrive à trouver. Il y a de la progression. J'arrive à trouver des clémentines ou des agrumes non traités, même en supermarché. Donc il y a, je trouve qu'il y a une évolution depuis l'année dernière par rapport aux produits non traités, en, même en supermarché.

*Speaker 2:* D'accord. Vous avez remarqué, y a plus d'offres [euh] que...

*Speaker 1:* Uh-huh. Ou alors c'est peut-être parce que j'ai un peu changé mes habitudes. Je ne vais plus au même supermarché, je sais pas. En tout cas, j'arrive à trouver. C'était ma priorité, en fait, trouver des produits non traités et maintenant j'y arrive en supermarché, ce qui était pas le cas [euh] avant. Mais j'ai changé de supermarché, c'est peut-être lié.

*Speaker 2:* C'est peut-être pour ça.

*Speaker 2:* Et est-ce que vous pouvez développer [euh] l'aspect que vous aimez bien y aller mais qu'il faut le temps ? Qu'est-ce qui fait que [euh] ?

*Speaker 1:* [Euh] ben je suis une mère de famille avec trois enfants qui ont beau..., j'ai, en fait mon rythme de travail a changé. Donc pour faire mes courses, c'est vrai que tout faire au même endroit, c'est plus facile. Mais [euh] j'aime bien prendre le temps [euh] aussi de [euh] enfin j'aimerais, en ce moment, j'y arrive pas, mais j'aime bien [euh] aller [euh] dans des magasins primeurs pour [euh], pour acheter des produits dont je suis plus sûre de la qualité.

*Speaker 2:* Oui.

*Speaker 1:* Ça répond à la question ?

*Speaker 2:* Oui, tout à fait.

*Speaker 1:* D'accord [rires].

*Speaker 2:* Oui, c'est dans l'idéal, vous préférez aller dans ces magasins-là ?

*Speaker 1:* Voilà.

*Speaker 1:* Mais j'ai pas trop le temps en ce moment.

*Speaker 2:* Y a un gage de qualité. Vous pensez, plus de, plus de confiance sur la qualité des produits.

*Speaker 1:* Tout à fait.

*Speaker 2:* Mais [euh] ça demande de faire plusieurs points de [euh] d'approvisionnement, quoi.

*Speaker 1:* Exactement. C'est exactement ça, vous avez bien, vous avez formulé nettement ma pensée.

*Speaker 2:* C'est mon, c'est mon travail de reformuler après [euh]. [inaudible] compris, pas compris.

*Speaker 2:* Et [euh] donc, est-ce que y a... le fait que vous aimez bien aller dans ce magasin, c'est exclusivement sur la qualité des produits ou y a un autre aspect qui fait que c'est un magasin dans lequel vous...

*Speaker 1:* Il y a deux autres aspects. Support de, du [euh] local.

*Speaker 2:* Ouais.

*Speaker 1:* Ça, c'est hyper important pour moi. Le support du commerce local, on va dire. [Euh] aussi bien pour le magasin lui-même qui a des employés que pour les producteurs locaux.

*Speaker 1:* En fait.

*Speaker 1:* C'est ça le plus important pour moi qui fait que j'ai vraiment envie d'aller dans ce genre de magasin. Et le deuxième aspect, c'est [euh] le côté saisonnalité. Retrouver les saisons.

*Speaker 2:* Oui.

*Speaker 1:* Ça, c'est important pour moi aussi. De plus en plus. Et j'essaye de l'enseigner à me enfants.

*Speaker 2:* Oui.

*Speaker 1:* Parce que je pense que, en tant que parents, on a aussi cette responsabilité-là, de faire que, pas tout, tout de suite, tout le temps.

*Speaker 1:* Oui.

*Speaker 1:* C'est, ça fait partie de l'éducation pour moi. C'est pour ça que [euh] même si je pourrais trouver, je sais pas moi, des fraises, je crois que j'en ai vu. J'ai vu du melon la semaine dernière, je crois.

*Speaker 2:* C'est vrai ?

*Speaker 1:* Ouais. Là, en ce moment, si vous regardez dans mes placards, y a du pamplemousse [euh], des noix et [euh] quelques pommes. Et [euh] beaucoup, beaucoup de clémentines, on se fait une cure de clémentines l'hiver, parce qu'on aime tout ça. Mais du coup, c'est bien parce que la saisonnalité, justement, c'est ça que j'aime, c'est que, là, quand on va arriver en mars, on n'en pourra plus des clémentines. Il y aura de nouveaux fruits qui arriveront et on sera super content de les trouver.

*Speaker 2:* Oui.

*Speaker 1:* Et à l'automne, on sera content de retrouver les clémentines, donc c'est pour ça. Ce que j'explique aussi à mes enfants, c'est que la saisonnalité, elle a ça, c'est qu'on est toujours content. À chaque saison, on est content de retrouver un truc. Il y a les kiwis aussi.

*Speaker 2:* Oui.

*Speaker 1:* Kiwis aussi, on a.

*Speaker 2:* Oui, je comprends bien.

*Speaker 2:* Et [euh] est-ce que ça vous arrive de [euh] devoir jeter, [euh] des produits que vous aviez achetés chez le primeur ?

*Speaker 1:* Oui.

*Speaker 1:* Mais ça m'a... Surtout que je prends donc, là les clémentines non traitées. Justement, c'est le gros truc chiant, c'est qu'il y en a toujours qui ne tiennent pas plus de deux ou trois jours.

*Speaker 2:* Oui.

*Speaker 1:* Ça, ça arrive [euh], mais bon, ça fait partie. Moi, pour moi, ça fait partie [euh] du jeu, quoi.

*Speaker 2:* Oui.

*Speaker 1:* J'ai eu de ce débat mignon tout plein avec ma maman qui me disait "Ah ben oui, moi j'achète plus les clémentines feuilles parce qu'à chaque fois j'en perds". Je dis ben oui, mais moi j'achète les clémentines feuille parce que je sais qu'elles sont pas traitées.

*Speaker 2:* Oui, oui.

*Speaker 1:* Chacun ses choix. Mais bon, j'en perds pas beaucoup non plus. Et j'ai trouvé là récemment, en fait [euh], je les sors, je les achète en sachets, quoi, je, en [inaudible] et là j'ai fait un truc, c'est je les ai mis dans des petits bacs bois, comme ça y en a cinq six, donc s'il y en a une qui, voilà, ça va, ça va...

*Speaker 2:* Ça se [euh], elles sont séparées ?

*Speaker 1:* Voilà. Elles sont séparées. Enfin, elles sont séparées, elles sont par quatre, cinq, six. Mais ça va faire qu'il y aura moins de contaminations. C'est un truc nouveau mis en place, là, récemment parce que j'en avais marre [rires].

*Speaker 2:* C'est une bonne technique.

*Speaker 1:* De toute façon, je crois [euh] j'en ai discuté avec votre collègue, que [euh] nous, on perd pas grand-chose et je pense que c'est l'histoire des claies.

*Speaker 2:* Ouais.

*Speaker 1:* Je sais pas si vous vous souvenez, chez nous, y a, donc c'est un panier avec des [inaudible] en fer. Donc c'est très chiant pour vos prélèvements. Par contre, l'air circule...

*Speaker 1:* Oui.

*Speaker 1:* Et du coup, moi je trouve que je perds beaucoup moins que dans tout ce que j'ai pu avoir avant.

*Speaker 2:* Oui.

*Speaker 1:* Ce que j'expliquais à votre collègue, c'est que j'ai eu l'idée parce que mon frère, il a des pommes et qu'il les met sur des claies et que du coup, il les garde six mois, quoi.

*Speaker 2:* Ouais. Oui, c'est possible que quand on y a pas de [euh], enfin moins y a de surface, moins il peut y avoir d'accumulation de [inaudible] de bactéries ou de [euh]...

*Speaker 1:* Ça, j'en ai aucune idée, mais empiriquement, c'est ce qui fonctionne le mieux.

*Speaker 2:* Mais je crois [euh] savoir ce qu'ils nous diront [euh], le Luben, mais [euh] que y a pas mal de corbeilles de fruits qui existent comme ça. Et apparemment, c'est là ou y aurait le moins, ils ont trouvé le moins de [euh] micro-organismes, justement. Que dans des corbeilles où y a, où y a toujours un fond. Donc ouais, c'est possible que vos...

*Speaker 1:* Je pense que le fait que l'air circule ou que y ait pas de surface de contact avec un truc étranger, j'en sais rien, en tout cas je trouve que nous, ça fonctionne mieux.

*Speaker 2:* Ouais, vous avez pas... [inaudible].

*Speaker 2:* Et du coup, si vous, quand vous êtes amenée à perdre [euh] un produit, qu'est-ce que vous ressentez ? C'est quand il vient de chez le primeur ? Est-que alors, est-ce que vous vous dites, ben [euh]...

*Speaker 1:* C'est la vie.

*Speaker 2:* C'est la vie ? Ouais, c'est ça c'est pas le choix de prendre comme ça.

*Speaker 1:* Mais que soit primeurs au supermarché [euh], là ou je suis vénère, c'est les [euh] carottes. Là, en ce moment j'en achète plus parce que je ne sais pas ce qu'il se passe cette année. Elles se noircissent très très vite.

*Speaker 2:* Ouais.

*Speaker 1:* Et là, j'avais jamais connu ça avant. Donc là, les carottes, ça m'énerve parce que j'arrive pas à en avoir qui tiennent en ce moment.

*Speaker 2:* Ok.

*Speaker 1:* Mais [euh] sinon, en général, non, c'est la vie. Et puis je perds, quand je dis que je perds, je pense que je perds pas beaucoup non plus. Je sais pas si vous avez mes résultats, mais [euh] j'ai pas fait non plus tant que ça, de jet, de, de perte.

*Speaker 2:* Oui.

*Speaker 2:* C'est assez, assez anecdotique.

*Speaker 1:* Je sais plus si j'ai la poubelle, toujours à vous rendre ou pas, c'est vous qui la récupérez ou si...

*Speaker 2:* [Euh] si, ouais, normalement Damien, il a pas...

*Speaker 1:* [Inaudible] m'a dit d'arrêter de l'utiliser. Et... Je crois que je l'ai revue dans le cellier.

*Speaker 2:* Oui, c'est le moment de descendre, quand je devais faire les rendez-vous. Au final, je les ai pas faits. Je dis quand même aux personnes d'arrêter de...

*Speaker 1:* D'accord.

*Speaker 2:* D'arrêter la poubelle [rires].

*Speaker 2:* OK. Alors si on fait une autre photo.

*Speaker 1:* Maintenant on change. On change de photo. Ouais ben.

*Speaker 1:* Ça c'est l'inverse, c'est le supermarché [rires].

*Speaker 2:* Alors quel, quel mot, quels mots [euh] vous viennent à l'esprit ?

*Speaker 1:* Alors usine.

*Speaker 2:* Ouais.

*Speaker 1:* C'est très, surtout votre photo, elle [euh], elle fait vraiment usine en fait. Votre photo amplifie le phénomène.

*Speaker 2:* Oui.

*Speaker 1:* Praticité quand même.

*Speaker 2:* Ouais.

*Speaker 1:* Côté pratique. Et vous avez besoin d'un troisième mot.

*Speaker 2:* Si vous l'avez, mais sinon...

*Speaker 1:* J'ai envie de dire uniformité.

*Speaker 2:* D'accord.

*Speaker 2:* Alors est-ce que vous pouvez me dire pourquoi vous avez pensé a usine ?

*Speaker 1:* Ben parce que là, on rentre dans [euh] la grande consommation, donc, les, et la surconsommation, quand on voit ça, ça fait un peu peur parce qu'on pense à tout ce qui va être perdu à la fin de la journée.

*Speaker 2:* Ouais.

*Speaker 1:* Ceci dit, je vois qu'il y a des étals qui sont pas trop remplis. Là, les pêches, il y en a pas beaucoup.

*Speaker 2:* Ouais.

*Speaker 2:* Donc, là, pour vous, c'est un aspect plutôt péjoratif [euh]...

*Speaker 1:* Ben la photo, en tout cas, l'angle de la photo fait pas envie, hein, ça c'est clair. Je pense que c'est aussi lié à votre photo. [Euh] le côté usine parce que, ben on sait que [euh] le producteur, il gagne moins sa vie avec ça.

*Speaker 2:* Ouais.

*Speaker 1:* Après, il a, il a un volume de ventes assuré, mais [euh] du coup, il gagne moins bien sa vie à chaque unité, donc c'est un équilibre pour lui à trouver. [Euh] le côté usine parce que y a un, un, comment dire, [euh] le côté force de frappe, quoi, de la marque du supermarché. Je sais pas comment on dit c'est une marque de supermarché, mais.

*Speaker 2:* Oui.

*Speaker 1:* Force de frappe de l'achat groupé, on va dire.

*Speaker 2:* Oui.

*Speaker 2:* Vous avez dit praticité ?

*Speaker 1:* Ben oui, puisqu'on fait toutes les courses au même endroit.

*Speaker 2:* Oui, c'est ce que vous disiez tout à l'heure.

*Speaker 1:* Oui voilà, donc ça fait un gain de temps pour le consommateur.

*Speaker 2:* Mm-hmm.

*Speaker 2:* Et vous avez dit uniformité ? Qu'est-ce que, pourquoi vous avez pensé à ce mot ?

*Speaker 1:* Mm-hmm ben c'est [euh] le côté on va tous acheter la même chose au même endroit et on va tous consommer pareil, quel que soit notre profil.

*Speaker 1:* Et c'est un peu un des défauts de la vie d'actuelle, j'ai envie de dire de façon générale, parce que [euh] on a beau [euh] être officiellement libre [euh], on est dans une période où chacun cherche à rentrer [euh] le plus possible dans le rang, à pas avoir la tête qui dépasse.

*Speaker 1:* Et à, à faire comme les autres. C'est assez drôle parce que ça, ça peut faire un amalgame [euh]. J'ai eu l'uniforme à l'école.

*Speaker 2:* Oui.

*Speaker 1:* Et je suis totalement pour le port de l'uniforme à l'école. Parce que quand on est tous habillés pareil, on cherche à se démarquer différemment.

*Speaker 2:* Mm-hmm.

*Speaker 1:* Et ce qui est très intéressant, c'est on est dans une société qui est anti-uniforme, où chacun est censé faire ce qu'il veut mais où à l'arrivée quand vous regardez une sortie de l'école, en particulier collège, lycée, tous tout le monde est habillé pareil.

*Speaker 2:* Mm-hmm.

*Speaker 1:* Tout le monde coiffé pareil [euh] et tout le monde est pareil. Alors que [euh] quand j'étais au lycée, oui, enfin oui, c'était juste, au collège surtout, on avait un uniforme, eh ben, comme on pouvait se coiffer à peu près comme on voulait, du moment qu'on avait pas les cheveux devant yeux, ben c'était là-dessus qu'on jouait [euh]. On décorait nos blouses pour que ben elles soient pas comme celle de la voisine. Des choses comme ça.

*Speaker 2:* Oui.

*Speaker 1:* Donc c'est assez [euh].

*Speaker 2:* Fallait être plus ingénieux pour se démarquer [rires].

*Speaker 1:* Voilà, c'est, ben ça demandait de réfléchir pour se démarquer. Mais voilà, c'est pour ça que je suis... enfin je pense que l'uniforme, ça a un côté bien parce que ça amène une différenciation par autre chose que par l'apparence.

*Speaker 2:* Ouais, [diaphonie] c'est vrai.

*Speaker 1:* Et ramené aux fruits et légumes, c'est [euh] ben là on a une offre qui est uniforme.

*Speaker 2:* Oui.

*Speaker 1:* Et du coup, ça, on a moins de découvertes possibles. C'est-à-dire que le supermarché va vous faire des prix canons, mais il va pas vous proposer le petit produit que vous connaissez pas [euh], adéquat à la découverte, contrairement à un primeur qui lui va prendre ce risque justement pour garder ses clients et les entretenir, de proposer des choses un peu inédites.

*Speaker 2:* Oui.

*Speaker 1:* [Euh] pour [euh] ben se démarquer et puis faire plaisir à son client qui va avoir le [euh], qui va [euh] découvrir une nouvelle saveur et puis qui, si ça se trouve, va l'adopter.

*Speaker 2:* Ouais.

*Speaker 1:* C'est pour revenir [euh].

*Speaker 2:* Oui, là, dans la grande surface, il y a tous les produits [euh] un peu [euh]...

*Speaker 1:* Standard.

*Speaker 2:* Oui, voilà que tout le monde prend, mais il y a pas de [euh]...

*Speaker 1:* Mais bon, j'ai jamais vu de panais, par exemple, dans une grande surface.

*Speaker 2:* C'est possible.

*Speaker 1:* Alors que tout l'hiver, y en a ici.

*Speaker 2:* Oui. Oui, oui. Je me suis jamais trop [euh]. Pourtant c'est un légume ici qui est très, ouais, qui est très...

*Speaker 1:* Qui est assez courant.

*Speaker 2:* Ben oui, mais c'est vrai que ça se trouve on en trouve pas dans. Donc ouais.

*Speaker 2:* C'est [euh] tous les nouveaux, les, tous les... C'est des vieux légumes qui reviennent un peu mode. C'est vrai que ça se trouve, ils ne sont pas encore dans les supermarchés. Donc ouais.

*Speaker 2:* Je vois bien ce que vous voulez dire. Et du coup, dans le supermarché [euh], comment vous vous sentez ? Est-ce que y a un plaisir de faire ses courses ou, non, c'est vraiment par nécessité ?

*Speaker 1:* C'est le côté pressé, c'est le côté pratique.

*Speaker 2:* Ouais.

*Speaker 1:* Le plaisir, non.

*Speaker 1:* À part au rayon livres. Mais...

*Speaker 2:* Ouais [rires].

*Speaker 1:* C'est le côté pratique.

*Speaker 2:* Oui. Et au rayon fruits et légumes, il n'y a pas forcément de plaisir à...

*Speaker 1:* Pas de plaisir particulier, non.

*Speaker 2:* Oui.

*Speaker 1:* Non, c'est vraiment le [euh] le supermarché, c'est le côté pratique [euh], le côté bon tarif.

*Speaker 1:* Voilà.

*Speaker 1:* Mais des fois, c'est compliqué de trouver, les. Des fois, je passe au rayon fruits et légumes, j'achète rien parce qu'il n'y a rien qui me tente. C'est pas tentant en fait, par rapport aux primeurs où il y a toujours, on se dit toujours ah c'est bien. Par contre, le prix, aïe.

*Speaker 2:* Ouais. Bon, c'est l'inverse quoi ?

*Speaker 2:* Chez le primeur, y a plein de choses qui peuvent vous faire envie, mais une fois, qu'on arrive devant, on fait ah en fait, non.

*Speaker 1:* Voilà, et le supermarché, c'est l'inverse.

*Speaker 2:* Et les prix, on s'en fiche un peu. Enfin, on sait que ça va être accessible un peu et [euh]...

*Speaker 1:* Voilà, c'est exactement ça.

*Speaker 2:* Mais qu'il n'y a rien qui donne vraiment envie.

*Speaker 2:* Et [euh] pourquoi c'est pas tentant selon vous ? Est-ce que [euh]...

*Speaker 1:* Le côté usine.

*Speaker 2:* Ouais.

*Speaker 1:* Côté usine, uniforme, qui fait que [euh] on est moins dans [euh] l'achat plaisir [euh]. Et l'envie que...

*Speaker 1:* Et en plus on n'est pas là uniquement pour les fruits et légumes, on est là pour des courses générales.

*Speaker 2:* Ouais.

*Speaker 1:* Les légumes c'est en plus, pour moi.

*Speaker 2:* Ouais.

*Speaker 1:* Parce que si j'ai le temps, moi je préfère aller dans, chez le primeur.

*Speaker 2:* Oui.

*Speaker 1:* Et toutes les semaines, d'ailleurs en ce moment, je me dis toutes les semaines, allez je passerai ici après, et toutes les semaines je suis trop naze [rires].

*Speaker 2:* Et au final...

*Speaker 1:* Là j'y suis pas allée.

*Speaker 2:* Je vois bien.

*Speaker 2:* Et du coup sur les produits, est-ce qu'il y a un peu cet aspect... En fait, les produits sont [euh], comment je pourrais dire, donnent pas forcément envie dans le sens où ils sont [euh] c'est ce que vous disiez, ils sont [euh]...

*Speaker 1:* Ils sont calibrés, ils sont trop calibrés pour moi.

*Speaker 2:* Oui. C'est...

*Speaker 1:* C'est le côté trop calibré. Moi, je trouve que [euh] quand je vois un, un stock de pommes, par exemple.

*Speaker 2:* Oui.

*Speaker 1:* Où toutes les pommes ont la même taille, la même couleur, le même aspect, je me dis Et les autres, que sont-elles devenues ? [Rires]

*Speaker 1:* Voilà.

*Speaker 1:* Et donc, ça m'a, ça me dérange dans l'idée où j'ai toujours dans l'esprit, moi, le côté gâchis. C'est ce qui m'a fait a-adhérer à votre enquête, d'ailleurs. C'est [euh] pourquoi on n'a pas le droit d'avoir des pommes qui sont irrégulières ou [euh] voilà.

*Speaker 1:* Et d'ailleurs, c'est très facile de savoir si les clémentines ont été traitées ou pas. C'est que si elles sont pas traitées, elles sont pas uniformes.

*Speaker 2:* Oui.

*Speaker 1:* Même au supermarché, ils sont, elles ne sont pas uniformes en différentes tailles, différentes couleurs. Et donc [euh] après je vérifie par acquit de conscience le panneau.

*Speaker 2:* Oui.

*Speaker 1:* Mais en général à l'oeil, on voit si c'est traité ou pas pour les, les clémentines. Pour les pommes, c'est plus compliqué par contre.

*Speaker 2:* Oui.

*Speaker 2:* Et du coup [euh], finalement, c'est un peu un paradoxe. Dans le sens, quand c'est limite trop beau eh ben en fait, ça donne moins envie.

*Speaker 1:* Ça fait moins confiance, j'ai moins confiance quand c'est trop beau. Voilà, exactement, c'est exactement ça.

*Speaker 1:* Je pense qu'il y a [euh], il se passe un truc, ça s'inverse [euh] par rapport à...

*Speaker 1:* Il y a dix ou quinze ans.

*Speaker 2:* Voilà, fallait faire... On faisait du beau parce que, enfin, les gens préféraient aller vers les...

*Speaker 1:* Moi, je veux du goût maintenant.

*Speaker 2:* Oui.

*Speaker 1:* C'est aussi une évolution personnelle, hein. C'est que je vois là toute cette année, j'ai acheté les tomates [euh] dites à l'ancienne, là, celles qui sont côtelées.

*Speaker 2:* Ouais.

*Speaker 1:* Parce que je retrouvais des tomates qui avaient du goût.

*Speaker 2:* Ouais.

*Speaker 1:* Et [euh] les côtelées, pareil. Y en a de toutes les tailles, y en a, les couleurs, c'est pas toutes les mêmes, etc. Donc je pense que oui. Moi je suis vraiment, j'adhère vraiment à ce côté, à ce courant on va dire, où l'aspect ben c'est bien joli, mais [euh] l'uniformité j'en veux pas parce que [euh] je pense que le goût est plus important.

*Speaker 2:* Ouais.

*Speaker 1:* Enfin c'est pas que je n'en veux pas l'uniformité, je m'en fous de ça. C'est, ce qui m'intéresse, c'est le goût.

*Speaker 2:* Oui.

*Speaker 2:* Selon vous, quand vous voyez qu'il y a [euh] des produits uniformes, vous avez...

*Speaker 1:* Je me méfie.

*Speaker 1:* Je me méfie plus [diaphonie].

*Speaker 1:* J'ai peut-être tort, hein. Je peux avoir tort certaines fois, mais [euh].

*Speaker 2:* Ouais, c'est des choses, je pense que [euh] c'est par expérience qu'on voit que, enfin, dans les produits...

*Speaker 1:* Ben la golden qui n'a pas de goût, par exemple [euh].

*Speaker 2:* Ouais.

*Speaker 1:* Elles sont toutes pareilles quoi.

*Speaker 2:* Ouais, ouais c'est ça.

*Speaker 1:* J'ai une expérience, je crois c'était il y a deux ans, avec une Golden comme ça, magnifique, hein, d'extérieur, en extérieur et en pique-nique [euh]. Je commence à cou-, à prendre la Golden pour la couper et en fait, elle s'est dissociée en deux. La peau, les premiers deux centimètres étaient parfaits, et puis après, elle était pourrie de l'intérieur, tout l'intérieur. Ça, voilà ça met pas, ça met pas en confiance. [Diaphonie]. C'est un produit qui permet de faire ça.

*Speaker 2:* Ouais. De masquer l'intérieur. C'est juste la peau qui est, qui brille.

*Speaker 1:* Qui permet d'avoir une pomme qui reste jolie, qui est pas du tout fripée. Et l'intérieur est tout pourri. Voilà, je veux pas savoir ce que ce que j'ai bouffé dans les autres Golden du, du paquet.

*Speaker 2:* Ouais, c'est clair.

*Speaker 2:* Donc les, les produits de grande surface, finalement [euh], qu'est-ce que, si on devait résumer, qu'est-ce que vous en pensez ? Les fruits et légumes que, est-ce des fois, ça vous arrive, du coup, d'en prendre ?

*Speaker 1:* Oui.

*Speaker 2:* [Euh] qu'est-ce que vous pensez de ces produits ?

*Speaker 1:* [Euh] qu'il faut faire attention [euh] à, il faut, il faut faire attention à l'étiquetage.

*Speaker 2:* Oui.

*Speaker 1:* Et c'est ça qui domine. C'est faire attention à l'étiquetage.

*Speaker 2:* Oui.

*Speaker 2:* Alors que [euh]...

*Speaker 1:* Quand je dis l'étiquetage, en fait, on va dire sur les fruits et légumes du supermarché. Ce que je vais regarder dans l'étiquetage, c'est le traitement, la provenance.

*Speaker 2:* Ouais.

*Speaker 1:* C'est ça qui me, dont je me méfie. Alors que sur [euh] les pri-, le pri-, chez le primeur, c'est plus le tarif qui va [rires] voilà. C'est pas les mêmes choses que je regarde sur les les étiquettes.

*Speaker 2:* Ouais ouais.

*Speaker 1:* C'est ça qui est marrant, ouais. Je le réalise en vous le disant. Mais c'est réel, c'est exactement ça.

*Speaker 2:* Ouais, c'est...

*Speaker 2:* Le choix n'est plus le même du coup. C'est [inaudible].

*Speaker 2:* Donc provenance et [euh] traitement, quoi ? C'est ça ?

*Speaker 1:* Ouais, ouais c'est vraiment...

*Speaker 1:* Même traitement avant la provenance [euh] quelque part.

*Speaker 2:* Oui.

*Speaker 1:* Parce que la provenance, c'est pour des convictions, on va dire, écologiques. Et le traitement, c'est pour la santé.

*Speaker 1:* Oui.

*Speaker 1:* Du coup la santé vient avant l'écologie.

*Speaker 2:* Oui. Puis après, ça se regroupe.

*Speaker 1:* Après, les deux sont intimement liés.

*Speaker 2:* Voilà, c'est ça, mais...

*Speaker 2:* Ça marche.

*Speaker 2:* Et [euh] est-ce que ça vous arrive de perdre des fruits et légumes que [euh] qui viennent de [euh] grandes surfaces ?

*Speaker 1:* Ben pareil, hein, comme je prends des trucs non traités, oui, la même chose. Exactement pareil que pour primeurs, c'est exactement pareil.

*Speaker 1:* D'accord.

*Speaker 2:* En termes de quantité, c'est la même chose ?

*Speaker 1:* Oui, il n'y a pas de vraiment de différence.

*Speaker 2:* Et [euh] sur le ressenti ? De [euh]...

*Speaker 1:* Pareil. Non, franchement, c'est pareil.

*Speaker 1:* Là-dessus au niveau de l'usage, quand je prends, comme je fais attention à prendre aussi du non traité.

*Speaker 2:* Ouais.

*Speaker 1:* Au niveau des pertes, c'est identique, au niveau de mon ressenti, c'est identique. Et les carottes, c'est pareil.

*Speaker 2:* Oui.

*Speaker 1:* Il y a que les carottes des sables qui résistent mieux, mais ça, c'est plutôt au primeur que je les trouve.

*Speaker 2:* Oui.

*Speaker 1:* Là c'est, de toute façon, là j'en achète plus [rires], du coup j'achète, le truc horrible, les carottes [euh] déjà toutes prêtes [rires] dans les sacs, sacs plastiques. Mais au moins j'en perds pas.

*Speaker 1:* Ben ouais, c'est ça.

*Speaker 1:* Je paye quatre fois cher. Mais bon, je [rires].

*Speaker 2:* Si à chaque fois on les perd, c'est vrai que [euh].

*Speaker 2:* Ben oui. Donc c'est la même chose, OK.

*Speaker 2:* Alors si on prend une troisième photo. Quels sont les mots qui vous viennent en tête ?

*Speaker 1:* C'est le marché.

*Speaker 2:* Oui.

*Speaker 1:* [Euh] que vous dire sur le marché ? Moi, j'y vais plus. J'y vais pas. Peu.

*Speaker 2:* Ouais.

*Speaker 1:* [Euh] j'allais beaucoup enfant. Donc vous trouver trois mots, c'est, j'ai envie de dire désuet. Il y a un côté désuet.

*Speaker 2:* Ouais.

*Speaker 1:* Dans le sens où [euh] dans la société d'aujourd'hui, on va toujours très vite. [Euh] aller au marché [euh] en semaine, le matin par exemple, c'est pas envisageable.

*Speaker 2:* Ouais.

*Speaker 1:* [Euh] il y a un côté [euh], ouais, c'est ça côté désuet. Il y a un côté sympathique aussi. C'est un truc qu'on prend le temps de faire quand on a les vacances éventuellement. Mais j'avoue que moi c'est pas, j'achète pas comme ça. Je le faisais enfant.

*Speaker 2:* Ouais.

*Speaker 1:* Mais [euh] à part parfois en vacances à Saint-Malo, parce que la halle est super pratique [euh tout est au même endroit, c'est plus du tout, c'est pas du tout mon mode de consommation.

*Speaker 2:* Oui.

*Speaker 1:* Et [euh] j'irai même plus loin, pour voir ce que, ce qu'achètent mes beaux-parents qui font régulièrement le marché, je pense qu'il y a parfois, je dirai même le mot arnaque. Mon troisième mot je prendrais le mot arnaque.

*Speaker 2:* Ouais.

*Speaker 1:* Ce qui est très méchant. Mais [euh] j'ai eu la dé-désagréable impression que pour vous donner un exemple, alors c'est pas des légumes, mais le poulet qu'achète ma belle-mère à un prix pharmaceutique au marché de Saint-Malo.

*Speaker 2:* Oui.

*Speaker 1:* [Euh] elle l'achète plus cher que moi [euh] en supermarché.

*Speaker 2:* Mm-hmm.

*Speaker 1:* C'est pas un poulet fermier.

*Speaker 2:* Mm-hmm.

*Speaker 1:* Donc y a un côté [euh], j'avais été, j'avais vu aussi des œufs [euh] 3 vendus [euh] comme œufs, comme oeufs de la ferme, des oeufs marqués 3, catégorie 3.

*Speaker 2:* Oui.

*Speaker 1:* Moi, j'achète du 0 ou du 1.

*Speaker 2:* Oui.

*Speaker 1:* Y compris en supermarché.

*Speaker 2:* Oui.

*Speaker 1:* Donc y a un côté arnaque, c'est-à-dire que ça jouit d'une image artisanale qui n'est pas toujours justifiée.

*Speaker 2:* Oui.

*Speaker 1:* Bon je sais pas si j'ai dit trois mots du coup.

*Speaker 2:* Si si.

*Speaker 1:* J'ai désuet, arnaque. Et puis le troisième, je sais plus. Côté nostalgie. Enfin voilà, du coup, je [euh]...

*Speaker 1:* Je me méfie un peu des marchés. Je trouve que c'est bien sympathique, mais je suis pas tellement cliente.

*Speaker 2:* Ouais. Je vois bien.

*Speaker 1:* J'ai pas grand-chose à en dire [rires].

*Speaker 2:* Et là, quand vous allez dire vous avez dit, vous avez [euh] parlé de nostalgie.

*Speaker 1:* Ouais.

*Speaker 2:* C'est par rapport à votre enfance ?

*Speaker 1:* Oui, où j'allais au marché avec mes parents.

*Speaker 1:* Ou avec mes grands-parents.

*Speaker 1:* Mais je trouve ça bien aussi que mes enfants y aillent avec leurs grands-parents, c'est [euh], ça leur permet de voir [euh] quelque chose d'ancien [rires]. Ça un côté visite historique, mais [euh] c'est [euh]... Voilà, c'est, je pense que aujourd'hui [euh] il va falloir réinventer le marché si on veut qu'il fonctionne.

*Speaker 2:* Ouais.

*Speaker 1:* Alors il y a le principe du gros, de grands frais, mais [euh] c'est encore [euh], Grand Frais quand on voit les tarifs pratiqués, ça donne pas envie non plus, donc [euh] c'est un équilibre à trouver. Je sais pas quel, comment eux s'en sortent.

*Speaker 1:* Mais [euh] voilà.

*Speaker 1:* Après il y a un côté sympathique, hein, à faire ses courses au marché, mais [euh].

*Speaker 2:* Ouais, qu'est-ce qui peut être sympa au marché ?

*Speaker 1:* Ben c'est prendre le temps, tout simplement.

*Speaker 2:* Ouais.

*Speaker 1:* Ce qu'on ne fait pas dans notre [euh], dans notre vie actuelle, prendre le temps. [Euh] mais, voilà, moi j'ai pas mal de méfiance vis-à-vis des produits. C'est-à-dire le, le primeur du marché, [euh] je trouve que son étiquetage est pas toujours clair et que [euh] les tarifs, au vu de, par exemple on va revenir aux clémentines, hein, mais [euh] pareil. Des clémentines du marché à [euh] 4 € le kilo, ben ça peut être des clémentines traitées, quoi.

*Speaker 2:* Ouais.

*Speaker 1:* Et ça se voit. Moi je dis, c'est, c'est mon dada, hein, mais les clémentines, ça se voit immédiatement.

*Speaker 2:* Oui.

*Speaker 1:* On revient avec [euh] un deux kilos de clémentines toutes brillantes, toutes pareilles, même taille, même couleur bien orangée [euh], pas besoin de se, pas besoin de se poser longtemps la question si elles ont été traitées.

*Speaker 2:* Oui, je vois.

*Speaker 2:* Donc ça, il y a pas de, le marché, c'est sympa, mais [euh] vous pensez qu'aujourd'hui, en tout cas, [euh] c'est pas [euh] justement, y a un, y a un faux semblant dans le [euh]...

*Speaker 2:* Ouais, tout à fait.

*Speaker 2:* On sait pas sur qui [euh].

*Speaker 1:* Voilà, on ne sait pas sur qui, sur quoi.

*Speaker 2:* Et vous pensez qu'avant, il y avait moins ce truc-là ou pas ? Ou c'était aussi déjà [euh] ?

*Speaker 1:* Je pense il y a 20 ans moins.

*Speaker 2:* Ouais.

*Speaker 1:* Mais je pense que c'est pas nouveau.

*Speaker 2:* Ouais, c'est pas, c'est pas d'hier.

*Speaker 1:* Mais peut-être aussi qu'on avait moins conscience de tout ça il y a 20 ans, on faisait moins attention.

*Speaker 2:* Peut-être qu'on se posait la question, c'est vrai. Ça. Mais d'un côté, il y avait moins de moins de, y avait moins de facilités de transport, je sais pas...

*Speaker 1:* L'information circulait différemment. Il y avait plus de vrais primeurs aussi.

*Speaker 2:* Oui.

*Speaker 1:* Parce que c'était [euh], j'ai connu moi, enfant [euh], où on allait [euh] chez la maraîchère.

*Speaker 2:* Ouais.

*Speaker 1:* Donc c'était les produits [euh], c'était ses produits à elle. Et [euh], et voilà, quand elle n'en avait plus, elle fermait, des fois, la boutique elle ouvrait pas parce qu'elle avait plus rien, quoi.

*Speaker 2:* Oui, voilà.

*Speaker 1:* Moi, j'ai connu ça enfant. Aujourd'hui, c'est, aujourd'hui, donc elle, elle faisait, je crois, quelques marchés. Mais pareil, si elle avait pas de produits à vendre, elle n'allait pas au marché. Aujourd'hui, il y a quelqu'un qui fait les marchés, il sera toujours là [euh] que ce soit ses produits ou pas, il trouvera toujours quelque chose à vendre. Aussi parce que sa vie est devenue différente. Parce que pour vivre aussi, c'est plus compliqué pour lui.

*Speaker 2:* Oui.

*Speaker 1:* Même si à la base, c'est un vrai maraîcher.

*Speaker 2:* Oui.

*Speaker 1:* Il va aussi faire de l'achat revente, je pense.

*Speaker 2:* Il y en a, il y en a pas mal.

*Speaker 2:* Après, c'est, ouais, faut, les personnes qui vont régulièrement au marché, faut trier aussi, quoi et c'est, quand on a l'habitude et qu'on sait toujours [euh], les gens peuvent retrouver le bon commerçant. Ils savent que c'est le producteur direct qui [euh], mais y a plein de gens finalement qui doivent se faire un peu arnaquer en pensant...

*Speaker 2:* Ils vont au marché, ils pensent prendre local. Du coup, parce que c'est le marché, mais en fait...

*Speaker 1:* Pas du tout.

*Speaker 2:* Pas du tout.

*Speaker 1:* C'est exactement ce que je crois.

*Speaker 2:* Quand on voit un ananas à Brest, on dit Monsieur celui-là, il est pas, pas forcément [rires]...

*Speaker 1:* Je doute qu'il soit, qu'il ait poussé [euh] au Jardin botanique.

*Speaker 2:* C'est ça. Donc après, des fois, ces revendeurs-là ont un prix, ça dépend, enfin...

*Speaker 2:* Mais ouais.

*Speaker 2:* Le marché, c'est plus forcément gage de [euh]...

*Speaker 1:* De qualité.

*Speaker 2:* Ouais, de qualité, de, qualité aussi. Donc vous n'avez pas forcément l'habitude de consommer des produits qui viennent [euh]...

*Speaker 1:* Non, j'ai pas du tout l'habitude.

*Speaker 2:* Alors si on prend une, la dernière photo.

*Speaker 1:* C'est tout mignon [rires].

*Speaker 2:* C'est quoi les mois, là, qui vous viennent en tête ?

*Speaker 1:* [Euh] projet en cours [rires]. Projet en cours. J'ai recouvert un bout de jardin de bâches pour l'hiver pour que, pour essayer de faire pourrir un peu toutes les racines, pour faire un peu, refaire un potager un peu plus grand [euh] au printemps.

*Speaker 2:* Ouais.

*Speaker 1:* Mais c'est aussi le plaisir. En fait, c'est plus le plaisir. Parce que vous voyez là, vous avez un bout de mon potager. C'est un potager hors sol, là [rires].

*Speaker 2:* OK [rires].

*Speaker 2:* Donc vous faites un peu pousser [euh] les...

*Speaker 1:* Je fais pousser les, les herbes [euh], les patates parce que ça amuse beaucoup les enfants. C'est eux qui font. [Euh] les fraises.

*Speaker 2:* Oui.

*Speaker 1:* Donc c'est ça que je fais pousser surtout. Quelques... la lavande, le thym, tout, des choses comme ça. [Euh] voilà, c'est vraiment [euh] le plaisir de voir grandir. Donc il y a un côté pl- très plaisir.

*Speaker 2:* Oui.

*Speaker 1:* [Euh] voilà, je sais pas quoi vous dire d'autre. [Euh] l'ambition aussi de faire un peu plus cette année, là, en 2022 que, qu'en 2021.

*Speaker 2:* D'accord.

*Speaker 1:* Mais c'est par plaisir. Et puis aussi toujours dans l'idée de bien élever mes enfants. Leur montrer que, ben le fruit il va pousser ou le légume il pousse lentement et que [euh], et que faut prendre le temps des choses et puis que c'est du soin, du travail et puis que c'est aussi un plaisir de [euh], ben de, de...

*Speaker 1:* Comment dire, c'est-à-dire construire, c'est pas le terme, mais de [euh], de mettre ça en place.

*Speaker 2:* Oui.

*Speaker 1:* Et puis c'est aussi l'occasion de faire quelque chose en famille.

*Speaker 1:* Voilà, de [euh].

*Speaker 1:* Ah il y a les radis. J'ai oublié, dans mes cultures [rires].

*Speaker 1:* On a eu un vrai échec l'an dernier.

*Speaker 2:* Un échec ?

*Speaker 1:* Oui [rires].

*Speaker 2:* C'est pas facile, les radis, apparemment.

*Speaker 1:* Ça a été un bel échec. Je les ai éclaircis, mais ils ont jamais. Du coup, je veux réessayer, mais en pleine terre parce qu'en pot ça a pas marché.

*Speaker 2:* Ouais, ouais.

*Speaker 2:* C'est possible. Quelqu'un me disait que pareil il arrivait pas à faire des radis [rires].

*Speaker 1:* Les radis, ça a été un échec. Moi, j'ai envie d'essayer radis, carottes. Des choses simples en fait, des tubercules.

*Speaker 1:* Des choses simples.

*Speaker 1:* La menthe, ça, j'ai pas besoin d'essayer, ça fonctionne très bien.

*Speaker 2:* Ouais.

*Speaker 1:* [Euh] et puis j'en ai mis aussi en pot, en cuisine, le basilic mourait toujours. Et puis maintenant, je le mets dans la cuisine, et ça a l'air de mieux fonctionner.

*Speaker 2:* Plutôt que dehors ? [Diaphonie].

*Speaker 1:* Ben de température apparemment [rires].

*Speaker 2:* Ouais mais si la menthe elle tient ça c'est pas mal, enfin, y en a c'est très robuste, voilà.

*Speaker 1:* Ouais, la menthe, c'est facile. [Euh] dans mon ancienne maison, j'en avais sans l'avoir plantée. C'était venu avec autre chose. [Euh] ici [euh], voilà, le basilic, ça a toujours été un échec. Et là, j'ai essayé. Le thym, j'y arrive arrive toujours pas.

*Speaker 2:* Ouais.

*Speaker 1:* Le problème avec le thym, je veux l'essayer en pleine terre cette année. [Inaudible]. Voir s'il se plaît mieux. Le romarin, j'ai le même pied depuis plus de dix ans.

*Speaker 2:* Ouais, ça, pareil, c'est robuste.

*Speaker 1:* Mais le thym je ne sais pas. Ça ne fonctionne pas.

*Speaker 1:* Voilà.

*Speaker 1:* Donc voilà, c'est un côté, donc plaisir, [euh] éduquer, [euh] le côté éducatif pour mes enfants.

*Speaker 2:* Ouais.

*Speaker 1:* [Euh] même pour moi. Parce que j'apprends des trucs en faisant pousser, les, pousser les...

*Speaker 1:* Les patates. Les choses comme ça, c'est rigolo.

*Speaker 2:* Ouais.

*Speaker 1:* Et puis [euh] la jouissance [euh] pure de pouvoir manger des choses qui ont poussé comme ça dans le jardin. C'est génial.

*Speaker 2:* Ouais.

*Speaker 1:* Et il y a le côté magique par rapport aux patates. On plante une patate, on en récolte 20. Ça, c'est génial aussi [rires].

*Speaker 1:* On adore ça, nous.

*Speaker 2:* Oui.

*Speaker 1:* On fait ça dans un pot [euh] qui est de ce diamètre-là, on plante trois patates et on en récolte deux kilos quand même. Donc [euh] on se dit en pleine terre, qu'est-ce que ça va donner ? Elles sont toutes petites, hein, mais [euh] [rires]. On a fait deux récoltes de patates quand même, hein, je crois. Deux. On n'a pas, la troisième, je crois qu'on l'a laissé pourrir [rires].

*Speaker 2:* Ouais ça arrive aussi, ça.

*Speaker 1:* Voilà.

*Speaker 2:* Et [euh] donc du coup c'est, oui, c'est vraiment pas du tout pour un but d'autosuffisance, c'est vraiment pour le plaisir ?

*Speaker 1:* J'ai, j'ai 300 mètres carrés de terrain [rires].

*Speaker 2:* Donc oui, ça va être compliqué [rires].

*Speaker 1:* 300 mètres carrés de terrain [euh]...

*Speaker 1:* Trois enfants en bas âge et un métier.

*Speaker 2:* Oui.

*Speaker 1:* Donc [euh] voilà. Après, j'ai [euh] j'ai une famille [euh] pas loin d'ici qui sont des copains. Le pa- le papa [euh] le papa de la maman, le grand-père, en fait, [euh] qui est à la retraite, lui, il adore ça. Donc il passe beaucoup de temps à faire son jardin et le jardin de mes amis.

*Speaker 2:* Ouais.

*Speaker 1:* Et du coup, il récolte pas mal de légumes et il me disait que finalement, plus de la moitié de leurs légumes, c'était [euh] maison.

*Speaker 2:* Oui. Mais ça...

*Speaker 1:* Mais ça demande, mais ça demande beaucoup de temps et de soins.

*Speaker 2:* Ouais.

*Speaker 1:* Donc là, moi, j'en suis pas là. Moi, c'est plus le côté éducatif, ludique et [euh] et le plaisir.

*Speaker 2:* Ouais.

*Speaker 1:* Que vraiment faire ses conserves. J'ai pas... Ou alors il faudrait que je, que je sois dans un terrain d'un hectare, enfin, plus grand.

*Speaker 2:* Oui, [diaphonie] voilà et puis avoir le temps.

*Speaker 1:* Mais bon, on n'exclut pas, nous, de se faire une serre à un moment, ou quelque chose comme ça, mais [euh]. Mais pour l'instant, c'est plus du projet que [euh], que du concret [rires].

*Speaker 1:* Plan sur la comète [rires].

*Speaker 2:* C'est l'ambition de, c'est des ambitions.

*Speaker 1:* Voilà faut avoir des ambitions [rires] et des projets [rires].

*Speaker 2:* Et du coup, quand vous les [euh] vous les trouvez comment les produits que vous faites vous-même pousser ?

*Speaker 1:* [Euh] ben forcément, ce sont les meilleurs, hein.

*Speaker 2:* Ben oui [rires].

*Speaker 1:* Non y a un côté fierté [euh], c'est moi qui l'ai fait.

*Speaker 2:* Ouais.

*Speaker 1:* Voilà [euh].

*Speaker 1:* Il y a le côté vraiment [euh] chasse aux fraises aussi pour la période des fraises où tous les jours, il y a un enfant qui va regarder [euh], qui va regarder... Des fraises, tout ça c'est top.

*Speaker 2:* Ouais.

*Speaker 1:* Donc c'est vraiment le [euh] côté. Je me suis mis de l'eau. Ben oui, je me suis mis du café, moi. J'ai mis du café partout, moi, je vais chercher [rires]. Oui, mais c'est vraiment le côté [euh] plaisir. Encore une fois.

*Speaker 2:* Ouais.

*Speaker 1:* Résultat de, de travail, hein.

*Speaker 1:* J'en ai pas mis sur vos...

*Speaker 1:* Je sais pas comment j'ai fait.

*Speaker 1:* Non, de toute façon, elles sont plastifiées.

*Speaker 1:* Non, non, c'est même pas moi. Non non, j'ai rêvé, c'est en fait, c'est une tache de feutre [rires]. Là, il y a du feutre bleu aussi, donc il y a du marron, du bleu. Je pense qu'ils ont fait du marqueur [rires].

*Speaker 1:* Pas de café, c'est bien ça.

*Speaker 2:* Donc c'est [euh] la, le plaisir de, de manger les produits, c'est plus dans le, le, c'est plus dans le...

*Speaker 2:* Vous dites si je me trompe, mais dans le, le plaisir de l'avoir fait soi-même que dans le, le goût en soi du...

*Speaker 1:* Le goût aussi. C'est bon.

*Speaker 2:* Ils sont bons ?

*Speaker 1:* C'est bon.

*Speaker 2:* Ils sont quand même bons.

*Speaker 1:* Ils sont quand même bons. [Euh] je trouve que ça manque un peu de productivité pour les fraises. On se bat pour trois fraises, c'est compliqué.

*Speaker 2:* Le rendement...

*Speaker 1:* Le rendement n'est pas là [rires] donc, donc voilà.

*Speaker 1:* Ça manque un peu de rendement. Mais sinon, c'est, c'est vraiment le plaisir et l'amusement, on va dire. C'est le côté ludique[euh] pour la famille.

*Speaker 2:* Ouais.

*Speaker 1:* C'est aussi, je pense pas que je serais, enfin que j'aurais envie d'avoir un immense potager parce que justement, je perdrais ce côté jeu ludique plaisir.

*Speaker 2:* Ouais.

*Speaker 1:* Et je serais plus dans le côté rendement et [euh] conserves et machin.

*Speaker 1:* Voilà.

*Speaker 1:* Mais c'est parce qu'on est citadin. Peut-être que si, si on vivait à la campagne, j'aurais envie de faire comme, comme mes belles-sœurs et avoir des grands potagers, et, et me nourrir avec, hein. J'ai des, j'ai un frère et une belle-sœur qui sont quasiment en autosuffisance.

*Speaker 2:* Ah oui ? Dans le...

*Speaker 1:* Mais c'est un travail quotidien. C'est [euh] ils passent quatre, cinq heures par jour en moyenne [euh] à s'occuper de ça.

*Speaker 1:* Et ils ont un terrain d'un hectare.

*Speaker 2:* Oui.

*Speaker 1:* Là, 300 mètres carrés, ça va.

*Speaker 1:* Même en permaculture, différentes couches, tout ça, ça le fera pas.

*Speaker 2:* Oui, ça va être compliqué.

*Speaker 1:* Oui, ça se complique.

*Speaker 1:* Mais c'est plus, voilà, je dis, mon potager, ça a vraiment un côté éducatif parce que je m'y suis mis quand les enfants ont commencé un petit peu grandir, donc [euh].

*Speaker 2:* Oui, c'est [euh].

*Speaker 1:* C'est vraiment, voilà, le principal objectif de ce potager, c'est le côté éducatif et ludique.

*Speaker 2:* Oui.

*Speaker 2:* Est-ce que ça vous est arrivé de jeter un produit que vous aviez récolté chez vous ou pas ?

*Speaker 1:* [Euh] des radis tout, tout [euh].

*Speaker 2:* Oui.

*Speaker 1:* Voilà.

*Speaker 1:* Ça, oui. [Euh] sinon, non, en général, on cueille, on mange.

*Speaker 2:* Oui.

*Speaker 1:* Après. Par le passé, quand on avait fait des salades, on en avait beaucoup trop. Elles étaient montées en graine, donc là, c'était pas mangeable.

*Speaker 2:* D'accord.

*Speaker 1:* Donc je sais pas si ça compte.

*Speaker 2:* Si ouais.

*Speaker 1:* Mais ça, c'était, c'était pas l'an dernier, c'était y a plus longtemps que ça, c'était notre ancienne maison. En fait, j'avais été, j'avais planté trop de salades [rires]. Donc au début, ça paraissait bien sympa. Puis au bout d'un moment [euh], ben on n'arrivait plus à suivre [rires]. Là par contre les salades, les radis ça a pas marché. Les salades, ça avait bien marché. Il faudrait que, je vais refaire ça cette année du coup [rires].

*Speaker 2:* Ouais.

*Speaker 2:* C'est vrai que c'est pas mal, ici, ouais, ça pousse bien. Donc finalement ce que vous aviez [euh] jeté, qui était...

*Speaker 1:* On les a pas jetés, on les a compostés déjà dans le jardin. C'est pas sorti du jardin. On les a compostés direct.

*Speaker 2:* Oui.

*Speaker 2:* Donc [euh] oui, c'est des produits soit qui avaient pas, des radis qui n'ont pas fonctionné. Qui étaient pas [euh]...

*Speaker 1:* Mm-hmm. Oui, c'étaient pas des produits qui étaient un moment, enfin...

*Speaker 2:* Qui étaient bons à la récolte et que vous avez [euh]...

*Speaker 1:* C'est pas le truc qu'on a récolté, mis dans la maison et laissé passer.

*Speaker 2:* Voilà.

*Speaker 1:* Non, c'est pas ça.

*Speaker 2:* Donc, [euh], oui.

*Speaker 1:* Donc je pense qu'on peut dire que je n'ai rien jeté ?

*Speaker 2:* C'est ça.

*Speaker 1:* Et puis les fraises, de toute façon elles arrivent même pas jusqu'à la maison souvent [rires].

*Speaker 1:* [Diaphonie]

*Speaker 1:* C'est un autre débat.

*Speaker 2:* Ça se mange direct à la cueillette [rires].

*Speaker 1:* Le pire, c'est les framboises, hein, et j'en ai pas, de framboisiers, il faut que je les plante. J'en ai deux en bas, mais c'est pas encore planté.

*Speaker 2:* Les framboises, c'est bien, ça.

*Speaker 1:* Ouais, c'est bien.

*Speaker 2:* Et puis ça coûte tellement cher dans le commerce que si on peut les cueillir soi-même [rires].

*Speaker 1:* C'est ça.

*Speaker 2:* Ok alors. En autre, petit jeu.

*Speaker 1:* Je vous rends vos photos.

*Speaker 2:* Oui, merci. Est-ce que vous [euh] mangez de la banane ou pas ?

*Speaker 1:* Oui.

*Speaker 2:* Ok [inaudible].

*Speaker 1:* C'est énorme, la banane chez nous, c'est un truc [euh]...

*Speaker 2:* Ah oui ?

*Speaker 1:* C'est un long débat permanent. C'est un sujet si vous voulez chez nous. Donc ça tombe bien, que vous soyez ici.

*Speaker 2:* Vous allez me dire du coup sur quoi porte le débat. Mais là c'est dix photos de banane.

*Speaker 1:* D'accord.

*Speaker 2:* Je vais vous demander de les ranger, de celles qui vous donnent le plus envie à celles qui vous donnent le moins envie.

*Speaker 1:* La vache [rires].

*Speaker 1:* Ah y a , y a, y a moyen [rires]. [Silence]

*Speaker 1:* On va faire comme ça [rires].

*Speaker 2:* Alors, vous allez pouvoir les mettre, on va faire deux rangées comme ça je vais pouvoir prendre en photo pour après me [euh] souvenir de votre rangement.

*Speaker 1:* Je fais deux colonnes.

*Speaker 2:* Ouais, voilà.

*Speaker 1:* Voilà. J'ai toujours du café. Hop. Je pourrais les mettre les unes en dessous des autres, non ?

*Speaker 1:* Hop.

*Speaker 1:* En fait, je vais vous mettre celles que je consommerais sans problème. Après, celle-ci, elle est entre-deux.

*Speaker 2:* Ouais, à partir de celle-là, ça commence à [rires]...

*Speaker 1:* Voilà. Pas pour les mêmes raisons.

*Speaker 2:* Alors justement, expliquez-moi votre rangement. Il y a des petites formes là pour que je puisse [inaudible], savoir de quelle banane vous parlez.

*Speaker 1:* Alors [euh] alors déjà nous, les bananes, c'est un vrai sujet puisque, ah je me suis trompée, celle-ci, j'aurais pu la mettre plus haute.

*Speaker 2:* Vous pouvez la remonter.

*Speaker 1:* [Euh] les bananes, c'est un vrai sujet parce que on n'est pas tous d'accord à la maison. Et [euh] il y en a au moins deux qui, dès qu'elles commencent à vraiment, à, à être mûres, les trouvent trop mûres immédiatement. Donc c'est pour ça que c'est un vrai sujet. Donc [euh] j'ai adopté une technique simple, c'est que [euh] j'achète, déjà j'achète toujours des bananes bio parce que, pour une question de goût, j'ai, on a constaté que les bananes bio avaient beaucoup plus de goût que les autres.

*Speaker 2:* D'accord.

*Speaker 1:* Donc [euh] c'est pour ça que là aussi, c'est un vrai sujet, le goût des bananes [rires]. Et [euh] je les achète et on les consomme dans les 24 ou 48 heures max.

*Speaker 2:* Ouais.

*Speaker 1:* Parce que sinon [euh] elles, elles me resteront sur les bras.

*Speaker 2:* Ok.

*Speaker 1:* Et donc c'est bibi qui devra se les manger toutes, forme rond blanc [rires]. Voilà.

*Speaker 1:* Et donc le rond blanc, ça devient la limite.

*Speaker 2:* Même pour vous ?

*Speaker 1:* [Euh] ben si, je peux les manger. Mais elles commencent à avoir un goût un peu plus liquoreux et [euh] voilà.

*Speaker 1:* Ça c'est pour ça qu'elle est entre-deux, c'est, pour moi, celle-là je vais la consommer encore sans souci, mais quand, moi je vois la tige là qui commence déjà à pourrir, donc [euh]. Enfin, on voit que là ça commence à venir là.

*Speaker 2:* Oui.

*Speaker 1:* Au bout de la tige. Donc là ça va être [euh], je peux la consommer, mais de, à ce stade-là, il y a plus que moi.

*Speaker 2:* Ok [diaphonie] rond blanc.

*Speaker 1:* Je commence pas par le début, excusez-moi mais, mais [euh] les autres je sais qu'à la maison les autres à part un de mes fils [euh].

*Speaker 2:* Ouais.

*Speaker 1:* Pour eux, c'est déjà mort [rires].

*Speaker 1:* Voilà, c'est, c'est déjà mort. Voilà.

*Speaker 1:* [Euh] pour l'autre entre-deux puisque on va, je commence par les entre-deux du coup. Celle, donc le triangle rose.

*Speaker 2:* Ouais.

*Speaker 1:* J'ai trouvé donc, la banane totalement verte, pour moi elle n'est pas encore bonne.

*Speaker 2:* Ok.

*Speaker 1:* Probablement que je ne l'achète pas dans le stade, dans le cadre où je vous dis que [euh] je les achète pour les consommer immédiatement.

*Speaker 2:* Ouais.

*Speaker 1:* Si je me retrouve avec des bananes comme ça, je vais essayer de les laisser mûrir.

*Speaker 2:* Ok.

*Speaker 1:* Donc donc c'est pour ça que je l'ai mise plus loin. Pour moi, c'est en attente, on va dire.

*Speaker 2:* Ok.

*Speaker 1:* Voilà. Après, celles que je peux consommer, alors je les ai mis, j'ai mis celles qui étaient bien jaunes en premier, donc elle est dans un triangle bleu.

*Speaker 2:* Ouais.

*Speaker 1:* [Euh] parce qu'elle me paraît mûre à point. Ni trop ni trop peu.

*Speaker 2:* Ouais.

*Speaker 1:* Après, un peu à égalité, [euh] le triangle vert et le [euh] carré blanc.

*Speaker 2:* Ouais.

*Speaker 1:* Parce qu'elles me paraissent l'une un tout petit peu, enfin, elles me paraissent un tout petit peu plus mûres l'une comme l'autre. Je sais pas si j'ai raison, si j'ai tort. Au niveau de la couleur [euh], paraissent un petit chouille plus mûres.

*Speaker 2:* Mm-hmm.

*Speaker 1:* Donc pour moi, ce sera parfait parce que c'est comme ça que je les aime. [Euh] je sais que chez moi, il y en a qui les trouve déjà un peu trop mûres. Dès qu'il y a plus de vert dessus, c'est déjà un peu trop pour eux.

*Speaker 2:* D'accord.

*Speaker 1:* Donc, c'est vraiment [euh], voilà, en particulier mon mari qui [euh], je sais pas, il a dû, il doit avoir un traumatisme d'enfance sur les bananes. Mais lui, s'il n'y a pas de vert sur la banane, il aura beaucoup de mal à, à l'ouvrir et à la manger.

*Speaker 2:* Ok.

*Speaker 1:* Voilà. Après donc je vous ai mis le carré orange.

*Speaker 2:* Ouais.

*Speaker 1:* Le carré orange, ben on voit que elle commence à avoir quelques jours, là. À ce stade-là, je pense que, que j'arriverais à la refourguer à mes enfants si je l'ouvre moi-même et qu'eux voient pas trop le noir sur la peau. Mais pour moi, elle est encore très bonne, il y a pas de soucis [euh]. Normalement, en général, ça, ça le fait très très bien.

*Speaker 2:* Ouais.

*Speaker 1:* Voilà.

*Speaker 1:* Ensuite, on va passer à celles que je rejette. Alors déjà là carré bleu. [Euh] il se peut que j'essaie de l'ouvrir, de la couper en deux, de mettre un quelques carrés de chocolat au micro-ondes pour la manger sous forme de dessert.

*Speaker 2:* Ok.

*Speaker 1:* Par contre c'est que moi [rires].

*Speaker 2:* Ah ouais ?

*Speaker 1:* Les autres, ils voudront pas. Et [euh] les trois autres. Donc le rond orange, le triangle blanc et [rires] le rond rose. Je suis juste une bonne élève, hein ?

*Speaker 2:* Ouais ouais, ouais [rires].

*Speaker 1:* Pour moi, bon, alors je les ai mis par ordre de dégradation, mais pour moi, [euh] ça me fait penser quand mon papa faisait des bananes flambées.

*Speaker 2:* D'accord.

*Speaker 1:* [Euh] donc chez moi, c'était pas, c'était pas jeté. [Euh] j'essaye de ne jamais en arriver là.

*Speaker 2:* Ouais.

*Speaker 1:* [Euh] si j'en arrivais là, je sais pas ce que je ferais. Honnêtement, je sais pas. Est-ce que j'essaierais de les cuire sachant que je pense que chez moi, ils sont pas très [euh] volontaires pour manger de la banane cuite ?

*Speaker 2:* Ouais.

*Speaker 1:* Est-ce que je les jetterais ? Je peux pas vous, voilà. Ça dépend. Est-ce que j'en ai une ? Dans ce cas-là, je vais la jeter. Est-ce que j'en ai cinq ? Dans ce cas-là, j'essaierais peut-être de faire des bananes flambées.

*Speaker 2:* Ouais.

*Speaker 1:* Voilà.

*Speaker 2:* Mais c'est rare que ça arrive ici [euh] ?

*Speaker 1:* Il faut vraiment que je l'ai zappé, que ce soit au fond du placard et que je l'ai pas vu.

*Speaker 2:* Ouais.

*Speaker 1:* Mais j'en arrive, normalement, j'en arrive pas là. Ça, ça a dû m'arriver une fois dans les deux dernières années [euh] d'avoir une banane que, qui était restée bloquée dans le fond du sac. J'ai pas vu [rires].

*Speaker 2:* Et donc [euh], qu'est-ce qui fait que vous préférez en termes de goût celle-ci plutôt que celle-ci, par exemple ? Qu'est-ce qui fait que la banane parfaite, qu'elle est, elle a, elle est comment au niveau du...

*Speaker 1:* La banane parfaite ? Ben elle est mûre, mais pas trop mûre.

*Speaker 2:* OK.

*Speaker 1:* Donc [euh] c'est pour ça que celle qui est moins mûre et celle qui est un peu, qui est plus mûre, qui est très, qui commence à marronner beaucoup, [euh] quand elle est pas mûre, je trouve qu'elle a, elle est, elle a une texture qui me plaît pas. Et puis elle a un goût, elle a moins de goût et quand elle est trop mûre, elle commence à avoir un goût un peu liquoreux. Je sais pas si c'est le terme.

*Speaker 2:* Ouais.

*Speaker 1:* Mais [euh] un peu...

*Speaker 1:* Voilà, un peu trop, enfin, elle est lourde en bouche, j'ai envie de dire, et du coup, ça me fait moins envie, quoi.

*Speaker 2:* Oui. Donc ça peut vous arriver aussi de les cuisiner. Mais c'est assez rare, c'est [euh]...

*Speaker 1:* C'est devenu assez rare, oui. Quand j'étais célibataire, je le faisais régulièrement.

*Speaker 2:* Oui.

*Speaker 1:* Mais [euh] là, vu qu'il y a pas de [euh], j'allais dire de [rires], derrière, ils veulent pas manger, donc [euh]...

*Speaker 2:* Oui.

*Speaker 1:* Oui, ça sert à rien [rires].

*Speaker 2:* Oui.

*Speaker 1:* Mais oui, ça m'est arrivé de faire des bananes flambées ou de les faire cuire. Et même [euh] quand les enfants étaient tout petits et qu'on était encore [inaudible], ça m'arrivait de temps en temps de me prendre une banane et de me la mettre au micro-ondes au chocolat comme je vous ai dit, c'est super.

*Speaker 2:* Ben oui.

*Speaker 1:* Je sais pas si vous faites, mais.

*Speaker 2:* Si [rires].

*Speaker 2:* Moi, la banane, limite, je la mange plus en [euh] dessert qu'en [euh], cuisinées que crue [rires]. Mais je les laisse, j'attends. Là, je suis, oh ben non ben non, faut que je la cuisine [rires]. Oui.

*Speaker 2:* Est-ce que, selon vous, [euh] il y a un moment donné où la banane, elle est potentiellement dangereuse pour l'organisme ou pas selon vous ?

*Speaker 1:* Non, je pense pas.

*Speaker 2:* Donc c'est pas, pas, c'est pas...

*Speaker 1:* Tant qu'elle, en fait, là y a aucune pourriture pour moi.

*Speaker 2:* D'accord.

*Speaker 1:* Donc pour moi, y a pas de, y a pas de souci.

*Speaker 2:* Ok.

*Speaker 1:* Je me trompe peut-être, mais [euh] de toute façon [euh] des bananes comme ça j'en ai mangé aussi. J'ai pas été malade derrière donc [euh] a priori.

*Speaker 2:* Donc c'est pas [euh] parce que vous dites attention, ça peut être dangereux que vous, vous, là vous serez amenée à la jeter.

*Speaker 1:* Non, c'est juste qu'elle est dégueulasse là pour moi [rires].

*Speaker 2:* Mais c'est tout [rires].

*Speaker 1:* Oui.

*Speaker 2:* Et [euh] dans la même idée, est-ce que selon vous, il y a un moment donné où elle perd ses apports nutritifs ou est-ce qu'il y a un moment donné où elle a un summum... ?

*Speaker 1:* Probablement, mais là, je n'en sais rien.

*Speaker 2:* OK. De la même façon, c'est pas parce que là, vous vous dites Ah là, je sais qu'elle a les meilleurs apports que vous la mangez à ce moment-là ?

*Speaker 1:* Non.

*Speaker 1:* J'ai tendance à penser, ben que si on les cueille mûres à point, elles ont plus de vitamines, mais si on les cueille vertes [euh] et qu'on laisse mûrir, les vitamines [euh]...

*Speaker 2:* Oui.

*Speaker 1:* Voilà, mais après, en dehors de ça [euh].

*Speaker 2:* Ouais, c'est plutôt au moment de la, de la cueillette, c'est savoir si elle est restée sur l'arbre [euh]...

*Speaker 1:* Voilà, c'est plus l'arbre. Savoir combien de temps elle est restée sur l'arbre qui me, me permettrait de me dire oui là, il y a plus de vitamines.

*Speaker 2:* Oui.

*Speaker 1:* Mais j'en sais rien du tout. J'avoue que je, c'est pas une question que je me suis posée par rapport à la banane. [Diaphonie]. Je suis plus dans le goût, le plaisir et le [euh] fait de manger, de manger du fruit, quoi.

*Speaker 2:* Ouais.

*Speaker 2:* Et c'est justement pour ça qu'on posait la question. C'est qui, au final [euh], je crois que personne ne m'a encore dit que [euh], que la, qu'il mangeait la banane en fonction de... [rires].

*Speaker 1:* Ça peut arriver, hein.

*Speaker 2:* Ben oui [euh], mais au final les gens mangent la banane, choisissent le moment de la banane uniquement par goût, quoi, par [euh] par plaisir.

*Speaker 1:* Mm-hmm c'est un vrai sujet, la banane.

*Speaker 2:* Ouais, c'est pour ça, c'est le plus [euh], c'est très rigolo et c'est pour ça que je prends en photo, le... y a des différences.

*Speaker 1:* Il y a des dates, ou pas ?

*Speaker 2:* Non, non. Oui, enfin, normalement, enfin, je crois qu'il y avait une date, mais c'est une vidéo qui était déjà faite.

*Speaker 1:* D'accord.

*Speaker 2:* Mais c'est [euh], il y a des gens qui vont vraiment la préférer [euh] là, quoi.

*Speaker 2:* Uh-huh.

*Speaker 1:* Et [euh] y en a qui vont vraiment, oui, la préférer [euh], ou même des fois presque verte.

*Speaker 1:* Ah mon mari, ça serait plus là, je pense.

*Speaker 2:* Ouais, ouais.

*Speaker 1:* Donc il transige là.

*Speaker 1:* Et là il commence déjà à douter.

*Speaker 2:* Ouais comment, ouais c'est ça. Il y a des gens qui me disent [euh].

*Speaker 1:* Moi, c'était plus, avant d'être avec lui, c'était plus ce genre de banane, vous voyez ?

*Speaker 2:* Oui.

*Speaker 2:* Moi aussi, je pense qu'il faut qu'elle soit un peu achetée.

*Speaker 1:* Faut qu'elle commence à être achetée. Faut pas qu'elle soit trop tachetée, mais qu'elle commence à être tachetée. Donc y a, y a un vrai débat là-dessus et j'ai pas les réponses. Je sais que si je veux qu'elles se mangent bien, il faut que je les prenne là.

*Speaker 2:* Oui, c'est ça [rires], les manger en deux jours max.

*Speaker 1:* Voilà.

*Speaker 1:* Là, là, j'arriverais encore, mais [euh] ça va être [euh].

*Speaker 2:* Il y en a qui m'ont même dit que y avait, même, que, le, si ça craque pas, eh ben pour eux elle est pas bonne. Alors c'est vrai que là ça craque, ça craque, mais...

*Speaker 1:* Que ça craque moins. [Diaphonie].

*Speaker 1:* La banane, ça c'est aussi un vrai sujet. Est-ce que vous la prenez par là ou par là ? Par la tête ou la queue ?

*Speaker 1:* Ah !

*Speaker 2:* Ben moi j'ouvre là.

*Speaker 1:* Eh ben les singes les ouvrent dans l'autre sens.

*Speaker 2:* Ah oui ?

*Speaker 1:* C'est plus facile de l'ouvrir dans l'autre sens.

*Speaker 2:* Ah bon ?

*Speaker 1:* Vous essaierez, vous penserez à moi. Vous essaierez, vous allez voir. En fait, vous appuyez un petit peu le bout. Ça se casse tout de suite et vous pouvez l'ouvrir très facilement.

*Speaker 2:* Ah ! [Diaphonie].

*Speaker 1:* Ça craque pas.

*Speaker 2:* Oui.

*Speaker 1:* Ça a eu craqué et ça craque plus.

*Speaker 2:* Ça craque plus [rires]. C'est marrant, ça, faudra que je regarde. Non, c'est, ça doit être une habitude de prendre la queue.

*Speaker 1:* Ben oui, on a tous l'habitude de faire ça. Et si vous regardez un singe, il le fait pas parce que c'est con de le faire.

*Speaker 2:* Oui ? Lui, il le fait dans le bon sens.

*Speaker 1:* Voilà [rires].

*Speaker 1:* Vous essaierez, mais ça changera votre vie [rires], surtout si vous mangez beaucoup de bananes [rires].

*Speaker 2:* Vous [inaudible] sur la banane, j'essaierai.

*Speaker 1:* Vous penserez à moi, vous verrez [rires]. Vous verrez que j'ai raison.

*Speaker 2:* Je ferai des bananes [inaudible]..

*Speaker 1:* Juste vous appuyez un petit peu, vous pincez un petit peu, et puis après, ça vient tout seul.

*Speaker 1:* Mais en général, quand on pince même, ça se casse.

*Speaker 2:* C'est marrant, ça.

*Speaker 1:* Je vous les remélange ?

*Speaker 2:* Oui, on peut les, hop. Merci. Alors ok, pour la banane.

*Speaker 1:* C'est à vous que j'avais envoyé mes photos de tomates en décomposition ?

*Speaker 2:* Ouais, alors je sais pas si, est-ce qu'il y en a une [euh], faudrait regarder s'il y a les vôtres. Justement.

*Speaker 2:* Est-ce qu'il y en a ?

*Speaker 1:* Là, j'ai fait fort avec les tomates. La vache, je les ai oubliées deux fois de suite.

*Speaker 2:* C'était...

*Speaker 1:* Et j'ai fait des champignons aussi.

*Speaker 2:* Il y avait des champignons.

*Speaker 1:* De la permaculture, de la, de la polyculture de champignons.

*Speaker 2:* Les champignons étaient d-, étaient bien...

*Speaker 1:* Oui, oui.

*Speaker 1:* C'était sympathique aussi, les champignons [rires]. Pour le coup. Ça, c'est des choses qui m'arrivent pas souvent.

*Speaker 2:* Ouais, et là, il y avait aussi ces tomates-là ?

*Speaker 1:* Oui [euh].

*Speaker 2:* Et est-ce que vous vous souvenez justement de l'histoire de ces [euh] tomates ? [Diaphonie] ouais c'est ça.

*Speaker 1:* Je savais plus que j'avais des tomates [euh] dans mon [euh]. J'avais complètement zappé.

*Speaker 2:* Ouais.

*Speaker 1:* En plus, j'en ai probablement racheté entre temps que j'ai dû manger, si vous voulez, ça, voilà.

*Speaker 2:* Oui.

*Speaker 1:* Elles étaient là depuis un moment.

*Speaker 2:* [Euh] oui.

*Speaker 1:* Mais je les ai quand même mangés, les morceaux [euh], je vous avais même mis les morceaux que...

*Speaker 2:* Oui, qui...

*Speaker 1:* N'étaient pas abimés.

*Speaker 2:* Ouais.

*Speaker 2:* Vous pensez, les champignons, c'est pareil, c'est un oubli ?

*Speaker 1:* Oui, oui oui.

*Speaker 2:* Ça, ça arrive. Alors tac. Alors là, elles sont où mes plus poubelles ?

*Speaker 2:* Il y a trois [euh] types de [euh] façons de se débarrasser entre guillemets de ces fruits et légumes. Est-ce que [euh], est-ce que vous avez des poules ou des animaux qui, à qui vous allez pouvoir donner [euh] ?

*Speaker 1:* Non, j'aimerais bien, mais non.

*Speaker 2:* Oui, donc y en a pas.

*Speaker 2:* Vous compostez ?

*Speaker 1:* [Euh] la plus grande partie des légumes, sauf les agrumes.

*Speaker 2:* OK, donc on peut [inaudible].

*Speaker 1:* Et les œufs aussi.

*Speaker 2:* Ok.

*Speaker 2:* [Diaphonie]

*Speaker 2:* Et alors là, j'ai différentes [euh] préparations et je vais vous demander de [euh] garder celles qui [euh) qui vous parlent, ce que vous, vous pouvez faire des fois, et d'exclure [euh] celles qui [euh] ne vous parlent pas.

*Speaker 1:* Ça, je sais pas ce que c'est.

*Speaker 2:* C'est un gaspacho, c'est faire des soupes froides, quoi.

*Speaker 1:* Ouais, et ça, c'est quoi, c'est jus de fruits ?

*Speaker 2:* Ouais, smoothie, jus de fruits [euh].

*Speaker 1:* Soupes froides, je fais pas.

*Speaker 2:* Ouais. Et ça vous faites ?

*Speaker 1:* Le reste, oui, grosso modo, je fais, ou je fais ou je consomme. Des fois, je vous ai dit, hein, les carottes [euh] carottes râpées en ce moment, c'est le supermarché qui me les fournit.

*Speaker 2:* Ouais ouais je vois [rires].

*Speaker 1:* C'est pas bien de [inaudible] en ce moment j'en ai marre.

*Speaker 2:* Ouais, pas de jugement, hein.

*Speaker 1:* C'est anonyme [rires], ne me balancez pas !

*Speaker 2:* Alors là, je vous donne des fruits et légumes qui ont été jetés.

*Speaker 1:* D'accord.

*Speaker 2:* Par [euh] les participants.

*Speaker 2:* D'accord.

*Speaker 1:* Y en a à moi ou pas dedans ?

*Speaker 2:* Eh ben non, je crois pas, y a pas les, y a pas vos tomates, ni vos champignons. Et là, l'idée, c'est de, que pour chacune, vous, vous me disiez ce que vous voyez. Et [euh] vous me dites si vous aussi vous l'auriez jeté ou si vous auriez fait quelque chose avec, comment vous avez, comment vous percevez...

*Speaker 1:* Je commence par la, l'orange.

*Speaker 2:* Ouais.

*Speaker 1:* [Euh] j'arrive pas à voir jusqu'où elle est atteinte en fait. Donc moi, les agrumes, je vais [euh] couper la partie que j'utilise pas.

*Speaker 2:* OK.

*Speaker 1:* Et la jeter.

*Speaker 2:* D'accord.

*Speaker 1:* Puis consommer le reste.

*Speaker 2:* D'accord. Vous auriez probablement consommé [euh]...

*Speaker 1:* En gros, ben de ce que je vois, si j'en juge par la photo, j'en aurais consommé au moins la moitié, oui.

*Speaker 2:* Ouais ok.

*Speaker 1:* Après par contre je la mets, je dois vous dire ou je la mets ?

*Speaker 2:* Eh ben là ouais, enfin on peut la mettre sur une salade de fruits. Enfin, voilà.

*Speaker 1:* Vous auriez quand mangé.

*Speaker 1:* La tomate, je vois pas ce qu'elle a de, je vois pas.

*Speaker 1:* Vous avez vu mes tomates là [rires], là pour moi, c'est. Alors je l'aurais pas achetée déjà, parce que [euh] on parlait des fruits et légumes parfaits, là elle a l'air très parfaite. Je pense pas que je l'aurais achetée ou alors ç'aurait été par hasard qu'il y en aurait une belle comme ça.

*Speaker 2:* Ouais.

*Speaker 1:* Mais voilà, moi je l'aurais consommée. Oui, sans problème.

*Speaker 2:* Ok.

*Speaker 1:* Les carottes comme ça [rires].

*Speaker 1:* Ça, ça m'arrive assez régulièrement.

*Speaker 2:* Ouais.

*Speaker 1:* Quand j'en suis à ce stade, je l'épluche comme je peux.

*Speaker 2:* Ouais.

*Speaker 1:* Et [euh] j'essaye de la faire consommer ni vu ni connu avec des carottes plus fraîches [rires].

*Speaker 2:* OK, en petit mélange.

*Speaker 1:* Voilà, un petit mélange.

*Speaker 2:* Ce serait plutôt cru, cuit ?

*Speaker 1:* Cru.

*Speaker 1:* Cru parce que cuit. Personne, cuit ils les aiment pas. Donc [euh]...

*Speaker 2:* Oui.

*Speaker 1:* Ni vu ni connu, je t'embrouille. Par contre, je sais que râpé, ça marche pas, ça.

*Speaker 2:* D'accord.

*Speaker 1:* Voilà, râpé, ça marche pas parce que [inaudible] en veut pas.

*Speaker 2:* Ouais, Ok.

*Speaker 1:* Les autres, voilà. Le poivron [euh] un poivron dans cet état ? [Euh] si je suis en train de faire une ratatouille, peut-être, mais j'arrive, je laisse pas les poivrons en, en arriver là en général. Quand j'achète des poivrons, c'est ciblé parce que j'ai prévu de faire quelque chose avec.

*Speaker 2:* D'accord.

*Speaker 1:* Donc, à moins que, comme pour mes champignons [euh], pareil, j'achète quand j'ai une idée derrière [euh].

*Speaker 2:* Ouais.

*Speaker 1:* Je les ai oubliés, ça j'en arrive pas là. Donc du coup, le jeter, pas le jeter, faudrait que je le tâte.

*Speaker 2:* Ok.

*Speaker 1:* Donc là je peux pas vous dire.

*Speaker 2:* Ok.

*Speaker 1:* Sorte de ratatouille. La salade [euh] ?

*Speaker 1:* Bah je suppose que c'est parce que c'est la [inaudible] salade ? [Euh] probable que j'aurais jeté, mais peut-être pas autant de dents de salades. Après ça dépend si j'ai 500 salades [euh] peut-être que je vais jeter. Après si j'en ai pas beaucoup, je récupère le maximum.

*Speaker 2:* Ouais.

*Speaker 1:* J'ai pas de réponse. Faudrait que, pareil faudrait que je voie. [Euh] la courgette, je comprends pas trop. Elle est, elle est brunie ?

*Speaker 2:* Apparemment, oui.

*Speaker 1:* Elle est brunie. Donc là, pareil, faudrait que je voie. Peut-être que j'essaierais de la, de l'éplucher pour en savoir plus.

*Speaker 2:* Ok.

*Speaker 1:* Et récupérer les morceaux [euh] qui sont encore consommables.

*Speaker 2:* Ouais.

*Speaker 2:* Ça partirait en... ?

*Speaker 1:* Bah la carotte, même motif, même punition. Sauf que là, bon celle-là [rires], il n'y en a qu'une. [Euh] elle a l'air vraiment [euh] plus atteinte que les autres. Donc là peut-être quand même que j'irais jusqu'à la poubelle.

*Speaker 2:* Ouais.

*Speaker 1:* Puis ça dépend aussi du courage. [Euh] le bout des feuilles de poireaux. Ça aussi, pareil, y a débat toujours. [Euh] si on fait une soupe, on peut les mettre. [Euh] je pense qu'il y a toujours une petite partie, effectivement malheureusement, qui part au compost.

*Speaker 2:* Ouais, c'est [euh] c'est [euh].

*Speaker 1:* Ah j'ai fait tomber ça au passage. [Euh] la poire, ben c'est comme beaucoup de fruits, c'est, je récupère ce que je peux. Sauf que celle-là, y a pas l'air d'y avoir grand-chose à récupérer.

*Speaker 2:* Celle-là, vous essayez quand même ? Ou elle part direct au compost ?

*Speaker 1:* [Euh] bonne question. [Mm-hmm] je pense que celle-ci, c'est foutu. Je vois qu'il y a du noir même derrière les pépins. Donc là, elle était ouverte. On voit que, je pense qu'elle est trop, trop fine. Elle est trop avancée.

*Speaker 2:* Ok.

*Speaker 2:* Donc elle irait là ?

*Speaker 2:* Compost, là, oui, oui.

*Speaker 1:* On peut la mettre [euh] ?

*Speaker 1:* D'accord.

*Speaker 1:* Ah c'est ce que je jette ?

*Speaker 2:* Oui.

*Speaker 1:* D'accord.

*Speaker 1:* Alors qu'est-ce j'avais jeté, [diaphonie] c'était celle-là.

*Speaker 1:* Compost aussi. Et ça, du coup, je ne sais pas.

*Speaker 2:* Ça dépend, oui.

*Speaker 1:* Ça dépend. Le citron, c'est comme pour la, l'orange. Je, je pense que je recoupe parce qu'en plus ça, je vais le découvrir au moment où je sers le poisson [rires]. Donc je sers le poisson. Hein, on va remettre dans le contexte. Je sers le poisson, je me dis Ah mince, le citron, je sors le citron, il est vert [rires]. On me dit je coupe la partie comme ça, j'ai du citron. Ni vu ni connu, je t'embrouille.

*Speaker 2:* Ouais.

*Speaker 1:* Je pense qu'on consomme.

*Speaker 2:* Ouais.

*Speaker 1:* [Euh] le radis, je vois pas ce qui cloche. [Euh] ah c'est, à moins que ce soit du moisi là ? C'est...

*Speaker 2:* Ouais, apparemment.

*Speaker 1:* Y a du moisi au bout du radis. Ben ça dépend si c'est, déjà un radis de supermarché qui fait ça, ça m'inspire pas confiance, donc c'est compost. Si c'est mon radis, je serais un peu vénère de, de perdre mon radis, donc je couperais la partie. J'essaierai de consommer le reste et en fait, ce serait dégueu. Donc on va plutôt mettre compost.

*Speaker 2:* Compost.

*Speaker 1:* [Euh] la banane. Ben nous avons parlé du débat.

*Speaker 2:* Ouais.

*Speaker 1:* [Euh] celle-ci, je la cuisine.

*Speaker 2:* Ouais. Ouais. Ok.

*Speaker 1:* Ah, ça, c'est peut-être moi. Ça me dit quelque chose, ça. [Euh] les, non, ça doit pas être moi puisque je, ah, non, je ne vous ai pas envoyé de photo, alors j'ai les endives. On est en train de parler des endives Monsieur le micro, [euh] [rires] les endives en fait, le truc, c'est que l'endive, quand elle en arrive là.

*Speaker 2:* Ouais.

*Speaker 1:* Elle est souvent très acide.

*Speaker 2:* OK.

*Speaker 1:* Donc [euh] essayer de récupérer, oui. Mais si c'est pour [euh] la trouver dégueu [euh] et écœurer les enfants, ça vaut pas le coup. Donc on va dire compost.

*Speaker 2:* Ok.

*Speaker 1:* Compost, ça permet d'avoir bonne conscience.

*Speaker 2:* Ouais.

*Speaker 1:* [Euh] et la tomate ? [Euh] ben je crois que celle-ci par contre, donc c'est une tomate avec du moisi, contrairement à l'autre où je voyais pas vraiment de défauts d'espèce, celle-ci, je crois quand même, y a un moment où faut, faut mettre au compost.

*Speaker 2:* Faut accepter le compost.

*Speaker 1:* Voilà, faut accepter le compost.

*Speaker 2:* Et vous avez dit le compost, [euh] ça donne bonne conscience. Qu'est-ce que [euh], qu'est-ce que vous voulez dire par là ?

*Speaker 1:* [Euh] ben moi j'essaye de diminuer mes déchets de façon générale.

*Speaker 2:* Ouais.

*Speaker 1:* Et [euh] j'ai un peu moins de mal à jeter au compost que à jeter dans la poubelle elle-même. Parce que je me dis derrière ça, ça sera réutilisé, ce sera recyclé direct.

*Speaker 2:* Ouais.

*Speaker 1:* Et ça passera pas par, [euh] par l'usine [euh] de déchets.

*Speaker 2:* Ouais.

*Speaker 1:* Voilà, donc c'est vraiment l'idée, c'est de réduire max les déchets [euh], l'impact, [euh] de remplir le moins possible le camion poubelle.

*Speaker 2:* Ouais.

*Speaker 1:* C'est vraiment ça, l'objectif.

*Speaker 2:* Et donc vous, vous utilisez votre compost [euh] aussi dans vos... ?

*Speaker 1:* Alors je ne fais pas dans mon jardin. J'ai le bac à compost qui est devant la porte.

*Speaker 2:* Ouais.

*Speaker 1:* Le bac [euh] du jardin de la MPT qui est, qui est à moins de 100 mètres.

*Speaker 2:* Ok, donc c'est là-bas que vous allez.

*Speaker 1:* Donc je le vide là-bas et [euh] maintenant pour [euh], comme j'en avais marre d'avoir du jus au fond, je mets [euh], là en ce moment, il y a les feuilles de chêne qui sont restées collées. Donc c'est toujours des feuilles de chêne. Mais sinon au, à la MPT ils ont du broyat.

*Speaker 2:* OK.

*Speaker 1:* Donc je mets un peu de broyat dans le fond de mon bac, et comme ça, ça évite qu'il y ait plein de jus et que ça, ça fermente trop.

*Speaker 2:* Ok, c'est une bonne idée parce que moi aussi je composte.

*Speaker 1:* Voilà, avec le broyat, il y a moins de, déjà j'ai moins de mouches.

*Speaker 2:* Ça aussi, ouais.

*Speaker 1:* Ça, c'est bien.

*Speaker 2:* Ouais, des larves des fois.

*Speaker 1:* Et voilà. Et donc j'ai, maintenant j'ai une petite, j'ai un petit bac à compost, mais vraiment c'est genre, c'est un tupperware, quoi, qui me sert dans la cuisine, que je vais vider dans le bac dehors.

*Speaker 2:* Ouais.

*Speaker 1:* Ça évite de sortir tout le temps.

*Speaker 2:* Oui.

*Speaker 1:* J'y mets aussi le café.

*Speaker 2:* Oui.

*Speaker 1:* J'y mets aussi, ou bien là je peux mettre le sachet par exemple

*Speaker 2:* En vrac, les papiers ?

*Speaker 1:* Oui, les sachets papier, je les mets aussi parce qu'on m'a expliqué que c'était bien d'avoir un peu de, de papier ou de broyat ou de choses comme ça pour que ce soit moins acide à l'arrivée. Donc [euh] du coup, oui, j'ai moins de complexes à mettre au compost que à mettre à la poubelle elle-même.

*Speaker 2:* Oui, je comprends. Ça marche.

*Speaker 2:* Sauf les peaux des agrumes.

*Speaker 2:* Oui, il paraît qu'il faut pas les mettre. Ça tue les, ça empêche apparemment la, les vers...

*Speaker 2:* Ça rend la terre plus acide, je crois.

*Speaker 2:* Ouais, du coup, je crois il n'y a pas de vers et du coup ben ça...

*Speaker 1:* Alors attendez je remets bien les photos.

*Speaker 2:* [Inaudible]. Super, merci.

*Speaker 2:* Ouais, je crois qu'il y a le lombric, non ? Je sais plus comment il s'appelle, le, enfin y a un ver, quoi, qui [euh].

*Speaker 1:* J'en sais rien [rires].

*Speaker 2:* Et apparemment les [diaphonie].

*Speaker 1:* Même là, j'ai été vider mercredi, enfin hier, puisqu'on est jeudi. [Euh] j'ai pas vu les moucherons en fait. Je me suis dit, tiens, c'est marrant, il y a plus de moucherons. Et en fait, quand j'ai rabattu le bac, ils étaient tous sous le couvercle, en fait [rires].

*Speaker 1:* Il y en avait peut-être un millier, quoi. Donc [euh] les légumes d'un côté.

*Speaker 2:* Oui.

*Speaker 1:* Et les scènes de l'autre. C'est ça ?

*Speaker 2:* Mais c'est, ouais, les larves, ça me, des fois ça me...

*Speaker 1:* Mais je le mets plus, je le mets dans la maison. Au début, il y a un moment où c'était dans la maison et c'était moisi.

*Speaker 2:* C'est ça, oui, je suis en appartement.

*Speaker 1:* Vous avez peut-être un balcon ?

*Speaker 2:* Eh bien maintenant, là, j'ai déménagé. Maintenant, j'ai un, je suis au rez-de-chaussée, et j'ai pas loin pour y aller, y a une cour extérieure, donc là, ça va. Mais avant, quand j'étais au quatrième étage [euh], j'avais pas de balcon et là c'était l'enfer.

*Speaker 2:* Et donc, des fois, j'avoue, je craquais. Je, je remettais tout dans la poubelle. Enfin, ça, du coup, ça n'avait plus aucun sens [rires]. Composter en intérieur, c'est ouais, c'est pas le même [euh].

*Speaker 1:* Y a des bacs à compost avec des vers ou je sais pas quoi, ça l'air intéressant comme idée, mais...

*Speaker 2:* C'est, oui, c'est carrément un lombricomposteur d'intérieur, quoi.

*Speaker 1:* Moi, ça m'intéresserait d'essayer. Mais j'ai un mari qui s'y, qui s'y oppose fermement [rires].

*Speaker 2:* Pas très vert [euh] ?

*Speaker 1:* Non, non, on essaye déjà la banane quand elle est pas verte. Pour lui, c'est déjà trop. Donc vous imaginez s'il avait des lombrics dans la cuisine [rires].

*Speaker 1:* Il aime pas trop tout ce qui commence à fermenter. Oui, non. [Euh] alors juste pour finir sur la, la poubelle, comment ça s'est passé ?

*Speaker 1:* Ben bien.

*Speaker 2:* Oui, pas de... ?

*Speaker 1:* Bien [euh]. Non, y a pas. RAS j'ai envie de dire.

*Speaker 2:* Ouais.

*Speaker 1:* Ça a bien fonctionné. Cette fois-ci, les piles l'ont tenu jusqu'au bout.

*Speaker 2:* OK.

*Speaker 1:* Contrairement à la dernière fois, où elle s'était mise en vrac.

*Speaker 2:* Ouais.

*Speaker 1:* J'ai un peu plus changé le sac aussi. Parce que voilà, c'est, je pense, la dernière fois avec la fermentation, ça a dû changer de poids dedans et ça a dû la, ça a dû, voilà.

*Speaker 2:* Ouais

*Speaker 1:* Mais non.

*Speaker 1:* Non, de toute façon j'utilise déjà ce genre de poubelle régulièrement, donc ça n'a pas changé grand-chose à ma vie.

*Speaker 1:* Ça oui. Les contraintes, ça a été. Et vous pensez avoir du coup moins jeté que... ?

*Speaker 1:* Non, j'essaie de pas changer mon comportement pour que votre étude soit la plus...

*Speaker 2:* La plus... ok.

*Speaker 1:* La plus propre possible, la plus proche de la réalité possible.

*Speaker 2:* D'accord. Vous avez pas eu de changement d'habitudes, non ?

*Speaker 1:* Non. J'ai l'impression d'ailleurs, je, je pensais que je jetais moins en automne. [Euh] et en fait, j'ai jeté à peu près, grosso modo, en fait, il y a eu plus d'oublis là que pendant l'été.

*Speaker 2:* Ouais.

*Speaker 1:* J'ai eu plus d'oublis. Ce que j'ai jeté, c'était vraiment des oublis alors que pour la première période, c'était des [euh] deux, trois fruits qui restaient, etc., alors que là c'était vraiment de l'oubli. Et voilà les champignons typiquement.

*Speaker 2:* Oui [diaphonie].

*Speaker 1:* Je les ai achetés pour faire un truc, ils sont restés dans le sachet. J'ai complètement zappé. Je les ai retrouvés quinze jours après. Et là, c'était trop tard, quoi.

*Speaker 2:* Oui, ça c'est pas assez, puisque souvent l'été [euh], on perd plus dans le sens où c'est des, des fruits et légumes qui sont beaucoup plus fragiles eh ben du coup à la fin de la semaine, il y en a quelques-uns qui n'ont pas supporté [euh].

*Speaker 1:* Donc à l'arrivée, je dois avoir grosso modo la même proportion de déchets, contrairement à ce que je pensais. Là c'est ma découverte à moi. Mais c'est lié, là, c'est lié à des oublis.

*Speaker 2:* Ouais, c'est pas les mêmes raisons, quoi.

*Speaker 1:* C'est pas les mêmes raisons. C'est ça qui est intéressant par contre.

*Speaker 2:* Oui.

*Speaker 1:* Pour mon côté de l'étude, c'est ça que j'ai trouvé ça, que j'ai trouvé intéressant.

*Speaker 1:* Oui. Ça marche.

*Speaker 2:* Bon ben merci beaucoup.

*Speaker 1:* De rien.

*Speaker 1:* Je vais vous rendre votre petite poubelle.

*Speaker 1:* Oui [rires], je fais le tour du campement.

**Household F09, Interview 1**

**C'est parti. Alors, dans un premier temps vous propose un petit jeu comme une devinette. Je vais vous demander de penser à un fruit ou un légume que vous aimez particulièrement. Vous allez pensez à ce fruit ou à ce légume sans jamais me dire son identité. L’idée est que je le devine uniquement grâce à votre description.**

OK.

**Est-ce que vous avez un fruit un légume en tête ?**

Oui mais si je le décris, ça va être facile.

***(rires)* Dites-moi tout ce que vous avez en tête sur ce fruit ou sur ce légume.**

C’est un fruit qui vient des pays chauds. Il a une peau assez particulière… et des feuilles assez volumineuses on va dire. Donc il faut bien découper toute la peau pour trouver le fruit à l'intérieur, qui est de couleur jaune. Il a un goût très sucré, un peu acide, parfois. C’est facile non ?

**Pas tant que ça ! Et qu'est ce que vous aimez particulièrement ?**

Dans ce fruit ? Le côté sucré et rafraîchissant parce qu'il est gorgé d'eau. Et que même la peau, je l'utilise pour faire… Quand je la boue avec de l'eau et ça me fait un peu de l'eau... comment on appelle ça… qui draine un peu quoi. Détox ! Voilà, c’est ce que je cherchais !

**D'accord, en général, quand vous le mangez, vous le mangez quand ? C'est plutôt le matin, plutôt le midi… N’importe quand ?**

Plutôt au dessert, midi et soir, peu importe.

**Donc, vous avez parlé de feuilles volumineuses, d’une peau épaisse ? Du coup voilà, est-ce que vous pouvez me raconter, voilà, vous avez le fruit devant vous… tout le procédé jusqu’à ce qu'il arrive dans votre bouche. Imaginez, vous l'avez là, qu’est-ce que vous faites ?**

Déjà j’enlève toute ce qui est feuilles. Et ensuite, je coupe les extrémités et ensuite j'enlève toute la peau sur toute la longueur.

**OK**.

Voilà…

**En général, vous l'achetez où ?**

En grande surface.

**En grande surface ?**

Ouais je l’achète en grande surface, par deux ou trois d’ailleurs.

**D'accord…**

On consomme vraiment ça à la maison.

**Et quand vous mangez, qu'est ce que vous ressentez ? Au moment où vous le mangez ?**

Ca me met… j’ai l’impression de boire un verre de jus en fait, il tellement gorgé d'eau que ça me rafraîchit et ça me… Pour un dessert, ça passe très bien, surtout quand il est bien frais, ça me… J’aime vraiment ce fruit.

**OK… Et est-ce que vous avez le sentiment qu’il vous apporte quelque chose en plus de côté rafraichissant…**

Peut-être l'apport de vitamines. Je pense que c'est un fruit qui en est plein et du coup, ça booste un peu, je pense.

**Et est-ce que vous lui trouvez des inconvénients ?**

C’est juste la préparation qui est un peu casse pieds : enlever toutes les peaux… découper en rondelles… C'est un peu long. C'est pas comme une pomme où on la lave…

**Je vois *(rires)* Alors, là, si je devais deviner… Je dirai l’ananas.**

Oui, bravo ! *(rires).*

**Yes ! Chouette. Alors maintenant, j'aimerais bien qu'on discute de vos pratiques d'achat.**

Oui.

**Et de votre organisation générale. Du coup, au sein de votre foyer, c'est vous qui êtes amené à faire des courses ?**

Oui.

**Du coup, comment vous vous organisez ?**

Je fais des drives. Les quatre-vingt pourcents du temps, c’est en drive. Vraiment, si j’ai des choses qui manquent, je vais en magasin mais sinon c’est en drive. En général je fais un drive tous les… pff.. dix jours à peu près, on va dire.

**D’accord.**

C’est-à-dire, si j’en fais un le lundi, je peux en faire un le mercredi de la semaine d'après… Voilà en général comment je m’organise. Après ça dépend… En général c’est comme ça et je commande tous mes fruits et légumes en drive.

**D’accord. Les fruits et légumes aussi.**

Oui.

**Du coup quand vous faites le drive, vous savez ce que vous souhaitez acheter… ?**

Oui.

**Comment vous…**

C’est les habitudes.

**C’est par habitude ?**

Ouais c’est par habitude, j'achète plus ou moins toujours la même chose. Que ce soit, les pommes ou les oranges… Euh pff… Au drive comme on est bien servi, je me casse pas la tête. Donc ouais…

**OK.**

Les légumes, c'est pareil : la salade, les carottes, les pommes de terre… C’est toujours plus ou moins la même chose qu’on commande.

**Ouais ? OK. Ca marche. Donc vous avez su ce lieu là, de drive. Est-ce que vous en avez d'autres ou c'est exclusivement cette surface là pour le drive.**

Alors, à l'occasion je vais au marché, mais vraiment à l'occasion. Ça peut être une fois tous les trois, quatre mois. Et de temps en temps en hiver, on va chez un maraîcher acheter des pommes, voilà. Ca nous arrive d’acheter quatre, cinq, six kilos de pommes, pour la compote, quand c’est la période des pommes en hiver.

**OK. d'accord. Donc marché très occasionnel, votre approvisionnement régulier ça va être le drive.**

Oui.

**OK. Alors, est-ce que vous pouvez dresser une liste des différents fruits et légumes que vous avez l’habitude de vous procurez, ceux vous consommez très régulièrement. Vous avez commencé là à citer, selon vos habitudes. Par exemple, si on commence par les fruits, est-ce que vous avez…**

Oui oui, bien-sûr. On consomme la banane… la pomme, l'orange, l’ananas… Après ça dépend des périodes. Ça peut être melon, pastèque, quand c’est l’été, en ce moment. En automne, c'est plutôt les raisins. On consomme beaucoup de raisins, que j’achète aussi au drive. Après des fois à l'occasion aussi des mangues, fruits de la passion aussi. Kiwi aussi, mais vraiment plus rarement. Oui, je crois que j'ai fait le tour.

**Et à l’inverse, ce que vous consommez moins ? Par exemple, le kiwi vous dites que c’est plus rarement… Est-ce qu’il y en a d’autres comme ça que vous…**

Euh… Les poires, c’est vrai que j’achète pas beaucoup de poires, je ne sais pas pourquoi…

**Pas de raison…**

Non… Parce que si, souvent quand je les achats au drive, elles sont hyper dures. Elles sont pas mûres, elles sont pas bonnes donc forcément au bout d’un moment, j’ai laissé tomber. Sinon, au niveau des fruits qu'on consomme pas trop... C'est plus ces fruits exotiques, après… qu’on consomme plus rarement… Les fruits de dragon et autres, c’est des choses qu’on consomme pas du tout… Papaye, tout ça, noix de coco, non. D’ailleurs, pêche, abricot, on en consomme.

**OK. Et sur les légumes du coup, ceux que vous achetez très régulièrement…**

Les légumes il y en a un peu moins… Ca va être plus… pommes de terre, carottes, poivrons courgettes, aubergines, tomates, tomates bien-sûr, on consomme beaucoup de tomates, tomates cerises ou tomates… normales. De la salade, poireaux. Ouais ça va être plus ça… Je vois pas d’autres trucs… Le chou, si le chou. La patate douce qu’on consomme aussi beaucoup. Après il y a beaucoup de légumes qu’on consomme pas comme les radis, céleri… euh.. ouais si champignon ça peut arriver… c’est plus rare. *(La personne s’adresse à son fils)* Quel légume on consomme pas *(nom enfant),* t’as une idée toi ? Navet, on consomme pas. Par exemple, les brocolis ou les chou-fleurs, on consomme mais surgelés quoi. Mais j’achète très rarement frais parce que c’est trop gros et que… enfin j’ai pas envie d’en manger pendant 3 jours *(rires)* donc j’en consomme pas beaucoup. Après je sais qu’il y a plein de légumes qu’on consomme pas… Tous les légumes anciens, on consomme pas trop.

**Du coup, qu’est-ce qui fait que vous… par exemple là vous m’avez dit brocoli, chou-fleur en surgelé.**

Ouais en surgelé, c’est plus facile ouais.

**Et par exemple le radis, le cèleri... pourquoi vous.. (10’50)**

C’est les goûts.

**C’est les goûts ?**

Ouais, le goût acide des fruits, des légumes on aime pas trop ça. Après il y a certain peut-être parce que je sais pas trop comment les cuisiner. Le panais, c’est pareil… Des choses comme ça. C’est pas des choses qui sont trop dans leurs habitudes. Il faut avoir le temps de cuisiner aussi. Epinards, c’est pareil… Epinards on consomme pas, ou surgelés mais rarement.

**OK. Alors, si on s'intéresse vos lieux de stockage des fruits et légumes, quand vous revenez de vos courses… Qu'est ce que vous faites de vos fruits ?**

Mes fruits je les mets tous dans la panière à fruits. Certains au frigo. Et exceptionnellement, genre la pastèque quand je veux consommer frais, le melon aussi, mais sinon très peu de fruits au frigo quand même, ça va être plus dans la panière à légume dans la cuisine ou le panier à fruit. Et les légumes, c’est plus dans ma logia, où c’est un peu plus frais.

**D’accord.**

Voilà, à part la salade que je mets au frigo et après les légumes que je consomme… enfin que je découpe mais que je ne consomme pas tout de suite, je les mets au frigo aussi.

**OK.**

Mais sinon ils sont tous dans la logia.

**D’accord. Est-ce que selon vous, ce sont des lieux de stockage idéaux…**

Oui, oui, je pense. Après, j'ai déjà essayé de mettre les carottes au frigo, j’ai vu que ça prenait vite l'eau. Je me suis adaptée… Je pense que c'est la meilleure façon que j’ai trouvé pour les stocker. Après il y en a peut-être d’autres, mais…

**Vous avez le sentiment que là, comme c’est, ça se conserve bien ?**

Ouais.

**OK. Ca marche. Et au niveau du nettoyage de ces lieux, surfaces, est-ce que vous les nettoyez ? Est-ce que ça vous arrive ou pas ?**

Pas à chaque fois, quand j’achète des fruits parfois j’enlève les fruits qui sont en dessous, je les remets… les nouveaux fruits en dessous et les anciens au-dessus. Mais… je nettoie pas forcément, après c’est pas toujours sale, à part s’il y a un fruit qui a pourri, là je vais nettoyer mais… c’est pas systématique.

**Alors, si maintenant on s'intéresse à votre façon de consommer les fruits et légumes frais. Si on prend un fruit que vous consommez régulièrement, vous avez dit par exemple, prenons la banane ?**

Oui.

**Sous quelle forme vous la consommez ?**

Alors, la banane on la mange souvent cru, ou nature quoi. Ca peut arriver parfois qu’on la met dans un gâteau, mais… c’est pas systématique. C’est souvent comme ça, cru au dessert ou même en en-cas, quand on a faim. Par exemple au bureau j’en ai amené une, cet après-midi j’ai mangé ça. Ca peut m’arriver.

**Donc ça arrive en gâteau mais c’est plus rare ?**

Très rare pour la banane. La pomme, ça peut arriver. Parfois, c'est pareil. Souvent on la mange cru la pomme, et de temps en temps en gâteau. Mais souvent les fruits je les cuisine pas plus que ça. Vraiment rarement. Ouais, c’est vraiment très rare.

**D’accord. Vous avez parlé de faire des compotes, de temps en temps ?**

Ouais voilà de temps en temps mais ouais c’est pareil.

**Et du coup, si on prend un légume.**

Par contre les légumes… ben’ c’est toujours cuit de toute façon.

**Ouais, il n’y a pas de légumes qui…**

Non. A part les tomates qu’on peut manger comme ça, le concombre aussi. On consomme aussi le concombre, oignons échalote, tout ça. J’ai pas dit tout ça mais on consomme aussi beaucoup. Ouais, certains légumes comme les carottes, on peut faire en carottes râpées. **Sinon, les trois quarts du temps, même quasi systématiquement c'est cuit, dans les quiches, dans les ratatouille, dans les gratins… En sauce…** On en mange…

**Donc les légumes sont souvent préparés.**

Oui, oui.

**Et est ce que vous avez en tête des légumes ou des fruits que vous allez manger comme ça sans rien éplucher ? Tout à l’heure, on disait que l'ananas demande une sacrée préparation avant de consommez, est-ce que vous avez en tête comme ça, un légume où vous pouvez, sans rien toucher, le manger….**

Ben la tomate et la pomme. La pêche aussi, la nectarine, qu’on va manger là ces jours-ci. La fraise aussi qu’on a mangé là, en ce moment. En légumes, c’est vraiment la tomate qu’on mange tout le temps. Des tomates cerises on en mange toute la journée, presque. C'est tout. Et la salade quoi. La salade faut juste la découper.

**Oui par exemple, est-ce que, quand vous préparez une salade, est-ce que vous avez de l’épluchures ou pas ?**

Ça m'arrive ouais.

**Ouais, c’est-à-dire, c’est..**

Les premières feuilles quoi que j’enlève ouais.

**Vous enlevez les premières feuilles ?**

Ouais, ouais.

**Vous diriez que c'est plutôt systématique ou non, ça dépend du…**

Ca dépend de la qualité de la salade, ouais.

**Alors, j’aimerai là qu’on échange sur le moment où vous jetez un légume ou une partie seulement quand cela arrive, pourquoi vous décidez de le jeter, plutôt que de le consommer ?**

Parce qu'il est pourri.

**Parce qu'il est pourri ?**

Ouais. Euh… C’est vraiment qu’il est pourri. Ouais.

**Et comment vous déterminez qu’un fruit et un légume est pourri ?**

A l'odeur ou au touché.

**A l'odeur ou au touché ?**

Mmh. C’est souvent si je touche le légume et que je vois qu’il est un peu flétri…

**Ouais.**

Ouais. L’autre jour on a voulu faire une patate douce, on a vu qu’elle sentait mauvais, on l’a jeté. On avait pas vu celle la, qu’elle était pourrie. (18’52). Mais c’est un peu trompeur parce que, on dirait pas comme ça qu'elle est pourrie, mais… elle est pourrie *(rires)*

**Et qu'est ce que vous ressentez quand vous touchez quelque chose de flétri, pourri ?**

Alors déjà, je suis un peu dégoûtée d'avoir laissé le légume pourrir mais… je… Alors certains légumes où je coupe ce qui est pourri quand même pour garder le reste. Si je vois que sur un concombre ou autre, il y a un bout qui a un peu pourri, je vais pas tout jeter. Mais en général si le légume est vraiment vraiment pourri, je le jette. Et j’aime pas gaspiller.

**En général oui, on aime pas gaspiller… OK et est ce que vous avez l'impression au sein de votre foyer, qu’il y a des différences entre vous, dans les préférences, par exemple dans la maturité du fruit ou du légume, que certains vont préférer très mûr, d’autres pas du tout mûr…**

Ouais, surtout la banane, les enfants, dès que c’est un peu noir, ils veulent pas manger. Moi, ça me dérange pas plus que ça.

**D'accord**

Sinon, non il y a pas trop de différence. C’est vraiment la banane. Je sais pas pourquoi… je pense parce que c'est noir : c'est noir, c'est pourri euh… *(rires)* Donc s’il y a une banane avec des petits points noirs, ils vont presque préférer prendre celle qui est presque verte, qui est plus belle que… Alors qu’elle sera moins bonne. Après voilà, c’est les enfants.

**Je vois !**

Sinon non, ils font pas trop la différence sur le reste.

**Ouais ? OK. Alors là, vous avez parlé justement du gaspillage. Si je vous dis gaspillage alimentaire, quels sont les mots qui vous viennent spontanément à l'esprit ?**

Gaspillage alimentaire… Ben’ pour moi c'est du gâchis… c'est de l'argent qu'on jette quoi… C’est… Moi franchement, ça me fait un peu mal au coeur de jeter parce que je me dis que j'aurais pu cuisiner avec, ou faire quelque chose... enfin Je me dis que j’ai pas géré quoi ! *(rires)* Ca me fait un peu mal au cœur de jeter moi, clairement. Avec les enfants, on a voyagé, en Afrique. Et quand on voit à quel point les gens font attention à ce qu'ils mangent, ne serait-ce que le moindre grain de riz, et nous… il suffit que ce ne soit pas la bonne couleur ou la bonne forme, les gens jettent facilement. Ça fait un peu mal au cœur. Donc je fais attention à ce qu’on ne fasse pas ça *(rires).*

**Ouais, donc ça vous fait mal au cœur pour.. et d’un point de vu financier, de perdre de l’argent et aussi d’un point de vu… euh**

Responsable quoi, enfin la responsabilité… Ouais.

**Ok, ça marche. Alors, ça pourrait être intéressant de donner une définition du gaspillage alimentaire, avec vos propres mots. Si vous deviez écrire une définition dans Le Petit Robert, qu'est ce qu'il y aurait après « gaspillage alimentaire ».**

Pour moi gaspillage alimentaire, c’est quand on jette tout ce qui pourrait être mangé. Parce qu'on va laisser pourrir... Parce que… on n'en a plus envie ... Parce que… Parce que oui, pour moi c’est ça. On jette la nourriture qu’on pourrait manger.

**Ok. Ca marche. Alors, si on parle de la poubelle connectée, que vous avez depuis quelques jours, racontez-moi comment ça se passe ? Du coup, son utilisation… est-ce que vous avez l'impression de l’utiliser fréquemment ou au contraire que très peu ?**

J’ai l’impression de l’utiliser très peu moi. Je pense que ça me responsabilise aussi. Du coup, je fais attention à ce que j’ai pour pas jeter des choses dedans *(rires).* Mais ouais, je l’utilise très peu après, je pense que je fais plus attention.

**Ouais du coup vu qu’elle est là…**

On a moins envie de jeter des choses dedans *(rires)*

***(rires)* Ok. Ca marche. Et*, es*t-ce que vous vous souvenez de ce que vous avez pu y jeter ?**

Oui, oui, ben la patate douce, entre autre !

**Oui, la patate douce.**

L’autre jour j’ai jeté un morceau de banane aussi un peu pourri. Euh… des tomates qui avait bien pourri aussi. Elles avaient pris un bon coup de chaud. Quelques pommes de terre qui avait germé… On a jeté un melon aussi qui avait un peu pourri. Je l’avais laissé au frigo et.. il avait un peu moisi donc on l’avait jeté. *(s’adressant à son enfant)* Tu te rappelles ? Du melon qu’était pas très bon ? *(rires)* Il se rappelle pas ! De toute façon il mange tout avec moi lui *(rires).*

***(rires)* Donc toutes ces choses, vous les avez jeté parce que c’était euh.. vraiment pourri ?**

Ouais vraiment pourri.

**Ok. Est ce que ça vous est arrivé de vous poser des questions avant ou après avoir jeté l'aliment : est ce que ça, je dois le mettre dedans ou pas… ?**

Non, pas spécialement. Je savais ce qu’il y avait à mettre dedans donc… euh..

**Ok. Chouette. Et est-ce que ça vous est arrivé de l’oublier ? De se dire voilà à un moment donné, j'ai jeté quelque chose et..**

Oui ouais, ça m’est arrivée. C’est des petites choses mais… un petit légume pourri que j'ai coupé en même temps que mes carottes et qui est parti avec les épluchures… Mais ce n'était pas grand-chose mais oui un petit peu.

**Oui, quand vous préparez des fois…, ça part avec les épluchures, le petit bout…**

Ouais voilà, c’est ça. Mais pas des choses significatives.

**Ouais, OK, super. Et est ce que vous avez changé de sac poubelle depuis…**

Oui, ouais, j’ai changé. Une fois.

**OK, ben’ super merci, j’ai posé toutes mes questions… Est-ce qu’il y a des choses sur lesquelles vous voulez revenir, des choses que vous pensez que je n'ai pas abordés ou non…**

Non, non je pense qu’on a fait le tour.

**Et ben’ super, merci beaucoup !**

**Household F09, Interview 2**

*Mme. 1:* Le concept qui- qui moi m'intéresse.

*Mme. 2:* D'accord.

*Mme. 1:* Hop. Alors, toc. Mon petit matériel.

*Mme. 2:* Oui, [rire] Ici. Ici, j'ai quatre illustrations.

*Mme. 1:* D'accord.

*Mme. 1:* Je vais vous demander d'en choisir une, de la re- de la retourner et de me dire [euh] spontanément ce qui vous vient [euh] à l'esprit. Au moins les trois premiers mots qui vous viennent.

*Mme. 2:* Les trois premiers mots.

*Mme. 1:* Oui.

*Mme. 2:* Alors, donc c'est un jardin.

*Mme. 1:* Ouais.

*Mme. 1:* [Euh] Voilà. Premièrement, c'est vert.

*Mme. 2:* Ouais.

*Mme. 2:* Parce que tout est vert. [Euh] Nature.

*Mme. 1:* Ok.

*Mme. 2:* Et [euh] sain.

*Mme. 1:* Oui.

*Mme. 2:* Et [euh] voilà.

*Mme. 1:* Ok. [Euh] Vous avez dit jardin. Qu'est-ce qui vous avez fait penser à -

*Mme. 2:* [Ben] Surtout les jardinières. C'est des jardinières de légumes. Donc [euh] avec différentes plantes. Je dirais je sais pas trop ce qu'il y a. Je suis pas très- j'ai pas trop la main verte. [Rire] Mais je sais pas ce que c'est. Je pense qu'il y a des poireaux.

*Mme. 1:* Oui. [Rire] C'est le- c'est le signe que j'ai bien eu.

*Mme. 2:* Ouais. Et voilà. Ouais.

*Mme. 1:* Vous avez dit vert.

*Mme. 2:* Céleri. Ouais, c'est ça. Vert. Oui.

*Mme. 1:* Et nature, pourquoi ça vous a fait penser à la nature ?

*Mme. 2:* [Ben] C'est [euh] c'est l'idée du vert de la terre. [Euh] Puis [euh] [bah] c'est vraiment les légumes qui sortent du sol. Enfin, c'est vraiment la- la nature quoi.

*Mme. 1:* Oui.

*Mme. 2:* C'est l'image. [Rire]

*Mme. 1:* Et [euh] vous avez dit sain. Qu'est-ce qui-

*Mme. 2:* Oui.

*Mme. 1:* Vous fait penser à ça ?

*Mme. 1:* [Ben] Comme c'est produit en petit.

*Mme. 2:* Oui.

*Mme. 1:* Voilà, ça- je pense pas qu'il y a d'engrais, tout ça. Donc pour moi, c'est- c'est bio, c'est- c'est sain quoi.

*Mme. 1:* Ouais. En termes de santé.

*Mme. 2:* Ouais, voilà. Ça a pas l'air de-- enfin, on dirait pas que des pesticides ou juste comme ça, c'est-- Voilà.

*Mme. 1:* Ouais. On voit qu'il y a une vie de nature.

*Mme. 2:* Voilà. C'est ça. L'idée de nature, ouais écolo. Tout ça quoi.

*Mme. 1:* Mm-hmm ! [Euh] Est-ce que vous avez déjà eu une ou deux expériences avec ce type de lieux ou- ou pas vraiment ?

*Mme. 2:* Oui.

*Enfant [Enfant de l'interviewé]:* Oui [rire].

*Mme. 2:* Où ça ? [Ben] Av- Avant, on habitait dans un immeuble où derrière, il y avait un jardin partagé.

*Mme. 1:* OK.

*Mme. 2:* Voilà. Donc [euh] ça nous arrivait d'aller récupérer [euh] on allait récupérer quoi ? Des panais, [euh] des framboises, des fraises, de la menthe. Ouais, des petites choses comme ça.

*Mme. 1:* Ouais.

*Mme. 2:* Ouais. Et sinon, oui j'ai mon père qui a un jardin. Donc ça nous arrive aussi d'aller dans sa jardinière.

*Mme. 1:* Donc vous avez déjà consommé des produits qui--

*Enfant [Enfant de l'interviewé]:* Et quand je vais chez papy, je vois le jardin.

*Mme. 1:* Ah ouais. Papy a le jardin.

*Mme. 2:* Ouais. Oui, oui, oui, ça nous arrive de consommer du coup [euh] Mais c'est à l'occasion. Franchement, c'est pas--

*Mme. 1:* Ouais.

*Mme. 2:* Ouais. C'est vrai, c'est pas tout le temps.

*Mme. 1:* Et [euh] globalement, vous pensez quoi de ces produits qui viennent de- est-ce que vous pensez qu'ils font en particulier ou c'est classiquement des produits de--

*Mme. 2:* [Euh] Souvent, c'est plus petit que ce qu'on trouve dans le commerce.

*Mme. 1:* Ok.

*Mme. 2:* [Euh] En ce moment, y a pas d'engrais. Donc forcément, il faudrait laisser- laisser plus de temps, mais moderne de goût, c'est [euh] c'est plus ou moins similaire.

*Mme. 1:* Ouais.

*Mme. 2:* Ouais. Je vois pas une grosse différence avec ce qu'on peut trouver dans le commerce.

*Mme. 1:* Ouais. Ok. Est-ce que [euh] quand vous êtes dans ce genre de lieux globalement, comment vous- vous vous sentez ? Est-ce que c'est un lieu qui vous inspire ou au contraire, dans lequel vous vous sentez pas forcément bien ?

*Mme. 2:* Si, si, si, ça m'inspire. J'aime bien, parce que c'est un peu [euh] la chasse aux trésors. Voir un peu ce qu'on trouve quoi. Donc- et puis en plus de savoir qu'on peut les manger, [euh] c'est assez agréable. Mais [euh]

*Enfant [Enfant de l'interviewé]:* Chasse au trésor.

*Mme. 2:* Après, voilà, comme je disais, j'ai pas la main verte, parce que je suis en- en appartement et que c'est pas un endroit que-- Mes parents ont acheté une maison il y a pas très longtemps. Donc j'ai pas grandi avec un jardin en fait.

*Mme. 1:* D'accord. Même petite, vous aviez pas de--

*Mme. 2:* Ouais. Non. Donc [euh] voilà. Donc à l'occasion, c'est pas mal. Si j'en avais un, je ne sais pas si j'arriverais à le gérer. [Rire]

*Mme. 1:* Ouais. Et est-ce que vous avez le souvenir ou pas d'avoir [euh] une fois [euh] jeté des produits [euh] enfin qui venaient du jardin partagé quand vous étiez ou [euh] ou des produits de votre papa ? Ou vous avez pas le souvenir d'avoir ?

*Mme. 2:* [Euh] Si c'est jardin partagé, c'est déjà arrivé. Parce que j'avais ramassé des choses qui étaient pas si bonnes que ça. Les na- les na-- C'était des panais.

*Mme. 1:* Des panais, ouais.

*Mme. 2:* Ouais, qui étaient un peu [euh] je pense qu'ils étaient restés trop longtemps [euh]

*Mme. 1:* Ah ouais.

*Mme. 2:* Ouais. Du coup, ils ét- c'était pas super bon. Ils étaient trop filandreux par rapport à ce que d'habitude quoi.

*Mme. 1:* Oui.

*Mme. 2:* Mais [euh] Il a mis un œuf dans du vinaigre. Et il fait une expérience.

*Mme. 1:* Ah ! C'est quoi le-- Qu'est-ce que c'est censé ?

*Enfant [Enfant de l'interviewé]:* En fait, on doit mettre du- On doit mettre- on doit prendre un verre, on doit mettre du vinaigre et on a- on doit attendre jusqu'à demain, il devient- il dev-

*M. 2:* Il va devenir tout mou après.

*Mme. 1:* Il va deve- il va se ramollir.

*Mme. 2:* [Ben] Apparemment, il perd sa coquille plus ou moins. Je sais pas.

*Mme. 1:* Il est- il est dur là où il est-- Non.

*Mme. 2:* Non, c'est un œuf. Ouais. Il a vu ça dans un livre là.

*Enfant [Enfant de l'interviewé]:* Ouais. Et ensuite, on- et ensuite-

*M. 2:* Pas J'aime lire, pas J'aime lire. C'est Youpi.

*Mme. 1:* Youpi.

*Mme. 2:* Voilà.

*Enfant [Enfant de l'interviewé]:* Oui. Et ensuite, on peut- ça- ça peut éclater.

*Mme. 2:* Ouais. Voilà.

*Mme. 1:* À voir.

*Mme. 2:* Voilà, c'est ça. [Rire]

*Mme. 1:* Du coup, qui vous avez déjà dépanné.

*Mme. 2:* Voilà. Parce que ouais- parce que ils étaient trop [euh]- mais sinon, non.

*Mme. 1:* Oui.

*Mme. 2:* En général, quand on ramasse, on mange tout de suite.

*Mme. 1:* Ouais.

*Mme. 2:* Ouais.

*Mme. 1:* Donc vous avez jamais eu trop à stocker finalement des produits qui venaient de ce jardin.

*Mme. 2:* Non.

*Mme. 1:* Mm-hmm. Alors, une autre photo maintenant. Celle que vous voulez.

*Mme. 2:* Laquelle Edito ?

*Enfant [Enfant de l'interviewé]:* I.

*Mme. 2:* Ok. Inventaire des fruits et légumes. Alors, [ben] c'est un pain plus ou moins, parce que [euh] moi je vais jamais dans un endroit comme ça, où on vend que des fruits et légumes. Je suis toujours que dans les grandes surfaces.

*Mme. 1:* D'accord.

*Mme. 2:* Donc [euh] acheter des fruits et légumes, oui, mais jamais chez- chez un prim- prim- primeur ?

*Mme. 1:* Ouais.

*Mme. 2:* C'est ça ? Jamais.

*Mme. 1:* Vous avez pas d'expérience particulière avec [euh]--

*Mme. 2:* Non. Non, non. C'est vraiment à l'occasion quoi. Je trouve que tu es censé-.

*Enfant [Enfant de l'interviewé]:* [Inaudible 00:07:39-00:07:444]

*Mme. 2:* Ouais. Aux marchés, j'y vais jamais. On va presque jamais au marché.

*Mme. 1:* Ouais.

*Mme. 2:* Oui, il y a des raisins. Oui.

*Mme. 1:* Et là, vous voyez la photo, comment- comment vous la décririez ?

*Mme. 2:* Après, [euh] [ben] moi, j'adore. J'adore les fruits de-- Ça me donne envie tout ça.

*Mme. 1:* Ouais.

*Mme. 2:* Ouais. Ça me donne envie. Mais (euh] il faudrait que je retourne plus facilement le drive. Mais oui, sur les raisins, les clémentines, [euh] les bananes, les mangues, tout ça, on achète souvent. Fruits à coque, ça peut arriver aussi. On consomme tout, mais [euh] c'est vrai que j'y vais jamais. C'est parfois si je trouve que c'est plus cher.

*Mme. 1:* Ouais. C'est [euh] ça peut être une des raisons. Et est-ce qu'il y en a d'autres des raisons qui font que vous privilégiez ?

*Mme. 2:* Je passe mon chemin quoi.

*Mme. 1:* Oui.

*Mme. 2:* Donc ça, je passe pas du tout devant les primeurs.

*Mme. 1:* Ouais.

*Mme. 2:* Non, c'est pas-- Puis quand je veux acheter quelque chose, je veux acheter que des fruits et légumes. Donc je préfère aller dans une grande surface où je vais acheter [euh]

*Mme. 1:* Oui.

*Mme. 2:* Mon jambon, mes œufs ou autres et en même temps prendre des légumes que de- enfin, mes fruits que d'aller [euh] directement dans un magasin juste pour ça.

*Mme. 1:* Ouais.

*Mme. 2:* Mais oui, forcément, ça donne envie. On consomme beaucoup. [Inaudible 00:08:58-00:08:58] Tu as reconnu quoi ? Les raisins. Et ça ?

*Enfant [Enfant de l'interviewé]:* Les oranges.

*Mme. 2:* Et ça ?

*Enfant [Enfant de l'interviewé]:* Les bananes.

*Mme. 1:* Ouais. [Rire]

*Enfant [Enfant de l'interviewé]:* Des ananas.

*Mme. 1:* Alors- vous pouvez prendre n'importe quel autre.

*Mme. 2:* OK.

*Mme. 1:* Alors, vous avez dit que vous allez pas souvent-

*Mme. 2:* Ouais, au marché. Ouais, marché [ben] non, j'y vais pas souvent. J'y vais pas souvent. C'est vrai que les états là ça donne envie parce que c'est pareil. J'aime- j'aime bien.

*Enfant [Enfant de l'interviewé]:* C'est presque pareil.

*Mme. 2:* Mais [euh] ouais, non, jamais. J'ai pas eu l'occasion. C'est pas dans ma- dans mes habitudes. Pas encore, je me dis peut-être que plus tard, quand j'aurai plus de temps. [Euh] Je prendrais plus de temps dans les- à choisir mes fruits et légumes, d'aller faire marcher les- les producteurs locaux, parce que c'est aussi le but quand on va au marché. Mais [euh]

*Mme. 1:* Ouais.

*Mme. 2:* Ouais. Moi, je suis toute seule avec eux, j'ai pas le temps. Je fais des drives, je vais aller chercher et basta quoi.

*Mme. 1:* Ouais, ouais, ouais. Donc il y a le-

*Mme. 2:* Après, 'est un petit plaisir. Ça peut nous arriver [euh] peut-être deux fois dans l'année à l'occase, d'aller au marché. On prend 20 euros, on achète tous ce qu'on a envie comme fruits, légumes et compagnie. Mais [euh] c'est pas habituel du tout quoi.

*Mme. 1:* Ouais.

*Mme. 2:* Ouais.

*Mme. 1:* Si vous y allez, c'est [euh] que vous avez le temps. Et dans ce cas, c'est plus par plaisir que par nécessité.

*Mme. 1:* Ouais, c'est ça. C'est vraiment quelque chose que je mets dans le planning. C'est pas quelque chose dans lequel je vais [euh] systématiquement. C'est- c'est le diman- je m'attends un peu à- à ça oui, mais c'est vraiment [euh] au marché, ça oui. Mais c'est vraiment à l'occase quoi.

*Mme. 1:* Ouais. Et qu'est-ce qui fait que [euh] donc qui fait que ce soit pas une- une habitude de [Diaphonie 00:10:47-00:10:47] C'est manque de temps [euh] pour vous ? Et qu'est-ce qui fait que par contre, il peut y avoir du plaisir à y aller ? Selon vous, qu'est-ce qui fait que c'est plaisant ce- ?

*Mme. 1:* [Ben] Justement, c'est de pouvoir [euh] enfin, voir des choses qui viennent [euh] enfin, qui ont l'air plus naturelles quoi. Qui viennent de la terre, avec les cho- avec les commer-- enfin, les agriculteurs qui sont là. Ouais. Et puis [euh] [ben] les fruits et légumes en général, ils sont plus beaux qu'en supermarché aussi quoi.

*Mme. 1:* Ils sont plus beaux vous avez dit ?

*Mme. 2:* Ouais.

*Mme. 1:* C'est-à-dire ? Est-ce que vous pouvez développer ?

*Mme. 2:* [Euh] Voilà, on voit les choux. Ils sont beaucoup plus gros qu'en- qu'en surfaces, ils ont l'air plus frais. [Euh] Ça se voit qu'ils ont pas été trimballés pendant des heures dans des- dans des voilà, dans des camions, superposés les uns aux autres. Enfin, c'est [euh] voilà. Et puis il y en a quelqu'un qui m'ont conseillé quoi.

*Mme. 1:* Pour vous ?

*Mme. 2:* Ouais. Donc [euh] c'est l'avantage aussi si on cherche quelque chose en particulier. Je sais que souvent ça peut nous arriver que on cherche des pommes pour faire des compotes ou autre, [bah] il y en a tellement que quand eux sont là, ils peuvent nous dire lesquels sont les meilleurs. Enfin, voilà. Alors qu'en supermarché, il y a personne.

*Mme. 1:* Ouais. Ouais, ça pour vous, c'est quand même [euh] l'avantage d'avoir un interlocuteur.

*Mme. 2:* Voilà ! Ouais. Et c'est pour ça que c'est un plaisir d'aller [euh] au marché dans ce moment là. Mais c'est vrai que- après, petite, j'ai jamais été vraiment au marché avec mes parents aussi. Je pense que c'est peut-être [euh] ma façon. Après, on peut- enfin, on peut changer en grandissant, mais--

*Mme. 1:* Les habitudes. Et du coup, après.

*Mme. 2:* Ouais, ouais. Ouais, je pense.

*Mme. 1:* Donc vous avez déjà consommé de ces produits [euh] là ?

*Mme. 2:* Oui. Ouais, à l'oc, mais vraiment à l'oc.

*Mme. 1:* À l'occase, ouais. Et donc [ben] vous me dites ils sont plus beaux, ils ont l'air plus- plus frais tout ça. Est-ce que-- Mais avec vos expériences, est-ce que ça s'est confirmé ou- ou la qualité ne semblait pas ?

*Mme. 1:* Si, si, si. Surtout, [euh] je me rappelle quand- enfin, que j'achète souvent des oranges, des clémentines. [Euh] Elles sont plus- plus juteuses, plus sucrées.

*Enfant [Enfant de l'interviewé]:* Et plus bon.

*Mme. 2:* [Ben] C'est vrai. Des fraises aussi, ça nous arrive d'acheter. [Ben] Elles sont plus-- enfin, elles sont belles. Quand elles viennent de Plougastel, ça se voit qu'elles ont été cueillies presque le matin même quoi.

*Enfant [Enfant de l'interviewé]:* Elles sont plus bonnes.

*Mme. 2:* Que qui ?

*Mme. 1:* Ouais.

*Mme. 2:* Donc c'est pour ça que- enfin, ouais. C'est plus frais. Ça vient d'ici quoi.

*Mme. 1:* Ouais.

*Mme. 2:* Ça vient du coin en général.

*Mme. 1:* Et ça, [euh] cet aspect local, qu'est-ce qui- qu'est-ce qui vous plaît dans cet aspect [euh] ?

*Mme. 2:* [Ben] Déjà, c'est écolo- écologique. C'est [euh] ça fait marcher les petits producteurs.

*Mme. 1:* Ouais.

*Mme. 2:* [Euh] [Ben] On est sûr de- enfin, de ce qu'il y a dedans, on va dire. Et puis [euh] et puis ouais, ouais, c'est plus frais quoi.

*Mme. 1:* Ouais. Quand vous dites : On est sûr de ce qu'il y a dedans, c'est quelque chose qui parfois peut vous- inquiéter, je sais pas si c'est le mot. Mais c'est quelque chose que- auquel vous pensez des fois.

*Mme. 2:* Ouais, parfois, ça me questionne un peu. Quand je vois certains [euh] légumes qui peuvent rester [euh] des semaines sans- sans bouger.

*Mme. 1:* Ouais.

*Mme. 2:* Souvent les poivrons par exemple. Je peux avoir des poivrons, ils peuvent [euh] rester un mois, ils ne bougent pas.

*Mme. 1:* Ouais.

*Mme. 2:* [Bah] Je me dis pas qu'est-ce que normal ? Ça devrait pourrir à un moment. Bon, non, pas du tout. [Euh] Ouais, il y a certains légumes comme ça. [Euh] L'aubergine aussi, l'aubergine qui bouge pas trop.

*Mme. 1:* L'aubergine aussi, vous avez remarqué ?

*Mme. 2:* Ouais, enfin des fois- des fois je me pose la question, voilà Est-ce qu'ils mettent des choses pour que ça dure ou pas ? À l'inverse, les bananes elles pourrissent très très vite.

*Mme. 1:* Vos bananes pourrissent [euh] vous avez l'impression que--

*Mme. 2:* Ouais. Et je me dis que peut-être d'où ça- enfin, de là- enfin, entre le moment où elles sont cueillies et le moment où on les consomme aussi.

*Mme. 1:* Ouais.

*Mme. 2:* Voilà. Quand ça vient du- de la Côte d'Ivoire ou autre.

*Mme. 1:* Oui. Je sais pas.

*Mme. 2:* Donc je pense que dans les marchés comme ça. Et alors, ce qui est bien aussi quand on en achète au marché, c'est que peut-être on achète en quantité raisonnable. Je sais pas. Quand on drive, j'ai tendance à acheter [euh] puis je pars un peu dans tous les sens.

*Mme. 1:* Ouais, c'est sûr, oui. Au marché.

*Mme. 2:* C'est vrai- Ouais, voilà. On achète vraiment ce qu'on a besoin. Je pense qu'on a plus- voilà. On a plus [euh] la main dessus, les yeux aussi pour voir quelle quantité prendre. Qu'est-ce qui nous suffirait ? Puisqu'au drive, on prend un kilo, deux kilos, on se rend pas compte. Et puis parfois--

*Mme. 1:* Et vous pensez que ça peut avoir un impact sur [euh]

*Mme. 2:* Le gaspillage, ouais.

*Mme. 1:* Ouais.

*Mme. 2:* Ouais, ouais. Parce que des fois [euh] je dirais que des fois, on peut prendre deux kilos de pomme, deux kilos d'oranges, un kilo de bananes, puis en semaine, on n'a pas le temps de tout manger. Ou on mange pas à la maison cette semaine là. Ou j'en sais rien. Et puis du coup, [bah] gaspillage.

*Mme. 1:* Ouais. Ok. Ouais. Dernière illustration. Vous vous doutez de ce que ça peut-être.

*Mme. 2:* Ouais. Supermarché. [Rire] Supermarché [ben] ouais.

*Mme. 1:* Qu'est-ce qui vous vient [euh] à l'esprit ?

*Mme. 2:* [Ben]C'est la quantité surtout.

*Mme. 1:* Ouais.

*Mme. 2:* Quand on voit tout vu tout sur le- toute la quantité, c'est impressionnant quand même.

*Mme. 1:* Ouais.

*Mme. 2:* C'est en grande échelle quoi. C'est super. [Rire] Ouais. Après [euh] après, ouais, ça c'est- ça c'est plus mon côté dark on va dire.

*Mme. 1:* Ouais.

*Mme. 2:* Parce que c'est pratique quoi. On a tous les uns à côté des autres. On a tout le choix qu'on veut. On est sûr qu'on trouvera ce qu'on a besoin.

*Mme. 1:* Ouais.

*Mme. 2:* Et en même temps, parfois on est perdu.

*Mme. 1:* Si. [Rire] Est-ce que des fois vous êtes perdue ?

*Mme. 2:* Oui, oui, oui. [Bah] Et surtout une fois, dans-- enfin, que ce soit- c'est souvent au niveau des pommes ou des- des- des- des poires justement. Je suis un peu perdue, puisqu'il y a tellement de choix différents. Puis je sais jamais quoi prendre.

*Mme. 1:* Ouais.

*Enfant [Enfant de l'interviewé]:* Il y a plein de fruits.

*Mme. 2:* Ou je me perds dans les rayons, parce que je ne sais plus où est rangé telle chose. Donc des fois, je me balade. Je me balade, je me balade. [Rire]

*Mme. 1:* Je vois.

*Mme. 2:* Ouais. Et ça m'arrive d'oublier certaines choses du coup, parce que je passe à côté de certains rayons. Oh, arrête avec ça toi. Allez-, file manger et prends ton dessert. Ouais.

*Mme. 1:* Glo- globalement, quand vous êtes dans le supermarché, vous- vous allez par [euh] par nécessité de faut acheter, il faut acheter.

*Mme. 2:* Ouais ouais, c'est ça.

*Mme. 1:* Et [euh] comment vous vous sentez dans ce milieu là ? C'est un plaisir de faire ces courses ou est-ce que--

*Mme. 2:* Moi, ça dépend des jours. Après [euh] Ouais. Alors, c'est- je vais toujours au même supermarché pour être sûre de pas [euh] de pas [euh] passer des heures en fait. Donc j'ai toujours un supermarché dans lequel je vais et je trouve ce que j'ai besoin. Donc ça me permet de savoir plus ou moins où sont les choses.

*Mme. 1:* Ouais.

*Mme. 2:* Et après, un plaisir, ça dépend ce que j'ai besoin d'acheter. Mais c'est pour faire mes courses. Sinon, toujours en drive comme ça, je suis sûre de gagner du temps et l'énergie, on va dire. Et [euh] un plaisir, quand j'ai de- quelques petites choses à acheter, que je prends du temps à choisir si j'ai besoin quoi.

*Mme. 1:* Oui. Finalement, si on prend, c'est [euh] le plaisir que il est [euh] pour le plaisir de faire vos- vos- vos achats en tout cas, les légumes ou autre chose, ça va être si vous avez du temps.

*Mme. 2:* Ouais, c'est ça.

*Mme. 1:* Ok.

*Mme. 2:* Ouais. Et après- et franchement, c'est pas ma priorité.

*Mme. 1:* Oui.

*Mme. 2:* Mais vraiment.

*Mme. 1:* Et du plaisir à aller faire ses courses.

*Mme. 2:* Ouais, voilà, c'est ça en fait. Faire ses courses quoi. C'est un peu dommage, parce que ce qu'on mange, c'est un peu tant, mais [euh] je prends le temps de faire mon drive.

*Mme. 1:* Ouais.

*Mme. 2:* Voilà.

*Mme. 1:* Oui.

*Mme. 2:* Je les choisis quoi.

*Mme. 1:* Donc c'est un gain de temps le drive. Qu'est-ce qui vous plaît dans- dans le fait de driver ?

*Mme. 2:* [Bah] C'est- c'est- c'est le gain de temps.

*Mme. 1:* Ouais.

*Mme. 2:* C'est- c'est ça la priorité.

*Mme. 1:* Ouais.

*Mme. 2:* C'est que je peux rajouter dans mon panier tout ce que j'ai besoin au fur et à mesure dans la semaine et que je valide dans le moment avec ma carte bancaire effectivement. [Diaphonie 00:20:08-00:20:15] C'est la pratique quoi.

*Mme. 1:* Ouais.

*Mme. 2:* Puis avec eux, c'est pas possible. Ils sont trop bavards, ils sont trop [euh] dans tous les sens, c'est pas possible. Ils veulent tout acheter. Enfin--

*Mme. 1:* Ouais.

*Mme. 2:* Les enfants quoi.

*Mme. 1:* Ah oui, avec les enfants.

*Mme. 2:* Ouais, voilà, c'est ça. C'est pas possible. C'est pas possible donc-- Là, c'est- voilà, ma manière de consommer actuellement, c'est comme ça. Après, je pense- enfin, ça évoluera sûrement.

*Mme. 1:* Ouais.

*Mme. 2:* Faut que seulement à un moment du plaisir faire mes courses, quand j'aurai le temps et que j'aurais envie de-- Mais là, actuellement non. Clairement pas.

*Mme. 1:* C'est pas un [Diaphonie 00:20:52-00:20:56]

*Mme. 2:* Ouais.

*Mme. 1:* Ouais. Mais de toute façon, vous drivez donc--

*Mme. 2:* Ouais, ouais.

*Mme. 1:* Vous allez pas forcément au supermarché.

*Mme. 2:* Ah [ben] si, si. J'y vais, j'y vais quand même. Mais à l'oc- vraiment à l'occase quoi. Quand j'ai oublié quelque chose.

*Mme. 1:* Ouais. Quand il vous manque quelque chose.

*Mme. 2:* Ouais, c'est ça. Je vais dire peut-être deux fois par mois, mais pas plus quoi.

*Mme. 1:* Ok. Ok. Alors, autre petit jeu. Et il y a dix bananes. Vous mangez de la banane ?

*Mme. 2:* Oui, bien sûr.

*Mme. 1:* Je vais vous demander de ranger [euh] ces bananes, de celles qui vous donnent le plus envie à celles qui vous donnent le moins envie.

*Mme. 2:* OK. [Rire] Allez. Olala, celle-là est noire. Alors, ça dépend. Pour faire quoi ?

*Mme. 1:* Ah, ça va être justement des petites--

*Mme. 2:* Parce que nous, on mange parfois les bananes bien noires aussi, mais on les frit. Alors, celle-là. Pas facile. Ouais, on va commencer par celle-là. Celle-là. Je vois pas trop de différences entre ces deux-là.

*Mme. 1:* Elle est pas si différente.

*Mme. 2:* Ouais. Celle-là est plus verte.

*Mme. 1:* Vous ouvrez les- n'hésitez pas à les poser sur la--

*Mme. 2:* Ouais. Toutes celles-là, je pense que elles sont toutes à la fin. Alors, je pense que c'est comme ça. [Euh] Celle-là qui donne la plus envie. Après, celle-là, celle-là, celle-là, ensuite celle-là, celle-là. Off. Non, on va mettre celle-là d'abord. Je préfère manger celle que ça. Et après les autres.

*Mme. 1:* [Rire] Ok. Donc que je vrais prendre en photo l'arrangement que vous avez fait.

*Mme. 2:* Oui.

*Mme. 1:* Et hop. Alors, est-ce que vous pouvez m'expliquer pouvoir vous avez rangé dans cet ordre ?

*Mme. 2:* Oui. Alors, [euh] celle-là, c'est celle que j'ai l'habitude de consommer. Parce que ouais, [ben] les trois prem- ouais, les trois premières.

*Mme. 1:* Ouais.

*Mme. 2:* Souvent, on les achète comme ça.

*Mme. 1:* OK.

*Mme. 2:* On les consomme-- oui, on a mangé une [Inaudible 00:24:00-00:24:00]

*Mme. 1:* Ouais. Qui a l'air-

*Mme. 2:* Ouais, voilà. Entre les deux.

*Mme. 1:* Ouais.

*Mme. 2:* Celle-là- enfin, celles qui pour moi sont moelleuses et sucrées, on va dire. Voilà. J'ai rangé les bananes entre celles que on veut le plus manger, celle que on veut le moins manger. C'est les bananes, différentes bananes, il y en a une qu'on va- c'est- j'ai rangée par celle qu'on aime le plus manger et celle qu'on aime le moins manger.

*Enfant [Enfant de l'interviewé]:* C'est lesquelles qu'on aime moins ?

*Mme. 2:* Là, le plus et après le moins. Donc celle-là, je disais que celles-là, on les aime ben parce qu'elles sont sucrées [euh] et elles sont molles. Celles-là, [euh] ça va encore. Même si elle est un peu verte, elle sera un peu dure.

*Mme. 1:* Ouais.

*Mme. 2:* Ces deux-là là.

*Mme. 1:* Ouais.

*Mme. 2:* Mais celle-là, elle est en- elle est la même chose que celle-là.

*Mme. 1:* Alors, ouais, c'est un peu- Je crois que dans la- C'est elle la plus verte.

*Mme. 2:* Ouais, après celle-là.

*Mme. 1:* Et c'est elle, et après, c'est elle.

*Mme. 2:* Ouais. Ok.

*Mme. 1:* [Diaphonie 00:25:11-00:25:15]

*Mme. 2:* Je comprends pas grand-chose, mais [euh] là, quand c'est trop vert comme ça, non.

*Mme. 1:* Ouais, c'est-- Vous la mangez quand même cru quand c'est comme ça ?

*Mme. 2:* Non.

*Mme. 1:* Non.

*Mme. 2:* Lui il peut, moi je peux pas. j'ai- j'aime pas ça. Donc je préfère encore la manger un peu plus mûre que- que verte. Après, ça, [ben] on le mange, mais dans les gâteaux quoi. Enfin, à niveau- à ce- à stade-là encore, ça va, mais c'est deux là, on jette.

*Mme. 1:* Donc à partir du- c'est le rond.

*Mme. 2:* Ouais, c'est ça.

*Mme. 1:* Le rond orange.

*Mme. 2:* Voilà.

*Mme. 1:* Vous la mangez pas cru.

*Mme. 2:* Non, on va la manger cuite.

*Mme. 1:* D'accord. Et pour vous, là, le carré blanc.

*Mme. 2:* Oui.

*Mme. 1:* Vous la mangez pas non plus.

*Mme. 2:* Si, je peux la manger. Ouais. [Ben] J'attendrais qu'elle soit--

*Mme. 1:* Ouais.

*Mme. 2:* Mais si j'ai vraiment envie, je peux la manger. C'est pas non plus [euh]

*Mme. 1:* C'est pas votre banane parfaite ?

*Mme. 1:* Ouais, c'est ça. C'est encore un peu trop mûre pour moi.

*Mme. 1:* OK.

*Mme. 2:* Alors, je sais pas si il faut que j'attende qu'elle détache pour qu'elle soit bonne, mais-- [Rire]

*Mme. 1:* C'est ça. Donc là, vous m'avez dit que elle était moelleuse et sucrée.

*Mme. 2:* Oui. C'est ça.

*Mme. 1:* Et [euh] quand elles sont un peu moins mures, comment vous les qualifieriez avec vos sens ?

*Mme. 2:* [Ben] Plus dures.

*Mme. 1:* Plus dures.

*Mme. 2:* Et un peu plus [euh] pâteuse peut-être, je sais pas.

*Mme. 1:* Ouais.

*Mme. 2:* Enfin pâteux, c'est plutôt pour ceux-là.

*Mme. 1:* Ouais.

*Mme. 2:* Mais plus dures- mais ouais, je sais pas. C'est- c'est un peu comme une poire quoi.

*Mme. 1:* Ouais.

*Mme. 2:* Ça manque de sucré en tout cas.

*Mme. 1:* C'est pas assez sucré. Ouais. [Rire] Ouais, c'est ça. Et quand elle est au carré bleu ici, qu'est-ce que vous en faites ?

*Mme. 2:* [Ben) C'est la cuisine en général.

*Mme. 1:* Ouais.

*Mme. 2:* Ouais, je vais cuisiner.

*Mme. 2:* Ouais.

*Mme. 1:* Et que vous faites par exemple, ouais des gâteaux.

*Mme. 2:* Des gâteaux, ça nous arrive. Des pancakes.

*Mme. 1:* Ouais.

*Mme. 2:* [Euh] On fait quoi ? Des crumbles aussi des fois.

*Mme. 1:* Ouais.

*Mme. 2:* Mélangés avec des pommes. Et des smoothies aussi.

*Mme. 1:* Des smoothies, ouais.

*Mme. 1:* Donc [euh] là, vous la- vous at- vous attendez ? Ici, vous la cuisinez. Sur le triangle blanc, vous jet- vous jetez.

*Mme. 2:* Ouais.

*Enfant [Enfant de l'interviewé]:* Mais là, on dir- mais là maman, on dirait qu'ils sont pareil ces deux.

*Mme. 1:* Ouais, elles sont presque pareilles. Celle-là, elle est un peu plus--

*Mme. 2:* Ouais, moisie.

*Mme. 1:* Ouais. Et c'est- et c'est même [euh] rétréci.

*Mme. 2:* Ouais. [Rire]

*Mme. 1:* Ok. [Euh] Est-ce que selon vous, il y a un moment donné où [euh] la banane, elle perd [euh] ses apports, nutritifs ou pas ? Ou même, que ce soit au début. Enfin, la-- sur [euh] ces apports nutritifs. Est-ce qu'il y a un moment donné que le gout, on juge, on dit : Ouais, voilà c'était pas le même goût ? Ou du sucre ?

*Mme. 2:* [Euh] Pour moi au tout début, [euh] moins d'apport nutritif. Quand elle est verte.

*Mme. 1:* Ouais.

*Mme. 2:* Mais après [euh] elles conservent les mêmes, sauf quand elle vra- elle coule vraiment.

*Mme. 1:* À partir de quand ?

*Mme. 2:* Quand elle remonte un peu quoi.

*Mme. 1:* Ok. Au rond--

*Mme. 2:* Au rond rouge, ouais.

*Mme. 1:* Ouais.

*Mme. 2:* Pour moi, c'est- elle fermente et du coup, [euh]

*Mme. 1:* Et est-ce que selon vous, il y a moment donné où elle devient- elle peut être- enfin, elle devient ou elle est au départ [euh] un potentiel danger quand même pour la santé ?

*Mme. 2:* Non, non, non. Non. Pour moi, c'est un fruit. Ça risque pas de-

*Mme. 1:* Oui.

*Mme. 2:* Je sais que dans certaines iles, ils les mangent très vertes.

*Mme. 1:* Ouais.

*Mme. 2:* Et puis on- on le mange souvent chez un ami des bananes toutes vertes là, plantains. Ouais, il est frit. Et nous, on mange beaucoup les bananes [euh] noires comme ça, plantains aussi, mais frits aussi. Ça n'a pas du tout le même goût, mais [euh]

*Mme. 1:* Alors, quand elle est en- là le triangle blanc, vous la- vous pouvez vous la frire ?

*Mme. 2:* Là- pas celle-là. La banane plantain.

*Mme. 1:* La plan- Ok, c'est que--

*Mme. 2:* Celle qui est- [euh] enfin, c'est une banane, elles sont beaucoup plus grosses.

*Mme. 1:* OK.

*Mme. 2:* Ouais.

*Mme. 1:* Et elles, on peut attendre qu'elles soient [euh] noires. Enfin, il faut être noires.

*Mme. 2:* C'est meilleur.

*Mme. 1:* C'est meilleur ?

*Mme. 2:* C'est plus sucré. Mais à partir du moment où elles sont frits, elles ressemblent à des frites, donc si on les prend trop [euh] trop vertes, elles sont dures. Elles sont pas sucrées en fait. Ça ressemble plus à- à un légume qu'à un fruit. C'est-

*Mme. 1:* Ouais. Il faut goûter pour savoir. [Rire] Ok. Je vais voir si j'ai pas d'autres questions.

*Mme. 2:* Mais à l'occasion, si vous aimez les bananes, il faut vraiment [euh] que vous puissiez les bananes plantains.

*Mme. 1:* Et les frire. On est--

*Mme. 2:* Ouais. Ouais, ouais.

*Mme. 1:* J'aime bien la banane.

*Mme. 2:* Eh [ben] il faudrait. [Rire]

*Mme. 1:* Et en plus, je la mange- je la mange rarement cru. Je m'achète des bananes, mais elles finissent souvent au-- Je suis pas une fan de la banane crue comme ça.

*Mme. 2:* Ah ok d'accord.

*Mme. 1:* Soit je la mets le matin dans un- dans un petit bol ou alors si j'attends le fait de se jeter sur mon gâteau.

*Mme. 2:* Ouais [rire].

*Mme. 1:* Du coup, [ben] il faudrait que j'essaye de le faire frire.

*Mme. 2:* Ouais.

*Mme. 1:* Je crois qu'on a fait le tour de la banane. Alors, autrement [Inaudible 00:31:21-00:31:22] petite activité. Manger les bananes. Hop ! Voilà. Merci. Hop ! [Rire]

*Mme. 2:* [Inaudible 00:31:41-00:31:42]

*Mme. 1:* Ouais. Alors, je sais pas si vous m'avez envoyé tout à l'heure.

*Mme. 2:* Ouais [Rire].

*Mme. 1:* Du coup, vous voyez, ce sont vos- vos fruits et légumes que vous m'avez envoyés. [Euh] Est-ce que vous pouvez me les décrire ? Si [euh] ces morceaux, ces fruits.

*Mme. 2:* Oui. Alors, le premier, donc c'est du chou vert.

*Mme. 1:* Ouais.

*Mme. 2:* [Euh] C'est toute la pellicule de surface quand j'avais laissé [euh] ça, c'est un peu moisi dans le frigo en fait. Ça a noirci. Et puis il y a des petites taches brunes, tout ça. C'était devenu sec quoi forcément, vu que c'était [euh] à l'extrémité du- du fruit.

*Mme. 1:* Oui.

*Mme. 2:* Donc j'ai coupé tout ça. Plus quelques feuilles qui avaient [euh] ramollies. Voilà.

*Mme. 1:* Ok.

*Mme. 2:* Pouvoir consommer le reste. Le deuxième, c'est une banane que j'ai laissée [euh] enfin que- en fait, cette banane là, physiquement [euh] elle était normale.

*Mme. 1:* Ouais.

*Mme. 2:* Mais quand on l'a ouverte, [euh] tout-- enfin, ça se voit pas, mais elle était vraiment toute moisie à l'intérieur. De là à là, c'était tout mou quoi. Alors que je pense qu'elle a du s'ouvrir à un moment donné et que c'est moi-- Je sais pas. Elle ressemblait à une banane--

*Mme. 1:* Ouais, sa peau ne disait pas le--

*Mme. 2:* Voilà- voilà, le contenu, voilà. La mauvaise surprise de banane. C'est même lui je crois même qui le- qui l'a ouvert, qui dit : Maman, regarde. Donc voilà. Donc je- je-- alors, je pense pas qu'elle était très vieille en plus. Dehors une semaine, même pas.

*Mme. 1:* Ouais.

*Mme. 2:* Voilà. Ça, c'est une clémentine qui est restée [euh] dans le bac à fruit [euh] pourri. En fait, ce côté-là était tout pourri. Ce côté-là était orange. Et donc j'ai pas vu. Pourtant, on m- on met l'autre main deux à trois fois par jour dedans chacun. Mais je- je sais pas pourquoi.

*Mme. 1:* Donc là, cette clémentine, elle était [euh]

*Mme. 2:* [Ben] Physiquement, elle était pas mal de ce côté-là, dans le bac à fruit. Et du coup, j'ai pas du tout fait gaffe qu'elle était--

*Mme. 1:* De l'autre côté.

*Mme. 2:* Ouais. Il y en avait deux. Il y en avait deux.

*Mme. 1:* Deux qui étaient comme ça, dans cet état-là.

*Mme. 2:* Ouais. Côte à côte. Je pense qu'il y en a qui était--

*Mme. 1:* Ouais. Ouais. Ok. Alors, si on revient sur le chou. [Euh] Est-ce que vous pouvez me raconter son histoire ? Du coup, d'où il vient ? C'est-à-dire, [euh] forcément, vous vous l'avez acheté. Donc--

*Mme. 2:* Le chou, c'est- un- c'est un légume que j'achète à l'occasion, [euh] peut-être une fois par mois.

*Mme. 1:* Ok.

*Mme. 2:* Je le mets dans les potées. Je sais pas, il vient du driver. Franchement, je sais pas d'où il vient.

*Mme. 1:* Mais non, c'est [euh] Mais ma question, c'est [euh]

*Mme. 2:* Ouais.

*Mme. 1:* Du drive ou-

*Mme. 2:* Du supermarché, oui. [Euh] Il vient du petit supermarché et [euh] j'ai dû cuisiner une partie il y a deux semaines et vouloir cuisiner l'autre partie après. Donc forcément, [bah] tous les-- Donc voilà pourquoi j'ai coupé tous ces parties-là, parce qu'avec les mamans.

*Mme. 1:* Ouais. En fait, vous avez coupé en plusieurs [euh]

*Mme. 2:* Morceaux, en plusieurs fois. Ouais, c'est ça. Et en deux- les deux.

*Mme. 1:* Ouais. Pisser. Donc il était stocké dans le frigo.

*Mme. 2:* Dans le frigo.

*Mme. 1:* Avant qu'il soit entamé.

*Mme. 2:* [Euh] Non. Avant qu'il soit entamé, il était dans ma loggia.

*Mme. 1:* OK. Donc il a fait loggia.

*Mme. 2:* Ouais. Et après frigo.

*Mme. 1:* Puis en ta- enfin, en première entame, le frigo. Et là, [euh] du coup en- c'est quand- ça- après le frigo, la deuxième entame.

*Mme. 2:* Voilà, c'est ça. Ouais.

*Mme. 1:* Ok. Et [euh] qu'est-ce que ça vous fait de jeter ce [euh] chou ? Vous vous êtes dit quelque chose en particulier ou c'est [euh]

*Mme. 2:* Non, parce que [euh] clairement le chou était tellement gros que si je jette une petite tranche, ça me fait pas trop [euh]

*Mme. 1:* Ouais.

*Mme. 2:* Ça me fait pas trop de la peine, ça va.

*Mme. 1:* Ouais.

*Mme. 2:* C'est une petite partie du- on dirait qu'il y en a beaucoup regardé comme ça, beaucoup. Mais finalement, c'est vraiment une petite tranche sur la quantité de choux. Donc on a pu encore bien en manger.

*Mme. 1:* Ouais, la quantité jetée fait que ça--

*Mme. 2:* Voilà.

*Mme. 1:* Ça impacte--

*Mme. 2:* Enfin, moi- très peu sur [euh]

*Mme. 1:* Oui.

*Mme. 2:* Sur ce qui reste quoi.

*Mme. 1:* Oui. La quantité jetée et grande partie mangée quoi.

*Mme. 2:* Voilà, c'est ça. C'est pas trop grave.

*Mme. 1:* Ouais.

*Mme. 2:* Et en même temps, ça fait longtemps que-- Donc

*Mme. 1:* Ouais.

*Mme. 2:* C'était un peu normal.

*Mme. 1:* C'est un téléphone.

*Mme. 2:* Ouais, c'est le mien. Tu peux regarder [Enfant de l'interviewé] ?

*Enfant [Enfant de l'interviewé]:* OK.

*Mme. 2:* Pardon.

*Mme. 1:* Il y a pas de souci. [Rire]

*Mme. 2:* Leur père a amené sa mère. Je savais même pas qu'elle était là. Elle vient de- d'Afrique.

*Mme. 1:* Elle est là, elle est à Paris. Pour des vacances ou ?

*Mme. 2:* Ouais, ouais, pour des vacances. Ouais. C'est la première fois qu'elle quitte pas- son- son petit village.

*Mme. 1:* Ah ouais ! Ça doit faire tout drôle. Ouais

*Mme. 2:* Pour arriver à Paris. En plus, il fait froid.

*Mme. 1:* Ouais.

*Mme. 2:* Parce qu'en fait, c'est l'été là-bas. Ils sont en décalage.

*Mme. 1:* Puisque leur hiver, ça dépend d'où e- d'où elle vient. Mais [euh] même leur hiver--

*Mme. 2:* Oui, oui, il pleut beaucoup, mais il fait chaud. Ouais.

*Mme. 1:* Voilà. [Rire] Ouais, ça doit changer de- de décor. De température, de décor.

*Mme. 2:* C'est ça. [Rire]

*Mme. 1:* C'est marrant. [Euh] Alors du coup, [euh] sur la fin du chou, si vous avez jeté ces parties-là, c'est [euh] pourquoi ? C'est- donc elle- vous m'avez dit que elles étaient pas belles.

*Mme. 2:* Ouais, c'était flétri et [euh] il y a des petites tâches noires un peu. Ça commence à moisir un peu.

*Mme. 1:* Et donc vous pensez que en termes- une fois mangée, qu'est-ce que ça ferait [euh] ?

*Mme. 2:* [Ben] Je sais même pas. Puis je me suis jamais posée la question, mais [euh] comme physiquement, c'est pas beau--

*Mme. 1:* Ouais.

*Mme. 2:* Et que quand tu le coupes, [euh] c'est tout blanc, [ben] je me dis qu'il y a un truc qui ne va pas donc-- Mais en termes de goût, [ben] je suppose que ça doit être un- ouais, peut-être un peu plus fort en gout quoi.

*Mme. 1:* Ouais.

*Mme. 2:* Mm-hmm. Je me suis jamais vraiment posée la question.

*Mme. 1:* Oui, oui. C'est- des fois, on a le réflexe, on voit que c'est pas beau.

*Mme. 2:* Ouais, c'est ça. On jette- enfin, ouais.

*Mme. 1:* C'est pas beau, c'est pas bon.

*Mme. 2:* Ouais, c'est ça. C'est ça.

*Mme. 1:* Ok. Alors, la banane, on m'a dit- vous m'avez dit qu'elle avait une semaine. Même part, intéressante. [Euh] Pareille, donc elle devait devenir du drive.

*Mme. 2:* Ouais, je pense qu'elle était déjà sûrement abîmée quand ils me l'ont amené du drive. Parce que c'est pas possible que ce soit si pourri en si peu de temps.

*Mme. 1:* Ouais.

*Mme. 2:* C'était dans un sachet [euh] transparent. Ces sachets qui viennent sûrement de je ne sais quel pays. Avec le transport et tout.

*Mme. 1:* Ouais. Ou elles sont déjà- c'est pas celles qui- ouais, qui sont pas emballés forcément.

*Mme. 2:* Oui, voilà, c'est ça. [Ben] Des fois, au drive, [euh] on a beau choisir des bananes, ils nous mettent des bananes en sachet, alors qu'on commande des bananes au kilo. Enfin--

*Mme. 1:* Oui. Ouais.

*Mme. 2:* Sauf que elle était pas très--

*Mme. 1:* Ouais, elle est pas arrivée en forme.

*Mme. 2:* Voilà, elle est pas arrivée en forme. Et puis [euh] franchement, il y en avait qu'une. J'ai pas envie de la cuisiner. Ouais, ouais. Ça m'a pas donné envie.

*Mme. 1:* Ouais.

*Mme. 2:* Donc je l'ai jeté.

*Mme. 1:* Ok. Et [euh] la clémentine.

*Mme. 2:* Clémentine pareil, pas très vieille. Peut-être une semaine aussi.

*Mme. 1:* Ok.

*Mme. 2:* Ouais. Parce que j'ai mangé les autres, elles étaient très bonnes. Donc du coup, j'ai pas compris.

*Mme. 1:* Ouais. Mais pourquoi elle [euh] et sa copine--

*Mme. 2:* [Ben] Ouais, j'en sais rien, je sais pas. C'était les moucherons à la cuisine qui ont commencé. Mais je sais pas. Normalement, les moucherons font pas ça. J'en sais rien. Je sais pas.

*Mme. 1:* Ouais. Vous avez pas compris pourquoi c'est --

*Mme. 2:* Non. Peut-être que c'était- peut-être qu'il y en avait une qui avait déjà un petit peu une petite piqûre-- enfin, un petit peu de moisi et puis ça a entamé.

*Mme. 1:* Ouais. C'est possible.

*Mme. 2:* Peut-être que comme c'était en dessous, ça manquait un peu d'air et du coup, ça c'est- je sais pas.

*Mme. 1:* Ok.

*Mme. 2:* Ouais.

*Mme. 1:* [Euh] Et est-ce que là- donc là, en termes de- qu'est-ce que vous pensez si jamais vous êtes amenée à manger quelque chose comme ça en terme de goût ? Ou est-ce que selon vous, il y a un danger à manger cette- cette- ces parties là ? Ou non, c'est juste que ça va vraiment être pas bon.

*Mme. 2:* Oh ! Ça va être pas bon. Ouais, acide [euh]

*Mme. 1:* Ouais.

*Mme. 2:* Ouais.

*Mme. 1:* Ok. Alors, [ben] un petit jeu. Ici, j'ai [euh] des légumes.

*Mme. 2:* D'accord.

*Mme. 1:* Et ici, j'ai différentes choses qu'on peut faire avec. Et je vais vous demander déjà de regarder ce- ces différentes façons de- de cuisiner ou de jeter les fruits et légumes, de garder celles qui vous- qui vous parle. Avec lesquelles, enfin voilà, vous pouvez faire quelque chose. Et après, les- ranger ces fruits et légumes-là, en fonction de ce que vous aurez pu faire avec. Selon vous.

*Mme. 2:* Ok.

*Mme. 1:* Alors, les légumes ne sont pas très explicites, vu que c'est le premier entretien. Donc il y en a qui me disent qu'en fait, les images ils comprennent pas forcément. Donc n'hésitez pas à me dire si vous--

*Mme. 2:* [Euh] Moi, c'est plutôt ça.

*Mme. 1:* Ouais.

*Mme. 2:* Quand je dois cuisiner des légumes, c'est plus un- en poli en fait.

*Mme. 1:* Ouais, ok.

*Mme. 2:* Je crois que c'est plus près. Enfin, quoique la soupe, ça peut être pratique aussi. Mais--

*Mme. 1:* Vous en faites de temps en temps ?

*Mme. 2:* Ouais, à l'occase.

*Mme. 1:* Ouais.

*Mme. 2:* Parce que ils en- ils en mangent pas forcément eux. Et puis--

*Mme. 1:* Ouais. OK.

*Mme. 2:* Ouais, mais c'est ça qui me vient.

*Mme. 1:* Ok. Et- alors, c'est donc la soupe, c'est assez rare. Là, c'est un compost [euh]

*Mme. 2:* Ouais.

*Mme. 1:* Pas de poules.

*Mme. 2:* Non.

*Mme. 1:* Des conserves, vous en faites [euh] jamais. En gros, c'est une poubelle, une poubelle classique. Dons- alors, si vous compostez pas, vous avez une poubelle [euh] classique. Salade de fruits, vous n'en faites pas.

*Mme. 2:* Non.

*Mme. 1:* Non.

*Mme. 2:* Franchement, après-- Confiture non plus.

*Mme. 1:* Confiture [euh] si on pense au salé, [euh] des bolognaises que vous mettez en bocaux, jamais. Et des salades de crudités, non plus ?

*Mme. 2:* Ça oui.

*Mme. 1:* Si.

*Mme. 2:* Ouais, ouais.

*Mme. 1:* Je vais le mettre. Ça, c'est- alors, je vais le mettre, parce que je sais que vous cuisinez la banane. Du coup, je vais le garder. Je pense qu'il faudrait que je trouve une image où le- la banane est autrement. Ou enfin, ou les fruits sont en gâteau. Et d'essayer de cuisiner un peu les fruits quoi.

*Mme. 2:* Ouais, ok.

*Mme. 1:* En compote, en smoothie.

*Mme. 2:* Ouais ok.

*Mme. 1:* Et là, c'est la congélation de fruits et légumes. Est-ce que ça vous arrive de les combiner ou pas ?

*Mme. 2:* Ça arrive, mais vraiment rarement. Mais ça peut arriver.

*Mme. 1:* Ça peut arriver.

*Mme. 2:* Ouais. Ça peut arriver. C'est plus [euh] le persil, juste comme ça que j'en mets trop.

*Mme. 1:* Ouais, que vous--

*Mme. 2:* Compote de persil que je peux congeler.

*Mme. 1:* Ouais. Et donc là, vous avez des fruits et des légumes que [euh] d'autres participants ont jetés. L'idée, c'est de voir si vous [euh] vous les aurez jetés à ce moment-là ou si vous vous en aurez fait autre chose. Et de les ranger un peu là où [euh] en me disant un peu pourquoi. Et là où vous, vous les aurez jetés.

*Mme. 2:* OK. Bon mais évidemment la tomate moisie je jette.

*Mme. 1:* Ouais.

*Mme. 2:* Je sais pas si on peut couper, ressortir du bon. [Euh] Ça, j'aurais pu couper et- et manger.

*Mme. 1:* Ouais.

*Mme. 2:* Même manger sans cuisiner en fait.

*Mme. 1:* Ouais, c'est vrai que--

*Mme. 2:* Enfin, indirectement dans l'assiette quoi.

*Mme. 1:* Ouais. Donc ça-- J'aurais pu mieux-- Enfin, il faut que je ressorte la salade de fruits pour avoir un visuel. On dirait que vous pouvez la manger crue quoi.

*Mme. 2:* Ouais. Voilà, c'est ça. Ouais.

*Mme. 1:* Ok.

*Mme. 2:* [Euh] Ça- Ouais, non, la poire. On sait que quand ça commence à moisir, c'est pas très bon.

*Mme. 1:* Ouais.

*Mme. 2:* Je vais jeter aussi. Carotte, [euh] ça m'est déjà arrivé d'en manger.

*Mme. 1:* Quand il était dans cet état-là ?

*Mme. 2:* Ouais. À part être plus flétries. [Euh] Celle-là, [euh] à l'occase, j'aurais fait [euh] spécialement une soupe pour les mettre dedans.

*Mme. 1:* Ok. Mettre la soupe.

*Mme. 2:* Ouais.

*Mme. 1:* Justement. Et souvent c'est des stratégies [euh]

*Mme. 2:* Ouais.

*Mme. 1:* Ok.

*Mme. 2:* [Euh] [Bah) Ça, c'est ma banane. Je l'ai mise à la poubelle. [Rire]

*Mme. 1:* Banane poubelle.

*Mme. 2:* Celle-là, elle a quoi ?

*Mme. 1:* Je ne sais pas. Je n'ai pas encore fait l'entretien avec la personne. Mais la personne m'a envoyé cette tomate. Comme quoi, du coup, elle l'a jeté.

*Mme. 2:* Okay. [Ben] Non. Je n'en mange pas. Je peux la faire en sorte de crudités.

*Mme. 1:* Ouais.

*Mme. 2:* Ça, [euh] c'est pareil. Salade. Là les endives, non, je jette parce que je sais- enfin, les endives, ces trois sites. Et quand ça commence à pourrir, c'est pas bon.

*Mme. 1:* Ouais, ok.

*Mme. 2:* Poivrons, [euh] derrière moisi un côté, peut-être un peu flétri de l'autre. [Euh] Pourquoi pas ? Je l'aurais fait en-- en--

*Mme. 1:* On peut [Diaphonie 00:48:31-00:48:32]

*Mme. 2:* Ouais. Ça, c- c'est quoi ? Une patate douce ?

*Mme. 1:* C'est une carotte.

*Mme. 2:* Ah, c'est une carotte. Pareil, j'aurais jeté aussi. La couleur, elle me faisait penser à une patate douce. Je me disais est-ce que ça peut se manger. Et ça, c'est ?

*Mme. 1:* C'est des [euh] des fanes de poireaux, c'est des vers de poireaux.

*Mme. 2:* Moi, je jette aussi.

*Mme. 1:* Vous les jetez.

*Mme. 2:* Ouais.

*Mme. 1:* Ok. [Euh] Pourquoi le poivron [euh] ce poivron là, vous le- vous auriez tendance à le cuire ? À le faire empoiler ?

*Mme. 2:* [Euh] Parce que les poivrons, déjà, j'ai pas l'habitude de les manger en soupe.

*Mme. 1:* OK.

*Mme. 2:* [Euh] Ni cru.

*Mme. 1:* Ok.

*Mme. 2:* [Euh] Donc ouais, voilà, c'est plus simple. Mais après, j'aurais fait une ratatouille par exemple.

*Mme. 1:* Ouais.

*Mme. 2:* Voilà.

*Mme. 1:* Donc oui, voilà. Une cuisson.

*Mme. 2:* Ouais.

*Mme. 1:* Et [euh] les carottes ici un peu, comme elles sont. Pourquoi du coup en soupe [euh] ?

*Mme. 2:* [Ben] Parce que je pense que si on veut, elles ont du- enfin, ils ont perdu de leur croquant.

*Mme. 1:* Ouais.

*Mme. 2:* Donc en salade, c'est pas possible. On voit que ça se passera trop- pas trop bien. Donc c'est pour ça que mixer avec d'autres choses, peut-être une pomme de terre ou- ça passerait quoi.

*Mme. 1:* Ouais. Ok.

*Mme. 2:* Oui.

*Mme. 1:* C'est la texture du croquant qui est perdue.

*Mme. 2:* C'est ça, qui est perdue. Ouais, que en on soupe, ce serait plus facile.

*Mme. 1:* Ok. Puisque là, il y a plus du coup de notion de croquer le--

*Mme. 2:* Ouais, voilà, c'est ça. Mais le goût reste quoi.

*Mme. 1:* Ouais.

*Mme. 2:* Donc c'est pour ça.

*Mme. 1:* OK. Et là, selon vous, il y a- il y a-- Qu'est-ce qu'il fait que vous pouvez le manger cru ? Allez-y, pas de souci.

*Mme. 1:* Je vais prendre une photo après.

*Mme. 2:* OK. Oui, oui bien sûr. Oui.

*Mme. 1:* Du coup, et là c'est crudités, [euh] c'est- enfin, c'est oui.

*Mme. 2:* [Ben] Ouais, des crudités. Moi, salade, forcément c'est en salade. Et puis tomate, [euh] j'aurai plus la cuire aussi. Mais là, elle me parait bonne pour être mangée crue.

*Mme. 1:* Ok.

*Mme. 2:* Enfin, je peux aussi la manger cuite.

*Mme. 1:* Oui.

*Mme. 2:* Ça dépend ce que je veux faire.

*Mme. 1:* Mais du coup, c'est quoi vos attentes par rapport aux -- quelles sont vos attentes du cru par rapport aux cuit ?

*Mme. 2:* [Ben] Je veux que ce soit bien croquant quoi. C'est pour ça que une carotte bien fraîche, je peux la faire facilement encore drapée.

*Mme. 1:* Ouais.

*Mme. 2:* Si elle perd son croquant, je vais la mettre dans la soupe ou-- Mais c'est ce que j'attends d'un légume pour une salade, c'est que ce soit bien croquant quoi.

*Mme. 1:* Ouais, ok. C'est la texture plus que le goût en soit qui fait que--

*Mme. 2:* Ouais, c'est ça.

*Mme. 1:* Vous allez changer de- de préparation quoi.

*Mme. 2:* Ouais. Et [euh] [ben] la tomate- enfin, la- la- l'orange, ça m'arrive de mon-- Enfin, ça peut arriver. Là, mon- ma clémentine était trop pourrie, sinon j'aurai pu couper le morceau.

*Mme. 1:* Oui.

*Mme. 2:* Mais quand c'est un peu moisi comme ça, je jette ça.

*Mme. 1:* C'est en tout début de--

*Mme. 2:* Oui. Voilà, c'est ça. Faut le manger tout de suite quoi.

*Mme. 1:* Ouais.

*Mme. 2:* On coupe le morceau et puis là, il y a plus de la moitié quoi. C'est dommage.

*Mme. 1:* Ouais, oui, c'est ça. C'est pour ça que [Inaudible 00:51:54-00:51:55] parce que même si peut-être à l'intérieur, il y a peut-être une ou deux quartiers qui commencent à--

*Mme. 2:* Ouais, c'est ça.

*Mme. 1:* Pas terrible, qu'on peut enlever le reste.

*Mme. 2:* Ça reste mangeable quoi.

*Mme. 1:* Ouais, ça peut encore être bon. Bon, très bien. Et donc là, les vers de poireaux, de toute façon, vous les mangez pas.

*Mme. 2:* [Ben] Non, je sais pas, ça m'a jamais inspiré. Je jette aussi les miens.

*Mme. 1:* Ouais. Mais [euh] il y en a plein qui--

*Mme. 2:* Ouais, mais c- c- ça se mange non-stop je pense.

*Mme. 1:* Ça se mange- ça se mange, ça se mange. Du coup, c'est vrai que- la plupart des personnes [euh] du foyer qui participent et avec qui je m'entretiens, ouais ont tendance à plus les mettre dans des soupes. Parce qu'apparemment, ça a quand même- c'est tr-c'est très fort en goût.

*Mme. 2:* Ouais, c'est ça.

*Mme. 1:* Ouais, c'est bon. Ça- ça a plein d'apports aussi, mais c'est très fort en goût. Et [euh] la texture est quand même différente que le reste du poireau.

*Mme. 2:* On est d'accord.

*Mme. 1:* Et du coup, c'est vrai que ça passe apparemment mieux en soupe quoi.

*Mme. 2:* Ok.

*Mme. 1:* Et donc là, les endives, vous m'avez dit que par expérience [euh]

*Mme. 2:* Ouais, les endives, quand elles sont un peu moisies, elles sont trop acides.

*Mme. 1:* Ouais. Et-- Du coup, c'est le goût direct qui va vous--

*Mme. 2:* Ouais, ça me bloque.

*Mme. 1:* Elles peuvent être encore croquantes, mais le gout, ça--

*Mme. 2:* Voilà ! Quand elles sont bien fraîches, pareil [euh] je peux les manger comme ça en salade, mais à partir du moment où elles deviennent un peu [euh] comme ça, ah non, je peux pas.

*Mme. 1:* Oui, le goût c'est pas trop. Bon [ben] là, ça vous donnait pas envie. [Euh]

*Mme. 2:* Voilà.

*Mme. 1:* Elle était toute seule comme ça.

*Mme. 2:* Voilà. C'est ça.

*Mme. 1:* Celle-là, elle est trop.

*Mme. 2:* Ouais.

*Mme. 1:* Trop moi-même pour une soupe, elle est trop.

*Mme. 2:* Ouais, non.

*Mme. 1:* Là, c'est pareil.

*Mme. 2:* Oui, c'est pareil. Les poires, quand elles sont trop [euh] trop mures comme ça, elles sont très pâteuses.

*Mme. 1:* Ouais.

*Mme. 2:* J'aime pas.

*Mme. 1:* La texture, ça--

*Mme. 2:* Ouais, ouais. Ça me- ça me donne pas du tout envie.

*Mme. 1:* Et celles-ci ?

*Mme. 2:* Trop moisies.

*Mme. 1:* Trop moisies aussi.

*Mme. 2:* Ouais, on peut rien y faire. On peut rien récupérer je pense.

*Mme. 1:* Ouais.

*Mme. 1:* Ok. Bon [bah] super. Je pars prendre une petite photo.

*Mme. 2:* Oui.

*Mme. 1:* Et puis on est tout bon. Alors--

**Household F10, Interview 1**

00:00:04
*Speaker 1:* Alors... du coup, vous êtes [euh) prête?

00:00:08
*Speaker 2:* Oui.

00:00:10
*Speaker 1:* Pour commencer, je vous pose un petit jeu, un petit jeu de devinettes, je vais vous demander de penser à un fruit ou un légume que vous aimez particulièrement. Vous allez penser à ce fruit et à ce légume, sans me révéler son identité. Le but, c'est que je devine (euh) uniquement grâce à votre description.

00:00:29
*Speaker 2:* Faut des trucs qui sont faciles à décrire, du coup.

00:00:35
*Speaker 1:* Ouais à la fin, je fini toujours par l'avoir. Je l'ai- je vais l'éplucher jusqu'au bout .

00:00:46
*Speaker 2:* Euh... Ouais, c'est bon.

00:00:47
*Speaker 1:* Alors allez-y, je vous écoute, dites-moi tout (euh) ce qui vous vient sur ce fruit ou ce légume.

00:00:53
*Speaker 2:* Alors, (euh) on va commencer par le légume, (euh) c'est un long- un long légume assez- ben, assez fin on va dire--

00:01:04
*Speaker 1:* Ouais.

00:01:04
*Speaker 2:* (euh) qui a une forme un peu cylindrique, (euh) ce légume est vert, (euh) on le mange souvent en été--

00:01:16
*Speaker 1:* Ouais.

00:01:17
*Speaker 2:* Euh, on le mange froid, je crois, je crois qu'on le mange que froid, (euh) on peut l'éplucher ou pas, selon les préférences--

00:01:27
*Speaker 1:* D'accord.

00:01:29
*Speaker 2:* et (euh) c'est tout.

00:01:32
*Speaker 1:* Ouais.

00:01:33
*Speaker 2:* Euh, Je sais pas trop comment d'autre le décrire.

00:01:34
*Speaker 1:* Qu'est-ce que vous aimez tout particulièrement en ce légume?

00:01:40
*Speaker 2:* Euh, j'aime bien parce que, il est frais quand on le mange, (euh)... J'aime bien le goût, (euh) je sais pas.

00:01:58
*Speaker 1:* Ouais. Vous aimez le goût et son, du coup qui vous donne une sensation de fraîcheur donc ce serait apparemment plutôt froid, (euh) en général à quel moment de la journée vous le mangez? plutôt le matin, le midi ?

00:02:11
*Speaker 2:* Euh, midi ou soir.

00:02:17
*Speaker 1:* Midi ou soir.

00:02:17
*Speaker 2:* Ouais. Souvent, je le fais dans une salade. Je le mets dans une salade.

00:02:23
*Speaker 1:* Alors est-ce que vous pouvez me raconter les différentes étapes que vous faites (euh) avant de le consommer? C'est à dire (euh), il y a le légume. Qu'est-ce que vous faites avant de le manger?

00:02:31
*Speaker 2:* Je le nettoie.

00:02:32
*Speaker 1:* Ouais.

00:02:34
*Speaker 2:* Après, soit j'ai le courage de faire une salade et là je vais le couper soit en rondelles, soit en petits morceaux, ou soit j'ai la flemme de faire à manger et je le mange directement sans le couper.

00:02:46
*Speaker 1:* On croque.

00:02:47
*Speaker 2:* C'est ça.

00:02:51
*Speaker 1:* OK. Et vous, vous l'épluchez pas?

00:02:52
*Speaker 2:* Non.

00:02:53
*Speaker 1:* OK. Mais vous le lavez systématiquement?

00:02:58
*Speaker 2:* Non.

00:02:59
*Speaker 2:* Non. Non plus.

00:03:00
*Speaker 2:* Quand j'y pense.

00:03:00
*Speaker 1:* Ouais. Pas systématique. OK. Euh, En général, vous l'achetez, où, le plus souvent?

00:03:12
*Speaker 2:* Dans le rayon fruits et légumes des supermarchés.

00:03:16
*Speaker 1:* Ouais, OK... Ç marche (, euh) donc il vous amène de la fraîcheur et vous aimez le goût. Est c-e que vous lui trouvez d'autres- d'autres avantages ?Des choses que voilà, qu'est-q u'est-ce que vous ressentez quand vous le mangez, est-ce que vous avez e l'occasion donnée u'il vous apporte quelque chose? Plus du goût ?Ou c'est exclusivement (euh) ?--

00:03:41
*Speaker 2:* Non, c' est exclusivement fraîcheur et--

00:03:50
*Speaker 1:* OK. Et (euh) est-ce que vous lui trouvez certains inconvénients, ou pas vraiment?

00:04:03
*Speaker 2:* Euh... Non, je trouve pas d'inconvénients.

00:04:06
*Speaker 1:* Non? C'est le légume parfait.

00:04:08
*Speaker 2:* Oui.

00:04:08
*Speaker 1:* Et pis si non, si vous êtes- si vous capable de le manger comme ça, il est en plus pratique. Alors je dois deviner, et je dirai, le concombre.

00:04:25
*Speaker 2:* Oui.

00:04:27
*Speaker 1:* Ç marche. Alors maintenant, je veux qu'on discute plutôt de vos pratiques d (euh) 'achat et de votre organisation en général, donc notamment sur les fruits et légumes. En tout cas vos achats alimentaires, notamment les fruits et légumes. ( Euh, dcoup, vous, au sein de votre foyer, donc, (euh) si je me souviens bien, vous êtes en couple, plus en coloc'.?

00:04:49
*Speaker 2:* Oui, et on a un colocataire, ouais.

00:04:52
*Speaker 1:* OK. Au sein de votre foyer, du coup, qui est amené à faire des courses alimentaires?

00:04:57
*Speaker 2:* Euh, tous les trois.

00:04:58
*Speaker 1:* Tous les trois. Alors, est-ce que vous pouvez me raconter comment- comment vous vous organisez du coup?

00:05:04
*Speaker 2:* Euh, ben, quand on a plus rien dans les placards (euh), il y a quelqu'un qui va faire les courses, mais on planifie pas forcément qui va acheter quoi. (euh) Après si (euh) nous on va faire des courses, et que il y a- si le colocataire il voulait autre chose, ben, il va aller faire d'autres courses aussi, mais on planifie pas (euh) qui va faire les courses (euh) à intervalle régulier, quoi.

00:05:31
*Speaker 1:* OK. Euh, donc, au final, (euh) vous avez pas forcément une liste en commun de ce qu'il faut acheter ou pas.

00:05:37
*Speaker 2:* Non, on regarde ce qu'il y a dans les placards, ce qui nous donne envie aussi dans le magasin, on va surtout pour tout ce qui est fruits et légumes, on va prendre plus (euh) ce qui est de saison, donc on va voir ce qu'il y a, les prix, tout ça. On va pas faire une liste (euh) les trucs de base, si on a des- on fait une petite liste mais, c'est les trucs de base qu'on sait qu'il faut acheter à chaque fois donc (euh).

00:06:00
*Speaker 1:* D'accord, et (euh) donc sur les fruits et légumes quand- quand, ben, par exemple quand c'est vous qui allez au supermarché, vous savez pas forcément ce que vous allez acheter avant d'y aller ?

00:06:14
*Speaker 2:* Non, non je regarde (euh) pour ma part, moi c'est plus au niveau du prix que je regarde. Et comme je suis très difficile en légumes que j'en mange pas beaucoup, je prends que ce que j'aime la plupart du temps. Même si les autres veulent autre chose, je prends ce que j'aime.

00:06:33
*Speaker 1:* D'accord. Et en termes de fréquence, du coup (euh), même si (euh) vous disiez que c'était pas forcément régulier--

00:06:42
*Speaker 2:* Oui, c'est à peu près toutes les- toutes les- une fois- une fois par semaine qu'on va faire les courses.

00:06:46
*Speaker 1:* Ouais, il y a au moins une personne dans le foyer qui va faire les courses--

00:06:48
*Speaker 2:* (euh) Oui mais, à environ- ça fait une semaine qu'on l'a pas fait, mais (euh), ouais en général c'est toutes les semaines, parce que les fruits et légumes, comme en plus il fait chaud, ça tourne assez vite, donc (euh), on en prend peut-être un peu moins pour (euh) retourner en acheter.

00:07:06
*Speaker 1:* Et donc (euh), ça justement c'est, comment vous gérez les quantités que vous achetez, vu qu'enfin voilà, vous y allez sans trop savoir ce que vous allez prendre, sachant que vous aimez certaines choses et il y a la saison et le prix, donc du coup, comment vous êtes devant le rayon fruits et légumes, comment vous gérez les quantités que vous allez acheter?

00:07:23
*Speaker 1:* Un peu au pif.

00:07:23
*Speaker 1:* Ouais, c'est une stratégie.

00:07:27
*Speaker 2:* Ouais. Après, comme je prends pas souvent- pas tout le temps en vrac, par exemple (euh), je sais que c'est pas des légumes, c'est que des patates et on prend le grand sac de patates (euh), les carottes pareil. On prend un grand sac de carottes de, je sais plus combien de kilos, on prend souvent, on va essayer ça, c'est par rapport aux prix, donc (euh) c'est pas forcément ce qui est en vrac qui est le moins cher, donc (euh), on prend le moins cher.

00:07:49
*Speaker 1:* Ça va être ça plutôt la stratégie, c'est de--?

00:07:50
*Speaker 2:* C'est de trouver le moins cher.

00:07:55
*Speaker 1:* OK. Je comprends. OK. Et du coup (euh), donc, vous allez acheter (euh), bon il y a les élémentaires (euh), enfin des choses peut être que, même en commun là, qu'il faut que vous achetiez, sinon, chacun achète un peu de façon sur le moment ce qu'il préfère. Aimez-vous partager (euh) à trois les- toutes vos courses?

00:08:16
*Speaker 2:* Oui, mais sauf si (euh) je sais pas moi, j'ai un paquet de gâteaux qui est pour moi, je garde pour moi, je le dis, c'est mon paquet de gâteaux, voilà. Mais sinon, on partage tout.

00:08:26
*Speaker 1:* D'accord...OK. Euh, alors, est-ce que (euh) là, on pourrait dresser une (euh)- une liste des différents (euh) fruits et légumes que vous avez l'habitude de consommer?

00:08:47
*Speaker 2:* OK.

00:08:48
*Speaker 1:* Alors, si on prend, je sais pas comme voulez, soit les légumes, soit les fruits.

00:08:53
*Speaker 2:* Euh, les fruits, c'est plus rapide déjà.

00:08:54
*Speaker 1:* Alors les fruits.

00:08:56
*Speaker 2:* Alors en fruits, il y a pommes, bananes, (euh) là en ce moment, on aime bien prendre des fraises--

00:09:02
*Speaker 2:* Ouais.

00:09:02
*Speaker 2:* (euh) fruits, fruits, fruits (euh) en hiver c'est les clémentines--

00:09:10
*Speaker 1:* Ouais.

00:09:15
*Speaker 2:* (euh) après, on n en a pas acheté beaucoup en ce moment, mais sinon on aime bien avec mon copain, on aime bien prendre raisins et (euh) brugnons aussi.

00:09:23
*Speaker 1:* Ouais.

00:09:27
*Speaker 2:* Je crois que c'est à peu près tout. J'ai quelques abricots, mais là, on en mange moins.

00:09:32
*Speaker 1:* D'accord.

00:09:34
*Speaker 2:* J'ai tout en fruit.

00:09:36
*Speaker 1:* Et du coup sur là, sur les fruits (euh), est-ce qui y en a que vous connaissez (euh), mais voilà, que vous- que vous ne consommez que très rarement, voire jamais?

00:09:47
*Speaker 2:* Ben oui hein, il y en a plein! (euh) qu'on consomme pas beaucoup, rarement. Ben, les poires--

00:09:55
*Speaker 2:* Ouais.

00:09:59
*Speaker 2:* (euh) mangues, noix de coco, c'est rare qu'on en prenne, mais on aime bien en prendre--

00:10:02
*Speaker 2:* Ouais.

00:10:04
*Speaker 2:* (euh) les pomelos, il paraît que c'est très rare, (euh) melon, c'est un fruit ou c'est un légume ?

00:10:12
*Speaker 2:* C'est un fruit, mais (euh)--

00:10:12
*Speaker 2:* Ben ouais, melon on en consomme régulièrement en été aussi.

00:10:18
*Speaker 1:* OK.

00:10:19
*Speaker 2:* Je sais jamais.

00:10:26
*Speaker 1:* Je suis en train d'essayer de regarder dans ma sacoche. Je sais pas.

00:10:26
*Speaker 1:* Alors en fruits (Mmmm)

00:10:32
*Speaker 1:* Si on (inaudible 00:10:32- 00:10:33) que vous avez de la poire, pourquoi vous la consommez, rarement voire jamais ?

00:10:38
*Speaker 2:* Moi, j'aime pas les poires.

00:10:41
*Speaker 1:* D'accord.

00:10:41
*Speaker 2:* Donc (euh), j'en consomme pas.

00:10:41
*Speaker 1:* Ouais. Vous avez dit (euh) la mangue, la noix de coco, vous aimez bien mais--

00:10:47
*Speaker 2:* Ouais la mangue c'est souvent assez cher, c'est bon, mais c'est souvent assez cher. La noix de coco, on aime bien aussi, mais c'est tellement horrible à ouvrir que, on prend pas forcément. Et après, pomelos pareil moi j'aime pas ça, il y a que mon copain qui aime et (euh) il les prend pas en grande surface, il prend dans une épicerie chinoise, par de gros pomelos, pas des tout petits.

00:11:11
*Speaker 1:* D'accord.

00:11:12
*Speaker 2:* Du coup, on prendra pas souvent dans l'épicerie chinoise et ils ont en pas tout le temps (euh)--

00:11:20
*Speaker 1:* Et sinon (euh), que ce soit vous, votre conjoint et (euh) votre colocataire, vous allez au même supermarché pour faire vos courses ou--?

00:11:31
*Speaker 2:* Euh, bonne question. Euh, globalement, on va dans les mêmes, mais en fait, on va dans, un peu beaucoup de supermarchés selon nos envies--

00:11:43
*Speaker 1:* Ouais.

00:11:45
*Speaker 2:* Euh, Je crois que oui, on veut à peu près dans les mêmes.

00:11:47
*Speaker 1:* D'accord et qu'est-ce qui dicte un peu, voilà pourquoi vous choisissez ces supermarchés là plutôt que d'autres?

00:11:53
*Speaker 2:* Euh, par exemple, on va aller au- quand on va aller au Lidl avec mon conjoint, c'est parce que on veut du poulet Knacki et y en que là-bas.

00:12:03
*Speaker 2:* OK.

00:12:03
*Speaker 2:* Euh, quand j'ai le courage d'aller jusqu'au Carrefour. C'est surtout pour l' ice tea. Parce que on boit que de l'al- que du froid ici avec mon copain, sauf que il y a que celui de Carrefour qui est sans sucre, donc que Leclerc, c'est souvent parce que j'ai pas le courage de faire beaucoup de route et c'est le magasin le plus près. Netto, c'est parce qu'il est à côté des FAC, donc quand je sors des cours, je vais passer par Netto.

00:12:24
*Speaker 1:* OK.

00:12:26
*Speaker 1:* Et voilà.

00:12:28
*Speaker 1:* D'accord. Donc et par praticité et par (euh) pour aller chercher un produit en particulier. Et du coup, les fruits et légumes sont susceptibles de venir de ces quatre là si jamais à un moment donné (euh)--

00:12:42
*Speaker 2:* Oui, ouais. Après Netto un peu moins, je crois, encore moins de fruits et légumes à Netto, il y a pas grand chose non plus. Pas très intéressant.

00:12:52
*Speaker 1:* D'accord. Et vous direz que c'est où le plus (euh)--?

00:12:56
*Speaker 2:* Celui où en prend le plus, c'est, je pense Lidl.

00:12:59
*Speaker 1:* OK. Les fruits et légumes ce serait là le, plus loin, quoi. OK, ça marche. Alors si on revient aux- aux fruits, (euh) alors, par exemple, vous mangez des fraises, des bananes, la mangue vous aimez bien, mais c'est (euh) assez cher, la noix de coco, c'est pénible à ouvrir. C'est vrai. (Euh) Est-ce que il y a, au niveau des fruits de la passion, les fruits exotiques, qu'est-ce que- est-ce que vous en consommez à part (euh) ben quelques fois la mangue et--

00:13:37
*Speaker 2:* Euh...

00:13:37
*Speaker 1:* Ou non, les ananas?

00:13:38
*Speaker 2:* Ah, j'ai oublié les ananas! Moi j'aime pas ça mais (euh), je sais même pas si on en a déjà acheté... Je crois que l'ananas on prend en conserve.

00:13:52
*Speaker 1:* Ouais.

00:13:55
*Speaker 2:* Je crois pas qu'on a acheté de- d'ananas frais, parce que déjà c'est grand donc il y en a beaucoup à manger, donc en conserve, c'est beaucoup plus pratique.

00:14:01
*Speaker 1:* Ouais.

00:14:02
*Speaker 2:* C'est moins bon, mais c'est plus pratique.

00:14:06
*Speaker 1:* OK. Euh, d'accord. Donc plutôt investir sur l'ananas, est-ce qui y en a d'autres comme ça, des fruits que vous allez acheter plutôt en conserve ou même, en surgelé parfois, ou non?

00:14:22
*Speaker 2:* Euh, fruits en surgelés non, (euh) en conserve, quelques fois on prend (euh) la mangue aussi en conserve--

00:14:30
*Speaker 1:* Ouais.

00:14:33
*Speaker 2:* Et (euh) quelques fois, des poires en conserve pour faire des gâteaux aussi.

00:14:39
*Speaker 1:* OK.

00:14:43
*Speaker 2:* Mais c'est ça.

00:14:50
*Speaker 1:* D'accord. Euh, des cerises, par exemple.

00:14:55
*Speaker 2:* Ah oui, ben les cerises, on n'en n'achète pas souvent. On nous en donne. Quand quelqu'un a un cerisier dans le jardin, on arrive à en récupérer.

00:15:03
*Speaker 1:* D'accord. Chouette! OK. Donc- donc finalement, les fruits que vous allez pas consommer, soit que vous les aimez pas trop, ou bien d'ailleurs peut-être le prix qui va (diaphonie 00:15:15- 00:15:17)

00:15:17
*Speaker 2:* C'est ça ouais, il y a le prix (diaphonie 00:15:15- 00:15:17) .

00:15:18
*Speaker 1:* Et (euh) pis ben des fois le côté un peu pratique, ouais l'ananas c'est (inaudible 00:15:19- 00:15:22). Et (euh) les pêches, vous m'avez dit brugnons aussi--

00:15:29
*Speaker 2:* Ah les pêches, oui aussi, ouais si

00:15:32
*Speaker 1:* Ç peut arriver?

00:15:32
*Speaker 2:* Oui mais, brugnons, pêches je mets tous dans la même case. Donc (euh), je fais pas trop la différence.

00:15:35
*Speaker 1:* (inaudible 00:15:35- 00:15:37)

00:15:37
*Speaker 2:* C'est ça.

00:15:40
*Speaker 1:* OK. Alors, les légumes (euh), désormais, les légumes que voilà, ceux que vous allez avoir très régulièrement chez vous (euh)--

00:15:48
*Speaker 2:* Alors, carottes- des carottes--

00:15:50
*Speaker 1:* Ouais.

00:15:50
*Speaker 2:* (euh) poivrons, courgettes, concombres--

00:15:56
*Speaker 1:* Mouais.

00:16:03
*Speaker 2:* (euh)... (euh) poireaux--

00:16:03
*Speaker 2:* Ouais.

00:16:04
*Speaker 2:* (euh) j'allais dire patates mais pas du tout.

00:16:10
*Speaker 1:* Mais si ben vous êtes- tiens, on peut les--

00:16:13
*Speaker 2:* Mais c'est pas vraiment des légumes.

00:16:14
*Speaker 1:* Ouais. Mais on les compte (euh)--

00:16:17
*Speaker 2:* Euh, alors qu'est-ce qu'on aime bien? Qu'est-ce qu'on prend souvent en légumes? Ben, des oignons--

00:16:22
*Speaker 1:* Ouais.

00:16:33
*Speaker 2:* (euh) Je sais plus- je sais pas.

00:16:33
*Speaker 1:* ça peut être ceux que vous consommez que--

00:16:39
*Speaker 2:* Ouais, parce que c'est ce qu'on va consommer le plus.

00:16:42
*Speaker 1:* Par exemple, est-ce que vous consommez ou pas (euh) de la salade?

00:16:50
*Speaker 2:* Euh, pas trop. Et en plus (euh), ben maintenant qu'on a le potager, on a de la roquette et de salade dans le potager.

00:16:56
*Speaker 1:* OK. Vous avez un potager?

00:16:58
*Speaker 2:* Oui.

00:17:00
*Speaker 1:* Salade et roquette. Est-ce que- qu'est-ce que vous avez d'autre dans le potager?

00:17:06
*Speaker 2:* Alors (euh), on n'a pas encore pu en consommer parce que c'est pas encore prêt, mais on a carottes, choux frisés, choux-fleurs, betteraves et pommes de terre. Et fraises et tomates.

00:17:20
*Speaker 1:* Betteraves, tomates, fraises, salade, roquette, carottes, choux fleurs, choux frisés, betteraves, tomates, fraises--

00:17:29
*Speaker 2:* Et patates.

00:17:29
*Speaker 1:* Et patates. Et du coup c'est--

00:17:33
*Speaker 2:* Ah il y a la tomate aussi qu'on consomme régulièrement, j'ai oublié ça.

00:17:35
*Speaker 1:* Ouais. Donc là par exemple, vous n'en avez pas encore des--?

00:17:36
*Speaker 2:* Non, on- on a juste la roquette qui comment- que on prend un peu de temps en temps, dans le jardin, et tout le reste n'est pas encore prêt.

00:17:47
*Speaker 1:* D'accord. Du coup, quand c'est- enfin quand c'est pas prêt dans le potager, vous allez (euh)--

00:17:53
*Speaker 2:* On va en acheter.

00:17:56
*Speaker 1:* Vous allez en acheter. OK. Et la salade du coup, vous en achetez--

00:18:01
*Speaker 2:* Ben la salade (euh), j'en achetais beaucoup à un moment, mais j'en achète beaucoup moins. Parce que à chaque fois, je- j'adore la salade en sachet. J'aime pas la salade fraîche.

00:18:12
*Speaker 1:* D'accord.

00:18:13
*Speaker 2:* Je crois que ma- que la laitue elle se gâte dans le sachet, parce que c'est ce que celle là que j'aime. Mais (euh) chaque fois, je la laissais périmer dans le frigo, donc j'ai arrêté d'en acheter.

00:18:21
*Speaker 1:* Ouais. OK. Qu'est-ce qui fait que vous la prépariez pas forcément, finalement?

00:18:28
*Speaker 2:* Euh, ben parce que (euh), ben... Souvent je commençais le paquet, pour faire une salade, tout ça, mais (euh) j'avais pas l'occasion de- je pensais pas à le finir quoi, il restait au fond du frigo caché par tout le reste et--

00:18:45
*Speaker 1:* Ouais. Ouais c'est une fois que c'était ouvert, après--

00:18:46
*Speaker 2:* C'est ça.

00:18:50
*Speaker 1:* OK. Est-ce que vous consommez de l'artichaut ou pas?

00:18:56
*Speaker 2:* Non.

00:18:56
*Speaker 1:* D'accord et pourquoi alors, par exemple l'artichaut, pourquoi ça vous--

00:19:00
*Speaker 2:* Euh... (euh), ben, je sais pas. Euh, ben, déjà de base, moi je- je- c'est pas moi qui achèterais des légumes- des légumes, vu que je ne suis pas fan, et ouais, c'est mon copain qui achète plus les légumes et là, c'est vrai qu'il n'en a jamais acheté.

00:19:23
*Speaker 1:* D'accord.

00:19:24
*Speaker 2:* Je sais pas pourquoi.

00:19:27
*Speaker 1:* C'est forcément par goût, c'est --

00:19:28
*Speaker 2:* Ben, moi je- moi c'est par goût mais (euh) après pour les autres, je sais pas.

00:19:34
*Speaker 1:* D'accord. Et donc, vous dans ceux que vous m'avez cités, vous les mangez tous là, les carottes, les poivrons?

00:19:38
*Speaker 2:* Euh, tout ce qui est chou, j'aime pas trop--

00:19:40
*Speaker 1:* D'accord.

00:19:40
*Speaker 2:* mais après (euh), si je pouvais je mangerais pas de légumes.

00:19:44
*Speaker 1:* OK.

00:19:44
*Speaker 2:* Mais (euh) courgettes, concombres, tomates, poivrons, ça passe.

00:19:51
*Speaker 1:* Ouais.

00:19:53
*Speaker 2:* Après poireaux en soupe, ça passe. Après, c'est assez- faut trouver à chaque fois un moyen de me faire manger le légume, sans que je vois que c'est le légume.

00:20:04
*Speaker 1:* Ah ouais? (Rires) Parce que du coup sans- vous dites sans que vous voyez que c'est le légume, il y a--

00:20:11
*Speaker 2:* Ben, je- j'ai beaucoup de mauvaises- quand je sais ce que c'est le légume, automatiquement, je suis (euh)- j'aime pas. Donc (euh), je vais pas aimer. Si je sais trop, par exemple, si on fait en soupe, on voit pas ce qu'il y a comme légumes dedans et ben, ce sera un peu plus facile déjà à- je saurai pas exactement ce que c'est.

00:20:27
*Speaker 1:* D'accord.

00:20:28
*Speaker 2:* Et j'aurai pas le goût avant de- d'avoir goûté.

00:20:33
*Speaker 1:* Des fois, si vous savez que c'est un légume qui est à la base, que vous aimez pas trop, si vous voyez--

00:20:36
*Speaker 2:* Ah, je- je mangerai pas.

00:20:37
*Speaker 1:* D'accord. Mais si au final, en mangeant et que vous étiez pas au courant, ça passe?

00:20:42
*Speaker 2:* Ben, je tire la tête mais--

00:20:43
*Speaker 1:* Si on vous a dit après, il y avait du chou. D'accord, vous êtes pas très (euh), très légume.

00:20:56
*Speaker 2:* Non.

00:20:56
*Speaker 1:* Et c'est pas un goût ou il y a autre chose, du fait que- enfin je sais pas, est-ce que c'est vraiment sensiblement au goût qu'il y en a aucun qui vous plaît?

00:21:03
*Speaker 2:* Ben, il y a eu le goût déjà, après, je pense aussi quand j'étais petite, on- c'était que de la soupe tout le temps que je mangeais le soir. Et ça m'a dégouté des légumes.

00:21:10
*Speaker 1:* OK.

00:21:11
*Speaker 2:* Je pense qu'il y a de ça aussi, mais ouais, c'est surtout par goût et (euh) je pense que- et j'ai pas envie de faire d'effort. Je pense aussi.

00:21:18
*Speaker 1:* Ouais.

00:21:21
*Speaker 1:* OK. Du coup, justement les- parce que le concombre, vous disiez que vous le mangiez du coup (euh) froid ou cru, est-ce qu'il y a des légumes que vous allez aimer manger cuits ou pas?

00:21:36
*Speaker 2:* Cuits. Eh ben, les courgettes.

00:21:38
*Speaker 1:* Ouais?

00:21:43
*Speaker 2:* Euh, cuits, les carottes- (euh) ouais, carottes c'est soit froid, soit chaud. Ouais ça dépend.

00:21:46
*Speaker 1:* Ouais.

00:21:46
*Speaker 2:* Euh, cuits ben, il y a les pommes de terre.

00:21:50
*Speaker 1:* Ouais.

00:21:52
*Speaker 2:* Euh, qu'est qui- qu'est-ce que je mange?

00:21:59
*Speaker 1:* Les tomates, vous les mangez cuites ou--?

00:22:00
*Speaker 2:* ça dépend, je préfère froides--

00:22:03
*Speaker 1:* Ouais.

00:22:04
*Speaker 2:* mais cuites, ça peut passer.

00:22:05
*Speaker 1:* D'accord.

00:22:05
*Speaker 1:* En poivrons cuits, poivrons (euh) j'aime pas trop cuits, je préfère (euh) froids.

00:22:13
*Speaker 1:* Ouais.

00:22:17
*Speaker 2:* Et (euh)--

00:22:19
*Speaker 1:* OK. Je vois. (Euh) les aubergines pour vous c'est--?

00:22:26
*Speaker 2:* Dans la ratatouille, c'est tout.

00:22:29
*Speaker 1:* On la voit pas trop.

00:22:31
*Speaker 2:* C'est ça.

00:22:36
*Speaker 1:* OK. (Euh) ça marche, qu'est-ce que j'avais d'autre en tête? Les courges. Est-ce que ça vous arrive de consommer des courges ou--?

00:22:44
*Speaker 2:* Courges- courges c'est les courgettes rondes?

00:22:46
*Speaker 1:* Il y a les- il ya les potimarrons, il y a les butternuts, c'est les- c'est les vieux légumes--

00:22:54
*Speaker 2:* Ah! Oui, butternuts ah mais des- ouais, ah non on n'a plus. On avait des butternuts, non, on n' en a plus. (Euh) Non butternuts (euh) j'aime pas. (Euh) Potimarrons, je crois pas en avoir compté.

00:23:06
*Speaker 1:* C'est- ça ressemble à une citrouille.

00:23:10
*Speaker 1:* Ouais, non, ça me dit rien.

00:23:16
*Speaker 1:* (Inaudible 00:23:12- 00:23:15) C'est la même famille. Si vous aimez pas le potiron, vous pouvez essayer quand même, c'est ça. OK. (Euh) Est-ce que j'ai d'autres légumes qui viennent en tête?

00:23:29
*Speaker 2:* Mais si on aura des- on doit planter aussi des citrouilles géantes dans le potager.

00:23:32
*Speaker 1:* Ah ouais? Pour les consommer?

00:23:35
*Speaker 2:* Oui.

00:23:38
*Speaker 1:* Pour votre plus grand bonheur ou--?

00:23:39
*Speaker 2:* C'est qu'elles seront immenses.

00:23:41
*Speaker 1:* Ouais.

00:23:42
*Speaker 2:* Ç fera beaucoup à manger.

00:23:48
*Speaker 1:* OK. Alors, (euh) du coup, il y a pas mal de légumes que vous allez pas manger? Oui, et c'est principalement par goût que vous allez (euh) vous en passer. OK. (Euh) Si on s'intéresse maintenant à vos, lieux de stockage, donc quand vous revenez des courses, qu'est-ce que vous faites de vos fruits, qu'est-ce que vous faites de vos légumes?

00:24:16
*Speaker 2:* Alors tous les légumes, on les met (euh) sur les étagères dans la pièce à côté.

00:24:24
*Speaker 2:* D'accord.

00:24:24
*Speaker 2:* On les (euh)- souvent on les enlève des sachets parce que depuis qu'on est en été, on a remarqué que le soleil est sur l'étagère, quelque part toute la journée. Du coup,(euh) on les enlève automatiquement des sachets, sinon ça pourrit plus vite. Et les fruits, on les amène à l'intérieur, sur la petite table où on met tous nos fruits.

00:24:49
*Speaker 1:* D'accord.

00:24:50
*Speaker 2:* Et s'il fait trop chaud comme là, l'autre jour, les fraises, on les a mises au frigo parce que, elles tenaient pas sinon.

00:24:59
*Speaker 1:* OK. Donc normalement, il n'y a pas de fruits et légumes au frigo, sauf si--

00:25:05
*Speaker 2:* Ou (euh) quand- quand par exemple le concombre, là que j'ai commencé (euh), il y en a certains de mes concombres, que je mets au frigo.

00:25:09
*Speaker 1:* OK.

00:25:10
*Speaker 2:* Mais, on essaye ouais, on n'a pas un grand frigo, donc (euh), et la buanderie faisait froid en hiver. Du coup, ça faisait- c'était bien pour les légumes, mais là, on commence à envisager de trouver un autre endroit pour l'été, pour mettre les fruits et légumes.

00:25:23
*Speaker 1:* D'accord, parce qu'au final, [inaudible 00:23:00- 00:25:00]

00:25:25
*Speaker 2:* Ben, il fait vite chaud. Et comme c'est au soleil (euh), ça- ça tourne plus vite.

00:25:30
*Speaker 1:* OK. Et donc (euh), là, si vous avez pas de contraintes matérielles ni financières, qu'est-ce que vous ferez pour (euh)- ce serait quoi votre stockage idéal de fruits et légumes? Si vous en avez un. [Inaudible 00:25:46- 00:25:47]

00:25:51
*Speaker 2:* Ben, cer- certains légumes, je pense dans le frigo.

00:25:54
*Speaker 1:* Mouais.

00:25:56
*Speaker 2:* Euh, Les fruits qui pourrissent vite. Ben, tout ce qui est pommes, tout ça, pas besoin de frigo, mais plus (euh) tout ce qui est fraises ou les- les fruits qui peuvent pourrir un peu plus vite, mettre au frigo, aussi je pense.

00:26:09
*Speaker 1:* D'accord.

00:26:11
*Speaker 2:* Et après (euh) c'est tout. Je pense qu' après (euh) une grande partie des légumes qui aiment bien quand même, en dehors du frigo.

00:26:19
*Speaker 1:* Ouais. Et vous disiez que là, ils aiment pas quand il fait être un peu trop chaud--

00:26:24
*Speaker 2:* Ouais, on va les (euh) peut-être les bouger juste de place dans un endroit, mon soleil, où- où il y a moins de chaleur, peut-être.

00:26:35
*Speaker 1:* Ouais. Donc, dans l'idéal un lieu (euh)--

00:26:38
*Speaker 2:* Un lieu frais, ouais.

00:26:40
*Speaker 1:* mais pas forcément réfrigéré, quoi.

00:26:42
*Speaker 2:* C'est ça, puisque il fait pas trop chaud, pas trop froid (euh), je sais même pas ce qu'il faudrait comme température, une température normale, quoi.

00:26:57
*Speaker 1:* OK. Et donc les fruits sont à température ambiante dans votre cuisine.

00:27:04
*Speaker 2:* Ouais.

00:27:11
*Speaker 1:* Donc là, si des fois vous en mettez pas au frigo, c'est plus par contrainte--

00:27:14
*Speaker 2:* Ben, on n'a pas un grand frigo. Après, on n'a pas- on n'avait pas la- le besoin de le mettre au frigo non plus.

00:27:20
*Speaker 1:* D'accord. Parce que justement, c'était (euh)--

00:27:28
*Speaker 2:* Ben, c'est assez bon pour qu'il tiennent assez longtemps.

00:27:34
*Speaker 1:* OK. Ça marche. (Euh) en termes de nettoyage des différents lieux, est-ce que vous avez une organisation systématique, ou c'est un- ou c'est (euh) quand (euh)--

00:27:48
*Speaker 2:* Ben, pour les légumes on nettoie, c'est- c'est dans des cageots où on nettoie pas.

00:27:53
*Speaker 1:* Ouais.

00:27:54
*Speaker 2:* Euh, après que pour les fruits, souvent ils sont dans un- (euh) dans un petit- un petit bol, un grand bol. Dès que, il est vide, on le nettoie, mais après le frigo, on ne nettoie pas parce que on pense (euh)- il y a de la place. Donc (euh,) on nettoie pas.

00:28:11
*Speaker 1:* Donc, finalement il y a que la corbeille--

00:28:13
*Speaker 2:* à fruits--

00:28:20
*Speaker 1:* qui, quand elle est vide (euh)--

00:28:22
*Speaker 2:* C'est ça.

00:28:24
*Speaker 1:* OK. Ça marche. (Euh) Là, si on s'intéresse à vos- à vos pratiques de préparation et de consommation des fruits et légumes, alors, si on- si on prend (euh) un fruit que vous avez dit consommer très régulièrement, ou on va dire, régulièrement. Si on prend, il y avait- vous avez dit d'abord pomme et banane. Alors, prenons la pomme, comment vous consommez la pomme? Sous quelle forme vous allez la consommer?

00:29:01
*Speaker 2:* Euh, ben, je croque par- donc, je la mange directement.

00:29:05
*Speaker 1:* Ouais, vous la croquez.

00:29:05
*Speaker 2:* Je la croque. Ou sinon, je la coupe en- je la coupe, parce que quelques fois je peux pas croquer parce que c'est trop dur, donc (euh) je coupe.

00:29:12
*Speaker 1:* OK.

00:29:12
*Speaker 2:* En plusieurs morceaux.

00:29:19
*Speaker 1:* Et vous n' épluchez pas?

00:29:21
*Speaker 2:* Non.

00:29:25
*Speaker 1:* OK. Et au sein de votre foyer, est-ce qu'il y en a qui ont tendance à l'éplucher ou non?

00:29:30
*Speaker 2:* Non, personne ne- personne ne l'épluche.

00:29:37
*Speaker 1:* OK. Et la banane? Sous quelle forme vous la mangez?

00:29:40
*Speaker 2:* Ben, pareil. On- on enlève la peau, et on la mange.

00:29:49
*Speaker 1:* OK. D'accord. (Euh) Donc, les pommes, les bananes, c'est exclusivement crues, que vous allez la---?

00:29:59
*Speaker 2:* (Mmmm) Si bananes, quand- quand je fais ben, ça fait longtemps que je l'ai pas fait non plus, parce que on n'en a plus assez à pourrir. Mais quand il y en avait trop de pourries, je le faisais en- en milkshake.

00:30:09
*Speaker 1:* OK.

00:30:13
*Speaker 2:* Mais trop pourri, pas non plus trop trop pourri, mais bien abîmé, quoi.

00:30:17
*Speaker 2:* Ouais.

00:30:22
*Speaker 1:* OK. (Euh) Pour vous, c'est quoi une- une banane abîmée?

00:30:31
*Speaker 2:* Alors (euh), faut qu'elle soit- quand bien bien abimée, c'est que, elle est quasiment (euh) ben que, que... Ben, qu'elle est en grande partie noire, quoi.

00:30:49
*Speaker 1:* Ouais

00:30:55
*Speaker 2:* Faut que- faut qu'il y ait au moins la moitié qui ait bien- bien- bien le bac, commence à être noir, que ce soit bien abîmé, sinon ça se mange.

00:31:03
*Speaker 1:* Ouais.

00:31:06
*Speaker 1:* À la base, vous vous aimez plutôt la banane quand elle est encore un peu verte (euh)--

00:31:11
*Speaker 2:* Ouais, quand elle est bien verte.

00:31:12
*Speaker 1:* D'accord. Est-ce qu'il y a des différences là-dessus, dans le foyer ou pas, ou c'est tout le monde qui préfère à la base la--?

00:31:19
*Speaker 2:* Euh, tout le monde (euh), la prend verte.

00:31:21
*Speaker 2:* Ouais. [inaudible 00:31:22- 00:31:25] D'accord. Donc, tout le monde a tendance à l'aimer verte, plutôt que tachetée (euh)?--

00:31:36
*Speaker 2:* Ouais.

00:31:36
*Speaker 1:* OK. Est-ce qu'il y a parmi les fruits que vous aviez dit consommer, certains fruits que vous allez (euh)- vous aller cuisiner, donc il y a la banane quand elle commence à être bien amochée, est-ce qu'il y en a d'autres comme ça, des- des fruits que vous allez pouvoir cuisiner ou non? Non?

00:31:56
*Speaker 2:* Non.

00:31:56
*Speaker 1:* Non?

00:31:56
*Speaker 2:* Non, je crois pas.

00:31:58
*Speaker 1:* Ils sont tous crus sinon (euh)--?

00:32:00
*Speaker 2:* Je crois.

00:32:04
*Speaker 1:* Et au sein de votre foyer, pareil? Il y a pas grand monde- il y a personne qui va--?

00:32:07
*Speaker 2:* Mais je crois pas, je sais pas, je les ai pas vu en faire, donc je pense pas.

00:32:12
*Speaker 1:* OK. Alors, si on prend là, le- les légumes, (euh) si on prend le- les carottes. Sous quelle forme vous allez (euh) consommer les carottes?

00:32:34
*Speaker 2:* Alors (euh) quelques fois, je les épluche juste pour les manger crues, directement comme ça, (euh) on peut- on les coupe sinon en rondelles, on les fait (euh) cuire (euh) à la poêle.

00:32:50
*Speaker 1:* Ouais.

00:32:50
*Speaker 2:* Donc on- souvent on les met, quand on fait des soupes, ben, on met des carottes dans les soupes.

00:32:54
*Speaker 1:* Ouais.

00:32:58
*Speaker 2:* Euh, Je crois que j'ai déjà fait des carottes râpées aussi--

00:33:00
*Speaker 1:* D'accord.

00:33:05
*Speaker 2:* mais ça fait longtemps. Euh, je crois que c'est tout.

00:33:06
*Speaker 1:* OK.

00:33:06
*Speaker 2:* Ou on met dans les pâtes ou (euh), on fait cuire avec les pâtes, pour revenir à côté plus longtemps, en fait.

00:33:16
*Speaker 1:* OK. Ou soit (euh)- soit râpée, mais c'était plutôt rare, plutôt comme ça, ou comme on pourra le manger cru. Sauf que là vous l'épluchez, la carotte?

00:33:27
*Speaker 2:* Oui. Oui j'épluche quand je la mange. Ben, automatiquement, de toute façon carotte c'est cru. Que ce soit cuit ou cru, j'épluche.

00:33:34
*Speaker 1:* OK et sinon, c'est plutôt en poêlée (euh)--

00:33:36
*Speaker 1:* Oui.

00:33:42
*Speaker 1:* On coupe en petits bouts et poêlée (euh) en accompagnement ou en soupe. OK, si on prend le poivron, sous quelle forme vous consommez le poivron?

00:33:54
*Speaker 2:* Euh, Souvent je coupe en lamelles et je le rajoute dans des salades,

00:33:57
*Speaker 1:* D'accord.

00:34:01
*Speaker 2:* Euh, quelques fois, je le coupe même pas, je le mange directement--

00:34:04
*Speaker 2:* OK.

00:34:05
*Speaker 2:* et (euh) quelquefois, mais rarement, parce que j'aime pas trop quand c'est cuit, On pareil- on les coupe en petits carrés ou en lamelles et on les fait revenir à la poêle pour en faire un accompagnement ou autre chose.

00:34:18
*Speaker 1:* D'accord. Mais vous préférez [inaudible 00:34:21- 00:34:22]?

00:34:21
*Speaker 2:* Moi, je préfère cru.

00:34:26
*Speaker 1:* OK, donc on prend notre [inaudible 00:34:28- 00:34:29]. (Euh) La courgette, en général, vous la consommez comment la courgette, soit vous, qui vous- quelqu'un de votre foyer, qui--

00:34:43
*Speaker 2:* Ben là, eh ben, on fait (euh), soit on va- on coupe en petits- en petits bouts pour faire des, ben pareil on la fait poêler, on accompagne en d'autres choses. Soit en soupe. Là, du coup, je- on l'épluche pas quand on le fait en soupe.

00:34:59
*Speaker 1:* OK.

00:35:00
*Speaker 2:* Euh, Courgettes farcies, quelques fois--

00:35:04
*Speaker 1:* OK.

00:35:06
*Speaker 2:* et, c'est tout.

00:35:12
*Speaker 1:* Et du coup, la courgette, si vous la poêlez, vous l'épluchez?

00:35:16
*Speaker 2:* Oui, il y a que quand- que je l'ai- que quand moi- pour moi en tout cas, que quand je mets dans la soupe, que j'épluche pas.

00:35:21
*Speaker 1:* D'accord.

00:35:21
*Speaker 2:* Puisque de toute façon (euh), je vais tout mouliner donc (euh)--

00:35:26
*Speaker 1:* Et pourquoi quand c'est en poêlée, vous l'épluchez, est-ce que vous saurez dire, ou--?

00:35:30
*Speaker 1:* Euh, parce que c'est- c'est épais, c'est--

00:35:33
*Speaker 1:* Ouais.

00:35:36
*Speaker 2:* Je sais pas. Une habitude, je pense aussi.

00:35:44
*Speaker 1:* OK. Donc, la peau (euh) crue comme ça, enfin voilà, la peau (euh) ne paraît pas (euh) très agréable--

00:35:56
*Speaker 2:* J'ai tant vu mes parents éplucher les courgettes, ben, je fais pareil.

00:36:00
*Speaker 1:* C'est ça. Et les courgettes, si vous arrive de les manger crues ou pas du tout?

00:36:11
*Speaker 2:* Ah non.

00:36:28
*Speaker 1:* OK, alors ça marche, OK. (Euh) Alors, là je voudrais qu'on échange sur le moment où vous jetez un fruit ou un légume ou une partie seulement, (euh) quand ça arrive, pourquoi décidez-vous de le jeter, plutôt que de le consommer?

00:36:48
*Speaker 2:* Ben, parce que, il est pourri.

00:36:51
*Speaker 2:* Ouais.

00:36:51
*Speaker 2:* Et c'est la seule raison, quand c'est pourri, quoi. Pas mangeable.

00:36:58
*Speaker 1:* Ouais. Et comment vous déterminez quel fruit et légume est pourri et pas mangeable?

00:37:06
*Speaker 2:* À la couleur, tex- à la texture--

00:37:10
*Speaker 1:* Ouais.

00:37:15
*Speaker 2:* (euh) ouais, c'est - je sais même pas comment- (euh) ouais c'est ça couleur et texture.

00:37:16
*Speaker 1:* Ouais.

00:37:24
*Speaker 2:* Il y a pas d'autres (euh) façons de déterminer que c'est pourri.

00:37:24
*Speaker 1:* OK. Et quand vous êtes face à du pourri. Qu'est-ce que vous ressentez à ce moment là?

00:37:41
*Speaker 2:* Ben, ben, on va dire du- une sorte de dégoût à voir (euh) les parties pourries.

00:37:44
*Speaker 1:* Ouais...Et (euh) du coup, qu'est-ce que vous faites, il ya un fruit, vous voyez qu'il y a du pourri. Qu'est-ce que vous faites à ce moment là?

00:38:02
*Speaker 2:* Je coupe une large zone de ce qui est pourri.

00:38:03
*Speaker 1:* Ouais. OK.

00:38:03
*Speaker 2:* Puis, j'ai tout le temps peur que c'est allé plus loin, mais je sais que après les- les deux guerres, à la maison, ils coupent juste vraiment, ce qui est pourri.

00:38:14
*Speaker 1:* D'accord. Et pourquoi vous avez besoin de--?

00:38:18
*Speaker 2:* Parce que j'ai tout le temps pour qu'il y a du pourri qui est allé plus loin, et que ça gâche le goût du truc.

00:38:28
*Speaker 1:* Selon vous, le pourri il gâche le goût?

00:38:30
*Speaker 2:* Ben, je sais pas, je goûte- j'ai pas goûté le pourri, mais dans ma tête, oui.

00:38:36
*Speaker 1:* Ouais. Dans votre tête, ça a quel goût le pourri?

00:38:42
*Speaker 2:* Ben, un goût de pourri--

00:38:43
*Speaker 1:* (Rire) C'est ça.

00:38:43
*Speaker 2:* et c'est pas bon, quoi.

00:38:44
*Speaker 1:* OK. Et si vous coupez, c'est pour éviter d'avoir ce- ce goût pourri mais, sinon c'est, enfin voilà.

00:38:57
*Speaker 2:* Ben, je sais pas quel goût ça a.

00:39:09
*Speaker 1:* OK. Euh, vous avez pas de dégoût, ça vous dégoûte plus qu'autre chose de- d'avoir du dé- du- du pourri? Euh, OK, le goût (euh) vous savez pas trop (euh) tellement quel goût ça a? Alors, vous avez dit que si vous enlevez (euh), une large zone, c'est pour être certaine que, il n'y ait pas de- ce goût du pourri qui atteint à l'endroit que vous voulez manger. Est-ce que selon vous ce pourri, il a d'autres conséquences qu'au niveau du goût ou non, c'est vraiment le goût qui va vraiment être (euh)--?

00:39:44
*Speaker 2:* Ah non, je pense qu'il y a aussi (euh), ben, ce qu'il y a des gens, mais je ne sais même pas s'il y a- je pense qu'il y a d'autres trucs. Mais (euh), il y a des (euh) merde, des- je sais pas, des sortes de germes, de microbes ou je ne sais pas trop quoi. Ben des- ouais, des germes, je sais même pas si- si c'est le mot mais, ouais--

00:40:08
*Speaker 1:* Moi non plus, je connais pas-- et pour vous, c'est pas- c'est pas--

00:40:09
*Speaker 2:* C'est pas, comment dire, je sais pas comment expliquer... c'est pas propre, quoi.

00:40:09
*Speaker 1:* Ouais.

00:40:09
*Speaker 2:* C'est--

00:40:27
*Speaker 1:* Donc, du coup, c'est pas propre. Ça vous- c'est pour ça que vous ne souhaitez pas le manger?

00:40:34
*Speaker 2:* Ouais, pareil quand on sait jamais si c'est pourri, il y a des bêtes qui viennent. Alors, manger des petites bêtes, non merci non plus.

00:40:45
*Speaker 1:* Ouais. C'est ça qui fait qu'un fruit ou un légume devient pas mangeable pour vous, c'est (euh)--?

00:40:54
*Speaker 2:* Ouais.

00:40:56
*Speaker 1:* Quand son goût va être (euh) différent, voire- voire, il y a des bêtes. OK, et vous avez dit que, donc les deux gars qui sont (euh) chez vous (euh) sont- vont moins couper (euh) cet aspect là. Est-ce que vous savez (euh)? Est-ce que vous saurez dire, voilà que s'il y a des différences entre vous face au- face à ce pourri? Est-ce qu'il y en a chez vous qui vont le cuisiner quand même? Est-ce que non, vous êtes quand même tous à peu près pareils ou--?

00:41:35
*Speaker 2:* Moi, je pense que, ils vont plus- moi, je vais voir que c'est un petit peu pourri, je vais couper automatiquement, que je pense que les deux gars, ils voient que c'est un petit peu pourri, mais que c'est encore mangeable, donc ils vont cuisiner.

00:41:48
*Speaker 1:* D'accord.

00:41:49
*Speaker 2:* Donc, ils voient que c'est encore mangeable, ils font- ils cuisinent.

00:41:59
*Speaker 1:* OK. ça marche. Alors là, je voudrais qu'on parle du gaspillage alimentaire. Quand je vous dis gaspillage alimentaire, quels sont les mots qui vous viennent comme ça, spontanément (euh) en tête ?

00:42:22
*Speaker 2:* Ben, jeter, poubelle,

00:42:29
*Speaker 1:* Ouais.

00:42:29
*Speaker 2:* (euh) ben gaspillage,

00:42:29
*Speaker 1:* Mm-hmm.

00:42:38
*Speaker 2:* ben, cout.

00:42:43
*Speaker 1:* Ouais. Alors, du coup, est-ce que vous pouvez tenter de définir le gaspillage alimentaire, si vous vous écriviez une définition dans le Petit Robert? Qu'est-ce qu'on y verrai après (euh) gaspillage alimentaire?

00:43:03
*Speaker 2:* Euh, ce serait une action qui consiste à jeter des aliments qui n'ont pas été mangés ou consommés, sachant qu'ils étaient encore (euh) consommables ou mangeables.

00:43:22
*Speaker 1:* Donc, action qui consiste à jeter des aliments qui n'ont pas été mangés, consommés, sachant qu'ils étaient-- OK, ça marche et du coup là, vous dites (euh), donc c'est le fait de jeter des aliments qui ont pas été mangés, consommés, en sachant qu'ils étaient encore consommables pour vous, quand un- quand vous tombez sur un fruit ou un légume qui est complètement pourri, c'est moins du gaspillage que si on le jetait alors qu'il n'était pas pourri. Vous, est-ce ce que vous faites une différence (euh) là dessus ou pas?

00:44:09
*Speaker 2:* Après c'est vrai, quand- quand je jette les fruits et légumes, comme il y a le compost, je trouve que c'est moins jeter. Puisque ça servira quand même (euh), il y a le compost donc, ça va faire du bon compost, du coup, ça fait- il y a moins la sensation de jeter déjà.

00:44:20
*Speaker 2:* Ouais.

00:44:22
*Speaker 2:* Mais sinon, je sais pas. Si quand c'est bien périmé, c'est pas mangeable, OK, c'est pas du gaspillage alimentaire, mais quand c'est un petit peu périmé, mais que c'est mangeable, c'est du gaspillage, quand on mange pas, voilà, c'est du gaspillage alimentaire.

00:44:46
*Speaker 1:* Mais pour vous, il y a quand même une différence dans le- dans le fait de jeter un fruit ou légume ou autre chose qui n'est pas encore (euh), enfin, qui serait encore mangeable ou quand c'est vraiment mangeable et que là on pas d'autre choix que de les-- OK. Et du coup, ben là, vous m'en avez déjà un peu parlé, mais qu'est-ce que ce que vous ressentez quand vous, ça vous arrive de jeter (euh)- de jeter un aliment fruit ou légume à la poubelle?

00:45:18
*Speaker 2:* Ben, déjà, c'est très rare qu'on jette.

00:45:20
*Speaker 1:* D'accord.

00:45:22
*Speaker 2:* Euh, c'est assez rare. Après là pour, tout ce qui est du coup, pour fruits et légumes, j'ai moins la sen- sensation de jeter parce qu'on a le compost.

00:45:28
*Speaker 1:* Ouais.

00:45:28
*Speaker 2:* Euh, si (euh) par exemple, c'est un plat que, on n'avait pas fini tout ça. Et ben, on a un chat à la maison qui mange tout. Donc (euh), on lui donne, elle le mangera, elle sera heureuse. Donc pareil, on n'a pas trop l'impression de jeter. Mais globalement, oui, on jette quasiment rien, on a du monde, donc (euh)--

00:45:51
*Speaker 1:* OK. Donc, vous jetez pas beaucoup, il y a le compost ou le chat. Donc, au final (euh), ça vous- enfin voilà, vous n'avez pas de moment là, où vous vous dites, je viens de jeter un truc à la poubelle--

00:46:07
*Speaker 1:* Non.

00:46:07
*Speaker 1:* un aliment à la poubelle?

00:46:09
*Speaker 2:* Non, non c'est vraiment très rare qu'on jette des aliments à la poubelle.

00:46:12
*Speaker 1:* Et quand ça arrive, qu' est-ce que vous ressentez à ce moment là, si ça arrive?

00:46:16
*Speaker 2:* Ben, que j'aurais dû le manger plus tôt.

00:46:18
*Speaker 1:* Ouais.

00:46:20
*Speaker 2:* Que c'est de l'argent jeté.

00:46:23
*Speaker 1:* Ouais. D'accord. Donc, pour vous, il y a une notion d'avoir jeté de l'argent, en jetant (euh)--

00:46:39
*Speaker 2:* Oui, ben oui c'est ce que, un truc tout bête, mais qu'on nous dit tout le temps qu'on est petit, oui, pense aux petits africains qui ont pas l'argent pour s'acheter à manger, et là tu vois- quand on se voit jeter de l'ar- de la nourriture , ben c'est l'argent quoi.

00:47:01
*Speaker 1:* Et du coup, vous avez dit j'aurais dû manger plus tôt?

00:47:03
*Speaker 1:* Oui.

00:47:04
*Speaker 1:* Qu'est-ce ce que vous entendez, du coup, par là?

00:47:05
*Speaker 2:* Ben, que d'attendre ben, de perdre- Ben, si je l'ai jeté c'est que c'était vraiment trop pourri. (Euh) mais si c'était autre que des légumes et fruits, par ce que là, on aurait mis dans le compost, mais autres que légumes et fruits, ça veut dire que j'ai trop attendu, que c'était trop pourri. Donc j'aurais dû manger un peu plus tôt quand ce n'était pas encore à ce point là.

00:47:23
*Speaker 1:* OK. Donc selon vous, il y a une part de- enfin, c'est (euh) l'organisation mais c'est qu'il y a eu un moment donné un défaut dans le--

00:47:37
*Speaker 2:* Oui, que j'ai- j'ai soit je l'ai caché au fond du frigo, je l'ai pas vu, soit que- que j'ai pas pensé à le manger ou je sais pas, donc (euh) il y a tout le temps des raisons d'oublier manger un truc qui est dans le frigo, ou j'aurais dû mettre au congélateur, et comme ça j'aurai pû le manger plus tard.

00:47:57
*Speaker 1:* Ouais. vous pratiquez un peu la congélation ou pas (euh)?

00:48:01
*Speaker 2:* Eh ben, souvent, quand on finit pas les plats, si on voit que, il y en avait beaucoup trop à manger, on met au congel.

00:48:07
*Speaker 1:* D'accord. Quand il y a des restes de marmites, quoi (euh)?

00:48:15
*Speaker 2:* C'est ça (euh) comme il reste beaucoup de restes, il y a pleins de petits Tupperware, pour mettre au congélateur. On fait des petites portions.

00:48:28
*Speaker 1:* OK. ça marche. Alors, pour finir juste sur la poubelle connectée que vous avez depuis quelques semaines maintenant, comment ça se passe avec cette poubelle?

00:48:38
*Speaker 2:* Ben, très bien.

00:48:39
*Speaker 1:* La cohabitation se passe bien?

00:48:41
*Speaker 2:* Elle se passe très, ben, très bien. On n'a quasiment rien jeté dedans.

00:48:47
*Speaker 1:* D'accord.

00:48:47
*Speaker 2:* [inaudible 00:48:47- 00:48:48] cette poubelle. OK, c'est bien pourri à l'intérieur, Parce qu'on jette quasiment rien, vraiment mais (euh)--

00:48:54
*Speaker 1:* Ouais.

00:48:54
*Speaker 2:* Mais non. Elle a- elle se porte bien.

00:48:57
*Speaker 1:* OK.

00:48:57
*Speaker 2:* Elle est en vie. C'est bon.

00:49:00
*Speaker 1:* Elle a pas pris feu?

00:49:00
*Speaker 2:* Non, elle n'a pas fait de caprice.

00:49:02
*Speaker 2:* OK.

00:49:03
*Speaker 1:* Adorable.

00:49:06
*Speaker 1:* Cinq sur cinq. OK. Euh, est-ce que ça vous est arrivé de vous poser des questions en mode est-ce que ça doit aller dedans, ou pas ou--?

00:49:17
*Speaker 2:* Alors, pour moi, non. OK, mais quand j'ai expliqué aux gars--

00:49:21
*Speaker 1:* Ouais,

00:49:22
*Speaker 2:* C'était pas tout de suite compris, mais ils comprenaient pas, par exemple, qu' on jette pas- si on mangeait la banane, pourquoi on jetait pas la peau de la banane? En fait, ils pensaient que c'était pour mettre vraiment tous les déchets.

00:49:33
*Speaker 1:* OK. Comme un compost--

00:49:34
*Speaker 2:* Comme un compost normal. Du coup, il a fallu leur expliquer un peu au début- après c'était bon. On a- on met nos deux poubelles de compost pour préparer le repas, et on mettait ce qu'il fallait dans les bonnes poubelles.

00:49:45
*Speaker 1:* Parfait. Euh, ça vous est pas arrivé, du coup, de l'oublier des fois? Ce qui peut arriver (euh) d'un moment donné faire à manger, puis mettre tout dans le compost, et s' être dit, mince, ça- ça aurait peut être pu (euh)--?

00:49:57
*Speaker 2:* Moi, non, mais peut-être que quelques fois ça a dû être oublié, parce que, ça- ca arrive.

00:50:00
*Speaker 1:* Ben oui, c'est pour ça.

00:50:04
*Speaker 2:* Peut être, mais moi non. Je cuisine pas beaucoup, donc il ya pas de risque d'oublier.

00:50:08
*Speaker 1:* OK. ça marche. Du coup, vous êtes à 10- à 15 sacs pour l'instant?.

00:50:17
*Speaker 1:* Oui. Ben, un sac, un en fond de sac.

00:50:23
*Speaker 1:* Un fond de sac. OK. Est-ce que vous avez le souvenir ou pas des choses qui ont été mises dedans? Pas du tout, quelques exemples?

00:50:31
*Speaker 2:* Alors on a quelques fraises,

00:50:33
*Speaker 1:* D'accord.

00:50:35
*Speaker 2:* quelques bouts de tomates, des bouts de poivrons, des bouts de carottes, et au tout début je sais pas.

00:50:45
*Speaker 2:* OK. Et il y a eu donc- les fraises, vous avez dit qu'elles étaient entières et très (euh)--

00:50:51
*Speaker 2:* Oui. les fraises, c'était qu'elles étaient vraiment- commençaient à être toutes blanches.

00:50:58
*Speaker 1:* Ouais, pas faux. Et (euh) tomates, poivrons, carottes, c'était que des bouts? Il y a eu des fois des--?

00:51:02
*Speaker 2:* C'était des- c'était des petits bouts à chaque fois.

00:51:03
*Speaker 1:* D'accord. OK. Est-ce que vous avez le sentiment des- ou pas de- qu'il y a des fruits ou légumes que vous allez (euh) jeter plus que d'autres, ou que voilà, qui peut-être se conserveraient peut être moins bien? Est-ce que vous avez, je sais pas, pensé à- en disant ben ça j'ai eu l'impression de le jeter plus que- que autre chose ou--?

00:51:23
*Speaker 2:* Euh... Non.

00:51:23
*Speaker 1:* Non?

00:51:23
*Speaker 2:* Je crois pas.

00:51:23
*Speaker 1:* Ouais, i l n'y a pas comme ça qu'il y a quelque chose d'évident qui vous vient?

00:51:43
*Speaker 1:* Non, il y a pas quelque chose d'évident.

00:51:47
*Speaker 1:* OK. Ça marche et ben merci, j'ai posé toutes les questions. Est-ce que, il y a des choses sur lesquelles vous aimeriez revenir? Je ne sais pas s'il y a des points que vous pensez que j'ai oublié sur la conso de fruits et légumes ou--?

00:52:04
*Speaker 2:* Non. ça m'a l'air tout bon.

00:52:13
*Speaker 2:* On a fait un bon tour. OK. Eh ben, ça marche. Ben, merci beaucoup. OK. Alors, j'ai deux trois petites choses à vous donner et à faire avant de- de repartir, je vais vous montrer, [inaudible 00:52:32- 00:52:34]

**Household F10, Interview 2**

*Mme 1:* N'y a pas de mauvaise réponse à ce que [euh]- Oui- vous me dit. Alors, Julie- On va voir quand même. On va devoir voir ça. Alors, il y a quatre illustrations, tu le permets ? Oui. Et, je vais vous demander d'en choisir une et de me direz spontanément ce qui vous vient à l'esprit. OK- donc, vous la retournez.

*Mme 2:* Oui, [euh] En tous cas où je vais pas, parce que ça coûte trop cher.

*Mme 1:* D'accord ?

*Mme 2:* [Euh] souvent c'est des- des légumes locaux.

*Mme 1:* Ouais. Donc, c'est un lieu dans lequel vous allez pas [euh]-

*Mme 2:* Non, non. Surtout au niveau du prix. De cette façon, c'est plus cher qu'en magasin.

*Mme 1:* Ouais. Et sinon, vous pensez quoi [euh] de ce lieu quand même s'il était plutôt accessible en termes de prix ?

*Mme 2:* [Ben] aucune idée. [Euh] si, ça pourrait être intéressant d'y aller [euh] parce que c'est moins [euh]- au niveau des prix, si c'était moins cher ce serait intéressant parce que c'est plus local. C'est tu vois-

*Mme 1:* Ouais.

*Mme 2:* Donc, c'est [euh] souvent, il n'y a pas mal de bio aussi donc [euh] c'est- ça peut être potentiellement des [euh] légumes [euh] mieux que ce qu'on achète.

*Mme 1:* Ouais. C'est un magasin dans lequel vous pourriez aller si les prix étaient [euh]- ouais- accessibles ? Oui. Oui. Mais [euh]-

*Mme 2:* [Ben] après [euh] je pas le temps que ça de légumes, comme on a le potager mais-

*Mme 1:* D'accord. Ok, les légumes ne vous parlent pas tant que ça ? Non. Ok, alors si on prend un [euh] une autre illustration ?

*Mme 2:* OK, pour le marché.

*Mme 1:* Le marché, qu'est-ce que ça vous évoque ?

*Mme 2:* Le lieu où je vais jamais.

*Mme 1:* Ouais.

*Mme 2:* [Euh] Ça m'évoque la même chose en fait.

*Mme 1:* Oui.

*Mme 2:* Sauf que là, ça me- [euh] marcher ou c'est jour le samedi. Et là, c'est [euh] un tour de marché qui à tous les jours en mar- dans un tour de magasins marché quoi. C'est un peu pareil.

*Mme 1:* Ouais, le Primeur c'est un- c'est un marché [euh] en magasin quoi ?

*Mme 2:* Oui, c'est ça. Donc, [euh] soit c'est la même chose marché et Primeur pour moi.

*Mme 1:* Ouais. Et [euh] pourquoi vous allez pas au marché ? Pour les mêmes raisons, ou-

*Mme 2:* Pas le temps. Pas le courage.

*Mme 1:* Oui.

*Mme 2:* [Ben] le prix aussi qui est souvent plus cher. Après souvent les légumes et les fruits tout ça, c'est de meilleure qualité aussi.

*Mme 1:* D'accord. Et [euh] est-ce que vous avez déjà été au- de- marché ? Oui. Est-ce que c'est un lieu dans lequel [euh] vous vous sentez bien ou non ? C'est pas [euh] une contrainte d'y aller ?

*Mme 2:* [Ben] Après quand j'y vais, c'est pas pour acheter, je- juste [euh] regarder ce qu'ils vendent au marché, mais j'achète jamais et- et j'aime pas trop [euh]. Dans les supermarchés, on- on choisit vraiment comme on veut [euh] tout ce que [euh]- [ben] On se sert nous-même qu'au marché, c'est eux qui servent pour nous. Du coup [euh] on ne peut pas- moins de choix peut-être.

*Mme 1:* Ouai. Est-ce que c'est un milieu dans lequel vous vous sentez bien, le marché ou non ?

*Mme 2:* Non, pas du tout. Il y a trop de monde.

*Mme 1:* D'accord. Si vous y êtes, c'est pas un moment agréable ?

*Mme 2:* Pas forcément.

*Mme 1:* Est-ce que vous avez quand même déjà consommé des produits qui venaient du marché ou pas, des fruits et légumes ?

*Mme 2:* Oui, oui, il y a fort longtemps. Oui.

*Mme 1:* Oui ? Et qu'est-ce que vous pensiez de ces produits ?

*Mme 2:* OK, je ne sais plus du tout. C'était [euh]- c'était quand j'étais petite, je ne sais plus.

*Mme 1:* Ouai. Pas de souvenir. Non. Pas de fouiller les produits du marché quoi ?

*Mme 2:* Non, ils ne m'ont pas marqué.

*Mme 1:* Ouais, voilà. Ok, alors, c'est un problème de photo, maintenant ?

*Mme 2:* Ah ! Potager, c'est mieux.

*Mme 1:* Potager, c'est mieux ? Alors, qu'est-ce que ça vous évoque ?

*Mme 2:* Et [ben] ça m'évoque mon jardin.

*Mme 1:* Ouais.

*Mme 2:* C'est des [euh]- [ben] on fait pousser ce qu'on veut [euh]. Pas cher du tout. Oui- On plante ce qu'on veut, on a ce qu'on veut quand on veut. Il ne faut pas se tromper dans les dates, mais-

*Mme 1:* Ouai. Vous avez un potager ?

*Mme 2:* Oui.

*Mme 1:* Et vous produisez quoi ?

*Mme 2:* Alors là, on n'a plus grand-chose mais, on a fait carotte, patates, choux, petits pois, radis, [euh] salade, roquette, mâche, oignons et [euh] betterave

*Mme 1:* OK.

*Mme 2:* Là, c'est tout.

*Mme 1:* Ok, c'est pas mal. Et du coup, ces légumes-là, vous les[euh]- vous n'avez pas besoin de les acheter ailleurs ?

*Mme 2:* C'est ça. Vous- [Ben] les patates, si ce qu'on en n'a pas mis assez. Mais [euh]- ouai- Le reste nous [euh] il nous en reste encore [euh]- Les carottes- on a encore un cageot entier de carottes de-

*Mme 1:* Ok. C'est pas autosuffisants quand même pour- [euh] Oui- Pour votre consommation ? Et [euh]- Qu'est-ce qui fait que vous avez un potager ? Pourquoi vous avez choisi de faire un potager chez vous ?

*Mme 2:* [Ben] déjà c'est que ça [euh]- ça coûte moins cher ?

*Mme 1:* Oui.

*Mme 2:* Et [euh] c'est toutefois agréable de- de- de- de manger ce qu'on a fait pousser nous-même. Ça fait plus plaisir.

*Mme 1:* Ouai. Est-ce que vous arrivez à développer pourquoi ?

*Mme 2:* [Euh] Parce que, on passe du temps à les faire pousser. On est [euh]- On voit notre petite graine au départ et on voit le légume à la fin. Et ça forme ne pousse pas du tout légume [euh] [ben]. Ça m'aide à manger des légumes, de me dire que c'est moi qui les ai fait pousser.

*Mme 1:* Oui.

*Mme 2:* Après, j'ai l'impression aussi que ça se conserve mieux. Et là, les carottes, ça fait un mois qu'elles sont dans- dans des cageots. Elles sont tout le temps pas pourri, alors que celles du magasin deux jours, elles sont noires. Ok- Et quand même [euh] on les conserve exactement au même endroit, donc-

*Mme 1:* Oui. Vous avez remarqué une réelle différence entre [euh] -

*Mme 2:* Ouai. Surtout sur les carottes. Les carottes [euh] on a- on a essayé plein de [euh] façons [euh] en les achetant en magasin, en mettant dans un cageot, au frigo, au frais. A chaque fois, au bout de deux, trois jours, elles sont noires et alors que les nôtres, elles sont tout le temps bien.

*Mme 1:* Oui. Et est-ce que, [euh] en termes de [euh] de- Donc [euh] la conservation est meilleure ? Ouai- Est-ce que vous trouvez d'autres qualités ou d'aut- des défauts, au contraire des produits que vous pouvez produire vous-même ?

*Mme 2:* [Ben] Ils sont moins jolis. D'accord- Après c'est pas [euh] ils sont un peu plus difformes quoi. Mais [ben] après [euh] on y mange quand-même [hein]

*Mme 1:* Oui.

*Mme 2:* [Euh] Après à défaut [ben] il faut- Non, il y a pas d'autres défauts.

*Mme 1:* Oui.

*Mme 2:* Que d'autres qualités ou pas. [Ben] on sait ce qu'on [euh]- on sait que- ce que- ce qu'il y a eu sur les- les- sur les légumes aussi- On sait qu'il n'y a pas eu de pesticide ou [euh]- [euh] On- on sait ce qu'il y a dans la terre- voir tout ce qu'il y a dans la terre. On sait comment [euh] on les a cultivés.

*Mme 1:* Oui. Et [euh] en termes de goût il n'y a pas forcément de [euh] différences selon vous ou c'est la même ?

*Mme 2:* Pour les radis, si, mais les autres, pas du tout.

*Mme 1:* Et donc les radis, qu'est-ce qu'ils avaient de-

*Mme 2:* Ils étaient beaucoup plus piquants.

*Mme 1:* D'accord. Et du coup, le potager, est-ce que c'est un endroit dans lequel vous pouvez- enfin vous vous sentez bien ou non ? Vous seriez contraint de le- de le faire comment-

*Mme 2:* Ça dépend. Ouais- Là, on est en train de repréparer le potager pour [euh]- pour le printemps. Et clairement, vu le temps, on a un peu [euh]- pas le courage de le faire. Et c'est- Bon en tout cas il y a un peu une contrainte. Mais après, [euh] quand en été, c'est [euh]- au printemps et l'été c'est agréable de s'en occupés, ça pousse à nous fait sortir donc [euh] ça nous repose un peu.

*Mme 1:* D'accord. Est-ce que ça vous arrive quand même du coup de- [euh] devoir jeter des fruits et légumes que vous aviez vous-même produits ? Ouai- Et comment vous vous sentez quand vous [euh] jetez quelque chose que vous avez produit ?

*Mme 2:* En soi, c'est moi- moins [euh] gênant. Parce que, on ne les a pas achetés. Ouai-Donc, [euh] on se dit pas qu'on jette de l'argent. Oui- Après souvent [euh] quand on jetait, c'est parce qu'on en avait [euh] pas exemple pour les fraises, on en avait tellement que, [euh] on n'avait pas le temps de toutes les manger. Ouais- Et même si on en donnait partout, [ben] au bout d'un moment [euh] elles- elles pourrissaient et [euh] on avait beaucoup trop pour nous. Donc -

*Mme 1:* D'accord. Donc, ça vous embête moins finalement de-

*Mme 2:* Oui, [ben] en tout cas, moi, ça m'embêtait moins.

*Mme 1:* Ok. Donc, il y avait les fraises, est-ce que vous avez le souvenir d'avoir jeté autre chose ?

*Mme 2:* [Euh] Alors les patates, on a tout mangé. [Euh] en soi, on a acheté des trucs, mais c'est pas vraiment jeter. C'est [euh] ce que ça poussait pas bien. Du coup, [euh] comme ça poussait [euh] c'était tout petit. Genre les radis [euh] ils étaient pas- ils étaient pas une forme de radis, ils étaient vraiment tout raplapla, ils avaient pas poussé. Du coup, on a jeté. C'étaient [euh]- C'étaient pas mangeable. Ok- Après certaines une betterave pareil qu'on pas pris, que du coup, on a jeté mais sinon, on a [euh] à part [euh] les fraises qui pourrissait. On n'a rien eu à pourrir. Donc, on a tout mangé.

*Mme 1:* Ok, une fois à la récolte, c'était déjà pas bien ?

*Mme 2:* Ouai, c'est ça ?

*Mme 1:* Ou c'était- c'était juste les fraises-là qui- Ouai. Effectivement, les fraises [euh] ça se passait, même si [euh]-

*Mme 2:* Ça se conserve pas très longtemps. Du coup, là, on en avait quand même [euh] au moins un kilo par jour- [euh] quasiment un kilo par jour. Ah ouais ! Ouais, on n'en pouvait plus. On avait- on a fait de la confiture, des trucs, mais [puff]- Au bout d'un moment-

*Mme 1:* Ouais. C'est [euh]- C'était trop- Oui- Trop de fraise ?

*Mme 2:* [Inaudible] l'année prochaine.

*Mme 1:* Ouais, d'accord. OK, dans la dernière photo ?

*Mme 2:* La dernière, je suppose que c'est un magasin.

*Mme 1:* Tu la dis.

*Mme 2:* Yes ! [Ben]

*Mme 1:* Est-ce que vous pouvez me dire ce que ça vous évoque ?

*Mme 2:* [Euh] [ben] j'achète tout ce que [euh]- on n'a pas dans le potager. D'accord- Ou les trucs qui ne sont pas forcément de saison aussi.

*Mme 1:* Ouai.

*Mme 2:* En fait qui ne sont pas produits [euh] pas productible ici. Et surtout les fruits en fait. Les légumes moins. Les fruits, on en achète beaucoup.

*Mme 1:* D'accord. Et qu'est-ce que vous pensez de ce type de magasin ?

*Mme 2:* Bon [euh] rien.

*Mme 1:* C'est un endroit ou vous vous sentez plutôt bien ? Oui, oui- C'est une contrainte.

*Mme 2:* Non, non. Je- voilà non, j'aime bien aller faire les courses. Donc-

*Mme 1:* Oui.

*Mme 2:* Puisque, on sait qu'il y a tout le temps. On va dire à peu près la même chose [euh] en rayon dans les fruits et légumes, donc on sait qu'on va trouver ce qu'on veut, [euh] même si ce n'est pas la saison ou n'importe quoi. On a- on sait ou c'est. Oui- C'est pas [euh]- On arrive sur le marché et au final, ah [ben] non, il n'y a pas ça, c'est pas de saison [euh]-

*Mme 1:* Oui.

*Mme 2:* On est sûrs d'avoir tout ce qu'on veut. Après, c'est [euh] pas for- Ça, ça vient pas forcément de France, quoi. Ça vient de loin.

*Mme 1:* Oui.

*Mme 2:* [Ben] les prix sont moins chers aussi.

*Mme 1:* Ouais.

*Mme 2:* Et, ça se conserve très mal.

*Mme 1:* D'accord. Moins cher mais, [euh] ça se conserve mal ?

*Mme 2:* C'est ça. Rapport qualité prix, il n'est pas haut quoi.

*Mme 1:* Oui. Mais du coup, quand vous êtes amené à jeter un- [euh] un fruit, [euh] ou un légume qui vient du supermarché, qu'est-ce que vous ressentez ?

*Mme 2:* Voilà, c'est plus embêtant, parce que, voilà [ben] on a dépensé des sous pour l'acheter. Donc, [euh] on jette des sous.

*Mme 1:* Oui.

*Mme 2:* Du coup, on essaye de les [rire] consommer [euh] au maximum [euh] d'enlever et de voir si c'est juste une partie qui est pourrie ou [euh] souvent on a les trucs qui sont pourri complètement. Mais, [euh] ça fait plus mal de jeter ça.

*Mme 1:* Ouais. [Silence 00:13:54- 00:14:07] Et donc, vous arrivez à [euh] enfin votre- votre consommation de fruits et légumes [euh] c'est- [euh] entre le potager et les [euh] grandes surfaces ?

*Mme 2:* C'est ça.

*Mme 1:* Et donc, [euh] voilà, vous allez sentir une différence dans vos moments de jeter. Mais, c'est- parce que là, il n'y a pas eu de [euh] sous dépenser ?

*Mme 2:* Oui. C'est ça. Par rapport à l'argent.

*Mme 1:* Par rapport à l'argent dépensé. [Silence 00:14:30- 00:14:37] Ok. Alors, est-ce que vous consommez de la banane ou pas ? Oui- Ouais, on va faire un autre petit jeu. Alors là, il y a dix photos de banane et je vais vous demander de la ranger de celle qui vous donne le plus envie à celle qui vous donne le moins envie.

*Mme 2:* Ok, [Silence 00:15:05- 00:15:22] [hm-hm]

*Mme 1:* On va ranger, c'est mieux. [Silence 00:15:24- 00:15:41] Je vais prendre la photo.

*Mme 2:* Normalement, c'est dans l'ordre.

*Mme 1:* Du coup, est-ce que j'observe, ça a l'air d'être dans l'ordre de [euh] de maturation ?

*Mme 2:* Ouai. On- on les mange très verte, nous les bananes.

*Mme 1:* Ok. Alors est-ce que vous pouvez me raconter votre choix-là ?

*Mme 2:* [Euh] [ben] De base [euh] on mange les- [euh] les bananes très- très vertes. On les achète [euh] exprès, verte parce que [euh] après elles sont trop molles. On les aime bien quand elles ne sont pas trop molles. Après arriver à peu près comme ça-

*Mme 1:* C'est le roux blanc là ?

*Mme 2:* Ouais, on les mange plus. Je les fais en milkshake.

*Mme 1:* Ok, donc à partir du roux blanc- ouais- milkshake.

*Mme 2:* Et à partir du roux jaune, là [euh] je ne vois même pas, ça me fait me dégoûter.

*Mme 1:* D'accord. Donc, et c'est pareil pour tout le- pour tout le monde au sein de la ville ?

*Mme 2:* Oui.

*Mme 1:* Je le trouve souvent votre coloc.

*Mme 2:* Moi, il y a plus de coloc. Mais ouais.

*Mme 1:* Il y a plus de coloc, mais c'était pareil ?

*Mme 2:* Ouai.

*Mme 1:* Donc, vous les manger très bien. Ouais- À partir de là, en milkshake. Ouais- Le carré bleu, en milkshake. En effet- ça passe aussi ?

*Mme 2:* [Ben] ça dé- après, je coupe les morceaux qui sont vraiment- s'il y en a des trop- trop marrons [euh] je les coupe et je les mets pas. D'accord- Faut pas non plus que ce soit trop.

*Mme 1:* Oui, pour la peller.

*Mme 2:* Là, je- si c'est tout noir, j'ouvre même pas, c'est direct compost.

*Mme 1:* Ok, direct il faut composter. Oui- Et là, carré [euh] orange, vous pouvez quand même la manger [euh] crue ?

*Mme 2:* [Euh] [ben] je regarde en fait. Quand j'ouvre, si je vois que [euh]- que c'est trop abîmé [euh] je ferai en milkshake. Mais sinon oui [euh] comme ça, je peux manger.

*Mme 1:* Oui. Et qu'est-ce qui fait que vous les aimez [euh] donc [euh] verte ? Mais, dit [euh]- la dureté- c'est la dureté ? Surtout- C'est la texture qui va-

*Mme 2:* Oui, la texture.

*Mme 1:* C'est tout ? C'est vraiment une question de texture ? Ou il y a-

*Mme 2:* Oui, oui c'est que ça. Je suis raciste des bananes.

*Mme 1:* Tu deviens narcisses ?

*Mme 2:* Ouai.

*Mme 1:* Ok, et [euh] est-ce que, selon vous, il y a des moments ou la banane, elle- [euh] elle est potentiellement dangereuse pour la santé ou pas ?

*Mme 2:* [Euh] si elle a- s'il y a des mouches et des asticots dedans, oui. Mais-

*Mme 1:* Ouais.

*Mme 2:* Si non- non, je ne pense pas.

*Mme 1:* C'est vrai, c'est plus en termes de goûts que vous allez préférer la jetée que [euh] ouai- par idée de danger [euh] potentiels ?

*Mme 2:* Je vous assure que c'est dangereux, une banane.

*Mme 1:* Oui.

*Mme 2:* Oh, c'est rien. [Silence 00:18:33- 00:18:43]

*Mme 1:* Et, sur ces apports nutritifs. Est-ce que selon vous, il y a un moment donné ou [euh] les perds ou les a pas encore vous- Donc, on sait que la banane reste une banane.

*Mme 2:* Une banane reste une bonne banane.

*Mme 1:* Une banane reste une banane ?

*Mme 2:* Je n'ai aucune idée.

*Mme 1:* Ouais- donc c'est pas- c'est pareil. C'est pas par volonté de se dire, là, la banane, elle va m'apporter plus ou moins en- oui- C'est vraiment par goût et surtout texture ?

*Mme 2:* C'est ça ?

*Mme 1:* OK. Ok, pour la banane. Alors. L'autre petit jeu. Alors, je vais séparer ça. Ok, alors là, il y a [euh] trois types de "poubelles"- ouai- est-ce que [euh] vous avez des poules ?

*Mme 2:* Non, mais on envisage d'en mettre.

*Mme 1:* D'accord. Pour l'instant, il y a- il y a pas- vous n'avez pas d'animaux qui vont manger votre légume ?

*Mme 2:* Non, [ben] il y a mon chat.

*Mme 1:* Il mange des fruits et légumes frais ou pas ?

*Mme 2:* [Ben] quelquefois si ça cuit. Donc, s'il y a une sauce de viande de poulet dessus, oui.

*Mme 1:* Ok. Donc, ça- on peut le laisser alors ? Donc, il y a le compost ?

*Mme 2:* Oui et la poubelle.

*Mme 1:* Et, [euh] est-ce que ça vous arrive de jeter des fruits et légumes dans la poubelle en dehors du compost ? Non- Alors là, je vous montre. Il y a plusieurs en gros préparation qu'on peut faire avec des fruits et légumes. Et, je vais vous demander de garder celle que [euh] qui vous parle, que- que- que vous pouvez faire ? Ok- et d'enlever celles qui ne ressemble pas ce que vous pouvez faire ?

*Mme 2:* Ça, la banane oui. Après la fraise il y a la banane.

*Mme 1:* Ouais. Oui, voilà, c'est ça. Oui, voilà, c'est pas forcément le bon fruits ou légumes.

*Mme 2:* [Inaudible] l'autre jour, [hum] ça, je sais pas ce que c'est. [inaudible] on le fait de temps en temps. Il y a fruit et légume, oui, ça non, ça non, ça non, ça non.

*Mme 1:* Ok, ça va comme ça. Vous congelez pas vos fruits et légumes ?

*Mme 2:* Globalement, non.

*Mme 1:* Ne faites pas de gaspacho ? Non- vous mangez pas de crudités ? Ah si ! Ouais, de temps en temps aussi, mais- Crudités et salade de fruits ? [Euh] non- non. Hop, et maintenant, il y a des fruits et légumes que des personnes que [euh] des personnes ont jeté. Ok- j'aimerais vous demander de me dire, si vous, vous les auriez jetés aussi ? Ok- si oui, où ? Et, sinon [euh] qu'est-ce que vous auriez pu en faire ?

*Mme 2:* Ok, comme ça, compost.

*Mme 1:* Là, la cerise part au compost ? Ouais- ouais.

*Mme 2:* [Euh] ça, je garde parce que je sais couper le [euh] bout du radile.

*Mme 1:* OK, ça, vous mangez ? Et du coup, vous le ferez quoi, peut-être en manger cru ?

*Mme 2:* Ah oui ! en cru. OK- la tomate, elle est très bien là.

*Mme 1:* Ouais.

*Mme 2:* [Ben] soit crue, soit cuite.

*Mme 1:* Ouais.

*Mme 2:* Les carottes, il y a juste à les- [euh] les éplucher et c'est bon. Oui. Par là. Ou en soupe, non c'est bon quand c'est en soupe donc, c'est par là.

*Mme 1:* Quand elles sont pas belles comme ça ?

*Mme 2:* Quand elles commencent à être moins belles, moi, je le fais en soupe souvent. OK. Ça, je leur mis au compost.

*Mme 1:* D'accord, le poivron pour vous, qu'est-ce qu'il fait qu'il va au compost, ce poivron ?

*Mme 2:* [Ben] il est [euh] tout flasque. Du coup, il est plus [euh] croquant comme il l'est au départ.

*Mme 1:* D'accord.

*Mme 2:* [Euh] citron, j'en mange pas. Et en plus, dès qu'il y en a au frigo qui est un peu pourri, je le mets au compost. D'accord- j'aime pas du tout ça. La salade, elles sont un peu bien. Ouais, en salade. OK- les courgettes [euh] ils sont un peu bien, non, juste à éplucher et elles sont toute belle. Ouais- milkshake.

*Mme 1:* La banane en milkshake.

*Speaker 1:* [Euh] ça, je mettrais au compost. D'accord- parce qu'elle est [euh]- comme c'est pourrir. [Euh] les endives, j'en mange pas, mais, non, j'en mange pas du tout. D'accord- pas du tout [euh].

*Mme 1:* Vous n'achetez jamais ?

*Mme 2:* Non. J'aime pas ça. [Euh] les poireaux, [euh] il y a juste à couper ce qui n'est pas beau, et je les fais en soupe.

*Mme 1:* D'accord. Le vert aussi, vous le faites ?

*Mme 2:* [Euh] pas forcément. Ça- ça dépend- Quand c'est vraiment trop abîmé, je mets au compost le- [euh] le vert qui est en hausse. Ouais- donc [euh] ça va, je mets au [euh] à la soupe.

*Mme 1:* À la soupe, OK.

*Mme 2:* Là, la carotte, quand elles commencent vraiment à tirer la gueule.

*Mme 1:* On est sur une carotte.

*Mme 2:* Compost. Ouai- Et la poire, [euh] clairement, je l'aurais pas mangé non plus.

*Mme 1:* Quand on est comme ça, vous- oui- la jetés ? OK. Alors, je vais prendre une photo de votre- quoi. Les endives, elles n'existent pas dans votre consommation ?

*Mme 2:* N'existe pas. Non.

*Mme 1:* Alors ? OK. Alors, et vous m'avez envoyé des photos. Je vais les retrouver. Alors- Ouais, mais ça suffit. Est-ce que vous vous souvenez de [euh] ça ? Est-ce que vous pouvez me raconter ce que c'était [euh] ?

*Mme 2:* Alors, qu'est-ce que ce que c'était ? C'était du- du céleri, çà ? Non, non, je ne sais plus si c'était du céleri. Je sais plus. C'était- [euh] On avait oublié qu'il y avait ça dans les cageots. D'accord- complètement oublié. Et du coup, [ben] on l'a retrouvé en dessous de [inaudible]. Il est apparu et il était- il était- dans cet état- c'est ça. Et là, clairement [euh] ouais- Il n'y avait plus grand-chose à manger dessus.

*Mme 1:* D'accord. Et, est-ce que vous avez un souvenir de [euh] savoir ou- Ou est-ce que vous l'aviez [euh] acheté ? À Leclerc- ouais, c'est pas possible de-

*Mme 2:* Non. Non pas pour l'instant.

*Mme 1:* D'accord. Et du coup, est-ce que vous avez un souvenir d'avoir pensé quelque chose en particulier quand vous êtes- vous avez été amené à le jeter ?

*Mme 2:* Ah, moi, ça me gênait pas tout parce j'aime pas ça.

*Mme 1:* OK. C'était pas pour vous ?

*Mme 2:* C'était pas pour moi, donc ça me gênait pas du tout.

*Mme 1:* Ok, je vois. Donc, vous l'avez jeté comme ça parce que- oui, il est plus bon.

*Mme 2:* Il est plus bon. Je- je les goute pas. Ça ne m'a pas gêné du tout.

*Mme 1:* Ok. Et, vous en avez une note.

*Mme 2:* Ouais, [ben] les carottes du magasin.

*Mme 1:* Ok.

*Mme 2:* C'était le truc qui pique [euh] au bout de [euh] à peine, une semaine, elles venaient toutes comme ça quoi ? OK- elle est toute noire.

*Mme 1:* Ok.

*Mme 2:* Du coup-là, je les ai- je l'ai est fait en tant que soupes. Je les avais épluchées [euh] en dessous, il y avait des parties qui étaient bonnes, donc je les avais faites en soupe. J'avais jeté juste les parties [euh] moins jolies.

*Mme 1:* Ok, vraiment [euh]-

*Mme 2:* Trop abîmées pour [euh]- trop blanche ou noire [euh]-

*Mme 1:* Même pour la soupe [euh]- ouais- Ok, est-ce que vous avez le souvenir d'avoir jeté du coup autre chose à cette [euh] période de [euh]-

*Mme 2:* J'ai jeté des radis. Ouais- parce qu'on avait ramassé tous les radis du jardin ouais- sauf qu'on n'a pas [euh] tout mangé. D'accord- et évidement, ils étaient plus- plus bon. Ok- ils étaient tout raplapla, tout- tout bizarre. On les a jetés. Et qu'est-ce que j'ai jeté [euh] je sais plus. La tomate, je crois aussi. Quand ça pourrir.

*Mme 1:* Ouais.

*Mme 2:* Bon, je sais. Après, je sais plus du tout [euh] les champignons, je crois aussi.

*Mme 1:* OK. Et [euh] est-ce que vous avez [euh] le sentiment qu'il y a eu une quelconque [euh] différence entre [euh] le- la première période de l'étude et celle-ci ? Est-ce que [euh] vous avez moins jeté, plus de jeter ou sensiblement la même chose [euh] ?

*Mme 2:* Oh oh ! Je pense qu'on a plus jeté.

*Mme 1:* OK.

*Mme 2:* Parce qu'on a un coloc qui est parti, donc, il était plus là pour surveiller, pour [euh]- oui- pour nous faire penser à manger les légumes.

*Mme 1:* D'accord, parce qu'avant, il avait plutôt ce rôle [euh] ?

*Mme 2:* [Ben] il- il pensait souvent, [euh] il regardait [euh] souvent le soir les légumes, il disait : "Il faut qu'on fait pas ça, qu'on fait pas ça." Mais là, [euh] on pense pas forcément à- ouais- à faire les légumes quand on voit qu'ils commencent à être- [euh] OK- et après [euh] ouais, je pense qu'on a plus jeté.

*Mme 1:* Ouais. Ah bon !

*Mme 2:* Un peu plus. Je pense pas beaucoup plus, mais un peu plus quand même.

*Mme 1:* D'accord. Parce que le coloc n'est plus là pour [euh]- vous dire ça- vous dire. Ok. Bon, ça marche. Ben voilà. OK- mes décisions- très bien- bon c'est rapide. [Hum-hum] On est large, alors hop ! Je vais couper.

**Household F15, Interview 1**

**Alors, vous êtes prête ?**

Oui !

**Du coup, dans un premier temps, je vous propose un petit jeu de devinette, où je vais vous demander de penser à un fruit ou un légume que vous aimez tout particulièrement. Vous allez penser à ce fruit ou à ce légume sans jamais me révéler son identité. L'idée, c'est que je le devine uniquement grâce à la description que vous allez en faire. Alors, est ce que vous avez un fruit ou un légume en tête ?**

Ouais ouais ouais

**Alors allez y, je vous écoute, dites-moi tout sur ce légume ou ce fruit**

C’est plutôt un fruit exotique… je sais pas, je donne une couleur peut être ? Orange. Sucré. Doux… je ne sais pas *(rires)*. Pas évident à éplucher *(rires)* très mauvais en France, mais très bon quand on les trouve sur place…

**D'accord. Ok… Qu'est ce que vous aimez tout particulièrement dans ce fruit ?**

Je sais pas, c'est le goût et la texture aussi, une texture qui est un peu… ouais qui est douce aussi, je sais pas comment dire. Oui, un peu fondant. Ouais… Sucré ouais et juteux.

**En général, vous le manger quand ? Plutôt le matin, le midi, l'après-midi, n'importe comment, le soir ?**

Quand j’étais à l'étranger, c’est vraiment mon fruit préféré donc j’en mangeait tout le temps en fait ! *(rires)* Ca pouvait aussi bien être dès le petit dej’, le soir en dessert ou sur un marché quand il y en avait.. C’était tout le temps *(rires)*. Je mange beaucoup de fruits de toute façon. Ouais, moi,je suis pas très sucre. Je suis pas quelqu'un qui va aller acheter des pâtisseries, ça m’interpelle pas du tout.

**Mais pas contre les fruits, ouais ?**

Mais par contre les fruits ouais, ça et le chocolat *(rires)*

**Et du coup, est ce qu'en France, vous en consommez ou pas vraiment ?**

Assez rarement. Parce que c'est de l'importation, donc ça m'embête. Et en général, ils sont quand même moins bons. C'est plus difficile de les trouver à bonne maturité, mais ça peut m'arriver de craquer ouais, évidemment. Mais c’est vrai que l'importation, j'évite en général mais bon…

**Et quand vous l'achetez, vous achetez où le plus souvent ?**

Moi, j'achète pratiquement tout sur les marchés, hein de toute façon, les fruits et légumes ouais c’est les marchés.

**D’accord, et est ce que vous pouvez me raconter un peu le procédé de préparation du fruit, c’est-à-dire là, vous l'avez devant vous… Quelles sont les différentes étapes avant que vous puissiez le mettre dans votre bouche ? Si étapes il y a.**

Ouais étapes il y a ouais ouais. Déjà on mange pas la peau donc de toute façon, il y a de lavage avant… Pas forcément, un petit peu mais…après, je l'ouvre au couteau et puis je le mange à la petite cuillère.

**Ok…D’accord… donc vous m'avez déjà dit que quand vous le mangez, vous aimez le goût sucré, juteux. Est ce qu'il y a d'autres choses quand vous le mangez, ce que vous pouvez ressentir ou le sentiment qu’il vous apporte quelque chose ? Ou bien non, c'est simplement gustatif ?**

Ouais je kiffe c’est gustatif *(rires)* Ça doit bien m’apporter des vitamines comme tous les fruits, mais c'est pas ma préoccupation première ! *(rires)*

**D’ accord *(rires)* Je vois. Et du coup, à l’inverse, est-ce que vous lui trouvez des inconvénients à ce fruit ou pas ?**

Ici en France ouais, parce qu’on ne peut pas le trouver… on pas le trouver effectivement, comme je voudrais le trouver. Et après, oui, c’est vrai qu’il est pas facile à manger, on s'en fout partout, c’est hyper juteux *(rires)* Mais bon, à part ça non je vois pas trop d’inconvénients, non *(rires)*

**Ok. Donc il est pas facile à éplucher, il est un peu juteux. Et en France, il n'est pas de très bonne qualité…**

Ben non, mais on en trouve quand même en France, c’est un des fruits exotiques qu’on trouve assez facilement en France quand même.

**Ok.. Donc là, faut que je trouve.**

Mais oui, c'est ça, c'est pas évident. Il y a un gros noyau

**Ah !**

Et c’est assez fibreux quand même.

**Niveau taille, il est rond, ovale ou pas ?**

Ouais rond ovale

**La chair est orange, mais la peau est un peu verte-orange?**

Orange vert ouais ça dépend de la maturité

**et ils sont à peu près gros comme ça ?**

Ouais… C'est ça.

**Je dirais la mangue ?**

Exactement, bien joué ! *(rires)* Quand je vivais en Thaïlande, mais c'est un carnage. Elles sont top là-bas, c’est formidable.

**Oui, là j'en ai acheté... C'était pas.. c’était pas la folie j’avoue, c’était la première fois que j’en achete en France.**

Ben’ non c’est pas génial… Et en Grèce, j’en trouvais des bonnes aussi, mais beaucoup plus petites. Mais qui étaient pas mal. Mais ouais en Thaïlande, en Malaisie, c'est… Mais là-bas c’est la folie quand on aime les fruits c’est l’éclate, vraiment ! Même les bananes n’ont pas du tout le même goût, c’est hallucinant !

**Mais oui, en France, c'est un peu…**

Mais c'est vrai qu'en France, c'est un des fruits exotiques ; comme la banane, qu'on trouve énormément. Parce qu’il y en a plein qu'on ne trouve pas du tout aussi.

**Oui, qu’on ne connait pas… c’est possible qu’il y ait plein de fruits que je ne connais même pas.**

Oui, moi, j'ai découvert les trucs en Thaïlande. Je ne savais pas du tout que ça existait. Les fruits du dragon, par exemple, c'est vraiment bizarroïde, on s'y attend pas du tout, c'est pas très bon d'ailleurs, je trouve mais c'est vraiment bizarre. En fait, ça ressemble à un chou-rave un petit peu, avec des espèces d'épines là, c'est vraiment rose. Et quand on coupe en deux, la chair est toute blanche avec des points noirs, avec plein de pépins noire… Mais c’est vraiment chelou comme truc mais c’est pas très goûtu. . . J’ai gouté des snake-fruits, je ne sais pas, comment on le dit en français. Des fruits du serpent. Un truc comme ça. Une espèce de peau en écailles aussi, un peu dur, assez difficile à croquer. Mais qui ont un goût vraiment bizarre. Il y a plein de trucs… et ça, non, ça ne vient pas en France. Je pense après qu’en terme de transport, et puis peut-être même en termes de goût, c’est vrai que la mangue c’est top aussi… Donc ouais, ça, ça me manque.

**Du coup, maintenant, si on s'intéresse à vos pratiques d'achat et de votre organisation générale, racontez-moi du coup, comment s'organisent vos achats, notamment sur les fruits et légumes. Vous avez dit que vous alliez au marché, est-ce que vous pouvez me raconter un peu, comment vous organisez ? Si vous avez une organisation particulière…**

Ouais plus ou moins, j’ai le marché en face, le marché de [Nom du marché], marché bio local, donc voilà c’est pas très compliqué. Le mardi et le samedi, donc, j'y vais le mardi et le samedi, dans la mesure du possible, parce que le mardi, je travaille, donc il y a des fois où je ne peux pas toujours… et j'achète bah 100% de fruits et légumes là en fait, c'est vraiment rarissime que j'aille ailleurs.

**Ok, donc c'est une à deux fois par semaine…**

Deux fois en général ouais. Du coup, j'en achète pas trop, ce qui évite les pertes.

**Ouais, comment vous gérez vos quantités du coup ?**

Je me connais, c'est toujours la même petite routine. Un petit citron tous les matins, la petite banane, tous les matins. C’est con mais je compte quoi… *(rires)* j’en achète 4, j’en achète 5… Bon, les légumes, j’y vais vachement au feeling. C’est vrai que des fois, je me fais un petit peu déborder, mais pas trop quand même. Je m'organise parce que je connais ma routine. Moi, je fais à manger le midi, je ne mange pas en cantine, mais non moi, je fais toujours à manger, j’achète pas de sandwitch et tout. Donc du coup, je calcule à peu près mes repas. Et puis voilà… L’autre jour je me suis fait avoir, j'ai jeté quelques radis dans votre poubelle là, parce que j’étais partie en formation encore et c’est vrai qu’ils ont pas bien résisté… à mon retour, ils étaient pas biens…

**Ouais ? Vous ne mangez pas à l'extérieur ?**

Non

**Enfin, si vous devez manger, vous préparez vous même un repas ?**

Je prépare toujours ma gamelle pour le midi.

**D'accord. Par contre, ça vous arrive d'avoir des déplacements de plus longue durée que la journée, vous disiez, ce qui fait que…**

Oui, mais après, ça dépend. C’est vrai que quand je suis en formation, bah forcément, je mange avec les autres le midi. Je vais pas faire mon original avec mes gamelles. Oui, mais même dans la journée, quand je suis en déplacement, c'est vrai qu’en général, sinon, je me prépare une salade que j’ai dans la voiture, je vais pas m'acheter un sandwich, j’aime pas trop… Je suis ingénieure en agro-alimentaire, je crois que ça m'a traumatisé *(rires)* Du coup j’ai jamais bossé dans cette branche là, mais ça m’a traumatisé leurs usines et leurs trucs, quand on voit la qualité de la bouffe. Puis j'ai été élevé comme ça, j'ai été élevé comme ça avec mes parents, mes grands-parents qui avaient un potager gigantesque et qui faisaient des conserves qui nous fournissaient tout en fruits et légumes. Ma mère qui cuisinait, donc je sais que mon frère est pareil enfin voilà, il y a vraiment aussi… on a été élevés comme ça quoi.

**Avec des produits frais…**

Avec des produits frais ouais c'est pareil, j'achète jamais de légumes... Je sais que ça se fait hein… Des patates déjà épluchées là dans des sachets, des poêlées de légumes et tout ça, non ça c'est pas mon truc. Moi, je mange mes fanes de betteraves, je mange mes fanes de radis,

**Ok, c’est intéressant aussi…**

Je mange tout quoi ! C'est super bon d'ailleurs, les fanes de betteraves, vraiment c’est top. Je ne comprends pas que les gens ne les cuisinent pas plus que ça.

**Comment vous les cuisinez vous du coup, les fanes ?**

En général les radis, je les fais en soupe, car quand même crus, c'est pas top. Les carottes aussi, ça m'arrive de les faire en soupe, mais j'aime un peu moins le goût et les betteraves, bah c’est cru ou cuit. Ça se fait très bien en salade.

**Ok, d’accord.**

Pareil, les petits oignons nouveaux, je mange toute la tige... J'aime bien, j'aime bien ouais, mais en plus, c'est bon. C'est ça le truc, c'est qu'on a pas l'habitude, mais en fait on trouve des recettes vraiment super sympa.

**Ouais… Ouais quand vous dites que nous ne comprenez pas trop pourquoi, pourquoi les personnes ne consomment pas forcément… Vous dites qu’on a pas l'habitude de consommer. Il y a peut-être ça, est ce que vous, petite, vous mangiez déjà, les fanes ou non, c'est venu plus tard… ?**

Non, c'est venu plus tard ouais ouais c’est venu plus tard

**Et qu'est ce qui a fait que, depuis quand à peu près ? Qu'est ce qui a fait que vous êtes mise à consommer les fanes ?**

Je pense que c'est le fait d'aller sur les marchés aussi parce que c'est vrai que sur les marchés, on nous fait des bottes de légumes nouveaux là en ce moment, et c'est génial et il y a les fanes, il y a tout et c’est vrai que je me suis intéressée à ce que je pouvais en faire. Et voilà, parce que souvent ils demandent s’ils les enlèvent ou pas et c'est un gâchis… Et puis c’est vachement bon, c’est surtout ça ! *(rires)* Je vois pas trop pourquoi on s'en priverait ? Moi, j'aime bien manger des soupes et du coup ouais, je les mets dans mes soupes, c’est top. Mais je pense que oui, c'est le fait d'aller sur les marchés parce que plus jeune, je le faisais pas, ouais, effectivement… On discute enfin c’est vrai que... J'ai même découvert, c’est vrai qu'il y a pas mal de légumes qui sont revenus aussi, qu’on ne mangeait pas étant plus jeunes

**Ouais, comme lesquels ? Vous pensez à quoi ?**

Je pense au chou rave. C'est vrai que moi le chou rave, c’est pas un truc que je mangeais étant plus jeune, les potimarrons non plus.. il y a des variétés de tomates qui reviennent. Bah oui, les fanes, on les mangeait pas…. Qu’est-ce que je mangeais pas ? Fenouil j’en mangeai pas… Ouais voilà des trucs comme ça, ce n'était pas dans l'air du temps. On a un peu changé quand même…

**C’est possible, ouais !**

Les maraîchers ouais, ils ont sorti des choses qui… tous les petits maraîchers locaux bio ont quand même remis au goût du jour tout un tas de légumes qu’on ne consommait plus du tout.

**Oui, vous pensez que les habitudes, les habitudes alimentaires ont pu changer au fil du temps… ?**

Pour les gens qui ont la sensibilité d'aller sur des marchés bio et locaux, oui, effectivement, le commun des mortels qui achète tout en surgelé, n'ont peut-être pas connaissance de l'existence du chou rave et de ces choses là *(rires).* Mais parce que je sais qu'il y a des gens, qui ont une consommation alimentaire qui est quand même particulière à mon sens. Mais bon, moi acheter du tout fabriqué en grande surface, je peux pas… mais je pense que oui, il y a de plus en plus de gens quand même qui ont cette sensibilité là, et que le maraîchage, ça se développe beaucoup. Et moi, j'ai l'impression en tout cas que ça change. Je n'avais pas ce souvenir là, étant gamine et même étant jeune, j'allais déjà sur les marchés et je trouve que ça a changé.

**C’est plus les mêmes propositions sur les étals quoi..**

Ouais, y a plus de variétés anciennes qui reviennent ouais.

**D’accord. Et quand vous allez sur le marché, est ce que vous savez ce que vous allez prendre ou pas ? Avant de partir, vous savez vraiment avant ou non…**

Alors pour les fruits, je suis assez routinière ouais, ça c’est vrai.

**Ah oui, avec le citron et la banane du matin**

Ouais un truc comme ça, non, après c'est plus au feeling parce que de toute façon, vraiment là comme c'est du local… Bah, faut faire avec les saisons. Là, on a eu un gros mois de creux, il y avait plus grand chose, mais là, ça commence à revenir des petits trucs nouveaux…. Mais c'est vrai qu'il y a des fois ben… patates, carottes quoi *(rires)* les choux, c'est fini…. Et puis, les épinards ne sont pas encore revenus. Ouais, donc, je fais un peu comme… comme ça, en fonction de ce que je trouve et ce qui me fait envie. Je mange beaucoup de blettes aussi. J'aime bien ça, mais il y en a pas toujours *(rires)*

***(rires)* Ah ouais ? Parce que c’est un légume qu’on a assez longtemps dans le temps, mais c’est vrai que des fois quand on y va… il y a toujours, des semaines à l’autre, c'est toujours les mêmes légumes… Et à la fin de la saison, c'est dur… Quand il y a un trou entre l'ancienne et la nouvelle, on se retrouve devant l’étalage avec… des pommes de terre *(rires)***

Ben oui, comme au niveau des fruits il y a plus grand-chose quoi c’est des vieilles pommes euh… *(rires)* Là ça revient on commence à avoir des cerises. Ceci dit, je ne sais pas trop d’où elles viennent… Ça me paraît un peu précoce. Mais bon.

**Oui, c'est possible.**

Les cerises, les fraises, voilà, c'est saisonnier quoi. C'est vrai que l'hiver bah ouais c’est… poires pommes, puis un peu d'agrumes quoi, puis des bananes, ça je sais que c’est pas bien, c’est importé aussi mais j’aime vraiment bien les bananes.

**Et bien écoutez, vous n’êtes pas la seule. Je remarque quelque chose autour de la banane qui est assez… *(rires)* Plein de gens qui évoquent, consommer assez local, limiter l'importation… Il y a souvent le discours… « euh sauf pour la banane » *(rires)***

*(rires)* Mais oui, c’est ça.

**C’est assez rigolo, je pense qu’elle fait partie de notre**

L'avocat aussi ! L'avocat pose problème.

**Tout à fait.**

Parce que c’est vraiment top, les avocats mais c’est vrai… j’en achète pas souvent, je me limite.

**Je comprends tout à fait ! Alors, du coup, la volonté d'aller au marché… c’est par praticité où il y a une volonté derrière, enfin voilà si on vous dit pourquoi vous allez au marché ? Comment vous vous positionnez ?**

Il y a deux raisons en fait pour moi. Déjà j'achète bio, alors je fume hein, vous pouvez remarquer que ça sent la clope.

**Je fume aussi, donc je ne dois pas sentir !**

C’est complètement ridicule, je mange bio et je fume, donc il y a bien un problème déjà, mais en tout cas moi, je mange bio, et ça, c'est plus pour ma santé, mais aussi pour l'environnement, parce que ça m'énerve. Je travaille dans l'agriculture, je suis ingénieur agro-alimentaire donc déjà ça part mal et je connais trop bien, en fait, je connais trop bien cette filière là, c'est ça le problème. C'est que je la connais trop, trop bien et ce que je constate aussi, c'est que malheureusement, tout ce système de grandes surfaces de prix tirés vers le bas… Bah moi je préfère aller donner mon argent et payer un peu plus cher, mais de donner à une famille qui est un exploitant, qui fait vivre sa famille avec une que d’aller donner à Carrefour, qui va payer sa caissière au lance pierre et enrichir ses actionnaires… Ou alors j'achète des actions Carrefour, dans ces cas là.... Mais euh ça fait très longtemps que moi, je suis là dessus et je sais, je sais, c'est un discours qui m'a toujours énervé en fait, mais depuis que je suis très jeune et avant, je passais vraiment pour une allumée. Sauf que maintenant, c'est plus dans les mœurs. Mais quand les gens me disaient c'est super il y a des promos chez Lidl, je disais « Mais putain, vous savez ce qu'il y a derrière quand même ? » Ca veut dire que nos emplois se barrent, ça veut dire qu'on importe la nourriture. Après nous, les jeunes, on trouve… Enfin les jeunes, maintenant, je suis plus jeune, mais à l'époque, c'était déjà galère de trouver du boulot…. Donc moi, c'est tout ce système qui me rend complètement malade et je voudrais qu'on revienne à ce qu'on avait avant avec la petite boucherie, la petite épicerie, la petite droguerie avec une famille derrière qui vit et c’est pas des espèces de grands groupes gigantesques, avec des actionnaires qui… qui traitent leurs salariés comme des merdes sous payés. Je… Enfin moi, c’est ça que j'essaie de faire aussi en achetant à manger quoi… Mais dans beaucoup de choses, j'achète très peu de choses d'ailleurs, je ne suis pas très consommatrice. Je ne vais pas sur Amazon, je n'ai pas d'abonnement à Netflix et toutes ces conneries… J’achète la moitié de mes fringues d'occasion dans des friperies… Enfin le système m’énerve, vraiment, je suis à fond là-dedans. Donc c'est très engagé aussi. C'est très engagé, mais je sais que c'est aussi excellent pour ma santé. Et qu'est-ce que c'est vachement plus agréable, plutôt que d'avoir une caissière qui fait la gueule, de passer une heure au marché avec des gens souriants quoi ! *(rires)* C’est toutes ces petites choses mises bout à bout… Et puis après, effectivement là j’ai le marché en face, donc ça serait en plus, vraiment ridicule de pas faire l'effort d'y aller quoi…

**Ouais, c’est vrai que là tout y est ! *(rires)***

*(rires)* Voilà, car je sais, je sais que ça peut être très compliqué pour certaines familles aussi. Tout le monde peut pas se permettre, avec les emplois du temps, les enfants, si on habite pas près d'un marché puisque malheureusement en fait, quand on habite en campagne, on se rend compte qu'on a moins facilement accès aussi à ces marchés là… faut sortir la voiture, machin… Finalement, en ville, on nous amène tout. C'est quelque chose de plus facile aussi je pense. C’est pour ça qu'on parle souvent des bobos des villes aussi, mais mine de rien, c'est vrai que eux, ils connaissent rien à la campagne, donc ils ont une vision un peu… Quand ils veulent s'installer en campagne, ils déchantent parfois. Mais n'empêche qu'on leur amène aussi tout ça plus facilement que la personne qui vit en campagne, je trouve. Quoiqu’il y a pas mal de petits marchés en Bretagne.

**Mais ça, effectivement. Quand on regarde Brest, tous les marchés, chaque petit quartier a quand même à un moment donné son marché. Quand on est en campagne, il faut parfois aller au petit bourg, mais des fois, ça peut être assez long à pieds… Donc effectivement la question de l’accessibilité à ces produits là…**

Ben oui, c'est ça qui est paradoxal !

**Alors que c'est plutôt des produits qui viennent de la campagne.**

Ben oui, c'est ce qui peut paraître paradoxal… après il y a la solution de faire son potager. Mais encore une fois, il faut du temps pour ça. Ouais, moi, j'ai pas d'enfant, donc je suis tranquille, mais j'imagine une famille, les deux parents qui travaillent et trois quatre gosses, c’est pas le même rythme hein !

**C'est clair.**

Je pense que les courses, à un moment donné, c'est plus facile d'aller à Leclerc et… voire au drive et de faire, de faire le plein. C'est dommage, mais…

**Tout à fait. Donc ok, le marché, toute une démarche qui rentre dans une consommation engagée, qui est assez générale, et l’alimentaire passe**

Ouais c’est le sans-emballages aussi… enfin voilà les emballages réutilisables, etc. Parce que ouais, acheter des trucs sous plastique…. En grande surface, le bio ils le mettent sous plastique, super ! C’est comme les Biocoop ! Je suis très fâchée avec les Biocoop.

**Ah ! Racontez moi. Pourquoi ?**

Parce que les Biocoop, au départ, je trouvais ça top. Enfin moi, j'avais des Néerlandais dans la ville où je suis née qui avaient monté un magasin bio. Alors eux, c'est pareil, c'était des allumés de la première heure, parce que c'était il y a déjà 30 ans. C'était assez rare, mais tout était en vrac, déjà beaucoup. Et là c’est vrai que les Biocoop, je trouve ça hallucinant. Oui, c'est bio, mais c'est sous plastique. Maintenant, ils font même des plats préparés bio. Moi, ça m'énerve profondément parce que je ne comprends pas où est la logique. Je ne vois vraiment pas où est la logique avec des trucs d'importation. Je comprends pas. J'y vais de temps en temps pour acheter du vrac. Mais c’est vrai qu’il y a des fois, je suis vraiment fâchée. Surtout quand je vois les rayons snacking aussi pour le midi. Alors on en a la mini salade avec la fourchette…. Non mais sérieux, le mini jus d'orange, le mini machin, tout est sous plastique et je comprends pas en fait. Je trouve que ça s'est égaré. C'est devenu aussi…. C'est devenu comme tout le reste de greenwashing. Bref, on a complètement perdu l'esprit du bio au départ. Et puis, ça coûte une fortune en plus, ils sont vachement chers. Alors que sur le marché, la viande me coûte cher, c'est clair, mais en terme de fruits et légumes, ça va quoi… c'est vraiment pas cher. Je pense qu'il y a aussi une image, « le bio c'est cher », mais ça dépend où on se le procure, vraiment là c'est pas cher du tout. Le fromage et la viande, oui bien sûr, parce que les animaux en bio, ça coûte cher, oui.

**Oui, parce que ça demande encore plus de travail pour que soit bio…**

Ouais puis beaucoup plus d'investissements parce que s'ils veulent faire l'abattage et la transformation à la ferme, ils faut qu’ils construisent des labos. Ça coûte une fortune, avec les normes et tout, c'est très compliqué, c’est compliqué… le maraicher lui c’est tranquille, il met ses serres, il fait son champ, il y a très peu de mécanisation. Mais dès qu’on commence à entrer dans une logique d'abattage à la ferme, de transformation. Moi, je suis des agriculteurs. J'ai des montants d'investissement purement délirants... Je me rappelle de gens qui faisaient du fromage comme ça. On était à un million d'euros quand même pour refaire entièrement la laiterie. Bon, c'est vrai que ça a marché mais vraiment… Enfin, ils exportaient sur Paris, ils exportaient de la Normandie, sur Paris. Ils avaient vraiment, leur fromage, faisait un carton, donc ils avaient voulu agrandir. Mais on est sur des échelles complètement dingue en terme de prix.

**Et donc forcément, après le produit.**

Donc forcément, le produit est cher et il faut le camion frigorifique alors que le maraîcher lui met des tréteaux et une planche, les légumes, il y a pas de souci quoi…, mais les contrôles d'hygiène et tout, c'est difficile pour eux de faire moins cher en fait.

**Oui, bien-sûr. Ok, Très bien. Du coup, vous avez déjà commencé. Mais si on essayait de faire une liste des différents fruits et légumes que vous avez l'habitude de vous procurer. Alors, pour commencer ce que vous allez consommer très régulièrement, donc par exemple sur les fruits, vous m’avez dit les citrons bananes, pomme…**

Ouais citron banane oranges

**oranges, poires vous m’avez dit.**

Ouais… beaucoup de gingembre et de curcuma aussi…. Qu’est-ce que je mange d’autre comme fruits…

**Vous m’avez dit les fraises, les cerises,**

Ouais, les fraises, les cerises. Non c’est rare que j’en achète.

**C’est plus rare ?**

Ouais, les fraises, mais là, c'est hors de prix, les fraises, c'est vraiment le produit du luxe là quand même, même sur les marchés hein ! Oh mon dieu ! *(rires)* Tous les petits fruits rouges. Franchement, c'est vrai que là, c'est compliqué. Je comprends que les familles elles aient pas les moyens de s'acheter ce genre de fruits. C’est vraiment vraiment cher. Après en légumes, pfiou, je mange de tout moi : pommes de terre, carottes, radis, des blettes, des oignons, de l’ail, je mange vraiment de tout, tout ce qu’on peut trouver.

**Quand vous êtes sur le marché, il n'y a pas de restrictions, vous prenez ce qui vous donne envie**

Ah ouais ouais. Poireaux, enfin plein de trucs, épinards, betteraves, je mange tout, vraiment tout, tomates quand il y en a… Salade. Je suis assez herbivore en fait, je mange pas beaucoup de viande ! *(rires).*

**Et du coup, s'il y en a que vous consommez que très rarement, voire jamais. Par exemple vous avez dit la fraise, du coup c'est notamment en termes de prix là que ça se joue ?**

Ouais, je trouve quand même que là pfiou ! *(rires)*

**Et est-ce qu’il y en a d'autres, des fruits que vous que vous connaissez, mais que vous n'allez pas consommer ? Donc les fruits exotiques vous avez dit que « oui et non », c’est plus rare.**

Oui, du coup, c'est plus rare mais c'est pareil. Ils sont très cher, très cher, mais c'est plus rare. Ca c’est l’importation, ça m'embête. Les fraises de Plougastel, c'est du local *(rires)*. Ça reste… enfin c’est dans des serres même temps *(rires)* Mais après ? Non, je n'ai pas de restriction particulière. Moi, j'ai les moyens, donc c’est pas le souci, c’est pas vraiment le souci, non après c’est plus tout ce qui est importations qui me gêne. Et puis, tout ce qui est en sous serres, ça m'énerve. Les gens qui cherchent des tomates en plein mois de décembre, faut arrêter de délirer quoi ! *(rires)*

**Complètement. Ok… Et les légumes, pareil du coup, ça va être de saison. Mais sinon, il n'y a pas un légume auquel vous pensez qui, que vous excluez de votre consommation en particulier ?**

Le fenouil et le rutabaga, parce que je ne trouve pas ça très bon.

**D'accord, donc là ce sera plus en terme de goût.**

C'est franchement dégueulasse *(rires)* de mon point de vue. Après…. non par exemple les asperges. Je pensais à ça, c’est vrai que j'aime bien, mais j'en fais pas parce que je trouve ça chiant à faire ! *(rires)*

**D'accord…**

Tout simplement *(rires)*. Puis ça m'éclate pas au point que j'ai envie de m'embêter, en fait !

**Je vois *(rires)***

Après non pas trop… ah si le chou, non comment c'est…. l'artichaut ! L'artichaut, je déteste. Mais voilà tout ça c’est des questions de goûts ouais.

**Goûts plus l'asperge, même si ça passe, c'est un peu chiant à faire…**

Ouais je trouve ça chiant à faire, clairement.

**C’est pas non plus le grand kiff**

Ouais voilà c’est ça *(rires)* Non mais après c’est vrai qu’il y a quand même une contrepartie quand même qui n'est pas négligeable quand on achète comme ça, c’est qu'il y a un temps de préparation qui est assez important. En général, quand je rentre du marché, déjà, il faut que je nettoie tout ces… tous ces légumes donc bah, les épinards, les blettes, toutes ces choses là, les radis, faut tout passer à l’eau, parce qu'il y a de la terre. Oui, c'est pas les trucs de supermarché qui ont été javellisés avant. Donc ça prend du temps. Après il y a la cuisson, c’est vrai que moi j'achète souvent les betteraves crues, donc je les cuits. En général, je passe bien deux heures à faire déjà de la préparation de légumes quand je reviens mon marché, ouais.

**Ca c'est intéressant, justement, quand… c’est dans mes prochaines questions. Quand vous rentrez de vos courses, qu’est-ce que vous faites avec vos fruits et légumes ? Typiquement est-ce que vous avez une organisation, est-ce que vous les rangez, vous les préparez ?**

Bah déjà ici c’est tout petit. Bientôt, j'aurai ma maison, ça ira beaucoup mieux, mais comme là c’est tout petit, bah de toute façon je lave tout, et je cuits tout, même pour stocker au frigo, ouais je lave tout avant quoi.

**D’accord.**

Je précuits beaucoup de choses. Puis, comme j'y vais deux fois par semaine, oui, souvent si j’achète des épinards, des blettes, des choses comme ça, je les cuits tout de suite, après ça tient bien deux trois jours au frigo, si je ne les mange pas immédiatement. Et puis je retourne au marché 3/4 jours après. Mais oui, ça demande un petit peu de travail. Les fruits, y'a rien à faire, je les mets dans la corbeille à fruits puis voilà. Les agrumes au frigo, parce qu'en bio, c'est vrai que ça a une fâcheuse tendance à pas se conserver du tout !

**Donc les agrumes au frigo et le reste**

Non dans la corbeille normalement ça tient ouais. Après c’est vrai qu’en bio, il faut faire attention parce que ça se gâte vachement plus vite.

**Ouais. Les légumes du coup, ils sont souvent déjà un peu préparés et vous les mettez au frigo. Est-ce que vous avez d’autres lieux de stockage ou pas, pour vos légumes ?**

Non.

**Ils vont tous au frigo ?**

Ouais bah sauf les patates qui restent… sinon tout le reste va au frigo. Patates, patates douces, je laisse ça à l’extérieur.

**Oignons, vous mangez de l'oignon ?**

Oui. Ah oui, les oignons. Ben oui, ça dépend. Oignons frais au frigo, oignons de conservation je les garde à l'extérieur du frigo, l’ail aussi.

**Sinon, tout est au frigo.**

Ouais quasiment ouais.

**Et là, vous avez dit que vous aviez un espace qui est plutôt petit. Si jamais vous n’avez pas de restriction d'espace, pas de restrictions financières, que tout était possible. Quel stockage vous imaginez ? Quelle méthode de conservation vous mettrez en place ? Est ce que vous avez des idées ou pas ?**

Bah moi, dès que j'aurai la maison, déjà, y'aura le potager. Ca c’est clair, je vais essayer d'en faire un maximum. Et puis là, du coup, ouais j’achèterai un congélateur. Mais on serait peut être dans la stérilisation aussi. Faut voir. Je sais qu’il y a la lacto-fermentation aussi qui se fait. Mais en termes de goûts, j'en ai mangé en Egypte des trucs comme ça, mais j'ai un peu de mal avec l'acidité. Mais encore, ce serait à tenter. Mais oui, je pense que ça va être plus de la stérilisation et de la congélation effectivement.

**Ok. Et vous pensez garder l'habitude du réfrigérateur, autant ou pas vraiment… ? 32’’’’20**

Bah, du coup, ça va être différent parce que si j'ai mon potager, j'irai chercher au fur et à mesure. Donc j'aurai certainement moins besoin de stocker au frigo… Enfin si tenté que j'arrive à faire pousser quelque chose dans mon potager *(rires)*

**Peut-être que les premières années seront creuses *(rires)***

J'en ai déjà fait par le passé et c'est vrai que des fois, ça s’improvise pas quand même… il y a quelques… quelques échecs retentissants. Mais si jamais ça marche, je sais que quand j'avais mon potager, ben oui, j'ai besoin de tomates, je vais en cueillir deux et puis voilà je les mange directement et puis après forcément sur l'arbre, elles ne vont pas s’abimer. C'est complètement différent, c’est comme les herbes aromatiques. C'est toujours chiant quand on achète un bouquet de persil au marché parce que c'est toujours des grosses quantités pour moi qui suis seule et donc, où je me gave de persil pendant 3 jours ou j’en jette la moitié, et là bah au jardin, il suffit de couper 3 brins quoi, ça change tout.

**Complètement. On se sert directement en fonction du besoin.**

Voilà c’est plus pratique, donc je pense qu'il y aura moins de stockage frigo et par contre plus de stockage congélateur, parce que c'est comme ça que je fonctionnais avant. C’est vrai que les courgettes, c'est un truc de dingue, quand ça marche, on n'en peut plus des courgettes, on en donne à tous les voisins, il y a des courgettes partout. Et donc souvent, ce que je faisais, c'est je les mettais au congélateur. Et puis l'hiver, ça passait dans les soupes, parce que c’est vrai que le congélateur ça les abîme un peu…. Soupe, ratatouille, même en ratatouille, c'est limite quand elles sont congelées, mais bon, ça passe. Et voilà, je conservais beaucoup les haricots et petits pois, tout comme le congélateur, c’est vraiment pratique, mais cela consomme un peu d'énergie… donc je sais pas

**C'est dur hein de… Oui *(rires)***

*(rires)* On peut pas être parfait mais voilà

**On peut pas être parfait, sur tout quoi, il y a forcément un moment où on est obligé de… Déjà d’en avoir conscience, de savoir quand on fait ce que ça fait, c’est déjà**

Oui, c'est déjà ça *(rires)*

**C’est la première étape !**

Après, mon rapport aux fruits et légumes est très santé aussi c’est vrai que je suis très... Il n'y a pas que le côté environnemental. Je mange peu de viande aussi à cause de ça mais ouais c'est très santé aussi… Les gens… J'ai l'impression que beaucoup de personnes ne mangent pas beaucoup de fruits et légumes. Je trouve ça toujours un peu bizarre, parce qu’en terme de santé… Les plats préparés, enfin… Je ne sais pas si…

**Je ne sais pas si….**

Quand je vois qu'on est obligé de faire la promotion des cinq fruits et légumes par jour. Je me dis mais merde ça veut dire qu'il y a un tant de gens que ça qui n'arrivent pas à manger… Ça va très, très vite les cinq fruits et légumes par jour quand même.

**Je pense qu'il y a eu un…**

Sur 3 repas, c’est….

**Je ne sais pas… c’est moi qui fabule peut être, mais qu'avant, on cuisinait les fruits et légumes parce qu'ils n'y avaient pas d’autres propositions, c'était comme ça qu’on pouvait manger, il fallait bien cuisiner… Et qu'après, il y a eu toutes ces propositions, avec une société où le temps s'est raccourci, avec des activités, un travail… Les femmes se sont mises à travailler, ce qui est très bien, mais du coup voilà… Et là j’ai l’impression qu’on revient un peu en arrière, sur un peu tout, parce qu'il n'y a plus trop de choix. Mais je pense que j'ai tendance à espérer que ça revient, que les jeunes générations…. Retournent sur le marché, etc**

Ca revient doucement puisque dans l'immeuble il y a que des étudiants et je les ai jamais croisé au marché *(rires)* Par contre des ubereats en bas pour leur amener je ne sais pas quoi à bouffer là… *(rires)*

**Là vous les voyez ? *(rires)***

Donc dès fois je désespère un peu !

**C'est vrai que non, c'est pas…**

Mais c’est vrai qu'après… le micro-onde, toutes ces choses que nos grands-parents avaient pas, nos parents, un peu. Ca a changé tout quoi, voilà on s'achète une pizza, on la mets au micro-onde, on la mange, c'est vrai que c’est bon hein, au niveau du goût il y a pas de souci. Moi qui fait de l'agro alimentaire, personnellement, on ne me fera jamais manger ça… Mais après ouais, il y a plein de propositions maintenant, de trucs qui se font en dix minutes maximum.

**C’est ça… Donc quand le temps vient à manquer… à mon avis, la question s'est pas posée.**

C'est vrai que c'est chiant un peu. Quand on cuisine, ça prend beaucoup de temps. Et puis, tout le monde n'aime pas ça. Moi, j'aime bien, donc ça va, car c'est un truc qui me déplaît pas du tout. Mais je conçois qu'il y a des personnes qui n'aiment pas ça, que ça peut gonfler assez rapidement.

**Vous aimez cuisiner ?**

Ouais moi, j'aime bien ça, donc c'est vrai que c'est plus un peu simple aussi.

**C'est sûr. Et justement, si on s'intéresse à vos différentes façons de consommer les fruits et légumes, si on prend les fruits, un fruit, par exemple la banane que vous mangez régulièrement, vous avez dit le matin. Racontez-moi, sous quelle forme vous pouvez consommer la banane ?**

Bah, cru comme ça.

**Cru ? Du coup, surtout le matin ?**

Ouais *(rires)* petit rituel de la banane tous les matins et en fait je crois qu'il y a vachement de gens qui font ça ! Je sais pas pourquoi, on a été élevé à la banane le matin *(rires)* Ca et les chocapic quoi,c’était vraiment le truc !

**C’est vrai, il y a des gens qui m’ont dit « je pars au boulot. Je prends pas le temps de déjeuner, mais c'est la banane que tous les matins, je prends là-bas pour petit déjeuner ».**

Mais ouais voilà, c’est la banane quoi. Les bananes et le porridge en ce qui me concerne mais après ouais…

**Donc la banane crue. Donc, vous faites quoi ? Vous avez la banane.. ?**

Et ben je l’épluche et…

**Vous la mangez comme ça ?**

Je la mange comme ça ouais. Ouais, c'est vrai que je ne prends pas le temps comme ça, je vois certaines personnes qui font des super…. Non moi c’est assez rapide, mais non, je ne fais pas trop ça et souvent les fruits, mais pratiquement tous hein, je les mange juste comme ça. Je les prépare pas plus que ça… les pommes et poires, je les croque directement… si je presse mes oranges, mes citrons, parfois, pour faire des jus mais sinon, non les fruits, je les mange comme ça.

**C'est marrant ça, vous êtes la première à me parler de jus parce que du coup, je demande, les fruits, est-ce que certaines personnes les cuisinent ? Donc par exemple les pommes on m’a dit « ouais des compotes ou des choses comme ça ». Et personne m'a dit faire des jus, alors je pense que certaines personnes doivent en faire, par rapport à comment ils…et pourtant ce n’est pas venu à leur esprit de parler du jus.**

Il y a beaucoup de gens qui ont des extracteurs de jus et tout ça quand même, c’est…

**Et là oui parmi les gens que j'ai rencontrés, il y en avait qui avaient vraiment essayé de faire le fruit sous toutes ses formes.**

Ouais parce qu’il y a le smoothie aussi qui est vachement à la mode. Et personne n’en a parlé ?

**Non ouais… sous la forme liquide je sais pas pourquoi ça n’a pas… c’est curieux**

Etant gamins, avec mon frangin, on faisait des milkshake, avec les fruits, à toutes les sauces. Ca c’était…

**Avec la banane…**

J’en fais plus parce que je suis devenue totalement intolérante au lait.

**D'accord…**

Mais c’est vrai que le milkshake, qu'est ce qu'on a pu en faire ? Je ne sais pas ouais si ça se fait encore beaucoup… le smothie.. le lait a perdu..

**Mais smoothie je mets du lait… mais j'essaye du lait de soja, c'était un peu moins bon… que du bon lait de vache ! *(rires)* Mais c’est possible, c’est une forme de smoothie !**

Mais c’est vrai que non, je ne fais pas beaucoup de jus. Il y a des gens qui en font pas mal et j'en… J'en faisais plus quand j'étais à l'étranger, justement ouais.

**Ouais, quand il fait bien chaud.**

Oui, puis là-bas c’était l’orgie aussi… Y'avait tellement de fruits hyper juteux, c’était idéal quoi.

**Oui, c'est clair, il y a même des bars à jus un peu partout.**

Ben oui, il y en a partout !

**C’est ça ! Et du coup, pas de pas de cuisine particulière, des fruits à part par les jus, ça vous arrive pas de faire des préparations sucrées ou ?**

Non..

**C'est cru ?**

Ben oui, puisque les vitamines sont là. C'est un peu bête de le cuir…

**Ouais...**

Et puis ben’ non, et j'aime pas manger sucré salé. Donc, c'est vrai que… je sais qu’il y a beaucoup de gens qui mettent des fruits dans leur tajine et tout. Alors moi, je déteste ça donc déjà ça restreint tout à fait ! *(rires)* C’est vrai qu’il y a beaucoup de gens qui font ça. Ça peut être sympa.

**Donc vous l’ananas dans la pizza, c’est hors de question ?**

Ah mais c’est hors de question. Ah non mais là, s’il y a bien un truc, c’est ça. Mais en plus, je pense que les Italiens doivent être mal avec ça ! Je travaillais avec une italienne, c’est vrai que je ne lui en ai jamais parlé. J’aurais dû !

**Je ne sais pas d'où ça sort.**

C'est un crime, quand même, ça doit être un truc des Américains, avec Hawaï ! Ils ont dû se dire aller ! *(rires)* Non c’est pas mon truc non !

**Ok *(rires) Et* sinon, les légumes… comment vous avez l'habitude de les consommer ? Même des choses un peu plus rares. Si on prend un légume que vous consommez régulièrement, on peut prendre… je ne sais pas ? La courgette qui arrive… est-ce que… comment vous la consommez, la courgette ?**

Après je fais plein de choses avec les légumes… je fais des poêlés. Oui, je fais des grattins, je fais des quiches aux légumes, des quiches, tartes aux légumes. Je fais des salades, des soupes. Je pense que tout y passe. J'aime bien cuisiner indien aussi, donc je fais pas mal de, dhal… de préparations avec pas mal d'épices dans les légumes… avec des sources… des noix de cajou, du lait de coco… enfin alors là, je suis très créative sur les légumes ! C’est vrai que les fruits, je sais pas pourquoi ! *(riers)* Mais avec les légumes, je pense que je fais à peu près tout ce qu'on peut faire

**D’accord, et ces idées recettes, vous les avez comment ? Est-ce que vous allez les rechercher ou c’est des choses que vous avez depuis longtemps ?**

Non je les ai recherché ouais

**Ouais** ?

Mmh. Je les ai recherchés ouais. Puis j'ai un peu voyagé, donc j'ai vu autre chose. Et puis, je suis une grande fan de cuisine indienne et j'adore ça !

**C’est très bon !**

*(rires)* Je me lance !

**Bon, c'est rarement aussi bon que là-bas, mais…**

Mais… c’est un peu moins épicé, donc on survit plus facilement, parce qu’en Thaïlande… J’ai pas été en Inde moi personnellement, mon frère l’a fait. Mais en Thaïlande, il y a des fois c’est des… Pfiou !

**Moi, j'y étais et ouais c'était... Pfiou ! Mais après, on s'y habitue et limite, on n'est pas content. Quand ils voient les petits blancs arriver, ils se disent « oh on va pas les épicer » et là on est devant le plat et « mais ça pique pas » ! *(rires)***

Oui c’est vrai qu’il y a des fois, en France quand je vais dans les restaurants. L'autre jour, je suis allée dans un restaurant thaï et il me dit « attention, c'est épicé ». Je lui dis oui, mais justement ! Cétait nul. C'était pas du tout épicé ! *(rires)* Enfin rien à voir avec ce qu’ils font là-bas, c’est une blague ! Normalement j’aurais du pleurer, être toute rouge et… mais ouais c’est vrai que les légumes moi, je fais plein plein de choses avec, mais je vais beaucoup chercher sur Internet, ça c’est clair ouais, des idées… j’aime bien !

**Et, est ce que ça vous est déjà arrivé d'acheter justement un légume et vous saviez même pas trop ce que c'est… Et après, justement, aller regarder ce qu’on peut en faire..**

Ouais, bah les fanes, c'est comme ça, par exemple. Je me suis dit il y a des gens qui les consomment... Je vais voir comment on fait.. le chou rave, je connaissais pas trop, mais j'ai gardé…

**Donc ouais, c’est pratique courante de se dire comment je vais cuisiner et du coup chercher sur internet.**

Ouais ouais.

**Et du coup là on a parlé de la courgette, mais au final, tous les légumes vous allez les cuisiner un peu… poêlés, etc.**

Il y a plein de trucs à faire… Crus cuits…

**Oui, ça vous arrive de faire des salades crues aussi avec les légumes que vous achetez ?**

Mmh Mmh, je fais beaucoup de salades, plus l'été soupe l'hiver. Après je fais plein de trucs hein, caviar d'aubergine au four, des choses comme ça..

**Ok… Alors là, c'est une question un peu bizarre, mais en fait, nous, on se pose la question de savoir s'il y a des légumes qu'on va pouvoir consommer et sans lequel on n'effectue pas de préparation. Il n'y a pas d'épluchures sur ce légume. Les fruits, ça… Quand on mange cru, il y a quand même souvent de l’épluchures. Mais sur un légume, est-ce que vous arrivez à visualiser un légume que vous pouvez cuisiner comme ça, sans éplucher, sans couper un bout…**

Bah je sais pas les radis

**Les radis vous mangez tout dedans du coup ?**

Bah ouais j'enlève la fane et puis je le mange, clac.

**Et vous mangez les fanes de radis de temps en temps ?**

Ouais en soupe ouais. Plutôt en soupe, parce que c’est un peu raide quand même, ça ne se prête pas trop à la salade. Après, qu'est ce qu’il y a… Bah les tomates si tenté que ce soit un légume, c’est toujours la même question. Bah la courgette ? Ah oui la courgette on coupe quand même les extrémités.

**Ouais, et sinon vous gardez la peau, de la courgette ?**

Ben ouais ouais… Qu'est ce qu'on mange comme ça ? C'est une bonne question ça ! Les carottes ! Les carottes nouvelles, j’enlève pas forcément la peau, je les croque comme ça, après lavage *(rires)*

**Vous les nettoyez d’abord dans ces cas là ?**

Ouais ouais quand même ouais.

**Et la courgette, aussi, vous la lavez aussi si vous gardez la peau ?**

Oui je lave quand même ouais.

**Les radis aussi ?**

Oui, oui. Ben oui, parce qu'après des légumes, qu'on puisse manger directement comme ça, c’est pas évident hein… C’est assez proche de la terre en général.

**Les pommes de terre aussi, est-ce que vous consommez la peau ou pas ?**

Rarement, mais ça peut arriver, si je les cuits au four ouais.

**Ca dépend de la recette ?**

Ca dépend plus de la recette ouais…

**Et les fruits, par exemple la pomme, vous avez tendance à éplucher la peau ?**

Je mange tout. C’est bio alors je me tracasse pas trop. Bah je les rince hein... Non, mais c'est pareil, moi la pomme je mange tout hein, il reste que la queue. Je mange les pépins, je mange la pomme, la poire aussi en général. Il y a plein de bonnes choses dans les pépins *(rires)* Après les gens achètent des compléments alimentaires aux pépins de pommes ! *(rires)*

**Oui, c’est vrai ! Donc vous, vous avez tendance à consommer tout le produit, au maximum quoi.**

Ouais ouais carrément ouais. Bon la banane je mange pas la peau quand même, il y a quand même des limites, celle des agrumes non plus.

**Ouais les agrumes ouais. Mais il paraît que… quelqu'un m'avait parlé d'une consommation de peau de banane, mais sous quelle forme ?**

Grillée peut-être ?

**Je ne sais plus, il faudrait que je retrouve ce que… parce que la personne ne faisait pas, mais elle avait entendu une recette, comme quoi c’était possible. C’est vrai que justement… la question de est-ce qu’à la base, tout est bon en soit, enfin tout est comestible..**

Ah oui, ça va pas nous rendre malade, je pense pas… c'est particulièrement désagréable la peau de banane quand même hein ! *(rires).* Ca ne me viendrait pas trop à idée et là pour le coup, on sait qu'elles sont un peu plus traitées. Mais après, il y a plein de bonnes choses aussi dans les vitamines.. Enfin il y a plein de vitamines dans la peau des fruits, donc c'est un peu bête. Et puis ben chez moi, ça m'énerve pas. On achète un fruit, puis on enlève l'intérieur, on enlève l'extérieur, enfin il reste plus rien.. Je trouve ça un peu con en fait ! *(rires)*

**Complètement !**

Mais après, je conçois, si on achète du conventionnel… D'ailleurs, moi, je la mange pas la pomme. Quand on sait le nombre de traitements qu’il y a sur une pomme…

**Ouais, si un jour vous vous retrouvez en pique-nique en formation, et que vous avez une pomme qui n'est pas bio, vous n’allez pas la consommer ?**

Je ne pense pas, non. Je ne suis pas complètement suicidaire. Mais en moyenne, il y a une quarantaine de traitements quand même sur une pomme. Donc euh… sur toute la saison. Donc bon… enlever la peau moi je veux bien, mais je pense qu'à un moment, ça doit rentrer dans le fruit. Mais à chaque fois qu'il pleut, ils sont obligés de remettre tout ce qui est fongicide et tout ça parce que ça a lavé les fruits, donc ils remettent une couche dessus quoi… c'est dramatique la pomme…. Je pense que… je sais pas la poire mais on doit être dans la même chose à peu près ouais. Les oranges, c’est un drame aussi ouais. Donc voilà, mais quand c'est bio, ou quand ça vient de l’arbre oui, je mange tout ouais.

**C’est ça, c'est le bio qui va faire la différence sur euh…**

Ouais bah après je sais bien que c’est pas à 100% mais de toute façon vu ce qu’on respire… A un moment donné, c'est compliqué, tout ça c'est très compliqué ! *(rires)*

**Oui oui ! Ok et là si on échange sur le moment où ça vous arrive de jeter un fruit ou un légume, ou bien une partie seulement… Quand ça arrive pourquoi décidez-vous de le jeter plutôt que de le consommer ?**

Ah bah typiquement, c’est quand c’est pourri quoi. Alors, on voit bien les poires, les pommes, il y a des fois, il y a un point noir qui se met. Et puis ça se creuse. C'est quand même pas bon, clairement. Euh… et les agrumes bon bah, quand elles sont complètement moisies, ce n'est plus la peine de les manger. Après, dans la mesure du possible, j'essaie de couper que la partie abîmée. Mais bon c’est vrai qu’il y a des fruits comme la poire. En général quand la poire s'abîme… C'est vrai, c'est dégueulasse quoi, clairement.

**Du coup, comment vous déterminez qu’un fruit et légume est pourri. Qu’est-ce qui va venir vous…**

Oh bah ça se voit, c’est tout mou, c’est tâché… Ou quand on l'ouvre, ça arrive avec les poires, tu les ouvres, c'est noir à l'intérieur, mais c'est la couleur, puis même le goût, on s’en rend très très vite compte… Il y a des fois, on croit que ça passe et ça passe plus.

**Ouais… Et du coup, vous le jetez parce que c'est pourri, admettons vous enlevez le bout, c’est pourri et c'est pas bon niveau du goût. Est ce que selon vous il y a une quelconque… est ce qu'il y a un risque pour la santé de manger du pourri ?**

Ah non.

**Ou… c’est exclusivement par goût que**

Ouais non c’est parce que c’est le goût ouais franchement c’est pas bon, c’est comme un vieux kiwi c’est… franchement c'est pas bon. Non non pour la santé, il y a aucun risque. Je pense pas, je me suis jamais posée la question.

**Je ne sais pas… je me pose la question de savoir pourquoi est-ce qu'on jette.**

Non, je ne pense pas qu'il y ait un risque pour la santé, mais c'est vrai que c’est hyper désagréable.

**Et du coup, quand vous êtes face à du pourri, qu'est ce que vous ressentez ? Qu’est-ce que vous faites pour vous avez là quelque chose de pourri dans les mains ?**

Bah rien de particulier en fait *(rires).* Je me dis « merde j’aurais du faire gaffe », c’est tout. Par exemple, je sais que les pommes font mûrir très, très vite les autres fruits et légumes donc il faut les mettre à part, jamais dans la même corbeille sinon c’est catastrophe assurée. Mais non, ça me fait chier quoi. Je me dis merde, voilà, c'est tout. Après moi, je ne suis pas repoussée par le pourri. En fait, je n'ai pas de réaction. J'avais une collègue qui était phobique des poils de pêche. Je n'avais jamais vu ça de ma vie, je sais qu'il y a des gens qu'ont est phobique comme ça, avec certaines choses, c’est pas du tout mon cas. Ça ne me fait pas de réaction particulière. Je ne m'inquiète pas du tout pour ma santé. J'enlève juste le morceau qui n’est pas bon quoi.. mais c'est vrai que ça m'ennuie parce que justement, j'essaie d'être raisonnable dans mes achats et de faire ça régulièrement pour éviter. Donc c’est vrai je me dis merde quoi, voilà. Après c’est pas la fin du monde non plus hein.

**Non… Ok donc c'est vraiment le goût qui n'est pas bon, du coup, tant pis, même si c'est toujours embêtant de jeter. On enlève le bout qui est pas bon et**

Ouais voilà, c'est toujours embêtant parce que j'ai été élevé comme ça, on jetait jamais rien.

**Ouais… Et vous avez dit que la pomme, vous la mettez à part**

Ouais les pommes, je les mets toujours à part ouais.

**Est ce qu'il y en a d'autres comme ça ? Des fruits que vous allez un peu positionner d’une façon stratégique ou il n'y a que la pomme que…**

Ben’ les agrumes au frigo ouais...

**Ouais, c’est ça que vous disiez…**

Les fraises aussi quand même… Après non, c'est vraiment la pomme parce qu'on sait qu'elle fait vraiment mûrir les autres trop vite. Donc c'est vrai qu'après, c'est tenter le diable aussi de mettre dans sa corbeille tout en même temps, mais… Après si c'est un peu stratégique quand même. C’est que quand arrive l'été, qu'on achète des pêches ou des choses comme ça, on évite de les écraser, ne pas les mettre au fond de la corbeille avec des trucs bien lourds au dessus… Voilà il faut faire attention un peu quand même. Quand je fais mon petit marché, je fais attention ! J’achète d'abord les trucs pas trop fragiles, que j'arrive pas avec des pêches qui 0sont déjà en bouillie ou des abricots dans le fond du cabas. Je suis sûr qu'il y a plein de gens qui gaspillent tout bêtement comme ça parce que ça supporte pas vraiment les chocs ce genre de fruits.

**Non, oui non pas du tout. Et puis, une fois que ça a pris un choc après, ça va pourir très vite.**

Ah oui ça va hyper vite ouais.

**Il vaut mieux commencer par des choses lourdes et solides que par ses pêches !**

Les patates d’abord

***(rires)* C’est ça ! Très bien alors là, si l'on discute du gaspillage alimentaire de façon assez générale, quand je vous dis gaspillage alimentaire, c'est quoi les mots qui vous viennent comme ça, spontanément à l’esprit ?**

Ca m’énerve forcément, vous vous doutez bien à m’écouter depuis une demi-heure. Ça me rend malade, ça me rend malade. Mais oui, c'est vraiment le sujet qui m'énerve particulièrement. Moi, je fais de la plongée sous marine, je suis très attachée à la mer et s'il y a bien un gaspillage alimentaire que je ne supporte pas, c'est le chalutage. Il y a 20% de ce qui est pêché qui est vendu… qui est vendu ! Et dans les 20% qui sont vendus, je ne vous explique pas tout ce qui perd en plus à la poubelle, mais c'est vraiment le truc qui me rend complètement dingue, ça me rend complètement dingue ! Et d'ailleurs, je ne mange pas de poisson.

**A cause de ça ?**

Ouais, sauf si je depêche moi même et que je le mange mais… J'achèterai jamais du poisson par un pêcheur. Non, le gaspillage alimentaire, c'est complètement dingue parce qu'il y a plein de gens qui n’ont pas à manger quoi. Et ouais non, je ne supporte pas ça. Je ne supporte pas ça et ça serait bien qu'on éduque un petit peu plus les gens, je sais pas... Je crois que maintenant, les grandes surfaces donnent beaucoup quand même, mais par exemple les DLC, ça, c'est un truc qui m'énerve énormément les dates limites de consommation, parce que la plupart des gens sont persuadés que c'est la loi qui oblige les entreprises à le faire. C'est complètement faux !

**D'accord**…

C'est en fait les entreprises qui sont tenues à un résultat. La loi leur dit vous ne rendait pas les gens malades. En gros, prouvez nous que vous n'allez pas le faire. C'est comme avec les vaccins et toutes ces conneries, bref, c'est à eux prouver qu'ils font bien donc évidemment ils vont pas dire qu'ils font mal, c’est la base. Et donc le problème, c'est qu'ils mettent des DLC qui sont complètement incohérentes. Maintenant, ça se démocratise sur le yaourt. Les gens le savent, mais moi, c'est pareil. Ça fait longtemps que je le sais  vu que c’est mes études. Je mange d'ailleurs des yaourts périmés depuis un mois et demi. J'avais tous mes potes qui me regardent avec des yeux « tu vas mourir » *(rires)* je ne vais pas mourir avec un yaourt. Mais il y a beaucoup d'autres choses comme ça et ça, les DLC aussi, les entreprises agroalimentaires les mettent très courte, aussi exprès, parce qu'ils savent très bien que les gens vont faire un gros plein de courses le samedi, tout borurer dans le frigo. Et puis, à un moment donné, ils regardent les dates et ils jettent parce qu’ils ont peur et 90% je pense de ce qui est jeté est consommable. Et ça en fait c’est pas du tout la loi qui les impose, ces industriels qui font ce qu'ils veulent. Et c'est très drôle. Parce que vous allez sur une chaîne de production, ils vont vous sortir des yaourts qui sont destinés à la métropole avec une DLC. Après, on change parce que ça va partir à la Réunion et ils rallongent la DLC. C’est le même yaourt ! C'est juste parce que voilà, il y aura le temps de trajet, donc le temps que ça arrive là bas, on pousse un peu… mais ils savent très bien que leurs produits, de toute façon, on peut encore les consommer après. Ils prennent une marge de sécurité et c'est même plus une marge de sécurité. C'est purement commercial.

**C'est à la base, ça partait d'une intention de se déresponsabiliser, d'assurer ses arrières et donc mettre une date très courte par rapport à la réalité pour être certain. Sauf qu’après, effectivement, il y a même un avantage commercial à dire que s’ils jettent, ils vont racheter.**

Et bien exactement ! Donc non, le gaspillage alimentaire, c'est un carnage, sans parler des restaurants, des cantines….

**Oui, les collectivités**

Parce que les cantines, moi je me rappelle quand j'étais gamine, je ne sais pas si ça a changé, mais c'est purement immangeable. Franchement, personne ne bouffe rien, donc c'est jeté. Mais j'ose même pas imaginer les quantités de nourriture qui partent dans chaque cantine

**C’est vrai.. Mais moi ce qui me… Encore dernièrement je devais manger dans un… je travaillais dans un lycée, donc un self, et j’avais beau demander un petit peu, un petit peu. C'est pas nous qui nous servons.**

Oui, en plus !

**Et c'est ça, c'est ça. Je pense que si même les jeunes qui se servaient eux-mêmes, bon après il y a eu le covid et tout, mais il y aurait beaucoup moins de gaspillage. Je ne sais pas pourquoi ils nous mettent des tonnes et des tonnes dans l'assiette, si on avait pas envie, j’en voulais qu’un petit peu quoi, le choufleur tout mou là, j’en voulais qu’un !**

Oui bah dans les restos, c'est chiant aussi. Parce que c'est vrai que parfois, il y a des quantités euh…

**Bah c’est ça… Je pense que dans les restaurants gastronomiques, il ne perdent pas beaucoup *(rires)* !**

Oui bah oui, et puis vu le prix que ça coûte, on mange tout ! *(rires)* Mais c’est vrai que par exemple il y a des pizzéria qui réagissent bien par rapport à ça en proposant des tailles de pizza. Moi je trouve ça vraiment chouette parce que du coup, un peu moins cher, et puis au moins je ne vais pas en laisser la moitié dans l'assiette. Et moi, je demande beaucoup, beaucoup à emporter. Maintenant, quand je finis par mes assiettes dans les restos. Il y a des fois on me regarde bizarrement mais bon, c’est pas grave !

**Je crois qu’ils sont dans l'obligation maintenant de donner aux gens les restes, si les gens veulent l'emporte**

Parce que c'est vrai que c'est fou, c'est fou. Et puis ce que je ne comprends pas, en plus, dans le gaspillage alimentaire ou encore l'agro-alimentaire je les comprends, ils veulent faire du fric, le capitalisme à fond mais voilà *(rires)* . Mais l'individu, les gens, il y en a plein qui jettent et ça ne leur pose pas de problème. Et ça, je ne peux pas. Moi, je ne peux pas, même un bout de pain. Tant pis, je vais faire autre chose avec mon pain. Du pain perdu ou je ne sais pas, mais je vais pas jeter mon morceau de pain. Je ne comprends pas et j'ai l'impression… Enfin, moi, forcément, on a toujours des amis qui nous ressemblent donc j’ai quand même pas mal d'amis qui sont un peu dans mon genre, mais j'ai l'impression qu'il y a beaucoup de gens qui jettent super facilement la nourriture

**C’est possible oui… sans faire les choses, consciemment, sans voir consciemment ce que ça représente**

Ouais non mais voilà, ils regardent dans le frigo. J'avais acheté ça, c'est périmé. Pouf, poubelle ! Ok… Moi, ça me choque. Mais c'est vrai que c'est un carnage le gaspillage alimentaire, ouais. Mais surtout qu'après. Toujours dans cet esprit, je sais pas si vous connaissez la collapsologie hein.. Je suis vraiment à fond. Et du coup, depuis très, très… bien avant qu'ils inventent le mot, mais moi, je n'ai jamais compris parce que c'est tout un système : c'est du carburant pour transporter ces aliments, pour les produire, sans parler quand on jette de la viande, alors là c'est l'horreur de l'horreur. Les litres d'eau, les litres de soja importés, la forêt qu’on déforeste… et les mecs, pouf c’est périmé, je jette ! Bon… ok, mais après sinon moi, si je laisse couler mon robinet quand je me lave les dents, limite on va bientôt me foutre une taxe parce que je pollue ! *(rires)*. Non mais c’est ça, à un moment donné, ça me rend complètement dingue parce qu’on nous culpabilise à mort sur tous nos choix et à côté de ça, au niveau alimentaire, c'est n'importe quoi.

**C'est ça aussi. Je pense qu'il y a de ça, en fait. Je vais pas dire que les gens sont pas compétents, mais vous, vous avez des connaissances sur le sujet et ça vous intéresse et du coup, vous savez ce que c'est, un un kilo de viande, toutes les ressources que ça demande. Moi, j'ai regardé des reportages, je suis devenue végétarienne parce que ça m'avait pout *(son de bouche)* et ma mère l'était, donc c'était éducationnel. Après moi, j'ai été chercher, je sais. Mais j'ai l'impression qu'il y a des gens qui ne savent pas et qu'on leur dit, l’écologie, effectivement c'est couper.. couper l'eau quand on se brosse les dents, c'est ça alors que non, c'est minime. Mais je pense que**

La culture, c'est un truc de dingue hein !

**Et je pense qu'on cache pas mal cette info**

Oui, bien sûr, c'est comme pour le numérique.

**Oui, c’est pareil, les mails et tout**

Parce que moi, au départ, c'est pareil. Mais je passe toujours pour une allumée. Mais quand j'étais plus jeune, quand on a commencé à nous dire on arrête le papier, on fait du tout numérique numérique euh l’électricité, il faut pas mieux des forêts autogérées ? Gérer correctement, je ne vois pas trop est le problème avec mon papier en fait. On commence à en parler de data center. Mais ça fait longtemps, ils font chier avec leur papier. Moi, je continue à écrire sur du papier, je passe pour un dinosaure aussi auprès de mes collègues « il faut tout faire sur ordinateur », vous me faites chier donnez moi un cahier, j’écris sur mon cahier ! *(rires)* Déjà parce que moi j’ai du mal un peu en fait à m'habituer, mais il y a plein de choses comme ça qu'on nous cache. La pollution, c'est vrai de la circulation, ça ne va pas. Effectivement, on utilise tous une voiture. On est souvent tout seul dedans, mais la pollution faite par les engins agricoles et le transport des marchandises, c’est un truc de malade et j’avais vu ça en écoles d'agroalimentaire. J'ai 43 ans, donc c’était il y a quand même 20 ans. On a fait le trajet d'une margarine. Non mais une margarine, elle fait 15 pays avant d’arriver au supermarché entre les huiles qu’ils mettent dedans, l'usine qui transforme, l'usine qui met dans la boite, la fabrication de la boîte… une quinzaine de pays, super ! Et en plus, il y en a qui jete après ! *(rires)* Non mais voilà le délire. Et c'est vrai qu’en fait... Ouais, mais c'est pareil, les bateaux de pêche.

Tout ça, c'est une pure folie. C'est une pure folie la pêche en terme de ressources de tout ordre. C'est horrible. Il faut arrêter de manger du poisson, quand je vois des gens manger du poisson, je leur explique à chaque fois. Et puis bah, la viande, c’est vrai qu'il y a beaucoup d'aliments qui sont importés. C'est un truc de malade. Et puis, c'est fou parce que les gens qui font de la viande allaitante, les jeunes veaux, sont pas… Alors il y en qui sont élevés en France, mais il y en a énormément qui partent sur l'Italie où ils sont engraissés. Ils sont engraissés en Italie, il y a un espèce de circuit aussi des animaux comme ça, on les fait naître ici, on les engraisse là, on les abat là… enfin c’est n’importe quoi… Et dans tous les domaines de l'agro alimentaire, quelque soit ceux qu’on prend c'est tout est comme ça, ça circule, ça voyage de partout.

**C'est là qu'il y a un souci et en plus, je pense qu'ils se font une joie de le cacher et de l’enfouir pour pas que**

Bah oui ! L'extraction des engrais, les phosphates, tout ça se fait pas en France hein, il y en a beaucoup qui se font au Maghreb. Enfin c’est un truc de dingue aussi les engrais, le broyage des engrais, le coût énergétique des engrais alors qu'on peut faire du sans labour et du bio, ça pousse tout seul… mais non… trop facile ! *(rires)*

**C’est ça ! Mais des fois on se demande. Car vu comme ça, c'est vrai que c'est quand même plus simple de faire localement, d'utiliser et ne pas**

Oui puis ça marche bien, en plus !

**Pourquoi se compliquer…**

Mais c'est vrai que le domaine de l'agro alimentaire, j'aimerais bien… On n'a pas de chiffres, c’est hyper caché. C'est une vraie mafia. C'est un truc de fou l’agroalimentaire.

**Bien sûr, il y avait un reportage qui commence à dater. C'était Cowspiracy, c’est comme Seaspiracy qui vient de sortir aussi. Dans cowspiracy, il disait, le jeune qui se sentait écolo dans l'âme et qui faisait tout ce qu'on lui demandait de faire et qu’en fait a découvert que la production ; etc. tout ce que ça coûtait, il a quand même contacté toutes les grandes ONG écolos et personne n'a voulu lui répondre sur la question. Jamais Greenpeace, etc. disaient que c'était finalement l'élevage qui allait.. enfin toute la production qui allait polluer le plus. Et il a fait je ne sais combien d'appels pour essayer de les confronter à ça ? Et c'était tu quoi. Donc il y a bien derrière, quelqu'un qui fait bien en sorte que l'on ne sache pas quoi.**

Ah oui oui mais c'est terrible l'agro alimentaire. Mais je pense qu’il y a pas que ça ; moi c’est parce que je connais

**Oui, c'est ça. J'ai tendance à dire à penser que ça se… m'enfin, les jeunes générations… bon après c’est pareil, on a les amis**

Qu’on mérite ! *(rires)* Enfin qu’on espère mériter !

**(rires) C’est ça ! Du coup, on n'est pas objectif sur la question, mais j’ai l’impression que l'information circule plus. Malgré tous les défauts que peut avoir le numérique, etc. Internet…**

Ah oui ça fait circuler l’information oui, tout à fait !

**Il y a des sources d'information qui sont différentes. Avant voilà, il y a avait cinq sources d'information et c’était la vérité absolue.**

Oui, c'est vrai que sur ce côté-là.

**Là, ça circule beaucoup plus.**

Mais c'est comme le recyclage des déchets, on s'oblige à recycler, moi j'étais en Malaisie, nos déchets vont là-bas quoi !

**Oui ! Ca c’est pareil, ça a fuité.**

On voit les décharges

**Oui, on voit la petite bouteille de lait, Bio Français… mais pourquoi ?**

Mais tout est comme ça, c'est incroyable. Moi, quand j'habitais en Normandie, il y avait une usine de recyclage de batteries, piles, batteries qui s'étaient installée donc ils ont touché des subventions de dingue. Cinq ans après, le mec s'est barré, on l'a jamais retrouvé. Ils ont découvert une fosse et tout était dans la fosse. Rien n'avait jamais été recyclé. Je sais pas, ça a dû tenir 5 ans un truc comme ça et puis il s’est barré avec le pognon. C’est vrai que voilà… *(rires)*

**Oui ! Mais que faire face à *(rires)***

Mais dans l'agroalimentaire, c'est vrai qu'il y a aussi euh.. quand un lot est pourri on le mélange avec un autre, les épices, on a un lot qui arrive moisie, on le met sous UV pour détruire les germes. On mixe tout ça. Toutes les.. toutes les petites poudres, comme celles qu'on achète, j’en ai un petit peu quand même… Faut voir ce qu’il y a dedans hein…

**Même les bio et tout ou ?**

J'ai essayé de prendre du bio, mais j'espère que… je me dis c'est moins pire mais… c'est pas forcément mieux. Mais voilà, tout est mélangé et ça repart. La viande est périmée, c’est pas grave, on la trempe un peu dans un truc machin et hop on a la remet… enfin… Le gaspillage alimentaire, c'est une folie et puis tout ce qui est fait aussi derrière pour qu'ils augmentent leurs marges. C'est une pure folie aussi. Et ça, c'est super, c'est super secret effectivement ! C'est difficile de s'attaquer à l'agroalimentaire. J'ai lu une BD récemment sur les algues vertes en Bretagne.

**Oui, je ne l'ai pas lu, mais il parait qu’elle est top.**

Ah bah faut avoir le moral hein ! ah bah je l’ai lu en deux fois après j’ai fait une déprim’ pendant une semaine *(rires)* , j’ai fait houuuuu *(onomatopé)* déjà je savais beaucoup de choses... J'ai lu le livre noir de l'agriculture aussi, de Saporta. Je crois que c'est comme ça qu'elle s'appelle. Alors là c’est pareil, il a fallu que je fasse chapitre par chapitre après on se regarde un petit truc bien bête, un Mr. Bean histoire de décompresser 5 minutes. C'est horrible, mais elles ont eu énormément de problèmes ces deux femmes journalistes. C’est hallucinant les menaces qu’elles ont reçues, c'est hallucinant. Surtout pas en parler, surtout pas y toucher, surtout en Bretagne l’agroalimentaire…

**Ah bah oui.**

Ah oui ! *(rires)* On va quand même pas dire que ça pollue.

**On va vire de quoi après ? Oui c’est un… C’est très large.**

C'est très large ! Moi, j'aimerais bien que tout le monde se mette à consommer local. J'ai l'impression qu'il y a effectivement pas grand monde qui se préoccupe tant que ça du problème. Il y a un manque d'information, d'éducation. Après les gens peuvent pas être éduqués sur tout non plus. C'est un problème. Moi, je dis c'est sur l’agroalimentaire mais ça se trouve je suis complètement nul sur d’autres trucs où j’ai aucune idée de ce qui se passe derrière. Très certainement.

**Oui**.

Mais c'est vrai qu'il y a pas trop de sensibilité je pense, globalement, les gens se rendent pas compte de tout ce qui est derrière. J'ai même un collègue l’autre jour, mais c’est quand même dingue, moi j'ai une télé ici parce que je vais le remettre en Airbnb mais moi je regarde jamais la télé. Mais il a vu un reportage, je ne sais plus sur quelle chaîne, il a découvert pourquoi il y avait de déforestation en Amazonie. Mais sans déconner, tu bosses dans l’agriculture et tu t'es jamais demandé d’où ça venait ?

**Oui en plus, avoir quelqu’un qui travaille**

Les trucs, qu’il donne à bouffer à leurs vaches enfin.. On fait… Enfin sur l'alimentation des vaches… C'est plus pratique, alors on leur donne le l'ensilage de maïs, c'est bourré de sucres, c'est super bien ça coute une fortune déjà un ensilage de maïs, c'est un carnage en termes de mécanisation et de coûts de polluant, enfin de carburant. Un carnage, mais c’est pratique donc on fait comme ça. Du coup, la vache, forcément elle est pas en forme, si elle mange que du sucre, donc on lui refile du soja pour qu'elle ait des protéines. Comme on fait plus pousser de protéines végétales en France, très peu, et bien on va les chercher au Brésil. On les foutrait dans un champ avec de l'herbe, elles seraient en pleine forme, les vaches ! *(rires)* Bah non mais ! Une vache, ça bouffe de l’herbe quoi. Là on s’est mit à leur faire bouffer d'autres trucs qui les détraquent donc après on leur fait manger un autre truc pour les remettre, on leur file un peu d’antibio’, un peu de truc, un peu de machin. Puis la vache elle va comme elle peut, elle vit 5 ans puis on l’abat quoi. Alors que normalement, la vache, elle est pénarde 20 ans dans son champ, tout va bien quoi ! Il y a des producteurs de lait bio qui donnent quasiment aucun aliment industriel à leurs vaches et elles mangent de l’herbe. Une vache quoi ! *(rires)*

**Oui c’est ça, à l’époque ils avaient une vache, elle mangeait de l’herbe…**

C'est complètement dingue, surtout quand on est dans des régions comme les nôtres, où le climat est tempéré, on peut faire pousser à peu près ce qu'on veut. On peut avoir du lait, des animaux, de manière naturelle. Mais évidemment, j'imagine bien que dans certains pays, ils sont un peu obligés d'irriguer, un peu obligés d'acheter des aliments. Mais Bretagne, Normandie, qu’est-ce que… C'est quoi le délire ?

**C’est clair. Oui dans le sud encore, les herbes deviennent toutes sèches. 1’13**

Ben voilà, ils ont des brebis et des chèvres et ils font pas de vache quoi. Il faut être cohérent.

**Oui, comme tout, ça revient… la viande peut être locale ausso**

Oui.. C'est un vaste débat et on est mal barré ! *(rires)*

***(rires)* Conclusion : on est mal, on est mal ! Et du coup, si on fait une.., si on tente une définition du gaspillage alimentaire. Si vous deviez écrire dans un dictionnaire après gaspillage alimentaire, qu'est ce que vous y mettrez comme définition ? Avec vos propres mots.**

Gaspillage alimentaire ? Bah, pour moi, ça serait l'action de jeter un produit consommable, en fait.

**Effectivement**

Bah ouais, et c'est complètement con ! *(rires)*

***(rires)* Alors, exemples ! Après, c’est la question de quand vous vous jetez vos fruits et légumes, qu'est ce que vous ressentez ? Mais ça on a déjà… J'ai la réponse !**

Je le vis mal ! *(rires)*

***(rires)* C’est ça, pour être polie, je le vis pas bien ! Et pour terminer sur la poubelle connectée, du coup vous l'avez depuis quelques jours. Et comment ça se passe ? Vous avez le sentiment de l'utiliser beaucoup à vous ?**

J’ai jeté deux trucs dedans, mais je suis pas fière !

**Ouais ? C’est deux de trop ! *(rires)***

Ouais non non, j'ai un citron qui m'a fait une blague là, qui a pourri en 2 jours. Et puis, mes radis là qui n'ont pas tenu le temps de la formation. Je pensais que ça allait le faire, mais ils ont pas… voilà.

**Et donc, vous m’avez dit pourquoi vous les avait jeté… Donc le citron c’était en 2-3 jours que pout’ *(son de bouche)* qu’il est parti…**

Oui il est parti très vite lui !

**Il est parti… et les radis il y a eu la formation qui a fait que**

Bah ouais c’est ça, c’est vrai que je pars à l’hôtel sur 2-3 jours. Du coup, ça m'a cassé dans mon élan d’organisation.

**Et est ce que vous avez le sentiment que…. Enfin est-ce que vous avez des fruits et légumes où vous avez le sentiment que vous allez les perdre plus facilement que d'autres ou pas ? Sachant que vous jetez pas beaucoup, mais quand vous vous dites qu'il y a des choses qui sont plus difficiles à…**

Oui clairement, il y a des choses beaucoup plus difficiles. C'est vrai qu'on parle de pêche, des abricots, c’est des fruits qui peuvent vite s'abîmer… les poires, ça dépend, mais ça peut aller vite. Les agrumes aussi. Les légumes… euh pff bah les blettes typiquement. Les blettes, il faut les cuisiner tout de suite parce que j'ai essayé de les mettre dans des pots d'eau et même pas hein, ça tient 24 heures et c’est foutu ! Ca s’abime super vite, c’est incroyable comment ça fane très très vite. Les épinards sont un peu moins chiant la dessus. Ouais, ouais. Ben, il y a beaucoup de choses dans les produits frais quaund même.

**Liée à la fragilité du fruit ou légume, quoi ?**

Ben ouais, surtout en bio, ça va encore plus vite. C’est vrai que les tomates conventionnelles ça pourri jamais quoi… Sérieux c’est un truc de fou ! Mais c'est vrai qu'il y a quand même, des légumes et des fruits qui sont assez simples à conserver. Les pommes de terre, les carottes, ça se garde très très bien, très longtemps. Après, j'ai des Tupperware aussi avec des systèmes d'ouverture là, c'est génial pour conserver les légumes.

**Quelqu'un m'a montré, j’étais chez quelqu'un et il m’a montré, il a, selon les fruits et légumes**

Voilà, il y a des schémas. En fait, on ouvre ou on ferme et c’est incroyable.

**Ah oui ?**

C'est incroyable, la salade, on peut la garder super longtemps. C'est fou, ça marche super bien. Je ne sais pas ce qu'ils ont fait dans le plastique du coup… *(rires)* C'est un autre débat, on refera une thèse là-dessus *(rires)* Mais en tous les cas, c'est bluffant. C'est ma grand mère qui m'avait filé ça et c'est bluffant.

**Et c'est l'ouverture qui fait… En fait c’est l’air qu’on laisse**

Faut croire ouais, qu’avec moins d’air ça s’oxyde peut-être moins ou je sais pas…

**Alors que d'autres ont besoin peut-être de plus d’air…**

Mais c'est bluffant, notamment sur la salade ouais ! C'est incroyable ! Parce que c’est vrai que les salades c’est un petit peu chiant aussi quand on achète une salade… Pff faut être nombreux quoi ! Sinon c’est pas pratique..

**Et ça se perd très vite. Et donc la salade, vous la préparez et, vous la mettez dans un tupperware?**

Ouais, ben oui, du coup, c’est top. Après, c'est vrai qu'ils font beaucoup sur les marchés, ça j’apprécie ils font beaucoup de mélanges de salades, ce qui fait que ça permet, plutôt que d'acheter une énorme salade, d'acheter juste un petit peu aussi.

**Ça, c'est bien.**

Après sinon on a le sachet plastique de salade dans les grandes surfaces, mais bon là j'évite ça, mais je comprends aussi un peu que les gens se tournent vers ça parce que c'est typiquement le genre de produit qui est un peu pénible quand même parce que ça se perd hyper vite.

**Mais c'est vrai que ça. Je suis en train de me demander pourquoi... Comme le poireau, est ce que vous, vous consommez le vert ou pas ?**

Presque, pas tout.

**Vous allez enlever une partie, mais vous avez également du vert. Il y a des personnes, elles prennent vraiment que le blanc. Alors je sais que dans les grandes surfaces, il propose des fois que blanc. Mais du coup, c’est en sachet, c‘est pas… et je me demande pourquoi on proposerait pas et déjà sur des marchés, que le blanc à certaines personnes qui ne veulent vraiment pas consommer le vert. Mais peut être proposer le vert moins cher pour ceux qui veulent le mettre dans une soupe ou quoi….**

Oula après s’il y a que le vert euh…

**Ah bah oui, après il faut mettre autre chose, plein de patates à côté ou… Ou après, je ne sais pas des structures qui au final…, comme des associations, etc. pourraient récupérer ces bouts là, qu'on ne veut pas, mais qui sont tout à fait consommables pour essayer de faire ce que les**

Ouais… Ou est-ce que les gens peuvent pas, tout simplement essayer de les consommer et d’être moins con ? *(rires)* Pardon mais là !

**Oui, si non, mais là, c'est sûr. Mais il y a des gens qui ne le feront pas. Et du coup, la moitié du poireau part à la poubelle…**

Oui, mais je ne sais pas ce qu'on pourrait en faire parce que moi, je consomme pas mal du vert, mais surtout parce que je fais des soupes.

**Oui, c’est surtout en soupe.**

Après, c'est un peu raide quand même à manger… quand on fait une fondue de poireaux, on privilégie plus la partie blanche, un peu de vert clair, mais pas le bout de la feuille, c'est compliqué. Après moi, quand j'avais un jardin, bientôt à nouveau, je fais mon compost avec donc ça repart dans mon potager donc bon… Et puis je me dis il y a bien d'autres animaux qui viennent grignoter ça, donc je culpabilise moins ! *(rires)*

**Alors, c'est ça le compost, c'est la pratique qui déculpabilise un peu.**

Bah voilà, un petit peu ! Parce que c’est vrai que malgré tout on mange quand même pas tout, il y a quand même un minimum d'épluchage sur différents fruits et légumes. Oui, peut pas non plus… Moi déjà je mange mes pépins de pomme, mais je veux dire il y a un moment donné aussi, où on ne peut pas non plus tout manger ! *(rires)* Donc il ne faut pas non plus se rendre malade avec ça, mais après moi, je n'aime pas trop la solution de proposer du prémâché aux gens parce que je me dis si on veut aller vers une éducation, justement, à la cuisine et à l'alimentation, ça ne va pas dans le bon sens, à mon avis.

**Oui c’est vrai, c'est toute la question, mais moi je suis sûre qu’il y a des gens qui ne se forceront jamais.**

C'est sûr il y en a qui se forceront pas

**A changer, s’ils trouvent le vert pas bon.. ils ne vont pas s’embêter à se dire que si, il faut le préparer quoi, mais c’est comme les fanes du coup, par exemple les carottes, vous avez dit que sur le marché, ils vous le demandent ?**

Ouais ils me demandent, souvent sur les betteraves, moi je leur dis que je les mange et c’est vrai qu’on doit pas être nombreux à le faire parce que l’autre jour le maraîcher il m’a regardé avec des grands yeux *(rires)* Mais oui je ne m'en fou ! *(rires)* Je pense que lui ne le faisait pas.

**Et qu’est-ce qu'ils font des fanes après ?**

Oh bah je pense que ça part dans la petite poubelle qu'on a juste devant là.

**J'ai entendu des interrogés là, des personnes qui me disent, ce que je ne savais pas, qu'ils font du pesto avec les fanes.**

Ah oui on peut faire du pesto aussi c’est vrai, ça j’ai jamais essayé.

**Il parait que c’est pas mal du tout ! On ne mange pas non plus tous les jours, mais de temps en temps.**

Oui, c'est vrai qu'on peut faire des tests

**Et après, je sais pas si on fait plein de pesto qu'on commercialise... *(rires)***

*(rires)* Mais je sais pas. Je sais qu'il y a des produits, une productrice là à côté de Quimper qui fait des pesto, justement. Mais je ne sais pas si c'est avec les fanes, parce que de ce que j'en sais, je n'ai pas encore vu ses…, je n'ai pas encore trouvé de magasin où il y avait ses produits, je la connais par le boulot, en fait, parce que j'ai vu son dossier passer des pestos d’ail des ours, enfin c’est pas du vrai ail des ours, tout le monde l’appelle comme ça dans la région mais c’est pas le vrai *(rires)* mais c’est très bon, j’en mange, je vais en cueillir et je vais cueillir pas mal d’herbes aussi. Mais voilà, je sais qu’elle fait des pestos mais je sais pas si elle fait avec des fanes ouais. C’est vrai que pourrait être une idée ouais.

**Devrait y avoir… mais oui, il faut aussi que le milieu un peu agroalimentaire s'intéresse aussi à ce… mais bon ça..**

Qu’ils emballent pas ça dans du plastique euh… *(rires)*

*(rires)* Ah oui c’est vrai qu’au bout d’un moment, ça va repartir…

Après, je ne sais pas si les gens sont tellement prêts non plus à acheter du pesto de fanes de carottes. Je ne sais pas si le succès en grande surface serait au rendez-vous.

Ou alors que les grands restaurateurs nous montrent des recettes.

Oui, à la télé, peut-être que si c'est un chef qui met ça à la mode. Ça va peut être marcher.

**Et par ailleurs, ça fait longtemps que je n'ai pas regardé ce genre d'émission. Mais ça m'était arrivé de regarder des trucs comme Top Chef Machin là. Eh bien, ça arrive tout le temps, ou très régulièrement, qu’ils demandent de préparer absolument tout le produit. Les restaurateurs sont souvent attachés aux produits et ils vont souvent le cuisiner... Quitte à après utiliser dans des bouillons, des machins. Et c'est assez rigolo, parce que quand il y a les participants, ils leur demandent souvent de veiller à vraiment tout prendre dans le légume, ce qui peut éduquer tous les gens qui regardent aussi à se dire que tout se mange dedans, il faut juste être un peu plus…**

Mais c'est un problème qu’on a aussi avec le bœuf, la vache, le boeuf. Maintenant, les gens mangent du steak haché.

**Ça se résume à ça.**

Mais du coup, on en fait quoi du reste de la bestiole ? *(rires)* Parce que ça coute un bras à produire ! C’est un pur problème. Donc ouais les restes après si, ça part dans les plats préparés des grandes surface mais…

**C'est aussi le travail des bouchers de prendre tous les bouts et de les travailler jusqu'à ce qu'il**

Oui mais faut les cuisiner et c'est qu'il y plein de gens qui savent pas cuisiner plus en fait.

**Il y a une boucherie près de Recouvrance qui s'appelle Delicatessen. Et apparemment, j’avais vu un reportage ? Je n'y vais pas, parce que je mange pas de viande *(rires)* mais apparemment, du coup il prend son prix sur les produits, mais il va vraiment aller cuisiner toutes les parties qu’on**

Ah oui d’accord, mais c’est déjà cuisiné déjà ?

**Oui je crois qu’il prépare. Ils préparent bien déjà les bouts de viande pour qu’ils qui soit prêt, peut être à recuire ou pas, mais il fait tout ce travail-là pour éviter de perdre en fait. Que tout le monde ait son steak et le reste bah… on oublie quoi. Mais effectivement ça demande toujours un travail supplémentaire, c’est comme on disait. Ne pas gaspiller, ça demande un travail supplémentaire, d'organisation, etc. ou de cuisine…**

Puis d'imagination ! C’est vrai que moi je vais chercher des recettes et tout, je trouve vraiment des trucs super sympas à faire mais faut le faire quoi !

**Ouais, complètement. Bon et ben écoutez… ah, juste la poubelle est-ce que ça vous est arrivé de l'oublier ou pas ?**

De l'oublier ?

**C’est-à-dire, bon après vous n'avez pas jeté grand chose, mais de se dire après avoir jeté ailleurs que dans la poubelle, mince.**

Ah non non je fais gaffe non.

**D’accord, super. Et est ce que ça vous est arrivé de vous poser une question du style, est ce que ça, ça doit aller dedans ou pas ?**

Ah non non c’était clair hein. Quand j’épluche les légumes, c’est dans la poubelle normale, c’est que quand c’est pourri quoi.

**C’est ça. C’est ce que d'habitude vous mangez et que là, vous avez décidé de ne pas manger.**

Oui, voilà, c'est ça.

**Bon bah très bien. Et du coup, vous n'avez pas changé de sac encore.**

Non.

**Merci beaucoup. J’ai posé toutes mes questions. Est ce qu'il y a des choses sur lesquelles vous voulez revenir ou des points que vous voulez aborder, que vous pensez que j'ai oublié et que j'aurais dû y penser ?**

*Discussion sur le recrutement des foyers.*

**Household F15, Interview 2**

*Organisatrice:* [Alors] On a un petit matériel, cette fois-ci. [Tac] On va commencer par celui-là. [Alors] Il y a quatre illustrations. Je vais vous demander d'en retourner une au hasard et de me dire spontanément [euh] quels sont les mots ou les expressions qui vous viennent à l'esprit.

*Participante:* Oui, ça marche.

*Participante:* [silence 00:00:28 - 00:00:36]

*Participante:* Épicerie.

*Participante:* [silence 00:00:36 - 00:00:41]

*Organisatrice:* Ouais ?

*Participante:* Lesquelles ? Ce qui peut ne pas l'être du tout d'ailleurs [mais bon].

*Participante:* [silence 00:00:50 - 00:00:56]

*Participante:* Ouais, ça fait magasin de producteurs un peu.

*Organisatrice:* Ouais.

*Participante:* Après... Ça ne doit pas être que du local et du bio, parce qu'il y a des légumes d'hiver et des légumes d'été.

*Participante:* [rires 00:01:13 - 00:01:20]

*Participante:* C'est plutôt conventionnel, on dirait. [Voilà] Sinon c'est vrai que ça-- [enfin] moi, personnellement, ça me fait plutôt envie quand je vois un étalage comme ça.

*Organisatrice:* Ouais.

*Participante:* C'est vrai, c'est bien présenté.

*Organisatrice:* Est-ce que ça ressemble à un lieu que vous pouvez fréquenter ? Ou pas vraiment ?

*Participante:* Non, pas vraiment.

*Organisatrice:* Non ?

*Participante:* Quoique si... [en fait] C'est vrai que je vais de temps en temps au magasin Bio Monde à Daoulas. Ouais, ça ressemble un peu à ça. Ouais.

*Organisatrice:* Et pourquoi vous privilégiez ce magasin-là quand vous y allez ?

*Participante:* Je n'en privilégie pas parce que moi, je fais plein de route pour mon travail, mais en tout cas, je n'achète quasiment que du bio. Du coup, je connais tous les Biocoop, magasin de producteurs. Et quand je passe pas loin pour les clients, j'en profite, je fais un crochet.

*Organisatrice:* C'est plutôt quand c'est sur la route [en fait].

*Participante:* [En fait] Depuis que je ne suis plus à Brest, je n'ai plus du tout d'habitudes [quoi]. C'est n'importe comment.

*Organisatrice:* Oui.

*Participante:* [Enfin] Parce que je ne peux pas rouler tout le temps partout. Et du coup, je m'adapte vraiment [quoi]. C'est-- mon bureau est à Landerneau, donc c'est le Biocoop de Landerneau quand j'ai le temps. Daoulas, il y a un magasin de producteurs aussi où ils font de la viande. Quand j'ai le temps le mercredi... [tchouk] Mais c'est vraiment quand je peux [quoi]. Donc c'est devenu un peu n'importe quoi. Parce que [en fait] c'est en ville qu'on a le plus accès aux choses.

*Organisatrice:* Oui.

*Participante:* Les patates, elles sont produites dans le champ derrière moi, je vais les glaner, mais personne ne veut me les vendre. Il faut que j'aille à perpète pour les retrouver dans un magasin.

*Organisatrice:* Ouais.

*Participante:* Donc oui.

*Organisatrice:* Donc ce n'est pas un magasin dans lequel vous avez forcément l'habitude d'aller, mais ça peut arriver que vous vous retrouviez dans un magasin de ce type-là [quoi].

*Participante:* Ouais, ouais. C'est vrai que les Biocoop-- [enfin] surtout, je pense à celui-là, le Bio Monde, [ouais] ça c'est vraiment très similaire à ça. Effectivement.

*Organisatrice:* OK. Et est-ce que c'est des magasins dans lesquels vous vous sentez bien ? Ou non, c'est quand même contraignant, c'est l'idée de faire ses courses.

*Participante:* Ah oui, non, moi faire les courses... Moi ça me-- oui, ça me casse bien les pieds, mais disons que je survis dans ce genre de magasin. Vous me mettez dans un Leclerc, je fais une dépression. Disons que c'est moins désagréable.

*Organisatrice:* Oui, je vois.

*Participante:* C'est quand même plus humain un peu, c'est quand même moins-- mais même tout [en fait] ! C'est tout qui est différent. Les employés qui n'ont pas l'air au bout du rouleau, c'est [euh] l'éclairage qui n'est pas agressif, il n'y a pas des espèces de pubs sonores dans tous les coins comme il y a maintenant dans les supermarchés.

*Organisatrice:* Oui.

*Participante:* Et puis [bah] je pense quand même qu'il y a-- pour moi, c'est plus de la qualité.

*Organisatrice:* Oui.

*Participante:* C'est beaucoup de choses. Mais-Mais faire les courses, non, ce n'est pas ma passion.

*Organisatrice:* Il y en a pour qui c'est une passion.

*Participante:* Il faudrait qu'ils fassent faire les courses pour moi !

*Organisatrice:* On devrait créer un truc comme ça.

*Participante:* « Vous avez une passion pour les courses, faites-en profiter les autres. »

*Organisatrice:* Je trouve de nombreuses idées. Est-ce que ça vous arrive de jeter des fruits et légumes que vous avez achetés dans ce type de magasins ?

*Participante:* C'est rare, mais oui.

*Organisatrice:* Ça peut arriver ? Et [euh] qu'est-ce que vous ressentez à ce moment-là ? Quand vous perdez des--

*Participante:* Ça me saoule. Non, non, ça me saoule, c'est nul. Mais c'est vrai que parfois... [bah ouais pff] les-- [voilà] ouais, on a acheté un peu trop et puis c'est-c'est ça ou se gaver. [Bon bah] Ça part au compost. Depuis que je suis là, je culpabilise un petit poil moins.

*Organisatrice:* Oui et pourquoi le compost ?

*Participante:* [Bah] Parce qu'après ça repartira dans mon jardin. Donc j'ai moins l'impression d'avoir complètement jeté... Que quand ça partait à Brest dans la poubelle, [bon bah] ça partait à la poubelle [quoi].

*Organisatrice:* Ouais.

*Participante:* Et puis là au pire, il y a des animaux qui vont aller le manger si c'est un peu abîmé.

*Organisatrice:* Ouais.

*Participante:* Parce que tu vois, j'ai des pommiers, je n'ai pas pu tout manger. [Pff] Il y a trop de pommes. [Bon bah] Elles sont mangées par les animaux, [enfin] il n'y a pas de problème [quoi]. Les oiseaux-- tout le monde se régale. Je culpabilise, moins

*Organisatrice:* Oui.

*Participante:* Mais c'est vrai que j'essaye de faire attention. Mais c'est justement un des travers aussi de la campagne. C'est que-- on est obligé-- comme on n'a pas tout au pied-- [enfin] sous la main, [en fait] il faut acheter en plus grosse quantité pour ne pas être constamment en train d'y aller. Et puis [bah] il suffit d'un imprévu, d'être invité à manger chez des potes, n'importe quoi et puis [pouf] on se retrouve avec trop et on perd. Ce n'est pas facile à gérer.

*Organisatrice:* C'est moins facile qu'en ville, effectivement.

*Participante:* [Bah] Ouais, soit on fait plein de kilomètres en voiture et à chaque fois que je brûle un litre de gazole, je m'en veux pour la planète. Non mais-- [en fait] c'est horrible d'avoir une conscience écologique.

*Participante:* Dès qu'on fait un truc, c'est hyper-- on se-- on s'auto-culpabilise, c'est un drame.

*Organisatrice:* Ouais.

*Participante:* Mais c'est vrai que c'est-c'est un peu compliqué d'être régulier.

*Organisatrice:* Ouais, ouais.

*Participante:* Et puis moi, je mange vraiment beaucoup de produits frais.

*Organisatrice:* Donc d'un côté, il en faut suffisamment.

*Participante:* Donc il en faut quand même.

*Organisatrice:* Ouais. Effectivement aussi, ce n'est pas comme si on avait l'épicerie en bas de chez soi.

*Participante:* [Bah] Pas à l'épicerie, comme j'aimerais [quoi]. Il y a un petit Utile [là] avec trois fruits et légumes qui viennent de l'autre bout du monde... Ça ne me fait pas rêver. Après j'ai quelques producteurs, mais [euh] ils ont des horaires-- mais ça, c'est normal aussi. Moi, je travaille que les agriculteurs, je l'ai déjà dit donc [euh] ils ne peuvent pas ouvrir leurs magasins tout le temps. Il faut aussi qu'ils s'occupent de leurs animaux et qu'ils soient dans le champ. Et c'est vrai que les horaires sont tellement réduits-- il y en a plein dans mon coin et [en fait bah] ce n'est pas-- ce n'est pas facile de les attraper.

*Organisatrice:* Ouais.

*Participante:* Il faut avoir que ça à faire aussi parce qu'ils ouvrent souvent le mercredi ou le vendredi pour des créneaux de deux heures, rarement plus. Et quand on bosse, [bah] ce n'est pas toujours évident. Parce qu'il y a une offre quand même.

*Organisatrice:* Oui.

*Participante:* Mais qui n'est pas si facilement accessible.

*Organisatrice:* [silence 00:07:38 - 00:07:48]

*Organisatrice:* OK, [alors] une autre photo maintenant. Le même exercice.

*Participante:* Allez [zou].

*Organisatrice:* Les mots qui vous viennent spontanément à l'esprit ?

*Participante:* [Ouhla] On a complètement changé de registre.

*Organisatrice:* Ouais.

*Participante:* [Ohlala] C'est en France, ça ?

*Organisatrice:* Je ne crois pas à la photo mais--

*Participante:* Parce que ça, par contre, ça me rappelle quand j'étais en Asie. Mais c'est vrai qu'ils ont beaucoup, beaucoup, beaucoup plus de choix, de-de fruits et légumes aussi. Il y en avait partout. Et il y en avait vraiment partout. [Bah] Là, moi c'est typiquement le genre où je ne vais pas.

*Organisatrice:* Ouais.

*Participante:* Ça ne m'inspire pas du tout confiance. Je-Je me dis que la provenance, ça vient forcément de serres ou de l'autre bout du monde. Je sais que derrière, c'est les producteurs qui ne sont pas rémunérés. Et ouais, non, ça me casse les pieds. Je n'achète pas là-dedans. Parce qu'après c'est tout le reste aussi [hein]. C'est vrai que quand on voit ce monsieur-là, on se dit : « Il est salarié du petit truc. Et puis probablement que ça, ça vient directement d'un producteur ». Enfin on n'a pas l'impression que ça fait quatre fois le tour du monde [en fait].

*Organisatrice:* Ouais.

*Participante:* Alors que là, clairement, j'imagine des caissières payées au SMIC, un producteur en face qui n'en vit pas... Les malades des pesticides qu'il y a mis dessus. [Enfin bon] Une grosse catastrophe [quoi]. Puis en plus, il y a plein de trucs emballés en plastique, en bouts de... [Bon] Ça, c'est pareil. On devrait offrir des économes à toute la population française quand même. On voit de plus en plus de barquettes, où les carottes sont râpées. [Enfin bon] Quand même, les gars...

*Participante:* [rires 00:09:24 - 00:09:28]

*Participante:* Je vais lancer une campagne « offrez un économe à vos voisins ».

*Organisatrice:* Pour une carotte achetée, un économe offert.

*Participante:* [Non mais voilà]. Ouais, ça, ce n'est vraiment pas mon truc.

*Organisatrice:* Oui.

*Participante:* Ça reflète vraiment tout ce que-- tout ce qu'il ne faut plus qu'on fasse.

*Organisatrice:* Ouais.

*Participante:* Que ça soit pour notre santé, en tant que consommateur de ces aliments-là, et puis [bah] pour tout le reste [quoi].

*Participante:* [silence 00:09:48 - 00:09:54]

*Organisatrice:* Donc ce n'est pas un endroit dans lequel vous vous sentez bien.

*Participante:* Non, moi, je n'irais pas là-dedans. Moi, j'ai un truc comme ça à côté de chez moi, je n'y mets même pas les pieds [hein].

*Participante:* Même pour d'autres [euh] produits, vous allez rarement en grande surface ?

*Participante:* C'est vraiment rare. [Mm-hmm]. C'est rare. Ça arrive, évidemment, mais pas beaucoup.

*Organisatrice:* Ouais. Donc vous ne consommez jamais de fruits et légumes qui viennent de--

*Participante:* C'est hyper rare. [Bah] Avec des amis, ou des choses comme ça, je vois pour le 1ᵉʳ janvier, on fait les courses, je ne fais pas chier tout le monde. Tant pis [hein], pour une fois. [Bon, bon voilà]

*Organisatrice:* Pour les occasions--

*Participante:* Les vacances... Mais quand c'est que pour moi, non, je suis-- [euh] oui, non, je ne fais pas trop ça, non.

*Organisatrice:* Et vous pensez quoi des produits que-- qu'il vous arrive des fois de consommer, des fruits et légumes qui viennent de grandes surfaces ?

*Participante:* [Bah] Il y a des fois, on ne se rend pas compte de la différence. Pour être honnête, [hein] c'est vrai que sur certaines choses... Pour le premier de l'an, ma copine, elle, avait ramené des mangues et des litchis, ce n'était pas désagréable. Moi-- après, moi qui ai vécu en Asie, [bon] le goût des mangues laisse à désirer, mais ça fait tellement longtemps. En même temps, c'était sympa quand même. On peut ne pas s'en rendre compte pour des légumes à soupe ou autre, mais une carotte râpée en grande surface, c'est dégueulasse par rapport à une vraie carotte qui viendrait de mon jardin ou d'un producteur bio, c'est-- les tomates, certains fruits, il y a une grosse différence.

*Organisatrice:* Oui, ça dépend des--

*Participante:* Mais pas pour tout, non. C'est vrai qu'un chou, c'est pareil. Un chou, ça ne change pas grand-chose.

*Organisatrice:* Ouais.

*Participante:* J'ai entendu dire que les gens avaient beaucoup de mal à digérer les choux-fleurs quand il y avait un-un produit dessus [là]... Je ne me souviens plus du nom du produit. Ouais, ouais, il y a des gens qui consomment que du choux-fleurs bio justement, parce que sinon il y a un produit qu'ils mettent sur le chou qui se digère mal. Mais-mais moi c'est vrai que je-- non, il y a tous ces produits-là, je ne me rends pas compte de la différence. Les tomates, les patates, tout ça. On ne se rend pas compte.

*Organisatrice:* Oui. Donc ce n'est pas-- des fois, ce n'est pas forcément le goût qui vient régir vos-vos choix ?

*Participante:* [Bah] Moi, c'est un peu tout, c'est toute la filière aussi. Je travaille avec les agriculteurs, [pff] ils ne sont pas payés... Quand je vois le prix où ça arrive sur les étalages... Et puis [bah] c'est l'eau que je bois qui va être polluée parce qu'ils-- [enfin] ils mettent des pesticides. C'est toute une filière qu'il faut refuser. Moi je suis très boycott [en fait]. Je ne vote pas trop, je ne manifeste pas des masses, mais je suis très boycott. Je me dis la seule solution-- [bon bah] on n'est pas prêts de le faire-- mais si on arrêtait tous d'aller chez Leclerc, [pff] on est pénards, dans un mois, ils mettent la clé sous la porte.

*Organisatrice:* [Mm-hmm][Bah] Oui. Oui, c'est un peu le seul pouvoir qu'on a.

*Participante:* Donc c'est vrai que je n'aime pas trop-- Je me dis [bah] « Non, je préfère donner mon argent à des gens qui travaillent correctement et puis je suis sûre que ça va dans leur poche ».

*Organisatrice:* Oui.

*Participante:* En plus, de plus en plus dans ces grandes surfaces, on n'a même plus de caissières. Il faut passer par des machines. Ça me rend complètement dingue.

*Organisatrice:* Ouais.

*Organisatrice:* [silence 00:12:58 - 00:13:04]

*Participante:* Voilà, c'est tout un modèle. C'est-Ce n'est pas le goût parce qu'il y a des fois où on ne se rend pas-- on ne se rend pas compte. Et puis c'est vrai que c'est pratique aussi les aliments ultra transformés. C'est sympa de temps en temps d'aller acheter une pizza.

*Organisatrice:* Oui.

*Participante:* Une connerie comme ça. Mais je le fais hyper rarement, oui. Tant pis, je sacrifie mon confort.

*Participante:* [rires 00:13:21 - 00:13:26]

*Participante:* Oui, ce n'est pas une habitude [quoi]. Et est-ce que ça vous est arrivé de perdre des fruits et légumes que vous aviez achetés en grande surface ?

*Participante:* Ah [bah] non ! Ça ne pourrit pas. Non mais-- une fois, j'avais halluciné parce que j''avais acheté beaucoup de tomates et-et il y en a une que j'ai poussé jusqu'au bout. Mais je crois bien que je l'ai gardée trois mois la tomate. Increvable le truc ! Je ne sais pas ce qu'ils mettent dessus. Non, ça pourrit beaucoup moins vite. Beaucoup, beaucoup, beaucoup moins vite.

*Participante:* [silence 00:13:58 - 00:14:04]

*Organisatrice:* OK, du coup... ça pourrit peu. Voilà la troisième photo, on les fait toutes. Même [euh] idée. Là, qu'est-ce qu'il vous vient, par la tête ?

*Participante:* Ça, c'est mon futur jardin. Ça, c'est mes carrés de potager que je suis en train de préparer, les pauvres. Et je vous voudrais dessiner des petits trucs dessus, je n'ai pas eu le temps de finir mes petits pochoirs. Donc ça, c'est bon, c'est chez moi, bientôt.

*Organisatrice:* Futur potager.

*Participante:* [Ah] Oui, excellent ! Ouais, ouais, moi je vais me faire un potager. Comme ça au moins, je sais ce que je mange. Et puis parce que j'adore ça... aussi. [Bah] Ça, ouais ouais, c'est ce que j'aimerais avoir chez moi. Ouais, un petit potager comme ça, là. C'est trop sympa.

*Organisatrice:* Et pourquoi ?

*Participante:* Pour plein de choses. Parce que déjà, j'adore être dehors. J'adore. J'ai un travail derrière un ordinateur donc c'est vrai que d'aller faire du jardin le soir, ça me fait un bien fou.

*Organisatrice:* Ouais.

*Participante:* Ça me détend, ça me déstresse. Et puis-- et puis [bah] j'adore ça. Je regarde mes petites plantes pousser, je leur parle. C'est dramatique. Non mais-- là, j'ai planté des arbres. Je vais leur parler régulièrement pour être sûre qu'ils s'adaptent bien chez moi, ça fait beaucoup rire mes amis. Non, mais j'adore ça. C'est vraiment-- c'est un truc-- puis après, évidemment, il y a l'argument santé et goût. Mais moi, c'est un peu ma médit-- puais, mon truc qui me permet de me sentir bien, c'est mon jardin, [quoi] j'adore ça.

*Organisatrice:* Oui.

*Participante:* Après, je sais qu'il y a des personnes qui peuvent trouve ça extrêmement contraignant. C'est vrai que c'est du boulot. Il y a intérêt de bien aimer quand même quand on se lance, parce que sinon... [Bah] Il faut être régulier [quoi].

*Organisatrice:* Oui.

*Participante:* Mais ouais, ouais, j'aime bien ça oui. Et puis [bah] je sais ce que j'ai mis dans mon jardin. [Bon] Je ne suis pas tout à fait sûre de ce qu'ont pulvérisé les agriculteurs autour, mais moi en tout cas, je ne mets pas de saloperies dedans [quoi].

*Organisatrice:* Oui.

*Participante:* Et puis, honnêtement, une vraie tomate de jardin, mais ça n'a rien à voir. C'est top.

*Participante:* Oui, c'est ce que vous disiez, il y a des produits où encore la différence n'est pas grande, mais sur des produits comme la tomate...

*Participante:* [Ah] Ouais, même les carottes râpées, des betteraves... Oui, il y a une vraie, vraie, vraie différence. Les salades... Et puis [bah] c'est génial parce qu'il suffit de- d'aller cueillir. Tout est tout le temps frais. En termes de gaspillage aussi, c'est top [hein]. Parce que les tomates, on va les chercher, la salade, les aromates, tout au fur et à mesure. Donc c'est à la fin de l'été où ça se complique un peu parfois, on est un petit peu débordé pour faire des conserves.

*Organisatrice:* Ouais, ouais.

*Participante:* Ça se bouscule ou il faut avoir beaucoup d'amis à qui ramener tout un tas de trucs. Mais sinon [ouais], je pense qu'il y a moins de gaspillage aussi, en y réfléchissant... Lorsqu'on parle de ça... Ouais, je pense que je jette moins. Parce que j'ai déjà eu il y a quelques années des jardins. Parce que c'est vrai que [en fait] je crois que je mets pas mal de trucs. Après, quand il y en a trop, j'en donne à tout le monde. Mais comme je cueille au fur et à mesure tout-tout-tout le milieu de l'été, normalement on ne perd rien [hein].

*Organisatrice:* [silence 00:17:10 - 00:17:16]

*Participante:* Puis c'est éviter d'aller en grande surface. Je suis vachement mieux à parler à mes tomates que d'être devant une machine, là.

*Organisatrice:* Oui, le potager c'est un endroit où vous vous sentez bien. Ce n'est pas contraignant-- il y a des contraintes de travail, mais--

*Participante:* Ouais, il y a une contrainte de travail, mais ça fait du bien surtout quand on passe sa vie derrière un ordinateur, moi, ça m'aère, ça me fait du bien.

*Organisatrice:* Oui. Admettons que vous êtes amenée à jeter des produits que vous avez vous même produits.

*Participante:* On fera une cérémonie. Non, non, mais je vais les mettre au compost, tant pis.

*Participante:* [rires 00:18:00 - 00:18:06]

*Participante:* Ouais [bon bah] de toute façon, après, on n'est jamais à l'abri d'attaques d'insectes ou de trucs comme ça. Non mais des fois, ils nous arrivent des trucs à une vitesse... Le mildiou, les choses comme ça. Il y a de la perte, un petit peu quand même. Ce n'est pas-- mais bon, j'aurais moins mauvaise conscience parce que-- parce qu'après tout, ça retourne à la terre. Et puis je n'ai pas pourri l'environnement en faisant pousser mes légumes [quoi].

*Organisatrice:* Ouais.

*Participante:* Donc fatalement, ça ne peut pas être 100 % de réussite.

*Participante:* [silence 00:18:30 - 00:38:35]

*Participante:* Puis il y a toujours des petits rongeurs, des petites bestioles qui viennent manger.

*Organisatrice:* Oui.

*Participante:* [silence 00:18:42 - 00:18:48]

*Organisatrice:* Vous pensez qu'il y a moins de culpabilité à perdre en fruits et légumes que vous aviez vous même fait pousser que quelque chose que vous aviez acheté ailleurs ?

*Participante:* Ouais. [Bah] Oui, carrément.

*Organisatrice:* OK, on va se faire la dernière photo.

*Participante:* [Ah Bah] Un petit marché. [Bah] Ça, j'adore aussi faire mes courses au marché. Ça, c'est ce que je préfère.

*Organisatrice:* Ce que vous sauriez dire pourquoi ?

*Participante:* Parce que-- [euh][bah] déjà parce que je suis dehors. Je déambule.

*Organisatrice:* Oui.

*Participante:* Il y a des gens, des vrais gens.

*Organisatrice:* Ouais.

*Participante:* [Bah] Toujours pareil [hein]. C'est l'ambiance. Moi, les grandes surfaces, où je suis obligée de marcher des kilomètres avec un caddie sous des néons. C'est insupportable ! Alors que là ce n'est pas du tout le-- ce n'est pas du tout pareil. C'est beaucoup plus cool. C'est beaucoup plus long aussi ! C'est sûr qu'il y a-- on fait la queue à chaque étalage. Le marché [Nom du marché], il y a des fois, quand il y a beaucoup de monde, c'est infecte. Il faut une heure pour acheter trois trucs. Donc ça oui, c'est un peu contraignant. Mais il y a plein d'avantages sur plein de choses. Déjà, il y a beaucoup moins d'emballage. Si on s'organise bien, on vient avec ses boîtes d'œuf, ses bocaux, ses sacs. Vraiment, on peut faire du zéro déchet. Donc ça, c'est plutôt pas mal. En plus je donne mon argent directement à un producteur normalement et pas à une caissière qui va tout redonner à ses actionnaires. C'est aussi hyper important. Après, [bah] oui, je privilégie le bio aussi. Pas ce qui vient de Rungis, évidemment.

*Organisatrice:* Maintenant que vous parlez de Rungis, ça me fait rire.

*Organisatrice:* [rires 00:20:39 - 00:20:46]

*Participante:* [Mais] Et puis j'aime bien l'ambiance ! Oui, je trouve ça sympa, il y a des petits producteurs de partout. On se revoit de semaine en semaine quand on y va régulièrement. Ce n'est pas mal-- ce n'est pas mal l'humain, c'est sympa.

*Organisatrice:* Oui. Il y a les produits, mais pas que [quoi].

*Participante:* [Bah] Et puis je pense qu'aussi pour celui qui vend [enfin] c'est plus-- c'est plus valorisant quand même d'avoir ses clients qui viennent lui dire que c'était-- que c'était bon, que c'était super.

*Organisatrice:* Ouais.

*Participante:* Après, on le fait peut-être moins que les maraîchers, mais [euh] ça aide souvent les producteurs de fromages, de yaourts, de choses comme ça. Ils ont souvent un retour assez positif sur ce qu'ils font.

*Participante:* [silence 00:21:24 - 00:21:30]

*Participante:* Parce que quand ils envoient ça dans une « coop »... [Pff] Ils ne savent même pas où ça va être vendu. J'ose espérer qu'ils gagnent mieux leur vie, je pense normalement aussi.

*Organisatrice:* Est-ce que ça vous arrive de jeter des produits que vous avez achetés au marché ?

*Participante:* [Alors] Rarement, comme pour le reste. Mais oui, bien sûr, oui.

*Organisatrice:* Et le sentiment, est-ce qu'il est différent quand ça vient du marché ou qu'en grande surface par exemple, ou pas ? Selon vous.

*Participante:* Non, ça me fait chier tout pareil. Ouais, ouais je n'aime pas ça, non. Un truc auquel je pense aussi, c'est bête, mais alors au marché on discute avec les gens. Même dans la file, quand on attend [et tout] alors que dans une grande surface, on est tous avec notre caddie en train de courir comme des débiles dans tous les sens. C'est une énorme différence. Les gens ne se parlent pas dans un supermarché. Jamais. Alors qu'au marché, c'est facile d'engager la conversation quand on fait la queue [et tout]. C'est bizarre d'ailleurs.

*Organisatrice:* [Bah] Justement c'est-- il y a une question d'ambiance [quoi]. Je ne sais pas. Les gens ne sont pas... Au supermarché, tout le monde a l'air stressé. On a l'impression de toute façon, que les gens, on n'a pas envie de les embêter non plus. [Enfin] Tout le monde est-- sait qu'on passe un mauvais quart d'heure. Donc chacun passe son mauvais quart d'heure, on ne va pas aller embêter le voisin. Alors qu'au marché, on est même-- pourtant, on fait la queue, même des fois plus longtemps, mais on est plus souvent de bonne humeur et du coup, on engage plus facilement une conversation.

*Participante:* Conclusion : il faut abolir les grandes surfaces.

*Organisatrice:* [Bah] Oui, c'est ça.

*Participante:* Ce n'est pas possible ces trucs.

*Organisatrice:* Mais [pff] malheureusement il y a des-- quand je fais les entretiens, il y a des personnes qui disent [bah], même des personnes-- des personnes plus ou moins sensibilisées mais-- il y a des personnes qui disent que pour le côté pratique [euh], ils ont du mal à s'en passer [quoi].

*Organisatrice:* Quand ils ont temps, mais ils vont essayer de faire un-- d'-d'autres fonctionnements, mais quand ils ont des semaines de zinzins avec le travail, les enfants... [et tout] Ils disent : « Mais [pff] Je suis sur le chemin du supermarché, je prends tout ».

*Participante:* Ouais, ouais c'est sûr, mais moi c'est vrai que je fais un peu ça aussi. Moi j'ai pas d'enfant donc déjà c'est plus-- le frigo se remplit-- [enfin] se vide moins vite aussi.

*Organisatrice:* Oui, oui.

*Participante:* Et c'est vrai que quand je vais dans un supermarché parce qu'il faut, [bah] je remplis le caddie et je n'y retourne pas pendant un mois et demi, deux mois.

*Organisatrice:* C'est ça, tant qu'à faire.

*Participante:* Après, c'est bon. C'est comme le dentiste [hein].

*Organisatrice:* La totale, il y passe l'aprem.

*Participante:* Mais je comprends quand on a une famille avec quatre, cinq qui mangent, qui mangent, qui mangent.

*Organisatrice:* [Bah] C'est ça ! Et donc sachant qu'ils doivent quand même y aller toutes les semaines... Donc si-- [ouais] mais du coup, je réfléchissais dans la voiture et je me disais : « Mais pourquoi ? ». Il faudrait créer un endroit assez grand [et tout], mais quand même aéré, pare que le côté dehors est très, très il est-- [enfin] ça ressort beaucoup dans l'interview du marché, mais qu'il y ait plein de petits magasins de proximité-- mais après j'y pense-- ça existe déjà, c'est des halles. Mais il y en a plus tant que ça.

*Participante:* Non, il n'y en a pas beaucoup, non.

*Organisatrice:* Parce que [en fait] c'est ça qu'il faudrait. Un endroit aéré où c'est un gros marché [quoi]. Mais-Mais il n'y a pas les néons et [voilà] il n'y a pas forcément les caddies, [et tout ça] juste des petits sacs. Mais ça existe, [en fait] oui.

*Participante:* Ça existe mais après c'est-c'est vrai que la grande surface, ils ont bien réussi leur coup quand même, parce qu'on peut se garer. C'est gratuit, c'est facile. Les Halles, c'est dans le centre-ville. Quelqu'un qui a besoin d'acheter pour une semaine pour une famille, avec son cabas qui pèse trois tonnes, sa bagnole qui va être garée à perpète... Il va falloir payer cinq balles de parking parce que ça coûte une fortune en ville. [Enfin] Ce n'est pas...

*Organisatrice:* Alors il faudrait découper les centres commerciaux et ne pas faire des supermarchés, mais faire vraiment plein de petites boutiques. Mais bon, on reste sous des néons [et tout] à ce moment-là ça-- [enfin] c'est--

*Participante:* [Bah] Oui, ce n'est pas évident... [hein] Il y a des magasins de producteurs après qui se montent à droite, à gauche, ça ce n'est pas trop mal...

*Organisatrice:* Oui, oui, ça serait ça le-- mais comme on ne trouve pas non plus tout, absolument tout, tout, tout... [quoi]

*Participante:* Non, puis c'est vrai que ce n'est pas toujours abordable non plus.

*Organisatrice:* Il y a la question du prix aussi, oui, oui.

*Participante:* Parfois c'est quand même très, très, très cher. Ça-Ça dépend mais--

*Organisatrice:* Oui, il y a des personnes qui disent : « Moi j'aimerais bien manger chez le primeur », mais-mais non...

*Participante:* [Bah] Encore les fruits légumes, je ne sais pas si en cuisinant-- si c'est une question de budget, mais c'est vrai que dans ces magasins-là, il y a aussi beaucoup de produits préparés, des confitures... Et là, vraiment, les prix sont différents.

*Organisatrice:* Ouais, ouais, là il y a une différence de prix. Ouais, ouais, des fois c'est--

*Participante:* C'est terrible.

*Organisatrice:* Donc oui, les familles qui dans un budget serré et qui ne peuvent pas...

*Participante:* Et c'est pareil, avoir le temps de cuisiner, ce n'est peut-être pas donné à tout le monde non plus, de cuisiner tout le temps.

*Organisatrice:* Donc oui il n'y a pas-- je ne sais pas si un jour on sortira du-- mais ce qui est rigolo c'est que même les-les personnes plus âgées disent qu'ils retournent il y a un in-- ça s'inverse. Et quand il y a eu les débuts des supermarchés, ils ont arrêté de-- ils ont arrêté les commerces de proximité. Maintenant, ils retournent aux commerces de proximité. Donc il y a quand même un-- il se passe quelque chose, là, je pense. [euh]

*Participante:* Mais je crois que les gens soient en train de comprendre aussi. Parce que moi j'en parlais déjà avec mes parents quand j'étais plus jeune, et c'est vrai que personne ne me comprenait trop. Mais je disais-- parce que ma mère, avec ses amies-- elle avait une amie, [alors] elle adore du shopping et passerait sa vie dans les magasins, je-je ne comprends pas, elle est complètement-- [mais bon] mais voilà, c'est Gifi, c'est machin pour acheter des bouinasses pour sa maison. Tout du « made in China ». Et moi j'étais en école d'ingénieur et je leur disais, mais il va nous rester quoi nous comme boulot si vous achetez que de l'import ? Elles s'en foutaient complètement. Très clairement. Ça ne dérange pas du tout les générations de nos parents, mais pas du tout ! Ils en ont rien à foutre, « après nous le déluge » ! J-J'hallucinais. Mais c'est vrai que je pense qu'aussi les gens sont en train de se rendre compte petit à petit de ça. On ne s'était peut-être pas rendus compte avant, au début, parce que c'est vrai que c'était pratique, ce n'était pas cher [tout ça]. Mais n'empêche qu'aujourd'hui...

*Organisatrice:* Il y a le retour du bâton.

*Participante:* On ne fabrique plus de médicaments, on ne fabrique plus de masques, on s'est rendu compte avec la crise [bah] qu'on ne fabrique plus rien. Il suffit que la Chine nous dise : « Écoutez la France, on ne vous livre plus », et on n'a plus rien dans notre pays [quoi]. Donc je pense que les gens vont peut-être commencer un petit à... J'espère.

*Organisatrice:* Oui, je pense que les gens oublient, ça vient--

*Participante:* Pour qu'il y ait du travail, il faut qu'on ait des usines, qu'on ait des magasins. Parce qu'avant, dans une ville [je ne sais pas moi] il y avait dix bouchers et ça faisait dix familles qui vivaient. Maintenant, il en reste un ou deux par ci par là. Et tout le reste, ça va aux actionnaires parce que les-les gens qui travaillent pour ces grandes surfaces-là, ils ne gagnent pas d'argent, ils sont payés au Smic.

*Organisatrice:* Oui, non, c'est clair.

*Participante:* C'est des gens qui vivent sous le seuil de pauvreté les trois quarts du temps. Ils sont à 24 heures, 30 heures payées au SMIC. Super !

*Organisatrice:* Oui, sous des néons.

*Participante:* C'est pour ça que qui-quitte à ne pas aller en sur-- en grande surface, je préfère encore, sinon prendre le temps d'aller chez un boucher ou--

*Organisatrice:* [Bah] C'est ça.

*Participante:* Ou une épicerie plus locale qui ne sera pas sous enseigne [quoi]. Mais--

*Organisatrice:* [Bah] Ça, à Brest, le magasin, là... Il n'y a pas mal de personnes qui-- du projet, qui-qui vont-- c'est Ti Coop !

*Participante:* Ti Coop, oui.

*Organisatrice:* Où là justement le but c'est de-- c'est l'épicerie mais sans--

*Participante:* [Ah] Oui, c'est top ça.

*Organisatrice:* Sans actionnaires derrière [quoi].

*Participante:* Même les Biocoops [hein], c'est limite [mais bon].

*Organisatrice:* [Enfin] Oui, Biocoop le principe, il était bien, mais c'est-c'est devenu-- je pense qu'il y a quand même des gens qui s'en mettent dans les poches au vu du prix des fois des produits. Où va cet argent ? Pourquoi c'est si cher ? Ça devient un peu curieux quand même.

*Participante:* Et ça c'est vrai que c'est un gros problème. Parce que tout-tout-tout-tout, l'argent est aspiré vers le haut, il n'y a plus de boulot... Ce n'est plus possible [quoi]. On ne peut pas continuer comme ça.

*Organisatrice:* Oui, je ne sais pas si-- [bah] c'est clair, mais je ne sais pas quand est-ce que ça va-- ça va se décanter. Je ne sais pas comment--

*Participante:* [Bah] Je ne sais pas parce qu'il y a de plus en plus de gens qui s'en rendent compte. Mais finalement, dans les actes...

*Organisatrice:* Je ne sais pas, je pense que ça va aller--

*Participante:* Ils n'ont pas le temps, ils sont pressés... On dit qu'on va le faire, on va une fois l'été au marché, on fait : « [Ah] Super, j'ai fait une bonne action » et puis le reste de l'année...

*Organisatrice:* Et ça-- mais le problème, c'est que c'est un tout parce que si on-- ça va-- ça va loin là-- mais si on arrêtait de faire travailler les gens 35 heures, qu'on autorisait des semaines à 25 heures, les gens auraient plus de temps pour justement changer les habitudes [tout ça]. Ça se trouve, ça arrange bien [en fait] que les gens n'aient pas le temps pour justement ne pas avoir de-- être beaucoup moins réfléchis sur--

*Participante:* [Bah] De toute façon, ça arrange évidemment puisque comme ça, ça permet de maintenir une grosse partie de la population au chômage. Donc ceux qui travaillent ayant tellement peur d'être au chômage acceptent tout et n'importe quoi. S'il n'y avait plus de chômeurs, on pourrait poser nos conditions face à l'employeur. Ce serait une catastrophe, ils ne vont pas s'en remettre les mecs.

*Organisatrice:* Oui, ils ont l'impression qu'un autre pourrait faire son-- un autre pourrait prendre notre place [quoi].

*Participante:* [Bah] Voilà, donc comme ça, ils arrivent à nous maintenir-- le chômage est voulu, il ne faut pas rêver.

*Organisatrice:* C'est une catastrophe. Oui, oui.

*Participante:* C'est une catastrophe, mais c'est réel. On vit dans un monde qui ne va pas bien.

*Organisatrice:* Non, c'est clair.

*Participante:* Et là depuis deux ans, on le voit mais--

*Organisatrice:* Oui, c'est plus palpable.

*Participante:* Avant, on le voyait moins.

*Organisatrice:* C'est plus palpable, on se rend compte. [euh]

*Participante:* Avant moi, j'avais déjà ce discours-là, on me prenait un peu pour une gentille allumée. « Bon, elle est gentille [prénom de la participante] avec ses petites idées. Elle est sympa, elle nous gonfle un peu, mais on l'aime bien ». Mon frère est pareil que moi, donc on est un peu-- les deux un peu « bizarres ».

*Organisatrice:* Ouais.

*Participante:* Sauf que là, depuis quelques temps, ça se voit [quoi]. Avant, ça se voyait moins. Il y a plein de gens qui en parlaient mais [bon] bof.

*Organisatrice:* Oui, là, les incohérences-- en temps de crise on--

*Participante:* Oui, ça nous saute plus aux yeux. Donc peut-être petit à petit. Espérons que les jeunes soient un peu moins cons.

*Organisatrice:* Malheureusement, des fois je vois des-- des fois je crois-- je suis naïve, je crois « [Ah non] Ces gens-là, [enfin] c'est des vieux qui suivent ces personnes-là ». Et puis [en fait] on regarde ces meetings [ou quoi], et on fait : « [Bah] Non, ils ont mon âge ». On ne va pas être sauvé par notre génération. [Enfin bon] Est-ce que vous mangez de la banane ?

*Participante:* Ouais, un peu.

*Organisatrice:* Ouais ? Alors je vais vous demander de ranger la banane. Dix photos, celle qui vous donne le plus envie à celle qui vous donnent le moins envie.

*Participante:* [Ah] Oui, c'est que des bananes.

*Organisatrice:* C'est la même banane.

*Participante:* [Ouhla] Quelle horreur ! Moi comme ça, je ne la mange pas. Moi je les mange quand elles sont toutes vertes, même ça c'est trop. Là, c'est vraiment pour pas l'acheter. Voilà, comme ça.

*Organisatrice:* Là c'est la banane idéale ?

*Participante:* Elle est un peu fluo votre banane.

*Organisatrice:* Ouais.

*Participante:* [Ah] Non mais ça ce n'est pas-- non mais de toute façon, moi elles n'atteignent pas ce stade-là, parce que je ne supporte pas quand c'est mûr.

*Organisatrice:* Ah ouais ?

*Participante:* [Bon alors] Je- Je prendrais celle-là très clairement, très, très clairement. Ça c'est--

*Organisatrice:* C'est la meilleure ?

*Participante:* Ouais, ce serait la meilleure.

*Organisatrice:* Allez, on la met là.

*Participante:* Ce serait vraiment la meilleure. [Alors] Ensuite, on passerait avec celle-là...

*Participante:* Celle-là, je ne pense pas que je la mangerais, on dirait un concombre.

*Organisatrice:* Ouais, ouais bon, c'est-- pour de vrai, on n'en avait pas de verte et il fallait une verte alors du coup elle a été retouchée, mais--

*Participante:* Non, mais je ne la mangerais pas, elle est trop verte.

*Organisatrice:* Elle est trop verte ?

*Participante:* Disons que ça je le mange, et après... Ouais, allez, c'est vraiment pour ne pas jeter. Après ça, je ne la mangerais même pas. Mais vraiment pas du tout.

*Participante:* Ouais donc celle-là, on pourrait la mettre là, dans le sens où-- à la rigueur, on peut attendre qu'elle-- [euh] qu'elle devienne ça.

*Participante:* Mais ça, je ne supporte pas.

*Organisatrice:* Donc ça je les mets en maturité, mais vous ne les mangerez pas [quoi].

*Participante:* Non. Mais elles n'arrivent pas à ce stade-là.

*Organisatrice:* Je prends une photo de votre-- [inaudible 00:33:18 - 00:33:20]

*Participante:* [Alors] Ouais, je suis terrible sur les bananes. [Alors] Je sais qu'il y a deux écoles : il y a des gens qui les aiment bien mûres et il y a des gens qui les aiment très vertes. Après il faut trouver des gens qui les aiment bien mûres, pour pouvoir les refourguer.

*Organisatrice:* C'est ça. C'est marrant la banane, comment les avis sont... Les-Les goûts sont différents.

*Participante:* Mais tout le monde en mange, j'ai l'impression, par contre.

*Organisatrice:* C'est ouais-- [Bah] Ceux qui n'en mangent pas, c'est vraiment que-- [voilà] C'est un goût-- comme on n'aime pas un truc. Mais il y a beaucoup de-- et c'est un des seuls, justement. Aux premières périodes des entretiens là, ce qui m'a fait rigoler, c'est que même toutes les personnes qui ont des-- qui font attention à l'importation, il y a la banane, c'est la-- c'est l'entorse pour tout.

*Participante:* C'est ça. Moi, je n'achète pas d'avocat, j'achète des bananes.

*Organisatrice:* C'est ça et c'est-- on le sait mais en plus, [bon] ça va des fois il y en a qui sont françaises. Du coup c'est-- [bon] c'est quand même que même en France ! Mais c'est l'entorse que-- des fois les gens ne se rendaient même pas-- c'est après, en parlant, après avoir dit : « [Oh] Je n'achète pas d'importation », vous savez. Et puis après on parle des fruits : « Qu'est-ce que vous avez ? - [Bah] Là, j'ai des bananes ». [Ah] Oui, donc oui, c'est-- OK, les bananes, ça vient de loin, mais c'est le seul.

*Participante:* C'est vrai. Ouais, on n'y fait pas gaffe.

*Organisatrice:* Non, [bah pff] on le sait, mais c'est que ça fait tellement partie de notre-- [enfin] c'est-- l'avocat, même si on trouve ça bon, on en a mangé, on en a tous déjà goûté [et tout], on sait que c'est un peu exceptionnel. Que la banane, on a l'impression que ça fait-- [enfin] ça fait partie de notre alimentation--

*Participante:* Oui quotidienne quasiment.

*Organisatrice:* [Bah] Oui, c'est ça.

*Participante:* Ouais, ouais, c'est clair.

*Organisatrice:* Donc ouais, beaucoup d'amateurs de bananes en France.

*Participante:* Je ne sais pas si je peux tenter les bananes dans la région, c'est peut-être un peu juste... Même si ça se réchauffe.

*Organisatrice:* [Pff] Je ne sais pas... Parce qu'on dit que justement à Brest [tout ça], il-il y a des-- ils sauvegardent des espèces tropicales parce que ça ne gèle pas. Donc ça se trouve... Pourquoi pas ? Vous pourriez essayer.

*Participante:* Je vais m'installer en tant que producteur de bananes.

*Organisatrice:* Ce serait dingue ! Les petites bananes... Ce n'est peut-être pas aussi-- [enfin] peut-être différentes du coup, mais peut-être.

*Participante:* Les bananes Lecken du Treyon. Ça aurait de la gueule, hein ?

*Organisatrice:* Ouais, carrément. [Alors] Du coup, vos bananes. [Alors] La préférée-- [Alors] J'ai mis des formats pour ça, pour qu'à l'audio je puisse savoir de quelle banane vous parlez.

*Participante:* Ma banane préférée moi, c'est le triangle bleu.

*Organisatrice:* C'est le triangle bleu. [Alors] Est-ce que vous pouvez me la décrire ? Pourquoi c'est votre préférée ?

*Participante:* [Oh bah] Elle commence juste à passer du vert au-au jaune. Et j'aime bien au goût comme ça. Quand elle est plus mûre, c'est trop sucré.

*Organisatrice:* D'accord. Donc à partir de quelle banane vous ne prenez plus de plaisir ? Vous mangez « histoire de ».

*Participante:* Le rond blanc.

*Organisatrice:* Le rond blanc ?

*Participante:* [Mm-hm] C'est vraiment pour ne pas jeter.

*Organisatrice:* Et donc, après en termes de maturité, la carré bleu-- carré bleu c'est--

*Participante:* [Ah] Ouais, là-- [pff] Je fais appel à un ami.

*Organisatrice:* 50/50

*Participante:* Est-ce que quelqu'un aime les bananes mûres ? Et après, non, je ne mange même pas. Non, non, non, non.

*Organisatrice:* Et est-ce que vous cuisinez des fois la banane ou pas ?

*Participante:* [Hmm] Des pancakes à la banane ?

*Organisatrice:* Oui ?

*Participante:* Et c'est à peu près tout.

*Organisatrice:* Et dans ce cas-là, vous utilisez quelles bananes ? Ça peut être n'importe laquelle ?

*Participante:* Ouais, n'importe laquelle. De préférence la plus mûre, effectivement, parce que je sais que celle-là ne me plaira pas au goût après. Et elle est plus facile à écraser.

*Organisatrice:* Mais ce n'est pas forcément une habitude de se dire « Là c'est-- [enfin] j'ai une banane comme ça. Du coup, je vais faire un pancake aux-- ».

*Participante:* Ah, ce n'est pas forcément ça qui va me pousser à en faire.

*Organisatrice:* Oui, c'est plus si vous voulez des pancakes, vous regardez vos bananes, vous prenez la plus mûre. Mais ce n'est pas « [Oh] Celle-là, ça y est, c'est trop tard. Je vais faire un pancake ».

*Participante:* Et en même temps, je fais vraiment gaffe-- je-je ne jetterai jamais de bananes. Je ne jette jamais de bananes, je fais attention. Donc oui, peut-être que si je l'ai laissés aller jusque-là, je ferais peut-être des pancakes pour ne pas la jeter.

*Organisatrice:* Oui, mais ça n'arrive pas--

*Participante:* Mais c'est vrai que je les achète très très vertes et peu-- par petites quantités pour être sûre de ne pas.

*Organisatrice:* Oui donc finalement--

*Participante:* Je-Je ne jette pas de bananes, non. Non mais parce que je suis maniaque avec ça. Ça, c'est trop bien, je n'aime vraiment pas du tout. J'ai remarqué, c'est un vrai sujet ça.

*Organisatrice:* [Bah] C'est-- [donc] il y avait une autre personne que vous, c'était [genre] ces trois là et c'était encore-- et vraiment-là, c'était trop tard. C'était très-- elle savait cette personne qui était très stricte sur la banane. Et il y en a un où là, ça commence à être bon [quoi]. Et avant, ils ne veulent pas du tout. Donc [enfin] c'est que-- c'est quand même-- c'est marrant [quoi].

*Participante:* [Bah] oui, mais ça fait partie des fruits qui ont vraiment un goût qui change.

*Organisatrice:* Oui.

*Participante:* Beaucoup [hein]. On passe d'un truc assez ferme et assez peu sucré à quelque chose de complètement pâteux.

*Organisatrice:* Oui, oui, la texture se transforme...

*Participante:* Hyper sucré. Un peu comme le kiwi aussi. Le kiwi, passé un certain stade est vraiment bizarre...

*Organisatrice:* Ouais et ça [bah] d'autres personnes le disent. « Là par contre, le kiwi trop mûr... », le goût change vraiment [quoi].

*Participante:* Là il faut le passer en smoothie avec autre chose...

*Organisatrice:* Ouais ouais, moi j'avoue que le kiwi mûr...

*Participante:* Il y a quelques fruits comme ça.

*Organisatrice:* [Bah] Quand il est dur, je n'aime pas. Je n'aime pas galérer avec ma cuillère.

*Participante:* Mais c'est pour ça que j'en achète jamais parce que ça ne va jamais, le kiwi.

*Organisatrice:* C'est dur avant.

*Participante:* Ouais, ou il est trop dur, hyper acide, ou alors il passe ce stade où on a l'impression de manger des antibiotiques qu'on prenait étant petit. Les trucs en poudre, c'est dégueulasse. Et comme c'est de l'importation, [bah] moi le kiwi, du coup j'ai abandonné.

*Organisatrice:* [Bah] Ils en font-- ils essaient. [Enfin] Des fois, j'en vois.

*Participante:* J'ai acheté un pied là, pour mettre dans mon jardin, tu vois. Je tente.

*Organisatrice:* C'est vrai que ça pousse--

*Participante:* Il paraît que ça pousse ici... Je me dis que je vais peut-être pouvoir me réconcilier avec le kiwi et le cueillir au bon moment.

*Organisatrice:* [Ah bah] Oui, oui. Mais vous allez être débordée de kiwis. S'il prend...C'est-C'est incroyable.

*Participante:* Ah oui d'accord. Je vais faire un marché.

*Organisatrice:* Chez mes parents, on avait un mur... C'était en Normandie, mais du coup, c'est encore pire niveau température, il fait plus froid qu'ici et c'était des-des kilos de kiwis [pff]. Ma mère faisait du Kiwi Curd le matin, c'était super bon. Mais ça pousse... Je ne comprends pas pourquoi on a des-- [enfin] on a des kiwi de Nouvelle-Zélande alors qu'ici ça pousse.

*Participante:* [Ah] Oui ? [bah] On verra bien.

*Organisatrice:* [Ah bah] Ouais, ouais. Là, il y a d'autres gens qui me racontait là-- c'est de-- [bah] c'est pareil, ils sont débordés de kiwis. Et donc c'est-- c'est quand même incroyable qu'on en fasse importer alors que-- ça pousse très-- c'est comme les figues. Apparemment, ça pousse-- on n'en fait plus pousser ici, mais il paraît que ça pousse très bien ici.

*Participante:* [Ah] C'est marrant parce qu'on en voit des figuiers, mais j'ai toujours l'impression qu'ils ne donnent pas beaucoup de fruits à cause du manque de soleil.

*Organisatrice:* Alors peut-être que-- je ne sais pas c'est-c'est une mamie qui me disait qu'avant [bah] dans son jardin il y avait plein de figues [quoi]. Alors que maintenant elle n'en trouvait plus, elle n'en trouve pas d'ici. [Alors] Est-ce qu'il faisait meilleur avant ? Je ne sais pas.

*Participante:* Je ne sais pas, parce que c'est vrai qu'en Grèce, j'en mangeais beaucoup. Quand j'étais en Grèce, j'en mangeais plein des fraîches. Mais ici en France... Je n'en trouve pas. Enfin, pas dans le nord de la France. Dans le sud, oui, mais...

*Organisatrice:* Ça-- mais je sais-- ouais, je ne sais pas s'il n'y a pas eu un moment donné où-- vu-- en termes de rendement, c'est peut-être quand même inférieur à d'autres endroits. Et du coup le commerce a fait que [bah] on a arrêté de-de faire produire des fruits et légumes qui [en fait] étaient-- se produisaient mieux à d'autres endroits de France.

*Organisatrice:* [Oh bah] Ça, de toute façon, le rendement, il n'y a que ça qui compte. Ce n'est plus possible. Mais les particuliers, on en voit [hein] quand on se balade.

*Organisatrice:* Il y en a des-- ?

*Participante:* Il y a des figuiers quand même par ici. Pas beaucoup, mais ça arrive.

*Organisatrice:* Des figuiers, ils n'ont pas beaucoup de figues au bout de l'arbre.

*Participante:* [Bah] J'en avais vu, ouais... C'est surtout qu'elles n'avaient pas l'air mûres. Elles avaient l'air...

*Organisatrice:* [Ah] Ouais ? C'est possible.

*Participante:* Ou alors ça vient beaucoup, beaucoup plus tard, forcément ? Je ne sais pas.

*Organisatrice:* Peut-être aussi. Je ne sais pas, la mamie disait qu'elle mangeait des figues dans son jardin [quoi].

*Participante:* [Ah] Ouais fraîches, c'est top.

*Organisatrice:* Mais ouais, kiwi à mon avis, pas de soucis, vous en aurez.

*Participante:* [Bon] On verra. Mais peut-être que j'arriverai les cueillir [ouais] « bien », parce que c'est vrai que j'en achète plus à cause de ça.

*Organisatrice:* Ouais.

*Participante:* Parce que je trouve ça désagréable [en fait]. Une fois sur deux, je me fais avoir dans un sens ou dans l'autre et du coup... [pff] Bof.

*Organisatrice:* [Bah] Oui si à chaque fois qu'on l'ouvre, il n'est pas comme on veut...

*Participante:* S'il n'est pas très bon, ouais. On ne prend pas de plaisir après donc...

*Organisatrice:* C'est ça. Toujours le même problème.

*Participante:* [Bah] Oui, quand même.

*Organisatrice:* Un minimum de plaisir, c'est sympa quand on mange. Et sur la dangerosité de la banane. Est-ce que selon vous il y a un moment donné où la banane est éventuellement dangereuse pour l'organisme ou pas ?

*Participante:* Je ne me suis jamais posée la question.

*Organisatrice:* C'est une réponse.

*Participante:* Ça peut être dangereux, une banane ?

*Organisatrice:* [Bah] Je ne sais pas non-- je ne sais pas non plus. Justement, je me-- je me demande si les-les-les-- au final, non, personne. Si des personnes choisissent leur consommation de bananes [enfin] à partir de tel moment parce qu'ils pensent que ça peut être dangereux pour l'organisme. Mais au final, non. Les gens ne se sont pas posés la question. C'est juste qu'ils choisissent par goût, par plaisir de manger.

*Participante:* Parce que même en termes de digestion, même quand on les mange très vertes, [bof] ça passe [hein].

*Organisatrice:* Ouais, ouais. [Bah] Per-- il y a des personnes qui me disent que là, pour eux, ça sera trop dur à digérer mais...

*Participante:* Moi c'est vrai que je digère n'importe quoi.

*Organisatrice:* Oui, c'est ça mais on n'est pas tous égaux face à-- face à notre transit.

*Participante:* Je n'ai vraiment pas de difficultés, il y a vraiment tout qui passe !

*Organisatrice:* Pourquoi se priver alors ?

*Participante:* Ouais, ouais c'est peut-être plus délicat. Non, dangereux ? Ça, je ne me suis jamais posée la question. C'est purement, purement gustatif.

*Organisatrice:* Ouais c'est ça. Et pareil sur les apports nutritifs. Est-ce que selon vous, il y a un moment donné où la banane en a plus ou moins ? Est-ce qu'elle en perd, est-ce qu'elle en gagne ou [pff] ?

*Participante:* Bah c'est vrai qu'on a l'impression de manger plus de sucre quand elle est mûre que quand elle l'est pas. Mais [bon] j'imagine que ce qui est dedans, reste dedans.

*Organisatrice:* Magnésium, fibres... [et tout]

*Participante:* Oui, ça doit être à peu près identique.

*Organisatrice:* C'est pareil, ce n'est pas pour ça non plus que...

*Participante:* Mais après, c'est quand même pour l'aspect santé [hein] qu'on mange des fruits et légumes.

*Organisatrice:* Oui.

*Participante:* C'est bon aussi, mais oui, on sait bien que-- il y a quand même des vitamines, il y a quand même des fibres... Il y a un tas de bonnes choses dans les fruits.

*Organisatrice:* [Enfin] C'est pour ça, admettons, on vous dit que là [en fait] elle n'a pas encore ses-- je n'ai jamais posé cette question-- Admettons, on vous dit là, elle n'a pas d'apport encore-- elle en a un peu, mais elle est à moitié-- [on va dire] à 25 % de ses apports.

*Participante:* Je vais attendre.

*Organisatrice:* Du coup, vous seriez d'avis à dire : « [Bon] Allez, je vais essayer quand même d'attendre jusqu'ici pour être-- ».

*Participante:* Bon, il y a des limites. On ira jusqu'au triangle vert, après on ne va pas s'emballer. Tant pis, il y a des trucs que je mange, ça n'a aucun intérêt en termes de santé, comme les carrés de chocolat mais c'est-- je continue à en manger.

*Organisatrice:* Oui, il y a quand même une partie plaisir. Mais du coup, si vous aviez une info comme ça, vous tenderiez à vous dire « Allez... ».

*Participante:* J'aurais tendance, oui.

*Organisatrice:* Ça marche. [Alors bon] OK pour la banane. Et alors... Dernier petit jeu.

*Organisatrice:* [silence 00:44:47 - 00:44:53]

*Organisatrice:* [Alors] Ça, ce n'était pas rangé de tout à l'heure. On a des fruits et légumes... [Alors] Là, on a soit-- [en fait] c'est tout ce qu'on peut faire avec des fruits et légumes. Je vais vous demander de garder ce qui vous parle. C'est assez... de manière générale, ça ne ressemble pas à votre poêle ou...

*Participante:* OK. Ouais, ouais.

*Organisatrice:* [Voilà]

*Participante:* Ça, ça ne me parle pas du tout

*Organisatrice:* Vous ne faîtes pas de gâteaux ?

*Participante:* Je n'aime pas le sucre. Je mange très peu de sucre. Le lait, je le digère pas, ça par contre...

*Organisatrice:* [Ah !] Ça, ça ne passe pas par contre !

*Participante:* [Ah] Non, j'ai toujours trouvé ça dégueulasse. Mes parents me forçaient étant petite à en boire. Mais dès que j'ai pu m'échapper--

*Organisatrice:* Ouais, la culture du lait--

*Participante:* Je n'ai jamais plus rebu de lait !

*Organisatrice:* Pour avoir des os solides !

*Participante:* Oui ! On prend un chinois, ils ne se casse pas en deux. [Enfin] C'est ce que je dis toujours aux gens, je dis : « C'est quoi ce délire ? ».

*Organisatrice:* Oui, oui, c'est vrai qu'en Asie, ils n'ont pas de lait de vache mais ils s'en portent très bien. Après j'ai vu--

*Participante:* C'est fou ça.

*Organisatrice:* Il y a encore le lobby qui fait des--

*Participante:* Ouais, ça s'est calmé un peu...

*Organisatrice:* Il y a-- C'était Hugo Clément, je ne sais pas si vous voyez qui c'est ?

*Participante:* Ouais, plus ou moins parce que je ne regarde pas trop la télé mais j'en ai entendu parler, ouais.

*Organisatrice:* [Bah] Il fait pas mal de trucs là, et il a une-- il a un enfant et il était fou. L'enfant est revenu de l'école avec-- il y avait une marque de lait, le lobby du lait qui était-- qui venait faire des interventions pour-- Vous pouvez y aller.

*Participante:* Non, non, non, ça peut attendre. Il est bavard, lui.

*Organisatrice:* Et du coup, oui. Ils venaient faire des interventions, les enfants avaient un petit cahier, ils devaient remplir : Pourquoi c'est si bon de manger du lait [quoi] ? Et donc là, c'était cette année dans une école élémentaire.

*Participante:* Ah oui. Mais ce n'est pas bon en plus.

*Organisatrice:* [Bah non] En plus on entend dire que ce n'est pas bon et là, on y est encore. On continue quand même à faire-- à dire aux enfants « Il faut manger du lait ».

*Participante:* Mais surtout que [en fait] ce n'est pas là où il y en a le plus, du calcium.

*Organisatrice:* Non, non, mais oui-- ce n'est pas-- c'est du lait de vache [quoi].

*Participante:* Donc [bah] moi tout ça, ça me parle à peu près. C'est plus les autres petites photos qui ne me parlent pas trop.

*Organisatrice:* [Ah] Oui, celle-là ? Oui, vous ne mettez pas dans la poubelle classique, vous faites pas forcément de-du jus. Vous en faites ou pas des fois ?

*Participante:* Pas trop. Du jus d'orange un peu, parce que c'est vrai que c'est sympa au goût.

*Organisatrice:* Ouais, on peut le mettre.

*Participante:* Mais pas trop de jus quand même, parce qu'on enlève une grosse partie de ce qui est bon dans le fruit, alors c'est dommage. Puis les gâteaux...

*Organisatrice:* [Alors] Non. Là, je vous donne des fruits et légumes qui ont été jetés par des participants du projet et je vais vous demander de-- en gros, de me dire ce que vous auriez fait. [Ah] Des poules, vous n'en avez pas encore ?

*Participante:* Non, mais je pense que je n'ai pas assez grand pour mettre des poules.

*Organisatrice:* [Ah] Du coup là, vous n'allez pas donner aux poules vos...

*Participante:* Et puis de toute façon... Après je ne peux plus trop partir en vacances si j'ai des poules. Qui va s'occuper des poules ?

*Organisatrice:* [Bah] C'est ça c'est des animaux.

*Participante:* [inaudible 00:47:57 - 00:47:58] J'aurais pu en faire un jour. Là, je ne vois pas ce que je peux faire pour elles [hein].

*Organisatrice:* Alors, un compost ?

*Participante:* [Oh bah] Là... [Pff, hé, hé]

*Organisatrice:* [Ah] Oui ça pour vous, c'est-- ce n'est pas possible.

*Participante:* [Bah] Je vais couper comme ça [quoi], j'enlèverai les deux petits bouts.

*Organisatrice:* Et encore, à mon avis, si elle est comme ça au niveau du goût, pour vous, elle va être un peu trop--

*Participante:* Qu'est-ce qu'il a, ce petit radis ? On peut le mettre dans une salade. Ça va, il n'est pas malade. Là c'est pareil, on enlève le petit bout qui ne va pas, ça va faire une petite soupe. [Oh] Non là, compost quand même. Ouais, c'est un peu limite. Elle est en pleine forme cette tomate, non ? Elle a été jetée ?

*Organisatrice:* [Mm-hmm]. Apparemment elle était--

*Participante:* C'est marrant, [alors] pourquoi ?

*Organisatrice:* Apparemment, [du coup] ça ne se voit peut-être pas [du coup] mais là, elle aurait été molle [quoi].

*Participante:* [Ah] Ouais ? Elle avait un coup... Ouais [bon], on met la moitié dans la salade haute et l'autre moitié dans la soupe, et puis ça passe.

*Organisatrice:* Oui.

*Participante:* Là, c'est pareil. On peut faire un jus, on en récupère une partie, il n'y a pas de soucis. [Ah] Là...

*Organisatrice:* Ouais, il y a des moments plus ou moins...

*Participante:* D'accord, oui. Là non plus. [Ah] Ouais, [bah] la poire gâtée comme ça, ce n'est pas bon.

*Organisatrice:* Ouais, celle-là, non...

*Organisatrice:* [silence 00:49:20 - 00:49:24]

*Participante:* Là je n'en sais rien. Parce que si ce n'est pas moisi à l'intérieur, je récupérerais peut-être un peu, mais souvent ils moisissent dedans aussi. À mon avis, il finira quand même plus ici.

*Organisatrice:* Oui, il est trop attaqué ?

*Participante:* Ouais, non. Ça, je ne mange pas ça non plus.

*Organisatrice:* OK, je vais prendre une photo.

*Participante:* Après c'est pareil, on est des enfants gâtés, on a suffisamment à manger. Je ne vais pas non plus me torturer à manger des trucs moisis.

*Organisatrice:* Oui.

*Participante:* Dans une autre circonstance, effectivement... Ouais, j'ai peut-être--

*Organisatrice:* Sur une île déserte où on a ça et on est-- on fait peut-être un effort différent.

*Participante:* Il faut que ça reste un plaisir de manger, on ne va pas se torturer non plus.

*Organisatrice:* C'est clair. [Bon bah] Ça marche. [Et bah] Merci beaucoup !

*Participante:* De rien.

*Organisatrice:* Et sur la poubelle, comment ça s'est passé du coup ? Cette deuxième période ?

*Participante:* [Bah] Bien. Je n'ai pas jeté grand-chose. Ouais, j'ai utilisé deux sacs, je crois ouais.

*Organisatrice:* OK. Et justement, est-ce que vous avez eu un-- le sentiment qu'il y a une différence entre [euh] la première période et le deuxième ? Printemps/été et puis là...

*Participante:* Bah oui, je vais vite en approvisionnement [quoi]. Avant j'avais le marché [Nom du marché] au pied de chez moi donc j'y allais deux fois par-par semaine et je ne prenais pas grand-chose. C'était impeccable. Alors que là, ça m'est arrivé deux, trois fois de m'emballer un peu...

*Organisatrice:* Ouais, ouais.

*Participante:* Parfois, je fais une soupe, et puis je la congèle parce que je vois que les légumes commencent à faire des [inaudible 00:50:49 -00:50:50]. Il me reste-- il m'en reste de la soupe que j'ai déjà faite d'avant, mais j'ai acheté trop de légumes, donc-- [bah] J'ai un congel maintenant, donc je congèle sinon. Quand je vois que c'est en train de mal tourner. Je cuis, je congèle.

*Organisatrice:* Oui, ça, ce n'est pas mal.

*Participante:* Ça limite beaucoup les dégâts. Mais c'est vrai que je me rends compte que-- ouais, c'est un peu plus dur à gérer.

*Organisatrice:* La campagne...

*Participante:* [Bah] Ouais, la campagne, et puis mon métier aussi [hein]. Un coup je finis tôt, un coup je finis tard. Un coup je suis à droite, à gauche... C'est-C'est déjà un métier où il n'y a pas d'horaire, rien n'est régulier donc fatalement... Je vois des gens, ils ont leur semaine organisée comme du papier à musique. Moi, je ne sais jamais ce que je fais.

*Organisatrice:* Ouais. Oui, il ne peut pas y avoir : [ouais] tous les mardis 16:00...

*Participante:* [Bah] Non, c'est ça parce que [en fait] il y a plein de producteurs autour, mais c'est souvent le mercredi. [Alors] Quand je peux, [bah] j'y vais, mais parfois [bah] ce n'est pas-- ce n'est juste pas possible. Je termine plus tard, je ne suis pas dans le coin, je suis à l'autre bout... C'est comme ça [quoi].

*Participante:* Ils n'ont pas la bonne idée d'ouvrir le samedi matin, tous ceux qu'il y a dans le coin. Ça serait un peu plus pratique.

*Participante:* On fait un conseil-- [euh] on demande au conseil municipal.

*Participante:* C'est vrai que c'est souvent le mercredi... Le mecredi, 9:00 le dernier délai, [bah] ça fait super tôt.

*Organisatrice:* C'est vrai que ça fait...

*Participante:* Donc ouais, ça s'organise différemment mais...

**Household F19, Interview 1**

00:00:05
*Mme. 1:* L'enregistrement est parti. Toc. [Alors]. Donc, dans un premier temps, je vous propose un petit jeu--

00:00:13
*M. 1:* Ok.

00:00:14
*Mme. 1:* --de devinettes. Je vais vous demander de penser à un légume ou à un fruit--

00:00:18
*M. 1:* Ouais.

00:00:19
*Mme. 1:* --que vous aimez tout particulièrement. Vous allez penser à ce légume ou à ce fruit sans jamais me dire [euh] son identité.

00:00:25
*M. 1:* Ok.

00:00:26
*Mme. 1:* L'idée, c'est que je devine justement uniquement grâce à votre description.

00:00:31
*M. 1:* Ok.

00:00:31
*Mme. 1:* Est-ce que vous avez un... ?

00:00:33
*M. 1:* Ouais.

00:00:33
*Mme. 1:* Ok. Alors je vous écoute, dites-moi tout sur ce légume ou ce fruit.

00:00:34
*M. 1:* Je précise si c'est un légume ou un fruit--

00:00:40
*Mme. 1:* Vous pouvez.

00:00:41
*M. 1:* --ou je laisse le flou jusqu'au bout ?

00:00:40
*Mme. 1:* Vous pouvez, mais souvent quand on fait--

00:00:41
*M. 1:* [inaudible 00:00:45- 00:00:46] difficile sans le nommer [fin voilà].

00:00:49
*Mme. 1:* [rire] C'est ça.

00:00:53
*M. 1:* Donc, un petit fruit.

00:00:53
*Mme. 1:* Ouais.

00:00:53
*M. 1:* Rouge. Qui pousse sur les arbres. Acidulé.

00:00:53
*Mme. 1:* D'accord.

00:00:58
*M. 1:* Il pousse parfois en grappe de deux ou trois.

00:01:00
*Mme. 1:* Ok.

00:01:04
*M. 1:* On fait de la confiture.

00:01:08
*Mme. 1:* Mm-hmm.

00:01:08
*M. 1:* La saison est assez courte.

00:01:09
*Mme. 1:* Ok.

00:01:09
*M. 1:* C'est des fruits qui sont très fragiles... [silence 00:01:25- 00:01:32] [Hmm] Ouais j'ai dit rouge mais ça existe blanc aussi.

00:01:20
*Mme. 1:* D'accord. Et qu'est-ce que vous aimez tout particulièrement dans ce fruit ?

00:01:44
*M. 1:* Le goût.

00:01:52
*Mme. 1:* Le goût.

00:01:58
*M. 1:* Le goût ouais. La texture est sympa mais ça reste comme tous les fruits, c'est une texture aqueuse [quoi].

00:01:58
*Mme. 1:* Oui.

00:01:58
*M. 1:* Nan, le goût surtout.

00:01:58
*Mme. 1:* D'accord.

00:01:58
*M. 1:* Je pense que le fait de pas en manger souvent aussi ça doit jouer.

00:02:16
*Mme. 1:* Ok. Et à quel moment de la journée vous le mangez le plus souvent ? Est-ce que c'est plutôt le matin, l'après-midi, le midi, en grignotant ?

00:02:25
*M. 1:* C'est n'importe quand. Assez grignoté. Les petits fruits comme ça j'ai tendance à les grignoter, à en avoir sous la main et à en manger à longueur de journée.

00:02:28
*Mme. 1:* [rire] Ok. Je vois. [Euh] où l'achetez-vous le plus souvent, où est-ce que vous--

00:02:39
*M. 1:* En général je l'achète pas ouais.

00:02:39
*Mme. 1:* Ouais ?

00:02:39
*M. 1:* Je récupère ça chez des amis qu'ont l'arbre d'où ça vient.

00:02:39
*Mme. 1:* [rire] D'accord. Donc vous m'avez dit que vous le grignotez la plupart du temps, est-ce que vous pouvez me raconter un moment, où [voilà], le fruit est devant vous. Est-ce qu'il y a des étapes ou pas avant que vous le mettiez dans votre bouche, ou c'est--

00:02:50
*M. 1:* En général c'est assez- c'est assez direct [rires]. [Inaudible 00:03:12- 00:03:13] je vais avoir une barquette, un bol, peu importe, un contenant.

00:03:26
*Mme. 1:* Ouais.

00:03:26
*M. 1:* Comme ça, et puis [fin voilà] c'est comme les cacahuètes (quoi] [rires].

00:03:26
*Mme. 1:* Est-ce que y a du déchet dans ce fruit ?

00:03:28
*M. 1:* Ouais. Y a un noyau, et puis y a la tige.

00:03:35
*Mme. 1:* Ok. Ça se concrétise [rire]. [Hmm] Est-ce que vous avez l'impression qu'il vous apporte quelque chose, ce fruit, ou pas ? C'est simplement gustatif ou vous avez l'impression qu'il y a--

00:03:51
*M. 1:* [Bah] ça apporte du sucre, du bon sucre, donc ça apporte de l'énergie. [Euh bah euh fin] c'est un fruit, ça a des qualités nutritives certaines, bon après l'excès c'est pareil c'est plein de sucre donc-- [fin voilà] ça a ses limites [quoi]. C'est pas parce que ça m'apporte quelque chose d'un point de vue nutritif que je le mange. C'est un plaisir gustatif.

00:03:57
*Mme. 1:* D'accord et à l'inverse est-ce que vous lui trouvez certains inconvénients ou pas, à ce fruit ?

00:03:57
*M. 1:* [inaudible 00:04:25- 00:04:26]

00:03:57
*Mme. 1:* Parce que le fait que la saison soit courte finalement pour vous c'était presque un avantage, du fait du plaisir de le retrouver.

00:03:58
*M. 1:* [Fff bah] c'est ça, ça ajoute un petit truc, c'est comme les fraises quand la saison des fraises elle commence on est toujours contents parce qu'on va manger des fraises

00:03:58
*Mme. 1:* Oui. [Bon alors] je pense avoir trouvé [rires], je dirais la cerise.

00:04:00
*M. 1:* Ouais.

00:04:05
*Mme. 1:* [rire] Yes.

00:04:07
*M. 1:* [Voilà] contrairement aux bananes, on en mange toute l'année, y a moins ce côté--

00:04:12
*M. 1:* Ouais.

00:04:12
*M. 1:* --y a moins ce côté cool quand la saison elle commence, de toute façon c'est pas-- Y a pas de saison de la banane.

00:04:12
*Mme. 1:* Ah oui nan. Chouette. Alors maintenant, j'aimerais qu'on discute de vos pratiques d'achat et de votre organisation générale. Du coup, au sein de votre foyer qui est amené à faire les courses alimentaires ?

00:05:20
*M. 1:* [Euh] on a tendance à les faire ensemble. Mais [voilà], après s'il y a un désequilibre il est plutôt du côté d'[Nom de sa fille]. Elle a la voiture, c'est, [voilà]--

00:05:20
*Mme. 1:* D'accord.

00:05:35
*M. 1:* Quand on doit, [fin] quand ça arrive tout seul, à moins que ce soit d'aller à la superette vite fait pour acheter une plaquette de beurre parce qu'on n'a plus de beurre. S'il y a quelqu'un qui doit le faire tout seul, c'est [Nom de sa fille]--

00:05:46
*Mme. 1:* D'accord

00:05:46
*M. 1:* --elle a la voiture [fin bref] Mais sinon on va sur le marché ensemble en général, on essaye de faire les courses ensemble, et puis [ben) avec le coronavirus [et tout] on s'est mis pas mal à faire des commandes drive.

00:05:54
*Mme. 1:* Ok.

00:05:54
*M. 1:* Ce qui fait que [ben] on fait la commande ensemble et puis après on s'en va la chercher à deux soit [inaudible 00:06:00- 00:06:00], mais la commande est faite ensemble.

00:05:54
*Mme. 1:* Ok. [Euh] donc vous avez dit que donc là vous faites un peu de drive et vous allez aussi au marché ?

00:05:55
*M. 1:* Ouais.

00:05:55
*Mme. 1:* Donc vos fruits et légumes globalement, [fin] la majorité--

00:05:55
*M. 1:* Ils viennent du marché. Une fois par semaine ouais on achète des fruits et légumes.

00:05:55
*Mme. 1:* D'accord donc c'est- c'est régulier.

00:05:55
*M. 1:* Ouais.

00:05:56
*Mme. 1:* Toutes les semaines vous allez--

00:05:57
*M. 1:* Tous les samedis matin ouais.

00:05:57
*Mme. 1:* Le samedi matin.

00:05:59
*M. 1:* Alors c'est pareil ça arrive que des fois en semaine on aille à la superette acheter un ou deux légumes parce que [ben] on en a besoin pour un-- une recette ou ce qu'on a envie, [fin bref].

00:06:04
*Mme. 1:* Oui.

00:06:14
*M. 1:* Ça, [voilà], le marché exclut pas toutes les autres sources de provenance. Mais c'est vrai qu'on essaye de faire le maximum [bah] sur le marché. Acheter pour la semaine [quoi].

00:06:55
*Mme. 1:* Et du coup, justement, quand vous- quand vous allez au marché, est-ce que vous savez ce que vous allez acheter ou pas vraiment ?

00:07:03
*M. 1:* En partie.

00:07:03
*Mme. 1:* Ouais ?

00:07:04
*M. 1:* En partie ouais. Parce que [bah] c'est pareil, c'est saisonnier donc [bah] on sait que là, en ce moment, on va se mettre de plus en plus à acheter des courgettes par exemple. On a commencé à en acheter, ça va être quelque chose [bah] qui va revenir toutes les semaines. [Ben] on va acheter au moins une ou deux courgettes par semaine. Les navets c'est pareil, en ce moment on achète pas mal de navets, de carottes [fin voilà]. C'est-- On essaie- on essaie de coller à la saison, déjà, parce que les légumes sont meilleurs et puis oui on achète régulièrement les mêmess choses parce qu'au final [bah] y a des routines de repas qui se mettent en place d'autant qu'on- y a ma fille qui a 5 ans et [ben] c'est plus facile, pour éviter de se battre pendant les repas, que y ait des routines qui soient mises en place et puis que y ait voilà des trucs-- L'apprentissage du goût est important donc on essaie de faire découvrir voilà des nouveautés. Mais en gardant les valeurs, des trucs où on sait qui vont-- ça va être mangé sans difficulté [et tout]. Donc voilà, la courgette est un exemple parfait parce que [ben] déjà la saison commence. Lou elle adore ça. C'est un truc qui revient régulièrement, toutes les semaines, on va acheter des courgettes là jusqu'à la fin de la saison. Les carottes pareil, elle aime bien les carottes donc on achète des carottes très régulièrement. Parce que c'est pratique les carottes, ça se mange en crudité aussi. [Fin voilà], c'est- y a des routines--

00:08:31
*Mme. 1:* Oui.

00:08:31
*M. 1:* --d'achats, parce que y a des routines de repas [donc voilà] ça entraîne des routines d'achats parce que c'est pratique quand même, mine de rien, d'arriver sur le marché puis pas d'y passer une heure et demi en cherchant--

00:08:31
*Mme. 1:* [rire]

00:08:32
*M. 1:* --" qu'est-ce que je vais manger cette semaine ? "

00:08:32
*Mme. 1:* Donc, si je reprends, vous allez au marché [du coup] toutes les semaines, vous allez acheter [du coup] en fonction de ce qu'il y a en terme de saisonnalité avec néanmoins des choses récurrentes. Vous savez qu'il va y avoir des repas à faire avec courgettes, carottes etc.

00:09:21
*M. 1:* C'est ça ouais. Des trucs, des classiques, le navet pareil [fin bref], des exemples parmi tant d'autres, mais voilà, ouais, c'est ça. Ça nous permet de gagner du temps ouais déjà, parce que [bah] la réflexion sur ce qu'on va manger les semaines où on est juste tous les deux avec [Nom de sa fille] c'est moins compliqué parce que déjà on peut se permettre de sauter un repas, de grignoter--

00:09:38
*Mme. 1:* Oui.

00:09:39
*M. 1:* --de manger à 22 heures au lieu de manger à 19 heures [fin bref].

00:09:47
*Mme. 1:* Ouais.

00:09:48
*M. 1:* Y a beaucoup plus de souplesse. Quand on est avec [Nom de l'enfant] c'est plus rigide et nous [du coup ben] pour se libérer de la contrainte de "Qu'est-ce qu'on va manger ce soir ? Qu'est-ce qu'on va manger ce midi ? Qu'est-ce qu'on va manger ce soir ? Qu'est-ce qu'on va manger demain ? Qu'est-ce qu'on-- " [fin voilà]. Il y a des trucs qui se mettent en place [voilà] comme ça, des automatismes.

00:10:09
*Mme. 1:* Et [du coup], quand [bah] vous êtes sur le marché, vous savez toujours à peu près ce que vous allez acheter, comment vous décidez des quantités que vous prenez ? Est-ce qu'il y a un raisonnement ou est-ce que c'est au feeling ?

00:10:34
*M. 1:* [Ben c'est des pff], feeling, c'est avec l'habitude, de savoir que par exemple que les courgettes selon la taille qu'elles font avec deux ou trois courgettes je vais faire un repas, [voilà] selon la taille je vais en prendre tant. Après, il y a des trucs on prend toujours une botte de carottes, je sais pas la quantité, de toute façon, les carottes sont mangées quoiqu'il arrive. Elles partiront dans un plat en sauce, on les râpera, on fera [fin] y a pas d'inquiétude. Il y a des trucs comme ça on prend parce qu'on sait que ce sera mangé et que [fin voilà] peu importe de quelle façon, on les fera passer dans un plat [quoi]. Les navets c'est pareil, on prend tout le temps trois, quatre navets parce que- [bah] parce que trois, quatre navets c'est bien pour une semaine, ça passe bien pour une semaine, c'est une question d'habitude je pense. Je m'étais pas réellement posé la question quel était le-- ce qui me faisait choisir la quantité, [ff, ouais] je dirais plus feeling habitude [quoi], c'est--

00:11:40
*Mme. 1:* Ok.

00:11:40
*M. 1:* Et question d'envie.

00:11:41
*Mme. 1:* Ouais ?

00:11:41
*M. 1:* On a pris un kilo d'épinards parce que j'avais envie de manger des épinards.

00:11:42
*Mme. 1:* [Donc] il y a des choses que vous achetez habituellement parce que vous savez que vous allez pouvoir les cuisiner et que ça va plaire à [Nom de l'enfant]--

00:11:47
*M. 1:* Ben ouais c'est ça.

00:11:48
*Mme. 1:* Et est-ce que parfois ça vous arrive de prendre des choses qui étaient pas- pas prévues ou pas ? De se dire--

00:11:55
*M. 1:* Ouais, y a toujours- y a toujours. On se laisse-- On fait-- C'est la façon-- Que ce soit sur le marché ou en supermarché, on a une petite liste de trucs à acheter et puis on se permet des achats ce qu'on appelle l'achat impulsif [quoi]. [Donc] il y a toujours des petits trucs comme ça, que ce soit sur le marché ou autre où on se permet voilà un ou deux achats impulsifs--

00:12:16
*Mme. 1:* Ouais.

00:12:16
*M. 1:* --histoire de dire.

00:12:17
*Mme. 1:* [rire]

00:12:21
*M. 1:* Puis ça permet de découvrir des trucs.

00:12:23
*Mme. 1:* Ouais.

00:12:23
*M. 1:* Moi, j'ai tendance à acheter des conneries en achat impulsif. La dernière fois je suis tombé sur des bières avec des parfums incroyables, alors j'en ai pris.

00:12:31
*Mme. 1:* [rire]

00:12:31
*M. 1:* C'était dégueulasse, mais ça a satisfait ma curiosité, j'étais content, j'avais ma bière au guarana machin truc qui avait le goût de dentifrice, [voilà] [rires].

00:12:38
*Mme. 1:* Au moins c'est découvert.

00:12:38
*M. 1:* [Ben c'est ça], au moins la prochaine fois je l'achèterai pas parce que c'est pas bon, mais je sais voilà. Sur le marché, pareil, y a des fois on tombe sur un truc-- La dernière fois on est tombé sur un truc, on connaissait pas du tout comme légume. Une espèce de-- Ou alors c'était pas sur le marché, c'était magasin-- de navet/adis, entre les deux--

00:12:55
*Mme. 1:* D'accord.

00:12:55
*M. 1:* -- [euh ouais] navet/radis/betterave. Parce que c'est une betterave je crois. Mais c'est une betterave qui est plus dense, [fin je sais pas] c'était étrange comme légume, donc on l'a acheté et c'était vachement bon. C'était un navet un peu sucré avec un petit goût de betterave [quoi]. Mélange ouais navet/betterave. C'était super cool et on s'est acheté ça parce que- parce que quand on l'a vu on était là "Wow, connais pas" [rires]. On comprend pas, voilà le type d'achat impulsif.

00:13:31
*Mme. 1:* Ok [rire]. Et du coup, quand vous êtes face à ce légume [là] inconnu, comment vous faites après pour le consommer ?

00:13:43
*M. 1:* On regarde sur-- On demande à Google.

00:13:43
*Mme. 1:* Ouais, c'est Google qui--

00:13:46
*M. 1:* Ouais, c'est ça. Puis après des questions de- de proximité. Là on savait que c'était une betterave. Parce que c'était marqué que c'était une betterave. Ça ressemblait à du navet, donc on s'est dit oh on va essayer de faire un truc de cuisine [fin voilà] on va le cuisiner un peu comme on ferait avec une betterave crue ou un navet. On s'est fait un plat en sauce, on a mis ça dedans et voilà, nickel.

00:14:22
*Mme. 1:* Ok.

00:14:22
*M. 1:* Mais voilà Google sinon est notre ami. C'est un peut tout Google. Même pour la cuisson des légumes [rire].

00:14:23
*M. 1:* [Euh] du coup, vous avez parlé aussi d'un magasin bio. Ça vous arrive aussi d'y aller et de vous-- d'acheter des fruits et légumes aussi là-bas ?

00:14:31
*M. 1:* Ça nous arrive ouais on va, [alors] parce que les courses, c'est compliqué. On achète [donc] les fruits et légumes au marché à côté là, marché [inaudible 00:14:40- 00:14:40].

00:14:36
*Mme. 1:* Ok, oui, je vois.

00:14:37
*M. 1:* On va à Daoulas, au magasin bio qui s'appelle, bio quelque chose. Parce qu'on achète le vrac là-bas. On achète la farine, les pâtes, le riz [fin voilà] on achète le vrac là-bas. Donc du coup quand on y est y a des fruits et légumes aussi en vrac donc [bah oui] on en prend aussi. On n'y va pas souvent. Le vrac on en achète pas souvent, on a des gros bocaux, ça fait des gros stocks. Donc quand on y va on en prend aussi parce qu'on est sur place, parce que- parce que y a des trucs qui sont pas sur le marché parce que le marché là c'est vraiment marché local, bio et tout donc c'est cool pour [ben] faire les courses hebdomadaires (quoi].

00:15:18
*Mme. 1:* Ouais.

00:15:18
*M. 1:* Mais effectivement pour un petit plaisir, ils ont pas de kiwi par exemple, j'aime bien les kiwis [rires]. Mais c'est pas très local le kiwi, donc on en trouve pas sur le marché. [Donc voilà] pour ce genre de conneries ça arrive que [fin] oui pour ce genre de connerie quand on est au magasin bio on prend des kiwis, on prend un régime de bananes, on prend des trucs comme ça.

00:15:18
*Mme. 1:* Et du coup ce magasin vous y allez, en terme de fréquence c'est plus rare, c'est pas hebdomadaire, vous sauriez dire à peu près--

00:16:07
*M. 1:* Grosso modo je pense une fois par mois. Ouais, en voilà large mais ouais une fois par mois je pense.

00:16:17
*Mme. 1:* Et le drive qui arrive en complément, est-ce que pareil vous sauriez dire-- Environ.

00:16:27
*M. 1:* [Euh] Tous les quinze jours, ouais tous les quinze jours à peu près parce que y a les gâteaux pour [Nom de l'enfant], et voilà. Les gâteaux pour le goûter [rires]. Vu qu'elle est là, [fin voilà] qu'on la récupère tous les quinze jours je pense qu'on tape là-dessus à peu près. C'est pareil, c'est pas une science exacte, des fois on fait des gâteaux maison, [et voilà]. Puis des fois y a des gâteaux à finir parce que [bah] forcément des paquets de gâteaux on met ça dans un tupperware et puis maintenant qu'ils sont dans un tupperware [ben] ils ont pas le même goût, ils sont plus bons, [fin voilà].

00:16:39
*Mme. 1:* Je vois.

00:16:39
*M. 1:* [Donc voilà] faut dire "Nan tu finis les gâteaux qu'il y a dans les tupperwares avant qu'on achète d'autres gâteaux". Donc ça peut-être variable mais ouais une fois tous les quinze jours je pense c'est au niveau de ouais--

00:16:39
*Mme. 1:* Ça marche.

00:17:13
*M. 1:* --au niveau de l'échelle on doit être pas mal.

00:17:21
*Mme. 1:* Je vois. Et [du coup], on a parlé pas mal des légumes, mais est-ce que vos fruits, est-ce que c'est la même routine ? [Du coup] si vous m'avez dit les kiwis [du coup] en l'occurence c'est les fruits.

00:17:32
*M. 1:* Ouais. Les kiwis on n'en achète pas souvent, parce moi j'aime ça mais [Nom de sa fille] par exemple elle aime pas ça et en plus elle est un peu allergique aux poils du kiwi [donc fin bref].

00:17:32
*Mme. 1:* Ah oui. Ok.

00:17:32
*M. 1:* Donc c'est pas très souvent les kiwis mais on achète des bananes, parce que [Nom de l'enfant] aime bien parce que j'aime bien, parce que c'est une bonne source de magnésium, et puis parce qu'une fois qu'elles sont trop mures, ça passe très bien dans mes muffins [ou quoi], fin c'est vraiment difficile à perdre des bananes [quoi]. Vraiment, c'est un fruit qui est est quand même, faut y aller- faut y aller sérieusement pour être obligé de le jeter [quoi]. [Donc ouais] les bananes pas mal. Les pommes c'est assez cyclique parce que c'est pareil, moi j'ai pas l'habitude de manger des pommes comme ça. [Nom de l'enfant] elle aime beaucoup ça mais elle va manger une pomme et puis après de la semaine elle va plus en remanger donc quand on achète quatre pommes comme ça après [ben] je suis obligé de faire une compote, je suis obligé de faire voilà pour récupérer les pommes. Donc voilà les pommes de temps en temps. Les clémentines beaucoup l'hiver. Là ça commence à être fini mais on mange pas mal de clémentines. [Ben] là en ce moment des fraises, on achète des fraises toutes les semaines. J'aime beaucoup les fraises aussi c'est comme les cerises. Il faut pas en plus [hein], c'est pas bien de manger des fraises, c'est pas bon, je crois que c'est le fruit qui a le plus de pesticides résiduels dessus.

00:18:21
*Mme. 1:* Ah oui ?

00:18:21
*M. 1:* Ouais les fraises quand on mange des fraises on mange vraiment plein de saloperies avec. Mais j'aime vraiment beaucoup ça.

00:18:21
*Mme. 1:* C'est bon.

00:18:21
*M. 1:* [rires] Ouais c'est bon. Donc voilà donc je les achète-- Puis je me dis avec un peu de chance elles sont locales, elles sont bio [fff] mais c'est dégueulasse les fraises, c'est vraiment une industrie c'est vraiment une horreur.

00:18:21
*Mme. 1:* Ah oui ?

00:18:21
*M. 1:* Ouais, mais voilà puis j'en récupère, mon petit frère est maraîcher en Sarthe, donc j'en récupère de temps en temps.

00:18:21
*Mme. 1:* D'accord.

00:18:30
*M. 1:* Celles-là sont bonnes, pas d'intrants dedans. Et voilà, les cerises quand ça arrivera c'est pareil je vais essayer de récupérer mon stock de cerises. On mange des fruits séchés sinon.

00:18:30
*Mme. 1:* D'accord.

00:18:30
*M. 1:* J'achète des bananes séchées, de la mangue séchée, de l'ananas séché, des fruits séchés.

00:19:50
*Mme. 1:* Ok.

00:19:50
*M. 1:* Même la noix de coco, séchée, confite [inaudible 00:19:46- 00:19:47], plus un bonbon qu'un fruit. C'est vrai ça rentre plus dans la catégorie sucrerie là, séché, confit.

00:20:18
*Mme. 1:* Oui. Ok.

00:20:18
*M. 1:* Et puis là oui on est l'été, va arriver les pêches, les abricots, les brunions.

00:20:18
*Mme. 1:* Ouais.

00:20:18
*M. 1:* Que j'aime beaucoup aussi pas mal manger je pense.

00:20:21
*Mme. 1:* Les fruits l'été.

00:20:21
*M. 1:* Ouais les fruits jaunes là comme ça. Mais sinon ouais classique, pomme, poire, banane.

00:20:21
*Mme. 1:* Ouais ça c'est ce qui va venir toute l'année à peu près.

00:20:21
*M. 1:* Ouais c'est ça, c'est ce qui est facile, on se fournit facilement, la banane voilà]ça se perd pas, la pomme, la poire, la compote c'est vite fait au final, donc c'est pareil, ça se perd difficilement. Ça joue pas mal, surtout avec un enfant de cinq ans où [bah] la consommation est très aléatoire [quoi], et puis les arguments de raison sont un peu plus difficiles à prendre en compte quand on a 5 ans [quoi]. Tu dis ah ouais mais là, sur ton goûter t'as très envie de manger tes gâteaux au chocolat, mais, les fruits sont en train de moisir, la pomme commence à être toute molle, oui elle fait moins plaisir, elle donne vachement moins envie mais il faut la manger sinon c'est poubelle et poubelle c'est dommage. Ce genre d'arguments, plus difficile à accepter quand on a cinq ans.

00:21:07
*Mme. 1:* Ouais.

00:21:07
*M. 1:* Donc voilà, ça reste aléatoire.

00:21:12
*Mme. 1:* Je vois. [Euh] donc là l'idée c'est de dresser justement des listes de fruits et légumes. Vous m'avez déjà fait pas mal de listes. Si on reprend par exemple les fruits que vous mangez régulièrement, pommes, bananes, poires notamment, kiwis quand vous vous pouvez vous en procurer.

00:21:37
*M. 1:* Ouais de temps en temps, parce que ça coûte cher en plus les kiwis. Et puis, et puis voilà je suis le seul à en manger donc c'est pareil faut acheter ça en petite quantité [fin bref].

00:21:48
*Mme. 1:* Et du coup, après, quand vient la saison, les fruits, l'été, les pêches [tout ça], abricots, brunions, et [en fait] qu'est-ce qui-- Qu'est-ce qui guide vos choix ? Pourquoi ces fruits là vont être très réguliers et à l'inverse y en aura peut-être d'autres que vous allez moins consommer. Vous m'avez parlé du prix du kiwi.

00:22:08
*M. 1:* Ouais, puis y a des habitudes alimentaires je pense. Des fruits que je mangeais quand j'étais enfant, les fruits jaunes d'été par exemple c'est des fruits que je mangeais beaucoup quand j'étais enfant donc je pense qu'il y a voilà des habitudes alimentaires qui se mettent en place à cet âge-là. [Fin] "je pense", c'est sûr qu'il y a des habitudes alimentaires qui se mettent en place à cet âge-là mais qui sont assez prégnantes encore maintenant. Surtout sur la consommation de fruits je pense--

00:22:35
*Mme. 1:* Ouais.

00:22:35
*M. 1:* --qui est vraiment- qui est en tout cas, pour moi, une consommation alimentaire très axée sur le plaisir. Donc voilà, qui va être, contrairement aux légumes, qui va être plus une consommation avec [bah] avec-- Une consommation raisonnable, c'est-à-dire que je vais chercher à manger équilibré et sain. Alors, pas sur tous mes repas [hein] on est d'accord, je suis pas un... J'aime bien manger un bon burger avec une bonne assiette de frites et tout, mais ça va ensemble, c'est-à-dire qu'à côté de ça j'aime bien aussi me faire ma poêlée de haricots verts frais. [Fin voilà], il y a des habitudes alimentaires, je pense, énormément pour les fruits surtout, même pour les légumes au final. Et puis [euh bah] ouais j'ai grandi à la campagne, ça va être très local [quoi], surtout les légumes. Parce que j'ai grandi à la campagne mais j'ai pas grandi dans un verger, donc voilà j'avais des fruits, pruniers, y avait des prunes en été, [voilà] un pommier, un poirier, [et donc voilà].

00:23:41
*Mme. 1:* Oui.

00:23:41
*M. 1:* Puis les fraisiers, les framboisiers, tous les petits fruits de jardin. Mais sur les légumes, par exemple, je sais que, [ben] typiquement, les courgettes c'est un légume qui poussait dans mon jardin, que j'aimais beaucoup, l'été [voilà], des souvenirs de moi gosse en train de semer les pieds de courgettes [et tout]. À m'en occuper. Donc y a voilà y a un côté très, je sais pas, on va dire nostalgique même si c'est pas, fin voilà y a une habitude qui s'est mise en place, des routines [fin] des routines, ouais des habitudes alimentaires, et puis des trucs que je bouffais quand j'étais enfant et qui me plaisaient et que j'ai gardés. Y a des trucs que j'aime bien cuisiner aussi--

00:24:31
*Mme. 1:* Ouais.

00:24:31
*M. 1:* --qui sont, qui sont [voilà] que je vais prendre parce que soit j'aime bien les cuisiner soit ils sont simples à cuisiner, ils sont rapides à cuisiner. Après y a la curiosité--

00:24:31
*Mme. 1:* Ouais.

00:24:31
*M. 1:* --des trucs que je connais pas, j'ai envie de tester, une recette que j'ai vue, dont on m'a parlée. Je sais que le chou-fleu, par exemple, le chou-fleur que j'aime beaucoup [et ben] j'ai goûté chez la mère de mon ex un risotto de chou-fleur, donc un chou-fleur fait, râpé pour être fait façon risotto, j'ai trouvé ça super cool et du coup [ben] pendant l'hiver qui a suivi j'ai fait un chou-fleur par semaine quasiment [quoi].

00:25:22
*Mme. 1:* En risotto [rire].

00:25:22
*M. 1:* [Ben] voilà en risotto, après un chou-fleur, à cette époque là je vivais seul, [fin] je vivais seul la moitié du temps, donc un chou fleur par semaine en risotto, la moitié du chou-fleur déjà en risotto ça faisait déjà une belle quantité pour une personne. Parce que y a ça aussi, y a, pendant toute une période de ma vie, je vivais seul une semaine sur deux et avec un enfant de trois, quatre, cinq ans, une semaine sur deux, donc [bah] au niveau des achats, c'était [fin] voilà fallait faire attention à la quantité et à la taille. Un chou-fleu-- Un chou-fleur ça va vite au final de manger un chou-fleur. Mais un chou vert, par exemple--

00:26:00
*Mme. 1:* Ouais.

00:26:00
*M. 1:* --c'est vachement plus compliqué de se manger un chou vert tout seul avec une enfant de cinq ans en une semaine c'est raide [quoi], faut bouffer du chou souvent [quoi]. Y a des trucs comme ça où [voilà], là je me remets à, [fin voilà] je vis de nouveau en concubinage, c'est plus simple au niveau des quantité, même dans la façon de faire à manger au final [ben] ça joue. Je me permets de faire des plats qui sont plus imposants, déjà je suis plus tout seul à faire à manger, donc ça diversifie beaucoup plus les recettes et du coup les ingrédients qu'on met dedans, parce que- parce que [ben] on a chacun nos habitudes de cuisine et que du coup [ben] chacun nos habitudes d'ingrédients, ce qui fait que y a plus de diversité. Et puis- et puis y a plus de possibilités du fait qu'on est deux, au moins deux, à être présents et puis deux adultes, y a plus de possibilités de plats, on peut se permettre de faire des, par exemple des plats en sauce, des gros plats en sauce, que ce soit de légumes ou de viande, peu importe mais des gros plats en sauces qu'on va finir on va sortir on va avoir 1,5 ou deux kilos dans la casserole [ben] c'est pas grave parce qu'on va manger déjà le midi et le soir déjà on aura fait un bon sort au truc--

00:26:09
*Mme. 1:* Ouais.

00:27:32
*Mme. 1:* --et puis après le reste ça peut se congeler ou ça peut se manger une journée après [fin]. C'est plus facile d'envisager de faire la cuisine quand on sait qu'il y a deux adultes, c'est [voilà]-- C'est beaucoup plus simple. Moi je sais que c'était le problème quand j'étais seul avec [Nom de l'enfant], c'est que [ben], vite, on se replie sur les plats préparés--

00:27:54
*Mme. 1:* Ouais

00:27:54
*M. 1:* --sur les plats préparés, pas forcément les plats préparés, mais des trucs qui sont quand même très industrialisés, très transformés. Le poisson pané, des trucs comme ça--

00:28:03
*Mme. 1:* Ouais.

00:28:03
*M. 1:* --on va vite retomber là-dessus. Parce que [ben]-- Parce qu'au niveau des quantités c'est plus facile. Parce que [fin voilà]-- Parce que y a beaucoup plus de facilité quand on vit seul, seul, avec un enfant, à consommer ce genre de trucs--

00:28:17
*Mme. 1:* Oui.

00:28:17
*M. 1:* --que à consommer [ben] les produits qu'il va falloir toucher, transformer nous-mêmes. Et puis moi j'arrive pas à faire un repas en mettant une carotte, une patate, un navet dans un plat, j'y arrive pas, j'arrive pas à doser, après j'arrive pas à épicer, j'arrive pas à saler, j'arrive pas à-- je sais pas faire [quoi].

00:28:26
*Mme. 1:* C'est plus facile de faire des gros plats.

00:28:26
*M. 1:* Oui, c'est plus facile de prévoir à-- au moins, un truc qui se mange à quatre, cinq, même si on est que deux on sait que quatre, cinq ça se mange avant de le perdre.

00:28:52
*Mme. 1:* Ouais.

00:28:53
*M. 1:* Alors que quatre, cinq tout seul [ben] je sais que je vais perdre. Donc je vais pouvoir faire les efforts que je veux [ben] je vais en manger quatre repas de suite, mais le quatrième repas ce sera déjà plus très frais et puis va falloir que je m'en sépare parce que je vais être malade à force [quoi].

00:29:04
*Mme. 1:* Ouais.

00:29:08
*M. 1:* Donc voilà, mais du coup- du coup ça permet de changer les habitudes de-- d'achat. Vous seriez venue me voir [ben] il y a un an, mes habitudes d'achats, ma consommation de fruits et légumes était complètement différente parce que-- [ben] parce que j'étais bloqué par certains trucs. Et puis parce que voilà faut voir aussi que les trucs industrialisés, style poissons panés, surgelés préparé et tout, ça coûte vraiment peau de balle [quoi], vraiment au kilo.

00:29:37
*Mme. 1:* Mm-hmm.

00:29:37
*M. 1:* On arrive à se faire de la bouffe. [Alors] c'est pas bon, c'est pas bon pour la santé, c'est pas bon gustativement [et tout], mais [ben] on se nourrit pour pas cher [quoi].

00:29:37
*Mme. 1:* [Ben] oui.

00:29:37
*M. 1:* Alors que là [ben] ouais les fruits et légumes c'est un peu-- C'est pas excessif mais ça demande un peu plus d'argent, mais ça demande surtout du temps. Il faut avoir le temps de se, [ben] le temps de les préparer les légumes, le temps de réfléchir à ce qu'on va faire avec, le temps de les éplucher, de les nettoyer, de les éplucher, de les préparer [fin voilà], faire en sorte de les conserver, y en a certains, c'est pas dur, ça reste dans le panier et puis ça se conserve, mais-- La salade par exemple c'est pareil, quand on achète une salade faut avoir envie de la manger en entier, ou alors sinon faut avoir des moyens de conservation. Moi je savais pas du tout comment conserver ma salade avant qu'[Nom de sa fille] me montre une technique miraculeuse qui fait que la salade elle tient 15 jours au frigo sans flétrir. Mais avant ça moi c'était impossible pour moi d'acheter une salade.

00:30:38
*Mme. 1:* Ouais.

00:30:39
*M. 1:* Je pouvais pas me permettre de m'acheter une salade parce que, outre le temps de l'éplucher, de la laver et tout, [fin], c'est anecdotique, parce qu'après je me retrouvais avec une salade entière [quoi]. Faut aimer ça.

00:30:51
*Mme. 1:* Ouais, complètement.

00:31:00
*M. 1:* J'aime bien la salade mais manger une salade comme ça entière en deux, trois jours [quoi]--

00:31:00
*Mme. 1:* C'est ça.

00:31:00
*M. 1:* --ça passe vite au final, c'est pareil faut s'accrocher [quoi]. Faut en avoir vachement envie de la salade.

00:31:00
*Mme. 1:* Et justement la salade là c'est quoi votre stratégie de conservation ? C'est de faire--

00:31:02
*M. 1:* [Ben alors] je connaissais pas ça, donc on l'épluche--

00:31:16
*Mme. 1:* Ouais.

00:31:16
*M. 1:* --on la nettoie. Normal. On la rince. Et après on met ça dans un pochon, dans un sachet plastique avec une ou deux feuilles de Sopalin pliées dedans. Et [en fait] la feuille de Sopalin elle va faire tampon au niveau de l'humidité--

00:31:34
*Mme. 1:* Ok.

00:31:38
*M. 1:* --elle va se remplir, et puis elle va se vider au fur et à mesure et les feuilles elles vont rester- elles vont rester fraîches. Elles vont pas se flétir. Et ça marche très bien avec la menthe, ça marche très bien avec le persil. Ça marche très bien avec-- Donc on fait ça aussi. Bon après la menthe et le persil ça se congèle aussi.

00:31:38
*Mme. 1:* Mm-hmm.

00:31:38
*M. 1:* La salade je pense ça se congèle pas. Mais ça permet de- ça permet de ouais de conserver toutes ces herbes, feuilles, au frigo, dans un sachet plastique et puis ça va ça reste vert ouais dix, 15 jours [quoi]. Frais et croquant. C'est assez cool.

00:31:38
*Mme. 1:* [Bah] oui du coup la salade c'est tout un combat, la salade justement sur la conservation.

00:32:24
*M. 1:* Nan là c'est dingue, c'est assez-- C'est assez cool. Je connaissais pas du tout. Ça vient de loin.

00:32:24
*Mme. 1:* Ah oui ?

00:32:24
*M. 1:* Ouais, elle avait appris ça d'un ami Brésilien qui vit en Suisse, [fin]--

00:32:39
*M. 1:* [rire] [inaudible 00:32:36- 00:32:37] ça vient de loin.

00:32:39
*M. 1:* Voilà c'est une technique d'un ami brésilien vivant en Suisse, il l'a peut être appris en Asie, [fin voilà], un truc, mais ça marche très très bien. C'est redoutablement efficace. C'est tellement simple de mise en œuvre en plus, [fin voilà] c'est pas la technique qui demande... Nan, je fais comme d'hab avec la salade, sauf qu'à la fin elle est dans un pochon plastique, pochon Ikea un litre et quelques, on met ça avec deux feuilles de Sopalin pliées dedans--

00:33:04
*Mme. 1:* Et ça marche.

00:33:07
*M. 1:* --et dans le bac à légume du frigo. Et ça marche.

00:33:16
*Mme. 1:* Chouette. Je note, j'essaierai [rire].

00:33:16
*M. 1:* Redoutable.

00:33:17
*Mme. 1:* Redoutable [rire].

00:33:17
*M. 1:* Redoutable d'efficacité.

00:33:22
*Mme. 1:* [Hum] et vous avez quoi d'autre dans le réfrigérateur ? On s'intéresse à tous vos fruits et légumes et leurs différents lieux de stockage.

00:33:30
*M. 1:* [Euh], réfrigérateur, en terme de fruits et légumes on va pas avoir grand chose, on va avoir de la salade--

00:33:40
*Mme. 1:* Ouais.

00:33:40
*M. 1:* --des champignons, et de temps en temps les carottes par exemple quand elles commencent à devenir vraiment molles, les radis pareil quand ils commencent à devenir vraiment vraiment tout mous ils passent leur dernière journée au frigo, et encore--

00:33:40
*Mme. 1:* Ouais ?

00:34:03
*M. 1:* --ils passent leur dernière journée au frigo avant d'être mangés sur le champ. Surtout on les refait tremper dans l'eau pour les radis c'est pareil, c'est redoutable, le radis tout mou là.

00:34:03
*Mme. 1:* Dans l'eau ?

00:34:03
*M. 1:* Dans un bol d'eau ouais, avant de le re-manger ouais. Au niveau de la conservation, je sais pas si ça se conserve plus longtemps dans l'eau ou pas, mais quand il devient tout mou un peu flétrie là, une demi heure dans l'eau, et puis il redevient-- Il reprend un peu de sa forme, il redevient croquant. C'est pareil c'est pour reconsommer tout de suite. Moi j'aime pas trop ça le radis donc ça--

00:34:18
*Mme. 1:* [rire]

00:34:21
*M. 1:* Mais ça marche, [Nom de l'enfant] par exemple elle les bouffe après les radis comme ça, sans problème.

00:34:21
*Mme. 1:* Ok. Et du coup vous faites quoi des autres--

00:34:21
*M. 1:* Fruits et légumes ?

00:34:21
*Mme. 1:* Ouais. Vous les mettez où les autres fruits et légumes ?

00:34:50
*M. 1:* Ils sont par terre dans une cagette.

00:34:50
*Mme. 1:* Oui. À température ambiante du coup ?

00:34:50
*M. 1:* Mm-hmm. Ouais, dans la cuisine donc voilà ils prennent pas la lumière directe, mais ils sont pas particulièrement, je fais rien de particulier, c'est des légumes-- Ils sont -- Normalement c'est des légumes c'est l'avantage aussi d'acheter ça en local, normalement ils ont pas passé deux mois au frigo, donc ils ont pas [fin voilà]-- Ils ont pas un retard à rattraper, ils pourrissent pas comme ça, ils se tiennent. Ils tiennent la semaine. En fin de semaine ils font un peu la gueule, mais ils tiennent la semaine, ils sont complètement consommables. Là depuis que vous êtes venue, que vous avez installé la poubelle on a mis dedans, on a dû mettre trois artichauts parce que on a craqué, on a acheté trois minis-artichauts, c'était des tout petits artichauts, et je suis parti, j'avais une formation après je suis parti pendant trois jours, et du coup [Nom de sa fille] elle aime pas les artichauts, elle s'est retrouvée toute seule avec [Nom de l'enfant] [fin bref].

00:35:36
*Mme. 1:* Oui, et ces trois artichauts...[rire]

00:35:36
*M. 1:* Poubelle. Trois artichauts, et je crois que c'est tout ce qu'on a mis dans la poubelle depuis que vous nous l'avez déposée, ces trois artichaut. Ah nan, nan nan ! On a mis cinq, six radis aussi.

00:35:36
*Mme. 1:* Ah ! [rire]

00:35:36
*M. 1:* Qui étaient passés. Vraiment, qui étaient passés. Mais voilà on en jette très très peu des fruits et légumes.

00:35:36
*Mme. 1:* Ouais. Parce que du coup là donc vos fruits et légumes ils tiennent la semaine--

00:35:37
*M. 1:* Ouais.

00:35:37
*Mme. 1:* Est-ce que selon vous tout de même, vos stratégies de conservation, à part la salade qui est redoutable--

00:36:34
*M. 1:* Redoutable ouais [rire].

00:36:34
*Mme. 1:* Est-ce que c'est des stockages des lieux de stockages idéaux ou pas, selon vous est-ce que c'est--

00:36:34
*M. 1:* Je pense que y a moyen de faire mieux. Je sais que les garde-mangers à l'époque où y avait pas de réfrigérateur par exemple, c'était des lieux qui était un peu plus réfléchis qu'une cagette sous le... [Fin voilà], des questions-- Taux d'humidité je pense y a moyen de faire. Je sais que-- Je sais que ça se conserve mieux dans le CO2 par exemple, mais là ça devient carrément plus complexe de mise en place de faire des lieux de stockage au CO2, mais [euh]- mais oui le frais par exemple, ça conserve mieux donc il y a des possibilités. Mais moi mon frigo il est au minimum, il fait vraiment trop froid [quoi]. Mais j'ai vu là dernièrement, c'était peut-être sur Brut ou une connerie comme ça, deux ingénieurs qui avaient construit une maison "autonome" entre guillemets, donc pas d'électricité et, [fin bref], un truc qui était assez réfléchi. Et pour leur garde manger vu qu'ils avaient pas de frigo ils avaient fait un truc qui donnait à l'extérieur de la maison, donc en dehors de l'isolation pour rester au frais, et qui était orienté de façon à ce que le soleil ne tape jamais dessus. Et donc, effectivement, ça doit être plus performant en terme de conservation que les méthodes qu'on utilise.

00:37:43
*Mme. 1:* Ouais.

00:37:43
*M. 1:* Il peut y avoir beaucoup de méthodes plus performantes.

00:37:45
*Mme. 1:* Ouais.

00:37:46
*M. 1:* Mais l'avantage de celle là, c'est que déjà, c'est la notre, que [ben] on s'en sort très bien avec, c'est à dire que y a vraiment peu de ratés [quoi], et qu'elle est très peu contraignante. Donc elle a quand même beaucoup d'avantages, pour peu d'inconvénients, parce que [bah] ça prend pas de place.

00:38:08
*Mme. 1:* Ouais.

00:38:09
*M. 1:* Et on perd pas, on jette pas [quoi]. Donc à partir de ce moment là, y a probablement des méthodes plus performantes, mais qui répondent aussi bien au cahier des charges, je sais pas.

00:38:24
*Mme. 1:* Ouais. Oui, c'est un système qui vous convient très bien--

00:38:26
*M. 1:* [Ben] c'est ça, c'est-à-dire que quand on aura une maison, qu'on aura un jardin et qu'on fera des récoltes, la problématique se posera différemment--

00:38:34
*Mme. 1:* Ouais.

00:38:34
*M. 1:* --parce que, effectivement, on aura une autre problématique de conservation, on aura trop par rapport à ce qu'on consomme, où en tout cas on n'aura pas-- Là on achète pour la semaine [quoi]. Donc c'est pas dur de conserver pour la semaine. Donc [voilà] y aura d'autres problématiques de conservation qui pour le moment [ben] sont pas les nôtres [quoi]. Donc effectivement, mais à ce moment là oui quand on aura un jardin et qu'on va avoir [ben] quatre kilos de courgettes qu'on va devoir récolter au même moment [ben] là oui va falloir trouver un truc pour les-- pour conserver ces quatre kilos de courgettes. Mais après, c'est pareil. On a déjà des idées comment on va faire--

00:39:07
*Mme. 1:* Oui.

00:39:07
*M. 1:* --on va transformer, on va faire des bocaux, on va [fin voilà], on fait une ratatouille. Ratatouille en bocal ça tient deux ans.

00:39:22
*Mme. 1:* Oui.

00:39:22
*M. 1:* On fait attention, pas de [inaudible 00:39:18- 00:39:18] et puis [voilà] c'est bon [quoi].

00:39:22
*Mme. 1:* Oui, c'est ça, ce qui fait que là même si c'est pas forcément-- C'est un lieu idéal pour vous dans votre organisation--

00:39:28
*M. 1:* Voilà c'est ça.

00:39:31
*Mme. 1:* --dans votre aménagement et par votre organisation interne d'achats, vu que vous êtes-- que vous achetez toutes les semaines effectivement, s'ils tiennent la semaine.

00:39:33
*M. 1:* C'est ça. Y a pas besoin de mieux [quoi]. Ce serait rajouter une contrainte là où y a pas besoin d'augmenter l'efficacité donc, voilà, le cahier des charges est bien rempli [quoi].

00:39:51
*Mme. 1:* Et les-- Donc les légumes sont dans une cagette, vos fruits ils sont--

00:39:56
*M. 1:* Les fruits, [alors] les bananes sont à part, parce que ça dégage je sais plus comment ça s'appelle [bref] le [inaudible 00:40:04- 00:40:05] qui fait pourrir [fin], qui fait mûrir les fruits et légumes. Donc les bananes sont à part, elles sont crochetées dans un coin dans la cuisine. Les fruits sont dans un bol, dans un [voilà] dans un bol--

00:40:20
*Mme. 1:* Corbeille ?

00:40:20
*M. 1:* --corbeille, [voilà], ça dépend des jours, ça dépend ce qui est propre, ça dépend [voilà].

00:40:20
*Mme. 1:* Oui. À température ambiante.

00:40:20
*M. 1:* C'est ça, ouais, pareil, à température ambiante. Ça dépend combien y a de fruits, ça dépend de pas mal de trucs. Ils sont dans un contenant sur la paillasse de--

00:40:23
*Mme. 1:* Posés dans la cuisine.

00:40:23
*M. 1:* -- Posés dans la cuisine c'est ça.

00:40:38
*Mme. 1:* Ok.

00:40:44
*M. 1:* Au niveau de la conservation il y a pas une attention particulière qui est mise à partir du moment où on perd pas. C'est à dire que effectivement, si on commence à perdre sur deux, trois achats de suite une quantité de légumes et de fruits, là on va se poser la question de soit on arrête d'en acheter, soit on le conserve différemment, on le consomme différemment. À partir de ce moment là, on va se poser la question. Mais temps que ça roule, c'est simple [quoi], on se prend pas la tête. On n'aime pas jeter donc on s'en rend compte tout de suite aussi. Vu que c'est pas-- Qu'on le fait pas, que c'est pas naturel et tout, dès qu'on va se mettre à jeter des produits frais comme ça, tout de suite on va tiquer et puis on va se dire qu'il y a un problème, qu'il y a un truc qui a déconné quelque part et donc [ben] essayer de rectifier le tir assez rapidement parce que, [ben]- parce qu'on n'aime pas ça. Parce que y a aussi cette conscience écolo, si on peut dire, de [ben], c'est débile de- d'acheter des trucs, de, ouais, de les acheter pour les jeter [quoi], c'est une perte d'argent, c'est une perte de tout parce que [ben] parce que ça a pousé dans des conditions qui sont, on le sait, dans la plupart des cas-- des conditions qui sont pas exceptionnelles [quoi], qui sont pas au top pour l'environnement. C'est quand même très, très rare de trouver dans ce genre de distribution, même dans un marché local bio et tout-- Dans ce genre de distribution c'est quand même très rare de trouver un truc qui a poussé dans des conditions qui sont acceptables, donc ce serait con de, en plus, les jeter par négligence. Donc ouais ça nous heurte à chaque fois qu'on est obligés de les jeter, donc du coup on y fait attention, quitte à se déplacer plus souvent pour aller en acheter plus souvent. On gère le truc comme ça. On n'aime vraiment pas ça jeter. Mais ça marche avec tout [hein].

00:42:42
*Mme. 1:* Oui, pas que les fruits et légumes.

00:42:55
*M. 1:* C'est pour ça que- c'est pour ça qu'on achète en vrac aussi la plupart du temps, parce que ça limite la quantité de déchets, ça limite la quantité de plastiques, ça limite...

00:43:08
*Mme. 1:* Oui il y a une attention particulière aux déchets de manière générale.

00:43:10
*M. 1:* De manière générale ouais, mais à notre environnement [en fait], ouais que ce soit ouais les déchets ouais tout, moi j'ai ma veste j'ai mes mégôts de cigarettes qui sont dedans par exemple. C'est dégueulasse, ça pu [fin voilà] c'est assez particulier mais [ben] je jette pas mes mégôts de cigarettes comme ça par terre, parce que ça se fait pas, parce que c'est crad' [quoi]. Je préfère avoir la poche qui pue et effectivement avoir [ben] effectivement quand je mets ma veste ça sent le cendrier mais à la limite j'assume cette odeur dégueulasse plus facilement que le fait de jeter mes mégôts dans la nature.

00:43:14
*Mme. 1:* Ouais.

00:43:50
*M. 1:* Voilà l'attention elle est globale [quoi]. On fait gaffe à notre environnement. Des gens assez respectueux des autres et de notre environnement et de l'environnement des autres et [fin voilà]-- C'est assez naturel au final. [Fin] ça nous paraît assez naturel. Je vais pas dire que de manière générale c'est naturel, mais de la façon dont on le fait c'est assez naturel. On se force pas, on se dit pas de faire ça pour être des parents vertueux ou quoi que ce soit, on fait ça parce que, [ben] parce que c'est comme ça qu'on a une notion de ce que la vie devrait se faire-- devrait être [quoi]. Et puis parce qu'on a un enfant mine de rien, [fin] "on a un enfant", c'est pas l'enfant d'[Nom de sa fille] mais on l'éduque ensemble. Et [Nom de sa fille] et moi et sa mère et son compagnon. Et [ben)-- Et que c'est important aussi pour l'éducation d'un enfant de savoir que [ben] que on peut pas tout se permettre, que les choses ne sont pas renouvelables--

00:44:18
*Mme. 1:* Ouais.

00:44:18
*M. 1:* --comme on cherche à nous le faire croire. Que le côté renouvelable c'est une arnaque, c'est marketing [quoi] le renouvelable.

00:44:18
*Mme. 1:* Mm-hmm.

00:44:18
*M. 1:* C'est pas renouvelable, on a un impact sur notre environnement. Maintenant, reste à savoir quel impact on veut avoir-- quel impact on tolère sur notre environnement et donc voilà se rendre compte que quand on fait telle chose, on a tel impact, est-ce que cet impact là, il est tolérable, est-ce que par rapport à ce qu'on fait c'est, [fin bref] toute une réflexion sur, [ben] sur notre impact [quoi], sur ce qu'on fait vivre à notre entourage, qui soit végétal, animal, humain peu importe.

00:45:22
*Mme. 1:* Ouais.

00:45:22
*M. 1:* C'est des notions qui sont importantes à avoir, sans aller dans un écologisme militant, particulier, ou quoi que ce soit, c'est juste ouais une question [ben] de savoir ce qu'on fait, et puis de perenité quoi. Moi j'ai fait un enfant, [euh euh alors] avec une ambition, mais j'ai fait un enfant, je sais que c'est pas pour lui dire [ben] notre façon de vivre elle est pas pérenne--

00:45:22
*Mme. 1:* Mm-hmm.

00:45:22
*M. 1:* --et démerde-toi avec ça, bon courage. Ouais notre façon de vivre elle est pas pérenne, ça c'est clair, [fin] la façon de vivre de la société elle est pas pérenne, donc, [ben] donc j'essaie de lui montrer un exemple de façon de faire qui pourrait avoir ouais une potentielle pérennité ou en tout cas, qui a une façon d'envisager et de réfléchir à comment ça se passe, qui va vers plus de pérennité. Et voilà, sans [inaudible 00:46:37- 00:46:38] moi les écueils marketing style [ben] machins renouvelables [quoi], ceux qui sont [fin], fallacieux.

00:46:42
*Mme. 1:* [rire]

00:46:42
*M. 1:* [rire]

00:46:48
*Mme. 1:* Ok. Ça marche, [hum] sur le-- sur cet aspect de l'environnement et du gaspillage en général on y reviendra, j'ai une question avant. [Euh] sur la façon dont vous préparez et consommez vos fruits et légumes. Vous m'en avez déjà un peu parlé par exemple de certaines recettes : le risotto au chou-fleur, etc. Si on prend un autre légume que vous avez dit manger régulièrement, par exemple, y avait la courgette, est-ce que vous pouvez me raconter justement la façon-- Sous quelle forme vous la consommez cette courgette ?

00:47:47
*M. 1:* Ouais la courgette c'est assez simple. [Fin] ça, doit y avoir deux grandes façons pour la courgette--

00:47:49
*Mme. 1:* Ouais ?

00:47:49
*M. 1:* --après selon on n'est jamais à l'abri d'une recette qui en met dedans-- Mais la courgette peut poêlée avec de l'ail et du persil.

00:47:52
*Mme. 1:* Ok.

00:47:55
*M. 1:* [Voilà] La façon la plus souvent. La plus souvent c'est ça et sinon après si c'est des courgettes rondes ou des grosses courgettes, courgettes farcies.

00:48:05
*Mme. 1:* Ouais.

00:48:05
*M. 1:* Mais voilà mais globalement, c'est poêlée avec de l'ail et du persil.

00:48:18
*Mme. 1:* Ok.

00:48:18
*M. 1:* La courgette je la lave.

00:48:18
*Mme. 1:* Ouais.

00:48:18
*M. 1:* Je la coupe en quatre, je fais des cubes, je mets ça dans la poêle, je fais dorer, avec de l'ail, et puis une fois que c'est fini je rajoute un peu de persil.

00:48:35
*Mme. 1:* Ok. Donc le plus souvent en poêlée.

00:48:35
*M. 1:* Ouais.

00:48:35
*Mme. 1:* Sinon farcies. Est-ce que y a d'autres recettes qui vous vient-- Nan ? Ça va être vos--

00:48:35
*M. 1:* Nan c'est vraiment-- ça va être nos deux. Après des fois on fait des ragoûts de légumes--

00:48:37
*Mme. 1:* Oui.

00:48:37
*M. 1:* --donc s'il y a une courgette qui traîne encore, dans les légumes, ça peut complètement passer dans le ragoût de légumes [quoi]. C'est pas, y a pas de, [fin voilà], c'est-- De la courgette ça passe bien pareil, de tous les côtés donc--

00:48:56
*Mme. 1:* [rire]

00:48:56
*M. 1:* Voilà, ça y va selon. Selon.

00:49:00
*Mme. 1:* Ok.

00:49:01
*M. 1:* Mais quand elles sont achetées, elles sont achetées plus tôt pour [fin voilà] pour faire une poêlée et sinon ça va être des courgettes qui vont être achetées pour faire des courgettes farcies.

00:49:11
*Mme. 1:* Oui les rondes.

00:49:18
*M. 1:* Voilà. C'est ça, les grosses rondes [fin] les "grosses", plus ou moins grosses mais [voilà] celles qui ont une forme.

00:49:18
*Mme. 1:* Et du coup, vous avez dit, vous la-- En terme d'étapes de préparation, vous la lavez--

00:49:25
*M. 1:* Ouais.

00:49:26
*Mme. 1:* Et vous la coupez.

00:49:29
*Mme. 1:* Ouais.

00:49:33
*Mme. 1:* Vous l'épluchez pas ?

00:49:33
*M. 1:* Nan. Je mets à la poêle la courgette.

00:49:33
*Mme. 1:* Ok. Et est-ce que y a quand même de l'épluchure quand vous la préparez cette courgette ?

00:49:37
*M. 1:* [Ben] y a les trognons, [donc voilà] le cul et la tige qui sont retirés et voilà c'est tout.

00:49:49
*Mme. 1:* Ok. Et si on prend un fruit que vous avez dit consommer régulièrement-- Vous avez déjà, par exemple la pomme.

00:49:59
*M. 1:* Ouais.

00:49:59
*Mme. 1:* La pomme. Vous avait dit déjà en faire des compotes.

00:50:06
*M. 1:* Ouais, quand je les perds.

00:50:08
*Mme. 1:* Ouais. Ça va être quoi par exemple les différentes façons de consommer la pomme ?

00:50:26
*M. 1:* [Alors, ben] classique, on croque dedans [euh], peu de gâteaux. Pas de gâteaux. On fait pas de gâteaux avec les pommes [inaudible 00:50:22- 00:50:24] [rires] donc ouais croquer, [Nom de l'enfant] des fois je lui épluche, mais de moins en moins. Ça dépend des pommes [en fait]-- ça dépend d'où elle viennent. Je les frotte très très fort.

00:50:28
*Mme. 1:* D'accord.

00:50:36
*M. 1:* J'ai fait une saison ouvrier agricole--

00:50:39
*Mme. 1:* Ah oui.

00:50:39
*M. 1:* --j'ai ramassé des pommes et j'avais les mains rouges, brûlées en fin de journée à cause des produits chimiques qu'il y a sur les pommes.

00:50:46
*Mme. 1:* D'accord.

00:50:46
*M. 1:* Donc je les frotte très, très intensément avant de lui donner à manger, elles sont brillantes comme les pommes dans La Belle au bois dormant.

00:50:56
*Mme. 1:* [rire]

00:50:57
*M. 1:* Elles sont vraiment-- elles sont brillantes. Je les lustre. Mais parce que c'est bourré de produits chimiques, c'est dégueulasse les pommes de verger comme ça. Mais sinon, je les épluche et je les coupe en quartiers.

00:51:09
*Mme. 1:* Ok.

00:51:09
*M. 1:* Voilà, ça, ou nature comme ça ou des fois avec un peu de sucre vanillé parce que c'est quand même assez bon avec un peu de sucre vanillé.

00:51:22
*Mme. 1:* Ouais.

00:51:22
*M. 1:* Donc voilà, croquer, couper, éplucher, sinon compote.

00:51:22
*Mme. 1:* Ouais sinon compote ? Ok. Ça marche. [Hum] est-ce que vous avez en tête des fruits ou des légumes que vous allez pouvoir consommer, mais sans qu'il y ait d'épluchures ? Sans, ouais, sans épluchures.

00:51:48
*M. 1:* Sans zéro épluchure ?

00:51:48
*Mme. 1:* Oui. C'est une question qu'on, justement, qu'on se pose [rire].

00:51:48
*M. 1:* [Ben] y a, la framboise.

00:51:51
*Mme. 1:* Oui.

00:51:51
*M. 1:* Où il n'y a pas d'épluchures. Et, c'est ça, ça va être difficile d'en trouver d'autres. Parce que la fraise y a la queue. [Euh], nan parce qu'après y a des noyaux.

00:52:07
*Mme. 1:* Ouais c'est ça.

00:52:08
*M. 1:* Ya des grappes, y a des-- Pas d'épluchure ?

00:52:08
*Mme. 1:* Effectivement la framboise.

00:52:17
*M. 1:* J'ai des amis qui mangent les pommes, tout.

00:52:25
*Mme. 1:* Tout ?

00:52:32
*M. 1:* Tout. Ouais, tout. Trognon, queue comprise. Donc y a pas d'épluchures là-dedans, la pomme elle est finie. Mais c'est hardore je trouve quand même [rires].

00:52:32
*Mme. 1:* C'est possible [rire].

00:52:32
*M. 1:* C'est possible mais c'est hardcore.

00:52:32
*Mme. 1:* Oui.

00:52:44
*M. 1:* [Euh], nan, nan. Mais après ça dépend où est-ce qu'on place la tomate là-dedans.

00:52:44
*Mme. 1:* Oui. Oui, parce que la tomate--

00:52:47
*M. 1:* Mais bon c'est quand même un légume. C'est rarement des--

00:52:58
*Mme. 1:* Même en légume c'est justement- c'est, ceux qu'on n'épluche pas.

00:53:05
*M. 1:* Des légumes où y a pas non plus de, pas du tout du tout de trucs qu'on jette.

00:53:11
*Mme. 1:* Ouais, comme si on n'avait pas besoin de préparer quoique ce soit dessus. Effectivement la tomate--

00:53:27
*M. 1:* La tomate ouais. Des fois y a la queue de la tomate, pareil c'est, c'est-- Légumes...

00:53:27
*Mme. 1:* [Bah] la tomate et la framboise.

00:53:27
*M. 1:* Ouais, c'est tout [rires].

00:53:27
*Mme. 1:* C'est déjà ça [rire].

00:53:27
*M. 1:* C'est pas mal.

00:53:27
*Mme. 1:* C'est vraiment un questionnement--

00:53:30
*M. 1:* Ah ouais ouais ouais. Nan mais complètement, complètement. [Ben] ouais mais y a rien [inaudible 00:53:40- 00:53:41] du tout. Je dirais patate nouvelle.

00:53:50
*Mme. 1:* Ouais.

00:53:50
*M. 1:* Deux, trois trucs comme ça où les épluchures sont tolérables quoi.

00:54:03
*Mme. 1:* Ouais.

00:54:03
*M. 1:* [inaudible 00:53:56- 00:53:57]

00:54:03
*Mme. 1:* Mm-hmm. Alors là, j'aimerais qu'on échange sur le moment où vous jetez un fruit ou un légume, ou une partie seulement. Donc comment-- Quand ça arrive, pourquoi vous décidez de le jeter plutôt que de le consommer quand-- même si ça arrive pas souvent ? Quand ça arrive, pourquoi là vous le jetez et vous le consommez pas ?

00:54:27
*M. 1:* [Hum] [alors], y a la partie où c'est plus consommable.

00:54:29
*Mme. 1:* Ouais.

00:54:29
*M. 1:* Où c'est vraiment passé et ce serait dangereux de consommer.

00:54:33
*Mme. 1:* D'accord.

00:54:34
*M. 1:* Même si avec les fruits et légumes avant que ce soit dangereux, [fin bon alors] pas dangereux, parce qu'avant que ce soit dangereux y a quand même une marge, mais gustativement, c'est vraiment- c'est compliqué de manger comme ça, ça rancit ça devient-- Faut vraiment avoir faim [quoi], on n'en est pas à ce point là. Donc ya ça, on jette à ce moment là parce que gustativement manger une pomme pourrie, un fruit qui est complètement [inaudible 00:55:02- 00:55:02] c'est pas agréable. [Euh] essentiellement, ça.

00:55:11
*Mme. 1:* Oui.

00:55:11
*M. 1:* Parce que, parce que, parce que que ouais ça va pas être agréable. Parce que bon, ce serait faux de dire que c'est pas consommable. [Fin voilà], une pomme même quand elle est devenue rance [et tout] elle est consommable. Elle continue à avoir des qualités gustatives, elle continue à pas être dangereuse pour la santé [fin]. Elle va avoir un petit goût d'alcool, ça va être très mauvais mais c'est pas impropre à la consommation [quoi], donc ce serait faux de dire que au moment où c'est impropre à la consommation on les jette. Après il y a les agrumes qui deviennent moisi où là je pense qu'on est sur un truc où c'est plus propre à la consommation. Là on jette aussi. Mais [euh], mais ouais c'est ça que je vais jeter, c'est que ça va pas être- ça va pas être bon à manger.

00:55:53
*Mme. 1:* Ils sont pourris et du coup, sans parler de est-ce que c'est vraiment encore dangereux ou pas c'est que ça va pas être bon.

00:56:01
*M. 1:* C'est ça, c'est que ça va pas être bon [quoi]. Va plus y avoir aucun plaisir à manger et [ben) c'est pas drôle de le manger sans plaisir [rire]. C'est important quand même le plaisir dans les repas.

00:56:16
*Mme. 1:* Et [hum] qu'est-ce qui fait que vous vous rendez compte justement qu'il n'est plus consommable ? [Fin] que vous vous n'avez plus envie de le consommer. Qu'est-ce que-- Comment vous le déterminez, que ce fruit ou que ce légume ne--

00:56:33
*M. 1:* [Ben] y a un aspect-- Y a un aspect et/ou une odeur. Donc voilà, selon la tronche qu'il a et/ou l'odeur qu'il a c'est que.-- qu'il a plus le même goût. Bon après des fois aussi c'est par accident, on croque dedans et puis on regarde "ah euh bon, d'accord [en fait]". Mais voilà c'est-- C'est visuel et olfactif principalement.

00:57:02
*Mme. 1:* D'accord. Et qu'est-ce que vous ressentez face à de la pourriture ? Quand vous la voyez où vous sentez est-ce que vous avez--

00:57:17
*M. 1:* Nan, je--

00:57:17
*Mme. 1:* Vous ressentez rien de particulier ?

00:57:17
*M. 1:* [Ben] je, ça me dégoûte pas si c'est ça la question. J'ai pas de dégoût, de rejet, de d'appréhension, de toucher ou quoi que ce soit. Je ressens ce qu'on ressent face un fruit pourri, de la déception [rire], [fin voilà], "oh merde !", je me suis-- J'ai merdé quoi. Mais non, y a pas de dégoût. J'ai pas de problème avec la pourriture en particulier. [Fin] j'aime pas ça, l'odeur me déplaît, mettre mes doigts dessus c'est pas mon grand kiffe de la journée mais ça va pas me déranger particulièrement.

00:57:57
*Mme. 1:* Ok. Donc face à un fruit et légume pourri, même si les-- visuellement ou même olfactivement c'est pas-- ou textuellement-- c'est pas des sensations très agréables, y aura pas de dégoût, de sensation extrême, nan ?

00:58:13
*M. 1:* Nan. Y a pas de rejet ou--

00:58:13
*Mme. 1:* Ce sera plutôt la déception d'avoir perdu--

00:58:18
*M. 1:* Ouais d'avoir merdé quelque part dans mon organisation et d'avoir perdu et d'être obligé de jeter un fruit ou un légume parce que- parce que [bah] j'ai pas été attentif, parce que j'ai eu la flemme, parce que puisque c'est souvent quand même une raison qui est pas une très, très bonne raison. C'est pas parce que y a un cas de force majeure qui m'a empêché de consommer mon fruit et légume. C'est parce que- c'est parce que j'ai merdé en général.

00:58:51
*Mme. 1:* Ouais. Ok. [Hum] et est-ce que vous diriez qu'au sein de votre foyer, il y a justement des différences face à ce pourri, ou pas ? Est-ce que vous avez l'impression que-- Donc y a votre conjointe, votre fille est-ce qu'elles ont le même rapport au pourri ou pas ?

00:59:09
*M. 1:* [Alors] pour [Nom de l'enfant] c'est assez flou. Et [Nom de sa fille] je sais qu'elle aime ça encore moins que moi mais enfin dans le côté déceptif--

00:59:16
*Mme. 1:* D'accord, ouais.

00:59:16
*M. 1:* --dans le côté où on a merdé, c'est vraiment-- C'est encore plus exacerbé chez elle.

00:59:22
*Mme. 1:* Ok.

00:59:24
*M. 1:* Sinon, sa, son, sa réticence par rapport à la pourriture, à la moisissure c'est-- Je pense que c'est à peu près la même chose. Ouais, c'est ça, on n'est pas-- On n'est pas très rebutés par le sale, l'odorant et tout [fin]. Moi je suis dans un milieu où on a les mains dans de la crasse, dans des trucs qui puent, qui sont sales à longueur de temps et [Nom de sa fille] [ben] en dehors de son activité universitaire elle a beaucoup travaillé dans des écuries, avec des chevaux [et tout], donc c'est pareil, on est dans un environnement qui est pas particulièrement-- Ça sent pas la rose. On met les mains dans le crotin, dans de la paille dégueulasse. [Fin], nan, je pense elle aussi elle a pas de- elle a pas de répulsion vis-à-vis de ça non plus.

01:00:17
*Mme. 1:* D'accord.

01:00:26
*M. 1:* Après on sait jamais, y a des fois y a des odeurs c'est un peu- c'est épidermique.

01:00:32
*Mme. 1:* Oui.

01:00:32
*M. 1:* Y a certaines odeurs qui nous révulsent et c'est comme ça.

01:00:37
*M. 1:* Et concernant les préférences des fruits et légumes sur la maturité du fruit et légume, est-ce que vous voyez dans votre foyer des différences ou pas ? Sur quelqu'un qui va accepter un fruit qui- qui est complète-- Qui commence à être vraiment mûr voire trop mûr, ou-- [Fin] est-ce que vous voyez des--

01:01:00
*M. 1:* [Euh] ouais. Moi j'aime bien les fruits quand ils sont encore un peu verts, un peu [hein]. C'est limite mais j'aime bien quand ils sont un peu acidulés, et que voilà j'ai tendance , les pêches, les brunions et tout, les prendre légèrement, légèrement avant que ça soit, que ça soit mûr. Donc voilà là-dessus va y avoir une petite divergence mais [pff] je saurais pas dire, vraiment. Je saurais vraiment pas donner de réponse claire. [Nom de l'enfant] c'est encore en train de se faire donc je suis pas sûr que ça soit vraiment défini et [Nom de sa fille] je sais pas vraiment--

01:01:37
*Mme. 1:* Ses préférences...

01:01:40
*M. 1:* [Ben] ouais, ouais, elle aime bien le fruit mûr, mûr [quoi]. Comme un peu tout le monde, donc c'est pas vraiment incroyable. Au niveau de jusqu'à quel point de maturité [ben] elle va être capable de manger, apprécier, de le manger tout ça, je saurais pas définir.

01:01:59
*Mme. 1:* Ouais, et vous. Même si vous préférez encore, normalement, encore un peu vert.

01:02:05
*M. 1:* Ouais, ben moi ça va dépendre des fruits. Y en a certains, je vais avoir du mal-- Ouais, pas tant. Nan, j'aime bien les fruits, j'aime bien les fruits [rires] donc tant que ça a pas tourné [quoi], qu'il y a pas de rance, d'alcool, de truc comme ça, tant que ça a pas tourné--

01:02:21
*Mme. 1:* Vous les mangez.

01:02:21
*M. 1:* [Ben] c'est ça, ça me plaît.

01:02:21
*Mme. 1:* Même si c'est pas le moment où vous les trouvez le meilleur.

01:03:03
*M. 1:* Ouais c'est ça ouais. J'aime bien. J'aime bien les fruits.

01:03:07
*Mme. 1:* Oui [rire]. Ok. Chouette.

01:03:19
*M. 1:* Nan et puis [voilà] quand ils deviennent un peu mou, que c'est moins agréable [ben] salade de fruits--

01:03:23
*Mme. 1:* Oui c'est ça, vous transformez.

01:03:23
*M. 1:* --compotes, tartes, [fin] y a tellement de moyens de manger.

01:03:23
*Mme. 1:* Ouais, c'est ce que vous disiez aussi avec la banane quand elle devient trop mûre.

01:03:33
*M. 1:* [Ben] ouais c'est ça, trop vieille, muffins banane-chocolat c'est délicieux [voilà]. Et les fraises la dernière fois, le week-end dernier, le week-end d'avant ma mère est venue voir ma fille, elle a ramené j'sais pas combien de fraises, y en avait trop. Ça plus de-- Merde, je perds mes mots. C'est long, c'est acide, c'est filandreux--

01:03:43
*Mme. 1:* Rhubarbe !

01:03:43
*M. 1:* Rhubarbe ! Merci.

01:03:44
*Mme. 1:* [rire]

01:03:44
*M. 1:* Elle avait ramené de la rhubarbe et puis j'étais en train de perdre les fraises donc [du coup] j'ai fait une tarte rhubarbe-fraise. Ça va très bien ensemble--

01:03:47
*Mme. 1:* Ouais.

01:03:47
*M. 1:* --c'est très bon, et [voilà] ça m'a permis [ben] de sauver les fraises qui étaient vraiment en train de se perdre.

01:03:47
*Mme. 1:* Ouais.

01:03:47
*M. 1:* Donc y a toujours un moyen si on est attentifs, si on a l'oeil, faut avoir l'oeil dessus c'est comme, c'est tout, c'est-- On perd. C'est pour ça que au final, je trouve ça mieux ça vient d'avoir mes fruits et légumes dans le-- En dehors du frigo, parce que je les ai à l'oeil. Mon frigo il est petit, le bac à légumes il est bas, mon frigo il est vite plein, c'est mon ancien frigo d'étudiant, donc-- Mais dès qu'il y a deux ou trois trucs dedans on voit plus. Donc il faut vraiment se mettre à genoux voire assis par terre pour avoir une visibilité correcte et du coup, [ben] vite on perd de vue nos fruits et légumes et c'est à ce moment là qu'on les perd. Là quand on les a l'oeil [ben] on le voit, on voit que le légume il est en train de-- Le fruit il est en train de [ben] il est en train de finir sa vie [quoi], donc quand on le voit on se dit "ah oui, il faut que je le mange, qu'est-ce que je vais faire avec, je vais faire ça", et voilà, c'est réglé, le fruit ou légume on le perd pas. Donc ouais avoir l'œil dessus je pense que ça joue aussi pas mal sur la conservation [quoi]. D'avoir un visuel régulier et de [ben] de voir l'évolution du truc [quoi]. Déjà, ça permet d'avoir une expérience et de savoir que [ben] à ce stade là d'évolution il nous reste deux, trois jours ou un jour [fin]-- Ça permet [voilà] d'avoir une expérience de l'évolution de maturité de ses fruits et légumes. Et puis, ça permet aussi [ben] de driver ce qu'on va cuisiner quoi, c'est [fin voilà].

01:05:19
*Mme. 1:* Mm-hmm, complètement.

01:05:19
*M. 1:* Je pense que, je pense que l'oeil dessus ça joue-- Ça joue pas mal.

01:05:24
*Mme. 1:* Ouais. Ça vient contrer l'oubli.

01:05:26
*M. 1:* Ouais c'est ça ouais, puisque que c'est souvent, ça au final [hein], c'est soit un défaut d'organisation, soit un oubli. Et je pense que principalement, c'est de l'oubli [quoi].

01:05:33
*Mme. 1:* Oui.

01:05:36
*M. 1:* Puisqu'après, un défaut d'organisation, faut vraiment, pour qu'il soit, [fin] c'est anecdotique le défaut d'organisation. Si on perd ses fruits et légumes à chaque fois qu'on en achète, si sur chaque panier de fruits et légumes, on perd, y a un moment on le sait, on change d'organisation d'achat, on change d'organisation de cuisine, on change-- Donc le fait de continuer à perdre sur ce genre de truc, c'est soit on fait exprès, soit on a une flemme monstrueuse et on peut pas du tout changer notre organisation, c'est quand même-- Les gens qui font ça c'est une extrême minorité je pense. Faut être vraiment pas futé [quoi] pour continuer dans ce genre d'erreurs. Donc c'est de l'oubli [quoi], quand on perd un fruit ou un légume comme ça c'est parce qu'on a zappé, parce qu'on n'a pas eu l'oeil dessus, parce qu'on n'a pas pensé, parce que [voilà] parce qu'on a acheté trop et on perd. Ça se fait une ou deux fois [quoi], et puis après on arrête d'en perdre. On arrête d'en racheter trop en tout cas.

01:06:24
*Mme. 1:* Oui c'est ça, après on--

01:06:24
*M. 1:* -- on perd autrement.

01:06:25
*Mme. 1:* --ajuste [rires].

01:06:24
*M. 1:* On trouve toute technique pour perdre nos fruits et légumes.

01:06:25
*Mme. 1:* Ok. [Alors] là j'aimerais qu'on discute du gaspillage alimentaire.

01:06:47
*M. 1:* Ouais.

01:06:47
*Mme. 1:* Quand je vous dit "gaspillage alimentaire", quels sont les mots qui vous viennent spontanément à l'esprit ?

01:06:47
*M. 1:* Yaourt.

01:07:03
*Mme. 1:* Yaourt ? [rires]

01:07:08
*M. 1:* Ah ouais parce que le truc qui part le plus à la poubelle je pense dans cet appartement je pense, c'est les yaourts.

01:07:09
*Mme. 1:* Ah ouais ?

01:07:09
*M. 1:* Ouais, les petits suisses et les yaourts.

01:07:09
*Mme. 1:* Ok. Quoi d'autre ?

01:07:11
*M. 1:* [Euh, ff], ça doit être "gâchis", et puis alors les mots de [Nom de l'enfant] c'est "gaspiller la planète".

01:07:11
*Mme. 1:* Gaspiller la planète.

01:07:11
*M. 1:* Ouais. J'aime beaucoup cette expression. "[Nom de l'enfant] faut fermer le robinet quand on se brosse les dents" - "Ah oui, sinon ça gaspille la planète !".

01:07:14
*Mme. 1:* [rire] C'est direct.

01:07:34
*M. 1:* Ouais [rire] ça fait gaspillet la planète--

01:07:35
*Mme. 1:* C'est très visuel.

01:07:35
*M. 1:* Je la trouve belle l'expression, en plus ça dit vachement bien ce que ça veut dire. [Euh] ouais nan c'est ça, gaspillage alimentaire.

01:08:07
*Mme. 1:* Et si on tentait de définir ce qu'est le gaspillage alimentaire, donc vous avec vos propres mots si c'était à vous d'écrire dans un dictionnaire : qu'est-ce que le gaspillage alimentaire ?

01:08:17
*M. 1:* Je pense qu'il y a deux trucs, deux grands-- deux grandes parties dans le gaspillage alimentaire. Y a le gaspillage alimentaire domestique.

01:08:25
*Mme. 1:* Ouais.

01:08:25
*M. 1:* Donc celui sur lequel vous travaillez, principalement, j'ai l'impression, le gaspillage alimentaire.

01:08:26
*Mme. 1:* C'est ça.

01:08:29
*M. 1:* Le gaspillage dans les foyers. Et après il y a le gaspillage alimentaire de toute l'industrie agroalimentaire.

01:08:35
*Mme. 1:* Ouais.

01:08:35
*M. 1:* [Voilà], toutes les pertes liées à ça. Toute la partie agroalimentaire je sais pas trop. Je sais que ça existe, je sais que c'est des quantités qui sont non négligeables, mais j'ai aucune idée des chiffres. J'ai aucune idée du pourcentage, [fin voilà]. Je sais que ça existe, je sais que [fin voilà] y a des-- C'est une belle partie du gaspillage alimentaire, que c'est une peine, quand même. Surtout que [ben] c'est, vu que c'est industriel, c'est quelque chose qui est calculé, margé, prévu. [Euh, fin voilà], c'est extrêmement cynique comme gaspillage alimentaire. Et puis après y a le gaspillage alimentaire domestique, gaspillage je pense de flemme, de flemme d'organisation, de flemme de, de flemme de plein de choses mais qui-- Qui est donc ouais le gaspillage des ménages. Moins cynique, plus je m'en foutiste.

01:09:38
*Mme. 1:* Ouais.

01:09:38
*M. 1:* ... Mais après ouais, le définir, le définir. [Ben] le côté domestique, définir dans un dictionnaire, [ben] c'est tout ce qu'on achète pour mettre à la poubelle [rire] Tous les produits-- Tous les produits consommables qu'on achète pour les mettre à la poubelle. Parce que c'est ça le gaspillage. Je le définirais comme ça en tout ça le gaspillage alimentaire.

01:10:17
*Mme. 1:* Ok. [Hum] donc là bon c'est une question à laquelle on a répondu, vous réalisez que vous jetez un fruits et légumes à la poubelle, ce que vous ressentez [rires] [diaphonie 01:10:40- 01:10:41] déception, j'ai merdé [rires]

01:10:39
*M. 1:* Ouais c'est ça ouais.

01:10:39
*Mme. 1:* Y a quand même une forme de remise en question--

01:11:00
*M. 1:* [Ben] ouais, y a pas rien, il se passe pas rien quand je jette un fruit et légume je dis pas "oh, tant pis". Nan ça me fait chier, parce que-- Parce que [ben] ça gaspille la planète [rires].

01:11:00
*Mme. 1:* Tout à fait. Il faut arrêter de gaspiller la planète.

01:11:02
*M. 1:* C'est con quand même. [Ben] c'est con surtout [fin] aussi connement ouais ça me dérange parce que parce que c'est ce que je dis, c'est de la flemme ou un manque de... volonté d'organisation, un truc en tout cas, qui est-- qui a des solutions tellement simples. Donc voilà ça me fait chier parce que se rendre compte que c'est simple à ce point et que je l'ai pas fait, je me sens quand même vachement coupable [quoi]

01:11:35
*Mme. 1:* Je vois. Y a une culpabilité.

01:11:43
*M. 1:* Oui.

01:11:44
*Mme. 1:* Ok alors pour finir, j'aimerais juste qu'on discute de la poubelle connectée dont on avait déjà parlé. Vous l'avez depuis quelques jours maintenant et du coup, comment ça se passe ? Vous avez l'impression de l'utiliser fréquemment ou que très peu ?

01:11:56
*M. 1:* On l'a utilisée deux fois je crois.

01:12:16
*Mme. 1:* Oui voilà [rire]. Ok. Donc oui vous m'avez dit que vous avez jeté, cinq, six radis et les trois artichauts.

01:12:16
*M. 1:* Ouais c'est ça, les trois artichauts. [Alors] je crois que j'avais noté un truc sur le livret avec les notes.

01:12:17
*Mme. 1:* Ah oui ?

01:12:18
*M. 1:* Ouais mais je me rappelle plus ce que j'avais noté et je me rappelle plus de ce que j'avais [inaudible 01:12:22- 01:12:23] alors on est biens.

01:12:18
*Mme. 1:* [rire]

01:12:18
*M. 1:* Là.

01:12:18
*Mme. 1:* Ah.

01:12:19
*M. 1:* Ok. Ouais. On fait une distinction entre fruits et légumes bio et conventionnels dans votre étude dans les trucs qu'on jette, dans--

01:12:37
*Mme. 1:* Et [ben en fait] on-- là, pour l'instant, nous, non, dans ce que vous jetez--

01:12:53
*M. 1:* Je pense que ça va être des prélèvements-- sur les prélèvements--

01:12:55
*Mme. 1:* Exact-- C'est ça qu'on va vous donner et que là par contre y aura une--

01:13:00
*M. 1:* --une distinction à mettre ouais.

01:13:00
*Mme. 1:* C'est ça, on vous demandera, parce que nous on sait pas justement si ça peut aussi avoir une influence.

01:13:02
*M. 1:* [Ben] c'est ça. Ok. Ouais je pense que c'était là-dessus parce que j'ai dû écrire ça dans la foulée de lire le manuel et ça doit être ça ouais. Sur l'étiquettage des--

01:13:14
*Mme. 1:* C'est, ouais c'est prévu qu'on vous demande et la provenance ou si par exemple pour l'épicerie est-ce que vous récupérez chez quelqu'un [ben] c'est différent que quand on achète [voilà], le circuit va pas être le même etc. donc oui là ce sera intéressant de savoir à chaque fois, tout ça.

01:13:14
*Mme. 1:* Ok. Du coup, sur les artichauts, le radis, vous avez dit la raison pour laquelle vous avez perdu c'est que vous étiez en déplacement, [Nom de sa fille] n'aime pas trop les artichauts--

01:13:43
*M. 1:* Ouais c'est ça, c'était un mauvais achat, on a acheté ça-- Ça faisait partie des achats compulsifs sur le marché et au final c'était un craquage on n'aurait pas dû les acheter parce que on les a pas mangés. Et les radis [bah] parce que ce week end ma mère est venue et... quand elle vient elle demande "Tu veux des légumes ? Tu veux des fruits et des légumes quand je viens ?" parce que [ben] voilà elle habite en campagne, mon petit frère est maraîcher, [fin voilà bref] y a plein de, elle propose d'en ramener et à chaque fois, je lui dis oui, sauf que--

01:14:11
*Mme. 1:* --c'est trop [rire].

01:14:13
*M. 1:* [Ben] ouais, elle délire complètement, elle a ramené-- Elle a ramené un kilo de fraises. Plus un kilo de courgettes, un kilo d'asperges. [Euh fin] un bon 500 grammes de radis.

01:14:31
*Mme. 1:* Oui, ça commence à faire.

01:14:31
*M. 1:* Des fruits à côté, [fin].

01:14:35
*Mme. 1:* Ouais, ça commence à faire.

01:14:38
*M. 1:* Ça fait beaucoup quand même [rire]. C'est-à-dire que moi je me suis bouffé un kilo d'asperges en une journée [fin] il a fallu consommer, consommer, consommer et on a perdu, ouais on a perdu quelques radis à force, voilà, ils sentaient le chou. Quand ça sent le chou les radis je les jette. C'est pas une odeur naturelle pour un radis [rires].

01:14:55
*Mme. 1:* Et est-ce que vous avez, en dehors de ces radis y a l'artichaut, du coup la circonstance que c'est nan y a l'achat compulsif et--

01:15:04
*M. 1:* --et on m'a ramené beaucoup trop de trucs.

01:15:04
*Mme. 1:* Et voilà. Ouais. Ok. Oui. Et sinon est-ce que vous avez l'impression qu'il y a des fruits ou légumes que vous allez jeter plus que d'autres ou pas ? Vous allez perdre plus facilement--

01:15:15
*M. 1:* Y avait les citrons, parce qu'on utilisait beaucoup de jus de citron à l'époque. Et du coup, on a arrêté d'en acheter. On achète des bouteilles de jus de citron vert parce que parce que [ben] les citrons, c'est assez incroyables mais on comprend pas comment ça mûrit, pourrit, moisit. C'est incroyable tu l'achètes, un jour après tu vas en avoir trois qui vont être pourris. On rachète le même truc dans le même magasin, la même marque [et tout] et puis ils vont tenir une semaine et quelques.

01:15:46
*Mme. 1:* Ouais. Vous avez pas--

01:15:48
*M. 1:* On n'a pas compris la logique du mûrissement, pourrissement, conservation du citron. C'est--

01:15:56
*Mme. 1:* Du coup maintenant vous préférez-- C'était exclusivement pour du jus que vous les consommiez du coup ?

01:16:00
*M. 1:* Pour les tartes au citron, je fais aussi beaucoup de tarte au citron--

01:16:02
*Mme. 1:* Ok.

01:16:04
*M. 1:* --qu'on en achetait. Mais du coup, maintenant, quand je fais une tarte au citron [ben] j'achète, la journée j'achète des citrons.

01:16:09
*Mme. 1:* Et vous la faites

01:16:24
*M. 1:* Et je la fais. En direct. Pour les jus de citrons. Parce qu'après les jus de citrons c'était [ben] dans les repas [quoi] mettre un peu de jus de citron dans les crudités, peu importe, et [du coup], [Nom de sa fille] elle consomme beaucoup le citron comme ça, et on en perdait, on en perdait toujours quand on en achetait quatre on en perdait toujours au moins un.

01:16:39
*Mme. 1:* Ouais. Ok.

01:16:39
*M. 1:* On a fini par arrêter. Les bouteilles de jus de citron c'est pas mal au finlk. [Voilà].

01:16:39
*Mme. 1:* Ça marche. [Euh] sur l'utilisation de la poubelle, est-ce que ça vous arrive de vous poser des questions du style, est-ce que ça, ça doit aller dedans ou pas, ou ça va ça été plutôt intuitif ?

01:16:55
*M. 1:* [Euh] ouais, ça été. [Ben] vu qu'on a quasiment rien jeté dedans ça été assez simple. Sur l'artichaut, on s'est posé une question : c'est quand même mettre beaucoup de poids sur un truc où on n'aurait pas mangé grand chose de toute façon si on l'avait consommé. Il y en aurait énormément qui serait parti dans la poubelle, qui aurait pas été dans la poubelle connectée parce que ça aurait plus été du gaspillage du coup.

01:17:13
*Mme. 1:* Effectivement.

01:17:13
*M. 1:* Mais du coup, ça faisait vraiment. On s'est dit on va mettre vraiment beaucoup de poids pour un truc que [bah] si on l'avait mangé y aurait pas eu beaucoup moins à jeter du poids.

01:17:21
*Mme. 1:* Effectivement. Oui parce que l'artichaut ça peut-être, c'est assez lourd avec la queue [et tout], alors qu'effectivement--

01:17:29
*M. 1:* Et puis en plus c'était des petits artichauts, ils étaient vraiment pas grands, ils étaient rikikis, il devait pas y avoir grand chose à bouffer dessus.

01:17:45
*Mme. 1:* Ouais et puis y a toutes les, même les feuilles--

01:17:45
*M. 1:* [Ben] ouais c'est ça. Ouais nan c'est. Au niveau du poids l'artichaut c'est de l'arnaque.

01:17:45
*Mme. 1:* [rire] C'est vrai. [Hum] est-ce que ça vous est arrivé de l'oublier ou pas, la poubelle ? C'est à dire à un moment donné d'avoir jeté des choses et en fait ne pas les avoir mises. Vous pensez pas ?

01:17:52
*M. 1:* Nan, nan, nan, nan, nan. [Ben] déjà on jette pas souvent et [du coup], vu qu'on a la poubelle, elle traîne tellement qu'on est content de l'avoir [rires] Nan mais c'est-à-dire que [ben] elle nous vient en tête tout de suite du coup puisqu'on est là "oh merde on avait perdu quelque chose, ah bah ouais ah bah [du coup] ça va dans la poubelle qui pèse" [rires] Parce qu'on met rien dedans donc--

01:17:58
*Mme. 1:* [Voilà], au final, est-ce que vous avez l'impression que ça a changé vos, [fin], d'avoir la poubelle, non pas que ça ait changé mais que y ait une réflexion différente, supplémentaire, ou pas ? Est-ce que vous vous rendez compte genre que là vous n'avez presque rien jeté, plus qu'avant, ou ça change pas grand chose ?

01:18:38
*M. 1:* Je sais pas, j'aurais tendance à dire que ça change rien.

01:18:40
*Mme. 1:* Ouais.

01:18:42
*Mme. 1:* Mais... Sans certitude. Si, ça change forcément quelque chose parce qu'elle est là, on y pense et tout ça change forcément quelque chose. Mais je vois pas l'impact. Y a bien quelque chose. Forcément. On peut pas croire qu'elle soit là que je suis au courant qu'elle est là et puis que non ça change rien. Ça doit changer quelque chose. Mais j'ai pas de notion.

01:19:12
*Mme. 1:* Consciemment, non, c'est ça. Et sur le sac, [du coup], vous avez pas changé de sac j'imagine ?

01:19:17
*M. 1:* Non.

01:19:18
*Mme. 1:* Très bien.

01:19:18
*M. 1:* Nan du coup on est tranquilles pour le moment.

01:19:18
*Mme. 1:* Ok super. Bon [bah], j'ai répon-- [Fin] j'ai posé toutes mes questions. Merci beaucoup.

01:19:30
*M. 1:* [Ben] pas problème.

01:19:31
*Mme. 1:* Est-ce qu'il y a des choses sur lesquelles vous voulez revenir, sur ce que j'aurais peut-être pas pensé, ou--

01:19:40
*M. 1:* Nan [bah] voilà j'avais cette question sur l'étiquetage mais on est bons.

01:19:50
*Mme. 1:* Ouais.

01:19:50
*M. 1:* Nan c'est assez clair. J'avais vu une coquille aussi.

01:19:50
*Mme. 1:* Oui y en a deux [rire].

01:19:50
*M. 1:* Je l'ai pas noté.

01:19:50
*Mme. 1:* [Euh] chouette. Je vais pouvoir éteindre. Hop.

**Household F19, Interview 2**

*Mme:* Ça Part.

*Mme:* Alors, pour commencer. Un petit jeu, [enfin] ça va, il y en a plein d'ailleurs. Il y a quatre illustrations là.

*Mr:* [Ouais].

*Mme:* Et je vous demande d'en choisir une, la retourner et de me dire ce qui vous vient à l'esprit spontanément. Ce sont les trois premiers mots ou expressions qui vous viennent à l'esprit.

*Mr:* [Euh] supermarché.

*Mme:* [Ouais].

*Mr:* Fruits et légumes.

*Mr:* Frais.

*Mme:* [Ouais]

*Mme:* Qu'est-ce qui vous fait penser à frais ?

*Mr:* Ça m'est venu, je ne sais pas ce que-- c'est que des produits frais. [Enfin], si on exclut vraiment les trucs, en bord-- en bordure-- encadré de la photo.

*Mme:* [Ouais].

*Mr:* On est sur le rayon frais, dans un-- dans un supermarché.

*Mme:* Est-ce que vous pouvez me décrire l'image plus en profondeur ? Qu'est-ce que vous y voyez ?

*Mr:* [Euh] [ben] ça a l'air d'être une grande, grande surface.

*Mme:* [Ouais].

*Mr:* Beaucoup, beaucoup-- il a l'air d'y avoir beaucoup de produits différents. Les fruits et légumes, ça a l'air d'être assez varié ?

*Mme:* [Ouais] [silence].

*Mr:* Je trouve ça rigolo la façon de gérer les couleurs.

*Mme:* [Rire].

*Mr:* Le-- non, je ne sais pas quoi dire à part ça. Si c'est marrant, c'est toujours des trucs pour peser à aiguille.

*Mme:* [Ouais].

*Mr:* C'est étonnant ! Ça fait très supermarché européen ou occidental.

*Mme:* [Ouais]. Et d'une manière générale, qu'est-ce que vous pensez de ce type de lieu ? Est-ce que ça vous arrive de-- ?

*Mr:* [Ben] [ouais], [ben] [ouais], c'est pratique.

*Mme:* [Ouais].

*Mr:* On ne peut pas toujours faire le marché. Et puis et puis ça permet de trouver des trucs qu'on ne trouve pas forcément sur le marché.

*Mme:* [Ouais]. Vous pensez à quoi par exemple ?

*Mr:* [Ben] dernièrement on s'est acheté [euh] [ben] c'est une bergamote ?

*Mme:* [Ouais].

*Mr:* On n'avait jamais testé [du coup]. [Et voilà], on a trouvé ça en grande surface. On n'aurait pas trouvé ça à ailleurs.

*Mme:* [Ouais].

*Mr:* "Ça, c'est chouette !"

*Mme:* [Du coup], vous vous y rendez régulièrement dans ce genre de lieu ?

*Mr:* [Hum] une fois par semaine, à peu près.

*Mme:* [Ouais].

*Mr:* [Ouais] une fois toutes les semaines. Une semaine, dix jours [quoi].

*Mme:* [Ouais]. [Silence].

*Mme:* Est-ce que c'est un lieu dans lequel vous vous sentez bien ?

*Mr:* Sûrement, pas.

*Mme:* Non. [Rire].

*Mr:* Sûrement pas, je n'aime pas ça du tous.

*Mme:* Pourquoi vous n'aimez pas ça ?

*Mr:* Je n'aime pas les supermarchés, il y a beaucoup de gens. Les gens sont bêtes.

*Mme:* [Ouais].

*Mr:* Ils se bousculent, ne te laissent pas passer, ce-- [enfin] je n'aime pas. J'aime pas l'ambiance. C'est un mal nécessaire, comme on dit.

*Mme:* D'accord. C'est, c'est par nécessité ? Ce n'est pas par plaisir ?

*Mr:* Non, absolument pas non.

*Mme:* [Et donc], [euh] concernant les produits-- donc vous dites qu'il y a des produits que vous ne trouvez pas forcément sur le marché-- ?

*Mr:* [Ouais].

*Mme:* Ce qui est plutôt intéressant de trouver des produits différents ? Et vous pensez quoi d'autres des produits que vous pouvez consommer ? Des fruits et légumes que vous pouvez acheter là-bas ? De manière générale.

*Mr:* [Euh] ce que j'en pense ? [pff] Se fier à tout là-- c'est-- [ben] il y a à boire et à manger parce qu'il y a des trucs, il y a des produits [enfin] c'est, c'est-- c'est dans un joli achalandage [et tout], mais je ne sais pas typiquement-- typiquement les avocats.

*Mme:* [Ouais].

*Mr:* On a trouvé là-dedans qui sont des jolis produits, nourriture ici [et tout] [machin]. Sauf que [ben] la culture d'avocats, c'est une catastrophe [euh], une catastrophe écologique, une catastrophe humanitaire [enfin] c'est--

*Mme:* [Ouais].

*Mr:* C'est vraiment dégueulasse. Mais [enfin] [voilà], on va retrouver ça à côté du, du bio, du circuit court [euh] de l'enseigne [enfin]. Il y a de tout [quoi]. Il faut-- il faut être attentif à ce qu'on achète. En ce moment les clémentines, ma fille, elle mange beaucoup de clémentines. [Donc voilà], on va avoir la clémentine française, les clémentines d'Espagne et les clémentines de, un peu n'importe où. Les champignons, c'est pareil.

*Mme:* [Ouais].

*Mr:* Donc, il faut-- [ouais] Il faut être [enfin], il faut être attentif [quoi] dans ce genre de, dans ce genre d'endroit si on veut être-- Si on veut avoir un minimum de, de, de contrôle sur les produits qu'on achète.

*Mme:* [Ouais].

*Mr:* [Enfin], une vue, en tout cas une traçabilité.

*Mme:* [Ouais]. Si je reprends, vous ne pouvez pas être déçu si vous voulez des produits en particulier, notamment sur la provenance [etc]. Vous ne pouvez pas [euh] déambuler et puis prendre comme ça-- ?

*Mr:* C'est ça [ouais] un peu. [Enfin] [voilà] c'est, c'est, c'est plus ou moins fait pour le design d'un supermarché, c'est quand même fait pour que [euh] on se promène avec son chariot, on attrape, on met dedans et puisqu'on passe plus loin. Mais [euh], mais en vrai, c'est pas comme ça que ça fonctionne quand on veut [enfin] [voilà] [quoi]. Quand on veut-- [enfin] il faut y mettre de l'attention [quoi].

*Mme:* [Ouais].

*Mr:* On ne peut pas-- Il faut y mettre de l'attention ou alors c'est quelqu-- c'est d'un côté où on s'en fout de soit, de la cause environnementale ou quoi que ce soi. Mais si on veut de la cohérence entre, entre ces idées et ces actions, ça-- dans la consommation de tous les jours, c'est important d'être attentif [quoi].

*Mme:* [Ouais].

*Mme:* Et quand vous avez parlé de l'avocat ou [euh] et que c'est une catastrophe écologique et [euh] et même--

*Mr:* Humaine.

*Mme:* Humaine [ouais]. Est-ce que vous pouvez développer les deux-- ?

*Mr:* Mais la culture de l'avocat, ça coûte-- ça demande énormément d'eau.

*Mme:* [Ouais].

*Mr:* Ça-- je ne sais plus combien de litres de flotte par avocats [euh].

*Mme:* [Ouais].

*Mr:* Bon, après, j'ai pas les chiffres en tête. J'ai pas non plus les échelles de grandeur donc je ne sais pas si c'est [euh], si ça rivalise avec la, avec [euh] la--

*Mme:* Merci beaucoup.

*Mr:* L'élevage de la viande. Merci. Mais [enfin] [voilà], ça demande énormément d'eau et [euh], et [euh] la main-d'œuvre. La main-d'œuvre est pauvre, maltraitée, c'est-- c'est vraiment, je pense-- c'est vraiment une culture qui est-- qui est faite pour engranger de l'argent chez les grands cultivateurs. Mais [euh] [enfin][voilà]. Encore une fois, une main d'œuvre exploitée, sous-payée.

*Mme:* [Ouais].

*Mr:* Maltraiter [enfin] c'est--

*Mme:* [Ouais].

*Mr:* "C'est pas jojo quoi !"

*Mme:* [Rire].

*Mme:* Et [euh], [ah] est-ce que vous aimez l'avocat en soit ?

*Mr:* Oui.

*Mme:* Le produit ?

*Mr:* [Ouais].

*Mme:* [Et du coup], est-ce que ça vous arrive de faire une entorse et de le consommer ?

*Mr:* Sur l'avocat ? Non.

*Mme:* Sur l'avocat, c'est votre [euh]--

*Mme:* Ça fait des années que je n'en ai pas mangé. Mais c'est parce que l'avocat, je sais exactement comment ça se passe. Il y a d'autres trucs, mais après il y a des trucs. L'huile de palme par exemple. On essaye de plus consommer d'huile de palme du tout, même si il y a une entorse à l'huile de palme. C'est la pâte à tartiner de la petite.

*Mme:* Oui.

*Mr:* Elle mange de la pâte à tartiner au spéculoos. C'est qu'il y a une seule marque qui fait de la pâte à tartiner au spéculoos et [euh]--

*Mme:* Lotus.

*Mr:* Oui, [voilà], c'est ça. Il y a une seule recette [et du coup], on a de la pâte à tartiner à l'huile de palme. Mais sur certains trucs, oui, quand je sais, c'est une question de cohérence [quoi]. Je sais comment ça se passe [euh], que je ne vais pas-- c'est bon l'avocat, et ça se fait pas pousser par ici--

*Mme:* Oui.

*Mr:* Mais tant pis, je ne mangerai pas d'avocat. Il y a plein d'autres trucs qui sont bons que je peux manger.

*Mme:* [Ouais]. Est-ce qu'il y a d'autres [euh] fruits ou légumes, comme ça, qui sont importés ? [Euh] où la culture est quand même plus et moins, et moins catastrophique [euh] que vous pouvez consommer quand même ?

*Mr:* Il y en a quelques-uns, on mange de temps en temps. On se prend un ananas.

*Mme:* [Ouais].

*Mr:* De la mangue. Plus régulièrement que l'ananas, parce que l'ananas, on doit en acheter genre deux par an.

*Mme:* [Ouais] ok.

*Mr:* Mais on mange quelques mangues de temps en temps. [Voilà] mangues ou des produits dérivés de la mangue. Sur le marché, on achète de la mangue séchée des trucs comme ça. On mange un peu. Il y a quelques produits comme ça-- Moi, j'aime beaucoup les Kiwis.

*Mme:* [Ouais].

*Mr:* Mais c'est pareil, c'est par période. Mais là, au mois de septembre, octobre, j'ai mangé plein de kiwis.

*Mme:* [Ouais].

*Mr:* J'ai acheté plein de kiwis qui viennent, qui viennent du bout du monde [tout ça] [enfin], ça pousse en Nouvelle-Zélande, donc c'est pas--

*Mme:* [Ouais]. Vous n'en trouvez pas qui viennent de-- Je sais même pas, c'est con, de-- du coin ?

*Mr:* [Pfff] ?

*Mme:* [Enfin], je ne sais pas. Est-ce que-- ?

*Mr:* Je sais pas trop si ça-- ? Je ne sais pas trop si ça se fait ?

*Mme:* Et [ben] si, pourtant si, le kiwi. Mais [ouais], [ouais], [ouais].

*Mr:* Le kiwi [ouais]. Ok.

*Mme:* [Enfin] en Normandie où j'ai grandi, on avait un grand--

*Mr:* D'accord.

*Mme:* [Euh] un endroit rempli de kiwis, ça pousse [euh].

*Mr:* Ok.

*Mme:* Je ne sais pas quelle est la période ?

*Mr:* Je-- je me renseignerai, mais non, j'ai pas, j'ai pas fait attention particulièrement.

*Mme:* Non, c'est que, non, c'est que vous n'en avez pas vu. [Enfin] si, en supermarché, vous aurez vu la--

*Mr:* [Ben] [ouais], c'est ça. Non et puis j'ai vu que, j'ai vu [enfin]-- c'est Nouvelle-Zélande exclusivement-- [enfin] quasiment exclusivement.

*Mme:* Oui, c'est ça.

*Mr:* Et j'ai vu qu'il y a une espèce de guerre commerciale. Parce que la Chine essaye de s'y mettre et que la Nouvelle-Zélande n'est pas contente [enfin].

*Mme:* [Rire] [ouais].

*Mr:* Mais [du coup] je n'ai pas vu de production française. Le reste, ça doit être très anecdotique dans l'histoire.

*Mme:* [Ben] oui. Parce que l'avocat, il paraît qu'ils essaient de le faire pousser en Bretagne.

*Mr:* [Ha] oui ? Ok.

*Mme:* Et apparemment, ça, ça marcherait.

*Mr:* [Ouais].

*Mme:* Mais [euh]--

*Mr:* [Ben] j'en achèterai [enfin], si ça fonctionne bien. Carrément en Bretagne, on ne sait jamais.

*Mme:* [Ben] [ouais]. Je sais pas-- [enfin] je ne sais pas si ça sera exactement la même. Le même avocat.

*Mr:* [Ouais] mais ça peut-- ça vaut le coup de tenter ?

*Mme:* [Rire] Oui c'est ça.

*Mme:* [Euh] donc, si on reprend sur vos idées, sur les produits, [euh] au final, pas d'avis forcément-- sur le produit en lui-même. [En tout cas], il faut juste faire attention. Il y a de tout [quoi].

*Mr:* [Ouais], c'est ça [ouais]. On trouve des trucs [enfin]. J'ai l'impression qu'on trouve des trucs bien dans un supermarché. [Enfin] moi, ça me-- de ce que j'en connais. Après, c'est toujours très opaque. Le problème avec les supermarchés, c'est que on a une provenance générale et puis un label quand il y a manière à labelliser. Et puis, et puis sinon c'est pas vraiment-- mais [voilà]. De ce que j'en vois, il y a quand même des trucs [enfin], [en tout cas] je continuerai d'y aller. Je continue à en acheter.

*Mme:* [Ouais].

*Mr:* Je continue de nourrir une grande partie quand même grâce aux supermarchés donc--

*Mme:* Oui.

*Mme:* Et [euh] est-ce que ça vous arrive de perdre des produits qui sont issus des fruits et légumes que vous aviez achetés en grande surface ?

*Mr:* Oui. Mais-- on n'achète plus trop de citrons [du coup], maintenant, puisque les citrons par exemple, c'est un truc où-- on a acheté, allez quatre citrons et ramener ici. Deux jours après, on avait deux qui étaient, qui commençaient à moisir [quoi].

*Mme:* [Ouais].

*Mr:* Très problématique les citrons là-dessus. Et puis [euh] [ouais], si ça nous arrive ? Ça nous arrive de perdre. Quand c'est genre, on prend trop de champignons.

*Mme:* [Ouais].

*Mr:* Les trucs comme ça, ça reste assez rare au final. Parce que [voilà], c'est ça, les produits qu'on ne connaît pas vraiment. Quand on les tests, on ne va pas en acheter 15 000 [quoi], on en achète quelques-uns, puis on va voir comment les cuisiner. Ça nous plaît. On a tendance à pas trop les perdre.

*Mme:* OK. Et quand ça vous arrive, qu'est-ce que vous éprouvez ?

*Mr:* Je vais dire c'est chiant, mais c'est pas-- [Rire]

*Mme:* Si ,si [Rire]

*Mr:* Non, c'est chiant effectivement, c'est un peu limité par exemple. [Ouais] s'est mélangé. Il y a un petit côté un peu honteux, un peu d'une forme de déception [quoi] d'avoir [euh] Je ne sais pas-- [fff] si je voulais vraiment intellectualiser le truc, je dirais une forme de contrat moral avec son produit.

*Mme:* [Ouais].

*Mr:* On achète son produit, on achète son produit pour se nourrir.

*Mme:* [Ouais].

*Mr:* Donc [euh] [enfin] [voilà], c'est ça. Et puis là, [ben] là, il y a un échec, il y a un échec. C'est un sentiment d'échec.

*Mme:* [Ouais] [Silence].

*Mme:* Ok. Alors, si on prend une autre illustration ? Même exercice. Vous me dites ce qui vous vient à l'esprit. [inaudible].

*Mr:* Donc, on est sur un marché.

*Mme:* [Ouais].

*Mr:* Un marché qu'à l'air du coin. [En tout cas], c'est que des produits, des produits [euh] de, de, de chez nous.

*Mme:* [Ouais].

*Mr:* Beaucoup de personnes âgées.

*Mme:* [Rire].

*Mr:* Quasiment que des vieux.

*Mme:* [Rire] c'est vrai [ouais].

*Mr:* Bon et puis ça doit être probablement l'hiver [hein]. Parce qu'on voit beaucoup des choux, des céleris. On doit être dans la saison. La saison actuelle.

*Mme:* [Hum] Et qu'est-ce que ça-- Qu'est-ce que vous pensez de ce lieu ? Qu'est-ce que ça vous évoque le marché ?

*Mr:* [Hum] J'aime bien le marché. Je me dis, contrairement au supermarché, ou on va quand même retrouver, à peu de chose près, les mêmes, les mêmes mécaniques avec les gens qui sont-- qui restent définitivement malpolis [quoi].

*Mme:* [Ouais].

*Mr:* Mais j'aime bien ce côté, déjà, avoir affaire à un commerçant. Quasiment à chaque produit que l'on va acheter. Avoir un vis-à-vis humain, avec lequel on peut discuter du produit. Même si certains sont, sont juste des vendeurs, ils vont vous dire ce qu'on a envie d'entendre.

*Mme:* [Ouais].

*Mr:* Alors, est-ce que [ben] je trouve ce contact et cette façon de faire agréable ?

*Mme:* [Ouais].

*Mr:* Et puis [euh] et puis ils ont au moins un truc dans les supermarchés-- où les supermarchés, les produits, faut qu'ils soient beaux.

*Mme:* [Ouais].

*Mr:* Il faut absolument que [euh] ils aient la tronche qui va bien. Sur le marché, on va moins retrouver ça, même s'il y a toujours [enfin] [voilà] c'est de la vente. Donc, il faut que le produit fasse envie. Mais vu qu'on est face à quelqu'un, il va peut-être plus facilement pouvoir un peu forcé sur le produit moche qui reste, mais tout à fait consommable et gustativement identique [enfin] à l'autre donc-- donc [voilà]. Et puis je trouve ça chouette puisque j'en ai eu des bons souvenirs quand j'étais enfant et [euh]-- les commerçants, [enfin], les commerçants de marché aiment les enfants, ça me donne des bons souvenirs. Et quand j'y vais avec ma fille, c'est pareil, ça se passe bien en soi avec une fleur, un truc à manger. [Enfin] [voilà] Je trouve, je trouve ça chouette [quoi]. Je trouve que c'est une ambiance agréable et une façon agréable d'aller faire ses courses.

*Mme:* [Ouais].

*Mr:* Après les produits, ça va dépendre du marché [quoi].

*Mme:* [Ouais].

*Mr:* Ici, on va au marché qui est en bas, un marché qui est un marché bio et local.

*Mme:* [Ouais].

*Mr:* En théorie [en tout cas]. Après les produits, je ne vois pas comment ils peuvent être locaux, mais--

*Mme:* [Rire] comme le kiwi [ouais].

*Mr:* [Ben] il y a des ananas par exemple.

*Mme:* [Ah] [ben] Oui.

*Mr:* L'ananas local, j'y crois moyennement.

*Mme:* [Ouais] [ouais].

*Mr:* Mais [euh], mais [voilà], c'est un marché qui n'est pas trop mal quand même [enfin] puisque pas de récolte qui est relativement cher.

*Mme:* [Ouais].

*Mr:* Mais qui est pas mal. [Enfin] sur les tarifs sur le marché. Il n'y a pas, il n'y a pas de règles [quoi]. C'est juste [enfin], c'est-- les produits peuvent coûter du simple au double entre un étal et un autre, un marché à un autre.

*Mme:* [Et du coup], quand vous êtes [euh] sur le marché, vous faites [du coup] plus attention. Justement, cette différence de prix, ou-- ? Parce qu'aux supermarchés, vous faites, notamment, attention à la provenance, mais aussi et peut-être le prix aussi.

*Mr:* Bien sûr, ça rentre en compte. On note, mais [euh] je fais plus attention aux prix ? Oui et non. Parce qu'[en fait]. Il y a bien plus une question d'habitude je trouve, [enfin] dans ma façon de consommer, en tout cas au marché.

*Mme:* [Ouais].

*Mr:* Je vais aller au même étal.

*Mme:* Ok.

*Mr:* Une fois que j'ai fait le tour du marché, quelques fois-- et puis je l'ai connu. Quand on va sur le marché, on va-- il y a plusieurs étals de fruits et légumes, mais on fait, globalement, toujours les mêmes étals de fruits et légumes. Que les prix rentrent plus en compte à ce moment-là, [quoi]. Ça, ça a [enfin], ça a eu à entrer en compte [euh] dans les premières fois au moment de choisir les habitudes.

*Mme:* Oui.

*Mr:* Mais ce n'est pas le seul critère.

*Mme:* [Ouais]. Ça va être quoi les autres critères ?

*Mr:* [Ben] [euh] [fff]. Il va y avoir d'une part la beauté des produits [hein] parce que je suis sensible comme tout le monde [enfin] que le produit est plus beau, il me fait plus envie.

*Mme:* [Ouais].

*Mr:* [Euh], le contact avec le, la commerçante.

*Mme:* [Ouais].

*Mr:* [Euh], donc le prix aussi en fait partie. Et puis, [euh] et puis [euh], [ben] selon les disponibilités. S'il y a un étal, je vais avoir besoin de, de [enfin], si j'ai besoin de quatre produits, qu'il y a un étal qui m'en propose deux, un étal qui propose les quatre et [ben] [enfin] je ne vais pas faire deux d'un côté deux de l'autre, je vais aller directement sur, sur celui qu'à un choix, qu'un choix qui me correspond plus.

*Mme:* Oui.

*Mme:* Ok. Donc quand, quand vous allez au marché, c'est pour vous plus agréable d'y faire vos courses que lorsque vous êtes dans les rayons du--

*Mr:* [Ouais].

*Mme:* Supermarché ?

*Mr:* [Ouais], je préfère [ouais].

*Mme:* Et [euh], et ça [du coup]--, c'est à quoi-- ? Est-ce que c'est l'ambiance générale ou si, c'est rigolo, vous avez dit quand même que les gens étaient quand même toujours mal poli ? [Rire]

*Mr:* [Ben] [ouais], parce qu'ils restent mal polis sur le marché. Il reste-- il y a toujours des gens qui bousculent sans dire pardon ou qui [enfin], [voilà]. Je parle des gens mal polis, parce que bousculer quelqu'un, ça arrive, ce n'est pas grave. Se retourner, dire pardon, c'est pas [euh]--

*Mme:* [Ben] oui.

*Mr:* Ça n'arrache pas la langue [quoi].

*Mme:* [Ouais].

*Mr:* Mais [euh] je ne sais pas. Le fait que ce soit en extérieur, ça doit être plus agréable déjà.

*Mme:* [Ouais].

*Mr:* Parce que, effectivement, les supermarchés, l'ambiance hangar, néons [euh]--

*Mme:* [Ouais].

*Mr:* [Pfff] ! Même s'ils font des efforts de présentation, ça reste un grand hangar sous néons [quoi].

*Mme:* [Ouais].

*Mr:* Donc [euh], donc c'est pas top. Et puis [voilà], c'est ça. L'extérieur me plaît plus. Géants, par exemple, je trouve ça très rigolo d'entendre les petits oiseaux et de voir les petits oiseaux qui sont dans le hangar, qui squattent depuis des années, dans le hangar, qui vole.

*Mme:* Il y a des oiseaux dans le-- ?

*Mr:* Il y a des oiseaux dans les rayons du Géant. Il y a des petits moineaux qui volent [enfin]. Ça rend le côté-- ça rend le lieu plus agréable. D'avoir un truc comme ça [enfin] [voilà], ce truc très artificiel, très industriel [et tout].

*Mme:* Oui.

*Mr:* Ça me fait pas envie [quoi]. J'y vais par nécessité. C'est pas un moment agréable que je passe. C'est pas non plus un enfer [hein].

*Mme:* Oui.

*Mr:* Mais c'est pas un moment agréable.

*Mme:* Oui. Donc le supermarché est plus industriel et vous retrouvez un peu plus [du coup] du naturel dans le marché-- ?

*Mr:* [Ouais], [en tout cas] pour l'ambiance. Parce que, parce que je sais bien que sur le marché, [ben] [enfin][voilà] il y a-- on va retrouver les mêmes produits, Savéol par exemple, [enfin] ils n'ont pas-- les supermarchés n'ont-- n'a pas le monopole de Savéol [quoi]. Savéol, c'est partout, même sur le marché, donc [euh] [voilà], il va y avoir une possibilité de trouver des produits qui viennent de, d'autres circuits [enfin] qui, [voilà]. Mais on reste sur les mêmes, les mêmes mécanismes de, de, d'achat vente et de consommation.

*Mme:* [Ouais].

*Mr:* On sait bien que [enfin], on sait bien comment ça se passe [quoi]. C'est pas parfait. C'est pas la révolution [quoi]. Par rapport aux supermarchés, on trouve les mêmes choses globalement. Mais oui, c'est plus agréable.

*Mme:* Ok. [Euh] [du coup], c'est quoi la raison première ? Si vous deviez résumer [enfin], la raison première qui fait que vous, que vous privilégiez le supermarché, le marché ? Enfin les deux mais [euh]-- ?

*Mr:* Je trouve, je trouve ça plus humain le marché.

*Mme:* Donc c'est vraiment plus pour l'ambiance que pour les produits en soi ?

*Mr:* [Ouais] [ouais], parce qu'il y a moyen de trouver autrement [enfin] [voilà]. Là, en ce moment, il fait moins beau, donc on va moins sur le marché par exemple. Mais on va, on va. à ICI, je sais pas si vous connaissez ?

*Mme:* [Ouais], j'en ai entendu parler.

*Mr:* [Voilà], c'est tout petit, c'est assez vite fait. Mais on est sur une ambiance supermarché, [quoi], plus que sur une ambiance marché. Même si on-- je pense à une espèce de Michelin.

*Mme:* Oui.

*Mr:* [Voilà]. Il n'y a pas de choix de produits. [Enfin] c'est très, très limité [quoi], c'est que du local. On a vraiment très, très peu de choix de produits, mais [euh], mais les produits sont cool. Et puis, [et puis voilà], c'est le bio local. On sait au niveau, au niveau du circuit, que ça se passe bien, il y a même les photos des cultivateurs.

*Mme:* [Ah] [ouais] ? Ils mettent [euh]-- ?

*Mr:* Oui, c'est ça, c'est très rigolo.

*Mme:* Ok. On parlait des photos des cultivateurs. Pour vous, il y a quand même un intérêt, même sur le marché, vous avez parlé de ça, le fait d'être avec le commerçant, même si des fois, c'est--

*Mr:* [Ben] là, la photo, c'est juste parce que je trouve ça fun. Ça n'a aucun intérêt. Sinon, pour moi, ça ne me pousse pas à acheter parce qu'il a une bonne tête ou [quoi] parce que--

*Mme:* C'est plus à la tête du produit, c'est à la tête du type en photo !

*Mr:* [Ouais], je trouve ça rigolo. La dernière fois, il y en avait en catogan [et tout]. Il était vraiment très décalé. Historiquement, c'est-- ça, ça ne collait pas [quoi]. Il était resté dans les années 90, c'est très, très fun.

*Mme:* [Ouais], [ouais], c'est plus pour le côté marrant.

*Mr:* [Voilà], c'est ça. Mais j'aime bien cette idée de mettre les photos.

*Mme:* Et [euh], mais vous privilégiez quand même les produits locaux [euh] ?

*Mr:* [Ouais].

*Mme:* Quand c'est possible ?

*Mr:* Toujours, le plus possible. Un local bio aussi, si c'est possible. Et puis [euh] [ouais], c'est ça le circuit, mais plus possible un circuit court. Les produits de saison et de, et d'ici [quoi].

*Mme:* Oui.

*Mr:* Histoire de s'assurer le plus possible, qu'ils aient, [ben], qu'ils aient déjà poussé sans trop d'électricité et puis, qu'ils n'aient pas consommé des tonnes de carburant pour, pour arriver jusqu'à, jusqu'ici [quoi].

*Mme:* C'est notamment écologique ? Oui,

*Mr:* Oui.

*Mme:* La volonté de consommer local ?

*Mr:* [Ouais], c'est quasiment et exclusivement écologique [euh]. Moi j'aime bien les produits de chez nous puisque c'est ceux que je connais aussi.

*Mme:* Ouais.

*Mr:* Mais [euh] je freine ma curiosité des autres produits pour des raisons écologiques [quoi].

*Mme:* [Ouais].

*Mr:* [Ouais], c'est parce que-- c'est pas ce que [voilà], je, je sais comment ça fonctionne. Si ça ne vient pas par avion, ça vient par bateau. Ça crame des tonnes et des tonnes de kérosène et dans les bateaux, je sais ce qu'on met pour les faire tourner et c'est pas jojo.

*Mme:* [Ouais].

*Mr:* Donc, non, c'est dégueulasse [quoi]. Et puis ça sert à rien, c'est stupide. Donc, on a largement de quoi produire et s'alimenter en local. Ce n'est pas pour faire du repli ou du nationalisme ou [quoi], c'est qu'il y a un moment, faut arrêter le commerce. Le commerce international, pourquoi pas ? Ça a des bons côtés. Faut arrêter de marcher sur la tête aussi. On n'est pas obligé de mettre-- les produits qu'on peut faire pousser chez nous, on n'est pas obligé de les acheter à l'autre bout du monde ou--

*Mme:* [Ouais].

*Mr:* On n'est pas obligé de manger des avocats sans arrêt, on n'est pas obligé de manger des tomates en février. On n'est pas obligé tout ça [quoi]. Donc, [ouais], c'est, c'est, c'est la principale raison, voire la seule [enfin].

*Mme:* [Ouais].

*Mr:* [Ouais]. C'est une question de cohérence [quoi]. Une question. Je ne sais pas, ça me paraît simple.

*Mme:* [Ben] [ouais], [ouais].

*Mr:* [Enfin] un maraîcher aussi. Donc [voilà], c'est--

*Mme:* Ok.

*Mr:* [Enfin], il y a un truc qu'avec-- tu fais pousser, tu fais pousser des produits, que tu les manges. C'est pas compliqué [quoi].

*Mme:* [Ouais]. Donc [en fait] finalement le supermarché, c'est là où vous allez trouver les produits que vous ne trouvez pas sur le super-- que vous ne trouvez pas sur le marché. Finalement, vous allez rarement acheter au supermarché des produits qu'on cultive de saison localement.

*Mr:* [Euh], [ben] on va en profiter, c'est-à-dire qu'on ne va pas-- pareil, c'est pas la partie la plus agréable de la journée, d'aller faire les courses. Donc, quand on va au supermarché acheter du PQ, des pastilles pour la vaisselle, [enfin] des trucs qui, de toute façon, on est obligé de passer par un supermarché pour aller, pour les avoir. On va prendre des produits frais aussi.

*Mme:* [Ouais].

*Mr:* Parce que, parce que c'est aussi le truc des produits frais, c'est que [euh] il faut en acheter régulièrement.

*Mme:* [Ouais].

*Mr:* Parce qu'il ne reste pas frais, vital, donc [ben] [voilà], on en profite, on achète [ben] des poireaux, des choux de Bruxelles [enfin] les trucs d'en ce moment et puis [euh] on les mange tous les deux ou trois jours. Mais [euh] on va en profiter pour acheter des, pour acheter quelques trucs.

*Mme:* OK, donc, même ce que vous pouvez trouver sur le marché-- ?

*Mr:* Ça nous arrive.

*Mme:* Quatre jours, cinq jours après, au supermarché, vous prenez quand même-- ?

*Mr:* C'est ça, ça va nous arriver de, [enfin], [voilà] de-- On les achète, on les achète aussi au supermarché.

*Mme:* [Ouais]. Et [euh] vous allez au marché de Kerinou ?

*Mr:* [Ouais].

*Mme:* Vous n'allez jamais à celui de Saint-Louis ou d'autres marchés dans le-- ?

*Mr:* Non.

*Mme:* Et carrément, il y a une volonté de-- pourquoi Kerinou ?

*Mr:* Parce que c'est à côté.

*Mme:* OK, c'est pas faux.

*Mr:* Parce que c'est un marché bio local à côté. Je sais où qu'il est et je sais quels jours, que les autres non.

*Mme:* Vous y trouver tout ce que-- ?

*Mr:* Oui, c'est ça, on avait essayé celui qui est du côté de l'église là-bas, Saint-Martin, Saint-- celui où il y a les halles là.

*Mme:* [Ah] ! C'est Saint-Martin.

*Mr:* [Ouais] et on a pas trouvé ça bien. Dans les halles, les halles, on n'a pas-- on trouvait ça cher et puis [euh]--

*Mme:* Oui, parce qu'il n'y a pas de marché alimentaire, c'étaient juste les halles [euh]. J'ai déménagé à côté, mais je n'ai pas été encore, mais je crois que c'est-- [ouais], il n'y a pas autant de monde, il y a un primeur, mais ce n'est pas un marché en soi, je crois.

*Mr:* [Ouais], c'est ça [ouais], on avait-- on était passé une fois acheté deux ou trois trucs.

*Mme:* [Ouais].

*Mr:* Non, Kerinou, il est à côté, il est bien.

*Mme:* [Ouais] donc--

*Mr:* [Voilà], il y a des trucs, [voilà], il y a les fruits secs. [Nom de l'enfant] aime bien donc, [voilà] c'est son truc, à cet endroit-là. Au niveau des fruits et légumes, c'est cool. Et puis ça s'arrête à peu près à ça.

*Mme:* [Ouais].

*Mr:* Je n'achète pas trop d'autres trucs. Si des fois, un bout de viande, un morceau de fromage [ouais].

*Mme:* [Ouais].

*Mr:* Mais [voilà], surtout les fruits et légumes.

*Mme:* [Ouais]. Ok.

*Mr:* Et des œufs.

*Mme:* Des œufs. Et vous-y aller [euh] toutes les semaines ? Ou c'est plus [euh] non ?

*Mr:* Non, on y va plus toutes les semaines non.

*Mme:* [Ouais].

*Mr:* Non, il fait moche.

*Mme:* [Ouais].

*Mr:* Non, non, il ne fait pas beau, on y va plus toutes les semaines. Mais [euh] non, ça fait un bout de temps qu'on n'est pas retourné d'ailleurs même. Je ne saurai pas dire mais, ça fait au moins trois semaines.

*Mme:* Ok.

*Mme:* [Voilà], après, le rythme a changé un petit peu puisqu'on a [Nom de l'enfant] toutes les semaines. Mon ex-compagne est en études américaines donc, on l'a toutes les semaines. En général, le samedi matin. On se repose.

*Mme:* [Ouais].

*Mr:* [Ouais], c'est pas [enfin] [voilà]-- on n'a plus trop [euh]-- sinon c'est [euh] je ne sais plus, le lundi ou mardi soir.

*Mme:* [Ouais], le mardi soir.

*Mr:* Mais c'est pas jouable, la petite, elle est crevée.

*Mme:* [Ouais].

*Mr:* On la couche tôt [et tout].

*Mme:* [Ouais]. Donc il y a quand même un côté, aller au marché, c'est, [euh], ce n'est pas forcément nécessaire [euh] pour vous, que c'est aussi, si c'est possible, en termes de temps et de--

*Mr:* [Ouais].

*Mme:* Et de plaisir de s'y rendre ?

*Mr:* [Ben] c'est ça, [Ben] là, on trouve, [ouais] c'est ça, on trouve, on trouve-- [Ben] entre le supermarché, ICI, on s'en sort. Et puis, et puis, c'est la saison des gros plats qui dure.

*Mme:* [Ouais].

*Mr:* Donc c'est pratique aussi pour ça.

*Mme:* Et quand [euh] ça vous arrive de jeter un produit que vous aviez acheté au marché, qu'est-ce que vous ressentez ? Est-ce que ça va être globalement le même sentiment qu'au supermarché ?

*Mr:* Pareil, pas de distinction une fois qu'ils sont dans le bac à légumes, c'est les mêmes, c'est les mêmes produits.

*Mme:* [Ouais]. Donc, c'est le même contrat moral avec-- ?

*Mr:* Avec nos légumes ? [Ouais]. [Ouais], [ouais]. La provenance.

*Mme:* OK. Alors si on prend une autre photo ?

*Mr:* Donc, c'est un carré de jardin.

*Mme:* [Ouais].

*Mr:* Avec [euh], on dirait une espèce de-- Ça, c'est, ça à l'air d'être du chou ?

*Mme:* [Hum].

*Mr:* Ça, j'ai absolument aucune idée de ce que c'est.

*Mme:* Moi, non plus je sais pas.

*Mr:* Mais ça, je sais pas, je sais pas trop.

*Mme:* C'est des poireaux au fond ?

*Mr:* [Ben] poireaux, oignons, un truc comme ça potentiellement, mais [ben] j'aime bien. J'aimerais bien avoir un petit jardin.

*Mme:* [Ouais].

*Mr:* Faire pousser des trucs.

*Mme:* [Ouais].

*Mr:* Mais après on des légumes, bien sûr. Parce que c'est bon qu'il faut en consommer. Mais j'aimerais bien faire pousser des aromates et des simples.

*Mr:* [Ouais].

*Mr:* Pour faire des infusions. Pour, pour aromatiser la bouffe [et tout]. [Ouais], j'aimerais bien avoir un petit jardin, un truc comme ça d'herbes. Mais [euh] vous voyez, je, [euh] [voilà], j'aimerais bien ce côté. Cette petite est bien droite. Je ne suis pas sûr d'aller jusque-là. Pareil l'osier autour, je ne suis pas sûr mais [voilà].

*Mme:* Oui, là on est sur un potager.

*Mr:* [Ouais], c'est propre.

*Mme:* [Ouais], là, on est [ouais].

*Mr:* Mais [ouais], ça me, ça me donne envie de déménager là, avoir un peu de terrain.

*Mme:* [Ouais].

*Mr:* Et puis avoir c'est-- [voilà]. Pousser un truc [quoi].

*Mme:* [Ouais]. Est-ce que vous pouvez développer ce que-- Pourquoi vous aimeriez avoir à faire pousser vos-- ?

*Mr:* [Ben] parce que, pour plein de raisons déjà, parce que je trouve ça plaisant. De façon assez, assez bateau, je trouve ça agréable et que, et que j'aimerais bien pouvoir le faire. Et puis parce qu'après, au niveau de la traçabilité du produit [enfin], il n'y a aucun problème [quoi]. Et puis il y a certains trucs qu'on va pouvoir faire pousser et qu'on ne peut pas acheter en supermarché. Les framboises, l'exemple type. Moi, je n'achète pas des framboises en supermarché, je sais comment ça pousse. Je sais que [voilà], tu mets deux pieds de framboises et puis c'est bon les framboises. Et puis, ça coûte quasiment 20 € du kilo. Donc oui, c'est fragile. Oui, ça se transporte mal. [Bah tant pis], je ne les transporterais pas, elles sont chez moi, donc sur un mur. Et puis ce sera cool. Les fraises, les fraises, c'est vachement bon. C'est un fruit qui est vachement sain en plus. Sauf ceux qu'on, les fraises, qu'on achète dans le commerce parce que en France, la fraise, c'est le-- ce doit être le produit qui a le plus de produits.

*Mme:* [Ah] [ouais], sur [euh]-- ?

*Mr:* Sur le fruit. Il y a rien qui dépasse les doses légales, mais ils ont tout mis. Donc, la fraise, c'est un nid à pesticides [euh] [enfin], c'est vraiment, c'est dégueulasse [quoi].

*Mme:* [Ouais].

*Mr:* Donc j'aime beaucoup ça. Je continue à en acheter, ça ne va pas du tout. Faudrait pas que j'en achète, mais je trouve ça très bon. On est quand même dans une région de fraises.

*Mme:* [Ouais].

*Mr:* Donc [voilà], mais j'ai hâte de pouvoir faire pousser les miennes parce que c'est pareil, les pieds de fraises, c'est rhizome, ça s'étend partout, ça pousse de peur donc--

*Mme:* [Ouais].

*Mr:* Donc [voilà], il y a des trucs comme ça. Les courgettes, les courgettes et les tomates. Mais même de saison. Quand j'achetais des tomates en supermarché, [ben] elles sont fades [quoi].

*Mme:* [Ouais].

*Mr:* Alors qu'une bonne courgette qu'à pousser, [enfin] qu'à pousser dans la terre au soleil, c'est quand même tellement bon [quoi].

*Mme:* [Bah] [ouais].

*Mr:* Pareil pour les tomates, [enfin] [voilà], il y a plein, c'est quand même, c'est quand même des bons produits qu'on fait quand on les fait chez nous, c'est que ça, ça a quand même meilleur goût.

*Mme:* [Ouais].

*Mr:* Et puis [voilà], et puis c'est un passe-temps. C'est un passe-temps qui est chouette [quoi]. C'est un truc qu'on peut faire ensemble en famille.

*Mme:* [Ouais].

*Mr:* [Enfin] [voilà] c'est pareil, j'en ai des bons souvenirs d'enfant et j'aimerais bien continuer et puis--

*Mme:* [Ouais].

*Mr:* [Ben], j'aimerais bien que [Nom de l'enfant], c'est pareil. Elle est cette [euh], cette connaissance [euh] nous-- du fait que ça existe, qu'on peut faire comme ça. Après, qu'elle ait envie de le faire ou non, c'est autre chose. Mais qu'elle ait au moins, [enfin] [voilà] que ça fasse partie des choses, qu'elle sache.

*Mr:* [Ouais].

*Mme:* Donc, vous aviez un potager enfant ?

*Mr:* Moi, j'ai grandi à la campagne.

*Mme:* [Ouais].

*Mr:* Ce n'est pas un potager. Il y faisait deux hectares et demi de terrain, il y avait le potager, mais c'était vraiment-- [enfin]. J'ai grandi, j'ai grandi au milieu des champs.

*Mme:* [Ouais].

*Mme:* OK. Donc globalement les produits, vous les trouver-- ?

*Mr:* Meilleurs.

*Mme:* Meilleur que-- qu'au supermarché ?

*Mr:* [Ouais], c'est ça. Rien à voir.

*Mme:* [Ouais].

*Mme:* Et donc, si vous êtes amené à perdre un produit [euh], [enfin] c'est votre frère qui maraîcher ?

*Mr:* [Ouais].

*Mme:* C'est-- peut-être-- il vous donne des choses des fois ou --?

*Mr:* [Ouais].

*Mme:* Si vous perdez un de ces produits, qu'est-ce que [euh] vous éprouvez ?

*Mr:* [Bah] [ouais] [fff], ça m'emmerde aussi, un peu. J'aime pas perdre, j'aime pas jeter de la bouffe, ça, ça me gêne.

*Mme:* [Ouais].

*Mr:* [Euh], [voilà], ça me gêne. [Ben] quand il me les donne, je trouve ça chiant parce que ce qui nous a filé et que-- il y aurait pu les refiler à quelqu'un d'autre ou [quoi]. Ça vient de chez moi, je pense qu'il y aurait moins de problème, parce que-- mais il faut déjà être-- avoir un peu d'expérience pour savoir la quantité dont on va avoir besoin.

*Mr:* [Ouais].

*Mr:* Donc même si on en fait trop, on a un extérieur comme ça, [ben] ça retournera là ou ça vient [quoi]. Un coup de compost et puis--

*Mme:* [Ouais].

*Mr:* Et puis [voilà]. C'est pas très grave parce que ce sont des produits effectivement où-- c'est toujours chiant de perdre. Mais ils n'ont pas été [euh], ils n'ont pas consommé d'électricité particulièrement. Ils n'ont pas consommé de carburant particulièrement. Ils n'ont pas été dopés avec, avec des engrais qui demandent une quantité de flotte et d'énergie pour les, pour les produire. C'est un cycle extraordinaire et [enfin] [voilà], des produits. Bon, il aurait fallu un peu d'eau, il a fallu de l'attention et du temps. Mais [voilà], ça reste, ça reste des trucs, pas très grave [quoi].

*Mme:* [Ouais].

*Mme:* [Ouais], selon vous, il y a-- ce qui est même un peu plus grave entre guillemets, si on perd un produit [euh], qui a coûté [euh]--

*Mr:* [Ben] [ouais], qui a, qui a, qui a un coup-- on reste sur l'écologie [hein]. Qui a un coup écologique important [quoi]. [Ben], on est en train de-- [ben] ce--. Les deux sont du gaspillage, mais il y en a un, c'est du gaspillage où en plus tu te donnes les moyens de gaspiller [quoi].

*Mme:* [Ouais].

*Mr:* Te dire que, je vais le gaspiller ce produit. Mais avant on va le blinder d'électricité, de pétrole, [euh], [enfin] on va tout mettre dedans et après [hop] on va le jeter.

*Mme:* [Ouais].

*Mr:* C'est, [voilà] [quoi]. C'est un souci [quoi]. Quand ça vient de chez, quand ça vient de chez nous, il y a moins cette problématique.

*Mme:* [Ouais].

*Mr:* Toujours. C'est chiant de jeter [quoi].

*Mme:* [Ouais].

*Mr:* C'est pas agréable de se dire, [ben], [voilà], j'ai de la bouffer. Mais il ne servira à rien [quoi].

*Mme:* Oui, quoi qu'il arrive, c'est un produit qui [euh], qui était supposé être manger et qui est perdu.

*Mr:* Oui.

*Mme:* Mais quand il y a eu un coût écologique important dessus, c'est carrément toute l'électricité, tout ça, toutes les ressources qu'on a utilisées--

*Mr:* [Ben] c'est ça, [ouais]. Et puis on sait que, on sait que, on fait du mal à notre environnement.

*Mme:* [Ouais].

*Mr:* [Enfin] [Voilà], il n'y aurait pas ce-- on ne ferait pas-- on n'aurait pas de problème avec l'environnement, on ne serait pas en train de tout défoncer. Ce serait moins grave, mais on le sait qu'on est en train de faire ça et qu'on y participe tous, chacun à notre niveau, chacun. Donc [euh], donc oui.

*Mme:* Oui.

*Mr:* J'y reste pas insensible, ça me parle. C'est un truc qui est agréable à faire [quoi].

*Mme:* Ça gaspille la planète.

*Mr:* Ça gaspille la planète. Je pensais, c'est exactement--

*Mme:* [Rire].

*Mr:* Exactement, on nous a engueulés, avant-hier, parce qu'on jetait ses masques en papier.

*Mme:* [Ah] oui !

*Mr:* Mais, on peut lui expliquer que oui mais--

*Mme:* [Ben] [ouais].

*Mr:* On peut lui expliquer que oui, on les jette quand même. Il y a des beaux pandas dessinés dessus [et tous]. Elle veut les garder à la maison, elle porte toute la journée à l'école, il va à la poubelle.

*Mme:* [Ouais].

*Mr:* "Mais ça gaspille la planète !"

*Mme:* Mais c'est vrai. Je crois qu'il y a un qui, je crois qu'il y a une [euh], j'avais cru voir vu une personne qui essayait de les recycler.

*Mr:* [Ouais] j'ai cru voir ça aussi. [Ouais], [ouais] [ouais], mais [euh]--

*Mme:* Mais c'est pas du tout développé, [enfin] on n'a pas de poubelle à masque. [Rire]

*Mr:* [Ben] c'est des déchets biohazard [quoi]. C'est des déchets médicaux sensibles, donc [euh] c'est compliqué. Rien que, [ben] rien que de déposer une poubelle, c'est compliqué. Puis après, l'acheminement est compliqué. Forcément, ça reste--

*Mme:* [Ouais]. Il faut que-- oui.

*Mr:* C'est pas facile [quoi]. Déjà que c'est une galère à avoir des trucs pour déposer des ampou, des ampoules ou des piles dans les supermarchés alors--

*Mme:* [Ouais]. On va les mettre pour chaque [euh]--

*Mr:* [Ouais], c'est ça, ça va pas être facile.

*Mme:* [Ouais], [ouais]. Ok.

*Mme:* Alors, la dernière [euh]--

*Mr:* Photo.

*Mme:* Photo [ouais].

*Mr:* [Wow] !

*Mme:* À quoi vous pensez ?

*Mr:* [Euh] [pfff]. C'est très tape-à-l'œil. Ça me fait penser à, [euh], au wall food américain. Alors, après que je ne connais pas [hein]. Que je ne connais que par le prisme de la série South Park, donc.

*Mme:* [Rire].

*Mr:* Très déformée.

*Mme:* Ok.

*Mr:* Du, [enfin] [voilà], mais ça fait vraiment, [enfin]-- un tout, la façon dont il est habillé [euh], la présentation [et tout]. Je trouve que ça fait, très tape-à-l'œil ou très, très fake.

*Mme:* OK.

*Mr:* Avec la petite paille pour mettre sous les légumes, [enfin] c'est gênant.

*Mr:* C'est "too much" [quoi].

*Mr:* Mais après pourquoi pas ? Ça fait, ces boutiques de ville [quoi]. Ça fait traite de type, [enfin], boutique de grandes villes [quoi]. Parisiennes [enfin], vraiment-- boutique citadine qui essaie de faire un, qui essaie d'en appeler au retour, retour à la terre [quoi]. On sent qu'il y a un-- une volonté commerciale qui n'est pas camouflée, que, que vraiment de dire chez nous, c'est sain, chez nous, c'est des, des vrais produits [euh]. Ce qui est peut-être vrai, j'en sais rien, mais je trouve que la mise en scène est un peu violente.

*Mme:* [Ouais], pour vous il y a une question de paraître dans le magasin que--

*Mr:* [Ouais], que je trouve très-- beaucoup trop mise en avant.

*Mme:* [Ouais].

*Mr:* C'est pas le genre d'endroit dans lequel j'irais facilement après [euh], mais après faut voir. Si ça se trouve, ça se trouve, je suis juste, je suis juste méchant et puis--

*Mme:* [Rire]. Non

*Mr:* C'est un très bon magasin [et tout]. [Voilà] je trouve--

*Mr:* C'est peut-être son tablier orange qui, qui parait, qui fait vraiment trop [quoi].

*Mme:* [Rire].

*Mme:* [Ouais], un côté. Pour vous, [du coup], le fait que ce soit un peu trop mis en scène, ça-- c'est contreproductif par rapport aux-- ?

*Mr:* [Ben] contre-productif, Il faut voir après ce qu'il y a derrière. Mais quand je vois ça--

*Mme:* [Ouais].

*Mr:* Toujours, je m'inquiète sur le, [ben] la volonté qu'il y a derrière et donc du coup les, les-- l'engagement moral qu'il y a derrière, est-ce que c'est quelqu'un qui surfe sur la mode du truc et qui fait juste ça pour faire du business et faire du pognon ?

*Mme:* [Ouais].

*Mr:* Ou est-ce que c'est quelqu'un qui a vraiment [euh], une conscience [euh] écologique [euh] et qui fait [du coup] ça pour, pour ça [quoi]. Mais est-ce que [du coup], ça va être quelqu'un qui va essayer de tirer sur les prix, d'arnaquer les producteurs, de [enfin], peu importe quelles sont [enfin] les techniques pour faire du pognon.

*Mme:* [Ouais].

*Mr:* Ce qui va nous ramener à des systèmes de grandes surfaces, [enfin], [voilà]. Des systèmes qui sont, qui sont des systèmes, comment dire-- [euh]. Merde--

*Mr:* De prédateurs [voilà]. Qui sont des systèmes de prédation et qu'on sait qui sont des systèmes dangereux [quoi]. Ce n'est pas bon, ce n'est pas dans l'intérêt commun. Ou alors, est-ce que c'est quelqu'un qui a une vraie volonté ? Mais quand je vois toute cette mise en scène, j'ai tendance à penser que c'est quelqu'un qui fait ça, parce que c'est à la mode et que c'est un bon plan, un bon plan business et que [voilà] ça va lui permettre de gagner du pognon, [quoi].

*Mme:* [Ouais]. [Ben] en fait, tout dépend en soi. En fait, c'est réel, entre guillemets.

*Mr:* [Ouais].

*Mme:* Mais c'est pas -- mais que [euh]--

*Mme:* Où le producteur est payé au juste prix.

*Mr:* [Ouais], c'est ça.

*Mme:* Où alors, au final, c'est le même, c'est la même, c'est le même système que le supermarché [enfin] dans le--

*Mr:* [Ouais], c'est ça la.

*Mme:* L'économie est la même--

*Mr:* On a bien un habillage, un habillage différent.

*Mme:* C'est juste une mise en scène.

*Mr:* [Hum].

*Mme:* Donc, ce n'est pas des lieux que vous fréquentez[euh] régulièrement ?

*Mr:* Non, j'aurais tendance, [bah] après, [ouais]. Après je suis quelqu'un de curieux, donc [euh] j'irai bien voir mais--

*Mme:* Il est à Brest.

*Mr:* Mais par exemple, il est à Brest ?

*Mme:* [Ouais].

*Mr:* Ok, parce que par exemple, les endives.

*Mme:* [Ouais].

*Mr:* Bon, même si ça pousse plutôt dans le Nord, on en fait chez nous.

*Mme:* [Ouais].

*Mr:* Donc, le coup des endives en sachet plastique comme ça par exemple, c'est toujours-- l'emballage sur le, sur des produits comme ça, [ben] [enfin]. Les endives, ça va, ça tient, ça tient le choc [quoi].

*Mme:* [Ben] [ouais], [ouais], [ouais].

*Mr:* Donc [euh], donc on est [euh]-- j'ai du mal à croire qu'il n'a pas réussi à trouver des endives locales [euh] sans emballages plastiques dessus par exemple.

*Mme:* [Ouais].

*Mr:* Et que probablement, c'est une histoire de prix. Ces endives-là, elles lui coûtent rien, il peut les vendre, il peut faire une marge qui est intéressante.

*Mme:* [Ouais].

*Mr:* Et donc, [du coup], il a des endives avec un emballage plastique dessus. [Enfin] [voilà], c'est le genre de truc que--

*Mme:* [Ben] oui, s'il y a bien un producteur [du coup], on a pas besoin de l'emballer.

*Mr:* [Ben] c'est ça, il n'y aurait pas besoin de [enfin]-- et puis on ne se ferait pas chier à mettre un emballage plastique. Donc [euh], donc [voilà], c'est toujours un-- tout genre de boutique. Donc, c'est à Brest, c'est ça ?

*Mme:* [Ben] oui. Parce que à la base [euh]-- les photos, j'avais pris un peu des photos un peu partout et l'ancien primeur que j'avais, ça ne marchait pas. Il avait un nom de pape, nœud pap' [et tout] les gens me disaient c'est quoi ça ? Ça ne marchait pas du tout [et du coup], lui, [ouais]. Et à Brest, je ne sais plus, je ne sais pas où ?

*Mr:* D'accord.

*Mme:* Je peux toujours choper--

*Mr:* Et ça, c'est une photo aussi récente ?

*Mme:* Non, non [enfin] c'est sur, oui [euh] c'est trouvé des choses libres de droit, mais [euh]. Je ne sais pas si c'est dans le coin [enfin], ça a l'air d'être des produits du coin de toute façon.

*Mr:* Oui, oui, oui, mais c'est parce que ça fait très années 90, oui.

*Mme:* Oui, c'est, c'est fort possible.

*Mr:* Oui, c'est ça.

*Mme:* Et lui, [ben] s'appelle Mon petit primeur.

*Mr:* [Ouais]. [ouais] et il y a--

*Mme:* Je crois que c'est-- [ouais]. Et c'est un article de journal [euh]. C'était sur Le Télégramme.

*Mr:* [Ah] [ouais], il y a peut-être de ça aussi dans la mise en scène. Une photo, c'est une photo de presse.

*Mme:* [Ouais], mais je pense qu'il y a toujours la paille quand même où la paille reste [euh]--

*Mr:* [Ouais].

*Mme:* Reste, un-- Mais [ouais].

*Mr:* [Ben] après--

*Mme:* Non, c'est bien de savoir. Je ne sais pas du tout.

*Mr:* [Ben] après c'est ça, la paille, le reste [et tout]. Mais bon, après, c'est pas parce que j'ai une conscience écologique que ça l'empêche d'être un commerçant le mec.

*Mme:* Oui.

*Mr:* Il peut très bien après, faire un truc pour appâter les gens. Et si ça fonctionne et si ça lui permet de vendre des produits qui sont des vrais produits de circuits courts [et tout], [enfin], je ne vais pas lui jeter la pierre parce que, parce qu'il cherche à vendre ses produits, [quoi].

*Mme:* [Ouais].

*Mr:* C'est juste, ça me questionne sur la motivation.

*Mme:* [Ouais].

*Mr:* Mais [euh], mais après, je ne voudrais pas, je ne voudrais pas préjuger non plus.

*Mme:* [Ouais], c'est bien. C'est rigolo de voir que, qu'il y a une volonté de rendre le magasin [euh]--

*Mr:* [Ouais], c'est ça.

*Mme:* Faut qu'on se demande si-- pourquoi il y a besoin de mettre une volonté de-- ?

*Mr:* C'est ça.

*Mr:* Dans l'étal comme ça, on est bien [et tout], [enfin]. C'est assez rigolo cette façon de faire.

*Mme:* Oui. Il faudra regarder [enfin], je ne sais pas, je connais-- je ne sais pas quels sont les produits.

*Mr:* Et puis c'est-- ce vert [quoi]. Avec [euh] les lumières dessus [et tout] [enfin]. C'est épatant.

*Mme:* Donc pourquoi pas ? Mais en fait, tout dépend de-- ?

*Mr:* [Ouais], c'est ça, pourquoi pas ? Après, il faut creuser. Il faut juste [euh], il faut juste aller voir et [euh] et puis voir comment ça se passe. Parce qu'il y a des magasins [enfin] [voilà], [euh] Biocoop par exemple, je--

*Mme:* [Ouais].

*Mr:* J'aime pas du tout. Je n'aime pas du tout [euh]. Et leur façon de faire du commerce en amont et [euh] l'ambiance qu'il y a dans leurs magasins [et tout], [enfin] [voilà], j'aime vraiment, Biocoop, c'est vraiment pas mon truc.

*Mme:* [Ouais].

*Mr:* Mais d'autres. Il y a Bio Monde, par exemple.

*Mme:* [Ouais].

*Mr:* Il y en a Daoulas, et un-- Je ne sais plus. Brest, de l'autre côté, je crois. Je ne sais plus. Bref.

*Mme:* [Ouais].

*Mr:* [Ben] je trouve ça vachement plus chouette [quoi]. Alors après--

*Mme:* Et c'est quoi la-- ? Qu'est-ce qui vous fait-- ?

*Mr:* [Ben], déjà parce que probablement, je connais moins Bio monde, alors que Biocoop, je sais comment ils travaillent et que [ben] j'utilise les mêmes systèmes de pressurisation, l'achat en gros [et tout] pour [euh], [ben] pour [euh]-- pour faire de la tune [quoi].

*Mme:* [Ouais].

*Mr:* [Euh] Bio monde, je connais moins. Ça a l'air d'être moins grand comme structure ou parce que les contacts que j'ai eu avec le personnel là-bas m'a plu.

*Mme:* [Ouais].

*Mr:* Donc [euh], leurs produits, je les ai trouvés [euh], bien.

*Mme:* [Ouais].

*Mr:* Et [euh] [ouais], leur façon de faire. Par exemple [euh], chez Biocoop, quand on veut acheter du vrac, il faut acheter le contenant en vrac Biocoop.

*Mme:* [Ouais].

*Mr:* [Voilà], donc on paye son contenant en vrac Biocoop pour pouvoir acheter du vrac, qui sont quasiment, exclusivement des contenants en plastique.

*Mme:* [Ouais].

*Mr:* Bio Monde [euh], il te propose les contenants en vrac, en plastique [et tout], machin. Tu viens avec ton contenant et il le tare et [voilà].

*Mme:* Oui.

*Mr:* Tu reprends, [enfin] [voilà]. Il y a plus qu'à remplir son contenant. Et puis ils s'en foutent. Tu mets avec ce que tu veux pour mettre ton produit dedans. Le but du jeu, c'est que t'achètes du vrac. Ce n'est pas que tu achètes le contenant vrac Biocoop.

*Mme:* [Ouais].

*Mr:* [Voilà], des petits détails comme ça qui font que, [ben] je, je trouve cette façon de faire plus honnête et plus cohérente que celle de Biocoop, qui va te vendre, qui va te faire, [enfin], [voilà] un exemple parmi d'autres mais qui va te vendre le contenant en plastique. Je ne veux pas de contenants en plastique [euh], [enfin]--

*Mme:* [Ouais].

*Mr:* [Voilà].

*Mme:* [Ouais].

*Mme:* Ok. Alors--

*Mr:* Ici, ici c'est pas bio, mais ici c'est pas mal.

*Mme:* Et il est ou ce-- ? Il est dans le coin ?

*Mr:* [Ouais], c'est ça. Il est derrière le Géant, [enfin] derrière, ça dépend de quel côté, de quel côté on se tourne. Mais, [euh] il est du côté du Géant. Je ne connais pas le nom des rues. C'est pas pratique.

*Mme:* Et [euh], et dedans c'est que-- c'est des produits locaux ? Non, c'est [euh]-- ?

*Mr:* C'est local.

*Mme:* [Ah], c'est que local.

*Mr:* Il doit y avoir du bio dans l'histoire. Mais le but c'est pas bio, c'est local, circuit, circuit court [euh]. Donc [voilà], il va y avoir quelques produits indus mais d'industrie du coin.

*Mme:* [Ouais].

*Mr:* Genre des chips Bret's, [euh] des trucs comme ça.

*Mme:* Oui.

*Mr:* Et puis après [ouais], c'est des, des produits de boucherie locale. Je suppose. J'ai pas acheté beaucoup de viande à là-bas. Je suppose que la provenance de la viande doit être aussi relativement locale.

*Mme:* [Ouais].

*Mr:* Et puis après on a tous les primeurs en local [quoi].

*Mme:* Ok.

*Mr:* Et que-- ils donnent même l'adresse de la ferme. Donc, au niveau de la traçabilité, l'adresse, le contact, on sait d'où ça vient [quoi].

*Mme:* [Ouais].

*Mr:* On peut appeler, il y a le nom du producteur. Donc on peut le--

*Mr:* On peut retrouver après. C'est assez, c'est assez transparent.

*Mme:* [Ouais]. Donc ça passe pas même.

*Mr:* [Ouais].

*Mme:* Ok, alors le prochain jeu. Vous mangez de la banane ?

*Mr:* [Ouais].

*Mme:* Alors, il y a 10 [euh]--

*Mr:* Quand est-ce que je m'arrête ?

*Mme:* C'est ça. Il faudrait les ranger à celle qui vous donne le plus envie à celle qui vous donne le moins envie.

*Mr:* [Ah] [ouais], c'est compliqué [du coup] parce qu'il y a deux sens. [Rire].

*Mr:* Je préfère ça. C'est compliqué, parce que celle-là, elle ne donne pas envie, mais je sais que dans quelques jours ce sera, ça sera bon.

*Mme:* C'est ça.

*Mr:* Mais alors que [euh], alors que celle-là. [Pfff]. C'est un peu tard [quoi].

*Mme:* [Ouais].

*Mme:* Ok, alors on va les mettre [euh], on va essayer de faire un [enfin]--

*Mr:* [Ah], ce truc dans l'ordre.

*Mme:* [Ouais]. Tu peux faire deux colonnes et je vais prendre en photo d'autre [euh]--. Allez, je prends en photo. Alors. Les bananes, elles ont des petites formes là ? Parce que-- pour que je puisse, après l'entretien en écoutant savoir de quelle banane vous parlez.

*Mr:* [Ouais].

*Mme:* Donc si on commence par la première. La ba--

*Mr:* Triangle vert.

*Mme:* Triangle vert [euh], si vous essayez de vous projeter, en imaginant la banane devant vous [euh] est-ce que vous pouvez me la décrire avec vos cinq sens [quoi] ? Là, cette banane, elle est comment pour vous ?

*Mr:* [Euh], [ben] elle dégage du parfum. Un parfum de banane classique, [enfin], pas [euh] un peu alcoolisée, acidulée, [enfin], là on est sur de la banane.

*Mme:* Oui.

*Mr:* [Euh], elle est encore ferme.

*Mme:* [Ouais].

*Mr:* Et puis [voilà], si on tient un peu là où si on appuie un peu là, elle doit, la peau doit craquer pour pouvoir pour s'ouvrir.

*Mme:* [Ouais].

*Mr:* [Hum] [voilà], après, c'est une peau de banane. Ça glisse.

*Mme:* [Ouais].

*Mr:* Elles sont bon-- [ouais], bien jaunes [enfin] [voilà], mais au top.

*Mme:* Au top de la banane !

*Mr:* Au top de la banane mûre. Pas toute verte, pas [enfin]--

*Mr:* [Ouais].

*Mme:* C'est comme ça que vous la préférez ?

*Mr:* [Ben] [ouais], c'est comme ça que [ouais]. C'est comme ça que je la trouve. Je la trouve mûre, bon après, entre celle-là et la suivante carré blanc--

*Mme:* [Ouais].

*Mr:* La différence n'est pas flagrante, c'est, ça mûrit tellement vite les bananes, il doit y avoir une demi-journée entre les deux.

*Mme:* [Ouais].

*Mr:* Mais [ouais], comme ça, un peu, une bonne, une banane que je mangerais sans problème.

*Mme:* Et, [euh], le carré orange [du coup] est-ce qu'elle est globalement pareil ou est-ce que-- ?

*Mr:* Elle est globalement pareille. La différence va être principalement visuelle, c'est-à-dire que la peau commence à, à noircir. Je ne suis même pas sûr que la chair ait commencé à noircir encore dedans, à moins qu'elle ait été tapée. Elle a l'air d'avoir pris un petit coup, par exemple. Peut-être qu'en dessous, il y a eu un petit peu de marque, [fff] mais elle est peut-être juste un petit peu plus molle, mais en fait, visuellement, elle est juste visuellement, un peu différente. Elle doit sentir plus fort aussi.

*Mme:* [Ouais].

*Mr:* Mais sinon, c'est pareil, c'est une banane qui se mange. Sans souci.

*Mme:* Ok. Alors la suivante est rond blanc.

*Mr:* Le rond blanc. Là, je pense que [voilà]. Là, on voit la queue, elle est bien, elle commence à être bien--

*Mme:* Oui.

*Mr:* Je ne sais pas. Je n'ai pas le terme. Elle est noircie en tout cas.

*Mme:* [Ouais].

*Mr:* Je pense que si elle était sur un arbre, elle serait tombée ou pas loin de tomber cette banane.

*Mme:* [Ouais].

*Mr:* Sinon après, [euh], la chair a dû brunir un petit peu, ça il y a quelques endroits, elle doit être fondante et assez sucrée. C'est pas les bananes que je préfère, mais ça reste, ça reste mangeable.

*Mme:* [Ouais].

*Mme:* OK, et [ben] le triangle [euh]--

*Mr:* Le triangle le bleu.

*Mme:* [Ouais].

*Mr:* C'est une banane verte, pas assez mûre, mais elle doit être, elle doit être ferme. Voir un peu dur, et puis pas sentir grand-chose, [enfin], c'est une banane que je ne mange pas, [en tout cas] pas tout de suite. Mais après, c'est pas très grave parce que--[bah] [voilà], ça c'est l'histoire de quelques jours [quoi].

*Mme:* [Ouais]. Donc là, vous attendez encore un peu [quoi] ?

*Mr:* C'est ça.

*Mme:* Ok. Alors le carré bleu.

*Mr:* [Euh], le carré bleu c'est [euh], une banane qui commence à être bien mûre. Je l'ai mange plus à ce moment-là les bananes.

*Mme:* Ok.

*Mr:* Mais [euh], après on en fait des muffins au chocolat et à la banane.

*Mme:* [Ouais].

*Mr:* Donc, ces bananes-là sont très bien pour faire ça. Elles sont très parfumées. Elles sont pas mal sucrées, puis assez grasses. Bien, elles se mélangent bien à l'appareil à muffin. [Donc voilà], ça reste-- [ben] [euh], [bah] c'est une banane que j'apprécie puisque j'aime beaucoup les muffins chocolat banane. [Du coup], quand elles sont dans cet état-là, pas de peine pour la banane.

*Mme:* [Ouais].

*Mr:* Elle part, elle part dans le muffin et puis c'est très bien.

*Mme:* [Ouais], donc il n'y a pas de regrets à l'avoir comme ça puisque--

*Mr:* Non, parce que je ne la jette toujours pas à ce moment-là. Elles sont toujours consommées. Pas de, pas de problème.

*Mme:* Ok. Et [euh], ce qui fait que vous ne la mangez pas crue, ça va être quoi ?

*Mr:* [Pff] Ça va être [euh], je pense déjà elle a du mal à se tenir. Je pense que la chair à l'intérieur, elle a vraiment, vraiment commencé à s'oxyder et que la banane a du mal à rester cohérente, je pense.

*Mme:* [Ouais].

*Mr:* Mais [euh], je ne la mange pas parce que j'aime bien les muffins. Je préfère en faire des muffins.

*Mme:* [Rire] Tu sais, c'est l'excuse pour faire mûrir.

*Mr:* [Ouais], c'est ça, exactement.

*Mme:* Et donc--

*Mr:* Je préfère que ça devienne un muffin qu'une banane qui va être un peu molle, que je vais apprécier moyennement. Parce que, parce que [voilà], elle sera pas, elle sera pas mûre comme, comme je les préfère.

*Mme:* [Ouais], donc c'est parce que vous allez préférer la manger en muffin, que la manger crue.

*Mr:* [Ouais], c'est ça.

*Mme:* Ok.

*Mme:* Alors, le triangle rose.

*Mr:* Le triangle rose. [Hum], elle est pas mûre. Je soupçonne même d'avoir rajouté du vert au Photoshop.

*Mme:* [Rire] Oui. oui.

*Mr:* Elle n'a pas l'air cool, cool cette banane.

*Mme:* Non, elle n'existe pas cette photo verte.

*Mme:* [Ouais], elle fait un peu [euh]--

*Mr:* [Ouais] , reverdi un peu par ici.

*Mme:* [Ouais]. [Rire].

*Mr:* Non, mais c'est une banane, c'est une banane que j'aurais même pas acheté.

*Mme:* Quand elles sont vertes, comme si elles sont mises sur le côté photoshopé.

*Mr:* [Ouais], non, bien sûr que non, je l'aurai même pas acheté. Elle n'est pas mûre [du coup], tous les régimes autour doit être pas mûre [enfin], ça ne sert à rien d'acheter. J'achète des bananes quand je peux les manger et qu'elles sont à une ou deux journées de les manger. Là, la chair, elle doit être dure, ça ne doit pas avoir de goût [euh], [enfin] c'est--

*Mme:* [Ouais].

*Mr:* Inintéressant.

*Mme:* [Ouais].

*Mr:* Donc, [euh], non. Bon, après [voilà], si j'en ai, si je l'ai mal-- malgré tout comme ça chez moi. J'attends qu'elle soit, qu'elle soit dans un des états précédents.

*Mme:* Oui.

*Mr:* Pour pouvoir la manger d'une façon ou d'une autre.

*Mme:* Mais quand vous achetez des bananes, vous les achetez de sorte à ce qu'elles soient-- ?

*Mr:* Je les achète dans l'état du carré blanc ou du triangle bleu, c'est-à-dire elles sont vraiment, elles sont, [voilà] le triangle bleu à peine mûr. Cela va me permettre de pouvoir les conserver un petit peu. Mais [euh], je vais pouvoir les consommer quand même aussi, assez rapidement.

*Mme:* [Ouais].

*Mr:* [Voilà], on stock pas du, de la banane pour la faire mûrir chez moi.

*Mme:* Oui.

*Mme:* Ok, et donc à partir de rond jaune ?

*Mr:* [Ouais], le rond jaune, je pense qu'il y en a encore à récupérer. Faut voire. Même très noircis sur le dessus, mais sur le dessous, la chair, la peau, elle a l'air d'avoir tenu le coup encore.

*Mme:* [Ouais].

*Mr:* Donc, je l'ai mise après le triangle vert, parce que le triangle vert, je suis sûr que la banane, je pourrai en faire quelque chose.

*Mme:* [Ouais].

*Mr:* Alors que le rond jaune, je ne suis pas certain, mais je pense qu'il y a quand même, il y a quand même, [enfin], je vais aller voir dedans pour récupérer, faire du muffin.

*Mme:* [Ouais].

*Mr:* Mais je vais aller voir dedans pour récupérer, pour voir ce qu'il y a à récupérer puisque je pense que tout n'est pas perdu, on peut encore en sauver une partie ou une grande partie. Et puis après moi, le triangle blanc, [pff]. Peut-être, on peut en sauver un peu. Peut-être si je suis motivé et qu'il me manque de la matière pour faire mon muffin. Je vais peut-être voir vraiment. Et [euh] le rose ? Non, c'est bon. Il faut, il faut apprendre à avoir perdu des fois et là, c'est un échec. Poubelle la banane !

*Mme:* Oui.

*Mr:* Même s'il faudrait aller voir à l'intérieur quand même parce que je soupçonne. La peau de banane c'est épais, ça va quand même vachement bien conserver la chair à l'intérieur.

*Mme:* [Ouais].

*Mr:* Mais bon, là, non, je ne vais pas mettre les mains dedans.

*Mme:* [Ouais]. Elle a commencé à être bien--

*Mr:* [Ouais]

*Mme:* Et [ouais].

*Mr:* Là, c'est, là, c'est bon.

*Mme:* Ok.

*Mr:* Non, non et puis ça, ça attire les insectes après.

*Mme:* [Ouais] comme [ouais]--

*Mr:* Après, il commence à y avoir des moucherons [et tout], [enfin]--

*Mme:* [Ouais], en fait, même elle, elle commence même à être un peu [ouais]. La peau commence à être--

*Mr:* [Ah] oui, être un peu--

*Mme:* En plus d'être noire. La peau, commence à être [euh]-- Ça commence à--

*Mr:* J'arrête là ! c'est fini. Non, la moisissure, c'est ma limite, vraiment--

*Mme:* [Ouais].

*Mr:* Sur les bananes.

*Mme:* [Ouais]. oui, il faut, c'est [enfin]--

*Mr:* [Ouais], [ouais].

*Mme:* C'est qu'elle a bien tardé.

*Mr:* Faut pas être malade non plus. Ça sert à rien.

*Mme:* [Ouais].

*Mme:* Et alors, selon vous, est-ce qu'il y a un moment donné ou la banane perd ou n'a pas encore, ça dépend, ces apports nutritifs ou-- ?

*Mr:* [Enfin], je me pose la même--

*Mme:* Seulement selon vous ? Justement un a priori sur ce que [euh]-- Ou alors là, dès le début et jusqu'à la fin-- ?

*Mr:* [Ben], non, ça devrait se modifier un petit peu puisqu'il y a des réactions chimiques qui se font dedans. Il y a de l'oxydation, il y a des trucs comme ça donc, [euh]. S'il y a oxydation, il y a une pi-, il y a une perte de quelque chose après.

*Mme:* Oui.

*Mr:* Après [fff], après dans quel sens ça fonctionne ? Parce qu'il doit pas y avoir que de l'oxydation. Il y a une-- [pff] ça se modifie, sûre, il y a un truc qui change. Dans quel sens ? S'il doit y avoir de la perte après, après, est-ce qu'il y a de la perte tout le temps ? Est-ce qu'il y a de la perte tout le temps ? Ou est-ce qu'on entre, par exemple, celle-là et celle-là ou celle-là, on ne va pas gagner des-- un peu, un peu en valeur. Parce qu'on va arriver sur une maturation qui va vraiment être optimale. Et après, quand on a commencé à avoir une détérioration de perte, je le mettrais plutôt dans ce sens-là.

*Mme:* [Ouais], comme si il y avait une--

*Mr:* [Ouais], c'est ça.

*Mme:* Une courbe.

*Mme:* Oui.

*Mr:* Mais sur une modification, [enfin], de toute façon, ça se voit là, au niveau, à part en nutritif rond, rose, ça ne doit pas être top, top [quoi].

*Mme:* [Ouais].

*Mme:* Et [euh], est-ce que, selon vous, il y a à un moment donné où la banane peut-être potentiellement dangereuse [euh] pour la santé [quoi] ?

*Mr:* Dangereuse, genre dangereuse, maladie grave ou dangereuse, vomissement, diarrhée ?

*Mme:* Ça peut être, qu'il y a plusieurs échelles comme [euh]--

*Mr:* [ouais]. J'ai tendance à penser que les fruits [euh]. Les fruits et légumes [euh], pour s'empoisonner avec, pour vraiment s'empoisonner gravement avec, il faut y mettre de la volonté puisque, [ben] il faut les manger ce truc-là et [enfin] moi, moi je vomis, ça ne reste pas dans mon organisme très, très longtemps.

*Mme:* [Ouais].

*Mr:* Un truc comme ça. Donc [euh]. Avec de la volonté, il y a moyen que ça soit dangereux, mais sinon, non, [enfin], ça se sent. Il y a pas, on ne peut pas se faire piéger avec un fruit, un fruit pourri et tomber malade en ayant mangé un fruit qui n'était plus bon. Une banane qui était plus bonne sans s'en rendre compte, comme ça peut arriver avec une viande qui est un peu passée.

*Mme:* [Ouais].

*Mr:* Un produit laitier qui est un peu passé avec une banane, non, il n'y a pas de risque. [Ben], à moins d'avoir perdu le goût [quoi], [enfin]. Et puis même la texture [et tout] [enfin].

*Mme:* [Ouais].

*Mr:* Après, [euh], après le moisi, probablement que ce n'est pas très bon pour l'organisme.

*Mme:* Oui.

*Mr:* Mais Il faut déjà que ça rentre.

*Mme:* Oui.

*Mr:* Déjà faut voir, [enfin], parce que, faut la manger la banane pour une fois.

*Mme:* Et [euh], donc pour vous c'est le, c'est vos sens qui vont vous dire [euh] non là ce n'est pas-- ?

*Mr:* [Ouais] c'est ça. Ouais, [voilà], c'est un mélange d'un sensé instinct de survie et moi, mon corps, [bah] non, t'es con, arrête [quoi]. N'insiste pas, c'est débile.

*Mme:* [Ouais].

*Mr:* Non, je, [enfin], je, je ne m'inquiète jamais, moi quand je mange un fruit. Je me dis jamais, [ouais], ça fait longtemps que je l'aie. Parce ce que je le vois. Il est bon ou il n'est pas bon.

*Mme:* [Ouais]. Et [euh], des fois, même s'il n'est pas bon, en goût, en texture, est-ce que à votre avis [du coup], ça signifie qu'alors c'est notre corps qui nous dit, c'est qu'il n'est pas sain ? Ou alors, des fois, il y a quand même le goût qui commence à être mauvais--

*Mr:* [Ouais], ça commence par juste le goût. Ça commence par un fruit qui va être, qui ne va pas être très bon. Qui va être un peu acide et un peu aigre, [enfin], [voilà]. Qui va avoir [ben] une caractéristique gustative de, un, un truc qui est en train de passer [quoi].

*Mme:* [Ouais].

*Mr:* Mais [euh], non, il n'y a pas de danger. Non, je ne pense pas qu'il y ait eu un danger particulier, un danger-- [mouais]. Après, ça peut dépendre du type de fruits. Il y a des fruits qui peuvent s'alcooliser par exemple. Donc, il faut se méfier avec l'alcool quand même, selon le type d'alcool en plus. Je ne suis pas sûr que les fruits qui s'alcoolisent fassent forcément de l'éthanol.

*Mme:* Oui.

*Mr:* Méthanol. Je ne sais plus lequel est, [enfin], [voilà], mais il y a des types d'alcool qui sont quand même un-- vachement plus poison, que l'alcool qu'on consomme.

*Mme:* [Ouais].

*Mr:* Donc, peut-être sur certains, mais il pourrait y avoir ce genre de phénomène. Mais, [pff], [ouais] fondamentalement, je pense que les fruits, même pourris [euh].

*Mme:* Oui.

*Mr:* On n'est pas sur, sur un grand danger [quoi].

*Mme:* [Ouais].

*Mr:* On est sur quelque chose qui va être, [ouais], de l'ordre de, [ben] [ouais] ça, je pense que ça peut un peu rendre malade puisque l'organisme va fournir, il va réagir.

*Mme:* [Ouais].

*Mr:* Il va réagir au fait d'avoir un produit pourri, mais il ne va pas réagir violemment. Il ne va pas y avoir, je pense, d'ulcères, d'occlusion, de trucs comme ça, on ne va pas, on ne va pas se faire de, de-- on va pas se faire grand mal [quoi]. On va passer une sale journée et puis, et puis ça sera passée [quoi]. En fait, ça sera évacué. Le corps va trouver un moyen de l'évacuer dans un sens ou dans l'autre. Et puis une fois que ce sera évacué. Plus de problème [quoi].

*Mme:* Oui. Oui, et puis vous dire ça, ça c'était trop ?

*Mr:* [Ouais], c'est ça. [Voilà], ça, c'était vraiment non, t'as, t'as, t'a, t'as déconné, celui-là, il était vraiment plus bon [quoi].

*Mr:* Mais non, je pense. Je pense que, même si gustativement, on arrive à faire abstraction et à avaler, le truc, on ne se met pas en danger.

*Mme:* [Ouais].

*Mr:* Après [voilà], toutes proportions gardées, si on fait ça, une qui fait 10 kilos.

*Mme:* [Ouais].

*Mr:* Potentiellement, qu'est-ce qui est plus important, [enfin] ?

*Mme:* Ça dépend des organismes aussi [euh]--

*Mr:* [Voilà], c'est ça. Quelqu'un de particulièrement fragile où [quoi], [enfin]. Mais dans un cas normal, avec une adulte--

*Mme:* [Ouais].

*Mr:* Qu'est-- qui a une santé [euh], une santé [euh] classique [quoi], sans--

*Mme:* Sans problèmes de--

*Mr:* Sans problèmes particuliers. [Ouais], c'est ça. On se met pas en danger.

*Mme:* Ok. Alors--

*Mme:* Ici--

*Mr:* Bon, après j'ai tendance à penser qu'on ne se met pas en danger. Je n'irai pas faire les expériences.

*Mme:* Oui.

*Mr:* [Rire], Ça reste [euh]. [Voilà].

*Mme:* Oui, vous vous mettez déjà vos propres limites.

*Mr:* [Voilà], c'est ça. J'ai tendance à penser que ça se passerait bien. Mais je n'irai--

*Mme:* Oui.

*Mr:* Pas vérifier.

*Mme:* Pas se rendre malade pour regarder à quel point-- le côté--

*Mr:* [Ouais], c'est ça.

*Mme:* [Ouais].

*Mr:* Et puis, si un jour on me dit que c'est pas vrai, je l'admettrai sans problème, je dirai [Ah] ! Ok. Je le ferais pas, je le ferais tout de même pas.

*Mme:* Et [ben] [ouais].

*Mme:* Alors. Là, vous avez des [euh] photos.

*Mr:* Oui.

*Mme:* De ce qu'on peut faire avec un fruits et légumes.

*Mr:* [Ouais].

*Mme:* Notamment lorsqu'ils commencent à s'abîmer ou pas. C'est vrai qu'il y a des différentes poubelles.

*Mr:* [Ouais].

*Mme:* Et il y a différentes préparations où [euh] lieu de stockage et d'essayer de garder celles qui [euh] vous évoquent quelque chose. Celle qui [voilà] qui vous parle. Ce que vous pouvez faire [euh]. Mais là, c'est général.

*Mr:* Pour la conservation ?

*Mme:* Pour la conservation ou la consommation.

*Mr:* D'accord, [Ah] oui, parce que [ouais]-- Ok.

*Mme:* Salade de crudités. Est-ce que cela vous arrive d'en faire ?

*Mr:* [Euh], [ouais]. J'en mange, j'en mange un peu, [Prénom de la conjointe] en mange beaucoup.

*Mme:* Ok.

*Mr:* Beaucoup de crudités, de salade de crudité [et tout]. Moi, moins en comparaison. Mais [ouais], ça nous arrive, [enfin], moi, ça m'arrive et [Prénom de la conjointe], c'est--

*Mme:* Fréquent.

*Mr:* C'est fréquent [ouais]. C'est une de ces sources de nourriture quand on fait des trucs sur lesquels elle est pas top. J'ai fait des croques monsieur, l'autre fois pour elle et pour moi, elle s'est fait une salade de crudités pour [euh]--

*Mme:* [Ouais].

*Mr:* Pour manger à la place. Donc [ouais], [ouais], c'est un mode de consommation pour certains produits, c'est même le mode de consommation principal.

*Mme:* [Ouais].

*Mr:* Moi j'aime en crudités, en salade comme ça.

*Mme:* [Ouais].

*Mr:* [Euh], donc, [voilà]. La salade ça marche, la salade par exemple.

*Mme:* [Rire]. Par exemple.

*Mr:* Par exemple. [Euh], congélation. [Euh], on fait mais peu parce que le congelé fait huit litres.

*Mme:* [Ouais].

*Mr:* Donc [euh], donc, on est limité. Une question d'espace. Mais on est, on en parlait encore à midi ou avant-hier de s'acheter un vrai truc congèle parce que, parce que c'est pratique, c'est un très bon mode de conservation et pratique quand on fait des gros plats pour pouvoir les congeler. Et puis c'est pratique dans la consommation. C'est-à-dire que on va avoir des trucs, alors là, c'est juste des trucs qui ont été épluchés ou détaillés, qui ont été--

*Mme:* [Ouais].

*Mr:* Qui ont été stockés comme ça. Alors ça reste pratique. Ça a l'air d'être pour des gens qui, genre pour une cuisine, ou un truc comme ça, mais. Mais [euh] [voilà] les trucs tout fait, quand ils sont congelés [euh], on ne sait pas quoi faire à manger le soir, on le sort, on le décongèle. [Voilà], c'est pratique pour conserver, c'est pratique pour consommer, je trouve [euh].

*Mme:* Le congèle [ouais].

*Mr:* [Ouais]. Salade de fruits. Mais on n'en fait pas tant. On n'en consomme pas tant de temps en temps pour des soirs de fête, des soirs de repas, genre repas de Noël ou, repas d'annivair--, [enfin] de goûter d'anniversaire. Des trucs comme ça, ça peut nous arriver. Mais c'est, effectivement, mais c'est pas un truc qu'on fait, qu'on pratique.

*Mme:* [Du coup] vous en faites trop ?

*Mr:* Non, non, non.

*Mme:* Ok.

*Mr:* Les fruits, maintenant, c'est les manger [euh]--

*Mme:* Tel quel, [quoi].

*Mr:* Tel quel [ouais] [euh]-- [Mouais] non, [mouais]. Si on les mange tel quel, c'est juste qu'on a des trucs pour les pertes, on ne mange pas, on mange pas beaucoup de fruits différents.

*Mme:* [Ouais].

*Mr:* Beaucoup des pommes, des bananes quand il n'y a pas de clémentines, parce que c'est surtout [Nom de l'enfant] qui mange les bananes. Et là, en ce moment, on mange des clémentines.

*Mme:* [Ouais].

*Mr:* Exclusivement des clémentines. [Et voilà], c'est surtout [voilà], des pommes, des bananes.

*Mme:* [Ouais].

*Mr:* Et les clémentines quand c'est la saison, donc [euh]--

*Mme:* Parce que vous allez faire [euh]-- ?

*Mr:* On va pas faire particulièrement de salade de fruits.

*Mme:* Ok.

*Mr:* Ça, c'est un--

*Mme:* C'est un gaspacho.

*Mr:* Gaspacho, velouté. Non.

*Mme:* Velouté de légumes, froid, [ouais].

*Mr:* La soupe, oui, mais en froid comme ça ? Après, faudra tester. Mais [euh]--

*Mme:* [Ouais].

*Mr:* Mais non. Le compost, non, parce que [euh]-- [ben]parce qu'on est pas équipé.

*Mme:* [Ouais].

*Mr:* Donc pas de compost. Mais [euh] dans l'idée, oui.

*Mme:* [Ouais].

*Mr:* [Enfin], [voilà], dans l'absolu, si on pouvait, on aurait un compost et on composterait. Mais à l'heure actuelle-- la compote ou j'aime beaucoup la compote en plus. Et puis pomme kiwi par exemple.

*Mme:* [Ouais].

*Mr:* Deux fruits que l'on mange, ça fonctionne très bien.

*Mme:* [Ouais].

*Mr:* [Euh], des poêlées de légumes, bien entendu.

*Mme:* [Ouais],

*Mr:* [Ouais].

*Mme:* Vous faites.

*Mr:* Bien sûr, souvent. [Euh] les--

*Mme:* [Rire].

*Mr:* Et non.

*Mme:* Non pas encore.

*Mr:* Non, mais non, de la paille, des poules, ça chient partout.

*Mr:* Quand on aura un extérieur, on en aura.

*Mme:* [Ouais].

*Mr:* C'est pareil. On en aura genre deux. Parce que après, faut faire quelque chose avec les œufs [quoi]. Vite, c'est--

*Mme:* [Ouais].

*Mr:* Vite les poules, c'est l'enfer, des boulets [euh)--

*Mme:* Smoothie, aussi pas trop--

*Mr:* Smoothie, pas trop non plus. [Ouais], si on doit pouvoir en faire, on doit pouvoir avoir les appareils.

*Mme:* Ou la passoire.

*Mr:* Non, pas smoothie.

*Mme:* [Ouais].

*Mr:* [Euh] ça, c'est la poubelle.

*Mme:* [Ouais], la poubelle.

*Mr:* [Ben], la poubelle, [ouais]. [Enfin], c'est pas pour le moment, c'est votre poubelle mais--

*Mme:* [Ouais], du coup, [ouais].

*Mr:* Mais oui. Tarte aux pommes. Oui, même si on ne les fait pas comme ça, ça reste--

*Mme:* Oui, tarte aux pommes ou [euh] même vos muffins bananes. [Voilà], c'est dans cette idée--

*Mr:* [Ouais], [voilà], c'est ça. Des soupes, des soupes oui.

*Mme:* Alors ok. Alors là, l'idée, c'est qui va y avoir des fruits et légumes.

*Mr:* [Ouais].

*Mme:* Que des personnes ont jeté.

*Mr:* D'accord.

*Mme:* Et dire pourquoi, ils l'ont jeté.

*Mr:* Est-ce que je les jette ?

*Mme:* [Et voilà], vous, qu'est-ce que vous faites ? Est-ce que [du coup] c'est pareil ? C'est poubelle ? J'ai, j'ai-- il me manque une photo, je sais pas pourquoi. Et [euh], normalement, il y avait des bocaux. Est-ce que vous faites des bocaux lors des confitures ou pas ?

*Mr:* Non.

*Mme:* Non.

*Mr:* Non, non, non on ne fait pas. [Prénom de la conjointe] a peur du botulisme.

*Mme:* [Ouais].

*Mr:* On fait pas, on ne fait pas de bocaux, mais [euh] ça viendra peut-être. [Enfin], c'est comme [euh], c'est comme [euh]. [Enfin] moi, j'en ferais parce que j'aime bien faire de la ratatouille, par exemple l'été, et j'aime bien en manger l'hiver.

*Mme:* [Ben] [ouais].

*Mr:* [Voilà].

*Mme:* Carrément.

*Mr:* [Euh] [pff], ça [euh], ça [euh] là, là ou là ça reste [euh]--

*Mme:* Alors, c'est le radis, ça, vous le consommez ?

*Mr:* [Mouais], [ben ] il m'a l'air, je vois pas ce qu'il en fait de ce radis. Non, mais [euh]-- Bon, après, faut voir. [Mouais] il m'a l'air très bien ce radis. Il m'a l'air très bien.

*Mr:* [Euh] ça [euh] [ouais], j'enlève à la limite, j'enlève peut-être le petit bout oxydé là, le petit, la petite queue un peu oxydé de la--

*Mme:* De la salade là ?

*Mr:* De la salade mais [euh]--

*Mme:* Sinon, vous [euh]--

*Mr:* Ça passe. Alors, c'est tomate, c'est pareil. [Ben], On a l'air très très bien en soupe, à la limite. Mais [euh] en crudités-- les agrumes-- bizarrement sur les fruits, par exemple les agrumes. Je vais vraiment pas [euh]--

*Mme:* Vous allez pas forcer [quoi].

*Mr:* Je ne vais pas forcer [ouais], je ne sais pas, je ne sais pas pourquoi [hein].

*Mme:* Les agrumes, quand ils commencent à-- ?

*Mr:* [Ouais], je sais pas. Il y a-- l'agrume, ça me paraît moins, moins friendly [ouais].

*Mme:* [Ouais].

*Mr:* Comme ça, moisi.

*Mme:* [Ouais].

*Mr:* Donc, [euh] je ne vais pas les tenter. [Euh] Ça c'est--

*Mme:* [Ouais].

*Mr:* C'est bien pourri aussi. Sinon--

*Mr:* [Pff], non je vais pas me faire chier.

*Mme:* [Ouais].

*Mr:* À gratter là.

*Mr:* Mais, c'est pareil, ça a bien noirci en dessous.

*Mme:* [Ouais], doutez pas--

*Mr:* Non.

*Mr:* La carotte, [euh], bon [bah] si à la limite ces petits bouts de carotte, à la limite.

*Mr:* [Euh], mais [euh] tout, c'est pareil. Ça tout à fait, bien sûr.

*Mme:* [Ouais].

*Mr:* C'est muffin, direct.

*Mme:* [Rire], [ouais] trop bien.

*Mr:* C'est ça. C'est du vert de poireaux ?

*Mme:* [Ouais].

*Mr:* [Euh], je consomme peu le vert de poireau, mais à la limite, dans une soupe, il m'a l'air-- De toute façon dans une soupe, tout passe.

*Mme:* [Ouais].

*Mr:* Et, effectivement, il faudrait peut-être le détailler sur certains endroits, à la limite, mais bon.

*Mme:* [Ouais].

*Mr:* [Ouais]. [Hum]. Ça passe mais je suis pas fan du poivron. Ça passerait [euh]-- je pense ça passe sur--

*Mme:* En poêlé ?

*Mr:* [Ouais]. On enlève. Après, faut voir dans quel état c'est a l'intérieur. Mais une fois ouvert, je pense qu'il est encore, qu'il est encore bon. Il est juste un peu, un peu flétrie mais--

*Mme:* [Ouais].

*Mr:* Mais-- et puis ça, ça passe aussi. Alors peut-être pas en crudités. Elles sont peut-être un peu molles pour faire des carottes râpées. Mais alors en soupe ou en souple ou en poulet.

*Mme:* [Ouais].

*Mr:* [Ouais], [ouais], [ouais]. [Ouais] [ouais] tout à fait.

*Mme:* Ce qui fait que vous n'allez pas la mettre en crudités. [Du coup], c'est plutôt la--

*Mr:* La, la, la-- [euh] [ouais]-- [euh]--

*Mme:* La texture que--

*Mr:* [Ben], j'ai peur qu'elle soit, qu'elle soit un peu mal ou avec [du coup] en crudités, ce soit moins agréable. J'aime bien quand c'est croquant.

*Mme:* [Ouais].

*Mr:* Surtout la carotte râpée dans une salade comme ça, ça, [enfin] ça ajoute quand même ce côté un peu [euh] croquant [et tout]. Où- là, je pense que ça, ce sera sans intérêt. [Enfin] [voilà]. Mais après ça, après ça dépend [voilà]-- ça va dépendre-- est-ce que je suis en train de faire quelque chose à manger et que je le vois à ce moment-là et je me dis [ah] tiens, ça passe dedans ? Ou est-ce que la volonté, ça va être de sauver quelque chose que je suis en train de perdre ? Et dans ces cas-là, je vais faire, [enfin], [voilà].

*Mme:* Et là, si vous étiez en train de faire des carottes râpées ?

*Mr:* Si j'étais en train de faire des carottes râpées que j'en avais déjà passé quelques-unes à la limite, pourquoi pas mettre celles-là en plus ? Ça, oui, ça ne pose pas vraiment de problème.

*Mme:* [Ouais].

*Mr:* Mais [euh], mais [voilà], mais c'est le but du jeu, ça va être de sauver les carottes [euh], ça va dépendre sur ce que j'ai en plus avec nous, ça va être soit une soupe, soit une poêlée.

*Mme:* [Ouais], vous avez, vous allez plutôt la cuire.

*Mr:* La cuire, [ouais], c'est ça. C'est pour pour que la texture elle ne soit pas [euh]--

*Mme:* [Ouais].

*Mr:* Ça va, je sais pas--

*Mme:* Ça, c'est une photo de-- et tout ça, vous jetez aussi ?

*Mr:* [Ouais], tout ça, [ouais], je jette. [Ouais], [ouais], les endives. Mais après, faut globalement-- celles-là, faudrait voir, mais ça m'a l'air d'être noirci en dessous. Ça vaudrait le coup d'ouvrir pour voir dans quel état c'est en dessous, mais--

*Mme:* Mais en fait--

*Mr:* J'ai peur qu'elle soit perdue quand même.

*Mme:* C'est vrai que c'est [euh]-- elle [euh]--

*Mr:* Elle seule.

*Mme:* C'est-- ok. Celle qui est noire, celle qui est caca--

*Mr:* [Ouais], c'est ça. Mais [euh] [voilà]. Celle-là, ça dépend de l'état en dessous mais--

*Mme:* [Ouais].

*Mr:* Parce qu'après un peu trempé dans l'eau, ça va redonne un peu, un peu de croquant et [euh]--

*Mme:* [Ouais], vous croquez vous aussi ? Enfin vous croquez ! Vous, vous remettez les-- je me souviens c'était les radis que vous faisiez ça.

*Mr:* [Ouais], les radis dans l'eau [euh], les carottes, ça marche aussi dans les carottes quand elles sont un peu molles. Un coup dans la flotte [quoi]--

*Mme:* Et ça-- ?

*Mr:* En fait, ça-- c'est juste pour les consommer [euh].

*Mme:* Oui.

*Mr:* Les consommer [quoi]. C'est pas pour--

*Mme:* C'est pas pour [euh]--

*Mr:* [Ben, c'est pas pour dire, [Oh] elle est belle [voilà], elle fait trois ou quatre jours [quoi].

*Mme:* Non.

*Mr:* Là, elle en peut plus la carotte, mais elle se regonfle, un coup de flotte et [du coup], [du coup], ça permet de retrouver un peu de croquant. La salade pareille, tous ces trucs-là [euh]--

*Mme:* Et [euh], vous mettez ça combien de temps ? C'est une journée dedans ou c'est juste [euh]-- ?

*Mr:* Non, c'est histoire d'une demi-heure. Après, ça dépend [euh]--

*Mme:* Ça suffit.

*Mr:* En général, c'est plutôt de l'ordre d'une demi-journée, mais en une demi heure, ça fait déjà--

*Mme:* [Ouais].

*Mr:* Ça a déjà fait le taf en général.

*Mme:* [Ouais].

*Mr:* C'est plutôt-- [ben] [voilà], ça va, ça va correspondre au moment où je me dis qu'est-ce que je fais à manger ?

*Mme:* Oui.

*Mr:* Donc, ça arrive vers 14 h en général. Le qu'est-ce que je fais à manger ? Qu'es- ce qu'on fait à manger ce soir ? [Et du coup], [voilà], je vais faire le tour des placards [et tout] et puis--

*Mme:* Et si [euh], si il y a des carottes un peu molles [enfin] là-- ?

*Mr:* [Ouais] c'est ça, je peux les mettre à ce moment-là dans l'eau pour qu'on le soit-- à la limite pour le lendemain midi.

*Mme:* [Ouais].

*Mr:* Mais [ouais], ça marche, ça marche très bien.

*Mme:* Ça marche bien, avec les endives, la salade ?

*Mr:* [Ouais], [ouais], les endives, la salade ou tout à partir du moment où il n'y a pas une peau qui est un peu, un peu imperméable, les tomates, ça va moins bien fonctionner parce que je pense que la peau est--

*Mme:* [Ouais].

*Mr:* Elle permet moins d'absorber la flotte, mais à partir du moment où [euh], où ça peut absorber l'eau [euh]--

*Mme:* Ça marche.

*Mr:* Ça les regonfle un peu. [Euh], [enfin] [voilà]. C'est juste de la flotte qui re-rentre dedans [quoi].

*Mme:* [Ouais].

*Mr:* C'est, je pense que c'est principalement ce qu'ils ont perdu à ce moment-là mais--

*Mme:* [Ouais], c'est qu'elles sont déshydratées ?

*Mr:* [Ben] [ouais], c'est ça. Elles commencent à sécher donc [euh]--

*Mr:* Juste un peu d'eau.

*Mme:* Ok, faudrait que j'essaie ça.

*Mr:* [Ben] [ouais], [voilà], ça joue, ça joue pas grand chose [hein]. Mais par exemple sur un radis, entre un radis croquant et un radis un peu mou [euh]--

*Mme:* [Ouais], on le préfère souvent [euh]--

*Mr:* [Ouais], c'est ça. Laisser dans un bol d'eau dans, pendant, pendant 1 h ou deux avant de le manger, ça coûte quand même pas grand chose et c'est plus agréable à manger après.

*Mme:* [Ouais]. [Ben], c'est marrant, c'est [euh]-- et la salade c'est pareil, c'est juste avant ou vous la-- ?

*Mr:* La salade, juste avant, mais avant on la conserve. [Prénom de la conjointe], elle a une technique, mais on en avait déjà parlé.

*Mme:* [Ouais].

*Mr:* En, sachet avec une feuille de Sopalin pliée dedans.

*Mme:* Pour l'humidité du--

*Mr:* Puis qui va faire tampon, qui va prendre ou redonner selon les écarts de température. Et [euh] ça les conserve très bien. Mais moi, quand elles commencent à être un peu, quand elles tiennent plus.

*Mme:* [Ouais].

*Mr:* Elles sont un peu fatiguées avant, avant d'être vraiment, avant de s'oxyder mais elle commence à être molle [quoi].

*Mme:* [Ouais].

*Mr:* Un coup de flotte, ça repart. Les feuilles de chou, c'est pareil.

*Mme:* D'accord.

*Mr:* On a un chou chinois. Il commence à être un peu vieux.

*Mme:* [Ouais].

*Mr:* Et [euh], on en mange en crudité des fois, le chou chinois [enfin]--

*Mme:* [Ouais].

*Mr:* En crudité ou tout cuit. Et[euh], je retrempe les feuilles dans l'eau quand c'est la fin. Comme ça, je retrempe les feuilles dans l'eau avant de les cuisiner. Ça leur redonne un peu de, un peu de croquant, un peu de-- un peu de, un peu de, texture [quoi]. [Enfin] elles deviennent un peu, un peu de ferme, fermeté.

*Mme:* [Ouais], c'est ça. Au final c'est [euh], c'est [euh], c'est exclusivement la texture que ça va relancer ?

*Mr:* [Ouais], c'est ça.

*Mme:* Pas forcément en goût ou [quoi], c'est juste le--

*Mr:* [Ouais], c'est ça, ça va les rendre [euh]. Ça va aller [ouais], ça va-- comme s'ils avaient un jour de moins [quoi], globalement, [enfin] un jour de moins.

*Mme:* Oui, oui.

*Mr:* Un peu ferme [quoi]. Mais oui, ça joue, ça joue exclusivement sur la texture. Et puis je pense pas que ça se conserve mieux ou que [euh], [enfin] [voilà], après les avoir fait tremper, que ce soit devenu ferme. Elles aient regagné la journée, qu'elle avait perdu ou quoi que ce soit, je pense que le processus de détérioration continue de la même façon. C'est juste [voilà] de façon ponctuelle. On rattrape un état--

*Mme:* [Ouais].

*Mr:* Mais [voilà], je pense que ça continue, [enfin] [voilà]. Sur le même processus et que ça, ça fait gagner aucun temps. Il n'y a pas, d'avantage d'un point de vue de conservation à faire ça. C'est juste pour la consommation.

*Mme:* [Ouais], c'est un peu le-- c'est le-- la stratégie de la dernière chance pour lui redonner un--

*Mr:* [Ouais], c'est ça, [ouais]. Le côté agréable, avant le, avant de le manger. C'est ça.

*Mme:* Oui.

*Mme:* C'est pas mal, notamment quand on veut manger cru [du coup].

*Mr:* [Ouais] c'est ça. C'est pour les crudités, ça,

*Mme:* [Ouais].

*Mr:* Ça joue pas mal parce que c'est les crudités. C'est cool quand c'est croquant [quoi]. À partir du moment où ça commence à être un peu mou, flasque [et tout]. Là, c'est moins kiffant le crudité là, c'est pas--

*Mme:* Oui c'est vrai. Je vois, je m'imagine une assiette de crudités et que des trucs un peu [euh]--

*Mr:* [Ouais], c'est ça.

*Mme:* Un peu mousse.

*Mr:* Une salade de carottes râpées un peu molles.

*Mme:* [Ouais]. C'est pas [euh]--

*Mr:* Le râpé de cantine là--

*Mme:* [Ouais]. Ça donne, ça donne moins--

*Mr:* Non, ça c'est pas-- quand on se dit que les carottes râpées, on ne pense pas à ça, [quoi].

*Mme:* [Ouais]

*Mr:* Pense à la bonne carotte fraîche, croquante.

*Mme:* [Ben] c'est ça [ouais]. On disait fraîcheur dans les crudités.

*Mr:* [Ben] [ouais], [ouais].

*Mme:* De fraîcheur en bouche et de fraîcheur même de, du produit en fait.

*Mr:* C'est ça [ouais]. Il faut que [enfin], [voilà] que-- c'est ce qui plaît dans les crudités. À mon sens, maintenant [en fait], c'est ce qui me plaît.

*Mme:* Mais oui oui oui [ouais].

*Mr:* Quand on en parle, ça avait l'air d'être ça.

*Mme:* Mais oui, au final, c'est ce qu'on retrouve un peu partout. [Et du coup], il va y avoir une attente, une attente sur les fruits et légumes.

*Mr:* [Ben] [ouais] [ouais].

*Mme:* Qui est supplémentaire par rapport à-- quand il part en cuit [quoi].

*Mr:* Et pour le radis, je trouve ça important parce que j'aime pas le radis particulièrement.

*Mme:* [Ouais].

*Mr:* Je ne mange pas de radis comme ça, mais j'en mets des fois dans les plats.

*Mme:* [Ouais].

*Mr:* Ou dans des salades composées.

*Mme:* [Ouais].

*Mr:* Mais c'est du radis que je mets [euh], alors dans les salades composées il est cru, coupé en lamelles et dans les plats, il est presque cru parce que en fait, je n'aime pas trop le goût du radis, mais c'est pas particulièrement fort [quoi].

*Mr:* [Ouais].

*Mr:* Mais j'aime bien le croquant que ça apporte dans les plats ou dans les salades composées. Donc j'en mets. Et donc, effectivement, quand il commence à être un peu mou, pour moi, le radis n'a plus aucun intérêt.

*Mme:* [Ouais].

*Mr:* Parce que j'aime pas trop le goût et il a plus le croquant.

*Mme:* [Ouais].

*Mr:* Donc, effectivement, le tremper dans l'eau comme ça, ça permet de retrouver de l'intérêt et de, du plaisir à le consommer derrière.

*Mme:* [Ouais].

*Mr:* Et puis [voilà], c'est juste une question de plaisir. Le produit, il est, il a, il a les mêmes bénéfices. [Euh] qu'ils aient été trempés dans l'eau ou non, il va faire, [enfin] [voilà] c'est-- il va apporter les mêmes, les mêmes trucs dans mon organisme. [Enfin], si-- ça ne change pas [quoi].

*Mme:* Oui.

*Mr:* C'est juste de la flotte, c'est pas magique [quoi].

*Mme:* [Rire] [Ouais], c'est juste le-- pour vous lu-- [enfin] il y a toujours l'intérêt de l'apport nutritif de tous les fruits et légumes.

*Mr:* Oui.

*Mme:* Mais ce qui va faire par exemple, que le radis que vous avez mangé, c'est juste le croquant que vous aimez. Donc s'il a perdu ce croquant-là ?

*Mr:* [Euh] plus aucun intérêt à le manger.

*Mme:* Plus aucun intérêt à le manger.

*Mr:* Je peux manger du navet. Qui peut m'apporter à peu près la même chose. Mais que je trouve bon.

*Mme:* [Ouais]. [Ouais].

*Mme:* Donc, pour que, pour retrouver de l'intérêt de manger, il faut lui redonner [euh]--

*Mr:* [Ouais], c'est ça.

*Mme:* Son croquant--

*Mr:* Son croquant, [ouais]. C'est à peu près-- pour moi, le radis, c'est le seul truc qui est intéressant. C'est un légume, c'est que, c'est que c'est croquant [quoi].

*Mme:* Oui.

*Mr:* Et que c'est pas très parfumé.

*Mme:* Oui.

*Mr:* Donc, ça passe. En vrai ça arrive de--

*Mme:* [Ouais]

*Mr:* Tomber sur un bout de radis.

*Mme:* Ça se marie bien [du coup] avec [euh]--

*Mr:* [Ouais], c'est ça [ouais]. [Ouais], c'est chouette.

*Mme:* Ok.

*Mme:* Bon, [bah] très bien, merci beaucoup. On a terminé les questions.

*Mr:* Pas de problème.

*Mme:* Alors, je vais couper ça.

**Household F20, Interview 1**

00:00:02
*Madame 1:* Je le mets près de toi c'est plus intéressant que moi, alors pour commencer, je te propose un petit jeu de devinettes, c'est-à-dire je vais te demander de penser à un fruit ou un légume que tu aimes tout particulièrement. Tu as pensé à ce fruit ou à ce légume sans me révéler son identité. L'idée, c'est que je devines uniquement grâce à la description que tu vas en faire.

00:00:28
*Madame 2:* OK.

00:00:28
*Madame 1:* Alors, est-ce que tu as un fruit ou un légume en tête ?

00:00:33
*Madame 2:* [Euh] Oui.

00:00:35
*Madame 1:* Je t'écoute.

00:00:36
*Madame 2:* C'est un légume vert.

00:00:37
*Madame 1:* Ouais

00:00:38
*Madame 2:* Longs, frais, qui se mangent bien en salade en été, qui se mange froid et pas chaud, qui se mange entièrement.

00:00:48
*Madame 1:* Ok.

00:00:48
*Madame 2:* Il y'a rien qui est jeté. Donc, c'est un légume. Je sais pas si je l'ai précisé [Euh] souvent on le fait. Moi, je sais qu'on le fait souvent à la crème ou en salade. Avec un peu de vinaigre.

00:01:08
*Madame 1:* Ok. Qu'est-ce que t'aime particulièrement dans ce légume ?

00:01:13
*Madame 2:* La fraîcheur. Il est gorgé d'eau. [Euh] et le goût, mais je pense que le goût, c'est lié plus à la fraîcheur que le goût en lui-même. Je pense.

00:01:29
*Madame 1:* Ok. Tu sens la fraicheur, fin le goût et frais quoi.

00:01:34
*Madame 2:* Ouais.

00:01:34
*Madame 1:* Ok. En général on mange plutôt où fin quand c'est plutôt le matin, le midi, le soir, l'aprem ?

00:01:41
*Madame 2:* Le matin, fin qu'est-ce que je dis, le midi ou le soir.

00:01:44
*Madame 1:* D'accord.

00:01:49
*Madame 2:* Après moi des légumes comme ça, je peux en manger n'importe quand.

00:01:51
*Madame 2:* Donc [Euh].

00:01:54
*Madame 1:* Tu serais capable de grignoter, même si d'habitude, c'est plutôt un repas.

00:01:56
*Madame 2:* Oui, oui, oui.

00:01:57
*Madame 1:* Ok, en général, tu l'achète où ?

00:02:05
*Madame 2:* [Euh] en supermarché. Après, je commence depuis quelques mois, je commence à essayer de tout prendre au marché. Du coup, il y a besoin d'une période de transition, mais jusqu'à présent, du coup, c'est un supermarché.

00:02:22
*Madame 1:* Ok. Ça marche. Est-ce que tu peux me raconter les différentes étapes, s'il y a des étapes que tu fais avant de pouvoir le manger, tu as ton légumes, ce que tu dois faire jusqu'à pouvoir le consommer.

00:02:39
*Madame 2:* Alors je passe un coup sous l'eau.

00:02:40
*Madame 1:* OK.

00:02:43
*Madame 2:* Et après soit, soit je suis vraiment motivée ou pressée et je pars en croquant dedans directement comme ça.

00:02:50
*Madame 1:* OK.

00:02:50
*Madame 2:* Soit, je coupe en rondelles. Voilà c'est tout.

00:02:59
*Madame 1:* Ok, donc tu l'épluches pas.

00:02:59
*Madame 2:* Non.

00:03:04
*Madame 1:* Et tu manges même les extrémités du légume ou tu les enlèves ?

00:03:07
*Madame 2:* Ah oui nan c'est vrai, j'enlève les tout petits bouts, vraiment, ce qui est très dur à croquer. Le tout petit extrémité.

00:03:16
*Madame 1:* Ok. Tu laves, après tu enlèves un petit bout, soit, tu croque, soit tu coupes en rondelles, tu mets peut-être dans la crème, l'huile, etc.

00:03:29
*Madame 2:* Nature généralement.

00:03:29
*Madame 1:* De nature. Ok, en plus de la fraîcheur que tu m'as, que tu as, que tu as et que tu as décrit, est-ce que tu lui trouve d'autres, d'autres avantages à ce légume ?

00:03:45
*Madame 2:* Il hydrate parce qu'il est gorgé d'eau. Mais y a vraiment un effet de, bah tu bois en même temps, tu manges. C'est comme pour La Pastèque. Le principe.

00:04:00
*Madame 1:* Ok.

00:04:01
*Madame 2:* Et ce n'est pas salé, c'est pas sucré, ça reste assez neutre. Je trouve, c'est ça fait juste du bien, quoi. Et à n'importe quelle saison, ce soit l'hiver ou l'été.

00:04:18
*Madame 1:* Ok. Est-ce que tu saurais dire ce que tu ressens quand tu le manges ?

00:04:28
*Madame 2:* [Euh]. Je dirais, fin ouais, du bien être quoi.

00:04:35
*Madame 1:* Ouais.

00:04:40
*Madame 2:* C'est [Euh]. Mais je pense que ça, c'est le côté frais et hydratant vu que j'aime ça, j'aime ce légume. Je suis toujours contente d'en manger quoi. Donc je déguste. Surtout un frais.

00:04:58
*Madame 1:* Je vois. En général, tu le stocke où ?

00:05:04
*Madame 2:* Frigo.

00:05:04
*Madame 1:* Tu mets au frigo ?

00:05:05
*Madame 2:* Quand il est, quand j'ai commencé à le manger. Si je n'ai pas commencé à le manger, je les mets pas forcément dans le frigo. Ça dépend. Si je ne consomme pas assez vite, je peux le laisser comme ça. Et puis.

00:05:14
*Madame 1:* OK.

00:05:17
*Madame 1:* Et est-ce que tu trouves des inconvénients à ce légume ou non ?

00:05:24
*Madame 2:* Bof, ce n'est pas un inconvénient, peut-être dès fois, j'ai tendance à commencer à le manger. Il reste un morceau et un morceau qui est à l'air un peu, même si je protège, ça reste à l'air et du coup, ça s'assèche assez vite. Je suis obligée de couper un peu le bout et le jeter puisque c'est vraiment pas terrible avant de continuer à manger, quoi. Ça s'assèche assez vite.

00:05:50
*Madame 1:* Ok, donc c'est quand tu le coupes et tu manges pas tout.

00:05:54
*Madame 2:* Il reste un morceau quoi.

00:05:55
*Madame 1:* Le bout qui a pu sa peau, sèche et du coup, t'es obligée de l'enlever celui là pour commencer à le manger. Au final t'es obligée de jeter un petit bout. A

00:06:08
*Madame 2:* Je pense que je pourrais le manger, mais sûrement pas très bon. Oui, peut être.

00:06:11
*Madame 1:* Au niveau du goût que c'est pas.

00:06:24
*Madame 2:* Ouais c'est sec, c'est un peu rapeux, fin, pas terrible quoi.

00:06:29
*Madame 1:* Ok, le moment fatidique où je dois trouver, je dirais, le concombre.

00:06:31
*Madame 2:* Ouais, bravo.

00:06:33
*Madame 1:* Ok chouette.

00:06:38
*Madame 1:* Alors là, si on s'intéresse à tes pratiques d'achat et ton organisation en général. Vous êtes combien à vivre dans votre foyer ?

00:06:53
*Madame 2:* Deux.

00:06:54
*Madame 1:* Donc vous êtes deux. Et qui est amené à faire des courses alimentaires ?

00:06:57
*Madame 2:* Moi.

00:06:58
*Madame 1:* Uniquement toi ?

00:07:00
*Madame 2:* Ça dépend des dispo mais en général c'est moi, parce que je travaille plus le soir.

00:07:05
*Madame 1:* D'accord.

00:07:07
*Madame 2:* Je suis là en journée. En général, c'est plus facile, sinon, il fait aussi.

00:07:11
*Madame 1:* Oui, mais c'est plus toi qui.

00:07:14
*Madame 2:* Ouais.

00:07:14
*Madame 1:* Donc est-ce que tu peux me raconter comment tu t'organises ? C'est quoi ton sur tes courses alimentaires ? Comment tu t'organise ?

00:07:23
*Madame 2:* Alors là, coup ce que j'essaye de faire, c'est le dimanche. J'essaie d'aller au marché, c'est pas j'essaye, c'est tous les dimanches, je vais au marché le dimanche matin à Saint-Louis et j'essaye de faire le plein de légumes. Donc je ne vais pas calculer à savoir qu'est-ce que je prends pour la semaine juste, j'y vais et je prends le maximum de légumes, de toute façon je fais des poelès de légumes et tous. Donc je mets tout dans une casserole. Une salade, c'est rapide, mais j'essaie vraiment de faire le plein de légumes et de tenir le plus longtemps possible, juste avec le marché de Saint-Louis. Et des fois, ça m'arrive de devoir aller faire d'autres courses ou des temps parce que il me manque un truc. Ou alors un homme est passé il a tout mangé [Rires], j'ai plus rien et j'essaye d'arrêter à tout ce qui est supermarché d'aller ici plutôt.

00:08:12
*Madame 1:* OK.

00:08:12
*Madame 2:* Donc essayer de faire plus du local. On voit un peu les magasins qu'il y a comme des day by bay où les choses comme ça, c'est plus dans les légumes. Mais voilà, on apprend petit à petit à se gérer comme ça.

00:08:28
*Madame 1:* D'accord et tu disais sur le concombre que, j'ai du sopalin je vais me moucher.

00:08:46
*Madame 2:* En plus, il y a pas mal de plantes ici.

00:08:48
*Madame 2:* [Inaudible 00:08-50-00:09:03].

00:09:08
*Madame 1:* Du coup tu disais sur le concombre que, avant c'était plutôt au supermarché, mais que maintenant tu essayais le marché, sur combien de temps t'as pris se plie d'aller toutes les semaines au marché ?

00:09:18
*Madame 2:* Ça fait un peu moins d'un an qu'on est ici. On se pose des questions depuis un an. Du coup, je pense ça doit faire 6 mois qu'on a vraiment, que je, que je vais vraiment toutes les semaines au marché. Avant, j'allais une semaine sur deux, une semaine sur 3, mais là, c'est vraiment. Je me pose vraiment des questions. Avant, j'y allais juste pour voir, mais je savais que j'allais faire mes courses au supermarché la semaine, donc, alors que maintenant, je me dis tant qu'à y aller le dimanche, autant que je fasse vraiment le maximum au marché. Et après, si vraiment j'ai un besoin j'ai la semaine pour aller ailleurs.

00:09:49
*Madame 1:* OK.

00:09:49
*Madame 2:* Mais ça a changé un peu, mais depuis 6 mois, on va dire.

00:09:53
*Madame 1:* Et qu'est-ce qui a fait que tu as voulu plutot aller au marché qu'au supermarché ?

00:09:57
*Madame 2:* Parce que ça fait un an, du coup, depuis qu'on est arrivé ici, on a commencé à apprendre, à cultiver, à jardiner tout ce truc là. Donc on commence à se poser des questions sur notre consommation.

00:10:07
*Madame 1:* Ouais.

00:10:07
*Madame 2:* Voir un peu d'où ça vient? On regarde des documentaires qui font un peu peur. [Rires] On fait des choses comme ça ses trucs. Je viens du monde paysan de base de la campagne et je me suis rendu compte que j'ai grandi le nez dans le glyphosate. Ça fait un peu peur. Je me dis il faut quand même, mais jamais trop tard. Maintenant, faut vraiment qu'on prenne de la distance avec ça et qu'on essaye de prendre un maximum de bio, de cultiver nous-mêmes. Mais là, pour l'instant, on apprend à cultiver donc on est pas autonome. Mais voilà, c'est l'entre deux entre supermarchés et l'autonomie. On va au marché.

00:10:47
*Madame 1:* Et qu'est-ce que vous cultivez ici ? Pour l'instant, du coup, si c'est qu'au début, est-ce que vous avez quand même quelques ?

00:10:56
*Madame 2:* Ouais, là on on ramasse fraises, radis, salades, épinards. Toutes les plantes aromatiques. Dès qu'on en voit des nouvelles, on essaye. On a betteraves, poireaux, petits pois. Comment ça s'appelle haricots verts. On a pris des topinambours, patates douces, patates, enfaite tous les plans. On a essayé, je crois. Là, on a tout à peu près. On a citronniers, orangers, vignes, melons, pastèques, je sais même plus ce qu'on a mais en gros tout ce qu'on peut trouver, on a des tournesols pour les graines.

00:11:40
*Madame 1:* Donc, c'est le début, ces, on est sur un beau potager là.

00:11:43
*Madame 2:* Ouais [Rires]. Et ça pousse. En plus, on en arrive à faire pousser. On a. Du coup, on a créé un compost en bois de Palette, un composteur. Et on essaye vraiment de.

00:11:57
*Madame 1:* Ok, et par exemple, si on prend le poireau, vous arrivez à être autonome au moins sur le poireau ou non. Faut quand même de temps en temps que vous allez rajouter du poireau en plus.

00:12:06
*Madame 2:* On est plutôt autonome en vrai. En un an, on a dû prendre une fois du poireau, une fois. Et encore, parce que nous, les poireaux, on les laisse en terre et en fait, on mange que les feuilles comme ça, on les ramasse pas en fait et pousse tout le temps. On coupe les feuilles du haut et ça, c'est comme du poireau, au final, on n'a pas besoin de récolter.

00:12:23
*Madame 1:* Donc, c'est le verre que vous avez, vous coupez que vous mangez, oK et le blanc reste en terre et vous laissez repousser à chaque fois.

00:12:32
*Madame 2:* C'est ça, et dès qu'on va commencer à monter en fleurs. Là, du coup, on prend le blanc, on mange, on attend vraiment le dernier au dernier moment et l'année dernière, on a fait, on a fait, alors on les a planté en juillet et on les a récolté en février cette année.

00:12:48
*Madame 1:* OK.

00:12:49
*Madame 2:* Donc vraiment, on a attendu le dernier moment et on a fait en manger tout le long.

00:12:56
*Madame 1:* Et du coup, comment vous le mangez le verre de poireau comme ça qu'est-ce que tu prépares?

00:13:02
*Madame 2:* Pendant l'hiver, on les mettait dans les soupes comme du poireau normal. Et sinon, soit on faisait ça soit, je mets à la poêle revenir un peu à la poêle puisqu'on mange les racines aussi quand on essaie de manger tout le poireau.

00:13:14
*Madame 1:* OK.

00:13:15
*Madame 2:* Donc, on fait revenir à la poêle avec des légumes qu'on a et et sinon, j'ai testé en salade crues comme ça, et ça passe bien aussi. Donc après, on en met pas beaucoup parce que je pense pas que ce soit terrible pour la digestion quand même cru, mais ça passe bien.

00:13:32
*Madame 1:* D'accord. Donc il y a quand même la même volonté d'épuiser le poireau de tout.

00:13:42
*Madame 2:* Ouais.

00:13:44
*Madame 1:* Du coup, pour un plan de poireaux, d'aller jusqu'au bout.

00:13:50
*Madame 2:* Et après, il y en a certains qu'on les laisse monter en graine, comme ça on récupère les graines, pour essayer de refaire un sommet, mais pas là. On est pas trop encore arrivés à là. On apprend justement à gérer les graines, pour les récolter et tout. J'essaye avec du radis noir. J'ai récupéré les graines de radis noir aussi.

00:14:07
*Madame 1:* Oui.

00:14:07
*Madame 2:* J'essaye de faire sécher là. Et voir comment ça fonctionne.

00:14:11
*Madame 1:* Ok, ça marche. Et si on retourne au marché, t'essaye de faire un bon plein de légumes quand tu y vas. Est-ce que tu achètes là-bas aussi des fruits ou pas ?

00:14:31
*Madame 2:* [Euh] Les fruits, on en mange pas beaucoup. Donc, dès que j'en achète c'est là-bas, sauf cas exceptionnels. Si on me demande un truc au dernier moment et que j'ai besoin. Je vais au magasin, mais sinon, sinon, on mange les fraises qu'on a énormément de fraises, ces temps-ci. On ne prends pas de légumes, fin de fruits parce que cela soit on prend au marché quand vraiment y a une envie, mais sinon on est plus légume que le fruit, donc [euh].

00:14:59
*Madame 1:* Ok. Alors du coup, que ce soit les fruits et légumes, mais plus les légumes du coup. Quand tu es au marché, est-ce que tu sais, ce que tu vas prendre à l'avance ou pas avant ?

00:15:09
*Madame 2:* Non, je vais. En fait, j'ai repéré un peu les paysans ou les revendeurs, pas forcément bio, mais en tout cas qui revendent ce qui a été fait ici.

00:15:21
*Madame 1:* Ouais.

00:15:22
*Madame 2:* Du coup, je vais les voir et je fais en fonction de ce qu'ils ont.

00:15:26
*Madame 1:* Ok.

00:15:26
*Madame 2:* Et des fois, je prends rien du tout parce que ça n'inspire pas trop que tout ce qui est oignon et tout en a énormément toute l'année ici.

00:15:32
*Madame 1:* Vous cultivez aussi les oignons ?

00:15:33
*Madame 2:* Oui, ouais, ouais, j'ai oublié de dire. Mais là encore, les oignons on fait toute l'année ce qu'on mange que les feuilles aussi, on prend que le vert qui sort.

00:15:43
*Madame 1:* OK, c'est les oignons en, en tige.

00:15:47
*Madame 2:* Ouais, normalement, c'est censé être des oignons blancs, mais en fait, ils font des tiges, du coup on mange les tiges.

00:15:55
*Madame 1:* OK, ça marche. Donc finalement, quand tu es au marché, tu vas voir les producteurs locaux voir bio, en plus, tu regardes ce qu'ils ont et selon ce que tu as toi chez toi, tu prends, d'accord. Et, est-ce que est-ce que tu cultive l'aubergine ?

00:16:19
*Madame 2:* Oui, donc quand; on essaye,

00:16:23
*Madame 1:* Mais ça commence là ou pas ?

00:16:26
*Madame 2:* On a une fleur.

00:16:26
*Madame 1:* Ok.

00:16:26
*Madame 1:* Et voilà, quand tu es au marché, tu veux des aubergines. Comment tu gères tes quantités ? T'achète comment ?

00:16:37
*Madame 2:* Une aubergine, par exemple. Si je veux des aubergines, je pense que l'aubergine, je prendrais qu'une, des tomates, par exemple, vu que c'est plus petit et que ça, ça se mange plus facilement que l'aubergine. Je prendrais 4 5, peut être je n'en prend pas énormément non plus parce que je sais que ça tient pas trop la tomate, par exemple. Mais après, sur les tomates, les tomates qui commencent à être un peu abîmée et vieille. On sent que ça commence à, il faut les manger vite. En général, on les fait revenir à la poêle pour une petite sauce tomate et du coup, ça se passe bien mieux qu'en salade ne sont pas terribles.

00:17:15
*Madame 1:* D'accord. Quand un produit est abîmé, tu as tendance plus à le cuir que.

00:17:22
*Madame 2:* Ouais. Et pour tout ce qui est par exemple carottes ou patates qui se conserve bien.

00:17:28
*Madame 1:* Ouais.

00:17:28
*Madame 2:* Mais là, j'ai tendance à prendre quand même pas mal de quantité puisque je sais que j'en prends rarement.

00:17:33
*Madame 1:* Donc ça, tu ne fais pas pousser encore ?

00:17:38
*Madame 2:* Si si des carottes j'en ai pleins.

00:17:40
*Madame 2:* Ah Ouais ?

00:17:40
*Madame 2:* Ouais, ouais. Et quand elles sont belles, là, on est content. Les patates aussi. Les patates, il y en a plein.

00:17:46
*Madame 1:* Ah ouais, donc, finalement, on n'achète pas tant que ça.

00:17:48
*Madame 2:* Mais en fait, c'est que les patates, c'est la première année qu'on le fait, on vient de les planter jusqu'à présent, on achetait quoi. Je pense que d'ici fin du monde on va pouvoir récolter. Je pense que c'est déjà très haut et et là, du coup, on n'achètera pas pendant un moment. Donc on aura pleins. Mais c'est ça aussi. Nous, on fait pas trop sous serre, on n'a que des, du piment, poivron et tomates sous serre.

00:18:12
*Madame 1:* Ok.

00:18:13
*Madame 2:* Du coup, à part ça qui peut venir tôt et encore, on ne plante pas très tôt. Du coup, bah, c'est vrai, il faut attendre assez longtemps pour avoir les fruits et légumes, donc.

00:18:25
*Madame 1:* Ça marche. Et donc, qu'est-ce qui fait que tu as envie de cultiver des légumes plutôt que de les acheter ? C'est quoi qui te motive ?

00:18:40
*Madame 2:* Parce que moi, j'aime bien faire. Je travaillais en jardinerie, mais ça, ça m'a permis d'aimer les plantes, en fait, parce qu'avant, je ne comprenais pas trop pourquoi les gens aiment ça. La plupart des gens, je comprenais pas trop l'intérêt. Et en fait, voir la vie, voir pousser, pour recommencer le cycle à chaque fois, voir que ça va abriter plein de bestioles. Là, je fais tout pour un maximum d'abeilles. Là, j'ai mis plein de fleurs spéciales pour les abeilles, donc on en a de plus en plus bourdons, abeilles, papillons. La là, ça commence à revenir et on essaie de créer une biodiversité comme ça et voir si ça, ça va permettre de réguler les fleurs de limaces. Les choses comme ça, c'est de tester. Par exemple, on a des gros problèmes de limaces au niveau des salades et des choux. On avait des choux aussi, mais du coup, ils ont été ravagés. Et du coup, là, j'ai commandé des coccinelles, des petits larves de ver, en fait rajouter des prédateurs. On essaye de créer comme ça un équilibre en fonction des problèmes qu'on peut avoir. On apprend petit à petit.

00:19:44
*Madame 1:* Bah oui oui, avec la biodiversité, c'est aussi. Donc, c'est par, finalement, tu cultive, c'est par amour de voir de la nature qui est là où il y a même autre chose derrière, ou c'est vraiment l'idée de créer quelque chose de naturel ?

00:20:06
*Madame 2:* Le début, c'était vraiment l'idée de créer quelque chose de naturel. Moi, j'aime la nature de base, donc il y avait l'idée de pouvoir donner un coup de main pour vraiment créer quelque chose de, qui aille tout seul après, qui se fasse tout seul.

00:20:22
*Madame 1:* Ouais.

00:20:22
*Madame 2:* Et après, on a commencé à se poser des questions. On a commencé à creuser un peu et quand je vois l'état actuel des choses, le fait qu'on mange que de la merde, désolée pour le terme, que je trouve ça assez violent. En fait, ce qu'on ingère et comment est-ce qu'on vit pour avoir tous les produits qu'il y a partout, tout le temps, même quand tu penses que c'est bio, même quand tu penses que c'est sain, en fait, ce ne l'est pas, l'eau qu'on boit n'est pas saine. On arrive à un stade où c'est tellement mauvais que je ne sais pas comment on va faire pour les futures générations, c'est vraiment une question qu'on se pose. Et après, on se dit, quand quand j'aurai des enfants. Je n'ai pas envie de leur donner ça non plus. Le fait d'être conscient de ça, ça me pose un problème éthique. Je me dis au moins essayer d'être autonome comme ça si jamais il arrive quoi que ce soit, si jamais la société s'effondre ou pas, qu'il y a quoi que ce soit. Nous, on aura au moins tendue vers l'autonomie, donc ce sera peut être moins violent pour nous et on pourra proposer quelque chose de meilleur aux autres après à voir s'ils veulent ou pas. Mais en tout cas, on aura quelque chose à proposer quoi. Et puis là, on est là, on est jeune, on a la chance d'avoir un terrain qui n'est pas à nous, qui était en friche, qui était en friche, en face donc du coup on a fait tant pis si on connait pas le propriétaire. Il aura une surprise quand il reviendra. Il n'est pas revenu, je crois toujours pas. [Rires]. Voilà, tant qu'à faire, on s'est dit de toute façon il va pas râler, parce qu'on l'a entretenu son terrain, il va rien me dire.

00:21:53
*Madame 1:* Ok, ça marche.

00:21:59
*Madame 2:* C'est vraiment. Il y a plein de choses, qui poussent à tendre vers quelque chose de plus naturel. Et du coup, créer un potager, quoi. Et même savoir ce qu'on mange peu, on s'est rendu compte d'un truc aussi, ce qui est fou, c'est quand on mange quelque chose qu'on a produit et bah on le mange moins rapidement.

00:22:17
*Madame 1:* D'accord.

00:22:20
*Madame 2:* On a l'impression qu'on consomme plus quand il suffit juste d'aller acheter le produit et le consommer, etc. On consomme plus que quand on fait avec ce qu'on a dans le jardin, presque instinctivement, on va essayer de limiter, de faire attention au dosage. De, en fait, on fera plus attention que quand on va faire nos courses. On a tout à profusion parce que c'est ça qu'on se dit. Ah bah voilà, c'est pas grave si je mange toutes les pommes, ce que je peux en rechercher demain. Bah là, c'est pas ça. Donc, en fait, on est obligé de faire attention.

00:22:49
*Madame 1:* Ok.

00:22:52
*Madame 2:* Il y avait ça aussi.

00:22:53
*Madame 1:* Tu as trouvé une différence dans la façon de consommer les produits par rapport à quand tu l'achète en supermarché qu'au marché et que dans ton jardin ?

00:23:05
*Madame 2:* Bah déjà. Je pense que c'est psychologique. Je pense pas que ce soit un vrai, mais en tout cas j'ai l'impression qu'ils sont meilleurs, mais je pense, c'est parce qu'on a vraiment sué pour les avoi. Du coup, en fait, on déguste vraiment le produit, mais là, c'est fou, il a du goût et tout. [Rires] Je pense que ça change rien par rapport au marché. Et on en profite plus des fruits.

00:23:31
*Madame 1:* Ok. Tu dégustes plus, t'apprécies plus et peut-être une meilleure qualité aux produits que tu as fait toi, que celui que tu as acheté ?

00:23:45
*Madame 2:* En général ouais.

00:23:47
*Madame 1:* Et tu penses que c'est proportionnel par rapport à plus, c'est plus t'es dans une pratique, un peu de ton jardin, puis le marché, puis le supermarché, parce qu'il y a une proportion à dire que, tu sais genre, le marché par rapport aux supermarchés tu as l'impression que ce sera peut être aussi une meilleure qualité ?

00:24:06
*Madame 2:* Je pense, c'est même pas je pense que déjà, dans leur rapport à l'autre, c'est quand on va au marché. J'ai fait la foire bio, par exemple, ou les marchés un peu comme ça qui soit, ou parce que moi, ce que je veux dire, c'est les marchés. Il y a des gros revendeurs qui vendent de l'ananas en hiver, ça, je vois pas l'intérêt. Pour moi, c'est exactement la même chose que le magasin pour le coup. Par contre, le le mec qui vend parce que là je pense, où je vais souvent, mais qui va en fonction de la saison et des fois en hiver, il n'a que 3 légumes. Il a du poireau et des patates. Et ce qu'il peut avoir quoi, du chou. Là, du coup, je vais vraiment plus profité aussi du légume, parce que je vais me dire il arrive à faire pousser ça et moi, je n'arrive pas [Rires]. Je sais pas comment il fait.

00:24:59
*Madame 1:* Donc, il y a ce rapport aussi, du coup avec le travail de la Terre qui peut être fait par le producteur.

00:25:10
*Madame 2:* C'est quelqu'un qui connaîtra toujours mieux, je pense, ce légume que des personnes qui vont juste acheter à l'entrepôt quoi. Par exemple, l'autre jour où je me suis dit ça fait presque un an que je lui achète des légumes et j'ai jamais demandé si c'était bio en fait, parce que ça me paraissait évident. Et en fait, c'est pas évident parce t'en a plus qui ne font pas bio, mais qui font eux-mêmes, mais qui ne font pas du biodu coup je suis allée le voir et je lui ai demandé mais il m'a expliqué qu'il faisait tout bio et que c'étaient des fermes qui avaient autour de chez lui et lui il revendait les produits des différentes fermes et que tout était bio, sauf, sauf les oignons et les échalotes, je crois où il mettait des produits dessus. Donc, il m'avait dit si vous voulez du bio, ne prenez pas ces produits là par contre vous pouvez prendre, ceux-là mais pas ceux-là. Donc, c'est ça que je trouve intéressant aussi. C'est qu'il est capable de dire il n'est pas là. Dire si tout est bio, c'est bon. Allez y, servez vous. Alors qu'au final, c'est pas le cas.

00:26:12
*Madame 1:* Et ce revendeur là c'est un que tu vois au marché de Saint-Louis ?

00:26:13
*Madame 2:* Oui. Mais même si j'ai eu une petite déception quand il a dit non, je ne produit pas les légumes, c'est mes voisins, encore un revendeur. Mais bon après on voit que c'est fait. Mais déjà, c'est ce que je disais à mon copain parce qu'il avait du mal au début. Mais je disais quand tu vas avoir les légumes, que tu vois qu'il y a la terre dessus qui ne sont même pas lavés. Ils viennent du champs, des patates, t'as intérêt de pas être très propres quand tu les prends, parce que c'est ça que je trouve bien. Moi, ce que j'aime en fait, c'est de pouvoir prendre les choses, de voir que c'est vrai, c'est bête.

00:26:54
*Madame 1:* Ok et donc, si on essaye de dresser une liste de, des fruits et légumes que tu as l'habitude de consommer, donc toi ou ton copain, que votre foyer consomme, tous les légumes que vous cultivez qui sont assez nombreux.

00:27:22
*Madame 2:* Il y en a qu'on a jamais mangé et qu'on a dans le jardin. [Rire].

00:27:25
*Madame 1:* C'est lesquels, par exemple ?

00:27:27
*Madame 2:* Topinambour, ça on a jamais mangé, les baies de goji. Moi, j'en ai jamais trop mangé non plus. Et on a un plan. [Euh], comment c'est, Les physalis. Moi, j'en mange pas trop. Lui, il adore ça. C'est son fruit préféré. Mais moi, je mange pas trop ça.

00:27:43
*Madame 1:* Je sais même pas ce que.

00:27:44
*Madame 2:* C'est les coquelet du Pérou. Il y a plein de monde, c'est comme des petites cerises. Il y a une sorte de fleurs séchées. Et c'est jaune comme une tomate cerise jaune. Mais et ça, ça pousse très bien, en fait, il y a des légumes qui poussent fruits et légumes qui poussent très, très bien ici, et pourtant on en trouve pas trop, et lui il adore ça, du coup on en a. Qu'est-ce qu'on peut avoir que je mange pas trop, de la patate douce. J'en mange quasiment jamais non plus de manger une ou deux fois tout.

00:28:13
*Madame 1:* OK.

00:28:15
*Madame 2:* Je ne sais plus trop. Après d'un principe de salade, je mange pas trop non plus.

00:28:20
*Madame 1:* Et la patate douce, pourquoi tu en consomme très rarement, voire jamais ?

00:28:25
*Madame 2:* Parce que c'est pas ce qui me vient à l'esprit quand je cuisine, j'ai l'habitude de cuisiner avec des pommes de terre. Et encore, je cuisine pas beaucoup de pommes de terre, mais quand je fais, c'est des pommes de terre. Donc j'aime pas me poser la question de savoir si je suis à la patate douce. C'est tout ce que je pense, c'est une habitude.

00:28:40
*Madame 1:* Ce n'est pas le goût, le goût, à la base tu pourrais aimer.

00:28:43
*Madame 2:* C'est ça. Il y a un truc dont j'ai horreur, c'est quoi? Ah oui, la coriandre.

00:28:50
*Madame 1:* Ah oui, moi aussi.

00:28:53
*Madame 2:* Ça sent fort j'en peux plus. Et on a mis, les voisins, ils n'arrivent pas à faire pousser, mais on a mis un plan et ça nous a fait un truc sur 3 mètres. Il est immense. C'est le truc tout en fleurs qu'on a et les gens en raffole. Du coup, ils me demandent tout le temps des bouquets et je coupe les bouquets et j'en ai plein partout. Ça pue, ça, ça m'insupporte.

00:29:14
*Madame 1:* Il parait qu'il y a un gène qui fait que sur la coriandre, je fais une aparté. Mais, j'ai été six mois en Inde, il en est absolument partout. Et il paraît qu'il y a un gène qui fait que les Perses, il y en a qui peuvent adorer ça et celles qui n'aiment pas. Il y a comme un goût de savon qui est très, très dérangeant. Il se goûte, j'ai l'impression que c'est chimique, c'est du vraiment ultra fin. Ils en mettent un grain, je sais qu'il y en a et c'est répulsif. Il y a un gène qui fait que.

00:29:47
*Madame 2:* C'est fou, ça.

00:29:48
*Madame 1:* Ça déclenche cette réaction chimique notre. Donc, il y a la team pro coriandre et la team anti coriandre. Et combien de fois je commande indien et je dis sans coriandre. Mais je vois mon plat direct je me dis ok tant pis je ne pourrais pas le manger, c'est terrible.

00:30:10
*Madame 2:* Donc je ne te propose pas un bouquet de coriandre.

00:30:13
*Madame 1:* Non mercie. Donc la patate douce après la patate douce tu a dis autre chose.

00:30:22
*Madame 2:* J'avais dit topinambour, physalys.

00:30:25
*Madame 1:* Ouais. Et le topinambour, pourquoi tu n'en consomme pas ?

00:30:30
*Madame 2:* Parce qu'on m'a jamais donné ça à manger et qu'encore une fois, je ne sais même pas, j'ai aucune idée de comment cuisiner. Je sais même pas.

00:30:40
*Madame 1:* Si tu en as dans le jardin comme tu vas appréhender les choses ?

00:30:44
*Madame 2:* Bah il y a Internet. Google.

00:30:48
*Madame 1:* D'accord.

00:30:48
*Madame 2:* Pas trop. En fait, c'est juste pour les topinambours, par exemple, on était un magasin vert et du coup, on regarde, on fait ah mais tient je connais pas, ça se plante. Du coup, on va tester voilà c'est tout. Ça se passe comme ça en général.

00:31:01
*Madame 1:* OK, mais au final, ça marche. Vous en avez ?

00:31:05
*Madame 2:* C'est trop tôt. Encore encore des plans. Il est comme ça, super beau déjà.

00:31:09
*Madame 1:* Vous en aurez probablement. Et à ce moment là, tu regardes ce que tu peux en faire à ce moment là.

00:31:15
*Madame 2:* Ouais. Il y avait de la citronnelle aussi qu'on a essayé, mais je trouve ça un peu minable aussi. [Rires] J'adore le citron et la citronnelle et c'est dans les plats aussi. Il y en a beaucoup aussi chinois, qui en mettent beaucoup. Je sais, en goûtant qu'il y a de la citronnelle.

00:31:38
*Madame 1:* Je sais pas s'il y a un gène pour celui là.

00:31:41
*Madame 2:* Et ça, par contre, c'est pire que la coriandre.

00:31:46
*Madame 1:* Et du coup, tu vas en faire quoi de la citronnelle ? Que tu as ici que tu plantes ?

00:31:51
*Madame 2:* Là, on en avait un peu, on en a plus [Prénom d'un membre de la famille], il mange je lui ai dit tu te débrouilles. Alors là ou alors je donne aux gens, c'est comme la coriandre, je leur dit servez vous, même été jusqu'à déterrer des morceaux parce que ça prend. En fait, il est trop motivé, c'est ça.

00:32:15
*Madame 1:* Est-ce que vous avez des courges ou pas ?

00:32:18
*Madame 2:* Oui.

00:32:18
*Madame 1:* D'accord. Vous en consommez ?

00:32:22
*Madame 2:* Oui, mais pas trop de ça non plus, ce qui est potiron, courge, c'est que en soupe en hiver c'est tout. Le concombre et tout aussi.

00:32:32
*Madame 1:* Est-ce que vous avez des courgettes ?

00:32:35
*Madame 2:* Ah oui, le problème, c'est qu'on a mélangé tous nos plans. Je crois qu'on a des courgettes, mais ça se ressemble et ça ressemble tout cela. Donc on sait plus qu'on a mis et on sait plus ceux qui sont morts. Donc on a peut être plus de courgette mais normalement on en a.

00:32:48
*Madame 1:* D'accord. Et du coup, la par exemple la courgette que, tu sais pas si tu vas en avoir ou pas, c'est un légume que vous aimez ?

00:32:56
*Madame 2:* Oui, moi, j'en mange beaucoup, beaucoup et j'ai mes grands parents qui qui ont un potager aussi. Ils ont beaucoup de concombres et courgettes, donc tous les ans, je fais la razzia un peu de courgette. En été, en général, c'est ce qu'on mange. Je sais que je me retrouve tout le temps avec des kilos de courgettes et du coup on fait que ça.

00:33:13
*Madame 1:* Et donc, OK, t'en manges pas mal. Et au final, à part, la patate douce en légumes que tu consomme très rarement, la topinambour, mais tu vas la regarder après. Est-ce que c'est tout? Il y a que la patate douce qui te vient à l'idée que tu consomme très rarement, voire jamais.

00:33:31
*Madame 2:* Après, il y en a sûrement beaucoup d'autres, mais ça dépend des saisons. Tout ce qui est citrouille, une courge tous les trucs comme ça, ça en plus en hiver en plus pour les soupes.

00:33:45
*Madame 1:* Et l'artichaut ?

00:33:47
*Madame 2:* L'artichaut on en a aussi.

00:33:49
*Madame 1:* Ok.

00:33:50
*Madame 2:* Et moi, c'est vrai que je ne suis pas très fan, mais je peux en manger. Mais [Prénom d'un membre de la famille] adore ça. Du coup, je lui donne des artichauts. En général, je ne sacrifie pour lui donner l'artichaut.

00:34:05
*Madame 1:* Ok. Ça marche et sur les fruits, tu as dit que vous n'êtes pas des gros consommateurs de fruits ?

00:34:11
*Madame 2:* Non, mais là, du coup, on a un cerisier, on a myrtille, mûre, framboise, fraise, pommier. Rien à voir avec citron orange. Après, je connais pas les noms, c'est tous les trucs chelou. On mélange citron orange et tout. C'est là qu'on mange un peu. Ça, c'est bizarre. Donc faut que je trouve, je pourrais faire de la confiture avec. Ça se passe bien.

00:34:37
*Madame 1:* OK.

00:34:40
*Madame 2:* Il doit e, avoir d'autres je sais plus.

00:34:42
*Madame 1:* Et sinon, toi, quand tu es au marché, est-ce que tu en achète d'autres ou pas ?

00:34:49
*Madame 2:* Non, non.

00:34:49
*Madame 1:* T'achètes jamais de fruit ?

00:34:49
*Madame 2:* Non, à part dès fois si vraient j'ai un coup de coeur pour des fraises avant que j'avais, avant que les fraises soit bonne ici, on en prenait un peu au début. Et sinon, une fois, j'ai pris des myrtilles peut être.

00:34:59
*Madame 1:* Ok. Et donc. Qu'est-ce qui fait tu n'achètes pas de fruits autres que ceux que tu produits ou de temps en temps, quelques fraises myrtilles au marché ?

00:35:15
*Madame 2:* Peut être parce que ce n'est pas un besoin. Je ressens pasle besoin de manger des fruits, même si j'adore les bananes, par exemple, ou j'aime bien l'abricot de temps en temps. Mais c'est pas un besoin, en fait, je préfère me faire prendre un yaourt frais, par exemple.

00:35:31
*Madame 1:* D'accord.

00:35:35
*Madame 2:* Et ouais. Je pense juste ça si on était vraiment fan de ça, on aurait eu beaucoup plus de fruits ici. Et se servir dans un second temps, c'était vraiment les légumes, le premier truc qu'on voulait mettre.

00:35:45
*Madame 1:* D'accord. Donc, au final, on trouvera rarement des fruits dans ton panier au marché.

00:35:53
*Madame 2:* Ouais, très rarement.

00:35:55
*Madame 1:* Donc, ce n'est pas un problème de goût. Ce n'est pas non plus.

00:35:59
*Madame 2:* Oui c'est ça.

00:36:00
*Madame 1:* C'est pas un plaisir immense de les consommer.

00:36:01
*Madame 2:* Si vraiment j'ai une envie un jour, tout ce que je fais, je me suis réveillé en me disant je veux ce fruit, j'irais le prendre mais c'est rare ça n'arrive pas souvent.

00:36:17
*Madame 1:* Ça marche. Alors là, si on s'intéresse à des lieux de stockage, quand tu viens du marché, qu'est-ce que tu fais de tes fruits, non de tes légumes ?

00:36:33
*Madame 2:* Les légumes, je vais les séparer en deux en général.

00:36:36
*Madame 1:* OK.

00:36:37
*Madame 2:* Donc il y a des légumes que je sais qu'ils tiennent bien à l'extérieur. Par exemple, les carottes. Ça je mets dans les bacs là-bas. Et sinon, les légumes du genre tomates si je sens qu'elle commence à être un peu fatiguée ou les fruits où je me dit va falloir aller manger assez vite. J'ai tendance à les mettre dans le bac du frigo ceux-là.

00:36:57
*Madame 1:* Ok.

00:36:58
*Madame 2:* Je sais pas si c'est une bonne chose, mais je fais ça assez instinctivement. J'ai jamais trop regardé.

00:37:06
*Madame 1:* C'est ma question d'après, est-ce que ça te semble être un stockage idéal ou pas vraiment. Est-ce que ?

00:37:11
*Madame 2:* J'en sais rien, juste je me dis, le froid ralentit un peu, le fait de pourrir vite quoi. Sauf les radis. Un truc, les radis. Ça, c'est la mort. J'arrive pas à les garder à l'extérieur. Du coup, je suis obligé de les mettre dans le frigo. Parce que sinon, il perd toute leur eau. Et du coup, ils sont à moitié morte, tout de suite.

00:37:36
*Madame 1:* Donc les radis à température ambiante, c'est impossible. Donc, ils vont tous au frigo.

00:37:43
*Madame 2:* Et avec enroulés dans un ce qu'on a du sopalin moitié coton bizarre que qu'on peut laver, du coup on les emballe dedans et on pose comme ça. Et même que même sans ça ils tiennt pas dans le frigo non plus. Du coup, on essaye. Et là, ça fonctionne bien. Je pense que ça doit plus ou moins garder l'humidité un peu autour. Et c'est ça qui.

00:38:10
*Madame 1:* Ok, du coup à température ambiante, on retrouve les carottes. Quoi d'autre ? Le concombre, quand tu, tu sais que tu vas manger bientôt.

00:38:21
*Madame 2:* Quand on a des pommes, on laisse les pommes là. Tout ce qui est poivrons, carottes, oignons, échalotes, patates, courgettes. En général c'est ce qu'on a ici, je crois, les champignons, ça dépend de mon humeur. [Rires]

00:38:40
*Madame 2:* Des fois, je sais pas pourquoi je les mets tout de suite dans le frigo, des fois dehors. Je ne sais pas trop.

00:38:46
*Madame 1:* Je comprends.

00:38:54
*Madame 2:* J'ai pas beaucoup de légumes comme ça. J'ai souvent ces légumes là.

00:38:59
*Madame 1:* Et donc, par rapport à ce que tu cultive ? Comment tu fais quand tout est arrivé à maturité ? Est-ce que tu laisses quand même en terre ? Est-ce que tu prélève pas mal de choses, comment tu fais ?

00:39:12
*Madame 2:* Alors j'essaye de laisser en terre au maximum, de toute façon on est dans 15 mètre carrés, donc je peux pas faire autrement, par exemple, les fraises. Je sais qu'on n'a que six plants de fraises et c'est énorme la quantité qu'on a tous les jours. Du coup, à chaque fois, j'essaye de ramasser, d'anticiper et donc de ramasser celles qui ne sont pas encore bien mûres. Donc déjà, ça évite que les bestioles mange avant nous. Et après, ça permet de manger au fur et à mesure aussi, et de ne pas avoir des grosses quantités. Dès qu'il fait beau en un jour, qu'on arrive à réguler comme ça. Et puis après, quand on voit qu'il y en a trop, qu'on les mangera pas, on laisse le parti encore accroché dans les plans et on régule un peu comme ça. Et si jamais il y en a trop, je fais une confiture ou un gâteau. Et puis comme ça, ça part pas à la poubelle.

00:40:03
*Madame 1:* Ok, donc finalement comme lieu de stockage tu as le jardin, mais on ne va pas dire, ça reste en terre. Tu as une espèce de panier à légumes avec une température ambiante ou dans lequel tu peux mettre des fruits ou des pommes. Et sinon, c'est dans le bac à légumes que tu va mettre des légumes que tu trouveras peut être un peu plus fragiles et des fruits si les fraises, parce que tu les mets du feu au frigo ou pas du tout ?

00:40:32
*Madame 2:* Non, les fraises sont là dans l'assiette qui est juste là.

00:40:35
*Madame 1:* OK, donc, il n'y a pas. Tu ne mets pas de fruits au frigo ?

00:40:39
*Madame 2:* Non, je ne crois pas.

00:40:44
*Madame 2:* OK, d'ailleurs, j'ai tué mes fraises hier, je les ai laissés au soleil et du coup, elles sont mortes.

00:40:53
*Madame 2:* J'essaie de les manger, mais je réfléchis à comment je peux les faire. Le Soleil a du coup ce que j'ai appris. Il ne faut pas les laisser au soleil.

00:41:04
*Madame 1:* Les fraises n'aiment pas.

00:41:05
*Madame 2:* Quand c'est sur le plan oui, quand c'est plus à l'aise, j'ai l'impression que c'est comme si tu la met sur une poêle, que tu laisses comme ça, mais c'est pas terrible.

00:41:20
*Madame 1:* Ok, en termes de nettoyage des lieux de stockage donc t'en as deux ?

00:41:26
*Madame 2:* Ouais.

00:41:27
*Madame 1:* Comment est-ce que c'est systématique, t'as une organisation? Est-ce que c'est au feeling ?

00:41:33
*Madame 2:* C'est vraiment au feeling et je fais pas souvent, je pense pas à faire. On est toujours un peu pressé quand on vient ici. Donc quand je vois qu'il y a des légumes qui ont commencé à pourrir ou vraiment pas être bien, je nettoie en mètre. Mais sinon, il n'y a pas eu de problème et que tout est normal. Je pense pas forcément nettoyer. Et le bac du frigo, c'est un peu pareil. En fait, quand il y a eu un truc qui a coulé comme ça, je nettoie tout de suite. Et sinon, je pense pas forcément à faire si je vois pas que c'est ce qui.

00:42:07
*Madame 1:* Je vois. OK, alors là, si on s'intéresse à des façons de préparer et de consommer les fruits et légumes, tu m'en as déjà un peu parlé, si on prend les fruits, par exemple la fraise en général, tu manges comment ?

00:42:25
*Madame 2:* Comme ça, je lave même pas vu que ça vient du jardin. Je sais qu'il y a rien dessus.

00:42:34
*Madame 1:* Tu la manges crue. Est-ce que tu as d'autres façons des fois de la consommer ou pas du tout ?

00:42:39
*Madame 2:* Non, je mange cru.

00:42:41
*Madame 1:* Alors on a parlé de confiture, etc. Est-ce que ça t'arrive d'en faire ou ?

00:42:47
*Madame 2:* Pas pour les fruits frais jusqu'à présent, j'en ai déjà fait pour les pommes, pas pour les fraises. Et pommes et je sais plus quand je récupère des trucs de chez mes grands parents. Des fois, ça tient pas trop la route. Du coup, je fais des mélanges.

00:43:05
*Madame 1:* D'accord, si je reprends la pomme, la pomme, tu la consommes comment en général ?

00:43:11
*Madame 2:* Bah la pomme, soit je me d'abord, je commence toujours par mettre dans le bac température ambiante. Après moi, je mange pas trop, c'est plus mon copain qui les mange. Puis un jour en prenant des légumes en général, je vais me dire oh elle commence à vieillir. Et tout ça, je fais un gâteau avec soit je fais une compote vu que j'ai de la menthe et tout, je fais un petit mélange. Et après, le problème, c'est de conserver la compote. C'est ça notre problème.

00:43:35
*Madame 1:* Ah oui ?

00:43:37
*Madame 2:* J'ai pas appris encore, ma grand mère m'a montré, tu fais, tu mets dans un pot de verre, tu fermes bien et tu mets à l'envers, soit hermétique, mais ça ne fonctionne pas. Je ne dois pas avoir le coup de main je pense.

00:43:48
*Madame 1:* Ça se conserve.

00:43:53
*Madame 2:* En général, cinq fois, je me suis fait avoir ou je pensais que c'était bon. Je me suis dit c'est bon, là, je touche pas, faut surtout pas ouvrir, je mets de côté. Le jour où je m'en servir, c'est mort. C'est complètement moisi et j'arrive pas à faire encore longtemps. Je mets du citron et tout, je fais attention et j'évite de mettre du sucre.

00:44:11
*Madame 2:* Par contre, c'est peut être ça aussi, je sais pas.

00:44:17
*Madame 1:* Ok. Donc, finalement, quand on a des fruits quand même qui commencent à être fatigués, ça t'arrive d'essayer de faire autre chose pour.

00:44:28
*Madame 2:* Ouais. A part si c'est trop fatigué.

00:44:30
*Madame 1:* Ok, c'est-à-dire ?

00:44:31
*Madame 2:* Trop fatigué. Si on a les trois quarts d'un fruit qui sont, qui sont blancs ou noirs, commencent à moisi, c'est vraiment vraiment mort, ou, sinon on coupe toujours la partie. Et puis dès fois, on a l'impression que c'est une petite partie. En fait, quand tu coupes en deux, tu te rends compte que ça a pris tout l'intérieur. On mangeait beaucoup de clémentines, par contre, un moment aussi. Ça fait longtemps qu'on en a pas pris.

00:45:05
*Madame 1:* C'est possible. Ok et les légumes si on prend un légume, tu m'as dis le poireau en soupe, en salade avec le verre notamment, ou en poellées aussi avec les racines, la courgette que tu as des fois par kilo. Est-ce que tu peux me raconter comment tu consomme la courgette ?

00:45:32
*Madame 2:* Poêlée.

00:45:33
*Madame 1:* Poêlée ?

00:45:35
*Madame 2:* Ouais. Et quand j'ai trop de courgettes comme ça m'est arrivé cet hiver. Mais du coup, je mets tout. Je fais des soupes. On prend tous les légumes morts et on fait une soupe avec.

00:45:48
*Madame 2:* On les prépare quand même un peu avant, on coupe le moisi, on n'est pas à ce stade. On essaye.

00:45:57
*Madame 1:* Donc exclusivement en soupe ou en poêlée ?

00:46:01
*Madame 2:* Pour la courgette? Ouais, je mange pas cru. Et voilà.

00:46:09
*Madame 1:* Ça marche. Qu'est-ce que tu as dit? Les carottes, par exemple, comment tu consommes les carottes ?

00:46:21
*Madame 2:* Alors carottes. J'en ai fait hier, c'est en salade.

00:46:25
*Madame 1:* OK.

00:46:26
*Madame 2:* Sinon beaucoup à la poêle aussi. Un peu moins en soupe, je pense. Parce qu'on en a moins. Du coup, on les mange plus. Je vais manger en salade ou même cru comme ça. Souvent j'en prends et puis je grignote.

00:46:54
*Madame 1:* J'étais en pique nique hier j'ai amené des carottes.

00:46:56
*Madame 2:* A chaque fois je passe que je passe pour quelqu'un de bizarre quand je fais ça.

00:46:58
*Madame 1:* En pique nique une carotte.

00:47:07
*Madame 1:* Les courgettes est-ce que tu les épluche ou pas ?

00:47:13
*Madame 2:* Ça dépend où j'ai pris la courgette.

00:47:18
*Madame 1:* D'accord.

00:47:18
*Madame 2:* Quand je prends en supermarché, j'épluche. Quand ça vient de chez mes grands parents, pas forcément. Même si je ne suis même pas sûr de ce qui mette dessus et mon grand père me dit que c'est bio, mais je pense qu'on a tous une définition différente du bio. Donc, du coup, je dis oui, oui, mais bon, voilà. Mais bon, normalement, il n'y a pas des trucs très nocif de toute façon, dessus ou là, je mange, puis ça dépend. En fait, si je suis pressé ou pas aussi. Dès fois je coupe vraiment à l'arrache, hop dans une poêle, et puis, c'est bon je ne vais pas m'embêter. Mais j'essaie de garder un maximum la peau parce que je sais, que c'est souvent la peau qui est le plus riche. Donc, j'essaie de garder ça, même si mon copain par exemple lui, il n'aime pas les peaux et trouve ça trop amère, acide et tout. J'essaye de faire aimer le petit concombre, j'arrive quand c'est bien mélangé à des salades et tout. Il arrive à manger maintenant la peau, il trouve pas ça bon.

00:48:19
*Madame 1:* Et la carotte, est-ce que tu épluche ou pas ?

00:48:23
*Madame 2:* Non, ça j'épluche pas.

00:48:25
*Madame 1:* T'épluches pas la carotté.

00:48:25
*Madame 2:* Non, je la lave bien parce qu'en général, il y a de la terre et tout au moins, je prends souvent des sableuses, elles sont assez sales. Mais non je lave bien. Et puis voilà. Sauf quand elles sont vraiment trop sales ou un peu noircies. Et tout, là quand elles sont vieilles, je passe quand même un coup d'économe dessus. Mais sinon, j'essaie au maximum de manger avec la peau.

00:48:46
*Madame 1:* Ok, ça marche. Et la salade, quand tu, quand tu as de la salade, tu as dit que vous en faisiez de la salade ? Comment tu la prépares la salade ?

00:49:04
*Madame 2:* La salade du jardin et il y a de la vie dedans. J'ai retiré cinq feuilles. On s'amuse à retirer chaque limace qui est dedans. Ca me gave un peu. Mais je passe sous l'eau, les feuilles. Mais en général, elles sont jamais nickel, quoi. C'est juste histoire d'écraser le plus gros des bestioles, en fait, principalement. Mais après, moi, je m'en fous un peu puisque que la terre, vu qu'on est tout le temps dans la terre, je ne fais pas attention si je mange un peu de terre. C'est, c'est plus au niveau des limaces et tout. Je passe un peu d'eau. Je laisse pas, je laisse dans un coin comme ça, l'eau elle part un peu quoi. Puis après on mange ça comme ça.

00:49:52
*Madame 1:* Est-ce que t'as de l'épluchur dans ta salade ou pas vraiment ?

00:49:52
*Madame 2:* Ça dépend des salades, souvent, quand je prends dans le jardin, que je la retourne au niveau du pied. Il y a des trucs qui sont morts les feuilles mortes, je tire et vu qu'on en a beaucoup trop de salades et qu'on sait pas quoi en faire. Là, là j'avais un peu moins de scrupules, donc je tirais un peu ce qui était, ce qui était sale, quoi. Mais sinon, j'essaye de tout manger, sauf là quoi, de toute façon d'habitude j'en ai pleins. On a trop planté, on arrivait pas les manger, on a distribué à tout le monde mais on n'arrivait pas manger, donc on a dû jeter.

00:50:32
*Madame 1:* Ok. [Euh] Après à ma connaissance, justement vous jetez un fruit ou un légume ou une partie seulement. Quand ça arrive, pourquoi tu décides là de le jeter plutôt que de le consommer ?

00:50:48
*Madame 2:* Parce que c'est moisi, en général. Ou alors ça peut être, ça nous arrive rarement, mais je sais pas si on prend une tomate ou un fruit. Il y a une partie très dure qui est à moitié séchée, qui est qui est abîmée ou qui est très, très dure. Bah là on la tire celle là.

00:51:08
*Madame 1:* Ok.

00:51:08
*Madame 2:* Parce que c'est pas trop mangeable. Sinon, c'est quand c'est, c'est juste quand c'est vieux quoi. Quand c'est, soit déshydratées, il y a des trucs comme ça, quand c'est déshydraté on mange pas trop.

00:51:27
*Madame 1:* Et comment. Qu'est-ce qui te permet de déterminer que c'est moisi, que c'est déshydraté ?

00:51:33
*Madame 2:* Mais là, je pense aux radis, par exemple, quand j'ai des radis, je les coupe toujours au milieu déjà pour voir si il y a pas des vers dedans, parce que des fois, on ne voit pas mais il y a des verts au milieu. Donc quand y'a des vers je jette je mange pas. Je vais donc couper en deux et je regarde quoi. Et quand je vois que c'est déshydraté en général, je goûte un morceau quand même parce que des fois, ça va. Ça commence à déshydrater, mais ça va. Puis d'autres, c'est quand même. Je sais même pas à quoi ça pourrait ressembler, mais c'est vraiment bien du caoutchouc. C'est vraiment bizarre, ça on mange pas trop. En plus, on en a plein dans le jardin.

00:52:08
*Madame 1:* C'est des radis noir, ou des petits ?

00:52:10
*Madame 2:* Des petits, des noirs on a pas réussi. Ils sont tous montés en fleurs tout de suite.

00:52:17
*Madame 1:* C'est marrant, j'ai interviewé quelqu'un aussi qui a perdu tous ses radis, qui les avez cultivé, il avait pas réussi à les déssecher.

00:52:31
*Madame 2:* Des radis noirs ?

00:52:31
*Madame 1:* Non des petits radis, il les a tous perdu.

00:52:34
*Madame 2:* Ah ouais ? C'est bizarre. Parce que nous on a fait tomber un sachet sans faire exprès enfaite. Du coup, il a pris la fuite comme ça partout, et on a des radis qui ont poussé partout. Mais on en a dans le frigo, mais des beaux, de gros radis rose et tout.

00:52:48
*Madame 1:* Après en terme de conservation, t'as du mal à les garder ?

00:52:52
*Madame 2:* C'est bon, j'ai le truc du coup. Là, c'est bon, mais avant, on devait essayer de les manger très, très vite.

00:53:00
*Madame 1:* Ok. Donc finalement, quand tu vois que quelque chose est déshydraté tu vas quand même essayer de le couper et regarder si c'est mangeable, et s'il n'y a pas de mauvaise surprise dedans, de savoir si c'est bon ou pas ? Et qu'est-ce qui fait que si tu goute, tu te dis non, là, je jette. Qu'est-ce qui va venir déterminer ce moment là.

00:53:25
*Madame 2:* Texture et goût, texture puisque j'ai un problème avec la texture en général, y a des choses que je n'arrive pas à manger juste à cause de la texture.

00:53:34
*Madame 1:* OK.

00:53:37
*Madame 2:* Et après. Soit je goûte et je me dis je ne supporte pas la texture, mais ça a l'air d'aller quand même. Et là, dans ce cas là, je donne à [Prénom d'un membre de la famille]. Je dis moi, je peux pas manger, mais c'est bon en vrai, donc vas-y soit au niveau du goût. Des fois, ça a l'air très bon et quand on goûte c'est affreux, alors c'est hyper acide et on sent qu'il y a eu un problème dans le truc. Et du coup, je mets dans le compost. En plus, on a moins de scrupules à jeter quand même vu qu'on a un compost, mais on se dit que ça ira dans le sol. Ça va.

00:54:14
*Madame 1:* Donc, c'est parce que soit toi la texture te dérange ou parce que c'est pas bon et c'est acide que tu veux que tu peux jeter.

00:54:24
*Madame 2:* C'est ça.

00:54:27
*Madame 1:* Finalement, si c'est juste, c'est juste parce que tu sais comment je pourrais dire. Le goût n'est pas bon. Du coup, tu trouves que c'est plus mangeables. En fait, est-ce que tu penses que c'est réellement plus mangeable ou c'est que toi là, tu te dis que tu n'as pas envie de le consommer dans cet état ?

00:54:47
*Madame 2:* Nan, en fait. En général, quand je goûte quelque chose pour voir s'il est bon, c'est que déjà, le fruit, il est fin quand je l'ai vu. Mais je savais qu'il était vieux déjà et que la question, c'est est-ce qu'il est toujours mangeable ou pas par la vue ? Et du coup, c'est le goût qui va me décider à prendre. Et si j'ai un doute, si je me dis moi, j'aime pas, mais que ça va, c'est comme la texture finale, au final je donne à [Prénom d'un membre de la famille] je lui dis vas-y goûte et dis moi ce que tu en penses, toi tu penses que c'est toujours mangeable. Est-ce qu'on peut le faire parce que des fois, je récupère quand même des morceaux et je vais faire la poèle. Du coup, je réchauffe, oui. Puis ça passe quoi.

00:55:26
*Madame 1:* Ok. Ça marche. Du coup. Est-ce que tu arriverais quand est ce que c'est déjà arrivé de ne pas avoir vu que quelque chose était pas mangeable et de l'avoir mangé ? Et dans ce cas là, que se passe-t-il si tu te dis que la t'as u moisi, probablement du moisi dans la bouche, qu'est-ce que [Rires].

00:55:53
*Madame 2:* Je m'en fout un peu. Il y a une fois, mais c'était pas un fruit ou un légume. C'est un gâteau quand je travaillais dans le magasin de ma mère. On vendait des gâteaux artisanaux et tout. Et puis j'en ai pris puisque la date était dépassée elle aussi. Un gâteau ça se périme pas, j'ai pris une madelaine, la madeleine objet entre la caisse et tout en courant, je mangeais un bout je l'ai posé je suis partie. Quand je suis revenue j'ai vu que la Madeleine, en fait, était verte et rempli de mousse. J'ai fait oh non. [Rires]. Mes collègues se sont un peu fou de moiet c'est la fois où j'ai regretté d'avoir mangé un truc. C'était quand même bien moisi. Je ne sais même pas comment j'ai fait pour pas voir. Mais bon, après, j'ai pas été malade. Je suis du genre, ce qui n'est pas bien. Mais quand je fais à manger tout, quand j'ai des plats, je mets dans le frigo et je mange pendant plusieurs jours. Mais des fois, c'est la viande ou du poisson, donc des fois, c'est très limite, donc très, très limite. Mais vu que j'ai jamais été malade, je pense que mon cerveau ne comprend pas encore qu'il faut pas manger. J'ai déjà mangé des trucs c'était pas ouf. J'ai jamais été malade et comme toujours, c'est comme ça. Donc, c'est vrai que le moisi c'est pas un truc qui me fait très peur, après, j'évite un peu trop moisi. C'est pas une partie de plaisir. C'est juste que j'évite de jeter quoi.

00:57:14
*Madame 1:* Ok.

00:57:15
*Madame 2:* Quand je peux.

00:57:16
*Madame 1:* Donc c'est pas bon en goût, mais finalement, des fois, si ça passe encore. Tu regardes de consommer jusqu'à un certain stade ou

00:57:27
*Madame 2:* En fait, c'est qu'il y a des il y a des fruits et des légumes qui moisissent un peu.

00:57:31
*Madame 1:* Oui.

00:57:31
*Madame 2:* Où ça va pas altérer le goût forcément je trouve.

00:57:36
*Madame 1:* Lesquels par exemple ?

00:57:37
*Madame 2:* J'en sais rien. Je pensais peut être un peu aux pommes, courgettes et tout. Quand on sent que ce c'est plus la même couleur, ça devient marron pour les pommes et tout, et que vraiment, c'est pas terrible. Même les bananes. Ça, je peux manger. Je trouve pas que ça altère énormément le goût. A part les bananes, vraiment, quand ça devient vraiment foncé, c'est pas bon. Mais sinon, sinon, ça va plus loin quand ça devient trop comme les tomates et tout, je tire que s'il y a du blanc, de la mousse, tout, je tire, je fais un rond et je mets dans la poêle.

00:58:10
*Madame 1:* Donc tu enlèves les bouts moisis et tu vas avoir tendance à cuire.

00:58:16
*Madame 2:* Cuire en général, si je fais une salade, je mets quand même dans la salade.

00:58:23
*Madame 1:* Est-ce que tu serais qualifié ou pas? C'est la question. Le goût du pourri, du moisi ?

00:58:32
*Madame 2:* Non, à part moi en général, je trouve que c'est quand même ça tire plus vers de l'amère et de l'acide. Un peu, ça tire un peu vers là, mais j'ai l'impression que ça dépend vraiment des fruits et des légumes. En fait, ça dépend. Puis, il y a des fruits, j'arrive pas à savoir, par exemple ou je sais pas tout ce qui est Clémentine ou Orange. Les fruits un peu comme ça, j'ai l'impression qu'ils sont plus bons avant même qu'il y ait le blanc, que ce soit visuellement, j'ai l'impression que c'est plus bon en mangeant parce qu'il y a quand même un truc, un goût en plus qu'il n'y a pas normalement, quoi. Un truc qui tire vers l'amer, plus acide encore que le fruit original, vraiment que ça change, alors qu'il n'y a pas forcément de tu vois qu'il est un peu vieux. Mais t'as pas le blanc ou encore et tout ce que tu veux visuellement quoi.

00:59:28
*Madame 1:* C'est avec des fruits ou légumes qui sont plus, qui mettent plus longtemps à créer une mousse qui montre visuellement que c'est pourrie.

00:59:38
*Madame 2:* Mais je pense.

00:59:39
*Madame 1:* Mais que l'intérieur le vieillissement est déjà un peu.

00:59:45
*Madame 2:* Souvent, ça nous faisait avec les clémentines et tout. Et je me dis c'est peut être à cause de l'écorce qu'on voit que comme. Et l'écorce d'orange et tout, je trouve que c'est quand même assez dur par dessus. C'est bien protéger.

01:00:02
*Madame 1:* Ok. Alors. Finalement, toi, quand tu te retrouves face à du face à du pourri, du moisi, qu'est-ce que tu ressent quand tu vois qu'un fruits et légumes, par exemple, est pourri, moisi,

01:00:21
*Madame 2:* Ca peut commencer par un merde je l'ai oublié. Ah Beurk! Ca me dégoute. Donc ça, ça dépend du degré de moisi, quoi. Et. Des fois, je me dis juste, juste je vois qu'il commence un peu vraiment à passer quoi. Comme comme la pomme qui devient marron je fais ah merde souvent, mais c'est un marron léger, quoi. Alors que quand vraiment ça se creuse, que tu vois qui a la vie dedans et tout ça devient compliqué. J'ai beaucoup plus de mal quand même. Là, j'ai juste envie de prendre des gants, de prendre le fruit et de jeter. Même si j'essaie de regarder si n'a eu que la moitié, c'est drôle de regarder. Mais je sais que dans ce cas là, en général, c'est toucher un peu partout à l'intérieur. Donc c'est pas.

01:01:12
*Madame 1:* Donc, si c'est le fruits ou légumes, commence à s'abîmer tu te dis mince j'aurais du le manger plus vite. Et quand il est vraiment vraiment pourri, ça.

01:01:24
*Madame 2:* Ca me dégoute.

01:01:28
*Madame 1:* Ca te dégoute.

01:01:28
*Madame 2:* Même si j'accepte de plus en plus ces bestioles parce que je pensais les bestioles depuis que ce que je jardine, j'arrive de plus en plus les prendre une main. Je me soigne avecça souvent, parce que vraiment, j'avais peur de ça. Alors c'est débile d'avoir peur de ça.

01:01:44
*Madame 1:* [Inaudible 01:01:45-01:01:48]

01:01:47
*Madame 2:* Oui voilà mais ça va pas manger quoi, je risque rien [Rires].

01:01:59
*Madame 2:* Et j'apprends ça aussi. J'apprends les fleurs aussi, là, à manger des fleurs comestibles.

01:02:08
*Madame 1:* OK, alors si on discute du gaspillage alimentaire, quand je te dis gaspillage alimentaire, c'est quoi les mois qui deviennent comme ça spontanément à l'esprit ?

01:02:21
*Madame 2:* Fruits, légumes ?

01:02:25
*Madame 2:* Gaspillage alimentaire je vois les grandes poubelles en dehors des magasins.

01:02:30
*Madame 1:* Ouais.

01:02:32
*Madame 2:* Je vois bien. Je l'ai entendu ça, mais ça me fait penser à la javel qui peuvent mettre sur les fruits et légumes pourpas être récupérés. Gaspillage alimentaire ? Ça peut me faire penser au début de la chaîne, quand le paysan va ramasser ses fruits et légumes dans la partie qui va jeter deéjà avant même que ce soit amené quelque part.

01:02:56
*Madame 1:* Ouais.

01:02:59
*Madame 2:* Ça me fait pas trop penser au particulier chez lui, avec ses fruits et légumes, en fait, mais plus en amont, c'est vraiment tout le gaspillage qui peut y avoir avant et du coup, en nouvelle, tout ce qui est de plus en plus nouveaulà les paniers, un peu de produits abîmés ou dates cours, des trucs comme ça. Moi, j'essaie de récupérer ça. Des fois, quand je passe par hasard et que je vois des panier, je les récupère en général. De fruits, de légumes passés.

01:03:27
*Madame 1:* Qui commencent à être abîmés, qui vont être jetés par les distributeurs. Si pas de consommateur. OK, donc, pour le gaspillage alimentaire, il a un peu. Quand tu entends gaspillage alimentaire, tu vois plutôt la production qui va produire, puis jeter déjà une partie, puis les distributions qui vont venir jeter tout ce qui n'a pas été vendu et vendu tout.

01:03:52
*Madame 2:* Ou tout ce qu'ils ont abîmé en les lançant pour le mettre dans le rayon

01:03:58
*Madame 1:* Ok. Ça marche alors? Et si on essayait de faire une une définition du gaspillage alimentaire ? Donc toi et tes propres mots et ta propre façon de concevoir le gaspillage, donc qu'est-ce qu'on y verrait à ta définition du gaspillage si tu devais écrire dans le petit Robert la définition ?

01:04:21
*Madame 2:* Le gaspillage alimentaire. C'est le fait de Jeter des fruits ou des légumes non consommés abîmés. Ou, non, en fait, non, je retire ce que j'ai dit, c'est le fait de jeter des fruits et des légumes enfaite tout court. Dès fois, il y a même pas de raison.

01:04:45
*Madame 1:* Oui.

01:04:46
*Madame 2:* Et même en fait, c'est tout aliment fait de jeter, c'est le fait de jeter tout aliment.

01:04:57
*Madame 1:* OK, quand toi, tu es amené du coup à jeter un aliment. Qu'est-ce que tu ressens forcément des produits pourris et l'action de jeter. Qu'est-ce que tu ressens ?

01:05:12
*Madame 2:* Bah ça m'mebête quand même.

01:05:13
*Madame 1:* Ouais.

01:05:15
*Madame 2:* Même, même si c'est des trucs, je sais pas comme la soupe des trucs comme ça, parce que ça nous est arrivé, on est que deux. On fait tous les trucs, un peu tous les légumes un peu vieux. Des fois, on n'arrive pas à finir la soupe avant qu'elle-même moissise. Mais du coup, ça m'embête. Je me dis, là, on a le composteur, donc ça va. On arrive à récupérer un peu ce qu'on jette, mais quand c'est de la soupe, je ne me vois pas mettre de la soupe dans le composteur. Je sais pas pourquoi, je trouve pas ça terrible pourtant ça reste des légumes et Ça m'embête enfaite.

01:05:50
*Madame 1:* Oui.

01:05:55
*Madame 2:* Ça me gêne et des fois, je me dis que j'arrête d'acheter ça si je suis pas capable de le manger, donc j'arrête. Par contre, ça m'est arrivé de jeter volontairement des produits qui ne sont pas périmés. Par exemple, ce week end, j'ai jeté du ketchup.

01:06:13
*Madame 1:* Ok.

01:06:14
*Madame 2:* Parce que des amis sont venus manger et nous ont laissé un pot de ketchup alors que nous on mange pas de ketchup, donc, et je cours. Je me suis dit oh zut ils sont partis sans et vu qu'ils habitent loin, j'allais pas les rappeler. J'ai mis dans le frigo. Je me suis dit on va bien avoir du monde à venir manger on va le ressortir, mais ça fait deux mois qu'il est là et on a pas touché au pot de ketchup. À un moment, je dis ça sert à rien. Ça, c'est de la merde. J'ai jeté sans aucun scrupule pour le coup. Je me dis c'est con parce qu'il y en a qui sont sûrement contents de manger ça. Mais nous, on refuse de manger ces trucs là. Tant pis. J'ai mis à la poubelle, mais le pot était plein. Il était rempli de trucs aussi, mais pourquoi il y a les gens qui laissent ça chez nous.

01:07:00
*Madame 1:* Et du coup, t'as dis c'est de la merde, j'ai jeté sans scrupule. Il y a bien un rapport avec le produit et du coup, comment tu vas te sentir au moment de le jeter ou ?

01:07:14
*Madame 2:* Ouais, ouais, les produits comme le ketchup, qui est ta qui est dit. En gros, c'est censé être du jus de tomate. Et quand tu regardes les ingrédients, on a presque pas de tomates dedans et qu'au final, c'est ultra transformé, t'as plein de sucre de vinaigre. Tous les produits transformés comme ça, je me dis au final, c'est pas des légumes que je jette vraiment. Du coup, ça reste que des trucs qui sont nocifs pour la santé qui sont faits dans je ne sais pas quelle condition. En fait, je me dis au final, ce n'est pas une perte pour nous. C'est quelque chose de mauvais qui va à la poubelle. Pas mauvais dans le sens périmé, mais mauvais quoi.

01:08:03
*Madame 1:* Ça marche. Alors si, pour terminer juste sur la poubelle connectée qui est du coup chez vous depuis quelques semaines maintenant, comment ça se passe ? Est-ce que tu as des trucs à dire ? Si vous l'utilisez beaucoup, au contraire pas beaucoup ?

01:08:20
*Madame 2:* Bah pas beaucoup, en fait, parce que des fois, je vais l'utiliser un peu. En général, je mets un légume dedans et puis je ferme, je me dis c'est bon et tout est vu que bon, on jette pas beaucoup. Mais du coup, je l'oublie. Et quand je l'ouvre, je vois que c'est tout moisi. Et je me dis oh merde j'ai oublié le truc, du coup je vide le sac. Vider les sacs vide en fait. Parce que c'est limite du jus. Et des fois, c'était vieux et qu'on mets rien dedans presque. Au final, je pensais qu'on jetait plus, mais non au final on jette les épluchures, mais par contre des épluchures. Ça j'en jette tout le temps, beaucoup. Mais les fruits et légumes, vraiment, des fruits et légumes encore qu'on peut jeter en entier ou presque. Ça en jette pas du tout.

01:09:14
*Madame 1:* Est-ce que tu peux te souvenir de quelques exemples de ce que tu as pu jeter dans la poubelle parce est-ce que t'as un souvenir ?

01:09:23
*Madame 2:* Je j'ai jeté des tomates. Je crois.

01:09:29
*Madame 1:* Ok.

01:09:31
*Madame 2:* Parce qu'elles étaient, je pense que avez dû avoir plein de choc avant qu'on les prenez et à peine prise y en a pleins qui ont commencé à noircir, à s'écraser et à perdre du jus. Et tout, et quoi? Du coup, j'en avais beaucoup, donc les plus abîmés, je les mises à la poubelle et puis voilà.

01:09:46
*Madame 1:* Est-ce que des fois, il y a eu des petits bouts que t'as pu enlever. Ou ceux-là elles sont plus passés avec les épluchures.

01:09:54
*Madame 2:* Ils sont plus plus passé avec des épluchures les petits bouts.

01:10:02
*Madame 1:* Est-ce que des fois, ça peut arriver à toi ou ton copain d'oublier la poubelle, de donner, d'avoir jeté un truc et se dire ça, ça aurait pu aller dedans. Et là, j'ai oublié ce qui est pas grave. Mais qui aurait pu ou on aurait pu dire il y aurait peut être pu avoir deux ou trois trucs en plus et il n'y a pas eu ou non t'as pas le souvenir ?

01:10:25
*Madame 2:* Moi, j'ai pas souvenir après c'est possible, par exemple les radis quand je les ai en morceaux pour voirles verts et tout, je pense pas avoir mis dans la poubelle parce que j'ai mis.

01:10:36
*Madame 1:* Ok. Est-ce que des fois, c'était arrivé de te poser des questions, est-ce que ça doit aller dedans ou pas ?

01:10:54
*Madame 2:* Non, ça me semble assez clair.

01:10:57
*Madame 1:* Ok. Et du coup, en termes de sac, t'en as utilisé combien pour le moment ?

01:11:06
*Madame 2:* 3

01:11:06
*Madame 1:* 3. T'es au troisième ?

01:11:07
*Madame 2:* Oui, il m'en reste deux fois.

01:11:18
*Madame 1:* Je vais t'en redonner du coup.

01:11:23
*Madame 1:* Je t'ai posé l'ensemble de mes questions. Merci beaucoup d'y répondre. Est-ce qu'il y a des choses sur lesquelles tu voudrais venir, est-ce que tu penses que j'ai pu oublier des points. Des éléments sur lesquels que je n'ai pas abordé sur notre conso de fruits et légumes.

01:11:40
*Madame 2:* Non, je crois que c'est bon.

01:11:42
*Madame 1:* On a fait un bon petit tour.

01:11:53
*Madame 2:* Peut-être un truc je sais pas si ça t'intéresse, mais nous on essaye vraiment d'essayer de consommer. Non pas que le fruit ou le légume, mais aussi les feuilles en fonction de ce qui est consommable ou pas. J'ai vraiment essayé de prendre le fruit dans sa globalité

01:12:05
*Madame 1:* Comme tu disais le poireau jusqu'à ce que, jusqu'à épuisement du légume,

01:12:13
*Madame 1:* Le chou c'est pareil ?

01:12:14
*Madame 2:* Le chou, on n'a pas mangé de chou parce que les vaches mangent avant nous,

01:12:20
*Madame 1:* Ah oui c'est vrai.

01:12:21
*Madame 2:* Du coup on a pas pu. Les seules choses et on commencé à se former. Et puis ils sont montés direct, en fleur et tout. Et après, ça a été bouffé partout où on n'a pas pu ou non plus pour cet hiver. Mais on essaye après. Voilà ce que je te disais, tout ce qui est fleurs et tout aussi. On essaye donc voilà.

01:12:41
*Madame 1:* Et tu as en tête des, des légumes que tu sais que la plupart des gens vont, ils vont jeter et que vous vous allez faire l'effort comme le même effort que pour le poireau.

01:12:54
*Madame 2:* Moi c'était le poireau qui m'avait le plus surprise.

01:12:56
*Madame 2:* C'était vraiment le poireau. Parce que quand je vois que depuis toute petite on me donnait que le blanc a mangé, quand j'ai vu tout se manger. Sinon. Qu'est-ce qu'on essaie de manger ? Les radis, les radis, on mange pas les feuilles, encore faut que je regarde d'ailleurs ce que je me disais parce que je suis sûr, ce mangeable, faut que je vérifie avant de le faire. Mais tout ce qui est racine, [Euh] les mets les oignons, du coup. Des betteraves on mange pas encore les feuilles. Les salades, ça, c'est normal, je sais pas trop.

01:13:45
*Madame 1:* Et est-ce que ça t'arrives par exemple les fans de radis et les fans de carottes, est-ce que tu les consomme ou pas?

01:13:52
*Madame 2:* Oui, j'ai jamais eu de carottes. Vraiment encore. Et justement, les fans de carottes, il faut que je regarde ce que je sais. Je sais que ça, ça se mange. Je pense que je garderais ça pour. Je ne sais même pas si cet hiver, je peux essayer une fois, comme avec des carottes prises au marché.

01:14:09
*Madame 1:* Alors, à force de faire les entretiens, j'ai appris qu'on pouvait faire du pesto avec les fans de carottes et faire pas mal de gens qui se mettent à faire du pesto quand on a beaucoup de fans, le pesto à mon avis c'est comme la compote, on perd. Mais n'empêche qu'il paraît qu'on peut faire du pesto avec les fans de carottes

01:14:32
*Madame 2:* Et les fans de radis se mangent du coup ?

01:14:34
*Madame 1:* Les fans de radisse mangent aussi, apparemment, des gens dès fois qui les mettent dans les soupes. Apparemment, les fans de radissont donc assez forts en goût. Mais en fait, ça se mange tout ça. Peut être aussi des formes, un peu comme des herbes, une salade, mais à petite doses, dans une grosse botte. Est ce qu'on est capable de manger vraiment toutes les fans ?

01:14:54
*Madame 2:* Ca passe vite.

01:14:55
*Madame 1:* Mais oui, c'est ça.

01:15:00
*Madame 2:* Je pensais Navet aussi. Oui, ça aussi, on essaye de langer.

01:15:03
*Madame 1:* Le navet c'est pareil à part en soupe.

01:15:09
*Madame 2:* Mais surtout, surtout, ne jamais que des navets pour une soupe, mon copain a fait ça pour me faire plaisir un soir il a prit tous les navets, il avait fait une soupe de navets. C'était affreux, hyper acide et le goût. Mais je lui ai dit qu'est-ce t'as fais, les gens autour de nous nous ont dit pouquoi vous avez mis que des navets dans une soupe.

01:15:35
*Madame 1:* Il se fait noyer par le reste des patates. Je l'avais cuisiné mais j'étais pas convaincue.

01:15:44
*Madame 2:* Doré au beurre.

01:15:46
*Madame 2:* Oui, on m'a dit il y a plein de gens. C'est leur façon préférée de faire les navets.

01:15:51
*Madame 1:* Je l'avais fait à la poêle et je trouve pas ça ouf.

01:15:56
*Madame 2:* Je trouve pas ça. Mais bon, ça se mange quoi? Mais bon, ce sont plus juste.

01:16:02
*Madame 1:* Et quand je me suis retrouvé coincé avec des navets parce que sinon j'en consommerais pas, je que je prenais des paniers quand j'étais l'Ubu, il y avait une, une, AMAP qui venait et du coup, pendant longtemps que je me retrouvais avec des navets, je ne savais que faire pour passer à la soupe. Et sinon, un peu galère. J'ai recommander des paniers de la semaine dernière.

01:16:28
*Madame 2:* Ca a réouvert ?

01:16:30
*Madame 1:* Mais j'ai pas pris les mêmes. Oui, j'habite moi à Recouvrance. Il y a un bar qui fait à côté de chez moi, qui récupère les, qui fait lieux de récupération des paniers. Et mon copain travaille à côté, vraiment à côté du bar, donc c'est ultra pratique. Donc là, j'ai repris, on a essayé et ça commence à me créer des de la perte, en fait, parce que je me retrouvais avec des légumes que je ne voulais pas consommer ou que j'avais la flemme de cuisiner. Et du coup, plus je retrouver à perdre des trucs bêtement.

01:17:05
*Madame 2:* Maintenant, c'est ça. J'ai pris un panier qui allait à la poubelle, donc je vais récupérer et il y avait de la rhubarbe dedans. Ouais, mais la rhubarbe, t'en avait qui était déjà morte de chez moi dans le jus.

01:17:16
*Madame 1:* Ouais.

01:17:18
*Madame 2:* Et les autres? J'ai hésité pour essayer. Mais comment je vais faire la rhubarbe? Et avec 3 tiges, elle est toute petite. Quand j'étais là, j'ai pas faire de la compote avec ça. Sachant que j'ai pas d'autres fruits. Je mange pas ça à la poêle. Tant pis j'ai mis dans le composte.

01:17:34
*Madame 1:* Et quelqu'un qui a dit ça aussi sur le fait fois le fait de quand on est investi contre le gaspillage alimentaire et que du coup, on va justement aller acheter ces produits là et en fait, on se retrouve à sur sa poubelle. Mais du coup, je me mets à jeter beaucoup, mais c'est parce que j'achète des choses qui sont déjà déjà pourries. Du coup, quand j'arrive, je viens de faire le tri et j'arrive à en sauver. Il y en a d'autres. Ben non, ils sont déjà pourris. Ça part dans la poubelle connectée. Du coup ça fait du poids comme si c'était une personne qui gaspillait beaucoup, mais en fait, c'est parce que c'est parce que non. Parce qu'ils achètent des produits qui sont déjà très abîmés, qui partiraient forcément à la poubelle si elle les avait pas acheté quoi et ça c'est à prendre en compte, forcément.

01:18:19
*Madame 2:* C'est vrai que c'est difficile. Nous ça va on a moins de scrupule à jeter, dans le sens où il y a le compost, dans tous les cas ça sera mieux quand dans une poubelle. Donc, c'est vraiment pour ça qu'on a mis le compostage. On se voyait jeter à la poubelle qui se remplissait vite d'épluchures et tout. Je me disais c'est pas possible qu'on jette autant, alors que la terre qu'on arrive ici, c'est une cata la terre ici. Quand t'es de ce côté là elle est bien, plus tu vas vers là plus c'est du cailloux et du sable. Et du coup, quand on voulait cultiver là, putain la galère. C'est pour ça qu'on a fait un compost. Puis on mélange tout le temps. J'ai planté des physalis encore, vu que c'est son truc préféré. Il négocie à chaque fois pour ajouter un plan. Il a réussi à mettre 5 plants juste dans l'espace.

01:19:13
*Madame 1:* Par raport à tout ce que vous cultivez c'est impressionnant, c'est ici là devant.

01:19:23
*Madame 2:* Ouais. Et puis un peu la. Là t'as pastèque et melon, pastèque c'est celui où tu vois la petite fleur, la.

01:19:34
*Madame 1:* OK.

01:19:36
*Madame 2:* En face, je crois que c'est un melon et un deuxième melon, donc. Quant à la lavande qui fait fuir les bestioles et sous la bâche, là, c'est des fraises. C'est le citronnier premier.

01:19:48
*Madame 1:* Ok et à côté c'est quoi ?

01:19:54
*Madame 2:* Mais justement, c'est le truc chelou qu'il y a entre les deux, on a goûté, ça se mange, c'est pas méga acide, mais c'est quand même assez acide qu'il faut aimer, ce qui est un peu acide. Et moi, j'aime pas trop trop.

01:20:09
*Madame 1:* Sinon il faut les mélanger dans des jus, un jus d'orange et rajouter un peu de ça.

01:20:14
*Madame 2:* Oui, on avait fait ça avec des fruits comme ça. C'était une grand mère qui parlait, qui avait fait une confiture avec des fruits comme ça, une courge de spaghetti, donc une confiture.

01:20:27
*Madame 1:* Ça avait contrebalancé.

01:20:28
*Madame 2:* Du coup c'était moins acide, mais c'est quand même très acide et j'ai pris ce que je voulais. Qu'est-ce que je vais foutre avec ça ? J'ai récupéré une mangue et j'ai fais une tarte, donc avec ça et la mangue. Et en fait, c'était nickel. Alors que j'aimais pas forcément.

01:20:46
*Madame 2:* Et là, du coup, ça avait équilibré et du coup, ça passait bien

01:20:49
*Madame 1:* Il y a des stratégies.

01:20:51
*Madame 2:* Mais la mangue c'est vraiment pire, je savais pas quoi faire de la mangue. Je les fais en tarte, allez tout le monde va manger ça, comme ça ça va aller vite.

01:21:00
*Madame 1:* C'est super dur à juger quand est-ce qu'elle est prête à être mangé. Je ne sais pas quand. Est-ce que si on peut faire pousser ça

01:21:11
*Madame 2:* ici, la.

**Household F20, Interview 2**

*Mme. 1:* Alors, pour commencer, c'est comme un petit jeu. Il y a des illustrations ici et je vais te demander, [du coup], [euh], d'en retourner une et de me dire spontanément ce qui te viens, [euh], à l'esprit.

*Mme. 2:* Le marché.

*Mme. 1:* Ouais.

*Mme. 2:* Je, [euh], [ouais], je dis plein de truc [quoi].

*Mme. 1:* Ouais ce que ça t'évoques.

*Mme. 2:* Légume frais.

*Mme. 2:* Rencontres.

*Mme. 1:* Ouais.

*Mme. 2:* Froid. [Uhm], abordable.

*Mme. 1:* Ouais.

*Mme. 2:* Sympa, convivial.

*Mme. 1:* Ok.

*Mme. 2:* Voilà, je pense que--

*Mme. 1:* T'as fais ton petit tour ?

*Mme. 2:* Ouais, je pense.

*Mme. 1:* Alors, tu as dit [euh], je pense qu'on peut-- rencontre [euh], sympa, convivial, qu'est-ce que-- qu'est-ce que tu veux dire par [euh]-- pourquoi tu penses à ces mots-là ?

*Mme. 2:* [Ben] parce que le marché nous permet de rencontrer du monde.

*Mme. 1:* Oui.

*Mme. 2:* Moi j'aime bien discuter un peu avec des mamies ou des papis qui sont là-bas.

*Mme. 1:* Ouais.

*Mme. 2:* [Euh] après, les- les vendeurs aussi j'aime bien discuter un peu. Leur demander d'où vient le produit, [enfin voilà].

*Mme. 1:* Ouais.

*Mme. 2:* Même si je n'attends pas forcément de bonnes réponses, mais juste, voir un peu leur [euh]-- [ben] [euh] comment dire-- leur [euh], qu'ils me disent si c'est pas eux qu'ils [euh], le font, qu'ils le disent, [quoi], [en fait].

*Mme. 1:* Ouais.

*Mme. 2:* Juste qu'ils soient-- merde je trouve pas le mot. Qu'ils soient--

*Mme. 1:* Transparents ?

*Mme. 2:* Ouais, transparents voilà. Merci. [Hum]. J'ai une voix chelou [du coup] !

*Mme. 1:* [Rire]. C'est pas grave.

*Mme. 1:* [Euh] ok donc c'est un lieu de rencontre et en plus [euh] tu aimes le contact avec les- les vendeurs, voire le producteur quand c'est [euh]- c'est eux qui- qui vendent eux-mêmes. [Euh], t'as dit froid. Pourquoi t'as pensé à froid ?

*Mme. 2:* Parce qu'on est en hiver et que ces temps-ci je me dis il faut que j'y aille le dimanche matin. Et que quand je vois la pluie, tout ça, je me dis je vais peut-être faire autre chose au final.

*Mme. 1:* Ouais.

*Mme. 2:* [Et du coup], c'est vrai que ces temps-ci j'ai un peu de mal. Pourtant l'année dernière j'ai fait tous les dimanche en hiver [et tout] et là [euh]--

*Mme. 1:* Ouais.

*Mme. 2:* C'est la fatigue [et tout] qui font que j'ai vraiment pas trop le courage de me lever tôt pour y aller. Et l'année dernière, je travaillais dans la nuit du samedi à dimanche.

*Mme. 1:* Ah oui ?

*Mme. 2:* Dans tous les cas, je finissais mon travail à 8 heures, le matin, donc j'y allais sauf que là, maintenant c'est passé du vendredi au samedi donc [euh] le samedi je préfère dormir.

*Mme. 1:* Bah oui, oui, oui !

*Mme. 2:* Et le dimanche [et du coup] [ben] je vais plus le matin, c'est un peu--

*Mme. 1:* Oui, parce que [du coup] tu étais debout à 8 h [euh]--

*Mme. 2:* Oui c'est ça. Donc je venais ici, puis je disais, je motivais mon copain à 8 heures "Aller hop ! Tu te dépêches, on y va !" et voilà.

*Mme. 1:* Ouais.

*Mme. 2:* Mais ouais. Ou alors, j'y allais toute seule.

*Mme. 1:* Ok. [Euh] t'as dit abordable. Qu'est-ce que tu veux dire par là ?

*Mme. 2:* [Ben], parce que moi, je trouve que c'est pas très cher.

*Mme. 1:* Ouais.

*Mme. 2:* [Enfin] quand je prends des sacs de légumes vraiment, je trouve que ce n'est vraiment pas cher [quoi]. Peut-être un peu plus cher maintenant qu'avant, [euh] par rapport il y a 2 ans.

*Mme. 1:* Ouais.

*Mme. 2:* J'ai l'impression que ça a pris un peu mais [euh], [ben] peut-être que je me trompe.

*Mme. 1:* Ouais.

*Mme. 2:* Mais en tout cas [euh], je trouve pas très cher.

*Mme. 1:* Ouais.

*Mme. 2:* Au niveau des légumes.

*Mme. 1:* D'accord. [Euh], globalement est-ce que le marché c'est un endroit dans lequel tu te sens bien ? Ou non c'est, [euh]-- t'y vas par nécessité ? Comment tu vois les choses quand t'es au marché ?

*Mme. 2:* Ça dépend des jours. [Alors] les jours où il ya énormément de monde [euh], je mets mon casque, je me mets dans ma bulle et je vais dans la foule.

*Mme. 1:* D'accord.

*Mme. 2:* J'ai horreur quand il y a du monde. [Enfin], j'aime pas ça [en fait]. Les gens n'avancent pas [et tout]. [Enfin], ça me stress.

*Mme. 1:* Ouais.

*Mme. 2:* Mais quand c'est un peu plus léger et que, il n'y a pas énormément de monde, j'aime bien prendre mon temps, discuter [enfin] tout ça.

*Mme. 1:* D'accord.

*Mme. 2:* Ça dépend vraiment de la densité de population [en fait].

*Mme. 1:* Donc s'il y a pas grand monde, t'aurais tendance à prendre ton temps ?

*Mme. 2:* Ouais c'est ça.

*Mme. 1:* Mais s'll y'a du monde [euh], tu traces [euh]--

*Mme. 2:* Je vais à mes objectifs directement [rire]. Je prends ce dont j'ai besoin et je repars. Limite chronométré [quoi].

*Mme. 1:* [Rire]

*Mme. 2:* Et quand quelqu'un me parle, je lui dis tout le temps "Non, je n'ai pas le temps". [Rire]

*Mme. 1:* Donc, [euh], au final c'est [euh]-- pour toi il y a un côté agréable quand il y a peu de monde. Mais au final quand il y a du monde c'est pas si agréable [euh] --

*Mme. 2:* Non, non, non c'est stressant même.

*Mme. 1:* Ça te stress.

*Mme. 1:* D'accord. [Du coup], ça t'arrives de consommer des produits qui [euh], sont issus du marché ?

*Mme. 2:* Oui.

*Mme. 1:* [Euh], t'en penses quoi de ces produits-là ? T'as dit déjà qu'ils étaient abordables mais [euh], qu'est-ce que t'en penses d'autres ?

*Mme. 2:* [Euh], moi je trouve qu'ils sont bons, après en général ils sont sales [et tout] mais au moins ça me donne une raison de les laver.

*Mme. 1:* Ouais. [Rire]

*Mme. 2:* Alors que, [ben] [du coup] je fais le parallèle avec les légumes et fruits des grandes surfaces. [En fait], ils sont propres. Je sais qu'il faut les laver.

*Mme. 1:* Ouais.

*Mme. 2:* Et pourtant, [eh ben] c'est comme si c'était un truc chiant [en fait].

*Mme. 1:* Ouais.

*Mme. 2:* Parce que, [ben] au final que-- quand je vois le légume avant le lavage et après le lavage, il a pas du tout changé. Il est exactement pareil.

*Mme. 1:* Ouais.

*Mme. 2:* Je sais même pas si ça a servi a quelque chose.

*Mme. 1:* Ouais.

*Mme. 2:* Alors quand ça vient du marché [ben] j'ai plus confiance [en fait] parce que je vois qu'il y a de la terre donc je vais retirer la terre. Puis même si je mange un peu de terre, ça me dérange pas [en fait].

*Mme. 1:* Ouais.

*Mme. 2:* Enfin, c'est différent je trouve.

*Mme. 1:* Ouais. Oui, quand tu-- t'as la la terre, tu vois, tu sais pourquoi tu le laves--

*Mme. 2:* Oui, c'est ça oui. Puis ça me fait plus-- [enfin], je ne sais pas pourquoi j'ai plus confiance, comme si [du coup], le fait d'avoir de la terre, il y avait moins de pesticides.

*Mme. 1:* Ouais.

*Mme. 2:* Mais bon, je sais que ce n'est pas logique.

*Mme. 1:* Donc, tu les trouves globalement bon [du coup], les produits ?

*Mme. 2:* Ah ! oui, oui, oui. Ouais. Oui, oui, ils sont très bons. [Enfin] c'est des produits normaux [quoi].

*Mme. 1:* Ouais. [Euh], Est-ce que ça t'arrive d'en jeter [du coup], des produits qui viennent du marché ?

*Mme. 2:* En général, pas trop. Parce qu'on fait de la soupe.

*Mme. 1:* Ok.

*Mme. 2:* Donc [du coup] ça va dans la soupe. Et puis je prends-- quand j'y vais en tous cas, c'est toutes les semaines et [du coup] ça n'a pas le temps de pourrir dans la semaine [en fait] [donc] non ça va. Après, là cette période-ci, je-- on- on mange beaucoup de plats déjà préparés.

*Mme. 1:* Ouais.

*Mme. 2:* Parce qu'on n'est jamais là, [enfin] on est toujours ailleurs [et tout], on rentre très tard.

*Mme. 1:* Ouais.

*Mme. 2:* Et [du coup] on n'a pas le temps-- enfin, on ne prend pas le temps de cuisiner le soir en rentrant [quoi].

*Mme. 1:* Ouais. Ouais faut du temps pour [euh] cuisiner.

*Mme. 2:* [Ben] au moins un peu ouais. Parce que moi là, je travaille au Crous, donc le midi on fait avec les repas du Crous.

*Mme. 1:* Oui.

*Mme. 2:* Et le soir [euh], le soir, on vient d'acheter une mini pelle à construire donc on a pas mal de boulot là, le soir.

*Mme. 1:* [Ah Ouais].

*Mme. 2:* On est dans le froid tous les soirs. Et là [euh] quand je rentre, moi je refuse de cuisiner tu vois ?

*Mme. 1:* Bah ouais, ouais.

*Mme. 2:* Je dis c'est bon je vais dans le lit et je bouge plus.

*Mme. 1:* Ben ouais.

*Mme. 2:* Et voilà !

*Mme. 1:* Ok. Et alors si [euh]-- si [euh], malheureusement on n'a pas le choix de jeter un produit qui vient du marché, qu'est-ce que tu éprouves à ce moment-là ?

*Mme. 2:* Oh rien.

*Mme. 1:* Rien de spécial ?

*Mme. 2:* Rien parce qu'en général quand je jette, je le mets dans le composteur qu'on a là.

*Mme. 1:* Ouais.

*Mme. 2:* Donc [en fait], je sais que je vais le remettre dans le terrain pour la terre donc [du coup] ça me dérange pas du tout.

*Mme. 1:* Ok.

*Mme. 2:* [Ben] je me dis que peut-être que c'est un peu bête d'avoir-- de- de pas se nourrir avec, dans l'idée.

*Mme. 1:* Ouais.

*Mme. 2:* Mais bon, après ça ne me gêne pas plus que ça dans le sens où, vu qu'on-- vu que c'est un cycle, ça repart à la terre [et tout]. Ça va nourrir la terre donc [du coup] ça va [quoi].

*Mme. 1:* Ouais, ouais vous avez un compost.

*Mme. 2:* [Hum]. Hé ! Tous les deux-là ! Ho !

*Mme. 1:* Et [du coup] c'est- c'est un compost et vous, vous avez votre potager. C'est vraiment votre potager.

*Mme. 2:* Oui.

*Mme. 2:* D'ailleurs, ça fait longtemps qu'on n'est pas allé dedans. Mais bon. Il y a des choses en plus, il y a des physalis tout là, faudrait qu'on les ramasse.

*Mme. 1:* Ok, alors si on prenait une autre photo ? C'était celle-là, oui [du coup] ?

*Mme. 2:* Ouais, [euh] non c'est l'autre qui faut que je commente.

*Mme. 1:* Ouais.

*Mme. 2:* Alors [du coup] potager.

*Mme. 1:* [Rire] ouais.

*Mme. 2:* Alors, je suis en train de me dire que ce n'était pas de la permaculture. Je sais pas si tu veux-- ?

*Mme. 1:* Ouais.

*Mme. 2:* [Euh], très carré je trouve.

*Mme. 1:* Ouais.

*Mme. 2:* Très propre.

*Mme. 2:* Ça donne envie.

*Mme. 1:* Ouais.

*Mme. 2:* Maison.

*Mme. 1:* Ouais.

*Mme. 2:* [Hmm] et je pense que ce type de potager nécessite beaucoup d'entretien.

*Mme. 1:* Ouais ?

*Mme. 2:* Ouais.

*Mme. 1:* Alors, t'as dit que ça donnait envie. Est-ce que tupeux me dire pourquoi t'as exprimé ça ?

*Mme. 2:* [Ben] parce que c'est frais.

*Mme. 1:* Ouais.

*Mme. 2:* Et l'idée du frais c'est propre pour le coup. T'as juste à cueillir puis c'est frais [quoi].

*Mme. 1:* Ouais.

*Mme. 2:* T'as pas à allé chercher comme chez nous dans- dans tout ce qu'il y a. [Euh] [ouais] puis ça à l'air assez avancé. Je sais pas si c'est de la menthe. Non, c'est de la-- une salade je pense. Mais ça à l'air assez avancé déjà ici. Il y a juste à couper pour- pour aller cuisiner. Les poireaux derrière aussi on peut couper un peu le vert, [enfin] le chou aussi un peu, [enfin voilà].

*Mme. 1:* Ouais.

*Mme. 2:* On peut couper [euh], donc c'est prêt [quoi] [en fait]. [Hum].

*Mme. 1:* Est-ce que, [euh]-- alors ce potager ou le tien, c'est un lieu dans lequel tu te sens bien ou non ? Tu fais ça par nécessité de -- ?

*Mme. 2:* Oh non je me sens bien. C'est pas du tout une nécessité.

*Mme. 1:* Ouais.

*Mme. 2:* Parce que le potager on l'a commencé non pas pour manger, d'ailleurs on oubli de manger ce qu'on a dans le potager. Pas grave ! Mais c'est plus parce que c'est un moyen de décompression [en fait]. C'est un moyen de faire autre chose que d'aller en cours ou d'aller au boulot.

*Mme. 1:* Ouais.

*Mme. 2:* Donc, c'est vraiment un moment où on fait quelque chose tous les deux et qu'on a un retour à la terre aussi qui est intéressant je trouve.

*Mme. 1:* Ouais.

*Mme. 2:* Ça permet de- [ben] de prendre son temps.

*Mme. 1:* D'accord.

*Mme. 1:* Donc [euh], la raison première pour laquelle vous avez fait un potager c'est pas pour vous nourrir, c'est [euh] plus pour pouvoir travailler la terre et -- ?

*Mme. 2:* Oui, puis moi peut-être aussi c'est un peu le truc d'expérience. Expérimenter des graines. C'est comme, [ben] la pastèque ou le melon [quoi]. Des choses où on nous disait que ça sert à rien de planter. Donc, on a quand même planté et au final on a eu une grosse pastèque et super bonne [quoi].

*Mme. 1:* Ah oui ?

*Mme. 2:* Au final ça a pris [quoi] ! Et on dit aux gens, [ben] [euh] essayez de planter quand même parce que ça dépend de chaque année, ça dépend de beaucoup de choses au final.

*Mme. 1:* Ouais.

*Mme. 2:* Donc, plantez et puis vous verrez. Ça coûte rien de mettre des graines en terre.

*Mme. 1:* [Ben] ouais.

*Mme. 2:* Et [euh] [ouais] nous c'est plutôt-- parce que [en fait] moi j'aime bien voir grandir, donc tous les jours, j'allais faire-- là maintenant plus mais, tous les jours [euh] [ben], printemps, été, automne, je vais de-- je vais dans le jardin, je regarde chaque plant s'il a poussé ou pas [rire]. Et- et au final, je le mange même pas quand il a-- quand il est atteint je dis "Oh bah ! c'est cool, il a grandi, super ! On a produit ça" [rire] [et voilà]. Ouais, c'est plus ça. Mais ouais.

*Mme. 1:* C'est plus le plaisir de faire pousser que de le manger finalement ?

*Mme. 2:* Ouais je pense.

*Mme. 1:* Et [euh] [du coup], est-ce que ça- ça vous arrive quand de manger des produits que vous avez produit [euh] ?

*Mme. 2:* Oui, oui.

*Mme. 1:* Quand même

*Mme. 2:* Je sais pas si tu as vu, mais il y a beaucoup de physalis dans le jardin parce qu'on a un amateur ici.

*Mme. 1:* Ouais.

*Mme. 2:* Et [du coup] [ben] l'amateur, il allait pendant une période tous les jours quand même ramasser les physalis.

*Mme. 1:* D'accord.

*Mme. 2:* Voire même les limiers de mûres.

*Mme. 1:* Ok.

*Mme. 2:* Donc, non, non. On mange quand même. Après les carottes elles sont prêtes, par exemple, les carottes dans le jardin là, on n'en a pas énormément, mais elles sont prêtes depuis [euh] septembre.

*Mme. 1:* D'accord.

*Mme. 2:* Elles sont toujours plantées là. Donc [euh] quand on est motivé un jour, on va prendre, mais on sait qu'elles sont prêtes, on sait qu'elles nous attendent, mais on les laisse dedans quand même. On se dit, bon au pire, les vers mangeront et puis nous ont prendra quand on aura envie, on coupera. Ça prend pas tout en général.

*Mme. 1:* Ouais.

*Mme. 2:* Et [euh] [voilà]. [Ben] on n'est pas trop stressé avec ça.

*Mme. 2:* Et puis, il y a un truc aussi je sais pas si c'est important, mais le fait que ce soit un potager en ville, je pense que ça me freine aussi pour les manger.

*Mme. 1:* D'accord.

*Mme. 2:* Parce que [en fait], je vois au niveau [euh], j'avais planté des- des concombres, des courgettes [et tout] là-bas.

*Mme. 1:* Ouais.

*Mme. 2:* Et [en fait], il y a une sorte de résidu bizarre qui se met sur les feuilles et comme si c'était de la pollution [en fait].

*Mme. 1:* D'accord.

*Mme. 2:* Et [euh], et vraiment, ça me-- [enfin], je me dis, on sort, je sens les pots d'échappements.

*Mme. 1:* Ouais.

*Mme. 2:* Donc je me dis ça veut dire le plant il respire [en fait] ça et nous on va manger le plant qui a respiré le pot d'échappement toute sa vie [quoi].

*Mme. 1:* Ouais.

*Mme. 2:* Donc [du coup], je pense que ça me pose un petit problème aussi.

*Mme. 1:* D'accord.

*Mme. 2:* Là-dessus. Et [du coup] je suis pas-- Même si c'est mes légumes, et que je suis contente de manger mes légumes quand on a des fraises [et tout] [quoi] [enfin voilà].

*Mme. 1:* Ouais.

*Mme. 2:* Mais [euh], ouais, j'ai toujours ce truc de quand même [ben] c'est pas-- c'est pas clean [quoi].

*Mme. 1:* Ouais.

*Mme. 2:* C'est pas nickel [quoi]. Et normalement c'est la dernière année.

*Mme. 1:* Ah oui ? D'ici ?

*Mme. 2:* [Ouais] [ben] là on a-- on achète un terrain. Là, on signe l'acte de vente, vendredi prochain.

*Mme. 1:* Ok. Un terrain carrément ?

*Mme. 2:* Ouais, ouais, ouais. On va-- on va vivre dedans avec une tiny house et on part [euh] vers Saint-Sauveur à côté des Monts d'Arrée.

*Mme. 1:* Ok.

*Mme. 2:* Donc normalement là-bas, l'air sera mieux.

*Mme. 1:* Ouais.

*Mme. 2:* Normalement !

*Mme. 1:* Oui il y a de quoi là-- oui.

*Mme. 2:* Puis on aura un petit ruisseau [et tout]. Donc on pourra faire des trucs pas mal. On va faire des marres [et tout] dedans.

*Mme. 1:* Trop bien.

*Mme. 2:* Ouais. Donc normalement, on est censé manger plus de légumes.

*Mme. 1:* [Rire] de votre potager ?

*Mme. 2:* [Ouais], normalement.

*Mme. 1:* Et [euh]--

*Mme. 2:* Aussi un truc, c'est que à Brest on a l'abon-- l'abondance de tout.

*Mme. 1:* Ouais.

*Mme. 2:* Je pense. [Du coup], quand on n'est pas sûr ou qu'on a pas trop envie de manger un truc, [ben] [en fait] on peut aller manger autre chose facilement ailleurs. Et c'est ce que je disais à [prénom], c'est que le fait d'être à Saint-Sauveur--

*Mme. 1:* Ouais.

*Mme. 2:* Je pense qu'on aura moins le choix et [du coup] on sera vraiment obligé de- [ben] de suivre un rythme différent [quoi].

*Mme. 1:* Ouais.

*Mme. 2:* Là, par exemple, hier en rentrant, on s'est arrêté au Burger King là pour prendre un sandwich. Et [euh], et je- [ben] je me disais je-- en plus j'y pensais hier. Je me dis ça on le fait parce qu'on est à Brest.

*Mme. 1:* Ouais.

*Mme. 2:* Mais on ne va pas le faire quand on sera à Saint-Sauveur.

*Mme. 1:* Ouais.

*Mme. 1:* Ouais, il y a la facilité en ville [euh] de s'ap-- s'approvisionner à droite à gauche [euh].

*Mme. 2:* [Ben] oui, y'a de tout de [en fait] de suite.

*Mme. 1:* Ouais, ouais.

*Mme. 1:* Et [euh], quand vous mangez des produits que vous avez produit, [euh] vous les trouvez comment ?

*Mme. 2:* Toujours meilleurs qu'ailleurs.

*Mme. 1:* Toujours meilleurs qu'ailleurs ?

*Mme. 2:* [Hum]. Et on parlait de ça aussi avec des autres personnes qui ont des potagers et qui ont l'impression que leurs patates sont vraiment les meilleures [quoi].

*Mme. 1:* Ouais.

*Mme. 2:* Alors que nous, les nôtres aussi étaient les meilleures. [Rire] Je leur ai dit dit, à mon avis c'est juste un-- c'est un biais cognitif [quoi]. On a vraiment l'impression de manger un truc de meilleur alors que pas du tout. Pour moi, c'est pareil qu'ailleurs.

*Mme. 1:* Ouais.

*Mme. 2:* C'est juste que, vu qu'on a mis un plus de sueur dedans [ben]-- parce que faut les ramasser les patates.

*Mme. 1:* Ben ouais.

*Mme. 2:* À la main, c'est pas ouf ! [Rire]

*Mme. 2:* Mais ouais, donc c'est pour ça.

*Mme. 1:* [Ouais], c'est parce que c'est vous qui les avez [euh] produits. Le travail que vous avez fourni qui font que [euh]--

*Mme. 2:* [Ben] qu'on les trouve meilleurs.

*Mme. 1:* Ouais.

*Mme. 2:* Bon, après pas tout quand même. Il y a des trucs où je me dis bon ça a un peu foiré là-dessus quand même. Mais-- non mais c'est vrai la majorité [bah] les- les physalis quand ils mangent, ils les trouvent wow largement meilleures que toutes les autres qu'il a mangé [rire]. Et ça me fait rire [quoi] parce que je sais très bien que c'est faux, que c'est un biais [en fait] et que [du coup].

*Mme. 1:* Ouais et alors si on faisait une expérience avec des-- où on ne dit pas la provenance, il faut dire laquelle est la meilleure, il y a des chances que ça--

*Mme. 2:* [Ben] non, mais c'est ça ! [Bah] oui. Non mais c'est sûr. En plus, c'est que moi, je-- [enfin], je prends vraiment le temps de déguster quand je prends une fraise. Je la découpe.

*Mme. 1:* D'accord.

*Mme. 2:* Je prends vraiment le temps de goûter. Je sais qu'en réalité, les fraises que j'avais dans mon appartement étaient meilleures que celles ici.

*Mme. 1:* D'accord.

*Mme. 2:* Parce que c'était une autre variété et j'arrive pas à retrouver la variété d'ailleurs et ça m'énerve. Mais [euh] je le-- je le sais, je les trouve super bonnes ici, mais moi je sais.

*Mme. 1:* Ouais.

*Mme. 2:* Que [du coup] dans mon autre appart c'était meilleur. [Rire] Et lui, c'est "Ah non, j'te jure, Ah non, non. Elles sont beaucoup plus sucrées [et tout]". [Rire] n'importe quoi ! [Rire].

*Mme. 1:* Ouais [rire].

*Mme. 2:* Ça me fait rire ! Et puis même, c'est marrant par ce que les personnes extérieures qui viennent, quand j'ai des potes qui viennent et qui-- parce que là, j'avais pas mal de potes enceintes [alors du coup] les fraises, ça y allait.

*Mme. 1:* Ouais.

*Mme. 2:* Et [du coup] à chaque fois, elles me demandaient d'allait prendre des fraises. Et quand elles avaient mangé les fraises, elles étaient là en mode "Wow ! Mais qu'est-ce qu'elles sont bonnes." [Rire] Ça c'est juste parce que tu les as ramassées chez ta pote, c'est tout. [Rire] [Du coup], ça me fait rire.

*Mme. 1:* [Rire].

*Mme. 2:* Je trouve ça drôle.

*Mme. 1:* Ouais.

*Mme. 1:* Ouais donc, il y a un plaisir autre de manger quand ça vient de- du jardin dans lequel on va cueillir ?

*Mme. 2:* Ouais je pense que le fait de cueillir aussi, je pense que ça joue, ça influence. Parce que quand tu-- quand on te donne tout prêt dans la barquette, c'est différent que si toi tu vas cueillir, les choisir limite dans le paquet, tu vois ?.

*Mme. 1:* Ouais.

*Mme. 2:* [Enfin] c'est différent je trouve. Et puis ça te demande un petit effort qui fait que [du coup], je pense il y a le besoin de récompense derrière [et tout] [enfin].

*Mme. 1:* Ouais.

*Mme. 2:* Je pense qu'il y a un truc [euh] psychologique dedans.

*Mme. 1:* Ben oui, oui, oui. Carrément. Et [euh], est-ce que ça t'arrives de jeter les produits que t'avais cueilli dans ton potager ?

*Mme. 2:* Oh oui, oui ! Plus souvent même.

*Mme. 1:* Plus souvent ?

*Mme. 2:* Parce que [du coup] ici, vu qu'il y a aucun traitement et rien, on a beaucoup de vers.

*Mme. 1:* D'accord.

*Mme. 2:* Beaucoup de limaces. Quand on nettoie [hein] on a beaucoup de limaces. Là ça va mais-- Ouais, donc on a les radis, par exemple, c'est-- une fois sur deux, c'est une hécatombe.

*Mme. 2:* D'accord.

*Mme. 2:* Donc [en fait] une fois-- si, [en fait] je pense, si on en a-- quand ils sont prêts, on a deux semaines pour les récolter.

*Mme. 1:* D'accord.

*Mme. 2:* Si on récolte pas, [ben] [du coup] les autres animaux arrivent et les mangent.

*Mme. 1:* Oui.

*Mme. 2:* Et si tu récoltes dans les deux semaines, ils sont nickels. Ils sont beaux [et tout] franchement nickel. Mais si jamais tu dépasses les deux semaines, [ben] alors là, c'est mort [quoi].

*Mme. 1:* Ouais.

*Mme. 2:* Alors là, t'as des vers partout dedans, [enfin].

*Mme. 1:* Ouais.

*Mme. 2:* Et nous, vu qu'on ne fait pas attention --

*Mme. 1:* Ouais.

*Mme. 2:* [Ben] [du coup] quand on a un apéro on va aller, "go prendre des radis dans le jardin" et puis non [en fait].

*Mme. 1:* [Rire].

*Mme. 2:* Et voilà.

*Mme. 1:* Et qu'est-ce que ça vous fait de -- toi ou ton conjoint, de jeter des produits [euh] -- ?

*Mme. 2:* [Ben] c'est un peu plus chiant parce que tu pensais vraiment-- surtout quand tu vas les chercher pour une occasion précise.

*Mme. 2:* Ouais.

*Mme. 2:* Tu dis "Wow ! ça va être trop bien" [et tout]. Tu vas les chercher mais là [en fait] t'as la déception de "je les voulais vraiment cette fois-ci [en fait] !" Tu vois ? [rire].

*Mme. 1:* Ouais.

*Mme. 2:* C'est pas quelque chose comme ça. [Enfin] y'a --ouais c'était plus une envie [euh]. J'étais sûre de moi que je les voulais [quoi] [rire].

*Mme. 1:* Ouais.

*Mme. 2:* Et [du coup] quand tu vois que les vers les ont mangés avant toi. Là, je me dis "putain, j'aurai dû les ramasser plus tôt" [rire].

*Mme. 2:* Ouais

*Mme. 2:* [En fait] mais bon. Après l'idée de jeter me pose pas de problèmes non plus dans- dans l'abs-- parce que ça va dans le composteur [et voilà]. Mais c'est plus l'idée de ne pas avoir pu les gouter [en fait]. Si j'en avais eu [euh], je sais pas la moitié ou un quart, j'aurai été contente dans l'idée.

*Mme. 1:* Ouais.

*Mme. 2:* C'est pas-- c'est pas une question de- de tout manger ou pas, c'est plus sur le fait de au moins goûter ce que t'as produit [quoi].

*Mme. 1:* Oui.

*Mme. 2:* Qui est plus gênant. Mais il y a plein de chose qu'on n'a pas pu manger parce que [ben], on a laissé trop longtemps et [du coup] les vers ont mangé à notre place.

*Mme. 1:* [Rire] Oui. Et [euh]-- donc là, c'est parce que vous n'avez pas ramassé à temps. Et [euh] est-ce que ça t'arrive de-- des fois, de jeter un produit que t'as ramassé et qui est resté stocké [euh] ici-- [enfin] chez toi ?

*Mme. 2:* Oui.

*Mme. 1:* Mais pas mangé à temps ou -- ?

*Mme. 2:* [Hum].

*Mme. 1:* Et là c'est-- t'éprouves quoi ? C'est pareil ou-- ?

*Mme. 2:* Je me dis "merde, on aurait dû le manger !"

*Mme. 1:* Ouais, c'est pas on aurait du le ramasser c'est on aurait du le manger ?

*Mme. 2:* C'est ça ! Ouais c'est ça. Je-- [en fait] comme on n'a pas beaucoup le temps de cuisiner.

*Mme. 1:* Ouais.

*Mme. 2:* [Ben] je prends je me dis comme ça je me dis je vais cuisiner un truc trop bon, j'ai le plat en tête [et tout], et [en fait], en général, quand je fais ça c'est des plats qui demandent pas mal de préparation.

*Mme. 1:* Ouais.

*Mme. 2:* Et [en fait], j'ai jamais le temps de faire.

*Mme. 1:* Ouais.

*Mme. 2:* Donc, ça reste là. Je me dis c'est bon, semaine prochaine ça sera bon. Mais quand tu comptes en semaine,

*Mme. 1:* Oui ça--

*Mme. 2:* [Ben] il y a pas beaucoup de délais [en fait]. [Du coup], c'est compliqué.

*Mme. 1:* Ok.

*Mme. 2:* C'est comme le- le potiron là, qui est là. Ça vient du jardin de mon grand-père et ça va faire bientôt un mois qu'il est là.

*Mme. 1:* Oui.

*Mme. 2:* Et faut qu'on fasse de la soupe. On a fait une soupe l'autre jour avec nos produits du jardin, les courges du jardin là pour les faire justement. Mais là faut vraiment qu'on fasse celui-là. Bon là ça se conserve ça va.

*Mme. 1:* C'est-- ouais t'as la chance que ça se conserve pas mal.

*Mme. 2:* Ouais, faut pas que j'oublie trop longtemps encore [quoi].

*Mme. 1:* Et [du coup] tu récupères des fois des choses du potager de [euh] --

*Mme. 2:* De mes grands-parents, de la famille éloignée aussi qui sont sur [inaudible 00:21:46] pas mal de choses. Donc, on récupère. Mais à chaque fois qu'on va là-bas, ils me disent "Alors, qu'est-ce que tu vas faire à manger ce soir ?" Et [du coup] et [ben] je fais mon --

*Mme. 1:* Tu te sers.

*Mme. 2:* Ouais, [rire] mes courses ! [rire]

*Mme. 1:* Ouais, pas mal

*Mme. 2:* Voilà, parce que'eux, ils ont des chose par exemple, les oignons. Nous, on a eu des oignons, mais [en fait] on n'a pas compris, vu que c'est la première année, on n'a pas compris le développement de l'oignon.

*Mme. 1:* D'accord.

*Mme. 2:* [En fait], on savait pas que ça allait prendre autant de temps pour se développer.

*Mme. 1:* Ouais.

*Mme. 2:* Parce que ça prend presque un an au final.

*Mme. 1:* D'accord.

*Mme. 2:* Et quand on voyait [ben], que du vert qui sortiat, on s'est dit [ben] non, ça a pas fonctionné [et tout]. Donc on a mangé tout le vert, donc, ça on était bon [hein], on a bien tenu [et tout]. On a fait tous nos plats avec le vert de l'oignon.

*Mme. 1:* Ouais.

*Mme. 2:* Et [en fait], quand on a voulu planter autre chose à la place et qu'on les a déterrés, on a vu des gros oignons comme ça ! Genre, rose [et tout] genre trop beau. Mais [euh] [rire], on allait le jeter [en fait]. Et on a fait ça, on en a donné à des potes [et tout] et ont les a laissés en terre [du coup]. Bon, il y en a qui étaient déjà morts. Et [euh], et quand on a voulu les reprendre, [ben] [en fait] c'était passé [en fait] on n'avait tellement pas vu. [En fait] parce que là, on connaissait pas du tout le développement de l'oignon.

*Mme. 1:* Ouais.

*Mme. 2:* Donc c'est--

*Mme. 1:* Vous n'avez pas [euh] eu la curiosité à un moment de regarder c'est que --

*Mme. 2:* [Ben] [en fait] on a fait, sauf qu'à chaque fois, ça fonctionnait pas [du coup]-- parce qu'on a regardé sur l'équivalent de six mois [en fait].

*Mme. 1:* Ouais.

*Mme. 2:* Et au bout de six mois, quand on voyait qu'on avait beau déterré, ça faisait rien, [ben] on s'est dit [ben] [en fait] il y a eu une erreur de-- c'était pas dans un bon endroit [et tout] alors qu' [en fait] c'est juste que c'était pas prêt.

*Mme. 1:* Ouais [rire].

*Mme. 2:* Et [du coup] ça a commencé à pousser après six mois. Donc, quand on regardait les pieds, [voilà] [rire]. Mais c'est bien, on a appris maintenant.

*Mme. 1:* Oui [ben] oui, oui, oui !

*Mme. 2:* [Rire]

*Mme. 1:* C'est ça !

*Mme. 2:* Mais la première année c'est drôle.

*Mme. 1:* [Rire]

*Mme. 2:* Il y a des trucs [euh] --

*Mme. 1:* Oui, il y a des petites découvertes.

*Mme. 2:* Ouais, c'est ça.

*Mme. 1:* Et [euh], quand tu [euh] -- les produits que tu as des potagers de ta famille, tu les trouves comment ? Est-ce qu'ils sont aussi bons que les tiens ?

*Mme. 2:* [Rire] Non ! Ils sont-- [Rire]. Non, ça dépend ouais. [Euh] parce que je sais que mon grand-père c'est souvent, concombres, tomates, potirons.

*Mme. 1:* Oui.

*Mme. 2:* Courgettes. Et [en fait] pour moi, c'est comme la-- comme la-- le marché.

*Mme. 1:* Ok.

*Mme. 2:* Chez le producteur tu vois ? [Rire]

*Mme. 1:* Ouais, d'accord.

*Mme. 2:* [rire], c'est au même niveau. [En fait] non je suis pas [euh] --

*Mme. 2:* Et mon copain non plus pour le coup.

*Mme. 1:* Là [bah oui], ce n'est pas les meilleurs de toute la terre [quoi].

*Mme. 2:* Non [rire] Non. Par contre, les tomates-- les tomates elles étaient vraiment bonnes parce que c'est vrai, moi aussi des fois j'ai [rire] un truc comme ça, et [euh], et ouais [en fait] les tomates ont les avaient oubliées aussi et [du coup], elles étaient hyper sucrées [en fait]. Elles étaient toujours bonnes, mais très, très, très sucrés. Bon, mais là je pense qu'elles étaient vraiment bonnes.

*Mme. 1:* Ouais, ouais.

*Mme. 2:* Mais [euh] ouais. Parce qu'après ça dépend [en fait] quand tu ramasses, je sais que mon grand-père, dès que c'est rouge, le premier jour, il ramasse.

*Mme. 1:* D'accord.

*Mme. 2:* PArce que [du coup] [ben] c'est bien de laisser encore au soleil mûrir [en fait].

*Mme. 1:* Ouais.

*Mme. 2:* Je pense c'est pour ça aussi et je pense que les producteurs, ils ont tendance à ramasser tous de suite.

*Mme. 1:* Ouais.

*Mme. 2:* Parce que le temps que les gens prennent, qui les mettent dans chez eux, qui les utilisent [enfin].

*Mme. 1:* Ouais, oui c'est pas pareil que pouvoir la cueillir juste là [quoi].

*Mme. 2:* [Ben] c'est ça. Donc moi dès que j'allais au travail, que je devais me faire une salade, j'avais ma salade de faite et juste avant de partir j'allais dans la serre au dernier moment pour prendre toutes les tomates cerise, et je mettais dans-- je remplissais la boîte [et voilà].

*Mme. 1:* Et si ça t'arrives de perdre des produits qui viennent de-- des potagers de- de ta famille, est-ce que-- comment tu-- comment tu le ressens [euh]-- est-ce que c'est pareil ou -- ?

*Mme. 2:* [Ben] ça m'embête aussi, [enfin] ça m'embête un peu [quoi]. Je me dis "Oh ! bah, c'est con, mon grand-père m'a quand même donné avec tout son coeur" [quoi]. Tout l'amour qu'il avait son-- sa courgette, et là je l'ai jeté alors c'est vraiment triste. [Rire]. Non, en vrai, c'est un peu [euh]-- un peu ça en moins extrême qui se passe dans ma tête. Je me dis ouais c'est con quand même.

*Mme. 1:* Ouais.

*Mme. 2:* Et puis après, je me dis bon [ben] de toute façon, [enfin], encore une fois ça finira--

*Mme. 1:* Oui.

*Mme. 2:* [Voilà] dans le cycle. Donc ça va, c'est pas comme si je jetais dans une poubelle noire.

*Mme. 1:* Ouais tu penses ce serait-- ?

*Mme. 2:* Ah ouais non parce que là je sais que c'est écrabouillé, et que c'est juste-- ça n'a aucun intérêt [en fait].

*Mme. 1:* Ouais]

*Mme. 2:* C'est pas revalorisé, ça sert à rien. Donc c'est juste du gaspillage pour du- du gaspillage.

*Mme. 1:* Ouais.

*Mme. 2:* Alors que là, je trouve que c'est pas forcément du gaspillage.

*Mme. 1:* Ouais.

*Mme. 2:* Parce que la terre si tu ne la nourris pas, [ben] elle arrêtera de donner, elle sera de plus en plus pauvre. Alors que là justement les produits passés vont permettre de la nourrir. Donc, c'est ce qui va permettre de relancer aussi. [Enfin] moi, je vois comme ça.

*Mme. 1:* Ouais.

*Mme. 2:* [Du coup], je me dis c'est pas du gaspillage non plus [quoi].

*Mme. 1:* Ouais.

*Mme. 2:* C'est juste utiliser les fruits autrement.

*Mme. 1:* Ok.

*Mme. 2:* C'est- c'est une revalorisation [quoi].

*Mme. 1:* Ouais c'est [euh]-- tu penses [du coup] soit ils sont mangés, soit-- [enfin] soit ils nourrissent les humains, soit ils nourrissent [euh] la terre [quoi].

*Mme. 2:* Ouais c'est pour ça je pense qu'on ne se stresse pas non plus.

*Mme. 1:* [Ouais].

*Mme. 2:* Mais quand on va être à Saint-Sauveur, et que la-- les magasins seront beaucoup plus loin.

*Mme. 1:* Ouais.

*Mme. 2:* Là je pense qu'on va voir les- les choses autrement. [Rire]

*Mme. 1:* Ouais.

*Mme. 2:* Ouais je pense.

*Mme. 1:* D'accord. Alors, une autre, [euh]--

*Mme. 2:* Carte.

*Mme. 1:* Ouais.

*Mme. 2:* [Hum] [hum] ! Magasin.

*Mme. 1:* [Hum] [hum].

*Mme. 2:* Alors, je pense à bio en premier et je pense que c'est du bio parce qu'il y a beaucoup de vert.

*Mme. 1:* Ouais.

*Mme. 2:* Mais quand je vois un truc comme ça qui a l'air trop dans le vert [euh], trop- trop, je me donne un style bio, [ben] je me dis est-ce que c'est pas une grosse arnaque derrière [quoi] ?

*Mme. 1:* Ouais.

*Mme. 2:* Que les supermarchés ont l'habitude de faire croire.

*Mme. 1:* Ouais.

*Mme. 2:* C'est toujours ça et puis je sais que le bio ça veut rien dire aussi.

*Mme. 1:* Ouais.

*Mme. 2:* Avant, je croyais beaucoup au bio et maintenant quand j'ai vu que c'était juste une norme Européenne et qu'au final, le bio en fonction pays [ben] t'as des produits [euh]-- si t'as du bio d'Espagne [eh ben] t'as des produits qu'ils mettent en Espagne et qui sont interdits en France et pourtant c'est mis comme du bio.

*Mme. 1:* Ouais.

*Mme. 2:* [Ben] [du coup], je trouve ça illogique [en fait] et je fais plus trop confiance au bio et mon copain non plus d'ailleurs. Quand on voit [euh] noix de coco bio.

*Mme. 1:* Ouais.

*Mme. 2:* [Ben] à un moment, il faut arrêter de se foutre de la gueule du monde [quoi]. [Enfin] bon, je sais pas si t'as compté le prix de la viande dans le bio mais-- [enfin] de--

*Mme. 1:* Ouais

*Mme. 2:* [Voilà] je trouve, y'a des choses comme ça que je trouve un peu [euh] hallucinantes.

*Mme. 1:* Ouais, tu préfères privilégier le local que le bio avec le prix [euh] -- ?

*Mme. 2:* [Hum]. Le bio-- le bio emballé aussi.

*Mme. 1:* Ouais.

*Mme. 2:* C'est intéressant le bio emballé ! [Rire]

*Mme. 1:* [Rire].

*Mme. 2:* Genre Lidl qui fait du bio emballé.

*Mme. 1:* Ouais.

*Mme. 2:* [Euh] --

*Mme. 1:* Ouais mais pas que-- même les [euh]-- Leclerc et Carrefour là.

*Mme. 2:* Oui, oui [voilà] mais c'est parce que Lidl j'avais vu l'autre jour un- un concombre bio mais tout emballé de plastique.

*Mme. 1:* Ouais.

*Mme. 2:* Mais un concombre !

*Mme. 1:* Un concom-- ouais, ouais. Mais là, je crois qu'ils-- mais [euh] pendant un moment, je faisais pas mal de drive et [euh]- et quand je prenais genre un concombre [et tout] il était [euh] en ce-- [euh] ouais, c'était au Carrefour [euh] --

*Mme. 2:* Ouais.

*Mme. 1:* Donc au Carrefour, il était en- en- en dans du cellophane [quoi]. [Enfin] emballé. Et- et c'est que les produits bio quoi limite qui étaient emballés. Limite si j'avais pris un concombre pas bio, il aurait pas été emballé. Faut choisir entre le plastique ou le-- [rire]

*Mme. 2:* Oh ! merde [rire] Oh putain, le monde avance, c'est beau ! [Rire]

*Mme. 2:* C'est ça ! ouais, ouais, ouais, ils font-- c'est [euh], il y a l'excuse de pas que les produits se-- que le bio soit pas contaminé par les produits non-bio.

*Mme. 2:* [Hum].

*Mme. 2:* C'est ça l'excuse [euh] première de-- des- des marques bio.

*Mme. 2:* Ok, donc il vaut mieux que ce soit contaminé par du plastique--

*Mme. 1:* [Voilà] que--

*Mme. 2:* Ce que le plastique rejette.

*Mme. 1:* Ouais que--

*Mme. 2:* Que le non-bio

*Mme. 1:* Ouais.

*Mme. 2:* Qui peut être à côté.

*Mme. 1:* Surtout des produits comme le concombre [euh], ça a pas de peau [et tout] [quoi], [enfin] ce n'est pas résistant du tout. [Rire].

*Mme. 2:* C'est ça. Ouais mais c'est ça. Puis même, tu peux pas le laver, tu peux rien faire [en fait].

*Mme. 1:* [Ben] c'est ça.

*Mme. 2:* [Hum], ouais je comprends.

*Mme. 1:* Bah oui, c'est pour ça. [Rire].

*Mme. 2:* Ils ont dû se casser la tête pour trouver ça. [Euh] sinon, [ben] ça me fait penser à de la bien-- [enfin] en voyant ça, c'est bienveillant.

*Mme. 1:* Ouais.

*Mme. 2:* Ce qui me vient en tête. Peut-être cher aussi.

*Mme. 1:* D'accord.

*Mme. 2:* Donc [voilà], j'ai pas grand-chose.

*Mme. 1:* Ouais, bienveillant, pourquoi t'as pensé à ça ?

*Mme. 2:* [Ben] parce que ça fait un peu [euh]-- Hé ! Qu'est-ce que vous faites là ? Oh !

*Mme. 1:* [Rire]

*Mme. 2:* Arrête toi ! Hé, tu te calmes ? Ça fait un peu [euh], [ben] le monsieur, il a l'air gentil avec-- leorange, c'est chaleureux.

*Mme. 1:* Ouais.

*Mme. 2:* C'est une couleur plutôt- plutôt chaleureuse. Le vert, c'est un peu, [voilà], la nature, ça donne confiance tout ça. Donc, si tu mélanges les deux ça me donne bienveillance.

*Mme. 1:* Ouais.

*Mme. 2:* C'est un peu ça [rire], dans idée je pense, c'est ce qui s'est passé dans ma tête.

*Mme. 1:* [Ben] c'est les couleurs qui t'ont évoquées [euh] --

*Mme. 1:* Et cher c'est-- t'as l'impression que ?

*Mme. 2:* [Ben] ça me fait penser à la Biocoop.

*Mme. 1:* Ouais.

*Mme. 2:* Ou alors à Mon panier bio là qui est vers Saint-Renan [et tout] là.

*Mme. 1:* Ouais.

*Mme. 2:* Et [euh]- et je trouve que c'est une grosse arnaque ce magasin par exemple.

*Mme. 1:* Ouais.

*Mme. 2:* C'est les prix-là, je suis allée une fois dedans, je trouve que c'est hallu-- hallucina ment cher.

*Mme. 1:* D'accord.

*Mme. 2:* Je trouve que c'est exagéré de fou et [euh] je me demande comment ils font [en fait] pour-- parce que je veux bien croire que quand tu fais bio-- bon [en fait], je crois-- j'y crois même plus trop mais quand tu fais du bio, tu peux avoir plus de perte [et tout].

*Mme. 1:* Ouais.

*Mme. 2:* Donc t'augmentes un peu-- un peu des prix mais [euh] quand je vois le bio qui peut avoir au marché et le bio qui a dans certain magasin, je me dis c'est pas possible [quoi].

*Mme. 1:* Ouais.

*Mme. 2:* [Enfin]-- et puis co-- comment tu veux démocratiser le bio si on garde un prix aussi élevé [en fait] ?

*Mme. 1:* Ouais.

*Mme. 2:* [Ben] moi c'est ça que je comprends pas trop. Alors que quand je vois la permaculture [et tout].

*Mme. 1:* Ouais.

*Mme. 2:* Y'a vraiment des méthodes qui permettent de passer moins de temps dans les champs. Donc au final, l'agriculteur, il va devoir passer plein de fois pour mettre ses produits [et tout] mais il y aura beaucoup moins de temps a passé dans les champs et dans les-- dans la production. Donc je me dis qu'il y a quand même des- des avantages dont on parle pas.

*Mme. 1:* Ouais.

*Mme. 2:* Et des inconvénients. Parce qu'on parle que des inconvénients. Oui [alors] [du coup] y a des pertes de légume parce que y'a les petits vers qui vont les manger.

*Mme. 1:* Ouais.

*Mme. 2:* Alors que [bah] pas forcément [en fait]. Ça dépend de comment tu cultures.

*Mme. 1:* Ouais.

*Mme. 2:* Et par exemple pour-- parce que mon copain il regarde des documentaires là-dessus, sur les paysans [et tout] et [euh] y'avait un mec qui testait le- le fait de retourner la terre pour-- par rapport aux graines, pour pas que les graines sortent. À la place de mettre genre du produit comme du-- comme-- comment ça s'appelle le truc qu'il brûle là ? Connu là ? [Hum] glyphosate [et tout] là. Au lieu de mettredu glyphosate. [Ben] [en fait], ils disent juste tu retournes ta terre. Parce que [du coup] [euh] les graines tombent au fond.

*Mme. 1:* Ouais.

*Mme. 2:* Donc, elles vont pas pousser.

*Mme. 1:* Ouais.

*Mme. 2:* Et [euh], et y'en a-- donc, il y avait un mec pro glypho [et tout] et qui disait "Oui mais t'as vu le temps que tu perds entre passer la charrue sur tout ton champ ou alors prendre un gros truc qui met du glypho partout d'un coup ?"

*Mme. 1:* Ouais.

*Mme. 2:* [Ben] ouais mais t'es là [euh] oui.

*Mme. 1:* Mais bon. [Rire].

*Mme. 2:* Ok, là t'es en train de jouer sur une journée à la place de deux heures. Bon, ça va.

*Mme. 1:* Ouais.

*Mme. 2:* Genre--

*Mme. 1:* Ouais c'est--

*Mme. 2:* [Voilà], je pense ouais que c'est pas-- [enfin] j'ai plus l'impression que c'est plus un argument de vente des fois.

*Mme. 1:* Ouais.

*Mme. 2:* Plutôt que vraiment une dém-- une vraie démarche [euh] développement durable [quoi].

*Mme. 1:* Ouais.

*Mme. 2:* Qui prend en compte beaucoup plus de chose que juste l'écologie dans l'idée.

*Mme. 1:* [Euh], est-ce que c'est un magasin dans lequel, toi, tu pourrais aller ? Ce genre de magasin ou non ?

*Mme. 2:* Ouais, par curiosité.

*Mme. 1:* Ouais.

*Mme. 2:* Quand je ne connais pas, je m'arrête. Et je me dis que peut-être un jour, je tomberai sur une perle rare.

*Mme. 1:* Ouais ? C'est-à-dire tu entends quoi par une perle rare ?

*Mme. 2:* [Ben] comme Les Bocaux d'Ana pour le coup.

*Mme. 1:* D'accord.

*Mme. 2:* Un petit magasin qui est mignon. Là pour les prix, je trouve que ça va en fonction des produits que tu prends.

*Mme. 1:* Ouais.

*Mme. 2:* Et, [hum], y'a vraiment des trucs un peu originaux [quoi].

*Mme. 1:* Ouais.

*Mme. 2:* Donc moi ça ne me dérange pas de mettre du-- le prix dans les produits originaux [en fait].

*Mme. 1:* Ouais.

*Mme. 2:* [Voilà]. Ou [euh] Day by Day que j'aime bien aussi. J'y vais moins souvent.

*Mme. 1:* Ouais.

*Mme. 2:* Mais [euh] je trouve que ça va. C'est pas hyper cher non plus.

*Mme. 1:* Ouais.

*Mme. 2:* [Voilà].

*Mme. 1:* Les Bocaux d'Ana, ils se mettent à faire des-- ils en faisaient pas avant des- des légumes, je crois ?

*Mme. 2:* [Hum].

*Mme. 1:* Et là, je crois qu'ils commencent à en faire.

*Mme. 2:* [Ben] [en fait], ils font [euh]-- ils ont un partenariat avec des producteurs. Et [du coup] les producteurs amènent les paniers que tu commandes sur internet.

*Mme. 1:* Ouais, ça, c'est les-- c'est le-- [enfin], comme une AMAP. Mais j'ai [euh]-- c'est les Embruns je crois ? Peut-être ? [Euh] je crois. Mais [euh], dans le même genre, je ne sais pas si tu connais, y'a Mister Pépin ?

*Mme. 2:* Ouais, mais j'ai jamais pu m'arrêter là-bas.

*Mme. 1:* Ouais. [Euh], moi non plus j'ai jamais été alors que-- [ben], [enfin], je crois que c'est le même principe un peu-- tu retrouves un peu les mêmes produits je pense que Les Bocaux d'Ana, [enfin] à voir, [euh]. Mais c'est le même principe d'épicerie vrac et eux, ils font les fruits et les légumes Qui sont pareil, [enfin] là du-- des producteurs [euh].

*Mme. 2:* [Hum]. Parce que tu vois Les Bocaux d'Ana et Day by Day ils n'ont pas les mêmes produits.

*Mme. 1:* Ouais.

*Mme. 2:* Et je trouve ça marrant, et Day by Day font plus supermarché je trouve.

*Mme. 1:* Oui, ouais.

*Mme. 2:* Que Les Bocaux d'Ana où tu rentres c'est un peu un truc [euh], [ben] bobo [quoi].

*Mme. 1:* Ouais, on dirait Nature et Découvertes presque. [Rire].

*Mme. 2:* [Ouais], c'est ça. Mais grave, c'est exactement ça ! Et moi j'adore, [enfin], moi j'adore cette ambiance un peu bois, chaleureux, tu vois ? Si on rentre dedans, ça donne envie de tout acheter alors que t'en a pas du tout besoin.

*Mme. 1:* Ouais [Rire].

*Mme. 2:* Et puis c'est cher.

*Mme. 1:* Ouais.

*Mme. 2:* Pour la plupart des produits quand même.

*Mme. 1:* Ouais.

*Mme. 2:* Comme [euh]- comme les masques ou les trucs comme ça, c'est super cher.

*Mme. 1:* Ouais.

*Mme. 2:* Quand je regardais.

*Mme. 2:* Bon [ben], après [ben] les filles-- [enfin] je trouve que c'est un ensemble parce que les filles sont super sympas.

*Mme. 1:* Ouais.

*Mme. 2:* [Enfin], c'est vraiment un truc que tu peux prendre tout ton temps, tu peux rester discuter de toute ta vie avec les- les vendeuses, elles te parlent de toutes leurs vies aussi.

*Mme. 1:* Ouais.

*Mme. 2:* Vraiment, c'est drôle je trouve. J'aime bien. Et puis t'as- t'as du pain, enfin t'as des trucs qui sont bons quand même. [Enfin], c'est bon même. Nous, on a nos pâtes préférées, elles sont là-bas.

*Mme. 2:* D'accord.

*Mme. 2:* C'est les pâtes aux algues. Je sais pas si tu vois ?

*Mme. 1:* D'accord. Ouais je vois, mais j'ai jamais mangé, mais ouais.

*Mme. 2:* Oh celles-là, elles sont méga bonnes de fou.

*Mme. 1:* Ah ouais ?

*Mme. 2:* Ah ouais ! Moi j'avais peur, j'aime pas les algues.

*Mme. 1:* [Ben] ouais.

*Mme. 2:* Je trouve ça fort.

*Mme. 1:* Ouais j'en ai acheté à -- [euh], en poudre là pour de temps en temps essayer de-- c'est plus pour mon organisme que je fais ça que par plaisir de manger. [Rire].

*Mme. 2:* Mais en vrai, goute les pâtes.

*Mme. 1:* Ouais.

*Mme. 2:* Parce que t'as pas-- t'as pas le goût d'algue, vraiment [en fait], c'est juste hyper léger, donc c'est très- très léger et ça donne un tout petit goût aux pâtes, alors que les pâtes n'ont pas de goût.

*Mme. 1:* Ouais.

*Mme. 2:* Normalement, en général. Là, ça donne un léger goût qui fait que genre on peut les manger natures avec un peu de fromage.

*Mme. 1:* Ouais, [ben] oui, toujours dans les pâtes.

*Mme. 2:* [Rire] Et [euh]- et ça passe nickel. En vrai, on a eu un coup de coeur de fou pour ces pâtes. C'est vraiment-- alors que blé noir, il aime pas [et tout] mon copain.

*Mme. 1:* Ouais.

*Mme. 2:* Il est pas habitué. Mais alors, celles-là [euh].

*Mme. 1:* Je vais essayer.

*Mme. 2:* C'est-- et elle me-- [bah] y'a une des vendeuses qui disaient que ces enfants c'est pareil aussi.

*Mme. 1:* Et--

*Mme. 2:* Depuis qu'ils ont goûté les pâtes mauves là, mauves et vertes aux algues ils adorent.

*Mme. 1:* En plus c'est coloré.

*Mme. 2:* Ouais, ils ont des formes [genre] ça fait des petites vagues et c'est mignon.

*Mme. 1:* Ouais. [Rire].

*Mme. 1:* Donc, [euh], ouais, t'es quand même adeptes en soit des- des- des magasins qui-- [rire] -- des pâtes au fromage.

*Mme. 2:* [Rire] tout le monde rigole.

*Mme. 1:* [Rire]. Ouais, des magasins qui proposent des- des produits quand même originaux, mais qui sont dans une, comment dire dans une volonté écoresponsable [euh] ?

*Mme. 2:* Ouais, ah ouais [ben] on adore ça nous, on adore. Dès que-- dès qu'il y a malheureusement, mais en tout cas, quand on voit qu'il y a une foire-- une foire bio à Landerneau [et tout].

*Mme. 1:* Ouais.

*Mme. 1:* Moi, j'adore ça. Dès qu'il y a un truc [euh], foire de l'artisanat ou des trucs comme ça, moi j'adore.

*Mme. 1:* Ouais.

*Mme. 2:* Vraiment- vraiment, j'ai envie de tous prendre et je préfère acheter des trucs débiles.

*Mme. 1:* Ouais.

*Mme. 2:* Dans ces endroits-là.

*Mme. 1:* [Rire] ouais.

*Mme. 2:* Que- que ailleurs et [en fait], même un truc débile, j'en suis trop fière. Parce que je me dis, c'est la personne qui l'a fait à la main [et tout]. Et un truc qui me sert pas [ben] c'est presque une oeuvre d'art au final.

*Mme. 1:* Ouais.

*Mme. 2:* Parce que je me dis c'est hallucinant [quoi].

*Mme. 1:* Ouais.

*Mme. 2:* C'est comme [ben] là, tu vois le tableau qu'il y a derrière toi ?

*Mme. 1:* Ouais.

*Mme. 2:* C'est [euh]-- tu vois Les Bocaux d'Ana ? [Ben] juste avant, t'as une petite dame qui est artiste.

*Mme. 1:* D'accord.

*Mme. 2:* Et elle a son petit truc et en allant aux Bocaux d'Ana, je me suis dit "Oh ! Bah tiens, c'est marrant, j'ai jamais vu ça" [et tout] et [en fait], j'aimais pas forcément ses tableaux.

*Mme. 1:* [Rire].

*Mme. 2:* [Rire] et [enfin], [voilà ], [enfin], si ceux que j'aimais bien mais ils étaient à plus de 1000 balles donc [du coup] --

*Mme. 1:* Ouais.

*Mme. 2:* Mais, [euh], les petits j'aimais pas forcément et genre, vraiment, elle m'a montré toutes ses machines. [Enfin], je suis allée dans- dans l'arrière atelier [et tout] et elle m'a tout montré, limite elle me proposait de faire [et tout] pour tester. [Enfin], c'était hallucinant [quoi].

*Mme. 1:* Ouais.

*Mme. 2:* Et je me dis ça, je n'ai pas envie qu'elle meurt [en fait].

*Mme. 1:* Ouais.

*Mme. 2:* Donc, [du coup], j'ai pris un tableau.

*Mme. 1:* Ouais.

*Mme. 2:* En me disant, [voilà], je vais essayer de participer à- à sa vie [en fait]. Au fait, qu'il faut que-- faut que ça vive les choses comme ça parce que c'est- c'est vraiment dans l'humain [et tout]. [Enfin], c'est tout ce qu'il faut je trouve.

*Mme. 1:* Ouais. Carrément.

*Mme. 1:* C'est faire perdurer des- des pratiques [euh] artisanales ou des petits commerçants ou [euh] toute initiative [euh] éco [euh] --

*Mme. 2:* Parce que si elle avait été conne, d'un autre côté.

*Mme. 1:* [Rire], ouais.

*Mme. 2:* J'aurai pas pris, [Ben] oui, mais c'est ça. Même si-- même si j'avais trouvé super beau [euh], le magasin, même si y'avait des toiles qui m'auraient plu [et tout], si elle avait été conne, j'aurai rien pris.

*Mme. 1:* Ouais.

*Mme. 2:* Donc c'est vraiment un côté humain aussi. Je me dis pour [euh]-- parce que moi, je travaille pas mal sur l'i-- sur l'isolement social.

*Mme. 1:* D'accord.

*Mme. 2:* Hé, Ho ! [Rire]. Sur l'isolement social. [Ben] [euh], quand je vois des petits commerces comme ça, je me dis, c'est- c'est primordial qu'ils subsistent [en fait], parce que c'est-- quand je la voyais sortir après, parce que j'étais derrière-- dans la voiture-- Tu veux venir ? Tu veux venir ? Non, t'en fou ! Je la voy-- [enfin] [du coup], je la voyais de la voiture et elle allait parler avec des petites mamies qui étaient à côté des immeubles. [Enfin], je me dis c'ets- c'est ça qui est cool [en fait].

*Mme. 1:* Ouais.

*Mme. 2:* Même là, j'ai un collier, ça vient d'un- un peu plus haut, il y a une créatrice de bijoux là qui fait des trucs en pierre [et tout]. Et [euh]- et je voulais me prendre un bijou et j'allais aller à Cache-Cache Bonobo là.

*Mme. 1:* Ouais.

*Mme. 2:* Pour voir. Je me suis dit, faut que t'arrêtes.

*Mme. 1:* Ouais.

*Mme. 2:* Faut que t'arrives à couper avec ces trucs-là.

*Mme. 1:* Ouais.

*Mme. 2:* [Du coup], je me-- je me suis dit je vais trouver un truc de créateur. Et je vais aller voir. Bon, je vais mettre un peu plus cher. Et encore, au final, c'est pas --

*Mme. 1:* Ouais.

*Mme. 2:* C'est pas excessif parce que les trucs de Bo-- de Bonobo [et tout], ils rouillent [en fait].

*Mme. 1:* Ouais.

*Mme. 2:* Moi, j'ai une peau assez acide et [du coup] ça rouille.

*Mme. 1:* Ouais.

*Mme. 2:* Et [euh], et là [ben] là je vais sous la douche [et tout]. J'ai payé 55 euros.

*Mme. 1:* Ouais.

*Mme. 2:* Contre [euh] allez--

*Mme. 1:* [Rire].

*Mme. 2:* Aller hop ! Tu descends, tu descends ! Contre [euh], à Bonobo ça va être 20 euros. 20, 25.

*Mme. 1:* Mais il te durera [euh]--

*Mme. 2:* [Ben] tout le temps [en fait], à part si je le perds mais--

*Mme. 1:* Oui, mais c'est vrai que oui, les produits qu'on achète dans les magasins, au final, ils te font une saison puis après ils sont tous rouillés donc t'en rachètes un. Donc au final--

*Mme. 2:* Oui, c'est ça. Ouais.

*Mme. 1:* Ça revient au même prix que si t'en avais un--

*Mme. 2:* Ouais.

*Mme. 1:* [Euh], et le côté humain, est-ce que c'est-- donc là, quand tu parles de- de créateurs ou d'artisans, est-ce que tu as la- la même volonté quand tu vas acheter des- des fruits et légumes auprès de producteurs ou- ou moins que-- ?

*Mme. 2:* Moins quand même.

*Mme. 1:* Moins ?

*Mme. 2:* Ouais. Moins, parce qu'il y a plus une nécessité et puis c'est surtout qu'il y a beaucoup de monde en général.

*Mme. 1:* Ouais.

*Mme. 2:* Donc [euh]-- donc, si je leur demande [euh]-- [enfin] même- même quand je-- quand je parle de créateurs [et tout], si je vois qu'il y a beaucoup de monde, je vais pas me prendre la tête à aller-- à aller discuter avec eux [quoi].

*Mme. 1:* Ouais.

*Mme. 2:* Non, non, c'est juste quand je vois qu'il n'y a personne, comme la petite dame là [euh], quand j'ai vu pour ses- ses oeuvres, déjà premier truc je me suis dit "Wow ! J'ai l'impression qu'il n'y a personne qui vient dans le magasin".

*Mme. 1:* Ouais, ouais.

*Mme. 2:* Je me suis dit "Merde !" Et [du coup], [ben] je suis allée vers elle pour lui poser des questions, pour voir si ça fonctionnait, si elle arrivait à survivre [et tout] [quoi].

*Mme. 1:* Ouais.

*Mme. 2:* Et [euh]-- et c'est pour ça, ça dépend vraiment de-- des milieux et du- du temps [en fait].

*Mme. 1:* Ouais.

*Mme. 2:* Parce qu'au final, j'étais pas censée avoir le temps ce jour-là. Du coup, je suis arrivée en retard à mes trucs. Mais j'ai quand même pris le temps de discuter avec elle et de--

*Mme. 1:* Ouais.

*Mme. 2:* De voir un peu. Et je préfère aussi, c'est con, mais je préfe-- je préfère aussi payer en espèce. Comme ça je me dis qu'ils font ce qu'ils veulent derrière. [Rire]. Quand c'est les gros trucs, j'ai la carte bancaire.

*Mme. 1:* [Ben] oui, oui, oui.

*Mme. 2:* Et quand c'est les petits, je me dis bon, allez --

*Mme. 1:* Ouais.

*Mme. 2:* J'ai de l'espèce, prenez ça, je ne sais pas si ça peut vous aider.

*Mme. 1:* Ben ouais, ouais, ouais.

*Mme. 1:* Pas obligé de déclarer tout. [Rire]

*Mme. 2:* Ouais, c'est ça. Il faut pas le dire ça. [Rire]

*Mme. 1:* Je l'écrirai pas ! [Rire].

*Mme. 2:* Non mais, oui c'est ça, c'est le-- alors, je me dis quand t'as-- quand t'es dans la merde de ouf pour survivre.

*Mme. 1:* Ouais.

*Mme. 2:* [Ben], c'est bien quand tu as de l'espèce, tu vois ?

*Mme. 1:* Ouais, carrément.

*Mme. 2:* Au moins tu ne déclares pas et tu peux survivre quand même. [Voilà] ce que je me dis [quoi].

*Mme. 1:* Et donc, admettons [euh]-- par contre, si t'es genre à- à Saint-Sauveur, y'a une ferme à côté [euh], qui est en-- qui, genre-- je- je crée un scénario là, mais qui est en-- qui va pas très bien, des producteurs, et qui propose de la vente directe à la ferme. [Euh], est-ce que tu serais dans une démarche d'aller, [du coup] --

*Mme. 2:* Ah bah carrément

*Mme. 1:* Ouais.

*Mme. 2:* Moi, c'est même le premier truc que je vais voir même d'ailleurs. C'est sans- sans qu'ils soient [euh], en difficulté [et tout]. Si j'ai des producteurs de poulet.

*Mme. 1:* Ouais.

*Mme. 2:* Même si c'est pas du bio, bio.

*Mme. 1:* Ouais.

*Mme. 2:* Je préfère quand même aller les voir et puis essayer de-- [ben] et [en fait], directement de la personne à la personne [en fait].

*Mme. 1:* Ouais. Ouais, privilégier la vente directe.

*Mme. 2:* Ouais, je trouve ça plus cool. Puis au moins, c'est ce que je disais à mon copain, c'est que moi, je veux pas forcément être végétarienne.

*Mme. 1:* Ouais.

*Mme. 2:* Par contre, je veux savoir ce que je mange.

*Mme. 1:* Ouais.

*Mme. 2:* Donc, c'est- c'est pour moi, c'est le même problème que la clope ou l'alcool. C'est quand tu consommes quelque chose, faut que tu saches exactement quels impacts ça a sur ton organisme.

*Mme. 1:* Ouais.

*Mme. 2:* Et comment ça été fait. Et quand t'as tous ces éléments-là, ça te donne le- le choix [en fait] tu peux vraiment choisir.

*Mme. 1:* Ouais.

*Mme. 2:* Mais [euh], [enfin], être végétarien sans-- [enfin] [en fait], parce qu'il y en a beaucoup et mon copain, il a fait un peu ça à un moment. C'est comme un effet de mode [en fait].

*Mme. 1:* Ouais.

*Mme. 2:* Les gens disent faut être végétarien pour être écolo, alors que pas forcément. [Enfin], je suis pas forcément d'accord.

*Mme. 1:* [Hum].

*Mme. 2:* Je medis t'as plein de petits fermiers qui élèvent très bien leurs poules. Moi, ma grand-mère, elle élevait elle-même ses lapins et ses poules qui étaient en liberté [et tout]. Qui étaient tranquilles [quoi].

*Mme. 1:* Ouais.

*Mme. 2:* Elles faisaient leur vie, puis après, hop après elles les mettaient à la casserole et puis [voilà]. Et je pense que ça posait pas problème dans le sens où c'était propre.

*Mme. 1:* Ouais.

*Mme. 2:* Alors que maintenant, on dit rien parce qu'on sait que c'est dégueu derrière [en fait].

*Mme. 1:* Ouais, c'est ça.

*Mme. 2:* Donc [euh]-- mais moi je ne suis pas-- [enfin], je veux pas forcément être végétarienne, par contre je veux être consciente de ce que je mange.

*Mme. 1:* Ouais.

*Mme. 2:* Et même jusqu'à mettre plus cher.

*Mme. 1:* Ouais.

*Mme. 2:* Je m'en fou de mettre plus cher, je mangerais moins de viande et c'est très bien de manger moins de viande, parce que je trouve qu'on en consomme trop de toute façon. Mais [euh], ouais vraiment même moins cher et prendre de la qualité. Mais ça, c'est quelque chose qui- qui prend énormément de temps, parce qu'[en fait], on n'a pas forcément les petits producteurs à côté [quoi].

*Mme. 2:* [En fait], c'est ça je trouve qui manque et on n'a pas la-- on a pas-- on n'est pas informé dessus.

*Mme. 1:* Ouais.

*Mme. 2:* Y'a pas de -- déjà de prévention, y'a pas d'information.

*Mme. 1:* Ouais.

*Mme. 2:* [Enfin], c'est vraiment [euh] si tu veux te- te détacher du système, [ben] t'as intérêt de forcer [quoi], faut y aller, faut se battre pour se détacher [euh]. C'est- c'est fou, je me dis quand même.

*Mme. 1:* Ouais. Ouais, c'est à toi d'aller chercher l'information, elle vient pas à toi [euh] toute seule [quoi].

*Mme. 2:* Ouais et puis même chercher, même avec internet, je trouve ça assez difficile quand même.

*Mme. 1:* Ouais, parce que souvent ce ne sont pas des gens qui sont forcé-- [enfin], c'est pas des--

*Mme. 2:* C'est souvent du bouche à oreilles, [enfin], avec les voisins donc [du coup] tu sais pas [en fait].

*Mme. 1:* En campagne, ils savent peut-être un-- [enfin] [voilà] il y a moins de propositions, ils peuvent aller [euh]--

*Mme. 2:* [Ben] nous, on a un producteur d'escargots qui habite juste à côté de chez nous là.

*Mme. 1:* Ouais.

*Mme. 2:* J'aime pas l'escargot, donc je vais pas trop là-bas, mais je sais qu'il vend, [du coup], ses petites boîtes d'escargots [euh] --

*Mme. 1:* Ouais.

*Mme. 2:* C'est marrant.

*Mme. 1:* [Ben] oui

*Mme. 2:* [Mais bon]. On a des porcheries aussi, donc c'est génial.

*Mme. 1:* Ah ben oui.

*Mme. 2:* Mais [euh], mais n'empêche, mon copain était choqué parce que quand on est passé dans les porcheries et [ben] tous les cochons couraient dans l'herbe.

*Mme. 1:* D'accord.

*Mme. 2:* [Enfin], ils étaient libres.

*Mme. 1:* Ouais.

*Mme. 2:* [En fait]. Et lui, il avait jamais vu ça. Première fois qu'il voyait des cochons--

*Mme. 1:* [Ben] j'avoue que [euh]-- alors, les moutons, les chèvres, les vaches [euh], j'en vois. Des poules, des fois, des poulets j'en vois. Mais des cochons gambader ?

*Mme. 2:* [Rire].

*Mme. 1:* Alors dans des fermes, près des [enfin]-- je viens de Normandie, donc des petites fermes vraiment on parle de basse-cour limite [quoi] où là, j'en ai vu tranquille.

*Mme. 2:* Ouais, c'est ça.

*Mme. 1:* Mais une porcherie qui fait que du cochon [euh], jamais vu de-- [Rire]

*Mme. 2:* Ouais, c'est ça, parce que nous on nous parlait de porcherie et puis on était là "Oh vas-y, on a un ruisseau, donc est-ce que ça vient pas directement de la porcherie [et tout] ?"

*Mme. 1:* Ouais.

*Mme. 2:* On a commencé à faire "Oh ! putain"

*Mme. 1:* Ouais.

*Mme. 2:* Et [en fait], quand on est passé devant-- bon le ruisseau, normalement la source est dans notre jardin aussi. Donc ça vient des nappes phréatiques en-dessous donc bon. On peut avoir une chance que ça soit pas pollué mais quand on passe devant la porcherie qui est assez grande, parce que c'est un grand bâtiment.

*Mme. 1:* Ouais.

*Mme. 2:* Et [en fait], [bah] ouais on voit les cochons dehors qui viennent jusqu'à la route comme les-- comme les vaches [en fait] qui viennent voir les voitures [et tout].

*Mme. 1:* C'est plutôt--

*Mme. 2:* [Hum], donc à voir [hein].

*Mme. 1:* Oui.

*Mme. 2:* Je me dis, là, y a peut-être un truc quand même. Peut-être !

*Mme. 1:* Peut-être un truc à aller regarder.

*Mme. 2:* Peut-être un truc qui est moins grave que les autres. Mais bon.

*Mme. 1:* Ok. Alors la dernière photo.

*Mme. 2:* Alors, supermarché.

*Mme. 1:* Ouais.

*Mme. 2:* Je cherche un nom mais je le trouve pas. [Euh] sur la quantité de fou [quoi], la-- quand ya-- quand y a une quantité qui est immense, comment on dit ça ?

*Mme. 1:* C'est pas abondance que tu-- ?

*Mme. 2:* Oui, abondance. J'avais omniprésence mais c'était pas ça. Abondance, ouais. Propre.

*Mme. 1:* Ouais.

*Mme. 2:* Nickel, carré. Aligné. Rangé.

*Mme. 1:* Ouais.

*Mme. 2:* Triste.

*Mme. 1:* Triste ?

*Mme. 2:* Ouais.

*Mme. 1:* Pourquoi tu penses à triste ?

*Mme. 2:* Parce que c'est trop droit, c'est froid je trouve.

*Mme. 1:* Ouais.

*Mme. 2:* Il y a personne.

*Mme. 1:* Ouais.

*Mme. 2:* Il y a pas une personne dans le sens vendeur, vraiment producteur, tu sais pas d'où ça vient. Donc, peut-être aussi [euh]-- ouais c'est pas-- c'est- c'est opaque.

*Mme. 1:* Ouais.

*Mme. 1:* Est-ce que c'est un milieu dans lequel tu te sens [euh] plutôt bien ou- ou non ? Pas vraiment ? Ce genre de lieu ?

*Mme. 2:* Neutre, je dirais.

*Mme. 1:* Ouais ?

*Mme. 2:* [Hum]. Je suis plus là par nécessité quand j'y vais.

*Mme. 1:* Ouais.

*Mme. 2:* J'y vais pas pour prendre un bon moment [quoi]. C'est vraiment pas [euh]-- au contraire même, c'est plutôt [euh] jJ'aime bien mettre ma musique, me mettre dans ma bulle. Prendre ce dont j'ai besoin et partir.

*Mme. 1:* Ok.

*Mme. 2:* Je ne reste pas.

*Mme. 1:* Ouais. Comme [euh] --

*Mme. 2:* Ouais ?

*Mme. 1:* Comme quand y a le monde, tout, la foule au supermarché ? Tu fais la même [euh]-- ?

*Mme. 2:* Ouais, ouais.

*Mme. 1:* Le même mécanisme.

*Mme. 2:* Ouais, c'est ça. Et [euh], quand- quand je vais là-bas, en général, je sais ce dont j'ai besoin. Donc j'y vais pas pour flâner.

*Mme. 1:* Ouais.

*Mme. 2:* Je sais exactement ce dont j'ai besoin. Et quand-- [bon] quand je me dis faut prendre des légumes, là, on en a plus [et tout] faut que trouve quelque chose à prendre et que j'y vais, je vais dans le rayon légume et à chaque fois c'est la galère.

*Mme. 1:* Ouais.

*Mme. 2:* Parce que il n'y a rien qui m'inspire vraiment [en fait].

*Mme. 1:* D'accord.

*Mme. 2:* Donc [euh] j'ai du mal même les tomates, les champignons, j'adore ça [et tout] et là-- je sais pas. J'ai pas trop envie [quoi].

*Mme. 1:* Et tu sais-- tu saurais pas dire pourquoi ?

*Mme. 2:* [Ben] je pense par ce que c'est banal [en fait]. [Enfin], c'est-- je sais pas.

*Mme. 1:* Ouais.

*Mme. 2:* C'est hyper banalisé au même titre que tous les autres fruits et tous les autres légumes. [Euh], les prix [ben], ils sont normaux. [En fait], tout est tellement creux, je trouve.

*Mme. 1:* Ouais.

*Mme. 2:* C'est- c'est plat. [Enfin], je-- c'est bizarre dis comme ça.

*Mme. 1:* Si je comprends.

*Mme. 2:* Ouais. C'est-- ouais, c'est normal [quoi]. [En fait] c'est un peu comme si on était des robots, tu vois ?

*Mme. 1:* Ouais.

*Mme. 2:* On arrivait dans le supermarché. On devait prendre juste des produits, limite sans regarder [en fait]. T'as pas besoin de regarder.

*Mme. 1:* Ouais.

*Mme. 2:* Tu prends, [voilà] tu te poses pas de question, tu vas pas chercher pas plus loin. Tu prends, tu payes, tu rentres chez toi. [Enfin], je trouve ça [euh] assez hallucinant [quoi]. T'as pas de contact, t'as pas [euh]-- les gens, ils sont tous soit sur leur téléphone quand ils marchent.

*Mme. 1:* Ouais.

*Mme. 2:* Soit [euh] en train de gueuler sur les gamins qui est à côté. Soit-- [enfin]-- [en fait], t'as-- t'es pas en vrai contact avec les gens. Plus avec des papis et des mamies je trouve.

*Mme. 1:* Ouais.

*Mme. 2:* Papi, mamie, j'arrive plus à discuter avec eux surtout dans les grandes surfaces genre [euh]-- parce que là, je pensais au Netto quand je parlais.

*Mme. 1:* Ouais.

*Mme. 2:* Mais [euh] je sais que dans les grandes surfaces surtout quand je vais le matin après le travail. [Euh], du genre grand Carrefour, Géant [et tout], les trucs qui sont immenses, [eh ben] là, je sais pas les papis et les mamies, j'ai l'impression qu'ils viennent là-bas pour parler [en fait].

*Mme. 1:* D'accord.

*Mme. 2:* Et [du coup], ils te mettent le grappin dessus. [Rire] et après, j'ai beau changer de rayon ils suivent [quoi].

*Mme. 1:* [Rire].

*Mme. 2:* Mais c'est-- oh, c'est vrai [hein]. Ya une fois, elle m'a suivi du début jusqu'à la fin et elle faisait "Oh mais c'est fou ! On tombe tout le temps en face !"

*Mme. 1:* [Ben] oui.

*Mme. 2:* C'est fou ça ! [Rire] Et oui, oui, bon après moi je trouve ça mignon donc ça va parce que je me dis que s'ils sont comme ça c'est qu'il y a un besoin donc [euh]--

*Mme. 1:* Ouais.

*Mme. 2:* Limite, j'ai envie de les inviter chez moi pour prendre un café après. Tant qu'à faire plutôt que de me suivre dans le magasin [tu vois ?] Non, mais--

*Mme. 1:* Donc, ouais vous trouv-- [enfin] tu trouves ça plutôt froid comme- comme lieu, les grandes surfaces ? Et quand t'as dit [euh] que ça ne te donnes pas envie, que tout est banalisé. Qu'est-ce que tu voulais dire par là ? Que les produits [euh]-- ?

*Mme. 2:* [Ben] [euh] qu'on se pose pas la question de savoir d'où ça vient [et tout] ça. [En fait], on- on ne nous amène pas à réfléchir.

*Mme. 1:* Ouais.

*Mme. 2:* C'est-- je sais pas comment dire parce que c'est pas vraiment des- des mots qui correspondent mais, c'est un peu neutre, banalisé, genre plat.

*Mme. 1:* Ouais.

*Mme. 2:* C'est vraiment ça. C'est-- t'as même pas à répondre. C'est juste t'as besoin de manger un truc, prends n'importe quoi, ça fera l'affaire.

*Mme. 1:* Oui.

*Mme. 2:* Tu vas pas réfléchir je trouve quand tu vas dans un truc comme ça.

*Mme. 1:* Oui.

*Mme. 2:* Bon si des fois, je rigole un peu quand même, quand je vois la provenance des trucs et qu'il y a marqué bio [et tout] donc en général, avec mon copain, je fais attends regarde, c'est bien drôle.

*Mme. 2:* Voilà, on discute un peu des prix mais [euh] --

*Mme. 1:* Ouais.

*Mme. 2:* [Voilà quoi].

*Mme. 1:* [Euh], donc ça t'arrives d'acheter des- des fruits et légumes quand même là-bas, même si c'est pas une partie de plaisir, [enfin] ils t'inspirent pas sur le moment, est-ce que ça t'arrives quand même d'en prendre ou-- ?

*Mme. 2:* [Euh] [ouais] parce que, vu que-- [ben] vu qu'on travaille pas mal et que là, la mini pelleteuse est prise, c'est surtout [inaudible 00:51:54].

*Mme. 1:* Ouais.

*Mme. 2:* Et vu que j'ai pas beaucoup de temps entre le boulot la fac [et tout], [ben] [en fait], on y va le dimanche. [En fait], on y va les jours de marché.

*Mme. 1:* D'accord.

*Mme. 2:* Et du coup, [ben], le marché il est pas là à 23 heures le soir.

*Mme. 1:* Non.

*Mme. 2:* Quand on rentre. Donc, [du coup], on peut pas vraiment s'arrêter [et tout] [quoi]. Donc [euh], bah là, on à la-- on est pris et puis la semaine, quand je veux cuisiner et que je me dis "Merde, je n'ai pas de champignons, j'ai rien." [Ben], [du coup], je vais au Netto parce qu'il est à côté.

*Mme. 1:* Ouais. Et tu penses quoi des produits que tu trouves là-bas, de manière générale ?

*Mme. 2:* [Ben], ça se mange [quoi].

*Mme. 1:* Ouais.

*Mme. 2:* [Hum]. C'est pas [euh]-- c'est pas super sucré, c'est pas un truc de fou [quoi], ce que je mange.

*Mme. 1:* Ouais. Pas une explosion de saveur mais [euh]--

*Mme. 2:* [Voilà], ça fait l'affaire [en fait], ça répond à-- ça répond aux besoins.

*Mme. 1:* Ouais.

*Mme. 2:* Ouais.

*Mme. 1:* Ça répond aux besoins, plus qu'aux envies du coup ? C'est un peu ça ?

*Mme. 2:* Oui, oui, c'est ça, oui. Mais après, j'ai pas forcément l'envie non plus. Quand je vais à Netto, je me dis pas "Wow ! J'ai envie d'un truc super-- à défaut de fraise sucrée" De base, je sais que ça sert rien de me dire ça, Donc, [du coup], je me dis pas ça de toute façon et si vraiment j'ai envie de produits de qualité, [ben] là, je vais [euh] -- c'est quoi le magasin qu'il y à ? Ici.

*Mme. 1:* Ouais.

*Mme. 2:* Tu vois je vais peut-être plus dans des trucs comme ça et encore je sais même pas.

*Mme. 1:* Ouais.

*Mme. 2:* Mais bon je-- [ouais]. Après, c'est vrai que, [en fait], j'ai plus l'habitude de dire "Ah ben tiens, je vais-- j'ai envie d'avoir de bons légumes !"

*Mme. 1:* Ouais.

*Mme. 2:* [En fait], je me dis plus ça.

*Mme. 1:* Oui.

*Mme. 2:* Je pense que tant qu'on n'a pas trouvé de producteurs vraiment dans le coin, et qu'on connait pas leurs méthodes [et tout] c'est difficile, à part, [ben] chez soi parce qu'on sait comment c'est fait [et tout]. Quoique même ici la pollution j'ai des doutes [du coup] mais--

*Mme. 1:* Ouais là, tes attentes sont un peu en mode [euh] réalistes par rapport là à ton rythme [euh], le fait que-- [enfin] là, tes provenances, tu ne te mets pas d'attentes spécifiques. Pour le moment, là, sur tes- tes fruits et légumes [quoi] ?

*Mme. 2:* Si je mets des attentes, au niveau gustatif.

*Mme. 1:* Ouais.

*Mme. 2:* C'est plus sur le poisson.

*Mme. 1:* D'accord.

*Mme. 2:* Quand je veux du poisson ou de la viande. Ça, c'est un truc [euh] j'achète presque plus de-- d'emballé déjà. Bon, à part du steak haché qu'il faut faire à l'arrache pour de la bolognaise ou des trucs comme ça, là je prends plus [euh] comme ça, mais sinon- sinon, je vais chez le poissonnier. Quand- quand, j'ai envie de prendre des noix de St Jacques, je vais en prendre deux.

*Mme. 1:* Ouais.

*Mme. 2:* [Ben] [du coup], deux grosses et puis, je veux savoir d'où ça vient, vraiment voir un poissonnier qui me donne confiance déjà en lui, ce qui va me permettre de-- d'acheter [quoi].

*Mme. 1:* Ouais.

*Mme. 2:* Parce que si je sens pas le poissonnier et que j'aime pas trop la façon dont c'est présenté, la couleur des produits [et tout]. Je vais pas les prendre.

*Mme. 1:* Ok.

*Mme. 2:* Je préfère limite, prendre sous plastique. Je me dis temps qu'à payer cher--

*Mme. 1:* Oui.

*Mme. 2:* [Voilà], faut que--

*Mme. 1:* Oui, si le produit est le même autant prendre [euh]--

*Mme. 2:* Et c'est vraiment des arna -- [enfin], y a vraiment des anar-- des arnaques de fou là-dessus [pardon].

*Mme. 1:* Oui.

*Mme. 2:* Parce que là, je me suis encore fait avoir l'autre jour [euh] sur des coquilles St Jacques.

*Mme. 1:* Ouais.

*Mme. 2:* Et [euh]- et j'avais pris ça au traiteur, bon là, j'étais à Carrefour, donc le traiteur de Carrefour. Je me suis dit je vais pas prendre emballé, je vais directement [euh] voir le traiteur.

*Mme. 1:* Ouais.

*Mme. 2:* Une blinde ! Ça m'a coûté une blinde la coquille. Arrivée ici, [genre] t'avais une pauvre noix de St Jacques dedans et que de la crème. Genre sur toute la coquille, c'était que de la crème. Ils avaient blindé de chapelure, que ça passe nickel.

*Mme. 1:* Oui, je vois pas [du coup], ce qu'il y a dedans.

*Mme. 2:* Mais j'étais là, mais c'est vraiment des- des cons [quoi] !

*Mme. 1:* [Ouais], c'est abusé. C'est pas-- c'est pas les petits champignons, sinon on a des petits champignons ou des-- je sais pas ce qu'ils mettent dedans.

*Mme. 2:* Ouais. Oui, oui. Mais, c'est-- moi, j'a-- j'attends au moins qu'il y en ai trois, quatre, [tu vois] des coquilles.

*Mme. 1:* Oui, oui en plus oui --

*Mme. 2:* Au moins, [enfin], que t'es-- que t'es une vrai coquille St Jacques [quoi] et --

*Mme. 1:* Oui, oui. Oui normalement à chaque bouché presque t'en as-- t'as ou alors elle est coupée en plein de morceaux mais t'en as une qui arrive {euh] --

*Mme. 2:* Oui, c'est ça. T'as des morceaux [quoi].

*Mme. 1:* Oui. [Rire]

*Mme. 2:* Tu croques.

*Mme. 1:* Oui, tu vas manger la St Jacques quand elle-- [Rire].

*Mme. 2:* Limite, tu pouvais la boire là [euh], [enfin] la boire c'était quand même de la purée [tu vois ?] Mais bon--

*Mme. 1:* Ouais.

*Mme. 2:* Oh ouais j'étais pas contente. Et puis c'est le principe des sushis aussi.

*Mme. 1:* Oui.

*Mme. 2:* Je sais pas, tu les vois faire leurs sushis en plus, ils les font devant toi, et pourtant ils sont dégueu. Alors, je sais pas comment ils font. Vraiment, genre je pige pas, parce que à chaque fois je prends les sushis que les dames font.

*Mme. 1:* Ouais.

*Mme. 2:* Et pourtant [euh], [ben], ça n'a jamais le même goût que quand tu fais toi-même.

*Mme. 1:* Ouais.

*Mme. 2:* Pourtant, elles le font avec leurs mains [quoi].

*Mme. 1:* Ouais, c'est le produit--

*Mme. 2:* C'est hallucinant, [ouais] c'est ça. Je sais pas d'où viennent leurs produits mais c'est [euh]--

*Mme. 1:* Ouais.

*Mme. 2:* Là, on prend même plus là [hein]. On a pris une fois.

*Mme. 1:* Oui.

*Mme. 2:* On prend plus aussi. Quand tu vois le prix. Parce que [du coup], [ben] là- là il se font plaisir.

*Mme. 1:* Ouais.

*Mme. 2:* Et [euh], surtout à Gouesnou, le Leclerc de Gouesnou, l'espace [euh], un peu nourriture chinoise [et tout] là.

*Mme. 1:* Ouais.

*Mme. 2:* Là c'est vraiment des arnaqueurs de fou là, mais vraiment.

*Mme. 1:* [Rire].

*Mme. 2:* Genre, on avait payé 11 euros, ça de pâtes.

*Mme. 1:* De pâtes ?

*Mme. 2:* Ouais. De nouilles chinoises. Et tu sais ce qu'elle nous fait en plus ? Elle met, elle fait "Ah bah 11 euros !" puis elle fait "Vous avez l'air sympa je vais vous rajouter des pâtes dedans !" Genre, j'étais là "11 euros ?" Tu te fous de ma gueule ?

*Mme. 1:* Des nouilles [quoi].

*Mme. 2:* Ouais, j'espère que tu peux rajouter des nouilles quand même. Beaucoup plus même. Pour arriver à 11 euros.

*Mme. 1:* Ouais ça fait [euh] 10 kilos de nouilles. [Rire].

*Mme. 2:* Non, mais c'est ça. Non, mais j'étais là, c'est quoi ce-- c'est une arnaque de fou [quoi].

*Mme. 1:* Ouais.

*Mme. 2:* C'est vraiment, c'est- c'est fait pour entuber les gens.

*Mme. 1:* Ouais.

*Mme. 2:* C'est hallucinant. Et moi, je ne pensais vraiment pas. Parce que bon, t'as- t'as des petites arnaques où ils se font une marge mais bon, ça reste raisonnable [quoi]. Mais là, c'est plus qu'une marge [euh]--

*Mme. 1:* Oui, oui, oui, là oui, oui.

*Mme. 2:* [Voilà], je fais 11 euros. On était à la limite de lui rendre la boîte et après on s'est dit, ça se fait pas trop quand même.

*Mme. 1:* Ouais.

*Mme. 2:* [Du coup], on était bloqué et j'ai fait, bon on va-- on va goûter les nouilles qui d'ailleurs n'avaient rien d'exceptionnelles en plus, [tu vois], je me suis dit peut-être qu'il y a de l'or [tu vois] dedans qui font que le-- les nouilles sont à 11 euros.

*Mme. 1:* Ouais, c'est ça. Sauce- sauce dorée.

*Mme. 2:* Ouais, [rire], c'est ça. Mais non, même pas. C'était même pas terrible en plus [alors bon].

*Mme. 1:* En plus, ouais]. [Rire]. Effectivement.

*Mme. 2:* On a vraiment jeté de l'argent.

*Mme. 1:* Ouais. [Ben] oui. Et [euh], est-ce que ça t'arrive de jeter des fruits et des légumes que t'as acheté au supermarché ? Ou pas ? Que t'as stocké chez toi et-- ou que tu n'as même pas eu le temps de stocker ?

*Mme. 2:* Oui, oui, oui, oui.

*Mme. 1:* Ouais ?

*Mme. 2:* [Ben] surtout les barquettes. Quand t'as des barquettes de champignons, de fraises.

*Mme. 2:* Ouais.

*Mme. 1:* Qu'est-ce qui fais qu'à ton avis que -- ?

*Mme. 2:* [Ben] parce que c'est humide.

*Mme. 1:* Ouais.

*Mme. 2:* Qu'ils mettent ça sous emballage alors que c'est humide dedans, qu'ils laissent pas respirer les produits. [Du coup], quand tu prends les champigons du dessus [eh ben] tu vois que tout est moisi en-dessous.

*Mme. 1:* Ouais.

*Mme. 2:* [Voilà], et ça, ça m'énerve un peu aussi.

*Mme. 1:* Oui ? Qu'est-ce qui-- [du coup], qu'est-ce que-- t'es énervée contre [euh] ?

*Mme. 2:* [Ben] parce que c'est-- pour moi, c'est une arnaque aussi [quoi]. À un moment, tu penses acheter, je sais pas, 500 grammes de champignons alors que tu manges que 200 grammes parce que le reste est déjà moisi moisi, moisi ? T'as de la vie dedans.

*Mme. 2:* Et c'est pas possible [quoi].

*Mme. 1:* Ouais. Ça justement c'est le-- quand en grandes surfaces tu peux trouver des champignons un peu en vrac, bon, [du coup] là qui peuvent être plus respirer. Mais les bio, ils seront toujours en barquette. [En fait] si tu choisis entre savoir que tu prends un bio et que t'as--

*Mme. 2:* Peut-être pas--

*Mme. 1:* Ou t'as de forte chance où la moitié en-dessous [enfin] qui soit déjà un peu tout mou [et tout], alors que si tu veux faire manger cru ou [quoi]. Ou alors tu prends en vrac mais ils sont pas bio, [enfin] tu [euh]-- c'est [euh]-- t'es obligée de faire une concession à un moment donné sur [euh]- sur ce que tu veux manger [quoi]. Ok. Alors, est-ce que tu manges de la banane ?

*Mme. 2:* Oui. Justement j'ai récupéré par ma soeur qui travaille au Lidl j'ai récupéré 50 bananes.

*Mme. 1:* 50 bananes ?

*Mme. 2:* 50 bananes, parce qu'elle, elle devait en avoir 70. Elle a récupéré une cagette de 70 bananes, des grosses bananes.

*Mme. 1:* Ouais.

*Mme. 2:* Et elle m'a dit je sais pas quoi faire. Donc elle m'a refilé 50 bananes.

*Mme. 1:* Et [du coup], c'était matin, midi et soir ? Ou comment tu-- ?

*Mme. 2:* [Ben] [euh], j'ai appelé mes potes. [Rire] Qui veut des bananes ? En plus, j'ai un- un gros amateur de banane donc il est venu, il m'a pris 25.

*Mme. 1:* Ok.

*Mme. 2:* Et [du coup] après, j'ai fait des gâteaux pour tout le monde.

*Mme. 1:* [Ben] ouais.

*Mme. 2:* [Du coup], les bananes sont passées. Et je crois qu'on en a jeté la moitié d'une en tout et pour tout parce qu'elle était vraiment noire, noire écrasée [quoi].

*Mme. 1:* Ouais ?

*Mme. 2:* Et [prénom] a pas voulu la manger.

*Mme. 1:* {Eh ben] justement, là l'idée t'as 10 bananes. C'est la même banane.

*Mme. 2:* Ouais.

*Mme. 1:* En état de-- tu vois les différences.

*Mme. 2:* Et je vais te demander de les ranger celles qui te donne le plus envie, celles qui te donne le moins envie de-- en mode de la manger là, tout de suite comme ça.

*Mme. 2:* [Silence 01:00:34 - 01:00:53] Juste, j'ai mis par couleur parce que ça va être compliquer.

*Mme. 2:* Je dirais [euh] celle-là, la première.

*Mme. 1:* T'as préféré carré. Carré orange.

*Mme. 2:* Deuxième.

*Mme. 1:* Ouais. Triangle vert.

*Mme. 2:* Troisième.

*Mme. 1:* D'accord.

*Mme. 2:* Quatrième.

*Mme. 1:* Ok.

*Mme. 2:* Cinquième, sixième en-dessous.

*Mme. 1:* Ok.

*Mme. 2:* Et après dans l'ordre. Hop, voilà.

*Mme. 1:* Ok. Alors, je note à chaque fois. Triangle bleu, après triangle bleu,triangle rose, carré blanc. J'ai pas mis de numéro parce que j'avais peur que ça influence les gens.

*Mme. 2:* Ah oui ?

*Mme. 1:* Oui. [Rire]. Carré bleu. Rond jaune. Rond bleu, non rouge. Triangle blanc. Ok. Alors, Est-ce que tu peux me dire [euh]--

*Mme. 2:* Il est 11h50 là ?

*Mme. 1:* Oui.

*Mme. 2:* Oh merde !

*Mme. 1:* T'as un truc ?

*Mme. 2:* Ouais. Je bosse à midi.

*Mme. 1:* Ah ouais ! [Ben] non, on arrête. [Rire].

*Mme. 2:* Oh merde déjà ? maist'es arrivées à quelle heure ?

*Mme. 1:* À 10h30. 10h30 à midi.

*Mme. 2:* Ça fait déjà 1 heure 20 qu'on est là ?

*Mme. 1:* Ouais qu'on parle [ouais]. [Ben] je pense après on a mis. J'éteins l'enregistrement, ça fais 1h03 que l'enregistrement tourne.

*Mme. 2:* Putain !

**Household F23, Interview 1**

00:00:05
*Mme. 1:* Alors, vous êtes prête ?

00:00:07
*Mme. 2:* Oui.

00:00:07
*Mme. 1:* [rire] Du coup, dans un premier temps, je vous pose un petit jeu de devinettes. Je vais vous demander de penser à un fruit ou à un légume que vous aimez tout particulièrement. Vous allez penser à ce fruit ou à ce légume, sans jamais révéler son identité. Le but, c'est que je devine uniquement grâce à une description.

00:00:27
*Mme. 2:* D'accord, OK.

00:00:28
*Mme. 1:* Alors est-ce que vous avez un fruit ou un légume en tête ? Oui, alors allez-y, je vous écoute, dites-moi tout ce qui vous passe par la tête sur ce fruit ou ce légume.

00:00:37
*Mme. 2:* Alors, il fait partie de la famille des- des fruits rouges.

00:00:40
*Mme. 1:* OK...

00:00:41
*Mme. 2:* Il est légèrement acidulé et se marie très bien avec de la pistache...

00:00:46
*Mme. 1:* OK.

00:00:47
*Mme. 2:* [Euh] c'est un petit fruit, une baie, avec plein de petits grains. [Euh] On peut en faire des tartes, on peut en faire des gâteaux, du coulis, du jus. [Euh]... Que dire ? Il est... couleur [euh] rosée.

00:01:06
*Mme. 1:* OK...

00:01:06
*Mme. 2:* [Euh] et si si c'est pas mûr, c'est vraiment pas bon... [rire]

00:01:10
*Mme. 1:* OK [rire].

00:01:12
*Mme. 2:* Voilà, c'- c'est un fruit qui fait appel à mes souvenirs d'enfance.

00:01:16
*Mme. 1:* D'accord... Est-ce que vous pouvez m'en dire un peu plus ?

00:01:28
*Mme. 2:* Mm-hmm..

00:01:28
*Mme. 1:* En tous cas là par exemple sur les souvenirs, pourquoi c'est des souvenirs d'enfance ?

00:01:32
*Mme. 2:* Parce que c'était un fruit que mon grand-père faisait pousser dans son jardin, un jardin en Bretagne, et que- que nous dans le Sud on n'avait pas, et que mon père n'arrivait pas à faire pousser. Alors je ne sais pas pourquoi, mais ça ne poussait que dans le jardin de mon grand père, enfin c'est ce que je croyais, parce que mes parents n'en achetaient jamais au supermarché, parce que ça coûtait très cher. [rire] Ça coûte d'ailleurs toujours très cher. [rire]

00:01:53
*Mme. 1:* OK. Et donc, au-delà de... ces souvenirs d'enfance. Qu'est-ce que vous aimez tout particulièrement, dans ce fruit ?

00:02:01
*Mme. 2:* Alors j'aime que ce soit un fruit saisonnier, qu'on achète pas tout le temps, parce que effectivement, il est trop cher, et que c'est bien de l'avoir dans son jardin. [euh] J'aime-- j'aime beaucoup son goût, entre le côté sucré et acidulé. [euh] J'aime bien sa forme, le fait qu'on puisse le mettre sur le petit doigt [euh]... J'aime-- Et comme j'adore énormément la pistache, je suis ravie de faire des mélanges entre la pistache et ce fruit-là, voilà. [rire]

00:02:34
*Mme. 1:* OK... Et en général, vous le mangez quand ? Plutôt le matin, plutôt le midi, l'aprem, le soir ?

00:02:39
*Mme. 2:* Plutôt au goûter, en général quand je fais un goûter avec mes copines et [euh], j'en achète, et... pas que ça fait sensation, mais les gens sont relativement contents qu'il y en ait sur la table, voilà.

00:02:50
*Mme. 1:* Je vois... OK. [euh] Alors, je pense avoir ma petite idée un peu, tout à fait honnête. [rire] Mais du coup, racontez-moi, il y a le fruit qui est là. Est-ce qu'il y a des étapes avant de le consommer, ou pas ? Racontez-moi votre fruit est là, qu'est-ce que vous faites ?

00:03:09
*Mme. 2:* Alors ça dépend s'il vient du jardin. S'il vient du jardin, je cueille et je le mange, [euh] s'il vient du supermarché, je- je- je le lave, d'abord, enfin je le passe à l'eau et... je l'égoutte. [rire] M'enfin en général, je- je le mange plutôt frais, sinon, ça m'arrive d'en acheter des surgelés, pour faire des gâteaux. Effectivement quand je fais des gâteaux, je le mets toujours en surgelés. [silence 00:03:33- 00:03:36] Je sais pas si ça se trouve en boîte, ça me dit rien, mais [euh] je sais pas, je crois pas-- je ne crois pas. [rire]

00:03:50
*Mme. 1:* Si j'ai le bon fruit, je vois pas non plus, mais on verra. [euh] OK. Donc en général vous l'achetez où, le plus souvent ? Des fois, vous l'avez-- vous vous le procurez dans le- dans le jardin.

00:03:58
*Mme. 2:* Je l'achète-- On fait toujours nos courses au Super U, la grande partie du temps, donc on l'achète là. Ça nous est déjà arrivé d'en acheter au marché de Brest, et sinon je l'achète toujours en surgelé [euh] au Super U à côté. [silence 00:04:10- 00:04:13]

00:04:14
*Mme. 1:* OK, ça marche. Alors en plus du plaisir, du goût [euh] et des souvenirs, est-ce que vous avez le sentiment qu'il vous apporte autre chose, ou pas vraiment ?

00:04:23
*Mme. 2:* Non, pas spécifiquement. C'est vraiment en toute petite quantité, c'est rare. Donc, c'est vraiment le plaisir. C'est un peu comme un- un bonbon, quoi. [rire] Plus qu'un fruit ou--

00:04:37
*Mme. 1:* Je vois. Et du coup, est-ce que à l'inverse, vous lui trouvez des inconvénients, à ce fruit ?

00:04:42
*Mme. 2:* Alors oui, il- il passe vite, surtout quand il vient du supermarché, il est vite en-- allez, en- même en 12 heures, il peut y avoir de la moisissure quoi, c'est assez incroyable. Donc c'est vraiment de la consommation instantanée parce que sinon, ça ne se garde pas.

00:04:57
*Mme. 1:* [rire) D'accord.

00:05:01
*Mme. 2:* Enfin comme la majorité des fruits et légumes du supermarché, j'ai envie de dire, qui passent tous très vite. [rire] Mais les framb-- pardon [rire, inaudible].

00:05:15
*Mme. 1:* [rire] De toute façon c'était la dernière question, j'allais le dire là. [rire]

00:05:20
*Mme. 2:* [rire] Ce fruit là en l'occurrence, il passe très vite. [rire]

00:05:20
*Mme. 1:* Il passe très vite. En 12 heures presque, c'est terminé quoi.

00:05:22
*Mme. 2:* Ah ouais non mais- c'est même pas une blague, ouais je pense en 12 heures, quand j'achète par exemple le goûter, le lendemain, au matin, y a de la moisissure dessus. Ah ouais, c'est incroyable.

00:05:29
*Mme. 1:* Ouais c'est vrai que c'est-- [euh] ben je- je reviendrai après sur [euh] ces questions-là. Si là on continue mon petit fil, [euh] j'aimerais que-- discuter de vos pratiques d'achat, et du temps de votre organisation en général. Donc, vous vous êtes deux, à vivre dans le foyer, et au sein de votre foyer du coup, qui est amené à faire les courses le plus souvent, ou alors, comment vous-- ? C'est vous ?

00:05:56
*Mme. 2:* Oui.

00:05:56
*Mme. 1:* Et du coup, comment vous vous organisez ? Est-ce que vous pouvez me raconter un peu ? Donc il y a le Super U ici--

00:06:00
*Mme. 2:* Alors en ce moment, c'est un petit peu chaotique, puisque j'ai énormément de travail, j'ai plus de 40 heures semaine, et je suis un peu fatiguée. Mais en règle générale, j'y vais plutôt [euh] le vendredi, [euh] ou le lundi soir, parce que j'ai un jour de RTT une semaine sur deux, donc le- le vendredi quand j'ai RTT c'est vrai que c'est pratique, j'y vais. Sinon voilà, ça peut se faire-- En fait, c'est un peu quand- quand on a besoin de quelque chose, parce que j'achète en gros, et puis- genre des fois, ça nous fait deux semaines, on achète juste des petits trucs ponctuels parce qu'on a oublié ça, ou voilà. Mais en règle générale oui, c'est des grosses courses toutes les deux semaines.

00:06:38
*Mme. 1:* OK.

00:06:39
*Mme. 2:* Quand je parle de grosses courses, je dis 60 euros quoi. Et sinon après voilà, on a, je pense, par semaine, 20 euros en plus, sur des petits trucs quoi, genre par exemple ben justement des fruits ou des légumes qui passent vite quoi.

00:06:56
*Mme. 1:* OK, d'accord. Et est-ce que vous savez ce que vous allez acheter, ou- ou pas quand vous vous rendez-- ?

00:07:02
*Mme. 2:* Oui, je fais des menus, des menus en essayant de les faire le soir et qu'il en reste pour le lendemain midi, pour emporter, (euh] j'essaye de faire assez équilibré mais bon, sur les deux dernières semaines, on a mangé plus de pizza que tout le reste de l'année donc [euh]. [rire] Voilà, avec le-- puis là avec le fait qu'on soit plus confinés non plus, on a des amis qui viennent, donc forcément on commande des pizzas, ou fast food, beaucoup plus que- qu'avant quoi, même plus qu'avant le- le- confinement, on faisait assez attention, mais alors là, c'est un peu, bouffe à tout va, quoi. [rire] Mais oui sinon je fais les courses, donc [euh] en règle générale, le gros, [euh) je le fais aux Bocaux d'Ana en vrac, donc tout ce qui est pâtes, riz, [euh] enfin les choses qui périment pas, la farine, voilà, j'ai-- la farine, des fois, je vais la chercher aussi [euh] au Moulin de- de [euh] Keriolet, près de Douarnenez, parce que je vais voir ma grand mère qui est là-bas, et elle est bonne cette farine. [euh] Je fais beaucoup de choses moi-même, gâteaux, pâtes... voilà en l'occurrence, pas maintenant, mais normalement oui. [rire] [euh] Et puis après ouais, les- les fruits et légumes donc, ça nous arrive souvent d'aller au marché, donc à Saint-Louis. Le pain, on fait nous-mêmes aussi. Normalement. [rire] Et [euh] voilà, les fruits et légumes, je dirais que, souvent je demande à [Prénom du conjoint] de- de passer les chercher [euh] en rentrant du travail, quoi. Je lui dis-- Il y a un petit primeur là, au coin, il a des sacs à vrac dans son sac et- et je lui dis : " ben tu me prends ça", puis il prend une courgette, ou il prend-- voilà [rire]. C'est un peu [euh] -- voilà. J'ai- j'ai des aliments [euh] genre des pâtes, du riz, qui sont là tous les jours, et puis- puis après voilà, en fonction des humeurs du jour, on prend ça comme fruits, on prend ça comme légumes, voilà. Et on mange très peu de viande, [euh] c'est-à-dire qu'on doit en manger une fois par semaine, quand on a du monde. Voilà donc [euh], et c'est souvent du poulet, parce que je ne mange aucune viande rouge, et je ne mange pas de porc, donc c'est s-- même tout le temps, du coup, voilà [rire] du blanc de poulet, pour être exact. [rire] Voilà, donc c'est la seule viande qu'on consomme ici. [euh] Sinon, des œufs, beaucoup, et [euh] beaucoup de légumes. J'achète aussi beaucoup de légumes, du coup, en surgelé, [euh] pour faire des soupes. Voilà, comme on a un Cook Expert, on en entend plus parler, voilà. Et ça j'en fais-- en règle générale, j'en fais deux par semaine, donc j'achète deux sachets par semaine, et ça nous fait toute la semaine. [silence 00:09:29- 00:09:33] Et on est gros consommateurs d'Uber Eats aussi depuis le confinement, ça veut dire que facilement, deux fois par semaine, on commande. Étant donné que [Prénom du conjoint] a des Tickets Resto, c'est vrai que c'est le- la façon de les passer, puisqu'on pouvait pas les passer, on allait plus au resto. Donc [euh], voilà. [rire]

00:09:47
*Mme. 1:* C'est clair ! [rire] Du coup effectivement, ça fait qu'il y a deux repas presque dans la semaine en moins, que vous- vous avez à--

00:09:57
*Mme. 2:* C'est ça, globalement, c'est ça, toujours deux repas.

00:09:59
*Mme. 1:* Ça marche. Alors du coup sur les fruits et légumes, [euh]... Donc, c'est de temps en temps au marché, ou ?

00:10:10
*Mme. 2:* Oui, c'est, je dirais, une fois par mois. Quand on a le temps en fait, on prend beaucoup, et en règle générale on prend plutôt ouais, des pommes de terre, des oignons, des carottes, qui se conservent et qu'on consomme dans le mois, [euh] et puis [euh], genre quand c'était l'époque des clémentines aussi, on allait beaucoup au marché, on les prenait là-bas, les clémentines. Elles étaient- elles tenaient plus longtemps que celles du- supermarché. Et [euh] qu'est-ce que-- Je réfléchis... Mm-hmm... Non après voilà, les- les fruits et légumes, ouais vraiment, on essaye de consommer bio aussi, on va toujours au stand bio, [euh] mais voilà, là on a beaucoup acheté aussi au supermarché des-des légumes et ils tiennent pas quoi. C'est une catastrophe. Genre en fait on les achète, et [euh] parce qu'on se dit ben-- moi mes plats sont prévus sur la semaine et en fait, ils font pas la semaine, donc [euh], donc obligés de jeter ou de- d’en récurer une bonne partie, genre l’autre jour on a acheté quatre nectarines bio au Super- au Super U, elles étaient pas mûres, et 24 heures plus tard, elles étaient moisies, mais toujours pas mûres, voilà, donc [euh] voilà. Et ça c’est un peu ouais-- ça- ça- ça dégoûte un petit peu quoi, de se dire on achète des fruits et légumes et ils sont pas mangeables et voilà donc [euh] on essaye d’acheter, voilà. Et puis c’est-- je trouve ici, c’est plus compliqué aussi, quand on vivait dans le Sud près de Toulouse, mes parents sont dans le Languedoc-Roussillon, les pêches, les nectarines, on va les chercher dans les arbres quoi donc [euh] c'est vrai que-- Moi je suis- enfin j'ai été élevée comme ça, le légume je vais le chercher soit dans la terre, soit dans le-- et vivre en ville, c'est ça que je trouve difficile, c'est qu'on a pas ce- cette facilité là, enfin ce rapport à la nourriture qui est super simple, quoi. Donc là, on n'attend qu'une chose, c'est de partir à la campagne et de faire un potager. r.

00:11:53
*Mme. 1:* Ah, c'est dans les plans ?

00:11:54
*Mme. 2:* Ouais, c'est dans les plans. On essaye, on cherche un peu là depuis quelque temps. On a failli acheter en septembre et--

00:11:59
*Mme. 1:* Ça le fait ?

00:12:00
*Mme. 2:* Non, y avait de la mérule partout. [rire] Maintenant on cherche.

00:12:06
*Mme. 1:* D'accord. Donc [euh] si on reprend, du coup, ça vous arrive tout de même, quand vous faites vos- vos menus pour la semaine, du coup de- d'acheter tout au supermarché, donc une fois à peu près la semaine, une fois la semaine-- non une fois tous les 15 jours, vous avez dit.

00:12:22
*Mme. 2:* Oui c'est ça plutôt, oui.

00:12:23
*Mme. 1:* Et vous prenez normalement vos fruits et légumes [euh] qui sont justement en fonction de vos menus. Et par contre, de temps en temps, ça peut vous arriver aussi d'aller au primeur, quand il manque un truc, d'aller juste passer sur le--

00:12:36
*Mme. 2:* C'est ça. Ou par exemple quand je fais, je sais pas, un sauté de- de- de nouilles, je sais que j'ai acheté mes pâtes- j'ai acheté [euh] tout le reste par exemple, le lait de coco, enfin ce genre de choses, et de dire à [Prénom du conjoint] le soir, genre avant de rentrer : "Est-ce que tu peux prendre du poulet et ces légumes-là ?", parce que [euh] c'est ça que je vais faire ce soir. Et ce qu'on fait aussi, c'est qu'on commande beaucoup sur Greenweez, c'est du bio [euh), et en fait ça nous permet de commander [euh) en-- des trucs qu'on trouve pas en fait forcément ici, genre des- des haricots azuki tout ça, du lait de coco un peu moins cher, ou enfin voilà.

00:13:08
*Mme. 1:* D'accord, OK. C'est- c'est comme un drive, mais c'est [euh]--

00:13:14
*Mme. 2:* C'est ça, ouais. On commande-- ça nous a-- c'est peut-être une fois tous les deux mois, quoi. Vraiment pour des trucs très spécifiques.

00:13:20
*Mme. 1:* Ouais, ouais je vois, c'est vrai que c'est des-- ça se développe sur les alternatives...

00:13:23
*Mme. 2:* Puis ça arrive vite quoi, en 24 heures 48 heures, bon après c'est-- ouais, si on pouvait faire autrement, on ferait autrement, mais c'est vrai que quand on a besoin d'un truc et qu'on le trouve-- qu'on est obligés de faire dix magasins dans Brest pour le trouver... Voilà. Donc [euh] on consomme comme ça, mais-- [rire] On fait des yaourts maison aussi. Le lait végétal maison aussi, à partir d'avoine, voilà.

00:13:50
*Mme. 1:* OK. Donc vous achetez de l'avoine et vous faites le lait-- ?

00:13:52
*Mme. 2:* Des flocons d'avoine et puis je les mixe, je les mixe et puis je les filtre. Sauf le-- les seules choses que je fais pas, c'est le lait de soja, parce que [Prénom du conjoint] est intolérant au lactose, depuis tout petit et [euh] les-- y a que le lait de soja qui prend comme les yaourts [euh], enfin comme les yaourts au lait de vache en fait, dans la machine à yaourts. Donc [euh]-- Sinon c'est d'autres préparations, il faut rajouter de l'agar-agar avec les autres laits végétaux, alors que le soja c'est le seul qui prend comme un lait de vache, quoi. Donc ça, j'achète aussi.

00:14:25
*Mme. 1:* Donc [euh] c'est pour ça, vos yaourts sont avec du lait de soja, c'est ça ?

00:14:26
*Mme. 2:* Lui lait de soja, et moi lait de vache.

00:14:27
*Mme. 1:* Ah oui, vous faites les-- [rire]

00:14:29
*Mme. 2:* J'en fais six de chaque. [rire]

00:14:34
*Mme. 1:* OK, ça marche. Alors-- et du coup, comment vous gérez vos quantités [euh] de fruits et légumes que vous achetez ? Est-ce que sur vos- quand vous préparez vos menus, c'est déjà bien-- voilà c'est, genre il faudra une courgette ou deux aubergines etc.a, ou c'est-- ou non, c'est plutôt écrit courgettes, aubergines, et c'est vous qui sur place [euh] gérez les quantités ?

00:14:56
*Mme. 2:* En fait, je fais mes menus par jour, par exemple je sais pas, lundi je mets- on va manger ça et y en aura pour tant de portions, et du coup j'ai mes recettes, et là je dis ben voilà il me faut deux carottes, il me faut ça, et en fait sur le côté-- après, quand j'ai fait tous mes menus de la semaine, je reprends une liste à part, et je dis bon alors j'ai combien de carottes ? J'ai combien de courgettes ? J'ai combien de grammes de riz, j'ai combien de-- voilà, et je fais comme ça.

00:15:16
*Mme. 1:* D'accord, OK. D'accord, donc c'est une liste qui est assez précise quand même, dans- dans ce que vous allez chercher [euh]--

00:15:24
*Mme. 2:* Sauf quand on achète voilà, au jour le jour comme ça, quand vraiment on a besoin d'un légume, par exemple une courgette, je sais qu'elles passent super vite, je vais pas lui dire dix jours à l'avance : "prends-moi trois carottes-- trois courgettes, quoi". [rire]

00:15:33
*Mme. 1:* Ouais, je vois. [rire]

00:15:34
*Mme. 2:* Là j'ai acheté des poivrons, ils sont là depuis longtemps, et [euh] j'ose même pas les regarder. [rire]

00:15:42
*Mme. 1:* Ah ouais. [rire] Ouais, OK. Donc c'est plutôt une fois tous les 15 jours, avec des compléments de temps en temps. OK. Alors [euh] là, l'idée, c'est de dresser une liste des différents fruits et légumes que vous avez l'habitude de consommer, comprenant ceux que vraiment-- voilà, qui vont faire partie de votre consommation [euh] pas quotidienne mais voilà, c'est très régulier, vous allez [euh] justement consommer ces fruits et légumes-là. Alors est-ce que vous pouvez m'en- m'en donner ?

00:16:11
*Mme. 2:* Sans- sans compter les saisons ou [euh] ?

00:16:12
*Mme. 1:* Si, vous avez le droit de me dire, si vous faites attention aux saisons, vous allez avoir envie de me dire [euh]--

00:16:15
*Mme. 2:* OK, donc dans la vie de tous les jours, ce qu'on achète tout le temps, c'est bananes, échalotes, oignons, ail [euh]. J'achète en conserve souvent des tomates concassées et du coulis de tomates tout ça, enfin [euh] ensuite, qu'est-ce que ce que- qu'est-ce qu'on achète ? En hiver, c'est très, très souvent des poireaux- [euh] poireaux, oignons rosés aussi, on fait beaucoup en hiver, [euh] au printemps, on continue sur le poireau [rire], je réfléchis [euh]... après voilà été forcément courgettes, aubergines, tomates, poivrons, fraises, framboises. [rire] En hiver beaucoup ouais, oranges, mandarines, ça on-- mandarines, je pense que l'hiver j'en mange quatre par jour, au moins. Je suis une grosse consommatrice de fruits, [Prénom du conjoint] moins, moi je peux en manger cinq ou six par jour, des fruits. [euh] [Prénom du conjoint] n'aime pas du tout les carottes, mais on en fait quand même très souvent, parce que moi j'aime ça. [rires]. Alors, la pomme de terre n'est pas un légume, mais à noter que [Prénom du conjoint] l'an dernier a acheté sur un coup de tête 60 kilos d'un coup. Donc l'année dernière, on a mangé énormément de patates, voilà. [rires]

00:17:33
*Mme. 1:* On le considère nous comme un, comme un légume, dans la consommation qu'on va avoir--

00:17:38
*Mme. 2:* Ben on en fait beaucoup. Je pense qu'on en fait deux fois par semaine, des pommes de terre. On en a toujours, patates douces énormément, genre c'est soit patates soit patates douces, [euh] je réfléchis, alors en automne, toutes les courges, butternut, potimarron. Je pense qu'un repas sur deux, c'est ça. [euh] Qu'est-ce que-- je réfléchis... Navet, beaucoup, plutôt sur les soupes d'hiver, les navets... [euh] J'en fais énormément en fait, donc il faut que je réfléchisse. [euh] L'été, nectarines, pêches, brugnons, les fruits rouges, le raisin, plutôt en septembre. [euh] Y a tellement de légumes, c'est ch-- ah, alors je fais beaucoup de mélanges si [euh] brocolis, chou romanesco, [euh], autre chou, y en a plein. [euh] Ce que j'aime pas du tout, c'est les endives, alors ça on en a jamais. J'aime pas le chou-fleur non plus, donc ça on en a jamais. En fait, je pense, voilà, on mange tous les légumes, sans exception, exceptés endives, asperges, chou-fleur. Si, les blettes, les blettes non plus. Mais après, tout, genre haricots verts, petits pois, je pense que haricots verts, on en a bien aussi une fois par semaine, souvent surgelés par contre, je prends souvent en surgelé [euh]. Je dirais vraiment, ce qui revient le plus, c'est [euh] ouais, le butternut, la courge en automne, en hiver, c'est le poireau, après, on a patates, carottes, peut-être douces, courgettes, énormément en été, je pense que c'est celui qu'on mange le plus avec les tomates. Et puis après voilà, ail, oignon, échalote, toute l'année, voilà. À chaque repas, je pense qu'il y en a au moins un des trois.

00:19:34
*Mme. 1:* OK, avec les pommes de terre.

00:19:35
*Mme. 2:* Puis on consomme beaucoup aussi de- de variétés anciennes, de carottes, de toutes les couleurs, les tomates de variétés anciennes de toutes les couleurs, de toutes les formes, des Green Zebra, des Cœurs de bœuf, enfin voilà. C'est mon père qui cultivait ça, donc il a toutes les variétés anciennes de tomates dans le Sud, et il nous a envoyé des graines, tout ça, donc [euh] donc on fait passer, ma grand-mère en a, et on a récupéré-- c'est pas le même goût que dans le Sud, mais elles sont bonnes quand même. [rire]

00:20:05
*Mme. 1:* Votre père, c'est son métier de-- ?

00:20:07
*Mme. 2:* Non, pas du tout. Mon père est CPE, mais grande passion pour le potager, fruits et légumes. Il a créé un potager dans le lycée, enfin voilà, il fait beaucoup de choses. C'est son passe-temps quoi, je pense qu'il est bien quatre heures dehors par jour sur le potager, voilà.

00:20:24
*Mme. 1:* Et du coup, petite vous aviez [euh]-- ?

00:20:27
*Mme. 2:* Oui, puis alors mes grands-parents, que ce soit côté maternel et paternel, le faisaient aussi, donc [euh], surtout des fruits, pour le coup, pas trop de légumes, et voilà des- des- des fraises, des framboises voilà, des petits arbres fruitiers, des pommes. Petites pommes aussi, ça, beaucoup, des pommes.

00:20:46
*Mme. 1:* Vous en mangez beaucoup ?

00:20:46
*Mme. 2:* Beaucoup, ouais. Finalement, ce que je mange pas beaucoup, c'est les poires. Je trouve ça galère à manger, mais j'aime bien ça. [rire]

00:20:54
*Mme. 1:* Alors c'est ça justement là, qui va m'intéresser. Vous a-- dans les fruits et légumes que vous mangez pas, donc là si on prend la poire, c'est plus par [euh]- c'est parce que vous trouvez ça pas- pas- pas dire vos mots à votre place mais, vous aimez ça, mais c'est plutôt parce que--

00:21:11
*Mme. 2:* Parce que, parce que je- je- la pomme on peut la manger comme ça, et la poire, je sais pas, en fait, j'ai été élevé en la coupant, et alors j'aime pas trop couper mes fruits, donc forcément, je fais pas l'effort. C'est bête, mais c'est une question d'éducation aussi... Qu'est-ce que, qu'est-ce qu'on mange pas comme ça ? J'adore la grenade, mais pareil, c'est pénible. Donc on en achète une fois l'an, puis ça- ça m'énerve et [euh]-- Alors ce qu'on n'achète pas souvent mais qu'on aime beaucoup aussi, c'est la mangue, mais parce que voilà, c'est un fruit exotique et que, on se dit aussi que question environnement, c'est pas non plus le meilleur des fruits à acheter mais ça, on aime beaucoup. On achète peut être deux fois dans l'année. Par contre, on n'a jamais acheté fruits de la passion tout ça, même si on les aime, mais là pour le coup, la mangue, on aime beaucoup on fait l'effort, mais le reste, on s'en prive. [rire] [euh] Qu'est-ce que- qu'est-ce que- qu'est-ce que j'aime pas pour cuisiner? Si, ce qui est pénible à couper, c'est les navets et les pommes de terre douces- les patates douces, alors on en fait souvent, mais alors c'est un peu le calvaire, quoi. [rire] Pareil les pommes de terre, moi je coupe pas. J'ai une blessure en fait au pouce, j'ai déchiré un ligament, donc c'est jamais moi qui épluche les pommes de terre, c'est toujours [Prénom du conjoint]. Donc [euh] il rechigne jamais, il faut éplucher 12 pommes de terre, il les épluche, parce qu'il a une passion incroyable pour la pomme de terre. [euh] Voilà, donc [euh]-- Mais après sinon c'est tout le temps moi qui épluche les légumes et qui fais la cuisine et voilà, lui il fait les pommes de terre.

00:22:40
*Mme. 1:* OK, je vois. Et donc, donc- donc- navet, patate douce, vous consommez quand même souvent, même si, c'est galère--

00:22:49
*Mme. 2:* Oui, les navets vraiment dans la soupe en fait. Des fois je m'embête même pas à les éplucher, ils sont bio, je les coupe, je les mets dans la soupe. Les pommes- les patates douces par contre, j'épluche toujours.

00:22:56
*Mme. 1:* Et qu'est-ce qui, selon vous, fait la différence justement sur ces épluchures [euh], le fait que la patate douce vous l'épluchiez, le navet pas forcément ?

00:23:07
*Mme. 2:* Bah le navet, je le mets dans la soupe donc [euh], tout va être broyé finalement, alors que la patate douce, c'est souvent en accompagnement d'un plat, ou dans des salades ou en frites et, même sur les patates, j'aime pas du tout la- la peau, en fait, des patates à manger, genre, par exemple, les pommes de terre en robe de chambre, j'enlève tout le temps tout. [rire] Autant y a beaucoup de choses que je mange avec la peau, ça me dérange pas, les courgettes, les- les nectarines, tout ça, les pommes, y a pas de souci, mais autant ouais les pommes de terre, la peau, j'aime pas ça. [rire]

00:23:40
*Mme. 1:* D'accord. C'est par- c'est par goût que--

00:23:42
*Mme. 2:* Oui c'est par goût, je- je trouve qu'il y a un petit goût un peu amer là, enfin je sais pas comment dire, mais l'amertume là, qui ne me plaît pas vraiment, mais sinon, voilà. Alors j'aime moins aussi les- les petites pommes de terre nouvelles là, les- les petites, ça je- j'aime moins. Voilà.

00:23:59
*Mme. 1:* Par goût aussi sûrement--

00:23:59
*Mme. 2:* Par goût aussi ouais, je- une petite amertume que, enfin un goût, un arrière-goût qui ne me plaît pas. Si vous avez compris, en fait, c'est moi qui décide de ce qu'on mange aussi. [rire]

00:24:10
*Mme. 1:* Ça a l'air, sauf quand [Prénom du conjoint] achète 60 kg de patates et qu'il veut bien préparer à manger. [rire]

00:24:15
*Mme. 2:* C'était une arnaque, en fait. C'est passé dans le journal et tout.

00:24:18
*Mme. 1:* Ah ouais ?

00:24:18
*Mme. 2:* Oui oui, 3€20 le kilo, ça fait cher la patate ! [rire]

00:24:23
*Mme. 1:* Ah oui, oui.

00:24:26
*Mme. 2:* [rire] Non non, mais on en a bien ri. Tout le monde s'est bien moqué de lui, le pauvre. On a mangé des patates bien pendant, huit mois quoi, au moins.

00:24:34
*Mme. 1:* Ah oui ? Ça faisait des patates à écouler, ouais. [rire]

00:24:38
*Mme. 2:* [rire] Ouais voilà, 60 kilos.

00:24:48
*Mme. 1:* Ouais. Bah c'est, c'est un humain. [rire] Et du coup, vous avez dit aussi, donc les endives, le chou-fleur--

00:24:53
*Mme. 2:* Oui, ça je, alors-- En fait, j'ai réellement jamais goûté, mais j'ai- j'ai une aversion pour ça depuis toute petite, je trouve ça vraiment, ça ne m'attire pas du tout et j'ai pas pris la peine de re-goûter étant adulte.

00:25:02
*Mme. 1:* Les deux ou-- ?

00:25:05
*Mme. 2:* Les deux, alors qu'y a plein de-- Alors, petite je mangeais presque rien comme légume, autant les fruits, j'ai jamais eu de souci, mais légumes, je mangeais pas d'haricots verts, pas de tomates, pas de-- rien en fait, que des pâtes et du jambon. Et en étant étudiante, j'en avais un peu marre de manger que des pâtes, et j'ai fait l'effort de goûter d'autres choses. Donc peut-être un jour, j'adorerai les endives et j'adorerai le chou-fleur, mais progressivement, voilà. [rire]

00:25:29
*Mme. 1:* Ouais, je vois. Et du coup, chou-fleur, essayez-les. Asperges, pareil--

00:25:33
*Mme. 2:* Alors ça les asperges non, ça j'aime pas du tout. Et endives, c'est- c'est l'odeur à la cuisson qui me dérange.

00:25:41
*Mme. 1:* D'accord, c'est les endives cuites, du coup. Et crues, ça vous-- ?

00:25:43
*Mme. 2:* J'ai jamais goûté, mais alors crues, ça me dérangerait pas, je pense. Et chou-fleur pareil, c'est l'odeur à la cuisson. Je- je suis vraiment très sensible aux odeurs, donc par exemple même si je fais une soupe, et tous les aliments, je les aime dedans, mais elle sent bizarre, je la mangerai pas. Je- j'y arriverai pas, en fait, je- voilà. [rire]

00:26:03
*Mme. 1:* OK.

00:26:04
*Mme. 2:* C'est un peu ridicule et enfantin, mais [euh]--

00:26:08
*Mme. 1:* Ben non non, c'est intéressant. Je veux dire, par exemple, moi je sens rien, donc je peux jamais me fier à mon odorat [rire] que vous, si--

00:26:17
*Mme. 2:* Même des aliments que j'ai l'habitude de manger, s'il y a un soupçon d'odeur qui ne me plaît pas, je le mangerai pas.

00:26:23
*Mme. 1:* D'accord, c'est avec l'odeur que vous-- c'est intéressant. Et par exemple le chou-fleur cru, pareil ?

00:26:28
*Mme. 2:* Chou-fleur cru, je pense que je pourrais.

00:26:30
*Mme. 1:* D'accord, mais vous faites pas, du coup, vous faites pas quoi. Mais bon--

00:26:33
*Mme. 2:* Je fais pas. Non, ça ne me viendrait pas à l'idée d'acheter. Alors par contre, chose que je ne me serais jamais vue faire à part- depuis l'an dernier, enfin avant l'an dernier, c'était acheter du brocoli et du chou romanesco. Et en fait j'en ai goûté à la crêperie dans une préparation, j'ai trouvé ça super bon, j'en ai racheté, j'ai trouvé ça super bon. Mais je les mangerais pas tout seuls, voilà. [rire]

00:26:51
*Mme. 1:* D'accord. Un plat, bien préparé.

00:26:54
*Mme. 2:* Dans une préparation, ouais voilà. Pareil, les aubergines. Tout seul, j'aime pas du tout, voilà. Mais je- je- dans un plat, de la ratatouille, dans le- dans d'autres préparations en fait où y a d'autres légumes en composition, ça, c'est pas un souci.

00:27:12
*Mme. 1:* D'accord, je vois. Et les blettes c'est quoi, c'est pareil, c'est plutôt-- ?

00:27:16
*Mme. 2:* Les blettes, c'est le mot. [rire] Horrible. C'est très proche de blatte, et ça me plaît pas. [rire]

00:27:25
*Mme. 1:* Ouais, c'est vrai. Sinon, par exemple on peut dire "bête", aussi mais [euh]--

00:27:27
*Mme. 2:* C'est pire encore ! [rire]

00:27:28
*Mme. 1:* [rire] Mais c'est- ouais c'est pas, c'est pas vraiment mieux. Sincèrement, c'est vraiment le mot qui vous- qui vous déplaît dans le-- ? Ça vous fait un blocage-- ?

00:27:38
*Mme. 2:* Alors, je pense à une autre chose, ça ressemble énormément à épinards, et j'aime pas non plus les épinards. [rire] Mais ça, j'ai jamais goûté non plus. C'est juste que je d-- alors, si, les épinards frais comme ça, comme ça, alors cuits pareil, ils ont une sale tête, [rire] ils me plaisent pas. En fait, j'ai un souvenir de- des épinards à la crème, en fait, on aurait dit des épinards dans de la purée, au collège. Et [euh], ça passe plus jamais depuis. Cette vision d'horreur, des épinards dans de la purée, enfin dans je ne sais pas quoi hein, mais [euh], non, je ne peux pas. Mais peut-être un jour, je ne-- [rire]

00:28:13
*Mme. 1:* Je vois. [rire)

00:28:17
*Mme. 2:* Ça, c'est un peu comme un enfant, c'est la diversification alimentaire à 23 ans, c'est-à-dire que je mangeais vraiment que des pâtes et du jambon, à 12 ans. Et puis depuis, chaque année allez, on va dire dix aliments rentrent dans- dans-- [rire] Je- je n'ai- je ne peux que progresser. [rire]

00:28:38
*Mme. 1:* [rire] C'est ça.

00:28:38
*Mme. 2:* Déjà, je mange des fruits et légumes. Ma cousine n'en mange aucun. Même les jus de fruits, elle peut pas quoi. [rire]

00:28:49
*Mme. 1:* Que vous, oui ?

00:28:49
*Mme. 2:* Alors moi, j'adorais le jus de fruits.

00:28:50
*Mme. 1:* D'accord.

00:28:50
*M. 1:* Bonjour

00:28:50
*Mme. 1:* Bonjour [rire]. OK, et donc [euh] est-ce qu'on a fait le tour des fruits ? Donc c'était bananes, vous m'avez dit, voici après tous les fruits [euh]--

00:29:07
*Mme. 2:* Alors moi tous les fruits, pas de soucis. J'aime un peu moins les litchis, j'en raffole pas, mais j'en mange. Et ce que j'aime pas du tout, par contre, c'est les bananes trop mûres, b- toujours lié à l'odeur, je ne peux pas. Ça me bloque. Les bananes pas mûres, c'est pour moi, les bananes trop mûres, c'est pour [Prénom du conjoint].

00:29:23
*Mme. 1:* D'accord donc ça c'est- c'est marrant, vous avez pas forcément des fois les mêmes goûts sur la maturité des--

00:29:29
*Mme. 2:* Non, c'est ça. [Prénom du conjoint] les aime pas quand elles sont pas mûres, et moi, je les aime pas quand elles sont trop mûres.

00:29:33
*Mme. 1:* [rire] D'accord, bon ben comme ça, c'est pratique. [rire] Faut pas que vous les mangiez toutes avant, quoi. [rire]

00:29:38
*Mme. 2:* Non, en général j'arrive quand même à lui en laisser.

00:29:40
*Mme. 1:* OK, chouette. Alors [euh] du coup, j'aimerais qu'on s'intéresse à vos différents lieux de stockage, donc les différents contenants où vous entreposez vos fruits et légumes. Donc quand vous revenez de vos courses, qu'est-ce que vous faites de vos fruits, par exemple ?

00:30:01
*Mme. 2:* Alors, en général, ils sont dans des sacs à vrac, déjà, donc j'enlève déjà les étiquettes sur mes sacs à vrac. Ensuite en règle générale, je les lave, pratiquement tous, je veux dire enfin les- les fruits et les- enfin surtout les- les- légumes en fait, pour que je les mette dans mon bac, dans le bas du- du frigo. Donc ça c'est pour ceux qui sont pas emballés. [euh] Donc je mets tout dans le bac voilà, enfin les fruits, genre pas les pommes de terre et les carottes, forcément, mais tout ce qui passe vite, je les mets dans le bac. Les pommes de terre, je les laisse dans le sac, je les mets donc, dans un endroit sombre. [euh] Les fruits, je les mets dans le panier à fruits, je m'embête pas. Ça arrive même qu'ils restent dans le sachet, donc le sac à vrac, je sais que je vais les manger rapidement. Y a juste voilà, les framboises et les fraises, les myrtilles, tout ça, je les lave directement et je les mets dans un bol qui est sur la table parce qu'il faut les consommer tout de suite. [euh] Quoi donc ? [euh] Les oignons, ils ont un petit compartiment aussi, un petit bol, donc oignons, ail, échalotes. Quand je fais des tartes aux oignons de Roscoff, j'ai plein d'oignons roses, du coup, je les mets dans un sac à vrac qui a des petits trous, et je le mets derrière la porte. Voilà, et après quand j'ai des fruits et légumes qui sont emballés, par exemple le bio du supermarché, ça arrive assez souvent, je les mets directement dans le frigo, avec l'emballage... Sauf si je vois que c'est un peu humide à l'intérieur. Dans ce cas là, je l'enlève et je les, voilà-- ça dépend de comment je sens l'emballage. [rire]

00:31:30
*Mme. 1:* Je vois.

00:31:30
*Mme. 2:* Parce que ça les courgettes, quand c'est- le carton est un petit peu humide, y a plus de courgettes au bout de deux jours. [rire]

00:31:40
*Mme. 1:* Donc, vous avez plusieurs lieux, il y a la corbeille à fruits, voir pour les petits fruits, les petites baies qu'on met direct sur la table. [euh] Il y a l'endroit à pommes de terre... y a- et patates douces, y a le petit compartiment pour mettre les oignons, l'ail et les échalotes ensemble, [euh] y a le bac à frigo, pour mettre les légumes, on a dit, pour éviter qu'ils passent vite. [euh] Et voilà. Et le sac avec les petits trous, c'est quoi ?

00:32:19
*Mme. 2:* [euh] Je vais vous montrer. Il faut que je trouve !...Comme ça, et on peut mettre des salades aussi normalement, mais moi, du coup, je les mets comme ça, et comme ça, je les vois quand même. Et je sais que j'ai des oignons, quoi. Parce que si je mets un sac où y a pas de petits trous et-- [rire]

00:32:39
*Mme. 1:* Oui, c'est vrai qu'on--

00:32:42
*Mme. 2:* Ça arrive aussi qu'on achète en vrac des pommes de terre, qu'elles aillent là-dedans, plutôt.

00:32:46
*Mme. 1:* D'accord.

00:32:48
*Mme. 2:* On en a deux comme ça, donc [euh]--

00:32:50
*Mme. 1:* C'est des petits sachets, c'est des- des filets quoi, un peu--

00:32:53
*Mme. 2:* Oui des sacs à vrac, mais normalement c'est spécial pour la salade. Mais, on achète rarement de la s- alors on achète rarement de la laitue, parce qu'on n'est pas des gros consommateurs de salade [euh] verte comme ça, celle qu'on a comme ça là et qu'après on recoupe. Et parce que jusqu'à il y a très peu de temps, on n'avait pas de panier à salade, et j'en voyais pas l'utilité. Et ma belle-mère nous a acheté un panier à salade, parce qu'elle trouvait ça inadmissible qu'on n'en ait pas, et par contre ce qu'on achetait souvent, c'est de la mâche. On achète en fait en sachet, par contre. Du coup, elle est déjà toute lavée, voilà.

00:33:32
*Mme. 1:* OK.

00:33:33
*Mme. 2:* Moi en général j'achète plutôt de la mâche pour me donner bonne conscience, c'est pas ce dont je raffole et puis j'en mets un petit peu à côté de mon plat, résultat, j'ai mangé deux feuilles et c'est trop bien, j'ai mangé de la salade. [rire]

00:33:44
*Mme. 1:* [rire] Je vois. Donc ouais donc c'est aussi pour ça, les endives enfin c'est pas- les endives crues, c'est pas dans vos habitudes de consommer en fait. Beaucoup de salade à côté. Parce que l'endive crue finalement elle fait aussi des fois ce--

00:33:55
*Mme. 2:* Ce- cette fonction.

00:33:59
*Mme. 1:* Oui voilà, c'est ça.

00:33:59
*Mme. 2:* Ce qu'on fait beaucoup c'est des salades, mais [euh] avec par exemple, des lentilles. Et là, je vais mettre des petites tomates, des concombres, un peu de carottes. Là, on va vraiment manger du cru. Mais sinon voilà, le- le cru, moi, j'ai jamais eu ça dans ma famille par exemple, mes parents ont jamais fait d'entrée, alors que la mère d'[Prénom du conjoint] tous les midis, c'est salade, genre petites carottes crues, tous les midis, tous les midis, tous les soirs. Et moi, c'est pas du tout dans- dans mon éducation, et je le fais pas donc [euh] voilà, mais je sais qu'il faudrait que je le fasse, c'est bien pour la santé, mais voilà. J'ai déjà du mal à faire un plat, alors en plus faire une entrée. [rire]

00:34:33
*Mme. 1:* Ouais vous êtes plutôt tendance à consommer les légumes cuits que crus, même si ça peut vous arriver de faire des salades un peu en--

00:34:45
*Mme. 2:* C'est ça, mais plus en été du coup, les salades. Ou quand je suis en déplacement, c'est vrai que c'est facile d'avoir une salade quoi, parce qu'on n'a pas souvent un micro-onde sous la main, donc [euh] voilà.

00:34:55
*Mme. 1:* Oui, c'est sûr. OK... [euh] alors en termes de nettoyage, est-ce que ça vous arrive de les nettoyer ? Ces- les différents contenants qu'on a cités, ou pas vraiment ? Voilà, comment vous vous organisez là-dessus ?

00:35:07
*Mme. 2:* Le bac à légumes c'est toujours [Prénom du conjoint] qui fait, donc je sais pas combien de temps, enfin tous les combien de temps il fait. J'ai eu des gros soucis d'épaule et je me suis fait opérer en février, et c'est des mouvements que je-- aller chercher, je pouvais pas, en fait, c'est des mouvements que je pouvais pas faire. Donc maintenant j'ai plus aucune raison de ne pas le faire, mais [euh] j'ai perdu l'habitude [rire] et par contre, voi- en fait, moi, je fais à manger et [Prénom du conjoint] fait la vaisselle, donc forcément en fait moi, je mets tout dans l'évier, et [euh] voilà. Donc je me pose pas trop la question de quand est-ce que c'est lavé ? À un moment, je dis : "ça, il faut que ce soit lavé", je mets dans la- dans l'évier, et c'est lavé. Et, magie. [rire]

00:35:44
*Mme. 1:* [rire] La magie opère. OK.

00:35:47
*Mme. 2:* Non, mais les f-- par exemple, ben y a des fruits aussi. Par exemple les kiwis, ils perdent plein de petits poils, bah quand on a fini le kiwi, c'est sûr qu'on met le contenant de fruits et légumes à laver. Mais si par exemple y a eu [euh] des pommes pendant un jour, je vais pas forcément le laver. Surtout si elles sont bio, quoi.

00:36:04
*Mme. 1:* OK. Donc la corbeille, c'est plus quand elle est un peu sale, qu'on se dit [euh]--

00:36:07
*Mme. 2:* C'est ça, oui. Bon après, c'est quand même une fois toutes les semaines quoi, ou toutes les deux semaines max quoi.

00:36:11
*Mme. 1:* OK, donc [euh] c'est quand même [euh]--

00:36:14
*Mme. 2:* Là vous allez voir l'état de notre cuisine, vous allez dire : "c'est pas possible, vous nettoyez pas cette cuisine", mais si si, c'est juste que- on nettoie tout d'un coup, en une semaine. [rire]

00:36:23
*Mme. 1:* C'est ça. Et moi d'un jour à l'autre, ma cuisine n'est pas la même. [rire]

00:36:26
*Mme. 2:* Nous c'est même peut-être d'un repas à l'autre. [rire]

00:36:30
*Mme. 1:* [rire] C'est ça. Je comprends. [euh] OK, donc y a la corbeille et après, sinon les autres contenants, passons là le sac à accrocher, et le bol à oignons [euh]--

00:36:44
*Mme. 2:* Ah celui-là il est jamais lavé par contre, le bol à oignons. [rire] C'est l'oublié. En fait c'est- c'est même pas un bol, c'est une petite corbeille en- en-- c'est pas du rotin, mais vous savez là, les- les trucs où on met les légumes normalement là les-- je sais pas comment dire les-- c'est un peu du bois mais [euh], enfin vous voyez ce que je veux dire là, les barquettes quoi les-- ouais, c'est- c'est ça en fait. Pour le- le- le- les oignons, c'est ça. Par contre les sacs à vrac, bon ben, quand je reviens des courses, que j'ai vidé mes légumes, ils vont direct à la machine. Voilà.

00:37:14
*Mme. 1:* OK, les sacs comme ça. En fait, les sacs à vrac sont en tissu--

00:37:16
*Mme. 2:* Ils sont en tissu, et [euh] quand j'ai vidé mes courses dedans ou quand y a plus rien dedans, par exemple les oignons, ben direct à la machine. Je me pose pas de questions, ils sont pas réutilisés, ils sont tous lavés à- à 30°C selon leur état, ou ça va même des fois jusqu'à 90°C quand y a eu de la terre ou-- Ça de laver assez chaud quand ça p, de faire un décrassage de la machine. à laver en même temps [rire]

00:37:40
*Mme. 1:* [rire] Donc [euh] ouais finalement, vos sacs de vrac vont-- ne touchent pas-- enfin les légumes [euh] qui sont achetés à ce moment-là, vont pas êt- vont pas toucher ceux d'avant, etc. Votre sac, il est--

00:37:51
*Mme. 2:* Non non, ils sont toujours lavés. Même mes sacs de courses, je veux dire, les sacs, les tote bags, tout ça, je fais les courses avec, ils passent avec les-- ça m'arrive. Je me dis, un de plus, un de moins, ça va pas... S'il y a eu un peu de terre dedans ou-- bien que ce soit pas... Mais voilà, c'est vrai que ça... En fait je- je- voilà, je m'occupe des courses, de faire à manger et de faire les lessives, donc forcément en fait, mon cycle il est là-dedans quoi. [rire]

00:38:18
*Mme. 1:* Donc ouais le tote bag (euh], il part [euh] il part à la machine.

00:38:22
*Mme. 2:* On fait pas souvent hein, mais [euh]. Comme effectivement, on fait des grosses courses toutes les deux semaines, ben ça fait une machine toutes les deux semaines quoi, avec autre chose, des torchons ou des draps et-, voilà.

00:38:35
*Mme. 1:* OK, ça marche. Alors maintenant, si on discute de votre façon de consommer les fruits et légumes. Alors vous en avez déjà parlé [euh] pas mal. Si on prend les fruits, si on prend un fruit que vous consommez régulièrement. Prenons la pomme, qu'on retrouve pas mal. La pomme, comment vous la consommez ? Tout à l'heure vous m'avez dit que par rapport à la poire, vous aimiez bien la manger [euh]--

00:39:05
*Mme. 2:* Oui je la mange comme ça, comme ça le plus souvent. Quand elles sont un peu passées, parce que s- j'ai oublié de les manger, ou parce qu'il y avait un fruit plus intéressant ou que- [euh] en règle générale, on fait de la compote, ou des pommes au four. [euh] Qu'est-ce qu'on fait sinon ? Je fais un peu de pâtisserie donc parfois, ça passe aussi dans les pâtisseries. [euh] Je réfléchis, qu'est-ce qu'on fait avec nos pommes ?... Dans les yaourts. Je les fais cuire un petit peu pour qu'elles soient molles, et puis je les mets dans les fond des yaourts avant de passer à la yaourtière. Voilà. C'est relativement l'utilisation qu'on a des pommes.

00:39:43
*Mme. 1:* Donc plutôt crues de base, mais après pas mal de--

00:39:46
*Mme. 2:* 70% elles sont mangées crues, quoi.

00:39:51
*Mme. 1:* OK. Mais ça vous arrive de les- de cuisiner [euh] ?

00:39:53
*Mme. 2:* Oui, des fois quand elles sont pas bonnes aussi, quand elles ont pas un goût agréable, elles passent en compote quoi.

00:39:59
*Mme. 1:* Est-ce qu'il y en a d'autres, des fruits que vous pouvez cuisiner, ou- ou non ? Est-ce que vous pensez à d'autres fruits ?

00:40:07
*Mme. 2:* La banane, ça arrive. [Prénom du conjoint] les passe aussi souvent-- parce que du coup, je les mange pas mûres, lui il les mange trop mûres, mais des fois, ça arrive qu'elles soient vraiment, beaucoup trop mûres. Et là, aucun de nous deux en veut, donc ça passe dans la compote de pommes. [euh] Ça nous est déjà arrivé de faire des banana breads aussi pour les passer. Et ça arrive clairement qu'elles passent à la poubelle. Quand on a vraiment pas le temps. Puis moi comme je vous dis voilà, l'odeur de la banane, si c'est moi qui dois cuisiner, je dis [euh], non. [rire] Voilà. Non je suis vraiment-- aux odeurs enfin c'est- c'est vraiment un blocage quoi, mais vraiment, c'est presque maladif quoi.

00:40:40
*Mme. 1:* Ouais les odeurs du cuit [euh]--

00:40:42
*Mme. 2:* Les odeurs du cuit, du trop- trop mûr, de tout [euh] enfin-- ouais, vraiment. Mais c'est comme ça pour tout dans la vie, toutes les odeurs. Voilà.

00:40:51
*Mme. 1:* Ouais. Une sensibilité... OK. Et si on prend les légumes, donc vous avez dit en fait déjà, si on prend la courgette. Donc, vous êtes plutôt du genre à consommer vos légumes cuits. Alors si on prend la courgette allez-y, vous pouvez me dire sous quelle forme vous--

00:41:14
*Mme. 2:* Alors, en soupe. En dés, et là je la place dans un curry ou dans une ratatouille. Dans quoi je la fais ? Sautée [euh]... Courgette- j'ai fait aussi des courgettes farcies, ça m'est arrivé. En règle générale, le plus souvent, c'est vraiment-- je fais des- je fais- je fais un curry de courgettes. Avec du riz, une sauce curry. C'est vraiment-- peut-être une fois toutes les deux semaines, en été, c'est ça. [rire]

00:41:23
*Mme. 1:* Ça a l'air pas mal. [rire]

00:41:49
*Mme. 2:* Ou avec un dahl, en accompagnement d'un dahl.

00:41:53
*Mme. 1:* C'est pas mal non plus, ça. [rire] OK. Et globalement ouais y a -- et par contre la tomate par exemple, si on prend la tomate ?

00:42:05
*Mme. 2:* Tomate, la- la- la fraîche ?

00:42:05
*Mme. 1:* Ouais.

00:42:06
*Mme. 2:* La fraîche en [euh] comme ça. Ça dépend si elle vient du Sud ou si elle vient du supermarché. C'est clairement pas la même, c'est la tomate gorgée de soleil, c'est quand même-- c'est dommage de les cuire, clairement. Mais sinon [euh] voilà-- quand j'étais chez mes parents, c'était clairement en entrée avec de l'huile d'olive. C 'est- c'est effectivement, peut-être le seul qu'on consommait cru, voilà. Tomates avec de l'huile d'olive. Sinon ici, ça va plutôt dans des- dans des préparations, genre dahl, un- un curry où je rajoute des tomates, une ratatouille, des tartes aussi, tomates chèvre. Dans quoi ? Dans des soupes. En été, je mets souvent des fraîches aussi (euh], des pizzas.

00:42:52
*Mme. 1:* Yes, des pizzas.

00:42:55
*Mme. 2:* Il est sorti, le mot. [rire] Voilà donc pour les fraîches, voilà. Après par contre, la tomate je veux dire, en coulis ou en- en- conserve clairement, j'en utilise bien trois fois par semaine.

00:43:09
*Mme. 1:* Ah ouais ?

00:43:09
*Mme. 2:* Ouais, ben pareil dans mes préparations, dans une soupe ou dans un dahl, dans- dans un- dans un chili sin carne, enfin voilà, je- je passe assez souvent de- voilà de, c'est des tomates, vous savez les-- c'est sans peau, y a quand même de la sauce avec, ouais voilà c'est ça, tomates pelées, exactement.

00:43:26
*Mme. 1:* Qui se conservent du coup, enfin qui sont en conserve.

00:43:32
*Mme. 2:* C'est bien pratique quand clairement on fait un truc un peu rapide, simple, hop, dans le dahl.

00:43:38
*Mme. 1:* Ouais, je vois.

00:43:38
*Mme. 2:* Avec la sauce et tout. Ça permet de cuire aussi, puisqu'il y a quand même du jus, et voilà.

00:43:46
*Mme. 1:* C'est dans vos sauces, quoi. Vos plats en sauce souvent, va y avoir ça.

00:43:49
*Mme. 2:* Oui, y a de la tomate.

00:43:51
*Mme. 1:* Ouais, OK. Alors, sur les-- En fait, nous on se pose des questions sur les épluchures, et on se demande comment les personnes épluchent ou non. Alors, il me semble que vous en avez déjà parlé. Qu'est-ce que vous épluchez ? Qu'est-ce que vous épluchez peu ? J'ai plus mes notes.

00:44:13
*Mme. 2:* Les pommes de terre !

00:44:14
*Mme. 1:* Ah oui, là, "aime pas la peau, c'est amer". Donc oui, vous épluchez les pommes de terre, les patates douces.

00:44:20
*Mme. 2:* Oui, par contre, quand- quand elles sont sombres, en général j'épluche presque jamais si c'est bio. Quand c'est bio, en rè-- ouais j'épluche jamais en soupe. Après voilà, quand c'est pas bio, j'épluche toujours.

00:44:32
*Mme. 1:* OK, est-ce que vous pouvez me raconter pourquoi ?

00:44:35
*Mme. 2:* Pour les pesticides voilà après, j'épluche pas le bio parce que dans la peau, y a plein de bonnes choses pour la santé.

00:44:42
*Mme. 1:* OK.

00:44:47
*Mme. 2:* ...Les fruits et légumes, à part les pêches, [euh] j'épluche pas grand chose, je ne crois pas. Enfin si, les kiwis, les bananes, et voilà. Mais parce que ça, ça ne se mange pas, mais après les fruits où on peut manger la peau, non honnêtement, je mange la peau partout quoi, sauf voilà- sauf les pêches. Je sais même pas si ça se mange d'ailleurs, la peau de pêche.

00:45:09
*Mme. 1:* Et vous épluchez la peau des pêches ?

00:45:13
*Mme. 2:* Oui. Bien que ça a dû m'arriver de les manger avec la peau. [rire]

00:45:17
*Mme. 1:* [rire] C'est curieux, quelqu'un m'a dit ça, ça. Et ça m'a-- Après c'est les habitudes qu'on a enfant ou-- Et pour moi, je savais même pas qu'on pouvait éplucher, enfin-- je, enfin si, toutes les peaux s'épluchent en soi, mais [euh], mais y en a qui m'ont dit que c'était une aversion, la peau des pêches.

00:45:35
*Mme. 2:* Moi j'ai- j'ai-- ma cousine, c'est ça, elle peut même pas toucher une peau de pêche.

00:45:38
*Mme. 1:* Ou bien y en a, c'est le kiwi, ou-- Pourtant une peau de pêche--

00:45:48
*Mme. 2:* C'est doux.

00:45:48
*Mme. 1:* Ben oui [rire], l'expression est plutôt positive. [rire]

00:45:48
*Mme. 2:* Ah non mais je- je pense que, en vrai, je pense que j'en achète moins parce que je préfère les nectarines, mais [euh] je crois que ça doit m'arriver de les manger avec la peau parce que j'ai tellement la flemme de les éplucher.

00:45:56
*Mme. 1:* Donc sinon [euh], sinon les fruits à part les peaux qui se-- qu'on estime pas consommables, le kiwi, la banane, est-ce qu'il y en a d'autres ?

00:46:13
*Mme. 2:* La mangue, ça on enlève aussi. Les litchis, faut enlever aussi. Bon après voilà, les fraises, les framboises, tout ça, bon y a pas vraiment de difficulté. [rire]

00:46:22
*Mme. 1:* Ce serait dommage. [rire]

00:46:23
*Mme. 2:* "J'ai enlevé toute la peau !", "Mais qu'as-tu gardé ?", "Il reste la feuille !", "Ah, d'accord"

00:46:30
*Mme. 1:* Ah les agrumes ?

00:46:32
*Mme. 2:* Oui, les agrumes. Ben oui, alors là clairement, ben oui, les mandarines, je les épluche. [rire] Ça va être compliqué sinon. Les pamplemousses...

00:46:47
*Mme. 1:* Et quand vous avez votre courgette-- est-ce que vous avez des fruits, enfin plutôt dans des légumes, prenons des légumes, que vous avez préparés, et sans- sans qu'il y ait des épluchures ? Donc que ce soit la peau, ou même la pomme, y a le trognon. Après, peut-être que vous le mangez ?

00:47:04
*Mme. 2:* Oui.

00:47:08
*Mme. 1:* Donc vous dans la pomme, si elle est bio, y a pas d'épluchures. Donc par exemple ?

00:47:11
*Mme. 2:* Je mange tout. La pomme, les aubergines, quand-- même si on aime pas trop ça quand j'en fais des- des-- en général j'enlève pas la peau, quand je les fais au four. La courgette, quand je la fais farcie, pareil. [euh] Quoi d'autre ? Les pommes de terre, quand mes parents viennent, on laisse la peau parce qu'ils aiment bien la peau. [euh] Je réfléchis mais... Plein de choses qu'on fait. Les navets dans la soupe, ouais dans la soupe on met en général-- la peau reste, donc [euh].

00:47:40
*Mme. 1:* C'est ça, non mais dans le- dans la courgette, y a-- même si vous gardez la peau, souvent on enlève les extrémités.

00:47:50
*Mme. 2:* Oui, ça j'enlève ouais, mais je fais exprès d'enlever le plus petit bout possible.

00:47:52
*Mme. 1:* Le plus petit bout possible. [rire]

00:47:54
*Mme. 2:* C'est ça pour tous les légumes, on a d'ailleurs acheté un économe exprès, qui enlève le moins de bout possible.

00:47:59
*Mme. 1:* Ah ouais ?

00:48:02
*Mme. 2:* Oui. C'était notre-- Dans le magasin : "on voudrait un éplucheur qui enlève presque pas la peau", "comment ça ?", "juste le dessus"--

00:48:05
*Mme. 2:* Les épluchures sont pas les mêmes.

00:48:14
*Mme. 2:* Ah non mais c'est, en fonction des économes c'est clair, y en a ils enlèvent un millimètre quoi, on se dit mais "wow !". [rire] Non, non non là, on en a un qui marche plutôt bien. Les tomates ! Ça je les mange en entière. Y en a qui enlèvent la peau, je cherche pas à savoir. Y en a qui enlèvent la peau et tout des tomates, moi j'enlève pas la peau. Mais pour des questions de digestion je crois, y en a qui digèrent pas les tomates, les poivrons, tout ça, enfin avec la peau. Si les poivrons, les poivrons j'enlève les pépins, mais sinon, j'enlève pas la peau quoi, mais je suis pas sûre qu'on puisse. Je réfléchis [rire], ça va être compliqué.

00:48:46
*Mme. 1:* À l'intérieur y a une peau fine, à l'intérieur du-- comme un oignon, mais-- ...Non je pense pas [rire. ] Mais les pépins, ouais les pépins...et les tiges, les petites tiges.

00:48:57
*Mme. 2:* Les pépins, on est obligé. Et les- les membranes blanches là, aussi.

00:49:03
*Mme. 1:* Et [euh] le poireau, que vous consommez beaucoup l'hiver. Qu'est-ce que vous consommez dedans ? Qu'est-ce que vous ne consommez pas, à votre avis ?

00:49:12
*Mme. 2:* Alors avant, je prenais que la partie blanche en me disant, "le vert ne se mange pas", et maintenant, honnêtement, je prends les trois quarts, et j'enlève vraiment les bouts qui sont vraiment abîmés quoi. Enfin, vraiment le dessus là, où c'est pas très, très beau et pas très bon. Et du coup, la partie verte, en règle générale, je la mets dans la soupe, et la partie blanche j'en fais une tarte, ou sautée à la poële, voilà.

00:49:35
*Mme. 1:* OK. Et [euh] alors vous l'avez pas dit, donc je sais pas si vous en consommez. Les champignons ?

00:49:41
*Mme. 2:* J'ai une horreur phobique des champignons. J'en parle même pas. [rire] C'est pire que tout. Le pire du tout, c'est le champignon. [rire] Ça me viendrait même pas à l'idée en fait, d'en avoir chez moi.

00:49:54
*Mme. 1:* Ah ouais ?

00:49:57
*Mme. 2:* Autant les blettes, je les tolère dans ma cuisine, autant les champignons, c'est dur. Enfin, je le toucherais même pas en fait, je ne pourrais pas.

00:50:01
*Mme. 1:* C'est rigolo. Est-ce que vous savez du coup d'où vient cette- cette aversion ?

00:50:07
*Mme. 2:* Pas du tout. Depuis toute petite, le champignon, ça ne passe pas. Mais du coup tous mes amis se- se moquent de moi, parce qu'on est allés une fois à la- à la- [rire] comment ça s'appelle ? La- le- le salon des expositions là, la foire gastronomique, je sais plus comment on appelle ça là, à Brest. Y a un gros truc en fait, où on peut manger plein de choses et tout, en novembre là, c'est trop bien parce que, y a plein de choses qu'on peut goûter et tout, et y avait un stand avec de la crème aux champignons, tous mes amis étaient là : "mais vas-y goûte" et tout, j'ai fait "mais jamais je goûterai ça", jamais genre rien que de vous voir en manger, je suis dégoûtée quoi. Et- et on est à un autre stand, et puis y a du saucisson et puis je me dis : "oh bah je vais goûter le saucisson", un saucisson aux cèpes, j'ai cru que j'allais décéder sur place. C'est tout juste si j'ai pas vomi en plein milieu de la salle [rire]. Terrible. J'étais pas bien, je transpirais, c'était une horreur quoi. Le goût, le goût, l'odeur. En fait, j'ai senti, genre, j'ai mis dans ma bouche et j'ai fait : "ah non"-- Je l'ai quand même avalé, parce que j'allais pas recracher devant tout le monde, c'est pas l'envie qui m'en manquait. [rire] Alors après du coup, j'ai mangé plein de trucs pour faire passer-- parce que le goût restait-- je ne peux pas. Mais même dans les- les nems, je trie. [rire]

00:51:26
*Mme. 1:* Ah oui, ça ne- ça ne passe pas. [rire]

00:51:34
*Mme. 2:* Le mot "champignon" me-- non.

00:51:34
*Mme. 1:* Ouais du coup, après ce- ce dégoût, il se- il s'étend au- à l'apparence visuelle d'un champignon cru.

00:51:42
*Mme. 2:* Même le mot, même le mot "champignon". [rire]

00:51:45
*Mme. 1:* C'est vrai que celui-là aussi, il est pas-- c'est pas des plus-- parce que les champignons c'est aussi enfin là des champignons y a des jolis champignons, que moi je vais trouver jolis et bons, et y a les champignons, de l'ordre de la bactérie, que je maîtrise pas trop-- Ouais c'est pas le plus beau terme non plus, c'est vrai.

00:52:05
*Mme. 2:* Non, non mais c'est vrai que le champignon, c'est vraiment-- Alors là, pour le coup, je pense que jamais je n'en mangerai. Genre autant les blettes, le chou-fleur, je veux bien faire un effort, mais le champignon c'est--

00:52:16
*Mme. 1:* Là non. C'est vrai que ça a un goût quand même, au niveau goût une texture un peu particulière.

00:52:22
*Mme. 2:* Rien que le mot "sauce forestière" me donne des sueurs. [rire] Quand je lisais ça sur les menus de la cantine, genre "oh non !".

00:52:31
*Mme. 1:* Mais pourquoi ?

00:52:32
*Mme. 2:* Mais c'était du poulet. [rires]

00:52:38
*Mme. 1:* OK bon ben je vois. [rire] Je vois pour le champignon.

00:52:41
*Mme. 2:* Mais y en a jamais-- alors si, y a eu des boîtes, de champignons, parce qu'on a des amis qui adorent ça, et on a fait une soirée pizzas, et ils nous ont dit : "on vient pas chez vous si y a pas une pizza avec du champignon". Du coup, on a acheté des boîtes. Et il doit rester une, qui restera là toute sa vie.

00:52:54
*Mme. 1:* Sauf si elle reçoit des pizzas. [rire]

00:52:59
*Mme. 2:* Sauf si elle reçoit des pizzas. [rire] Voilà. Non, non je- c'est bizarre hein, mais non les champignons je ne peux pas. Mais j'ai beaucoup de blocages alimentaires, en fait, j'ai pas-- J'ai- je pense que j'ai un truc à régler avec des aliments, dans ma vie. Je mange pas de fromage, je mange pas de viande, à part du poulet. Et même- j'aimais le jambon petite, et maintenant je ne l'aime plus, le jambon. [rire] Parce qu'en fait si je suis végétarienne ou flexitarienne, c'est parce que je n'aime pas la viande. [rire] Mais du coup, je fais genre, je sauve la planète, je mange pas de viande. [rire)

00:53:32
*Mme. 1:* Pourquoi pas en profiter, du coup. [rire] OK. Alors si là, on- on s'intéresse au moment où vous jetez un fruit et légume, ou une partie seulement, quand ça arrive, pourquoi vous décidez de le jeter, plutôt que de le consommer ?

00:53:48
*Mme. 2:* Parce qu'il y a des champignons dessus. [rire] Non parce que [euh], soit c'est moisi clairement on le voit, y a de la pourriture enfin voilà, en général j'essaye de sauver ce que je peux mais-- souvent c'est du jour au lendemain, c'est comme je vous dis c'est, on comprend pas c'est-- la veille il était très bien, le lendemain il est mort. Et c'est pour ça du coup qu'on achète un peu au jour le jour parce que [euh] on a peur que- que ça vire vite. [euh] Sinon, ça arrive des fois qu'on ait des courgettes qui soient vraiment pas bonnes quoi. Et du coup, en général je les goutte, même crues, avant de les mettre dans ma soupe ou dans une préparation, parce que ça gâche tout le plat quoi, et la soupe est immangeable. Donc ça je goûte, et si clairement elle est pas bonne, je suis obligée de la jeter quoi, je peux rien en faire quoi. On n'a pas de- on n'a pas de poules, on n'a pas- voilà. On aurait des poules, ce serait autre chose quoi.

00:54:37
*Mme. 1:* Donc, bien souvent parce que c'est moisi, ou alors elle n'est pas bonne, mais ça, vous le savez à partir du goût, du coup est-ce que vous pouvez me résumer comment, à chaque fois, vous allez pouvoir déterminer quel fruit ou légume, là, n'est pas ou plus consommable ?

00:54:54
*Mme. 2:* À sa tête.

00:54:56
*Mme. 1:* Sa tête. [rire]

00:55:00
*Mme. 2:* Sa tête me revient pas. [rire]

00:55:03
*Mme. 1:* Donc c'est plutôt, c'est son apparence qui va vous-- ?

00:55:05
*Mme. 2:* Son apparence, oui. En règle générale voilà c'est, quand c'est pourri, ça se voit. Et puis sinon, la courgette, je suis habitué maintenant, je goûte quoi. Même si elle a l'air super bonne, je goûte toujours un petit bout, avant de mettre à cuire.

00:55:18
*Mme. 1:* D'accord donc ouais, c'est même pas parce que là vous avez un doute sur la courgette, ça va être systématique, vous allez vérifier qu'elle est-- ?

00:55:27
*Mme. 2:* Surtout celles du Super U en fait, clairement, je veux dire- je pense- une courgette de- de- du primeur d'un petit- d'un petit agriculteur local je me poserais la question, mais là [euh], on a eu tellement de mauvaises surprises avec ces courgettes-là, et comme bah c'est tellement ça, on va avoir du travail, on a pas un magasin bio juste à côté ou voilà, un producteur ben on s'arrête au Super U souvent, y a un primeur là, juste à la place, mais souvent il est fermé aussi aux horaires où nous on en a besoin.

00:55:55
*Mme. 1:* Je sais pas s'il fait du bio ou pas ?

00:55:59
*Mme. 2:* Et... Non, il fait pas du bio, c'est un peu le- le dilemme parce que du coup, au Super U, ils font du bio mais, je ne sais pas quel bio, et qui en plus, tient 24 heures. Et au primeur c'est pas bio, mais ils ont des choses locales, qui tiennent plus longtemps. Donc [euh], après en général on mixe un peu. Et sinon, on achète bio au marché, quand on a le temps. Parce que comme on va à la messe le dimanche matin, c'est en même temps que le marché. Et voilà, soit on y va plus tôt, mais on est des gros dormeurs, donc en règle générale, on peut pas tout faire. Soit on y va après, et là y a plus rien. [rire] Le marché en fait je crois, on pourrait y aller toutes les semaines, si on se boostait un petit peu à dire, on y va plus tôt. Mais, voilà.

00:56:43
*Mme. 1:* Sinon, y en a un, quand je sors un peu de mon-- y en a un le samedi matin. Après faut, c'est le samedi matin, mais il est vraiment tout en haut...faut monter la- la rue. C'est avant Saint-Pierre, c'est au niveau des Quatre Moulins. Et c'est samedi matin, et on retrouve des- enfin les petits, du coup, mais par contre beaucoup de moins de monde, ce qui est son côté agréable, et on retrouve certains producteurs, qui sont- qui sont à Saint-Louis le lendemain matin.

00:57:12
*Mme. 2:* D'accord, bon à savoir. Y a celui de Kerinou aussi, le samedi.

00:57:14
*Mme. 1:* Ouais, mais du coup qui est plus loin plus loin. Mais, quoique--

00:57:20
*Mme. 2:* Nous en fait là, Kerinou, on passe sous le pont là, après on y est rapidement.

00:57:30
*Mme. 1:* Ah ouais ?

00:57:30
*Mme. 2:* En cinq minutes en voiture, on y est.

00:57:30
*Mme. 1:* Ah ouais, donc [euh]--

00:57:32
*Mme. 2:* Ouais par Pasteur là, par le bas.

00:58:00
*Mme. 1:* Et lui par contre, là c'est que le bio.

00:58:00
*Mme. 2:* Là, c'est que bio, voilà. Et ils ont le pain de Lannilis, qui tient quand même super longtemps. Y a un pain là, la boulangerie Izard. C'est le pain qui était prévu pour les marins, et c'est- il est connu comme un des pains qui tient le plus longtemps. On en a acheté, c'est la première fois que j'ai goûtais, j'en ai acheté y a deux semaines, il est encore bon, quoi. Il est pas dur enfin, il est pas- il est pas immangeable quoi.

00:58:00
*Mme. 1:* C'est vrai que ça c'est, le- le pain, les produits de boulangerie, ont été montrés comme- comme un aliment très- très gaspillé par nous, les ménages.

00:58:08
*Mme. 2:* Mais les baguettes, on achète une baguette, elle est plus bonne le lendemain.

00:58:11
*Mme. 1:* Ouais, ouais.

00:58:12
*Mme. 2:* Là j'ai acheté une baguette ce matin parce que j'avais un entretien ce matin, j'avais faim en sortant, mais c'est une baguette, je la mange dans la journée, sinon c'est pas-- le lendemain elle est plus bonne du tout, quoi. C'est pour ça qu'on fait nos pains aussi parce que-- quand on a le temps, parce que c'est trop-- sinon on prend à la Fournée, quoi, ils ont du bon pain aussi, au levain. Au levain, ça tient plus longtemps quoi.

00:58:34
*Mme. 1:* Ouais, ouais, tout est stratégie de--

00:58:36
*Mme. 2:* Ouais c'est un peu ça, le problème c'est que tout est tellement stratégie que pour avoir des courses [euh] pour avoir des courses qui nous correspondent et qui- qui- qui tiennent bien, tout ça, sans se dire ça va- ça va pourrir faut le manger maintenant maintenant... En fait il faut un peu, ruser, quoi.

00:58:51
*Mme. 1:* C'est ça. OK, et du coup sur le pourri. Si- quand vous êtes face au pourri, est-ce que vous ressentez la même sensation que face à un champignon, ou pas ? [rire]

00:59:06
*Mme. 2:* Non, honnêtement ça- ça- ça- ça me- ça me plaît pas non plus hein, je vais pas aller faire un bisou aux fruits. [rire] Mais c'est pas. Disons que je vais passer sous l'eau ou je vais enlever la partie sans la toucher, honnêtement, j'ai pas envie de mettre mes doigts dedans, mais je vais prendre le fruit et je vais- je vais faire ce qu'il faut pour le récupérer, quoi, j'vais pas-- Ça va pas directement à la case poubelle forcément quoi.

00:59:31
*Mme. 1:* OK... D'accord. Et [euh] est-ce que dans votre foyer, donc du coup avec [Prénom du conjoint], est-ce qu'il y a une différence, selon vous, entre justement vous-- on a dit pour la banane, la maturité, mais face au pourri, est-ce que vous avez un rapport différent, ou-- ?

00:59:56
*Mme. 2:* Non, je pense pas. Non, je pense que clairement quand on dit il est pourri, il est pourri. [rire] On n'est pas à se dire genre : "oui, non, peut-être", non il est pourri. [rire]

01:00:02
*Mme. 1:* Oui, c'est ça. [rire] OK. [euh] Alors--

01:00:08
*Mme. 2:* Il va peut-être le garder un peu plus, genre je vais passer derrière et puis je vais lui dire : "non mais regarde ça" et puis il va faire : "oui, t'as raison", et puis il va le faire. Quoiqu'il est têtu, alors je pense qu'en vrai- en vrai-- non mais si vous-- il est têtu donc je pense que s'il pense vraiment un truc, c'est que là je pense il était pas sûr, et il a essayé, si je dis non, il dit bon effectivement, elle a raison, ça va pas être bon. [rire] Mais en règle générale, c'est moi qui jette, puisque c'est moi qui fait la cuisine donc--

01:00:43
*Mme. 1:* ...Bah oui. OK.

01:00:44
*Mme. 2:* Alors c'est souvent [Prénom du conjoint] qui me rappelle aussi genre : "on a ça à manger, n'oublie pas qu'on a ça à manger !", parce que moi des fois je peux faire pendant une semaine des trucs s- enfin, puis je me dis que j'ai oublié que j'ai des courgettes, ou j'ai oublié que j'ai des poivrons, ou genre, mais pour moi on a un frigo c'est ça il faut que je les fasse, mais en fait c'est-à-dire que j'ai pas envie de faire un plat-- en fait, si je fais mes menus, et puis des fois je me dis : "oui mais j'ai pas envie de manger ça aujourd'hui", ça m'arrive. C'est pour ça voilà, on essaye vraiment de faire un peu au jour le jour aussi, parce que voilà je suis-- un peu comme les odeurs, je suis très changeante sur ce que j'ai envie de manger le jour-- ...La nourriture peut me mettre dans un état incroyable, c'est-à-dire que si, genre par exemple, c'est- c'est bête hein, mais si on reçoit un plat, genre qu'on commande à Uber Eats, et que c'est pas ce que j'avais espéré, genre s'ils se trompent sur un ingrédient, ça peut me mettre mais dans un état de- de dévastation immense. Pareil, quand genre j'ai pas-- j'ai la flemme de faire-- enfin flemme, j'ai pas envie de faire un plat alors qu'on n'a que ça à manger, si clairement c'est pas ce qui me fallait à ce moment-là, c'est- mais vraiment comme un enfant, quoi. Ça peut prendre des conséquences dramatiques, bon je vais pas pleurer ni tamer du pied, mais-- je vais en ressortir avec une frustration immense, quoi. [rire] Comme dit, mon rapport à la nourriture est un peu conflictuel. [rire]

01:01:53
*Mme. 1:* D'un extrême à l'autre.

01:01:55
*Mme. 2:* Ça m'est arrivé de pleurer en fait de- de- de- pas de tristesse, mais de- ouais de frustration quoi genre en mode genre : "y a plus ça !" genre "mais y avait plus" "mais non, mais c'est pas possible ! J'en voulais tellement !"

01:02:12
*Mme. 1:* [rire] Je vois.

01:02:12
*Mme. 2:* Un peu de fatigue et-- "Y a plus de courgettes", "comment ça ?" [rire] Non, non, une vraie enfant. Je- je- je l'accorde.

01:02:28
*Mme. 1:* Mais ça ouais, je comprends le fait de-- enfin les menus anticipés, c'est vrai que ça, ça a de gros avantages pour- pour- ben pour bien préparer ses courses et faire des courses adaptées aux besoins, mais c'est vrai que selon la journée qu'on a passée, on n'a pas forcément envie de-- [rire]

01:02:46
*Mme. 2:* La petite salade de mâche après dix heures de travail, on a plus envie quoi. On a envie d'un tacos, et puis voilà, c'est bon. [rire]

01:02:54
*Mme. 1:* Ouais, exactement, donc [euh] ouais, je pense que des fois y a du- y a forcément des changements de programme. [rire]

01:02:56
*Mme. 2:* Non puis alors moi, chaque semaine, je me dis : "on va manger trop healthy cette semaine", résultat : tacos, pizza, bières.

01:03:03
*Mme. 1:* Petit bilan ? Bon. [rire]

01:03:07
*Mme. 2:* Il reste une boîte de maïs ? [rire] On mange beaucoup du maïs aussi.

01:03:12
*Mme. 1:* En conserve du coup ?

01:03:14
*Mme. 2:* En conserve, mais alors on adore le maïs, le vrai- le vrai, le maïs hein, grillé au barbecue, ça c'est--

01:03:18
*Mme. 1:* Ah ouais ?

01:03:21
*Mme. 2:* Ouais, mais on n'a pas de barbecue, on n'a pas de jardin, donc [euh] voilà--

01:03:23
*Mme. 1:* Pour le moment, c'est--

01:03:24
*Mme. 2:* C'est ça. Je pense quand aura une maison avec un jardin, et ce sera quand même plus simple. Et puis plus d'endroits où stocker, parce qu'en fait finalement notre cuisine fait grande, mais y a pas beaucoup de rangements, et en fait, on est vite ensevelis sous toute la masse de- de vaisselle ou de- de légumes. On n'a pas d'endroit vraiment où couper, donc souvent je viens là, mais alors ça nous fait un, voilà-- Donc [euh] une cuisine ouverte ou avec un- un plan de travail, ça serait chouette. C'est un peu notre "goal" cuisine. [rire]

01:03:53
*Mme. 1:* C'est vrai que ça joue... Je pense même, ça donne envie ou non de cuisiner quand on a l'espace qui permet, qui donne envie de le faire quoi, ça joue forcément, et sur le stockage, je vous avais pas demandé, si [euh] voilà, si là y avait pas de contraintes matérielles, financières, qu'est-ce que vous ferez comme stockage de fruits et légumes ? Est-ce que vous resterez sur le même principe ?

01:04:22
*Mme. 2:* Alors, moi j'aimerais bien un légumier. Je trouve ça incroyable. J'ai découvert ça y a pas longtemps et j'ai dit mais dans la cuisine, il faut un légumier.

01:04:23
*Mme. 1:* C'est quoi, c'est des trucs par [euh]-- ?

01:04:25
*Mme. 2:* C'est des tiroirs ouais, c'est des tiroirs avec des bah, c'est bien aéré donc forcément le légume, il est pas, il pourrit moins vite, et puis c'est quand même grillagé un peu noir, donc la lumière ne rentre pas non plus, voilà, et ça permet aussi de voir tous ses fruits et légumes en même temps et de se dire, bon ben j'ai ça, alors que nous en fait on est obligés de tout stocker dans des petits endroits parce qu'on n'a pas la place pour tout au même endroit, [euh] et finalement le contact ça fait pourrir aussi d'autres fruits et légumes, donc on met un peu partout, et finalement on se rend compte : "bah ouais mais en fait on avait ça, mais comme c'était là on l'a pas vu, ou on l'a oublié", enfin voilà. Et ouais, un légumier, franchement, ça me plairait bien. Comme une petite mamie : "j'ai acheté un légumier".

01:05:08
*Mme. 1:* Et les- les- et les fruits, par contre, en corbeille.

01:05:12
*Mme. 2:* Une corbeille, une grande corbeille du coup. Un jour j'aurai une grande corbeille. Pour l'instant, j'ai une petite corbeille. [rire]

01:05:26
*Mme. 2:* [rire] Bébé corbeille.

01:05:32
*Mme. 2:* Un gros bol. Ouais puis alors, moi j'en mange souvent des fruits, donc forcément. J'aimerais bien les avoir dans le salon.

01:05:50
*Mme. 1:* Alors, pour presque finir, j'aimerais bien qu'on discute du gaspillage alimentaire, de façon assez générale. Quand je vous dis "gaspillage alimentaire", quels sont les mots qui vous viennent spontanément à l'esprit ?

01:05:59
*Mme. 2:* "Fruits rouges", "courgettes", "plus envie de manger ce que j'avais prévu de préparer" [euh]... "j'ai dû manger dehors parce que j'ai dépassé les horaires au travail et du coup y avait autre chose de prévu, et on n'a pas eu le temps de le manger" , "on a mangé Uber Eats plutôt que de manger la tarte à la tomate qui était prévue" ou "cette tarte aux poireaux ne sent plus comme la veille, donc elle ne me donne plus envie" [rire], ça aussi [euh], quoi d'autre ? "gaspillage alimentaire", acheter trop, aussi, par moments, pas réellement des besoins, [euh] ou acheter pas assez, aussi, et du coup attendre de trouver un plat pour passer le truc, le petit truc qu'on a acheté, et qui est pas assez pour un gros plat. Je sais pas- je sais pas si vous voyez ce que je veux dire ? [rire] Voilà, après je pense que le gaspillage alimentaire... Ou, ce kiwi n'est pas mûr, aujourd'hui. Aujour- demain, il est pourri, quoi, ça arrive. [rire]... (et toi, t'en penses quoi ? Tes croquettes elles sont-- ? ça ne t'arrive jamais. On devrait tous manger des croquettes) Les croquettes du fond du bol elle n'en veut plus le matin, il faut les jeter. C'est une princesse. Elle n'a pas le droit d'aller sur la table en fait, mais elle s'en moque.

01:07:40
*Mme. 1:* Là oui, ça a l'air. Et [euh] si on tente une définition du gaspillage alimentaire, donc voilà, si c'est vous qui deviez écrire dans Le Petit Robert la définition du gaspillage alimentaire, qu'est-ce que ça serait, avec vos mots et votre façon de- de voir ce- ce terme ?

01:08:05
*Mme. 2:* Alors [euh] je réfléchis, c'est une vaste question. [rire] [euh] Le fait d'acheter plus que ses besoins réels, et de ne pas utiliser en fait un produit qui devrait l'être dans- dans l'optique en fait d'avoir un- une consommation responsable. De ouais de surconsommer en fait, et c'est--

01:08:46
*Mme. 1:* Et du coup, vous, quand ça vous arrive de jeter, comment vous, comment vous vous sentez ?

01:08:56
*Mme. 2:* (euh] J'ai un peu honte quand même, parce je me dis y a des gens qui ont pas à manger, quoi, je veux dire même en France, ou des gens qui achètent des plats tout préparés parce que c'est- c'est vrai que les légumes et les fruits, ça coûte cher, quoi. Donc [euh], d'un l'impression de lancer de l'argent par la fenêtre, deux d'être irresponsable en fait, et pas du tout respectueuse en fait de- de ces aliments-là, en fait. Des gens qui les ont produits, qui les ont cultivés, qui les ont ramassés, qui les ont vendus [euh] et qui auraient pu en fait aller dans une autre famille ou chez d'autres gens, et qui auraient été consommés quoi. C'est ça que j'ai un peu honte. Après, je me dis, bon ben les légu-- par contre si la courgette, je viens de l'acheter, elle n'est pas bonne en fait là je-- c'est pas que je culpabilise, je suis plutôt un peu fâchée de me dire bon ben y a quand même des choses qui sont commercialisées, et qui sont vendues même plutôt cher, alors que c'est pas forcément bio, ou que c'est pas forcément fait en France. Et, voilà. C'est plus ça aussi des fois qui-- Un peu ouais- un peu de- de- d'énervement en fait face à des- des manières de consommer en fait, enfin, nous, de consommer en fait des aliments qui, normalement, on aurait pas eu besoin de les acheter si on consommait local, si on consommait enfin des choses, je sais pas si vous voyez ce que je veux dire. Parce que c'est rare quand même d'avoir une courgette [euh] bio, cultivée ici qui est mauvaise quoi, voilà, c'est-- Voyez, y a un traumatisme avec la courgette. [rire]

01:10:16
*Mme. 1:* Si vous la goûtez à chaque fois, effectivement, c'est qu'y a- y a vraiment des moments où ça passe pas, quoi.

01:10:19
*Mme. 2:* Ah oui c'est- c'est vraiment très mauvais.

01:10:25
*Mme. 2:* ...Ça arrive ouais, trois fois sur quatre, peut-être, quand on achète au Super.

01:10:29
*Mme. 1:* Ah oui ? Oui c'est quand même, c'est quand même beaucoup ouais, donc [euh]-- Puis même si dans la soupe elles passent pas, c'est que enfin voilà--

01:10:36
*Mme. 2:* Là la soupe est immangeable, quoi. Ça donne un goût très amer, le goût d'une courgette trop mûre quoi, en fait.

01:10:48
*Mme. 1:* Donc ouais, donc [euh] un peu de honte quand c'est un peu de- ouais voilà, c'est un peu parce qu'on aurait pu s'organiser différemment pour-- on avait pas envie-- [rire]

01:10:59
*Mme. 2:* [Prénom du conjoint] c'est de la colère contre moi, qu'il ressent. Il aime pas passer de légumes.

01:11:06
*Mme. 1:* Du coup oui, mais bon, malgré--

01:11:09
*Mme. 2:* Ce à quoi je réponds : "Eh ben t'avais qu'à faire !". Voilà non non, oui, parce que lui me rappelle souvent, et puis je fais : "je vais faire, je vais faire", puis en fait, je ne fais pas. Oui ça ouais, des fois j'ai un peu de honte, mais le fait est que je continue à le faire, donc c'est que je dois pas avoir beaucoup honte.

01:11:29
*Mme. 1:* Ouais, nous sommes dans des dissonances- des dissonances cognitives entre ce qu'on voudrait faire et ce qu'on fait, et ce que-- on est toujours [euh], toujours des conflits--

01:11:39
*Mme. 2:* Conflits d'intérêts de légumes.

01:11:44
*Mme. 1:* [rire] Bon d'accord. Alors [euh] pour terminer, terminer, sur la poubelle connectée. Donc vous l'utilisez depuis quelques jours maintenant, [euh] eh ben racontez-moi comment ça se passe. Vous avez le sentiment de l'utiliser beaucoup, pas beaucoup ?

01:11:59
*Mme. 2:* Alors on n'est pas d'accord sur l'utilisation avec [Prénom du conjoint]. [Prénom du conjoint] dit : "à chaque fois qu'on jette un truc, il faut le mettre dedans, et après il faut l'enlever tout de suite". Et moi, je dis : "non, tu le mets, puis tu laisses dedans, et après tu rajoutes".

01:12:10
*Mme. 1:* Oui.

01:12:11
*Mme. 2:* [Prénom du conjoint] ! J'ai raison !

01:12:16
*M. 1:* Ça chose pas la balance de le mettre, elle se souvient du poids précédent ?

01:12:28
*Mme. 1:* Ouais. En fait à chaque fois, y a un- y a le poids relatif qui est pesé. Donc, on a le- le- le nouveau gramme, la carotte, on va avoir le poids de la carotte. Et après, il y a le poids absolu qui va être cumulé, donc du coup, non. Après vous pouvez tout enlever si vous voulez, mais c'est pas obligatoire.

01:12:47
*M. 1:* Bon après de temps en temps j'ai enlevé tout quand même parce que, à force ça fait quand même-- ça moisit quoi.

01:12:50
*Mme. 2:* Je suis d'accord que nous nous soyons mis d'accord sur notre désaccord.

01:12:52
*Mme. 1:* Bah oui oui oui, c'est pas vrai en fait, elle elle en mettait, puis moi au bout d'un moment je voyais que ça faisait longtemps, je sortais et je refaisais la pesée à zéro, nickel.

01:13:00
*Mme. 2:* Oui parce qu'il est de mission poubelle en fait, c'est très segmenté dans notre- nos- nos missions de- de- de- ménagères. Moi j'aime bien la poubelle. Non après non, pas de, voilà-- vous avez les chiffres, du coup, sur notre poubelle ou pas ?

01:13:18
*Mme. 1:* Mais oui, mais je peux pas les dire.

01:13:20
*Mme. 2:* Vous pouvez pas me dire combien on jette ? Même pas à la fin ?

01:13:23
*Mme. 1:* À la fin de l'étude, on vous le dira. Alors, pas à la fin de là, la première période, à la fin de la seconde, donc en décembre en gros, on vous dira du coup sur les deux périodes où est-ce que vous vous situez un peu par rapport au panel. On pourra vous dire-- après nous ouais voilà, justement, y a tout un travail de savoir qui- quelle structure de foyer peut-être gaspille plus ou moins, enfin ça veut pas dire gaspille plus, mais voilà sera amenée à plus jeter de choses, est-ce que c'est une grande famille, est-ce que non c'est une personne seule, est-ce que-- tout ça on verra justement à la fin, et après en articulant avec toutes les- avec toutes les- toutes les pratiques différentes qu'on a les uns les autres en fonction de combien de fois on va aux courses, où est-ce qu'on va, justement voilà, les fameuses courgettes qu'on achète et finalement qui sont- sur lesquelles on est déçu parce qu'elles sont pas bonnes, enfin, y a tout ça à- à articuler, et puis ouais après on pourra vous dire, bien entendu.

01:14:15
*Mme. 2:* D'accord. Parce que là, on a jeté pas mal. [rire] En ce moment ouais, on a balancé pas mal de trucs, notamment des framboises. Impossible !

01:14:24
*Mme. 1:* Ouais c'est, on n'y échappe pas. Moi je, des fois, je vous avoue, je- je complexe, je fais une étude sur le gaspillage alimentaire des fruits et légumes et-- en plus, j'attends le dernier moment quoi. Là j'ai mangé un bout de radis noir ce midi, je l'ai recraché il est tellement pas bon, mais j'ai quand même laissé encore le reste du radis-- oui si ça se trouve, le reste est bon quoi, là c'était vraiment la petite partie mauvaise. [rire] Mais, non, je pense qu'il est foutu. Du coup bah-- donc euh, voilà, on n'y échappe pas. Tout le monde jette. Enfin tout le monde, peut-ê-- enfin si, je pense que tout le monde est amené des fois à jeter, des imprévus des machins.

01:15:04
*Mme. 2:* Ouais puis il suffit ouais, de rester manger chez un copain et y a un truc qui passe pas ou-- Ouais, ou un légume juste qui est pas bon aussi, ça arrive.

01:15:12
*Mme. 1:* Ouais, ça ça arrive aussi.

01:15:14
*Mme. 2:* Des pommes des fois, elles ont zéro goût.

01:15:17
*Mme. 1:* Ouais. Après faut aussi, ben faut les mettre en-- faut les cuisiner et tout mais, des fois après avec les contraintes de temps, on n'est pas non plus dans des sociétés très relax. [rire]

01:15:25
*Mme. 2:* Ah si, ah si, j'étais femme au foyer, pourquoi pas mais ? L'an prochain, je passe l'agreg, à mon avis ça va être-- Je pense la période de- de septembre à décembre, ça va être rigolo là, va y avoir, je pense ça va jeter. (rire]

01:15:32
*Mme. 1:* Y aura plus de tickets resto à la fin du- à la fin des- des trois mois.

01:15:43
*Mme. 2:* Je pense qu'[Prénom du conjoint] sera obligé de faire à manger. On va manger des pâtes, et des patates. [rire] Y aura pas de légumes, en fait.

01:15:54
*Mme. 1:* [euh] Et sur la poubelle, est-ce qu'il y a des moments où vous vous êtes posé des questions du style : "est-ce que ça, ça doit aller dedans ?"ou pas ? Non, c'était clair. (euh] Est-ce que, selon vous, y a des fruits et légumes que vous avez le sentiment de jeter plus que les autres ? Donc y avait la courgette peut-être parce que fréquemment mauvaises--

01:16:11
*Mme. 2:* Les bananes, du coup, quand [Prénom du conjoint] les mange pas. [euh] Qu'est-ce qu'on a- ? Là j'ai man- j'ai jeté quatre nectarines quoi. Parce que la veille elles étaient vraiment pas mûres, et le lendemain elles étaient moisies. Et là ça- ça- c'est-- Mais là du coup, c'est pas de notre faute quoi je veux dire, moi j'adore les nectarines, si j'avais pu les manger je les aurais mangées quoi, mais [euh] voilà donc ça les nectarines ouais, le- ouais, bananes, nectarines, courgettes. Des bouts de pommes de terre je pense aussi, abîmés. [euh] Qu'est-ce qu'on a pu jeter ? Bouts de carottes abîmés aussi, ça voilà, bouts de carottes abîmés, bouts de pommes de terre abîmés, et [euh] courgettes, bananes, et nectarines, je pense que, après, pas grand chose d'autre. J'ai jeté deux fraises hier, qui avaient complètement moisi, alors que je les avais achetées la veille. Pour le coup des bio, genre locales, mais elles ont pas tenu. Les fraises, ça passe tout de suite. Faut les faire tout de suite--

01:17:06
*Mme. 1:* Ouais. C'est ce que vous, ce que vous disiez avec les- avec tous les petits fruits, les baies et tout. Les cerises, enfin les cerises ça va.

01:17:15
*Mme. 2:* Les myrtilles. Oui, deux trois jours je pense, mais au-delà. Enfin moi de toute façon si j'achète des cerises, en deux heures, y a plus rien. [rire] En règle générale, les fruits- les fruits rouges, c'est ça en fait je me dis : "je vais en laisser à [Prénom du conjoint]", et il les mange pas, et du coup ben... Moralité, je les mangerai toute seule. [rire]

01:17:40
*Mme. 1:* [euh] OK. Alors est-ce qu'il vous est arrivé de l'oublier la poubelle ? C'est-à-dire des fois, ce qui est complètement possible, de se dire non ça, j'aurais dû jeter ce petit truc-là, non ?

01:17:50
*Mme. 2:* Ça peut-être dû m'arriver une fois, sur une pomme de terre. J'ai jeté avec les épluchures, peut-être.

01:17:54
*Mme. 1:* Souvent c'est, oui souvent c'est plus quand-- enfin, si on épluche et que là y a un petit bout-- Donc ça a pu arriver.

01:18:02
*Mme. 2:* Sans doute parce que c'étaient des pommes de terre neuves, qu'on venait d'acheter et je me dis bah en fait ça dépend pas de nous en fait, et ça va dans l'épluchage, j'ai du mettre-- genre c'est plus dans ce, voilà--

01:18:13
*Mme. 1:* Oui c'était de la petite épluchure de pomme de terre, au pire quoi. OK. Et le sac du coup, est-ce que vous avez changé de sac, ou-- ? Alors combien de fois vous l'avez changé ? Vous vous rappelez, ou pas ?

01:18:34
*Mme. 2:* Faut demander à [Prénom du conjoint]. Moi j'ai dû changer une fois, [Prénom du conjoint] a dû changer plusieurs fois. [Prénom du conjoint] ? [Prénom du conjoint] ? Tu as changé de sac combien de fois ?

01:18:37
*M. 1:* Le sac de quoi ?

01:18:37
*Mme. 2:* De la poubelle. [rire]

01:18:39
*M. 1:* Ah, trois ou quatre.

01:18:41
*Mme. 1:* OK, donc trois ou quatre, plus un. OK, donc je vais pouvoir vous en redonner alors, vous êtes bientôt- bientôt en rade.

01:18:51
*Mme. 2:* C'est possible, je vais- je vais regarder.

01:18:51
*Mme. 1:* J'en ai ramené.

01:18:58
*Mme. 2:* Vous nous en aviez donné pas mal du coup, parce que là il nous en reste... trois.

01:19:22
*Mme. 1:* Je vais quand même vous en redonner. Alors du coup, ben là j'ai terminé toutes mes questions. Donc un grand merci, et néanmoins est-ce qu'il y a des choses sur lesquelles vous aimeriez revenir, ou peut-être aborder des points que voilà que- que vous pensez que j'ai pas- que j'ai oubliés ? Des choses qui vous semblent-- ?

01:19:34
*Mme. 2:* Non, c'est bon.

01:19:37
*Mme. 1:* On a fait un bon petit tour des fruits et légumes, OK parfait. On les a bien épluchés.

01:19:44
*Mme. 2:* Quel jeu de mots !

**Household F23, Interview 2**

*Organisatrice:* Voilà, c'est ça. Ce n'est pas un test de connaissance. [Alors] Pour commencer, il y a quatre illustrations. Et je vais vous demander de-d'en choisir une, de la retourner et de me dire spontanément [euh] les trois premiers mots ou expressions qui vous viennent à l'esprit. Trois ou plus.

*Participante:* Peu importe--

*Organisatrice:* Ouais, non, justement c'est fait exprès pour que ce soit aléatoire.

*Participante:* Elle ne veut pas venir.

*Participante:* [silence 00:00:26 - 00:00:32]

*Participante:* Déjà j'ai eu « Didier », mais [alors] rien à voir.

*Organisatrice:* Didier ?

*Organisatrice:* [Euh] Ça me fait penser [euh] la-la tenue, à « Day by day », [enfin] les couleurs.

*Organisatrice:* Oui.

*Participante:* Je dirais « sans emballage ». C'est trois, c'est bon, j'ai trois mots ?

*Organisatrice:* Pourquoi Didier ?

*Participante:* Je-Je ne sais pas. Didier. Il a une tête à s'appeler Didier.

*Organisatrice:* [Euh] Et « day by day » ?

*Participante:* À cause du tablier.

*Organisatrice:* C'est le tablier ?

*Participante:* Orange. Les petites écritures vertes. Et son magnifique pull vert marinière qui-qui fait un petit rappel sur les fruits et légumes et-et le mur derrière. On est sur un truc exceptionnel.

*Organisatrice:* Oui.

*Participante:* D'accord.

*Organisatrice:* Et sans emballage ?

*Participante:* [Bah] Sans emballage, tout est en vrac ?

*Organisatrice:* Oui. Est-ce que vous avez déjà eu des expériences avec ce type de-de magasin ?

*Participante:* Avec « day by day » ou des primeurs en vrac ?

*Organisatrice:* Ouais, [bah] par exemple « Day by day » ou ce genre de--

*Participante:* [Bah] Oui, je vais essentiellement dans ce genre de choses.

*Organisatrice:* OK.

*Participante:* « Day by day » je n'y vais plus parce qu'ils ne font pas trop de bio, mais je vais aux [Nom d'un commerce à proximité] à côté.

*Organisatrice:* Oui.

*Participante:* Où tout est bio et local, le plus possible. Et puis après, je vais au petit primeur qui est juste à côté, là, sur la place [euh] pour prendre en vrac.

*Organisatrice:* Qu'est-ce qui motive vos choix de privilégier ce type de magasin ?

*Participante:* [Bah] Consommer local, bio, faire vivre les commerces locaux. Et puis [voilà] sans-sans-sans emballage. De pouvoir avoir mes-mes petits sacs. J'ai l'impression d'être une super héroïne de l'alimentation quand je fais ça.

*Organisatrice:* Pourquoi ?

*Participante:* [Bah] Parce que c'est trop facile de tout prendre en-en-- sous plastique [quoi], c'est-- [genre] tu mets tout ça dans un sac et puis tu rentres chez toi et puis après tu déballes les sacs, tu te dis : « Wow, tout ça pour faire 100 mètres, c'est super ». [Donc voilà]. C'est un truc qui me dérange, c'est les petits panneaux noirs, là. Je trouve que ça fait très vieillot alors que je ne sais pas si-- ça fait vraiment « mon marché » quoi.

*Participante:* [rires 00:02:46 - 00:02:52]

*Participante:* Je vais arrêter de critiquer Didier. Il a l'air super fier de sa boutique. Il a l'air super fier.

*Participante:* [chuchotements 00:02:58 - 00:03:03]

*Participante:* Elle n'a pas le droit d'être sur la table. Elle est contente que je ne dise rien. Tu sais, hein ? Que tu n'as pas le droit d'aller sur la table.

*Organisatrice:* Et est-ce que c'est un magasin dans lequel vous vous sentiriez bien ou pas ?

*Participante:* [Euh] Bien... Je n'ai rien contre Didier, ma foi. [Euh pff] Oui, oui, je veux dire-- je ne vois pas les prix [en fait]. J'aimerais bien voir les prix, pour savoir si c'est intéressant en rapport qualité-prix. Après, ça m'a l'air quand même d'être des tomates [je dirais] « industrielles », peut-être pas, mais [genre] bien rondes, [euh] bien calibrées. [Euh] Les carottes pareil, [et tout] les-les courgettes, ça ne m'a pas l'air bio et local [quoi] forcément. Ce n'est peut-être pas vers ce point que je-je-- qui m'attirerait [en fait] en termes de fruits et légumes. [Euh voilà] Quand j'ai le choix entre ce genre de fruits légumes et des-des bio, locaux qui ne sont pas très jolis. [Bah] Je préfère prendre les « pas très jolis », ils me donnent plus envie [quoi]. Je me dis qu'il y aura peut-être plus de goût et quelque chose qui a été fait en petites quantités, avec amour et juste à côté.

*Organisatrice:* Oui.

*Participante:* Quitte à payer un peu plus cher.

*Organisatrice:* D'accord. Finalement le beau, quand c'est trop beau--

*Participante:* [Bah] Ça cache quelque chose.

*Organisatrice:* Ouais ?

*Participante:* [Mm-hmm] C'est trop parfait. On a l'impression que ce n'est plus naturel.

*Organisatrice:* Oui.

*Participante:* Qu'est-ce que t'en penses ? Toutes tes croquettes, elles sont toutes rondes, et t'es contente. Alors Lili a peur des légumes.

*Organisatrice:* Ah ?

*Participante:* Il ne faut pas lui montrer une courgette.

*Organisatrice:* [Ah] Mais c'est comme les vidéos qui ont circulé où les-les chats font des bonds quand on leur montre--

*Participante:* Ouais, mais elle est traumatisée. Elle met ses oreilles en arrière et puis elle part en courant.

*Organisatrice:* C'est incroyable, ça.

*Participante:* Elle tremble [et tout] si on approche la courgette [et tout]. Un truc de fou.

*Organisatrice:* Moi j'avais essayé avec mon chien, ça n'avait pas du tout... [Pff] Non.

*Participante:* [Ah] Ouais, [bah] elle, elle n'aime pas du tout. Peut-être l'odeur [en fait]. Pareil, les oignons, c'est trop drôle.

*Organisatrice:* [Ah] Oui ? C'est marrant.

*Participante:* Elle a peur de tout, pour sa défense. Donc... [euh]

*Organisatrice:* Donc l'oignon en fait partie. [Euh] Et du coup, les produits que vous trouvez dans [bah] les primeurs que vous faites, qu-qu'est-ce que vous en pensez ?

*Participante:* Ça dépend lesquels. Parce que [bah] là, au primeur, ils ne proposent pas que des choses bio, locales. Et parfois je trouve que pour ce que c'est, c'est un peu cher. Par contre [euh], je trouve que [bah] en l'occurrence, ils ont quand même les meilleurs avocats que je n'ai jamais mangé. Je ne suis jamais déçue, c'est super bien, ils sont vraiment bons. Alors que quand je vais au Super U, une fois sur deux, l'avocat au bout de deux jours, il n'est plus bon [quoi].

*Organisatrice:* Ouais.

*Participante:* Là, vraiment, je ne suis jamais déçue. En général, quand j'achète des-des trucs là, c'est quand même assez bon. Il y a juste les choses que je n'achète-- je n'achète pas c'est : tomates [tout ça]. Je préfère aller en Biocoop ou-- [enfin] en Biocoop ou magasins équivalents. Et plutôt sur le marché d'ailleurs, en été.

*Organisatrice:* Sur le marché en été ?

*Participante:* Oui. [Bon] Ici les tomates, ce n'est pas trop... Mais comme je viens de-du sud de Carcassonne, c'est vrai que les tomates [euh] ça pousse bien ! Donc j'ai plus de plaisir à acheter mes tomates là-bas qu'ici [quoi].

*Organisatrice:* Ouais, mais oui. Le marché en été, c'est celui de-- du coup, de Carcassonne ?

*Participante:* Oui, [bah] oui, oui. Quand je vais voir mes parents. On achète [bah] les abricots, les-les figues après [enfin] un peu plus-plus début septembre, [enfin] c'est là, c'est trop-- c'est trop bon. Ça n'a pas du tout le même goût ici. [Enfin] Ce qu'on trouve, ce n'est pas pareil. Les abricots, pareil.

*Organisatrice:* Ouais.

*Participante:* [Enfin] D'ailleurs, en règle générale, je vais même les chercher directement dans le champ parce qu'on a des voisins qui ont plein d'arbres fruitiers et un grand potager. Donc [en fait] on récupère souvent avec les voisins des fruits et des légumes tout frais [quoi].

*Organisatrice:* Oui.

*Participante:* [Bon] Qu'ils disent bio, mais avec tout ce qu'ils épandent sur les vignes, à mon avis... on en est loin. On te dérange ?

*Organisatrice:* Et est-ce que ça vous arrive de jeter des fruits et légumes que vous aviez achetés dans-- chez les primeurs ou en Biocoop ? Enfin, ce style de petits magasins.

*Participante:* Beaucoup plus rarement que quand j'achète en supermarché puisqu'au bout de deux jours, il n'y a plus rien déjà. Donc j'exagère peut-être un peu, mais très rapidement, ça moisit. [Oh] Ce pourquoi on est embêté aussi, c'est qu'on a un tout petit freezer [en fait], on n'a pas de congèl' donc [euh] ça nous bloque beaucoup parce que [bah] tout doit être consommé vite. On n'a pas moyen de dire [bon], ça, ça va peut-être aller mauvais, on le coupe et on le met au congèl', on s'en resservira après. Je pense que ça, ça nous permettrait de-de beaucoup moins gaspiller.

*Organisatrice:* Oui. Et si vous êtes amenée à jeter un fruits et légumes que vous avez acheté chez le primeur ou aux [Nom d'un commerce à proximité] [ou quoi], qu'est-ce que vous ressentez ?

*Participante:* [Euh] Un peu de la frustration [quoi]. Je-- d'un, je l'ai payé assez cher. Je me dis : c'est carrément du gaspillage. C'est un fruits et légumes qui-qui-qui était bon [quoi]. Après, ça m'arrive quand même assez rarement.

*Organisatrice:* Oui.

*Participante:* [Mm-hmm] Parce que [en fait] comme des-- [enfin] ils sont généralement bons au goût. [Bah] On a envie de les manger vite, donc... Et puis on achète peut-être plus en-en petite quantité que quand on va au supermarché, c'est souvent par lots. Donc [en fait] on se retrouve avec par exemple six tomates ou trois avocats. Et là, on se dit : « [Bon bah] Il va falloir les manger, sinon ils vont aller mauvais ». Tandis que quand [bah] on peut prendre à la pièce, on a exactement ce dont on a besoin aussi.

*Organisatrice:* Oui.

*Organisatrice:* [silence 00:08:38 - 00:08:47]

*Organisatrice:* OK. On se fait une autre illustration.

*Participante:* Une autre ?

*Organisatrice:* Oui.

*Participante:* [Alors] Comment va-- comment va s'appeler cette personne ?

*Organisatrice:* S'il y en a une.

*Participante:* Il n'y a pas de personne, je suis un peu déçue.

*Organisatrice:* C'est quoi les mots qui vous viennent en tête ?

*Participante:* [Euh] Carré, [genre] monastère et [euh] organisé.

*Organisatrice:* Pourquoi vous pensez à monastère ?

*Participante:* [Bah] On dirait les-- ce qu'ils faisaient dans les monastères, là, avec les-les plantes aromatiques, [tout ça] même avec le petit écriteau.

*Organisatrice:* Est-ce que c'est un lieu qui vous parle ? Que ce soit un petit jardin de monastère ou un jardin d'un particulier, ou un jardin partagé.

*Participante:* Moi, j'aime beaucoup. C'est le genre de jardin que j'aimerais avoir. Petit, mais mignon et puis efficace avec-- [euh] on sait où sont les choses. C'est affiché, [tout ça] c'est ce qui permet de se repérer assez facilement. Et puis en même temps, ce n'est pas non plus en trop grosse quantité. On a quand même plaisir à se dire : « [Bon bah] Je vais récolter mes fruits et légumes, mais c'est un peu une denrée rare » [quoi], parce que j'aurai quatre carottes et quatre patates. Mais du coup, c'est-- je trouve ça chouette. Avec la vigne derrière, on voit qu'il y a plein de-de variétés [euh] différentes. Là, je vois des épinards, des framboises, peut-être à gauche, la vigne... Après...

*Participante:* [silence 00:10:18 - 00:10:24]

*Organisatrice:* Quand vous dites que c'est une denrée rare, c'est quoi que vous-- qu'est-ce que ça-- qu'est-ce que ça changerait [du coup] dans votre façon de considérer votre carotte ?

*Participante:* [Bah] Comme c'est moi qui l'ai faite pousser, une valeur sentimentale [quoi]. L'idée d'avoir planté la graine, d'avoir été arrosée tous les jours et de se dire : « [Bah] ces carottes, on va vraiment les savourer parce qu'il n'y en aura pas avant l'an prochain, si j'ai le courage d'en replanter » [quoi]. C'est ça, le truc. Puis il y a cette fierté de dire c'est quand même « mes » légumes.

*Organisatrice:* Ouais.

*Participante:* Il y a un peu cette image de-des légumes des grands-parents [quoi], ceux qui ont un potager et qui font leur potager [genre] : « [Ah] Oui, mais ce qui fait toute la recette, c'est les légumes du jardin ».

*Organisatrice:* Oui.

*Participante:* « Cultivés avec amour ».

*Organisatrice:* Est-ce que vous avez déjà consommé des fruits et légumes qui venaient de ce type de potager ?

*Participante:* Oui.

*Organisatrice:* Ouais ?Qu'est-ce que vous en aviez pensé ?

*Participante:* Qu'ils étaient très bon, mais aussi bons que ceux de la Biocoop.

*Organisatrice:* Oui.

*Participante:* Alors si, il y a quand même-- ce que j'aime beaucoup, c'est les tomates, [hein] j'y reviens, mais mon père, il avait-- donc c'est-- il plantait ses tomates et ce qui est trop bon, c'est de prendre la tomate directement sur-sur la plante. Et comme elle est gorgée de soleil, elle encore un peu chaude du soleil. Et là, c'est la meilleure tomate au monde [quoi] ! Pareil pour les fruits [hein], les abricots qu'on vient de cueillir, les mirabelles, les cerises... Il y a ce côté vraiment instantané et [euh] tout frais qui est-- [euh] je trouve que c'est toujours meilleur.

*Organisatrice:* Oui.

*Participante:* Puis de faire l'effort d'aller le chercher aussi, ce n'est pas la même chose que quand c'est dans un plat, sur la table du salon [quoi].

*Participante:* [silence 00:12:03 - 00:12:10]

*Organisatrice:* Vous pensez que ça joue un peu sur l'appréciation du...

*Participante:* Je pense que c'est totalement psychologique. Mais-- [euh bah] non, parce que quand elle est gorgée de soleil, il y a quand même ce-ce-ce goût-- Ce n'est pas ton jardin potager, laisse Didier tranquille. Mais après [je veux dire], [euh] ce sera la même tomate-- [euh, enfin je veux dire].

*Organisatrice:* Je vois.

*Participante:* Je suis assez évasive, désolé.

*Organisatrice:* Non, non, non, c'est très bien ! Du coup l'idée de jeter un fruits et légumes qui viendraient de-de ce type de potager, ça vous ferait quoi ? Est-ce que c'est la même frustration que ce que vous pouvez acheter chez le primeur ou-ou c'est différent selon vous ?

*Participante:* [Euh] C'est différent parce que l'idée du fait que ce soit un peu cyclique et que, par exemple, la tomate, [bah] je la cueille et elle est-- [euh] par exemple, elle-elle pourrit assez vite ou elle n'est pas très bonne. Je me dis, je la mets au compost et je me dis : « [Bah] Elle retourne à la terre ». Et [en fait] comme c'est moi qui l'aie faite pousser, il n'y a pas eu cette personne qui a pris le temps, [vraiment] en tant que travail, de la faire pousser, de la récolter de-- [euh] puis de la payer aussi [quoi]. Là, c'est mon propre travail, c'est moi qui l'aie faite pousser. Donc si ça ne va pas, [bah] elle servira à la terre et aux petits asticots. Et voilà, c'est le cycle de la vie aussi [quoi] !

*Organisatrice:* Oui. Donc il y a plus le fait que vous les avez-- vous n'avez pas dépensé d'argent pour ce fruit ou légume--

*Participante:* Et puis que c'est moi qui aie fait le travail [quoi].

*Organisatrice:* Oui, il n'y a pas de la--

*Participante:* Après, ça m'embêterait de jeter une grande quantité. [Enfin, je veux dire] J'aurais vraiment l'impression de-de-- en jeter une parmi 100, ça ne me dérange pas. Mais 100, par exemple, sur 200... Je me dis : « J'aurais très clairement pu en donner », c'est-- ou les mettre en conserves, ou faire un effort supplémentaire [quoi].

*Organisatrice:* Oui.

*Participante:* Je ne dis pas qu'il faut forcément les-les gaspiller nous-- comme c'est nous qui les avons faites. Mais [voilà] je parlais d'une par exemple.

*Organisatrice:* OK.

*Participante:* Là par exemple, j'ai acheté quatre tomates bio au Carrefour, ça m'a coûté [euh] 4,50 €. Quand la tomate, elle vaut plus d'un euro, ça m'embête quand même d'en jeter une. Là, franchement...

*Organisatrice:* C'est vrai qu'1€ la tomate...

*Participante:* Je n'avais pas vu le prix [hein] quand j'ai acheté ça, mais j'avais besoin de tomates.

*Organisatrice:* Oui, c'est vrai que là ça commence-- on pense direct, forcément, au porte-monnaie qui...

*Participante:* Et puis ce n'est pas du tout la saison, j'ai un peu cherché aussi.

*Organisatrice:* C'était un risque. Du coup, est-ce que le potager, c'est un endroit dans lequel vous pensez vous sentir bien ou pas ?

*Participante:* [Oh] Oui, j'aime bien. Ma foi, moi, je fais-- les plantes viennent mourir chez moi. J'aimerais bien avoir un potager, mais je ne suis pas sûre d'être très douée. [Donc voilà] Ça me fait rêver [hein], je trouve ça super beau, c'est très apaisant... J'aime bien l'idée d'avoir plein de variétés différentes de pouvoir voir comment elles grandissent... [Tout ça et tout] Mais-Mais je sais que chez moi, elles viendront mourir. Et puis... [voilà] C'est tout.

*Organisatrice:* Pas beaucoup d'espoir sur--

*Participante:* [En fait] C'est ça. Moi, si j'arrive vraiment à avoir une tomate, si j'en ai-- si je dois en jeter une, [en fait] ce sera peut-être un crève-cœur parce que ce sera peut-être la seule que j'aurais réussie à faire pousser sur 10 ans [quoi]. Là-Là, j'avoue, ça m'embêterait. Mais [euh] mon mari est plus doué que moi, donc je me dis que lui y arrivera et puis-- [voilà].

*Organisatrice:* Oui, c'est sûr [Prénom du conjoint]...

*Participante:* [Enfin] J'ai encore des plantes vivantes, mais elles perdent-- [voilà] mon Ficus, je ne sais même pas comment--

*Organisatrice:* Il est quand même-- il est très beau !

*Participante:* [Bah] Ça va mieux, là ! Parce qu'à un moment il avait perdu toutes ses feuilles et je me suis demandée...

*Organisatrice:* Mais j'en ai un, [enfin] qui est beaucoup moins grand, et il avait aussi perdu toutes ses feuilles. Il y a eu des branches qui n'ont jamais repris. Là, il est quand même-- mais de toute façon, il y a un cycle quand même où-- peut-être que-- mais [bon] en hiver, il ne les perd pas.

*Participante:* Là, je pense qu'il faudrait que je le rempote dans quelque chose de plus grand parce que quand je l'ai acheté, il faisait la moitié de ça [quoi]. [Bon] Il y a trois ans quand même.

*Organisatrice:* C'est une très belle plante !

*Participante:* Ouais, le rempoter puis mettre un tuteur plus grand, parce que là, il est en train de pencher, à un moment, il va tomber.

*Organisatrice:* Ouais, [bah] c'est possible. Et il peut aller-- il peut aller haut [hein] !

*Participante:* Oui, [bah] j'aime bien. Mais [euh] en vrai, il avait beaucoup plus de feuilles, il était plus touffu quand je l'ai acheté. Je pense qu'il-il subit un peu la vie en appartement aussi.

*Organisatrice:* [Bah] Ouais...

*Participante:* Dans la nouvelle maison, il sera mieux.

*Organisatrice:* Oui, c'est vrai. Et c'est-- oui. Je connais le problème. [Euh] J'ai déménagé, mes plantes ne se plaisent pas du tout dans mon nouvel appartement.

*Participante:* Oui, moi, j'ai du lierre comme ça. À Toulouse, j'ai toujours réussi à faire pousser du lierre. Et ici, dès que j'ai un plant, il meurt. Et j'essaye à chaque fois, parce que j'adore le lierre. Alors là, j'en ai un beau dans le-- je suis contente, il survit pour une fois. D'habitude, il meurt. Au bout de trois mois, [genre] adieu.

*Organisatrice:* J'ai le même problème. J'en avais pris, [euh] même là, chez la fleuriste. Plein de fois, j'ai essayé. Au bout d'un mois ou deux, il était-- [bah] il n'y avait plus rien à--- plus rien à sauver dessus. Et là j'en ai un qui naît, qui-qui est pas mal imposant, mais il ne se plaît pas dans mon nouvel appartement. Du coup, je suis en train de le reperdre.

*Participante:* Peut-être le changer-- le changer de place ?

*Organisatrice:* Ouais, [enfin] c'est-- il n'est pas-- c'est un appart' qui n'est pas lumineux, je savais avant de le prendre, mais... Le combo changement d'ambiance, pas de lumière, [euh] hiver...

*Participante:* Je pense qu'il n'a pas...

*Organisatrice:* C'est l'hécatombe.

*Participante:* Nous, c'est notre chat, là. En déménageant, je ne sais pas. Je pense qu'elle va péter un câble.

*Organisatrice:* [Ah] Oui ?

*Participante:* Elle est flippée de tout donc... [Euh] Là, on l'a amenée chez mes parents à Noël. La maison n'est pas très grande non plus. Elle nous a fait la gueule pendant deux semaines et quand on est rentrés ici, c'est : « [Ah bah] Enfin ! ».

*Organisatrice:* Le retour ! Ouais. Si elle n'aime pas sortir, elle ne sortira pas...

*Participante:* Ouais, là-là, elle est bloquée. Elle est cassée.

*Organisatrice:* Elle ne sortira pas si elle ne veut pas.

*Participante:* [Ah bah] Elle aura un grand jardin pourtant. Muré en plus ! J'espère qu'elle voudra bien sortir !

*Organisatrice:* Oui, ça s'adapte assez--

*Participante:* Lili ? Ça y est, là c'est l'heure de la sieste.

*Organisatrice:* Ça y est, ça c'est-- sommeil profond.

*Participante:* Coma.

*Organisatrice:* Coma.

*Participante:* Elle est forte pour ça. C'est sa meilleure activité de la journée, elle ne sait faire que ça.

*Organisatrice:* Ils ont cette chance.

*Participante:* Franchement, c'est-- je crois qu'il n'y a pas plus belle vie qu'une vie de chat [hein].

*Organisatrice:* Oui, oui, je crois que ce n'est pas mal. Encore mieux que chienne.

*Participante:* [Ah] Mais c'est incroyable, on ne lui demande rien, qu'est-ce qu'il veut ? Il dort. Il a les meilleurs endroits de la maison. Il fait des bains de soleil. Il a des croquettes de luxe. Celui-ci, du moins. Et elle est outrée quand on l'amène ailleurs.

*Organisatrice:* C'est ça, c'est reconnaissant. [Alors] La suite... On les fait toutes.

*Participante:* Comment va s'appeler cette personne ? [Ah] Il y en a plein ! [Hmm] Supermarché.

*Organisatrice:* Oui ?

*Participante:* [Hmm] Surconsommation. Multicolore.

*Organisatrice:* [Alors] Surconsommation pour vous, qu'est-ce que ça signifie ?

*Participante:* Que-- qu'il y a beaucoup trop de choses, de variétés, de quantité pour que ce soit consommé par, je pense, l'intégralité du magasin-- [enfin] des personnes, [enfin] des consommateurs sur la durée de vie des fruits et légumes.

*Organisatrice:* Oui.

*Organisatrice:* [silence 00:19:30 - 00:19:35]

*Organisatrice:* Et multicolore. Qu'est-ce que ça vous évoque ?

*Participante:* [Bah] Il y a toutes les couleurs [hein] : du vert, du jaune, du orange, du rouge... Du-Du marron au sol. Non, mais-mais peut-être pas toutes les couleurs de l'arc-en-ciel. Il y a du violet, du rose... Ça en fait pas mal.

*Organisatrice:* C'est coloré. Et est-ce que vous avez déjà vécu une ou des expériences avec ce type de lieux ?

*Participante:* [Oh bah] Oui, je vais au Carrefour donc... Mais il y a trop de-- il y a trop de choix et pas des bons choix [quoi]. C'est-à-dire que... Parfois-- la plupart du temps, ce n'est pas bon, puis ça pourrit tout de suite. [Enfin] Ça, je l'ai dit 56 fois.

*Participante:* Oui, vous avez remarqué ? Autant au niveau du goût que de la conservation. Puis c'est souvent fade, même si c'est au rayon bio. [Euh alors] Parfois, il y a petit rayon local. En règle générale, j'arrive à trouver là. Et pareil. J'ai acheté, il n'y a pas très longtemps, des oignons de Roscoff et en deux jours, ils étaient pourris. Je n'ai pas compris, des oignons ! Et [euh] j'ai dû en racheter en urgence, c'était pour une tarte. Sur 10, il m'en restait deux potables. Donc je pense qu'ils étaient pourris avant [hein] mais [euh] du coup, même là, on se dit : « Super, c'était là depuis quand ? ». [enfin]

*Organisatrice:* Oui.

*Participante:* Parce que comme c'est des consommables qui se conservent assez longtemps, peut-être que ça n'avait pas été vérifié et ils n'étaient déjà pas bien depuis longtemps. Comme c'est des sacs en kraft, on-on ne voit pas...

*Organisatrice:* Oui. Donc des fois des déceptions, des mauvaises surprises.

*Participante:* C'est ça. Après, ça peut arriver partout [hein], mais en l'occurrence plus souvent dans ce genre de-de commerce que... Puis là, [bon] même si c'est du vrac... Avec tous les petits sachets en plastique et les petites-- là, il y a les petites fraises et framboises en barquette.

*Organisatrice:* Oui.

*Participante:* [En fait] On a l'impression qu'ils nous vendent du rêve avec plein de fruits multicolores, c'est trop bon [et tout] alors que [en fait] c'est plein de pesticides et la qualité n'y est pas.

*Organisatrice:* Oui.

*Participante:* De l'autre côté, on a le droit à « Mangez cinq fruits et légumes par jour, c'est trop bien ! ».

*Organisatrice:* Et est-ce que c'est un lieu dans lequel vous vous sentez relativement bien ou pas ?

*Participante:* Non, parce que je n'aime pas mettre six ans à choisir une variété de pommes.

*Organisatrice:* Oui.

*Organisatrice:* [silence 00:22:07 - 00:22:11]

*Participante:* Puis quand on veut chercher des courgettes et qu'on est allé du côté des clémentines et qu'il faut refaire tout le tour, c'est un peu pénible. Je suis un peu une flemmasse, si vous remarquez bien.

*Organisatrice:* Je comprends bien. Il y a une sensation de perte de temps dans ce type de--

*Participante:* C'est ça. De-De temps et puis d'énergie. [Puis alors] Là, il n'y a pas mais j'ai souvenir quand j'étais petite là, sur les-les salades, ils mettaient des-des brumisateurs.

*Organisatrice:* Oui.

*Participante:* Et ça, ça me-- [enfin] c'était un truc de ouf quand j'étais petite, je disais : « Wow, il pleut dans le magasin ».

*Organisatrice:* C'est vrai qu'il n'y a plus...

*Participante:* [Ah] Ils ne font plus trop maintenant. Ça doit exister encore, mais ça fait très longtemps que je n'ai pas vu ça.

*Organisatrice:* Oui. Où est-ce que j'ai vu... J'étais-- ce n'était pas Brest. Je les ai vus, mais ça m'avait choquée, ouais, de revoir des brumisateurs. Et pour vous, le brumisateur, ça-- c'est une bonne chose ou une mauvaise chose ?

*Participante:* [Bah pff] Je veux dire, en soi, c'est-- si elles sont fraîches, s'il y a une petite quantité, on peut les vendre dans la journée, c'est bon. [Bah là] Ça veut vraiment dire qu'il faut essayer de les conserver le plus longtemps possible pour que le consommateur les trouve belle. En les réhydratant, en les--

*Participante:* [silence 00:23:15 - 00:23:23]

*Organisatrice:* Et quand vous êtes amenée à jeter un produit qui vient du supermarché, qu'est-ce que vous ressentez ?

*Participante:* [Bah] Je suis frustrée parce que forcément je l'ai-- je l'ai acheté aussi. Mais en même temps, s'il n'est pas bon et qu'il a pourri tout de suite, je n'y peux rien [quoi].

*Organisatrice:* Oui.

*Participante:* Par exemple les groseilles, si on les achète-- ou les framboises, si on les achète, allez le-le soir, le lendemain matin, il y en a qui sont déjà moisies [quoi]. Ça... C'est ouf !

*Participante:* [silence 00:23:45 -00:23:51]

*Participante:* Même en les mettant au frais.

*Organisatrice:* Oui. Ça vous est déjà arrivée de vous dire : « [Bon] Là, je change de méthode de conservation » et au final, ça ne change pas--

*Participante:* [Ah] Non, ouais, j'ai essayé. À la fin, j'ai congelé donc-- Je les mange soit directement en sortant, soit je les-- je les congèle dans mon tout petit freezer.

*Organisatrice:* OK. [Alors] La dernière illustration.

*Participante:* Marché.

*Organisatrice:* Oui ?

*Participante:* [Euh] Je cherche un terme... Raisonnable.

*Organisatrice:* Oui ?

*Participante:* Et frais.

*Organisatrice:* Raisonnable, qu'est-ce que--

*Participante:* [Bah] Raisonnable en qu-quantité [quoi]. Là, on voit qu'il y a--[euh bon] il y a un bac d'oignons [euh], il y a-- il y a des-- il y a quatre, cinq bottes de carottes et en même temps, on a l'impression [bah] que le-- [bah du coup] la personne qui vend, le producteur, il amène sa récolte [en fait] de la semaine, qu'il vend sa récolte de la semaine [quoi] en fonction de ce qu'il a et sans-sans surconsommation. Et le fait d'avoir aussi [bah] les fannes sur les carottes, on se dit « C'est vraiment des carottes qui ont été récupérées ». Bien qu'elles soient très, très propres pour des-des carottes...

*Organisatrice:* C'est vrai. Les fannes, pour vous, c'est gage de...

*Participante:* [Bah] de fraîcheur. Je me dis que c'est quand même quelque chose qui passe assez vite les fannes, alors que les carottes, on peut les conserver assez longtemps quand même [euh] un peu comme-- [enfin] moins que les pommes de terre, mais elles pourrissent moins vite [quand même] que d'autres fruits et légumes. Et par contre, les fannes, elles, elles deviennent très vite jaunes [quoi]. Là ça commence, mais-mais c'est quand même assez vert [quoi].

*Organisatrice:* Oui, vraiment. Oui, ça peut indiquer déjà l'état de la carotte [en fait]. Sa durée de--sa fraîcheur. Est-ce que c'est un lieu [euh] dans lequel vous-vous vous rendez ou pas vraiment ?

*Participante:* Oui, oui, oui. [Bon] Au marché, j'y vais assez souvent. Mais je vais plutôt du côté rayon bio du coup, au marché.

*Organisatrice:* OK.

*Participante:* Et généralement ils n'ont pas trop ce genre de-de caisses-là, en plastique, c'est plutôt soit directement sur le stand ou dans des cagettes en carton-- en bois [quoi].

*Organisatrice:* Oui.

*Participante:* Ce qui fait aussi [euh] plus responsable de l'environnement que des cagettes en plastique.

*Organisatrice:* Oui.

*Participante:* Là, pareil, il y a des bouteilles en plastique, il y a des sacs en plastique aussi. Donc c'est quand même des-des producteurs qui-qui continuent [en fait] à distribuer des sacs plastiques [quoi]. Mais ça m'attire déjà plus que-- que ça et que ça.

*Organisatrice:* Donc ça veut dire plus que le primeur et le supermarché.

*Participante:* C'est ça.

*Organisatrice:* Mais moins que le potager.

*Participante:* Moins que le potager.

*Organisatrice:* Et quand vous êtes au marché, est-ce que vous vous sentez bien ? Ou non plus, ce n'est pas...

*Participante:* [Bah] Quand il y a trop de monde, je ne me sens pas bien. Après, en l'occurrence, s'il n'y avait pas trop de monde, je me sentirais bien. J'aime bien le marché et j'aime bien faire le tour et voir que tel producteur propose « ça », mais que l'autre « ça ». Et du coup, imaginer [en fait] aussi les petites recettes que je peux faire [en fait] en fonction de ce qui est disponible sur le moment [quoi]. J'aime bien le côté surprise [quoi]. Quand je vais au marché, je ne me dis pas : « Il faut absolument que je ramène ça, ça, ça ». [Enfin] J'ai des petites attentes [quoi]. En termes de-de ce que je veux comme type de légumes. Je ne vais pas me dire il me faut absolument deux kilos de patates douces, [euh] du cresson... S'il n'y a pas de cresson, il n'y a pas de cresson. Je ne vais pas l'inventer. Et puis en fonction de ce que les gens ont pu laisser aussi, parce que si on arrive un peu tardivement au marché, [bah] il n'y a plus, il n'y a plus.

*Organisatrice:* Oui, aussi.

*Participante:* Ça demande quand même-- [enfin] peut-être pas ce terme-là, mais une certaine rigueur. [En fait, enfin, je veux dire] Le marché, c'est à une heure précise et on ne peut pas y avoir accès toute la semaine. [Euh] Le marché, c'est de 7:00 à 13:00 le dimanche matin. [Bah] C'est là qu'il faut y aller, sinon-sinon on n'aura pas de fruits et légumes pour la semaine si on souhaite consommer qu'à cet endroit-là.

*Organisatrice:* Oui. Est-ce que vous y allez toutes les semaines ? Ou c'est-- non, en ce moment.

*Participante:* [Bah là] En ce moment-- en ce moment, non. Trop travail, trop de-- Là, c'est un peu au jour le jour [en fait] de ce que j'ai envie de manger. En ce moment, c'est plutôt blinis/houmous [euh] plus qu'autre chose.

*Organisatrice:* Je vois.

*Participante:* [Bah] Comme je suis seule [en fait], je n'ai aucune obligation. Puis [en fait] je mange-- là, aussi depuis la rentrée, je mange au lycée [en fait] le midi. Donc déjà ça m'enlève énormément de repas. Puis j'ai un vrai repas, je prends des fruits et des légumes à tous les repas. Je ne sais pas d'où ils viennent, mais normalement c'est bio [hein] ce qu'on nous propose. Ce n'est pas exceptionnel, mais ça fait l'affaire. Et du coup, [bah] le soir-- le soir, je me prends un fruit si-si j'en ai. [Enfin] Mais je ne vais pas chercher à me faire un truc [euh] très construit, comme j'ai beaucoup mangé le midi. Parce que mine de rien, j'avais oublié, mais au self, on nous sert quand même des grosses portions, [euh] il y a une entrée, un plat, un dessert, un pain, des fruits. Et du coup, [bah en fait] ça me cale toute la journée. Même, je ne petit-déjeune plus parce que [en fait] en tant que surveillant, on mange à 11:00.

*Organisatrice:* Oui.

*Participante:* Je me dis : « Si je petit-déjeune, je ne mangerai rien et puis après j'aurai faim plus tard. » Donc là, [bah voilà] j'ai mangé à 11:00 tout à l'heure. Et [bah là] je n'ai pas faim [quoi]. [Je veux dire] Ce soir, je vais me prendre un truc léger. [Alors] J'ai des avocats et du-- et des tomates [voilà]. Et du houmous. Tout va bien.

*Organisatrice:* Oui, c'est ça. Ça m'a l'air pas mal. Donc oui, [enfin] là, vous faites-- vu que vous cuisinez-- cuisinez moins en ce moment, il y a moins aussi cette rigueur de--

*Participante:* Oui et puis comme on est en déménagement. Là, j'essaye d'écouler [en fait]. D'écouler les denrées qu'on-qu'on a [bah] qui sont un peu plus-- qui se conservent un peu plus sur la durée, [quoi] que j'avais gardées. Et je n'achète que des petits trucs [en fait] pour compléter, mais [euh] là je ne fais pas des grosses courses où j'achète [euh] plein de fruits, plein de légumes. J'essaie vraiment d'écouler [quoi]. Et puis quitte à manger que des patates douces surgelées, à un repas, parce qu'il faut finir le paquet de patates douces, mais...

*Organisatrice:* Je vois.

*Participante:* C'est dans deux semaines, mais je ne suis pas prête.

*Organisatrice:* Ça va aller vite. Ça va aller, ça finit-- ça va finir, ça va toujours. C'est toujours la panique avant, mais ça finit toujours par bien-- par bien se passer.

*Participante:* J'ai quand même un doute ! On a tellement de choses.

*Organisatrice:* On en passe, hein ?

*Participante:* C'est incroyable ! Parce que quand on est arrivé ici, on avait deux meubles et trois valises et là, on est plutôt sur un 60 cartons et un trois mètres cubes.

*Organisatrice:* Ouais, ouais, je compatis.

*Participante:* Et on a que 60 mètres carrés ! Qu'est-ce qu'on va faire dans 300 mètres carrés ? Je ne veux pas le savoir.

*Organisatrice:* [Bah] Ça va, vous avez fait ce sens-là. Moi j'ai fait l'inverse. Je suis passée de 80 à 40. [Bah] Là... Là, on s'amuse pour le tri.

*Participante:* Le tri et le Tétris.

*Organisatrice:* Ouais, c'est ça. C'est-- là, [bon] ça va vous pouvez encore empaqueter pas mal. Dessous...

*Participante:* C'est ça, puis nous, là le souci, ça va surtout être d'avoir des meubles.

*Organisatrice:* Oui. Au début, il va y avoir des pièces...

*Participante:* Vides.

*Organisatrice:* Oui, où l'on peut se projeter !

*Participante:* Les proprios nous laissent pas mal de trucs, comme eux vont dans plus petit aussi, ils nous laissent tout le deuxième étage, donc on a cinq lits.

*Organisatrice:* [Ah] Ouais ! C'est une sacrée demeure alors !

*Participante:* Ouais, [en fait] on achète le [Nom de la propriété]. C'est un gîte [en fait], on achète un manoir de 1715. [En fait] Ils faisaient des gîtes et ils louaient. Il y a 18 couchages.

*Organisatrice:* Et vous allez continuer à faire un gîte/chambres d'hôtes [et tout] ?

*Participante:* Ouais, on aimerait faire pour Pâques, mais à mon avis, ça va être...

*Organisatrice:* Ça va être chaud.

*Participante:* Ce n'est même pas envisageable. [Enfin] [Prénom du conjoint], lui, il est à fond mais... La déco, la déco est horrible [en fait]. C'est années 1970, ils-ils ont tout donné, ils ont tout donné [quoi] ! Il y a de la moquette partout donc il faut qu'on enlève ça. On a des poutres partout, mais des poutres noires [quoi], hyper foncées. Moi, j'ai une photo, mais du coup il faut qu'on ponce tout ça. Il y a vachement de trucs à faire [en fait].

*Organisatrice:* Avant de-- Oui, peut-être plutôt qu'objectif printemps, celui d'été... Et bien, sera plus simple à atteindre.

*Participante:* [Rhoo] Pardon, mon patron qui m'a demandé de faire des heures supp'. J'ai fait 27 h supplémentaires là, sur les deux dernières semaines... [Pff]

*Organisatrice:* Ça commence à faire.

*Participante:* Oui, j'en ai un peu marre.

*Organisatrice:* Oui.

*Participante:* Évidemment, je ne trouve plus la maison. Trop de photos. Qu'est-ce qu'il s'est passé ici ? [Alors] On a des trucs, des lambris comme ça au dernier étage et des poutres. Ça, il faut poncer et repeindre. Et [euh voilà] on a que des boiseries comme ça.

*Organisatrice:* [Ah] Oui, je vois.

*Participante:* Et le salon...

*Organisatrice:* C'est quand même joli !

*Participante:* Elle est top, mais il y a du taff. Les chambres, il n'y a que des boiseries comme ça...

*Organisatrice:* [Ah] Oui, oui.

*Participante:* Le papier peint à enlever, de la moquette partout. Partout, partout, partout, partout. Et ouais dans la cuisine... La cuisine, il va falloir poncer aussi, pareil. Le salon... Le salon, c'est le pire [hein].

*Organisatrice:* Oui. Il y a un côté-- ouais, mais ça, je-- ça-ça peut-être pire.

*Participante:* Non mais ça va rester dans son jus une bonne partie du temps.

*Organisatrice:* Ouais [voilà]. Je pense qu'on peut quand même accueillir, il y a des gens qui-- après plus vous referez, plus ce sera accueillant, plus on peut faire monter un peu les--

*Participante:* [Oh bah] Là, ils louaient sans soucis, oui. [Enfin, je veux dire] Ça va. Ils louent aux artistes des Vieilles Charrues [en fait].

*Organisatrice:* [Ah] Oui ?

*Participante:* Il y a eu Vianney là, trois semaines en septembre. Ils ont eu-- ils ont eu l'équipe Canal+ pour les Vieilles Charrues de l'an dernier ou celle d'avant, je ne sais plus. Je-Je ne sais plus lesquelles ont été annulées, mais ils ont eu l'équipe de Canal+ qui filmait du coup.

*Organisatrice:* [Roh] Trop bien ! Il va y avoir du beau monde !

*Participante:* Ouais, c'est trop bien ! Nous, on est trop contents !

*Organisatrice:* [Bah] Oui, c'est clair.

*Participante:* Voilà.

*Organisatrice:* Ouais, c'est un beau projet !

*Participante:* Oui, oui, oui. Ça a demandé une énergie folle, mais on est contents.

*Organisatrice:* Oui, oui. [Bon] Ça fait-- ouais, ça fait des-- ça-ça occupe, ce n'est pas ça que je veux dire, mais c'est...

*Participante:* Un projet.

*Organisatrice:* Ouais, voilà, c'est un projet, [quoi] ça-ça anime. [Euh] Alors sur les-- [euh] sur le marché, est-ce que ça vous arrive de jeter des fois des produits que vous aviez acheté ?

*Participante:* Très rarement.

*Organisatrice:* C'est rare ?

*Participante:* Très rarement parce que. Je ne jette pas en grandes quantités sur le marché. Je prends vraiment ce dont j'ai besoin [en fait] et puis je le fais assez rapidement. Si j'achète le dimanche, je fais du-- comment on appelle ça ? Du « batch cooking » ? C'est ça qu'on dit ?

*Organisatrice:* C'est quoi ?

*Participante:* C'est cuisiner en avance [en fait].

*Organisatrice:* Oui, oui.

*Participante:* Je-je prépare. Je prépare le dimanche après-midi et puis je fais mes petites préparations, puis après, j'ai juste à cuisiner un féculent ou--

*Organisatrice:* Ouais.

*Participante:* À côté [quoi]. Et donc du coup, [bah] les légumes sont déjà transformés. Donc pas [euh voilà]... Si, ce qui passe souvent à la poubelle, c'est [euh] j'ai envie d'être trop une « fit girl » et je m'achète de la salade et [en fait] je ne la mange jamais. Ça souvent...

*Organisatrice:* Ouais, la salade...

*Participante:* [Ah] La salade, je ne sais même pas pourquoi j'achète ça encore. Pourquoi je me fais du mal à en acheter, et pourquoi je lui fais du mal. Il faut que j'arrête.

*Organisatrice:* La salade, ça finit souvent mal. Et [euh] ça vous fait quoi quand vous jetez un produit qui vient du marché ? Est-ce que c'est la même chose partout ? Est-ce que c'est un peu différent ? Comment vous...

*Participante:* La salade, ça m'embête. Je me dis : « Tu n'aurais pas dû l'acheter ».

*Organisatrice:* Oui.

*Participante:* Mais après-après [voilà], c'est vraiment très, très rare que je jette. Et si je jette, c'est qu'il y a une raison. [Et voilà] C'est vraiment le supermarché [quoi]. Le supermarché, c'est n'importe quoi.

*Organisatrice:* Oui.

*Participante:* Tout ce qui est passé dans la poubelle, [hein] c'est du supermarché.

*Organisatrice:* [Ah] Oui ?

*Participante:* C'est 90 % des supermarchés. Supermarché ou les bananes qui sont devenues trop mûres. Et une fois qu'elles ne sont plus vertes, je ne les aime plus. Et du coup, je dis à [Prénom du conjoint] : « Tu mangeras les bananes ? – Oui, oui, oui, oui ». Et [en fait] il ne les mange pas et puis elles deviennent immangeables. Et-Et moi, comme ça-- l'odeur des bananes trop mures, ça me dégoûte, elles finissent à la poubelle.

*Organisatrice:* C'est vrai que vous aviez un truc avec l'odeur.

*Participante:* Oui, oui.

*Organisatrice:* Je m'en rappelle. Et bien, très belle transition puisque là, c'est un autre [euh] petit jeu sur la banane.

*Participante:* Je vais peut-être allumer.

*Organisatrice:* Ouais, si vous voulez.

*Participante:* Parce que bientôt, on va devenir aveugles. Est-ce que vous voulez un bonbon ?

*Organisatrice:* [Euh bah] Pourquoi pas se laisser tenter par un petit bonbon ?

*Participante:* Ou un fruit ?

*Organisatrice:* Un bonbon, c'est bien !

*Organisatrice:* [rires 00:36:22 - 00:36:27]

*Organisatrice:* Hop ! [Alors]

*Participante:* [Alors] Là, ça, ce n'est pas possible.

*Organisatrice:* Ah ouais ? [Alors] Justement, il y a dix photos de banane et je vais vous demander de les ranger, de celle qui vous donne le plus envie à celle qui vous donne le moins envie.

*Participante:* [Mm-hmm]

*Participante:* [onomatopées 00:36:40 - 00:36:55]

*Participante:* Ça existe ça ?

*Organisatrice:* Et [bah] apparemment...

*Participante:* Parce que moi, à ce stade-là, elle est à la poubelle depuis six ans déjà.

*Organisatrice:* Oui.

*Participante:* En règle générale, je dis à [Prénom du conjoint] : « T'es sûr ? Tu ne veux pas la manger ? », et puis... [Mm-hmm et voilà] Fin de la vie de la banane. [Han] Mais pourquoi tant de haine ? Là c'est-c'est chaud, mais il ne faut pas voir des problèmes avec les couleurs.

*Organisatrice:* Non.

*Participante:* [Hmm] Ça, c'est... Peut-être pas assez mûr. Là, elle commence déjà à être un peu abîmée. Je la mangerais, mais... [en fait voilà, mm-hmm]

*Organisatrice:* C'est quoi la banane parfaite ?

*Participante:* [Mm-hmm] Comme ça, on va dire.

*Organisatrice:* [Alors] Hop ! Je vais prendre une photo de votre rangement.

*Participante:* C'est un peu-- [voilà] moi, elle est parfaite, elle est là et je la mange comme ça, et après... [bon]

*Organisatrice:* [Alors]

*Participante:* Il ne faut pas exagérer non plus. Trop peu mûr, c'est moyen.

*Organisatrice:* [Alors] Les bonbons, le café, on est bien reçu !

*Organisatrice:* [rires 00:38:17 - 00:38:24]

*Organisatrice:* [Alors] Du coup, il y a des petites formes sur la-- ici pour savoir de quelle banane on parle à l'audio. Donc votre préférée, c'est la banane triangle bleu. Et pourquoi ? Est-ce que vous sauriez dire ? Qu'est-ce qu'il fait que--

*Participante:* Parce que le bout, il est encore blanc, là.

*Organisatrice:* Oui.

*Participante:* Et en même temps, elle n'est pas toute verte. Elle commence à-- elle est plus jaune que verte. Là, pour moi, elle est parfaite. Ça, c'est-- c'est la banane que j'aime bien. Je les achète généralement comme ça en me disant : « Je peux encore la manger [bon allez] pendant un ou deux jours, elle sera bonne ».

*Organisatrice:* OK.

*Participante:* Ensuite [bah voilà] ça-- ça c'est encore avant. Non, ça, c'est après. Parce que là-là, c'est du noir. Donc ça je mange encore.

*Organisatrice:* Carré blanc, on mange encore.

*Participante:* Ça aussi, mais le-le bout est-- commence vraiment à devenir noir. Et puis il y a des-- non, mais je peux montrer. Elle est un peu noire.

*Organisatrice:* Le triangle bleu, ça passe encore. Non c'est triangle vert, pardon.

*Participante:* Carré orange [hm] on commence à voir des petits points noirs. Ça va. Ça va, ça va, mais là, je commence à dire : « [Prénom du conjoint], mange les bananes ». Parce que là, à un moment, je ne vais plus pouvoir les manger toute seule. Là, je la mangerais, mais plutôt dans une préparation. Si elle n'est vraiment pas assez mûre, plutôt dans un muesli [tout ça et tout.] Pas juste comme ça, [quoi] je vais trouver ça moins bon,

*Organisatrice:* D'accord.

*Participante:* Une préparation, ça passe.

*Organisatrice:* Pas tout seul.

*Participante:* Là, l'odeur, le [euh] rond blanc, commence réellement à me déranger.

*Organisatrice:* D'accord.

*Participante:* Donc je ne l'ouvre même pas. Puis après, que dire... Là c'est pire. Donc là, c'est poubelle. Là, elle est la poubelle. Carré-Carré bleu, elle est la poubelle. Même pas je-- même pas j'ose la présenter à-- À la limite si, elle va peut-être finir dans une compote de pommes.

*Organisatrice:* D'accord.

*Participante:* Mais là, rond jaune, elle est à la poubelle. Et-Et du coup, est-il nécessaire de parler de l'existence de triangle blanc et de carré rose ? Non.

*Organisatrice:* Pas besoin. Donc si on reprend, la banane parfaite c'est-- [on va dire] les deux premières, la troisième ça-- c'est toujours correct.

*Participante:* Je la mangerais sans souci.

*Organisatrice:* Oui ? Vous prenez toujours du plaisir à la manger [euh] là ? Et là, ça commence à être [euh] limite.

*Participante:* [Bah] Là, je la mangerai si elle n'a pas trop d'odeur.

*Organisatrice:* D'accord. Et là, triangle rose, [euh] qu'est-ce qui fait que vous la-- vous ne pouvez pas-- [enfin] vous ne la consommeriez pas comme ça toute seule ?

*Participante:* [Bah] Il y a un côté un peu amère [quoi] de la banane qui n'est pas mûre [quoi], et qui n'est pas agréable. Je n'aurais pas de plaisir à la manger, je pense, juste comme ça, pour le plaisir de manger une banane [quoi]. Mais dans un muesli mélangé à d'autres fruits ou avec les petits grains de muesli et du chocolat, et du yaourt, ça me--

*Organisatrice:* Oui.

*Participante:* Ou dans un smoothie ou... [voilà]

*Organisatrice:* OK. Et [euh] dans une préparation, vous préférez le triangle rose que le rond blanc ?

*Participante:* Dans une préparation, par exemple gâteau ou compote, je préférerais ça parce que ça aura le côté plutôt sucré de la banane, alors que là, pas forcément de l'apport, il ne sera pas... [Voilà] C'est-à-dire que même en coupant la banane, là, elle va coller. Et puis on va s'en mettre plein sur les doigts. [En fait] Je n'aime pas ça non plus. Tandis que là, la banane, quand elle n'est pas trop mûre, ça s'épluche bien. Ça ne colle pas. Il n'y a pas les petits filaments pénibles qu'on est obligé d'enlever de la banane parce qu'elle est trop mûre. Je ne sais pas si vous voyez ce que je veux dire.

*Organisatrice:* Oui.

*Participante:* Et là, là, là à partir de ce stade-là, on n'en parle plus.

*Organisatrice:* Donc il y a les odeurs et il y a le côté pratique à--

*Participante:* Oui. Et puis même là, quand on « peel » la banane, elle se défait toute seule. Tandis que là [bah] si on n'a pas de couteau, c'est compliqué. Ou alors ça se déchire, mais du coup, ça, ça s'enlève totalement. Et-- [euh] ouais, non mais un vrai-- un vrai traumatisme avec les bananes vraisemblablement, j'ai dû beaucoup y réfléchir pour arriver à ce-- à ce-- à ce genre de réflexion.

*Organisatrice:* Non mais vous n'êtes pas la seule.

*Participante:* Non mais je rêve. Tu ne fais pas ça quand on est toutes les deux.

*Participante:* [silence 00:42:42 - 00:42:47]

*Organisatrice:* Elle a des grands yeux.

*Participante:* Oui.

*Organisatrice:* Dérapage. Donc oui, vous avez des attentes assez spécifiques de la banane, mais ça vous arrive de les cuisiner de temps en temps.

*Participante:* Oui, c'est ça.

*Organisatrice:* Et [Prénom du conjoint], du coup, est-ce qu'il les mange quand elles sont--

*Participante:* Non.

*Organisatrice:* Lui, il n'aime pas quand elles sont trop...

*Participante:* Lui c'est ça globalement.

*Organisatrice:* Carré orange.

*Participante:* Carré orange. Là, il ne la mangera pas, c'est sûr, celle-là.

*Organisatrice:* Ouais.

*Participante:* Celle-là, il la mangera. Et je pense qu'à partir de là, il va se dire : « Non, elle est trop mûre ».

*Organisatrice:* OK, donc lui c'est-- [euh], ça va être plus des trucs-- celle-ci, qui commence à être tâchetée.

*Participante:* Hop ! Elle-Elle n'a pas mal [hein].

*Organisatrice:* Non. Triangle vert, carré orange, rond blanc... Et carré bleu, ça commence à être trop-trop tard.

*Participante:* Mais ça suffit ?

*Organisatrice:* Elle est trop mignonne.

*Participante:* Tu arrêtes !

*Organisatrice:* Et selon vous, est-ce qu'il y a un moment donné où la banane peut être éventuellement dangereuse pour la santé ou pas ?

*Participante:* Je me dis que peut-être pas assez mûr, ce n'est pas très bon.

*Organisatrice:* D'accord.

*Participante:* Après [euh] trop mûre... [Bah] Quand elle est pourrie... Pas non plus. Ce n'est pas meilleure des solutions non plus. Mais je pense que jusque-là, il n'y a pas de soucis pour la manger quand même.

*Organisatrice:* D'accord.

*Participante:* Mais c'est juste que je n'aime pas après.

*Organisatrice:* Voilà, c'est ça. Ce n'est pas ça qui va venir régir votre décision.

*Participante:* C'est ça. Mais là, à partir de là, je me poserais sérieusement des questions quand même.

*Organisatrice:* À partir du rond jaune, sur une la-- l'éventuelle toxicité...

*Participante:* Toxicité, peut-être pas, mais... Je ne sais pas. Je n'ai pas du tout envie de manger cette chose.

*Organisatrice:* Ouais.

*Participante:* C'est quoi la réponse ? Du coup, il y a une toxicité ou pas ?

*Organisatrice:* Moi, je ne sais pas encore. Il doit y avoir une réponse, mais je ne sais pas. Mais [euh] justement ça m'intéresse de savoir si les personnes-- [en gros] si elles choisissent le moment de consommation en fonction peut-être-- [enfin] du moment où elles jettent en se disant : « [Bah] Non là, ça y est. Ça devient dangereux, je ne peux plus la manger », ou alors : « Là, c'est trop dangereux », ou : « Je ne digère pas assez » ou je n'en sais rien, du coup j'attends. Ou si c'est plutôt relatif au-au goût [oui] et c'est surtout relatif au goût.

*Participante:* Moi, j'ai toujours entendu qu'une banane pas mûre, ce n'était pas bon pour la santé.

*Organisatrice:* Oui. C'est plus ça que--

*Participante:* Trop mûre, je pense que c'est [alors] peut-être sur l'indice glycémique aussi, ça joue.

*Organisatrice:* Oui ?

*Participante:* [En fait] Une banane moins-- moins mûre, l'indice glycémique, c'est-- comme c'est peut-être moins chargé en sucre, je ne sais pas comment expliquer ça-- mais ça a un indice glycémique plus faible tandis qu'avec une banane comme ça, à mon avis, l'index glycémique, c'est--

*Organisatrice:* Oui. [Alors] Ouais, justement sur les-- sur les apports nutritifs de la banane. Est-ce que selon vous il y a un moment donné ou elle est optimale ou pas ? Est-ce que-- [euh] est-ce que ça reste ?

*Participante:* Je n'en sais absolument rien.

*Organisatrice:* Donc c'est pareil, ce n'est pas ça qui va guider votre-- [euh] forcément votre façon de-de manger la banane.

*Participante:* Par contre, par rapport un autre fruit, je vais me dire je vais choisir une pomme plutôt qu'une banane, c'est moins calorique.

*Organisatrice:* D'accord.

*Participante:* Les clémentines, par exemple, moi c'est-- l'hiver, c'est les clémentines.

*Organisatrice:* Oui, oui.

*Participante:* [Mm-hmm] Par contre, quand j'ai très faim, et que j'ai besoin d'être calée. Je mange la banane.

*Organisatrice:* Oui, donc c'est-- s'il n'y a pas de notion sur le moment de son apport nutritif, il y a quand même une connaissance sur ses calories et que ça-- que ça cale bien.

*Participante:* C'est ça. T'arrêtes de te gratter ? T'as des puces ou quoi ?

*Organisatrice:* OK pour la banane.

*Participante:* Ça fait literie.

*Organisatrice:* Elle a quel âge ?

*Participante:* Elle a trois ans.

*Organisatrice:* Ça va, là, c'est calme. En même temps trois ans...

*Participante:* [Han] Heureusement, c'était une terreur.

*Organisatrice:* [Ah] Oui ?

*Organisatrice:* [chuchotements 00:46:31 - 00:46:41]

*Organisatrice:* [Alors] Le dernier petit jeu. [Alors] Est-ce que vous composter ? Vous m'avez dit que oui, non ?

*Participante:* On n'a pas de composteur ici. On aimerait bien mais...

*Organisatrice:* [Ah] Oui, c'était que si vous aviez un potager. [Alors] Du coup, c'est plutôt une poubelle classique. Pas de poules, j'imagine. Hop ! [Alors] Poubelle classique... Ici, j'ai différentes choses qu'on peut faire avec [euh] des fruits et légumes frais. Je vais vous demander de garder-- de disposer celles qui vous parlent, des choses que vous pouvez faire, et de-- d'exclure ce que-- [voilà]

*Participante:* Ce que je ne ferais pas.

*Organisatrice:* Oui.

*Organisatrice:* [silence 00:47:22 - 00:47:37]

*Participante:* [Oh bah] Je fais tout donc...

*Organisatrice:* [Ah bah] Très bien. Parfait. [Alors] Oui, on peut les disposer un peu dispatchées, un peu partout. Merci. Et [alors] là, je vais vous donner des fruits et légumes et je vais vous demander de décrire ce que vous voyez et de me dire ce que vous aurez fait et de les mettre aux endroits estimés.

*Participante:* D'accord.

*Organisatrice:* Là-Là où il y a la poubelle, ou sinon il y a plein de préparations ou de conservations.

*Participante:* Ça ne peut pas aller sur plusieurs ?

*Organisatrice:* [Bah alors] Dites-moi, qu'est-ce que vous--

*Participante:* [Bah] Là, pour le trognon, j'aurais pu le mettre en compote, en smoothie ou en-en tarte.

*Organisatrice:* OK. Donc [euh] vous la cuisinez [quoi].

*Participante:* Je la cuisine.

*Organisatrice:* OK.

*Participante:* Ça, je jette.

*Organisatrice:* Le verre de poireaux.

*Participante:* [Bah] Je sais que je peux en faire des bouillons de légumes là, [enfin] des-des réductions, mais j'ai jamais pris le temps de le faire.

*Organisatrice:* Oui.

*Participante:* Ça, je coupe un bout, mais je mets le reste en salade, ça ne me dérange pas.

*Organisatrice:* OK.

*Participante:* Ça, c'est pareil, je recoupe un bout et [hop] ça va sur la salade, par exemple.

*Organisatrice:* OK.

*Participante:* Je n'aime pas les radis. Mais là pareil, je coupe un bout et ça va dans ma salade.

*Organisatrice:* OK. Ça, je jette. La tomate est trop-trop moisie.

*Participante:* [Alors] Je ne mange pas d'endives, [alors] je ne sais pas comment-- si on l'épluche, si ça peut être mieux ?

*Organisatrice:* [Bah] Si vous n'en mangez pas, c'est vrai que c'est compliqué. On peut l'enlever, si vous voulez, parce qu'il n'y aura pas d'endives donc [euh]...

*Participante:* Je vous les échange en bonbons ?

*Organisatrice:* Pas de soucis.

*Participante:* [Hm] Ça...

*Organisatrice:* C'est une courgette.

*Participante:* J'enlève les bouts qui ne vont pas et j'en fais une soupe.

*Organisatrice:* OK.

*Participante:* [Alors] Le problème, c'est que parfois, quand c'est trop-trop mûr, ça donne un goût pas bon du tout à la soupe.

*Organisatrice:* Oui, je--

*Participante:* Donc en règle générale, je goûte pour savoir si... Ça je ne cherche pas.

*Organisatrice:* La carotte est trop-trop atteinte.

*Participante:* [Alors] Ça, j'ai eu le cas hier, j'ai enlevé les cas pas-- les trucs pas bons, j'ai congelé. [Enfin] J'ai coupé en petits bouts, j'ai congelé.

*Organisatrice:* OK. C'était sur un poivron aussi ?

*Participante:* Oui, exactement, poivron rouge et poivron jaune. J'avais un petit bout moisi, j'ai enlevé le moisi, puis j'ai-- mis en petits bouts et j'ai congelé.

*Organisatrice:* OK.

*Participante:* Parce que je ne comptais pas en faire, là, dans les jours à venir. Enfin, j'avais prévu à l'origine, puis j'ai trouvé une autre recette et j'ai fait autre chose. [Oh] Ça, je coupe ce qui ne va pas et puis, salade de fruits, ou jus, ou smoothies...

*Organisatrice:* Oui.

*Participante:* Ça, j'enlève les bouts pas bons et compote ou smoothie.

*Organisatrice:* OK.

*Participante:* Mais sans grande conviction. Ce n'est pas que je préfère.

*Organisatrice:* Oui.

*Participante:* [Oh bah] La tomate en salade ou en tarte, ou en-- ou si je ne veux pas les faire tout de suite, qu'il m'en reste, je les congèle. Ou dans une préparation, il y a plein de préparations. [Hm] Ça, je coupe les bouts qui ne vont pas et je le mets en soupe.

*Organisatrice:* OK. [Bon bah], il y a quand même pas mal de fruits et légumes de sauvés !

*Participante:* [Bah] J'essaye [hein] mais [bon] là, celle-ci, je ne l'aurais peut-être pas gardée, mais celle-là, elle me semble encore capable.

*Organisatrice:* Oui. Vous n'auriez pas jeté les deux ? Je vais prendre en photo.

*Participante:* Du coup c'est un peu...

*Organisatrice:* Ouais mais ne vous inquiétez pas, ça me donne une idée. On voit la tête du chat, elle me fait rire.

*Participante:* C'est une vraie princesse, hein Lili ?

*Organisatrice:* Elle est marrante. [Bon bah] Chouette, merci beaucoup. J'ai fait tous mes petits jeux. [Euh] Pour finir sur la-- sur la poubelle connectée. Comment ça s'est passé sur la deuxième période ?

*Participante:* [Bah] On n'a pas jeté grand-chose parce que les fruits-- les fruits d'hiver et les-- et les légumes d'hiver tiennent beaucoup plus longtemps que ceux d'été.

*Organisatrice:* Vous avez remarqué que vous avez moins jeté ?

*Participante:* Ouais, beaucoup moins. Puis je fais beaucoup plus de soupes donc un légume qui est un peu abîmé [hop] ça va dans des-des tartes. Donc pareil pour les fruits, très facilement, ça va dans une tarte.

*Organisatrice:* Donc vous avez le sentiment-- par exemple, du coup, l'été, il y a moins de [euh] solutions [euh] comme ça de--

*Participante:* [Bah] Surtout des fruits qui partent super vite [quoi].

*Organisatrice:* Oui.

*Participante:* En 24 heures parfois, c'est fini [quoi].

*Organisatrice:* Ouais, ça, c'est vrai. [Bah] C'est bien pour ça qu'on avait regardé la saisonnalité, justement, [euh] je pense que vous êtes nombreux, nombreuses, à avoir fait ce constat. Vous jetez quand même moins l'hiver que-que l'été, et parce que les fruits et légumes se conservent relativement mieux. Et en plus, parce qu'il y a les soupes [et tout], ça--

*Participante:* Ça part plus vite [quoi].

*Organisatrice:* Oui, plus facilement.

*Participante:* On achète peut-être moins l'hiver aussi, parce qu'on se dit : « [Bah] Les courses-- [tout ça] » ; après on se dit : « [Bah] Il y en a-- ». Avec l'été [en fait] il y a ce côté hyper facile [genre] : « [Oh] Là, j'ai envie plein de tomates, j'ai envie de plein de fruits, j'ai envie... »

*Organisatrice:* Oui, oui. Toujours, on a envie de manger de plein de choses fraîches et--

*Participante:* C'est ça.

*Organisatrice:* Et on n'a pas envie de manger des trucs lourds donc les fruits et légumes ont du succès, et puis au final, des fois, on mange autre chose.

*Participante:* Finalement, ça finit en coquillettes au beurre.

*Organisatrice:* C'est ça !

*Participante:* On va au magasin : « [Oh] Des fruits, pastèque, trop bien ! ».

*Organisatrice:* C'est ça. Et puis quand la fin arrive [allez hop], la plâtrée.

*Participante:* Moi, aussi je fais 35.

*Organisatrice:* C'est ça, je connais. [Bon bah] Chouette, merci beaucoup !

*Participante:* Non, [bah] avec plaisir !

*Organisatrice:* Hop ! On va couper.

**Household F27, Interview 1**

**Pour commencer, je vais vous demander de penser à un légume ou à un fruit que vous aimez tout particulièrement. L'idée, c'est que vous allez penser à ce légume ou à ce fruit sans jamais révéler son identité. Je vais devoir le deviner juste avec votre description, comme un petit jeu de devinettes. Est-ce que vous avez un légume ou un fruit frais en tête ?**

Ouais

**OK, alors je vous écoute, dites-moi, tout sur ce légume ou ce fruit.**

Alors, ça va être assez… c'est jaune, c'est un peu courbe.

**Ok…**

C'est tout. Avec ça, vous devriez trouver. Et c’est un fruit.

**Qu'est-ce que vous aimez tout particulièrement dans ce fruit ?**

Je ne sais pas pourquoi on aime un fruit… Le goût. Y a pas mal de magnésium je crois dedans. Donc... J'aime bien, surtout le goût.

**Ouais… En général, vous le mangez quand plutôt le matin, plutôt le midi, l’après-midi…**

Souvent l'après midi

**L’après midi ?**

Ouais

**C’est un fruit que vous pouvez grignoter comme ça ?**

Oui, ouais ouais. Souvent l'après midi, quand j'ai… à 4 heures, quand j'ai un petit creux, je prends ça, un fruit.

**Ok. Ca marche. En général, vous l'acheter où, ce fruit ?**

Sur le marché

**Sur le marché ? Alors, est ce que vous pouvez me raconter les différentes étapes avant que vous consommiez votre fruit ? Là, il y a votre fruit… Qu'est ce que vous fa14ites avant de le mettre dans votre bouche ?**

Je l’épluche.

**Vous l’épluchez ?**

Après c’est tout, il y a que… et je le mange.

**C’est la seule façon que vous le mangiez ou des fois vous le manger différemment ?**

Celui-là, il y a pas 36 façons de le manger *(rires)*. Il faut l’éplucher d'abord, et le manger après. Je n'ai jamais vu encore de personnes manger la peau

**D’accord *(rires).* Selon vous, la peau ne se consomme pas ?**

Selon moi, oui, mais bon, maintenant, on trouve un tas de trucs.

**Mais vous, non, vous ne la mangez pas.**

Non

**Vous m’avez dit qu’il vous apportait du magnésium, que vous aimez le goût, que vous le mangez pendant un petit creux. Est ce que vous lui trouvez d'autres avantages ? Est-ce que vous avez le sentiment qu'il vous apporte d'autres choses encore, ou pas ?**

Souvent, quand j'ai un petit creux, il cale quoi.

**Ok…**

D'autres avantages… ? Euh.. non, non.

**Ok, et est-ce qu’à l’inverse vous trouvez des inconvénients, à ce fruit ou pas ?**

Non.

**Non ?**

Je n'aime pas trop quand il est un peu trop mûr, donc je préfère… qu'il soit bien, mais pas trop mûr.

**D’accord. Sur la maturité, il faut que vous le mangiez assez vite, sinon vous n’aimez pas le...**

Ouais

**Ok. Je pense trouver…, je dirais la banane ?**

Ouais. C'était pas trop que difficile je pense *(rires)*

**C’est vrai que jaune et courbe, j’avais déjà une idée *(rires)***

Ouais

**Désormais, j'aimerais qu'on discute de notre organisation générale en termes de courses alimentaires. Vous avez dit acheter, par exemple les bananes sur le marché. Est ce que c'est le cas de tous vos fruits et légumes, ou pas?**

Tous mes fruits et légumes, oui.

**D’accord…**

Pratiquement… ça m’arrive d'en acheter quelques uns, rarement, au supermarché. Mais pratiquement**,** tous sur le marché.

**Et pourquoi vous choisissez de prioriser le marché ?**

Parce que d'abord, c'est souvent des produits de la région et ils sont moins manipulés par tous les gens que dans un supermarché, on ne sait pas qui manipule les. Et le prix aussi, bon la qualité est meilleure déjà, prix et qualité meilleurs… encore que le prix, je sais pas trop, mais bon, la qualité, le prix, c'est… Ça tient, ça revient un peu dans… alors comment expliquer ça, c'est pas le souci primordial.

**D’accord… Vous pensez que c'est un avantage au niveau du prix, mais ce n'est pas pour ça que vous allez au marché quoi..**

Non non. Si c'était un peu plus cher le marché, j'achèterais quand même sur le marché, parce que c’est des produits de la région, souvent des produits français, donc..

**Et quand vous dites qu'ils sont moins manipulés, pourquoi pour vous, c'est… pourquoi c'est mieux quand ils sont moins manipulés ?**

Il y a moins de bactéries déjà… moins de… ils sont moins flétris et pourrissent moins vite.

**Ouais. Et le marché, vous y allez… Ça fait combien de temps que vous avez cette habitude d'aller au marché ?**

Ca fait un moment… Ça fait un ou deux ans je pense.

**Et avant vous les achetiez où vos…**

Chez Leclerc, au supermarché quoi.

**Et vous trouvez une différence du coup dans la façon dont on se conserve ?**

Euh, oui. Souvent, il se conserve beaucoup mieux quand j'achète sur le marché... Et la qualité je trouve est meilleure aussi. Et ça permet de…sur le marché aussi, de discuter avec la vendeuse pour savoir quels sont les fruits de saison…. Voilà.

**Oui, ça vous arrive de demander conseil ?**

Oui, par exemple pour les pommes de terre, lesquelles pour les pommes sautées ? Lesquelles pour la purée ? C'est pas les mêmes, donc…

**Selon la recette, vous n’allez pas prendre les mêmes.**

Ouais ouais.

**D’accord. Et justement, à ce propos, est ce que quand vous allez sur le marché, vous savez ce que vous allez acheter ou pas vraiment ?**

Ouais, si. J’ai ma liste, après des fois, je rajoute, je rajoute un ou deux trucs en regardant sur le marché où je change quoi. Mais j'ai, j'ai ma liste définie avant d'y aller, quoi.

**D’acord. Et comment vous la faites cette liste ? Comment vous rédigez vos…**

D'après ce qui… d’après ce que j'aime, par exemple ces temps ci, on va prendre ces temps ci, c'est souvent des tomates quand il m'en reste plus. Donc je prends un avocat par semaine. Melon, ces temps-ci parce qu’il y du melon et j'adore le melon. Euh… des carottes pour faire des carottes râpées, je change des fois, c’est des concombres. Après, c'est des fruits, donc des pommes, généralement, ou des… après je vois ce qu'il y a, avant, il y a une semaine ou deux, c'était des clémentines, mais il y en a plus, donc je vais prendre d'autres choses, je vais prendre des abricots, ou voilà. Après je peux rajouter des radis, ces temps-ci, parce que j’adore les radis. Voilà, c’est à peu près le style.

**D’accord, donc si je reprends ce que vous dites, vous allez noter sur votre liste ce que vous aimez que là vous n’avez plus, ce que vous allez vouloir consommer dans la semaine et que vous n'avez plus. Et les fruits, du coup, vous prenez... Comment vous gérez vos quantités, finalement ?**

Euh… je vois à peu près ce qu'il me faut par semaine, là, il me reste quatre pommes, des fois il m'en reste une ou deux, donc j’en prends un peu moins…Voilà. Je sais à peu près… je fais les courses le vendredi matin, donc je m'organise pour la semaine quoi, sachant que ça tient, ça tient une semaine quoi les fruits.

**D’accord. C’est tous les vendredis matin que vous allez ?**

Ouais quand je peux. Généralement oui.

**Si vous avez autre chose… le marché est plusieurs fois dans la semaine ?**

Oui, le mardi, vendredi et samedi, donc sans ça c’est le samedi donc euh...

**D’accord. Et le reste de vos courses ?**

Le reste de mes courses, je les fais en supermarché, chez Leclerc ici.

**Et en termes de fréquence, est-ce que c’est pareil, une fois la semaine que vous allez…**

Une fois, une fois la semaine chez Leclerc et une fois la semaine, les fruits.

**D’accord. Ok… Alors, vous l'avez déjà un peu fait... Là l'idée c’est de dresser un peu une liste de tous les fruits et légumes que vous allez consommer régulièrement. Vous avez déjà dit tomate, avocat, melon, carotte ou concombre, radis… Est ce qu'il y a d'autres légumes que vous allez vraiment consommer régulièrement ?**

Ah oui, après, il y a certains légumes, c'est du surgelé, quoi. Ou de la conserve mais plus du surgelé, donc tout ce qui est brocoli, j'achète en surgelé, mais là, c'est sur… c’est chez Leclerc, où j'achète certains trucs chez Thiriet et en… Mais c'est sur place donc brocolis, choux fleurs, haricots verts… Bon c’est à peu près tout… ouais petits pois.

**D'accord, et pourquoi vous préférez consommer ces produits là, surgelés ? Est-ce qu’il y a une raison particulière ?**

C’est plus fort.., c'est plus pratique en surgelé. J'achète souvent des surgelés précuits. Donc après, on les met 3 minutes au micro-ondes, et c'est prêt.

**D’accord, c'est surtout pour un côté pratique que**

Ouais.. Moi, je ne suis pas un fin cuisinier, quand même.

**Vous aimez cuisiner ou pas vraiment ?**

Ça m'arrive. J'aime bien cuisiner. Oui, mais après… j'achète aussi des courgettes, mais là, je les prépare, c'est en… c’est sur le marché aussi. Courgettes, je mets des poivrons avec.

**D’accord. Est ce qu'il y a des légumes que vous connaissez et que vous allez vraiment, jamais consommer, ou très rarement ?**

Ouais les salsifis… ça fait un moment que je n'ai pas consommé, mais je n'aime pas trop. C'est à peu près tout, le reste... Lentilles, j’en fait assez régulièrement… Haricots, les autres haricots là, les flageolets aussi, donc… non…

**Non, vous n’excluez rien ?**

Non, asperges aussi, j'en fais. Asperge, en frais ou en boîte quoi. En frais, suffit de les cuire à la poêle, c'est très bon aussi. J'en fais de temps en temps. Non. C'est à peu près tout.

**Donc vous avez en tête le salsifi, parce que vous n’aimez pas trop finalement ?**

Ouais, sans ça le reste… si la rhubarbe... Je ne sais pas trop comment ça se cuisine. J'adore les tartes à la rhubarbe, mais on n’en trouve pas beaucoup dans le coin. Avant, il y en avait une pâtisserie qui en faisait mais elle a fermée depuis.

**Ah… Et du coup, vous, est-ce que vous achetez de la rhubarbe ou non ?**

Non, non, non, j'ai demandé une fois, mais je sais pas trop. Je ne me rappelle plus trop comment il m'avait dit de la cuisiner, mais bon, c'est assez compliqué d'après ce que je me rappelle là donc euh *(rires)*, je vais pas me lancer dans des trucs…

***(rires)* Donc vous l’aimez mais vous ne savez pas trop comment la cuisiner…**

Je l’aime en tarte, ça c’est sûr.

***(rires)* Du coup vous ne vous êtes pas lancé dans la tarte, vous-même..**

Ouais non.

**Et si on s'intéresse aux fruits, justement, est ce que vous avez… donc vous achetez régulièrement vous dites les pommes, les bananes euh… les clémentines**

Ouais des clémentines, des poires… Ça dépend des saisons, quoi… Des cerises quand ils ne sont pas trop chers… Des fraises aussi. Ça m'arrive de temps en temps d'acheter une barquette. Qu'est-ce qu’il y a… les abricots , j'aime bien aussi… les prunes. J'aime bien à peu près tous les fruits alors… donc euh… Melon aussi, quand c’est… je ne sais même pas si c’est un fruit ou un légume le melon donc euh… *(rires)* c’est un fruit à priori

***(rires)* Je ne sais pas non plus…**

*(rires)* Vous devriez savoir, alors !

**Je ne suis pas encore experte en fruits et légumes !**

Oui non, si même pruneau je prends aussi… J’ai fait le tour à peu près.

**Il n'y a pas de fruits que vous allez exclure de votre consommation ?**

Des fruits que j’exclue ? Non, à priori ça me dit rien…

**Les fruits de la passion, par exemple est-ce que vous en consommez ? Les fruits exotiques, il y a la banane**

Ouais les kiwis, j'en prends de temps en temps. Ouais, ouais, fruits de la passion. Non, je suis pas.. ! J'aime bien en glace ou en… mais bon comme ça.

**Ouais… Par exemple l'ananas, ça vous arrive d’en acheter ou pas du tout ?**

Ah ouais l’ananas en boîte parce que… ouais, l’ananas ouais.

**Et pourquoi, par exemple, l’ananas, vous allez l'acheter en boite et pas en frais ?**

Enfin, c'est… pour moi, c'est plus pratique. Ou alors je l’achète en barquette. Des fois, ils font des barquettes chez Leclerc en tranches quoi donc euh....

**Du coup c’est au niveau de la préparation que vous trouvez ça plus simple ?**

Ouais… faudrait que j’essaye une fois, acheté un demi… parce qu’un ananas entier… Moi, je suis tout seul. Donc ou alors les mettre en congélation après. Donc, je sais même pas… ouais.

**Ouais sinon ça vous demande de manger une partie et de congeler le reste..**

Ouais, ouais.

**Ok… Ca marche. Alors désormais, j’aimerais que l’on parle de vos lieux de stockage. Donc là, il y a une corbeille. Et justement, quand vous rentrez du marché, qu'est ce que vous faites de vos fruits ?**

Oui, je les range, donc les fruits… Donc, il y a une partie dans la corbeille, une partie dans le bac à pratiquement tout dans la corbeille, une partie dans le bac à légumes, les fruits comme le melon, pruneau aussi je les garde au frigo… euh… bon pruneau, des fois, je l'achète, des fois en frais sur le marché, des fois chez Leclerc, des marques Pruneau là, Maître Prunille. Donc voilà, c’est tout en fruits. Chez soit la corbeille, soit là quoi… Quand c'est des fraises, je les prépare, je les lave, je les prépare. J'en mange une partie le midi et après, le reste, je mets au frigo pour le lendemain.

**D’accord, vous faites des restes... Et les légumes ?**

Les légumes, donc les légumes… il y a une partie qui vont là

**Donc c’est comme une panière.**

Ouais.

**D’accord**

Et une partie qui va dans le bac à légumes aussi. Et j'ai acheté un bac pour les pommes de terre aussi pour mettre les pommes de terre, je ne sais pas si on en avait parlé l'autre fois, que ça germait assez vite, donc j'ai acheté un bac. Je vous ferai voir tout à l'heure. Ils sont comme ça, à l’abri. Ils sont à l'abri du soleil aussi.

**C’est ça. Et comment vous allez décider des légumes que vous allez mettre dans la panière et ceux que vous allez mettre plutôt dans le bac à légumes au frigo ?**

Alors il y a certains, certains légumes, par exemple les tomates, il parait que ça se met pas au frigo. L'avocat aussi, par exemple… D'autres, alors, souvent, les carottes, je les mets parce que je les prépare pas... Il m'en reste toujours un peu… ou c’est pas le jour où que je prépare, donc je vais laisse au... Après, par exemple, les radis, quand je les achète, ils vont… je les épluche généralement le jour même et ils vont au frigo après, parce que… ouais après il y a certains légumes qui vont au frigo, d'autres pas du tout.

**D’accord donc les tomates que vous laissez en dehors, des oignons aussi.**

oignon, avocat,

**avocat, pomme de terre…**

Pommes de terre, euh, qu’est-ce qu’il y a… courgettes aussi quand j’en achète. Quand j'achète un concombre, je le mets au congélateur euh au réfrigérateur en attendant que je le prépare.

**Ok… Est ce que cela vous semble être un stockage idéal ou pas ? Est ce que vous voyez… est-ce que pour vous, c'est…**

Pour moi, oui, je perds, je perds rien. Je m'arrange pour que ce soit, d’être mangé ou préparé avant que ça se perde, donc..

**Par exemple, vous m'avez parlé de la pomme de terre, qui était peut-être mieux sans lumière. Est ce que vous avez d'autres idées comme ça, de conservation pour les fruits et légumes ou pas ? Par exemple vous m’avez dit aussi que pour vous les tomates et l’avocat ça ne va pas au frigo. Est-ce que vous avez d'autres idées de conservation ?**

Non…. J'avais lu les tomates, pas les laisser au frigo. Je sais pas… Après, c'est une question d'habitude, j'avais vu que les carottes, quand on les laissait… si je les prépare pas le jour même, quand on les laisse dehors, ils dessèchent. Alors, je les mets… je les laisse dans leur emballage papier, et je les mets au frigo en laissant le papier ouvert. Donc ça permet, ça les garde plus longtemps. Elles bougent pratiquement, elles bougent pas. Pareil pour le concombre. Si je le fais pas le jour même, je le mets au frigo. Ça bouge pas.

**D’accord. Et vos tomates ici vous trouvez qu’elles se conservent bien ? 25’00**

Ah oui oui, bah je surveille, après là je les ai acheté… ce samedi là, ils sont pas encore…. ils sont pas trop trop mûrs, mais je vais commencer ce soir, je pense. Je vais en prendre une et je surveille. Et au fur et à mesure qu’elles mûrissent je les prépare. Je m'arrange pour pas perdre de fruits ni de légumes.

**Ouais là par exemple, vous avez 5-6 tomates, vous regardez**

Ouais je regarde pratiquement tous les jours voir dans quel état ils sont. Et si par exemple, ils sont un peu un peu plus mûrs qu’il m’en reste deux ou trois, je peux faire des tomates farcies, j'achète de la farce et voilà. Ou alors les mettre avec les courgettes pour faire des courgettes cuisinées et après… et après, je fais des bacs que je mets au congélateur, de courgettes qui me servent après.

**Quand vous voyez que… si vous trouvez trop mûr pour être mangé au final un peu cru, vous avez tendance à la cuisiner quitte à après mettre la préparation au congélateur.**

Ouais, ouais ouais.

**Ok. Et sur les lieux de stockage, est ce que ça vous arrive de les nettoyer du coup ? Que ce soit la corbeille, la panière…**

La panière ? Non, ça fait un moment que je l’ai pas nettoyé ça… La corbeille, ça arrive de temps en temps, et le bac aussi. Bon, c'est une fois tous les… je sais pas trop… Ça dépend comment c'est. Quand je vois que c'est un peu trop sale, je nettoie. Mais bon, c'est pas, c'est pas régulier, je me dis pas « faut que je le fasse tous les… » après, peut-être, ça suffit pas, faudrait peut-être faire plus souvent, ça, on ne sait pas *(rires)*

***(rires)* Je ne sais pas non plus ! donc quand vous voyez que c’est un peu sale, c’est là que vous vous dites que je passe un coup quoi.**

Ouais ouais.

**Ok ça marche. Alors là, je voudrais qu'on discute justement de votre façon de préparer et consommer les fruits et légumes, vous m’avez dit déjà par exemple, les tomates quand elles commencent à murir vous avez tendance plutôt du coup à les préparer un peu cuite pour éviter de manger cru. Si on prend un fruit…**

Ouais

**Euh… est-ce que banane vous m’avez dit la manger cru. La rhubarbe vous l’aimez en tarte mais au final vous ne la cuisinez pas. Est-ce ça vous arrive de cuisiner des fruits ou pas ? Ou vous êtes plutôt dans le cru ?**

Le plus souvent, c'est du cru. Les fruits, ça m'arrive de temps en temps… Les pommes, par exemple, quand je mange du boudin noir, je fais des pommes à côté dans, à la poêle avec un peu de beurre. Ça c’est très bon, le boudin pommes. Sans ça, les autres… Non, rarement les fruits, je les cuisine.

**D'accord. Et si on prend la pomme, par exemple, comment vous la… vous avez la pomme, tout à l’heure vous m’avez raconté pour la banane vous l’épluchez et vous la mangez. Et la pomme comment vous la consommer ?**

Comment je la consomme ? Je la lave d'abord, bon avant je l’épluchais parce qu’on nous raconte qu'il y a plein de pesticides et donc j'ai posé la question il y a pas longtemps, il y a deux semaines ou trois semaines, s'il y avait des pesticides sur les fruits, on m’a dit il y en a de moins en moins. Donc maintenant, je la lave et je mange la peau, donc je la coupe en quartiers, j'enlève le milieu et je la mange.

**Donc avant, vous aviez l’habitude de l’éplucher, mais maintenant vous mangez la peau.**

Oui, pareil pour la poire, les autres euh... Bon, de toute façon, je les lave toujours les fruits.

**Systématiquement vous lavez vos fruits ?**

Ouais.

**Ok. Et si on prend les légumes, si on prend un légume que vous consommez régulièrement, par exemple la courgette, vous avez commencé à me raconter… alors est-ce que vous pouvez me raconter comment vous la consommez ?**

Généralement, je la prépare donc je l’épluche, je prépare en, soit en rondelles, soit en cubes, je la coupe… Donc je la mets dans une sauteuse avec de l'huile d'olive, je rajoute un demi poivron avec une tomate et voilà et un fond de veau aussi généralement et un peu d'eau. Et je laisse cuire. Des fois, je fais avec de la saucisse aussi que je mets après aussi… voilà

**Ok, vous la faites à la poêle bien souvent ?**

Oui ou à la sauteuse. La courgette, c'est souvent comme ça que je le fais. Mais après, comme j'en prépare deux ou trois, ça me fait quatre ou cinq repas, ça dépend. Donc une fois, j'en prends une fois. Après, j'en laisse une autre fois dans le frigidaire pour le lendemain ou le surlendemain. Et le reste, je mets en petite barquette et je mets au congélateur pour sortir quand j'ai besoin ou quand j'ai envie.

**Ok. D’accord. Et vous avez dit que vous l’épluchez la courgette ?**

Oui. Il y en a qui après, ça dépend aussi. Parce que des fois, je ne l’épluche pas. Mais je la lave à ce moment là, je la grappe un peu. Je la lave en la brossant un peu. Et là aussi, la peau, il paraît qu’elle est plein de vitamines, donc euh… voilà.

**Ouais, et la carotte ?**

Alors, la carotte, c'est souvent des carottes râpées ou des carottes en rondelles, préparées pareil, un peu pareil que les courgettes. Je rajoute fond de veau, un oignon, toujours… voilà. Les carottes, je les épluche et je les rappe. J'ai un truc pour râper et je mets 2 ou 3 oignons euh, avec échalotes dedans comme ça il y a ma préparation, échalotes et carottes, faites pour deux ou trois jours.

**Ok. Plus généralement, vous la mangez la plus souvent en râpé ?**

Le plus souvent, oui.

**Et ça peut vous arriver de faire un peu une préparation chaude comme la courgette ?**

Oui, ouais ouais.

**Ok, ça marche.**

Les autres, que ce soit brocolis, choux fleurs, tout ça, c’est plus en en surgelé, par exemple ce midi, j'ai sorti des brocolis, ce matin, surgelés, donc ils sont dans la boite là et je mettrais au micro-ondes 3 minutes et c’est prêt.

**D’accord, ah oui, car ils sont déjà pré-cuirts ?**

Oui, ils sont précuits.

**Donc ils sont déjà préparés, assaisonnés, tout ça ?**

Ah non, j’assaisonne pas beaucoup. Après, il y en a qui cuise au beurre et tout ça. Donc j'évite tout ça parce que j'ai tendance à grossir. Donc voilà… *(rires)* non, non. Ou alors si je mets un peu de beurre, c'est du beurre non cuit, quoi, du frais. Et encore, ce n'est même pas du beurre, c'est… une sorte de margarine quoi.

**D’accord, je vois. Donc là les brocolis, vous les mangez comme ça, un peu réchauffé quoi.**

Ouais

**Ok. Alors, est ce que vous avez en tête, des légumes que vous allez consommer sans… et ne laisser aucune épluchure, ou pas ? Par exemple la courgette, des fois vous l’épluchez pas, vous la brossez, mais bien souvent… il y a les extrémités.**

Oui, les extrémités, je les coupe.

**Donc vous les enlevez, et est-ce que vous avez en tête des légumes que vous allez consommer, où il n'y aura pas d'épluchures, pas de reste**

Hoff…

***(rires)* C'est une question qu'on se pose.**

Non, je vois pas. Les légumes, carottes… les légumes sans épluchures euh… faudra me trouver ça parce que…

**C’est ça ! Et les pommes de terre, est-ce que vous épluchez la peau ou pas, par exemple ?**

Généralement oui, oui, mais là, j'en ai fait... Quand c'est dimanche là, a priori, c'est des pommes de terre nouvelles, donc j'ai pas épluché, j'ai gratté la peau et je les ai fait à l'eau, pour faire des pommes vapeur, quoi. Mais souvent, alors soit c'est ça, soit de la purée, donc pareil, mais je les coupe en plus petit pour l'écraser après, mettre un peu de lait et de beurre, pour faire une purée ou sans ça, je l'ai fait poêler avec un peu de graisse de canard aussi, au lieu de… du beurre.

**Vous mettez la canard à la place du beurre ?**

La graisse de canard, ouais.

**Ah ouais ?**

Oui, c'est très bon ça, ça, c'est une recette de Dordogne. On était en Dordogne, j'ai de la famille en Dordogne et on vend ça de la graisse de canard. Faut pas en mettre énormément. Il faut pas en manger tous les jours, bien sûr, au lieu de mettre du beurre, on met de la graisse de canard et c'est… après, vous coupez vos pommes de terre en cubes et vous laissez dans une poêle cuire vos pommes de terre et c'est excellent !

**Et ça vient de Dordogne. Ça marche. A présent, j'aimerais qu'on échange sur le moment où vous jetez un fruit ou un légume, ou une partie seulement. Quand ça arrive, pourquoi décidez vous de le jeter plutôt que le consommer ?**

Souvent, c’est parce qu’il est pourri hein.

**Parce qu’il est pourri ?**

Ouais.. il y a que cette solution là, je jette presque rien. Pratiquement rien dans….

**Et comment vous déterminer que là, le fruit ou le légume**

Des fois, ça arrive où il y a de la pourriture dessus… Mais il n'y a que dans ce cas là, parce que après, quand ils sont trop mûrs, ça passe quand même pas. Faut pas qu'il y ait de pourriture dessus. Mais j'attends pas… Je m'arrange pour… pour manger les fruits avant que ça soit trop mûr.

**Du coup, quand vous voyez de la pourriture, comment réagissez-vous ?**

Par exemple, ça m'est arrivé il y a un petit moment sur une courgette où la courgette avait pratiquement pourrie, une partie donc.. J'ai coupé la partie qui était pourrie. Je l'ai jeté et j'en ai parlé à ma commerçante qui m'a dit « vaut mieux les garder », je les avait laissées là et il vaut mieux les garder au frigidaire et elle m’a changée quand même, ma courgette.

**D’accord, donc vous avez eu une courgette, une partie de la courgette, pourrie, vous avez jeté juste la partie pourrie.**

Ouais

**Cette courgette étaient stockées dans la panière, et sur le marché, la personne à qui vous vendez vous a dit qu’il fallait la stocker au…**

Oui, oui. Et ça m'est arrivé sur des clémentines aussi. Pareil, là aussi on m'avait dit de les garder plus au frigidaire, parce que…

**Ah ouais ?**

Ouais ça les déconsommait assez vite. Après quand c'est comme ça, je consomme ceux qui, par exemple, les pommes ça se gardent assez longtemps, donc je consomme les fruits qui sont plus délicats, donc en priorité, après la pomme… De toute façon j'essaye de manger deux fruits par jour, donc généralement c'est une pomme avec autre chose quoi.

**D'accord. Et pourquoi vous avez cette… s’il y a une raison, d'avoir ces…**

Deux fruits par jour ? Donc j'avais été, j'avais été voir un nutritionniste qui m'avait fait un plan de repas et il m'avait dit, vous avez le droit à deux fruits par jour, mais pas en même temps. Donc j'essaye de… J'ai perdu 10 kilos, même plus. J'en ai repris un peu, depuis. Mais bon, maintenant, j'ai… donc.. J'essaye de continuer un peu de ce qu’il m'a dit quoi. Donc pour les repas, j'ai le droit à 150 grammes de féculents, 150 grammes de légumes et 120 grammes de viande, viande ou jambon ou deux oeufs et des yaourts à 0%.

**D’accord. Du coup, vous mesurez toujours ?**

Ouais, ouais ouais. Bon, quand il me reste 10 grammes, je fais un peu moins attention, donc j'avais fait... Au départ, j'étais monté jusqu'à 87, là je fais 76. Donc c'est beaucoup mieux quand même. Donc je fais un peu moins attention, mais je fais attention quand même sur mes repas, je… tâche de… après quand il reste 10 grammes, au lieu de 150, c'est 160, je vais pas laisser 10 grammes, on va pas chipoter quand même *(rires). 42’50* Ouais j’ai ma bascule, ouais je…

**Et vous pesez**

Généralement ouais, presque tous les jours quoi.

**D’accord. Comment vous faites ? Parce que du coup, il faut diviser les repas en… ?**

Après, je m'organise. Par exemple en parlant de ce midi, donc j'ai des légumes, c’est des brocolis. Donc faut que je mange en entrée… Je vais manger du taboulé comme ça chez mes féculents et mes mes légumes. Voilà. Après, je mange un yaourt à la fin. Je mange plus de pain. Normalement j’ai pas le droit au pain ou alors c'est 80 grammes et ça retire un féculent donc voilà… Il y a que le matin que je mange du pain, j'ai le droit à 80 grammes de pain le matin, plus de 10 grammes de beurre, quand c'est du beurre, normal. Voilà c’est…

**Et ça vous convient, sur votre poids…?**

Des fois, c'est un peu dur, mais bon, je compense avec un peu de chocolat, de temps en temps quand j’ai la… il m'a dit le chocolat noir sans truc dedans, on peut en prendre pratiquement… pas trop, mais ça ne fait pas grossir. Je prends du 70% donc voilà. Des fois, c'est un peu plus dur, mais bon, après l'estomac, se réadapte et ça va.

**Et c’est vrai qu’en terme d’organisation ça demande un peu… mais j’imagine**

Ouais mais après c'est pas excessif comme organisation, j’ai le temps je suis en retraite donc euh…

**La notion du temps... Vous pensez que si on vous travaillerez, ça serait plus compliqué ?**

Oui, parce que après, quand je travaillais, j'allais souvent à la cantine, au restaurant d'entreprise. Donc après, c'est difficile de… voilà

**De demander combien ça pèse *(rires)***

*(rires)* Voilà. Des fois, quand je vais, quand je vais acheter de la viande, je leur dis les tranches de viande à peu près 120 grammes, quoi. Donc ils me font.

**Vous allez chez le boucher ?**

Ouais

**Et vous demandez 120 grammes ?**

120 grammes ouais.

**Et du coup, vous mangez pas forcément de la viande le midi et le soir ?**

Non, généralement, le midi, c'est viande et le soir, jambon, oeuf ou poisson. Même le midi, ça arrive d'être poisson, quoi. Je, je change jambon ou tranche de poulet, bon c’est de la viande le poulet…

***(rires)* Oui, vous avez dit le midi viande et le soir jambon poulet. Pour vous c’edt pas vraiment la même chose ?**

Oui, oui, c'est la même chose

**Mais c’est pas un gros plat de viande ou quoi ?**

Non, non, non plus. C'est plus léger.

**Oui, entre l'Entrecôte et une tranche de jambon, c’est pas la même, c'est différent.**

Ouais, ouais.

**D’accord. Pour terminer, j'aimerais qu'on discute du gaspillage alimentaire. Quand je vous dis gaspillage alimentaire, quels sont les mots qui vous viennent à l'esprit ?**

C'est difficile parce que je gaspille pratiquement rien, donc j'essaye de gérer au mieux…

**Donc la vous dites « gérer », donc il y a quelque chose… pour vous le gaspillage alimentaire a peut être un lien avec la gestion ?**

Oui, il faut.. il faut gérer ses fruits et légumes… Après en prenant du surgelé au niveau des légumes, là, il y a moins de gestion à faire. On peut en sortir. Ça me permet d'en sortir quand je veux quoi, le matin, une fois que j'ai déjeuné, je sors ce que j'ai besoin pour le midi. Voilà.

**Ok. Et si on essaye de définir ce qu'est le gaspillage alimentaire ? Si vous, vous essayez de faire une définition comme dans le Petit Robert, le dictionnaire, qu'est ce que vous… Comment vous le définiriez ?**

C’est un manque d'organisation quoi.

**Ouais ? Pour vous le gaspillage alimentaire, c’est un manque d’organisation. Ok. Quand vous réalisez que vous êtes en train de jeter un fruit et légume… comment ? Comment vous réagissez ?**

C'est pour ça que… moi, ça m'embête de… sachant qu’il y a des personnes qui meurent de faim dans le monde. Ça me dérange de jeter des légumes. C'est pour ça que j'ai… depuis un moment. J'en ai… Ça fait longtemps que je n'en ai pas jeté, donc j'évite le plus possible d’en jeter quoi. Oui, il y a assez de monde comme ça qui crèvent de faim dans le monde. On peut s'organiser pour ne rien perdre.

**D’accord. Donc vous n’aimez pas jeter, notamment parce qu'il y a des gens qui n'ont pas la chance d’avoir à manger.**

Et en plus acheter, ça coûte de l'argent en plus. Si on achète des trucs, qu'on le jette…

**C'est se dire d’un côté, il y a des gens qui ne mangent pas et en plus, c'est aussi jeter de l'argent…**

Oui, oui oui.

**D’accord, alors pour terminer, juste qu’on discute un peu de la poubelle connectée que vous avez depuis quelques jours. Comment ça se passe ? Vous avez le sentiment de l’utiliser beaucoup ou très peu ?**

*(rires)* Non, pas du tout. Franchement, non, j’ai rien jeté. Et comme je vous dis… j'aime pas jeter, donc j'ai rien à jeter à la poubelle. Ma poubelle, j'ai un petit sac, c'est tout, c’est les épluchures de légumes que je mets dedans, c’est pratiquement…. Il y a que ça dedans. Donc…

**Donc là, vous n’avez rien jeté depuis, vous n’avez rien perdu ?**

Non, non. Même quand je suis parti une semaine en vacances. J'avais avec moi ce qui me restait, donc comme c'était pas trop loin..

**D’accord, ce qui vous a permis de ne pas rentrer…**

Ouais ouais… Et après j'avais vu avec ma marchande de légumes pour préparer les légumes le samedi matin et qu’elle les dépose quelque part. Je m'étais organisé une… C’est une organisation, après j'ai le temps. Après, c'est sûr que d'autres personnes qui travaillent quand il y a la mère et le père qui y travaillent, c'est peut être moins facile de s'organiser comme ça.

**Ouais… Ok. Bon, bah du coup.**

Des légumes que je consomme aussi, j'ai oublié de parler des endives aussi. J'aime bien endives, salades.

**Et la salade. Comment vous la conservez, la salade ?**

J'en prends plus beaucoup. Généralement, j'ai des boites, des boites pour… des boites pour gérer les fruits normalement… je vous ferais voir ça tout à l'heure et donc je l’épluche et je le mets dans une boîte voilà… et ça dure deux ou trois jours. Mais les salades, j'achète de moins en moins. J'aime bien les endives, donc j'achète les endives plus plutôt.

**D’accord, et pourquoi, du coup ? C’est une préférence de goûts ?**

Préférences de goûts.

**Plutôt endives.**

Ouais plutôt endives.

**Bon, du coup, vous n'avez pas d'exemple de ce que vous avez jeté, puisque vous n’avez rien jeté *(rires)***

Non.

**Bon admettons, là ça vous est pas arrivé, mais quand vous jetez, est ce que vous savez… enfin est-ce que vous avez en tête, un fruit ou un légume que vous allez plus souvent perdre qu’un autre. Un fruit et légume qui…**

Ben’ comme je vous avez dit. J'avais perdu un morceau, c'était presqu'une courgette, une mandarine. Et après le reste, je m’en rappelle plus trop, non.

**Et du coup, en termes de raisons, la courgette, elle était**

Ouais elle était pourrie assez vite, donc je ne l'avais pas mise au frigidaire. Maintenant, je les mets au frigidaire quand j'en achète, donc, mais quand j'en achète, souvent, je les cuisine, les courgettes quand je les achète le vendredi… Je les cuisine le samedi, donc c'est assez vite. Pour le reste, je sais que les mandarines, je fais gaffe maintenant.

**Est ce que vous avez eu un moment donné, des questions, pour savoir si par exemple, sur les épluchures est-ce que ça doit aller dans la poubelle connectée ou pas ? Ou non, vous n'avez pas eu de questions.**

Non, vous me l'aviez dit le premier, la première fois que ça n’allez pas dedans.

**Du coup, j'imagine que vous n'avez pas changé de sac poubelle…**

Non j’ai rien fait elle est restée là, comme était au tout débat.

**Ok, ça marche. J’ai fini de poser toutes mes questions. Merci beaucoup d’avoir pris le temps d’y répondre. Est-ce qu’il y a des choses sur lesquelles vous voulez revenir ? Est ce que vous pensez que j'ai pu oublier quelque chose autour de votre consommation ?**

Non… On a fait le tour à peu près des consommations. J'essaye de consommer à peu près tous les fruits et légumes possible, pour faire changer un peu…

**Ouais vous essayez de varier quoi ?**

Ouais varier ouais.

**Et vous faites aussi en fonction de la saison.**

Ouais ouais. Après les légumes, il y en a en surgelés maintenant… c'est plus pour les fruits, pour la saison.

**Oui, ouais. Très bien. Merci beaucoup.**

**Household F27, Interview 2**

*Speaker 1:* [hop] Fine deux alors? Normalement le protocole. [hop] [rire]

*Speaker 2:* [Rire] on est repartis [Ouais] je, j'ai fait la troisième dose déjà [donc] --.

*Speaker 1:* Oui je me souvenais que vous étiez vacciné assez tôt déjà.

*Speaker 2:* [Donc euh] -- comme si je ne faisais pas la, la troisième dose. Le pass sanitaire était plus disponible à partir du 15 [euh]. Donc je l'ai fait assez vite.

*Speaker 1:* Je vais devoir aussi -- j'ai deux mois de latence, il faut que j'aille --.

*Speaker 2:* [Ah oui, oui ,oui] c'est -- après [euh] chez ma belle sœur hier à Paris. C'est -- c'est compliqué ils disaient pour avoir [euh] un rendez vous, c'est la galère.

*Speaker 1:* Ouais moi je regardais déjà là mais -- Moi c'est à partir de mi décembre que je dois le faire et -- il n'y a pas de rendez vous pour l'instant.

*Speaker 2:* [Ouais, ouais] Moi, j'étais avec la pharmacie à côté là et -- ça a été assez vite [hein], en une semaine j'avais mon rendez vous. --

*Speaker 1:* C'est pas plus mal. [rire]

*Speaker 2:* [Ouais, ouais, ouais] [rire]

*Speaker 1:* [Hop] alors -- que je me trompe pas. [Ouais] on a plein de matériel.

*Speaker 2:* Plein de photos.

*Speaker 1:* [Alors] là je vous en mets plusieurs

*Speaker 2:* Oui.

*Speaker 1:* L'idée, c'est que vous en choisissez une ou vous la retournez. Quand vous la retournez, vous me dites spontanément.

*Speaker 2:* Si j'ai gagné ou si j'ai pas gagné le lot. [c'est ça ?] [rire]

*Speaker 1:* Les mots qui vous viennent [euh] -- en tête. De nouvelles inscriptions, qu'est-ce que ça vous évoque [quoi]. --

*Speaker 2:* Là ça m'é, m'évoque un jardin ; potager.

*Speaker 1:* [Ouais]

*Speaker 2:* partagé aussi, je pense.

*Speaker 1:* [Ouais] -- qu'est-ce qui vous fait penser ça ?

*Speaker 2:* [hum] -- Il y a des -- C'est encerclé, là, je pense [euh]. C'est le premier truc qui m'est venu à l'esprit parce que -- à priori [euh], a priori, on voit pas, on voit pas une maison à côté., donc je pense que c'est peut être des [euh], des [euh] des jardins par, [euh] partagés, ça, ça se fait de plus en plus. Donc. --

*Speaker 1:* Oui, c'est en ville.

*Speaker 2:* [Ouais] et ben bon -- Généralement, les jardins dans les -- [euh] dans les maisons ne sont pas comme ça. C'est des allées ou des trucs comme ça. C'est pas encadré comme ça. [euh] --

*Speaker 1:* ok. -- Et est-ce que vous avez déjà vécu une ou plusieurs expériences avec ce type de lieux ? avec des --

*Speaker 2:* Non.

*Speaker 1:* Vous n'avez pas de potager?

*Speaker 2:* Non, pas de potager. --

*Speaker 1:* Ok. Et globalement, vous pensez quoi? Des produits qui peuvent être issus de -- ce type de lieu.

*Speaker 2:* [euh, pff] Généralement c'est des bons produits. Après, ça dépend [hein] s'ils mettent des [euh] -- des insecticides ou [je sais pas] des, des produits, donc [euh] s'ils font ça écologiquement ou quoi. Mais généralement, c'est des produits [euh] plus sains que ce qu'on trouve en surface ou [euh] en grande surface. Ce matin, lundi matin. -- Pas trop demander [hein]. [rire]

*Speaker 1:* [rire] ok.

*Speaker 2:* C'est pour ça que moi, j'achète [euh] -- pratiquement rien en grande surface. Je fais tous sur le marché -- [euh] des produits locaux.

*Speaker 1:* [donc] vous n'avez pas forcément de [euh] . Goûter des produits comme ceux issus d'un potager, que se soit le vôtre ou d'un voisin ou d'un --

*Speaker 2:* [Euh] Si j'ai eu des poireaux qu'un collègue m'a donné, [euh] -- Bon, après la différence on -- on la sent pas tout de suite [euh]. C'est sûr qu'il y a un peu plus de goût sur les poireaux ou -- je sais plus ce qui m'avais donné aussi --. J'ai vu des châtaignes dernièrement donc [euh] --

*Speaker 1:* [Ouais] donc une autre photo -- celle que vous voulez. -- Qu'est-ce que ça vous évoque ?

*Speaker 2:* [Oh] ca c'est une grande surface.

*Speaker 1:* [Ouais]

*Speaker 2:* Un produit en grande surface. Donc là [euh] tout le monde touche les produits, les légumes, donc [euh] -- c'est pour ça que j'en achètent de moins en moins parce que [euh] -- en plus maintenant avec le, avec le covid, on ne sait pas ce qui se passe. -- Donc ouais, ouais, tout le monde touchent. [ouais] Donc moi j'achète [euh] sur le marché maintenant.

*Speaker 1:* [Ouais] -- Principalement pour ça -- par le, le fait que --

*Speaker 2:* Les produits sont [euh] -- déjà il y a, Il y a moins de -- d'intermédiaires, donc [euh] à priori ça revient plus [euh] à l'agriculteur [euh] le prix de la vente [euh] revient plus à l'agriculteur qu'aux -- parce que il prend une marge, ils prennent une marge donc [euh]. Et bon, [euh] à priori c'est plus sain quand même. Les produits. --

*Speaker 1:* ils sont plus sains sur le marché ?

*Speaker 2:* Oui. -- [ben] généralement, ce sont des produits de saison qui ne restent pas en, en -- en frigo [euh] un certain temps parce que là, en -- en grande surface, je pense qu'il y en a une partie qui reste en frigo donc [euh] ou en congélation donc. [Euh] -- [ouais]

*Speaker 1:* [Ouais] -- Et du coup, ça vous arrive des fois de -- d'aller au supermarché, même si ce n'est pas pour acheter vos. --

*Speaker 2:* Ah oui, [bah] je vais une fois par semaine en supermarché. Mais [euh] j'achète [euh] je n'achète pas de, [euh] je dis j'achète pas, ça m'arrive -- une fois de temps en temps quand il me manque vraiment quelque chose. Mais [euh] -- j'achète pas de fruits [euh] ni de légumes [euh] Les légumes souvent c'est en -- en surgélation donc [euh] je les achète soit en supermarché, soit [euh] avec un -- fournisseur de -- à domicile.

*Speaker 1:* [Ouais] vous commender des --

*Speaker 2:* [Ouais] des --

*Speaker 1:* Du surgelé -- Et est-ce que vous sauriez dire quand vous êtes dans des [euh] -- grandes surfaces ? Comment [euh] vous sentez? Est-ce que c'est un moment [euh] -- que vous qualifieriez d'agréable d'aller faire vos courses ou non? Vous y allez par nécessité. Comment ? --

*Speaker 2:* Moi, j'aime bien faire les courses.

*Speaker 1:* [Ouais ?]

*Speaker 2:* [Ouais]

*Speaker 1:* En Grande surface ? [euh] --

*Speaker 2:* [Ouais] [euh] -- Là, j'y vais, il y à pas trop de monde. Je m'arrange le [euh] -- le matin,le mercredi matin il y a pas [euh] -- Je vais généralement à 8 h et demie, ça ouvre à 8 h et demie. Il n'y a pas grand monde que je suis de -- donc j'ai le temps de -- de regarder, [euh] -- de choisir donc [euh] -- Mais [ouais].

*Speaker 1:* [Ouais]

*Speaker 2:* ça m'a jamais déplu de faire les courses. Je sais que certaines personnes ont du mal à faire, -- mais moi, ça me dérange pas. --

*Speaker 1:* [ok].

*Speaker 2:* Même quand je fais mes courses, je fais tous les rayons pour voir s'il me manque pas quelque chose ou --

*Speaker 1:* [Ouais] -- vous prenez le temps de --

*Speaker 2:* [Oauis, ouais, ouais] je ne suis pas -- mainteant que j'ai -- avant je faisais moin ça mais maintenant j'ai le temps [euh] --

*Speaker 1:* [Ouais] --

*Speaker 2:* Après [euh] -- on rencontre des collègues [euh] -- on discute [euh].

*Speaker 1:* Ouais ? ça vous arrive de -- de rencontrer du monde ? [euh]

*Speaker 2:* [Ouais, ouais, ouais]

*Speaker 1:* En grande surface ?

*Speaker 2:* [Ouais, ouais]

*Speaker 1:* Ok et donc alors maintenant vous achetez vos fruits et légumes [euh] principalement sur le marché. [euh] Mais donc vous avez déjà consommé des fruits et légumes [euh] issus du [euh] --

*Speaker 2:* Même temps depuis un moment? Non, je ne me rappelle plus. [euh] -- J'ai dû acheter des citrons dernièrement, c'est tout. Des citrons bio, c'est tout.

*Speaker 1:* [Oui]

*Speaker 2:* Oui, un sachet et il m'en manquait donc [euh] -- et comme il n'y a pas le marché tous les jours donc bon [euh] -- sans ça le reste non [euh]. Je j'achète pratiquement pas, [euh] je fais, je fais ma liste de -- j'y vais le vendredi matin, je fais ma liste le jeudi [euh] soir pour voir ce qui manque [euh] Donc je prends des tomates, là Il y a de l'avocat, des endives, des pommes, des oranges. Pour la semaine [quoi] je m'arrange et --

*Speaker 1:* [Ouais] Vous prenez tout [euh] mis à part qui vous manque quelque chose [euh].

*Speaker 2:* [Ouais] c'est rare --

*Speaker 1:* Et vous pensez quoi des produits des fruits et légumes qui peuvent être vendus sur le [euh] -- en grande surface? Vous trouvez de qualité ou --

*Speaker 2:* De moins bonne qualité que sur le marché.

*Speaker 1:* D'accord, c'est quelque chose que vous avez remarqué ?

*Speaker 2:* Oui.

*Speaker 1:* Et dans "moins bonne qualité". Vous entendez quoi ? C'est plutôt en terme de saveurs, en terme de -- de conservation, en terme de --

*Speaker 2:* Conservation [euh] déjà, ça m'est arrivé il y a quelques années de perdre des -- des fruits. Ça m'arrive aussi avec le marché. Mais bon [euh] -- ça, il n'y a pas de problème. Comme je la connais, elle me le remplace, je lui ai dit Tiens, ça m'est arrivé il y a trois ou quatre mois [euh] -- j'avais perdu un truc donc [euh] --

*Speaker 1:* Oui ça me parle. Ce n'est pas une courgette [ou] --

*Speaker 2:* Ouais une courgette -- il y a -- [ouais] Il y a un moment quand même. [Bah] depuis je -- je [euh] perd pratiquement rien. -- Je m'arrange pour regarder les tomates, comment ils sont -- si ça peut attendre, et il y a d'autres fruits [au] -- au frigidaire [euh]. Bon, après les -- les fruits là, ça -- ça part assez vite. J'en prends deux par jour. Donc [euh] --

*Speaker 1:* Oui.

*Speaker 2:* Ça ne reste pas à traîner normalement.

*Speaker 1:* Ouais ça, finalement, vos fruits et légumes sont pas longtemps stockés.

*Speaker 2:* [Non, non]. Après les légumes, c'est souvent en surgelés. Quoi -- Que ce soit -- à part les petits pois que je prends en conserve, ce que je trouve meilleur, en conserve que -- sans ça brocolis, chou fleur, haricots verts, [euh] Ah ! sauf les pommes de terre [euh] -- que j'achète. Mais le reste c'est en -- en surgelés quoi.

*Speaker 1:* Donc ça jamais vous en achetez finalement sur le marché.

*Speaker 2:* Non, cela non, c'est plus, c'est plus pratique [euh] Par exemple, le chou fleur [euh], quand on l'achète sur le marché, faut le cuire [je ne sais pas combien de temps]. Donc on l'achète en surgelés [euh] Il y a trois minutes à le réchauffer au micro-ondes [euh] -- J'essaye de prendre des produits précuits, donc comme ça, il n'y a plus qu'à les -- les réchauffer au micro-ondes trois minutes. Et c'est bon. [quoi]

*Speaker 1:* [Ouais] un côté plus pratique [euh] pour vous [quoi]

*Speaker 2:* [Ouais] c'est plus pratique [euh] --

*Speaker 1:* Et [euh] -- même en gestion de quantité quand on en avait parlé la dernière fois de -- vous prenez que ce d'on --

*Speaker 2:* [Ouais]

*Speaker 1:* Quand viens le chou-fleur aussi

*Speaker 2:* [ouais, ouais] [rire]

*Speaker 1:* Du coup, vous disiez que [euh] -- ça vous ai déjà arrivé de perdre des fruits et légumes [euh] qui étaient [euh] --

*Speaker 2:* [Euh ouais] bah là y a. -- Ça, ça fait un moment parce que ça fait deux ou trois ans que j'achète plus de [euh] produits [euh] chez Leclerc ou ailleurs [quoi] donc [euh] ça m'est -- ça m'est arrivé.

*Speaker 1:* [Ouais] Donc en terme de conservation, sinon en termes de saveurs, vous les trouvez tout aussi bons qu'ailleurs ?

*Speaker 2:* [Oh] bah non, je trouve les produits [euh] du marché [euh] meilleurs quand même.

*Speaker 1:* [Ouais]

*Speaker 2:* Après il y a pas de grosse, grosse différence --

*Speaker 1:* Ça va plus être aussi ce que vous disiez sur le fait que les gens [euh] les manipulent.

*Speaker 2:* Maintenant, avec le Covid, [là] [euh] tout le monde manipule [euh] tout ça. Je ne sais pas comment --

*Speaker 1:* [Ouais] [rire]

*Speaker 2:* Après, faut bien les laver, je pense.

*Speaker 1:* [Oui]. Ça marche. [Euh] Quand vous perdez par exemple admettons là le citron que vous avez acheté quand, -- admettons que vous le perdez, il se gâte et vous devez le jeter. Qu'est-ce que vous ressentez quand vous jetez des produits qui peuvent venir du supermarché ?

*Speaker 2:* [Bah] ça me, bon pour un citron c'est pas [euh] --ça m'énerve un peu, mais bon c'est pas trop trop grave quoi. Mais [euh] -- j'aime pas perdre la nourriture quoi --donc [euh] c'est pour ça que je fais attention de consommer des produits avant qu'ils soient périmés. Ou -- ou pourrie

*Speaker 1:* Ouais, ok. Alors une autre [euh] photo. Qu'est-ce que ça vous évoque ?

*Speaker 2:* Oh, ça, c'est -- ca c'est un marché couvert, je pense

*Speaker 1:* Oui.

*Speaker 2:* Style, [heu] style grand frais quoi. Je connais ça que je ne sais pas si vous connaissez le grand frais.

*Speaker 1:* Non.

*Speaker 2:* C'est un [heu] c'est -- c'est un -- un magasin de -- de légumes et de -- de fruits qu'on trouve souvent dans la région parisienne.

*Speaker 1:* OK

*Speaker 2:* donc c'est un peu pareil. Les gens touchent, [euh] touche un peu à tout [quoi]. Mais. C'est un peu plus saint que -- parce que c'est. -- Certains produits sont sous brumisateur. Ils mettent un -- un peu de -- de pluie dessus [euh] donc [euh] donc c'est --

*Speaker 1:* Vous vous achetez pas dans ce genre de --

*Speaker 2:* Ah non, non il y en a pas dans le coin. Il y a -- s'il y a un sur la route de Brest, les primeurs de Landerneau -- mais je n'ai jamais été. Donc non, au choix il y a le marché à côté, je suis à côté.

*Speaker 1:* C'est sûr.

*Speaker 2:* Ouais donc [euh] --

*Speaker 1:* Et sinon, c'est un lieu dans lequel vous pourrez -- vous pourriez, si jamais vous n'aviez pas le marché. C'est un lieu dans lequel vous pourriez aller [euh] acheter --

*Speaker 2:* [euh] Ouais s'il n'y avait pas le marché. Je sais que quand je vais à Paris chez ma -- chez ma belle sœur elle fait tous ses produits frais [euh] dans un magasin comme ça [quoi] en grand frais et donc ils font les légumes, ils font la viande aussi [euh] sur Paris [euh] je ne sais pas s'il y en a dans le coin grand frais, mais c'est pas sûr. Donc --

*Speaker 1:* [Ouais]

*Speaker 2:* [Ouais] -- Donc [euh] voilà bah elle, elle fait ses courses là bas parce que [euh] ou elle habite, il n'y a pas de marché. Donc [euh] non.

*Speaker 1:* C'est vrais qu'il y en a plus [euh]. -- Ce genre de magasin, il y en a plus dans les grandes villes.

*Speaker 2:* Oui.

*Speaker 1:* [Et puis] [enfin] en campagne qu'en campagne [quoi].

*Speaker 2:* [Oui, oui, oui] Nous on a de la chance d'avoir [euh] --

*Speaker 1:* [C'est ça]

*Speaker 2:* [rire]

*Speaker 1:* Ok alors, La dernière photo qui va un peu plus vous parler, je pense du coup.

*Speaker 2:* Ah ça c'est le marché.

*Speaker 1:* [Rire]

*Speaker 2:* Tout le monde en a parlé. [euh] Moi, je fais pratiquement toutes mes, mes courses légumes. Et -- sauf les surgelés, quoi. Carottes, oignons, [ruh] échalotes, ail. percil, radis [euh] tout ça, j'achète. Concombre, courgette -- au marché après pomme orange aussi donc [euh] -- après j'essaie d'acheter des produits des saisons, [quoi] c'est pas toujours [euh] --

*Speaker 1:* Pourquoi vous essayez de prendre des produits de saison ?

*Speaker 2:* [Euh] les produits de saison viennent de [euh] à côté [quoi] Sans ça, ça vient du Maroc ou de, d'Israël, ou -- je dis ça mais les oranges, c'est pas de l'hiver. [rire] Si on veut manger des oranges, c'est pas ici qu'on en fait [hein]

*Speaker 1:* Non, c'est sûr.

*Speaker 2:* Ouais donc [euh] --

*Speaker 1:* Puis un avocat c'est pas --, on en a pas encore.

*Speaker 2:* Non, non, les avocats non plus.

*Speaker 1:* Il parait qu'ils sont en train d'en faire en Bretagne.

*Speaker 2:* [Ah ouais] donc [euh] ouais

*Speaker 1:* D'accord. Donc c'est parce que du coup les -- les pour vous, les produits de saison sont locaux. Du coup, c'est pour ça que vous prenez --. D'accord. Globalement, quand vous allez au marché, est-ce que [euh] comment vous vous sentez ? C'est un moment agréable ou c'est une nécessité de vous rendre là bas. -- Qu'est-ce que ça vous fait d'aller au marché ?

*Speaker 2:* Moi, j'aime bien. -- C'est une nécessité parce qu'il faut les acheter déjà. Donc [euh] -- moi, c'est un peu différent je vais à -- je suis le premier tous les matins, [euh] tous les vendredis parce que je suis assez tôt. Donc c'est à 7 h 15, j'arrive à peu près. Donc --

*Speaker 1:* Ah oui !

*Speaker 2:* je suis réveillé de bonne heure le matin, donc à 7 h 15, je suis servi tout de suite, on reste discuter. J'aime bien, je reste discuter avec les -- la patronne et son ouvriere donc [euh] voilà.

*Speaker 1:* Donc vous allez tout le temps avec la même [euh].

*Speaker 2:* Ouais avec la mème -- quand elle est là, ouais, bah j'ai mes mes habitudes après [euh] [ouais] je trouve [euh] ses produits sont bons. Quand il y a un problème. S'il y a un problème, je lui dis et --

*Speaker 1:* Oui, ça oui. C'est ce que vous racontiez tout à l'heure. Ça vous est déjà arrivé que--

*Speaker 2:* [Ouais, ouais] ouais ça fait un petit moment. Depuis, j'ai -- j'ai rien eu donc [euh] Non, non.

*Speaker 1:* Et quand c'est comme ça, elle vous redonne [euh] tout de suite ?

*Speaker 2:* Ouais, elle m'a, ouais elle m'a re donné, elle m'avais re donné ou elle me l'avait [euh] sois qu'elle me lavait déduit parce que j'en avais pas besoin ce, cette semaine là me l'avait déduit de ma commande.

*Speaker 1:* D'accord.

*Speaker 2:* Ouais.

*Speaker 1:* Et quand vous discutez avec vous, discutez de la pluie et du beau temps ou vous discutez des produits ou c'est --

*Speaker 2:* Bah des [euh] un peu de tout, de -- de l'actualité, du temps, des produits aussi. [euh] défois Je lui demande s'il faut mieux les garder au frigidaire queà l'air libre sur certaines, sur certains fruits, surtout les bios. Vaut mieux les laisser au frigidaire parce que il y a moins de. -- Il y a moins de produits, donc ils périssent un peu plus vite donc [euh].

*Speaker 1:* Oui. Et du coup, la productrice chez laquelle vous allez, elle produit du bio ? ou non c'est ...

*Speaker 2:* [Oui, oui oui]

*Speaker 1:* Ok

*Speaker 1:* [Bon] il n'y a pas qu'elle, il y a un marchant de fraises aussi à côté, [euh] en face quand c'est la saison de -- de fraises, j'achète aussi avec lui. Je sais que c'est lui qui les fait. Puisqu'Il est de Plougastel, donc [euh] [voilà]

*Speaker 1:* Donc ouais vous achetez directement auprès du producteur.

*Speaker 2:* [Ouais, ouais, ouais]

*Speaker 1:* Ok [euh] et donc globalement, vous pensez quoi des produits que vous trouvez [euh] sur le marché ?

*Speaker 2:* Moi je, je les trouvent très bien. Il n'y a pas de problème.

*Speaker 1:* [Ouais] vous avez --

*Speaker 2:* Moi j'ai, même, j'essaye. Il y a différentes sortes de pommes, j'avais [euh] il y en a une que je prends régulièrement ou je sais plus laquelle que s'est et j'avais essayer une autre qui m'avait pas -- j'avais moins aimé donc je suis revenue [donc]

*Speaker 1:* [Ouais] vous trouverez quand même de la variété sur --

*Speaker 2:* [Oui, oui, oui] bah les pommes -- ouais il y a pas mal de variétés quand même. Mais c'est un marché de Brest ça ? Non ?

*Speaker 1:* [Euh] Je ne sais pas du tout

*Speaker 2:* [Rire] [Ouais] ok

*Speaker 1:* Et [euh] si jamais ça vous arrive, même si vous jetez très peu de -- de perdre un produit du marché, qu'est-ce que [euh] -- qu'est-ce que vous ressentez ?

*Speaker 2:* [Bah] là aussi c'est pareil. Comme j'aime pas, c'est. [euh] -- je suis un peu déçu, mais bon, après -- j'évite [hein] de -- de les perdre donc c'est -- c'est très rare, donc [euh] je ne sais pas, -- dans l'année je dois en perdre [euh] un ou deux, pas beaucoup plus donc [euh] --

*Speaker 1:* Oui donc éffectivement c'est pas très marquant

*Speaker 2:* C'est très, très, très, rare donc [euh] je prends ce qu'il me faut et bon -- j'entasse pas

*Speaker 1:* Oui, puis la dernière fois finalement, même quand vous l'aviez perdu, le fait que vous -- vous connaissiez la productrice [euh]

*Speaker 2:* Ouais, ouais, mais même si c'est remplacé, c'est -- c'est un peu gênant de perdre quelque chose quoi, sachant qu'il y a plein de personnes qui crèvent la dalle et que -- nous on fonce. Il y a plein de gens qui paient, foutent plein de -- bouffe à la poubelle, donc c'est --

*Speaker 1:* Ouais, c'est ça. C'est le fait que -- tout le monde ne mange pas à sa faim qui vous --

*Speaker 2:* Oui

*Speaker 1:* Qui vous énerve le plus quand vous jetez vous?

*Speaker 2:* [Ah oui, oui]

*Speaker 1:* Ok, -- ça marche -- [alors] merci on a finis le premier petit jeu [rire]

*Speaker 2:* [Ah] Il y en a plusieurs ? [bon] qu'est-ce que j'ai gagné alors ? [Rire]

*Speaker 1:* [Alors] -- mes bananes, elles sont toutes là ?

*Speaker 2:* [hum] des bananes

*Speaker 1:* Des bananes, vous mangez de la banane ?

*Speaker 2:* Ça m'arrive.

*Speaker 1:* Ca vous arrive. Alors il y a dix [nomalement] images de bananes, je vais vous demander de les ranger, un peu comme vous pouvez, comme ça, celle qui vous donne le plus envie àcelle qui vous donne le moins envie.

*Speaker 2:* Le moins envie [bon]

*Speaker 1:* Je prend les -- les bananes

*Speaker 2:* [Bah] le plus envie c'était la première, je crois c'est -- Il n'y a pas de grosse différence [hein]

*Speaker 1:* C'est subtile [rire]

*Speaker 2:* [Ouais] -- ça c'est par là -- celle la c'est entre, entre les deux, c'est presque la même photo [hein].

*Speaker 1:* Oui

*Speaker 2:* [Euh] j'aime pas déjà les bananes comme ça -- un peu -- après celle la elle est un peu trop verte je pense. -- [après] celle la elle est un peu taché mais c'est gras.

*Speaker 1:* [rire]

*Speaker 2:* Après [euh] dans ceux là -- il y a pas grosse différence [hein].

*Speaker 1:* [Oui]

*Speaker 2:* [euh] elle a l'air d'être un peu plus mure que [euh] -- [Ouais] allé, on va pas chipotter.

*Speaker 1:* Ça, c'est vos préférées, et après, ce serait comme ça la ?

*Speaker 2:* Ouais, ouais [euh]

*Speaker 1:* Je vais prendre en photo vôtre -- vôtre rangement.

*Speaker 2:* [Ouais] Celle là, on peut même la mettre dans les préférées, [bah] je sais pas [euh].

*Speaker 1:* Quand elle est verte, ça va, vous l'aimez quand même ?

*Speaker 2:* Ouais, ouais. Ouais, ouais. [Bah] ce que j'aime pas, c'est quand elle ait un peu comme ça, un peu blettes [euh] -- Ça, [euh] j'aime pas.

*Speaker 1:* Pour vous en fait, quand ça commence à être un peu trop tacheté comme ça, c'est --

*Speaker 2:* Un peu tacheté ça va.

*Speaker 1:* [Hop] [tac] parfait, pour chaque banane, je vais vous demander de vous projeter comme si elles étaient devant vous et de me la décrire du coup, à l'aide de vos --cinq sens.

*Speaker 2:* [rire]

*Speaker 1:* Qu'est-ce que voilà? Qu'est-ce qu'elle vous? Qu'est-ce qu'elle vous évoque, cette banane? par exemple donc là, on a, j'ai mis ça comme repère les petites formes.

*Speaker 2:* Ouais

*Speaker 1:* Donc là, vous, votre préférée, c'est la carré blanc.

*Speaker 2:* Oui.

*Speaker 1:* Selon vous, c'est comment elle est cette banane ?

*Speaker 2:* [bah] elle est, je pense que elle devrait être [euh] -- elle doit être mure, mais [euh] -- pas, pas trop donc impeccable je trouve. Après bon, ça c'est qu'une photo, donc [euh] après faut voir, non...

*Speaker 1:* Elle vous semble parfaite comme ça ?

*Speaker 2:* Ouais, ouais, ouais. -- Après celle là, c'est pareil j'aurais pu la mettre làaussi c'est à peu près le même style celle là et un peu taché donc elle doit pas être doit pas être taché à l'intérieur, donc [euh] elle doit être un peu plus mure que celle là, mais encore [euh] bien.

*Speaker 1:* Ok

*Speaker 2:* Celle là ce doit être un peu près pareil que l'autre, donc [euh] bien ça me -- là, celle-là commence à être un peu mure donc [euh]

*Speaker 1:* C'est quoi? C'est la carte orange ? là pour vous elle est encore bonne ?

*Speaker 2:* [Euh] oui encore bonne oui, après -- oui, oui,oui. Bah toute façon, même celle là est bonne, mais bon on ne sait pas -- on peut la manger, mais bon. -- Et celle là, a priori, elle est pas assez, pour moi, pas assez mure [quoi].

*Speaker 1:* Triangle rose elle est pas assez mure ?

*Speaker 2:* Oui.

*Speaker 1:* Donc quand vous avez [euh], oui vous attendez quand --

*Speaker 2:* attendez -- un ou deux jours qu'elle se, qu'elles murisent un peu.

*Speaker 1:* Ouais. Et donc après celle du rond blanc vous.

*Speaker 2:* Non, bon après ça commence à être trop trop mure.

*Speaker 1:* Et qu'est-ce qui fait que là, vous commencez à pas trop aimer en termes de saveurs ou de textures ? qu'est-ce que ? --

*Speaker 2:* Déjà elle est un peu plus molles, elle est un peu [euh] Comment expliquer ? Oui, elle est un peu plus molle [euh] Ouais, c'est la texture molle [euh].

*Speaker 1:* Ouais, vous préférez qu'elles soient un peu plus --

*Speaker 2:* Ouais, un peu plus --

*Speaker 1:* Un peu plus ferme, ouais, je cherchais le mots

*Speaker 1:* Et donc, dans votre organisation, vous vous attendez jamais qu'elle arrive en fait, jusque içi, quand vous voyez qu'elle arrive là, vous --

*Speaker 2:* Ah non, non, non. Déjà c'est rare que j'en achète, j'en achète quand j'en achète dans c'est deux ou trois, pas beaucoup plus. Donc je les mangent quand elles sont comme ça à peu près dans les trois premières. Et après j'évite [euh] -- là après c'est encore faisable, mais bon, je préfère quand ils sont comme ça.

*Speaker 1:* Ouais.

*Speaker 1:* Et donc là, vous avez dit après que c'était encore. Mangeable. [enfin] que vous avez dit --

*Speaker 2:* Celle la doit être, mais après, je sais pas trop [ehin] celle là, ça doit être juste [euh] ça, non je pensse pas.

*Speaker 1:* [rire] c'est pourri

*Speaker 2:* Ah peu être pour des personnes qui aiment bien ça mais [euh]

*Speaker 1:* Mais pour vous à partir de quand elle est pourrie la banane ?

*Speaker 2:* Quand on voit qu'elle est un peu tachée à -- à l'intérieur aussi où on voit des fois [euh] des fois qu'elle est [euh] tachée à l'intérieur.

*Speaker 1:* Oui. -- Des fois, il faut l'ouvrir pour bien se rendre compte que --

*Speaker 2:* [Oui, oui] Mais bon, quand ils sont comme ça, on voit déjà l'extérieur que -- presque pas la peine d'ouvrir [hein].

*Speaker 1:* Ouais, sur le carré bleu, on sait que c'est déjà --

*Speaker 2:* [Rire] Ouais, ouais, ouais

*Speaker 1:* Et selon vous, est-ce qu'il y a un moment donné où le où la banane, elle perd ses apports nutritifs ou pas?

*Speaker 2:* Ou je pense qu'à partir de là elle doit perdre [euh] la même elle doit perdre elle doit ommencer à perdre, je ne sais pas trop, [allors là] je suis pas un spécialiste [hein]

*Speaker 1:* Ouais non c'est selon vous [hein] [rire] moi non plus je ne sais pas. Ok et selon vous est-ce qu'il y a un moment donné où la banane est potentiellement dangereuse pour notre organisme ou pas ? Pareil son sans être un professionnel, juste vous à votre avis est-ce qu'il y a un moment donné ou c'est pas très saint de la manger ?

*Speaker 2:* Ouais, quand elle est comme ça, quand elle commence à être blettes, ça ne doit pas être très sain de la manger [hein].

*Speaker 1:* Ouais. -- Pour vous quand elle commence à être noire [Euh]

*Speaker 2:* Ouais, ouais

*Speaker 1:* Ok

*Speaker 2:* Après si on prend la ferveur gustative de ceux là sont pas terribles, terribles moi je, après il y a certaines personnes qui préfèrent peut être comme ça que comme ça. Donc ça dépend des goûts. Mais moi, je préfère quand ils sont. -- [euh] bien ferme, pas trop mure.

*Speaker 1:* Ouais donc là même le donc c'est le carré bleu là et le rond blanc pour vous. Au gust, même gustativement, toute à l'heure vous parliez de la texture, mais même gustativement [euh] --

*Speaker 2:* Ouais c'est, c'est moins bon.

*Speaker 1:* ok

*Speaker 2:* Ouais

*Speaker 1:* Et vous avez bien conscience qu'il y en a que ça se trouve qui préfèrent comme ça que --.

*Speaker 2:* Ah oui, oui, oui.

*Speaker 1:* C'est des préférences

*Speaker 2:* Après ça dépend des goûts de chacun. Donc [euh]

*Speaker 1:* Ok. Alors.

*Speaker 2:* On a finit sur la banane [rire]

*Speaker 1:* On a finis sur la banane. -- Alors je vais vous montrer d'autres photos.

*Speaker 2:* Il y en a plein là.

*Speaker 1:* Alors là, ce sont des illustrations de choses qu'on ont, que des personnes ont dit pouvoir faire défoiss avec leurs fruits et légumes, que ce soit du stockage ou des lieux, ou on jette ou des préparations. Et je vais vous demander de garder ce que vous [euh] ce que vous [euh] vous pouvez avoir chez vous quoi. parce que vous pouvez faire ou-- et ce qui ne vous parle pas, on mets de côter.

*Speaker 2:* Bon, ça, ça doit être des produits surgelés. J'en ai, [euh] -- ca c'est une préparation, mais je sais pas qu'est-ce qu'il y a dedans, il y a de la banane, je pensse. Là tu as --bon, ça ça me parle un peu [euh], ça j'ai pas, ça à laire d'être du woke ça, je sais pas trop --

*Speaker 1:* Ouais c'est une poilée

*Speaker 2:* Ouais une poilée, je -- je fais de moins en moins de poilée.

*Speaker 1:* Ouais

*Speaker 2:* Ca, ça non plus je sais pas qu'est-ce que s'est, c'est une espèce de compote ?

*Speaker 1:* C'est ça, là c'est la préparation de fruits, donc en compote, -- en smoothie.

*Speaker 2:* Je je je prend de la compote de temps en temps, mais j'achète. J'achète en pot, donc [euh]

*Speaker 1:* Ouais, jamais vous, vous n'avez transformé vos fruits

*Speaker 2:* Non, oh non.

*Speaker 1:* D'accord.

*Speaker 2:* Des fois, ça m'arrive de faire des -- des salades de fruits, donc [euh] j'en fais de t'en, c'est temps-çi j'en ai pas fait depuis un moment, mais [euh] j'en ai eu fait. J'ai des recettes de salade de fruits [euh]

*Speaker 1:* C'est plus ça. Si vous préparez les fruits, ce serait en salades -- mais pas en --

*Speaker 2:* Bons. Ça, c'est une salade. Bon, ça m'arrive d'en faire aussi, salade, carottes. Les soupes [euh] ça aussi. C'est quoi ça ? ça doit être la poubelle ça

*Speaker 1:* Est-ce qu'elle peut ressembler à la vôtre ?

*Speaker 2:* [Euh] Oui, ça peut ressembler, bah [euh] alors on voit pas assé. C'est des épluchures ?

*Speaker 1:* Ouais.

*Speaker 2:* On dirait qu'il y a un pot là, non ?

*Speaker 1:* Oui, en plus c'est une poubelle classique où on met tout dans quoi

*Speaker 2:* Moi je mets plus les pot là, mais bon.

*Speaker 1:* Oui. [Rire]

*Speaker 1:* Mais parce qu'en fait il y a après.

*Speaker 2:* Ouais ben là comme je fais le tri donc [euh]

*Speaker 1:* Mais en fait les autres poubelles, c'est ça, l'idée c'est, est-ce que vous vous [rire] Il y en a qui compostent. Il y en a qui donnent aux poules ou à d'autres animaux. Et sinon, c'est poubelles classiques, quoi, en gros, laquelle ressemble le plus à --

*Speaker 2:* [Bah] la poublle classique parce que je composte pas.

*Speaker 1:* Ouais donc ça -- Pas de poules ? [rire]

*Speaker 2:* non, j'ai pas de poules.

*Speaker 1:* Et ça, c'est si vous faites des bocaux, défois.

*Speaker 2:* [Euh] non.

*Speaker 1:* Qu'ils soient sucrés ou salés.

*Speaker 2:* Non

*Speaker 1:* Non ?

*Speaker 2:* Non.

*Speaker 1:* Ok

*Speaker 2:* Donc, on notera.

*Speaker 1:* Et le, et la poilée, vous, vous c'est pas comme ça que vous préparez vos légumes ? Ça vous arrive pas de ? --

*Speaker 2:* Non. Avant, j'en achetait assez souvent des poêlées en surgelé, mais je fais, ça m'arrive [euh] le dimanche, je fais souvent des patates douces avec de la graisse de canard.

*Speaker 1:* Oui

*Speaker 2:* donc, c'est une poêlée [euh]

*Speaker 1:* Oui

*Speaker 2:* [Ouais] donc on peut dire que ouais.

*Speaker 1:* Oui, ça, c'est juste savoir si vous préparez des fois des légumes cuits [quoi].

*Speaker 2:* [Oui, oui, oui]

*Speaker 1:* Ou à la poêle.

*Speaker 2:* [Ouais] à la poêle les pommes de terre, je fais de temps en temps --

*Speaker 1:* Ouais, ok alors validé. et vous avez tout ça, et en faite je vais vous donner [rire] encore des photos, et je vais vous demander ce que vous vous auriez fait, de ces différentes photos. Vous pouvez me la décrire un peu puis me dire : Alors ça, voilà. Qu'est-ce que ce que vous en faites ?

*Speaker 2:* On à poubelles [hein]

*Speaker 1:* Pourquoi c'est la tomate cerise ? Pour vous c'est direct poubelle.

*Speaker 2:* [Euh] ouais -- on voit pas en dessous, mais elle a l'air d'être bien, bien esquinté, donc [euh] là c'est poubelle.

*Speaker 1:* Ok, alors regardes on peut la mettre -- on peut la renlever en dessous -- comme ça je prend une photo après [rire]

*Speaker 2:* Bon si [euh] c'est bien esquinté --

*Speaker 1:* Poubelle

*Speaker 2:* [Euh] ça il y a peut être quelques petits bouts à récupérer, mais en partie [euh] poubelle.

*Speaker 1:* Oui.

*Speaker 1:* [Euh] ça les andives elles ont l'aire d'être dans un salle état.

*Speaker 1:* Ce sont des photos données par les, par les participants comme vous et on, au moment de jeter donc [euh] et là on essaie de voir si tout le monde jette ou pas ou si d'autres diraient bon celle-là je la cuisine.

*Speaker 2:* Ouais donc [euh], ouais bah là poubelle aussi, il n'y a rien à récupérer, je pense ça là [ouais] poubelle aussi.

*Speaker 1:* [Ouais] les verres de poireaux vous, vous les --

*Speaker 2:* [Euh] j'achète pas beaucoup de poireau

*Speaker 1:* Oui

*Speaker 2:* j'y ai pensé hier ou avant hier d'acheter en surgelé des -- vers de poireaux pour faire [euh] des Saint-Jacques -- à poireau avec des poireaux donc -- mais non, sans ça [euh], quand j'achète des poireaux, les [euh] le vert, je je fais une soupe avec des pommes de terre.

*Speaker 1:* Daccord

*Speaker 2:* Donc [euh] le blanc, je le cuit [euh] avec la soupe, mais je mange en -- en poireaux vinaigrette et le reste le vert je fais -- dans la soupe [quoi].

*Speaker 1:* Ouais

*Speaker 2:* Bon ça, poubelle [euh]. Ça, ça dépend de [euh] comment elle est à l'intérieur, on voit que l'extérieur, mais a priori, ça a l'air , ça a l'air d'être bien taché [quoi].

*Speaker 1:* Vous l'orange comme ça vous la --

*Speaker 2:* Moi je la coupe pour voir si [euh] si il y a une moitié qu'est pas [euh] -- qu'est pas touchée [euh] il y a une moitié que je peux manger.

*Speaker 1:* Oui

*Speaker 2:* Oui, après [euh] à voir à l'intérieur, quoi

*Speaker 1:* OK.

*Speaker 2:* [Euh] ouais, qu'est-ce que.

*Speaker 1:* C'est vrais que [ouais] si vous voulez la manger on peut la laisser là

*Speaker 2:* [Ouais bah] j'ai pas dit que je la mangais, je vais regarder

*Speaker 1:* On regarde, on check [rire]

*Speaker 2:* Et ils mettent à la poubelle ça ?

*Speaker 1:* [Rire] apparemment

*Speaker 2:* Elle a l'air d'être [euh] belle. Pas de -- bon, à voir [euh], -- c'est quoi ça ? Ca m'a l'air d'être des andives mais bon.

*Speaker 1:* C'est une salade ouais

*Speaker 2:* Carottes, [euh] elles ont l'air d'être -- ouais, non, je sais pas trop là, là faut voir comment ils sont [euh] -- des fois, ils sont encore bons, ils sont que -- parce qu'elle a dû les garder un petit moment, donc [euh] en les épluchant peut être [euh] je sais pas [euh]. J'en ai jamais comme ça donc [euh]

*Speaker 1:* Bah oui.

*Speaker 2:* Ouais, [bon] poubelle

*Speaker 1:* Poubelle [rire]

*Speaker 2:* Oh c'est quoi ça ? un poivron ça ?

*Speaker 1:* C'est un poivron ouais

*Speaker 2:* Il a l'air d'être [euh] -- poubelle

*Speaker 1:* Poubelle

*Speaker 2:* [Ouais]

*Speaker 1:* Je vais prendre en photo, -- donc ça ce seraient les deux seuls que normalement vous -- gardez [euh]

*Speaker 2:* [Ouais, ouais] ouais celle là, elle a l'air d'être -- à moins que l'autre côté est complètement pourrie donc [euh] -- on ne sait pas

*Speaker 1:* On ne sait pas

*Speaker 2:* Et là -- mon full, moi je l'ouvrirait voir si, si l'autre côté est, est saint quoi

*Speaker 1:* Ouais, ouais celle la oui c'est marrant je -- quand je l'ai reçu, je savais pas si -- je me suis dis que la personne s'est trompée, mais peut-être pas j'ai pas encore, je ne l'ai pas encore [euh]

*Speaker 2:* Ouais [rire]

*Speaker 1:* Alors, grande table, ok, parfait. Donc ouais finalement, vous [euh] ça vous arrive [enfin] c'est -- ça vous arrive [euh] très très très rarement de vous retrouver avec des fruits et légumes dans cet état là? En fait.

*Speaker 2:* [Ouais, non, ouais]. Parce que [bah] par exemple, j'ai acheté des carottes là y sont. Je vais les, je vais les acheter demain [euh] ils seront -- je les achent assez vite, et donc [euh] c'est pour manger dans la semaine donc [euh]

*Speaker 1:* Ouais

*Speaker 2:* Ouais

*Speaker 1:* Et quand vous parliez des -- des poireaux [euh] qu'est-ce qui fait que le vert, vous allez le mettre dans la soupe et que le blanc, par contre, vous allez prendre en vinaigrette ?

*Speaker 2:* Parce que j'aime -- j'aime bien le poireau vinaigrette

*Speaker 1:* Oui.

*Speaker 2:* Oui.

*Speaker 1:* Et le vert en vinaigrette ?

*Speaker 2:* Ouais. Après -- non, je préfère c'est meilleur le blanc tout seul que [euh] avec le vert. Je sais que ma ma belle sœur, quand elle fait des poireaux vinaigrette elle fait le vert avec et je préfère que il y ait que le blanc.

*Speaker 1:* Oui.

*Speaker 2:* Et donc, mais [euh] après ça va bien dans une soupe [euh] le vert.

*Speaker 1:* [Ouais] en termes de goût, quand vous mangez quand même le poireau [euh] --de façon un peu [euh] -- nature, le vert pour vous, c'est pas --

*Speaker 2:* [Bah], c'est, c'est un peu différent je trouve que du blanc

*Speaker 1:* Oui. Mais c'est un peu --

*Speaker 2:* Je le trouve moins bon

*Speaker 1:* [Ouais ouais] la plupart des gens c'est ce qu'ils font finalement c'est que le verre --il y en a qui jettent le verre.

*Speaker 2:* [Ouais] bah non

*Speaker 1:* il y en a qui mettent en soupe.

*Speaker 2:* [Ouais] bah moi, je -- je jette pas le verre, je fais souvent soupe -- ouais

*Speaker 1:* Ouais

*Speaker 2:* Ouais

*Speaker 1:* Bon bah ça marche

*Speaker 2:* Poireaux, pommes de terre.

*Speaker 1:* Oui, les soupes c'est pratique, c'est toujours un peu --

*Speaker 2:* Ouais ouais [rire]

*Speaker 1:* Mettre tous les légumes, c'est toujours une réussite [rire] -- bon bah ça marche --bien, très bien, merci beaucoup.

**Household F29, Interview 1**

*Mme 1:* Je lance l'enregistrement. Et là, c'est parti ! F 29. [Alors], [rire] vous êtes identifiée sous-- Pour les causes de-- des raisons d'anonymisation, [en fait], vous avez un numéro. Voilà !

*Mme 2:* Très bien ! Nous sommes donc le département Finistère.

*Mme 1:* Vous êtes en plus le département Finistère, [rire] effectivement, c'est tout à fait ça. [Euh] donc [en fait], l'idée du, de l'entretien, c'est de vous poser un certain nombre de questions sur votre consommation en termes de fruits et légumes, vos pratiques, comment ça se passe ? Enfin voilà, tout ce qu'il y a autour de ça. Il n'y a pas de bonne réponse. Il n'y a pas de mauvaise réponse, [en fait].

*Mme 2:* Ouais, ouais !

*Mme 1:* C'est vraiment votre expérience qui nous, qui, qui nous intéresse. [Euh] donc voilà ! Donc c'est enregistré mais anonymisé, [euh] voilà ! Donc, j'ai juste besoin que vous me disiez que ça vous convient et puis, on peut y aller.

*Mme 2:* Ça me convient très bien. [rire]

*Mme 1:* [Ben] c'est parfait, impeccable ! Et du coup, on va commencer par une [euh], une petite devinette.

*Mme 2:* Oui !

*Mme 1:* Pour débuter, [euh] je vais vous demander de- de penser à un légume ou un fruit [euh] que vous aimez particulièrement.

*Mme 2:* Oui !

*Mme 1:* Et puis [euh] d'essayer, sans me dire ce que c'est--

*Mme 2:* Mm-hmm !

*Mme 1:* De me le faire deviner en me le décrivant. [rire]

*Mme 2:* [Bon] ! [Alors], c'est un fruit que l'on a plutôt tendance à classer dans les légumes, qui est un fruit de saison, actuellement, et qui est rouge.

*Mme 1:* [rire] [Alors], [bon] je pense que j'ai déjà un petit peu deviné.

*Mme 2:* D'accord ! [rire]

*Mme 1:* On va creuser un petit peu. [rire] Pour- pour vous [en fait] [euh] si vous devez le- le décrire,

*Mme 2:* Mm-hmm, mm-hmm !

*Mme 1:* Au-delà donc des quelques descriptions que vous venez de me donner, [euh] qu'est-ce qui vous vient à l'esprit, [en fait] ? Si vous deviez le- le décrire, quelle est, je ne sais pas, sa texture ?

*Mme 2:* Je dirais que c'est un- c'est un produit [euh] que l'on trouve un peu toute l'année maintenant, mais qui ne devrait pas se trouver toute l'année, que l'on peut faire en pleine terre, sous serre, ordo. [Euh] que l'on peut-- Moi, j'aime bien sa fraîcheur.

*Mme 1:* Ouais !

*Mme 2:* Le nombre de ses variétés, les enfants en raffolent parce qu'elle existe-- Ça existe aussi en petite taille [rire] et c'est un fruit très ancien, [en fait]. Voilà !

*Mme 1:* D'accord ! [Euh] vous le mangez comment ? Vous le mangez [euh]--

*Mme 2:* Cru, cuit ?

*Mme 1:* Cru, cuit ? Voilà !

*Mme 2:* En salade, farci, en ratatouille, en ragoût. J'en mets de partout de toute façon. Donc [euh], je fais mes lasagnes avec, mes sauces perso aussi. [Euh] J'en ai sous forme de concentré, j'en ai en cubes, j'en utilise tout le temps.

*Mme 1:* Et finalement sur [diaphonie 00:02:56-00:02:57] fraîche, vous l'utilisez [euh]-- C'est la première chose que vous avez dit, que c'était un produit de saison ?

*Mme 2:* Oui !

*Mme 1:* Donc, j'imagine que--

*Mme 2:* [Ah] moi, je suis très [euh] très, je suis très à cheval là-dessus, [en fait]. Je n'achète que rarement des produits qui ne sont pas de saison. Il y a bien entendu des exceptions. Quand on a des enfants [ben] les bananes, c'est ce qu'il y a de plus souvent dans la coupe à fruits. [Euh] ouais, mais je fais quand même très attention d'acheter des agrumes en hiver, de-- Les fruits d'été, je les prends qu'en été, enfin, voilà ! Déjà les prix m'aident aussi [rire] parce que bon, voilà, mais c'est aussi un choix.

*Mme 1:* C'est aussi un choix ?

*Mme 2:* [Ah]! Oui ! Oui !

*Mme 1:* D'accord ! [Alors], [bon] [bah] vous avez répondu à toutes ces petites questions de relance, mais [euh] si vous deviez dire en un mot, mais vous l'avez peut-être dit, je vais vous le faire répéter, ce que vous aimez particulièrement dans ce fruit ou légume. Voilà !

*Mme 2:* [Euh] moi, c'est sa, sa fraîcheur.

*Mme 1:* Sa fraîcheur ?

*Mme 2:* Ouais, ouais ! C'est un fruit juteux. C'est un fruit qui s'accommode avec tout. En tout cas, avec beaucoup de choses, que ce soit fruit-- Enfin, que ce soit sucré ou salé parce que moi, j'en fais des confitures aussi.

*Mme 1:* D'accord !

*Mme 2:* C'est très, très bon !

*Mme 1:* Mm-hmm !

*Mme 2:* En fonction des variétés, vous avez [euh] des saveurs vraiment, complètement différentes, des textures différentes aussi, en fonction des variétés et [euh] je trouve ça hyper intéressant à cuisiner, [en fait].

*Mme 1:* OK !

*Mme 2:* Oui, je suis une maman qui cuisine beaucoup. Donc, ouais, j'aime bien, [en fait], travailler ce produit-là.

*Mme 1:* Travailler ce produit-là ! [Euh] Et si [euh] vous deviez lui trouver un inconvénient ?

*Mme 2:* Parfois le prix. Ouais !

*Mme 1:* OK ! Bon ! Je crois que votre fruit [diaphonie 00:04:38-00:04:39] qui est pris pour un légume est une tomate. [rire]

*Mme 2:* Tout à fait ! Bravo ! [rire] Vous avez droit à un café ! [rire]

*Mme 1:* Et ce que vous dites est très, très juste. La plupart des gens pensent que c'est un légume.

*Mme 1:* [Eh] Oui !

*Mme 1:* [En fait], c'est un fruit. Et oui ! Vous en cultivez d'ailleurs ?

*Mme 2:* [Ben] oui !

*Mme 1:* Et oui, voilà ! [rire]

*Mme 2:* Là, on a mis des tomates cerises pour les enfants. Donc, c'est-- ce sont eux qui s'en occupent.

*Mme 1:* Mm-hmm !

*Mme 2:* [Bon] elles ont eu un petit peu de mal quand elles sont passées de- sous la verrière à l'extérieur. Elles n'ont pas aimé trop le changement de température mais elles repartent donc je pense qu'on en, on en aura bientôt. Il y a deux variétés différentes. Là, il y en a des oranges allongées et des tomates cerises classiques, rondes et rouges.

*Mme 1:* Oui, des classiques rondes rouges. D'accord !

*Mme 2:* Voilà ! Ils mangent ça comme des bonbons, mes enfants, c'est impressionnant. Vous savez, on achète parfois les barquettes de un kilo en grande surface ou même parfois à la ferme, les enfants, si je leur enlève pas dans dix minutes, il y en a plus. Mais c'est impressionnant comme ils aiment ça. Certaines sont très sucrées. Donc, c'est vrai que c'est un- c'est un fruit que vous pouvez manger très bien. Je suis sûre que si je vous faisais goûter, soit le-- Des fois, je fais des tatins de tomates cerises avec un tout petit peu de sucre. Vous avez l'impression de manger un gâteau, tellement que c'est bon, [quoi] ! Donc les enfants, c'est vraiment le fruit qu'ils mangent le plus, le plus facilement et sans rechigner, quoi !

*Mme 1:* En saison ! [Bon] d'accord, super ! Maintenant, on va parler un petit peu de vos, vos pratiques d'achat, de stockage, voilà, ce genre de choses. [Alors] déjà, globalement, [en fait], comment vous achetez vos fruits et légumes frais ? On reste bien dans le domaine du frais. Et puis, [ben] comment vous vous organisez, de manière générale ? Qui achète par exemple ? Qui fait les courses ?

*Mme 2:* C'est moi la plupart du temps. [Alors] non, il n'y a pas que moi qui fais les courses, mais en général, quand c'est mon mari qui y va, c'est moi qui lui fais la liste et il prend ce que je, ce dont j'ai besoin. C'est moi qui cuisine principalement, même si mon mari cuisine très bien aussi.

*Mme 1:* Mm-hmm !

*Mme 2:* Mais [bon], c'est ponctuel chez lui. C'est moi qui prépare, on va dire, neuf repas et demi sur dix [rire] en général. J'ai plusieurs modes d'approvisionnement. Le plus rapide et le plus classique et le moins onéreux, c'est la grande surface.

*Mme 1:* Mm-hmm !

*Mme 2:* [Euh] on en a plein aux alentours, [alors] ça peut être en drive ou en magasin. Ça dépend le temps que j'ai, [en fait], pour faire mes courses. Il faut être très organisé.

*Mme 1:* J'imagine.

*Mme 2:* Sinon [euh], une fois par mois, je vais chercher mes légumes parce qu'il ne- elle ne trouvait pas de fruits. Mais je vais chercher mes légumes à Plougoulm, à la ferme.

*Mme 1:* D'accord !

*Mme 2:* Chez un agriculteur bio.

*Mme 1:* OK !

*Mme 2:* Pourquoi Plougoulm ? Parce qu'[en fait], j'ai sympathisé avec cette personne via les réseaux sociaux, qui avait mis une annonce parce qu'il est-- Elle s'est lancée avec son mari-- C'est un jeune couple avec un jeune enfant.

*Mme 1:* Mm-hmm !

*Mme 2:* [Euh] qui est donc sur Plougoulm et dont le mari a repris, si j'ai bien compris, l'exploitation de ses parents.

*Mme 1:* D'accord !

*Mme 2:* Sauf que ils ont mis plusieurs années à revenir au bio, c'est-à-dire qu'avant, c'était une- une exploitation de- de culture raisonnée, je crois. Et ils ont voulu passer au bio. Donc, ils ont dû attendre trois ou quatre ans, je ne sais plus, pour couvrir la terre. Enfin, je ne sais plus, elle m'avait expliqué, mais je ne pourrais pas vous redire, pour pouvoir, elle, avoir le label bio. Et donc on se- on se fournit chez eux. Là, c'est ultra frais, ultra de saison parce que quand il y en a plus- il y en a plus. [rire] Voilà ! Pour mes fraises, je vais à Plougastel, acheter mes fraises. Ou [alors] j'achète sur le marché un petit, [euh] un petit exploitant indépendant qui vient de Plouvien.

*Mme 1:* D'accord ! Sur le marché de Lannilis ?

*Mme 2:* Non, ici !

*Mme 1:* Ici ? D'accord !

*Mme 2:* On a le samedi et le dimanche, [en fait] une [euh], une personne qui vient de Plouvien et qui propose des fraises. Et quand je veux faire mes confitures, je vais directement à Plougastel parce que c'est vachement moins cher. Voilà ! [rire]

*Mme 1:* C'est bon ! [toux] Et du coup [euh], si, enfin, vous avez aussi parlé du [euh]- des-- Comment dire ? Des grandes surfaces.

*Mme 2:* Ouais !

*Mme 1:* [Euh] vous achetez quoi comme fruits et légumes frais en grande surface ?

*Mme 2:* Ça dépend.

*Mme 1:* Ça dépend ?

*Mme 2:* Ça dépend. J'achète le plus souvent tout ce qui est salade verte. [Euh] qu'est-ce que je prends là-bas ? Les tomates quand je n'en ai pas. J'achète [euh] [toux] [ben] en fait, en gros, c'est difficile de vous répondre parce qu'[en fait], j'achète ce que je ne trouve pas forcément à Plougoulm.

*Mme 1:* Voilà, c'est ça.

*Mme 2:* Donc, ça varie, en fait. C'est pas toujours la même chose. Puisque à Plougoulm principalement, j'y prends mes pommes de terre, mes oignons, mes échalotes, mon ail, mes courgettes, mes carottes. Elle ne fait pas de tomates. Donc, c'est principalement tomates, en grande surface. Le céleri, j'achète en grande surface aussi, quand c'est l'hiver et que je fais des soupes, j'aime bien. Et en gros, c'est tout parce que sinon tout le reste, ils font-- Tout ce qui est courge, je les prends à Plougoulm. Tout ce qui est navet, quand c'est la saison, je prends à Plougoulm. Et tout ce qui est butternut, potiron, à l'automne, c'est pareil, je prends là-bas. Les brocolis, je prends là-bas, les artichauts, je prends là-bas.

*Mme 1:* Donc, [en fait], c'est plus--

*Mme 2:* C'est de l'appoint en fait, en grande surface.

*Mme 1:* C'est un complément. Voilà, c'est ça. Ouais, c'est le complément de--

*Mme 2:* Ouais, voilà ! Ou entre deux cultures, [euh] voilà, les légumes ne sont pas encore tout à fait à point. [Bon] je fais l'appoint, moi, en grandes surfaces, mais je privilégie plus le petit exploitant du coin, quoi !

*Mme 1:* Et [alors], du coup, ça me fait revenir deux questions, [en fait].

*Mme 2:* Oui ?

*Mme 1:* [Euh] sur votre organisation. Déjà, vous, enfin, premièrement, vous avez parlé du fait que vous y allez une fois par mois.

*Mme 2:* Oui, je prends en gros.

*Mme 1:* Donc du coup, vous prenez en gros. J'imagine que c'est aussi lié [euh] [ben] à la structure de votre foyer ? Puisque--

*Mme 2:* Absolument, ouais !

*Mme 1:* Vous êtes huit en tout, si j'ai bien compris ?

*Mme 2:* Oui ! Et puis, pour rentabiliser aussi le- le trajet, parce que c'est quand même pas à côté.

*Mme 1:* Mm-hmm !

*Mme 2:* Si finalement, le prix du trajet vous revient plus cher que d'acheter vos légumes sur place, non ! Il n'y a pas d'intérêt. Alors que là, en plus, les légumes se- se conservent beaucoup plus longtemps [en fait] que en grande surface.

*Mme 1:* D'accord !

*Mme 2:* Vous verrez sur mon petit livret là, là où j'ai le plus de pertes, [en fait], c'est les grandes surfaces.

*Mme 1:* C'est les grandes surfaces.

*Mme 2:* Certainement parce qu'ils passent en chambre froide ou je ne sais quoi. Et ici [euh] [ben] moi je trouve que c'est une hérésie de mettre des tomates au frais, par exemple. Voilà ! Mais on est obligé parce que si vous commencez [euh] à casser la chaîne du froid [ben] vous êtes sûre que deux jours après, vous jetez vos tomates, [quoi].

*Mme 1:* Ouais !

*Mme 2:* C'est dommage, mais ici, c'est comme ça ! Moi, je viens du sud de la France. Les tomates, les citrons, tout ça, enfin, j'en ai connu depuis mon enfance. On n'a jamais mis [euh] au réfrigérateur quoi que ce soit en légumes, quoi.

*Mme 1:* Et du coup, ça veut dire qu'[en fait], pour vos stockages, vous faites finalement d'une certaine manière la différence par rapport à--

*Mme 2:* Oui !

*Mme 1:* Comment vous vous décidez, finalement, si vous stockez dans le frigo ou si vous stockez pas dans le frigo ?

*Mme 2:* [Alors], ça dépend de l'endroit où je les achète.

*Mme 1:* C'est ça ?

*Mme 2:* Je sais que ce que j'achète à la ferme n'est pas conservé en chambre froide. Donc, je peux sans problème et ça se vérifie, garder mes légumes beaucoup plus longtemps dans mon entrée parce qu'il y fait plus frais que dans la maison. Et il ne nécessite pas de conserver cette chambre froide, quoi, si vous voulez.

*Mme 1:* Oui, oui !

*Mme 2:* Et puis, ça permet, entre guillemets et entre parenthèses, [euh] de ne pas avoir de problème avec les légumes et les fruits que vous achetez. Parce que parfois, en grande surface, les fruits et légumes que vous achetez ne sont pas à maturité. Et le fait de les mettre en chambre froide, ça ralentit considérablement, en fait leur, leur progression quoi. Donc, je n'aime pas trop [rire] acheter en grande surface, mais des fois par obligation, on est, enfin voilà, on n'a pas le choix. Donc, quand je n'ai pas le choix, je le fais, mais je ne trouve pas ça bien.

*Mme 1:* D'accord !

*Mme 2:* Voilà !

*Mme 1:* OK ! [Euh] je commence à- voilà, à comprendre le- le système. Et [alors], la deuxième question qui m'est venue à l'esprit par rapport à ça, c'est que vous avez- vous m'avez expliqué que finalement, vous [ben] vous preniez aussi de saison.

*Mme 2:* Oui !

*Mme 1:* Donc, j'imagine, vous y allez une fois par mois et puis vous prenez ce qu'il y a.

*Mme 2:* Oui !

*Mme 1:* Du coup, comment fonctionne votre anticipation par rapport à ça ? C'est-à-dire que, est-ce que--

*Mme 2:* Je sais à l'avance ce que je vais chercher.

*Mme 1:* D'accord !

*Mme 2:* Elle est très organisée. Donc, [en fait], elle m'envoie des tableaux--

*Mme 1:* Mm-hmm !

*Mme 2:* De ce qui est disponible la semaine suivante ou pas. Et [euh] comme moi, elle sait que je viens qu'une fois par mois, elle anticipe un peu plus, c'est-à-dire qu'elle me les envoie dix jours avant le jour du retrait pour que je puisse avoir le temps de faire mes calculs et, et calculer mes quantités ou [euh] préparer mes menus, parce que je suis un peu psychopathe là-dessus, je sais toujours, enfin, pour une semaine, je sais ce que je vais cuisiner en fonction de ce que j'achète, voilà.

*Mme 1:* D'accord !

*Mme 2:* Et j'achète en fonction de ce que je cuisine. C'est-- Voilà ! Donc c'est pour ça que je vous disais, mon mari, il lui arrive de faire des courses, mais [euh] je sais déjà mes menus qui sont préétablis plus ou moins. Bien sûr, c'est pas rigide, mais plus ou moins pour ce qui est produit frais, en tout cas, c'est ce que je fais.

*Mme 1:* D'accord !

*Mme 2:* Voilà ! Et il y a une chose que je ne vous ai pas dit, c'est que, pareil, les œufs, je vais les chercher à la ferme aussi.

*Mme 1:* D'accord !

*Mme 2:* Voilà !

*Mme 1:* D'accord !

*Mme 2:* De préférence. Il m'arrive d'en acheter en grande surface, mais c'est quand je n'ai pas d'autres choix non plus.

*Mme 1:* D'accord !

*Mme 2:* Voilà !

*Mme 1:* Donc, [en fait], vous privilégiez la ferme avec l'organisation quand même qui est, en fait, vous êtes très organisée là [hein]

*Mme 2:* Ouais ! [Bah] on est obligé. [rire]

*Mme 1:* Et c'était la question que j'allais vous poser.

*Mme 2:* Ouais !

*Mme 1:* [Euh] est-ce que ça a toujours été comme ça ou est-ce que c'est finalement aussi--

*Mme 2:* Non, j'ai toujours été comme ça.

*Mme 1:* Vous avez toujours été comme ça ?

*Mme 2:* Ouais, même avant d'avoir les enfants. Mais je pense que je le serais moins si on était financièrement plus à l'aise, certainement.

*Mme 1:* Mm-hmm !

*Mme 2:* Mais comme on est obligé de faire attention, [ben] ça se goupille très bien parce que les courses, c'est pareil, c'est cadré. Là, on parle des produits frais, des légumes, fruits. Mais pour tout le reste, c'est de la même façon que je fonctionne. Donc les courses d'appoint, je sais que je vais faire une fois les courses par semaine. Et [euh] et dans ces courses-là, je sais déjà ce que je prends.

*Mme 1:* D'accord !

*Mme 2:* Voilà ! Il peut y avoir bien sûr des variations, mais en général, on étudie [euh] les prix de ce qu'on achète, quoi.

*Mme 1:* Oui, ça fait aussi partie des choses.

*Mme 2:* [Eh] oui, malheureusement ! [rire]

*Mme 1:* [Euh] je vous ai posé la question pour le petit-- On avait-- On a une petite question sur ce qu'on appelle l'auto approvisionnement, donc le fait de cultiver--Enfin, j'imagine que vous avez pas forcément la--

*Mme 2:* [Bah] [en fait], on est-- Pour l'instant, on est obligé de tout cultiver en pot ou en jardinière. Donc, on a des fraises, des framboises et des tomates cette année, plus mon petit jardin aromatique. Mais c'est prévu que dans l'aménagement [euh] du jardin, on ait un petit, [euh] un petit potager, [en fait]. Voilà !

*Mme 1:* Vous feriez une, une différence vous, entre un produit que vous avez cultivé vous-même, qui a poussé dans votre jardin et puis quelque chose que vous allez acheter [alors], peut-être par étape chez le producteur et puis quelque chose que vous achetez au supermarché ?

*Mme 2:* [Ah] [ben] oui, bien sûr ! Je crois qu'il y a quand même une, une dimension affective. Quand vous l'avez chez vous, c'est vos enfants qui s'en occupent, [euh] que vous voyez le fruit ou le légume poussé, ça n'a pas le même impact, en tout cas sur les enfants que quand vous allez l'acheter sur un étal, qu'il soit au marché, en grande surface ou à la ferme. Enfin, bien sûr, c'est différent. Après, moi, je ne vous cache pas que je me lancerai pas dans les cultures compliquées. Je ne mettrai jamais de pommes de terre chez moi, par exemple. Ce n'est pas que c'est compliqué techniquement, mais il faut avoir de la surface, il faut avoir du temps pour ça. Voilà ! Moi, je suis adepte du [euh] naturel dès qu'on peut, mais aussi avoir un peu de temps pour vivre quoi. Donc, il ne faut pas n'avoir que ça à faire. Voilà !

*Mme 1:* Et c'est un métier [hein] !

*Mme 2:* [Ah] oui, oui, absolument ! Quand je vois les amis qui sont de vue, les amis où je m'approvisionne à Plougoulm, ils n'ont pas de vacances, ils n'ont pas de week-end, ils partent jamais. Ils n'arrivent même pas à se faire remplacer quand ils veulent partir ne serait-ce qu'une semaine. Enfin, c'est une vie de dingue, en fait. [Bon] ils en ont, je ne sais combien d'hectares, bien sûr, mais ils n'ont que ça. Donc c'est hyper hyper dur quoi.

*Mme 1:* [Euh] vous m'avez donné une liste assez importante de fruits et légumes, enfin, et principalement de légumes puisqu'on a listé sur-- [Euh] est-ce que, par exemple, les fruits que vous consommez régulièrement à côté de ça, ce sont lesquels ?

*Mme 2:* Non, il y en a plein. [Euh] [alors], pommes, kiwis, bananes, poires, en fonction des saisons [hein] Oranges, pamplemousses, les fraises, bien sûr ! Framboises, myrtilles, je vous donne ce que les enfants préfèrent parce qu'il y en a, il y en a d'autres mais [euh] qu'est-ce qu'on prend d'autre ? Ouais, c'est à peu près tout. Pour les enfants, c'est ce que je prends le plus souvent.

*Mme 1:* D'accord ! Et puis sur les légumes, donc vous avez cité les courgettes, cité les carottes--

*Mme 2:* [Ah] oui, les légumes oui. On a pommes de terre, courgettes, carottes, tomates, beaucoup de tomates. Salades, beaucoup de salades. [rire] [Euh] les navets, les poireaux, ils aiment beaucoup les poireaux. [Euh] tout ce qui est courge, potiron, butternut, [euh] comment on appelle ça ici ? Les courges longues rouges là ? Les courges rouges. Les courges musquées, je crois, vous appelez ça ici. En fait, ce sont de longues courges, la peau est plutôt orangée et l'intérieur est orange, très vif, avec des gros--

*Mme 1:* C'est pas des butternuts, celles-là ?

*Mme 2:* Non !

*Mme 1:* Non ?

*Mme 2:* Non, c'en est encore une autre. Je crois que c'est la courge musquée qu'elle s'appelle.

*Mme 1:* D'accord !

*Mme 2:* Donc voilà ! Ça on en mange beaucoup. Potimarron, on en mange beaucoup. Qu'est-ce qu'ils aiment bien encore ? En produit frais [hein] parce que sinon, il y en a d'autres mais pas frais. Les artichauts, brocolis, [euh] moins souvent choux de Bruxelles parce que-- Après, c'est des histoires de goût mais je leur en fais quand même. [Euh] tout ce qui est choux en général.

*Mme 1:* Mm-hmm !

*Mme 2:* Les blancs, les rouges, les choux pommes, les choux frisés, les choux machin. Et voilà [hein] !

*Mme 1:* Et les choux là, vous avez cité les choux de Bruxelles--

*Mme 2:* Oui !

*Mme 1:* Ça va être la même, la même fréquence ou-- ?

*Mme 2:* Non, pas du tout !

*Mme 1:* Non ?

*Mme 2:* Non, les choux de Bruxelles, c'est quand j'ai envie de faire plaisir à mes garçons parce que je ne sais pas pourquoi ils adorent ça. Moi, j'aime moins. Je trouve le goût plus prononcé, enfin, c'est que j'aime moins. Mais je leur en fais quand les prix descendent un peu et que j'ai, ou alors que j'ai l'opportunité d'en avoir.

*Mme 1:* Mm-hmm !

*Mme 2:* Soit à la ferme, soit ailleurs, j'en prends.

*Mme 1:* D'accord ! Donc, ça fait partie, effectivement de ces--

*Mme 2:* Oui !

*Mme 1:* Et [euh] par exemple, un fruit que vous mangez [euh] rarement ou jamais.

*Mme 2:* La mangue, malheureusement.

*Mme 1:* La mangue ?

*Mme 2:* J'adore ça. Tout ce qui est fruits exotiques, [en fait]. [Euh] oui, il y a l'ananas aussi qu'on mange beaucoup.

*Mme 2:* Mm-hmm !

*Mme 2:* L'été [ben] il y a toutes les pêches, abricots, nectarines. Un peu plus tard, il y a le raisin. [Euh] Ils adorent tout ce qui est fruits rouges, [en fait]. Donc, tout ce qui est framboises, mûres, myrtilles, le cassis un peu moins mais [euh] ouais, voilà, tout ça, ils adorent. Les kiwis, ils aiment bien. Qu'est-ce qu'on a après ? Ouais, pommes, poires, c'est les classiques, bananes, bien sûr ! L'hiver, c'est tout ce qui est agrumes, oranges, pamplemousses, citrons aussi parce que j'ai la chance d'avoir mes parents qui habitent à Menton. Donc, ils m'envoient des colis de, d'agrumes--

*Mme 1:* D'accord !

*Mme 2:* Que j'accommode de plein de façons différentes, en sirop, en confiture, en-- Je fais du [inaudible 00:20:58-00:20:59] enfin, je fais plein de trucs pour pouvoir les garder plus longtemps, ou en citronnade ou orangeade. Et voilà ! Je crois qu'on a fait à peu près le tour.

*Mme 1:* Et la mangue, c'est pour quelle raison finalement [diaphonie 00:21:10-00:21:11]

*Mme 2:* J'adore ce fruit !

*Mme 1:* Oui !

*Mme 2:* Et le prix ?

*Mme 1:* Et le Prix.

*Mme 2:* Le prix et le, l'éloignement surtout parce qu'on ne vit pas à côté. Donc, je ne connais pas d'exploitation de mangues en Finistère. [rire] Donc ouais, mais je dis mangue, mais [en fait], c'est pareil pour la papaye, la goyave. Enfin, moi j'adore tous ces fruits-là.

*Mme 1:* Mm-hmm !

*Mme 2:* Mais ils sont relativement chers. La patate douce aussi, on aime bien, mais c'est cher. [Euh] on consomme parfois du gingembre aussi, frais mais peu, enfin voilà ! Tout ce qui est-- [Ben] on a un petit souci quand même, mon mari et moi, avec l'empreinte carbone.

*Mme 1:* Mm-hmm !

*Mme 2:* Donc, on se dit, à quoi ça sert de faire toute l'année, attention à rentabiliser les allers retours [euh] en [inaudible 00:21:52-00:21:53] tout ça machin, pour acheter sain, naturel et, et rentable pour après derrière, acheter ce genre de produits. Donc, quand on le fait, c'est généralement pour se faire plaisir à Noël ou des choses comme ça, voilà ! On s'achète quelques petits fruits exotiques, mais- mais c'est rare et ce n'est pas tous les ans.

*Mme 1:* C'est un cadeau, [quoi].

*Mme 2:* Voilà, c'est le petit plaisir qu'on se fait nous, [quoi].

*Mme 1:* D'accord ! [Euh] [alors], si vous deviez, enfin, m'expliquer, me montrer, vous revenez de faire vos courses, par exemple, de légumes.

*Mme 2:* Ouais !

*Mme 1:* [Euh] du coup, vous m'avez dit que vous achetez en gros. Donc, j'imagine que vous arrivez avec la table pleine, voilà ! [Euh] comment ça se passe ? Qu'est-ce que vous faites ? Voilà ce qui--

*Mme 2:* [Eh] [ben] j'organise en fonction des modes de conservation. Je, je les range aux endroits appropriés, par exemple, tout ce qui est [euh] oignon, ail, échalotes, pommes de terre, enfin, les légumes qui ne nécessitent pas de chambre froide sont stockés dans mon entrée parce que j'ai la place et parce qu'à terme, il y aura de toute façon un endroit dédié pour ces légumes-là.

*Mme 1:* D'accord !

*Mme 2:* Ça peut être le cas des fruits aussi parce que les pommes, moi je les mets pas au frigo. [Euh] Les bananes, généralement, j'essaye de les prendre un peu plus vertes pour qu'elles aient le temps de, d'arriver à maturité, voilà ! Et c'est stocké dans mon entrée aussi. Et tout ce qui nécessite, [en fait], d'être au frais, j'ai [euh] plusieurs solutions. Alors que je ne dis pas de bêtises. Non, je ne crois pas qu'il m'arrive de congeler des fruits ou des légumes. Si ! Mais ce sont des légumes que je blanchis avant.

*Mme 1:* D'accord !

*Mme 2:* C'est-à-dire que je les pré-cuisine.

*Mme 1:* Ouais !

*Mme 2:* Et quand j'en ai vraiment en grosse quantité et que je sais qu'ils vont se perdre, si je les fais pas rapidement, je les blanchis, je les congèle. Donc, j'ai un congélateur dans ma buanderie et j'en ai un autre dans mon abri de jardin.

*Mme 1:* D'accord !

*Mme 2:* Si c'est pas suffisant. Comme je fais mes courses en gros, en général, [rire] ce frigo-là ne suffisait pas, et ce congélateur non plus. Du coup, on a doublé tout en fait.

*Mme 1:* D'accord !

*Mme 2:* Pour pouvoir [ben] à la fois, profiter des promotions quand il y en a et pouvoir stocker justement plus. Donc voilà ! Et sinon, [ben] j'ai mon, mon réfrigérateur qui me permet de stocker pas mal de choses qui nécessitent du froid.

*Mme 1:* Qui nécessitent du froid.

*Mme 2:* Voilà ! Comme par exemple, je vous disais tout à l'heure, mes parents m'envoient des cartons de, d'agrumes. Au départ, ils sont stockés dans mon entrée parce que ils ne nécessitent pas d'être au frais. Mais quand je vois qu'ils commencent à-- Que ça fait déjà un moment que je les ais et je ne veux pas qu'ils s'abîment, je les mets dans le tiroir de mon réfrigérateur. Vous allez voir, il y en a, par exemple.

*Mme 1:* Il y en avait sur la photo.

*Mme 2:* Ah [ben] voilà ! [rire] Mais c'est pas les mêmes depuis. [rire] On m'en a renvoyé.

*Mme 1:* J'imagine- j'imagine.

*Mme 2:* Voilà !

*Mme 1:* OK ! [Euh] il y a une question qui m'a traversé l'esprit, oui. Est-ce que vous-- Il y a des des, des légumes par exemple, ou des associations fruits légumes que vous évitez de faire ou au contraire que vous faites ? Quand vous stockez.

*Mme 2:* J'ai pas-- [Eh] [ben] [diaphonie 00:24:56-00:24:57] [Alors], les fruits sont d'un côté, les légumes de l'autre, déjà. Mais effectivement, si j'achète des kiwis et qu'ils sont pas très mûrs, je vais les mettre avec des pommes.

*Mme 1:* D'accord !

*Mme 2:* [Euh] si [euh]-- [Ben] je vais éviter de mettre des, des légumes lourds sur des légumes fragiles, par exemple. [Euh] après, oui, vous allez me dire, elle est psychopathe jusqu'à ce point-là ? Oui, c'est vrai que j'ai une manie, c'est que je mets le-- J'ai plusieurs étagères et je mets au-dessus les légumes qu'il va falloir que je cuisine en premier.

*Mme 1:* Oui !

*Mme 2:* Voilà, puis en dessous [ben] et cetera, et cetera. Donc oui, c'est vrai, je suis comme ça. Mon mari se moque de moi suffisamment, avec ça. Mais sinon, non, je n'ai pas de--

*Mme 1:* Et ça, c'est très intéressant. [rire] Vous faites-- Non, mais c'est vraiment intéressant. Vous le faites parce que ça vous rappelle ou ?

*Mme 2:* Oui !

*Mme 1:* Oui ?

*Mme 2:* Parce que je sais que j'ai cette organisation-là et que si je le fais pas, j'oublie.

*Mme 1:* D'accord ! Ouais !

*Mme 2:* Et comme je n'aime pas le gâchis, [ben] je préfère faire ça [en fait].

*Mme 1:* D'accord !

*Mme 2:* Voilà !

*Mme 1:* Donc c'est une façon en fait de stocker--

*Mme 1:* Ouais !

*Mme 2:* D'organiser mon- mon esprit et ma- ma cuisine quoi.

*Mme 1:* Votre cuisine ?

*Mme 2:* Ouais !

*Mme 1:* D'accord ! OK !

*Mme 2:* C'est pour ça que je vous disais tout à l'heure, j'ai des menus dans la semaine, mais ce n'est pas figé, c'est-à-dire que le menu que j'ai prévu le vendredi, si je vois que les légumes vont s'abîmer pour le mardi, je vais le faire mardi quoi ! Je ne suis pas idiote non plus à ce point-là. Donc voilà, c'est en ça que je disais ça peut varier.

*Mme 1:* Ça peut varier.

*Mme 2:* Voilà !

*Mme 1:* [Euh] Est-ce que ça vous arrive de nettoyer tous ces endroits de stockage ? Ou est ce que vous avez [diaphonie 00:26:29-00:26:30] par rapport à ça ? Vous vous organisez comment ?

*Mme 2:* Non, [en fait], je n'ai pas de routine. Mon frigo, il est lavé deux fois par mois. Mon entrée, je la nettoie quand j'ai besoin parce que les pommes de terre à la ferme, c'est pas comme les pommes de terre toutes propres [hein] qu'on achète en grande surface. Donc, quand il y a besoin, je le fais. [Euh] Si je stocke mes fruits ou légumes sur un papier absorbant, par exemple, je veille à ce qu'il ne soit pas trop crade, enfin, c'est normal, sinon je le remplace. Mais sinon, [ben] tout ce qui est coupe à fruits, tout ça, oui. Enfin, je ne saurais pas vous donner la fréquence à laquelle je la nettoie, mais quand il y a besoin, je le fais quoi. [Diaphonie 00:27:12-00:27:13] Et de toute façon, je nettoie tous fruits ou légumes avant de le consommer moi.

*Mme 1:* D'accord !

*Mme 2:* Donc, [euh] même si par mégarde il avait été entreposé dans un endroit qui n'est pas forcément propre à cent pour cent, de toute façon, mes enfants ne mangent pas les fruits avec la peau.

*Mme 1:* Mm-hmm !

*Mme 2:* Et [euh] par exemple les fraises--

*Mme 1:* Mm-hmm !

*Mme 2:* Moi je les fais baigner d'abord dans de l'eau, avec de l'eau froide, avec un peu de bicarbonate dedans.

*Mme 1:* D'accord !

*Mme 2:* Voilà ! Et effectivement, on se rend compte qu'il y a quand même plein de saletés [en fait] qui partent. Donc [euh] voilà ! Et je mets du jus de citron en général quand je fais des fraises.

*Mme 1:* D'accord !

*Mme 2:* Voilà. Mais ça, c'est, je crois, une des formations du Sud. On met du citron partout.[rire] J'en mets dans la salade, non mais partout.

*Mme 1:* [rire] OK ! [Euh] Vous avez parlé du papier absorbant. Et [en fait] quand j'ai regardé les photographies hier soir pour préparer mon entretien avec vous ce matin, j'ai vu ce papier absorbant. Et [euh] à quoi il nous sert ?

*Mme 2:* [Alors], il me sert principalement dans le tiroir de mon réfrigérateur parce qu'il y a parfois de la condensation ou de l'humidité qui peut altérer le fruit ou le légume qui est contenu dedans. J'ai oublié de vous dire, concombre aussi, c'est gros consommateur de concombre ici. [rire] Et [euh]-- Et [en fait], pour éviter ça, tout simplement, [ben] je mets un papier absorbant. Et parce que parfois il peut arriver aussi [ben] dans les agrumes que mes parents m'envoient, qu'il y en ait un qui a eu un choc, mais je le vois pas de suite et qui s'abîme. Donc auquel cas ça, ça contient quand même le fruit avant que je m'en aperçoive et quand je m'en aperçois, je change le tout. Voilà ! Mais c'est principalement pour ça.

*Mme 1:* C'est principalement pour ça et donc c'est aussi une mesure, totalement, de conservation, de bonne conservation.

*Mme 2:* Ouais, d'hygiène quoi. Ouais ! Ouais ouais, absolument !

*Mme 1:* D'accord ! On a commencé un petit peu à aborder la phase suivante [euh] il y a un instant, c'est sur la préparation [en fait] et la consommation des fruits et légumes frais.

*Mme 2:* Mm-hmm !

*Mme 1:* [Euh] Donc, enfin, on a déjà évoqué, par exemple, les tomates que vous avez, vous m'avez dit, voilà, comment vous les consommiez, de façon très, très diverse et variée. [Euh] Je ne sais pas, on va en prendre un autre, par exemple, est-ce qu'il y en a un autre qui vous vient à l'esprit ? Est-ce que vous pouvez, pareil, me décrire un petit peu, [ben] finalement, sous quelle forme [euh] vous le consommez ? Est-ce que c'est, enfin, voilà, du cru, du cuit ? Je sais pas, on va prendre le concombre, on a parlé du concombre.

*Mme 2:* [Alors] Le concombre, moi, je ne le mange que cru.

*Mme 1:* Mm-hmm !

*Mme 2:* Je sais qu'on peut le faire cuire mais [euh] je ne m'y suis pas encore aventuré. [Euh] Donc le concombre, [en fait], on le mange principalement en crudités. Soit nature comme ça avec un, juste un, un filet de sel, soit [euh] les enfants aiment bien quand je le prépare avec soit un yaourt, soit de la crème fraîche allégée avec plein d'herbes dedans. Donc, ça ils aiment beaucoup et voilà, principalement ce qu'on fait avec le concombre.

*Mme 1:* [Euh] quand vous le préparez, c'est--

*Mme 2:* Je l'épluche.

*Mme 2:* Oui, vous l'épluchez.

*Mme 2:* Je le lave après l'avoir épluché.

*Mme 1:* D'accord !

*Mme 2:* Je le sèche avec un papier absorbant et je le coupe seulement après.

*Mme 1:* D'accord ! C'était ma, c'était ma question, [en fait]

*Mme 2:* Non, je me mange pas la peau.

*Mme 1:* Les les-- [Euh] [alors] j'ai, c'est une question-- C'est pas dans le guide, mais c'est une question qui me vient à l'esprit.

*Mme 2:* Mm-hmm !

*Mme 1:* On a parlé tout à l'heure des fruits et légumes qui sont dans le, le jardin. [Euh] Est-ce que vous avez cette même routine de les faire tremper dans du bicarbonate ? Enfin, voilà ! Non ?

*Mme 2:* Non, parce que-- [Alors], les fraises, oui. Il n'y a que les fraises [hein] que je- je- je consomme comme ça. [Euh] Sinon, [ben] les tomates, je les lave. Les framboises ne se lavent pas, donc-- Mais elles ne touchent pas la terre non plus trop, donc c'est pas bien gênant. L'herbe aromatique, ça dépend si je l'avais-- Si je la trouve sale, je le lave mais généralement, j'essaye qu'elle ne le soit pas trop parce que ça enlève quand même des propriétés de la plante. Donc, tout ce qui est basilic, le thym [euh]-- Là j'ai de la ciboule. Enfin, voilà, en général, non, ça, je ne lave pas, sauf si vraiment c'est sale avec de la terre dessus.

*Mme 1:* D'accord !

*Mme 2:* Et [euh] et voilà ! Les framboises, non, je ne les lave pas. Les fraises, oui. Les fraises, je les lave. Voilà !

*Mme 1:* D'accord ! Et si vous achetez par exemple des herbes aromatiques, [euh] est-ce que vous allez les laver ?

*Mme 2:* [Ah] Oui ! Je n'ai pas confiance. Je ne sais pas d'où ça vient. Donc dans le doute, si, je nettoie.

*Mme 1:* Voilà, c'est ça ! Donc il y avait une différence entre [diaphonie 00:31:46-00:31:47]

*Mme 2:* Chez moi parce que je sais ce que je mets dessus, c'est-à-dire rien. [rire] Je ne les traite pas. Tant pis ! Si un fruit est moche, il est moche, mais ce n'est pas pour ça qu'il est pas mangeable. Un légume, pareil. Mais d'ailleurs, à Plougoulm, des fois, on a des carottes qui ont des tailles, des des formes un peu biscornues, mais elles sont très, très bonnes quand même. Donc, on s'en fout, [enfin] on les nettoie, on les épluche et puis on les mange pareil quoi. La carotte, on la mange crue, cuite, sucrée, salée.

*Mme 1:* Et finalement, est-ce que il y a des légumes ? Je ne parle pas des fruits, on va revenir dessus puisque vous en avez parlé. Mais est-ce qu'il y a des légumes que vous consommez sans rien éplucher ? Juste comme ça, Pouf !

*Mme 2:* [Ben] Oui, la tomate, je ne l'épluche pas. Je la nettoie mais je ne l'épluche pas. Je ne l'épépine pas non plus. [Euh] C'est une bonne question. La salade, je la nettoie, deux fois. [Euh] Oui, les courgettes, je ne l'épluche pas, [diaphonie 00:32:52-00:32:53] je la nettoie mais je l'épluche pas, ou partiellement. Ça dépend ce que je vais en faire. Le brocoli, je le nettoie. Le chou fleur, je le nettoie. Les choux, en général, j'enlève les premières feuilles et je le nettoie. Je ne vois pas. La carotte, je l'épluche et je la nettoie. Non, c'est tout. Je ne pense pas-- À part la courgette, non, c'est vrai que je ne garde pas beaucoup de, d'épluchures. [rire]

*Mme 1:* Non non, mais c'est, c'est une question. [En fait] c'est, c'est vraiment une question qu'on se pose parce que finalement. Enfin, ces pratiques-là sont variées. [Euh] Je vais prendre un autre exemple, par exemple, le brocoli.

*Mme 2:* Oui ?

*Mme 1:* Vous l'avez cité mais [euh] quand vous le préparez, vous préparez comment ? Est-ce que--

*Mme 2:* À la vapeur. Souvent, c'est à la vapeur. [Euh] Les enfants préfèrent et mon mari plus, les choses naturelles finalement, que cuisinées en béchamel ou voilà ! Donc, je le coupe, je coupe les fleurettes.

*Mme 1:* Oui !

*Mme 2:* [En fait] Je les mets à l'eau claire. Je les rince. [Euh] Je les mets ensuite dans un panier à vapeur. Voilà ! Et ça cuit comme ça, avec juste du gros sel. On mange comme ça, il peut m'arriver d'en faire de la purée avec des pommes de terre. Et en gratin aussi.

*Mme 1:* Et la partie [euh] tige du brocoli ? [diaphonie 00:34:22-00:34:23] Voilà, ça, par contre, c'est une partie qui-- D'accord ! OK ! C'était juste pour avoir un petit, enfin, quelques notions justement sur les parties finalement, [en fait] et puis quel était le degré de de de votre préparation, [en fait] de-- Voilà !

*Mme 2:* Ouais ! Pareil pour l'artichaut, je sais qu'il y a des gens qui mangent ici le début de la tige. [Euh] Nous, on n'aime pas ça. Donc, on mange le cœur, on mange les feuilles, mais on ne mange pas le début là de-- Je ne sais pas comment ça s'appelle--- De la tige.

*Mme 1:* De la tige. [Euh] Je pense comme ça, enfin, c'est quelque chose qui me vient à l'esprit. [Euh] Je ne suis pas sûre de vous avoir entendu parler d'aubergine.

*Mme 2:* [Ah] Oui !

*Mme 1:* Vous venez du sud !

*Mme 2:* [Ah] [ben] Oui ! [rire] Malheureusement, les fermes d'ici n'en produisent pas ou peu ou très chères. Donc ça, c'est le produit, oui, que j'achète en grande surface entre le milieu du printemps et la fin de l'été quoi. Avec les poivrons. Ouais ! Ça c'est, c'est mon petit péché mignon. Je fais beaucoup de ratatouille [en fait] ou de légumes farcis.

*Mme 1:* Mm-hmm !

*Mme 2:* Et c'est très bon ! Donc, c'est vrai que ça, c'est le fr- le légume, pardon, que j'achète volontiers, même si c'est très cher et j'en mets moins.

*Mme 1:* Les aubergines, vous épluchez ou vous épluchez pas ?

*Mme 2:* [Alors] ça dépend. [rire] Si je l'ai fait farcie, je ne l'épluche pas, mais je la nettoie. Si je fais en ratatouille, j'aime bien le faire une bande sur deux.

*Mme 1:* D'accord !

*Mme 2:* Parce que ça cuit mieux.

*Mme 1:* D'accord !

*Mme 2:* Voilà ! Plus vite quoi !

*Mme 1:* D'accord ! Donc effectivement, il y a cette [euh], cette histoire d'apprêter aussi en fonction de la cuisson et [euh]-- D'accord ! [Euh] [Alors] On vient de le faire mais [euh] voilà, [en fait], on a un certain nombre de questionnements qu'on souhaiterait aborder avec vous sur [euh] le moment [en fait] où [euh] vous jetez [euh] un fruit ou légume, enfin, on n'en a pas encore parlé de ça, effectivement, [euh] ou une partie seulement. Donc finalement, [euh] à partir de quand vous avez l'impression que, enfin, vous vous dites non là, celui-là, je ne vais pas le consommer. Malo, je ne vais pas le consommer, je vais le jeter. Voilà !

*Mme 2:* Quand, déjà, visuellement, il ne me paraît pas consommable.

*Mme 1:* Oui !

*Mme 2:* Parce qu'il y a [euh] des points de moisissure qu'ils ont, qui sont apparus. Ou parce que-- [Alors] moi, je vais un peu plus loin que l'aspect extérieur. Je vais prendre comme exemple la banane.

*Mme 1:* Mm-hmm !

*Mme 2:* Il peut arriver que la peau soit très noire, mais que le fruit soit très sain à l'intérieur. Auquel cas je ne le donnerais pas forcément à manger comme ça à mes enfants, mais je l'accommoderais dans une compote ou une tarte ou un gâteau, enfin voilà ! Mais je vais m'en servir. Mais [euh] ouais, je pense que l'aspect visuel déjà il fait beaucoup ou [euh] parfois, c'est l'odeur aussi [hein] du légume ou du fruit. Une pomme de terre qui est pourrie ça pue quoi. Donc là, on s'en rend compte rapidement.

*Mme 1:* Ouais !

*Mme 2:* Et on l'enlève vite avant qu'elle ne contamine toutes les autres. Mais [euh] sinon, si j'ai un légume qui est partiellement abîmé, [ben] je vais enlever la partie abîmée mais je vais consommer l'autre, [quoi]. Je ne vais pas m'amuser, non, je ne suis pas du genre à jeter un, un fruit ou un légume entier parce qu'il a un défaut, quoi. Ouais !

*Mme 1:* Et [euh] si jamais vous avez un point comme ça, enfin voilà, une partie défaut vous, votre pratique, c'est d'aller, d'aller prendre [inaudible 00:37:52-00:37:53] ou au contraire de, d'être serré sur le--

*Mme 2:* Je ne me pose pas la question, je coupe et c'est tout [hein] ! C'est instinctif [en fait] je ne sais pas.

*Mme 1:* C'est ça.

*Mme 2:* Je-- J'analyse pas [en fait]. [rire]

*Mme 1:* [rire] J'imagine que vous n'analysez pas.

*Mme 2:* [Euh] Je fais comme je le sens. [Euh] ouais ! Et puis si j'ai, si j'ai coupé trop court,[ben] je, je recoupe le bout--

*Mme 1:* C'est ça !

*Mme 2:* Que j'aurais pas eu quoi. Mais [euh] non, je ne me pose pas cette question.

*Mme 1:* [Ah] Ouais ! Et là, du coup, c'est visuel, j'imagine.

*Mme 2:* [Ah] Oui, [ben] oui, oui ! Pour le coup, là, c'est visuel.

*Mme 1:* C'est visuel.

*Mme 2:* Mais après, des fois, vous, vous avez [euh] une pomme de terre quand vous l'épluchez, avant de l'éplucher, elle vous paraît saine et vous l'épluchez, et puis elle est toute abîmée à l'intérieur. Donc [ben] par, par essence, vous allez enlever la partie endommagée de la pomme de terre. Et là, pour le coup, vous ne pouvez pas savoir à l'avance combien vous allez enlever de la pomme de terre. [Alors] effectivement, s'il y a trois quarts de la pomme de terre abimée,[euh] je ne dis pas que je vais garder le dernier quart si vraiment il fait deux centimètres carrés, [quoi].

*Mme 1:* C'est ça.

*Mme 2:* Mais [euh] si-- On va, on va partir du principe que si une majorité du fruit ou du légume est abîmée, je vais le jeter. Mais s'il y a moins de la moitié, je vais le garder. On va enlever la partie abîmée et je vais consommer l'autre. Voilà, c'est mon idée de la rentabilité à moi, quoi. [rire]

*Mme 1:* D'accord ! Oui, vous analysez. [rire]

*Mme 2:* Oui, mais en le faisant, c'est vrai que je ne me pose pas la question.

*Mme 1:* [rire] Et [euh] quand, par exemple, vous avez parlé des pommes de terre, vous, vous trouvez une pomme de terre comme ça qui a, qui est abîmée dans un lot, [euh] vous ressentez quoi ? Ça vous fait quoi ?

*Mme 2:* [Ben] je dis que c'est naturel, enfin, c'est pas grave.

*Mme 1:* Oui !

*Mme 2:* Ça, ça vient de la terre [hein] donc, je ne sais pas si vous avez déjà cultivé des fruits ou des légumes dans votre enfance ou quoi. Moi, mes parents n'ont fait que ça. Donc [euh]-- Ils n'étaient pourtant pas jardinier ou cultivateur, mais [euh], mais ils aimaient ça, ils avaient un grand jardin. Moi, j'ai toujours mangé les fruits et légumes de mon père, donc [euh]-- D'ailleurs ça me fait un peu de la peine, maintenant, quand je vois combien on paye les choses ici qui n'ont pas de goût. Mais [bon], soit ! Mais, mais [euh] ça arrive que vous ayez tout un pied de de, de tomates ou de petits pois, vous savez pas pourquoi [ben] celui-là, il est abîmé. Vous avez parfois des pommiers qui sont véreux. Enfin, c'est- c'est la nature quoi, c'est comme ça. Pour moi, c'est gage aussi que ça n'a pas été [euh] trop traité. Donc c'est, bizarrement, plutôt rassurant.

*Mme 1:* Plutôt rassurant.

*Mme 2:* Ouais !

*Mme 1:* D'accord !

*Mme 2:* Je trouve.

*Mme 1:* OK ! [Euh] est-ce que vous avez, pour le coup [euh], parfois des-- [Alors], je ne suis pas censée dire le mot mais, quand vous touchez un produit comme ça, qui a altéré ou [euh] qui a un peu moisi, tout ça, il y a une réaction particulière ou finalement, c'est ce que vous venez de me décrire ?

*Mme 2:* Ça dépend du produit.

*Mme 1:* Oui ?

*Mme 2:* Ouais ! [Euh] qu'est-ce que j'ai comme exemple ? Non, en général, je n'ai pas de dégoût. J'ai pas, j'ai pas de réticence à, à me dire que je vais manger la partie saine de ce fruit-là. Les enfants, oui, c'est beaucoup plus compliqué pour eux. Chez eux, l'aspect visuel est [euh] franchement, je crois, primordial. Si le fruit est beau, s'il est tout lisse, s'il est tout bien préparé, machin, c'est, ça va. Mais s'il y a [euh] [alors] je dis pas un petit pas quand même, je parle vraiment la moitié du fruit abîmé, ils ne vont pas le prendre dans leurs mains.

*Mme 1:* Ouais, ouais !

*Mme 2:* [Ah] non ! Maman ! [rire]

*Mme 1:* [rire] D'accord ! Maman, il y a quelque chose dans la tanière, c'est ça ?

*Mme 2:* Oui ! Maman, maman, il va falloir que tu regardes la coupe à fruits, s'il te plait, parce que moi-- [Ben] Vas-y ! Fais-le ! [Ah] Non, moi je ne touche pas à ça. Et je vous parle d'ados [hein], pas de petit- petit [hein].

*Mme 1:* Ouais, ouais, ouais !

*Mme 2:* Ouais, ouais ! [Ah] oui, oui, [inaudible 00:41:43-00:41:44] c'est oulala ! Ouais !

*Mme 1:* D'accord ! Et, et si jamais, par le plus grand des hasards, je sais pas, vous croquez dans une pomme et puis [en fait], vous apercevez qu'il y a une partie qui est un peu--

*Mme 2:* Abîmée ?

*Mme 1:* Ouais !

*Mme 2:* Ouais, ça arrive.

*Mme 1:* Ça arrive ?

*Mme 2:* Ouais, ouais ! C'est-- J'aurais peut-être un-- Ouais ! Un petit- petit-- Ouais ! Un petit dégoût quand même.

*Mme 1:* Ouais !

*Mme 2:* Ouais ! Autant, ça me choquerait pas de me dire que ça peut arriver et que c'est naturel, que-- [Euh] non, je ne suis pas fan de tout ce qui est [euh] insecte.

*Mme 1:* Oui !

*Mme 2:* Tout ça, c'est pas mon truc. [rire] Non ! Je préfèrerais que ça n'arrive pas. [rire] En même temps, je, je ne vois pas trop dans quel cas ça pourrait m'arriver. Parce que c'est vrai que je regarde quand même avant de consommer quelque chose ou de faire consommer quelque chose. Je regarde quand même que ce soit viable entre guillemets à la consommation, mais effectivement ça pourrait arriver. Voilà !

*Mme 1:* OK ! [Bon] c'est très, très intéressant parce que là vous-- Voilà ! Vous avez décrit plusieurs choses et finalement [euh] cette idée que c'est naturel. Donc, c'est rassurant. Mais que en même temps, quand ça vous arrive--

*Mme 2:* Ouais, ouais !

*Mme 1:* C'est pas agréable. Voilà ! Il y a une sorte un peu de [diaphonie 00:42:58-00:52:59].

*Mme 2:* C'est ça !

*Mme 1:* D'accord ! [Euh] on arrive presque à la fin de de, de notre entretien, mais [euh] concernant le-- On va maintenant un petit peu parler, justement, [alors] vraiment de- de, du gaspillage alimentaire.

*Mme 2:* Ouais !

*Mme 1:* De cette- cette- cette idée de- du gaspillage alimentaire. [Euh] et quand je vous dis ça, gaspillage alimentaire, finalement, c'est quoi les mots ou des expressions courtes qui, spontanément, vous viennent à l'esprit ?

*Mme 2:* On parle toujours de fruits et légumes [hein] ? Pas d'autres choses ?

*Mme 1:* On parle, si possible, de fruits et légumes. S'il y a autre chose qui vous vient à l'esprit, n'hésitez pas à parler.

*Mme 2:* [Ben] le premier mot qui me vient, c'est dommage, parce que quand on pense à toutes les personnes qui en auraient besoin et qui avec un tout petit peu de volonté pour en faire quelque chose-- Il y a des grands chefs [hein] maintenant qui [euh]-- Mais ça a été médiatisé [alors] maintenant, tout le monde dit que c'est super. Mais [euh] il y a des gens qui faisaient ça déjà depuis très longtemps dans le Sud [en fait]. Ici, je ne sais pas parce que ça ne fait pas assez longtemps que je suis arrivée pour le dire. Mais dans le Sud, ça fait déjà très, très longtemps que, à la fin des marchés ou [euh] à la sortie des, des inventaires de grandes surfaces, de choses comme ça, il y ait des gens, des associations qui viennent récupérer ou même des particuliers parce que les marchés ici, ça ne se fait pas. Mais [euh] la première fois que je suis arrivée sur un marché en Finistère, à la fin du marché où j'ai demandé : "Il vous resterait des fruits [euh] à confiture, [en fait] ?" [En fait], dans le Sud, on vous les donne. Ici, on vous les vend. Mais je suis restée bête, je ne m'y attendais pas. [rire] Surtout que [bon] [euh] ils vous vendent ça, pas franchement donné.

*Mme 1:* Ouais !

*Mme 2:* Donc [euh] [bon] OK ! Et ce n'est pas du hors gabarit ou-- Non, non [hein] ! C'est des fruits abîmés à l'intérieur et puis vous faites votre tri quoi. [Bon] ! Donc [euh], je me dis dommage, ouais ! Parce qu'on pourrait [euh], vous voyez ? Un exemple concret, factuel. Mes enfants sont à l'école publique, ici. La cantine de l'école, ils n'y vont pas, tant mieux. On a la possibilité de les avoir le midi, ici. Entre mon mari et moi, on jongle tous les deux. Mais il y a plein d'enfants qui n'ont pas le choix et qui mangent à la cantine. [Euh] avant les élections municipales, on avait [euh]--parce qu'avant, on faisait partie de la PE de l'école. On avait amené ce sujet au sein de l'école parce qu'ils viennent de refaire la cantine, les locaux. Et avant qu'ils les refassent, on avait dit : "Est-ce qu'il faudrait pas en profiter ?" Compte tenu que l'école publique, l'école privée est juste à côté et il y a un ehpad au milieu.

*Mme 1:* Ouais !

*Mme 2:* Est-ce qu'on ne pourrait pas en profiter ? Parce qu'en plus, on avait avec nous un parent d'élève qui est cuisinier mais qui faisait partie d'une cuisine centrale avant. Donc, la grande [euh], enfin, en nombre, il sait faire. On a dit pourquoi ne pas en profiter pour prévoir un espace cuisine, ici ? Vous avez que ça, des agriculteurs ici dans le coin, dont deux bio qui sont à l'entrée et à la sortie de la commune. Pourquoi on en profiterait pas pour associer ces gens-là ? Faire un projet de territoire. Et voilà ! La réponse a été, ça nous coûte moins cher de faire venir Sodexo. [Ben] c'est pour ça que je dis dommage parce que si tous les fruits et légumes qui étaient en fin de vie pouvaient être [euh]-- surtout que faut voir les critères de fin de vie [hein]. Moi, j'ai déjà eu des, des récupérations de panier parce que je suis adhérente de deux, deux organismes. Je ne sais pas si vous connaissez Too Good to go ou [euh] comment s'appelle l'autre ? [Euh] Phoenix. Donc, ce sont des paniers à sauver entre guillemets en fin de journée. Mais il faut voir, il y a des fois, vous récupérez des trucs qui sont encore bons pendant deux ou trois semaines quoi. Donc [bon], je comprends pas bien, mais [bon], les grandes surfaces doivent avoir des- des- des directives plus drastiques, sans doute que l'on aurait nous chez nous. Mais [euh], mais on se dit : "Mais pourquoi ils ne font pas ça à grande échelle ? Ça coûterait quoi ?" Il y en a qui le font à Paris maintenant et qui [euh] qui font des repas pour [ben] les personnes sans domicile ou [euh] ou [euh] en faibles revenus. Ou [euh] [ben] c'est juste logique [en fait], mais ça n'existe pas ou trop peu en tout cas.

*Mme 1:* Voilà !

*Mme 2:* [Toux] donc, voilà !

*Mme 1:* OK ! Le mot principal, c'est dommage ?

*Mme 2:* [Ben] Voilà ! Ouais ! C'est dommage et c'est [euh] un peu [euh] colère aussi quoi.

*Mme 1:* Ouais ! Colère aussi.

*Mme 2:* Parce que je-- C'est dommage dans le sens pas normal, [quoi].

*Mme 1:* Mm-hmm !

*Mme 2:* Et après [euh] vous entendez aussi [euh] les pauvres, les agriculteurs qui râlent parce que-- Ou les éleveurs de- de vaches laitières qui râlent parce que eux on leur paye le litre de lait, je dis n'importe quoi, 10 centimes quand vous-- on vous le vend à un euro 20, quoi. Donc [ben], c'est pas très cohérent tout ça, je trouve.

*Mme 1:* Mm-hmm ! Oui, donc, il y avait aussi pas cohérent [diaphonie 00:48:02-00:48:03] comme- comme expression et comme--

*Mme 2:* [Alors] c'est vrai que maintenant ça s'est un peu plus généralisé dans les grandes surfaces. Vous avez de plus en plus de rayons [euh] anti-gaspi, justement.

*Mme 1:* Ouais !

*Mme 2:* Où tout est mélangé. Vous avez du bio, pas bio, machin, mais c'est bien, c'est déjà un progrès. Maintenant, je pense qu'il faudrait que les- les gérants [en fait] de grandes surfaces ou de- d'autres établissements maraichers [en fait]-- Aller un peu plus loin dans la démarche et [euh] se disent de toute façon c'est perdu, donc perdu pour perdu. Soit on en fait don à des gens qui en ont besoin. Soit [ben] on le brade à petit prix, mais ça sera déjà ça de pris. Et ça peut dépanner un restaurant. J'en sais rien moi, enfin, quelqu'un qui, qui aurait le courage de le faire. Moi, je le fais bien pour huit, mais je pense que quand on tient un restaurant, on doit savoir le faire pour 50 ou 100 repas quoi. Bref ! [rire]

*Mme 1:* D'accord !

*Mme 2:* [inaudible 00:49:01-00:49:02]

*Mme 1:* Et du coup, [rire] [euh] finalement, si je vous demandais, à l'aide de vos mots et des mots [hein] que vous venez de citer, de me donner votre définition du gaspillage alimentaire, qu'est-ce que ça donnera ?

*Mme 2:* Ah ! [Ben] [alors], il y a, il y a plusieurs, plusieurs [euh] cas de figure chez moi. Il y a le cas de figure où je n'ai pas assez anticipé. Donc, c'est de ma faute.

*Mme 1:* Mm-hmm !

*Mme 2:* Et [euh] là, c'est déception parce que j'ai- j'ai pas réussi à aller au bout de ma démarche. Mais ça m'arrive peu, heureusement. Soit, malheureusement, le plus souvent, c'est [euh] d'avoir trop fait confiance à- au côté visuel et me rendre compte que finalement, la qualité du produit n'est pas celle que j'escomptais [quoi].

*Mme 1:* D'accord !

*Mme 2:* Voilà ! Et ça c'est, c'est [euh] neuf virgule neuf cas sur dix, c'est ça.

*Mme 1:* D'accord !

*Mme 2:* Malheureusement ! C'est-à-dire que vous pensez avoir acheté un bon produit et vous vous rendez compte que deux jours après, il faut déjà le jeter quoi.

*Mme 1:* D'accord !

*Mme 2:* Malgré le réfrigérateur, enfin, voilà ! Parce que c'est pas de bonne qualité. Ce qui n'arrive jamais ou très rarement quand vous achetez à la ferme. Il n'y a pas photo.

*Mme 1:* [Euh] on a quasiment terminé sur la partie fruits et légumes. Merci beaucoup ! C'était très riche. [rire] Non non, mais vraiment, extrêmement riche et extrêmement intéressant. [Euh] je voulais juste faire un petit-- Enfin, faire un petit point sur la poubelle connectée.

*Mme 2:* Oui !

*Mme 1:* Du coup, donc [euh], vous l'avez utilisé pendant à peu près un mois, je pense.

*Mme 2:* Oui ! La première fois, c'était lui mais voyez.

*Mme 1:* Oui, [ben] voilà c'est ça. Donc effectivement, on est vraiment-- [rire]

*Mme 2:* Nickel !

*Mme 1:* Nickel, voilà ! [Euh] Est-ce que vous avez le sentiment de l'utiliser fréquemment ou au contraire, peu ?

*Mme 2:* Ouais ! Malheureusement, trop fréquemment pour moi, ouais !

*Mme 1:* D'accord !

*Mme 2:* Parce que quand on pense qu'on ne met pas dedans tout ce qui est épluchures régulières, [euh] voilà, et qu'on n'y met que les fruits ou légumes qui sont totalement ou partiellement abîmés avant de les consommer et qui ne devraient pas, entre guillemets, l'être, moi je trouve qu'il y en a quand même trop.

*Mme 1:* Oui !

*Mme 2:* Et j'avais pas conscience de ce trop avant d'avoir ça.

*Mme 1:* D'accord !

*Mme 2:* C'est à force de-- Je me suis dit : "Merde !" Mais c'est vrai que ça fait beaucoup quand même. Et comme je vous disais tout à l'heure, quand on regarde les provenances des produits que l'on met dedans, [ben] je peux vous dire que je vais revoir ma- ma façon d'acheter.

*Mme 1:* Vous pouvez me donner quelques exemples [diaphonie 00:51:41-00:51:42] [rire]

*Mme 2:* [Alors], pour pas les- pour pas les balancer, [hein] Leclerc [rire] est le plus mauvais élève [en fait].

*Mme 1:* D'accord !

*Mme 2:* Parce que, à chaque fois que j'ai eu à jeter quelque chose, ça venait de chez eux.

*Mme 1:* OK !

*Mme 2:* Voilà ! Sachant que je n'ai pas qu'un Leclerc de prédilection [hein], j'en ai trois. Donc, tout dépend [euh] en fonction de l'organisation de la famille. Ça peut être Brest, ici, Lannilis ou le [inaudible 00:52:12-00:52:13] aussi. Et peu importe la provenance, je pense que c'est le système de conservation qui [euh] qui va pas.

*Mme 1:* En termes de- en termes de produits, c'était quel type de produits ?

*Mme 2:* [Alors] ! Ne bougez pas ! [Euh] Premier, c'était des pommes de terre. [Bon] à leur décharge, je les avais achetées depuis un petit moment et elles étaient conservées dans l'entrée. Donc ça, on va dire, [bon]. Ensuite, c'était endives et champignons de Paris. Donc là, pour le coup, très frais. Donc là, il n'y a pas eu de cassure du froid puisqu'ils sont arrivés du magasin. Je les ai mis immédiatement dans le tiroir de mon réfrigérateur. Et dans les deux jours, ils étaient abîmés, au réfrigérateur. Alors que d'aspect, quand je les ai achetés, ils étaient corrects. Là, je me dis : "[Bon], il faut te poser des questions quand même". Ensuite, il y avait des tomates à J plus trois, donc tomates dans l'entrée. Des pommes, au bout de deux semaines, sachant que moi, les pommes, quand j'étais petite, on les gardait, je sais pas combien de mois, dans la cave et elle bougeait pas. Là, [euh] pomme a-- je ne sais plus combien il fait dans l'entrée. Je crois qu'il fait entre 16 et et 19 degrés. Enfin, on avait regardé par curiosité la dernière fois, je crois que c'est ça, entre 16 et 19 degrés. Donc, ce n'est pas non plus 30 quoi. Donc, au bout de deux semaines, il a fallu que j'en jette. Mais c'est peut-être dû aussi au transport, parce qu'une pomme, c'est fragile. C'est comme une agrume, quand il prend un coup, vous le voyez pas forcément à l'achat, mais après le coup, voilà. [Alors], est-ce qu'ils se les envoient pour les stocker ? J'en sais rien. Mais voilà, après mon retrait du sac. Ensuite, ça a été des courgettes parce que Plougoulm n'en produit plus puisque normalement là, on est dans une période où ils changent les cultures. Donc, ça va revenir plus tard dans l'été. Du coup, j'avais acheté mes courgettes chez Leclerc. À J plus trois, il a fallu que j'en jette. Courgettes, Leclerc égal [euh] frigo, c'est pas celles de Plougoulm. Celles de Plougoulm, je les garde dans l'entrée, elles durent je ne sais pas combien de temps. Après re-pommes, c'est les mêmes [hein] ! La fois d'avant et c'est tout. Et à chaque fois, c'est Leclerc !

*Mme 1:* Ouais ! Et du coup, là, ce que je note, c'est que finalement, il n'y a pas forcément un produit plus qu'un autre que--

*Mme 2:* Non ! Non !

*Mme 1:* Enfin je-- C'est peut-être une impression. Peut-être que c'est, non. C'est pas [euh]--

*Mme 2:* C'est tout-- En général, tout ce qui vient de là-bas, fruits et légumes--

*Mme 1:* Donc là, c'est pas un produit plus qu'un autre. C'est finalement un lieu d'achat--

*Mme 2:* Oui, c'est ça !

*Mme 1:* D'accord !

*Mme 2:* OK !

*Mme 1:* [Euh] [Bon] vous m'avez parlé du- du- du caractère [euh] pas forcément [euh] pratique de la poubelle. Donc, ça-- Du coup je le-- Je l'enregistre [euh]--

*Mme 2:* Oui, c'est juste voilà-- Aux changements de sac et au nettoyage que c'est pas forcément [euh] pratique.

*Mme 1:* Par contre, vous vous-- Ça vous est arrivé de vous poser des questions sur, est-ce que je dois le mettre dedans ?

*Mme 2:* Non ! Parce que-- Non, non, parce que j'avais le petit livret et c'est écrit dessus. Il y a un autocollant sur le couvercle. Il suffit de savoir lire [en fait].

*Mme 1:* Oui ! Donc, il n'y a pas des--

*Mme 2:* Et puis, on a eu [euh] séance de sensibilisation. On m'a-- Charlotte m'avait installé le 7 mai, la poubelle, justement, connectée. Et le 7 mai, on a fait une petite formation familiale, même pour les tout petits. D'ailleurs, ils étaient très intéressés. Ça m'a vachement étonné. Ils en ont parlé à l'école et tout.

*Mme 1:* [Ah] c'est intéressant !

*Mme 2:* [Ah] ouais ouais, parce que ça y est, c'est des stars. Ils font l'objet d'une étude. Donc, [rire] ça m'a trop fait rigoler. La maîtresse, elle me dit : "Vous avez une poubelle connectée ?" Je dis : "[ben] oui". [rire] [Alors] du coup, c'est cool parce que j'ai pu lui en parler. Elle dit, c'est génial ! J'en ai pas entendu parler. Ouais, mais c'est normal. Et [euh] du coup, je lui ai expliqué, je lui ai montré le petit livret. Elle me dit : "Mais c'est super bien ça". Et c'est vachement pédagogique parce que aussi ils ont des actions [euh] anti-gaspi, [euh] le tri sélectif, tout ça. Donc, c'est dans la même démarche et ça l'a vachement intéressé. Donc, les tout petits m'ont posé plein de questions : "Justement, maman, est-ce que tu vas mettre les épluchures [euh] au, dans la poubelle-là ?" [Alors] J'ai dit : "Non ! Ça, ça ne rentre pas. Mais pourquoi ? Mais parce que ça ne rentre pas". [rire] Je n'ai pas su expliquer vraiment pourquoi il ne fallait pas. Mais je pense que, voilà, j'ai tourné les choses en disant parce que c'est habituel.

*Mme 1:* Mm-hmm !

*Mme 2:* C'est, de toute façon, des déchets qu'on aurait générés dans tous les cas de figure. Donc, ça n'intéresse pas. Ce qu'on veut savoir, c'est [euh] est-ce qu'il faut traiter les fruits et légumes pour les garder plus longtemps ? Si oui, est-ce que ça a une incidence sur la santé ? Tout ça, enfin-- Ils étaient hyper intéressés. Donc voilà, les ados, il a fallu que j'y revienne deux fois parce qu'ils sont là, mais pas là. Vous leur parlez, mais ça passe à travers. Donc [euh], il y a eu un loupé [hein]. J'ai marqué dans le- dans le truc, je ne sais plus où c'est. Mais il y a-- [Ben] C'était le lendemain [hein] [en fait]. Il y a mon grand à moi, mon fils, qui [euh] a ouvert. [Ah] c'est quoi ce truc ? J'ai dit [inaudible 00:57:28-00:57:29]. Donc [bon], c'est pas grave, je l'ai noté que c'était ça, là. Mais sinon, ça a été.

*Mme 1:* [Ben] OK ! Super ! Moi j'ai pas vu [euh] sur les cumuls puisqu'[en fait] on a accès, donc j'ai été regardée sur la, ce qu'on appelle la timeline. J'ai rien vu de- d'anormal.

*Mme 2:* [Ben] si, [en fait] vous verrez le huit.

*Mme 1:* Le huit.

*Mme 2:* La troisième pesée [en fait].

*Mme 1:* OK !

*Mme 2:* La troisième pesée, c'est une erreur. Il n'y avait rien qui-- de changé [en fait]. C'est juste mon fils qui s'est planté. Il a ouvert pour voir ce qu'il y avait dedans. Cet abruti. [rire]

*Mme 1:* C'est la curiosité.

*Mme 2:* Alors que je lui avais expliqué la veille. [rire] C'est pas grave, il a voulu voir. [Oh] C'est génial ! C'est quoi ? [rire]

*Mme 1:* Déjà, s'il a dit : "C'est génial!" Ca veut dire que [bon], voilà ! [diaphonie 00:58:13-00:58:14]

*Mme 2:* Mais parce que c'est l'effet nouveauté. Voilà ! C'est quoi ce truc ? Donc voilà. Mais sinon, ça, c'est l'utilisation normale. J'ai changé deux ou trois fois le sac, j'ai [inaudible 00:58:24-00:58:25] deux fois.

*Mme 1:* D'accord ! Il y a eu un moment où vous avez eu l'impression de l'utiliser moins ou pas ?

*Mme 2:* Oui, parce qu'il y a un gros-- [Alors], au début, ça a été vachement régulier, je trouve. Il y a le 8, le 10, le 11, le 12, le 18 mai. Après, c'est passé au 31 mai et après plus rien jusqu'au 7 juin. Donc, oui, bizarre ! J'ai pas plus consommé de-- Plus ou moins consommé de fruits et légumes dans cette période.

*Mme 1:* Mais j'sais pas. Vous en avez moins acheté ?

*Mme 2:* Ouais, j'en ai moins acheté [hein].

*Mme 1:* [inaudible 00:59:01-00:59:02].

*Mme 2:* Tant mieux.

*Mme 1:* D'accord, mais bon. [rire] Non mais comme quoi, c'est effectivement je pense quelque chose qui est assez i-irrégulier. [Euh] quand on avait fait nos tests, on l'avait testé. Voici ce que j'avais constaté. Il y avait des périodes où--

*Mme 2:* Après, vous savez, ça dépend peut-être aussi des approvisionneurs [hein] parce que, comme c'est principalement le même [euh] fournisseur [euh], moi, c'est toujours chez Leclerc que j'ai des soucis a priori de gaspillage. [Euh] tout dépend s'ils ont changé de producteur ou peut-être de mode de transport ou de mode de conservation ou de stockage. Je sais pas quoi. Ça influence peut-être aussi [euh] l'arrivée des fruits chez nous [hein] au moment des légumes [hein].

*Mme 1:* Et du coup [euh]- et du coup, ce qui se passe dans- dans nos [euh] nos paniers. [Euhm] moi, je me suis arrivée au bout des questions que j'avais à vous poser. Donc, je voulais savoir si [euh] [euh]-- je vous remercie déjà d'y avoir répondu.

*Mme 2:* Mais je vous en prie ! [rire]

*Mme 1:* Comme détail, voilà une précision. [Euhm] est-ce qu'il y a des choses sur lesquelles vous, vous souhaiteriez revenir ou des choses qu'on n'a pas abordées, vous aviez envie de dire [en fait] sur ce sujet ?

*Mme 2:* Non. Moi, je trouve que c'est très bien ce que vous faites, ce-ce que [euh) l'étude que vous êtes en train de mener parce que [euhm] ça permet aussi aux gens qui- qui le font avec vous, [en fait], de prendre un certain nombre de [euh]-- fin de prendre conscience (en fait] d-des choses [euh) de notre façon de consommer, de notre façon de [euhm]- de nous organiser aussi. Et [euh] on est tellement tous différents que je pense que ça va être hyper riche pour vous après. Bon courage pour trouver [euh] [rire] le lien dans tout ça. Mais [euh) moi, en tout cas, ça m'a fait prendre conscience effectivement de mes habitudes de consommation qui ne sont pas forcément celles que je pensais avoir.

*Mme 1:* D'accord !

*Mme 2:* Finalement, je trouve que j'achète relativement trop souvent chez Leclerc. Donc, ça m'a permis en tout cas de revoir ma [euh) façon de- de consommer tout simplement. Voilà ! Après, j'espère que [euhm] ça va avoir une incidence plus une-- d'un- d'un point de vue plus national auprès des- des fournisseurs de fruits et légumes. Je parle pas forcément de- du pauvre agriculteur qui [euh) trime toute l'année, mais du- de la centrale d'achat qui [euh] vous la revend à-- derrière à un prix qui est pas forcément raison, tu trouves, en fonction de- de ce qu'il propose et [euh] d'inciter les gens à plus [euh] [euh] auto consommer. Après, [bon] tout le monde n'a pas la chance d'avoir un jardin ou- ou un balcon [euh]. Voilà ! Mais quand on voit [euhm]-- moi, je suis en train de-- Ça fait un moment qu'on est en train ici de passer à [hm] à n-notre façon de- de manger. On revient à des- des bases beaucoup plus [euh] saines et anciennes [en fait] que- que cette société de consommation-là qui vous pousse à acheter [euh], je sais pas des fraises au mois de février ou je sais pas quoi. [Euh] vous voyez, on a mangé de l'ananas hier. Je me suis gardé le- la houppe (en fait] de l'ananas pour pouvoir le replanter. On fait des expériences maintenant avec les enfants. [Euhm] on va essayer de le replanter parce que j'ai vu que sur Internet, il y avait tout un tas de- de légumes [hein]. [Alors], il y en a que je faisais déjà. Le céleri, vous pouvez le replanter [euh]. Vous avez un pied de céléri. [Euh] on a gardé des [hm]- des- comment ils s'appellent- des graines de- de fraises pour pouvoir ravoir nos semis l'année prochaine. Enfin voilà, on essaye de repenser un petit peu tout ça pour [euh] limiter [en fait] notre consommation aux choses vraiment [euh] à la fois nécessaires et à la fois saines. Voilà ! On revient aux- aux légumes secs [euh], aux fruits secs [euh], des choses comme ça, quoi,

*Mme 1:* OK !

*Mme 2:* aux choses plus- plus simples.

*Mme 1:* Plus simples [en fait].

*Mme 2:* Ouais,

*Mme 1:* Ouais oauis.

*Mme 2:* moins trafiquées.

*Mme 2:* Tout en sachant qu'on a quand même [euh] des- des enfants qui vivent [euh] à l'époque d'aujourd'hui et qui sont très, très, très influencés [euh] et influençables par les lobbies et- et compagnie. Donc [euh], [bon] j'en suis pas encore à cultiver mes céréales pour leur faire les céréales le matin. [rire] [Bon], je pense que pendant un moment encore, on aura les céréales en boîte [hein]. Mais [euh] [bon], voilà ! On essaye quand même de limiter, ouais.

*Mme 1:* De limiter là.

*Mme 2:* Faire attention, ouais.

*Mme 1:* D'accord ! On va écouter [inaudible 01:03:25-01:03:26] impeccable. [Euhm] ce que je vous propose, c'est que je vais arrêter l'enregistrement.

*Mme 2:* Oui, bien sûr.

*Mme 1:* On va [euh] passer aux pérations [diaphonie 01:03:33- 01:03:34] prélèvements.

**Household F29, Interview 2**

00:00:00
*Mme. [Prénom de l’intervieweuse]:* [inaudible 00:00:00-00:00:04] Du coup, on a pas mal de matériel pour ce petit entretien. En vrai, je vais mettre quatre illustrations.

00:00:16
*Mme. 2:* Oui.

00:00:16
*Mme. [Prénom de l’intervieweuse]:* Je vais vous demander d'en choisir une, de la retourner et de me dire spontanément les [euh] trois premiers mots ou expressions qui vous viennent à l'esprit.

00:00:24
*Mme. 2:* D'accord. Alors les trois premiers mots qui me viennent à l'esprit, c'est-- Je vous montre d'abord.

00:00:37
*Mme. [Prénom de l’intervieweuse]:* Ouais.

00:00:38
*Mme. 2:* C'est [euh] marché local. Mm-hmm, local, Mm-hmm quand on regarde des étiquettes pas trop, mais bon, on va dire marché local et saison.

00:01:01
*Mme. [Prénom de l’intervieweuse]:* Ok. Qu'est-ce qui vous fait [euh] penser au marché ?

00:01:06
*Mme. 2:* Bon, les dispositions en fait, [euh] c'est un peu [euh] les- les étales qu'on retrouve [euh] en extérieur. Il y a des fleurs, il y a [euh] un stand qui pourrait très bien être [euh] l'application de ce que vous achetez-là. Vous pouvez le- le- le consommer après qu'il les ait modifié ou apprêté. [euh] Mm-hmm, mais c'est un marché couvert, quoi où- où l'intérieur d'un restaurant, un truc comme ça, mais non, les fleurs me font dire que c'est plutôt un marché.

00:01:39
*Mme. [Prénom de l’intervieweuse]:* Ok.

00:01:43
*Mme. 2:* Mais c'est pas chez nous.

00:01:44
*Mme. [Prénom de l’intervieweuse]:* Pas chez vous ?

00:01:50
*Mme. 2:* Non.

00:01:50
*Mme. [Prénom de l’intervieweuse]:* Vous avez dit local aussi ?

00:01:52
*Mme. 2:* Oui, mais je sais pas pourquoi j'ai dit local parce que… [rire] Finalement quand on regarde, il y a ananas. C'est pas d'ici, le raisin d'Italie, c'est pas ici. [euh] Les grenades, je ne crois pas qu'il en pousse en Bretagne. [rire]

00:02:06
*Mme. [Prénom de l’intervieweuse]:* Pas encore.

00:02:08
*Mme. 2:* Mais je sais pas quand j'ai vu tout ça, en fait, ça me semblait plutôt cohérent que dans le lieu où ça se trouve, on trouve tout ça, tout ce qui est fruits exotiques, [euh] bananes, ouais, les grenades, les châtaignes. C'est ce qui m'a fait penser à la saison, en fait. Je pense que c'est des châtaignes, ça. On est encore fin de l'automne tout ça. Il y a eu les- les châtaignes, il n'y a pas longtemps.

00:02:30
*Mme. [Prénom de l’intervieweuse]:* Ouais.

00:02:33
*Mme. 2:* Mais après, ça vaut guère mieux. Il y a là, c'est des fruits de la passion. Là, c'est du citron vert, enfin tout ça. Ça se trouve quand même dans- dans une même région quoi. Donc comment s'appelle les gros pamplemousses-là et mon truc là, comment on appelle ça ? Les trucs chinois, là, les énormes pamplemousses. Bah tout ça, ça peut se trouver en tout cas [euh] au même endroit.

00:02:54
*Mme. [Prénom de l’intervieweuse]:* Ouais, c'est local, mais pas- pas de la Bretagne.

00:02:58
*Mme. 2:* Voilà, je pense. Je pense que--

00:02:59
*M. [Prénom du conjoint]:* C'est- c'est du local de l'importation.

00:03:01
*Mme. [Prénom de l’intervieweuse]:* C'est du local d'importation, oui.

00:03:06
*Mme. 2:* J'ai dit local parce que j'ai pensé au marché. Donc, moi, j'associe le marché, en fait, à ce qui est local. Mais- Mm-hmm mais pas forcément. [euh] Maintenant qu'on regarde avec précision la photo. Voilà, on se rend compte que c'est pas d'ici.

00:03:20
*Mme. [Prénom de l’intervieweuse]:* Oui.

00:03:23
*Mme. 2:* Après le gars, il est quand même vachement bien apprêté et tout ça. Et bah, ouais, c'est ça, c'est le- le must du fruits et légumes quand même hein. [rire]

00:03:31
*Mme. [Prénom de l’intervieweuse]:* Ouais, alors la photo ne fonctionne pas trop par rapport à ce que [euh]-- Normalement, vous avez raison, c'est pas local, c'est même mieux. C'est même pas quand on regarde l'intérieur des étiquettes.

00:03:41
*Mme. 2:* C'est en anglais ?

00:03:42
*Mme. [Prénom de l’intervieweuse]:* C'est vrai, c'est pas français.

00:03:43
*M. [Prénom du conjoint]:* C'est ce que j'ai regardé.

00:03:44
*Mme. [Prénom de l’intervieweuse]:* Parce qu'on voulait prendre des photos libres de droits etc. Et à l'origine, l'idée c'est plutôt d'évoquer un lieu, [euh] un primeur ou un magasin spécialisé de fruits et légumes. Mais en plus [euh] vous êtes la première bien marché et remarquer le nœud papillon et tout ça donc la photo. Je pense qu'il faut qu'on en trouve une autre pour évoquer les primeurs parce que là on est pas.

00:04:06
*M. [Prénom du conjoint]:* Vous auriez dit marché ou hall, mais c'est pareil.

00:04:08
*Mme. 2:* Ouais mais le marché couvert, c'est ça j'ai dit.

00:04:10
*Mme. [Prénom de l’intervieweuse]:* Ouais, c'est ça.

00:04:12
*M. [Prénom du conjoint]:* Ou alors- ou alors oui, un magasin de standing quoi. Standing du fruits et légumes c'est vrai que l'homme derrière lui c'est rangé.

00:04:19
*Mme. 2:* Ouais, mais tu vois, il a le nœud papillon, mais regarde à droite, ça a l'air vachement moins bien rangé. Il y a toute la partie fleurs. [euh] Enfin, ça fait un peu tu te serres et puis tu- tu pars avec.

00:04:30
*Mme. [Prénom de l’intervieweuse]:* Quoi ?

00:04:32
*Mme. 2:* Regarde là, c'est vachement moins bien rangé déjà.

00:04:36
*M. [Prénom du conjoint]:* Oh oui, c'est fait !

00:04:37
*Mme. 2:* Et puis moi, personnellement.--

00:04:39
*M. [Prénom du conjoint]:* Ça fait grossiste un peu.

00:04:40
*Mme. 2:* Ça fait grossiste. Voilà si moi, je vais chez un primeur. J'attends pas d'y acheter des fleurs.

00:04:46
*Mme. [Prénom de l’intervieweuse]:* Ouais.

00:04:46
*Mme. 2:* Voilà, là, ça fait plus hall, effectivement, marché couvert.

00:04:56
*Mme. [Prénom de l’intervieweuse]:* Oui, c'est difficilement, je trouve la photo.

00:04:59
*Mme. 2:* Désolé.

00:04:59
*Mme. [Prénom de l’intervieweuse]:* Mais bon, c'est parfait là. [rire] Et [euh] alors si on imagine que c'est un primeur, admettons [euh] est-ce que c'est un lieu [euh] dans lequel vous allez de temps en temps. Pas de primeurs ou de magasins spécialisés [euh].

00:05:17
*Mme. 2:* Pour plusieurs raisons. Déjà parce que moi, j'ai ce que [euh] ce dont j'ai besoin à la ferme.

00:05:22
*Mme. [Prénom de l’intervieweuse]:* OK.

00:05:22
*Mme. 2:* Et ensuite parce que ce que je pourrais être amené à acheter [euh] dans un magasin spécialisé sont les agrumes. Et elles m'arrivent directement du jardin de mes parents. Donc [euh] pas besoin d'en- d'en acheter ailleurs.

00:05:33
*Mme. [Prénom de l’intervieweuse]:* Ok. Alors du coup, si c'est quelque chose de très bon, vous pouvez. [euh] C'est pas un endroit dans lequel vous allez. Mais sinon, qu'est-ce que vous en pensez ? Nom de ce type de magasin. Est-ce que-- Qu'est-ce que vous pensez des lieux et- [euh] et des produits qui y sont souvent proposés ?

00:06:07
*Mme. 2:* Mais moi, ce qui m'embête en fait chez un primeur, c'est que justement, [euh] Mm-hmm, il y a parfois une incohérence entre l'idée de départ qui est de proposer des produits de qualité. Principalement locaux, [euh] à des prix abordables et la réalité sur le- sur le- sur le tas dans la région. Voilà. Mm-hmm. Alors moi, je ne vais plus chez les primeurs depuis que je suis en Bretagne parce que honnêtement, j'en vois pas l'intérêt. Mais j'ai eu fait ça à Paris et que quand j'habitais Paris, parce que pour manger de façon un peu équilibrée [euh] ben, voilà, vous n'avez pas trop le choix. Et pis, il y en a plein sur tout.

00:06:49
*M. [Prénom du conjoint]:* C'est ça. C'est exactement ce que j'allais dire. Le truc de faire d'habiter à Landéda, le primeur, moi, ce que je vois là aussi, ce serait à Lambézellec quoi.

00:06:55
*Mme. 2:* Non, il y a Saint-Renan.

00:06:57
*M. [Prénom du conjoint]:* La Maison du théâtre là, il y en a qui travaillaient à la Maison du théâtre. Oui, j'allais prendre le temps, mais il y a le--

00:07:03
*Mme. 2:* À Paris, il y en a plein en fait-- [diaphonie 00:07:04-00:07:05] Voilà, c'est ça. Il y en a vachement plus [euh] sur la région Parisienne en tout cas. Mais par contre, au niveau des prix, ils sont-- ils se valent pas tous. C'est-à-dire que vous avez le primeur [euh] comme lui, il est avec son nœud papillon. Vous allez payer ultra cher parce que vous êtes dans un quartier de Paris qui est ultra cher. Et vous avez aussi [euh] ben, le petit guest du coin que tout le monde connaît, qui est ouvert, limite H 24 et qui propose des trucs super aussi, mais [euh] cinq fois moins chers. Donc en fait, ça dépend.

00:07:37
*Mme. [Prénom de l’intervieweuse]:* Ok, donc pour vous, le primeur, c'est [euh] d'accord si c'est un commerce de proximité [euh], il n'y a pas d'intérêt.

00:07:46
*Mme. 2:* Non.

00:07:46
*Mme. [Prénom de l’intervieweuse]:* On va mettre en place. Il y en a bien à Landéda. Potentiellement, vous pourrez y aller ou non.

00:07:52
*Mme. 2:* Non, non parce que Ulti fait déjà ce rôle-là. La supérette d'ici a déjà ce rôle un peu de primeurs parce que ils choisissent des producteurs locaux ici. Et [euh] il n'y a pas que ça. Mais [euh] en tout cas en fruits et légumes, si vous ne voulez acheter que du local, vous pouvez.

00:08:11
*Mme. [Prénom de l’intervieweuse]:* Ok.

00:08:11
*Mme. 2:* Il y a pas mal d'autres.

00:08:13
*M. [Prénom du conjoint]:* Il y à tous les mardis aussi.

00:08:14
*Mme. 2:* Il y a le marché bio qui vient tous les mardis matin. Donc franchement, celui qui ouvre un primeur ici, je ne suis pas sûr qu'il fasse [euh] fortune. Et puis, vous avez des fermes de partout hein, c'est surtout ça. C'est qu'en fait [euh] le primeur, vous avez encore un intermédiaire. Vous allez à la ferme, vous achetez directement du producteur au consommateur. Et moi, [euh] enfin, j'y crois beaucoup à ça parce que je préfère aider un agriculteur qui- [euh] qui se lance dans cette activité ou qui a besoin de vivre de ça en dehors de ce qu'il vend aux coopératives, etc. Enfin ça me parle plus si vous voulez que un primeur qui- qui déjà doit payer son loyer [euh] pour son magasin, qui doit [euh] faire venir la marchandise, enfin voilà, après ça dépend, je vous dit si en milieu urbain, ça s'entend carrément. Parce que là, vous êtes loin des fermes, vous êtes loin de des producteurs, on va dire. Mais ici, honnêtement ou alors c'est une volonté politique de vouloir faire augmenter le cadre de vie des gens [euh], de vouloir que les- les- les gens qui s'installent ici soient des gens à au moins un grand.

00:09:25
*Mme. [Prénom de l’intervieweuse]:* Oui, donc vous, vos expériences, elles sont plus liées à la ferme et vous allez directement à la ferme. Ok, alors on va prendre une autre photo, vous allez faire le même petit exercice. Et vous, les trois premiers mots qui vous viennent…

00:09:50
*Mme. 2:* Grande surface.

00:09:52
*Mme. [Prénom de l’intervieweuse]:* Ouais.

00:09:53
*Mme. 2:* Quantité, diversité.

00:10:00
*Mme. [Prénom de l’intervieweuse]:* Alors est-ce que vous pouvez me dire pourquoi vous avez pensé à ces- ces mots-là, pis que--

00:10:04
*Mme. 2:* On voit que c'est une grande surface, parce qu'il y a plusieurs types de rayons différents. [euh] Vu le volume ça ne peut pas être un petit produit-- enfin un petit [euh] primeur justement parce que sinon ben v'là le- [rire] la quantité de gaspillage, je suppose. Et [euh] parce que Mm-hmm on voit qu'on peut trouver, par exemple, plusieurs types d'un même fruit ou d'un même légume. Ce qui, chez un primeur, serait vachement moins [euh] important, en fait.

00:10:39
*Mme. [Prénom de l’intervieweuse]:* Ouais.

00:10:41
*Mme. 2:* Et surtout [euh] ben, ouais tous les frigos là qui sont- [euh] ils sont énormes. En fait il y en a beaucoup. Voilà.

00:10:52
*Mme. [Prénom de l’intervieweuse]:* Donc, c'est la quantité [euh] des produits et le- et les nombres de variétés de chaque produit qui vous font penser que c'est [euh] une grande surface.

00:11:01
*Mme. 2:* Oui, la façon dont c'est rangé aussi.

00:11:04
*Mme. [Prénom de l’intervieweuse]:* Ouais.

00:11:05
*Mme. 2:* Enfin, c'est surtout les volumes qui me font dire que c'est une bonne chose.

00:11:09
*Mme. [Prénom de l’intervieweuse]:* Ouais. Et vous en pensez quoi du coût de ce lieu ?

00:11:16
*Mme. 2:* Ben quelque part, heureusement qu'ils existent parce que [euh] si tout le monde devait- [euh] devait-- Alors il y a plusieurs raisons. D'abord, il faut que ce soit accessible à tous. Le fait que nous, on achète à la ferme, c'est un choix. On pourrait très bien l'acheter qu'en grandes surfaces. D'ailleurs, il m'arrive d'acheter des choses en grandes surfaces, notamment les fruits quand je trouve pas en local hyper de proximité. Mais [euh] il m'arrive d'aller [euh] moi en grandes surfaces, voilà aussi. Et heureusement qu'elle existe parce que au niveau des prix, il ne faut pas se leurrer, c'est quand même pas le même- même prix qu'un primeur par exemple. Et vous avez la variété justement.

00:12:02
*Mme. [Prénom de l’intervieweuse]:* Ouais

00:12:03
*Mme. 2:* Donc [euh] Même si c'est pas écolo. Ben on peut quand même trouver [euh]. Faire des tomates en hiver [euh], des bananes toute l'année. [euh] Enfin, voilà. Après, c'est un choix qu'on fait d'acheter ou non. Mais même si les fruits et légumes qui ne sont pas de saison sont plus chers que lorsqu'ils sont de saison, ils sont quand même abordables en grande surface. Par contre, il ne garde pas.

00:12:35
*Mme. [Prénom de l’intervieweuse]:* Vous trouvez que les fruits et légumes que vous achetez hors saison en grandes surfaces restent abordables, même si c'est un peu plus cher. Mais par contre, ils se conservent mal.

00:12:45
*Mme. 2:* Mais tous, même les fruits de saison, tout ce que vous achetez en grande surface. Moi, je sais ce que l'étude a révélé chez nous en tout cas.

00:12:51
*Mme. [Prénom de l’intervieweuse]:* Ouais.

00:12:52
*Mme. 2:* C'est que c'est une catastrophe en fait. [euh] Les seuls fruits et légumes que j'ai jetés, il y en a aucun qui venaient de la ferme. En revanche, ils venaient tous d'une grande surface. Je pense que leur façon de conserver et de [euh] ouais de conserver surtout [euh] ces aliments-là, ces produits-là font que lorsque vous arrivez chez vous, si vous ne les conservez pas dans la même- dans les mêmes conditions, alors que vous vous les voyez sous cette apparence-là, vous les voyez pas dans les frigos.

00:13:27
*Mme. [Prénom de l’intervieweuse]:* Ouais.

00:13:28
*Mme. 2:* Mais vous savez qu'ils sortent de frigo, en fait. Parce que si vous ne les remettez pas, vous immédiatement dans les frigos le lendemain déjà, des fois, vous voyez le fruit qui s'altèrent corps.

00:13:40
*Mme. [Prénom de l’intervieweuse]:* Ouais, alors que les-- Donc, c'est-- vous changez vos méthodes de conservation selon la provenance de vos fruits et légumes. S'ils viennent de la ferme, vous les mettez pas forcément.

00:13:50
*Mme. 2:* Je les mets jamais au frais. Il y a pas besoin, en fait.

00:13:54
*Mme. [Prénom de l’intervieweuse]:* Oui.

00:13:55
*Mme. 2:* Ils ont eu l'habitude d'être stockés déjà [euh] quand vous allez les chercher à la ferme, ils viennent d'être cueillis. Donc, ils n'ont déjà pas passé cette étape de conservation au froid ou je sais pas quoi. Au mieux, ils sont dans un hangar, donc c'est un hangar qui est ventilé mais qui est à température ambiante, en fait et quand ils arrivent chez vous, c'est rebelote. C'est-à-dire que vous, les reconservez à température ambiante. Ils n'ont pas- ils n'ont pas subi de modification de température. En tout cas, pas significative pour altérer le- le légume. Et il se garde vachement longtemps, en fait.

00:14:27
*M. [Prénom du conjoint]:* Dans une pièce non chauffée.

00:14:30
*Mme. 2:* Dans une pièce non chauffée, bien sûr, ouais

00:14:34
*Mme. [Prénom de l’intervieweuse]:* Oui, c'est température ambiante--

00:14:35
*Mme. 2:* Si la température ambiante il mûrisse forcément, il va falloir le plus vite.

00:14:39
*Mme. [Prénom de l’intervieweuse]:* Ok, et du coup donc, à part cette mauvaise conservation, qu'est-ce que vous pensez des produits que vous vous trouvez [euh] en grande surface ?

00:14:53
*Mme. 2:* Ben finalement, moi j'en suis plutôt déçue parce que des fois [euh] on se dit bon, tiens. Mes enfants adorent les myrtilles, par exemple. Myrtilles en ce moment d'en trouver plein, mais ils viennent pas d'ici. Elles viennent du Chili, elles viennent de je ne sais pas où donc on peut dire, on fait l'effort, de- de mettre un peu un mouchoir sur notre côté un peu écolo et se dire, bon, on veut faire plaisir aux enfants, donc on achète, on les achète chers.

00:15:21
*Mme. [Prénom de l’intervieweuse]:* Ouais.

00:15:22
*Mme. 2:* Voilà et [euh] sous 24 heures, il faut les avoir mangé en fait, même en les gardant au frigo.

00:15:29
*Mme. [Prénom de l’intervieweuse]:* Ok.

00:15:31
*Mme. 2:* Donc [euh] c'est quand même bon.

00:15:33
*M. [Prénom du conjoint]:* De toutes les façons la chambre froide, tue le goût enfin, moi, j'aime autant aimer les légumes depuis qu'on prend les légumes de la ferme.

00:15:38
*Mme. 2:* Oui, c'est vrai.

00:15:39
*M. [Prénom du conjoint]:* Mais en termes de goût, oui, tu sais ça n'a rien à voir.

00:15:44
*Mme. [Prénom de l’intervieweuse]:* Vous trouvez une différence.

00:15:45
*Mme. 2:* Ça par contre, c'est important, oui, pour vous, parce que moi, j'ai été élevé à la campagne, donc mes parents m'ont toujours fait leurs légumes.

00:15:51
*Mme. [Prénom de l’intervieweuse]:* Ouais.

00:15:51
*Mme. 2:* Et c'est très rare que mes parents achètent [euh] leurs légumes autrement que ceux qu'ils cultivent, mais ça arrive. Alors que toi, par contre, t'a toujours [euh] été élevé par ça, quoi, les grandes surfaces, les trucs comme ça. Et ils redécouvrent aujourd'hui le vrai goût du légume. [rire]

00:16:06
*M. [Prénom du conjoint]:* Enfin [euh] c'est ça justement. Après avoir- Après, les goûts évoluent aussi hein quand on est enfant. Oui, en même temps, même il y a dix ans. On est allé nous faire manger les légumes.

00:16:16
*Mme. 2:* Et la façon de les cuisiner peut-être aussi.

00:16:18
*M. [Prénom du conjoint]:* Oui, la façon de-- Mais non, mais même enfin ça se voit, les enfants mangent pas les courgettes de grandes surfaces.

00:16:24
*Mme. 2:* Oui, les tomates surtout.

00:16:25
*M. [Prénom du conjoint]:* Les tomates et alors [euh] Les tomates, c'est le mode de conservation aussi. Parce que même les tomates hors sol, quand elle est cueillie et mangée tout de suite à la bouche, une tomate à la fois quand elle est passée par la chambre froide, ça y est, c'est fini.

00:16:38
*Mme. 2:* Mais en général, les légumes [euh] bretons achetés en grande surface [euh] pas terrible.

00:16:44
*Mme. [Prénom de l’intervieweuse]:* Ok.

00:16:47
*M. [Prénom du conjoint]:* Mais si les chambres froides, les chambres froides à la cueillette ils mettent dans les chambres froides après les amènent dans des camions réfrigérés, dans les centrales ou les chambres froides et les remette dans un autre camion réfrigéré pour arriver dans les chambres froides dans le magasin et après ?

00:17:01
*Mme. 2:* Oui, mais en plus, il y a le- le facteur lorsqu'il les cueille, ça dépend du légume ou du fruit. Il ne les cueille pas à maturité, pour qu'il puisse encore avoir le temps de mûrir chez vous, en fait. Mais [euh] c'est ce qui fait que ça s'altère en fait. Au niveau des bananes, les choses comme ça. Vous voyez la différence entre un fruit [euh] acheté [euh] souvent à faire après chambre froide ou [euh] un fruit que vous achetez chez le primeur par exemple, vos bananes vont durer plus longtemps.

00:17:32
*Mme. [Prénom de l’intervieweuse]:* Donc c'est plus elles viennent de fermes, plus elles ont plus d'intermédiaires, plus elles sont passées dans la chambre froide quoi.

00:17:39
*Mme. 2:* Mais c'est vrai qu'au niveau de la saveur de- de l'aliment ça n'a rien à voir, c'est vrai. Les fraises, tout.

00:17:49
*Mme. [Prénom de l’intervieweuse]:* Donc seuls les fruits ou les légumes.

00:17:50
*Mme. 2:* Ouais.

00:17:52
*M. [Prénom du conjoint]:* Ce sont les fruits ou légumes, oui, oui.

00:17:53
*Mme. 2:* Le plus flagrant- le plus flagrant ici que j'ai remarqué, c'est effectivement les courgettes et les tomates. Pour les légumes qui perdent carrément leur goût, c'est dégueulasse. Et [euh] au niveau des fruits, c'est les fraises qui sont passées au frigo. Elles ont plus de goût et oui, les pommes aussi, je trouve que les saveurs des pommes [euh] renferment, personnellement- personnellement, je savais même pas que vous pouvez mettre des- des pommes au frigo. Donc [euh] voilà. Mais enfin, chez nous, on les conserve pas comme ça, mais c'est vrai que il y a une différence.

00:18:28
*Mme. [Prénom de l’intervieweuse]:* Donc globalement et par rapport aux produits que vous avez directement à la ferme, voir cette tendance chez le primeur, vous dites qu'ils sont peut-être moins pire que les grandes surfaces. Il y aurait moins de saveurs. Ils se conservent moins que la ferme. Et [euh] vous avez parlé de la déception des myrtilles. Vous avez dit qu'elles étaient chères. Deux elles venaient d'ailleurs. Donc au moment des caisses. Est-ce que, selon vous, il y a- il y a une différence dans le fait de perdre la myrtilles avec tous ces éléments là par rapport à d'autres produits ou par le fait qu'elle est meilleure que ce plus cher. Est-ce que sur le moment de la perte de la jetée, si jamais elle s'est altérée, il y a-- la conséquence est plus lourde ou- ou pas ?

00:19:17
*Mme. 2:* Non, c'est pareil. Mais [euh]- mais [euh] ce qui est embêtant, c'est [euh] oui d'avoir mis de côté l'aspect un peu écologique en se disant ouais, bon ok, elle n'est pas d'ici, bon, carbone machin, hein. En plus, on les paye plus cher effectivement pour qu'elles durent pas. Voilà c'est, mais ça m'aurait fait le même effet si ça avait été un fruit local [euh] acheté au prix, ben moi, j'aime pas le jeter de toute façon en général. Donc oui, je vais avoir encore plus ce sentiment parce que- [euh] parce que j'aurais fait l'impasse là-dessus sur mes valeurs. Mais- [euh] Mais ça m'embêterait pareil avec un fruit d'ici quoi.

00:19:54
*M. [Prénom du conjoint]:* En plus sur les- sur les valeurs, je trouve qu'on fait attention.

00:19:56
*Mme. 2:* Oui, oui, on fait vachement gaffe.

00:19:58
*M. [Prénom du conjoint]:* Mais [euh] c'est complètement différent d'acheter [euh] un produit d'ici. Il peut y en avoir en France, mais quand on veut de l'avocat ou je sais pas quoi, on sait qu'il y en a pas en France.

00:20:08
*Mme. 2:* Si, il y en a. En Bretagne, en plus, il y en a plein, mais ils les vendent pas.

00:20:11
*M. [Prénom du conjoint]:* Voilà, donc, ils vont commencer.--

00:20:12
*Mme. [Prénom de l’intervieweuse]:* C'est vrai, ça va.

00:20:13
*M. [Prénom du conjoint]:* Du kiwi- du kiwi aussi. Il va y en avoir plus jusqu'à présent, il y en avait pas trop. Donc France, si on en voulait, on était bien obligé de prendre le kiwi qui venait de Nouvelle-Zélande de chauffe après. Par contre les tomates d'Espagne, c'est directement un geste que j'ai toi.

00:20:26
*Mme. 2:* Mais non.

00:20:27
*Mme. [Prénom de l’intervieweuse]:* Oui, ce qui-- est-ce qui peut être produit en France--

00:20:28
*M. [Prénom du conjoint]:* C'est ça- C'est ça- C'est l'écologie c'est- c'est faut- faut être parmi deux poids, deux mesures. C'est quand on peut acheter [euh] quand même du produit qui est le local, oui, là, il y a un impact écologique qui est plus grand que quand de toute façon, enfin si c'est un produit qui ne pousse pas ici [euh].

00:20:41
*Mme. 2:* Mais c'est vrai que c'est un véritable choix parce que le produit local, vous le payez vachement plus cher souvent que celui qui vient d'Espagne ou du Maroc ou de je ne sais pas où, hein. Il faut en être conscient aussi [euh]. Cet été, on l'a vérifié [euh]. Par exemple, pour les melons, oui, les melons, genre melons de Cavaillon, la lys, voilà orange à l'intérieur là. Eh bien [euh], les melons d'ici étaient deux fois plus chers, même sur les marchés que ceux qui venaient du Maroc qui étaient vachement plus gros et que vous- vous payez 1,50 € quoi.

00:21:14
*Mme. [Prénom de l’intervieweuse]:* Oui.

00:21:16
*Mme. 2:* Donc ça incite pas les gens non plus à faire cette démarche parce que on en est tous au même point hein, se dire que ben les fins de mois parfois sont compliquées [euh] c'est un budget. Et les cours, ça, nous on est huit. Donc si on fait pas gaffe, on est vite débordé avec ça. Donc il y a des choix parfois qu'on fait, mais [euh] par défaut, pas par conviction.

00:21:44
*M. [Prénom du conjoint]:* Par contre, des fraises d'Espagne, on en a jamais annoncé qu'on n'a aucun plaisir à manger des fraises d'Espagne.

00:21:49
*Mme. 2:* Ah ! Non, on achète des oranges sous l'Espagne.

00:21:52
*M. [Prénom du conjoint]:* Oui.

00:21:53
*Mme. 2:* Mais elles ne sont pas mauvaises, les oranges espagnoles.

00:21:57
*Mme. [Prénom de l’intervieweuse]:* Donc vous pouvez faire des entorses [euh]

00:21:59
*Mme. 2:* Oui.

00:22:00
*Mme. [Prénom de l’intervieweuse]:* Mais à condition que vous vous y retrouver dans le désir, le budget et le plaisir de- [euh] de manger.

00:22:06
*Mme. 2:* C'est ça.

00:22:09
*Mme. [Prénom de l’intervieweuse]:* Mais dès que vous pouvez, vous préférez [euh] manger les locales. Parce que c'est meilleur et en plus pour des valeurs écologiques.

00:22:17
*Mme. 2:* C'est ça.

00:22:19
*Mme. [Prénom de l’intervieweuse]:* Ok. et [euh] quand vous vous rendez en grande surface--

00:22:24
*Mme. 2:* Oui.

00:22:25
*Mme. [Prénom de l’intervieweuse]:* [euh] Comment vous vous sentez globalement, parce que c'est un lieu dans lequel vous aimez aller faire vos courses ou pas, qu'est-ce que [euh].

00:22:30
*Mme. 2:* Ah ben par nécessité, nous, on ne va que en grandes surfaces pour nos courses hein [rire]

00:22:34
*Mme. [Prénom de l’intervieweuse]:* Oui.

00:22:37
*Mme. 2:* On achète tout là bas, [euh] au drive ou- ou sur place. Mais, Mm-hmm tout ce qui est produit [euh] hygiène enfin tout ! On achète tout en grandes surfaces, nous.

00:22:52
*M. [Prénom du conjoint]:* On a essayé le marché de la limite si par exemple le marché, c'est un marché qui est cher. Donc j'ai Plouguerneau un peu moins cher. Mais bon, j'ai Plouguerneau, vous y allez tous les jeudis matins, c'est plus long. Mais [euh] c'est ça aussi. Je pense qu'à Brest, si on était à Brest, on y arrive souvent à un marché.

00:23:06
*Mme. [Prénom de l’intervieweuse]:* Ouais.

00:23:07
*M. [Prénom du conjoint]:* Ouais, le Saint-Martin, on va les…

00:23:10
*Mme. 2:* Après, il y a un marché qui semble être bien, c'est celui de Saint-Renan. Mais ça nous fait vachement moins pour y aller. Donc du coup, voyez ce que vous retrouvez sur le prix de l'étal. Vous le retrouvez pas parce que vous avez fait [euh] votre déplacement [euh] avec licence. C'est- C'est nul. Voilà. Là, le- le fonctionnement de la ferme, ça nous convient bien parce que j'y vais une fois par mois.

00:23:31
*Mme. [Prénom de l’intervieweuse]:* Ok.

00:23:32
*Mme. 2:* J'y vais une fois par mois, je prends un gros. Je groupe les commandes aussi parce que j'ai nos voisins, nos amis qui en profitent aussi. Du coup, eux, les fermiers, ça les arrange parce qu'ils ont de plus grosses commandes. Moi, ça m'arrange parce que de toute façon, j'y vais pour moi. Donc [euh] ça me dérange pas de les amener après pour eux. Ils sont ici, donc ils viennent les chercher après ici et tout le monde s'y retrouve parce que on prend des conditionnements qui nous permettent de tenir vraiment un mois quoi.

00:24:02
*M. [Prénom du conjoint]:* Ça fait un seul trajet.

00:24:03
*Mme. 2:* Et ça fait un seul trajet pour Montréal.

00:24:06
*Mme. [Prénom de l’intervieweuse]:* Ouais Mm-hmm et [euh], quand même, c'est des quantités assez [euh] importantes. Si vous êtes huit et [euh] une fois par mois et vous ne perdez pas de fruits et légumes ?

00:24:20
*Mme. 2:* Non, quasiment pas.

00:24:22
*Mme. [Prénom de l’intervieweuse]:* Oui.

00:24:22
*M. [Prénom du conjoint]:* Vraiment quasiment pas.

00:24:23
*Mme. 2:* [diaphonie 00:24:23-00:24:24] Ça peut arriver, c'est ça. C'est ce que je disais.

00:24:31
*M. [Prénom du conjoint]:* C'est ça. Rien que les légumes achetés à la ferme.

00:24:33
*Mme. [Prénom de l’intervieweuse]:* Oui, oui.

00:24:34
*Mme. [Prénom de l’intervieweuse]:* Il n'y a pas de- de on n'a pas encore fait pourtant, il pourrait y avoir des pommes, il y en a à Milizac, etc. On n'a pas encore fait ça.

00:24:40
*Mme. 2:* Ouais, le- le verger, la balade.--

00:24:42
*M. [Prénom du conjoint]:* Il y a le verger, pourquoi pas ? On les fait y faire un fait pour l'instant, mais, je pense qu'on conserverait bien aussi un bon comme les poires. Ce n'est pas ce qui se conserve le moins bien, mais.--

00:24:51
*Mme. 2:* Les poires, moins bien que les pommes.

00:24:53
*Mme. [Prénom de l’intervieweuse]:* Oui, les poires sont conflictuelles chez les-- pour pas mal de personnes [inaudible 00:24:55-00:24:56] des poires. Donc, on dit c'est que les légumes, mais du coup, ils se conservent.

00:25:05
*Mme. 2:* Ah ! Mais un mois sur l'autre, il y a aucun problème en fait.

00:25:07
*Mme. [Prénom de l’intervieweuse]:* Ça, c'est impressionnant.

00:25:08
*Mme. 2:* Même les potimarrons tout ça. Ça va faire trois semaines que je les ai là.

00:25:13
*Mme. [Prénom de l’intervieweuse]:* Oui, ils peuvent pas…

00:25:15
*Mme. [Prénom de l’intervieweuse]:* Non.

00:25:16
*M. [Prénom du conjoint]:* Quant à les prix du radis noir, il n'avait pas bougé. Il n'en servait là. La dernière fois qu'on en a acheté en grandes surfaces, [diaphonie 00:25:21-00:25:23]

00:25:24
*Mme. 2:* Il était au frigo.

00:25:25
*M. [Prénom du conjoint]:* Il était au frigo et il y a eu du moisi dessus.

00:25:26
*Mme. [Prénom de l’intervieweuse]:* Oui.

00:25:32
*Mme. 2:* Après, je sais-- je connais pas [euh] Mm-hmm la façon dont il conserve exactement, est-ce qui- est-ce que le producteur met un produit dessus avant qu'il arrive en grande surface pour la conservation, la maturité du truc, j'en sais rien du tout, mais ce que je suis sûr par contre, c'est [euh] le fermier où on va, lui n'en met pas. Donc, même si [euh] vos pommes de terre, en grande surface parfois, germe au bout d'une semaine,--

00:25:57
*M. [Prénom du conjoint]:* Ça par exemple, elle ne germe jamais, les pommes.

00:25:58
*Mme. 2:* Elles, ne germe bah, elle germe un peu enfin, rien à voir [euh] au bout d'un mois quoi.

00:26:03
*M. [Prénom du conjoint]:* Rien à voir.

00:26:05
*Mme. 2:* Mm-mm et elles sont encore consommables.

00:26:07
*M. [Prénom du conjoint]:* Les oignons, les échalotes, pareil.

00:26:08
*Mme. 2:* Les oignons, j'en ai jamais perdu un, je crois.

00:26:10
*M. [Prénom du conjoint]:* Et [euh] c'est--

00:26:11
*Mme. 2:* Alors que c'est gorgé d'eau, hein.

00:26:12
*Mme. [Prénom de l’intervieweuse]:* Oui, oui, ça peut faire--

00:26:14
*M. [Prénom du conjoint]:* Pour moi, la solution à lire, c'est que dans les chambres froides, de toute façon.

00:26:17
*Mme. 2:* Ça rajoute de l'eau.

00:26:18
*M. [Prénom du conjoint]:* Ça rajoute de l'humidité tout le temps.

00:26:20
*Mme. [Prénom de l’intervieweuse]:* Mais oui, oui.

00:26:20
*M. [Prénom du conjoint]:* Mais l'humidité- l'humidité- l'humidité. Alors que [euh] là-bas, c'est dans un hangar à l'air libre. Donc la manipulation à la naturelle.

00:26:28
*Mme. [Prénom de l’intervieweuse]:* Oui.

00:26:32
*Mme. 2:* L'ail, ça va faire un an que je l'ai. C'est impressionnant. L'ail déjà en- en--

00:26:38
*Mme. [Prénom de l’intervieweuse]:* L'ail, c'est pareil.

00:26:39
*Mme. 2:* [diaphonie 00:26:39-00:26:41]

00:26:42
*Mme. [Prénom de l’intervieweuse]:* Peut se perdre. [euh] C'est en plus, c'est des produits on ne sait pas qui sont perdus, mais une fois qu'on joue,

00:26:47
*Mme. 2:* C'est incroyable. Alors vous perdez une ou deux gousses, peut-être sur une tête entière.

00:26:52
*Mme. [Prénom de l’intervieweuse]:* Oui, au bout d'un moment, c'est ça.

00:26:55
*M. [Prénom du conjoint]:* C'est vrai, mais on même fait la réflexion. Moi, je ne suis pas contre. On ne se pose pas la question, mais c'est vrai que. Les pommes de terre qui germe, avant moi, j'achetais les pommes de terre en grandes surfaces et tout le temps.

00:27:06
*Mme. [Prénom de l’intervieweuse]:* Oui, oui.

00:27:08
*Mme. 2:* Et même si tu les conserves dans un abri fermé sans la lumière, ça germe quand même, oui.

00:27:15
*M. [Prénom du conjoint]:* Là, franchement, c'est vrai que--

00:27:16
*Mme. 2:* Ils mettent rien sur la pomme de terre, ils vous le disent. D'ailleurs, ils ont même le label, je sais plus quoi là. C'était quoi le label ? [rire]

00:27:26
*Mme. [Prénom de l’intervieweuse]:* Il y a tellement de label.

00:27:27
*Mme. 2:* Non, mais eux, c'est un label qui est in-- intéressant parce que c'est [euh] Haute qualité, je sais pas quoi en fait, sans traitement.

00:27:36
*Mme. [Prénom de l’intervieweuse]:* Ok.

00:27:36
*Mme. 2:* Voilà.

00:27:36
*M. [Prénom du conjoint]:* C'est pas le label bio, parce que ça--

00:27:38
*Mme. 2:* Ils font plus--

00:27:39
*M. [Prénom du conjoint]:* Deux trois contraintes supplémentaires. Mais en fait, c'est quand même c'était bio- c'est du bio sans l'estampillé.

00:27:45
*Mme. [Prénom de l’intervieweuse]:* Ils savent que des fois ça les fait entrer dans des cases.

00:27:48
*M. [Prénom du conjoint]:* C'est ça. [diaphonie 00:27:49-00:27:50] Qu'ils ont dit [euh].

00:27:53
*Mme. 2:* Et bon, c'est vrai que parfois vous avez des légumes qui ont une forme un peu bizarres, qui ne créent pas en grandes surfaces par exemple. Il y a eu à un moment donné les légumes moches, y'a plus.

00:28:02
*M. [Prénom du conjoint]:* Ouais, mais ça a pas trop pris, je crois, pour en vendre.

00:28:03
*Mme. 2:* Non. Mais [euh] bon, voilà, pourtant c'est intéressant. Vous les achetiez moins cher en grand surface.

00:28:09
*Mme. [Prénom de l’intervieweuse]:* Je vous rappelle plus [euh]- Je vous me rappelle toutes les campagnes de- de prévention faque ils avaient le même goût les légumes moches, mais ils faisaient moins cher.

00:28:17
*Mme. 2:* Ah oui, oui.

00:28:18
*M. [Prénom du conjoint]:* Intermarché, en fait.

00:28:19
*Mme. 2:* Intermarché faisait Leclerc a eu fait un peu aussi. Mais ça, c'est pas bon, ça n'a pas poursuivi [euh] C'est dommage parce que moi, personnellement, pour faire de la soupe, je m'en fous hein. Si la carotte est droite ou pas.

00:28:31
*M. [Prénom du conjoint]:* Ouais, mais les gens étaient tellement habitués à changer les habitudes.

00:28:35
*Mme. [Prénom de l’intervieweuse]:* Ouais.

00:28:36
*Mme. 2:* Voilà.

00:28:37
*M. [Prénom du conjoint]:* Les gens, ils veulent un beau légume.

00:28:38
*Mme. 2:* C'est important.

00:28:40
*Mme. [Prénom de l’intervieweuse]:* C'est-- La plupart, après les personnes qu'on met en interroge dans le cadre de faudrait, ce- ce sont des personnes qui ont été sensibilisées et qui se sont portées volontaires pour le projet. J'ai eu le sentiment que les légumes moches, si les personnes sont- sont ok avec certains difformes. Mais finalement, je pense que devant l'étalage, inconsciemment, on va quand même rendre ce qui compte. Mais à la base, je pense qu'il y a quand même un sentiment que ça a marché cette- cette campagne. Non, ce n'est pas grave. Si les moches sont finalement moins choisis, je pense.

00:29:14
*Mme. 2:* Que celui qui est beau ne fait pas. Suffisamment bien. Je suis un peu déçue. On l'a tellement passé longtemps que--

00:29:22
*Mme. [Prénom de l’intervieweuse]:* Mais en fait, c'est un peu--

00:29:24
*Mme. 2:* Non, mais le prix, il était quand même- le prix était quand même [euh] significatif. Quand tu mets ton 2,50 €, ton kilo de pommes, c'était un 50 l'autre. [euh]

00:29:34
*Mme. [Prénom de l’intervieweuse]:* Là, dans ce cas-là, le coup, c'est [euh] vrai. Mais bon, franchement, ils savent qu'il n'y a pas de problème.

00:29:39
*Mme. 2:* Oui.

00:29:39
*Mme. [Prénom de l’intervieweuse]:* Et s'il y a pris [euh] avec une cuillère, plus d'intérêt à prendre.

00:29:44
*Mme. 2:* Je n'avancerai pas inaperçu que je n'ai pas vu, des fraises, il y a longtemps.

00:29:47
*M. [Prénom du conjoint]:* On a eu à acheter des fraises- des fraises [euh] en Californie. On va acheter ça par exemple. Et- et on avait le jus-- Puis combien de kilos de fraises, c'est juste parce que c'était pas des calibrés pour mettre dans les boîtes.

00:29:56
*Mme. 2:* Mais je m'en foutais, c'était pour faire de la confiture.

00:29:58
*Mme. [Prénom de l’intervieweuse]:* Donc [euh] même pour les manger, on en a mangé un--

00:30:02
*Mme. 2:* On en a mangé des- des-- comme ça, ouais.

00:30:04
*M. [Prénom du conjoint]:* C'était juste pas calibré, mais au contraire, enfin.

00:30:07
*Mme. 2:* Mais dans la nature des fruits, ils n'ont pas tous le même corps.

00:30:13
*Mme. [Prénom de l’intervieweuse]:* [euh] Vous dites c'était pas grave, c'était pour faire la confiture. Vous avez des visiteurs pour la carotte, c'était pour la soupe.

00:30:18
*Mme. 2:* Ouais, moi je fais des confitures, je fais des conserves, je fais du sirop avec les queues de fraises. Si-- j'ai découvert ça cette année, c'est vachement bon. En fait, on ne jette plus rien, quasiment. [euh] Mais ce que je fais avec les fruits et légumes, je le fais aussi dans d'autres choses. Par exemple, là, cette année, j'ai trouvé du- du thon, qui était pas cher du tout, du thon blanc. J'en ai fait à l'huile, en conserves, comme ça, j'en aurais cet hiver à l'huile d'olive--

00:30:46
*M. [Prénom du conjoint]:* Avec des fans de radis, on fait la soupe.

00:30:48
*Mme. 2:* Ouais. Avec les fans de radis, on fait la soupe.

00:30:50
*Mme. [Prénom de l’intervieweuse]:* Ouais, je disais les fans.

00:30:53
*Mme. 2:* Ouais, on peut le faire avec les carottes aussi.

00:30:54
*Mme. [Prénom de l’intervieweuse]:* [inaudible 00:30:55-00:30:56]

00:30:57
*Mme. 2:* On essaye toujours de valoriser le truc, pas le jeter bêtement.

00:31:01
*M. [Prénom du conjoint]:* On valorise pas, mais on ne va pas jusqu'à valoriser les épluchures de légumes.

00:31:05
*Mme. 2:* Mais on pourrait, hein, je pourrais faire des- des Mm-hmm--

00:31:07
*M. [Prénom du conjoint]:* Ben, oui ça se fait hein, il y a plein de trucs à faire, hein.

00:31:08
*Mme. 2:* Vous savez, les fond de sauce-là, les- les bouillon de légumes.

00:31:12
*M. [Prénom du conjoint]:* Bien sûr.

00:31:13
*Mme. 2:* Il y en a qui font des chips.

00:31:15
*M. [Prénom du conjoint]:* Des chips, c'est ce que j'allais dire.

00:31:16
*Mme. 2:* Ça, j'ai envie d'essayer. Des chips de légumes. On est-- Maintenant en plus à la ferme, on a les paniers. Donc [Prénom du conjoint] s'est découvert une passion pour les paniers. [rire] C'est vachement bon. On peut les accommoder de plein de façons. Donc ça avec la peau des paniers, je sais qu'on peut faire des chips.

00:31:36
*Mme. [Prénom de l’intervieweuse]:* Ouais, donc de façon général, vous disiez de [euh] vous disiez valoriser le produit.

00:31:40
*Mme. 2:* Oui, j'essaye.

00:31:42
*Mme. [Prénom de l’intervieweuse]:* Perdre un minimum de jus, quoi.

00:31:47
*M. [Prénom du conjoint]:* On le voit, on ne jette pas beaucoup de- de- de ce qu'on a le plus comme déchet. C'est tous les emballages.

00:31:52
*Mme. 2:* Des emballages.

00:31:53
*M. [Prénom du conjoint]:* Ça n'arrête pas, mais par contre la- la poubelle, en fin les déchets [euh]--

00:31:56
*Mme. 2:* Déchets ménagers, ouais.

00:31:58
*M. [Prénom du conjoint]:* Nous, on a fait un ramassage que tous les quinze jours [euh], on a jugé important. Et [euh] ce n'est jamais plein. Des fois, il est a moitié pleine seulement quoi.

00:32:05
*Mme. [Prénom de l’intervieweuse]:* Oui.

00:32:06
*M. [Prénom du conjoint]:* Quand les enfants font le ménage dans leur chambre, déjà ils remplissent parce que--

00:32:13
*Mme. 2:* C'est plus du ménager, c'est du incinérateur. [rire]

00:32:17
*M. [Prénom du conjoint]:* [inaudible 00:32:17-00:32:18] C'est que des déchets ménagers. C'est vrai qu'on jette pas beaucoup.

00:32:21
*Mme. 2:* Non.

00:32:27
*Mme. [Prénom de l’intervieweuse]:* Ok, ça marche. Alors si on prend une autre photo ?

00:32:30
*Mme. 2:* On va toutes les faire ?

00:32:31
*Mme. [Prénom de l’intervieweuse]:* Oui.

00:32:35
*Mme. 2:* Ah ! J'adore. Potager de quelqu'un. [rire] Ça, c'est du- de home made ça.

00:32:44
*Mme. [Prénom de l’intervieweuse]:* Donc tout ça vous évoque, le potager de quelqu'un ?

00:32:47
*Mme. 2:* Moi, j'adorerais avoir la même chose ici, mais je peux pas.

00:32:50
*M. [Prénom du conjoint]:* Tout ça nous évoque qu'il y ait une friche pour l'instant. [rire]

00:32:51
*Mme. 2:* C'est ça. Ça nous dit qu'il- qu'il va falloir qu'on fasse notre jardin. Et ça, c'est génial en fait, parce qu'en fait vous avez la possibilité de ne produire que ce que vous consommez. Donc vous pouvez ajuster en fait [euh] vos cultures à vos habitudes alimentaires. Et vous pouvez même vous permettre de varier puisque vous pouvez changer autant que vous le souhaitez. Et là, pour le coup, c'est hyper rentable parce que dans des endroits comme ici en Bretagne où il pleut tout le temps n'est ce pas ? Ben, je pense que vous faites de larges économies en fait.

00:33:29
*Mme. [Prénom de l’intervieweuse]:* Oui.

00:33:29
*Mme. 2:* Vous pouvez maîtriser et le-- la culture en elle-même, c'est à dire le choix du légume ou du fruit que vous cultivez, mais aussi la façon dont vous le faites. Donc, si vous n'avez pas envie de mettre d'engrais, vous n'en mettez pas. Si vous avez envie d'en mettre, vous en mettez, enfin, voilà.

00:33:46
*Mme. [Prénom de l’intervieweuse]:* Ouais.

00:33:46
*Mme. 2:* Pour moi, ça c'est le top parce que c'est même pédagogique pour les petits. C'est génial, c'est le retour aux sources en fait.

00:33:53
*M. [Prénom du conjoint]:* Et puis c'est maintenant la conservation.

00:33:56
*Mme. 2:* C'est mon enfance, il n'y a pas le problème. Alors si ça peut poser un problème de consommation en cité comme mon père où ils sont deux où tu- tu cultives comme s'ils étaient 50, tu as 60 000 couches d'un coup par exemple. [rire]

00:34:08
*M. [Prénom du conjoint]:* C'est ça. Tu prends un exemple extrême parce que le terrain est grand.

00:34:11
*Mme. 2:* Mais voilà.

00:34:12
*M. [Prénom du conjoint]:* Non, mais même pour faire [euh] enfin le--

00:34:15
*Mme. 2:* Mais on avait commencé.

00:34:16
*M. [Prénom du conjoint]:* Oui, on avait une année.

00:34:17
*Mme. 2:* Moi, j'avais fait mon- mon jardin de [euh] Mm-hmm de plantes aromatiques et on avait fait des fraises pour les petits cette année, l'année prochaine, on aura des framboises, on va planter des myrtilles. Enfin, voilà. Moi, je trouve que c'est important aussi d'enseigner ça aux enfants. Parce que ben il y en a plein qui ne savent pas ce qu'ils mangent en fait, on s'en aperçoit chez les plus grands déjà [euh]. Ben moi je cuisine quand même pas mal et des fois je leur fais des- des blind test, c'est à dire qu'ils doivent trouver ce qu'il y a dans le plat, en fait.

00:34:47
*Mme. [Prénom de l’intervieweuse]:* Ouais.

00:34:49
*Mme. 2:* Si c'est une catastrophe. Et même quand vous leur montrer, parfois, c'était *[prénom]*-là qui avait confondu une asperge avec un poireau. J'ai truc, un truc énorme quoi. Je dis non, mais tu-- tu rigoles ou quoi ?

00:35:02
*M. [Prénom du conjoint]:* Encore- encore on est en France.

00:35:02
*Mme. 2:* Oui, quand même.

00:35:03
*M. [Prénom du conjoint]:* Pas aux Etats-Unis.

00:35:04
*Mme. 2:* Ouais, ouais, mais bon je vais me contenter de la France, ça m'a assez perturbé. Et pour vous, c'est important, ça se peut pédagogique, voilà ou on devrait savoir ce qu'on veut.

00:35:16
*Mme. [Prénom de l’intervieweuse]:* Ouais, je trouve, ben oui, quand même.

00:35:22
*Mme. 2:* Si tu sais pas ce que tu manges, on peut te donner n'importe quoi. [rire] J'aimais- j'aimerais qu'il espère à autre chose quoi. Les frites, tu peux les faire toi-même par exemple. Ce n'est pas que du McCain.

00:35:40
*Mme. [Prénom de l’intervieweuse]:* Donc le potager, vous aimerez bien, notamment [euh] d'un point de vue économique, [euh] que vous y retrouverez. D'un point de vue pédagogique aussi. [euh] est-ce qu'il y a d'autres raisons qui feraient que le potager serait pour vous un-- Faute d'en avoir un ou si c'est principalement ça.

00:36:03
*Mme. 2:* C'est principalement ça. Et après, pour l'avoir vécu, je vous dis toute mon enfance, c'est aussi quelque chose de- de-- qui peut devenir solidaire aussi parce que moi, j'ai vu mes parents toute leur vie [euh] en faire profiter les voisins, les amis. [euh] Et- et c'est ce qui m'a donné le goût de la cuisine aussi. Enfin, je veux dire [euh] si j'avais pas eu [euh] une maman cuisinière, un père cultivateur [euh] à ses heures perdues [euh], bah, je serais peut-être pas une maman qui cuisine aujourd'hui, qui a envie de transmettre ça à ses enfants. [euh] Et j'aimerais peut-être pas manger comme [euh]-- Je n'aurai pas ce plaisir [euh] de faire à manger et de partager des bons moments. Tout ça, c'est tout est lié, en fait.

00:36:45
*Mme. [Prénom de l’intervieweuse]:* Selon vous, le fait d'avoir connu [euh] le légume dans son jus en son milieu naturel [euh] a eu une influence sur aujourd'hui le plaisir que vous avez.

00:36:56
*Mme. 2:* Et sur ma curiosité aussi. Parce que du coup, ben j'ai été un petit peu habitué, formaté à un cer- à un certain type de légumes et d'alimentation, parce que moi, je vivais dans le sud. Donc on cuisine d'une certaine manière très très [euh] influencée par l'Italie, [euh] le- le- le Maroc, enfin l'Espagne, tout ça. Et quand je suis devenu, moi, adulte, bah, j'ai eu envie de voir d'autres trucs. Donc je me suis orienté vers les épices, davantage vers des légumes qu'on connaissait pas. Le pané, des choses comme ça, j'en mangeais pas chez mes parents.

00:37:29
*Mme. [Prénom de l’intervieweuse]:* Oui.

00:37:29
*Mme. 2:* Donc voilà, ça m'a éle-- ça m'a élevé à cette espèce de curiosité aussi.

00:37:37
*Mme. [Prénom de l’intervieweuse]:* Est-ce qu'il y a de produits petits que vous connaissez. Vous l'avons mangée aujourd'hui que vous n'aviez pas ?

00:37:41
*Mme. 2:* Absolument, oui.

00:37:45
*M. [Prénom du conjoint]:* Après on peut partir à un endroit où sa cuisine n'est pas trop vieille, mais elle cuisinait quand même. [rire]

00:37:48
*Mme. 2:* Ben oui.

00:37:49
*Mme. [Prénom de l’intervieweuse]:* Dans les deux cas de figure.

00:37:53
*M. [Prénom du conjoint]:* Enfin, moi, je ne sais pas si en étant assis en même temps. Si c'est merci mes grand-mères qui m'ont donné envie de cuisiner, je pense.

00:37:57
*Mme. 2:* Oh ! Mais cuisinier vachement aussi ta grand-mère.

00:37:59
*M. [Prénom du conjoint]:* Ma grand mère, elle, a acheté ses trucs au marché à cuisiner.

00:38:01
*Mme. 2:* Ben voilà. Moi, j'ai connu ça aussi. Ma marraine était une personne âgée, en fait, elle n'achetait que [euh] au marché sa viande, son poisson, ses légumes, ses fruits. Et puis elle cuisinait royalement bien.

00:38:14
*M. [Prénom du conjoint]:* Quand j'étais petit. C'est très souvent ma mère, moi, au moins une fois par semaine et tout ça est lui aussi ça, à aimer voir les produits--

00:38:20
*Mme. 2:* Mais c'était culturel à avoir manger vachement plus que maintenant.

00:38:23
*M. [Prénom du conjoint]:* À avoir un- un vendeur qui dit tiens et goût ça.

00:38:26
*Mme. [Prénom de l’intervieweuse]:* Ouais.

00:38:27
*M. [Prénom du conjoint]:* Un type de abricot, un truc, toi, tu diras oui, ça sent vachement bon.

00:38:31
*Mme. 2:* Ben oui.

00:38:32
*M. [Prénom du conjoint]:* Et [euh] seuls les abricots de grande surface, tiennent seul.

00:38:36
*Mme. 2:* Bah oui, c'est dégueulasse. Mais même si vous faites de la confiture avec, elle n'a pas de goût. Il faut mettre trois tonnes de sucre [euh], il faut la cuire [euh] 50 ans. Et puis [euh], enfin vous n'avez pas. Pour acheter des abricots dans le sud, c'est là qu'ils poussent. [rire]

00:38:55
*Mme. [Prénom de l’intervieweuse]:* Ben oui.

00:38:56
*M. [Prénom du conjoint]:* En fait, on devrait manger que les choses qui poussent autour de nous.

00:38:59
*Mme. 2:* Mais oui- oui- oui.

00:39:02
*M. [Prénom du conjoint]:* Donc on connaît le reste.

00:39:03
*Mme. [Prénom de l’intervieweuse]:* Oui.

00:39:04
*Mme. 2:* C'est compliqué.

00:39:04
*Mme. [Prénom de l’intervieweuse]:* C'est le genre de fois du passé.

00:39:08
*Mme. 2:* Ouais puis bon, faut quand même pas déconner. C'est quand même un des derniers plaisirs accessibles qui nous reste. [euh] Quand il y a eu le colis, il y a eu si, il y a eu çà, le seul plaisir qu'on avait, c'était de manger. [rire] Manger et jouer en famille et se retrouver dans des moments sympa quoi. Mais si ça a été un lien. Moi, ça m'a permis de créer une souris pour ça où je me suis dit, mais oui, en fait. [euh] Cuisiner en famille, c'est vachement bien. Et ça, m'a hop, allez faire bondir sur un truc. Et voilà. Mais [euh] je trouve que c'est-c'est une des dernières choses qui reste abordable et accessible à tous en fait.

00:39:48
*Mme. [Prénom de l’intervieweuse]:* Donc pour vous cuisiner, c'est au-delà d'une [euh] on va dire une tâche ménagère.

00:39:53
*Mme. 2:* Ah ! Oui, carrément, c'est- c'est du bonheur à l'état pur hein. Donc moi, c'est-- [euh] J'aime cuisiner pour- [euh] pour- [euh] pour vous faire plaisir en fait. Pour-- Mais je me mets, moi dans ce--

00:40:07
*M. [Prénom du conjoint]:* Même quand tu fais du poisson ?

00:40:08
*Mme. 2:* Oui, même quand je fais du poisson. [rire] Ouais, mais les enfants, ils aiment.

00:40:14
*M. [Prénom du conjoint]:* Moi, c'est pareil hein enfin quand on cuisine, voilà pas le 12 heures. C'est pas ça qu'on fait cuire vite fait des pâtes ou quoi ?

00:40:20
*Mme. [Prénom de l’intervieweuse]:* Non.

00:40:21
*M. [Prénom du conjoint]:* Non, parce qu'ils ont bien dit, ils sont sortis de l'école et que je ramène les pieds l'après-midi.

00:40:24
*Mme. 2:* Oui.

00:40:25
*M. [Prénom du conjoint]:* On n'est pas dans le même truc, mais sinon. Même le pain rassis et tout maintenant, je fais mes pudding et tout

00:40:32
*Mme. 2:* C'est vachement mou.

00:40:35
*M. [Prénom du conjoint]:* Un référendum, ça explique le raisonnement tatoué à l'appui. Un pudding salé. C'était superbe.

00:40:41
*Mme. [Prénom de l’intervieweuse]:* Je connais pas mes voisins après, mais à eux.

00:40:43
*M. [Prénom du conjoint]:* On peut le faire à tout.

00:40:44
*Mme. 2:* Sucré salé.

00:40:45
*M. [Prénom du conjoint]:* Savoir tartiflette, savoir tout ce qu'on veut. Mais c'est une nouvelle [euh]- c'est une nouvelle maison.

00:40:53
*Mme. [Prénom de l’intervieweuse]:* C'est un super plat anti-gaspi à zéro euro quoi. Mais pudding.

00:40:57
*M. [Prénom du conjoint]:* Ouais ouais ouais.

00:40:58
*Mme. 2:* En fait, vous gardez tout ce qui est pain, viennoiserie, panini.

00:41:03
*Mme. [Prénom de l’intervieweuse]:* Oui.

00:41:04
*Mme. 2:* [euh] Vous les gardez.

00:41:05
*Mme. [Prénom de l’intervieweuse]:* Ok. Mais j'ai même moi, je mets même les restes de pâtes aussi dedans.

00:41:08
*Mme. 2:* Oui, il y a des pâtes, du riz [euh].

00:41:10
*M. [Prénom du conjoint]:* Il y avait un reste de pâtes, des pâtes aux légumes là [euh]

00:41:13
*Mme. 2:* Vous pouvez le faire avec du blé, des lentilles de toutes en fait, oui, incroyable

00:41:20
*M. [Prénom du conjoint]:* Des oeufs, des épices, le reste de ratatouille dedans et un peu de parmesan et puis--

00:41:25
*Mme. 2:* Au fromage et basta ! Et c'est franchement ultra bon.

00:41:29
*Mme. [Prénom de l’intervieweuse]:* Trop bien vous savez croustibat du coup, c'est dans le jus.

00:41:31
*Mme. 2:* Mais oui.

00:41:32
*M. [Prénom du conjoint]:* Mais oui, oui et puis ça fait [euh] un repas complet.

00:41:35
*Mme. 2:* Ouais, avec une salade verte ou des tomates.

00:41:38
*Mme. [Prénom de l’intervieweuse]:* Oui.

00:41:39
*Mme. 2:* Et basta.

00:41:40
*M. [Prénom du conjoint]:* Ouais, ouais.

00:41:43
*Mme. [Prénom de l’intervieweuse]:* Qui veut une recette. [rire]

00:41:45
*Mme. 2:* Un métier, vous en appréciez ?

00:41:46
*M. [Prénom du conjoint]:* C'est- C'est hyper complexe à faire.

00:41:49
*Mme. [Prénom de l’intervieweuse]:* Non.

00:41:49
*M. [Prénom du conjoint]:* Le robot. [rire]

00:41:51
*Mme. [Prénom de l’intervieweuse]:* Et hop, là.

00:41:52
*Mme. 2:* Il te faut le plat. [rire]

00:41:54
*Mme. [Prénom de l’intervieweuse]:* Parfait.

00:41:56
*Mme. 2:* Ouais.

00:41:57
*Mme. [Prénom de l’intervieweuse]:* Non, le bœuf. Ok, [euh] vous avez dit sur le bonheur de cuisiner ? Vous avez parlé de mettre un peu de vous dans- dans le plat. Vous pouvez développer ce que vous vouliez raconter par là ?

00:42:14
*Mme. 2:* Alors ce que je veux dire par là, c'est que [euh] D'abord, moi, j'ai envie de donner envie à mes enfants de faire comme moi plus tard. [euh] Puis j'ai envie de leur montrer comme je les aime parce que je cuisine, parce que je sais que [euh] ce que je suis en train de faire, ça va leur plaire, particulièrement parce qu'ils m'auront dit, maman, ça c'est trop bon machin. Donc je vais avoir envie de leur refaire parce que [euh] je sais que je leur fais du bien en [euh] en préparant les repas [euh] de cette façon-là. Parce que [euh] parce qu'on aime bien [euh]. Ben passer [euh] Mm-hmm ce moment-là ensemble. Ça représente quelque chose pour nous. Donc on aime bien aussi se faire nos petits [euh] pour Halloween, on se fait des repas Halloween sur le thème de Halloween. Donc ça- ça- ça pousse aussi la créativité [euh] de tous en fait [euh] pour Noël, on fait un repas spécifique Noël, enfin on aime bien. On est donc dans ce- dans ce délire là, un peu.

00:43:20
*Mme. [Prénom de l’intervieweuse]:* C'est chouette.

00:43:22
*Mme. 2:* [rire] Mais voilà, au moins j'essaye de leur donner envie. Ça ne marchera peut-être pas. Mais en tout cas, ils finissent leurs assiettes. [rire]

00:43:28
*Mme. [Prénom de l’intervieweuse]:* Quand ils mangent des plats bons. [rire]

00:43:33
*M. [Prénom du conjoint]:* Chacun fait son petit tri du truc qu'il n'aime pas.

00:43:35
*Mme. 2:* Oui, mais ça oui. Ah ! Par contre, ça, c'est un truc, effectivement. Tu fais bien de le dire, je suis une maman qui cuisine, mais je ne m'amuse pas à faire un plat par personne. C'est-à-dire que [euh] s'il y en a un qui n'aime pas les oignons, l'autre, il n'aime pas [euh] le fromage, l'autre, il n'aime pas [euh] la tomate. Si, par exemple, je fais des lasagnes, je ne vais pas m'amuser à faire un côté sans oignon, l'autre sans viande, l'autre machin, non. Le- le- le but, c'est, tu goûtes, t'aimes pas, t'aime pas. Tu tries, si tu veux, ça te regarde. Mais tu- tu manges la même chose que tout le monde, voilà. Mon mari, lui, il est vachement plus sympa que moi. Lui, il adapte. C'est-à-dire que s'il y en a un qui n'aime pas le fromage, il va faire un plat spécifique ou c'est la même chose, mais sans le fromage. [rire] Il y en a une qui aime pas la tomate [euh] crue. C'est pas grave, il va lui mettre du maïs ou du concombre, mais [euh] voilà. Ah non, moi, je ne suis pas dans ce délire-là de tout le monde.

00:44:33
*Mme. [Prénom de l’intervieweuse]:* Ben c'est vrai qu'à huit ça peut vite te--

00:44:34
*Mme. 2:* Ben, c'est ça.

00:44:35
*Mme. [Prénom de l’intervieweuse]:* [diaphonie 00:44:35-00:44:36] Systématique après--

00:44:38
*Mme. 2:* Arrête t'es un papa poule. Il y a une fois, on a mangé du pudding. Ma fille n'aime pas le fromage, il lui a fait une omelette.

00:44:43
*Mme. [Prénom de l’intervieweuse]:* Ouais.

00:44:44
*Mme. 2:* Ben oui. Mais du coup, elle n'a pas fait l'effort de le goûter ton pudding.

00:44:46
*M. [Prénom du conjoint]:* Elle ne mange jamais.

00:44:47
*Mme. 2:* Mais c'est pas grave, je ferais mieux demain. Mais non, t'es trop gentil. [rire]

00:44:56
*M. [Prénom du conjoint]:* C'est souvent [Prénom de l’enfant].

00:44:57
*Mme. 2:* Ben oui, c'est souvent [Prénom de l’enfant]. Moi, c'est souvent pour [Prénom de l’enfant] où je me dis Ah ! Merde, c'est vrai, elle n'aime pas les tomates. C'est difficile de faire des- des lasagnes à la bolognaise sans tomates, mais elle les mange quand même hein.

00:45:09
*Mme. [Prénom de l’intervieweuse]:* Oui.

00:45:10
*Mme. 2:* Après, il y en a un qui n'aime pas le jambon, l'autre, il n'aime pas.

00:45:14
*Mme. [Prénom de l’intervieweuse]:* Oui. Mm-hmm

00:45:15
*Mme. 2:* Hein, voilà, tu manges pas ce soir, c'est pas grave, tu mangeras mieux demain. [rire] Mais j'essaye toujours. Ouais, voilà si un jour je fais un plat [euh] qui ne fait pas l'unanimité, le lendemain, je ne suis pas psychopathe au point de leur- leur proposer quoi. Je vais me le garder, moi, pour manger au travail par exemple, [euh] diviser le tout. Donc voilà. Mais [euh] eux, je vais leur faire un truc le lendemain, qu'ils aiment.

00:45:39
*Mme. [Prénom de l’intervieweuse]:* Oui, oui. [rire]

00:45:40
*M. [Prénom du conjoint]:* [inaudible 00:45:40-00:45:43] [rire]

00:45:44
*Mme. [Prénom de l’intervieweuse]:* Les autres, c'est sûr.

00:45:45
*M. [Prénom du conjoint]:* Avec la sauce barbecue. [rire]

00:45:47
*Mme. 2:* C'est bien dégueulasse.

00:45:48
*Mme. [Prénom de l’intervieweuse]:* Ouais, c'est ça.

00:45:49
*M. [Prénom du conjoint]:* Oui, c'est bon.

00:45:51
*Mme. 2:* Disons que j'ai pas envie qu'ils ne mangent que ça. J'ai envie de les éduquer à manger un peu autre chose. À s'ouvrir un peu sur autre chose.

00:45:59
*Mme. [Prénom de l’intervieweuse]:* Ça va faire des entorses, non ? Ok.

00:46:04
*M. [Prénom du conjoint]:* On y tient, par exemple.

00:46:05
*Mme. 2:* Ouais, ils adorent ça.

00:46:06
*M. [Prénom du conjoint]:* Je les mange, les brocolis. Pour me faire manger, il fallait y aller.

00:46:10
*Mme. [Prénom de l’intervieweuse]:* Depuis que vous [euh] vous découvrez--

00:46:12
*M. [Prénom du conjoint]:* Brocolis de la ferme.

00:46:13
*Mme. [Prénom de l’intervieweuse]:* Oui.

00:46:13
*Mme. 2:* Le chou-fleur.

00:46:15
*Mme. [Prénom de l’intervieweuse]:* Le chou-fleur--

00:46:15
*Mme. 2:* On les mange même cru, le chou-fleur

00:46:17
*M. [Prénom du conjoint]:* Et puis le chou-fleur, il est trop bon.

00:46:19
*Mme. 2:* Il est doux, il est--

00:46:20
*M. [Prénom du conjoint]:* Il est encore meilleur, plus dur.

00:46:22
*Mme. [Prénom de l’intervieweuse]:* Ouais. Ben, c'est vrai qu'en plus les brocolis, les choux-fleurs, typiquement c'est [euh] des souvenirs de- de cantine quoi surpris compris.

00:46:32
*M. [Prénom du conjoint]:* Voilà.

00:46:33
*Mme. [Prénom de l’intervieweuse]:* Qu'un gros con y'a rien du tout. [rire] [diaphonie 00:46:34-00:46:38]

00:46:39
*M. [Prénom du conjoint]:* C'est dégueulasse.

00:46:40
*Mme. [Prénom de l’intervieweuse]:* Ouais.

00:46:41
*Mme. 2:* Peut-être qu'un jour j'arriverai à vous faire manger des épinards. [rire]

00:46:44
*Mme. [Prénom de l’intervieweuse]:* Ah !

00:46:45
*M. [Prénom du conjoint]:* Ça, les épinards, je mange pas.

00:46:46
*Mme. [Prénom de l’intervieweuse]:* Ah ouais ! Avec la crème.

00:46:49
*Mme. 2:* La crème et les œufs durs.

00:46:51
*M. [Prénom du conjoint]:* Je regouterais même.

00:46:52
*Mme. 2:* Mais t'avais pas à détester le dur hein. T'avais goûté un peu.

00:46:56
*Mme. [Prénom de l’intervieweuse]:* Dans un cake à du cher.

00:46:58
*Mme. 2:* Ouais.

00:46:59
*M. [Prénom du conjoint]:* Ouais, Ça-ça s'agace, le- le cake avec le du cher, c'est doux.

00:47:02
*Mme. 2:* Pourtant, mes, du coup, est un peu masqués. On le--

00:47:04
*M. [Prénom du conjoint]:* Alors, j'essaye.

00:47:07
*Mme. 2:* Et là, je vais essayer une recette bientôt. [Prénom], elle, m'a parlé d'une recette d'épinards froid. Il paraît qu'elle déteste l'épinard. C'est la seule façon dont elle le mange. C'est une recette d'une de ses copines. Et le fait qu'il soit froid, en fait, c'est vrai, il ne doit pas avoir de goût.

00:47:21
*M. [Prénom du conjoint]:* Cuit froids.

00:47:22
*Mme. 2:* Cuits froids, c'est une entrée.

00:47:25
*Mme. [Prénom de l’intervieweuse]:* Et ça ne fait que froid.

00:47:26
*Mme. 2:* Je ne sais pas ce qu'elle met dedans. Je lui ai demandé de me filer la recette parce que c'est très bon.

00:47:30
*Mme. [Prénom de l’intervieweuse]:* Elle a plein de trucs à-- Alors je vous invite à ouvrir la deuxième et dernière [euh] photo.

00:47:38
*Mme. 2:* Allons-y ! Ah ben voilà, un vrai marché.

00:47:40
*Mme. [Prénom de l’intervieweuse]:* Un vrai marché.

00:47:41
*Mme. 2:* Voilà.

00:47:42
*Mme. [Prénom de l’intervieweuse]:* Ensuite là, vous y allez pas forcément ?

00:47:44
*Mme. 2:* Non.

00:47:44
*Mme. [Prénom de l’intervieweuse]:* Mais vous avez dit que vous y aller petit.

00:47:46
*Mme. 2:* Oui, même si j'y allais petit.

00:47:48
*M. [Prénom du conjoint]:* Ah oui ! On y allait- on y allait avant mais bon.

00:47:50
*Mme. [Prénom de l’intervieweuse]:* Oui, aujourd'hui, il y a plus trop de-- Est-ce que vous pouvez me raconter comment--, en termes de-- [euh] quand vous y allez ou quand vous y allez avant, comment vous vous sentez dans ce type de lieux ?

00:48:00
*Mme. 2:* Ah ! Moi, j'adore. Moi, c'est à-- ça a le- le marché franchement, c'est- c'est-- J'adore ça quoi.

00:48:07
*Mme. [Prénom de l’intervieweuse]:* Oui.

00:48:08
*Mme. 2:* Surtout les marchés du Sud, parce que les marchés d'ici, c'est pas pareil, je trouve. Mais dans le Sud, la photo que vous avez là, c'est- c'est vraiment ça quoi.

00:48:15
*Mme. [Prénom de l’intervieweuse]:* Oui.

00:48:16
*Mme. 2:* C'est le bordel, il y a plein de monde, ça crie partout [euh] Le poisson à côté des légumes. Ouais, non, moi j'adore, j'adore. Et vous ne trouvez que ce qui est de saison normalement sur un marché. Vous n'avez pas-- Enfin maintenant, oui, ici en tout cas, oui.

00:48:35
*M. [Prénom du conjoint]:* Maintenant, oui, on trouve des choses que tu dis sur les marchés.

00:48:38
*Mme. 2:* Bon, ça n'a rien à foutre-là, mais avant, en tout cas, moi je me souviens, quand on était petit, le marché, c'était vraiment [euh] le petit producteur du coin qui venait vendre sa récolte et qui n'avait que ça quoi, il n'y avait pas [euh]-- Ah ben, désolé, j'en ai plus. [rire] Mais c'était presque bien que ça soit comme ça en fait.

00:49:01
*Mme. [Prénom de l’intervieweuse]:* Donc c'est un lieu dans lequel vous aimez [euh] vous rendre.

00:49:07
*M. [Prénom du conjoint]:* Le monde sur un marché est moins des-- Il y a trop de monde, c'est pas très agréable, mais c'est moins désagréable dans le monde, dans la grande surface.

00:49:15
*Mme. 2:* Oui, c'est bon, c'est vrai.

00:49:17
*Mme. [Prénom de l’intervieweuse]:* Pourquoi selon vous ? Ça, vous savez ça ?.

00:49:19
*M. [Prénom du conjoint]:* Je sais pas, déjà, les gens sont stressés.

00:49:20
*Mme. 2:* Ben, c'est une démarche hein, déjà d'aller à un marché hein.

00:49:22
*M. [Prénom du conjoint]:* Je crois que les gens sont plus stressés dans les grandes surfaces, c'est tout ça.

00:49:24
*Mme. 2:* Oui, c'est le gain du temps. Le marché, vous prenez votre temps, vous- vous êtes chiner, vous regardez ou comparez [euh]. Grande surface, vous en foutez. Vous regardez les étiquettes. Vous avez besoin de ça, c'est sur ma liste. Allez boum, je paye et je me casse. Ça n'a rien à voir en fait. Le marché, j'adore parce que vous prenez le temps de parler aux gens.

00:49:44
*Mme. [Prénom de l’intervieweuse]:* Oui.

00:49:44
*Mme. 2:* En fait, [euh] le petit vendeur, ben, il vous connaît, il vous appelle par votre prénom. Vous êtes sont habitués [euh]. Il sait quelles sont vos- vos préférences. Il vous les met de côté. [euh] Enfin, voilà, seulement, c'est pas du tout la même dimension.

00:50:00
*M. [Prénom du conjoint]:* Il y a beaucoup plus d'interactions sociales, ça, c'est sûr.

00:50:04
*Mme. [Prénom de l’intervieweuse]:* Oui.

00:50:04
*Mme. 2:* Parfois, il y a des gens qui préfèrent aller au marché, même si c'est plus cher pour ça.

00:50:09
*Mme. [Prénom de l’intervieweuse]:* Ouais.

00:50:11
*Mme. 2:* Et si moi, si j'avais trouvé un marché qui convenait, je pense que je ferais plus ça, que grande surface. Mais j'ai pas encore trouvé.

00:50:19
*M. [Prénom du conjoint]:* On n'a pas vraiment essayé le marché de Plouguerneau, mais pour ce jeudi matin.

00:50:23
*Mme. 2:* Je ne vois pas comment je pourrais y aller.

00:50:24
*M. [Prénom du conjoint]:* Mais [euh]--

00:50:25
*Mme. 2:* Si je t'envoie en- en é[Prénom de l’intervieweuse]ur.

00:50:28
*M. [Prénom du conjoint]:* Il faudrait qu'on aille de temps en temps à Saint-Renan, de servir le marché de Saint-Renan.

00:50:30
*Mme. 2:* Saint-Renan, il était bien, mais je n'y suis pas allée souvent. Mais le peu que j'y suis allée, on a possibilité de tout trouver. Vous trouvez aussi bien le nœud papillon que le--

00:50:40
*Mme. [Prénom de l’intervieweuse]:* À faire, non ?

00:50:42
*M. [Prénom du conjoint]:* Non, il y a bien celui de Lesneven, il est bien aussi.

00:50:44
*Mme. 2:* Je suis jamais y aller.

00:50:45
*M. [Prénom du conjoint]:* Les marchés le lundi matin, marché de Lesneven est vraiment bien. Je peux même acheter des poulets et tout ça.

00:50:50
*Mme. 2:* Parce que c'est- c'est bizarre ici, il n'y a pas de marché de journée.

00:50:53
*Mme. [Prénom de l’intervieweuse]:* Marché pendant la journée ?

00:50:56
*Mme. 2:* Oui. Dans le Sud, [euh] le marché, c'est toute la journée, en fait.

00:51:00
*Mme. [Prénom de l’intervieweuse]:* On sait que le matin--

00:51:01
*Mme. 2:* C'est que le matin est tard. Ça commence, il est 8 heures et demie, quoi. Dans le Sud, vous y allez à 7 heures 7 heures et demie. Vous avez déjà du monde, quoi.

00:51:09
*Mme. [Prénom de l’intervieweuse]:* Oui, oui, si ça a été 9 heures 13 heures quoi.

00:51:14
*Mme. 2:* C'est ça ?

00:51:14
*Mme. [Prénom de l’intervieweuse]:* Mm-mm

00:51:14
*M. [Prénom du conjoint]:* Alors que ça marche la nuit marque-- les marchés, je sais pas à 16 heures le vendredi.

00:51:18
*Mme. 2:* Ben carrément.

00:51:19
*M. [Prénom du conjoint]:* Il y a endroit où ça se fait. Mais [euh] c'est des tout petits marchés, mais il y en a.

00:51:23
*Mme. 2:* 16 19 par exemple.

00:51:24
*M. [Prénom du conjoint]:* 16 19, le vendredi.

00:51:25
*Mme. 2:* Il y aura du pétrole.

00:51:26
*Mme. [Prénom de l’intervieweuse]:* Ouais, je pense, ouais.

00:51:29
*M. [Prénom du conjoint]:* [euh] Enfin--

00:51:29
*Mme. [Prénom de l’intervieweuse]:* Toutes les personnes qui ne vont pas le wee- avant le week…

00:51:31
*Mme. 2:* Oui avant le week end, après le travail, les vendredis, les gens finissent parfois plus tôt.

00:51:36
*Mme. [Prénom de l’intervieweuse]:* Oui.

00:51:37
*Mme. 2:* [euh] et puis [euh] c'est--

00:51:38
*M. [Prénom du conjoint]:* Il n'y en a pas trop--

00:51:38
*Mme. 2:* C'est pas que ça vous donne envie de cuisiner dans le week-end en plus, quoi.

00:51:41
*M. [Prénom du conjoint]:* Moi, j'y allais- j'y allais tous les dimanches matin. Il y avait plein marché à Tours, il y en avait le dimanche matin.

00:51:46
*Mme. 2:* Mais en Montauban aussi, il y en a plein de partout [euh]

00:51:49
*M. [Prénom du conjoint]:* Je ne sais pas où ils sont les marchés le dimanche matin, ils sont tous en semaine et c'est vrai qu'en semaine ben…

00:51:54
*Mme. [Prénom de l’intervieweuse]:* Ouais, si les producteurs qui sont-- Ils sont pas à Brest [euh] le dimanche matin. C'est Saint Louis c'est le-- Il y à plusieurs- plusieurs marchés dans la semaine à Brest, mais le gros de Saint-Louis.

00:52:06
*M. [Prénom du conjoint]:* C'est vrai que celui-là aussi, Saint-Louis, le dimanche matin, oui, l'intérieur de Saint-Louis.

00:52:11
*Mme. [Prénom de l’intervieweuse]:* Ah ! Il y a Saint-Louis aussi, Saint-Martin.

00:52:13
*M. [Prénom du conjoint]:* Saint-Martin, oui.

00:52:14
*Mme. [Prénom de l’intervieweuse]:* Il y a des halles, mais du coup, c'est fermé, forcé. À l'intérieur aussi, des magasins sont là à l'année. Mais le Grand Saint-Louis, il y a et ça brasse du monde-là. Il faut aimer [euh] être dans la foule et avoir le temps de flâner.

00:52:28
*Mme. 2:* Ouais, c'est ça. Moi, je j'adore.

00:52:32
*Mme. [Prénom de l’intervieweuse]:* Et les produits du marché, ils sont similaires aux autres ou ?--

00:52:37
*Mme. 2:* Non, les produits du marché, ils sont un peu assimilés à celui de la ferme hein.

00:52:41
*Mme. [Prénom de l’intervieweuse]:* Oui.

00:52:42
*Mme. 2:* Parce que souvent les marchés tels que je les connais moi, c'est des produits [euh], je vous dis qui arrivent du producteur. Généralement, c'est lui qui vous les vend d'ailleurs. Dans le Sud, vous avez même les- les producteurs d'huile d'olive. Voilà. C'est-- Ce sont des gens à qui appartiennent les moulins, en fait où les olives sont pressés et ils vendent le produit de-- des récoltes des gens quoi. Donc, bah, il n'y a pas photo. C'est- c'est le top en fait.

00:53:12
*Mme. [Prénom de l’intervieweuse]:* Et [euh] alors, du coup, si on reprend selon vous, les produits vont être de meilleure qualité [euh]. Dans le potager ou au marché ou à la ferme, ce sont les mêmes. Si vous êtes amené à jeter des produits, donc vous, c'est quelque chose que vous évitez un maximum. Est-ce qu'il y a une différence pour vous dans la façon dont vous allez vous sentir au moment de jeter le produit--

00:53:35
*Mme. 2:* Bien sûr que ça va m'embêter davantage de jeter un produit qui sort d'un producteur auquel j'ai acheté sur un marché ou de mon jardin ou [euh] du fermier parce qu'il y a pas la même- [euh], la même connotation derrière. C'est pas de la grosse échelle, c'est pas [euh], c'est quelqu'un qui a mis [euh] le temps pour faire [euh] pousser ce que vous êtes en train de manger, qui y a mis toute son attention et voilà, c'est du travail [euh] qu'il a effectué et rien que par respect pour ça déjà [euh]--

00:54:10
*M. [Prénom du conjoint]:* C'est impersonnel, c'est le mot, c'est ça impersonnel la grande surface. Alors qu'au marché, on a vu le prix, ou au producteur, on a vu le producteur enfin voilà c'est différent.

00:54:19
*Mme. 2:* Un peu qui vous a expliqué [euh]. Voilà ce que vous allez manger enfin, je veux dire, moi, ça m'est déjà arrivé de discuter avec ben le producteur d'huile d'olive, par exemple. Mais c'est un passionné. C'est un-- Il vous donne envie de faire de lui ça. Rien comme le bio. Je tends une bouteille, quoi. Et pareil pour les œufs. Moi, je me souviens de mes parents [euh] avant d'avoir eux-mêmes leurs poules tout ça. On allait acheter les œufs au marché. Vous en aviez toujours treize à la douzaine.

00:54:46
*Mme. [Prénom de l’intervieweuse]:* Oui.

00:54:47
*Mme. 2:* C'est le genre de trucs aller en grandes surfaces et demandé en treize si vous en avez douze quoi. Ben voilà, mais--

00:54:53
*M. [Prénom du conjoint]:* Ça en plus, c'est le kilo, c'est un kilo 100.

00:54:55
*Mme. 2:* Le persil chez le poissonnier, les citrons, il vous les donne ben, je veux dire c'est- c'est une autre approche, vraiment. Mais oui, c'est impersonnel. Mais chez le primeur, c'est un peu impersonnel aussi.

00:55:11
*M. [Prénom du conjoint]:* Oui, c'est- c'est un peu moins, mais ça l'est quand même hein.

00:55:15
*Mme. [Prénom de l’intervieweuse]:* Voyez sur une élève de graduation.

00:55:19
*Mme. 2:* Et ben, le plus bas, c'est la grande surface, après le primeur et après le marché quoi.

00:55:22
*Mme. [Prénom de l’intervieweuse]:* Ouais.

00:55:23
*M. [Prénom du conjoint]:* Et après, le producteur en hérite.

00:55:25
*Mme. 2:* Et ça, c'est le top du top.

00:55:27
*Mme. [Prénom de l’intervieweuse]:* Oui, vous avez directement...

00:55:28
*Mme. 2:* Maintenant, [Prénom de l’intervieweuse] [euh], elle sait que j'aime les courgettes qui sont pas trop grosses, elle me les garde, [euh] voilà les poireaux, les trucs comme ça. Elle sait que j'aime les petits, elle me les garde. Enfin, j'ai même réussi à avoir des fleurs de courge

00:55:42
*Mme. [Prénom de l’intervieweuse]:* Ok.

00:55:43
*Mme. 2:* Truc qui- qui- qui-- Vous trouvez ça-- Vous trouvez pas ça ici normalement. Ben, elle me les mets de côté, quoi, parce qu'elle sait que ça me fait plaisir et que je les cuisine donc, oui.

00:55:55
*Mme. [Prénom de l’intervieweuse]:* Et en grande surface, si vous voulez du persil, vous l'achetez ?

00:55:57
*Mme. 2:* Oui.

00:55:58
*Mme. [Prénom de l’intervieweuse]:* Le céleri aussi.

00:55:59
*Mme. 2:* Oui.

00:56:01
*Mme. [Prénom de l’intervieweuse]:* Et donc [inaudible 00:56:02-00:56:03]. Si vous êtes amenés à la perdre, à la jeter, ça vous embête plus que ça?

00:56:07
*Mme. 2:* Ouais ça va me faire chier.

00:56:08
*Mme. [Prénom de l’intervieweuse]:* Ouais.

00:56:11
*M. [Prénom du conjoint]:* Mais alors qu'elle ne te les a pas vendus.

00:56:13
*Mme. 2:* Elle ne me les a pas vendus, elle me donne.

00:56:15
*Mme. [Prénom de l’intervieweuse]:* Donc ce n'est même pas le fait de les avoir acheté. [rire]

00:56:17
*Mme. 2:* Non.

00:56:17
*Mme. [Prénom de l’intervieweuse]:* C'est pas le fait de les avoir achetés.

00:56:19
*Mme. 2:* C'est le fait de les perdre tout court. Ce que ça représente pour- pour moi, pour elle [euh]. Enfin, moi, je sais que j'ai un énorme respect pour eux parce que ils travaillent tout le temps et c'est des gens qui sont hyper courageux. Ils sont plus jeunes que nous, mais en plus, ils ont un tout petit bout. C'est tout ce qu'il y a autour aussi, quoi. On ne fait pas que leur acheter des légumes. Ils sont un peu rentrés dans nos vies aussi. Ça fait combien ? Trois ans que je vais chez eux. Et [euh] voilà, ils sont des gens qui ont voulu se lancer dans le bio. Ils ont tenu bon et tout [euh] parce qu'ils ont eu des menaces, des- des voisins. Parce que là-bas, à Saint-Pol-de-Léon, il n'y a que ça. Des agriculteurs, mais il y a pas beaucoup des bio hein.

00:56:56
*Mme. [Prénom de l’intervieweuse]:* Oui.

00:56:57
*Mme. 2:* Quand vous avez le courage de vous lancer dans le bio et que ça marche en plus, vous avez d'énormes pressions. Donc ils ont, eux fait le choix d'arrêter le bio, mais d'être en agriculture hyper raisonnée la machin avec leur label bidule-là. Et ils s'y retrouvent mieux parce qu'on leur fout la paix maintenant avec ça. Mais [euh] je trouve ça- ça enfin dingue quoi.

00:57:18
*Mme. [Prénom de l’intervieweuse]:* Le combat pour faire quelque chose de bien.

00:57:20
*Mme. 2:* C'est ça. Et en fait de pas pouvoir.

00:57:22
*M. [Prénom du conjoint]:* Ouais ben ici, [euh] Maïlys avait eu les mêmes problèmes hein.

00:57:25
*Mme. 2:* Maïlys ouais.

00:57:26
*M. [Prénom du conjoint]:* Qui fait vraiment avec son âne etc., le tout sur de petites parcelles. Et il y a eu [euh]- il y a eu [euh] enfin la violence.

00:57:32
*Mme. 2:* Ils lui ont saccagé son champ.

00:57:34
*M. [Prénom du conjoint]:* Saccagé son champ et tout ça parce que ça les fait chier.

00:57:36
*Mme. [Prénom de l’intervieweuse]:* C'est incroyable.

00:57:38
*M. [Prénom du conjoint]:* Mais ça mettra du temps.

00:57:39
*Mme. [Prénom de l’intervieweuse]:* Oui. Oui, [inaudible 00:57:40-00:57:41] Il y a tout- tout- tout ce qu'il y a autour de la personne qui- qui a produit le- le produit qui entre en jeu quand vous- même quand vous le préparez, peut être quand vous--

00:57:57
*Mme. 2:* Disons que si je perd un--

00:57:59
*M. [Prénom du conjoint]:* Ah oui, c'est sûrement hein parce que moi, je cuisine un peu les légumes, mais pas trop avant hein. Et maintenant, cuisiner les légumes quand ça vient de la ferme, etc., je ne-- c'est différent que les légumes des grandes surfaces que de prendre le filet en haut des courgettes. Là, c'est complètement autre chose.

00:58:13
*Mme. [Prénom de l’intervieweuse]:* Vous préférez cuisine ?

00:58:15
*M. [Prénom du conjoint]:* Je préfère cuisiner maintenant, j'ai le plaisir à cuisiner les légumes avant de cuisiner un peu d'autres trucs que les légumes, moi.

00:58:21
*Mme. 2:* C'est ce que j'allais vous dire un peu, parce que Mm-mm, j'allais vous dire en me disant que quand je perds un produit qui vient de grandes surfaces, c'est l'aspect économique qui va primer. C'est que merde j'ai payé un truc qui- qui est pas viable quand je perds un fruit ou un légume, que j'achète [euh] a un producteur quel qu'il soit, que ce soit la ferme, mon jardin ou le marché. C'est toute la dimension [euh]--

00:58:47
*M. [Prénom du conjoint]:* Humaine.

00:58:48
*Mme. 2:* Humaine, en fait, voilà qui prime. Et comme c'est celle qui m'anime, moi, au quotidien, forcément, ça va me toucher plus. [silence 00:58:55-00:59:05]

00:59:06
*Mme. [Prénom de l’intervieweuse]:* Ok. Alors, Merci ce petit jeu est terminé. [rire] [diaphonie 00:59:18-00:59: 10]

00:59:21
*Mme. 2:* C'est peut-être plus la peine de chercher--

00:59:23
*M. [Prénom du conjoint]:* Si si il est allé les chercher. Il n'a pas trouvé l'endroit, je pense.

00:59:27
*Mme. 2:* Il n'a pas envoyé de message. Ah ! J'ai un message. Bien.

00:59:30
*M. [Prénom du conjoint]:* Il y a 4 heures environ, il n'a pas réussi à sonner ?

00:59:35
*Mme. 2:* J'ai eu un contretemps. Je ne serai là que vers 16 heures 30.

00:59:38
*Mme. [Prénom de l’intervieweuse]:* Ok.

00:59:39
*M. [Prénom du conjoint]:* Alors.

00:59:40
*Mme. 2:* Pas de souci. Pierre-Louis- Pierre-Louis

00:59:44
*Mme. [Prénom de l’intervieweuse]:* C'est ça oui.

00:59:51
*Mme. [Prénom de l’intervieweuse]:* Ici !

00:59:52
*Mme. 2:* Oui une banane bien mûre.

00:59:54
*Mme. [Prénom de l’intervieweuse]:* Nous en avons dix.

00:59:57
*Mme. 2:* Ouh là, d'accord.

00:59:59
*Mme. [Prénom de l’intervieweuse]:* Je vais vous demander de les ranger. Celle qui vous donne le plus envie, celle qui vous donne le moins envie.

01:00:04
*Mme. 2:* D'accord. [silence 01:00:05-01:00:18]

01:00:24
*Mme. 2:* C'est une banane ça ou c'est un truc qui sort de la planète Mars ?

01:00:27
*Mme. [Prénom de l’intervieweuse]:* C'est une banane verte. [rire]

01:00:36
*Mme. 2:* Du plus au moins.

01:00:36
*Mme. [Prénom de l’intervieweuse]:* Ouais. [silence 01:00:37-01:00:59]

01:01:01
*M. [Prénom du conjoint]:* Moi, j'aime bien quand elles sont bien mûres.

01:01:02
*Mme. 2:* Mais oui, mais moi aussi. Mais celle-là, elle est vraiment bien bien-- Celle-là, moi je l'utilise dans les gâteaux par exemple, je la mange pas comme ça.

01:01:10
*Mme. [Prénom de l’intervieweuse]:* Mais elle est [euh] comme enfin n'importe quoi. On n'a pas encore un homme. Vous allez dire justement ce que vous faites de chaque- [euh] de chaque banane. Elles ont un petit repère, une forme- une forme et une couleur à côté pour que, sur l'enregistrement, je puisse reconnaître ce que vous dites.

01:01:29
*Mme. 2:* Franchement, pour moi, ça, c'est pas une banane, c'est un- c'est un truc qui sorte je sais pas où, mais pas une banane. Enfin, moi, je mange pas ça hein. Alors voilà, on va dire que ces trois-là--

01:01:44
*Mme. [Prénom de l’intervieweuse]:* Je prends en photos montrant au moins une partie [euh] voilà. Ce serait ça.

01:01:55
*Mme. 2:* À peu près.

01:01:57
*Mme. [Prénom de l’intervieweuse]:* À peu près. Merci. Alors si on commence par la première.

01:02:04
*Mme. 2:* En fait, on peut commencer par les trois premières, celles si je les mange.

01:02:08
*Mme. [Prénom de l’intervieweuse]:* Donc vous les manger tel quel. On a le triangle vert, le carré orange et le rond blanc. Ces trois-là mélangées--

01:02:18
*Mme. 2:* Sans problème. Celle-ci, je pense qu'elle pourrait aller aussi-là.

01:02:23
*Mme. [Prénom de l’intervieweuse]:* Le carré blanc, ok .

01:02:24
*Mme. 2:* Le carré blanc, ça va. Tout ça, on mange.

01:02:28
*Mme. [Prénom de l’intervieweuse]:* Ok.

01:02:30
*Mme. 2:* Ça, c'est pas mûr.

01:02:31
*Mme. [Prénom de l’intervieweuse]:* Le triangle bleu pour eux, il n'est si mûr.

01:02:33
*Mme. 2:* Oui, il n'est pas si mûr, il y a encore du vert, tandis qu'une banane, pour qu'elle soit bonne, il faut qu'elle soit quand même un peu tachetée. Sinon, c'est qu'à l'intérieur, elle n'est pas mûre.

01:02:41
*Mme. [Prénom de l’intervieweuse]:* Ok.

01:02:42
*Mme. 2:* Et le goût se sent aussi. Si vous voulez un vrai goût de banane, il faudra vous rapprocher de ça que de ça quoi.

01:02:50
*Mme. [Prénom de l’intervieweuse]:* D'accord. Et c'est quoi la différence de goût. Son goût de la banane.

01:02:55
*Mme. 2:* Elle est plus sucrée, en fait, elle devient plus sucrée quand elle est plus mûre et plus molle aussi.

01:03:00
*M. [Prénom du conjoint]:* Ouais, les enfants ont tendance à les prendre presque un peu. Un peu, pas le mur.

01:03:04
*Mme. 2:* Oui, les enfants, ils aiment bien ça.

01:03:05
*M. [Prénom du conjoint]:* Voilà, ils aiment la texture. En fait, c'est une question de texture. Les enfants aiment la texture un peu plus, mais ça commence un peu à être fondant dans la bouche. Là, c'est moins, mais le goût est plus- plus présent.

01:03:15
*Mme. [Prénom de l’intervieweuse]:* Ils préfèrent soit encore un peu trop croquant plus que ça.

01:03:19
*Mme. 2:* Oui, voilà.

01:03:20
*M. [Prénom du conjoint]:* Un peu plus ferme.

01:03:21
*Mme. 2:* Un peu plus ferme.

01:03:22
*Mme. [Prénom de l’intervieweuse]:* Un peu plus ferme. Eux, ils mangeaient le triangle bleu.

01:03:25
*Mme. 2:* Les enfants [euh] Triangle bleu sans soucis.

01:03:27
*Mme. [Prénom de l’intervieweuse]:* Et vous, vous faites quoi face au triangle ?

01:03:29
*Mme. 2:* Moi, si j'ai un triangle bleu, j'attends qu'elle mûrisse en fait. J'attends qu'elle devienne carré blanc.

01:03:34
*Mme. [Prénom de l’intervieweuse]:* Ok, au moins

01:03:36
*Mme. 2:* Au minimum.

01:03:38
*Mme. [Prénom de l’intervieweuse]:* Ok, [euh] le carré bleu, vous faites quoi avec ?

01:03:42
*Mme. 2:* Alors le carré bleu, moi, je les mange aussi, mais soit j'en fais de la compote, soit j'en fais des gâteaux ou de la confiture.

01:03:48
*Mme. [Prénom de l’intervieweuse]:* Ok.

01:03:48
*M. [Prénom du conjoint]:* Oui une banane écrasée.

01:03:49
*Mme. 2:* Ou une banane écrasée avec du sucre et du citron.

01:03:52
*Mme. [Prénom de l’intervieweuse]:* Ok, mais pourquoi vous- vous décidez de la transformer à ce moment-là ?

01:03:56
*Mme. 2:* Parce que je sais que les enfants ne la mangeront pas-t-elle. Déjà parce que quand vous allez l'ouvrir, elle va se déliter.

01:04:02
*Mme. [Prénom de l’intervieweuse]:* Ouais.

01:04:03
*Mme. 2:* Et [euh] parce que le goût très sucré et la texture très molle en fait [euh] de l'aliment, fera que je vais même pas essayer de leur proposer en salade de fruits par exemple.

01:04:12
*Mme. [Prénom de l’intervieweuse]:* Ok, donc c'est pour garder le- le goût de la banane, mais pas sous la forme.

01:04:18
*Mme. 2:* Voilà, c'est ça.

01:04:19
*Mme. [Prénom de l’intervieweuse]:* Et vous, vous serez capable de la manger [euh] cru ou--

01:04:22
*M. [Prénom du conjoint]:* Ah ! Oui.

01:04:23
*Mme. [Prénom de l’intervieweuse]:* Ok.

01:04:23
*Mme. 2:* Ouais, ouais.

01:04:26
*M. [Prénom du conjoint]:* Ouais, c'est quoi ? [inaudible 01:04:27-01:04:32]

01:04:33
*Mme. [Prénom de l’intervieweuse]:* Oui, c'est enregistré, mais tu peux parler. [rire] [euh] Alors le rond [euh] jaune orange, là, qu'est-ce que vous en penser ?

01:04:44
*Mme. 2:* Ouais, je l'assimile un peu aussi. Il faut voir à l'intérieur comment elle est.

01:04:48
*Mme. [Prénom de l’intervieweuse]:* Ok.

01:04:49
*Mme. 2:* Si à l'intérieur elle est encore à peu près saine et que je peux en faire un-- la même chose que le carré bleu, je l'utiliserai. Si elle est abîmée, en revanche, je vais pas la garder.

01:04:59
*Mme. [Prénom de l’intervieweuse]:* Ok.

01:04:59
*Mme. 2:* Voilà.

01:05:00
*Mme. [Prénom de l’intervieweuse]:* Ça marche. Et celle d'après ?

01:05:02
*Mme. 2:* Mais concrètement, l triangle blanc et le rond rose [euh] C'est difficilement mangeable, je pense.

01:05:09
*Mme. [Prénom de l’intervieweuse]:* Ouais.

01:05:09
*Mme. 2:* Voilà. C'est qu'elle est- elle est passée, quoi.

01:05:11
*Mme. [Prénom de l’intervieweuse]:* Ouais.

01:05:12
*Mme. 2:* Voilà, et ça, pour moi, je persiste à dire que c'est pas une banane [rire] je- je sais pas d'où elle sort, mais fallait la laisser sur l'arbre, en fait.

01:05:21
*Mme. [Prénom de l’intervieweuse]:* C'est- c'est un trucage du verre parce que[euh] il y avait pas de plus vert. Les personnes qui aiment le vert. Mais c'est que on voit que c'est pas très vert. C'est un peu artificiel ça. [rire] C'est une bonne verte. Voilà. Et vous, vous allez comme ça mettons les vertes.

01:05:37
*Mme. 2:* Mais je l'achète même pas moi là.

01:05:39
*Mme. [Prénom de l’intervieweuse]:* Ah ! Oui.

01:05:39
*Mme. 2:* Ouais, non.

01:05:40
*Mme. [Prénom de l’intervieweuse]:* En vert comme ça ?

01:05:40
*Mme. 2:* Ah ! Non, elle est- elle est-- C'est non. Non. C'est même pas qu'elle n'est pas assez mûre. C'est qu'elle mûra jamais votre banane. Elle va abîmer avant même que vous ayez le temps de la manger, quoi. [rire]

01:05:55
*Mme. [Prénom de l’intervieweuse]:* Ok [euh] Mm-hmm, selon vous, est-ce qu'il y a un moment donné où le goût, il se transforme, de la banane ?

01:06:03
*Mme. 2:* Je pense entre ces deux-là déjà.

01:06:05
*Mme. [Prénom de l’intervieweuse]:* Entre le rond blanc

01:06:06
*Mme. 2:* Entre celle-là là.

01:06:08
*Mme. [Prénom de l’intervieweuse]:* Ouais.

01:06:08
*Mme. 2:* Ici, vous avez un goût très, enfin, quand elles sont comme ça, souvent, les bananes, elles sont un peu âpres. Elles vous laissent un petit goût [euh] en bouche après l'avoir [euh] avalé.

01:06:21
*Mme. [Prénom de l’intervieweuse]:* Ouais.

01:06:22
*Mme. 2:* Quand elle est là, c'est [euh] le carré blanc. En fait, c'est [euh] le plus classique. On va dire hein c'est le goût que le-- que les gens connaissent le plus de la banane, c'est ça, en fait.

01:06:32
*Mme. [Prénom de l’intervieweuse]:* Ok.

01:06:33
*Mme. 2:* C'est le carré blanc, mais le goût qui- qui- qui est le plus proche, je pense du fruit en lui-même peut-être qu'ils le mangent aux Antilles, par exemple, c'est rond Blanc.

01:06:44
*Mme. [Prénom de l’intervieweuse]:* C'est le rond blanc.

01:06:44
*Mme. 2:* Voilà. Sachant qu'entre le rond blanc, le carré orange et le triangle vert. Ouais, c'est subtil quoi.

01:06:53
*Mme. [Prénom de l’intervieweuse]:* Ouais.

01:06:54
*Mme. 2:* Voilà, c'est pas- c'est pas hyper différent au niveau du goût, mais par contre le carré bleu, lui, il a vraiment un goût très sucré.

01:07:03
*Mme. [Prénom de l’intervieweuse]:* Ok.

01:07:07
*Mme. 2:* Voilà. En fait la saveur de la banane. Je pense qu'elle- elle se développe au- entre le rond blanc et le carré bleu.

01:07:15
*Mme. [Prénom de l’intervieweuse]:* Ok, et après le carré bleu en termes de goût, comment ça se passe selon vous ?

01:07:21
*Mme. 2:* Après, pour moi, c'est pas une question de goût trop. Entre le carré bleu et le rond jaune. C'est plus une question de matière.

01:07:28
*Mme. [Prénom de l’intervieweuse]:* La texture.

01:07:29
*Mme. 2:* La texture. [inaudible 01:07:30-01:07:48]

**Household F30, Interview 1**

**Alors, dans un premier temps, je vous propose un petit jeu comme une devinette, je vais vous demander de penser à un légume ou à un fruit que vous aimez tout particulièrement. Vous allez pensé à ce légume ou ce fruit sans révéler son identité. Le but est que je le devine uniquement grâce à la description que vous allez en faire.**

Ça, c'est difficile parce que j'aime beaucoup tous les légumes.

**Ah ! Est-ce qu’il y en a un**

Bon, oui voilà

**Alors je vous écoute, dites moi tout ce qui me vient à l'esprit sur ce légume ou ce fruit**

Comment est ce que je pourrais vous en parler ? Tout d'abord, c'est un légume, effectivement, on en profite toute l'année. Il apporte de la provitamine A, du sucre aussi, mais modérément. On peut même si on l'achète avec des fanes, certain moment, employer les fanes pour faire du potage, par exemple, et on peut le préparer de mille et une façons. Qu’est-ce que je peux vous dire encore ? Pas grand chose, pas grand chose d'autre.

**Qu'est ce que vous aimez particulièrement dans ce légume ? Qu'est ce que vous aimez tout particulièrement dans ce légume ?**

Et bien son goût, sa consistance, sa mache et qu'on peut le consommer crus ou cuits et que du point de vue, de comment dirais-je des bienfaits, il en apporte quand même pas mal quoi. Et j'adore son goût de toute façon !

**Donc déjà ! *(rires)* En général vous le mangez plutôt quand dans la journée, plutôt le matin, midi… l'après midi le soir, tout le temps… ?**

Je suis assez conventionnelle. J'ai un petit déjeuner, mais qui comporte surtout une boisson chaude, le thé que je sucre le matin, avec une cuillerée de miel et après pour les boissons, il y a plus de sucre *(rires)* et 2 tartines, une de beurre et une de confiture. Après, j'ai un repas à midi que je prends à 13 heures d’ailleurs ! C’est mes horaires et donc bon légumes, protéines soit poisson soit viande. Un goûter vers 5 heures où j’ai un fruit et une boisson, froide en été, c’est plutôt de l’eau et chaude jusqu’à maintenant *(rires)* Et puis deux carrés de chocolat qui sont un peu plus que deux ! *(rires)* Et le repas du soir que je prends entre sept heures et demie et 8 heures et qui comprend toujours un potage de légumes et du légumes broyés, en quelque sorte vous voyez ?

**Oui, donc si vous consommez ce légume, c'est plutôt le midi ou le soir en potage ?**

Il est toujours dans le potage le soir et plusieurs fois…. Plusieurs fois par semaine en légume. J'aime bien, le préparer en… à la marocaine avec du cumin, c’est très bon.

**Ok… En général, vous l'achetez où ce légume ?**

Alors j'ai deux endroits épicerie bio

**D’accord..**

Mais ça m'arrive aussi de l'acheter en grande surface tout à fait au printemps, quand il a ces fanes justement quand les bottes ont les fanes.

**D'accord… Est ce que vous pouvez me raconter quand vous avez votre légume, qu’-est ce que vous faites avant de pouvoir consommer ? C'est quoi les différentes étapes qu'il va y avoir ?**

En général, je commence par laver. Je l’épluche et je le relave *(rires)* et ensuite… où je le rappe, mais c'est de plus en plus rare que je fasse des carottes râpées parce que… oh bah je vous ai dit le légumes !

***(rires)* Je l’avais !**

Les carottes rapées, parce que je digère moins bien les légumes crus qu'autrefois. Et après… Quand je fais des carottes en potage, quand je mets en potage, je la coupe en deux ou trois et quand je le fais, au cumin, justement, je le coupe en rondelles. Je le mets aussi quand je fais une jardinière de légumes, évidemment, en petits morceaux, en jardinière de légumes.

**Ok… Vous l’épluchez systématiquement la carotte ? ?**

Euh oui… enfin quand elles sont vraiment toutes nouvelles nouvelles nouvelles, je gratte. Mais enfin, bon j’enlève… oui.

**Et du coup, vous me parlez des fanes, ça vous arrive de les utiliser ?**

De les utiliser dans le potager oui

**D'accord. A chaque fois, vous utilisez les fans et vous les mettez dans le potage ou c’est de temps en temps ?**

Oh, c'est de temps en temps, de temps en temps.

**De temps en temps.**

C'est d'ailleurs la saison des carottes avec fanes dure pas très, très longtemps. Mon corps ne

**D’accord, alors vous m'avez parlé des avantages de ce légume avec son apport, son goût, qu’on le prépare de plein de façons.. Est-ce que vous lui trouvez en revanche des inconvénients ou pas, à ce légume ?**

Moi, je n'ai trouvé aucun inconvénient.

**Ok, il est parfait.**

Franchement non

**Et du coup, vous en consommez toute l'année ?**

Oui, toute l’année.

**OK. C'est donc la carotte *(rires)*Alors là, si on s'intéresse à vous, à votre organisation en termes de courses alimentaires qu'au sein de votre foyer qui va faire les courses alimentaires ?**

Moi je vis toute seule ! *(rires)*

**Et du coup, vous pouvez me raconter comment vous vous organisez ?**

Comment je m'organise… Ecoutez, quand il y a pas de.. confinement et quand je vais régulièrement à mes…. activités à la piscine de Lanilis, j'en profite et je vais au magasin bio de Lanilis. Et c'est là que j'achète mes fruits et légumes, en particulier et que je fais mes courses alimentaires. Bon, si j'ai besoin de… Et si j'ai besoin d'aller par ailleurs acheter quelque chose d’alimentaire, j'essaye… Je vais à la petite épicerie de Lilia, où il y a aussi beaucoup, beaucoup de bio, mais alors l'inconvénient du bio, c'est que ça m'arrive assez souvent d'avoir des fruits qui s'abîment parce que ça s'abîme beaucoup plus vite. Ça, j'avoue… Malheureusement, en ce qui concerne la viande et le poisson, je dois aller… je vais à la grande surface à l’Intermarché parce que à part quelques… quelques éléments, par exemple je sais que un bifteck en tournedos, je l'ai congelé à l'épicerie bio ici à Lilia. Et autrement, du point de vue poisson, je ne peux avoir que le saumon fumé. Enfin bon, la truite fumée, ces choses comme ça. Donc, pour ça, c'est la grande surface.

**Et donc, ça vous arrive parfois, vous m’avez dit pour les carottes que ça peut vous arriver quand c'est la saison avec les fanes, d'acheter aussi, du coup, des légumes en grandes surfaces quand vous y allez ?**

Ca arrive quand je ne trouve pas, par exemple, les patates douces, c’est… Ça m'arrive de les prendre en grande surface oui.

**D’accord et vous vous parlez d'épicerie bio ? Qu'est ce qui fait que vous souhaitez consommer les fruits et légumes, notamment en bio ?**

Euh… (*le téléphone sonne)*

**Allez-y il y a pas de souci. *(…)* Alors du coup, je vous demandais pourquoi vous privilégiez les épiceries bio, notamment pour vos fruits et légumes ?**

Et bien, parce que je pense que c'est meilleur, parce que ça évite les pesticides, les… Et tout ce qui peut...

**Donc c'est meilleur pour…**

C’est meilleur pour ne pas avaler de pesticides, justement, dont certains sont, parait-il cancérigènes, d'autres peuvent apporter d'autres ennuis

Mh donc c’est pour votre santé que vous privilégiez, notamment le bio ?

Bah oui.

**OK, alors quand vous partez faire vos courses, est ce que vous savez ce que vous allez acheter ou pas vraiment ?**

En tout cas, j'ai en gros…, en gros, par exemple j’ai marqué légumes, légumes, fruits, poissons, etc. Oui, je fais, je fais une liste et quand je la regarde pas des fois j’oublis *(rires)*

***(rires)* donc c'est plutôt écrit légumes, fruits. Mais c'est pas détaillé, forcément**

Non parce que ça dépend de ce que je vais trouver aussi

**D’accord… Et comment vous gérez les quantités ? Du coup, quand vous allez acheter, quand vous êtes face aux étalages de fruits et légumes, comment vous gérez vos quantités ?**

Ben… avec les bio, je fais attention, j'en prends assez peu parce que justement, j'ai des ennuis. La semaine dernière, par exemple, j'ai eu deux énormes citrons qui sont passés à la poubelle, à votre poubelle ! Et ils étaient en l'espace de trois jours, ils étaient moisis. Ça m'a fait un peu grincer des dents ! *(rires)*

**Donc les fruits bio, vous essayez de prendre en petites quantités**

En petites quantités oui.

**Et vos légumes ? Comment vous gérez vos quantités ?**

Et bien là aussi, je prends petite quantité, par exemple quand je prends du brocoli et qu'il est en tête assez grosse et bien j'ai des fois un peu des problèmes aussi.

**D’accord…**

Alors j'essaye de jamais… mais l'autre jour, j'ai dû jeter. Il m'en restait deux petites têtes qui avaient moisi parce que je les avais... J'avais attendu trop longtemps, trop longtemps, parce que j'en avais vraiment… avec eux quand ils sont emballés en plus, c'est en assez grosse proportion. Et c'est trop quoi. Et ça, quand on est seul, on a vraiment des problèmes parce que c'est pareil. Tout ce qui est emballé… Jamais vous n'avez jamais des cuisses de poulet une par une. Vous n'avez jamais de…. Donc là, quand je prends, je prends 2, j’en prépare une et je congèle l'autre forcément.

**Ouais… D'accord, et donc, vous disiez que, c’est quand vous alliez à la piscine avant notamment la période du covid et sinon en termes de fréquence, vous vous rendez acheter des fruits et légumes tous les combien à peu prêt ?**

Sûrement deux fois par semaine,

**Deux fois par semaine ? Et est-ce que vous privilégiez un jour dans la semaine, c'est assez régulier, ou plutôt quand**

Et bien quand c'est à la piscine, c'est régulier, c'est… autrement bon. Quand je passe ici c'est…. De toute façon, déjà le lundi. Ni l'un ni l'autre n'est ouvert alors *(rires)*

**D’accord déjà *(rires)* OK. Ca marche. Alors là si on essaie de dresser une liste des différents fruits et légumes que vous avez l'habitude de consommer très régulièrement, ce qu'on a vraiment retrouvé chez vous souvent comme la carotte.**

Comme la carotte, le fenouil

**D’accord**

Le brocoli… Qu'est ce que je consomme très régulièrement ? Et bien de la salade bien sûr. En général, de la feuille de la feuille de chêne et si possible rouge *(rires)* des pommes de terre, oui aussi. De la patate douce… Alors quand c'est la saison des épinards au moins une fois par semaine si, ce n’est pas plus. Qu'est ce qu'il y a d'autres encore ? Alors, en saison, en saison… J'essaye d'avoir des petits pois… Et puis, plus tard, des haricots verts en saison aussi. Qu'est ce que je consomme régulièrement… Ah si les courgettes, les tomates, les poivrons et les poivrons, je les consomme même l'hiver. Mais à ce moment-là, ils sont en conserves, en bocaux dans de l'huile.

**Sinon, la courgette tomate, vous attendez la saison ?**

Ah oui j’attends la saison.

**D’accord. Est ce que vous avez en tête des légumes que vous connaissez et que vous ne consommez que très, très rarement, voire jamais ?**

Les artichauts parce que je les digère pas.

**D’accord….**

J'adore ça ! Mais je ne digère pas. La seule façon que j'ai de les consommer, c'est pareil, c'était les Italiens qui font ça… en les cœurs en bocaux.

**Ça vous les digérez ?**

Ca, ça, ça passe encore. Je ne sais pas pour quelle raison, mais en tout cas… Les radis, je ne peux pas en manger non plus,

**C’est pareil, c'est pour des raisons**

Oui c’est pour des raisons digestives. Ils passent pas du tout. Quoi encore ? Le poireau, je ne le manche qu'en potage parce que autrement… Je mangerai le vert, mais le blanc passe pas, il est trop sucré. J'aime pas du tout, et ça depuis que je suis gamine.

Vous aimez le vert, mais pas le blanc ?

Oui

**C'est original, je dois vous l’avouer *(rires)* Et donc dans le potage, vous mettez tout ?**

Oui dans le potage, je mets tout. Mon mari adoré ça, je lui en faisais, je mangeais un peu de vert, mais.. *(rires)* A les asperges, j'aime beaucoup en revanche, j'ai oublié de vous parler des asperges oui, à la saison des asperges.

**En saison ouais. Les courges, ce que ça vous arrive de consommer des courges ?**

Alors j’aime bien le potimarron, la… La courge butternut, mais les autres grosses courges je n'aime pas du tout, j’en mange pas.

**OK. Qu'est ce qu'on a d'autre… L'aubergine ? Est ce que ça vous arrive ?**

Oui, mais oui, j'en mange quand je fais de la ratatouille je mange de l'aubergine.

**La betterave, est ce que ça vous arrive…**

la betterave ? J'en mange beaucoup, j’avais oublié, j'avais oublié à la saison de la betterave. La betterave rouge, j'aime beaucoup oui.

**D’accord.. donc finalement, vous consommez pas mal de légumes différents, sauf l'artichaut, radis, pour des problèmes digestifs et le poireau par manque d'appréciation du goût du blanc. OK, si on s'intéresse aux fruits**

Ça m'arrive aussi, mais assez rarement parce que c'est très gros maintenant, etc. de manger du chou, mais par exemple… Mais Le chou rouge quand j'invite l'hiver. J'en fais volontiers mais cuit, je ne mange jamais de chou rouge cru, ça non plus, ça passe... Ça passe pas, trop difficile à digérer.

**Ouais, je vois.. Et le chou fleur, ça vous arrive de**

Alors le chou fleur, ça fait des années que je ne peux pas en manger.

**Pareil pour les mêmes raisons ?**

Parce que le chou fleur oui c’est

**Ah, c'est trop gros ?**

Mais non, ça passe pas

**Ah ça ne passe pas, même cuit ?**

Ben non. Quand, mon mari était vivant, j'en faisais, j'en faisais en gratin ou autre mais moi, j'en mangais très peu quoi parce que j'ai des difficultés à… C'est comme les artichauts, je gonfle ! *(rires)*

**Ah ouais, ça fait des ballonnements !**

Des flatulences et..

**Oui. Du coup, quand on peut éviter...**

C'est pas…

**Très agréable ! J’imagine**

Non c’est pas agréable même quand on vit seule ! *(rires)* C'est marrant parce que le brocoli ou le romanesco, ça m'arrive de prendre aussi du romanesco, mais de temps en temps, ça ne me fait pas cet effet là. C'est curieux. Alors que le chou fleur …

**Ah et les champignons. Est-ce que ça vous arrive d'en consommer ou pas ?**

Ça m'arrive assez rarement. J’en mets… quand je fais un plan en sauce. Comme je ne sais pas ce que je pourrais vous dire des blanquette ou des… mais c'est le genre de choses que je fais de moins en moins parce que toute seule, c'est pas évident. Mais si ça m’arrive. J'aime, j'aime bien.

**Ça marche. Alors, les fruits… quels sont les fruits que vous allez consommer très régulièrement ?**

Suivant la saison ! Donc l'hiver, c’est pommes, poires, banane. J'hésite de plus en plus parce qu'elles viennent de loin, mais je prends systématiquement les antillaises parce que c'est quand même la France ! *(rires)* Qu'est ce qu'il y a encore en hiver ?

**On retrouve des fois des kiwi, est-ce que vous en consommez des kiwi ?**

Les kiwi, oui. Oui, mais je commence à manger des kiwis quand ils sont français.

**D’accord, vous attendez la saison.**

De Nouvelle-Zélande, non. Et pendant très longtemps, j'ai consommé des kiwis bretons parce que j'avais des cousins qui avaient un immense kiwi Quimerc'h, dans le village où on est né et bon, ils étaient plus âgé que moi. Ils ont disparu tous les deux. On ne va plus à l'automne avec ma sœur cueillir des kiwi. *(rires)*

**Oui et ça peut... Quand il y en a, il y en a plein d'un coup.**

Oui, oui, mais ça se garde longtemps. Ça se conserve longtemps. Vous pouvez en manger.

**Mes parents en avaient dans notre ancienne maison et quand ils étaient là, on était obligé de donner à tous les voisins, ça pousse bien ! *(rires)*** Donc, pommes, poires, bananes, kiwis

L'hiver.

**L’hiver…**

Et puis bon, maintenant maintenant, il y a fraises. Je fais des confitures de fraises, d'ailleurs. J’en fais J’en ai fait dix pots là, la semaine dernière. Oui, et c'est d'une première tournée, j'espère que j'en ferai d’autres *(rires)*

**D’accord, donc les fraises, en confiture.**

Fraises et donc abricots et abricots, pêches, cerises. Mais c'est… quand je dis pêche. C’est pêches et nectarines hein d’ailleurs. C’est un bon… Melon et bon bah… Je ne vois pas. Je vois pas d’autres.

**C’est déjà pas mal ! Et en hiver, vous parliez de citron. Sur les agrumes. Est ce que vous en consommez, des agrumes ?**

Mandarine, citron oui citron c'est toute l'année. J’en mets dans les sauces, j'en mets dans les machins. Je me fais un citron pressé. Ca, c'est tout le temps, mais je ne consomme pas beaucoup, de temps en temps des… comment des pomelos, machin mais pas d'orange

**D'accord.**

J'aime pas l'orange.

**D'accord, c’est par goût**

C'est un peu… C'est bizarre, mais les mandarines, oui, mais pas l'orange.

**D’accord et clémentines ?**

Mandarines ou clémentines, c'est pour moi, c'est

**Oui c’est assez similaire**

Et je consomme aussi des fruits secs, des raisins secs. Amandes, noisettes, noix de cajou. Et puis, bien sûr, les noix, les noix noix. Alors justement. Il y a une discordance entre deux, deux nutritionnistes. Il y en a un, le docteur Fischer que je vois souvent à mon émission médicale de l'après midi, qui dit que des oméga 3, il y en a que dans les noix.

**D'accord.**

Et le docteur Cohen qui a fait un énorme bouquin Nutrition et machin truc et tout ça, prétend que dans tous les toutes les graines, dans tous les machins, il y a des oméga 3. Ils ne sont pas d'accord *(rires)* Bon, bref

**Du coup vous mangez un peu de tout ?**

Ben oui, je mange un peu. De toute façon. Et puis, il y a les noix de cajou, les noix, les noix, noix, noix, la noix de Grenoble.

**Et du coup, le raisin. Vous ne mangez que le raisin sec ou**

Alors le raisin j'en mange oui, à la saison, justement, donc le raisin, ça ne sera pas avant fin août septembre. Si si le raisin, et plutôt le raisin noir que le raisin blanc.

**D'accord. Ok… est ce que vous avez en tête des fruits que vous connaissez, mais que vous allez très rarement consommer ?**

Ben oui, ça m'arrive l'ananas, par exemple.

**Qu'est ce qui fait que vous le consommez que très rarement ?**

Bien, parce que c'est trop gros pour une personne seule. Par exemple, quand je vais avoir ma soeur et mon beau frère et qui vont rester quelques jours et que ma jeune sœur de… jeune enfin pour moi jeune *(rires)* qui habite Landéda, ma belle soeur, des fous, on se rassemble, on est juste 6. Et bien, je fais un apéritif. Mon apéritif, c'est que des fruits et des graines. Pas de petits gâteaux, de charcuterie pas de machin… Et donc, là, je vais mettre de la mangue et la mangue, j'adore. Ça, ça m'arrive d'en prendre pour moi toute seule. De temps en temps, mais en général, c'est quand j'ai du monde et je prépare… l'apéritif avec de la mangue.

Ok, est-ce que vous en voyez d’autres, des fruits que vous connaissez, mais que vous ne consommez pas ?

Des fruits que je connais que je consomme pas. La grenade, par exemple. Mais c'est tout, c'est pareil. C'est des fruits plutôt exotiques.

**Qu'est ce qui fait que vous en mangez pas du coup, la Grenade ?**

Parce que j'en mange quand ça se trouve chez les autres, quand je suis invitée, mais spontanément, j'ai pas, j'ai pas envie d'en acheter. Ce que je consomme sous forme de gelée, c'est de la mure parce que je fais les gelées moi-même, *(rires)* Je vais aller chercher mes mures !

**Oui c’est encore meilleur ! Et les murs et les framboises, tout ça, ça vous arrive ou**

Bah, les framboises ? C'est assez rare, elles ne veulent pas pousser chez moi et quand elles poussent dans des coins où je les ai pas mises *(rires)* mais c'est bien. Alors que les fraises, parfois, j'en ai chez moi, je les cultive quoi, j'en ai quelques unes. Mais bon, en dehors de ça, j'ai commencé la saison, la saison depuis un moment.

**Est ce qu'il y a d'autres choses que vous cultivez autre que les fraises ou une tentative de framboises ?**

Comme fruits ?

**Et légumes**

Alors les légumes. Oui, de toute façon, les légumes, j'ai une serre et je cultive les tomates. Et j’y mets du basilic aussi dans ma serre

**D’accord..**

Et dans mon potager, j'ai mis les petits pois. Ils ont plus ou moins raté cette année. Je mets des haricots verts. Parfois, j'en ai bien, d'autres fois, non. L'année dernière, j'ai eu plein de petits pois. J'en avais même congelés. Et puis, j'ai mis justement des potimarrons, des butternuts et des courgettes. Ça dans mon potager, et j'ai aidé des herbes, par ailleurs de la ciboulette, du persil, j'en ai mis une année dans mon potager et maintenant j'en ai partout dans le jardin, y compris tout l'hiver. Mon persil, je fais le tour du jardin quand j’ai besoin.

**Pratique !**

La ciboulette, du persil, j'ai… et j'ai planté un pied d'estragon mais l'estragon on a du mal à l'avoir. C'est pas facile à…. L’estragon… Qu'est ce que j'ai aussi alors qui est qui, qui pousse comme… J'en ai mis un pied depuis, c'est une vraie engeance, c’est le fenouil, le fenouil branche. Alors le fenouil bulbe j'ai jamais essayé, j'en consomme beaucoup mais j'ai jamais essayé.

**A ce propos, les oignons, l'ail, est-ce que vous en consommez ou…**

Alors moi, de l'ail, un peu, oui, j'en mets dans le potage, systématiquement, puis j'en mets autrement quand je fais un plat en sauce, de l’osso buco. Quand je fais bon… Qu’est-ce que je pourrais vous dire encore. Oui j’en mets pas mal jamais, de temps en temps cru, dans une salade oui ça m’arrive.

**D’accord**

L'oignon, pas trop trop. Parce que j'aurais tendance à avoir aussi des flatulences avec les oignons, mais j'en mets quand même de temps en temps, mais je ne mange pas souvent cru et autrement cuit. Oui. Quand je fais, quand je fais une ratatouille, quand je fais…. un plat, un plat en sauce.

**Ouais, et l'échalote, de la même façon ?**

Pareil, mais j'ai plus tendance à prendre de l'échalote que de l'oignon.

**D'accord… OK, alors si on s'intéresse maintenant à vos lieux de stockage, c'est à dire quand vous, quand vous revenez de vos courses avec tous ces fruits et légumes. Qu'est ce que vous en faites ?**

Alors, les fruits sont dans mon arrière cuisine, dans des coupes. Les légumes vont au garage qui est plus frais et très peu… La salade ne va au frigo que quand elle a été préparée, nettoyée, machin. Les pommes de terre, elles, elles sont dans un placard, dans l'arrière cuisine. Tout à fait au noir, quoi parce que si je les mets dans le garage, elles ont de la lumière tandis que là, elles n'en n’ont pas.

Bon, bah je vois pas autrement

**D’accord et est ce que ça, vous convient ? Est ce que ça vous convient comme, comme lieu de stockage ? Est ce que vous trouvez que vos fruits et légumes se conservent plutôt bien avec cette façon là de les ranger ?**

Je n'ai que des problèmes avec les légumes et les fruits bio quoi.

**Et pourquoi vous choisissez de mettre les fruits dans l’arrière cuisine et non pas au garage ou au frigo ? Qu'est ce qui fait que vous choisissez ? Ou alors il n’y a pas vraiment de choix, c'est comme ça**

Parce que je pense qu’au frigo, ils perdent leur… de leur valeur nutritive, ce qui est vrai pour les tomates, il faut absolument pas mettre les tomates au frigo.

**D'accord…**

Et puis. Je ne sais pas. Peut être pour les avoir plus sous la main ? J’en sais rien *(rires)* Oui, parce que très régulièrement. Avant le repas de midi oui ça je peux vous dire, je mange un fruit, mais un quart d'heure, 20 minutes avant, avant le repas.

**Pourquoi vous avez cette petite habitude ?**

Parce que… les fruits mangés en fin de repas vont fermenter facilement, parce qu'ils se digèrent plus vite et donc ils vont stagner. Et donc, il vaut mieux les manger avant le repas, un quart d'heure. Ils sont déjà en quelque sorte près de la sortie, si j’ose dire, de l’estomac.

**D’accord… En terme de digestion, c'est mieux de**

De les consommer avant qu'après.

**D’accord. Ça marche.**

C'est comme le thé, vous le buvez 20 minutes avant votre repas ou 20 minutes après la fin du repas. Parce que le thé vous mobilise le fer et empêche donc votre fer d'arriver jusqu'au sang si on le prend trop tôt après le repas.

**Et trop tard après, c'est pareil ? Parce que moi, si je veux… juste après, ce n'est pas bon non plus ?**

Ben non, c'est 20 minutes avant ou 20 minutes après, mais à la fin du repas, finalement, on peut traîner un petit peu avant prendre son thé.

**Il faudrait d'abord tout ranger avant de prendre le thé *(rires)***

Oui c’est ça *(rires)*

**Est-ce que vous avez… Vous m'avez parlé du garage qui est plus frais, les pommes de terre dans le noir, les tomates pas au réfrigérateur ? Est ce que vous avez comme ça d'autres stratégies de stockage et de conservation de fruits et légumes ? Ou essentiellement, ces trois là sur la température ?**

Oui, les oignons et l'ail sont aussi au noir dans un placard. Je ne sais pas si c'est valable, mais bon, j'ai l'habitude et puis il faut dire aussi que j'essaye aussi d'avoir les choses à portée de main, quand même quoi parce que… L'ail, l'oignon, bon bah, c'est près, c'est dans la cuisine et au noir. Autrement bah... C'est aussi que là, ils sont plus ou moins au frais quand même. Et puis, disons, c'est une question de place aussi. Les, quand même, les carottes, ça, ça n'a pas besoin d'être, d'être au frigo…

**Ouais… Ok. En termes de nettoyage de ces différents lieux, est ce que vous avez une organisation particulière ou non c'est, comme bon vous semble. Sur les contenants, que ce soit là où vous entreposez dans le garage, ou peut être les petits endroits dans le frigo où est la salade..**

Là où j’entrepose dans le garage, en général, je les laisse plus ou moins dans le contenant papier. Et j'ai une grande coupe en liège sur lesquelles je pose mes paquets.

**Et les coupes de fruits ?**

Quand je… rachète mes fruits, je nettoie la coupe pour remettre les suivants.

**OK... Alors maintenant, je voudrais qu'on discute de la façon que vous avez de consommer les fruits et légumes. Vous avez déjà cité déjà plusieurs préparations. Si on prend les fruits donc par exemple la fraise, vous m'avait dit que vous la consommez… vous faites aussi de la confiture.**

Oui, oui, mais je consomme cru aussi enfin comme ça.

**Oui, donc cru, confiture. Est ce que vous faites autre chose ou pas avec la fraise ?**

Non, non, je ne suis pas gâteau de toute façon. J’aime bien faire la cuisine, mais pas la pâtisserie. Ca, ma fille a appris à faire de la pâtisserie avec une de ses tantes *(rires)*

**Mais pas avec vous !**

Mais pas avec moi. A part les Clafoutis parce que quand j'étais à Vitry, on avait… des cerisiers Montmorency dans le jardin et je faisais des… je faisais des, pendant très longtemps, des cerises à l'alcool. Puis j'ai arrêté parce qu'il n'était pas question de… d'offrir des cerises à l'alcool après le repas, je faisais des clafoutis et je faisais des confitures avec mes Montmorency, mais en dehors…

**De cela ?**

En dehors de ça, donc la fraise, je mange cru quoi ou je fais des confitures.

**Oui, vous pensez pas un autre fruit que vous pouvez cuisiner ? Par exemple, la pomme ?**

Si la pomme, ça m'arrive de faire des compotes. De temps en temps me faire une compote de temps en temps, mais en général, j'aime beaucoup plutôt les croquer avec la peau si possible. C'est pour ça que je les prends bio. Et donc. Quand d'hiver, j'ai des invités, je fais comment ça s'appelle, les crumbles, crumbles pommes, poires.

**D’accord. Les bananes, quand vous en avez, ça vous arrive de les cuisiner ou pas du tout ?**

Non, non, non.

**Et les fruits d'été, comme les pêches, les abricots, ce sera plutôt cru ou…**

Pratiquement toujours cru. A la limite, j'ai une recette d'un gâteau, d'un gâteau aux abricots que j'ai dû faire une ou deux fois, mais bon, je vous dis c'est pas…

**Votre tasse de thé ? *(rires)***

Mmh !

**Ok ! Et donc la pomme vous m’avez dit que vous préférez croquer la peau que l'enlever ? Pourquoi vous préférez croquer avec la peau ?**

Croquer avec la peau parce que c'est dans la peau qu'il y a plein de vitamines, qu’il y a plein de bonnes choses. Et puis, c'est un réflexe aussi. Quand j'étais gamine, on avait des pommiers dans le jardin et la pomme….

**On croquait dedans ?**

Oui on croquait directement de l’arbre.

**OK, est-ce qu’il y a des fruits que vous épluchez ? Par exemple les pêches, etc. Vous les épluchezou vous consommez la peau ?**

Les nectarines, je consomme la peau, la pêche, ça dépend généralement non pour la pêche, j'aime pas trop le contact de la peau de pêche. J'avoue que ça, ça descend moins bien.

**Donc, c'est le contact, le contact par goût du coup ? La texture qui vous…**

Oui la peau est un peu plucheuse... c’est pas, c’est la consistance que je j'aime pas trop.

**Je vois. Ok… Si on prend un légume maintenant que vous avez dit consommez régulièrement, on en avait pas mal. Prenons la courgette. Sous quelle forme vous consommez la courgette ?**

Alors la courgette, ou je la fais en ratatouille… Ou bien je la fais simplement… Disons je la coupe en tronçons… pas trop, pas trop épais. C'est pas en rondelles ni en carrés, c'est en petits bâtonnets et je fais la même chose avec des poivrons très fins et un peu un peu… Un peu d'ail, et donc je fais revenir tout ça. Je mouille un petit peu et beaucoup d'herbes et de condiments, et en particulier du curcuma. Je fais ça.

**C'est comme une poêlé, quoi ?**

Oui une poêlé oui c'est ça

**Est ce que ça vous arrive de mettre de la courgette dans le potage ?**

Euh non… je n'ai jamais fait... Non, je ne l'ai jamais fait.

**Ok… Est ce que vous épluchez la courgette ou pas ?**

Moitié moitié. J’épluche et je laisse… surtout quand elles, quand elles sont, quand elles sont jeunes, quand elles sont petites, j'aurais tendance à moins les éplucher

**D’accord… qu'est ce qui fait que vous épluchiez à moitié celles qui sont un peu plus grosses ?**

Mmh.. Je ne sais pas ! *(rire)s* J’ai pris un peu cette habitude. Je veux garder un peu de peau parce que je sais que dans les peaux souvent, il y a plein de bonnes choses, mais en même temps, toute la peau, c'est un peu.

**C'est un peu trop…**

Un peu trop dur.

**Ok. Alors le poireau vous m’avez dit exclusivement en potage. Si on prend la tomate. Comment vous avez l'habitude de consommer la tomate ?**

La tomate, je la consomme crue, bon et souvent, quand je mets de la tomate dans les plats, c'est de la tomate pelée en en.... boîte.

**D'accord, sinon c’est plutôt crue ?**

Pas forcément quand je fais un tian, par exemple là elle est cuite. Et ça m'arrive quand j'en ai pas mal de tomates dans ma serre, justement, de faire de la soupe à la tomate aussi. Et je me prépare même des sauces que je, que je congèle et que j'ai pour plus tard pour maintenant dans des plats.

**D'accord et sur les tomtes, vous êtes autonome avec votre serre ou des fois, ça vous arrive d'en acheter ?**

Ben là, je vais. J'ai commencé à en acheter maintenant et après, je serai autonome jusqu'en septembre et même en courant septembre.

**OK. Vous là elles ne sont pas encore arrivées**

Non non… Elles ne sont pas encore arrivées, je commence seulement à voir des quelques unes qui sont comme ça et qui sont vertes encore.

**D’accord. La tomate qu'on vous la préparez, comment est ce que vous.. Vous la pelez ou non ?**

Non, je ne pèle pas. Je sais qu'on ne digère absolument pas les peaux de tomates.

**Mais si ça passe chez vous**

Ah bah ça va passer sous forme de fibres insolubles, mais…

**OK. Et vous les creusez ? Vous enlevez les pépins, l'eau ou non, vous mettez tout ?**

Moi, je les mange comme ça, cru je mets tout, tout y passe. Ça m'arrive de faire des tomates farcies.

**D’accord.**

Et à ce moment là, donc, j'enlève les, j'enlève les pépins. Effectivement, le centre, mais dans le centre, j'enlève le plus de pépins, mais je reprépare… toute la chair qui était au centre, je la remets avec la farce quoi ! J'en garde le plus possible de chair de tomates *(rires)*

**OK, alors là je voudrait qu'on échange sur le moment où vous jetez un fruit ou un légume, ou bien une partie seulement. Quand ça arrive, pourquoi décidez vous de le jeter plutôt que de conserver ?**

En général, quand je peux, j'enlève juste la partie abîmée. Mais mes deux citrons l'autre jour, c'était pas possible. Donc j’ai tout jeté.

**Ouais… Et comment vous déterminez que cette partie là, vous ne souhaitez pas la consommer ou que le fruit entier n'est plus..**

Parce que… la partie que je ne consomme pas, c'est qu'elle est vraiment abîmée, gâtée, pourrie quoi. Et quand je jette le fruit entier, c'est qu'il n'y a plus rien à sauver quoi.

**Et selon vous, pourquoi on ne mange pas du fruit ou un légume qui est abîmé, gâté, pourri ? Qu'est ce qui fait que ce n'est pas consommable pour vous ?**

Parce que je pense que le… L'organisme disons qui a fait pourrir le fruit, peut être aussi dangereux pour nous. Sans compter que l'aspect n'est pas engageant.

**Quand vous dites pas engageant, qu’est-ce que vous voulez dire ?**

En général, c'est mou… ça coule… c’est… Comment je dirais encore… et parfois, on voit qu'il y a… On voit déjà qu'il y a des… Quand c'est un champignon on voit qu’il y a des spores. Mais de toute façon… Mes deux citrons, ils étaient, ils étaient gris vert. Il y avait la couche de… déjà des spores, de champignons dessus.

**Ça, ça vous engage pas à…**

A essayer de l'ouvrir ! Même pas *(rires)* Ce n'était pas possible.

**Et justement, quand vous êtes face à du… quand vous avez dans vos mains un fruit ou un légume qui est pourri, moisi ou carrément on voit une couche de champignons, qu'est ce que vous ressentez à ce moment là, face à ce pourri ?**

Pff… Je me sens un peu râleuse parce que je me dis… Je me dis flute, ça, ça s'est gâté vite, ça s'est abîmé vite et c'est… Même sans être radin, c'est râlant de perdre…. D'autant plus qu'en général, les fruits et légumes bio sont plus chers en plus.

**Ouais… Oui, quand vous perdez un fruit et légume, vous perdez de l'argent finalement ?**

Ben oui, oui.

**OK, alors si justement on intéresse au gaspillage alimentaire de façon assez générale, quand je vous dis gaspillage alimentaire, quels sont les mots qui vous viennent spontanément à l'esprit ?**

Gaspillage alimentaire ? De quelle façon ? Vis à vis de moi ou

**Comme ça, quand on vous dit ça, quelles sont les images que vous avez en tête ? Quels mots vous viennent ?**

J'avoue pas grand-chose hein… Parce que j'ai pas l'impression que ça me concerne trop, en tout cas. Ce… Ce que…. Du point de vue de l'alimentation, ce que je trouve de plus. Plus. Plus grave, ce sont le système des plats préparés, ça j'avoue que c'est vraiment… parce que pratiquement, ils sont tous nocifs. Et même les bio. Même préparé en bio, il y a des tas de choses dedans qui sont pas bio et qui en bio comme dans les autres, on rajoute du sucre, on rajoute du sel et des trucs comme ça... Et le gaspillage alimentaire ? Je pense surtout, ça se fait de moins en moins et on récupère de plus en plus, le gaspillage au niveau des grandes surfaces quoi, quand ils jettent alors que c'est pas encore… abimé ou fané.

**Ouais…**

Il y a quelque chose que je vous ai pas dit aussi, c'est que je ne, je ne peux plus consommer de concombre.

**Ah oui, c'est vrai le concombre.**

C'est comme, c’est comme les radis,

**c'est sur la digestion ?**

La seule façon de le consommer que j’ai et que ça passe encore. C'est quand je le fais en tagliatelles. En long machin très fine, ça passe, mais en rondelles ordinaires. Là, ça ne va plus du tout. C'est curieux, mais en vieillissant comme ça, on a des trucs. ..

**Le corps change, on devient plus sélectif *(rires)***

**Si on essaie de faire une définition du gaspillage alimentaire. Si vous deviez écrire une définition dans le dictionnaire, comment vous définiriez le gaspillage alimentaire ? Selon vous, qu'est ce que c'est finalement, ça ?**

J'aurais du mal hein…

**Vous m’avez parlé des grandes surfaces, par exemple…**

Mais pas. Mais je vois pas autrement. Tellement de façons de gaspiller… Je sais pas, c’est peut être parce que… Quand j'étais jeune on ne gaspillait rien ! *(rires)* et pour les légumes, on vivait pratiquement avec le potager.

**Ouais…**

On achetait pas… En dehors du potager, on achetait rien du tout de légume. L'année dernière, j’ai planté quelques salades parce que on m'avait… quand j'ai acheté mes plants de tomates on m'avait offert plein de salades. Et puis ma petite voisine, qui habite un peu plus loin, elle était venue et m’en avait redonné aussi de la salade et malheureusement, disons que il faudrait quand je mets de la salade, je mette du mesclun simplement parce qu'on coupe ce dont on a besoin. Et puis parce que mes salades, ça m'est arrivé d’en apporter chez [Prénom de sa fille]. Parce que… elles poussaient tout en même temps et c’est trop quoi. Donc c'est pour ça que cette année, je n'ai pas pensé à en remettre. Mais quand vous, quand vous en achetez en… c'est pareil quand vous en achetez... Des petits plans en jardineries c’est par 6 ou par 10. C’est jamais… vous pouvez jamais piqué 2 ou 3 *(rires).*

**Ouais…. OK, alors pour terminer juste sur la poubelle connectée que vous avez depuis un petit moment maintenant. Comment ça se passe avec cette poubelle ?**

Elle a un petit problème, c'est que là, un mauvais contact avec le voyant de la pesée. Et souvent que mais [Prénom de sa fille] a vérifié, ça pèse bien mais moi, je vois pas le voyant… le voyant ne s'allume pas la plupart du temps. Des fois, oui des fois, non.

**D'accord il faudra qu’on change**

Autrement. Ça se passe euh…

**Comme une poubelle ?**

Une poubelle oui.

**Vous avez le sentiment, de l’utiliser beaucoup ou au contraire, vraiment pas beaucoup ?**

Comment je vous ai dit quand j'ai un petit machin, mais je trouve que je l’emploie trop !

**D’accord, selon vous c'est trop.**

Bien que je sache qu'après, ça va quand même au compost, mais bon…

**Vous compostez, oui ?**

Oui, je compost

**Alors, vous m’avez parlé des deux citrons. Est ce que vous avez d'autres exemples de ce que vous avez pu y jeter ?**

Deux fois des morceaux de pêche plate, pareil, bio, bon, etc. Euh… Un ou deux morceaux de brocolis. Ça doit être, ça doit être tout. Ah si, quelques quelques feuilles de salade qui avaient été triées, qui étaient au bas du frigo, mais qui se sont abîmées quand même, ça aussi je vous l’ai mis *(rires)*

**Merci ! *(rires)***

Dans cette poubelle là, parce que je les ai laissé s’abimer sans les consommer dans…

**D'accord. Est ce qu'à un moment donné, vous vous êtes posée la question du style est ce que ça, ça doit aller dedans ou pas ? Ou ça va c'était assez clair sur ce qui devait y être.**

Pour moi, c'est assez clair quoi

**Ouais ? ok… Est-ce que ça a pu vous arriver de l'oublier la poubelle ? En préparant quelque chose d’enlever des petits bouts, de se dire mince, ça aurait pu aller dedans ? Et puis, au final, tout est parti un peu dans le compost, ce qui peut arriver ?**

Je crois pas, non…

**Ouais ?**

Non je crois pas

**Vous n’avez pas le souvenir d’avoir oublié.**

Parce que dans le compost, c'est vraiment les épluchures, les…

**Ouais tout à fait. D'accord…, est ce que vous avez changé de sac poubelle ?**

Oui.

**Ouais ?**

D'ailleurs, c'était une cata. Il y avait quelque chose qui avait pourri. C'est [Prénom de sa fille] qui me l'a changée.

**Elle m'a raconté !**

Elle me la nettoyé et là, depuis, j'ai changé une fois *(rires)*

**Je pense que finalement**

Là ça va être changé samedi. *(discussion sur sac poubelle)*

**Bon bah écoutez, super, j'ai posé toutes mes questions. Merci beaucoup d'y avoir répondu. Est-ce qu’il y a des choses sur lesquelles vous souhaitez revenir. Si vous pensez que j'ai pu oublier des petites choses sur votre consommation de fruits et légumes ?**

Je crois pas…

**Ouais ? On a fait un bon petit tour ? Bon chouette, bon écoutez ça marche !**

**Household F30, Interview 2**

*Mme 1:* Alors ! Donc, des petites photos. Pour commencer, je vais vous demander de choisir une de ces images. Hop ! N'importe-- n'importe laquelle, de la retourner et de me dire spontanément [euh] ce qui vous vient à l'esprit.

*Mme 2:* N'importe laquelle ou lesquelles ?

*Mme 1:* Ouais, on va toutes les faire. [rire]

*Mme 2:* [Bon] [ben] ça, c'est un-- un petit potager en carré. Je sais pas comment on les appelle. C'est pas les-- C'est pas les potagers [euh] surélevés, c'est ça ?

*Mme 1:* Ouais, c'est aussi--

*Mme 2:* C'est des potagers en carré ?

*Mme 1:* Ouais, c'est ça. Mm-hmm! Qu'est-ce que ça vous évoque [euh] le potager, alors celui-là ou-- ou des potagers de manière plus générale ?

*Mme 2:* [Ben] J'aime bien faire du potager.

*Mme 1:* Ouais !

*Mme 2:* Mais [euh] [ben] cette année, particulièrement, ça n'a pas marché.

*Mme 1:* [Ah] ?

*Mme 2:* Non, la saison a été nulle parce qu'il a fait sec en avril et très froid en-- en mai.

*Mme 1:* Ouais !

*Mme 2:* Donc [euh] pas de petits pois, pas de haricots verts et mes courgettes, ça n'a pas marché non plus.

*Mme 1:* [ah] [rire]

*Mme 2:* Donc ça, c'est la photographie d'un-- d'un joli [euh] carré bien entretenu.

*Mme 1:* Ouais !

*Mme 2:* Avec une vigne, au fond, je crois ?

*Mme 1:* Ouais, apparemment une vigne. [silence 00:01:57-00:02:01] Et qu'est-ce que vous aimez [euh] dans le potager ? Qu'est-ce que vous aimez ? Pourquoi vous aimez ça, faire votre potager ?

*Mme 2:* [Ben] ce que j'aime, c'est de pouvoir avoir des-- des fruits [euh] des légumes [euh] forcément bio.

*Mme 1:* Ouais !

*Mme 2:* Parce que je vais-- et [euh] et frais et [euh] ce que je-- j'aime bien quoi.

*Mme 1:* Ouais !

*Mme 2:* Les petits pois, les haricots verts, les courgettes [euh], les potirons.

*Mme 1:* Ouais !

*Mme 2:* Et puis [euh], ce qui est bien aussi dans le potager, c'est qu'on peut toujours avoir ces [euh] ces herbes fraîches quoi : son pied de thym,

*Mme 1:* Ouais !

*Mme 2:* Sa ciboulette [euh] et ce genre de choses.

*Mme 1:* Et ce que vous aimez, c'est le fait de-- d'avoir les produits que-- que vous souhaitez et dont vous connaissez [euh]

*Mme 2:* Oui ! Voilà !

*Mme 1:* Caractère bio quoi ? Est-ce qu'il y a autre chose qui fait que vous aimez le potager ou c'est essentiellement ça, le fait de produire vos légumes ?

*Mme 2:* [ben] c'est- c'est surtout ça. Oui, d'avoir-- d'avoir ses ses propres légumes l'été, etcétéra. Parce que mes tomates, je les cultive sous serre. Parce qu'ici, les tomates d'un potager, c'est pratiquement pas possible.

*Mme 1:* Ouais, c'est [silence 00:03:27-00:03:30] ouais, vous arrivez à vous subvenir en tomates ? Ouais ?

*Mme 2:* Il y a des années, cette année, non, parce que j'étais absente à un moment où ils auraient dû être très à dos, très [euh] arrosé, [euh] mais [euh] il y a des années où j'arrive à faire [euh] quelques beaucoup de conserves quoi.

*Mme 1:* D'accord !

*Mme 2:* Il y a des sauces.

*Mme 1:* OK ! Globalement, est-ce que le potager, c'est un lieu dans lequel vous vous sentez bien ou non, vous le faites par nécessité [euh] ? Voilà, c'est--

*Mme 2:* Non, non, c'est-- pour moi de toute façon, tout le jardinage, c'est-- c'est un peu du [silence 00:04:15-00:04:18] du du passe-temps agréable quoi ?

*Mme 1:* Ouais !

*Mme 2:* Évidemment, j'suis un peu déçue quand ça marche pas, mais [euh] je- j'aime toujours. J'aime bien quoi.

*Mme 1:* Ouais ! [silence 00:04:34:00:04:37] Est-ce que vous sauriez dire pourquoi c'est un passe-temps agréable ? Si vous arriveriez à développer ?

*Mme 2:* Non. Disons que disons que j'ai j'ai toujours connu un potager, oui, quand j'étais gamine aussi et que [euh] de toute façon [euh] j'aime-- j'aime bien voir pousser et puis [euh] [euh] c'est pas seulement pour récolter, c'est le plaisir de de de de voir que ça que ça marche. [euh] ça, c'est comme comme pour le jardin d'agrément, [bon] [ben] on est on est content quand les fleurs poussent comme ça. Quand [euh] quand on a un bel arbuste [euh], etcétéra.

*Mme 1:* Ouais ! Quand ça marche pas, on est déçu, mais par contre quand ça marche [euh]

*Mme 2:* [ben] oui. [rire] On se sent un peu récompenser si [diaphonie 00:05:42-00:05:43] [rire]. Mais c'est jamais une corvée en tout cas.

*Mme 1:* Ouais. [silence 00:05:47-00:05:50] D'accord ! Et donc [euh] qu'est-ce que vous pensez des des produits que-- des fruits et légumes [euh], des potagers ? Qu'est-ce que vous vous [diaphonie 00:06:02-00:06:03]

*Mme 2:* En ce qui concerne en tout cas les tomates, c'est sûr que ces-- les tomates-là ont un goût et alors que-- de toute façon, je n'achète plus du tout de tomates, [euh] sauf vraiment, au tout début de la de la saison ou j'en ai pas encore ou vraiment il y en a sur le marché qui sont-- qui sont [euh] bio et belles et-- mais en général, c'est vrai que celles qu'on cultive soi-même, elles ont du goût. Voilà ! Et et suivant les les races, elles n'ont pas le même goût. Elles sont vraiment différentes et vraiment bonnes.

*Mme 1:* Et donc ça, ça marche, notamment pour la-- ?

*Mme 2:* Notamment pour la tomate, oui. Pour les autres [euh], pour les courgettes par exemple, c'est moins évident, mais bon [euh] quand même--

*Mme 1:* Ouais ! [silence 00:07:02-00:07:05] Elles sont bonnes. Après, est-ce qu'elles sont énormément [euh] enfin, est-ce que c'est [euh] la différence de goût est [euh] est peut-être moins-- moins significative que--

*Mme 2:* Oui, c'est [inaudible 00:07:14-00:07:15]

*Mme 1:* Avec la tomate. Et est-ce que ça vous arrive de-- de perdre des produits qui sont issus de votre potager ou pas ?

*Mme 2:* Non ! En général, non. [silence 00:07:27-00:07:34]

*Mme 1:* Est-ce que vous vous-- vous savez pourquoi vous arrivez à pas perdre vos fruits et légumes ou du potager ?

*Mme 2:* Parce que j'ai j'ai pas une [euh] une récolte [euh]-- je je mets un nombre de plants qui qui marche avec ma ma consommation en quelque sorte quoi. Et [euh] par exemple, pour les petits pois, il y a eu des années ou j'ai pu j'ai pu en congeler et donc, je ne je ne les perds pas.

*Mme 1:* Oui, quand vous avez une trop grosse-- quand vous voyez que c'est une trop grosse récolte [euh], vous-- vous congelez [euh]. Ça ça peut vous arriver de congeler et de--

*Mme 2:* Ça peut m'arriver de congeler en fait. Certains des petits pois, ça se congèle bien. Les haricots verts, ça ne se congèle pas.

*Mme 1:* D'accord !

*Mme 2:* Mais, [euh] [bon] [ben] ça m'est arrivé peut-être depuis quelques années, une fois ou deux, d'en donner un-- une poignée à [prénom] mais c'est tout quoi.

*Mme 1:* Oui !

*Mme 2:* Mais je perds jamais de-- je perds jamais de légumes, non.

*Mme 1:* [silence 00:08:31-00:08:38] D'accord ! [silence 00:08:40-00:08:43] Pourquoi ? Est-ce notamment dû au--

*Mme 2:* Si, il y a un seul fruit que que j'ai que j'ai planté [euh] parce qu'il s'est trouvé qu'il y a des plants qui ont poussé sur mon sur mon compost et les les coquerets du Pérou. Et les coquerets du Pérou, je les ai donc replantés dans mon [euh] jardin potager, sur le bord du potager, et pas cette année. Mais l'année dernière, j'en ai eu assez pour faire des confitures.

*Mme 2:* D'accord !

*Mme 2:* J'en ai fait des quelques pots de confiture. Donc du coup, maintenant [ben] je je laisse cette rangée dans le potager [rire], mais j'en ai profité à manger comme ça, quoi.

*Mme 1:* Ouais ! Et vous avez d'autres fruits ou pas ?

*Mme 2:* [euh] D'autres fruits dans le jardin ici, non.

*Mme 1:* Non ?

*Mme 2:* Non, non ! Je peux pas avec le vent. [diaphonie 00:09:43-00:09:45] Je peux pas avoir un seul [euh] un seul [euh], comment dire [euh] ? Arbre fruitier, quoi.

*Mme 1:* Ouais. [rire]

*Mme 2:* Je réussis encore à avoir quelques arbres [euh] décoratifs, mais par exemple, mon mimosa qui est là, et [ben] depuis, il est-- j'ai pas eu de fleurs depuis 2016.

*Mme 1:* Ouais, ça commence à faire.

*Mme 2:* Il est déplumé par les tempêtes tous les ans avant de fleurir. Et là, [euh] les deux derniers coups de vent qu'il y a eu là [euh)

*Mme 1:* Ouais. Oui, et puis là, vous êtes [euh] ouais.

*Mme 2:* Oui, oui, donc. [rire] Mais c'est c'est très net depuis [euh] disons, ça commence à faire [euh] [silence 00:10:32-00:10:35] la première grande tempête, on a eu en 2008. Après, on a eu plusieurs années tranquilles et depuis 2014, finalement, c'est rare qu'on ait un hiver sans sans coup de vent. Et depuis depuis quatre ans, c'est tous les ans, des coups de vent sans arrêt.

*Mme 1:* Ouais ! Oui, oui, plusieurs, il y en a des fois, deux ou trois dans l'hiver qui--

*Mme 2:* [ah] oui, oui, oui ! [ben] là déjà, on a eu [euh] on a eu de des journées [euh] dimanche, il y a des-- il y a dix jours, ça a été affreux et depuis, on a eu deux autres journées [diaphonie 00:11:11-00:11:12] et du c'est-- ça n'arrête pas.

*Mme 1:* [ah] oui ! Donc du coup, pas de fruit ? Pas de fruit ? [rire]

*Mme 2:* [ben] c'est même pas la peine d'essayer quoi.

*Mme 1:* OK ! Alors, si on prend une autre photographie. Est-ce que celle-là [diaphonie 00:11:38-00:11:39] Ouais, celle que vous voulez.

*Mme 2:* [Ah] [bon] [ben] c'est un bel [euh] [silence 00:11:47-00:11:52] comment on dit, un bel-- on dit pas un étalage, on dit un-- [rire] de de fruits et je suppose qu'ils sont bio ?

*Mme 1:* Ouais !

*Mme 2:* [silence 00:12:04-00:12:13] Et qu'on est forcément l'été. Poivrons, courgettes, aubergines, tomates. Mais il y a quand même des-- encore des légumes d'hiver parce qu'il y a des carottes et des céleris.

*Mme 1:* Ouais !

*Mme 2:* Des céleris boules. Parce que le céleri branche d'ailleurs, je sais pas si le céleri branche, c'est plutôt [euh] printemps été que que l'automne. Je sais pas. Mais disons que ce sont quand même [ben] les légumes classiques que l'on trouve dans dans la région, quoi.

*Mme 1:* Ouais ! Pourquoi vous trouvez ça beau ? Ouais, c'est un bel étalage de fruits et légumes.

*Mme 2:* Oui, c'est-- [bon] [ben] [ben] disons, assez classique quoi. [rire]

*Mme 1:* Ouais !

*Mme 2:* Assez classique.

*Mme 1:* Et qu'est-ce qui vous amène à supposer que c'est bio ? Qu'est-ce qui vous fait un peu penser à ça ?

*Mme 2:* [Ben] Ça me fait penser à ça parce ce que [euh] parce qu'il y en a, un peu de tout ce qu'on peut cultiver dans le coin, déjà.

*Mme 1:* Ouais !

*Mme 2:* Y en a quelques que je ne vois pas très bien là, mais c'est peut-être de la betterave. [euh] et [euh] disons qu'ils ont un air de fraîcheur qui fait penser qu'ils n'ont pas trop, dû trop trop voyager ou trop longtemps.

*Mme 1:* Ouais !

*Mme 2:* Enfin, peut-être que je me trompe [hein] même.

*Mme 1:* Ouais mais justement, c'est-- à quoi ça fait penser, ce n'est pas forcément certain, certain. Et [euh] est-ce que c'est un lieu dans lequel vous vous sentiriez bien ou pas vraiment ?

*Mme 2:* C'est assez difficile à dire parce que [euh] disons que, comme je fais mes courses, ce n'est pas le genre de question que je me pose, quoi.

*Mme 1:* D'accord !

*Mme 2:* Mais je choisis, je choisis mes endroits pour faire des courses quand je sais que je vais trouver justement des produits, des produits bio et et, toutes ces choses, donc. Si, si on veut, quand je vais faire mes courses, je sais ce dont j'ai besoin, ce que je choisis et [euh] ce n'est jamais des lieux où j'ai envie de traîner ou de rester, quoi. C'est pas-- je traîne plus volontiers dans les magasins de de plantes.

*Mme 1:* D'accord ! [rire] Ouais !

*Mme 2:* Ou de fleurs. Et où il y a des graines, des choses comme ça. [rire]

*Mme 1:* Les pépinières ? D'accord ! Et donc quand vous allez acheter vos fruits et légumes, ce n'est pas par plaisir que vous allez vous promener dans le magasin, c'est pour aller chercher des produits, vous savez, que vous allez trouver--

*Mme 2:* Mais oui, voilà ! Parce que j'ai besoin de fruits, parce que j'ai besoin de légumes.

*Mme 1:* Ouais ! Et [euh] est-ce que vous avez déjà vécu une ou des expériences avec ce type de lieux ? Est-ce que ça ressemble ? Est-ce que ça peut ressembler globalement à un magasin dans lequel vous allez ou pas ?

*Mme 2:* [euh] Oui, on peut, on peut dire ça enfin--

*Mme 1:* Ça vous arrive d'aller chez ce, enfin-- Je ne me rappelle plus ce que vous avez dit la première fois qu'on s'était vu. Vos fruits et légumes. [euh] Alors c'est quand vous allez à la piscine, je crois. [rire] Vous faisiez--

*Mme 2:* Oui, voilà, c'est ça. C'est exactement ça. Quand je vais à la piscine, quand je sors tout de suite à côté, il y a ce qu'ils appellent le magasin, c'est le Grand potager, je crois, le Grand potager bio. Je, j'ai même en en achetant des trucs pas du tout, du genre légumes, mais parce qu'ils font des tas d'autres choses bio.

*Mme 2:* Ouais !

*Mme 2:* J'ai gagné un un sac. Qu'est-ce que-- où est-ce que j'ai posé ce sac pour [euh] [inaudible 00:17:42-00:17:43] Le grand panier bio.

*Mme 1:* Le grand panier.

*Mme 2:* Il est, il est beau [hein] !

*Mme 1:* Eh oui ! Ça ils vous l'ont offert ?

*Mme 2:* Voilà ! Là ils me l'ont offert parce que j'avais plus, plus de 80€ de d'achats.

*Mme 1:* D'accord !

*Mme 2:* Parce que j'ai acheté mon thé--

*Mme 1:* OK !

*Mme 2:* Mon thé bio, [euh] c'est, comment ça s'appelle ? J'ai vraiment des des pertes de mémoire immédiates. [rire] J'ai du mal à-- Le thé de la pagode. Ils font le thé, ils font le thé de la pagode. D'habitude, je les achetais par correspondance et eux ils les font, donc je les achète. Mais bon, c'est quand même pas assez gros comme boîte.

*Mme 1:* Ouais, ouais !

*Mme 2:* Et si on en prend plusieurs, on arrive vite à--

*Mme 1:* Ouais, ça monte.

*Mme 2:* On arrive vite à plus de 80€ [rire] parce qu'il y avait le reste des achats aussi. Donc [euh] voilà ! J'ai eu mon grand sac pour aller la prochaine fois.

*Mme 1:* [rire] Et dans ce magasin-là, du coup, on trouve de tout ? Il n'y a pas que des fruits et légumes ? Du coup, ils faisaient [euh]--

*Mme 2:* [ah] Non, non, non, il y a tout. Vous avez tout, tout, tout, produits [euh] Le produit pour la lessive--

*Mme 1:* Ouais, les produits ménagers, les--

*Mme 2:* Les produits ménagers, vous avez les fruits secs en--

*Mme 1:* Ouais, en vrac.

*Mme 2:* En vrac. Vous avez aussi des alcools, si vous en voulez.

*Mme 1:* Ouais !

*Mme 2:* Vous avez toutes les, toutes les les céréales. Enfin [bon] bref, les produits de beauté. Les compléments alimentaires.

*Mme 1:* [ah] C'est une épicerie générale mais tout est bio quoi ?

*Mme 2:* Voilà, c'est ça.

*Mme 1:* Et quand vous allez pas à la piscine, vous allez toujours à cet endroit là ou pas ?

*Mme 2:* [Euh] oui, moins.

*Mme 1:* Ouais !

*Mme 2:* Parce que je vais du coup moins à Lannilis et du coup [ben], je fais mes achats de légumes bio à la petite épicerie de Lilia ici.

*Mme 1:* D'accord, oui, c'est ça. Vous trouvez du bio aussi là-bas ou c'est pas bio ? À la petite épicerie de Lilia.

*Mme 2:* Il y a du bio et du pas bio. Il y a les deux.

*Mme 1:* D'accord !

*Mme 2:* Il y a les deux.

*Mme 1:* Et c'est pareil, en plus pour une épicerie, il n'y a pas forcément que des fruits et légumes, quoi.

*Mme 2:* Non ! Non, non.

*Mme 1:* Oui voilà !

*Mme 2:* Il n'y a pas, il y a par exemple de la viande congelée.

*Mme 1:* Ouais ! Un peu de tout ?

*Mme 2:* Il y a les produits laitiers, il y a les œufs, il y a les produits d'entretien et ils font des dépôts de pain aussi.

*Mme 1:* Donc finalement, vous, ça vous arrive quasiment jamais de vous rendre dans un magasin de primeurs que de fruits et légumes ? Vous, c'est des épiceries, des épiceries, quoi. Que ce soit le grand panier bio ou la petite épicerie de--

*Mme 2:* Non, où il y a peu des fruits et légumes. Non, parce que [euh] j'en connais pas-- Il y en a, il y en a, mais [euh] ou bien on va à la ferme.

*Mme 1:* Ouais !

*Mme 2:* Et ça m'est arrivé d'y aller assez longtemps. Mais depuis deux ans là, [bah] depuis le Covid finalement, je--

*Mme 1:* Ouais.

*Mme 2:* Je suis plus, j'y suis plus allée. Et il y avait-- Il y a une autre aussi qui ouvre-- C'était le vendredi après-midi. Là, c'est, une ferme.

*Mme 1:* Oui.

*Mme 2:* [euh] Ils font direct, directement la vente quoi. Et c'est bio. Et là c'est pareil. Depuis le, depuis le Covid, j'y suis pas allée. Et dans la mesure où je trouve mes légumes bio soit ici à la machin, soit au grand panier--

*Mme 1:* Ouais !

*Mme 2:* J'y vais plus quoi.

*Mme 1:* Oui.

*Mme 2:* Parce que là-bas c'est soit le samedi matin à l'une des fermes, soit, qui là n'est pas bio, c'est raisonné mais pas forcément bio. Et à la ferme bio, c'est seulement le vendredi après-midi.

*Mme 1:* Oui, d'accord, c'est que des petits créneaux, quoi ?

*Mme 2:* Oui, des créneaux, donc ça me fait aller plus loin et ça m'intéresse du coup.

*Mme 1:* D'accord ! Alors si on prenait une autre photographie ?

*Mme 2:* [bon] Allez hop ! [rire] Ça c'est au marché.

*Mme 1:* Ouais !

*Mme 2:* C'est au marché.

*Mme 1:* Qu'est-ce que ça vous évoque le marché ?

*Mme 2:* [Ben] Que les étals sont très remplis, quoi.

*Mme 1:* Oui.

*Mme 2:* C'est vrai que c'est assez, assez attirant, mais [bon] il y a quelques années, ça m'arrivait d'aller au marché le jeudi à Plouguerneau.

*Mme 1:* Ouais !

*Mme 2:* Mais [euh] ça fait très longtemps que j'y suis pas allée parce que ce n'est pas toujours que l'on trouvait justement-- que venait le marchand de légumes bio, des fois ils étaient là, des fois ils n'étaient pas là.

*Mme 1:* D'accord !

*Mme 2:* Et donc [bon] [ben] bref, j'ai laissé un petit peu tomber. D'autant plus que c'est très difficile d'arriver à se garer. [rire]

*Mme 1:* Ouais !

*Mme 2:* Donc [euh], j'ai arrêté. [Bon] c'est, c'est un marché, un marché classique.

*Mme 1:* Oui ! Et [euh] est-ce que c'est un lieu dans lequel vous vous sentez bien ? Le marché ou pas vraiment, enfin voilà, il n'y a pas trop de différence avec d'autres magasins.

*Mme 2:* Disons que ça m'attire moins depuis que j'ai pris des années.

*Mme 1:* D'accord ! [rire]

*Mme 2:* Je trouvais ça agréable.

*Mme 1:* Pourquoi vous trouviez ça agréable avant ?

*Mme 2:* [ben] Je-- Parce que [euh] on peut comparer, on peut déambuler entre les différent-- Il n'y a pas que les fruits et légumes, il y a-- Ça m'arrivait par exemple de me prendre un demi poulet rôti à la broche ou des choses comme ça. Ou bien, il y a un peu de jambon, du jambonneau rôti, quoi. Puis, il y avait, il y avait même parfois des marchands de plantes.

*Mme 1:* Oui !

*Mme 2:* Pas seulement des fleurs, mais les plantes et à l'occasion. Mais ça fait, disons que ça fait assez longtemps que je n'y suis plus allée.

*Mme 1:* Ouais !

*Mme 2:* Dans les dernières années, c'est vrai qu'il y avait de temps en temps, mais pas régulièrement, justement, les marchands de légumes bio.

*Mme 1:* Oui. C'est pour ça que vous y alliez du coup ?

*Mme 2:* [Ben] aussi, oui. Et puis [euh]--

*Mme 1:* Et [euh] qu'est-ce que vous pensiez des produits que vous achetiez en, au marché ?

*Mme 2:* En général, c'était de bonne qualité. C'était oui, oui-- D'ailleurs justement les gens qui ouvraient leur ferme le Samedi matin, étaient au marché de Plouguerneau, là.

*Mme 1:* D'accord ! Ils vont vendre leurs produits [euh]--

*Mme 2:* Oui, oui, bien sûr ! Et ils faisaient certainement d'autres marchés.

*Mme 1:* Oui, tout à fait. OK ! Alors si on prend une autre, la dernière photo. [rire]

*Mme 2:* Alors, ça c'est vu du haut.

*Mme 1:* [rire] Oui.

*Mme 2:* [Bon] [ben] disant que, vu d'en haut comme ça, ça [ah] c'est assez esthétique, disons les différents carrés, mais [bon] [ben] ça ne donne absolument pas une idée de-- Ni des différents [inaudible 00:28:02-00:28:03] ni de leur fraîcheur, ni de leur-- Pour moi, c'est vu de trop loin, quoi. [rire]

*Mme 1:* Ça vous fait penser à quel [euh] quel style de magasin ?

*Mme 2:* [euh] C'est certainement pas une grande surface. Parce que disons qu'il y a trop de distance entre les différents étals.

*Mme 1:* D'accord !

*Mme 2:* Et que ça paraît bien, bien agencé du point de vue des couleurs et des machins comme ça. Et j'ai pas l'impression que dans un grand magasin, on prenne autant de, de temps, disons, pour arranger aussi bien.

*Mme 1:* Ouais !

*Mme 2:* D'autant plus qu'à côté, ça fait un peu salon si vous voulez. [rire] Là, le petit carré là--

*Mme 1:* Oui !

*Mme 2:* Je sais pas du tout, c'est--

*Mme 1:* Moi non plus, à la base, je ne sais pas.

*Mme 2:* Ça vous donne l'impression d'être une grande surface ?

*Mme 1:* À la base ouais. Mais c'est vrai que pas forcément. C'est vrai que les couleurs sont bien agencées.

*Mme 2:* Je ne sais pas, parce que [diaphonie 00:29:34-00:29:35] un peu la distance qu'il y a entre les-- J'ai l'impression qu'on- que c'est bien plus resserré dans un [diaphonie 00:29:43-00:29:44]

*Mme 1:* Quand on voit la personne, c'est vrai qu'il n'y a pas non plus en fait, tant d'espace dans le-- Mais ouais, vous avez peut-être raison. La grande surface, c'est de plus en plus, plus serré. [rire] Et alors--

*Mme 2:* Je ne sais pas, ça me donne l'impression d'être vraiment-- Ou bien si c'est dans une grande, enfin, grande moyenne surface, c'est, c'est, disons, déjà un sacré standing quoi.

*Mme 1:* D'accord !

*Mme 2:* C'est pas-- ça me donne pas l'impression d'une grande surface people.

*Mme 1:* Oui. Pas celle qu'on a-- Et vous, les grandes surfaces, est-ce que ça vous arrive d'y acheter des fruits et légumes ou pas ?

*Mme 2:* Ça m'arrive, mais très rarement. Ça m'arrive encore, oui. Par exemple, quand j'ai pas trouvé, je n'ai pas trouvé de brocoli par exemple dans mes deux machins, j'achèterais une tête de brocoli à la grande surface, quoi.

*Mme 1:* Ouais !

*Mme 2:* Mais--

*Mme 1:* Donc c'est plus pour des éléments précis ?

*Mme 2:* C'est assez rare. Ça m'est arrivé aussi d'acheter au début de la saison des endives, d'en acheter à la grande surface parce qu'il y en avait pas du tout dans mes deux, deux autres magasins, quoi.

*Mme 1:* D'accord ! C'est quand vous trouvez pas ailleurs que vous vous rendez--

*Mme 2:* Oui, voilà ! Oui, c'est tout.

*Mme 1:* Et [euh] comment vous vous sentez quand vous êtes dans une grande surface ? C'est un moment plutôt agréable ou non, vous y allez vraiment par nécessité ?

*Mme 2:* Ouais ! C'est pas désagréable [hein] c'est pas désagréable mais [bon] [ben] j'y vais parce que vraiment j'ai besoin d'y aller. Ça veut, ça va pas le genre-- La seule chose que je trouve plus agréable dans ma grande surface, c'est que j'ai aussi accès à un rayon librairie.

*Mme 1:* D'accord !

*Mme 2:* Et que du coup [ben] ça m'arrive d'acheter un bouquin et [rire] ça m'arrive de-- Oui ! Et puis il y a des choses que j'achète là quand même c'est ma lessive, la machine à laver le linge, par exemple. Je ne vais pas forcément la prendre bio. Je prends une lessive bio du genre Le Grand Arbre Vert ou quelque chose, mais dans ma grande surface, quoi.

*Mme 1:* Les écolabels, ce qu'ils appellent les--

*Mme 2:* Voilà !

*Mme 1:* D'accord ! Et vous pensez quoi des produits, donc des fruits et légumes que vous trouvez en grande surface ? De manière générale ?

*Mme 2:* Là, en général, je les choisis parce que [bon] [ben] et [euh] en général ils sont de, ils sont de la région.

*Mme 1:* Ouais !

*Mme 2:* Ils sont de la région et [euh] [bon] [ben] ils me semblent quand même assez propres entre [euh] entre guillemets, quoi. [rire]

*Mme 1:* Ouais, vous entendez quoi par propre ? C'est quoi que vous voulez--

*Mme 2:* [ben] C'est à dire que peut-être quand même cultiver sans, sans excès de produits phytosanitaires comme ils disent.

*Mme 1:* Ouais ! Et en termes de qualité, de saveurs ou de conservation, est-ce que vous trouvez que c'est sensiblement pareil ?

*Mme 2:* Je vous dirai franchement que du point de vue saveurs, je n'ai jamais trouvé tellement de différence entre un chou romanesco ou [euh] un chou ordinaire, un chou de Bruxelles, enfin tous les choux, entre les bio et puis ceux que j'achète là-bas, j'ai pas de-- Pareil pour les endives, quand ça m'arrive.

*Mme 1:* Ouais !

*Mme 2:* Il y a, il n'y a pas vraiment de différences de goût.

*Mme 1:* Et de conservation, vous en trouvez une ou pas vraiment ?

*Mme 2:* Pas vraiment !

*Mme 1:* Non ?

*Mme 2:* [euh] Pour les fruits, pour les fruits, quand ça arrive que j'achète des fruits-- pour les fruits, si !

*Mme 1:* Ouais !

*Mme 2:* Ils sont plus vite abîmés quand c'est des bios, ça c'est sûr. En particulier, les citrons.

*Mme 1:* Ouais !

*Mme 2:* Alors, je ne sais pas pourquoi, mais les citrons bio-- [rire] C'est du champignon qu'ils font.

*Mme 1:* Oui ! [rire]

*Mme 2:* La moisissure.

*Mme 1:* Et cela peu importe, où est-ce que vous-- enfin, peu importe où vous les trouvez ? Que ce soit dans-- par exemple, les paniers [euh] les paniers bio ou des bio que vous trouvez en grandes surfaces, la conservation sera mauvaise pour les, pour les deux ?

*Mme 2:* [euh] À vrai, à vrai dire [euh] les citrons de grandes surfaces, je n'en trouve jamais des bio.

*Mme 1:* [Ah] ouais ?

*Mme 2:* À la grande surface, non. Ils n'en ont pas. Ils ont deux provenances. Il y a le citron vert aussi et le citron jaune. Et ils ont deux, [euh] enfin, pas deux provenances, je ne suis pas sûre encore. Mais deux emballages différents, c'est à dire, il y a le vrac.

*Mme 1:* Oui !

*Mme 2:* Ou il y a le filet.

*Mme 1:* D'accord !

*Mme 2:* Le filet, j'en prends jamais parce qu'il y en a trop dedans pour moi, le temps que je--

*Mme 1:* Ouais, ouais !

*Mme 2:* Même dans le pas bio, ils finissent par s'abîmer. Donc je les prends en vrac et non [euh] en grande surface, ici, ils s'abîment pas, quoi. Alors c'est, [ben] c'est, c'est préciser qu'il n'y a pas eu de produits phytosanitaires mis après la récolte. Ça, ils le disent, mais [bon], mais c'est tout [hein]. C'est la seule [euh] --

*Mme 1:* La seule indication.

*Mme 2:* La seule que l'on a quoi.

*Mme 1:* Oui ! Donc quand vous êtes en grande surface, du coup, vous n'achetez pas forcément bio ? C'est [euh]--

*Mme 2:* [Ben] en général, en général, je-- C'est rare que j'en prenne mais j'en prends quand même. Et c'est vrai qu'ils se conservent plus longtemps.

*Mme 1:* Ouais !

*Mme 2:* Ça, c'est sûr ! Ce qui fait que maintenant [ben] il y a des, il y a des fruits que je prends qu'en vraiment petite quantité.

*Mme 1:* Ouais !

*Mme 2:* Parce que sinon, sinon je les perds. Alors les pommes, là, ça n'a pas d'importance. Les clémentines non plus, mais les poires par exemple, j'en prends quand, j'en prends, j'en prends trois quoi.

*Mme 1:* Oui !

*Mme 2:* Et je retournerai. [rire] J'en reprendrai quatre jours après ou cinq jours après.

*Mme 1:* Oui ! Qu'est-ce qui vous amène à faire des courses assez régulièrement ?

*Mme 2:* Alors (euh] en revanche, pour les bananes, je n'ai pas trouvé de différence. Entre les bananes bios et les autres, c'est à dire, il n'y a pas de différence du point de vue [euh] du fait que ça s'abîme ou pas.

*Mme 1:* Oui ! Ouais !

*Mme 2:* C'est pareil ! Et du point de vue du goût, de toute façon, non.

*Mme 1:* Ouais ! D'accord ! Et donc [euh] au moment de de de de jeter un fruit et légume-- Alors vous m'avez dit que le potager [euh], c'était [euh] enfin que vous-- Jamais vous jetiez des fruits et légumes que vous aviez vous même plantés. Et est-ce que ça vous arrive de, dans d'autres, donc soit dans vos magasins, vos petites épiceries ou la grande surface, [euh] quand vous en perdez des fruits et légumes, qu'est-ce que vous ressentez ?

*Mme 2:* Ben [euh] ça m'agace un peu. [rire]

*Mme 1:* Ouais !

*Mme 2:* C'est évident, [euh] quand c'est un fruit où on peut sauver des choses et [ben] je j'enlève la partie abimée [hein]. Et fruits ou légumes--

*Mme 1:* Mm-hmm.

*Mme 2:* Et puis [ben]-- En revanche, là, j'ai jeté aujourd'hui, [euh] un peu de purée de potimarron que j'avais laissé dans mon frigo moisir.

*Mme 1:* Ouais !

*Mme 2:* Parce qu'en fait, quand je l'ai faite et que je l'ai mangée, je ne l'ai pas trouvée terrible. Et du coup, je l'ai oubliée. [rire] Et là, aujourd'hui, j'ai regardé ce qu'il y avait dans ce container. Je me suis dit [bon], c'est un peu trop tard. Voilà, c'est la dernière chose que j'ai mise à la poubelle ce matin. [rire]

*Mme 1:* Donc ouais, ça, c'est [euh] parce que vous l'aimiez pas trop.

*Mme 2:* [Ben] Parce que je sais pas comment, je l'ai préparé pourtant, comme d'habitude et tout et je ne l'ai pas trouvée vraiment bonne quoi, appétissante. Donc je l'ai carrément oubliée. Après j'ai plus envie d'y retourner, quoi. [rire]

*Mme 1:* Ouais !

*Mme 2:* Et du coup, [ben] là, c'était--

*Mme 1:* Il était temps ?

*Mme 2:* C'était juste bien pour que je la jette, quoi.

*Mme 1:* Ouais. [rire] OK ! Et [euh] est-ce que vous trouvez qu'il y a une différence dans-- Quand, quand vous jetez un produit de grandes surfaces ou d'autre part, est-ce que vous ressentez une différence dans votre agacement ou non, c'est le même, c'est le même agacement d'avoir perdu un produit ?

*Mme 2:* [ben] C'est un peu le même agacement parce que c'est, c'est de l'argent fichu en l'air, quoi. [rire]

*Mme 1:* Ouais ! Ouais, ce qui vous agace, c'est le fait d'avoir dépensé quelque chose [euh]--?

*Mme 2:* [ben] oui, c'est--

*Mme 1:* [ben] ui !

*Mme 2:* Enfin, des fois, c'est-- pour l'histoire de la machin potimarron, là, en fait, c'est moi qui-- Je m'en veux un peu parce que, un, je ne sais pas si ça venait du produit lui-même, mais ça m'étonnerait. Donc j'ai pas su le mettre en valeur et ça n'a pas été-- ça ne m'a pas satisfait et du coup, j'ai, je l'ai laissé perdre quoi. Mais là, la fautive, en fait, c'était moi. [rire] C'était ni le légume, ni--

*Mme 1:* Ouais !

*Mme 2:* Ni le-- Et là où je l'avais acheté, d'ailleurs, ça devait être un potimarron bio qui venait de- du Panier, du Grand Panier Bio.

*Mme 1:* Alors là, du coup, si je reprends ce que vous dites, si le fruit ou le légume s'abîme [euh] trop vite par rapport à ce que vous pensiez, [euh] ça va vous agacer d'avoir perdu de l'argent. Mais là, en l'occurrence, vous pensez que, vous vous en voulez parce que [euh]

*Mme 2:* [ben] je- je- oui ! Mais [euh] mais même, par exemple, quand je perds un fruit parce qu'il s'abîme un peu plus vite, parce qu'il est bio, justement, [ben] je me dis bon [ben] la prochaine fois, au lieu d'en prendre quatre, tu en prends trois ou tu en prends deux. Voilà ! Mais--

*Mme 1:* [silence 00:42:45-00:42:55] Oui, ça va.

*Mme 2:* Mais c'est pas toujours facile de faire des courses quand on est seul, parce qu'on n'a pas envie de revenir trop souvent, parce que c'est pénible de se déplacer aussi, il faut-- et- et d'un autre côté, [ben] on sait bien qu'on ne peut pas en prendre des tas parce que ça va justement s'abîmer quoi.

*Mme 1:* Alors autre petit jeu. On m'avait dit, vous consommez des bananes. Ça vous arrive de prendre des bananes ?

*Mme 2:* Oui.

*Mme 1:* D'ailleurs, c'est un jeu sur la banane. Alors, ici vous avez dix bananes. C'est la même, mais il y a des états d'avancement différents.

*Mme 2:* Il y en a qui sont très avancées. [rire]

*Mme 1:* Très avancées.

*Mme 2:* Est-ce que--

*Mme 1:* Et l'idée, je vais vous demander de les ranger. Celles qui vous donnent la plus envie et celles qui vous donnent le moins de goûts.

*Mme 2:* [silence 00:44:06-00:44:10] La moins envie entre celles-là, je- j'aurais du mal à [rire] à choisir. Pas trop forte. Celle-là est trop verte. Celle-là est un peu trop verte aussi.[silence 00:44:25-00:44:29] entre ces deux-là.

*Mme 1:* Ça, c'est laquelle la première ? Vous pouvez la mettre là et faire une--

*Mme 2:* [silence 00:44:36-00:44:40] C'est cette-- peut-être celle-là. Mais il n'y en a pas vraiment une qui est [euh] je ne sais pas parce que celle-là, elle a l'air d'avoir des- des marbrures noires-là qui me-- qui me gêne. Et celle-ci, j'ai l'impression qu'elle est quand même un peu verte.

*Mme 1:* Oui, ça c'est un peu plus verte.

*Mme 2:* Ouais, j'sais pas. Il y a celle-ci qui commence à avoir des-- je me demande si c'est pas la même [rire] qui a été photographiée.

*Mme 1:* C'est la même banane partout là, en fait.

*Mme 2:* [ah] c'est la même ?

*Mme 1:* Oui. C'est juste qu'elle a été pris à des moments [euh]

*Mme 2:* Il me semblait bien. Oui que--

*Mme 1:* Donc, c'est à quel moment que vous la préférez en gros ? Vous préférez [euh] la manger ?

*Mme 2:* J'hésite entre ces deux-là. [rire] [silence 00:45:30-00:45:37] Celle-là est un peu moins mûre que celle-ci. Mais [euh] elle manque déjà quand même des-- [oh], je vais peut-être prendre celle-là quand même.

*Mme 1:* C'est elle votre préférée ? Donc la deuxième, ce serait une orange, ce serait elle la deuxième ?

*Mme 2:* Oui.

*Mme 1:* Ensuite, la troisième, ce serait laquelle ? Il y a celle-ci.

*Mme 1:* [silence 00:45:58-00:46:02} Oui, celles-là, on les élimine [alors] parce qu'elles sont trop ou pas assez- pas assez mûres ou bien est-ce que--

*Mme 1:* On les met de côté pour l'instant ?

*Mme 2:* Oui.

*Mme 1:* Après, serait laquelle que- qui vous donne plus envie ?

*Mme 2:* Celle-ci à la limite. Celle-ci. Là, ça commence déjà à être un peu- [rire] un peu [hein]. Celle-là, je crois que je la mangerai pas [hein].

*Mme 1:* Oui. Et les vertes, est-ce que vous donnons ? Et alors, on-- si on devait comparer ces deux-là, laquelle vous fait la plus envie ?

*Mme 2:* Non, [ben] celle-là.

*Mme 1:* Celle-là, [alors] il faudrait la mettre [euh] celle-là. [Ben) et entre ces deux-là, si vous deviez en-- vous avez pas le choix d'en manger une, ce serait laquelle entre ces deux-là ?

*Mme 2:* J'ai pas le choix d'en manger une ?

*Mme 1:* [rire] [inaudible 00:46:44-00:46:45] manger la banane.

*Mme 2:* J'sais pas. Je mangerai quand même plutôt celle-là.

*Mme 1:* Donc, ça serait elle, d'accord !

*Mme 2:* Ça, c'est la verte verte là [euh]

*Mme 1:* Ouais. Et entre autres ces deux-là ?

*Mme 2:* [Ah] [ben], je mangerai celle-ci quand même.

*Mme 1:* La verte ?

*Mme 2:* [rire]

*Mme 1:* Et puis après, on a le choix.

*Mme 2:* Après les autres. [Ben] oui, c'est vrai. C'est la même.

*Mme 1:* Oui.

*Mme 2:* Celles-là, je les mangerais pas.

*Mme 1:* Celles-là, vous les mangerez pas ? [rire] On peut limite [euh]. Là, mais elles finissent à la poubelle. Je vais prendre en photo la manière dont-- ma banane préférée ?

*Mme 1:* [Alors], tac ! Je m'en souviens pas. Tac ! [Alors], si on prend la première, donc celle qui a le triangle vert, à votre avis, elle a quel goût ? [Alors], si vous dev- si vous deviez [euh] imaginer qu'elle est devant vous, comment vous la trouvez cette banane ? Vous pouvez me la décrire avec vos- vos cinq sens.

*Mme 2:* Comment est-ce que je peux vous dire ? [Ben] une banane pour moi, c'est-- [rire] elle a-- elle est quand même assez onctueuse, on dirait quoi [euh] [silence 00:47:56-00:48:02] bon, mais fff !

*Mme 1:* Donc, c'est-- et entre ces deux-là, entre la triangle vert et la rond et la carrée mm blanche ?

*Mme 2:* Y a pas tellement de différence quoi. Je pense que la- la verte est un peu plus mûre, donc [euh] pour moi [euh] à mon goût, [euh] elle est plus agréable quoi.

*Mme 1:* D'accord ! [silence 00:48:26-00:48:30] Et alors, le carré [euh] orange ?

*Mme 2:* [Ben] c'est un petit peu la même chose. Mais là, elle commence à devenir-- celle-ci surtout, un peu plus pâteuse.

*Mme 1:* La rond blond, un peu pâteux ?

*Mme 2:* Ouais, un peu pâteuse, oui. [rire] Certainement !

*Mme 1:* Et ça [euh], vous--

*Mme 2:* J'aime moins.

*Mme 1:* Vous aimez moins ?

*Mme 2:* Oui, oui. J'aime moins.

*Mme 1:* D'accord !

*Mme 2:* Celle-là, en- [en fait], on a plus l'impression d'un- d'un fruit pas mûr, c'est-à-dire que elle est- [silence 00:49:05-00:49:10] elle est presque un peu croquante, ce qu'on ne cherche pas dans une banane, en général. Et puis [euh], elle ne donne pas cette impression d'un peu sucré quoi, la banane.

*Mme 1:* Ouais. [silence 00:49:22-00:49:28] Donc, ça, c'était pour le triangle bleu. Ensuite, j'ai un carré bleu.

*Mme 2:* Mais le- le- le triangle bleu, je ne la mangerais que vraiment si j'avais rien d'autre à me mettre sous la dent. [rire]

*Mme 1:* Le carré bleu ?

*Mme 2:* [Ah] oui, parce que [rire] [euh] je pense qu'elle est-- qu'elle doit être très farineuse, pâteuse, agréable. Celle-ci, je la mangerai pas.

*Mme 1:* Les triangles roses, vous faites quoi de la triangle rose ?

*Mme 2:* [Ben] shhh ! [silence 00:49:55-00:49:58] J'ai pas l'habitude de le f- de faire ça. Je ne mange jamais mes- mes- mes fruits comme ça, mais si j'étais obligée, à la limite, je la cuirais.

*Mme 1:* D'accord ! Un triangle rose, vous le-- [euh] ?

*Mme 2:* À la limite, si je devais vraiment la- la consommer et je-- peut-être que je- je la ferais- [euh] la ferais frire avec un petit peu de miel dessus [euh]. C'est bon !

*Mme 1:* Oui. Et donc, celle-là, vous la cuisinerez, mais pas le- la carrée-- pas la carrée bleue ?

*Mme 2:* Non, non. [silence 00:50:29-00:50:34] Non. [silence 00:50:34-00:50:36] Non, pour une fois, j-j'étais. [rire]

*Mme 1:* Ouais, dès là- [euh] dès le carré bleu, vous- vous pouvez la jeter pour ça ?

*Mme 2:* Oui. [silence 00:50:45-00:50:49]

*Mme 1:* Oui. Seulement, vous avez plein dans le frigo [euh] [rire].

*Mme 2:* Donc, quant aux autres, n'en parlons pas.

*Mme 1:* Et donc là, oui, tout cela, vous faites rien avec. Elles partent à la poubelle ou quoi.

*Mme 2:* Ouais. [Ben] [euh] aux poules peut-être.

*Mme 1:* |Alors], vous avez des poules ?

*Mme 2:* J'ai des poules et c'est pas possible que [euh] oui [euh], celle-là [euh] à partir de là, ça part aux poules, quoi. Mais celle-là, je pourrais la cuisiner effectivement.

*Mme 1:* Ouais. Puis [euhm] le- là, celle du triangle rose, la banane verte, est-ce que [euhm] y a- y a des personnes qui m'ont dit pendant, qu'elles attendaient qu'elles deviennent un peu plus comme ça ?

*Mme 2:* Oui aussi.

*Mme 1:* Vous serez-- Oui.

*Mme 2:* Aussi oui.

*Mme 1:* Ça vous arrive [euh] jamais d'avoir des bananes comme ça presque chez vous finalement ?

*Mme 2:* Pratiquement, non. De toute façon, quand je les achète, je veille à ce qu'elles ne soient pas comme ça.

*Mme 1:* D'accord ! [rire]

*Mme 2:* [Alors], vous pouvez mettre [euh] [silence 00:51:41-00:51:45] ou bien- ou bien effectivement, j'attends qu'elles arrivent à ce stade ou à ce stade. Oui, voilà !

*Mme 1:* Oui. Mais aussi au final, quand vous les achetez, elles sont--

*Mme 2:* [Oh] en général, elles sont comme ça.

*Mme 1:* Elles sont vos triangles, mais--

*Mme 2:* Je m'arrange pour qu'elles soient pas trop vertes quand même.

*Mme 1:* Ouais. Donc, [en fait], vous pouvez [euh] quand vous les achetez [euh] ses limites [euh]. [diaphonie 00:51:55-00:52:07].

*Mme 2:* Je peux les garder plusieurs jours quand même, mais je les consomme au fur et à mesure.

*Mme 1:* Ouais. Et [euh] donc selon vous, est-ce qu'à un moment donné où [euh] la banane, elle [euh]- elle perd ou elle n'a pas encore ses apports nutritifs ou pas ? Le moine avait parlé tout à l'heure sur [euh]-- C'est inutile ! Ouais. Est-ce que selon vous, [ben] on peut- [euh] est-ce que quand elle est [euh] verte, par où on va dire ? Pas encore [euh] mûre, est-ce qu'elle a des apports nutritifs ? Est-ce qu'elle en gagne ? Est-ce qu'elle en perd au fur et à mesure ?

*Mme 2:* Je- je pense que [euh] jusqu'à-- de là, à là [euh] elle [euh] [silence 00:52:57-00:52:59] je sais pas dans quel sens, mais elle- elle doit gagner en digestibilité, en tout cas, de sucre parce que je sais que [Nom de l'enfant 2] et [Nom de l'enfant] qui ont des problèmes [euh] avec les sucres, [euh] elles peuvent manger des bananes mûres, mais pas vertes et pas trop mûres.

*Mme 1:* Ouais.

*Mme 2:* Il y a un équilibre de sucre dans- dans le fruit quoi.

*Mme 1:* [silence 00:53:25-00:53:32] D'accord ! Et [euh] selon vous, quand elle-- voilà- plus elle mûrit comme ça, est-ce qu'elle garde quand même ses apports [euh] nutritifs ou pas ?

*Mme 2:* [Ben] jusqu'à- à mon avis, jusqu'à ce stade-là peut-être et encore.

*Mme 1:* Jusqu'au carré bleu ?

*Mme 2:* Oui.

*Mme 1:* Et vous pensez qu'elle en a perdu quand même ? Ou [alors] en digestibilité ? [diaphonie 00:53:57-00:53:59]

*Mme 1:* Ça, je peux-- je- [euh] plus plus en digestibilité oui que en apport, je sais pas. Je suis pas assez spécialiste [rire] pour savoir.

*Mme 1:* Oui. Mais vous posez pas la question au moment de jeter-- de savoir si elle a encore des des bienfaits nutritionnels ?

*Mme 2:* Non, mais elle avait- elle est- peut-être qu'elle a des bienfaits nutritionnels, mais elle n'est plus appétissante et à la mâche, si on ose dire, la machin, elle est- elle n'est pas agréable quoi. Elle n'est plus agréable. [silence 00:54:29-00:54:34] Non, mais c'est vrai que quand- quand [euh] j'ai un fruit ou un machin abîmé, mais que mes poules peuvent en profiter, ça- ça m'est m- m- moins égal de les-- les jeter quoi.

*Mme 1:* Vous pensez que le fait d'avoir des poules [euh], ça- ça fait que vous avez moins de- peut-être de- de remords entre guillemets à- à aller sur-- Ouais, à jeter vos--

*Mme 2:* À jeter un fruit ?

*Mme 1:* Ouais.

*Mme 2:* Enfin, oui ! Nous- parce qu'elles ont-- elles en profitent, mais de toute façon [euh] Je fais un compost [hein] je- je ne jette rien à la poubelle. [rire] Belle poubelle quoi.

*Mme 1:* Et ça, vous pensez où est pareil, le compost peut-- ça [euh] ça peut changer un peu la façon dont vous voyez le moment de- de jeter les fruits ou légumes ou pas ?

*Mme 2:* Pas tellement, non ! Vous cuisinez quand vraiment-- En général d'ailleurs, c'est- c'est le compost, c'est- c'est plus les épluchures, les trucs comme ça. C'est- c'est pas le-- c'est pas le légume ou le fruit lui-même quoi. C'est [silence 00:55:54-00:55:58] sauf [euh] sauf quand tu- quand tu- j'ai un- un fruit abimé, donc je- je garde une partie. L'autre va au compost. Dans votre poubelle [rire] en ce moment, mais au compost d'habitude. [ben] elle finit et va sur le compost de toute façon.

*Mme 1:* Oui. Et [euh] est-ce qu'il y a un moment où la banane carrée bleue, vous avez dit, vous pouvez la donner aux poules ? Est-ce que la rond [euh] [diaphonie 00:56:24-00:56:26]

*Mme 2:* Oui, elles iraient- elles iront aux poules aussi [hein].

*Mme 1:* Elles iraient toutes aux poules ?

*Mme 2:* [oh] le- la- si elles se tiennent encore à peu près. [rire] [euh] quand on a enlevé la- la peau [euh] oui, si [euh] d'ailleurs, je verrais bien. Si elles n'en veulent pas, elles la mangeront pas.

*Mme 1:* Ouais. C'est sûr ! [rire]

*Mme 2:* C'est d'ailleurs [euh] c'est d'ailleurs assez- assez curieux parce que [euh] j'en ai- j'en ai deux là. [euh] elles ne- elles ne sautent pas forcément sur les mêmes aliments que les deux que j'avais avant. Elles ont aussi des- [rire] des goûts.

*Mme 1:* Leurs préférences. [silence 00:57:12-00:57:17] et [euh] directes questions sur les bananes. Est-ce que, selon vous, il y a un moment donné où elles peuvent être dangereuses pour la santé ou non ? C'est exclusivement en termes de goût et puis peut-être de digestion [euh] pour faciliter la digestion

*Mme 2:* [euh] je ne sais pas. Ça, j'avoue que--

*Mme 1:* C'est pas une question que vous vous posez quand vous--

*Mme 2:* J'en sais rien parce que [silence 00:57:37-00:57:41] je ne l'ai- je n'ai pas entendu dire, mais de toute façon [euh] là, c'est- c'est- c'est au goût que j'élimine.

*Mme 1:* [silence 00:57:49-00:57:58] OK ! Alors, presque pas rangé et on a un autre petit jeu. [silence 00:58:03-00:58:12] Alors, [00:58:12-00:58:20] pas de photo. [silence 00:58:21-00:58:25] Ici, on a des fruits et légumes que des personnes ou des participants en projet voyaient une poubelle non jetée. Hop ! Et là, il y a des photographies de ce qu'on peut faire des fruits et légumes. Donc là, en gros, on a une poubelle classique. Ici, on a un compost. Ici, on a des poules. Et l'idée, c'est que pour chaque photo, vous gardiez celle qui [euh] qui vous parle, c'est-à-dire que vous, vous avez chez vous que vous pouvez faire. Par exemple, dans le cas-là, des poubelles [euh] vous, celles-là, c'est pas votre poubelle. Vous, c'est plutôt compost ou--

*Mme 2:* Compost et poules.

*Mme 1:* Compost et poules. Donc ça, on peut les garder. Et là, l'idée, c'est de faire la même chose, mais là, c'est plutôt avec des préparations. Alors, est-ce que ça vous arrive de faire des poêlées de légumes, des gâteaux, et cétéra ? Et vous gardez celles qui vous parlent et [euh] celles qui vous parlent pas. On les- on les prend pas.

*Mme 2:* Et pour les légumes, les salades composées, oui. Des soupes, oui. Ça, c'est une soupe de- de tomates [diaphonie 00:59:27-00:59:29]

*Mme 1:* [rire] Elle est appétissante.

*Mme 2:* Ça, c'est une compote ?

*Mme 1:* Ouais.

*Mme 2:* Oui, ça- ça j'en fais aussi. [inaudible 00:59:38-00:59:39] Ça, c'est un smoothie ?

*Mme 1:* Ouais.

*Mme 2:* Je fais jamais.

*Mme 1:* Vous ne faites pas trop aussi.

*Mme 2:* Je veux pas de smoothie. [rire] Non.

*Mme 1:* Pas de gâteaux non plus. Vous en faites pas des gâteaux ?

*Mme 2:* [euh] pas souvent. Quand on-- quand chez des gens de temps en temps, mais bon--

*Mme 1:* Oui, c'est pas une pratique [euh]

*Mme 2:* Je fais un crumble ou je fais [euh]

*Mme 1:* Oui, c'est pas-- quand vous êtes toute seule, vous ne faites pas de gâteaux ?

*Mme 2:* [ah] non !

*Mme 1:* Tout ça, ça va être congelé, c'est comme vous avez fait avec vos petits pois [euh] ?

*Mme 2:* Oui, ça, ça- ça, ça m'arrive de conclure.

*Mme 1:* Ouais.

*Mme 2:* Et ça, c'est--

*Mme 1:* Ça, c'est un- c'est un gazpacho. Donc c'est un--

*Mme 2:* Despacho [diaphonie 01:00:17-01:00:19] non plus [silence 01:00:19-01:00:21] et ça, c'est une salade ?

*Mme 1:* Une salade de fruits, oui.

*Mme 2:* Une salade de fruits, oui. Ça, oui.

*Mme 1:* Ça vous arrive ? Et alors, j'ai perdu une [euh] une photo chez quelqu'un. Normalement, il y avait aussi des- des confitures. Des confitures ou des bocaux de ratatouille ou de la confiture [diaphonie 01:00:35-01:00:37]

*Mme 2:* Des confitures, j'en fais.

*Mme 1:* Donc, on va mettre [euh] enfin, on va dire, c'est [rire] si tu veux.

*Mme 2:* Des confitures, j'en fais, oui. Et cette année, [euh] ça fait deux ans, dès que j'ai pas pu faire de confiture d'abricots parce que [ben] il n'y a pas-- il n'y en a pas sur le marché quoi.

*Mme 1:* Ouais.

*Mme 2:* Et ça- ça me manque parce que j'ai du mal à faire la soudure du coup.

*Mme 1:* [ah] oui, avec les--

*Mme 1:* [ben] je fais de la fraise, de la gelée de pomme, des confitures de- de mûres et je faisais de l'abricot. Et donc, comme ça, j'arrivais à faire le--

*Mme 1:* Tous les autres fruits. [rire] Alors ! Là, je mets donc-- c'est des photos de fruits et légumes que des personnes ont jetés. L'idée, c'est de voir si vous vous les-- vous auriez jeté. Si oui, où ? Et si non, qu'est-ce que vous auriez pu faire avec ? Et de les mêmes du coup sur les photos [euh] en question. Donc là, c'est l'orange.

*Mme 2:* L'Orange, oui, mais c'est surtout le dessus quoi qui est abîmé. Faut voir si dans un cas- un cas comme ça, la pourriture est allée vers l'intérieur quoi parce que-- je sais pas. Je crois pas que j'en-- j'aurais pu en faire quelque chose.

*Mme 1:* Et du coup, vous la mettez au compost ou aux poules ?

*Mme 2:* [euh] au compost.

*Mme 1:* Au compost ? Elles ne mangent pas d'oranges, les poules ?

*Mme 2:* Non, [rire] non. Ça, si, ça, je me-- ça, je me débrouille avec. J'en- j'en ferais-- je- je pourrais les faire en- en petits légumes comme ça, en poêlé. Ça, c'est quoi ? C'est une [euh] une courgette ?

*Mme 1:* Ouais, qui apparemment un peu noircie.

*Mme 2:* [inaudible 01:02:20-01:02:21] vraiment abîmée ?

*Mme 1:* Mm-hmm !

*Mme 2:* Ouais. Celle-là, elle va aller au compost. [rire]

*Mme 1:* Les champs de compost.

*Mme 2:* Ça, ça ira bien aux poules, ça !

*Mme 1:* Eh ! [ben] Elle, c'est la fameuse banane. On a dit qu'elle est très bien pour les poules. [rire]

*Mme 2:* [silence 01:02:34-01:02:40] Celui-là, il vient de chez vous.

*Mme 1:* Ouais. [rire] C'est ça.

*Mme 2:* Parce qu'il y a un compost. [silence 01:02:47-01:02:51] Oui, il n'y a pas vraiment besoin- moyen d'en faire grand-chose là [hein].

*Mme 1:* C'est quoi là ? Ce sont les poires ?

*Mme 2:* Les poires, oui. Elles sont vraiment laides quoi. À la limite, on revient bien, j'aurais mangé ça, mais bon.

*Mme 1:* Compost.

*Mme 2:* La petite tomate [euh] [rire] même pas les poules [hein].

*Mme 1:* Même pas les poules ?

*Mme 2:* [ben] je crois pas qu'elles la mangeraient. Elles mangent pas tellement de fruits [euh]. Elles mangent plus volontiers de l'herbe et de la salade.

*Mme 1:* Ouais.

*Mme 1:* Mais que- que le- le fruit fruit autrement, est- est- elles mangent les trucs [euh] cuits quoi.

*Mme 1:* Oui, vos restes [euh]

*Mme 2:* De légumes cuits, ça arrive [euh] qu'elles les mangent [hein].

*Mme 1:* Et ça, cette tomate-là, un peu moisie. Vous pensez qu'elle est--

*Mme 2:* Je crois pas. [rire]

*Mme 1:* [ben] une tomate fraîche, elles la mangeraient ?

*Mme 2:* [euh] oui, elles la mangeraient et surtout, elles apprécient les graines à l'intérieur. Ça, c'est un radis ?

*Mme 1:* C'est un radis, ouais.

*Mme 2:* Les radis, j'en mange jamais. Je peux pas, je les supporte pas. [rire]

*Mme 1:* [ah] oui ! Bon, on peut l'enlever alors ?

*Mme 2:* Mais oui, on peut- on peut l'enlever ou le mettre [euh] le mettre au compost. Je ne sais pas.

*Mme 1:* De toute façon, c'est mieux si vous en achetez pas.

*Mme 2:* De toute façon, s'il avait été comme ça, je l'aurais mis au compost.

*Mme 2:* OK. [rire] je veux dire compost.

*Mme 1:* [ben] j'en ai mangé pendant des- des tas d'a- d'années [hein].

*Mme 1:* Oui, et maintenant, vous avez--

*Mme 2:* [ben] non, je le digère pas quoi. Le cocombre non plus.

*Mme 1:* Oui, ça, je me souviens du concombre.

*Mme 2:* Ça, c'est de la salade ?

*Mme 1:* Ouais.

*Mme 2:* Abîmé, ça ?

*Mme 1:* Ça, c'est vraiment aux poules.

*Mme 2:* Les poules.

*Mme 1:* Il faut voir les trop abîmés pour être mangés la- la- la salade ? [hum] !

*Mme 2:* La salade. Là, elle est-- la salade elle est- elles en prendront ce qu'elles [euh] [inaudible 01:04:46-01:04:47]

*Mme 1:* Ou là- là, il n'y a rien que vous gardez dans cette salade ?

*Mme 2:* [ben] elle est abîmée là, elle est abîmée là, elle est abîmée ici. Il n'y a pas grand-chose à en sortir. [rire] Ça m'arrive [hein] de trier comme ça parce que bon [ben] une salade, quand j'en achète une, c'est- c'est- c'est beaucoup. Je mets un bout de temps avant de la manger et c'est vrai que je la nettoie et cetera, je la mets au-- dans un [diaphonie 01:05:10-01:05:11] au frigo. Mais [euh] donc souvent, je perds ça effectivement, mais quand le reste est bon [ben], je la consomme. Bien sûr, il ne faut pas qu'elle soit quand même dans un état comme ça quoi. Celle-là, qu'est-ce qu'elle a ?

*Mme 1:* [rire]

*Mme 2:* C'est pas rien ? [ben] oui, moi, je la mange en--

*Mme 1:* En salade ?

*Mme 2:* En salade. [silence 01:05:33-01:05:36] Qu'est-ce que c'est que ça ?

*Mme 1:* C'est une carotte. [rire]

*Mme 2:* Une carotte ?

*Mme 1:* Je crois que c'est une carotte, oui.

*Mme 2:* Alors là, elle est vraiment abîmée [hein].

*Mme 1:* Ouais.

*Mme 2:* Et si je la mets aux poules, elles n'en mangeront pas. Non, non ! Cruel, elles n'en mangeront pas.

*Mme 1:* Et si elle était fraîche, elles en mangeraient ? Elles mangent des carottes fraîches, mais pas comme ça ?

*Mme 2:* Elles mangent les carottes cuites.

*Mme 1:* D'accord, mais jamais des carottes crues ?

*Mme 2:* Non, mais c'est délicat nos poules. [rire] C'est ça en fait.

*Mme 1:* [inaudible 01:06:08-01:06:09] en fait.

*Mme 2:* Les endives, on les laissait un petit peu aller loin quand même parce qu'en général, on s'en occupe avant. [rire]

*Mme 1:* Ouais.

*Mme 2:* On s'en occupe avant. [silence 01:06:22-01:06:26] Vous avez récupéré ça chez Leclerc ? [rire]

*Mme 1:* C'est le- c'est un- c'est une personne du- de l'étude qui m'a envoyé la photo pour me dire qu'il a demandé à quelques personnes de prendre des photos au moment où ils jetaient leurs fruits ou leurs légumes. Donc, tous les fruits et légumes que vous voyez là, c'est des gens qui participent comme vous à l'expérience et qui ont pris en photo le moment où ils jetaient leurs fruits ou légumes. En tout cas, c'est une personne qui avait acheté ces-- on dit vos Leclerc et qui a dû les oublier à un certain [rire] moment.

*Mme 2:* Les oublier ou quelque chose comme ça.

*Mme 1:* Ouais.

*Mme 2:* Donc [ben] ça, ça irait au- au compost.

*Mme 1:* Ouais. pareil ça, elles en mangent pas les- les poules ?

*Mme 2:* [ah] non, non. Crue, non.

*Mme 1:* Crue, non ?

*Mme 2:* Enfin, si ça dépend. [euh] ça m'arrive de leur donner les- les [euh]

*Mme 1:* Ouais, les trognons-là ?

*Mme 2:* Celles qui [euh] les premières feuilles quoi, qui sont dessus, qui sont un peu abîmées, mais qu'on n'épluche normalement quand on fera un machin. Et ça- ça, elles les mangent. Mais dans cet état-là, à la limite, si je l'ouvrais, peut-être bon [ben], mettre au compost. [rire] Et ça, c'est quoi ? C'est des- c'est du- du poireau ?

*Mme 1:* Ouais. C'est du vert, du poireau.

*Mme 2:* Le vert du poireau. Mais ça, c'est presque dans les épluchures ?

*Mme 1:* Ouais.

*Mme 2:* Ça, c'est le vert d'épluchures.

*Mme 1:* Vous, vous mangez pas le vert de- le p- le vert de poireau ?

*Mme 2:* [euh] le vert de poireau [ben] j'en mets dans ma soupe, mais là, il me paraît abîmé quand même, non ?

*Mme 1:* Ouais. Oui, cette partie-là, oui. Oui, il est abîmé.

*Mme 2:* Non, ça, c'est- ça, c'est la partie quand j'épluche le poireau, c'est la partie. S'il y a des feuilles abîmées comme ça, du vert, abîmé comme ça, il va de toute façon au compost.

*Mme 1:* Ouais. Et bien, aux poules ou au compost ?

*Mme 2:* Au compost.

*Mme 1:* Au compost ?

*Mme 2:* Les poules, elles n'en font rien. [rire] Et celui-là, qu'est-ce qui lui est arrivé ?

*Mme 1:* [ah] je ne sais pas son histoire encore.

*Mme 2:* Parce que je ne vois pas très bien là. Il est- il est moisi, il est pourri ou c'est la façon dont est prise la- la photo [diaphonie 01:08:35-01:08:36] il est abîmé ?

*Mme 1:* Là, ils sont-- En fait, là, il fait un peu flétri, il est un peu plissé ici. Peut-être, là, ils sont-- il fait un peu ridé un peu partout, là. Et là, peut-être un petit poc. Il commence à être un peu [euh]

*Mme 2:* Oui, non, un peu flétri. Il n'y a pas moyen d'en tirer quelque chose pour [euh] pour faire quoi que ce soit.

*Mme 1:* Ouais.

*Mme 1:* Parce que s'il y avait eu qu'un petit coin abîmé encore, on aurait pu le mettre à faire une poêlée de légumes. Mais là [silence 01:09:04-01:09:08]

*Mme 1:* Compost aussi.

*Mme 2:* Je mets beaucoup au--

*Mme 1:* [ben] de toute façon, c'est que des produits ont été jetés finalement. Vous avez quand même sauvé [euh] la tomate et [rire] les carottes.

*Mme 2:* Oui, mais celle-là, je vois pas pourquoi.

*Mme 1:* [ben] moi non plus. Je vous avoue [euh]

*Mme 2:* Finalement, je vois pas pourquoi elle a été jetée ?

*Mme 1:* Au début, quand la personne m'envoyait la photo. Si elle n'a pas compris, la dame. Elle m'envoie des- ces- tous ces fruits et légumes. Mais au moins, je suppose que ça se voit, il y a des choses qu'on ne voit pas [euh]. Je prends en photo le nom de votre logement. Ça se voit, il y a des-- on ne voit pas, mais elle était peut-être très molle, mais c'est vrai que là, on dirait qu'elle va très bien.

*Mme 2:* [ben] oui, elle a l'air bien quoi.

*Mme 2:* Oui.

*Mme 1:* J'sais pas trop. Hop ! [ben] très bien ! J'ai posé toutes mes questions. [rire] On pourra ranger tout le- tout le matériel. Parti ! Alors, toc !

**Household F31, Interview 1**

00:00:01
*Speaker 1:* Moi les engins!

00:00:04
*Speaker 2:* Ouai. Oh, ma voix. Vous allez la voir ma voix de casserole.

00:00:06
*Speaker 1:* Oh, bah ecoutez. Bah c'est la mienne que j'entends tout le temps, donc, [euh]. Je préfère entendre la votre. Alors du coup, pour commencer, je vous propose un petit jeu de devinette.

00:00:16
*Speaker 2:* Ouai, ouai.

00:00:16
*Speaker 1:* Je vais vous demander de penser à un fruit ou à un légume que vous aimez tout particulièrement.

00:00:20
*Speaker 2:* Ah! Y en a plein.

00:00:21
*Speaker 1:* Vous allez penser à ce fruit ou à ce légume--

00:00:22
*Speaker 2:* Ouai.

00:00:23
*Speaker 1:* Sans me dire son identité. L'idée, c'est que je devine--

00:00:25
*Speaker 2:* Ah, d'accord!

00:00:26
*Speaker 1:* Grâce à votre description.

00:00:26
*Speaker 2:* Ah! D'accord.

00:00:28
*Speaker 1:* Alors est-ce que vous avez un fruit ou un légume en tête?

00:00:28
*Speaker 2:* Oui, bah, tout de suite, jaune.

00:00:30
*Speaker 1:* OK.

00:00:32
*Speaker 2:* Et légume: vert.

00:00:34
*Speaker 1:* Légume vert. OK. Alors vous avez un fruit ou un- ou un légume en tête?

00:00:40
*Speaker 2:* Bah! Les deux- Les deux.

00:00:41
*Speaker 1:* Ah!

00:00:42
*Speaker 2:* Ouai.

00:00:42
*Speaker 1:* Alors, choisissant- choisissez-en un. Celui que vous préférez.

00:00:45
*Speaker 2:* Bon, on va dire jaune.

00:00:47
*Speaker 1:* OK. Allez-y raconté moi tout sur ce--

00:00:51
*Speaker 2:* Bah! De forme ronde.

00:00:52
*Speaker 1:* OK. Quoi d'autre?

00:00:53
*Speaker 2:* Bah, j'ai pas d'idée, je suis en train de l'éplucher.

00:00:59
*Speaker 1:* [rire] Ça va, je l'ai.

00:01:00
*Speaker 2:* Ah bon, ça va.

00:01:04
*Speaker 1:* OK. Qu'est-ce que vous aimez dans ce fruit?

00:01:07
*Speaker 2:* Bah! La fraîcheur!

00:01:09
*Speaker 1:* Ouai.

00:01:09
*Speaker 2:* Et bon, la saveur, [euh], voilà.

00:01:11
*Speaker 1:* Ouai.

00:01:12
*Speaker 2:* Ouai. C'est vraiment- Et puis, bon, c'est un fruit qui revient, en fait, tout l'ann- toute l'année, quoi.

00:01:16
*Speaker 1:* Ouai.

00:01:17
*Speaker 2:* Ouai, c'est--

00:01:20
*Speaker 1:* Ouai.

00:01:21
*Speaker 2:* C'est local, aussi, on va dire, voilà. Celles-là elles sont du 35. C'est pas si local que ça, mais c'est pas très loin, en fait.

00:01:27
*Speaker 1:* Oui.

00:01:28
*Speaker 2:* Je regarde souvent les provenances.

00:01:30
*Speaker 1:* Ouai?

00:01:30
*Speaker 2:* Ouai.

00:01:31
*Speaker 1:* En général, vous mangez quand ce fruit? Plutôt le matin, le midi, le soir, tout le temps?

00:01:37
*Speaker 2:* Oh, bah, ouai, c'est plutôt l'après-midi.

00:01:38
*Speaker 1:* Ouai?

00:01:38
*Speaker 2:* Mm-hmm. Ou est en fin de repas, on aime bien aussi, mais, ouai, c'est surtout l'après-midi parce que, [euh], c'est- c'est quelque chose que je dois éplucher. Et du coup, bah, pour les enfants, si j'en mange une, forcément, je vais en devoir faire- faire 10.

00:01:51
*Speaker 1:* Ouai.

00:01:51
*Speaker 2:* Et du coup bah, je les épl- je l'éplu- je les épluche entre les écoles, quoi. Donc, [euh], souvent, avant- après les avoir déposé, vers 14h, je...je fais ma petite salade, quoi.

00:01:59
*Speaker 1:* OK. Vous l'é...vous l'épluchez systématiquement?

00:02:02
*Speaker 2:* Ouai.

00:02:02
*Speaker 1:* Ouai?

00:02:02
*Speaker 2:* Oui, c'est ça. Pour les plus jeunes, en fait. Et puis, comme, en fait, j'en fait- je me dis: «Bah, allez, je fais la série», je fais pour les plus grands aussi.

00:02:07
*Speaker 1:* Oui, oui.

00:02:08
*Speaker 2:* Et puis, comme ça, c'est plus rapide. Moi, c'est quelque chose qui passe bien.

00:02:13
*Speaker 1:* Ouai. Oui, c'est consommé par à peu près tout le monde dans votre foyer?

00:02:14
*Speaker 2:* Ouai.

00:02:14
*Speaker 1:* Ouai?

00:02:17
*Speaker 2:* On a des pommiers aussi. On a un verger.

00:02:22
*Speaker 1:* D'accord. Ok. [euh], donc du coup vous l'épluchez. Est-ce que vous faite autre chose, [euh]--

00:02:25
*Speaker 2:* Avec, [euh]?

00:02:26
*Speaker 1:* Avant de les consommer?

00:02:29
*Speaker 2:* Avec ces- ces- ces- C'est des pommes?

00:02:32
*Speaker 1:* Ouai.

00:02:32
*Speaker 2:* Ouai, je fais des crumbles, en hiver, ça c'est plutôt l'hiver. Ouai, parce que, bon, l'hiver, décembre/février, ça va être ça, décembre/février, ça va être des crumbles, [euh], des compotes, éventuellement.

00:02:43
*Speaker 1:* Ouai.

00:02:43
*Speaker 2:* Donc, voilà. Ouai. Ça tourne autour de ça. Donc, c'est très salade après.

00:02:46
*Speaker 1:* Vous aimez ça en salade!

00:02:49
*Speaker 2:* Ouai, ouai, ouai.

00:02:49
*Speaker 1:* En salade de fruits?

00:02:52
*Speaker 2:* Oui, salade de fruit, ouai. Ou, alors, si vraiment j'ai pas le temps, j'ai pas la boite, ou--parce que je complète avec une boîte, c'est pas l'idéal, bah, [euh], ça va être seulement des pommes parce qu'ils adorent ça.

00:02:59
*Speaker 1:* OK.

00:02:59
*Speaker 2:* Et puis, bah, voilà. C'est très basique et ça marche bien.

00:03:04
*Speaker 1:* Ben, ouai. C'est ça le principal. OK. Où est-ce que vous l'achetez le plus souvent?

00:03:12
*Speaker 2:* Alors, à part mon jardin, bah c'est ici à Leclerc, [euh], celle-ci, [euh], c'est, [euh], en fait, ouai c'était 49, c'était pas 35. J'avais vu 35 à Casino, bah, c'est Leclerc, en fait. Leclerc ou Casino.

00:03:14
*Speaker 1:* Ok. C'est le supermarché .

00:03:26
*Speaker 2:* Ou Intermarché. Ouai, c'est ça. Parce que, bon, au marché, pas le temps. J'ai pas eu le temps de faire le tour des marchés.

00:03:31
*Speaker 1:* Oui, oui.

00:03:32
*Speaker 2:* Moi, je fais le marché le mercredi, mais pour ça, j'ai pas le temps. Il y a beaucoup de queue, [euh].

00:03:39
*Speaker 1:* Vous allez acheter autre chose sur le marché, mais pas les fruits et légumes, c'est ça?

00:03:42
*Speaker 2:* Ouai, c'est ça.

00:03:42
*Speaker 1:* Ouai?

00:03:43
*Speaker 2:* Ouai, ouai, tout à fait. Bah, les légumes non. Les légumes, c'est avec le maraîcher du coin, mais il ne fait pas de fruits. Donc, les fruits c'est vraiment le truc à part que je vais acheter en grandes surfaces, ouai.

00:03:51
*Speaker 1:* D'accord. ouai.

00:03:53
*Speaker 2:* Ouai. maraîcher est en face de chez nous par rapport à la Berre et- elle c'est- c'est chez elle que je prends les carottes, les patates, que je prends en grosse quantité, les oignons, les échalotes, les choux fleurs, les choux verts, enfin, toutes les séries de choux. Elle a des fraises quand même. Ah! Elle a des fraises quand même, je note quand même.

00:04:08
*Speaker 1:* [Rire]

00:04:09
*Speaker 2:* Ah! Non, elles sont revenues et on s'est régalé. Voilà, c'est déjà ça, ça complète bien la salade de fruits.

00:04:17
*Speaker 1:* Ouai, c'est sûr. Et [euh], Alors [euh], En plus de la fraicheur [euh], de la saveur...--

00:04:25
*Speaker 2:* Oui.

00:04:25
*Speaker 1:* Que vous aimez, et puis le fait que tout le monde l'apprécie dans votre foyer--

00:04:28
*Speaker 2:* Ouai.

00:04:28
*Speaker 1:* Est-ce qu'il y a autre chose que vous- que vous trouvez dans la pomme? Est-ce que, selon vous, elle vous apporte autre chose ou pas?

00:04:34
*Speaker 2:* Ouai, des vitamines. En principe elle devrait m'en apporter, mais.

00:04:39
*Speaker 1:* Oui. Pourquoi en principe?

00:04:41
*Speaker 2:* Parce que ça devrait être de la, bon, bah, pas que elles soient traitées vraiment, quoi.

00:04:42
*Speaker 2:* Ouai.

00:04:42
*Speaker 2:* Parce que là, moi, je prends grand- grande série, [euh], je sais pas comment elles s'appellent ces pommes, mais elles sont pas toujours dans le bio, évidemment. Moi, je cherche à privilégier le bio, mais après, [euh], c'est du bio de grande surface, donc, [euh].

00:04:56
*Speaker 1:* Ouai.

00:04:56
*Speaker 2:* Clairement j'irai pas m'acheter ailleurs, parce que ça va être 10 fois plus cher.

00:05:00
*Speaker 1:* Ouai.

00:05:00
*Speaker 2:* Il y a aussi la question budget, quoi.

00:05:02
*Speaker 1:* Oui.

00:05:04
*Speaker 2:* Ça c'est--C'est vrai que c'est-- chez nous, c'est-- le plan bon est bio, [euh].

00:05:07
*Speaker 1:* Ouai.

00:05:08
*Speaker 2:* C'est pas terrible non plus, mais. On essaie de se donner un maximum bonne conscience. Bon, après, je me dis dans les pommes, c'est peut-être pas là qui en a le plus par rapport aux vignes, par exemple.

00:05:19
*Speaker 1:* Ouai.

00:05:19
*Speaker 2:* Je me dis c'est peut-être pas-- Les traitements sont pas si élevés, je sais pas. Je pense pas.

00:05:24
*Speaker 1:* Donc, si vous pouvez vous privilégiez le bio mais si--

00:05:25
*Speaker 2:* Ouai, voilà, c'est ça.

00:05:25
*Speaker 1:* Mais si c'est pas du bio de grande surface et des fois, bah--

00:05:29
*Speaker 2:* Exactement.

00:05:29
*Speaker 1:* Au niveau du prix vous pouvez pas toujours faire bio et--

00:05:32
*Speaker 2:* Ouai, voilà.

00:05:33
*Speaker 1:* Ok. Et sinon, la pomme, est-ce que vous lui trouvez un- un ou des inconvénients ou pas?

00:05:39
*Speaker 2:* Oh, bah, les inconvénients de grandes surfaces, ouai. En fait, c'est que, [euh], elle a pas du tout la même saveur que celle du jardin.

00:05:43
*Speaker 1:* D'accord.

00:05:43
*Speaker 2:* Evidemment, si on compare, alors là, celles du jardin, ça va être les crumbles qui n'auront pas du tout la même saveur. Et, en plus, j'aime beaucoup l'acidité et y en a pas du tout ça, en fait, c'est assez fade.

00:05:54
*Speaker 1:* Ouai, je vois- je vois. OK. Ça sa marche. Alors là, je voudrais m'intéresser à vos pratiques d'achat. Vous m'avais déjà commencé à me- m'en parler. Donc les fruits ça va être en grandes surfaces et par contre les légumes vous allez au maraîcher du coin.

00:06:11
*Speaker 2:* Oui.

00:06:12
*Speaker 1:* Alors, est-ce que c'est comme ça pour tous vos fruits et légumes ou ça peut varier? Vous avez d'autres?

00:06:17
*Speaker 2:* Non, ça varie pas, en fait. C'est très- C'est très quotidien. Ça c'est, ouai, c'est très routinier chez nous.

00:06:24
*Speaker 1:* Ouai.

00:06:24
*Speaker 2:* Ouai, ouai, c'est ça. Je vais fermer ici, y des corrants d'air, on va faire «Atchoum», en plus je vais être en retard. Y a une vieille patate lá qui comence à se ressentir et elle germe c'est la- la dernière cuvette de pommes de terre.

00:06:38
*Speaker 1:* Ouai.

00:06:39
*Speaker 2:* Elles sont dans un état.

00:06:43
*Speaker 1:* [Rire]. Et du coup pendant vos courses vous vous organisez comment? Est-ce que vous savez ce que vous allez acheter avant ou pas vraiment?

00:06:48
*Speaker 2:* Non, ça va être--

00:06:49
*Speaker 1:* Non?

00:06:50
*Speaker 2:* Ça dépend de ce qu'il y a dans l'étalage. Bon, les pommes de terre, très clairement, je passe chez elle parce que je les prends par 10 sacs de 25.

00:06:56
*Speaker 1:* D'accord.

00:06:56
*Speaker 2:* Donc, j'ai deux approvisionnements par an. Un en octobre et un autre en janvier/février, plutôt au début février.

00:07:04
*Speaker 1:* D'accord.

00:07:04
*Speaker 2:* Voilà. Comme ça, je fais- je fais trois, quatre mois, [euh], en autonomie en pommes de terre.

00:07:10
*Speaker 1:* Donc, c'est deux sacs- 10 sacs de 25?

00:07:12
*Speaker 2:* Ouai.

00:07:13
*Speaker 1:* De 25, [euh]--

00:07:14
*Speaker 2:* Kilos.

00:07:15
*Speaker 1:* Kilos?

00:07:15
*Speaker 2:* Ouai.

00:07:15
*Speaker 1:* Ah ouai, ça commence à faire [imperceptible].

00:07:17
*Speaker 2:* Ouai, ouai, c'est ça, ouai. Et en fait, en quantité d'oignons je peux vous le dire aussi, c'est un sac à chaque fois que j'y vais pour mes sacs de pommes de terre. Donc ça va être un sac de 10 ou 15 kilos, je sais plus. Je crois que c'est 10.

00:07:27
*Speaker 1:* Ouai.

00:07:28
*Speaker 2:* Voilà. Là au début de saison, en octobre, j'en avais fait deux de 10 et puis j'en avais fait un autre de 10 en février.

00:07:33
*Speaker 1:* D'accord, alors tous les quatre, cinq mois, 10 kilos, quoi.

00:07:35
*Speaker 2:* Voilà.

00:07:35
*Speaker 1:* À peu près.

00:07:35
*Speaker 2:* Ouai, c'est ça.

00:07:39
*Speaker 1:* OK. [euh], et donc vous n'avez pas forcément d'organisation spécifique--

00:07:45
*Speaker 2:* Non.

00:07:45
*Speaker 1:* Vous y allez selon ce qu'il y a, sauf, bah, pour les patates, quand vous avez que--

00:07:49
*Speaker 2:* Ouai, voilá, c'est ça. La patate ça va être le- le- la substance constante à chaque fois.

00:07:52
*Speaker 1:* Ouai.

00:07:53
*Speaker 2:* Mais après, [euh], moi, [euh], souvent, quand je- je vais dans son étalage, c'est tous les mercredis matin, donc j'y vais de bonne heure parce que-- pour pas trop faire la queue.

00:08:02
*Speaker 1:* Ouai.

00:08:03
*Speaker 2:* Et alors-- Et ben, là, [euh], il y a des choux fleurs, j'en ai pris. Il y a eu aussi avant des épinards--

00:08:07
*Speaker 1:* D'accord.

00:08:08
*Speaker 2:* Choux verts. Bon, ça va dépendre de ce qu'elle présente dans-- Puis j'ai pris des asperges aussi.

00:08:12
*Speaker 1:* OK.

00:08:13
*Speaker 2:* Voilà.

00:08:14
*Speaker 1:* Et vous y allez tous les combien de temps à peu près, [euh], au maraîcher?

00:08:17
*Speaker 2:* Tous les mercre--Ah, oui, alors chez elle, [euh], au marché c'est tous les mercredis matin.

00:08:21
*Speaker 1:* D'accord.

00:08:22
*Speaker 2:* Et ben, le vendredi soir, chez elle, [euh], bah, des fois je me fais un petit complément, ça dépend. Alors, combien ça va être? [euh], allez on va dire deux fois par mois.

00:08:31
*Speaker 1:* D'accord.

00:08:31
*Speaker 2:* Ouai. Ou je complète, [euh], je me dis "Ben, le mercredi ça ne m'a pas suffi." Donc je complète avec elle en fin de semaine.

00:08:37
*Speaker 1:* D'accord.

00:08:39
*Speaker 2:* Voilà.

00:08:39
*Speaker 1:* Donc, tous les mercredis matin et quelque fois un vendredi sur deux--

00:08:42
*Speaker 2:* Ouai, voilà ou trois même.

00:08:43
*Speaker 1:* Ou trois, de temps en temps.

00:08:46
*Speaker 2:* Des fois elle ferme aussi. Elle n'ouvre pas toujours au public chez elle, donc, [euh].

00:08:49
*Speaker 1:* Ouai.

00:08:49
*Speaker 2:* Mais bon, ça va.

00:08:50
*Speaker 1:* OK. Et donc, les fruits, par contre, ce sera plutôt du côté-- quand vous faites vos courses au supermarché.

00:08:56
*Speaker 2:* Ouai, voilà c'est ça.

00:09:00
*Speaker 1:* [Rire] Et au supermarché, vous y allez tous les combien à peu près? vous savais le dire?

00:09:01
*Speaker 2:* [euh], ouai, j'y vais deux fois par semaine.

00:09:04
*Speaker 1:* Deux fois par semaine?

00:09:06
*Speaker 2:* Ouai, ouai. C'est pas du tout synthétique. Mais enfin, en même temps, si je prenais des bananes pour 15 jours, je suis sûr que ça tiendra pas la route, donc.

00:09:14
*Speaker 1:* Ouai.

00:09:16
*Speaker 2:* En fait, elles sont vertes, donc une semaine avant, faut que j'anticipe, je me dise: «Ah, ouai, tiens je vais peut être faire un smoothie dans 15 jours ». Parce qu'ici, c'est le problème, c'est que les bananes sont vertes, quoi, quand on les achète. Et du coup là, moi je- je- je les mange comme ça, quasiment très noir, quoi.

00:09:29
*Speaker 1:* Ouai.

00:09:30
*Speaker 2:* Je les adore, elles ont de la saveur. Avant, c'est pas bon.

00:09:33
*Speaker 1:* Ouai.

00:09:33
*Speaker 2:* C'est immangeable. Même les enfants s'en rendent compte.

00:09:34
*Speaker 1:* Donc, les enfants ils préfèrent aussi la--

00:09:37
*Speaker 2:* Ouai. Je leur est fait comprendre qu'il fallait attendre et que des fois elles sont vertes, et on a plus que les vertes. Et on attend. [Rire]

00:09:41
*Speaker 1:* Ouais. Il faut que vous résistez. [rire]

00:09:43
*Speaker 2:* Ouai, c'est ça. Il faut lutter. On fait les pommes autrement à côté.

00:09:45
*Speaker 1:* OK. Vous essayez d'anti- Vous êtes obligé anticiper et que--

00:09:49
*Speaker 2:* Ouai, c'est ça.

00:09:50
*Speaker 1:* Elles vont être bonne dans plusieurs--

00:09:51
*Speaker 2:* Voilà, c'est ça. Il faut que j'achète mes bananes deux semaines avant.

00:09:51
*Speaker 1:* D'accord.

00:09:51
*Speaker 2:* Volià, c'est ça la particularité.

00:09:51
*Speaker 1:* D'accord.

00:09:56
*Speaker 2:* C'est assez chaud.

00:09:57
*Speaker 1:* Ouai.

00:09:57
*Speaker 2:* Bah, du coup, voilà, à ce moment il faut que j'en rachète à chaque fois, voilà, donc, [euh].

00:10:00
*Speaker 1:* Ouai. [rire]. Et en terme de quantité, justement, comment vous arrivez à savoir ce qui-- ce qu'il vous faut?

00:10:07
*Speaker 2:* Ouai, bah, ça va être--ça, ça va être deux grappes comme ça, quoi, on va dire.

00:10:12
*Speaker 2:* Ouai.

00:10:12
*Speaker 2:* Donc, en quantité--Je peux aller vous les peser parce que j'ai une petite balance.

00:10:13
*Speaker 1:* [silence]

00:10:21
*Speaker 2:* J'ai ça. Bah ça, c'était, [euh], c'était pour cette semaine. Il y a déjà une semaine que je les ai acheté. Donc, lá déjà, vous avez presque 250, on va dire.

00:10:27
*Speaker 1:* Ouai.

00:10:30
*Speaker 2:* 250 plus 250 parce qu'il était entamé. Je pense qu'il y en a-- Ouai, il y en a presque 500. Il y en avait pour 500 grammes à peu près ou alors on va dire, [euh], un kilo. On va dire un kilo.

00:10:39
*Speaker 1:* Pour une semaine--

00:10:40
*Speaker 2:* Ouai, pour une semaine.

00:10:40
*Speaker 1:* Un kilo de--

00:10:41
*Speaker 2:* Voilà, c'est ça.

00:10:44
*Speaker 1:* D'accord.

00:10:44
*Speaker 2:* La pomme, bah, en ce moment, je dis oui. Parce qu'on ne va pas faire que des pommes non plus. J'aime bien varier alors-- Celles-là je sais pas combien c'est.

00:10:46
*Speaker 1:* Vous en avez de--

00:10:46
*Speaker 2:* Ouai, [euh], je sais pas trop. Un kilo cinq.

00:10:49
*Speaker 1:* Un kilo cinq? Ça vous fait combien de temps ça, par exemple.

00:10:50
*Speaker 2:* Un kilo cinq, bah, ça va me faire une semaine.

00:11:04
*Speaker 1:* Ouai.

00:11:04
*Speaker 2:* Mm-hmm.

00:11:04
*Speaker 1:* Et pour le reste, les légumes comment-comment vous gérez, [euh], comment vous savez quelle quantité vous devez prendre? Alors que les pommes de terre, c'est différent, vous faites un gros aprovisionnement.

00:11:13
*Speaker 2:* Ouai, c'est ça.

00:11:13
*Speaker 1:* Mais pour le reste, par exemple le chou-fleur--

00:11:16
*Speaker 2:* Ouai. Le chou-fleur j'en prends un, parce que tout le monde n'aime pas.

00:11:18
*Speaker 1:* D'accord.

00:11:19
*Speaker 2:* Mais à côté de ça, je vais prendre, [euh], un chou vert, peut être pour compléter.

00:11:24
*Speaker 1:* D'accord.

00:11:24
*Speaker 2:* Ils sont pas très chou.

00:11:25
*Speaker 1:* Ouai.

00:11:25
*Speaker 2:* Ça dépend. Après il faut que je mette aussi toujours de la pomme de terre pour ceux qui n'aiment pas.

00:11:27
*Speaker 1:* Ouai.

00:11:27
*Speaker 2:* Je m'adapte à ceux qui n'aiment pas quand même.

00:11:29
*Speaker 1:* [rire]

00:11:33
*Speaker 2:* [inaudible]. En ce moment je fais pas mal de cougettes avec poivron. Alors, je sais que c'est pas trop la saison des poivrons, mais. Les poivrons aussi---

00:11:43
*Speaker 1:* Ça a tendance a commencer.

00:11:47
*Speaker 2:* Ah ouai?

00:11:48
*Speaker 1:* Ouai, ouai, ouai.

00:11:48
*Speaker 2:* Ah ouai! Alors il y en pour combien? Ouai, j'en ai pour deux fois cinq, en fait. J'en ai pour un kilo cinq de courgettes.

00:11:51
*Speaker 1:* Donc, un kilo cinq de courgettes.

00:11:57
*Speaker 2:* De courgettes-- Ah, oui, mais alors attendez, ça va peut être me faire, ouai, j'en met pas systématiquement parce que lá j'ai fait une- un petit peu de poulet, donc j'en ai juste découpé une.

00:12:05
*Speaker 1:* Ouai.

00:12:05
*Speaker 2:* Ça m'arrive de faire des petits trucs sporadiques.

00:12:07
*Speaker 1:* Ouai.

00:12:07
*Speaker 2:* Parceque comme maintenant, on est dans une saison où on a bien un peu mélangé de tout.

00:12:11
*Speaker 1:* Ouai.

00:12:11
*Speaker 2:* Donc, un kilo cinq. On va dire un kilo la semaine.

00:12:14
*Speaker 1:* D'accord.

00:12:14
*Speaker 2:* C'est honnête. Alors, ça va être plus en hiver. Forcement, je complète mes soupes avec ça.

00:12:21
*Speaker 1:* Ouai. Dans vos-- Par exemple, ouai, dans vos soupes vous mettez quoi souvent?

00:12:30
*Speaker 2:* Alors courgettes, patates, oignons, ail. Ça, c'est très rapide. Après, ça va être-- quoi d'autre? Bah, forcément, des poireaux, quoi.

00:12:39
*Speaker 1:* Ouai.

00:12:39
*Speaker 2:* Ouai, ça fait un peu de fibres. Et puis, voilà quoi, c'est à peu près- Carotte, éventuellement, mais je suis très peu fan de la carotte en soupe.

00:12:47
*Speaker 1:* D'accord.

00:12:48
*Speaker 2:* Bah, après ça dépend. Il y en a qui vont me dire: « Oh! Je préfère une purée de-de carottes», à ce moment, je vais en faire une.

00:12:52
*Speaker 1:* D'accord.

00:12:52
*Speaker 2:* Ça a plus de saveur, bon. En soupe sa casse.

00:13:00
*Speaker 1:* [rire]. OK.

00:13:00
*Speaker 2:* Y a des courges aussi, [euh], courges, [euh], potimarrons, potirons. Mais maintenant c'est fini ça, en fait. On est plus dans une saison, non plus, de--de courges, quoi.

00:13:07
*Speaker 1:* Mm-hmm. C'est vrai. C'est plutôt l'hiver.

00:13:12
*Speaker 2:* Ouai, c'est très bon.

00:13:12
*Speaker 1:* Ouai. [rire]. OK. Et donc du coup, [euh], finalement, les quantités. Si vous savez que c'est un--un légume qui--ou un fruit qui est très consommé chez vous, vous allez presque faire--partir sur un kilo par semaine.

00:13:28
*Speaker 2:* Ouai.

00:13:28
*Speaker 1:* Et sinon, ça va être plutôt, par exemple le chou-fleur, ça va être un chou-fleur par ci, par là.

00:13:32
*Speaker 2:* Ouai, c'est ça.

00:13:33
*Speaker 1:* Parce que vous savez que ça va pas être non plus le--

00:13:34
*Speaker 2:* Oui, oui, ça c'est-- Ouai, c'est ça.

00:13:39
*Speaker 1:* Ok.

00:13:41
*Speaker 2:* Mm-hmm. C'est vrai que je refléchi pas trop, [euh], à mon avancement mais, ouais--je fais un peu comme ça, quoi. Ouai. C'est un peu menu à la carte ici.

00:13:49
*Speaker 1:* [Rire].

00:13:49
*Speaker 2:* Bon, après ce qu'on aime aussi, en hiver ou en entrée parce qu'en ce moment c'est plutôt concombre alors qu'ici c'est pas trop la saison, concombre et tomates. Parce que c'est la fraîcheur qui recherche. En hiver, ça va être plutôt des-des choux rouges, des choux verts râpés avec carottes râpées aussi, parce qu'on est plus carottes râpées que carottes cuites.

00:14:05
*Speaker 1:* Ouai.

00:14:07
*Speaker 2:* Oui, c'est vrai qu'on y revient dans notre pensée aujourd'hui, on se dit qu'on ne fait pas de jus de légumes, de jus crus.

00:14:12
*Speaker 1:* Mm-hmm.

00:14:13
*Speaker 2:* Et on se dit: « Pourquoi pas?». Parce que, [euh], avec notre enfant, qui-- qui a des problèmes de toux, je me dis: «Pour drainer tout, on devra faire des jus de légumes». Et ça, c'est quelque chose qu'on ne maîtrise pas encore.

00:14:23
*Speaker 1:* Il faudrait--

00:14:23
*Speaker 2:* On va essayer de maîtriser ce côté là. Il y a des machines qui existent d'ailleurs.

00:14:27
*Speaker 1:* Ouai. C'est vrai que tout le monde fait un peu de soupe, enfin, voilà les soupes--

00:14:31
*Speaker 2:* Ouai. Ouai c'est ça.

00:14:33
*Speaker 1:* C'est comme [imperceptible]. Les légumes, les crudités,quoi--

00:14:37
*Speaker 2:* Bah, oui.

00:14:37
*Speaker 1:* qui sont--

00:14:38
*Speaker 2:* Ah, bah, le cuit vapeur par contre, non, c'est--C'est cuit vapeur chez nous plus ou moins.

00:14:40
*Speaker 1:* D'accord.

00:14:40
*Speaker 2:* Mais mon- ma cuve elle a dans-- le fond de ma marmite est tellement près de l'eau que je me dis, [euh], c'est très relatif quoi. Donc je pense que tout est grillé, cuit, quoi.

00:14:49
*Speaker 1:* D'accord.

00:14:51
*Speaker 2:* Pour l'instant, c'est pas bien maîtrisé, c'est vrai. C'est pas bien. Enfin bon, il vaut mieux ça que--

00:15:04
*Speaker 1:* Donc, OK. Et sinon--Alors, si on fait une liste des fruits et légumes que vous avez l'habitude de consommer, vous en avez déjà cité pas mal.

00:15:11
*Speaker 1:* Ouai.

00:15:11
*Speaker 1:* Les fruits, vous m'avez dit les pommes, les bananes. Est-ce qu'il y en a d'autres que--les fraises qui sont--

00:15:16
*Speaker 2:* Oui, c'est ça. Les fraises jusqu'en septembre/octobre là.

00:15:18
*Speaker 1:* D'accord.

00:15:19
*Speaker 2:* Donc là, j'en prends, [euh], ouai, un kilo par semaine aussi.

00:15:22
*Speaker 1:* Un kilo de fraises?

00:15:23
*Speaker 2:* Ouai, c'est ça. C'est 500 et plus 500. Alors là, j'ai fait deux variétés, mais j'ai repéré une nouvelle variété qui est super, [euh], sucrée, donc.

00:15:29
*Speaker 1:* Ouai.

00:15:31
*Speaker 2:* Elle est beaucoup plus rouge foncé, d'ailleurs.

00:15:32
*Speaker 1:* D'accord.

00:15:34
*Speaker 2:* Je ne sais pas quel est l'appellation, mais elle était vraiment, [euh], plus près, [euh], de la fraise des bois. Ce que je recherche depuis longtemps. [rire]. Et là comme il n'y a pas assez de soleil non plus, c'est peut être qu'elles ne sont pas développées non plus dans le sucre, les- celles que je connais.

00:15:48
*Speaker 1:* Oui.

00:15:49
*Speaker 2:* Enfin, il faut faire la guête.

00:15:50
*Speaker 1:* Et [euh], donc les fraises, les pommes, les bananes? Est-ce qu'il y a d'autres fruits que vous allez consommer régulièrement?

00:15:55
*Speaker 2:* Ouai. En dehors des boîtes de conserve, hein.

00:15:58
*Speaker 1:* Ouai. Oui en frais, parce que--

00:16:00
*Speaker 2:* Ouai, en frais, c'est ça. Bah, l'abricot ça va être juste, hein, parce que franchement-- l'abricot est dur, donc ça va être en boite quoi.

00:16:08
*Speaker 1:* D'accord. Sinon après--

00:16:09
*Speaker 2:* Poire, éventuellement, je vais à Casino parce qu'elles sont- elles sont souvent bien molles en pleine saison.

00:16:13
*Speaker 1:* D'accord.

00:16:13
*Speaker 2:* Elles sont bien fraîches jusqu'en--oh, jusqu'en décembre. Je pourrais en avoir des bonnes jusqu'en décembre.

00:16:18
*Speaker 1:* D'accord.

00:16:18
*Speaker 2:* Je sais pas comment ils font pour en avoir-- leur conservation, mais elles sont bien. Non, non parce que même je dirais qu'on arrive à la galette des Rois, que je fais encore des tartes poire-chocolat.

00:16:28
*Speaker 1:* D'accord.

00:16:29
*Speaker 2:* Donc, ils adorent et les poires elles sont--elles sont super délicieuses, donc là encore, ça va.

00:16:34
*Speaker 1:* D'accord. Et sinon, le reste c'est plutôt en conserve?

00:16:37
*Speaker 2:* Ouai, ouai, c'est ça. Bah oui, ça doit être les conserves, [euh]-- S'en allaient, voilà.

00:16:41
*Speaker 1:* Les cocktails. Et vous faites beaucoup des, [euh], des salades avec?

00:16:47
*Speaker 2:* Ouai. Et puis des gâteaux aussi.

00:16:48
*Speaker 1:* Et des gâteaux.

00:16:48
*Speaker 2:* Ouai.

00:16:50
*Speaker 1:* OK. Au moins--enfin vous allez--vous consommez assez, [euh], les fruits sont consommés plutôt crus ou souvent cuisinés? Comment vous--

00:17:02
*Speaker 2:* Oh non, alors, [euh], en ce moment c'est plutôt cru parce que ça va être beaucoup de salades. En hiver ça va être cuisiné.

00:17:06
*Speaker 1:* D'accord.

00:17:07
*Speaker 2:* Et ça, ça va être dans les gâteaux, quoi. Ouai c'est ça. Ça va être un quatre-quart aux pommes, ça va être un gâteau aux abricots, ou sinon mes tartes poire-chocolat. Il ne faut pas que j'en parle trop parce que sinon, je vais ressortir ça.

00:17:18
*Speaker 1:* (Rire).

00:17:18
*Speaker 2:* Et j'aurai pas les poires.

00:17:20
*Speaker 1:* Banane- Banane-chocolat.

00:17:23
*Speaker 2:* Ah! Ouai, ouai, c'est sympa aussi.

00:17:25
*Speaker 1:* D'accord. OK. Donc l'été plus [imperceptible] en salade et l'hiver du coup en gâteau quoi!

00:17:33
*Speaker 2:* Ouai, ouai.

00:17:33
*Speaker 1:* OK.

00:17:33
*Speaker 2:* Et puis finalement, en hiver, ça va être plus les soupes, qu'on va délaissées un peu en été.

00:17:37
*Speaker 1:* Oui.

00:17:39
*Speaker 2:* Ouai, c'est ça.

00:17:41
*Speaker 1:* D'accord. Et est-ce que donc--après-- En terme de légumes vous m'avez déjà dit tout ceux que vous avez l'habitude d'aller-d'aller- de manger. Est-ce qu'il y en a que vous connaissez, que vous allez par contre pas--pas consommer pour diverses raisons?

00:17:53
*Speaker 2:* [euh], ouai. Les patates douces là.

00:17:56
*Speaker 1:* Ouai.

00:17:56
*Speaker 2:* Je sais pas comment celles-ci s'appelle. Ouai, je crois que c'est une patate douce.

00:17:58
*Speaker 2:* Patate douce, ouai.

00:17:59
*Speaker 2:* Ça n'a pas marché chez mes enfants et moi--moi-même non plus. Je n'aime pas trop la saveur, voilà c'est tout, c'est une saveur qui passe pas.

00:18:07
*Speaker 1:* Ouai.

00:18:07
*Speaker 2:* Il y a quelques courges aussi, [euh], mais elles sont très, [euh], très pointues en goût. C'est à dire qu'il y en a une qui avait vraiment un goût de noisette, noix, je ne sais pas ce que--c'était une Blueberry je crois, quelque chose comme ça, et ça n'a pas passé, mais bon, voilà, c'est vraiment spécifique.

00:18:22
*Speaker 1:* OK. Donc les légumes que vous n'avez pas consommés, c'est plutôt parce que ça plait pas forcément, [euh]--

00:18:27
*Speaker 2:* Ouai. Ça va être--- ces deux lá c'est parce que ça plait pas au goût et c'est ça-- c'est dans ma tête. Après, qu'est-ce qui ne pourrait pas passer? Ouai. Ou alors il faudrait m'en citer pour comprendre que--

00:18:37
*Speaker 1:* Que vous les consommer pas?

00:18:39
*Speaker 2:* Ouai.

00:18:39
*Speaker 1:* Dans la même stratégie, par exemple que là, vous avez la-- Il y a des, [euh] fruits que vous allez pas acheter frais parce que vous ne les trouvez pas, [euh], assez, [euh], sensibles, enfin, ce que vous disiez, je crois que--

00:18:51
*Speaker 2:* Ouai. Ah! Oui les abricots, par exemple, qui est dur comme du caillou, là.

00:18:53
*Speaker 1:* Ouai.

00:18:53
*Speaker 2:* Ouai, c'est ça.

00:18:55
*Speaker 1:* C'est-- Et du coup les légumes, non? Vous trouvez toujours votre compte, [euh]

00:18:58
*Speaker 2:* Ouai, je pense ouai. Ouai, ouai. Si, si chez le maraîcher. Bah oui, parce que j'ai aussi, [euh], la facilité d'accès chez elle, parce que c'est en face de chez moi, de l'autre côté de la route là, alors il faut passer la berme et il faut aller de l'autre côté, c'est la facilité, puis, ouai, le fruit, [euh], ouai, je ne vais pas trouver de- de verger, vraiment quoi.

00:19:16
*Speaker 1:* Ouai.

00:19:16
*Speaker 2:* En fait, on n'a pas cherché vraiment, quoi. Ça fait 12 ans qu'on est là, mais [rire] on est débordé. Ça fait beaucoup 12 ans, hein, mais--

00:19:23
*Speaker 1:* Mais du coup voilà, ça va être supermarché, vous avez quand même trouvez vos bananes, vos pommes--

00:19:26
*Speaker 2:* Ouai.

00:19:26
*Speaker 1:* Voire des poires--

00:19:27
*Speaker 2:* Ouai, c'est ça.

00:19:28
*Speaker 1:* Quand c'est la saison, et des fraises au maraîcher. Et sinon le reste c'est plutôt en--

00:19:31
*Speaker 2:* Abricots, salades. Ouai, bah, les cerises aussi en ce moment, chez Casino, quoi. Elles sont pas mal, ouai, bon voilà, c'est pas forcément bio, mais je me dis c'est la saison et les enfants adorent, donc.

00:19:40
*Speaker 1:* OK. Les cerises, les kiwis, par exemple ce qui vous--

00:19:42
*Speaker 2:* J'en ai dans le jardin. J'en ai 10 kilos ou 15 kilos par an. Et là, je crois que--

00:19:45
*Speaker 1:* Ah oui!

00:19:46
*Speaker 2:* On va en avoir encore plus, ouai, c'est ça. C'est au mois--au mois de décembre, ça.

00:19:50
*Speaker 1:* D'accord.

00:19:51
*Speaker 2:* Donc, ça fait décembre/janvier, ouai, où je- je les étalent sur ces deux mois.

00:19:55
*Speaker 1:* OK.

00:19:56
*Speaker 2:* On se fait des smoothies.

00:19:57
*Speaker 1:* Ouai.

00:19:58
*Speaker 2:* C'est bon.

00:20:00
*Speaker 1:* Et donc--

00:20:00
*Speaker 2:* Non, pas forcément. Là aussi, c'est pareil, le kiwi c'est vrai que je vais en acheter pour compléter dans la salade, mais c'est pas du tout la même saveur.

00:20:08
*Speaker 1:* Ouai.

00:20:08
*Speaker 2:* Et là, franchement, [euh], il y a beaucoup d'écart.

00:20:10
*Speaker 1:* Ouai.

00:20:10
*Speaker 2:* Ouai, ouai, c'est ça. Ça déplaît beaucoup aux enfants. Ouai, ouai. Et ceux du commerce, ouf, c'est de l'eau quoi, en fait, c'est de l'acidité pour rien. Il n'y a pas de--ouai.

00:20:24
*Speaker 1:* Ouai. Et du coup, vous avez donc des pommes, des kiwis?

00:20:27
*Speaker 2:* Ouai.

00:20:28
*Speaker 1:* [Euh], la dernière fois vous m'aviez montré des courges?

00:20:30
*Speaker 2:* Oui.

00:20:31
*Speaker 1:* Et qu'est-ce que vous avez d'autre dans votre jardin?

00:20:34
*Speaker 2:* Ah, dans mon jardin, sinon, bah, on fait des courgettes.

00:20:35
*Speaker 1:* D'accord.

00:20:36
*Speaker 2:* Ouai. Courgettes. Et, ben ouai, courges, courgettes, salades aussi, des laitues,

00:20:40
*Speaker 1:* D'accord.

00:20:41
*Speaker 2:* J'en ai déjà 24 qui sont sorties lá et je refais un semi aussi de je sais pas combien. Mais bon, il y a toujours de la perte, mais. Il y a beaucoup de salades ont été, ouai.

00:20:52
*Speaker 1:* D'accord. Alors courgettes, salades,

00:20:54
*Speaker 2:* Ouai, j'ai des grosses courgettes, énormes. Des fois, je les vois pas. Puis c'est après, sous les feuilles «Oh bah mince, alors», il y a des gros records chaque année.

00:21:02
*Speaker 1:* D'où viennent les courgettes que vous avez? C'est les vôtres? Ou-- Ah ouai c'est---

00:21:07
*Speaker 2:* Ouai, ouai. Je fais des semis, ouai.

00:21:08
*Speaker 1:* D'accord.

00:21:09
*Speaker 2:* Bon lá cette anné, [euh], j'avoue que j'ai triché parce qu'on était un peu en retard avec nos contretemps. Donc, là, les laitues j'en ai acheté à Point Vert et puis les courgettes, j'en ai trouvé aussi chez Point Vert. Donc j'en ai- j'en ai pris trois ou quatre.

00:21:21
*Speaker 2:* D'accord.

00:21:21
*Speaker 2:* Pour aller plus vite. Parce que là, je viens de les planter hier les autres, les semées.

00:21:24
*Speaker 1:* D'accord,

00:21:25
*Speaker 2:* Donc, ça va être des courgettes de fin de saison cette année. Je me suis dit: «Ça va être un peu chaud quand même». Ah, ouai, parce que là en grande quantité, c'est- y a beaucoup de gratin, quoi, les courgettes, c'est super bon quoi.

00:21:36
*Speaker 1:* Bah oui.

00:21:39
*Speaker 2:* Ouai. Et puis ratatouille, quoi. Énormément. Là, c'est vrai que ça va avec le barbecue.

00:21:46
*Speaker 1:* Point vert c'est le maraîcher du coup?

00:21:47
*Speaker 2:* Ah, non à Point vert, vous savez c'est les gammes verts et c'est les magasins de bricolage, enfin, de jardinage.

00:21:54
*Speaker 1:* D'accord. Et--

00:21:55
*Speaker 2:* C'est là qu'on a des plans d'un peu de tout quoi, d'aubergines et tout ça, quoi.

00:21:58
*Speaker 1:* Ah, vous avez vos plans?

00:21:59
*Speaker 2:* Ouai. On les achète là-bas, mais en fait, moi, aubergines ici, j'arrête d'en planter parce que ça- ça prend pas. Ça se fait dévorer par--

00:22:05
*Speaker 1:* Ah oui!

00:22:06
*Speaker 2:* Ou alors, sinon, les poivrons aussi, ça donne--on dirait que c'est des piments, mais c'est des poivrons en réalité, c'est tellement petit, donc. On est dans une région un peu, [euh] où il faut avoir une serre je pense.

00:22:15
*Speaker 1:* D'accord!

00:22:15
*Speaker 2:* Les melons c'est pareil. C'est pas la peine, j'en fais plus. Ouai, les haricots verts on se les fait manger et j'ai vu un lièvre, l'autre jour, dans mon jardin, donc.

00:22:23
*Speaker 1:* (Rire).

00:22:23
*Speaker 2:* Je comprends pourquoi. Les petits pois c'est pareil, on est déçu. Il y a beaucoup de tueurs pour rien quoi. Enfin, il faut avoir la serre. Et puis, le temps c'est plus que ça, quoi.

00:22:35
*Speaker 1:* Oui, ça demande peut être plus d'investissement que la courgette qui va très bien poussée d'elle-même. D'accord. Si on s'intéresse à vos lieux de stockage--

00:22:46
*Speaker 2:* Oui.

00:22:46
*Speaker 1:* [Euh], est-ce que vous pouvez me raconter, justement quand vous venez de vos courses, qu'est-ce que vous faite de vos fruits et de vos légumes?

00:22:53
*Speaker 2:* Alors mes légumes, comme c'est-- les patates elles sont en gros--en grosse quantité, c'est au hangar.

00:22:57
*Speaker 1:* D'accord. Oui.

00:22:59
*Speaker 2:* Et là, par dessus, je mets une bâche opaque qui empêche la lumière de pénétrer dans les filets, parce que elles sont dans des filets.

00:23:05
*Speaker 1:* D'accord.

00:23:06
*Speaker 2:* On m'a dit, éventuellement, de mettre de la--de la fougère, j'avais essayé il y a quelques années, ça n'avait pas rajouté grand chose, donc, voilà, pour l'assainissement. Et puis sinon, bah, le reste, les- les courgettes, les poireaux, les poivrons en ce moment, bah, ça va être le bac à légumes, quoi. Si vraiment, je n'utilise pas dans la foulée puisque j'y vais quand même deux fois par semaine. Et je me dis des fois, ça va être cuisiné dans la foulée quoi.

00:23:32
*Speaker 1:* D'accord. Oui, finalement, c'est pas des légumes qui vont être stockés très longtemps.

00:23:35
*Speaker 2:* Non. C'est ça, voilà, c'est ça.

00:23:40
*Speaker 1:* Oui, donc ça va être deux, trois jours max.

00:23:41
*Speaker 2:* Ouai, ouai, c'est vrai.

00:23:45
*Speaker 1:* D'accord. Et les fruits? Vous les entreposés où?

00:23:49
*Speaker 2:* Bah, dans--dans--dans--dans un espèce de saladier en verre, dans ma cuisine.

00:23:51
*Speaker 1:* D'accord. Comme une corbeille, [euh]--

00:23:54
*Speaker 2:* Transparent comme ça au moins, je les vois tous jusque dans le fond. Je me dis: «Ah tiens, c'est quoi ce bout de pomme là, en train de pourrir», c'est ça. Ouai, non, c'est ça.

00:24:07
*Speaker 1:* Oui.

00:24:08
*Speaker 2:* Et puis, bah, tout ce qui est petit truc, les échalotes, les oignons, l'ail, ça va être dans une petite corbeille en bois à côté de mes fruits.

00:24:16
*Speaker 1:* D'accord.

00:24:17
*Speaker 2:* C'est pas très loin de la gazinière comme ça au moins j'ai tout de suite ça sous la main pour éplucher rapidement.

00:24:21
*Speaker 1:* OK.

00:24:23
*Speaker 2:* Donc, c'est à l'air ambiant, ça.

00:24:24
*Speaker 1:* Ouai, avec les fruits tout ça.

00:24:28
*Speaker 2:* Ouai. Et les gros stock d'oignons, d'échalotes, ça va être au hangar ou dans--Ouai c'est dans le hangar.

00:24:34
*Speaker 1:* Oui [imperceptible] très grosse partie, [euh].

00:24:37
*Speaker 2:* Ouai, je prends des petits--des petits seaux quand je ramène dans la cuisine. Ça c'est les petits trucs de [prénom], alors pour une poubelle connectée, je mets celle là. C'est bien pratique les seaux à popcorn.

00:24:48
*Speaker 1:* [Rire]. Et après vous emmener. Vous avez raison pour pas faire des aller-retour.

00:24:56
*Speaker 2:* Oui, mais sinon- sinon ça--

00:25:05
*Speaker 1:* OK. Et donc, selon vous, est-ce que c'est des lieux de stockage qui sont--que vous trouvez adéquates? Vous--comment vous estimez vous conservation? Est-ce que vous trouvez ça plutôt bien?

00:25:13
*Speaker 2:* Ouai, je pense--C'est plutôt bien du fait aussi que, bah, les--les compléments bah, je les fait dans la foulée aussi. Il n'y a pas beaucoup d'attente dans le frigidaire, donc ça c'est--

00:25:22
*Speaker 1:* Ouai. OK.

00:25:27
*Speaker 2:* Puis les fruits, bah, comme j'attends qu'une chose, c'est qu'ils mûrissent, et ben, voilá, vaut mieux--

00:25:32
*Speaker 1:* Ouai.

00:25:32
*Speaker 2:* Même les activés à côté de la gazinière. «Tu mûrira». [Rire]. Je sais pas pourquoi elles sont toujours vertes leurs bananes.

00:25:54
*Speaker 1:* Et en terme de nettoyage, alors, est-ce que vous les nettoyer? Donc, tous les endroits, vous direz-- Comment vous gérez ça? Est-ce que c'est organisé? Est-ce que ce n'est pas organisé?

00:26:06
*Speaker 2:* [Euh], non. Enfin, oui. Quand j'arrive avec- pour mettre mes patates qui sont en grosse quantité, là, on va bien déblayer, [euh], l'em-l'emp- l'emplacement. Et puis oui, je vais rendre propre l'endroit où je vais mettre-- Et que je vais remettre plein de poussière de terre dessus.

00:26:20
*Speaker 1:* Ça va être de la nouvelle poussière.

00:26:21
*Speaker 2:* On fait en sorte que ce soit quand même décapé. Ouai, c'est ça.

00:26:21
*Speaker 1:* Ouai. Et, [euh], la corbeille, vous direz que ça--ça vous arrive? Ou--

00:26:29
*Speaker 2:* Oui. Ah, oui, oui, c'est fréquent. Parce que quand je ne vois plus de fruit, je me dit: «Ah, bah tiens, il y a un truc qui a pourri». Bah, là, je me dis: «Oh! C'est crade, je vais le laver, quoi».

00:26:34
*Speaker 1:* OK.

00:26:38
*Speaker 2:* Ouai, ouai.

00:26:38
*Speaker 1:* Et puis--

00:26:41
*Speaker 2:* Le bac à légumes, [euh], bah, en fait, [euh], le bac à légumes ça va être en même temps que le frigidaire, souvent quoi.

00:26:44
*Speaker 1:* Ouai.

00:26:44
*Speaker 2:* Alors ça va être une fois tous les deux ou trois mois. Enfin, on va dire que je le fais pas toutes les semaines, mais quand je trouve ça sale, quoi, en fait.

00:26:49
*Speaker 1:* Oui, c'est--

00:26:49
*Speaker 2:* C'est pas vraiment une fréquence plus que ça.

00:26:56
*Speaker 1:* Ça marche. Ce qui est-- Alors, sur les préparations, on en a déjà un peu discuté. Vous m'avez dit que les fruits vous les manger et cru en salade et cuit en gâteau.

00:27:09
*Speaker 2:* Oui.

00:27:10
*Speaker 1:* Est-ce que y a des fruits qui vont être consommés par vous, par votre foyer, plutôt comme ça, sans- sans même que vous les ayez mis dans les salades?

00:27:18
*Speaker 2:* Ah, oui, enfin, salades ou épluchés, quoi.

00:27:20
*Speaker 1:* Oui.

00:27:20
*Speaker 2:* Ouai. Non, les pommes je n'aime pas trop les voir manger la pomme comme ça à la bouche. Je suis exigeante sur, [euh], sur la peau de la pomme. Parce que bon, c'est pas forcément bio, celles-là ne sont pas bio en occurrence, et je leur dirais: «Bah, non attends je te l'épluche».

00:27:35
*Speaker 1:* D'accord.

00:27:37
*Speaker 2:* Ouai, ouai. Vraiment la peau, c'est quelque chose pour moi de-- C'est important de l'enlever quoi.

00:27:41
*Speaker 1:* D'accord.

00:27:45
*Speaker 2:* Même celles du jardin. Les courgettes du jardin- Les courgettes du jardin quand je fais-- ouai d'accord, il peut y avoir un peu de peau de la courgette une fois sur deux, mais je suis assez vigilante là-dessus.

00:27:53
*Speaker 1:* D'accord. Donc, même ce qui est de votre jardin vous avez tendance à éplucher?

00:27:58
*Speaker 2:* Ouai. Bah, les Kiwis, on ne va pas manger la peau, c'est sûr, mais la pomme, [euh], ouai, la pomme du jardin, en fait, comme il y a des vers dans les vraies pommes du jardin, là aussi, c'est par rapport au vers que je leur dis: «Bah, non attends-attends je vais peut être découper».

00:28:08
*Speaker 1:* Ouai.

00:28:08
*Speaker 2:* Parce que la surprise après.

00:28:10
*Speaker 1:* D'accord. Et, donc, sur les légumes, si on prend un légume que vous consommez, par exemple, la pomme de terre?

00:28:10
*Speaker 2:* Ouai.

00:28:10
*Speaker 1:* Vous consommez beaucoup?

00:28:10
*Speaker 2:* Ouai, ouai.

00:28:10
*Speaker 1:* Est-ce que vous pouvez me citer un peu les différentes façons que vous avez de- de consommer la pomme de terre?

00:28:28
*Speaker 2:* En plat?

00:28:30
*Speaker 1:* Ouai. Un plat à préparer.

00:28:33
*Speaker 2:* Purée, [euh], comment je veux dire, soupe, purée, pommes de terre sautées.

00:28:37
*Speaker 1:* D'accord.

00:28:38
*Speaker 2:* Et éventuellement frites, mais bon, on préfère la pomme de terre sauter. Qu'est-ce que ça va être d'autre? Ouai, je crois que j'ai fait le tour, en fait, c'est assez basique.

00:28:46
*Speaker 1:* Ouai, d'accord. Et la pomme de terre vous l'épluchée aussi, [euh], vous l'épluchée?

00:28:54
*Speaker 2:* Ouai. Bien, en-- même en-- ouai pas mal, ouai. Je vous l'avais montré l'autre jour. Mais je déplure beaucoup la pomme de terre.

00:28:59
*Speaker 1:* Est-ce que vous pouvez me raconter, là, de nouveau, pourquoi vous avez ce besoin de--

00:29:03
*Speaker 2:* Surtout la pomme de terre, parce que la pomme de terre, je sais que ça fait partie des légumes ultra-traités, même si c'est de l'agriculture raisonnée, parce que c'est ce qui sont en face de chez moi, chez le maraîcher. Bah, je sais que c'est traité un petit peu en dessous dans--dans, [euh] en dessous du colza, je crois.

00:29:19
*Speaker 1:* D'accord.

00:29:19
*Speaker 2:* La pomme de terre c'est ultra traitée, c'est ce que j'avais--j'avais toujours su, donc je me méfie. Puis, en fait, je digère très mal la pomme de terre en finale.

00:29:27
*Speaker 1:* D'accord.

00:29:27
*Speaker 2:* Et du coup, je me dis: «Est-ce qu'elle est si comestible que ça la pomme de terre?». C'est une réflexion qu'on mène depuis très longtemps.

00:29:33
*Speaker 1:* D'accord.

00:29:34
*Speaker 2:* Il y a des années où--Quand je mange moins de pommes de terre, je me sens beaucoup mieux.

00:29:38
*Speaker 1:* D'accord.

00:29:38
*Speaker 2:* Donc, c'est moi qui-- On essaie d'alterner, mais alterner c'est pas évident.

00:29:47
*Speaker 1:* Donc, c'est à cause du-de-du traitement qui est fait sur les pommes de terre que vous voulez les éplucher et vous allez faire, [euh] [diaphonie].

00:29:52
*Speaker 2:* Ouai, ouai, c'est ça. J'ai pas d'économe. J'aurais jamais d'économe, j'épluche tout à mon-- avec mon couteau.

00:29:59
*Speaker 1:* Oui. Parce que l'économe n'épluche pas assez?

00:30:04
*Speaker 2:* Ouai. Puis j'ai toujours eu l'habitude du couteau, en fait, depuis toute jeune, donc c'est un truc que je renouvelle même pas avec le- l'économe. Ouai, et puis j'épluche vite, en fait, c'est aussi la rapidité quoi.

00:30:17
*Speaker 1:* Ouai.

00:30:17
*Speaker 2:* Je rends un peu cubique la pomme de terre tant pis. Mais bon, c'est--ouai, c'est efficace à tous les niveaux, quoi, je pense.

00:30:23
*Speaker 2:* Ouai. Et est-ce que vous avez, en fait, des fruits ou des légumes que vous--que vous allez laisser une peau ou pas du tout? Il n'y en aura aucun que vous allez--

00:30:34
*Speaker 2:* Non.

00:30:34
*Speaker 1:* À chaque fois vous épluchez?

00:30:37
*Speaker 2:* Ouai, ouai, ouai. Ouai, c'est ça. Même les pommes de terre nouvelles jeunes, j'aime- j'aime pas.

00:30:43
*Speaker 1:* Ouai.

00:30:43
*Speaker 2:* Ouai, j'ai toujours la phobie de plein de choses. J'ai vécu un peu dans la paranoïa. C'est ce qu'on se disait souvent, avec mes deux sœurs, parce que mes parents nous-- étaient vigilants aussi sur ce mode de vie. Il faisait un peu cette façon de faire, quoi.

00:30:58
*Speaker 1:* D'accord, donc, dès votre plus jeune âge il y avait cette, [euh], habitude d'éplucher, [euh].

00:31:02
*Speaker 2:* Ouai. Si vous saviez. Ouai c'est ça. Bah, oui, parce qu'on était face à des-- Mon père était-- tenait un laboratoire d'analyses médicales, donc on était, [euh], confronté aux maladies de-d'un peu tout-tout le monde.

00:31:15
*Speaker 1:* Ouai.

00:31:16
*Speaker 2:* Moi, je- le laboratoire, en fait, il y avait le côté bactériologie et là, c'était terrible, donc, voilà. On vit dans les parasites et finalement, à force de ça, on a peur de beaucoup de choses, quoi.

00:31:26
*Speaker 1:* Ouai.

00:31:26
*Speaker 2:* Puis on se pose des questions aussi. Tous ces cancers et ces maladies auto immunes, c'est vrai que-- En plus, c'est ce qui nous arrive encore aujourd'hui, donc.

00:31:39
*Speaker 1:* Ouai. Oui, donc, ça va être, selon vous--enfin, voilà, la façon dont vous allez consommer les fruits et les légumes avec [imperceptible] d'épluchure et tout c'est pour--C'est d'un point de vue de santé que vous--

00:31:48
*Speaker 2:* Ouai. Ouai, c'est ça.

00:31:48
*Speaker 1:* Que vous allez être vigilante sur--

00:31:48
*Speaker 2:* Ouai. Bah sur les peaux, ouai.

00:31:55
*Speaker 1:* D'accord. Et par contre, bah, les fraises, par exemple, vous faites comment?

00:31:58
*Speaker 2:* Ouai, bah, là c'est de l'agriculture raisonnée, donc j'ai dit aux enfants: «Je veux bien vous en passer», mais, voilà, quoi. C'est-- Ils le savent quoi. Ouai, ça c'est évident. Après elle m'a garantie, en face, que elle mettait rien. Je suis septique quand même.

00:32:03
*Speaker 1:* (Rire).

00:32:06
*Speaker 2:* Il y a une mise en confiance aussi.

00:32:14
*Speaker 1:* Oui, c'est sûr. Quand c'est comme ça, est-ce que vous les lavées ou non, vous--

00:32:19
*Speaker 2:* Ah oui, oui, oui.

00:32:20
*Speaker 1:* Si? vous lavez--

00:32:21
*Speaker 2:* Oui, je les lave bien, ouai, ouai, c'est ça. Ouai. ouai. Moi je les fais une para une.

00:32:28
*Speaker 1:* Oui.

00:32:28
*Speaker 2:* Ouai, c'est comme les épinards, c'est pareil. Ça me suffit ça. Je dis à mon mari: «Ah, là aujourd'hui c'est épinards, je peux pas te dire le temps que j'ai mis à les- à les laver»

00:32:35
*Speaker 1:* Ah! Oui?

00:32:36
*Speaker 2:* Ouai. Puis les salades aussi, en fait. Les salades parce que c'est quelque chose de cru.

00:32:39
*Speaker 1:* Ouai.

00:32:39
*Speaker 2:* Et puis, je suis vigilante sur toute la pari- parasitologie. Je fais attention. Le cresson, je n'en achète jamais. Je sais que c'est- C'est par ces petites feuilles qu'on attrape le plus de saloperies.

00:32:49
*Speaker 1:* Ouai. D'accord, alors quand c'est des choses qui vont être mangés crues--

00:32:54
*Speaker 2:* Ouai.

00:32:54
*Speaker 1:* Que vous ne pouvez pas éplucher! Bref, y a pas de--

00:32:59
*Speaker 2:* Ouai, bah, là, je les lave très--en détail, quoi.

00:32:59
*Speaker 1:* D'accord.

00:32:59
*Speaker 2:* C'est un par un. Ouai, c'est fastidieux, mais bon, c'est un régal, quoi.

00:33:09
*Speaker 1:* OK. Alors, j'aimerai là qu'on discute sur le moment ou vous jettez un fruit ou un légume.

00:33:15
*Speaker 1:* Ouai.

00:33:15
*Speaker 1:* Ou une partie seulement. Donc, quand ça arrive, pourquoi décidez-vous de le jeter plutôt que de le consommer, ce fruit ou ce légume?

00:33:22
*Speaker 2:* Ah, oui, d'accord, [euh]. À quel moment? Parce qu'il est dégradé, en fait, il va être complètement noir. Ça va être ça.

00:33:27
*Speaker 1:* Ouai.

00:33:28
*Speaker 2:* Ou alors, sinon, ça m'est arrivé, j'y a pensé dernièrement à une pomme de terre qui était tellement, [euh], dans tous les sens--

00:33:34
*Speaker 1:* Ouai.

00:33:34
*Speaker 2:* Que je me suis dit: «Mais comment veux tu que j'épluche ça. Aller bon, je la balance quoi». C'est pas toujours, mais, dans tout- dans les- le tas de pomme de terre que j'ai eu, [euh], j'en ai pas- j'en ai pas eu plus de 10 quoi, on va dire.

00:33:45
*Speaker 1:* Ouai. D'accord.

00:33:51
*Speaker 2:* Ouai, c'est la forme aussi.

00:33:54
*Speaker 1:* Ok. Oui, qui vous empêche vraiment de--

00:33:56
*Speaker 2:* De l'éplucher, ouai, ouai. c'est ça. D'aller vite et d'éplucher, quoi.

00:34:04
*Speaker 1:* D'accord. Et comment vous déterminez, du coup que, un fruit ou un légume est dégradé comme vous dites? Qu'est-ce qui vous fait penser à ce moment là que-qu'il n'est plus consommable?

00:34:15
*Speaker 2:* Ben, c'est- c'est son aspect, en fait , c'est un aspect tout noir. Et puis, [euh], ouai, ben.

00:34:26
*Speaker 1:* D'accord, et quand vous êtes face, [euh], à cet aspect noir et comment vous--comment vous sentez face--si vous voilà-- si vous êtes amené à toucher sur le-le--

00:34:37
*Speaker 2:* Bah, la banane,[euh], bah, je la prends, j'enlève le petit truc noir si c'est qu'un- une petite zone, vous voyez?

00:34:42
*Speaker 1:* Ouai. D'accord.

00:34:42
*Speaker 2:* Après, [euh], ouai, ça dépend. Après si tout est vraiment--si je prends la- la pomme des fois, elle font-- il y en a qui font «pchit».

00:34:46
*Speaker 1:* Ouai.

00:34:51
*Speaker 2:* Donc on se dit: «Oh, il y a plein de jus là-dedans. Bah, c'est bon». Je-- Parce que je pense qu'en y mettant le couteau j'avancerai pas grand chose.

00:34:56
*Speaker 2:* Oui. Donc, pour reprendre, quand c'est des petits bouts abîmés, vous allez les enlever.

00:35:02
*Speaker 2:* Ouai, voilà c'est ça.

00:35:02
*Speaker 1:* Et si vous voyez que--- que---que ce--

00:35:06
*Speaker 2:* Que c'est la pomme entière, ouai. Ou alors, sinon, si je vais avoir que ça à extraire, je me dis je- j'ai à peine le temps d'extraire ça. Donc ça va être toute la pomme, ça dépend la proportion que ça représente, mais, [euh].

00:35:15
*Speaker 1:* Un petit bout, on enlève, mais si c'est un-- si ça commence à être un gros--

00:35:21
*Speaker 2:* Ouai, c'est ça. On va dire si c'est trois-quarts d'une pomme qui est déjà fichue [euh], je crois que, [euh]-- Ça dépend, hein, si je suis tentée par la grosse pomme qui- qui peut être encore exploitable, mais ouai, c'est ça.

00:35:29
*Speaker 1:* Vous n'allez pas sauver le dernier quart.

00:35:32
*Speaker 2:* Ouai, pas forcément. Et pouvant savoir que ça fait de la perte.

00:35:48
*Speaker 1:* (diphonie)

00:35:48
*Speaker 1:* Donc, OK. Est-ce que dans votre- vous m'avez dit déjà que, en général tout le monde aime les choses bien mûres!

00:35:53
*Speaker 2:* Ouai. Ouai, ouai.

00:35:54
*Speaker 1:* Et face au-- Est-ce qu'il y en a-- Est-ce que vous avez des différences dans votre foyer? Est-ce qu'il y en a qui vont aimer les choses crues? Les choses vraiment très mûres? Est-ce que vous voyez des différences ou c'est plutôt--

00:36:05
*Speaker 2:* Oh, non. C'est tout le monde.

00:36:05
*Speaker 1:* Tout le monde pareil?

00:36:06
*Speaker 2:* Ouai, je pense, ouai. Ou alors, c'est parce que moi, je- je fonctionne comme ça, que les enfants fonctionnent comme ça, mais non. Ils me le diraient sinon. Bon c'est vrai que la banane verte, je vois bien que ça--que ça plait pas plus que ça.

00:36:16
*Speaker 1:* Ouai. Et face au--Par exemple s'il a des bouts abîmés, est-ce que, [euh], il y a des différences, selon vous, face aux-- à ces--à ces morceaux qu'on peut qualifier un peu de--vous m'avez dit dégradé, noir. Est-se qu'il y a eu des gens qui vont tolérer un petit bout ou--

00:36:34
*Speaker 2:* Ah, bah, [euh], ouai, j'ai des enfants qui vont manger, [euh], pourri, sans faire attention.

00:36:37
*Speaker 1:* D'accord.

00:36:38
*Speaker 1:* Parce que ça [imperceptible]. Bah, voilà, c'est même à l'envers de ce qu'on pourrait penser.

00:36:43
*Speaker 1:* (Rire).

00:36:47
*Speaker 2:* Bon, si ça marche, tant mieux.

00:36:47
*Speaker 1:* D'accord. On dirai qu'ils vont--

00:36:52
*Speaker 2:* C'est sûr qu'ils vont pas faire l'inverse, c'est à dire que si y a un petit bout pourri, ils vont pas balancer et c'est déjà ça, quoi.

00:37:00
*Speaker 1:* Ouai?

00:37:00
*Speaker 2:* Ouai. Bon, après, il faut les freiner, quoi.

00:37:04
*Speaker 1:* [rire].

00:37:05
*Speaker 2:* Les jeunes--les jeunes de maternelle apprennent, ils savent quand même.

00:37:06
*Speaker 1:* Ouai. OK. Alors, pour finir, j'aimerai qu'on discute du gaspillage alimentaire.

00:37:16
*Speaker 2:* Oui.

00:37:16
*Speaker 1:* Donc, [euh], vous disie- Quand je vous dis gaspillage alimentaire, c'est quoi les mots qui vous viennent spontanément à l'esprit?

00:37:22
*Speaker 2:* C'est trop de quantité achetée sur un moment présent, enfin, à un moment donné, on va dire et après, donc, s'est ne pas gérer son stock, son--sa consommation, en fait, le gaspillage.

00:37:33
*Speaker 1:* Ouai.

00:37:33
*Speaker 2:* S'est ne pas gérer sa consommation et ne pas, [euh], se dire que, bah voilà, si j'en achète un kilo de plus, ouai, pourquoi pas, mais se dire: «Bah, mince, finalement, ouais, je--ma quantité, elle est là, elle n'est pas au-delà», quoi.

00:37:45
*Speaker 1:* Et pour vous c'est lié à la--une mauvaise gestion?

00:37:50
*Speaker 2:* Ouai, ouai, c'est ça voilà, ouai. Un mauvais calcul, en fait. Bon, après, il n'y a pas que ça, bien sûr. Comme en ce moment, on a un enfant hospitalisé, donc il va y avoir des contextes à part où forcément, [euh], quelque chose qui désorganise la--la famille, qui va faire que--ouai, c'est-- Le gaspillage et--et là ça peut plus s'expliquer de cette façon, quoi.

00:38:12
*Speaker 1:* Du coup quand ce que vous dites, c'est forcément lié, à un moment donné, à un problème d'organisation.

00:38:17
*Speaker 2:* Ouai.

00:38:17
*Speaker 1:* Et des fois, cette organisation elle dépend de faits extérieurs qui--

00:38:21
*Speaker 2:* Ouai c'est ça. Ouai, ouai.

00:38:22
*Speaker 1:* Sont indépendants de la personne qui organise, [euh].

00:38:26
*Speaker 2:* Ouai.

00:38:27
*Speaker 1:* D'accord. Et, sinon, au niveau du coût des courses, vous c'est--c'est vous qui gerez toujours--

00:38:31
*Speaker 2:* Oui.

00:38:31
*Speaker 1:* Ou ça fait partie de votre foyer?

00:38:33
*Speaker 2:* Non.

00:38:33
*Speaker 1:* Non, c'est toujours vous qui-- [rire].

00:38:36
*Speaker 2:* C'est mon rôle, je suis payée pour ça.

00:38:43
*Speaker 1:* [euh], OK. Donc, là l'idée c'était de définir. Vous m'avez déjà donné la définition. Quand vous vous êtes amenée à jeter un fruit ou un légume, qu'est-ce que vous ressentez à ce moment là? Est-ce que--

00:38:53
*Speaker 2:* Bah, je me dis: «Ouai, mince, si j'avais su j'aurais mieux sélectionner mon--mes fruits», quoi.

00:39:00
*Speaker 1:* D'accord.

00:39:00
*Speaker 2:* Il y a ça aussi. Parce que ce n'est pas le fait d'avoir mal vieilli, parce qu'ils vieillissent tous pareils, je me dis: «Moi, j'aurais mieux fait de regarder si toutes les bananes n'étaient pas abîmées», parce que souvent ça arrive aussi. Donc, je suis assez exigeante dans le sens où aux bananes je passe du temps à les sélectionnées.

00:39:13
*Speaker 1:* Ouai. Parce que des fois ça vous arrive de--de--

00:39:14
*Speaker 2:* Bah, c'est mon mari des fois. Parce que le week end, je lui dis: «Là franchement, j'étais à la bourre», c'est exceptionnel et lui va prendre le premier truc de bananes et je dis: «Bah, regarde il y a déjà la moitié qui est esquintée là».

00:39:27
*Speaker 1:* Ouai.

00:39:28
*Speaker 2:* Et ça, je sais que ça va mal vieillir.

00:39:29
*Speaker 1:* D'accord.

00:39:31
*Speaker 2:* Déjà c'est pas beau à la- à la base et après ça pourrit très rapidement.

00:39:36
*Speaker 1:* D'accord. Mais bon, finalement, c'est pas forcément dans le stockage qui va être long, ça va être, des fois, sur le choix, [euh], d'achat dès le départ.

00:39:44
*Speaker 2:* Ouai, exact. Ouai. Le stockage, oui, en fait, c'est là. C'est vrai qu'on s'est dit: «Ma cuisine on l'a pas encore étudiée dans sa fonctionnalité».

00:39:53
*Speaker 1:* Ouai.

00:39:53
*Speaker 2:* Depuis qu'on est arrivé, on a posé nos meubles et on a fait beaucoup de choses, mais-- sauf la cuisine.

00:39:57
*Speaker 1:* D'accord.

00:39:57
*Speaker 2:* Donc c'est vrai que dans notre réflexion on se dit: « Bon, on va y re-on va y revenir un jour».

00:40:03
*Speaker 1:* D'accord. Vous aimeriez qu'elle soit différente?

00:40:05
*Speaker 2:* Ouai. Mieux organisée, [euh], ouai dans le stockage. Même là, la moitié de ce meuble on- on l'utilise juste pour--pour rien du tout, quoi. [rire]. Ouai, mais c'est ça, le stockage

00:40:16
*Speaker 1:* Est-ce que vous connaissez, justement, des stratégies de stockage, [euh], que là par exemple, vous ne pouvez pas forcément faire, vous faites déjà sur les pommes de terre qui ont été à l'abri de la lumière dans des filets?

00:40:25
*Speaker 2:* Non, justement, pas tant que ça. Parce que pour les pommes d'hiver, on nous avait dit: «Il faut les étaler sur un filet pour que l'air passe». Donc, on avait fait ça, mais on n'avait pas trouvé que ça- ça se conservait mieux. Bon, ceci dit, on les entasse pas trop non plus. On a de la capacité de stockage au hangar, on les étale facilement dans la pièce le temps que je les consomme, mais consomme, [euh], rapidement.

00:40:47
*Speaker 1:* Ouai ça. OK. Ça marche. [euh], alors, pour terminer juste sur la poubelle connectée, [euh]. Est-ce que vous avez le sentiment de l'utiliser fréquemment ou au contraire--

00:41:01
*Speaker 2:* Un peu par un coup parce que finalement, je mets dans mon seau et des fois je me dis: «Oh! J'ai pas eu le temps, j'ai pas eu le temps», puis finalement,

00:41:06
*Speaker 1:* D'accord.

00:41:10
*Speaker 2:* Du coup, ouai, ça va être par à-coup, mais j'y pense. Alors, j'y pense. J'aurais collectionner mon truc comme vous aviez dit -- bon bah, le hangar c'était pas top, mais --

00:41:18
*Speaker 1:* Après, si vous y pensez, c'est l'essentiel, c'est pas-- Du courrier?

00:41:31
*Speaker 2:* Ouai c'est ça, du courrier à domicile, c'est sympa. Par une copine.

00:41:37
*Speaker 1:* Alors du coup, [euh], oui, est-ce que ça vous arrivez peut être de l'oublier ou non vous avez quand même, plus ou moins, toujours penser à--

00:41:44
*Speaker 2:* Non. Non, j'ai respecté parce que bon, en plus, ouai, voilà, ça nous amuse.

00:41:52
*Speaker 1:* Ouai. Parce que y a d'autres personnes--des foyers qui on oublié des fois--

00:41:53
*Speaker 2:* Ah oui? Ah non, pas nous. Parce que nous, c'est assez amusant, enfin, tout le monde s'y retrouve, se plaisent à ce truc, donc je dis à mon mari: «Ah! ça c'est pour la pou- poubelle connectée». Franchement c'est dans un tel bazar que--

00:42:09
*Speaker 1:* Faut qu'il choisisse. [euh], alors, est-ce que vous avez des exemples de ce que vous avez jeté? Est-ce que vous vous souvenez de quelques petites choses, de la pomme de terre,

00:42:19
*Speaker 2:* Le noir- le noir de--de banane au début, [euh], des--des pommes qui avait été entamées par les enfants et pas fini, retrouvées une semaine après.

00:42:27
*Speaker 1:* D'accord.

00:42:28
*Speaker 2:* Et après des--bah, dernièrement là, j'ai eu des pommes de terre que javais épluchées en grosse quantité parce que j'avais anticipé de pas être là un soir, que mon mari est arrivé derrière et il a fait autre chose.

00:42:39
*Speaker 1:* D'accord.

00:42:39
*Speaker 2:* Du coup, elles sont restées deux jours sans être utilisées, donc ce midi je les aie détouré.

00:42:43
*Speaker 2:* Ouai.

00:42:43
*Speaker 2:* Donc là, j'ai jeté à la poubelle ce qui avait été détouré, quoi.

00:42:46
*Speaker 1:* D'accord.

00:42:47
*Speaker 2:* Ouai, mais mine de rien, ça nous arrive quand même--

00:42:49
*Speaker 1:* Oui.

00:42:50
*Speaker 2:* D'avoir ce côté là, [euh]-- J'épluche plus de carottes qu'il faut parce que je me dis: «Tiens, je vais en faire après un petit truc avec la dinde», donc je fais des carottes râpées en un premier lieu.

00:42:58
*Speaker 1:* D'accord.

00:42:58
*Speaker 2:* Et puis après j'oublie celles des--quelques-unes que j'ai épluchées en plus quoi.

00:43:02
*Speaker 1:* Oui.

00:43:02
*Speaker 2:* Mais, enfin, bon.

00:43:03
*Speaker 1:* OK.

00:43:09
*Speaker 2:* Ouai. Quest-ce qu'il y avait d'autre? Il y avait un oignon qui avait germé aussi, je vous l'avais montré l'autre jour, je l'ai mis dans la poubelle connectée. Je me suis dis : «Avant de le planter, je le met là-dedans», et je ne l'ai pas encore planté, (rire). Il attend toujours là, mais je l'ai plan- je l'ai rejeté ce matin je l'avais mis hier soir.

00:43:24
*Speaker 1:* D'accord. OK. [euh], quoi d'autre? Est-ce que vous vous êtes posée des questions, des fois, du style: «Est-ce que ça doit aller dedans ou non?

00:43:38
*Speaker 2:* Non.

00:43:39
*Speaker 1:* Non, ça a été?

00:43:39
*Speaker 2:* Ouai, ça été.

00:43:42
*Speaker 1:* OK.

00:43:42
*Speaker 2:* Ben, ouai.

00:43:43
*Speaker 1:* Et est-ce que vous savez si la poubelle fonctionne avec les voyants? Est-ce que la Wi-Fi-- Je suppose que la Wi-Fi fonctionne.

00:43:52
*Speaker 2:* Ah ouai. Voilà, ça s'allumait là-bas, je me suis dit c'est en cours de pesée. Mais alors aujourd'hui et ce matin, juste ce matin, je me suis posée la question parce que j'avais vidé en me disant: «Bon bah, ça va être fini» et puis, en fait, après, je me suis dit: «Ben non, ce n'est pas fini puisque j'ai encore mes épluchures ici de--de pommes de terre détourer».

00:44:07
*Speaker 1:* Ouai.

00:44:07
*Speaker 2:* Donc je l'ai vidé et j'ai mis mes pommes de terre détourer. Je me suis dit: «Mais si ça se trouve la pesée a été déjà--», enfin, vous voyez, c'est pas--ça c'est pas, [euh], rénitialisé, parce que j'avais vidé.

00:44:17
*Speaker 1:* D'accord. Normalement, si vous videz et que vous refermer--

00:44:20
*Speaker 2:* Ouai c'est ce que j'ai dû faire.

00:44:20
*Speaker 1:* Il va comprendre que c'est vide.

00:44:22
*Speaker 2:* Ah oui. Mais ça a été rapide après le-- J'ai pas attendu un quart d'heure pour remettre mes--

00:44:27
*Speaker 1:* Normalement si--

00:44:27
*Speaker 2:* Ah ouai, d'accord.

00:44:28
*Speaker 1:* Si le voyant--

00:44:28
*Speaker 2:* Oui, oui.

00:44:29
*Speaker 1:* C'est éteint, c'est bon.

00:44:31
*Speaker 2:* Ah, ouai. Non, parce qu'il y a un temps où il s'éteint quand même.

00:44:33
*Speaker 2:* Ouai. Je vais--

00:44:33
*Speaker 2:* J'avais remarqué qu'il y avait bien--un quart d'heure, je pense pour-- pour se re-éteindre. Bah vous verifierez--

00:44:37
*Speaker 1:* Et bien on va regarder si-- peut être que- peut être que de là-bas il capte pas la Wi-Fi . Ce qui est pas grave du coup parce que les données sont dans la carte SD.

00:44:44
*Speaker 2:* D'accord.

00:44:45
*Speaker 1:* Mais c'est à dire que c'est peut être pour ça que c'est long, en fait. Vue que la poubelle--

00:44:48
*Speaker 2:* Ah! C'est ça. Parce que l'allais le ramenée par là pour le jetter--Et c'est ça. Et puis j'allais pas remmené ça là-bas pour la mettre dedans, oh, oui.

00:44:53
*Speaker 1:* Mais si s'est jetté, ça va être compté dans--dans la carte.

00:44:57
*Speaker 2:* Mm-hmm. Ouai, ouai.

00:44:57
*Speaker 1:* Dès que vous mettez quelque chose, ça va être pesé au fur et à mesure.

00:45:01
*Speaker 2:* Ah, ouai, d'accord.

00:45:01
*Speaker 1:* Mais j'ai regardé là-bas, tout à l'heure si--voir si c'est le Wi-Fi--

00:45:04
*Speaker 2:* D'accord.

00:45:04
*Speaker 1:* Qui fait une faute, mais c'est pas grave, si le Wi-Fi-- Et, alors, au niveau des sacs poubelle, est-ce que vous- vous en avez utilisé? Est-ce que vous vous souvenez? Ou--

00:45:16
*Speaker 2:* Bah, j'en ai utilisé un, le premier que vous avez mis en place et après, je me suis dit: «Non, le plus simple ça va être de les mettre direct dedans et de l'enlever après le lendemain pour quelque chose».

00:45:26
*Speaker 1:* D'accord.

00:45:26
*Speaker 2:* C'est ce que j'ai fait dernièrement.

00:45:27
*Speaker 1:* D'accord.

00:45:28
*Speaker 2:* Ouai.

00:45:31
*Speaker 1:* Donc là, il n'y a eu qu'un sac, [euh].

00:45:32
*Speaker 2:* Ouai. Voilà.

00:45:32
*Speaker 1:* Ok.

00:45:32
*Speaker 2:* Mais, en fait, le problème du sac, c'est que vite, [euh], j'ai trouvé que ça s'entassait trop, enfin, qu'il y avait trop- Après, il y a eu des petits vers, si vous voulez. Je me suis dis : «Ah, ben, non. J'ai pas envie d'avoir une poubelle comme ça où ça va monter-grimper aux rideaux les petits vers, là». [Prénom du conjoint], mon mari, il me disait: «Mais ça vient d'où?"«» Et puis-- Comme j'avais mis des bananes, au début, je- j'ai identifié que ça venait des bananes.

00:45:52
*Speaker 1:* Ouai. Je pense, ça va vite quand même.

00:45:57
*Speaker 2:* Bah, oui, c'est ça.

00:45:57
*Speaker 1:* OK. Ça marche et bien je vous ai posé toutes mes questions.

00:46:00
*Speaker 2:* Ah ouai?

00:46:00
*Speaker 1:* Merci beaucoup d'avoir pris le temps d'y répondre.

00:46:01
*Speaker 2:* Ouai, c'est ça.

00:46:04
*Speaker 1:* (euh), est-ce que, [euh], il y a eu des choses que vous pensez que j'ai pu oublier ?Des choses, [euh] sur lesquelles vous voudrez revenir? Sur la consommation de fruits et légumes?

00:46:12
*Speaker 2:* Non. Non, c'est vrai que les pommes de terre je les épluche en gros comme je--

00:46:18
*Speaker 1:* Ouai.

00:46:18
*Speaker 2:* C'est la seule particularité.

00:46:19
*Speaker 1:* Oui, si vous avez un truc a-a--si vous- ce que vous pensez, admettons «gaspiller», le plus ce serait quoi? Si, [euh] -- Est-ce que vous avez l'impression qu'il y a des choses que vous allez jeter plus que d'autres ou--

00:46:31
*Speaker 2:* Peut-être, [euh], le- la- le légume que, [euh], je- j'épluche, [euh], le moins délicatement ça va être la pomme de terre, très clairement.

00:46:37
*Speaker 1:* Ouai. Et donc là, vous savez qu'il y a toujours un peu de--

00:46:37
*Speaker 2:* Ouai ,ouai.

00:46:40
*Speaker 1:* Et sinon au terme des bananes, tout ça, vous en perdez pas tant que ça?

00:46:44
*Speaker 2:* Non. Non, pas tant que ça, non plus.

00:46:48
*Speaker 1:* Ouai.

00:46:48
*Speaker 2:* Mm-hmm

00:46:48
*Speaker 1:* D'accord. C'est l'épluchure, [euh]-

00:46:49
*Speaker 2:* Ouai, c'est ça. Ouai,

00:46:51
*Speaker 1:* Ou de temps en temps le noir de banane qui commence à se--

00:46:53
*Speaker 2:* Ouai, ouai, c'est ça.

00:46:55
*Speaker 1:* OK. Bon, bah, très bien. Merci.

00:46:57
*Speaker 2:* Mais de rien.

**Household F31, Interview 2**

*Mme. 2:* Mm.

*Mme. 1:* Je crois qu'il n'a pas mesuré, [euh], le seul lot quasiment le gros lot que j'ai mis de pommes du jardin dedans parce que, [euh], le truc, il déborde.

*Mme. 2:* [Ah], [ah], [ah].

*M. [Prénom du conjoint]:* Ça veut dire qu'on balance et on gaspille.

*Mme. 2:* Je ne sais pas, [en fait], il n'a pas resté de- de resto de date rouge.

*Mme. 1:* Ouais.

*Mme. 2:* Parce que ça débordait un peu et le couvercle n'était pas fermé.

*M. [Prénom du conjoint]:* [Ah] Oui, du coup, oui, c'est-à-dire qu'il--.

*Mme. 2:* Il faut qu'on soit ferme, [quoi].

*Mme. 1:* Ouais, il pensait que ce n'était pas--.

*Mme. 2:* Tiens, [Prénom du conjoint], pendant ce temps-là, tu me-, [euh], tu feras, [euh], la pesée avec les pommes un petit peu, [euh], séquencée, il faudrait les--.

*M. [Prénom du conjoint]:* Tout le filet-là ? Tu veux que--.

*Mme. 1:* Ouais, ouais parce qu'elles sont trop petites. Moi, honnêtement, [ah], t'as pas vu ce qu'on a eu en pomme cette année ?

*M. [Prénom du conjoint]:* Mm-hmm.

*Mme. 2:* Alors, va.

*Mme. 1:* C'est les pommes du jardin ?

*M. [Prénom du conjoint]:* Ouais.

*Mme. 2:* Ouais.

*Mme. 1:* Ouais, il y en a plein.

*Mme. 2:* Il faut pour les pâtisseries.

*Mme. 1:* Mais j'ai été tellement dépassé--.

*Mme. 1:* Si vous les perdez-, [euh], [en fait], si vous les avez stockées chez vous, oui.

*Mme. 1:* Ouais.

*Mme. 1:* Oui.

*Mme. 2:* Si par contre, elles sont tombées de l'arbre, elles étaient déjà abîmées, non. Enfin, [en fait], on fait, on- on part de ce principe-là.

*Mme. 1:* Ouais, ouais, ouais, je comprends, je comprends, ouais, ouais, c'est ça.

*Mme. 2:* Si elles étaient bonnes à la cueillette--.

*Mme. 2:* Ouais, ouais, c'est ça.

*Mme. 2:* Et vous les avez perdues--.

*Mme. 2:* Ouais.

*Mme. 2:* Avec le temps.

*Mme. 1:* Maintenant on va faire- on a fait une grosse cueillette.

*Mme. 2:* Ouais.

*Mme. 1:* Mais moi, après ce que je fais, je le divise en- en trois.

*Mme. 2:* Ouais.

*Mme. 1:* Les grosses, les moyennes et les petites, et ça, c'est les petites.

*Mme. 2:* D'accord.

*Mme. 1:* Je n'y touche pas, c'est que je n'ai vraiment pas le temps.

*Mme. 1:* Ouais.

*Mme. 1:* Déjà, il faut gérer les grosses et moyennes.

*Mme. 2:* Ouais, ouais, [eh], [ben], oui, oui, après, [euh], ça--.

*Mme. 1:* Je ne sais- je ne sais pas combien on a de pommiers, nous, [euh], pas loin d'une- de bonne dizaine, donc, [euh]--

*Mme. 2:* Ouais, ça va vite une fois que c'est- ouais.

*Mme. 1:* Ouais et puis là, c'est-, [euh], c'est- c'est pas des pommes qui se conservent tellement, j'ai l'impression.

*Mme. 2:* Ouais.

*Mme. 1:* Alors, [euh], il faut aller vite, [quoi].

*Mme. 2:* Oui, c'est le jardin et ce n'est pas--.

*Mme. 1:* Alors, c'est un peu dommage de laisser pourrir les plus grosses qui sont les plus faciles à les éplucher, [hein].

*Mme. 1:* C'est vrai.

*Mme. 2:* Alors qu'est-ce que, [euh]--

*Mme. 2:* Alors, ouais.

*Mme. 2:* En plus, il y a des petits--.

*Mme. 2:* C'est un petit jeu.

*Mme. 1:* [Ah], [waouh) J'adore.

*Mme. 1:* Il y a quatre photos et je vais vous demander de retourner, [euh], celle que vous voulez.

*Mme. 2:* Ouais.

*Mme. 1:* Et de me dire spontanément ce qui vous vient à l'esprit.

*Mme. 1:* [Ah] Là, là, là, les trucs dont j'ai horreur, il faut se concentrer.

*M. [Prénom du conjoint]:* Mais pourquoi ? AAA

*M. [Prénom du conjoint]:* Ça me rappelle les-, les-, les entretiens des, des-, des concours de recrutement-là, [euh].

*Mme. 2:* [Ah] Ouais, c'est- [eh], [ben], oui, [en fait], on-, c'est- c'est-, [euh], les photos, elles servent à-, au moi, [en fait], on sait que les personnes, elles- elles visualisent la même chose.

*M. [Prénom du conjoint]:* Mm-hmm.

*Mme. 2:* Pour après développer ce qu'elles pensent, mais-, mais c'est-, [euh], c'est plus un support histoire de que--.

*Mme. 1:* AAA, d'accord ?

*Mme. 1:* Oui, allez-y, Patrice.

*Mme. 2:* Je vais prendre la première qui se présente, marché.

*Mme. 2:* Ouais.

*Mme. 1:* Ce n'est pas forcément le chou vert que j'ai acheté dernièrement, mais--.

*M. [Prénom du conjoint]:* Vous pouvez rester sur celle-ci pour le moment.

*Mme. 2:* [Ah] Oui, d'accord.

*Mme. 2:* Ouais et [euh], me dire ce que voilà, qu'est-ce que ça vous évoque le marché ?

*Mme. 1:* Le marché, [euh], c'est les légumes surtout parce que, [euh], les fruits, je n'en achète pas sur le marché.

*Mme. 2:* D'accord.

*Mme. 1:* Voilà, légumes, poissons.

*Mme. 2:* OK.

*Mme. 2:* Mm-hmm.

*Mme. 1:* Et galette, à la fois, ça ne rentre pas dans les--.

*Mme. 1:* Ouais

*Mme. 2:* Dans votre domaine. Voilà, c'est ça alors c'est tout, [quoi). Après ce que je peux dire ?

*Mme. 1:* Oui, globalement, vous pensez quoi, [euh], du marché ? C'est un lieu- qu'est-ce que vous pensez de ce lieu ?

*Mme. 2:* [Eh] [Ben] Local, [en fait], je pense que j'ai- j'ai des producteurs du coin parce que je la connais. Je vais souvent des fois aussi chez elle directement, c'est en face de, de notre auberge-là.

*Mme. 1:* Oui.

*Mme. 2:* Et, [euh], je la connais, donc, [euh], je- je la- je la retrouve le mercredi au marché et sinon, c'est le vendredi soir chez elle, donc des fois, je fais des compléments. Parce que des fois, je n'ai pas toujours le temps. Le marché, c'est aussi des petits vieux qui parlent.

*Mme. 1:* Oui.

*Mme. 2:* Et, [euh], il faut avoir de la patience ou alors il faut se lever très tôt, mais des fois je le fais, je peux kiffer.

*Mme. 1:* Ouais.

*Mme. 2:* Non, pour trouver des bons- des bons produits, c'est- c'est plaisant, [quoi].

*M. [Prénom du conjoint]:* Ouais.

*Mme. 1:* Ouais.

*Mme. 2:* Une fois- le- le jeu en vaut la chandelle, [quoi].

*Mme. 1:* Ouais.

*Mme. 1:* Mm, moi, c'est un truc, [euh], que je fais souvent, [quoi].

*Mme. 2:* Vous allez du coup tous- tous les combien à peu près au marché ?

*Mme. 1:* [Euh], on va dire, [euh], deux fois minimum par m- par mois par mois.

*Mme. 2:* OK.

*Mme. 1:* Ouais, si- si j'avais un- une-, [euh], une moyenne à tirer sur l'année, [quoi].

*Mme. 1:* Ouais.

*Mme. 1:* Parce qu'il y a des moments où je vais y aller vraiment tous les mercredis- mercredis et puis après il y a des périodes où--.

*Mme. 1:* Mm-hmm.

*Mme. 2:* C'est- c'est des intersaisons, on est plus motivé alors bon.

*Mme. 1:* Oui. Du coup, qu'est-ce qui fait que, [euh], vous- vous aimez aller sur le marché ?

*Mme. 2:* La qualité des produits.

*Mme. 1:* Ouais.

*Mme. 1:* Moi, la saveur aussi, [eh], [ben], voilà, c'est- c'est- ça revient à la qualité, [hein], je pense.

*Mme. 1:* Ouais.

*Mme. 2:* Voilà, la qualité, c'est pour prendre en- globalement, [euh], tout ce qui ressort de- du goûter, du- de la conservation aussi. Plus ou moins parce que bon, en général, c'est parce que moi, comme je le fais tous les mercredis, vous voyez, ça- c'est- j'ai le temps de consommer, [quoi], enfin--.

*Mme. 1:* Ouais.

*Mme. 2:* Ça ne va pas être consommé si vous voulez pour-, je ne vais pas au marché pour- [euh) pour trois ou quatre mois, [quoi].

*Mme. 1:* Ouais.

*Mme. 1:* C'est souvent pour consommer dans la- dans la foulée, [quoi].

*M. [Prénom du conjoint]:* [Euh], et puis on n'a pas de congélateur.

*Mme. 1:* [Ah], oui, c'est vrai, ouais, c'est vrai. Je ne sais pas si je vous l'ai précisé par le passé, [hein],

*Mme. 2:* Vous n'avez pas de--.

*Mme. 2:* Non, on n'a pas de congélateur.

*Mme. 2:* [Ah], oui, du coup, vous avez- oui.

*Mme. 1:* [Ah], [ah].

*M. [Prénom du conjoint]:* Du coup, on est obligé d'aller au marché, [hein].

*Mme. 1:* Du coup, ouais.

*Mme. 2:* [Eh] [ben] Ouais, c'est ça. Je- je fais au--. Ouais, j'ai en--. [Silence 00:04:27- 00:04:44] [Euh] Le marché, j'évite parce que quand il pleut, [eh], [ben], je n'ai pas envie de sortir tout le monde.

*Mme. 2:* Ouais.

*Mme. 2:* Souvent je vois aussi que, [euh], le marché, c'est un but aussi de- [euh] de sortir sa famille, je vois ça souvent le mercredi.

*Mme. 1:* Ouais.

*Mme. 2:* Je vous dis ça, mais, [euh], pour moi, ce n'est pas forcément le- le cas, [quoi]. Le mercredi, je sais qu'il faut y aller de bonne heure comme ça au moins, je profite plus de mes enfants après, [hein].

*Mme. 1:* Ouais.

*Mme. 1:* Voilà, Ils font la grasse mat pendant ce temps-là.

*Mme. 2:* Donc, [euh], la première raison pour laquelle vous allez au marché, c'est pour la qualité des produits ?

*Mme. 1:* Ouais.

*Mme. 2:* Que ce soit sur la saveur ou la conservation.

*Mme. 1:* Et c'est local, [quoi], ouais, c'est ça.

*Mme. 2:* Et c'est local en plus.

*Mme. 1:* Ouais, ouais, ouais, voilà.

*Mme. 2:* Vous avez remarqué une différence sur la conservation par rapport à d'autres, [euh]--.

*Mme. 1:* Mm, [eh], [ben), je n'ai pas trop, [euh], regardé, mais, [en fait]--.

*Mme. 2:* Ouais.

*Mme. 1:* C'est vrai, je pense que ouais, ça doit se valoir, mais la saveur pour- pour moi, ce n'est pas du tout pareil.

*Mme. 2:* OK.

*Mme. 1:* Pour moi, ça se ressent dans les fromages, [quoi].

*Mme. 2:* D'accord.

*Mme. 1:* [Ah], [ben] Ouais, franchement, comparer un fromage, [euh], d'artisan avec un fromage de grande surface. Le- l'autre jour, j'ai essayé avec- tiens, je vais aller à Leclerc pour essayer de comparer.

*Mme. 2:* Ouais.

*Mme. 1:* [Eh], [ben] Ce n'est pas du tout pareil.

*Mme. 1:* Ouais.

*Mme. 1:* Et pourtant, Lecler, c'était à la- la- la coupe qu'elle faisait ça, [hein].

*Mme. 1:* Oui.

*Mme. 1:* Le-Le petit brie de Néo-là.

*Mme. 2:* Ouais.

*Mme. 1:* [Eh], [ben] Ce n'est pas du tout pareil.

*Mme. 2:* Ce n'est pas le même.

*Mme. 1:* [Ah] Ouais, ouais.

*Mme. 2:* Donc, surtout ouais, on--.

*Mme. 1:* L'affinage n'est pas le même, ouais.

*Mme. 2:* Et, [euh], vous avez dit, il y a un côté plaisant sauf quand les petits vieux qui--.

*Mme. 1:* Ouais, ouais, c'est ça, voilà, ouais, c'est ça.

*Mme. 2:* Et, [euh], oui, vous trouvez que le marché, c'est plutôt un lieu agréable dans lequel faire ses courses--.

*Mme. 1:* Ouais.

*Mme. 2:* Ou c'est vraiment par nécessité que vous y allez ?

*Mme. 1:* Bon, c'est quand même la nécessité.

*Mme. 2:* Ouais.

*Mme. 1:* Parce que je vais vous dire- je vais vous dire que ce n'est quand même pas facile d'accès. Comme tout le monde y va, c'est une fois par semaine, [euh], forcément, il y a un engorgement qui se crée et il faut se garer à une grande surface là-bas pour aller là.

*Mme. 2:* Ouais.

*Mme. 1:* Il faut se motiver, [quoi].

*Mme. 2:* Il n'est pas très pratique finalement, [euh].

*Mme. 1:* [Eh], [ben], moi, je trouve que d'accès, c'est- c'est nul, [quoi], mais bon.

*Mme. 2:* Ouais.

*Mme. 1:* Après, mais- à mon avis, il n'y a pas le choix, dans tous les patelins, c'est pareil remarquez. Je pense que--.

*M. [Prénom du conjoint]:* Mm.

*Mme. 1:* C'est ça ou alors faudrait vraiment une grande surface énorme.

*M. [Prénom du conjoint]:* Et même si t'allais à une supérette, [euh], du centre-ville, [euh].

*Mme. 1:* Ouais.

*M. [Prénom du conjoint]:* T'as pas 36 places.

*Mme. 1:* [Eh], [ben], c'est ça.

*Mme. 2:* Ouais.

*Mme. 1:* S'il fallait se mettre à leur place, je comprends bien le problème, [quoi).

*Mme. 2:* Ouais et- et, [euh], est-ce que ça vous arrive de perdre, [euh], des- des fruits et légumes que vous avez achetés sur le marché ?

*Mme. 1:* Non.

*Mme. 2:* Non, c'est rare.

*Mme. 1:* Non, non, franchement, non, non, non, non parce qu'il y a quand même un budget dans l'histoire, je pense, [hein].

*Mme. 2:* Ouais.

*Mme. 1:* Peut-être, je- je ne compare pas, je ne suis pas là pour comparer, [en fait].

*M. [Prénom du conjoint]:* On assume, on sait que faire une soupe avec des produits du marché, c'est plus cher que, |euh]--.

*Mme. 1:* Ouais, tu crois ?

*M. [Prénom du conjoint]:* Que d'aller acheter une soupe, je ne sais pas.

*Mme. 1:* [Ah] Je ne suis pas sûr, [hein].

*M. [Prénom du conjoint]:* Ouais.

*M. [Prénom du conjoint]:* Mm.

*M. [Prénom du conjoint]:* [Eh], [ben] Le prix des haricots, par exemple l'été.

*Mme. 1:* Mm.

*M. [Prénom du conjoint]:* C'est cher, on n'en achète pas.

*Mme. 1:* [Ah], c'est vrai, ouais, c'est sept, huit euros le kilo, on ne te dit pas, [quoi].

*M. [Prénom du conjoint]:* Mm.

*Mme. 1:* Ouais, c'est casque, [hein].

*Mme. 2:* Et puis, [euh], vous, vous avez pas mal de bouches à nourrir.

*M. [Prénom du conjoint]:* Mm-hmm.

*Mme. 1:* Ouais, souvent, j'y vais par trois, quatre kilos les haricots parce que sinon, [hein], on va se- on va s'étriper pa- parce qu'on n'en a pas assez.

*Mme. 2:* Ouais.

*Mme. 1:* Ouais, c'est ça.

*Mme. 2:* Donc, [euh], globalement, tu dirais quand même qu'il y a un budget. C'est- c'est peut-être un peu plus cher--.

*Mme. 1:* Ouais.

*Mme. 2:* Au marché que--.

*Mme. 1:* Peut-être, ouais, [eh], [ben], c'est sûr, |hein].

*Mme. 2:* Ouais.

*Mme. 1:* Mm, moi, je pense, ouais, c'est- si tu vois les choses comme ça.

*M. [Prénom du conjoint]:* [Eh], [ben] Par exemple, les pommes de terre.

*Mme. 1:* Mm-hmm.

*M. [Prénom du conjoint]:* C'est clairement moins cher chez le producteur.

*Mme. 1:* Ouais.

*Mme. 2:* OK.

*Mme. 1:* C'est que moi, je les prends en grande quantité aussi.

*Mme. 1:* Ouais, on va acheter, [euh]--.

*Mme. 1:* Mais même que--.

*M. [Prénom du conjoint]:* Huit sacs de 25 kilos, [euh].

*Mme. 1:* Vingt-cinq kilos, c'est 10 euros, donc, [euh]--.

*M. [Prénom du conjoint]:* Mm.

*Mme. 2:* Ouais, OK.

*Mme. 1:* Pour moi, ce n'est pas grand-chose.

*M. [Prénom du conjoint]:* En- en- en supermarché, on ne verra pas, [euh]--.

*Mme. 1:* Non, je ne crois pas.

*M. [Prénom du conjoint]:* Ces prix-là.

*Mme. 1:* C'est des petites quantités, [quoi].

*M. [Prénom du conjoint]:* Mais parce qu'on y va en- en grossiste, [quoi].

*Mme. 1:* Mais pour- pour nous-.

*Mme. 1:* Ouais.

*Mme. 1:* Il n'y a pas assez de g- de, [euh], justement de grossiste, je trouve.

*M. [Prénom du conjoint]:* Mm.

*Mme. 1:* Ouais.

*Mme. 1:* Rapport aux légumes, choux et tout ça pareil, je- je- j'en prendrais bien, [euh], plus que ça, [quoi], mais bon.

*Mme. 2:* Ouais.

*M. [Prénom du conjoint]:* Mais après ça ne se conserve pas.

*Mme. 1:* Ouais, c'est ce que je me dis, ça va être piqué, c'est- c'est ça.

*Mme. 2:* Ouais.

*Mme. 2:* Il y a le co- côté conservation, [quoi]. Les carottes, c'est pareil, [hein]. Non, par contre, moi, en conserve, il n'y a que le-, [euh], enfin, on conserve en grosses quantités, il y a les pommes de terre et les- les oignons

*Mme. 1:* Et les oignons, ouais, là, en feuilles, c'est que ça se conserve.

*M. [Prénom du conjoint]:* Mm.

*Mme. 2:* Mm.

*Mme. 1:* Vous pouvez conserver presque toute l'année, [euh].

*M. [Prénom du conjoint]:* Ou au moins plusieurs mois.

*Mme. 1:* Ouais, ouais.

*Mme. 2:* Mm.

*Mme. 1:* Ouais, ouais, d'une année. D'accord, donc, [euh], ce qui fait- la première raison pour laquelle vous- vous ne voulez pas jeter, [euh], donc vous faites tout pour éviter de jeter--.

*Mme. 2:* Ouais.

*Mme. 1:* C'est aussi une question de budget, le fait de ne pas perdre vos, [euh]--.

*M. [Prénom du conjoint]:* Ouais.

*Mme. 2:* [Eh], [ben] Ouais, quand même et puis c'est triste de- de voir des- des- des bons produits de qualité, [euh] jetés, [euh], ça, c'est chiant, [hein]. Ça ne nous arrive pas, [quoi], quand même.

*Mme. 1:* Ouais.

*Mme. 2:* Hein.

*M. [Prénom du conjoint]:* Il y a quand même comme un- une sorte de respect pour la nourriture.

*M. [Prénom du conjoint]:* Ouais.

*Mme. 2:* [Ah], [ben], oui, oui, c'est sûr, [hein].

*M. [Prénom du conjoint]:* Mm.

*Mme. 1:* [Ah] Ouais.

*M. [Prénom du conjoint]:* Mais c'est aussi l'éducation qu'on donne--.

*Mme. 2:* Mm.

*M. [Prénom du conjoint]:* Aux enfants à table, on ne joue pas avec, [euh].

*Mme. 2:* [Ah], ouais, on termine l'assiette parce qu'alors là, alors là, qu'est-ce qu'on a gueulé pour ça ?

*M. [Prénom du conjoint]:* Là, il y a super exemple, heureusement que l'enregistrement--.

*Mme. 2:* [Ah], ouais.

*M. [Prénom du conjoint]:* Il n'est pas en vidéo, là c'est des--.

*Mme. 2:* Non, mais la cuillère à soupe du-, [euh], du bébé.

*Mme. 1:* Ouais.

*Mme. 1:* Non, il n'avait pas faim, il revenait d'hôpital, le pauvre.

*Mme. 1:* Ouais.

*Mme. 1:* Ouais.

*Mme. 2:* Non.

*Mme. 1:* Et du coup, il l'a finira, [euh), plus tard ?

*M. [Prénom du conjoint]:* Ouais.

*Mme. 2:* [Ah] Je ne sais pas.

*M. [Prénom du conjoint]:* Alors souvent quand il n'y touche pas, on resserre.

*Mme. 2:* Ouais, mais rare sont les fois quand même tout ce qui est purée, on ne les réchauffe pas.

*M. [Prénom du conjoint]:* Alors il y a des choses--.

*Mme. 1:* Ouais.

*M. [Prénom du conjoint]:* Qui ne se réchauffent pas comme les épinards, les carottes.

*Mme. 2:* Mm.

*M. [Prénom du conjoint]:* Les patates, ce n'est recommandé.

*Mme. 2:* Les patates, ce n'est pas bon quand c'est réchauffé.

*Mme. 1:* [Ah], ouais ?

*M. [Prénom du conjoint]:* Ouais.

*Mme. 2:* Ouais.

*Mme. 1:* Il ne faut pas re- réchauffer ?

*Mme. 2:* Il y a un goût.

*M. [Prénom du conjoint]:* Il y a un goût.

*Mme. 2:* [Eh], [ben] Les pommes, enfin, je veux dire, [euh], quand j'ai en- en soupe, je vais le faire deux fois.

*M. [Prénom du conjoint]:* Mm.

*Mme. 1:* D'accord.

*Mme. 2:* Donc, là, il y a un deuxième, [euh], donc il y a- il y a un réchauffage, [quoi].

*Mme. 1:* Ouais.

*Mme. 2:* Mais on sent que la pomme de terre, déjà, elle vire de goût.

*M. [Prénom du conjoint]:* [Ah], elle vire de goût.

*Mme. 1:* OK.

*Mme. 1:* Et la pomme de terre pure, si elle est toute seule, je la balance.

*Mme. 1:* D'accord.

*Mme. 1:* Mais, [euh], les poules, [euh], bon, pour recycler.

*Mme. 1:* Ouais.

*M. [Prénom du conjoint]:* Et, [en fait], quand on balance, c'est recyclé aux poules, c'est recyclé.

*Mme. 2:* Ça- voyez-vous, ça part aux poules.

*M. [Prénom du conjoint]:* Mm.

*Mme. 2:* Ouais.

*M. [Prénom du conjoint]:* Ou au compost ou aux poules, mais, [euh]--.

*Mme. 2:* Ouais, c'est ça.

*M. [Prénom du conjoint]:* Tout ce qui est comestible pour les poules, c'est--.

*Mme. 2:* Mm.

*M. [Prénom du conjoint]:* C'est elle qui ont la-, la primeur. Quelquefois, il y a le chat du voisin qui passe et--.

*Mme. 1:* Ouais.

*Mme. 2:* Ouais.

*M. [Prénom du conjoint]:* Il n'est pas encore végétarien.

*Mme. 2:* [Ah] Ouais, c'est ça.

*Mme. 2:* OK, alors si on prenait une autre photo.

*Mme. 2:* [Ah], [ben] voilà !

*Mme. 1:* Qu'est-ce qui vous vient à l'esprit ?

*Mme. 2:* [Ah], [ben] Là, ça va être plutôt, [euh], vous savez, les marchands de légumes, là.

*Mme. 1:* Ouais.

*M. [Prénom du conjoint]:* Le job d'été pour les enfants, moi, je dirais.

*Mme. 2:* Ouais, c'est pas mal, c'est- c'est--. Moi, je vois le chat vu plus toi mec dans, les champs, tu vois, Là, j'aurais dit, job d'été, ouais, AAA dans un magasin.

*Mme. 1:* [Euh].

*Mme. 2:* Moi, ça, j'y vais jamais.

*Mme. 2:* Ouais.

*M. [Prénom du conjoint]:* Ça, c'est un- c'est un magasin de vente de produits de proximité.

*Mme. 2:* Ouais.

*M. [Prénom du conjoint]:* On dirait.

*Mme. 2:* On va dire une Biocoop ou peu importe.

*M. [Prénom du conjoint]:* Mm, ouais, enfin--

*Mme. 2:* Enfin, ça va être dans ce principe.

*M. [Prénom du conjoint]:* Il s'en- il s'en est monté un il n'y a pas longtemps, là.

*Mme. 1:* Mm.

*M. [Prénom du conjoint]:* Et ils ont fermé, je crois.

*Mme. 2:* [Ah], ouais, [ben], je ne sais pas. Bon, en tout cas, moi, je n'y vais pas.

*Mme. 1:* D'accord.

*Mme. 2:* Parce que comme il y a le marché une fois par semaine, je sais que je fais mon approvisionnement exclusivement là-dessus.

*Mme. 1:* Ouais.

*M. [Prénom du conjoint]:* Et en-, [en fait], ouais, ce petit magasin, on avait vu que c'était, [euh], super cher.

*Mme. 1:* Et puis, c'est- c'est vachement plus cher, [eh], [ben], du coup, parce qu'on sait que ce n'est plus le producteur, mais c'est dire-, c'est il y a un intermédiaire alors là, il y a une plus-value, enfin, je ne sais pas.

*M. [Prénom du conjoint]:* Ils utilisent le- les- l'étiquette locale, [euh].

*Mme. 2:* Ouais, c'est ça.

*M. [Prénom du conjoint]:* Bio local et après--.

*Mme. 2:* Ouais.

*M. [Prénom du conjoint]:* Je crois que c'est exactement le nom de--.

*Mme. 2:* Et toi t'es tombé sur des arnaques où ils ne mettaient pas des étiquettes bios et en fin de compte--.

*M. [Prénom du conjoint]:* Ouais, [en fait]--.

*Mme. 2:* Il n'y avait rien de bio.

*M. [Prénom du conjoint]:* Devant le stand--.

*Mme. 2:* Mm.

*M. [Prénom du conjoint]:* Il y avait une banderole agriculture biologique.

*Mme. 2:* Ils peuvent faire ce qu'ils veulent aussi.

*M. [Prénom du conjoint]:* Et en fait, ils vendaient des choses non bio, ouais.

*Mme. 1:* Ouais, ouais.

*Mme. 2:* [Eh], [ben] Ouais, enfin bon. Après ces gens-là, [euh], ils font un noble métier dans le sens où quand c'est en- en pleine ville, t'es content d'avoir ça, [quoi], donc tu n'as pas toujours--.

*M. [Prénom du conjoint]:* [Euh] L'outil, il est noble que quand- quand c'est eux qui cultivent et qui vendent.

*Mme. 2:* Ouais alors c'est ça.

*M. [Prénom du conjoint]:* Très bien, il y a- il y a des gens qui s'in--.

*Mme. 2:* Ouais, c'est ça.

*M. [Prénom du conjoint]:* Qui- qui s'inquiètent juste d'acheter ça cher--.

*Mme. 2:* Ouais, c'est vrai que--.

*M. [Prénom du conjoint]:* Et de le revendre plus cher.

*Mme. 2:* [Eh) [ben] Ça, c'est le co- c'est le commerce d'une façon générale, [hein].

*M. [Prénom du conjoint]:* Ouais, mm.

*Mme. 2:* Regarde le textile, ce n'est pas mieux, [hein], voilà.

*Mme. 1:* Ouais.

*Mme. 2:* Enfin, bon, on ne va pas re- rentrer dans les trucs de-, [euh], de philosophie politique, mais c'est vrai que c'est- que c'est assez exécrable, je n'aime pas.

*Mme. 1:* Ouais, donc vous, parce que, [euh], vous avez enfin--.

*Mme. 2:* Il y a--.

*Mme. 1:* Accès au marché--.

*Mme. 2:* Ouais.

*Mme. 1:* Vous ne voyez pas forcément l'intérêt de vous rendre dans un--.

*Mme. 2:* D'un intermédiaire.

*Mme. 1:* Un lieu comme ça, ça sera plus cher, [euh].

*M. [Prénom du conjoint]:* Après parce qu'on n'en a pas des masses ici, [hein].

*Mme. 2:* Non.

*M. [Prénom du conjoint]:* On a eu une tentative qui était pour moi un peu trop cher, donc on a- on a jugé, OK, la qualité des produits est là.

*Mme. 2:* Mm, [oh], ouais.

*M. [Prénom du conjoint]:* Mais, [euh], le prix élevé n'est pas justifié.

*Mme. 1:* Ouais, OK.

*M. [Prénom du conjoint]:* En plus, il devrait être moins cher puisqu'il n'y a pas de- d'intermédiaire.

*Mme. 1:* Ouais.

*M. [Prénom du conjoint]:* Donc, on n'a pas compris pourquoi on n'y- on ne gagne pas.

*Mme. 2:* [Ah] C'est vrai, c'est- c'est le bio local, là ?

*M. [Prénom du conjoint]:* Ouais, le bio local.

*Mme. 2:* [Ah], ça n'est pas tenu, [hein], ils sont- ils ont fermé en peu temps.

*Mme. 1:* [Ah] Ouais.

*M. [Prénom du conjoint]:* Et, [euh], par contre, ta- ta copine- ta copine à Nantes.

*Mme. 2:* Ouais.

*M. [Prénom du conjoint]:* Ils ont des- des espèces des grandes halles.

*Mme. 2:* [Ah] Ouais et c'est ça.

*M. [Prénom du conjoint]:* Ou, [euh], on retrouve cet esprit où tout le monde vient à Nantes sur la- avec sa propre- sa culture.

*Mme. 2:* Ouais, non, mais alors c'est de grosses quantités, ouais, [eh], [ben], franchement, c'est génial, [quoi].

*M. [Prénom du conjoint]:* Ouais, mais ça, c'est des- des produits locaux.

*Mme. 2:* Mais, [euh], ouais.

*M. [Prénom du conjoint]:* Et pas chers, [euh], disponibles en grande quantité, [euh].

*Mme. 1:* Ouais.

*Mme. 2:* Mm.

*M. [Prénom du conjoint]:* Enfin, quand je dis pas cher, c'est abordable, bien sûr.

*Mme. 2:* C'est à Ancenis.

*Mme. 1:* Oui.

*Mme. 2:* Ouais, ça se trouve à Ancenis à sa- sa grosse zone alors il y a un gros carrefour ou je ne sais pas. Une grosse grande surface et de l'autre côté, il y a tout ce qui est textile, [euh]--.

*M. [Prénom du conjoint]:* Mm.

*Mme. 2:* Légumes comme ça, mais un gros truc.

*Mme. 1:* Ça fait des marchés couverts quand- un peu ou ?

*Mme. 1:* C'est incroyable, ouais, c'est ça et c'est super facile d'accès, [quoi].

*M. [Prénom du conjoint]:* Mais je le- c'est un marché de gros et ouvert aux particuliers.

*Mme. 2:* Ouais.

*Mme. 1:* Ouais.

*M. [Prénom du conjoint]:* Souvent, les marchés de gros sont pour les commerçants.

*Mme. 1:* Ouais, elles sont- c'est ça.

*M. [Prénom du conjoint]:* Alors quand je suis allée la voir, [euh], il y avait des barquettes, [euh], deux fois ça de-, [euh], d'abricot. Elle me dit :"[Ah] C'est pour les confitures, t'en veux ?" et je lui dis : "[Ah], ouais, [eh], [ben], tiens, je vais en prendre", mais on les a même consommés comme ça, [quoi].

*Mme. 1:* Ouais.

*M. [Prénom du conjoint]:* Mm-mm.

*Mme. 2:* Mm-hmm, on était content d'en avoir, [quoi].

*M. [Prénom du conjoint]:* Mm-mm.

*Mme. 2:* En plus, c'est le sud là-bas.

*M. [Prénom du conjoint]:* J'ai fait de la confiture, moi.

*Mme. 2:* Ouais, on t'en a fait un, deux.

*M. [Prénom du conjoint]:* Deux.

*Mme. 2:* Pas grand-chose, [quoi].

*M. [Prénom du conjoint]:* Ouais, il m'arrive--.

*Mme. 1:* Parce qu'on avait tout raflé.

*Mme. 2:* Tout mangé avant.

*Mme. 2:* Ouais, avant ici, [euh]--.

*M. [Prénom du conjoint]:* Je crois qu'il n'a pas eu le temps de refroidir.

*Mme. 2:* [Eh], [ben] Alors c'est ça. Ici, les fruits sont quand même pas chers, très sucrés et développés et tout, donc on les consomme ici dans le Sud.

*Mme. 1:* Ouais.

*M. [Prénom du conjoint]:* Non, quand même on a- on a des beaux légumes verts et des patates, mais moins de fruits.

*Mme. 2:* Ouais, c'est ça.

*Mme. 1:* Ouais.

*M. [Prénom du conjoint]:* À part les pommes.

*Mme. 2:* Ouais, c'est ça.

*Mme. 1:* Ouais, donc, [euh], c'est un lieu dans lequel vous, vous n'y allez pas--.

*Mme. 2:* Non.

*Mme. 1:* Parce qu'il n'y a pas d'intérêt, [euh], premier--.

*Mme. 2:* Non.

*Mme. 1:* Mais si vous étiez en grande ville et que, [euh], vous- vous n'aviez moins d'accès peut-être au marché ou quoi ?

*Mme. 2:* Ouais, peut-être.

*Mme. 1:* À la rigueur, pourquoi pas ? Mais sinon, là, il n'y a pas de, [euh]--.

*M. [Prénom du conjoint]:* Non, c'est le rendez-vous de- de toute façon, c'est--.

*Mme. 2:* Ouais, c'est ça.

*M. [Prénom du conjoint]:* Une chose qu'il y a dans les marchés, donc--.

*Mme. 1:* Ouais.

*Mme. 2:* Ouais.

*M. [Prénom du conjoint]:* Pourquoi pas, moi, je ne suis pas contre.

*Mme. 2:* Mm.

*M. [Prénom du conjoint]:* Après, encore une fois, |hein], nous, on est--.

*Mme. 2:* Mm.

*M. [Prénom du conjoint]:* Consommateur, on essaye de choisir--.

*Mme. 2:* Ouais.

*M. [Prénom du conjoint]:* De la qualité, mais aussi on regarde, [euh]--.

*Mme. 2:* [Eh], [ben], le producteur aussi, [hein], moi, je regarde

*M. [Prénom du conjoint]:* Les- les quand je parle de l'éthique, elle est dans les deux sens. OK, [euh], le producteur, il faut qu'il trouve ses comptes.

*Mme. 2:* Non, on va regarder, [hein].

*Mme. 1:* Ouais.

*M. [Prénom du conjoint]:* Mais le consommateur, je veux dire, ce n'est pas un pigeon non plus, [quoi].

*Mme. 2:* Non et puis en- en plus--.

*Mme. 1:* Ouais.

*Mme. 2:* Il ne faut quand même pas oublier que je fais le mercredi, mais je fais aussi le vendredi soir directement chez le producteur.

*M. [Prénom du conjoint]:* Mm, mm.

*Mme. 1:* Ouais.

*Mme. 2:* Là, je ne veux même plus l'avoir sur le marché, donc, [en fait], si j'avais ça en ville, [euh)--.

*M. [Prénom du conjoint]:* Mm-hmm.

*Mme. 2:* Je vous avouerais que j'irais quand même pour les grosses quantités chez un producteur.

*Mme. 1:* [Eh], [ben] Ouais.

*M. [Prénom du conjoint]:* Ouais, ouais, mais nous, on est une famille un petit peu particulière.

*Mme. 2:* Ouais, ouais, il faut le dire aussi, [hein]. Ouais, parce que--.

*M. [Prénom du conjoint]:* C'est genre-là, si on se sert, on- on--.

*Mme. 2:* [Eh], [ben].

*M. [Prénom du conjoint]:* On enlève quatre rayons, [quoi].

*Mme. 2:* Ouais, [en fait], c'est- c'est- si je lui demande, [euh], [eh], [ben], veuillez me mettre 25 kilos de patates s'il vous plait, je crois. Que ça--.

*Mme. 1:* Ouais, ce n'est même pas sûr qu'il les ait même là, [euh].

*Mme. 2:* Ouais, c'est ça.

*Mme. 1:* C'est ce que lui va chercher en grossiste, [euh].

*Mme. 2:* Mm-mm, ouais.

*M. [Prénom du conjoint]:* C'est qu'est-ce qu'il écoule dans la- dans la journée durant--.

*Mme. 2:* Ouais, ouais, ça se voit, [hein], tu m'étonnes.

*Mme. 1:* Et donc, [euh], le producteur chez qui vous- que vous retrouvez mercredi au marché--.

*Mme. 2:* Mm.

*Mme. 1:* Vous alliez aussi le vendredi, [euh] ?

*Mme. 2:* Ouais, [eh], [ben], ouais, des fois, je fais--.

*M. [Prénom du conjoint]:* [En fait] Il est ouvert le mardi soir.

*Mme. 2:* Alors, moi, je--.

*M. [Prénom du conjoint]:* Et le vendredi.

*Mme. 2:* Ouais.

*Mme. 1:* Ok.

*Mme. 2:* C'est vachement pratique.

*M. [Prénom du conjoint]:* Et on n'y va- on n'y va que, [euh], le vendredi parce que mardi, on fait d'autres choses, [quoi]. Il y a les enfants à l'école, [eh], [ben], enfin--.

*Mme. 1:* Ouais.

*M. [Prénom du conjoint]:* Vendredi aussi, mais--.

*Mme. 2:* Mm.

*M. [Prénom du conjoint]:* [Eh], [ben], [en fait] Le mardi et mercredi le lendemain, [quoi].

*Mme. 2:* Ouais, ouais, c'est ça. Ouais, il y a mercredi, le lendemain.

*Mme. 2:* Mm.

*Mme. 2:* Et puis, [euh], le vendredi soir, c'est bien parce que ça me finit la semaine. Et puis je sais que si je prends de la mâche le mercredi, ça ne va pas forcément être top le vendredi.

*M. [Prénom du conjoint]:* Ouais, ouais.

*Mme. 1:* Ouais.

*Mme. 2:* Ça AAA, donc je reprends de la mâche. En ce moment, il y en a énormément à- super bonne.

*Mme. 1:* Ouais, ouais.

*Mme. 2:* Je vais reprendre vendredi soir, [eh], [ben], c'est génial.

*Mme. 1:* Oui, ça vous coupe la semaine en deux, [euh].

*Mme. 2:* Ouais, c'est ça, mm et puis une heure pareille, [hein] et voilà.

*Mme. 1:* OK alors une autre photo, on va toutes les faire.

*Mme. 2:* [Ah], ouais, [eh], [ben], super.

*M. [Prénom du conjoint]:* Mais c'est la même.

*Mme. 2:* Je ne la vois pas, [ah], oui, [eh], [ben], là, c'est--.

*M. [Prénom du conjoint]:* Non, là, c'est le supermarché.

*Mme. 1:* C'est le supermarché, [hein], mm.

*M. [Prénom du conjoint]:* Mm et alors, c'est quoi ? C'est des- des--.

*Mme. 2:* Pour moi, ça va être plus que les fruits quand je n'en ai plus, [euh], avec le-, [euh], les- les pommiers du jardin, [quoi].

*Mme. 1:* Ouais.

*M. [Prénom du conjoint]:* C'est un supermarché, mais c'est- ouais, il y a ces-, ces--.

*Mme. 2:* Ça va être, [euh], poires quand elles sont bien développées. En ce moment, ça va encore, les poires et des bananes, [hein].

*M. [Prénom du conjoint]:* Non, on va prendre- on va prendre, [euh]--.

*Mme. 2:* C'est un truc qui revient tout le temps, les bananes, ça serait le planning.

*M. [Prénom du conjoint]:* Ouais, exceptionnellement, on va prendre des carottes parce que, [euh], envie de faire un plat avec des carottes et puis il fait ça avec--.

*Mme. 2:* Mais ça, c'est vraiment le truc d'urgence, [quoi].

*M. [Prénom du conjoint]:* Mm.

*Mme. 2:* Mm, pour les légumes, c'est les trucs d'urgence. Par contre, les fruits, [eh], [ben], oui, je les prends-là, [quoi).

*M. [Prénom du conjoint]:* Ouais parce qu'ici, [euh]--.

*Mme. 2:* Mm

*Mme. 1:* Ouais.

*M. [Prénom du conjoint]:* Les détaillants n'ont pas beaucoup de fruits, [hein], alors AAA.

*Mme. 1:* Non.

*Mme. 2:* Mm.

*Mme. 2:* Oui et en plus, c'est une région de, [euh], légumes que de--.

*M. [Prénom du conjoint]:* Mm.

*Mme. 2:* Ouais, c'est ça.

*Mme. 1:* Que de fruits, donc, et en fruits, ce serait exclusivement bananes et poires.

*Mme. 2:* Et poires.

*Mme. 1:* Ouais.

*Mme. 2:* Et pommes, [euh], et après quand c'est hors saison, enfin.

*M. [Prénom du conjoint]:* Mm.

*Mme. 1:* Ouais.

*Mme. 2:* Ouais.

*M. [Prénom du conjoint]:* On peut trouver de temps en temps des- ce- non les cerises, on les prend au marché.

*Mme. 2:* C'est- les cerises, [eh], [ben], si.

*M. [Prénom du conjoint]:* Au marché ? On les prend au supermarché ?

*Mme. 2:* [Ah] Moi, je les prends au supermarché à Intermarché, je t'assure, elles ne sont pas mauvaises en juin, là.

*M. [Prénom du conjoint]:* Cerises ?

*Mme. 2:* Mm.

*M. [Prénom du conjoint]:* [Euh] Ici, les fraises, on les achète au détaillant-là.

*Mme. 2:* [Eh], [ben] Non, au marché.

*M. [Prénom du conjoint]:* Non, au marché, oui, oui, c'est ça.

*Mme. 2:* [Ah], [bah] Moi, c'est au marché chez Pardo, [hein].

*M. [Prénom du conjoint]:* Oui, oui, oui.

*Mme. 2:* Toujours ma- ma productrice de patates.

*Mme. 1:* [Ah] Oui, c'est des fraises.

*Mme. 2:* C'est des- des fraises.

*M. [Prénom du conjoint]:* C'est des fraises AAA et qui sont très bien.

*Mme. 2:* [Ah] Ouais, ouais, elles sont très bien.

*M. [Prénom du conjoint]:* [Euh].

*Mme. 2:* Mais on ne prend pas des quantités importantes.

*M. [Prénom du conjoint]:* Juste pour comprendre comme légumes des- des fois, alors les Kiwis, on en prend de temps en temps.

*Mme. 2:* On en a.

*M. [Prénom du conjoint]:* Mais on les produit maintenant, [euh].

*Mme. 2:* D'accord, vous avez des-, [euh], des kiwiers ?

*Mme. 2:* Ouais, normalement, on en a plusieurs.

*Mme. 1:* Ouais.

*Mme. 2:* Là, je- on les a pas encore récoltées, mais il va en avoir.

*M. [Prénom du conjoint]:* Ouais, ça va être, [euh]--.

*Mme. 2:* L'année dernière, dans les 10 kilos, je pense que cette année, [euh]--.

*Mme. 2:* Là, c'est- c'est nickel, je crois.

*Mme. 1:* Ouais, ouais, c'est marrant, il y a des gens qui- enfin moi, petite, j'en avais un chez moi, [euh], chez mes parents.

*Mme. 2:* [Ah] Ouais, ouais, c'est embellissant.

*Mme. 1:* En Normandie, ça poussait, oui, c'est- c'est incroyable ce qu'on avait.

*Mme. 2:* [Ah] C'est ça.

*Mme. 1:* Et-, [euh], et les personnes qui consomment des kiwis mais qui ne savent pas qui ça se pousse-, [euh], enfin que ça pousse très bien, [euh], ici.

*Mme. 2:* [Ah], c'est incroyable. Ici, c'est impeccable.

*Mme. 1:* On les fait importer de Nouvelle-Zélande, mais--.

*Mme. 2:* [Ah], [ben], c'est ça.

*M. [Prénom du conjoint]:* C'est- ouais, il y a--.

*Mme. 2:* Et c'est un cadeau qu'on nous a offert quand on est arrivé en AAA, on s'est dit, mais il- il se moque de nous.

*M. [Prénom du conjoint]:* [Euh], non, non c'était pour--.

*Mme. 2:* Si, c'était Bostik, ton directeur de test.

*M. [Prénom du conjoint]:* Oui, oui, mon directeur de thèse, mais, [bof].

*Mme. 2:* Qui t'avait offert ça.

*M. [Prénom du conjoint]:* Et c'est pour la naissance de--.

*Mme. 2:* [Ah] Ouais, je ne sais plus.

*M. [Prénom du conjoint]:* De Flavie je crois non ?

*Mme. 2:* Enfin, on était- on était arrivé ici, il n'y a pas longtemps.

*M. [Prénom du conjoint]:* Ou des jumeaux, ouais, mais c'est après ils nous ont offerts ça--.

*Mme. 2:* Ouais, trois, deux, trois ans.

*Mme. 1:* Ouais.

*M. [Prénom du conjoint]:* Pour la naissance de- d'un nos enfants et- et depuis-, [euh], depuis trois ans, on fait des- des- les plusieurs, peut-être 10 kilos de kiwis.

*Mme. 2:* Ouais, ouais, ouais, c'est énorme.

*M. [Prénom du conjoint]:* Et alors on se fait- on fait des smoothies avec.

*Mme. 1:* Ouais.

*Mme. 2:* Ok alors quand j'ai ça, ouais, [eh], [ben], c'est génial. Franchement, ouais, ça nous tient au moins jusqu'à janvier, [hein], en décembre, janvier, février, [hein].

*M. [Prénom du conjoint]:* Au plus.

*Mme. 2:* [Eh], [ben], parce qu'ils sont durs comme des cailloux.

*M. [Prénom du conjoint]:* [Ah] Si, on prend- on prend des avocats aussi au supermarché.

*Mme. 2:* [Ah] [bah] Voilà ça, j'en avais pris, j'en ai pris au- au supermarché-là.

*M. [Prénom du conjoint]:* On va sur la table ?

*M. [Prénom du conjoint]:* Ouais.

*Mme. 2:* Mais rare.

*M. [Prénom du conjoint]:* On ne va pas se mentir.

*Mme. 2:* C'est rare, Franchement.

*Mme. 1:* Ouais.

*Mme. 2:* Je t'assure que, [euh], c'est rare parce que moi, j'attends qu'il soit super mou comme l'arbre, souvent--.

*M. [Prénom du conjoint]:* Ouais.

*Mme. 2:* Ils sont presque-, [euh], et on appuie, [euh], c'est--.

*Mme. 1:* Ouais, puisque- alors ouais.

*Mme. 2:* On sent que c'est moyen.

*M. [Prénom du conjoint]:* Mm.

*Mme. 1:* Ouais.

*Mme. 2:* Sinon, quand ils sont durs comme du caillou, je sais qu'ils vont mal vieillir.

*M. [Prénom du conjoint]:* Ouais.

*Mme. 1:* Ouais.

*M. [Prénom du conjoint]:* Mais c'est vrai qu'on a-.

*Mme. 2:* Je t'assure.

*M. [Prénom du conjoint]:* On en a peu-, [euh], on achète peu des choses qui viennent loin.

*Mme. 1:* Ouais.

*Mme. 2:* Ouais, c'est ça.

*M. [Prénom du conjoint]:* À l'exception des bananes, je dirais la banane, c'est--.

*Mme. 2:* Et le citron peut-être.

*M. [Prénom du conjoint]:* Ouais, peut-être. Le citron, on en prend un, on n'en prend pas beaucoup.

*Mme. 2:* Comme on se fait du détox.

*M. [Prénom du conjoint]:* Et si, on prend des agrumes des fois, oui, oui.

*Mme. 2:* Ouais.

*M. [Prénom du conjoint]:* Mais, [euh], mais bon, les citrons, on n'en prend pas des masses, [hein].

*Mme. 1:* Ouais.

*Mme. 2:* Mm, les oranges aussi, ça- ça vient d'Espagne.

*M. [Prénom du conjoint]:* Mm.

*Mme. 2:* Oui et encore, du coup, ce n'est pas- ça va, ce n'est pas très, très loin.

*Mme. 1:* Je prends des clémentines de Corse en ce moment.

*M. [Prénom du conjoint]:* De Corse, ouais.

*Mme. 1:* Oui, voilà.

*Mme. 2:* Ouais.

*Mme. 1:* Oui, c'est des produits qu'on a dans le Sud--.

*Mme. 2:* Mm.

*Mme. 1:* En tout cas du Sud de l'Europe, bon, ce n'est pas loin.

*Mme. 1:* Ouais, c'est ça.

*M. [Prénom du conjoint]:* Il paraît que c'est encore en France, la Corse.

*Mme. 1:* [Eh], [ben], pour le moment, en tout cas, ouais, ouais.

*Mme. 2:* Ouais.

*Mme. 1:* Et, [euh], globalement, vous pensez quoi des produits que vous pouvez trouver en- en grande surface ?

*Mme. 2:* [Eh], [ben] Il faut les tester, enfin, [euh], les palper, [euh], Pour les- les poires, moi je sais ce que je fais.

*M. [Prénom du conjoint]:* [Eh], [eh], Les ferments- il faut- [eh], [eh], [ben], tous les fruits qu'on prend, ils sont à peine mûrs, [quoi].

*Mme. 1:* [Ah], oui, [ah], [ben], après les- les bananes, [euh], honnêtement, oui.

*M. [Prénom du conjoint]:* Il faut laisser mûrir.

*Mme. 2:* Comme je vous avais dit--.

*M. [Prénom du conjoint]:* Et--.

*Mme. 2:* Je prends deux semaines d'avance parce que là, je n'ai plus une seule banane. Mais je pense que, [euh]--.

*M. [Prénom du conjoint]:* Quand on- quand on revient de course avec les bananes, les enfants veulent en prendre une, on dit, non, non, elle est verte, il faut la laisser mûrir et on attend trois jours.

*Mme. 1:* OK.

*M. [Prénom du conjoint]:* Et ça, c'est- c'est frustrant.

*Mme. 2:* Ouais, c'est ça.

*M. [Prénom du conjoint]:* Ça atteste que, [euh], [eh], [ben), le cycle naturel n'est pas fini.

*Mme. 2:* Ouais, c'est ça.

*M. [Prénom du conjoint]:* Donc, j'imagine que la qualité nutritive de la banane, elle n'est pas au rendez-vous, [euh], et la conservation, [euh]--.

*Mme. 2:* Ouais.

*M. [Prénom du conjoint]:* Encore moins, [quoi].

*Mme. 2:* Mm.

*Mme. 1:* OK.

*Mme. 2:* Ouais, c'est ça. Enfin bon, en vrai, c'est des suppositions, mais c'est sûr qu'on n'est pas au top, [hein].

*M. [Prénom du conjoint]:* Ouais.

*Mme. 2:* Les bananes ça, je sais toujours était le truc AAA.

*M. [Prénom du conjoint]:* Même- même, je crois- je crois qu'on a déjà eu des--.

*Mme. 2:* Les poires.

*M. [Prénom du conjoint]:* Très souvent des pommes et des poires pas mûres.

*Mme. 2:* [Oh], |bien] Les poires sont dures

*M. [Prénom du conjoint]:* Ouais.

*Mme. 2:* Mais moi, je ne les prends pas dans ces cas-là, j'aime bien les prendre quand elles sont ramelots. Je sais au moins qu'elles vont être savoureuses parce que quand elles sont dures, je vous dis, [euh], ça me pourrit, ça--.

*Mme. 1:* Ouais.

*Mme. 2:* C'est comme les- les avocats, c'est des trucs qu'il faut pas acheter, [euh], dur, [quoi].

*Mme. 1:* C'est vrai qu'il y a plein de personnes qui sur les poires, c'est, [euh]--.

*Mme. 2:* Ouais, je ne sais pas, il y a un truc.

*Mme. 1:* Ouais.

*Mme. 2:* Ouais.

*Mme. 1:* Il y a les gens, ils disent que c'est une kata, enfin ils les reçoivent, elles sont- enfin, ils les achètent, elles sont dures.

*Mme. 2:* Voilà.

*Mme. 1:* Donc, ils les laissent, [euh], mûrir et puis en fait--.

*Mme. 2:* [Eh] [ben], voilà, c'est ça.

*Mme. 1:* Elles passent du dur à--.

*Mme. 2:* Ouais, c'est ça.

*Mme. 1:* À pourri ou alors au goût, elles ne sont pas bonnes.

*M. [Prénom du conjoint]:* C'est ça.

*Mme. 2:* C'est ça, ouais, c'est ça.

*Mme. 1:* C'est assez, [euh]--.

*Mme. 2:* Mm.

*M. [Prénom du conjoint]:* Et abricot, pêche, les pêches, les brugnons, [euh].

*Mme. 2:* [Oh] Ça aussi.

*Mme. 2:* [Oh].

*Mme. 2:* [Oh], là-, là, ce n'est pas-.

*Mme. 1:* Ça l'é- ça l'été, [euh]--.

*Mme. 2:* Les pêches plates.

*M. [Prénom du conjoint]:* Très souvent, [euh], même dans les restaurations collectives--.

*Mme. 2:* [Oh].

*M. [Prénom du conjoint]:* On tient un brugnon, on croque dedans.

*Mme. 2:* [Oh].

*M. [Prénom du conjoint]:* Il est super dur.

*Mme. 2:* Ouais.

*M. [Prénom du conjoint]:* [Ah], [ben], c'est comme ça, je vais ramener ma caisse, [euh].

*Mme. 2:* Ouais, ça vous arrive des fois de ramener, [euh] ?

*M. [Prénom du conjoint]:* [Ah] Ouais, en restauration collective ou à la cantine.

*Mme. 2:* [Ah] Oui.

*Mme. 2:* [Ah], [ben] Ouais, ouais, c'est ça.

*M. [Prénom du conjoint]:* C'est pareil, je prends un brugnon, il n'est pas mûre, j'amène- je vais voir le gars, je fais, il n'est pas mûr votre brugnon.

*Mme. 2:* Ouais, c'est vrai.

*M. [Prénom du conjoint]:* [Eh], [ben] Posez-le et allez chercher autre chose.

*Mme. 2:* C'est vrai que la moindre des choses, quand tu es en-, en cantine, c'est horrible, [ah], [ben] c'est vrai.

*M. [Prénom du conjoint]:* Je fais ça, surtout pour les narguer, pour leur dire que ce n'est pas correct de vendre des choses comme ça.

*Mme. 2:* [Eh], [ben] Ouais.

*M. [Prénom du conjoint]:* [Ah] Ouais, quelle horreur ?

*M. [Prénom du conjoint]:* Parce qu'on ne fait rien, ils vont continuer de le faire.

*Mme. 1:* Ouais, ouais, ouais, oui, oui.

*Mme. 2:* Et puis le repas à cinq euros, merci, [quoi].

*Mme. 1:* Oui, c'est ça. Oui et puis ça fait le- ça finit à la poubelle inutilement enfin ça--.

*Mme. 2:* Ouais.

*M. [Prénom du conjoint]:* Ouais.

*Mme. 2:* Ouais.

*Mme. 1:* Enfin on se retrouve à nous-mêmes jeter le--.

*Mme. 2:* Ouais.

*Mme. 1:* Le produit, [eh], [ben], ça, abricot, brugnon, pêche, l'été, vous en achetez où ?

*Mme. 2:* [Eh], [ben], c'est rare.

*M. [Prénom du conjoint]:* [Eh], [ben], du coup, du coup, rarement.

*Mme. 2:* C'est rare.

*M. [Prénom du conjoint]:* Quand on a- alors des fois, il y a un producteur--.

*Mme. 2:* Ma mère m'en passe de son marché à elle.

*M. [Prénom du conjoint]:* Ouais, quand on va sur-, [euh], sur--.

*Mme. 2:* Elle est à La Baule ma mère et du coup--.

*Mme. 1:* [Ah] Oui.

*Mme. 2:* Là-bas, c'est vachement développé dans les goûts, [hein], c'est bon.

*M. [Prénom du conjoint]:* C'est- ouais, il y a une meilleure saveur.

*Mme. 2:* Même, [euh], les melons.

*M. [Prénom du conjoint]:* Ici des fois et sur le parking de-, d'un parking Gamedair, il y a--.

*Mme. 1:* Ouais.

*Mme. 2:* [Ah] Ce n'est pas bon.

*M. [Prénom du conjoint]:* Il y a un bungalow.

*Mme. 2:* [Ah] Ouais alors là, c'est--.

*M. [Prénom du conjoint]:* Il y a quelqu'un qui vient de la Drôme, je crois.

*Mme. 1:* D'accord.

*M. [Prénom du conjoint]:* Et qui vient ici vendre ses produits.

*Mme. 2:* Ouais, ils ne sont pas bons.

*M. [Prénom du conjoint]:* Mais on sent que pareil.

*Mme. 2:* Ouais, mais on a essayé. Non, mais c'était même l'arnaque.

*M. [Prénom du conjoint]:* C'est à peine mûri, c'est à peine mûri.

*Mme. 2:* Je t'avais dit.

*Mme. 1:* Ouais.

*Mme. 2:* [Ah] Ce n'était pas bon, [ah].

*Mme. 1:* Ouais, c'est pris trop tôt.

*M. [Prénom du conjoint]:* Ce n'est pas- c'est des- des fruits--.

*Mme. 2:* C'est horrible, là.

*M. [Prénom du conjoint]:* Des fruits cueillis trop tôt.

*Mme. 1:* [Ah].

*M. [Prénom du conjoint]:* Pas de saveurs, [euh], pas juteux, pas sucré, [euh].

*Mme. 1:* Ouais.

*M. [Prénom du conjoint]:* Et puis oui, ça ne peut aller, [quoi]. Les trucs, ça fait rêver d'acheter ça, [quoi].

*Mme. 2:* Ouais, c'est ça. Non, non, tu as- tu as envie d'acheter, [quoi].

*Mme. 1:* Ouais.

*Mme. 2:* Oui, non, c'est ça. Ouais, c'est- c'est pauvre, [hein], en fibres et tout ça. Je trouve qu'en, [euh], ait pauvre--.

*M. [Prénom du conjoint]:* [Eh], [ben] on dirait, on aurait presque plus de plaisir à acheter de la salade de fruits en boîte et--

*Mme. 2:* [Eh], |ben] C'est ça.

*Mme. 1:* Ouais.

*M. [Prénom du conjoint]:* Et en manger, [quoi].

*Mme. 2:* Comme dans l'hiver, [quoi].

*M. [Prénom du conjoint]:* Ouais.

*Mme. 2:* Ouais, c'est ça, ouais.

*Mme. 1:* Et, [euh], quand- quand vous êtes dans les grandes surfaces, est-ce que c'est un moment--.

*Mme. 2:* Ouais.

*Mme. 1:* Que-, [euh], que vous appréciez de faire des courses ? Ou c'est--.

*Mme. 2:* Pour moi, c'est assez lourd comme caddies parce que c'est les eaux que je prends.

*Mme. 1:* Ouais, ouais.

*Mme. 2:* C'est tout, voilà, qui dit grandes surfaces, dit les packs d'eau.

*M. [Prénom du conjoint]:* Ouais.

*Mme. 1:* Ouais.

*Mme. 2:* Ou alors les couches.

*M. [Prénom du conjoint]:* Mm.

*Mme. 1:* Ouais.

*M. [Prénom du conjoint]:* C'est presque, c'est quasiment tous les jours. Comme on disait, on n'a rien pour conserver.

*Mme. 2:* Non, ça dépend et en vrai, il me manque toujours un petit truc, [quoi], c'est ça.

*M. [Prénom du conjoint]:* On ne pas- on fasse les ventes, il y a dans la semaine, on doit y aller au moins trois ou quatre fois aux courses.

*Mme. 2:* Ça, c'est toi quand tu anticipes très mal.

*M. [Prénom du conjoint]:* Oui, ça, c'est vrai, ça, c'est vrai.

*Mme. 2:* Je suis désolée, mais moi je suis plus efficace.

*M. [Prénom du conjoint]:* Moi, je consomme les AAA, ça c'est clair.

*Mme. 2:* Mm.

*Mme. 1:* Ouais.

*M. [Prénom du conjoint]:* Ouais, elle est- en principe, [euh]--.

*Mme. 2:* Non, je suis plus efficace dans ce- je- j'anticipe.

*M. [Prénom du conjoint]:* C'est- c'est un peu, [euh].

*Mme. 2:* Parce que moi, je stocke, [en fait].

*M. [Prénom du conjoint]:* Elle, c'est dans le rôle de la ménagère et--.

*Mme. 2:* C'est ça, moi, je stocke énormément.

*M. [Prénom du conjoint]:* Et moi, je suis dans le suppléant pendant le week-end, [hein], ça c'est--.

*Mme. 2:* Et tu me rachètes des pâtes alors j'en ai 36 000 tonnes.

*M. [Prénom du conjoint]:* [Eh], [ben), ouais, mais écoutes, de toute façon, c'est jamais en trop, [hein].

*Mme. 2:* Ouais, je sais, mais bon.

*Mme. 1:* Oui, c'est des produits qui ne se bannissent pas.

*Mme. 2:* Voilà, ce sera consommer de toute façon.

*Mme. 2:* Mais il y a moins de gestion du stock que, [euh], chez, [eh], [ben], chez que--.

*Mme. 2:* Ouais, c'est ça, ouais, je pense, [quoi].

*M. [Prénom du conjoint]:* Et des fois, comme celle qui organise les placards à nourriture, elle sait où se trouve.

*Mme. 2:* Ouais, ouais, c'est ça.

*M. [Prénom du conjoint]:* Moi, je ne trouve plus le riz ou les pâtes, [eh], [ben], j'irai acheter du riz et des pâtes et quand je reviens, elle me dit, [oh], on en a déjà plein.

*Mme. 2:* Non, mais je vais mettre des panneaux clignotants, je te l'ai dit, là.

*M. [Prénom du conjoint]:* Ouais, c'est ça, je te les réclame.

*Mme. 1:* Donc, [euh], les courses, ce n'est pas forcément moments de plaisir, c'est, [euh]--.

*Mme. 2:* Non, c'est la corvée pour moi.

*Mme. 1:* C'est la corvée.

*Mme. 2:* C'est ça, ouais.

*M. [Prénom du conjoint]:* Mais du coup, c'est rodé, [quoi], c'est expédié, [hein].

*Mme. 2:* Ouais, [ah], ouais, ouais, c'est ça, ouais, ouais, ouais.

*Mme. 1:* Donc, vous prenez, [euh]--.

*Mme. 2:* Souvent conditionné par les promos, je vous avouerais, pour les eaux.

*Mme. 1:* Ouais.

*Mme. 2:* Parce que je me dis, [euh], [eh], [ben], tiens, c'est trois packs pour le prix de deux, [eh], [ben], allez, je fonce dans les--.

*M. [Prénom du conjoint]:* [Eh], [ben] [en fait], c'est des- c'est sites de pro, [hein].

*M. [Prénom du conjoint]:* Ouais.

*Mme. 2:* Et je vois qu'il y a des cycles comme ça, [euh] et ça fait que, [euh], quand je prends un- une promo, ça va ve- aller jusqu'à l'autre promo de l'autre, [euh] grande surface et- et sur--.

*Mme. 2:* Ouais.

*M. [Prénom du conjoint]:* Ouais et, [en fait], il y a plusieurs magasins ciblés dans les cycles.

*Mme. 2:* Ouais.

*Mme. 1:* D'accord.

*M. [Prénom du conjoint]:* Donc, on a Intermarché, Leclerc, [euh].

*Mme. 2:* C'est ça.

*M. [Prénom du conjoint]:* Casino et puis, [euh]--.

*Mme. 2:* Mm, ouais.

*M. [Prénom du conjoint]:* Voilà, on passe notre temps à suivre les cycles.

*Mme. 1:* Et du coup vous changez de grande surface en fonction, [euh] ?

*Mme. 2:* Ouais, c'est ça, ouais, pour les eaux et surtout les eaux, les couches et les eaux.

*M. [Prénom du conjoint]:* On n'a- on n'est- on n'a pas forcément de fidélité. Après bon, il y a Leclerc, ici, [euh], c'est celui qui est le plus proche.

*Mme. 1:* Ouais.

*M. [Prénom du conjoint]:* Et c'est là où on peut dire c'est un--.

*Mme. 2:* [Ah] Alors là, il n'est pas terrible.

*M. [Prénom du conjoint]:* Un choix, on va dire moyen, plus. Il y a de la- il y a un peu près de tout, [euh].

*Mme. 2:* Ouais, [bof].

*Mme. 1:* Ouais.

*M. [Prénom du conjoint]:* Mais c'est décevant, les stocks, ils ne sont pas renouvelés, [hein], parce que moi, j'ai--.

*M. [Prénom du conjoint]:* [Ah] Ouais ?

*Mme. 2:* J'ai- j'ai certains produits ciblés dans le bio.

*M. [Prénom du conjoint]:* Alors leurs produits, les produits frais, là que ça s'est amélioré.

*Mme. 2:* [Ah] Ouais, [ah].

*M. [Prénom du conjoint]:* Ils font un stock de bio, ils font du local, ils font--

*Mme. 1:* C'est ce que j'allais dire quand je finis le- le stock, je sais que pendant un mois, il n'y en aura plus [hein].

*Mme. 1:* Alors, sur certains produits, c'est vrai.

*Mme. 1:* On voit qu'il y a qu'une feuille- une rose qui a fondue dedans.

*M. [Prénom du conjoint]:* Le lait maternisé par exemple.

*Mme. 1:* Ouais.

*M. [Prénom du conjoint]:* Ils ont très peu de réapro dans la gamme Compromis.

*Mme. 1:* D'accord. Des fois, vous n'avez plus rien.

*M. [Prénom du conjoint]:* On prend trois et puis il faut attendre un mois pour qu'ils renouvellent.

*Mme. 1:* Ah ouais, c'est une catastrophe. Ah oui, lui-même, des fois il supprime carrément le- le- l'article. En ce moment, il y a des trucs qui sont supprimés.

*M. [Prénom du conjoint]:* Mais c'est ça qui est particulier. Mais après, il y a des périodes où quand il y a des- des blocus, des grèves, ils ont du mal à se réapprovisionner.

*Mme. 1:* C'est ça aussi, le lait.

*M. [Prénom du conjoint]:* Et là, c'est [euh] que des- des matières premières, c'est le- les transporteurs qui conduisent plus. Voilà. Et puis on- on meurt pas de faim quand même.

*Mme. 2:* Oui, il faut adapter. Il faut adapter les coûts quoi. Et donc [euh] la corvée du- enfin, est-ce que- selon vous, est-ce que vous prenez plus de plaisir à vous rendre par exemple au marché ou le soir chez le producteur ? Ou ça reste quand même une corvée [euh] ?

*Mme. 1:* Non, non, je suis plus fan du producteur. C'est- c'est là que ça va me donner des idées beaucoup plus, parce que [ben] je sais que déjà c'est savoureux. Je me dis : Ah (bah] tiens, elle a des betteraves cuites aujourd'hui. [Ben] Allez, j'en prends pour ce soir.

*M. [Prénom du conjoint]:* On fait malgré tout des- des bonnes affaires. Puis c'est- c'est une personne avec qui on va parler, alors que on va faire nos courses.

*Mme. 1:* Oui, oui, c'est sûr.

*M. [Prénom du conjoint]:* On dit bonjour à la caissière, on lui dit au revoir et puis c'est tout quoi.

*Mme. 2:* Ouais.

*M. [Prénom du conjoint]:* Et [euh] et la personne à qui on va parler là, chez le producteur, on peut lui demander--

*Mme. 1:* Enfin, même si on raconte pas notre vie. Moi, je suis assez efficace là-dessus. Je raconte pas ma vie non plus. Mais c'est ça. C'est quand tu dis--

*M. [Prénom du conjoint]:* Mm-hmm.

*Mme. 1:* On échange aussi [euh] sur leur agriculture. Comment ça s'est passé ? Parce que cette année, ils m'ont dit les patates, [euh] mettez-moi les- les quantités que vous voulez de bonheur parce que j'en ai eu pas eu des tonnes.

*Mme. 2:* Ouais.

*Mme. 1:* Donc voilà. C'est aussi échanger sur des petits trucs qui sont sympas à comprendre quoi.

*Mme. 2:* Donc ouais, sur le- comment s'est passée la- la- ?

*Mme. 1:* Ouais, le site de production.

*Mme. 2:* Ouais, le site de production. Et puis il y a aussi peut-être des idées- enfin, sur les quantités, tout ça.

*Mme. 1:* Ouais, ouais, c'est ça.

*Mme. 2:* Des conseils quoi sur--

*Mme. 1:* Ouais, ouais.

*M. [Prénom du conjoint]:* Je vais pas dire les références, mais les [euh] types de fruits et légumes qu'on peut s'approvisionner.

*Mme. 1:* Ouais. Tout à l'heure, tu parlais de job d'été, même mine de rien, on en a parlé avec Monsieur [Nom de famille] et il m'a dit : [Ben] Si vous avez un jeune qui a plus de seize ans, je le prends.

*Mme. 2:* Ouais, ouais.

*Mme. 1:* Pour déconner là-dessus, je me dis mais ça peut faire des sous aux enfants. C'est bien.

*Mme. 2:* Ouais, ouais.

*M. [Prénom du conjoint]:* Pour apprendre ce que le travail, c'est pas mal.

*Mme. 2:* Ouais.

*Mme. 1:* Ouais.

*Mme. 2:* Oui, là, c'est-- Ouais, ouais ouais. Donc [euh] oui, vous préférez quand même vous approvisionnez chez le producteur que dans la base- en grande surface.

*Mme. 1:* Clairement, ouais.

*Mme. 2:* Et [euh] et ouais, vous disiez que ça vous donnait même des idées dès que vous arrivez [euh]

*Mme. 1:* Ouais. C'est ça, ouais.

*Mme. 2:* Devant les produits.

*Mme. 1:* Je vais pas penser. Dans les choux, il y a plein de choux différents en ce moment, donc ouais.

*Mme. 2:* Ok. Est-ce que ça vous arrive de perdre des produits de grande surface ?

*Mme. 1:* Ouais, beaucoup plus je trouve. Dans les bananes qui--

*M. [Prénom du conjoint]:* [Ben] Ouais, on a parlé des bananes. Une fois, les bananes, dans les--

*Mme. 1:* Moi dernièrement, je vais faire du citron parce qu'ils ont pourri. Je les ai acheté il y a quelques jours, je peux te les montrer, je les ai balancé, mais il était déjà [Diaphonie 00:26:15-00:26:16] Ouais. Ça pue à trois kilomètres, deux citrons qui étaient en contact. Ça y est, ils ont développé des espèces de truc vert-là.

*Mme. 2:* Ouais.

*M. [Prénom du conjoint]:* [EUh] Il y avait pas des carottes qu'on avait jetées là dernièrement ?

*Mme. 1:* Ouais. Mais alors je les ai épluchées et elles ont pourri tout de suite.

*M. [Prénom du conjoint]:* Ouais. Bon, enfin tout de suite.

*Mme. 1:* Bon, tout de suite.

*M. [Prénom du conjoint]:* Je crois que elles ont été oubliées sous une casserole. [Rire]

*Mme. 1:* C'est ça.

*M. [Prénom du conjoint]:* Elle fait des expériences de--

*Mme. 2:* Ouais, ouais, des fois, ça--

*Mme. 1:* Tu sais, ces- ces fameuses carottes bios que t'achètes des fois en sachet.

*M. [Prénom du conjoint]:* Ouais, en sachet.

*Mme. 1:* Et j'ai l'impression que celles-là, eh [ben] elles- elles pourrissent beaucoup plus vite.

*M. [Prénom du conjoint]:* Ouais.

*Mme. 1:* Que celles que je vais acheter chez le producteur.

*M. [Prénom du conjoint]:* Ouais. Mais en fait, elles sont emballées sous azote.

*Mme. 1:* Ouais, c'est ça.

*M. [Prénom du conjoint]:* Et quand on les déballe, elles prennent contact avec l'oxygène et là, je crois que c'est- c'est --

*M. [Prénom du conjoint]:* C'est explosif. Et j'ai vu que le pourrissement de ça, quand je les épluche parce que je me dis : [Ben] Tiens, je vais faire- j'oublie- enfin, je passe à autre chose, eh [ben] ça va beaucoup plus vite.

*Mme. 2:* Ouais. Bizarre. Bon. Est-ce qu'il y a quelque chose que les gens remarquent assez- que de conservation du produit (euh] pour les supermarchés, c'est perdu un peu.

*Mme. 1:* Mm-hmm.

*Mme. 2:* Et citrons reviennent aussi.

*Mme. 1:* Ah ouais. Citron et orange aussi. Là, tout à l'heure, j'avais citron et orange. Le duo, [euh] je le sentais à trois kilomètres.

*M. [Prénom du conjoint]:* [Euh] L'orange ouais, mais on les mange quand même.

*Mme. 1:* Il y en avait qu'une. Mais ouais, il y en avait qu'une. Elle était à côté des citrons. Alors, peut-être que ça a transmis [euh] le virus. [Rire]

*M. [Prénom du conjoint]:* C'est- ouais.

*Mme. 2:* Et quand vous perdez un produit du supermarché, qu'est-ce que- qu'est-ce que vous éprouvez à ce moment-là ?

*Mme. 1:* [Ben] De la déception, parce que c'est quand même acheté cher aussi, mine de rien. C'est- c'est de l'argent fichu quoi. Moi, après je le mets aux composts. De toute façon, je veux pas les manger, mais je les mets au compost quoi. Voilà, c'est- c'est la consolation. C'est que j'ai un potager après qui attend que le compost. Donc il y en a-- C'est un peu cyclique, ça rev- ça revient à la terre si vous voulez.

*M. [Prénom du conjoint]:* Sur les marchés ou pas, c'est toujours [euh] voilà.

*Mme. 1:* Ouais, c'est ça. Après [euh] que l'homme n'y- n'y passe pas dans ce cycle, c'est quand même le comble quoi.

*Mme. 2:* Oui, oui, ça a été produit à la base pour eux.

*Mme. 1:* Ouais.

*Mme. 2:* Ok. Alors, dernière photo.

*Mme. 1:* Un potager je pense. Il est très classe. Regarde. Inspire-toi parce que là-- [Rire]

*Mme. 2:* On est sur un potager de compét.

*Mme. 1:* Ah ! Parce que là, le nôtre, c'est vraiment le bordel. [Rire] Tu vois là, j'adore ce truc très carré. J'ai une copine, c'était pareil. Elle a de petites allées, avec des petits gravillons. Je sais pas comment--

*M. [Prénom du conjoint]:* Ça c'est- ça, c'est- à mon avis, c'est cultivé en ville, ça.

*Mme. 1:* [Ben] Oui.

*M. [Prénom du conjoint]:* Regarde bien la photo.

*Mme. 1:* Enfin.

*M. [Prénom du conjoint]:* Non, même pas.

*Mme. 1:* Chez peut-être des personnes âgées, mais des personnes soigneuses au moins.

*M. [Prénom du conjoint]:* Si, il y a un muret. Il y a un muret.

*Mme. 1:* Alors, [ben] nous [euh] potager, [ben] oui.

*M. [Prénom du conjoint]:* Ça me fait presque penser à la culture hors sol.

*Mme. 1:* Non, non, pas du tout, non.

*M. [Prénom du conjoint]:* En tout cas, c'est bien tenu.

*Mme. 1:* J'ai une copine qui avait la même chose. Et là, elle avait dans mes âges.

*M. [Prénom du conjoint]:* Du coup, c'est [euh]

*Mme. 1:* En tout cas, elle avait plus de temps peut-être.

*M. [Prénom du conjoint]:* Relatif à produire sa propre nourriture.

*Mme. 1:* Ouais.

*M. [Prénom du conjoint]:* [Ben] Nous, on est adepte. On fait--

*Mme. 1:* On le fait tous les ans. Alors par contre, c'est sûr qu'on limite parce que moi j'ai trouvé des lapins de garenne dans le jardin. Et j'ai compris pourquoi j'avais pas de haricots verts.

*M. [Prénom du conjoint]:* C'est que la- la première fois où on s'est installés ici, on a fait- la première année, on a planté des patates. On a eu 300 kilos et on en a eu pour six mois. On était contents. La deuxième année, j'ai récolté ce que j'ai planté.

*Mme. 1:* Rien.

*Mme. 2:* Donc nul.

*M. [Prénom du conjoint]:* C'est-à-dire [euh] minable quoi.

*Mme. 2:* Ah ouais.

*Mme. 1:* Ouais, c'est ça.

*M. [Prénom du conjoint]:* Je faisais de toutes petites patates. Je plante des petites patates grosses comme des gâteaux. Et [euh] et après, quand je récolte, je- je retrouve ces patates-là [euh] qui en fait j'étais- elles étaient remplacées par d'autres.

*Mme. 2:* Ok.

*M. [Prénom du conjoint]:* Mais [euh] pas- je vais pas éplucher ça. [Euh] Nous, on perd beaucoup de temps à les éplucher. Alors, nous on pratique ça, mais à condition que on- on a--

*Mme. 1:* Déjà d'avoir une surface raisonnable.

*M. [Prénom du conjoint]:* Voilà.

*Mme. 1:* Parce que maintenant, avec de la- avec deux longueurs d'avance, je dirais que la surface interminable, c'est fini. Ouais.

*M. [Prénom du conjoint]:* Ce qu'il y a, c'est que on a le temps de- on a le temps de planter. On a le temps- on trouve le temps de récolter, mais on n'a pas le temps de faire les entretiens entre les deux quoi. Genre buter les patates, [euh] couper les gourmands et tout ça. Nous, il faut qu'on fasse des choses qui poussent tout seul et qu'on ramasse. C'est pour qu'on a courgette, courge.

*Mme. 1:* Courgettes, courges.

*M. [Prénom du conjoint]:* [Euh] Salades.

*Mme. 1:* Ouais, salades. Ça, c'est vrai. Les laitues.

*M. [Prénom du conjoint]:* Après, on a- on a fraises, cassis.

*Mme. 1:* Ouais, ouais, on a des framboisiers. Ouais, on a des framboisiers, ouais, c'est ça.

*M. [Prénom du conjoint]:* Pommiers.

*Mme. 1:* C'est vrai qu'on a eu pas mal--

*M. [Prénom du conjoint]:* On a des œufs.

*Mme. 2:* Ouais. Donc voilà.

*M. [Prénom du conjoint]:* Et je crois qu'on a- on a tenté les échalotes et les betteraves. Mais c'était pas convaincant.

*Mme. 1:* Oh oui. Ouais, les échalotes, elles étai- elles étaient belles, mais c'est des échalotes de jardin. Donc c'est pas des gros trucs de compétition.

*M. [Prénom du conjoint]:* C'est ça. Après nous, comme on n'utilise pas d'engrais, rien. On- on a la terre qu'on a, on ne fait pas d'efforts en fait.

*Mme. 2:* Ouais.

*M. [Prénom du conjoint]:* On ne fait pas d'effort. On- on a- avec les--

*Mme. 1:* [EUh] Si, betterave. Betterave aussi, j'ai eu.

*M. [Prénom du conjoint]:* Oui, oui, on-

*Mme. 1:* Alors, tu vois- ah oui, betteraves parce que je fais des semis. Et alors, après les semis, c'est des tout petits trucs qu'il faut replanter. Là, c'est la chirurgie

*M. [Prénom du conjoint]:* C'est ça.

*Mme. 1:* Alors, j'ai dit aux enfants : Si vous voulez faire de la chirurgie, [euh] [Diaphonie 00:30:58-00:31:01]

*M. [Prénom du conjoint]:* Des- des rangées d'haricots, de petits pois, mais on ne les ramasse pas. Ça pousse, ils sont gros. Mais on n'a pas le temps de les ramasser. Quand on trouve le temps, ils sont déjà [euh] trop gros.

*Mme. 1:* Non, mais des fois, ça ne pousse pas du tout [Prénom du conjoint]. T'exagère.

*M. [Prénom du conjoint]:* Des fois, [bah] ça pousse pas parce que ils passent-- Les lapins sont passés par là.

*Mme. 2:* Ouais.

*Mme. 1:* Une horreur.

*M. [Prénom du conjoint]:* C'est la vie- c'est la vie, c'est la campagne.

*Mme. 2:* C'est ça.

*M. [Prénom du conjoint]:* Il faudrait qu'on plante des lapins.

*Mme. 1:* Non, mais tu sais, on a une petite serre qui sert à personne. Je te dis, on va mettre les haricots l'année prochaine là-dedans. Déjà, pour [euh] limiter les lapins.

*M. [Prénom du conjoint]:* On peut- on peut faire ça.

*Mme. 1:* Moi, je voudrais tenter- re- retenter le haricot parce que ça, c'est super cher en- en- dans le marché. Et puis bon, c'est pas--

*M. [Prénom du conjoint]:* Et il y aura ni oiseau, ni lapin.

*Mme. 1:* Ouais. Donc c'est ça.

*M. [Prénom du conjoint]:* Ma petite tomate. On a essayé les tomates, mais c'est trop humide.

*Mme. 1:* Les tomates cerises. Si, on a peut-être eu quelques années. Mais ça prend vite l'humide du haut aussi. C'est tellement humide, c'est ça comme tu dis.

*Mme. 2:* Ouais, ouais.

*Mme. 1:* Alors, les melons, j'ose même pas imaginer. Parce que moi, j'en avais plein dans le sud-là, vers Nantes. Mais ici.

*Mme. 2:* Le soleil.

*Mme. 1:* Ouais, il y a pas de soleil. Ici, les voisins, ils ont tout ça en serre donc--

*Mme. 2:* [Ben] Oui, oui. Et [euh] qu'est-ce qui fait que vous- vous appréciez quand même faire un potager [euh] quand vous pouvez ?

*Mme. 1:* [Ben] Le- le

*M. [Prénom du conjoint]:* C'est- c'est d'avoir sa-

*Mme. 1:* Le gain.

*M. [Prénom du conjoint]:* Le côté sur la qualité qu'on- qu'on y met.

*Mme. 1:* Ouais, ça c'est sûr. Mais le gain aussi de l'argent.

*M. [Prénom du conjoint]:* Voilà ! Ouais.

*M. [Prénom du conjoint]:* Pas spécialement. Non, c'est vrai qu'on n'est pas gagnant.

*M. [Prénom du conjoint]:* Tu sais, je pense que si on- si on payait au- au- à l'heure pour le jardinage, on serait clairement perdant. J'ai mieux fait d'aller brosser à--

*Mme. 1:* Non, mais c'est sûr. Regarde tes courgettes. Honnêtement, tu balances une graine, ça prend et ça te donne- incroyable ça. Là, honnêtement [euh] là, je pense que financièrement, t'es gagnant et qu'il y a pas grand-chose à faire comme- comme dépenses.

*M. [Prénom du conjoint]:* Si tu balances dans- dans la terre que j'ai retourné.

*Mme. 1:* Ouais [Rire].

*M. [Prénom du conjoint]:* Voilà. C'est ça la différence.

*Mme. 1:* Un petit peu mieux, oui.

*M. [Prénom du conjoint]:* Et puis retourner la terre, [ben] on- on le fait avec le coude quoi. J'ai une petite- une petite bineuse. Mais ouais, il faut-- Il y a du temps derrière.

*Mme. 2:* Oui.

*M. [Prénom du conjoint]:* Et ce temps-là, [euh] en fait, je- je vais me payer [euh] en étant au travail quoi.

*Mme. 2:* [Ben] Oui.

*M. [Prénom du conjoint]:* Que ce que me rapportent les légumes que je plante.

*Mme. 2:* [Ben] Oui. Du coup, [euh] après les acheter- enfin ça oui.

*M. [Prénom du conjoint]:* On- on maîtrise la qualité, on maîtrise l'approvisionnement. Et ça, pour nous, c'est important.

*Mme. 1:* Et puis vu la grosseur de nos courgettes.

*M. [Prénom du conjoint]:* Surtout avec les histoires de confinements là, [euh] courgettes en- en supermarché, [ben] nous, on a les deux trois.

*Mme. 1:* Et là, j'en ai encore pour vous dire. Ça nous maintient encore jusqu'à maintenant.

*Mme. 2:* Ok. Et est-ce qu'on sert [euh] ?

*Mme. 1:* Ah [ben] c'est impeccable, je pense. J'ai pas été les voir l'autre jour, mais j'en ai encore mangé une cette semaine.

*M. [Prénom du conjoint]:* Voilà. Des fois, si il y a un petit peu de perte. Mais--

*Mme. 2:* Ouais.

*M. [Prénom du conjoint]:* Mais on- on s'en veut de là par contre, plus que quand on les achète.

*Mme. 2:* Oui, vous avez plus de--

*M. [Prénom du conjoint]:* Plus de remords. Parce que-- [Ben] Ouais, c'est- c'est- on les a montré aux enfants, on les a planté avec les enfants, on a récolté avec les enfants. Et alors, on- sans les enfants.

*Mme. 2:* Sans les enfants. [Rire] [Diaphonie 00:33:42-00:33:43] Mais elles sont énormes.

*Mme. 1:* Mais encore, ça c'est des petites. Parce que je veux dire, celles que j'ai utilisées, c'était une qui faisait les deux en même temps.

*M. [Prénom du conjoint]:* Ouais. Ouais, c'était-

*Mme. 1:* Ça, c'est des trucs de compétition.

*Mme. 2:* C'est énorme. [Rire]

*M. [Prénom du conjoint]:* Ouais, c'est aussi--

*Mme. 2:* Et du coup, vous disiez que ça vous embêtait plus de les jeter [euh] du fait d'avoir vu tout le processus du--

*M. [Prénom du conjoint]:* C'est ça. Puis c'est- on- on jette de la qualité. Là, on est sûr. Elles sont arrosées à la rosée du matin tous les jours.

*Mme. 1:* T'as vu nos courges aussi, [euh] qui sont marr- qui sont toutes grises là. Elles sont énormes aussi. On a des gros trucs de compétition. Ça, c'est bien parce que pour les soupes, moi, ça va vite. [Rire]

*Mme. 2:* Donc ouais, vous jetez de la- quand vous jet- quand vous perdez des produits du jardin, [euh] c'est que vous perdez la qualité du produit.

*M. [Prénom du conjoint]:* C'est ça. Et on- on perd- on n'a pas utilisé [euh] le potentiel qu'on a en auto production sur ces--

*Mme. 2:* Ouais.

*Mme. 1:* C'est peut-être une courge qu'on a perdue en fin de se- saison, voilà. Qui--

*M. [Prénom du conjoint]:* Oui, oui, on en perd quand même un petit peu.

*Mme. 1:* Ouais. Un petit peu, ouais. C'est relatif quoi.

*M. [Prénom du conjoint]:* [Euh] On va dire [euh] une sur dix. Peut-être 10 pourcent.

*Mme. 2:* Ouais.

*Mme. 1:* Ouais.

*M. [Prénom du conjoint]:* C'est très très approximatif.

*Mme. 2:* Oui, oui. Oui, c'est- c'est pas- c'est rare quand même que vous en perdiez quoi. C'est vrai qu'on a beaucoup de récolte.

*M. [Prénom du conjoint]:* Des fois-- puis on-- des fois, on prend le fruit abîmé et quand on le stocke, il s'abîme encore plus. C'est surtout ceux-là en fait qu'on perd.

*Mme. 2:* [Ben] Oui, oui.

*M. [Prénom du conjoint]:* Ou alors, une- une chute de manutention, on l'abime et c'est- le pourrissement arrive.

*Mme. 2:* Mm-hmm. Ok. Alors, si on fait un autre petit jeu. Vous consommez de la banane ?

*M. [Prénom du conjoint]:* Oui.

*Mme. 1:* Oui.

*Mme. 2:* Vous m'avez dit. Je vais vous donner dix images de bananes et je vais vous demander de les ranger dans l'ordre de celles qui vous donnent le plus envie à celles qui vous veulent le moins envie.

*Mme. 1:* De votre côté--

*Mme. 2:* Non, vous pouvez les mettre de votre côté. Vous faites genre des rangées.

*Mme. 1:* D'accord. Ouais, ouais. Celles qui me donnent le plus envie. D'accord. Ok. Je vais me permettre de les étaler.

*M. [Prénom du conjoint]:* Oui, oui, allez-y.

*Mme. 2:* Celle pas du tout. Et celle-là aussi, je l'aime bien. Voilà. Non. Celle-là, je l'aime un peu plus. Celle-là, [bah] ouais. En fait ou [euh] c'est celle que je ne veux plus ou celle que je vais utiliser d'abord où ?

*Mme. 2:* Celles qui vous donnent le plus envie d'être consommée là tout de suite.

*Mme. 2:* Ah ouais. J'hésite entre les deux premières bananes. Mais c'est pas grave. Et celle-là, [bah] ça va être dans les dernières du coup. Laquelle je pourrais préférer ? Je sais pas trop en fait.

*M. [Prénom du conjoint]:* Elles sont noires.

*Mme. 1:* Oui, elles sont noires. [Bah] Ça, ça va être la- vraiment la dernière de dernière ça, ça va être avant celle-là, parce que j'ai l'impression que celle-là est plus écrabouillée que celle-là. À vrai dire, je préfère entre la verte ou celle-là, je préfère celle-là. Tu vois ? Et [euh] du coup [euh]

*Mme. 2:* Poursuivez dans l'autre sens.

*Mme. 1:* Voilà ! Ah ouais, pardon. Voilà ! Ça, c'est la dernière.

*Mme. 2:* C'est la dernière.

*Mme. 2:* Ouais, c'est ça. Alors, attendez. Voilà ! Ça va être dans ce sens-là. Moi, je le vois tout de suite. Pardon !

*M. [Prénom du conjoint]:* Là, tu vois, elle est- elle est pétrie ici là.

*Mme. 1:* Ouais. C'est ça. Celle-là, elle est trouvée- elle est vraiment à jeter. Maintenant, si tu la prends, elle est molle. Et du coup, tu la mets--

*M. [Prénom du conjoint]:* Ouais, faut la mettre au pourri.

*Mme. 1:* Tu la mets [euh]

*Mme. 2:* Je vais me mettre devant vous pour vous faire parler dessus. Je vais prendre en photo votre rangement.

*Mme. 1:* Ouais. [Inaudible 00:37:22-00:37:24] pas très.

*Mme. 2:* Juste pour avoir-- après pour savoir de quelle banane vous parlez.

*M. [Prénom du conjoint]:* Ah ouais, ouais, ouais, d'accord.

*Mme. 2:* Alors, est-ce que je les ai toutes ?

*Mme. 1:* Ah ouais, c'est intéressante comme jeu ça en fait.

*M. [Prénom du conjoint]:* Mais pourquoi t'as mis la verte ? Elle est pas trop pourrie la verte.

*Mme. 2:* C'est pas selon la maturité, selon l'envie de la- la croquer quoi.

*Mme. 1:* Ouais, c'est ça. Moi, celle qui me donne le plus envie, c'est celle-là.

*M. [Prénom du conjoint]:* Ah ouais, d'accord.

*Mme. 1:* Après, celle-là, c'est pour cuisiner. Celle-là, c'est après celle-ci. Et celle-ci quoi.

*Mme. 2:* Alors, si on prend justement la première et la- alors, j'ai mis des petites formes pour voir desquelles on parle. Si on prend la rond blanc, [euh] est-ce que vous pouvez me la décrire avec vos sens-là, si vous- si vous l'aviez devant vous là ?

*Mme. 1:* Ouais, je- je la sens bien. [Euh] Je la sens savoureuse, développée.

*M. [Prénom du conjoint]:* Et bien mûre quoi.

*Mme. 1:* Ouais, bien mûre. Avec un goût de banane quoi. Alors, après celle qui est à côté, elle est un tout petit peu plus avancée. J'aurais tendance à hésiter là avec les deux, mais celle-ci, j'aurais tendance à la cuisiner en smoothie ou quelque chose comme ça. Plutôt que de la croquer si vous voulez.

*Mme. 2:* OK.

*Mme. 1:* En banane flambée. Alors, ça c'est-- Oh làlà, avec du beurre et du sucre.

*M. [Prénom du conjoint]:* Quand elles sont bien mûres comme ça, en général, elles passent à la poêle. Et-

*Mme. 2:* Donc dès le carré bleu pour vous, là c'est [euh]

*Mme. 1:* C'est plutôt cuisson. Ou alors, mousse- smoothie, c'est pas cuit. C'est- c'est le broyer. Ouais. C'est ça.

*Mme. 2:* C'est mixer ou--

*Mme. 1:* Ouais, ouais.

*M. [Prénom du conjoint]:* Après [euh] le carré suivant aussi est consommable.

*Mme. 1:* Ouais, ces- ces deux-là aussi.

*M. [Prénom du conjoint]:* Celle-là est bien, mais elle peut- elle peut attendre avant celle-là.

*Mme. 1:* En fait, je les ai pas classés par rapport à-- comment je peux les-- Tu vois, c'est je leur ai amené au-dessus aussi celle-ci quoi.

*Mme. 2:* La carré orange, vous faites quoi avec ? Là- vous vous attendez ? Vous mangez cru ? Vous--

*Mme. 1:* Je pense que par rapport à celle-là, je vais attendre.

*Mme. 2:* Vous atte- là, le carré orange, vous mangez pas ?

*M. [Prénom du conjoint]:* Je la mangerais crue celle-là.

*Mme. 1:* Oui, je la mangerai. Mais j'attends par rapport à celle-ci. Celle-ci, je la mange la première. Et--

*Mme. 2:* D'accord.

*Mme. 1:* Ouais, celle-ci, elle peut encore attendre pour moi, je trouve. En saveur.

*Mme. 2:* Ok.

*Mme. 1:* Ouais.

*Mme. 2:* Et le--

*Mme. 1:* Par contre, celle-ci, je l'ai mise là. Mais c'est pour [euh] pour ne pas avoir- j'ai- j'ai mis selon comment je- ça, je le mettais en même temps que ces deux-là. Parce que celle-ci, j'aurais tendance à la cuisiner, pareil. Que celle-ci, je la- je- j'attendrais avant de la croquer [euh] par rapport à celle-ci.

*Mme. 2:* Et donc là, la rond orange vous, est-ce que vous êtes- enfin, est-ce que vous pourriez la manger crue ou non, celle-ci ?

*Mme. 1:* Ouais. Oui, oui, je peux la manger. Oui. Ah oui, oui, oui. Ainsi que la- la bleue d'ailleurs. Moi, ça me ferait pas peur.

*Mme. 2:* Ok. Et-

*Mme. 1:* Je pense que--

*Mme. 2:* Vos enfants- vos enfants la mangeraient ou--

*Mme. 1:* Moins- moins que celle-ci déjà.

*Mme. 2:* Ouais.

*Mme. 1:* C'est sûr. Ceci, elle est- elle est douteusement trop moins.

*M. [Prénom du conjoint]:* Il reste un petit peu jaune. Je pense que eux, ils la laisseraient pourrir. Ouais.

*Mme. 1:* Ouais, c'est ça.

*M. [Prénom du conjoint]:* Alors qu'ils savent pas qu'ils vont l'avoir dans le smoothie.

*Mme. 2:* [Rire] Chut ! On leur donne du pourri à manger tous les jours. Attends, je me suis trompée. Celle-ci est plus développée, je sais pas en fait.

*Mme. 2:* Celle-là est un peu plus verte.

*Mme. 1:* Ouais, ouais, elle est plus développ- elle est moins développée. Ouais.

*Mme. 2:* Et là, celle avec le triangle vert ?

*Mme. 1:* Celle-là qu'on commence à-- [Ben] Celle-ci commence à mûrir. Moi, j'attends encore désespérément, une semaine au moins je pense.

*Mme. 2:* Ouais.

*Mme. 1:* [Rire] Et alors, les deux autres d'après. Enfin, la série des autres, alors celle-là, je pense que-- je vois pas pourquoi j'ai mis au-dessus. Je me disais peut-être qu'elle est-- mais celle-là, je pense qu'elles sont à balancer ces deux-là. Ces deux-là.

*Mme. 2:* Oui, celle- le triangle blanc, vous- vous la cuisinez pas.

*Mme. 1:* Ouais, ouais. [Ben] Je sais pas, mais ça dépend encore de si je la prends et qu'elle est toute ramollo, (ben] là, il y a plus rien à en faire. Elle est trop pourrie quoi.

*Mme. 2:* Ouais.

*Mme. 1:* Ça, c'est pas évident à- à tester quoi. Sur un regard comme ça, j'ai du mal. Et celle-ci, [ben] j'attends quoi.

*Mme. 2:* Ouais.

*Mme. 1:* Là- là, j'attends au moins deux semaines. Elle est verte comme je sais pas quoi.

*Mme. 2:* D'accord. Et [euh] notamment, vous avez pas justement des apports nutritifs [euh] de la banane. Est-ce que selon vous, il y a un moment donné où- où la banane perd ses apports nutritifs ou- ou justement ne les a pas encore ? Enfin, comment vous pensez [euh] ?

*Mme. 1:* Ouais. Je pense que celles-ci, le- elles ont toutes des apports nutritifs je pense. En soi.

*Mme. 2:* Ouais.

*Mme. 1:* Mais c'est le goût quoi qui me dérange.

*Mme. 2:* Ouais.

*Mme. 1:* Donc c'est ça. Peut-être que-- [Prénom du conjoint], qu'est-ce que t'en penses ? C'est peut-être nut- plus nutritif en vert que en pourri comme ça.

*M. [Prénom du conjoint]:* Je sais pas.

*Mme. 1:* Mais [euh] honnêtement au goût, c'est-- [Diaphonie 00:41:20-00:41:27]

*M. [Prénom du conjoint]:* En général quand ils ramassent. J'ai pas confiance, parce que quand ils ramassent des fruits trop [euh] trop tôt, comme dans le cas des bananes, il est possible qu'ils mettent des- des antis mûrissement. Enfin, des produits de conservation.

*Mme. 1:* Ouais. Peut-être va savoir dans des gros containers. Ça peut être ajouté, on n'en sait rien. Ouais, c'est ça. Donc du coup, c'est sûr qu'on prend de la ba- la banane, il doit- il doit y avoir du potassium, c'est par rapport à ça quoi. C'est tout.

*Mme. 2:* Ok. Et [euh] quand vous l'achetez la banane, elle est- elle ressemble plutôt à laquelle ?

*Mme. 1:* Eh [ben] elle ressemble à ça quoi.

*M. [Prénom du conjoint]:* [Diaphonie 00:42:01-00:42:03]

*Mme. 1:* Celle-là ou celle-là ou celle-là. Enfin, jamais ça.

*M. [Prénom du conjoint]:* Ouais, c'est ça.

*Mme. 1:* Ou alors, ça va être rare quoi.

*M. [Prénom du conjoint]:* Rarement comme ça, mais--

*Mme. 2:* Au plus grand max ce triangle vert, sinon c'est plus vert.

*M. [Prénom du conjoint]:* C'est ça. C'est la saison des verts.

*Mme. 1:* Mais alors, si jamais j'en trouve des comme ça, forcément je fonce dans le tas.

*Mme. 2:* Ah oui, vous- si vous en avez, vous les prenez comme ça.

*Mme. 1:* Ouais. Parce que si j'en ai pas, [euh] qui- qui est en train de mûrir à la maison quoi. Je- si j'ai un besoin aussi, parce que je dois en prendre.

*Mme. 2:* Ouais, c'est ça. Oui, s'il y a plus de banane chez vous et que--

*Mme. 2:* Oui, c'est ça.

*Mme. 2:* Comme ça, ça va vous permettre de--

*Mme. 1:* Je gagne du temps quoi.

*Mme. 2:* Ok. Pas attendre quinze jours.

*Mme. 2:* Ouais, c'est ça. [Rire]

*Mme. 2:* Ok. Et est-ce que selon vous il y a un moment donné où la banane, elle peut être [euh] potentiellement dangereuse pour la santé ou pas ?

*Mme. 1:* Quand c'est vraiment pourri comme ça, dans les extrêmes.

*Mme. 2:* Le triangle blanc et--

*Mme. 1:* Ouais. Et rose.

*M. [Prénom du conjoint]:* Abon ? Rose, ça peut être dangereux ?

*Mme. 1:* [Bah] Quand c'est pourri, [euh] il y a que du pourri. C'est pas terrible.

*M. [Prénom du conjoint]:* C'est vrai que c'est ton père qui me disait que les extrémités des bananes, c'est- c'était plein de vers.

*Mme. 1:* Non. C'est moi qui te disais, parce que je l'ai su de mes parents, mais ouais c'est pareil.

*Mme. 2:* Ah ouais ?

*Mme. 1:* Ouais, ouais. Les petites queues de banane. Ouais.

*Mme. 2:* Donc là, à partir de ce moment-là, [euh] vous pensez que c'est potentiellement dangereux.

*Mme. 1:* Non, ces deux-là, je- ouais. Mais celle-là aussi, je la mangerai pas en fait, la verte. Je pense que vert aussi, c'est pas bon. Les patates vertes par exemple, eh [ben] il faut les détourer énormément parce que même nos voisins nous ont dit que les patates vertes sont toxiques.

*Mme. 2:* D'accord.

*Mme. 1:* Donc banane verte pour moi, c'est pas--

*M. [Prénom du conjoint]:* C'est toxique à ce moment-là ?

*Mme. 1:* Ouais, je- je suis même pas sûre que ce soit digeste une banane verte.

*Mme. 2:* Oui. Oui. Selon vous, là, ça commence vraiment à être pas bon. Tout est question de digestion.

*Mme. 1:* Ouais, ouais.

*Mme. 2:* Ok. Alors, on fait un dernier petit jeu. Un petit jeu [rire].

*Mme. 1:* Très bien.

*Mme. 2:* Alors.

*Mme. 1:* Olàlà, il y en a des trucs.

*Mme. 2:* Alors, là j'ai des photos.

*Mme. 1:* C'est plus au propre maintenant.

*Mme. 2:* Bon. Elles sont plastifiées exprès. C'est des- [euh] en fait, c'est ce qu'on peut faire avec des- des fruits ou des légumes, notamment quand ils commencent à s'abîmer ou pas. Enfin, il y a tout bon. Et l'idée, c'est que vous choisissez les photographies qui vous parlent. Par exemple--

*Mme. 1:* Ouais, d'accord. Ça, c'est compost.

*Mme. 2:* Du coup, ça, non. Du coup, vous qui compostez, ça, c'est pas un domaine qui vous ressemble.

*Mme. 1:* Ouais, non.

*Mme. 2:* Donc ça, vous l'enlevez. Salade de crudités.

*Mme. 1:* Ça, c'est déjà cuisiné quoi. Qu'est-ce que je fais ? Alors, ça c'est--

*Mme. 2:* Oui. C'est ce que vous- est-ce que ça vous arrive de faire des- des salades de crudité ?

*Mme. 1:* Oui.

*Mme. 2:* Donc ça, vous gardez.

*Mme. 1:* Ça, je le garde.

*Mme. 2:* Des soupes.

*Mme. 1:* Là, c'est des conserves. Ouais, je garde.

*M. [Prénom du conjoint]:* On ne fait pas tant que ça.

*Mme. 1:* Non. On a fait des petits pois il y a quelques années quand on en a eu.

*Mme. 2:* Oui.

*Mme. 1:* Ça, c'est une espèce de compote, c'est ça ?

*Mme. 2:* Ouais.

*M. [Prénom du conjoint]:* Ça, c'est bon.

*Mme. 1:* Alors oui, c'est très bon. Ça aussi, c'est très bon.

*Mme. 2:* Ça, c'est [euh] ouais des tas. Enfin, tous les gâteaux que vous pouvez faire avec.

*Mme. 1:* Ouais, c'est des wokes. Moi, je fais une patate sautée, des trucs comme ça.

*Mme. 2:* Des poêlés.

*Mme. 1:* Un smoothie.

*Mme. 2:* Un smoothie.

*M. [Prénom du conjoint]:* Smoothie.

*Mme. 1:* Les œufs, on a ça.

*Mme. 2:* C'est ce que- oui, ça va- les fruits et légumes.

*Mme. 1:* On va pas manger la poule. [Rire]

*Mme. 2:* Oui.

*Mme. 1:* Ça, c'est le compost.

*M. [Prénom du conjoint]:* On vous écoute. Pardon.

*Mme. 2:* Le compost, vous avez. Vous prenez. C'est ça.

*Mme. 1:* On met en compost.

*Mme. 2:* Et vous avez les poules aussi.

*Mme. 1:* Ah oui.

*Mme. 2:* En fait, après je vais vous demander de mettre des- des- des- voilà.

*M. [Prénom du conjoint]:* Donc on prend tout en fait.

*Mme. 2:* Oui, il y a pas mal de choses. [Rire] Oui, mais ça, du coup, c'est-- vous, vous mettez pas vos- vos épluchures dans une poubelle classique.

*Mme. 1:* Ah d'accord. Non, non, non, non, non, non.

*Mme. 2:* Vous mettez tout [euh]

*Mme. 1:* Non. Je- Moi, je croyais que c'était--

*Mme. 2:* C'est pour ça que je vous la mets pas.

*Mme. 1:* Ah oui d'accord. Parce que moi, je mets dans des bassines. Alors, le sien, c'est--

*Mme. 2:* Oui, oui. Pour les personnes qui n'ont pas de compost, du coup, [ben] c'est une poubelle classique.

*Mme. 1:* Donc ça, c'est pour recycler les déchets. D'accord.

*Mme. 2:* C'est ça. Et là, je vous mets des photos de fruits et légumes ou de parties de fruits et légumes que des personnes ont jetées. Et je vais vous demander de me dire ce que vous vous auriez fait. Est-ce que vous auriez--

*Mme. 1:* Ça, c'est-- Ouais, je jette. Donc je mets ça dans ma poubelle.

*Mme. 2:* C'est ça. Dans le compost ou dans les poules.

*Mme. 1:* Ça, je jette. Ah [bah] tiens, ouais je vais mettre ça dessus. Là.

*Mme. 2:* Ouais.

*Mme. 2:* Ah voilà. Ok. Ça c'est pour poule. Et ça, ça peut être pour poule aussi. Ma foi, les tomates. Les courgettes, [euh] voilà, il- il en reste un petit peu de courgettes pour picorer dedans. [Ben] Ça, orange, c'est acide. [Euh] Ça, on a beau leur mettre de la verdure, je veux bien mettre avec-- pour moi, je mets ça en compost. Je leur mets pas de verdure, parce que elles aiment pas ça.

*Mme. 2:* Et-- mais du coup, ça, vous jetez ? Vous- vous la consommez pas ce--

*Mme. 1:* Ah, elle est pas pourrie totalement.

*Mme. 2:* C'est ça en fait. L'idée, c'est-- là par exemple ça, quand vous vous voyez un--

*Mme. 1:* Ah ouais, ouais, ouais, d'accord. Je vois. J'ai pas tilté. Moi, j'étais un petit temps pourri quoi.

*Mme. 2:* Ouais. L'idée, c'est- vous, qu'est-ce que vous auriez fait ? Est-ce que vous voulez.

*Mme. 1:* Oh [ben] ça, je veux bien le cuisiner.

*M. [Prénom du conjoint]:* Le demi-citron, tu l'aurais pris ?

*Mme. 1:* Ouais, ouais, j'aurais pris le demi-citron. Parce que j'ai des cakes au citron. Et qu'un demi-citron, ça me suffit.

*Mme. 2:* Donc ça a été- ça aurait fait un gâteau par exemple.

*Mme. 1:* Ah oui, pardon. Ouais, c'est ça.

*Mme. 2:* C'est ça. Voilà.

*Mme. 1:* Oui, oui, d'accord.

*Mme. 2:* Là, c'est un gaspacho. On va dire.

*Mme. 1:* Alors attendez, ça, j'aurais fait un jus d'orange moi.

*Mme. 2:* [Euh] Un jus--

*Mme. 1:* Ou un gâteau, pareil.

*Mme. 2:* Ou un gâteau. On peut mettre aussi--

*Mme. 1:* Non, pas salade de fruits, gâteau. Non, je pense.

*M. [Prénom du conjoint]:* Ouais, ok.

*Mme. 1:* Salade, [euh] ça oui, avec une salade composée. Tiens, je veux mettre ça là.

*Mme. 2:* Oui, c'est--

*Mme. 1:* C'est super bon. En plus, ils adorent ça quoi.

*Mme. 2:* Celle-là, vous la- vous ne l'auriez pas jetée vous ?

*Mme. 1:* Moi, j'aurais pris ce qui est encore mangeable. C'est flétri-- à mon avis, elle s'est flétrie en surface. Peut-être que le cœur est encore récupérable.

*Mme. 2:* Ouais, vous auriez essayé quand même de la manger.

*Mme. 1:* Ouais. J'aurais essayé.

*M. [Prénom du conjoint]:* On va jou- on va jouer les bons samaritains. Ce serait passé à la compost.

*Mme. 1:* Et ça, je mettrais aux poules, parce que elle a l'air complètement flagada.

*Mme. 2:* Ouais.

*Mme. 1:* Ça [euh] [ben] c'est pareil. C'est mon- je- je pourrais récupérer ça, mais genre franchement, je- je vais perdre du temps.

*M. [Prénom du conjoint]:* Ouais, pizza ça.

*Mme. 1:* Ça dépend. Il y a un petit quart, c'est--

*M. [Prénom du conjoint]:* Non. C'est un bout de feuille dessus, c'est flétri. C'est à mon avis pas pourri. Moi, j'aurais mis pizza.

*Mme. 2:* [Euh] Alors, c'est- on prend- c'est plutôt cuit quoi. On l'a mise dans plat cuit.

*Mme. 1:* Il y a plus grand-chose à faire là-dessus. Franchement.

*M. [Prénom du conjoint]:* Ouais. Enfin, c'est que tu- tu enlèves le pourri, il reste plus rien. Donc ça va aux poules ça.

*Mme. 2:* Ouais, ça va aux poules ça. Là, c'est comme nos- nos carottes.

*M. [Prénom du conjoint]:* Non, c'est mieux ça.

*Mme. 1:* Ouais, c'est déjà mieux. Ouais, c'est vrai. Voilà, je détoure et je- je suis un peu comme le--

*Mme. 1:* Là, là cuit- cuit.

*Mme. 1:* Ah ouais. Ça aurait été comme celle-ci. Ça dépend ce que je vais faire.

*M. [Prénom du conjoint]:* Quand c'est flétri ou --

*Mme. 1:* Ah ouais !

*Mme. 2:* Ouais.

*Mme. 1:* Ce sera des tomates [euh]

*M. [Prénom du conjoint]:* Elle est- elle est verte là non ? C'est ça ? C'est quoi l'arnaque là-dedans ?

*Mme. 2:* Je sais pas. Je suis pas. Quelqu'un l'a mis--

*Mme. 1:* Moi, je sais pas. Je vais les toma--

*Mme. 2:* Ouais, c'est vrai.

*M. [Prénom du conjoint]:* Crudité. Et là, c'est ?

*Mme. 1:* [Ben] Là, il y a quelques salades. Ouais, ben, le cœur est bien là, je sais pas. Et ça, c'est pareil. Très bien.

*Mme. 2:* Ok. Alors, je vais prendre en photo votre rangement. Ne vous inquiétez pas, ça va prendre--

*Mme. 1:* Comme ça. Donc on voit à peu près tout.

*Mme. 2:* Ce que je vois. Est-ce que j'ai tout ?

*Mme. 1:* Ouais. On peut tout rétrécir.

*Mme. 2:* C'est moi qui ai mal cadré. Très bien, super. Merci. Et du coup, est-ce que vous pouvez me dire pourquoi [euh] quand ça commence à flétrir, vous- vous privilégiez le- le cuit ? Qu'est-ce qui fait que vous préférez mettre [euh] ?

*Mme. 1:* Parce qu'en fait, il y a pas grand-chose à récupérer pour une salade. Enfin, il y a aussi la quantité quoi.

*Mme. 2:* Ouais.

*Mme. 1:* [Euh] Alors du coup, [ben] je me dis : Tiens, si je fais des patates sautées, ouais, pourquoi pas ? Je vais mettre ça. Ou alors, oui on fait de la pizza avec-- ouais, c'est ça.

*Mme. 2:* Ouais. Et [euh] c'est pour ça que vous voulez-- enfin--

*Mme. 1:* Sinon, ça passe à la cuisson quoi, pour si peu de quantités.

*Mme. 2:* Ouais, ok. C'est que vous avez du coup transformé avec d'autres [euh] mélangés avec d'autres produits. Et dans les- et pourquoi le vers de poireaux ici, [euh] ne va pas chez les poules ?

*Mme. 1:* Parce que la verdure [euh] elles- elles accrochent pas.

*Mme. 2:* Vos poules ou c'est toutes les poules ? Enfin, je sais pas.

*Mme. 1:* Ah, toutes les poules.

*Mme. 2:* Ah ouais ?

*Mme. 1:* Ouais. Parce que comme elles gambadent dans- dans l'herbe, nos poules grattent toute l'année dans l'herbe comme [euh] enfin, des fois, je libère. En ce moment comme il fait pas beau, donc je lièvre. C'est que de la gadoue dans le- dans le poulailler. [Bah] Du coup, [euh] non, ça ils grattent pas dedans.

*Mme. 2:* OK. [Rire] [Diaphonie 00:49:33-00:49:35]

*Mme. 1:* Des réflexes. Ouais, c'est ça. Donc je me dis, bon avec le compost, ça va avec les épluchures de pommes de terre aussi.

*Mme. 2:* Oui. Les épluchures de pommes de terre, par exemple les poules, elles en mangent pas.

*Mme. 1:* Non.

*Mme. 2:* Non ? Non plus.

*Mme. 1:* Banane, elles adorent et [euh] ça, pêche aussi. Et [euh] courgette, je- des fois je mets, mais ça dépend de la quantité quoi.

*Mme. 2:* Ouais. Et (euh] est-ce qu'il y a des fois où--

*Mme. 1:* C'est entre les deux quoi.

*Mme. 2:* Ouais.

*Mme. 1:* J'aurais dû faire ça. Un truc au milieu, ça dépend. [Rire]

*Mme. 1:* Est-ce que des fois il y a des [euh] des fruits et légumes que vous trouvez trop abimés pour donner aux poules ou non ? Quand elles sont pourries, elles--

*Mme. 1:* Bon, ça c'est encore faisable, mais à voir quoi. En fait, je vais mettre dans un pou- un endroit du poulailler où ça va faire un simili compost quoi en fait.

*Mme. 2:* Ouais, d'accord. Ok. Et donc vous voyez pas de moment où là, vous dites : Non, non, ça c'est trop pourri pour-- Oui, elles vont se débrouiller toutes seules.

*Mme. 1:* Ouais. C'est pareil. Quand c'est trop- trop pourris, ouais [ben] là, dans-- une pomme toute pourrie, voilà je la balance dans le compost.

*Mme. 2:* Ouais. Vous la mettez pas--

*Mme. 1:* Si par exemple, les trois quarts encore mangeable, eh [ben] elles vont picorer dedans quoi.

*Mme. 2:* Ouais, ok, ça marche. Alors, j'en ai six autres. Donc là, vous auriez coupé les citrons.

*Mme. 1:* Ouais, ouais, c'est ça. Ouais. J'aurais fait un cake. Ouais. C'est ça. C'est pas une tarte, mais un cake- un cake.

*Mme. 2:* Oui, voilà une pâtisserie. Et là, tout ça, vous aure- vous auriez pu manger aussi cru ?

*Mme. 1:* Ouais, une salade ouais. Ils aiment bien l'endive comme ça mes enfants.

*Mme. 2:* Ok.

*Mme. 1:* Ouais. Une salade avec un [Inaudible 00:50:50-00:50:50] et tout. Ouais.

*Mme. 2:* Ok. [Ben] Ça marche. Merci beaucoup.

*Mme. 1:* Attendez, je vais vous remettre les trucs.

*Mme. 2:* Ouais, mais ne vous inquiétez pas.

**Household F39, Interview 1**

**Du coup, pour commencer, je vous propose un petit jeu de devinettes. Je vais vous demander de penser à un fruit ou à un légume que vous aimez tout particulièrement. L'idée, c'est que vous allez penser à ces fruits et légumes sans révéler son identité. Je vais devoir le deviner grâce à votre description.**

D'accord, ok.

**Est ce que vous en avez un en tête, un fruit ou un légume ?**

Fruits j’en ai deux, mais il faut que j’en choisisse un tout de suite là, c’est bon j’en ai choisi un ! Et il faut que je choisisse aussi un légume ?

**Non, c'est un des deux.**

J'ai choisi un fruit.

**OK, je vous écoute, dites-moi tout sur ce fruit.**

Il est de saison. Il est évidemment sucré parce que je ne sais pas s'il y a beaucoup de fruits non sucrés enfin si, si on joue dans les détails avec les tomates et les avocats et machin. Il est… j’ai pas envie de vous donner trop d'informations si ? il faut que je fasse durer le plus longtemps possible ?

**Vous me dites tout ce qui vous vient, de toute façon à la fin je vais l’avoir !**

Il y a un noyau. Ce qui est agaçant d’ailleurs, c'est quand on ouvre le fruit des fois le noyau s’ouvrent en 2 laissant apparaître l’amande en son cœur.

**Ok**…

Noyau strié… Le fruit existe de différentes couleurs. C’est difficile d'en trouver des bons, disons que j'ai le sentiment, mais ça, c'est une généralité que les fruits étaient vachement meilleurs il y a 20 ans ou il y a 30 ans. C'était plus facile c’est à dire, on était peut-être plus dans du fruit qui arrivait… Je suis en train de faire de l'histoire, ce n'est pas le sujet.

**Si si !**

Non non… On était peut-être dans des fruits qui venaient de producteurs de réel. Et alors que maintenant, ça vient de fermes en Espagne. On sait très bien, en plus avec le gel, cette année, c'est un voeux pieux que de croire qu'on va manger du fruit français, on est sûr de manger des fruits espagnols. Voilà, je regrette que ce fruit là n'existe pas toute l'année, mais pour autant, je ne suis pas prêt à le manger en boite. De toute façon, je trouve ça pécher de… De manger ce fruit là en dehors de la saison, sans jeu de mots. Tu as pas compris ?

**J’ai pas compris**

C’est pas grave

**Je réfléchis !**

On va dire que c’est le pollen *(rires)*

***(rires)* Ah si ! J’ai, j’ai ! Alors, qu'est-ce que vous aimez tout particulièrement dans ce fruit, mis à part son côté sucré ?**

Le fait que ce soit juteux.

**Ok… Juteux ?**

Il a un parfum d’une autre pareille quoi. Il n'y a pas de, il n' a pas de ressemblant, il n'a pas de frère jumeau. Il n'a pas de… dans sa famille, il est un peu seul je trouve. Il ressemble pas aux autres, car il a un parfum… Un parfum qui est estival, quoi. Et puis, il s'associe bien à d'autres fruits. Il appelle les copains dans une salade de fruits assez facilement. En fait, je le mange uniquement cru, jamais cuit, éventuellement compotée ouais éventuellement mais ça c’est quand le fruit est fatigué.

**Ok…**

Comme ce que j'ai fait avec des abricots tout à l'heure, j'en avais trois qui étaient un peu fatigués. Je me suis dis je vais quand même pas les offrir à la poubelle de Charlotte, non mais oh ! Un peu de sucres dans un… au micro-onde deux trois minutes et ça fait une petite confiture express. Euh voilà… Il faut que je remplisse encore du vide ? Que je dise autre chose ? Je peux dire, je peux dire que… Il est sensuel, ce fruit. De par la douceur de sa peau. En revanche, bon c'est énervant parce que quand il n’est pas mur c’est *(tape le point sur la table – pour signifier « dur »)* . C'est commun à tous les fruits, mais je dirais que je ne maîtrise pas trop sa maturation. Non pas que je maîtrise la maturation de quoique ce soit *(rires)* quand je vois la gueule de ma corbeille avec un gingembre qui germe avec un oignon qui pousse et les pommes qui survivent, elles survivent. Elles ont au moins trois semaines de corbeille ces pommes là mais elles sont bien, elles seront mangé à un moment donné, cuites ou crues. On ne sait pas encore.

***(rires)* En général vous le mangez quand ce fruit ? Plutôt matin, midi après midi…**

Franchement ? Tout le temps ! Il y a pas de règle parce qu’en fait j'ai vraiment… Il n'y a vraiment qu'en déplacement que je continue à prendre un petit déjeuner, car je ne me… je pars du principe qu'il faut que je mange quand j'ai faim. C'est mieux pour moi, d’ailleurs à tel point, ma petite de neuf ans et demi quand elle est chez moi, je la force plus à manger, je considère que si elle a pas faim, elle a pas faim, elle mangera mieux plus tard. Le matin, je vais prendre un café, vous voyez je me suis fait ma petite compotée d'abricot donc je prends trois, quatre cuillerées là dedans direct. Ca peut être un yaourt, ça peut être un oeuf sur le plat, juste ça donc ce fruit là je… Non non il y a pas de règles, je peux le manger à n'importe quel moment, et il est facile à emporter aussi. Même si ça coule sous les doigts, il est quand même plutôt facile à manger, plus facile à manger qu'un kiwi, par exemple. Parce kiwi, il y a deux écoles donner un coup de couteau et manger ça à la cuillère. Mais on risque d'arracher la peau et de s’en foutre partout, moi je préfère le faire à épluche légumes à la maison et tac tac tac et picorer après, alors que celui là, il se mange facilement comme une pomme en fait.

**Alors, est ce que vous pouvez me dire, justement, comment… vous avez votre fruit, c’est quoi les différentes étapes avant que vous puissiez le manger ?**

Je passe sous l’eau d'abord. Je le passe sous l'eau, parce que sa peau étant veloutée, j'ai l'impression qu'elle accroche plus les poussières, car il y a des fruits que je vais évidemment pas laver, la fraise, par exemple, je ne la lave pas. C'est peut être d’ailleurs le seul fruit que je ne lave pas. Les cerises, hier je les ai lavées. Si vous voulez des cerises d'ailleurs vous dites ! Donc, première étape, je passe sous l’eau. Ensuite, je la coupe en deux. Un coup de couteau qui fait le tour, « clac » j’ouvre le fruit, j’extrais le noyau. Et puis généralement, ce que je fais, je suis vraiment dans une grosse période salade de fruits depuis quelques temps, donc j’ai tendance à couper et mettre ça dans un bol avec d'autres fruits, d’autres fruits copains. Mais sinon le demi-fruit, je gobe, comme [Prénom], mon goéland.

***(rires)* OK, ça marche en général, vous l’achetez où le plus souvent ?**

Le plus souvent, alors, ce fruit-là comme les autres, le plus souvent c'est au marché, car j'ai un marchand qui… je vais au marché de Saint Louis le dimanche matin quand je suis Brestois en semaine et si je n'ai pas fait le marché Saint-Louis, je peux aller au marché amirale lillois parce que mon marchand de fruits et légumes est également là bas. En deuxième situation, ça va être les commerces de proximité spécialisés comme Siam Primeurs ou la Corbeille d'or, même si je trouve que ce sont tous deux des voleurs. Quand je vois le prix pratiqué, bon, mais parce que je le veux bien, mais c'est quand même abusé par rapport aux prix constatés ailleurs. Et puis, il y a dans fruits, en fait c’est comme tout produit vivant, il n'y a pas de règles, hier midi, sur la nappe à la plage, il y avait deux tupperwares de melons, un qui venait de chez Leclerc, un qui venait de chez un producteur, je ne sais plus quoi. Leclerc était fameux. On n'a pas. Il n’y a pas de règle. On ne peut pas savoir à l'avance et d'ailleurs je trouve qu’il y a des choses à … Je sais pas si vous avez entendu à propos du différents variants du Covid que des chiens ont été dressés et peuvent détecter, tout comme les chiens savent détecter les cancers très, très tôt. Pourquoi est ce qu'on n'a pas un chien au marché qui va dire que ce melon là, il est bon ? Celui là, il n'est pas bon. Je suis sûr, ça, mais techniquement, c'est faisable. Et là, je rêve. J'achète des fois des avocats en barquette et ça me fait… J’ai un peu mal au bide de le faire parce que l'avocat n'appelle pas de suremballage, mais dans la barquette où ils sont tous les deux logés, neuf fois sur.. Enfin pratiquement tout le temps, ils sont parfaits, ils sont parfaits. Est ce que ça tient au fait qu’il y ait des petits trous, ils respirent, ils sont pas choqués… Vous êtes en train de vous foutre de moi là ?

**Non non *(rires)***

Si si !

**Non non je vois très bien ces deux avocats en barquette !**

Et donc, j'aimerais bien qu'on ait des fruits un peu garantis bons à manger maintenant et avec une sucrosité parfaite. Mais bon, c'est peut être… ça enlève au charme et au naturel de la chose.

Ouais… Je vois

Non parce que c'est quand même vachement… C'est presque humiliant, vexant. On a l’impression de s’être fait baisée la gueule quand on achète un melon et qu'il a un goût de navet. C’est trompeur !

**Mmh. Je vois.**

C’est comme si je vous sers un café et qu’il a un goût de thé. Non, c’est pas ce que j'ai demandé.

**Et euh… sur ce fruit, donc quand il est bon du coup. Qu'est ce que vous ressentez quand vous le mangez ? Est-ce que vous avez, le sentiment qu’il vous apporte des choses… ? Qu'est ce que vous ressentez ?**

Les portes du soleil. Parce que c'est un fruit d'été. La priorité, c'est ça. Il me désaltère puisqu'il est juteux, généralement. Il m’apporte de l'agacement lorsqu'il est pas mûr. Là je m'en veux aussi parce que je me dis tu avais qu’à attendre ou le faire mûrir mieux. J'ai expérimenté un truc hier, je savais qu'il y avait deux avocats dans le bac à bières. Vous savez, il y a des gens qui mettent des fruits dans les bacs à bières du frigo ! Je les ai ouvert, ils étaient là depuis au moins 3 semaines. Alors en couleurs ils étaient parfait, en revanche bah ils étaiant dur. Ils étaient durs parce que par peur qu'ils mûrissent trop vite, je les ai mis au frigo et en fait ils se sont figés. Ça avait le goût de ce que c'était. Mais c’était pas wahou non plus. Je les ai mis en salade avec des tomates cerises, des échalotes, du vinaigre balsamique blanc, un filet d'huile d'olive et du poivre, et du sel. Et à la plage, c'était bien quand même. Ça faisait le job.

Ouais j'imagine ! Et du coup, à part le fait que des fois c’est pas facile de savoir quand est-ce qu’il est mur ou pas, est-ce qu’il y a d'autres inconvénients à ce fruit ?

Ah oui, il est fragile, il est fragile. Si on n'y prend pas garde, il va pourrir assez rapidement, donc faut gérer le stock en fonction de la consommation, sans tout stocker au frigo, mais c'est quand même une nécessité. J'avais fait le point hier soir, j’ai regardé cette semaine en fait, il n'y a pas un seul moment où je vais manger chez moi cette semaine, ni midi ni soir, parce que un dej à Quimper ce midi, parce qu'une réunion ce soir, parce que des trucs tout le temps qui font que ou d'apéros avec des potes qui font que du coup, je n'ai pas fait… Je n'ai pas fait le plein du frigo, il commencent à raisonner, ce qui est plutôt bien, comme ça ça va me permettre de le nettoyer. J'ai rien fait. J'avais complètement oublié que vous alliez inspecter mon.. Horreur malheur !

***(rires)* Non, c'est très bien. Donc, il pourrit facilement et d'un des fois on le mange alors qu’il n’est pas encore mûr. C’est un peu… c’est son inconvénient !**

Ben oui

**C’est dur de jauger.**

Ben il est fragile. Disons que c'est voilà… le fruit idéal, c’est la pomme. Ca pourrit pas facilement… C’est rare, sauf quand on prend une Granny Smith, on sait qu’elle va être dure, mais c'est ce qu'on veut. Généralement quand on achète une pomme… bah elle est à maturation quoi. Enfin, quand on achète des pommes avec une étiquette dans les magasins machin, etc. Si j'achète chez des petits producteurs, je vais avoir plus de surprise, je vais avoir des habitants dedans, des choses comme ça quoi.

***(rires)* Ok. Alors, c'est le moment de révélation. Je dirais que c'est la pêche.**

Oui, bravo, trop forte.

**J'ai eu un indice, mais bon, j’aurais trouvé quand même je pense !**

Mmh Mmh !

**Alors ok. La suite, j'aimerais qu'on discute de vos pratiques d'achat de votre organisation en général. Du coup, vous vivez avec vos filles ?**

Non. Pardon. Je vis seul, en normal, je vis seul et ponctuellement, j’ai mes filles.

**Et c'est régulier ou pas ?**

Pas que mes filles, j'ai une copine aussi qui est ponctuellement ici. Alors on commence par qui, du coup ?

**Ma question, c'est qui fait les courses alimentaires au sein de votre foyer ? Du coup ça être vous ?**

Ah c’est moi ! Evidemment, je ne laisse ça à personne ! C’est comme remplir le lave-vaisselle, c’est comme faire tourner une machine, personne d'autre n’a le droit de le faire.

**Ok ! *(rires)***

Oh non mais ça va non ? Mais même quand mes potes viennent manger, ils savent maintenant non non « je t’ai posé ça sur la table hein ! Je ne vais pas me risquer à me faire engueuler parce que j'ai mal rangé le lave-vaisselle avec la vaisselle sale ». Je dis « t’as intérêt ! » Non, non, les courses c'est moi, et personnes d’autres.

**Ok, ok !**

Bordel, on peut rajouter bordel ! Ah merde c’est enregistré, j’avais oublié ! *(rires)*

**De toute façon c’est moi qui récrit *(rires)*. Ok et du coup, comment vous vous organisez, pour vos courses alimentaires ?**

Comment je m'organise ?

**Oui**

C'est-à-dire où je les fais, quand ? À quel moment de la journée ? Tout ce qui me vient à l'esprit. Déjà, je l'ai fait. Je fais les courses lorsque j'en ai besoin. Mais il n'y a pas une règle. Ce n'est pas le samedi de 8 heures 32 à 9 heures 14. Je suis chez Leclerc. Non. C'est quand j'en ai besoin. Et quand est ce que j'en ai besoin ? Lorsqu'il y a des événements parti.. enfin des évènements, parce qu'il y a un repas prévu, lorsqu'il y a un apéro prévu avec des potes qui viennent ici lorsque ma fille est là, ça va être le cas vendredi soir, je rentre de Rennes avec elle, donc je veux qu'il y ait deux, trois basiques qui soient là pour faire au moins le repas du vendredi soir. Sachant que le samedi, on verra bien si on va au resto ou si on va se balader en fonction de la météo. Donc je ne vais pas, je ne vais pas planifier longtemps à l'avance. Je vais aller prioritairement dans le commerce dans lequel je sais ce que… je vais trouver ce que je cherche, c’est-à-dire, je m'explique. Euh.., demain, sans doute là aujourd'hui, je vais pas avoir le temps, je crois. Et puis ça risque d'être fermé. Je vais aller chez Faisan Doré, près des Halles Saint-Louis, pour acheter deux escalopes cordon bleu parce que là bas, ils ont des escalopes ccordon bleu extraordinaires, que je vais laisser au réfrigérateur jusqu'à vendredi soir et vendredi soir, quand on arrivera, *(sifflement)* je les mettrai au four et voilà, ça fera le job avec des pâtes ou autre chose.

**Ok…**

J'achète vraiment que ce dont j'ai besoin, mais c'est paradoxal que je suis en train de vous dire parce que… j’ai l’impression d’être allongé sur le canapé rouge. Parce que souvent, mon frigo est blindé mais blindé, alors si je vire les bouteilles, il y a plus grand-chose… Ah oui, j'ai toujours peur de manquer. Je pense que c’est parce que j'ai été élevé en partie par mes grands parents. Ils ont fait la guerre, la peur de manquer. Donc au cas où des amis arrivent, il faut du jus de fruit. Il faut du machin, il faut du cidre. Il faut…. Donc les courses, je vais attaquer la grande surface… alors la grande surface, la plus grande surface que je fasse c’est Leclerc du centre ville hein.

**Ok.**

Je m'interdis euh… sauf allez une fois par an, je vais être à Leclerc Kergaradec ou des trucs comme ça. Mais j'aime pas parce que sur le temple de la tentation, c'est le temple de la perte de temps. Quand je connais les magasins, je sais où trouver les affaires. Pas besoin d'une greffe de cerveau pour savoir où est rangé le Sopalin. Je vais généralement au leclerc du centre ville pour acheter plutôt des produits d'entretien, généralement pour la maison, et je vais greffer dessus tout l'alimentaire que je peux acheter chez eux, sachant que si je n'ai que de l'alimentaire à acheter, je vais plutôt privilégier les commerces de proximité autour des Halles. Sauf le lundi puisque le lundi ils sont fermés. Puis, je pars du principe que nous sommes ce que nous mangeons. Donc, je m'interdis de bouffer de la merde.

**Nous sommes ce que nous mangeons…**

Ouais complètement.

**Qu'est ce qui vous fait penser…**

Que c'est vrai ce que je dis ?

**Oui.**

Parce que je le dis déjà donc c'est vrai ! *(rires)*

**Point, c’est tout ! *(rires)***

Bah oui d’abord ! Ben oui, évidemment. Si je bouffe du nutriscore D ou E à longueur de journée, je vais grossir. Je le vois. Nous sommes ce que nous mangeons. J'ai arrêté autant que faire se peut tout ce qui est pain, crêpe, brioche, pain au chocolat, viennoiseries, pâtes, riz, pommes de terre, depuis quatre mois, je suis à moins dix kilos. J'ai pas arrêté de picoler, j’ai pas arrêté la charcuterie, j’ai pas arrêté le fromage vous imaginez ? et je n'ai pas fait de sport ! Vous imaginez si, si je coche en plus toutes ces cases là ? Je vais devenir gauler de ouf ! Mais c'est pas le but, on s'en fout. L'idée, c'était de perdre un petit peu et de…

**Juste en enlevant, pain, pommes de terre, brioche, les crêpes… Tout ce qui est à base de blé**

L'amidon oui l'amidon tout ça, absolument. Donc cela veut dire qu'il faut se... Il faut se bousculer un peu les neurones pour savoir qu'est ce que je vais faire à bouffer ? Ma fille bon avec le cordon bleu, je peux lui faire des pâtes, je peux lui faire des petits pois, je peux lui faire des haricots verts.. moi en accompagnement d’une viande ou d’un poisson bah…. J'ai acheté en fait une poêle-wok là et je fais revenir des légumes, des légumes frais que je taille… C'est bon, c'est croquant, c'est savoureux. Je joue avec les épices parce que l'amidon, on fait tout ce qui est riz, pommes de terre, pâtes, Un litre de flotte un peu de sel. On fait chauffer, pof pof. Je pense que c'est la première recette, entre surtout riz et pâtes parce que la patate, elle germe assez facilement quand même. C'est quand même la solution de facilité quoi… guillemets, qu'un parent apprend à son enfant. Les enfants, logiquement, quand ils participent, participent un petit peu à la maison. Ils savent assez rapidement cuire des pâtes avec le danger que représente évidemment une casserole d’eau bouillante. Ça, c'est autre chose. C'est dommage et c'est dommage mais en même temps, je crois que ça ne sert à rien de croire que l'on éduque ses enfants en les faisant cuisiner parce que [Prénom de l'enfant] qui a 9 ans et demi, bientôt 10 ans, elle se tenait assise que déjà elle était sur un plan de travail. Là où j'habitais avant. Et elle regardait, elle me regardait, faire et elle mangeait de tout ! Et elle allait chez la… chez la nounou, tous les jours avant d'aller à l'école. Enfin, avant d'être en âge d'aller à l'école, donc elle passait les journées chez la nounou et à l'époque, avec la maman d'[Prénom de l'enfant] on savait où la nounou faisait ses courses. C'est un apriori à la con, elle faisait ses courses chez Lidl. Et on se disait « elle fait ses courses chez lidl donc elle doit acheter de la merde ! » On lui avait dit non, nous on apportera son repas. Et donc le week end, je faisais le bourguignon, la blanquette, le kig ha farz, le machin et je préparais des petits pots moulinés, etc. Donc, elle ne mangeait que ce que j'avais préparé et j'étais persuadé que c'était bien pour son goût. Bon maintenant, aujourd'hui, c'est difficile de lui faire bouffer autre chose que du jambon pâtes ou des petits pois quoi…

**Ah…**

Les légumes, des courgettes, des machins non… Alors, elle en a mangé plus petites mais je pense que son, son papi à Rennes, mon ex-beau-père a dû la dégoûter tellement il lui.. il faisait pousser des courgettes et il a dû lui faire bouffer des courgettes à tour de bras. Donc ras le bol des courgettes. Elles ne mangent pas de carottes, pas de tomates, elles ne mangent pas de salade. Bon, c'est comme ça. Ça évoluera plus tard.

**C’est bien possible !**

Oui.

**Mais là, elles ne mangent pas beaucoup de légumes quoi.**

Non, c’est pas son… ce n'est pas sa tasse de thé, sa préférence quoi voilà.

**A 10 ans…**

Oui voilà.

**Ok, du coup. Alors quand vous allez faire vos courses, est ce que vous savez ce que vous allez chercher ou pas vraiment ? Est ce que ça peut être au feeling ? Ou est ce que c'est quand même défini ? Parce que vous avez dit, que vous ailliez pour une raison précise**

Alors, si, non non. Quand je vais, quand je vais faire uniquement l’alimentaire dans les commerces de proximité, si je vais chez le poissonnier… alors non en partant, si… je sais généralement si je vais aller chez le poissonnier ou si je vais chez le boucher.

**Ouais.**

Et quand je vais chez eux, généralement, je sais ce que je veux. C'est assez rare. Je vous dirais qu'à 80%, je sais ce que je veux et je me laisse séduire par le conseil du professionnel à 20%. Mais j'ai des idées assez arrêtées. Comme par exemple, si je veux des crevettes que je vais faire en marinade avec du citron vert, du gingembre et de l'ail euh… je vais au rayon poisson de Leclerc parce que des crevettes à 7,95, ça sera suffisant. Alors, c'est de l’élevage. Elles sont certainement nourries avec de la merde. Je peux prendre de la sauvage qui va coûter quatre fois le prix, mais j'ai expérimenté déjà. Et finalement, quand je les ai mises dans la marinade avec l’ail et le gingembre. Finalement, à l'arrivée, il n'y a pas de grosse différence...

**Oui…**

Donc mon principe, il est en train de voler en éclats, c’est ça ? ( ??) Evidemment que je bouffe certainement de la merde de temps en temps.

On est plein de contradictions sur plein de

Ouais

**Après c’est soumis voilà il y a la fourchette du prix aussi !**

Oui, parce que oui. Alors, le prix, vous voyez je vous le dis, je vais à la Corbeille d'or et des fois, je casse mon PEL pour acheter des tomates cerises des jardins Rabelais là, je sais plus où ils sont. Ça vous parle non ?

Non ?

Les jardins Rabelais je crois qu'ils sont dans la région de Tours et ils produisent des tomates, des petites tomates cerises en grappe qui sont joliment rangées dans un carton avec une jolie petite étiquette. Mais le prix au kilo oscille entre 28 et 30 euros le kilo de tomates cerises. C’est-à-dire, moi qui suis beaucoup plus âgé que vous, je peux vous dire que 30 euros le kilo, ça fait 200 francs. 200 francs le kilo ! Ma grand mère se retourne dans sa tombe et donc, de temps en temps, je vais me laisser séduire. Pourquoi ? C'est juste pour.. parce que c'est Instagram. Parce que la petite grappe est bien rangée sur la petite planche en bois, avec deux, trois trucs machins. C'est joli et ça flatte l'oeil parce que le plaisir de la table commence avec le plaisir du regard. Il faut que ce soit beau avant d'être bon. En revanche, quand je sais qu'à la plage, on va manger à pleine poignée des tomates cerises, la barquette qui était sur la table tout à l’heure, qui est ou ? Que personne ne sorte ! Vous avez piquez mes tomates ?

**Non ! *(rires)***

J’ai du les mettre au frais. Elles on où ? Elles sont là. Voilà, ça, Princes de Bretagne ou Savéol, ça fait le job.

**Ouais. Ok. Les fruits et légumes. Si on s'intéresse uniquement aux fruits et légumes, vous avez dit par exemple, tout à l’heure, que la pêche principalement au marché Saint-Louis ou à Amiral Linois, ou ailleurs…**

La Corbeille d’Or, c’est Siam primeurs ou La Corbeille d’Or, les deux commerces les plus proches, qui vendent des fruits a priori de qualité, non c’est pas à priori, ils sont de qualité assurément. Ils sont hors de prix, mais ils sont de qualité.

**Ouais, d’accord. Et du coup, quelle est la fréquence ? Est-ce que c'est régulier ?**

Des fruits et des légumes ?

**C'est pareil, c’est selon vos besoins ?**

Généralement, c'est quand même le dimanche matin. C'est vraiment là que je fais l'essentiel de mes courses, j’ai pas fait hier parce que j'étais un peu dans le jus et la priorité, c'était de faire mon ramassage scolaire, ma maman, ma soeur, tout le monde pour aller au Trez Hir on s’est retrouvé à 8 à 2 voiture, mais sinon, généralement, c'est le dimanche matin.

**A Saint-Louis du coup ?**

Dimanche matin, Saint-louis, sinon, ça va être Leclerc du centre-ville lorsque j'ai un évènement enfin j'appelle ça un événement… un repas ou un repas ici et donc je vais faire les courses globales. Mais vous voyez, je pense, à un truc là qui va me conduire à aller chez Leclerc prochainement. C'est juste parce que j'ai terminé ma bouteille de vinaigre balsamique blanc. C’est un truc tout con. Je vais aller chez Leclerc juste pour ça et je vais scanner les rayons que je connais par coeur et me dire « tiens ça j'ai besoin pour la lessive, ça, j'ai besoin » et je vais rajouter au fur et à mesure, mais pas nécessairement avec un besoin alimentaire particulier. Parce que vous voyez là je m'attache à vider mon frigo, à vider les placards, non pas que je veux pas que… parce que, parce qu'en fait on… entassent, on entassent, on entassent vous voyez là, c'est blindé. Bon, c'est le bordel déjà. Mais… et en plus, je consomme pas la moitié des choses, le chocolat et l'autre côté pareil. Il y a des conserves, il y a des pâtes, du riz, machin. Je peux consommer avant d'acheter. J’ai du stock.

**Oui, il y a toujours de quoi faire.**

Houlà oui !

**Ok. Et le marché ? Est ce que… par exemple là hier, vous n’avez pas été parce qu'il y avait autre chose de prévu. Mais sinon, vous y allez toutes les semaines ?**

Oui, oui, mais il y a autre chose de super important. Ben oui, c’est qu’après il y a l’apéro ! Eh ben oui, non mais c’est pas une blague !

Qui est revenu !

Et bien oui ! L'apéro, l'apéro du dimanche midi au central où on s'installe à midi, midi et demi et on fait la fermeture à 15 heures. Et on a un demi tour dans le corner, c'est le côté sympa avec les potes. C'est pas le prétexte parce qu'il y a le cadis quand même, qui est blindé avec des huîtres, des langoustines, du poisson, de la viande, des fruits, des légumes, qui est lourd en fin d’apéro. Mais en effet, quand on habite au centre ville, je trouve que c'est le côté, le moment à la fois de convivialité et facile pour faire les courses. Et puis, on voit plein de monde dans les allées on cause avec plein de gens. C'est un peu notre vie de village à nous au centre-ville.

***(rires)* Alors du coup, vous vous rendez au marché aussi par côté convivial et un peu festif.**

Oui bien-sûr

**Est-ce qu’il y a une autre raison pour laquelle vous privilégiez le marché, notamment pour acheter vos fruits et légumes ou pas ? C’est vraiment pour aller se promener, cet aspect…**

Pourquoi je privilégie ? Bah il y aussi le relationnel avec mon marchand de fruits et légumes que je connais depuis plusieurs années et donc je trouve, c'est…. comment dire, je respecte son travail, le fait qu'il se lève à pas-d’heure et je trouve logique de faire marcher sa petite économie à lui plutôt que Michel-Édouard, dans la mesure du possible. Mais en hiver, si j'ai pas envie d'aller au marché, il pleut. C'est pas agréable… Voilà, ça commence déjà par un moment de plaisir. Dans toute la chaîne de la bouffe, il faut du plaisir, y compris quand on fait les courses. Mais là où il n’y aura pas de plaisir, ce sera vraiment lorsque en hiver, voilà je vais faire mes courses au Leclerc, rapidement, et puis c’est une contrainte plus qu’un choix..

**Et du coup, vous pensez que dès le moment de l’achat, s’il y a du plaisir, ça influence le reste de la chaîne alimentaire ?**

Bah oui ! Je prends déjà du plaisir à imaginer ce que je vais faire manger. Parce qu'il faut que ce soit beau et bon, surtout bon d'abord, bon, le beau arrive au dernier moment, la mise en œuvre. Et oui, ça influe. Oui, bien sûr. Bah… si je m'en fous de ce que je vais bouffer et que je bouffe une boîte de cassoulet à même la boite parce que j'ai chauffé. Enfin bref, voilà c’est pas le même process quoi, c’est un global, c'est un tout.

**Ok. Et alors, quand vous êtes sur le marché, comment vous gérez les quantités que vous allez acheter, de vos fruits et légumes ?**

Je gère les quantités en fonction du nombre de consommateurs qu'on va être dans la semaine… c'est à dire si je suis seul, je vais prendre allez, 3, 4 fruits de chaque, pamplemousse, des oranges… Ca ce sont les fondamentaux, que j'ai toujours au réfrigérateur et kiwi également. Après, j’attaque sur les produits plus de saison, mais je vais effectivement en prendre plus si je sais que mes filles ou si j’ai des repas avec du monde ici. Je gère uniquement comme ça dans ma tête en fonction des événements à venir.

**Vous n'avez pas forcément une liste de courses ?**

Ah non. Ah non. Alors la liste de courses, elle est faite elle est là-dedans *(montre son portable)*. On va aller dans liste de courses. Chez Leclerc, balsamique blanc, un grand parasol, une capeline chapeau pour ma maman, on s'est rendu compte hier à la plage, à 78 ans, elle veut se protéger du soleil donc une grande capeline ce serait bien ! Une chaise pliante aussi pour la plage, du papier hygiénique, du déboucheur, de l’acide chlorhydrique et ( ??), et ça déboucheur, acide chlorhydrique et ( ??) ça figure sur ma liste depuis au moins trois ou quatre semaines. Je n'ai pas acheté pour l'instant parce que ce n'était pas une urgence. Je me suis dit « tiens, quand je penserai, on va.. Enfin quand ça sera vraiment l'occasion, quand il y aura besoin », alors je suis allé chez Leclerc, peut être une fois ou deux depuis. Mais bon, ce n'était pas une absolue nécessité. J'en aurais besoin, mais pas tout de suite.

**Donc, quand vous voyez qu’il y a quand même un produit qui manque, que vous avez l'idée de l'acheter, vous allez le noter quelque part ?**

Oui, oui.

**Ce qui constitue votre liste, le jour où vous irez plus tard.**

Oui. Mais l'alimentaire requiert assez peu de listes. Parce que c'est l'inspiration du moment ou voilà, par habitude, je sais ce que je vais prendre ou les 20% d’improvisation quoi.

**Ok, je vois. Donc il y a l’habitude et l’improvisation. Et si vous savez que vous accueillez quelqu'un la famille des amis pour un repas, est ce que vous savez déjà ce que vous allez vouloir cuisiner ? Ou non, c'est justement sur le marché où vous vous dites « ah ouais ça ça peut être bien » ?**

Non non… Généralement si, si sais. Parce que c'est en fonction de la météo aussi. Même si j'habite en appartement, le repas ne sera pas le même selon que ce soit en hiver. En hiver, je fais un kig ha farz, par exemple, avec un jour où il fait 5 degrés, du vent, de la pluie et un temps à rester sous la couette toute la journée… S'il y a un repas qui était prévu parce que, ça fait longtemps qu'on ne s'est pas vu, parce qu'un anniversaire, parce que c'est un rassemblement, etc. En hiver voilà, et en été, ce sera pas la même chose. C'est comme si j'avais une terrasse ou un jardin, il y aurait du barbeuc’, évidemment, de la côte de boeuf ou autre chose. Donc, je sais non, je sais généralement ce que je vais faire, ouais. Et puis, je veux pas me gratter la tête. C’est-à dire, si par exemple, vous me dites demain soir, on est six ici à l'apéro, je vais commencer à griffonner peut-être sur un petit papier quand même, deux, trois idées de ce que je pourrais faire, c'est à dire il y aura un plateau de fromages, il y aura de la charcuterie de chez ( ??), il y aura des crevettes marinées, peut-être un plateau, peut-être quelques huîtres. Voilà, je mets ça dessus, mais je ne vais pas marquer le détail de tout ce que je vais acheter. Je sais qu'en fonction de ce que je vais faire, je sais ce que doit acheter je vais plutôt fais une liste des plats, des aliments que je veux servir plutôt que la liste des achats.

Je **vois**.

Ca tient peut-être aussi au fait que mon père est restaurateur, que j'ai très tôt eu cette mécanique-là de… des achats pour le restaurant. J'ai fait une école hôtelière, donc en termes d'organisation bouffe. Attention, je ne suis pas exemplaire hein, mon frigo est dégueulasse et bordélique, mais quand il s’agit d’acheter ça ne me stresse pas.

**Oui, vous avez quand même l’habitude de gérer des quantités, pour un repas savoir combien**

Oui !

**Ce dont vous avez besoin. Ok. Alors, donc ça vous avez déjà un peu commencé à le faire. L'idée, c'est de dresser une liste des différents fruits et légumes que vous consommez très régulièrement.**

Oui, les fruits d'abord.

**Les fruits d'abord.**

Pomeloss, parce que moi j’appelle ça pamplemousse mais les marchands appellent ça pomeloss. Orange, kiwi. Ça, ce sont les trois basiques. Banane, pommes, poires lorsqu'il y en a évidemment en saison, les pêches, les abricots, les cerises. J'aurais dû mettre avant parce que c'est plus tôt les fraises les framboises. Ça j’en mangerai sur ( ??). Les fruits de la passion, l'ananas, la mangue, la mangue, j’aurai pu même mettre avant le fruit de la passion et l'ananas. Parce que c'est plus souvent. Pratiquement. ..C'est rare qu'une semaine, je n'en achète pas. Voilà. Parce qu'en fait j'ai réussi à stabiliser ma recette de salade de fruits. Voilà donc en fonction des différents épices que je mets dedans et tout ça et puis, c'est pas mal.

**OK !**

Ca, ce sont les fruits. Vous permettez deux secondes ?

**Bien-sûr**

*(la personne est au téléphone)*

Pardon. On passe aux légumes ou bien ? Alors les légumes, les légumes, alors les légumes… Poireaux, oignons, branches de céleri et poivrons. Fenouils. Carottes. Courgettes, aubergines, alors tout ça, évidemment, en bazar. Dans le désordre, en fonction de la période de l'année évidemment. Comme pour ce que je vous ai expliqué, plus jamais de patates. J’achète jamais. Mais qu'est ce que j'ai évidemment ? Des tomates bien-sur des tomates. J'ai des oignons, des oignons, des oignons, je prends, je précise, sauf cas exceptionnel là, oignons rouges, plutôt rouge parce que ça va bien dans les poêlés wok de légumes. Et j'ai arrêté d'acheter, sinon les oignons et les échalotes, privilégiant plutôt ces produits là, c'est du produit tout à fait naturel. C'est clairement de l'oignon, c'est purement de l’oignon et de l’échalote qui ont été déshydratés et qui permettent de réduire… sans que ça pourrisse en fait voilà ! Sans remplir la poubelle, la poubelle de Charlotte !

***(rires)* la poubelle de Charlotte ! Ok !**

En légumes, j’ai fait à peu près le tour, non ? Légumes, légumes, donc j’ai dit poivrons. J'ai dit poireaux, tomates ouais je tombe toujours sur les mêmes hein…

**Les courges, vous en consommez ?**

Si si aussi ! Les courges, des potirons, les potimarrons, les choses comme ça. Généralement, en soupe classiquement, avec un peu de poireaux, avant je mettais de la patate, maintenant j’en mettrai plus mais, un peu de poireau, un peu de cette branche de céleri… Voilà.

**Et du coup, si on prend les légumes que vous consommez pas, rarement ou pas du tout. Car la pomme de terre et c’est par raison de santé**

Ouais, santé régimé ! Bien-être !

**Bien être alimentaire. OK, est ce qu'il y a d'autres légumes que vous avez consommés que très rarement, que ce soit pour cette raison là ou non ?**

Pour cette raison là, non. Ce que je consomme assez rarement, c'est la salade. Parce que la salade, c'est pour sa complexité de mise en œuvre. C'est tout con alors que j'ai bien cuisiné. La salade. Alors je la prends, j'achète, j'achète, mais souvent, je la jette parce que elle est restée. C'est un peu le parent pauvre de l'organisation du repas. C'est le repas. C'est une entrée ou un apéro, un plat, un dessert, le fromage, « ah merde la salade ». Quelqu'un ? Personne veut de la salade. OK, elle reste au frigo, elle reste au frigo et puis elle va s'abîmer, s'abîmer. Et puis, finalement, je n'aime pas le principe d’acheter de la salade toute faite, mais c'est tellement pratique. Mais voilà, c'est plutôt le j'aime bien, j'aime beaucoup. Ah si, il y a quelque chose, j'oubliais : les endives ! Les endives, mon ami, mon ami de l'hiver, poêlées, au beurre avec un peu de miel. En gratin évidemment… en salade avec les cerneaux de noi, du roquefort et du vinaigre balsamique c’est une tuerie ! J’adore les endives. Qu'est ce que j'ai oublié comme légume et que je puisse réagir… A part la salade ? Qu'est ce qu'il y a comme légume que je n'achète pas ? Du chou ! Si chou fleur, j’achète du chou fleur ! Chou-fleur oui, artichauts non. Non, parce que… parce que j'y pense pas, parce que c'est peut-être aussi le principe de passer du temps à manger la bête ! Alors que j’en ai mangé chez, quand j'étais gamin, chez ma grand mère. Ça fait des années que j’ai pas mangé d'artichaut pourtant j’aime bien ça. J’achète des asperges aussi. J'achète des navets, le navet, c'est plutôt le compagnon dans la soupe, dans le pot au feu machin voilà, il est pas prépondérant. A quoi je n'ai pas pensé en légumes ?

**On commence à être pas mal. Les légumes racines ?**

Les panais ?

**Panais, gingembre.**

Le gingembre et le citron vert, je les ai tout le temps chez moi parce que c'est le petit condiment facile, enfin c’est surtout depuis que j'ai acheté la micro-plane. Vous connaissez le micro-plane ?

**Non.. Ah ça zeste ?**

Voilà, c'est exactement ça. Vous mettez le gingembre directement dessus. Mais il est ou *(la personne cherche son citron)* Je l'avais hier. J'ai utilisé pour la… tant pis on va pas passer deux heures dessus ! Je l’ai utilisé pour la salade de fruits hier matin, je l’ai peut-être mis au réfrigérateur.

**En zeste du coup ?**

Ouais. Et sinon, oui il y a aussi deux choses dans ma mécanique, dans ma façon de faire. Le gingembre, deux fois l'an à peu près, j'en achète en grosse quantité. Je coupe en petits morceaux que je passe dans mon extracteur à jus ici pour en faire un jus vraiment pur gingembre. Et que je mets dans des bacs à glaçons. Je congèle. Et quand les glaçons sont formés, je mets ça dans un grand sac plastique au congélo. Et je fais la même chose avec l'ail. Ca me fait un jus d'ail et des glaçons d’ails et des glaçons de gingembre me permettent de faire des marinades facilement, me permettent de mettre dans un plat facilement un plat un peu exotique avec du lait de coco des choses comme ça, c’est rapide, quoi. Je passe du temps à le faire, mais c'est pour du gain de temps par la suite.

**Ok, et les fruits, est-ce qu’il y en a que vous avez en tête, que vous savez, vous connaissez ces fruits là, mais vous les consommez très rarement, voire jamais ? Est-ce qu’il y en a ou non ?**

Oula… Je réfléchis donc, quand je regarde, je passe en revue l'étal de fruits, que ce soit chez mon marchand ou chez Leclerc, ou est ce qu'il y a des fruits que je n'achète jamais ?

**Ouais.**

Si les fruits qui viennent de Chine, comment ça s'appelle, des grosses de sorte de poires chinoises, je sais plus comment on voit ça dans les commerces. Clairement, ça, non. En fait, en général, j'évite d'acheter ce qui vient de loin. C'est pas tant le fruit parce que je ne le connais pas. C'est un a priori, mais c'est a priori de bilan carbone et tout et tout. Alors après si je me suis mis en tête de faire une recette qui requiert l'usage d'un fruit et que on est encore sur les ailes de saison et qu'on en n'a pas encore, je mangerai quand même des framboises qui vont venir du Portugal, d'accord, mais j'évite de prendre un truc qui vient de.. par exemple des haricots verts du Kenya, quand c'est pas la saison des haricots verts non. Je m'y refuse. Donc dans des fruits, sinon question de goût ou quoi que ce soit…. Est-ce qu'il y a des fruits vous, qui vous viennent à l'esprit ?

**Et bien non, vous en avez cité pas mal donc euh…**

Bon la noix de coco, par exemple, je n'achète pas parce que c'est compliqué, il faut la casser… alors on en trouve maintenant des barquettes dans lesquelles il y a la pulpe de coco si j'en avais besoin. Qu’est-ce que j'achète pas encore en fruits ou légumes… euh je ne vois pas.

**OK. Les légumes il y avait l'artichaut, j'aime bien, mais j’y pense pas. La salade parce que c'est complexe, on finit par la perdre bien souvent. Les oignons jaunes plutôt maintenant déshydratés et les pommes de terre pour des raisons de conforts alimentaires. OK, ça marche. Si on s'intéresse au lieu de stockage de vos fruits et légumes… Quand vous revenez de vos courses, qu’est-ce que vous faites de vos fruits et légumes ?**

Je mets au réfrigérateur tout ce qui va au réfrigérateur. C’est-à-dire sauf les bananes, évidemment, qui ne vont pas au froid. Les pommes généralement, je les mets aussi dans la corbeille et les produits plus fragiles qui peuvent mûrir vite bah je vais les mettre au réfrigérateur. Tiens, j'ai oublié les avocats, car j'en consomme. Il fait partie des basiques, tout le temps. Bon, je regarde pas trop d’où ils ont pris l'avion. Mauvaise exemple voilà nous sommes plein de contradiction voilà... Donc généralement voilà, ça va au réfrigérateur pour les produits fragiles et les non-fragiles, restent à l'air libre tout simplement.

**Les légumes, au réfrigérateur ?**

Oui, oui pratiquement tous parce que, mis à part oignons quand il y a un oignon il va pas au réfrigérateur, sauf quand il est entamé, parce que quand j’ai commencé à taper dedans…. Je ne vais pas nécessairement manger tout un poivron. Je vais avoir besoin d'un demi poivron rouge et de morceaux de différents légumes. Après, je les enferme dans un sac zippé au réfrigérateur. Bon, au bout de quelques temps, peut être que ça va aller dans la poubelle euh LEGO ? Sinon vous allez être vexée si je l’appelle Poubelle Charlotte.

***(rires)* Ok, donc c'est plutôt réflexe réfrigérateur, sauf oignons, bah pommes de terre vous n’en avez plus. Bananes, pommes… Ok.**

Que ce soit, kiwi, abricot en saison, que ce soit les pêches, tout ça, ça va au réfrigérateur.

**Ok et est-ce que ça vous convient ? Comme manière de stocker vos fruits et légumes, ou pas vraiment ?**

Pas vraiment non parce que je trouve que c'est extrême, le réfrigérateur, c'est extrême dans la conservation. J'aurais un cellier, un peu comme les anciens. C'est-à-dire une arrière cuisine ventilée naturellement avec des clayettes en bois sur lesquels viendrait poser délicatement toutes mes affaires là et ça respire plus naturellement, je préfèrerai donc non non, ça ne me convient pas parce que je dis… c'est extrême, c’est extrême, même quand je fais de la viande, par exemple. Il faut absolument que je pense à, une côte de boeuf ou une belle entrecôte, à la sortir trois à quatre heures avant. Une viande, il faut surtout pas la mettre froide dans une poêle. Sinon, la meilleure des viande du monde peut devenir sans intérêt.

**Ouais du coup là, c'est parce que vous n'avez pas d’arrière-cuisine, de celliers, que vous choisissez la condition extrême réfrigérée, mais si vous n'aviez pas de contraintes matérielles ce serait plutôt en arrière-cuisine.**

Ah oui oui, complètement tout à fait ! Je me rappelle, mes grands-parents ils stockaient des pommes dans un cellier avec terre battue, ça respirait. Ça sentait bon. Et puis, il y a une durée…

**C’est une température un peu modérée**

Exactement, avec des murs épais qui gardent l'inertie de la température. Il n’y a pas de grandes variations, jamais. C’est comme une cave quoi ! Ca va très bien, ça va très bien aux fruits et légumes. Ça va très bien aux viandes, aux charcuteries, aux salaisons. Ça va très bien... Au vin ! Ca va très bien à plein de choses.

**Les fromages**

Des fromages, bien sûr. Absolument. Tout à fait.

**Ok ! Et en termes de nettoyage du coup, vous avez une corbeille et le réfrigérateur. Comment vous procédez ? Est ce que c'est systématique, réglé, quand vous vous semble ?**

Quand c’est sale, quand c’est sale. Quand quelque chose a coulé. Quand je vois qu'il y a quelque chose qui est coulé et que lC'est honteux hein... Mais j'ai emménagé ici en septembre 2016. Je n'ai jamais vidé complètement le réfrigérateur ni congélateur. C’est-pour-ça, je me dis, le vider un peu ça serait bien, vider le congélateur, tout ça, ne serait ce que dégivrer, le faire un peu respirer et lui redonner un peu d'air. Il a quelques années, lui, il doit avoir 15 ans maintenant ce réfrigérateur euh voilà, j'y attache pas non plus une très, très grande… un très, très grand soin. 'étagère est dégueulasse, je vide l'étagère, je la sors, je la nettoie, je la rince, je sèche bien et je la remets en place. Je n’ai jamais… Je me dis que quand il aura fait son temps, je passerai à autre chose.

**OK donc sur le nettoyage, c'est**

Non, c’est quand ça a besoin, c’est tout !

**OK. Ça marche alors après l’idée là c’est de parler de vos pratiques de préparation et de consommation. Vous avez déjà pas mal raconter ce que vous faites, mais si on reprend un fruit, que vous avez dit consommer très régulièrement.**

Vous voulez un fruit que je consomme régulièrement ?

**L'Orange ?**

Par exemple, l'orange, c'est toute l'année ouais

**Alors, est ce que vous pouvez me raconter tout ce que vous faites avec l'orange ?**

Oh bah l’orange principalement, je la pelle à vif. Je la coupe en petits morceaux. Je mets ça dans un bol et je mange à la fourchette. Ou alors ça peut être un peu plus travaillé, là je mets dans un grand saladier avec d'autres fruits, et il va être accompagné, accompagné d'autres fruits pour faire une salade de fruits. Je ne la mange que comme ça, je ne fais jamais de jus d'orange.

**OK.**

Le pomelos c’est pareil.

**Ok. La banane, par exemple ?**

La banane, majoritairement, alors ça peut être le type… La banane ça va être le coupe faim. En mode coupe faim comme ça paf ! En mode salade de fruits parce qu’elle est vachement sympa en salade de fruits, la banane. Et puis, évidemment, quand elle est un peu fatiguée, elle va se jeter dans une poêle avec du beurre salé et elle va se faire flamber au rhum. Caramélisée et compotée et versée après sur deux boules de glace vanille. C'est une tuerie internationale.

**OK ! Donc, tout à l’heure c’était les abricots, les pêches, et là la banane, ça vous arrive, quand vous voyez qu'un fruit commence à être un fatigué, vous arrivez à le cuisiner**

Ah oui ! Pratiquement, c’est tout le temps, mais vraiment tout le temps parce que je jette pas. Non, je jette peu, alors qu’est-ce que, il aurait peut-être fallu que je donne ce que je mettais dans la poubelle ? Euh… j'ai ouvert un melon, sur mon premier sac poubelle parce que là je suis au deuxième sac poubelle, sur le premier sacs poubelle, il était super lourd, ça avait rendu plein de flotte parce que j'avais mis un melon. Et puis, en fait je laisse comme je remplis pas beaucoup, ça reste longtemps, vous voyez en un mois il y eu deux sacs. Donc un sac fait 15 jours, ça sentait un petit peu. Bon, on est dans la période où je peux ouvrir la fenêtre. Le melon était dégueulasse, là j’ai pas réfléchi, j'ai mis… il avait aucun goût quoi ! Là faut à moins de rajouter du sucre, le faire cuire pour faire une confiture de melon... C'est pas quelque chose qui… dans mes principes j’ai jamais fait de confiture de melon, donc, mais les fruits en petite quantité, vous voyez ce matin, j'avais 3 abricots parce que j’ai acheté des abricots au marché du Trez Hir hier matin, les trois qui étaient abîmés vraiment et bah je me dis, ils sont trop trop écrasés pour que je prenne plaisir, parce qu'il faut aussi du croquant, il faut satisfaire certains… ouais, enfin, je sais pas comment expliquer. Quand on mord dans un fruit, on mord ! On n’est pas à l’EPAD ! On n'est pas en compote. Bon, ben là, je vais effectivement le transformer en compote. Donc, je jette extrêmement rarement des fruits entiers.

**Ok et par contre, s'il n’a plus cet aspect croquant que vous cherchez, frais, que vous cherchez dans le fruit, vous allez vite le transformer**

Mh. Et s’il y en a beaucoup, ça va être une tarte à l’abricot et romarin. Par exemple.

**Donc vous cuisinez quand même euh..**

Bah je cuisine euh

**Pas mal vos fruits finalement !**

Pas mal mes fruits oui. Enfin, si vous regardez cette semaine entre lundi midi et vendredi soir, il n’y a que vendredi soir que je vais manger ici, tous les autres jours, même midi ou soir, je suis pas là, donc je ne vais pas cuisiner, donc il y a pas de mérite. Oui, quand je cuisine, je cuisine un petit peu oui !

**Ok. Et les légumes ? Si on en prend un que vous avez dit consommez régulièrement. Le poireaux. Qu’est-ce que ovus faites avec le poireau ?**

Le poireau, je le nettoie. C’est-à-dire, je lui coupe les cheveux, je le fends, je passe un peu d'eau… de l'eau… Ca consomme beaucoup d'eau le poireau parce que mine de rien, il y a de la terre entre les différents trucs là. Voilà et après, je coupe en tronçons et je mets dans un sac et j'utilise après soit en fondu avec un peu de crème, du curry, pour accompagner du poisson, soit en soupe, beaucoup en soupe, majoritairement en soupe, alors majoritairement souple et en…. quand je fais la soupe, je peux en garder un ou deux que je vais garder entiers et ficelés comme ça après, je les fais en vinaigrette en entier quoi.

**Ok. D'accord.**

Poireaux vinaigrettes en soupe

**Ou en fondue.**

Voilà, généralement c’est comme ça.

**OK quand vous avez le poireau. Donc le poireau il est comme ça. Comment vous…**

Ah ! Ah ! Entre le vert et le blanc ? Généralement pour la soupe, je garde tout le vert, sauf les parties qui sont vraiment abîmées ou voilà… Pour la soupe, je vais mettre tout le vert. Même pour… ouais je garde vachement vert en fait, je retire vraiment que les parties abîmées. Parce que le vert à du goût. Je surveille l’heure, à 10 heures, j'ai un rendez vous téléphonique, mais je ne sais pas *(discussion sur temps restant d’entretien..)*

**Quand vous jetez fruit ou un légume… Pourquoi avez vous décidé à ce moment là de le jeter plutôt que de le consommer ?**

Parce qu'il n'est juste pas consommable, parce qu'il est pourri

**Ok, et comment vous déterminez ?**

Oh bah ça se voit ! *(rires)* bah des fruits pourris des fruits pourris ! Vous ne savez pas ce que c'est un fruit pourri ? A votre âge ? et bah merde alors *(rires)* Euh non, que je jette et que je ne fais pas un plan B… Je n’ai pas d’exemple… Si, ce qui me vient à l'esprit sur les courges et potirons, la machin quand il y a une grosse partie là qui a, parce qu'il est posé, parce que le problème des courges, c'est que ça conserve longtemps, mais on le voit, mais il y a une partie cachées derrière qu’on ne voit pas. Ah merde, dégueulasse. J'enlève tout ça et je fou à la poubelle parce que c'est vraiment pas utilisable.

**Et selon vous ? Pourquoi c'est pas utilisable, pas consommable ? Pourquoi ne pas le consommer ce pourri ?**

Oui, parce que la nature de départ n'est plus au rendez vous. C'est à dire le goût, la texture ne sont plus là. Ils ont été complètement transformé par les bactéries. Je crois. Et ça, c’est même pas super propice à la consommation. Si j'avais des poules, je donnerais, je mettrais tout au poule, ce serait mangé. J'ai eu des poules par le passé, quand j'étais en maison. D'ailleurs, si j'étais en maison aujourd'hui, je reprendrais des poules. C'est pratique, les poules, ça bouffe tout !

**Et ça fait des œufs, des fois.**

Et ça fait des oeufs. Absolument.

**Donc ce n'est pas propice… Enfin vous m’avez dit qu’on ne retrouve plus ce qu’on recherché avant dans le… Et en plus, pour vous, c'est pas propice à la consommation, il y a le goût et autre chose ou…**

Euh.. Non en fait le goût n'est plus là, alors le goût n'est plus là. La consistance n'est plus là. Et surtout, je pense, ça peut peut-être être dangereux pour la santé de manger quelque chose qui a été complètement altéré.

**Ouais donc poubelle à ce moment là.**

Ouais poubelle mais c'est extrêmement rare. Si c’est 1% de ce que j'achète, c'est le bout du monde.

**Comme le melon, la dernière fois c’est juste qu’il n'était pas bon.**

Franchement pas bon, non mais ouais. La promesse n'était pas au rendez vous. Quand j'achète, vous savez, je vous ai dit… je dirai le plaisir... L'idée de la survenance du plaisir, c'est déjà du plaisir. Quand j'achète un melon, je me dis « ouais c'est le goût du melon, ça va être chouette ». C'est du plaisir. Mais si, quand je l’ouvre il est dégueulasse, je suis super déçu, allez vire.

**Est ce que vous avez… Quand vous êtes face au pourri, qu'est ce que vous ressentez ? Vous êtes face à une courge pourrie, à un..**

Oui. Je m'en veux de l'avoir laissé pourrir. Je vois de l'argent qui part à la poubelle. C'est presque honteux. Je dis presque, il faut pas non plus se taper la tête contre les murs.

**Ouais ok et justement, si on parle de gaspillage alimentaire, quand je vous dis gaspillage alimentaire, c'est quoi les mots qui vous viennent spontanément à l'esprit ?**

Cantine, spontanément, cantine. Gaspillage alimentaire ? Bioseau ? Bizarrement, parce que bah oui, mais que dans Bioseau on va certainement trouvé des choses qui ont été mises et qui auraient pu être mangés. Gaspillage alimentaire ? Qu’est-ce qui me vient à l’esprit avec gaspillage alimentaire…la restauration au sens large quoi, le nombre d'assiettes qui reviennent, sur lesquelles il y a des trucs, c'est du gaspillage alimentaire. Alors vous avez peut-être regardé les DLC de certains yaourts dans mon réfrigérateur, mais je crois que je détiens un record du monde. J'ai dû manger un Activia nature qui avait 3 mois de dates. Il était parfait. Il était très bien. Faut qu'on arrête de nous enfumer avec des pseudo dates machin… Donc gaspillage alimentaire ? Bon, quand je regarde mon congélateur, je vais penser à, allez un truc que j'ai mis au congélateur en disant tiens plutôt que de le perdre, c'est un plat que j'ai préparé moi-même. Je le mets au congélateur. Puis, au final, si je l’ai pas bouffé au bout de 6 mois il va peut-être partir à la poubelle, c'est du gaspillage alimentaire.

**Vous congelez, vous pratiquez la congélation ?**

Ouais congélation maison, ouais ouais. Il est 57 je…

**Oui oui allez-y ! Faites, faites.**

Je vais là-bas. Vous revoulez un café ?

**Non non merci c’est gentil.**

**Household F39, Interview 2**

*Mme.1:* Non, non, je mets ça près de vous.

*Mr.2:* Oui.

*Mme.1:* Alors l'idée, c'est que vous retournez une de ces photos.

*Mr.2:* Oui.

*Mme.1:* N'importe laquelle--

*Mr.2:* Oui.

*Mme.1:* Et que vous me disiez spontanément ce qui vous vient à l'esprit.

*Mr.2:* Je retourne une photo.

*Mr.2:* Oui.

*Mr.2:* Pour moi, c'est un marché. Ça peut être le marché du dimanche matin, aux Halles Saint-Louis.

*Mme.1:* Oui.

*Mr.2:* Des produits frais [euh] des produits plutôt bio, des produits qui viennent de pas loin.

*Mme.1:* Oui.

*Mr.2:* [Euh] des produits sains. Je pense, ce sont des produits pas chers [euh], ce qui me vient à l'esprit, parce que c'est tout ce qui me vient a à l'esprit, il n'y a pas d'emballage, vous voyez individuels.

*Mme.1:* Oui.

*Mr.2:* Le fait qu'il n'y ait pas d'emballages individuels, il n'y a pas d'emballage à payer [euh] il y a moins de pollution. [Euh] Alors après, je- je- je regarde que les fruits et légumes derrière, je vois une clientèle plutôt âgée.

*Mme.1:* [Rire]

*Mr.2:* [Euh] parce qu'on est sur un horaire, peut être de travail aux horaires de travail par définition, sur lequel les gens qui travaillent ne peuvent pas aller au marché.

*Mme.1:* Oui.

*Mr.2:* Et donc ça veut dire aussi que de fait, seuls les vieux peuvent bien manger et pas cher.

*Mme.1:* C'est ça.

*Mr.2:* On peut, on peut imaginer. Donc ça veut dire que quand on travaille, quand on est à un rythme de débile comme le nôtre à tous.

*Mr.2:* [Rire]

*Mr.2:* [Euh] [en fait], on est condamné à bouffer de la merde chère un petit peu. Donc dans l'absolu, la solution, ce serait quoi ? Qu'il y ait des marchés en horaires décalés.

*Mme.1:* Oui.

*Mr.2:* Par exemple, [alors] les marchés ouvrent tôt le matin limite celui qui va bosser peut, s'il le souhaite et s'il le peut en termes d'organisation personnelle, familiale [euh] se rendre au marché avant d'aller au boulot. Mais après, il faut revenir à la maison, reposer ses courses,

*Mme.1:* Oui.

*Mr.2:* [Euh] avoir les marchés-- parce que les marchés sont tout le temps le matin. D'ailleurs tiens, ça m'interpelle ça. Pourquoi les marchés sont-ils tout le temps le matin ? Il est extrêmement rare [alors], sauf dans Pari-- Paris où il peut y avoir des quartiers ou des-- je crois passées 13h a mon avis, il n'y a pas grand-chose [euh] alors que les gens qui bossent, il faut bien qu'ils fassent leurs courses. Il y aurait des marchés qui fonctionneraient de 16h à 21h. Après, on va me dire [ben] oui, mais le producteur qui vend ses carottes, il a le droit d'avoir une vie de famille aussi. Alors là, [ben], on s'en sort, on s'en sort plus.

*Mme.1:* [Rire]

*Mr.2:* Hein.

*Mme.1:* Je crois que celui de de Kérinou [euh] je crois qu'il a un créneau le soir.

*Mr.2:* Oui, oui, oui, mais alors c'est celui de Kérinou.

*Mme.1:* Oui.

*Mr.2:* Ça veut dire, il faut aussi, en accessibilité, [en fait] avoir l'information déjà. Après, vous me direz aujourd'hui, si on veut l'information, on la trouve. C'est pas faux, mais il y a une différence. Il y a deux-- il y a deux niveaux d'information, il y a l'information que je cherche, j'en ai besoin eil y a celle qui se présente à moi et dont je ferai bon usage.

*Mme.1:* Oui.

*Mr.2:* Voilà. [Euh] qu'est-ce que je peux dire d'autres sur la photo ? [Ben] rien. Il y a des patates et des carottes il y a des choux.

*Mme.1:* [Rire]

*Mr.2:* Voilà.

*Mme.1:* Donc quand vous avez vu le marché, vous avez très rapidement parlé de produits frais, bio, locaux.

*Mr.2:* Ouais.

*Mme.1:* Donc c'est [euh]-- vous, vous avez l'habitude de consommer ce genre de produits.

*Mr.2:* Même pas.

*Mr.2:* Même pas ?

*Mr.2:* Même pas, mais attendez vous m'avez demandé ce que ça m'inspirait. J'ai pas dit que c'était moi. Parce qu'en fait quand je vais au marché, [bah] j'achète [euh] j'ai mes habitudes. Toujours chez les mêmes marchands et jamais, au grand jamais, je n'ai-- je ne l'ai ni questionné sur la provenance des produits, ni demandé explicitement à avoir des produits bio ou de proximité. Jamais.

*Mme.1:* Ouais.

*Mr.2:* C'est ce que ça m'inspire, mais- mais dans mes usages, si j'ai besoin de bananes, j'ai besoin de banane, je vais pas me faire des nœuds au cerveau. Savoir s'il est de la Martinique, de Cuba ou d'ailleurs.

*Mr.2:* Oui.

*Mr.2:* [Euh] je- je ouais j'ai besoin de patates, j'ai besoin de cour-- quels que soient les produits, je veux juste que les produits soient beaux et bons. Alors, beau et bon. Ouais, parce que quand c'est beau, on se dit que c'est meilleur déjà. [En fait], le- le- le plaisir du- du bien manger commence par le visuel.

*Mme.1:* Oui.

*Mr.2:* Même si je sais très bien qu'il y a-- il y a des rayons-là dans les magasins qui ont commencé à se lancer sur [euh] en gros, une pub avec les fruits, ouais je suis moche, mais je suis bon quand même, achetez-moi etc. Et c'est vrai que dans un rayon évidemment, qu'entre une belle grappe de raisin et une grappe de raisin moche comme dirait l'autre, la question est vite répondue.

*Mme.1:* Oui.

*Mr.2:* Bon, mais [euh] bon, je dirais que les fruits moches, ça va être plutôt pour- pour la cuisine, pour- pour une transformation, alors que le beau fruit ça va être dans la coupe, ça va être sur une belle table.

*Mme.1:* Ouais est-ce que vous pouvez développer pourquoi le fruit moche [euh] en transformation ça- ça va ?

*Mr.2:* [Ben], ça va, [ben] le beau aussi. Disons que ça va être-- alors attention, vous voyez, quand on dit moche dans mon esprit, à tort, [euh] il est peut-être un peu abimé, il est un peu taché, un peu gâté, c'est pour ça. Alors que moche, ce n'est pas ça.

*Mme.1:* Oui.

*Mr.2:* Je connais,-- je connais des gens qui sont moches, qui ne sont pas malades.

*Mme.1:* [Rire].

*Mr.2:* Ils sont juste moches. [Euh] développer sur les fruits moches [euh] ouais [ben] j'en achète pas beaucoup, mais c'est je-- ça me pose pas de problème d'en acheter si c'est pour faire une cuisson.

*Mme.1:* Ouais.

*Mr.2:* En gros les pommes, typiquement, je m'en fous de la gueule des pommes à partir du moment où je veux faire une compote.

*Mme.1:* Ouais. Est-ce que vous savez déjà pourquoi ?

*Mr.2:* Pourquoi [euh] ça commence à être compliqué les questions.

*Mme.1:* [Rire] on se doute, mais essayer de--

*Mr.2:* Non non mais pourquoi ça ne me pose pas de problème-- alors disons que je trouve-- la beauté du fruit, c'est presque dommage de la sacrifier en détruisant le fruit pour le transformer. Ça vous va ?

*Mme.1:* Oui.

*Mr.2:* Faudrait presque que je l'encadre celle-là. C'est dommage parce que le fruit, effectivement, il y a-- il y a des des-- alors la nature se fait chier à nous faire des beaux trucs. Les agriculteurs se font chier à nous-- à- à faire en sorte que les produits soient beaux. Bon [ben] donc [euh] c'est pour qu'on-- que cette beauté-là soit aussi utilisée d'une certaine façon. Alors évidemment, c'est pour l'acte d'achat. C'est un coup de foudre, mais si ça peut durer un petit peu et d'ailleurs, il y a des artistes qui ont fait des natures mortes avec des coupes de fruits depuis presque la nuit des temps. Je pense qu'ils ont eu à cœur aussi de choisir des belles pommes rouges, des beaux fruits, [euh] un beau faisan qui venait d'être tué. Mais bon [euh] le faisan qui était à moitié handicapé, je pense qu'il avait pas sa place sur la nature morte quoi.

*Mme.1:* [Rire].

*Mr.2:* J'imagine.

*Mme.1:* C'est vrai.

*Mme.1:* Et donc le marché--

*Mr.2:* Oui, allez-y, questionnez-moi.

*Mme.1:* C'est un lieu dans lequel vous vous allez.

*Mr.2:* Oui.

*Mme.1:* Et comment vous vous sentez dans le marché ? Est-ce que c'est un lieu de course par nécessité ? Est-ce que c'est [euh]--

*Mr.2:* Mais non-- mais non, c'est un prétexte pour voir des potes.

*Mme.1:* [Rire].

*Mr.2:* C'est un lieu-- j'ai failli vous répondre comment vous vous sentez, presque en représentation. Parce que je suis certain que je vais croiser des gens que je connais. Et c'est un moment agréable. Alors c'est un moment agréable quand il fait beau. Je vais pas aller au marché s'il pleut, ça m'emmerde royalement. [En fait], les courses, je les fais [alors] indifféremment marché, supermarché [euh], ici, rue de Paris ou celui qui est le plus pratique.

*Mme.1:* Ouais.

*Mr.2:* Je cherche avant tout la praticité et la praticité pour moi, ça rime avec rapidité, simplicité. Il faut que je puisse me garer facilement ou que ce soit accessible à pied facilement. [Euh] il faut que je sois certain de trouver le produit parce qu'il y a rien qui me gonfle plus que de faire deux ou trois magasins pour trouver un produit. [Euh] et puis [euh] bon le prix ne rentre pas en considération parce qu'on n'est pas sur des dépenses inconsidérées [quoi]. Alors évidemment que oui des mangues vont coûter un peu plus cher qu'un kilo de patates ou une botte de poireaux d'ici en bonne période.

*Mme.1:* Ouais.

*Mr.2:* Mais effectivement, c'est quelque chose que je- je- je regarde pas. Et donc le marché oui, j'y vais-- j'y vais. [Alors] quand il fait beau, du moins qu'il ne pleut pas [euh] été comme hiver, peu importe. Plus l'été quand même. Parce qu'en hi-- en hiver, [euh] [alors]-- et puis j'y vais lorsque je ne vais pas être trop en déplacement la semaine suivante.

*Mme.1:* D'accord.

*Mr.2:* Parce que il y a-- ça sert à rien que je fasse des courses le dimanche si je suis parti le lundi soir ou le mardi matin.

*Mme.1:* Ouais.

*Mr.2:* Je vais pas stocker les produits sinon après je serais obligé de les découper, les mettre dans un sac plastique, appeler Charlotte, il faut venir machin etc. Compliqué.

*Mr.2:* Le petit bruit à l'enregistrement, je mange mon œuf.

*Mme.1:* [Rire]. Et [euh] du coup le-- vous y-- donc vous y allez pas forcément parce que vous avez besoin de produits, c'est plutôt par plaisir d'aller sur le marché.

*Mr.2:* C'est le mix des deux. Je me dis je vais sur le marché même des-- la plupart du temps, sans savoir ce que je vais y acheter.

*Mme.1:* Oui.

*Mme.1:* C'est pas ça le plus important. Je vais certainement ramener un ou deux repas ou quelques fruits. [Euh] je vais peut-être allé aussi chez la fromagère qui est aux halles. [Euh] et puis bon, c'est vrai que la plupart du temps quand je vais au marché aux beaux jours, ça se prolonge pendant deux heures, deux heures et demie en terrasse d'un café et il y a des potes qui arrivent, il y a du monde, il y a de la famille. On s'est vu faire des tablées où on était quatre au début, 12 à la fin.

*Mme.1:* Ce que vous aimez dans le marché c'est surtout le- le fait de- de--

*Mr.2:* La convivialité. Pardon, j'ai coupé la parole.

*Mme.1:* Non [bah] non, justement, je préfère que vous exprimez vous même le mot.

*Mr.2:* Ouais, ouais, ouais. La convivialité, absolument. La convivialité-- la convivialité, [euh] le bruit, la diversité, diversité de produits, diversité de- de- de- de clientèle.

*Mme.1:* Oui.

*Mr.2:* Parce que là, il y a tous les âges. Après, ça dépend des horaires.

*Mme.1:* Oui.

*Mr.2:* Si vous allez le matin, effectivement, vous allez voir les anciens.

*Mme.1:* Oui.

*Mr.2:* Si vous allez en-- vers 10h, 11h, vous avez les familles avec les jeunes enfants et en [euh] fin de matinée, vous avez ceux qui ont fait la nouba la veille et qui ont la tête à l'envers.

*Mme.1:* [Rire].

*Mr.2:* On est d'accord ?

*Mme.1:* Au dernier moment, à 13h.

*Mr.2:* C'est ça.

*Mme.1:* OK. [Euh] est-ce que ça vous arrive de perdre des produits que vous aviez acheté au marché ?

*Mr.2:* De les perdre ?

*Mme.1:* De devoir les mettre à la poubelle.

*Mr.2:* Ah ! Parce que perdre, il m'est arrivé en rentrant de vider mon caddie et deux jours après de me dire merde, j'avais acheté des avocats mais je- je les aient rangé où ? [En fait] comme ils sont noir au fond de mon caddie noir, ils sont restés depuis deux jours dans mon caddie. Voilà, c'est pour ça que je-- la notion de perdre-- non de perdre, de laisser-- perdre, de laisser gâcher c'est ça des produits que j'ai acheté au marché, spécifiquement ?

*Mme.1:* Ouais, au marché.

*Mr.2:* C'est rare.

*Mme.1:* C'est rare ?

*Mr.2:* Ouais, [euh] c'est rare parce que-- alors les produits que je gâche le plus, je dirais, sont les agrumes et le raisin. Pourquoi ? Parce que ce sont des des fruits que j'achète pour en avoir de façon un peu façon piquorage [euh] aisément. [Euh] je les laisse devant moi, je suis absent plusieurs jours, j'ai pas le réflexe de les mettre au réfrigérateur. Et donc là oui, ils peuvent être un peu gâtés quand je reviens. Et transformer un agrume gâté, c'est pas extraordinaire. Autant la pomme y a un morceau qui est pourri, on vire et le reste sera parfait. Autant [euh] un agrume gâté, je n'arrive pas à- à trouver une solution. Le raisin [euh] ouais, le raisin [bah] non, pareil je vais pas faire du vin, je vais pas faire du cidre, du cidre c'est avec des pommes pardon.--

*Mme.1:* [Rire].

*Mr.2:* [Euh] je voulais dire du vinaigre. Non je vais rien faire. Voilà. Mais c'est pas-- c'est pas très souvent.

*Mme.1:* D'accord.

*Mr.2:* Et puis, [euh] c'est pas très souvent parce que quand même dans la majorité des cas, lorsque j'achète les produits, ils sont mangés dans les 48h, parce que je- je suis gourmand.

*Mr.2:* Vous voulez un carré de chocolat ? Non vous voulez pas, je vais pas insister lourdement. Sinon j'ai l'impression que je-- d'être comme ma mère que vous voulez. Comme vous voulez.

*Mme.1:* Je peux me laisser tenter.

*Mr.2:* Il y a différents parfums. Vous faites ce que vous voulez, vous ouvrez.

*Mme.1:* Je prends ce qui a été ouvert.

*Mr.2:* Ah bon, non. Mais non, mais non tenez, celui-là, il est très bon, c'est [euh] rocher au lait. Allez-y, tapez dedans. C'est noix de coco si vous en voulez, vous prenez aussi allez-y.

*Mme.1:* Merci. Et quand vous êtes amené à les jeter. Qu'est ce que vous éprouvez ?

*Mr.2:* J'ai une pensée pour ma grand-mère.

*Mme.1:* Ouais pourquoi ?

*Mr.2:* On ne jette rien, on ne jetait rien. Elle devait être née en 1916 un truc comme ça, morte en 1983. [Euh] non, on jette-- on jette pas. Jeter un produit qu'on a acheté, c'est exactement comme jeter des pièces dans la poubelle.

*Mr.2:* Voilà il y a ça. Ça c'est la première-- mon premier sentiment. Le second, c'est [euh] alors je jette dans la poubelle oui, mais [alors] est ce que je pars en déplacement ? Parce que selon l'état d'avancement de ma poubelle, si je mets des produits frais, légumes ou enfin fruits à la poubelle, je ne veux pas laisser pourrir pendant trois jours dans la poubelle.

*Mr.2:* Donc, [euh]. Pardon.

*Mr.2:* Allez-y.

*[Conversation téléphonique qui a interrompu l’entretien]*

Mr.2 : Pardon, on y va, on continue, désolé.

*Mme.1:* Pas de souci. Vous disiez que quand vous jetiez-

*Mr.2:* Je pensais à ma grand-mère.

*Mme.1:* La grand-mère, c'est les pièces qui part a la poubelle et après il y avait le départ en déplacement.

*Mr.2:* Le départ en déplacement qui fait que ma poubelle, [euh] [en fait]- [en fait] d'un côté ça m'emmerde de jeter des trucs qui vont pourrir dans la poubelle. Si ma poubelle est presque neuve, je suis obligée de la- la- la vider alors qu'elle est pas pleine. J'ai un truc-- truc de débile, il y a des potes qui m'ont déjà dit que j'ai un vrai problème c'est que je peux dépenser. Je regarde pas la dépense, j'achète du vin, j'achète des tableaux de-- Voilà machin, par contre, ça me fait chier de jeter un sac poubelle à trois centime et demi qui est pas-- hein ?

*Mme.1:* Qui est pas plein.

*Mr.2:* [Bah] qui est pas plein. Exactement. C'est logique, non ?

*Mme.1:* [Rire] je suis peu pareille.

*Mr.2:* [Euh] voilà. Qu'est-ce que j'ai à dire d'autre là dessus sur ce point précis, sur ce à quoi je pense quand je mets à la poubelle ? [Euh] non, rien qui me vient.

*Mme.1:* OK. Si on prend une deuxième photo.

*Mr.2:* Oui.

*Mr.2:* Tac je prend celle-ci, diamétralement opposée. Oh là [euh] on a l'impression que c'est la caméra qui la prise. La caméra de surveillance qui a pris d'en haut la photo d'un rayon de fruits et légumes dans un-- Alors je dirais pas un supermarché, vraiment, si-- [alors] ça se veut être un supermarché. Mais vu comme ça, je dirais que ça a l'air d'être un supermarché plutôt qualitatif. Enfin- enfin j'en sais rien moi. Parce que [en fait], ce qui fausse la lecture, c'est qu'il y a pas d'enseigne, pas de marque. Ça n'oriente pas [en fait] le jugement de la qualité, vous voyez ce que je veux dire ?

*Mme.1:* Ouais.

*Mr.2:* [Euh] pre-- première lecture comme ça, je me dis tiens, c'est [euh]-- c'est du [euh] un peu style-- ça pourrait ressembler à du Monoprix en région parisienne, mais c'est le sol-- le sol marron comme ça, c'est pas courant, ça fait un peu tradi. Alors est-ce-- ça-- le- le sol marron pourrait laisser à penser que c'est un- un lieu de vente de producteurs. Vous voyez ce que je veux dire ?

*Mme.1:* Ouais.

*Mr.2:* Un peu comme chez ici là. Mais vu la quantité et vu comment c'est rangé, j'y crois pas trop. J'y crois pas trop. Non parce que-- parce qu'un lieu de vente de produits-- de produits de producteurs ça-- par essence, ça veut dire on-- qu'ils viennent de proximité. Et là, il y a beaucoup trop de diversité pour que ça soit que de la proximité.

*Mme.1:* Oui.

*Mr.2:* [Euh] je vois qu'il y a des balances. Les balances, pourquoi il y a des balances comme ça ?

*Mr.2:* [Rire].

*Mr.2:* Attends, je vois des balances, des balances sur lesquels on pèse et on voit le poids, mais on s'en fout. Je vois pas le truc d'étiquettes, alors il y a un support pour mettre-- pour les sacs en plastique. Donc la photo date un petit peu parce qu'il a des sacs en plastique. On dirait ils ont toujours des sacs en plastique. Ah oui, mais ils sont pourissables. Mais vous savez, je vais essayer de faire comme ça. Essayer d'agrandir [rire] spontanément ce que j'ai envie de faire c'est d'agrandir. Je dis à l'enregistreur, je pose mes doigts dessus et j'essaye d'agrandir la photo, mais ça marche pas.

*Mme.1:* Non.

*Mr.2:* [Euh] un support papier, c'est un iPad en panne. Alors il y a le support avec machin-- mais alors je vois pas comment fonctionne leur balance. J'ai jamais vu ça en France, donc j'en déduis que c'est peut-être pas en France. Et la tonalité du carrelage me laisse à penser qu'on est plutôt en Europe du Sud.

*Mme.1:* C'est possible, mais effectivement c'est pas-- c'est pas en France je crois.

*Mr.2:* Non, parce que-- parce que la balance, ça-- c'est-- elle a- elle donne l'impression d'une indication. Je sais combien j'ai pris. Mais la pesée se fait peut-être en caisse.

*Mme.1:* Ouais.

*Mr.2:* Pour une question, pour éviter que les gens ne grugent. Nombre de fois ou j'ai vu ça dans les magasins. En tout cas, mon Leclerc centre ville des gens qui y vont peser, deux pommes qui vont en rajouter 12 dans le sac.

*Mr.2:* [Rire].

*Mr.2:* Mais les caissières, si c'est une caissière qui a un peu de bouteille, elle est pas con. Et esclandre machin sécurité tout ça.

*Mme.1:* [Rire].

*Mr.2:* D'ailleurs à ce sujet, moi je-- quand je peux ne pas prendre de sac, je ne prends pas de sac.

*Mme.1:* Ouais.

*Mr.2:* Si j'achète par exemple à un- un régime de bananes, je vais étiqueter directement sur le régime de bananes.

*Mme.1:* Ouais.

*Mr.2:* La botte de poireaux pareil. Enfin, tout- tout ce qui est tout ce qui peut éviter l'emballage, oui. Quand je vais chez Sephora, j'achète un flacon de parfum.

*Mme.1:* Ouais.

*Mr.2:* Je le déballe en caisse, je leur laisse le carton, je pars avec le --

*Mme.1:* Juste le--

*Mr.2:* [Ben] oui, je suis venu acheter du parfum, pas un emballage. Regardez- regardez ici dans mon-- le coin de ma cuisine, là le nombre de cartons qu'il y a. Non, mais lui je le rappellerai plus tard. [Euh] je te rappelle plus tard. Voilà. [Euh] la quantité de déchets que l'on produit. C'est hallucinant. Les capsules Nespresso.

*Mme.1:* Ouais.

*Mr.2:* Il y a déjà plusieurs mois, je leur avais dit-- j'avais mon- mon panier comme ça, je dis "Bah écoutez, vous savez quoi ? On va déballer parce que moi, les emballages, je préfère qu'ils soient chez vous, quand vous en aurez marre d'en avoir, Peut être que vous en produiraient moins". Et parce que le jour ou on paiera vraiment effectivement au poids de ce que chaque ménage produit, là on verra notre copie.

*Mme.1:* Ouais.

*Mr.2:* Et donc je leur ai dit "Je prends que les capsules", ils m'ont dit "oui, mais vous êtes obligés de prendre"-- ils m'ont découpé un- un endroit ou il y a la DLC--

*Mr.2:* Ouais.

*Mr.2:* Les dates [euh] de façon industrielle sur les trucs Nespresso, parce que légalement ils n'ont pas le droit de laisser partir des clients sans cette information là. Ils me disent après je peux très bien mettre dans la poubelle au coin de la rue, on s'en fout. Mais [euh] vous êtes obligés de partir avec. Je dois descendre, là-dedans il y a encore du pif, Il faut descendre à la cave, c'est autre chose.

*Mme.1:* [Rire].

*Mr.2:* Donc emballage, ouais. [Euh] il y a de la diversité. [Euh] le vert est mis-- il y a les produits qui sont réfrigérés apparemment. Sur un présentoir, je pense réfrigéré. Donc c'est doublement la raison pour laquelle je pense qu'on est peut-être dans l'Europe du sud ou il y a tous les systèmes peut-être de- de brumisation pour garder les produits frais etc., tout le vert, on voit que c'est de la salade, des trucs comme ça et les autres les fruits et peut-être des légumes qui nous craignent pas sont au milieu. Ouais d'ailleurs on voit que c'est pas chez nous, la gueule du chariot, c'est pas un chariot comme chez nous ça. [Euh] voilà,. J'ai pas grand chose d'autre à dire, je crois en regardant la photo.

*Mme.1:* Ouais. [Euh] du coup, vous avez raison. C'est pas-- je crois que c'est pas chez nous. [Euh] l'idée, c'est de vous évoquer une grande surface.

*Mr.2:* Oui, oui.

*Mme.1:* Est-ce que--

*Mr.2:* J'en ai fait des caisses alors que--

*Mme.1:* C'est très bien. C'est bien détaillé. Est-ce que vous, ça vous arrive de-- d'aller en grandes surfaces acheter vos fruits et légumes ou pas ?

*Mme.1:* Bah bien sûr. Mais je ne vais pas aller en grandes surfaces pour n'acheter que des fruits et légumes. Si je dois acheter que des fruits et légumes, je vais aller chez le primeur du coin.

*Mme.1:* OK.

*Mr.2:* Ou je casse mon PEL pour acheter des tomates cerises Rabelais ou des trucs comme ça. Mais bon, j'en ai-- j'en ai besoin tout de suite et- et j'ai, j'ai pas de temps à perdre. Donc je vais au plus pratique pour moi. La praticité, c'est- c'est l'élément essentiel dans le choix du lieu d'achat.

*Mme.1:* Ouais.

*Mr.2:* [Euh] je vais en supermarché, [euh] et je vais en profiter pour acheter des autres [euh] basiques dont j'ai besoin.

*Mme.1:* Oui. Et du coup, ça veut dire que quand vous allez en grandes surfaces, c'est que vous avez besoin de- de diverses choses.

*Mr.2:* Oui.

*Mme.1:* Et vous en profitez pour prendre des fruits et légumes.

*Mr.2:* Exactement, c'est ça.

*Mme.1:* Mais c'est pas parce que vous avez besoin des légumes que vous allez à la base en grande surface. S'il y a besoin que de fruits et légumes--

*Mr.2:* Non, non, non.

*Mme.1:* Et [euh] quand vous êtes en grande surface, comment vous vous sentez ? Par rapport au marché, est-ce que c'est le même rapport ou pas du tout ?

*Mr.2:* Ça a rien à voir. Enfin, rien à voir, rien à voir, rien à voir. [Euh] je vous ai dit qu'au marché, effectivement, j'y vais [euh] avec le prétexte de voir du monde. Et puis je suis-- le marché, c'est le dimanche. Il y a le marché [inaudible 00:26:48] le mardi aussi. Mais j'y vais jamais parce que mardi, je bosse. Le dimanche est un moment de détente. [Euh] la grande surface en l'occurrence, ça va être soit Leclerc du centre-ville, soit Carrefour Express rue Amiral Linois. Enfin, les plus proches toujours. [Euh] j'y vais au pas de charge.

*Mme.1:* Oui.

*Mr.2:* Je marche vite.

*Mme.1:* OK.

*Mr.2:* Ouais. Je marche vite. [Euh] au marché, je flâne.

*Mr.2:* Ouais.

*Mr.2:* On marche-- et puis il y a la foule. Il y a l'effet de masse qui fait que bon, si on veut marcher vite, on peut dans l'absolu, mais il faut un peu slalomer. [Euh] supermarché, je marche toujours vite parce que je veux vite me débarrasser de ce que je considère comme une corvée.

*Mme.1:* OK.

*Mr.2:* Pour autant, je garde le contact visuel avec le-- les gens dans les allées au cas où je connaîtrais quelqu'un pour discuter. Mais c'est assez rare que je rencontre des gens que je connaisse au supermarché.

*Mme.1:* Oui.

*Mr.2:* Ou alors les personnes que je croise sont comme moi, elles sont pressées, elles ont pas de temps à perdre. Et donc on va pas rester bavasser pendant des heures.

*Mr.2:* Permettez, deux secondes.

*Mme.1:* Ouais allez-y.

*[Conversation téléphonique]*

Mr.2 : On disait quoi ? Ouais les gens son speed aussi dans le magasin et donc [euh] on perd pas de temps.

*Mme.1:* Ouais.

*Mr.2:* Et mon état d'esprit, c'est de faire toujours le même circuit dans le magasin aussi.

*Mme.1:* D'accord.

*Mr.2:* Je commence toujours au même endroit, par la lessive. Lessives, blanchisseurs assouplissants, etc. Je remonte dans l'allée du- du papier fesses. [Euh] je vais faire un tour aux boissons [euh]-- enfin bref, c'est toujours mon parcours pour-- histoire de rien oublier quoi.

*Mme.1:* OK.

*Mr.2:* C'est-à-dire la profusion d'offres du supermarché ne m'atteint pas parce que je sais ce que je veux. Tout au plus, [euh] je vais aller à un moment donné en rayon des boîtes de conserve, maquereaux, thons catalanes, sardines laquelle je prends j'en sais rien. Et là, c'est allez celle-là cette fois-ci.

*Mr.2:* OK.

*Mr.2:* [Euh] plutôt des produits simples, mais bon ils sont-- ils sont tous transformés, évidemment.

*Mme.1:* Oui. Et [euh] qu'est-ce que vous pensez des produits donc des fruits et légumes que vous trouvez au supermarché ?

*Mr.2:* Ils sont de belle qualité.

*Mme.1:* Ouais.

*Mr.2:* [Euh] ils sont-- ils sont-- ils sont propres, ils sont beaux. Alors a-- après, bon il y a évidemment du personnel qui fait-- ils sont-- ils sont assurément moins bien traités qu'au marché. Parce qu'au marché, on est servi généralement par le marchand, le responsable de l'étal, alors qu'au supermarché c'est tripoté toute la journée.

*Mme.1:* Oui.

*Mr.2:* C'est certainement ce qui fait que il y a-- [bah] on arrive, il y a des trucs qui sont pourris, machin, il suffit que la- la nana ou le gars du rayon ne soit pas passé pour faire le petit nettoyage qui va bien. Oh des fois, on peut avoir des fruits un peu abîmés mais sinon dans l'ensemble les prix sont sages quoi. Je- je vois un exemple [euh] là il y a plus de raisin muscat là, on est arrivé à la fin de la saison de production aux raisins de muscat. Ça, c'était un petit truc sympa en octobre-novembre, j'en achetais beaucoup. [Euh] mon produc-- mon marchand [euh]-- Alors au marché, je crois qu'on était dans les 6,50 ou sept euros le kilo.

*Mme.1:* Oui.

*Mr.2:* Ce qui faisait hurler ma maman qui disait "Ouais ça va pas moi, j'achète à- à trois euros le kilo".

*Mme.1:* [Rire].

*Mr.2:* Ouais mais il est moins bon, alors c'est peut-être faut il être aussi bon. Et au- au su-- au supermarché, je trouvais facilement à 1,50 euros ou 2 euros de moins au kilogramme et à la corbeille d'or aux halles Saint-Louis, où là c'est du racket, hein, la dernière fois que je l'ai regardé, il était à 11,95 je crois le kilo.

*Mme.1:* Ah ouais.

*Mr.2:* C'est-à-dire-- c'est à dire [euh] 83 francs le kilo. Vous êtes trop jeune, vous n'avez pas connu ça [rire]. Donc [euh] sinon, voilà la qualité est là, le prix est raisonnable, la quantité, la variété, il y a de la disponibilité. [En fait], quel que soit le plat que je vais préparer, je sais qu'en allant chez Leclerc, je vais tout trouver. Alors que chez Carrefour Express un peu moins. Il peut manquer des fois des produits ou j'ai moins de choix [quoi].

*Mme.1:* Et [euh] est-ce que ça vous arrive de perdre des produits que-- de perdre, les jeter.

*Mr.2:* Oui, de gâcher.

*Mme.1:* Des produits que vous aviez achetés au supermarché ? Les fruits et légumes.

*Mr.2:* Le- le comportement est exactement le même là-dessus que ceux du marché. C'est-à-dire, je vais pas être plus attentif parce que j'ai payé plus cher, moins cher, non, ça n'a pas l'impact.

*Mme.1:* Ça marche. Alors si on en prend une [euh]-- une autre photo.

*Mr.2:* Une autre photo, tac. [Euh] plantes potagères. Ça, c'est un-- oh c'est choupi ce petit jardin-là. [Euh] alors [euh] je suis pas certain que ça soit chez un particulier. Il y a une information de plantes potagères, donc c'est un petit jardin. Soit c'est un jardin partagé, soit c'est le jardin d'un restaurateur dans un beau parc de-- d'un château. [Euh] il fait pousser à la fois pour avoir ces produits frais à domicile et il le fait savoir.

*Mme.1:* [Rire]. Ouais.

*Mr.2:* Vous êtes d'accord avec moi ou pas ?

*Mme.1:* Ouais c'est pas mal.

*Mr.2:* [Euh] qu'est-ce qu'il y a dessus ? C'est peut-être des herbes-- ils disent plantes potagères. Peut-être des herbes aromatiques, c'est quoi ? Je vois pas si bien ce que c'est comme feuilles, alors derrière du poireau ou de l'oignon, on sait pas bien. Derrière des côtes de blettes, peut-être. Devant est-ce que c'est du chou ? Premier plan, je sais pas ce que c'est. Derrière, on a l'impression que c'est-- alors soit c'est de la vigne, ça a l'air d'être de la vigne. Au début, je pensais que c'était des-- vous savezdes- des- des poir-- des poiriers, des pommiers en espalier là. On met contre un mur. Mais là, je pense ça ressemble plutôt à de la vigne.

*Mr.2:* [Euh] le reste, on ne voit pas bien ce que c'est, mais bon, il y a le petit che-- chemin autour pour aller se promener-- enfin et cueillir. Mais voilà, il y a-- il y a le- le double-- c'est à la fois, c'est- c'est de la déco et de l'animation au-- à destination de la clientèle parce que c'est à-- c'est- c'est c'est dans l'air du temps. C'est rigolo, bio, beau, voilà, c'est proximité. On le fait sa-- on le fait savoir que les produits sont de là. Et puis le- le chef ou le-- l'équipe de cuisine a tout à disposition sans avoir à s'emmerder à acheter du cerfeuil, du persil plat, des machins truc comme ça.

*Mr.2:* Et puis il y a un mur en pierre derrière, [euh] je sais pas, bizarrement- bizarrement, je-- alors je- je- je suis tordu, mais moi je vois pas ça en Bretagne. Je vois ça plutôt dans-- je situe ça dans la vallée de la Loire ou un peu plus bas parce que la couleur de la pierre est peu claire. Et puis le mur-- le mur dans sa partie basse, a l'air d'être un peu [euh] comme, vous savez quand- quand vous arrosez une surface avec une eau ferrugineuse et ça fait des traces rouges, etc. Un peu rouille. Ça me fait penser à ça. Voilà.

*Mme.1:* OK. Et qu'est-ce que vous pensez du fait de- de produire soi-même ses légumes ?

*Mr.2:* Produire ou alors à avoir à disposition comme ça, il m'est arriv-- il m'arrive tous les ans d'acheter un plant de basilic chez Leclerc et de le garder à la maison, de l'arroser tout doucement et de- de façon à avoir du basilic frais. Parce qu'en fait, je trouve ça très bien, parce que [euh] [en fait], je- je suis [euh] il y a-- il y a-- incohérent dans mon comportement puisque [euh] chaque fois que j'achète des herbes emballées individuellement dans la petite barquette en plastique avec le plastique par-dessus au rayon frais, il y a toute une gamme extraordinaire, chaque fois que j'achète ça, ça va à la poubelle.

*Mme.1:* D'accord.

*Mr.2:* Parce que j'ai acheté, je m'étais dit tient on a du monde ce soir, on fera des mojitos. Finalement, on est resté sur le champagne ou le pif. On n'a pas fait de mojitos, donc finalement ça reste, ça reste. Et puis au bout de quelques jours, c'est-- ce sont des produits très fragiles, [en fait] les herbes, extrêmement fragiles. Donc [euh], je trouve que c'est très bien de les faire pousser.

*Mme.1:* D'accord.

*Mr.2:* Et d'ailleurs [euh] je vois le succès de ces petites solutions-là, de migno-- micro jardins éclairés [euh] vous trouvez ça chez Ikea, vous trouvez ça dans les magasins de bricolage, vous avez trois pots où vous faites pousser vos trucs. Il y a un petit grand plaid dessus qui fait que voilà ça aide le-- la bébête à pousser.

*Mme.1:* [Rire] Ce sont des solutions d'appartements.

*Mr.2:* Oui, absolument.

*Mme.1:* Est-ce que vous avez déjà vécu des expériences avec des potagers ou pas ?

*Mr.2:* Chez moi, à avoir-- à faire pousser moi ?

*Mme.1:* Oui, vous ou votre entourage.

*Mr.2:* De potager disons que j'ai vécu en maison effectivement et en vie-- vivant en maison, oui j'ai fait pousser du persil, des radis, des petits trucs [quoi], des petits formats.

*Mme.1:* OK.

*Mr.2:* Pas des grands. Après, mes beaux-parents avaient un jardin où lui fait pousser les cucurbitacées, les patates, le machin ça c'est-- c'est presque la petite agriculture familiale. Mais moi [euh], en- en agrément, on va dire oui j'ai déjà eu l'expérience, mais pas de façon [euh]-- comment dire que le terme que j'emploie, systématique.

*Mme.1:* Ouais.

*Mr.2:* Si c'était systématique, j'aurais reproduit avec ma petite solution d'appartement ici.

*Mme.1:* Oui. Et vous pensez quoi des produits qu'on trouve dans les potagers ? Globalement.

*Mr.2:* [Euh] ils sont-- alors savou-- plu-- plus savoureux, plus frais. Parce que justement, ils ne sont pas passés par les étapes de transformation, de conditionnement, d'adjonction de gaz pour les garder. Enfin bref, tout ça. [En fait], on est au plus proche de la nature.

*Mme.1:* Oui.

*Mr.2:* Et en plus, ils ont poussé avec ce que moi, propriétaire de la petite parcelle, je leur ai donné à bouffer.

*Mme.1:* Oui.

*Mr.2:* [Euh] du marc de café peut-être [euh] des- des petits déchets alimentaires. Enfin bref [euh] bon. C'est pas-- ça a pas poussé hors sol, ça a poussé avec du lombric dessous. Si lombric il y a, il y en a de moins en moins, je crois.

*Mme.1:* Oui. OK.

*Mr.2:* Il est 9h45.

*Mme.1:* Ah oui, vous devez y aller ?

*Mr.2:* Je dois être au port à 10 heures. En bas, [euh], j'en ai pour deux minutes à descendre. Il faut juste que je remette ma ceinture, mes chaussures machin. Mais si dans les dix minutes maximum, on peut avoir terminé, c'est bien. Et s'il faut qu'on se revoie, on se reverra.

*Mme.1:* Oui. La dernière ? Ah c'est vous qui la retournez du coup.

*Mme.1:* [Rire] oui.

*Mr.2:* Alors je pense, vous êtes déjà vu chez le coiffeur.

*Mme.1:* [Rire].

*Mr.2:* Il est chauve.

*Mr.2:* Vous allez dire quoi ?

*Mme.1:* Non, j'allais dire "c'est vrai ?", parce que c'est une des seules photos qui est de Brest.

*Mr.2:* Ah non, c'est une blague [euh] il est chauve, il est chauve.

*Mme.1:* [Rire].

*Mr.2:* Alors légumes, légumes, alors là il y a très peu de choix. Là, on est vraiment-- alors attends. Alors, j'ai envie de dire qu'on est sur un- un magasin de proximité. On est locavores et avec, [euh] mais c'est le-- non. Alors je pense pas que ce-- j'hésite à dire, c'est pas un jeu de devinette, il y a pas de bonnes ou mauvaises réponses, j'ai bien compris. Première approche, je m'étais dit, c'est [euh] comment ça s'appelle peut-être comme chez ici, là.

*Mme.1:* Il y a [Prénom].

*Mr.2:* [Prénom] est là ? Salut camarade, je vais lui donner un pain au lait, il a le droit. [Euh] alors, non, non attends je vais lui donner une craquotte parce que j'ai du beurre rance ici et le beurre rance c'est dommage de le jeter. Donc, en plus, c'est l'hiver. Donc je lui fais une bonne grosse tartine. Avec tout ça dessus. Que si je vous donne des craquootes seules, il dit que ça a pas de goût. Je le vois à son comportement. Tiens mon pépère. Alors attends, [euh] non ce n'est pas [Prénom] c'est un autre. C'est pas grave.

*Mme.1:* [Rire]

*Mr.2:* [Euh] c'est-- comment ? Des producteurs locaux qui viennent avec leurs produits mais je crois pas. Non, non. Non, non parce qu'il y a tous des étiquettes qui vont bien, etc. Je pense, c'est juste un- un marchand de primeurs local. Dans- dans un quartier [euh] qui sélectionne-- je pense qu'il sélectionne ses produits, il n'y a pas, il n'y a pas pléthore d'offres de choix.

*Mme.1:* Oui.

*Mr.2:* [Euh] i y a une courgette, il y a un poivron, il y a un machin, les tomates, un petit peu de variété. Ouais, [euh] il n'y a pas de grosses quantités. C'est- c'est-- il y a une théâtralisation, c'est-à-dire, c'est mis en valeur, c'est bien mis en valeur. Mais il n'y a pas une abondance, il n'y a pas-- c'est pas à profusion. Donc je me dis qu'il a une gestion [euh] des circuits courts, des quantités. Il a peut-être pas beaucoup de places de stockage non plus et il n'a pas non plus énormément de clients. Donc ce qu'il met là, ça suffit pour sa clientèle.

*Mme.1:* Ouais.

*Mr.2:* Et je pense à une boutique dans laquelle il y a pas que des fruits et légumes. Ça, c'est-- si c'est marqué légumes, c'est que c'est le rayon légumes. Il y a peut-être un autre endroit où on vend de la picole, des bocaux de pâté, enfin des trucs comme ça (quoi].

*Mme.1:* D'accord.

*Mr.2:* Ça fait penser un peu à ce qu'on appelait chez Mauricette les Vergers du Ponant, à Lambé.

*Mme.1:* OK. Je connais de nom mais je...

*Mr.2:* Où il y a majoritairement des fruits, légumes. Mais il y a aussi de la charcuterie, il y a aussi des œufs, il y a aussi plein de trucs.

*Mme.1:* D'accord. Est-ce que c'est un magasin dans lequel vous vous sentiriez bien ou pas ?

*Mr.2:* Oui, si absolument oui. Mais une fois de plus, je vais y aller-- je vais pas traverser la ville pour y aller. [Euh] je vais y aller parce que c'est pratique.

*Mme.1:* Oui. Et donc vous n'avez pas d'expérience particulière avec ce type de- de lieux ?

*Mr.2:* [Bah] je pense-- Les Vergers du Ponant, justement. Mon expérience, [euh] c'est quoi ? Pourquoi est-ce que chaque fois que je suis allé là-bas, j'y suis allé? Parce que ce n'est pas le plus proche de chez moi.

*Mme.1:* Oui.

*Mr.2:* [Euh] les prix sont sensibles non un peu moins cher que Siam Primeur ou La corbeille d'or eux, ils sont dans le triangle d'or on appelle ça avec une clientèle à fort pouvoir d'achat qui est pas trop regardante, donc ils en profitent. Bon, en même temps, on va dire aussi une clientèle qui est exigeante, qui ne tolérera pas de l'à peu près. Et donc- donc je pars du principe qu'il y a de la qualité et je pense qu'on trouve la même qualité au Vergers du Ponant, à Lambé. Mais c'est pas la même clientèle, c'est pas le même prix de l'immobilier. Résultat, les prix sont plus sages.

*Mme.1:* Oui.

*Mr.2:* Mais mon temps étant compté, je vais pas non plus traverser la ville pour aller acheter mon raisin là-bas. En revanche, si j'y vais, c'est une mutualisation. C'est-à-dire, je vais chez Guillaume Baïkal comment The gusto, l'épicerie caviste et boire un gorgeon avec lui, acheter quelques bouteilles. Et je vais aller chez Ali, le traiteur à côté, pour acheter deux ou trois trucs éventuellement.

*Mme.1:* Oui. OK, alors dernière question. Est-ce que par rapport à tous les- les lieux là qu'on a-- qu'on a vu, est-ce que [euh] il y a, selon la provenance du produit, un effet sur le- le plaisir que vous aurez appris à les préparer ou les manger ?

*Mr.2:* Non. La- la- la provenance de ces produits non, non, non, non. [Euh] bof, bof, non, parce que normalement, dans l'absolu, je vais peut-être pas acheté exactement le même produit non plus.

*Mme.1:* Oui.

*Mr.2:* C'est-à-dire que [euh] la tomate, je vais prendre l'exemple de la tomate, [euh] la tomate que je vais acheter dans le supermarché. [Euh] alors attention, abstraction faite des évolutions, parce que depuis quelques années, ils font des efforts également pour se rapprocher de la qualité des autres. Si je peux avoir des tomates pleines terre que j'ai acheté au marché chez une petite productrice, je sais que la saveur sera toute autre par rapport à de la tomate hors-sol [euh] achetée ailleurs. Et dans l'absolu, ma tomate pleine terre aujourd'hui, je peux la trouver partout.

*Mr.2:* C'est-à-dire je peux la faire pousser dans mon jardin. Je peux l'acheter au marché à mamie qui est en train de vendre ses produits avec-- sur son petit étal. Je peux l'avoir au supermarché parce que le supermarché fait des efforts depuis des années et- et chez mon marchand de primeurs aussi, il peut peut-être avoir rentré des-- comment on appelle ça ? Des vieilles, [euh] des vieilles--

*Mme.1:* Des tomates anciennes ?

*Mr.2:* Des tomates anciennes, des trucs comme ça, effectivement, et qui vont avoir une saveur extraordinaire. Donc c'est pas tant le lieu, c'est le choix du produit lui-même. Mais le lieu ne va pas influer sur la qualité sur mon-- le plaisir gustatif ou le le- le moment que je vais vivre avec le produit quand je vais le déguster.

*Mme.1:* C'est joliment dit [rire].

*Mr.2:* Oh la la la.

*Mme.1:* Très bien, bon on va s'arrêter là pour aujourd'hui. Je vous remercie.

*Mr.2:* N'oubliez pas d'arrêter votre truc. Ça enregistre toujours ?

*Mme.1:* Oui, ça enregistre toujours.

**Household F45, Interview 1**

00:00:00
*Speaker 1:* Trente neuf, en fait. Exactement !

00:00:00
*Speaker 1:* Il y a de quoi faire.

00:00:00
*Speaker 1:* Eh bien, c'est parti, voilà. Oui, il y en a, on a [euh], en fait, on a eu un foyer [euh], il y avait 50 foyers qui avaient été sélectionnés et on a eu un foyer où [euh], la personne au moment où on devait aller [euh], lui installer la poubelle connectée, avait [euh], un souci de santé.

00:00:00
*Speaker 2:* Ah, c’est pas bon ça.

00:00:00
*Speaker 1:* Speaker 1 : Ah, souci de santé du moment. Donc [euh], on a évité, hein, évidemment.

00:00:00
*Speaker 2:* Ah oui, c'était pas la peine hein. C’est pas la peine d’aller s'exposer, c'est clair, hein.

00:00:00
*Speaker 1:* C'est clair. Et du coup, je pense que c'est- c'est- c'est on a du mal à- à reprendre contact avec elle. On ne sait même pas si c’est suite à ces problèmes de santé, ce qui est possible hein. Malheureusement, c'était un peu compliqué. Voilà. Donc c'était juste pour [euh] voilà pour vous rappeler que il y avait cet- cet entretien mais, que c'était anonymisé et vérifié que ça vous convenait. Voilà.

00:00:00
*Speaker 2:* Vous l'avez dit la dernière.

00:00:00
*Speaker 1:* Parfait [euh], donc en fait on va- on va commencer ce petit entretien par une- par une devinette. Voilà. Un jeu [euh], alors c'est moi qui vais devoir deviner. En fait [euh] [euh] je vais vous demander, pensez à un fruit ou un légume [euh] que vous aimez tout particulièrement. Alors, on va peut-être le faire en deux temps puisque vous êtes deux.

00:00:00
*Speaker 2:* Ouais.

00:00:00
*Speaker 1:* Chacun votre tour. [Euh] et en fait vous allez [euh] ben [euh] y penser et puis [euh] en faire une description de manière à ce que j'arrive à le deviner. Donc, l'idée, c'est de me faire deviner votre fruit ou légumes [euh] [euh] préférés. [Euh] au travers notamment ben de ses qualités, de sa description.

00:00:00
*Speaker 2:* Voilà ! Je commence.

00:00:00
*M. [Prénom du conjoint]:* Comme tu commences. Description physique et tout ?

00:00:00
*Speaker 2:* Physique.

00:00:00
*Speaker 1:* Ce qui vous vient à l'esprit. Ce qui vous vient l’esprit.

00:00:00
*M. [Prénom du conjoint]:* Moi, c'est un fruit de forme [euh] plutôt- plutôt allongée, sucré, la plupart du temps sucré.

00:00:00
*Speaker 1:* Hum hum.

00:00:00
*M. [Prénom du conjoint]:* Donc [euh] il faut de temps en temps laisser mûrir.

00:00:00
*Speaker 1:* Okay.

00:00:00
*M. [Prénom du conjoint]:* Et qui a une peau.

00:00:00
*Speaker 1:* D'accord !

00:00:00
*Speaker 2:* Okay.

00:00:00
*Speaker 1:* J'ai- je- je- je vais- je vais creuser un peu. Je pense que j'ai une petite idée. Mûre quand même. [Euh] vous le mangez tel quel ? Cru cuit ?

00:00:00
*M. [Prénom du conjoint]:* Pas forcément. Cru et cuit.

00:00:00
*Speaker 1:* Cru et cuit. D'accord. [Euh] et est-ce qui a des- une préparation à faire avec ce- ce- ce fruit ou ce légume particulier ? Vous avez dit qu'il y avait une peau donc c'est un déguisement, il se mange pas ?

00:00:00
*M. [Prénom du conjoint]:* Non la peau se mange pas.

00:00:00
*Speaker 1:* D'accord.

00:00:00
*M. [Prénom du conjoint]:* Enfin, pas à ma connaissance, en tout cas. Non, je pense pas, on mange pas. Il faut les toucher.

00:00:00
*Speaker 1:* D'accord.

00:00:00
*M. [Prénom du conjoint]:* Qui soit crus ou cuits.

00:00:00
*Speaker 1:* Qui soit cru ou cuit. [Euh] c'est un- un- un fruit ou un légume que vous mangez à quel rythme ? C'est fréquent ou …

00:00:00
*Speaker 1:* Oui enfin, fréquent [euh] oui j'ai quand même [euh] une fois au minimum une fois par semaine.

00:00:00
*Speaker 1:* D'accord. Toute l'année?

00:00:00
*M. [Prénom du conjoint]:* [Euh] oui. Même si on n'a pas toujours des- des- très- très- très bons toute l'année.

00:00:00
*Speaker 1:* D'accord.

00:00:00
*M. [Prénom du conjoint]:* Et pas un fruit qui se cultive ici.

00:00:00
*Speaker 1:* D'accord.

00:00:00
*Speaker 1:* Speaker 2 : Oh là tu donnes beaucoup d'indices là. [Rire]

00:00:00
*Speaker 1:* Et qu'est-ce qui fait que vous l'aimez particulièrement ?

00:00:00
*Speaker 1:* Speaker 3 : Oh non, je pense que ça doit- ça doit être un petit peu dans ma, je sais pas, c'est- c'est un fruit que j'ai connu quand j'étais beaucoup plus jeune et que là j'ai longé sur [euh] je dirais sur le sur pied presque et c'est vrai que ça m'a j'ai toujours- j'ai toujours trouvé très- très bon là. Il y a peut-être maintenant des *réminiscence* de- de ça qui font que j'en mange [euh] ben que je- que je l'aime bien.

00:00:00
*Speaker 1:* Ouais ouais. C'est attaché une expérience en fait, particulière à un moment quoi.

00:00:00
*M. [Prénom du conjoint]:* Oui, j'ai toujours, depuis tout petit, j'ai toujours aimé ce fruit.

00:00:00
*Speaker 1:* Speaker 1 : [Sonnerie téléphone]. Excusez-moi, je suis absolument désolée, normalement je le coupe. Voilà, je coupe ça. Bon, c’est coupé. Okay, bon, je pense que j'ai deviné. Ah, enfin, peut-être, c'est pas sûr. [Euh] pour finir [euh] donc on a parlé de pourquoi vous l’aimer particulièrement. Est-ce que vous avez l'impression que c'est finalement un fruit ou un légume qui a des avantages particuliers et d'un autre côté est-ce qu'il a des inconvénients ?

00:00:00
*M. [Prénom du conjoint]:* [Euh] Inconvénients [euh], non, du tout pas. L'avantage, il est bon.

00:00:00
*Speaker 2:* Il y a autre chose quand même.

00:00:00
*M. [Prénom du conjoint]:* Quoi ?

00:00:00
*Speaker 2:* Tu peux le transporter partout comme ça.

00:00:00
*M. [Prénom du conjoint]:* Oui oui !

00:00:00
*Speaker 1:* D’accord ! Donc, ça fait partie des avantages.

00:00:00
*M. [Prénom du conjoint]:* Oui oui !

00:00:00
*Speaker 2:* Pour ma part, s'en est un.

00:00:00
*M. [Prénom du conjoint]:* Tu devines quel fruit c’est ?

00:00:00
*Speaker 2:* Ah oui.

00:00:00
*Speaker 1:* J'essaye, je me lance. Une banane.

00:00:00
*Speaker 2:* Et voilà, j'étais sûre. C'est pas compliqué un fruits long hein.

00:00:00
*Speaker 1:* Non non c'était très bien décrit dès le début. Et c'est intéressant parce que vous avez dit sur [euh] cette- cette [euh] certain comme ça ou légumes [euh] [euh] qui [euh] qui- qui résonnent d'une manière un peu particulière parce que par exemple on a eu une expérience spécifique avec, on les a mangées sur l'arbre.

00:00:00
*M. [Prénom du conjoint]:* Ah ben quand j'étais petit pour vous dire enfin, quand j'avais une dizaine d'années, 10 ou 15 ans, oui j'en mangeais à peu près [euh] je pouvais dire une demi-douzaine par jour.

00:00:00
*Speaker 1:* D'accord.

00:00:00
*Speaker 1:* Speaker 3 : C'est toujours des…

00:00:00
*Speaker 1:* Speaker 2 : C'était des petites bananes ?

00:00:00
*Speaker 1:* Speaker 3 : Non non non, oui, j’en mangeais sur l'arbre, effectivement j'ai vécu en Martinique pendant trois ans.

00:00:00
*Speaker 1:* Ouais.

00:00:00
*M. [Prénom du conjoint]:* Et dans notre jardin, on avait des bananiers- des bananiers là qui avaient eu des bananes comme ça.

00:00:00
*Speaker 1:* Ouais. Ouais.

00:00:00
*M. [Prénom du conjoint]:* C'est vrai que quand on mangeait une banane mûre, prise sur un bananier, ça n'a rien à voir avec toutes les bananes que vous pouvez manger autrement hein.

00:00:00
*Speaker 1:* Qu’on peut manger autrement.

00:00:00
*M. [Prénom du conjoint]:* Quand vous faites le transport et puis on met tout quoi.

00:00:00
*Speaker 1:* C’est clair.

00:00:00
*Speaker 2:* C’est ma nature.

00:00:00
*M. [Prénom du conjoint]:* Hein, mais c'est ça, oui, c'est ça, ce sont mes fruits préférés.

00:00:00
*Speaker 1:* Préférés ! Bon ben super. Eh bien, à vous le tour.

00:00:00
*M. [Prénom du conjoint]:* : Alors, moi, je suis plus attirée par un fruit rond. Speaker 1 : Hum hum.

00:00:00
*Speaker 1:* Qui peut être, plusieurs couleurs, qui se mangent quasiment toute l'année et qui se prêtent aussi à de nombreuses préparations.

00:00:00
*Speaker 1:* D'accord, donc quand vous dites de nombreuses préparations [euh], c'est pareil, ça peut se manger aussi bien [euh], [euh] cuit.

00:00:00
*Speaker 1:* Speaker 2 : Que cuit.

00:00:00
*Speaker 1:* Speaker 1 : Que cuit.

00:00:00
*Speaker 2:* Un fruit qui peut être incorporé, a pas mal de préparation.

00:00:00
*Speaker 1:* D'accord. [Euh], donc vous avez dit que vous le mangez quasiment toute l'année. C'est quelque chose par exemple que vous achetez [euh], ici ou …?

00:00:00
*Speaker 2:* Oui !

00:00:00
*Speaker 1:* Voilà ! Et qui peut être produit par exemple [euh]…

00:00:00
*Speaker 2:* Oui, c'est ici, c'est en France- c'est en France- c'est en France.

00:00:00
*Speaker 1:* D’accord !

00:00:00
*Speaker 2:* On en trouve ici hein.

00:00:00
*Speaker 1:* D’accord ! Au supermarché par exemple, c'est là que vous l'achetez ou …?

00:00:00
*Speaker 2:* Oh oui essentiellement, quand même ou sur le marché.

00:00:00
*Speaker 1:* Sur le marché. D'accord. [Euh] et alors même question qu'est-ce que vous aimez en fait particulièrement dans- dans ce fruit ?

00:00:00
*Speaker 2:* Bah le petit le- le goût- le goût qui diffère selon les variétés et puis avoir des- des fruits très sucrés, d'autres qui le sont moins certains- certains qui ont un petit coup acide, c'est surtout ce côté-là.

00:00:00
*Speaker 1:* D'accord, donc la variété en fait, la diversité qu’on a le même fruit en fait.

00:00:00
*Speaker 2:* Voilà ! On s'en lasse pas parce que finalement au fil des mois, on trouve différentes variétés donc on a l'impression en fait [euh] de changer [euh], presque de fruits en fonction des- en fonction des- des mois ou des saisons.

00:00:00
*Speaker 1:* D'accord. Et du coup [euh], un peu la même question, est-ce que [euh], il y a un sentiment particulier en fait quand vous [euh], vous mangez ce fruit, est-ce qu'il a, rattaché à quelque chose de- de particulier ?

00:00:00
*Speaker 2:* Ah, spécialement, je pense que c'était un fruit effectivement que, qui était pas mal consommé quand j'étais petite aussi. Parce qu'on en trouvait quand même, il y avait eu- il y avait moins de fruits, je pense, il y a 30, 30, 40 ans que maintenant.

00:00:00
*Speaker 1:* Oui !

00:00:00
*Speaker 2:* 49, 50. On va dire ça comme ça.

00:00:00
*Speaker 1:* D'accord. Et du coup, en- en quelques mots [euh], ces avantages et éventuellement des inconvénients s'il y en a ?

00:00:00
*Speaker 2:* Des avantages déjà, c'est qu'on le trouve pratiquement toute l'année. Il est facile aussi à transporter et puis il se prête à pas mal de- de préparation.

00:00:00
*Speaker 1:* La préparation. La diversité des préparations et la variété [euh].

00:00:00
*Speaker 2:* Oui oui !

00:00:00
*Speaker 1:* D'accord. Alors c'est pareil je vais tenter ma chance. Je pense que c'est une pomme.

00:00:00
*Speaker 2:* Voilà. Mais déjà vous avez quelques indices quand vous regardez la corbeille.

00:00:00
*Speaker 1:* Je me suis empêchée de la regarder. C’est bien parce que ça veut dire que…

00:00:00
*Speaker 2:* Vous voyez qu’il y a des bananes et des pommes.

00:00:00
*Speaker 1:* Exactement ! Mes deux préférés.

00:00:00
*Speaker 2:* Bon, ceci étant [euh], moi j'aime beaucoup les fruits [euh], fruits et légumes. Il y a plein de fruits qu'on aime bien hein.

00:00:00
*Speaker 1:* Oui oui oui.

00:00:00
*Speaker 2:* Toi aussi, t'aimes bien les fruits. Vous en mangez [euh]…

00:00:00
*Speaker 2:* Tous les jours.

00:00:00
*Speaker 1:* Tous les jours.

00:00:00
*Speaker 2:* Tous les jours. C'est vrai que ouais, ouais, ouais.

00:00:00
*Speaker 1:* Et du coup, on va parler un petit peu de votre façon de les [euh] les acheter. Alors là plus généralement hein que ça soit fruits, mais légumes aussi hein puisque on- on est vraiment sur ces deux [euh] [euh] ces- ces deux idées. [Euh] et globalement [euh] voilà, quel est votre [euh], alors déjà est-ce que vous faites tous les deux les courses alimentaires ou est-ce qu'il y en a plutôt un de vous ou plutôt que l’autre.

00:00:00
*Speaker 2:* En général, on y va pratiquement tous les jours. Tous les…

00:00:00
*M. [Prénom du conjoint]:* En ce qui en ce qui concerne fruits et légumes, c'est quand même toi qui fait le…

00:00:00
*Speaker 2:* C'est plus moi qui, c'est plus moi qui me charge.

00:00:00
*M. [Prénom du conjoint]:* Moi je suis beaucoup plus, bon, les pommes j'aime bien les pommes, mais j'aime pas, je n'aime pas toutes les variétés de pommes, donc je vais rester [euh] je suis assez traditionaliste.

00:00:00
*Speaker 1:* Hum hum.

00:00:00
*M. [Prénom du conjoint]:* J'aime bien, je mangerais, je pourrais manger- je ne pourrais manger que celle-là par exemple, ça ne me dérangera pas.

00:00:00
*Speaker 2:* Comme tu les trouves pas toute l’année, ben, tu es obligé de…

00:00:00
*M. [Prénom du conjoint]:* Non mais je suis heureux. Mais je suis, bon, c'est vrai qu'autrement [euh], aussi quand-quand Martine me les épluche et qu'elle me coupe je les mange.

00:00:00
*Speaker 1:* D'accord. Donc, il y a aussi la- la- l'aspect préparation. On va y en revenir. [Rires]. Alors, très très concrètement je sais pas, quel est votre organisation en termes de course par exemple, est-ce que vous faites vos courses [euh] donc vous m'avez dit que vous faisiez des courses quasiment tous les jours.

00:00:00
*Speaker 2:* Quasiment !

00:00:00
*Speaker 1:* Est-ce que c'est au même endroit à chaque fois ? Est-ce qu'il y a des choses particulières par exemple des alternances marchés, supermarchés ? Enfin, comment ça se passe, en fait ?

00:00:00
*Speaker 2:* Ben en fait on fait les- l'essentiel des courses, c'est plutôt Leclerc hein le coût de la Miso. Et puis le vendredi, on va sur le marché [euh].

00:00:00
*Speaker 1:* D'accord.

00:00:00
*M. [Prénom du conjoint]:* Et sur le marché quand même Miso pour acheter les, souvent, c'est pour acheter des pommes. La plupart du temps, c'est pour acheter les pommes. Mais c'est toujours aussi le même [euh] le même étale.

00:00:00
*Speaker 2:* Ouais.

00:00:00
*Speaker 1:* D'accord.

00:00:00
*M. [Prénom du conjoint]:* Parce qu'il y en a, bon, c'est quand même celle-là, c'est quand même cette personne-là qui est plus souvent, maintenant, il y en a plusieurs. C'est- c'est vrai que c’est- c'est rare qu'on change.

00:00:00
*Speaker 2:* Après on a ses habitudes aussi je pense, qu'il y a une confiance qui s'installe [euh].

00:00:00
*M. [Prénom du conjoint]:* Faudrait pas la nommer, c'est le verger de Carambrou.

00:00:00
*Speaker 1:* D'accord, donc c'est un producteur local.

00:00:00
*Speaker 2:* Oui, oui, oui !

00:00:00
*Speaker 1:* D'accord.

00:00:00
*Speaker 2:* En fait, je crois que elle- elle produit des- des fruits.

00:00:00
*Speaker 1:* Hum hum.

00:00:00
*Speaker 2:* Mais elle en achète également auprès de son frère qui doit exploiter [euh] je sais pas si c'est pas en *Anjou*.

00:00:00
*M. [Prénom du conjoint]:* Ouais, mais les pommes elle en a parce que…

00:00:00
*Speaker 2:* Les- les pommiers, elle en a.

00:00:00
*M. [Prénom du conjoint]:* Puisqu'ils faisaient des…

00:00:00
*Speaker 2:* Pommes mes poids.

00:00:00
*M. [Prénom du conjoint]:* Bon là maintenant, ils peuvent plus en faire à cause de la pandémie, mais tous les ans, ils faisaient deux fois par an.

00:00:00
*Speaker 2:* Non, une fois par an.

00:00:00
*M. [Prénom du conjoint]:* Une fois par an, il fait que vous alliez chez eux une canette [euh].

00:00:00
*Speaker 1:* D'accord.

00:00:00
*M. [Prénom du conjoint]:* Vous vous cueillez carrément les- les, vous, une caisse quoi et vous cueillez ce que vous voulez cueillir [euh].

00:00:00
*Speaker 2:* Ben là, directement quoi.

00:00:00
*Speaker 1:* Ça vous est déjà arrivé d'y aller ?

00:00:00
*Speaker 2:* Oui.

00:00:00
*Speaker 1:* Ouais ? D’accord ! Et [euh], cette question vient tout de suite à l'esprit donc [euh], je vous la pose. Pour vous est-ce que il y a une différence entre [euh], [euh], un fruit que vous avez acheté et un fruit que vous avez comme ça cueilli vous-même, que ça soit par exemple dans ce contexte-là de cueillette ou [euh], dans votre jardin si vous avez [euh].

00:00:00
*Speaker 2:* Ben, nous, on n'a- on n'a pas de- d'arbres fruitiers dans le jardin. [Euh] Je pense pas qu'on fasse une très grosse différence parce que c'est pas produit chez nous. On s'en est pas occupé [euh] toutes, pendant plusieurs mois. Mais c'est très agréable quand même d'aller cueillir les- les fruits directement, voilà.

00:00:00
*M. [Prénom du conjoint]:* Ceci dis, le code [euh] le code [euh] le creux que l'on produit nous [euh] sans doute, c'est du chauvinisme, je trouve que c'est meilleur que- que ce que j'achète.

00:00:00
*Speaker 1:* Ouais.

00:00:00
*M. [Prénom du conjoint]:* Même si c'est pas bon. Malgré que ce sont que des radis ou des…

00:00:00
*Speaker 2:* Ouais, mais je pense que c'est parce que- parce que on.

00:00:00
*M. [Prénom du conjoint]:* Oui, c’est vrai que c’est nous qui avions…

00:00:00
*Speaker 2:* On a consacré du temps.

00:00:00
*M. [Prénom du conjoint]:* Oui, oui !

00:00:00
*Speaker 2:* On a réfléchi à la…

00:00:00
*M. [Prénom du conjoint]:* On a réussi quand même.

00:00:00
*Speaker 1:* Hum.

00:00:00
*M. [Prénom du conjoint]:* Même si, ça a mis du temps.

00:00:00
*Speaker 2:* C’est pas toujours tout à fait réussi non plus. Il faut dire ce qui est hein. On est pas non plus des agriculteurs donc.

00:00:00
*Speaker 1:* Non mais c'est intéressant de, cette idée que peut-être [euh] le fait de l'avoir fait soi-même c'est enfin, il y a une petite [euh] [Inaudible, 00 :13 :05-00 :13 :05].

00:00:00
*M. [Prénom du conjoint]:* Je croyais pas par exemple pour revenir sur une année d'aller en acheter avec [euh] sur le verger, c'est-à-dire qu'on avait pris beaucoup.

00:00:00
*Speaker 2:* Ouais on avait pris beaucoup.

00:00:00
*M. [Prénom du conjoint]:* Ben ça a pas été le bon plan parce que il y en avait beaucoup qui se sont datés quoi.

00:00:00
*Speaker 1:* Ah.

00:00:00
*M. [Prénom du conjoint]:* Parce qu'il faut avoir un local [euh] presque [euh] presque dédié pour les, quand vous en prenez. Ce qu'on dit que les pommes doivent pas se toucher.

00:00:00
*Speaker 2:* Oui. C’est compliqué hein, c’est vrai que c’est compliqué.

00:00:00
*M. [Prénom du conjoint]:* Il faut pouvoir les étaler toutes sans qu'elle touche l'autre. Alors c'est vrai que ça suppose beaucoup donc [euh] c'est pour ça que vraiment [euh] il y a- il y a des producteurs qui vous proposent une fois par semaine et d'aller acheter des, je ne pense pas, ça dépend des goûts et saveur ou saveur.

00:00:00
*Speaker 2:* Le jardin des saveurs.

00:00:00
*M. [Prénom du conjoint]:* Le jardin des saveurs. Est-ce que ça casse un peu bête hein. On est passé il y a pas longtemps.

00:00:00
*Speaker 2:* Pas le *cabinet*.

00:00:00
*M. [Prénom du conjoint]:* Non, d'une veste.

00:00:00
*Speaker 2:* D’une veste.

00:00:00
*M. [Prénom du conjoint]:* Bon, c’est, il faudrait faire la démarche d'y aller [euh], ah si si, y aller, si si y aller une fois par semaine par exemple bah, je sais pas, je trouve enfin, parce qu'on va se retrouver avec des gros stocks qui vont pas forcément non plus rester bien quoi.

00:00:00
*Speaker 1:* Oui, alors ça me fait rebondir hein, sur ce que vous disiez, le fait pour vous d'aller finalement faire les courses [euh], parce que là si je- je comprends bien la situation, il y a certains produits finalement vous achetez une fois par semaine quand vous allez au marché notamment par exemple, c'est pomme.

00:00:00
*Speaker 2:* Voilà !

00:00:00
*Speaker 1:* Mais je sais pas, il y a peut-être d’autres fruits et légumes que vous achetez plutôt.

00:00:00
*Speaker 2:* Et qui aussi souvent, [euh] du kiwi, des poids aussi, j'achète très souvent.

00:00:00
*M. [Prénom du conjoint]:* Parce que l'avantage par exemple les kiwis, parce que c'est vrai qu'on en- on en mange tous les jours [euh], si vous en achetez [euh] ben [euh] une vingtaine, s'ils sont tous à la même maturité ben les derniers vont être un peu moins.

00:00:00
*Speaker 2:* Et c'est souvent le cas quand on les achète en grande surface. Ou ils sont bien mûrs ou ils sont pas du tout mûres et quand- quand on ne les achète pas du tout mûres ben ils nourrissent pratiquement sur…

00:00:00
*M. [Prénom du conjoint]:* Jusque-là- là elle parlera de panache qu'on lui demande vingt kiwis.

00:00:00
*Speaker 2:* C'est ça l'avantage.

00:00:00
*M. [Prénom du conjoint]:* On va- on va lui demander, est-ce qu'on peut en avoir dix [euh] à peu près un peu plus mûres et puis dix autres qu'on va garder [euh], les mêmes- mêmes deux emballages [euh] distincts des fois.

00:00:00
*Speaker 1:* D’accord !

00:00:00
*M. [Prénom du conjoint]:* Donc c'est vrai que c'est, en plus qu’on fasse le marché ici. Je trouve que c’est- c'est sympa aussi quoi.

00:00:00
*Speaker 2:* C'est le gros avantage d'acheter au marché. Parce que quand vous achetez au Leclerc [euh], vous n'avez pas ce service rendu. Voilà, c'est, le dimanche dernier, j'avais acheté un, on a acheté un melon [euh], le vendredi pour le dimanche. En général, je vais, je les choisis relativement bien, et là, elle me dit, c'est pour quand ? Parce que c'est pour dimanche elle me dit bah, celui-là est parfait. Certes il était plus petit que du, d'autres qui étaient sur les tables, mais c'est vrai qu'il était délicieux.

00:00:00
*M. [Prénom du conjoint]:* Je rappelle, des fois, il faut faire attention parce que il y a une année, on sait que vous avez peut-être abordé un peu du sujet. Il y a une année marquée fait souvent de la confiture d'abricot donc [euh] Leclerc, c'est pas rare que tous les ans. Moi, il y ait une fois- une- une fois des promotions sur les caisses de l'abricot pour qu'on, spéciale confiture. Donc elle a dit tiens on va en prendre. Je regarde- je regarde les cadeaux ben sans mentir j'ai dû en enlever [euh] cinq six sans mentir, dans- dans chaque- dans chaque cagette comme ça, il y en avait au moins 4 ou 5 qui étaient pourris, mais on les voyait hein. J'ai été voir le chef de rayon, [prénom] il y a il y a quand-même beaucoup de- il y a quand même beaucoup d'abricots pourris pour la confiture. Ah oui, oui, pour la confiture, c'est pas grave.

00:00:00
*Speaker 2:* C'est- c'est un peu dommage que de raisonner comme ça [euh] enfin.

00:00:00
*M. [Prénom du conjoint]:* Je trouve que quand-même qu'il y en ait un qu'on ait peut-être pas vu, je dis pas, mais systématiquement je trouve que c'est, et le lendemain, ils étaient toujours là hein.

00:00:00
*Speaker 2:* On s'est dit que peut-être qu'il- il allait les enlever du rayon, même pas.

00:00:00
*Speaker 1:* Ça vous fait quoi ça ?

00:00:00
*Speaker 2:* Ben moi, je suis très [euh] très maniaque je veux dire quand je fais de la confiture hein, c'est- si y a un bout gâté. Je vais enlever, parfois ça m'arrive d'enlever la moitié de l'abricot et ça mènerait pas l'idée de mettre des- des fruits pourris pour faire de la confiture.

00:00:00
*M. [Prénom du conjoint]:* Par exemple [euh] là maintenant depuis la- depuis la pandémie, vous avez des affiches [euh] enfin comme en gros autant que faire sous peu. Prenez le fruit que vous avez touché.

00:00:00
*Speaker 1:* Hum.

00:00:00
*M. [Prénom du conjoint]:* Mais moi je suis désolé parce que en plus dans la grande surface ils ont tendance, c’est pas étonnant s’ils le font systématiquement quand vous avez un cajou de pommes par exemple, elles sont toutes rouges. Si vous prenez la pomme et vous la retournez, bien souvent, de l'autre côté peut-être des fois être blancs.

00:00:00
*Speaker 2:* Ah.

00:00:00
*Speaker 1:* Même des fois, il va être même marqué tout ça. Ben je suis désolé, mais même pandémie ou pas moi je vais pas aller prendre des fruits comme ça [euh].

00:00:00
*Speaker 2:* Moi quand on voit le budget qu'on y consacre, on a pas franchement envie d'arriver à la maison, de- de devoir mettre ses fruits et légumes à la poubelle avant même d'avoir [euh] pousser un dégout.

00:00:00
*M. [Prénom du conjoint]:* Mais on est quand même relativement bien [euh] c'est comme relativement correct, quoi, globalement quoi.

00:00:00
*Speaker 2:* Ouais si, de toute façon, on n’a pas tant que ça l’embarras du choix, sauf à aller euh directement chez le producteur, ça puis, il y a des quand même des contraintes [euh] horaires, des- des jours où on…

00:00:00
*M. [Prénom du conjoint]:* Des fois, c'est des jours particuliers dans la semaine.

00:00:00
*Speaker 2:* On n'est pas forcément disponibles sur ces créneaux-là où ces jours-là. Mais c'est- c'est vrai que c'est ça l'idéal hein quand on peut de l'attacher en circuit de cou, on se rend bien- on se rend bien compte hein que c'est mieux hein. Les fruits se conservent mieux quand ils n'ont pas été [euh] quand ils n'ont pas circulé [euh] mais bon on a pas toujours non plus [euh] que les facilités hein. Il y a pas- il y a pas une pléthore non plus de producteurs locaux aussi hein.

00:00:00
*M. [Prénom du conjoint]:* Des fois, enfin, pour les légumes, c'est souvent des- c'est souvent sous forme de panier.

00:00:00
*Speaker 1:* Hum.

00:00:00
*M. [Prénom du conjoint]:* Donc, vous êtes quand même obligé d'avoir envie de tout ce qui est obligé d'avoir dans le panier quoi, donc c'est vrai que c'est…

00:00:00
*Speaker 2:* Puis on sait pas toujours ce qu'il va y avoir. On avait acheté par intermédiaire de notre fils qui est enseignant, puis, c'est une opération [euh] pour- pour l'école quoi. On dit oui okay, mais c'est vrai quand il m'a amené le cajou déjà je trouvais que c'était, il y avait pas grand-chose pour le prix qu'on avait payé et puis franchement il y avait des légumes, bon on les découvrait certes, mais franchement je sais pas trop quoi faire avec hein.

00:00:00
*Speaker 1:* Ouais.

00:00:00
*Speaker 2:* Donc [euh], et puis bon, je pense que l'école avait fait un petit bénéfice, c'était le but aussi, hein. Mais franchement, je trouvais que c'était- c'était relativement cher pour ce qu'il y avait quoi.

00:00:00
*M. [Prénom du conjoint]:* Et ceci dit, je trouve dommage que le circuit court se développe pas plus [euh], enfin, plus que ça quoi parce qu’il y dans le coin, enfin, on est quand même- on est quand même en région où y a pas mal de, même de petits producteurs et tout hein.

00:00:00
*Speaker 2:* Il y a toute une logistique qui- qui est difficile je pense.

00:00:00
*Speaker 1:* Ouais, il y a différentes- il y a différentes raisons ?

00:00:00
*Speaker 2:* Je pense que c'est compliqué.

00:00:00
*M. [Prénom du conjoint]:* Je trouve que c'est- c'est, enfin d'après ce que j'en vois, je trouve que c'est davantage le consommateur qui doit faire l'effort plus que le- que le producteur.

00:00:00
*Speaker 2:* Moi je suis pas d'accord avec toi quand même parce que pour les producteurs ça suppose qu'ils quittent leurs- leurs exploitations, qu'ils aient un local dédié et qu’ils aient du personnel disponible, qu’ils aient tel créneau.

00:00:00
*M. [Prénom du conjoint]:* On voit des fois dans des émissions vers styles *diesel*, la commission sur des régions des points bien précis, on voit, on a vu, c'est pas rare de voir des- des groupements de petits producteurs et pas forcément que des fruits et légumes hein, ça peut être des- ça peut être des bergers, ça peut être de la viande qui ont effectivement loué ou acheté un local genre [euh], hangar qu'ils ont retapé et qui sont, ils tiennent une permanence. Alors, ça le boucher qui va te servir des fruits et légumes hein, s’il est de permanence et ainsi de suite. Bon, après, c'est souvent aussi, ce qu’il y a, c'est que c’est souvent dans des endroits où il y a pas d'offres non plus, il y a pas de- il y a pas de grande surface, il y a pas de choses comme ça donc les gens sont même obligés de, d'être dépourvu comme ça quoi, mais des fois, j'ai du mal à comprendre pourquoi c'est- c'est- c'est si compliqué que ça.

00:00:00
*Speaker 2:* Bah, seulement moi, je pense que c'est pas ça le problème des producteurs non plus hein.

00:00:00
*M. [Prénom du conjoint]:* Mais c'est souvent ça.

00:00:00
*Speaker 2:* De répondre à ces attentes-là, je pense que c'est pas toujours évident.

00:00:00
*M. [Prénom du conjoint]:* C'est souvent beaucoup plus cher quand même.

00:00:00
*Speaker 2:* Ça dépend.

00:00:00
*Speaker 1:* Il y a un facteur de prix effectivement.

00:00:00
*M. [Prénom du conjoint]:* Des fois, c'est, des fois, c'est bénin. Enfin des fois le, l'exemple est, c'est vrai que quand vous achetez du gingembre qui vient du Chine je comprends très bien. Il a fait 40000 je sais pas, 50000 kilomètres, bon d'accord. Et [euh] un jour, on discutait avec des amis qui ont des- des- des- des copains, des parents qui sont producteurs bio, dans les, ils font du fromage.

00:00:00
*Speaker 2:* Ah oui.

00:00:00
*M. [Prénom du conjoint]:* [Inaudible, 00 :21 :15-00 :21 :17] mandarin. Mais un jour j'ai regardé le lieu où ils gardent les mandarins, au rayon de fromagerie de Leclerc. Il y en avait de cru au kilo.

00:00:00
*Speaker 1:* Ouais.

00:00:00
*Speaker 2:* C'était dans les- dans les 35 euros.

00:00:00
*M. [Prénom du conjoint]:* C'est presque, c’est- c'est un acte volontaire et c'est plus c'est- c'est plus- c'est plus vouloir manger que bio ainsi de suite. Il faut vraiment être militant quasiment quoi et malheureusement, c'est pas donné à tout le monde quoi.

00:00:00
*Speaker 1:* Ouais. Ouais. C’est clair.

00:00:00
*Speaker 2:* Tous les foyers ne peuvent pas.

00:00:00
*Speaker 1:* Et ça, j'ai du mal à comprendre. C’est produit dans les mandarins, donc c'est quand même pas, c'est- c'est même pas à 200 kilomètres d'ici.

00:00:00
*Speaker 2:* Le mandarin, c'est à quoi, à 70 kilomètres d’ici ?

00:00:00
*M. [Prénom du conjoint]:* C'est sûr ça coûte plus cher à produire, en tout cas il y a pas de frais de transport par rapport à d'autres fromages qui viennent de- qui viennent des Pyrénées ou d'ailleurs quoi donc c'est- c'est et c'est toujours, c'est vrai que c'est toujours, c'est ça- c'est ça que j'ai du mal, alors, j'aimerais bien discuter avec des gens qui soient de la profession pour essayer d'avoir un peu leur point de vue.

00:00:00
*Speaker 2:* Nous on connaît peut-être pas non plus tous les- tous les paramètres.

00:00:00
*M. [Prénom du conjoint]:* Non, on connait pas Mais des fois, il y a des différences hein.

00:00:00
*Speaker 2:* Je pense que c'est pas si facile que ça pour les producteurs hein.

00:00:00
*Speaker 1:* On- on- on s'éloigne un petit peu du sujet, mais ce que- ce que vous dites est très intéressant. Et- et [euh] et on- on va travailler [euh] dans peu de temps là, on va démarrer un programme avec, alors sur la- la région de Lorient. Mais [euh] qui porte un petit peu sur ça, c'est-à-dire cette [euh] en fait on se rend pas forcément compte du travail des producteurs et cetera et cetera. Et d'un autre côté les producteurs ne se rendent pas forcément compte des contraintes que ces développements de circuits courts, mais où l'offre est difficilement accessible. On le fait peser sur [euh]- sur [euh] ben sur leur compte client et consommateur et du coup, on aimerait bien voir comment pouvoir [euh] ben pour le coup rapprocher un petit peu. Voilà ce que vous disiez pouvoir échanger on- on- on a un petit peu cette hypothèse cet échange que, il faut reconstruire, rebâtir cet échange, ce qu'on a un peu perdu dans notre société.

00:00:00
*Speaker 2:* Tout à fait !

00:00:00
*Speaker 1:* Alors aussi parce que- ben parce que on va au supermarché, et du coup, le producteur, il est- il est invisible en fait.

00:00:00
*Speaker 2:* Oui, c'est vrai. C'est un peu dommage hein. Oui, c’est un peu dommage.

00:00:00
*Speaker 1:* Voilà. Donc [euh] je dirais à suivre. [Rire]. Très bien. Bon, donc, si je- je- juste je fais un petit récapitulatif là de ce que vous m'avez dit par rapport vraiment [euh] [euh] aux aspects fruits et légumes. [Euh] vous achetez finalement très régulièrement une partie [euh] sélectionnée plutôt sur le marché parce que vous avez finalement des producteurs et des habitudes, voilà. Et que [euh] vous trouvez aussi [euh] [euh] un avantage à aller sur le marché parce que ça vous permet [euh] d'avoir [euh] [euh] une meilleure qualité finalement et un étalement [euh] de- de- du degré de maturité en fait, des produits que vous achetez. Ce qui vous permet finalement dans- dans- dans- de les consommer tous.

00:00:00
*M. [Prénom du conjoint]:* Puis en plus quand vous achetez- quand vous achetez des pommes chez elles [euh] c'est le même prix.

00:00:00
*Speaker 2:* Toutes les variétés sont au même prix. Vous pouvez les panacher. C'est intéressant, ça permet de découvrir aussi d'autres variétés.

00:00:00
*Speaker 1:* C'est ça.

00:00:00
*Speaker 2:* Qu'on aurait pas forcément acheté autrement sans, souvent ça m'arrive, je prends une pomme, de lait, une pomme de lait, puis, on le goûte et puis on se dit tiens ça, c’est pas mauvais.

00:00:00
*Speaker 1:* Alors, la diversité- la diversité de goûter, et cetera.

00:00:00
*Speaker 2:* Tout à fait.

00:00:00
*Speaker 1:* Okay ! Et quand vous faites vos- vos courses comme ça [euh] vous anticipez sur vos recettes puisqu'on a beaucoup parlé des fruits. Alors c'est vrai que les fruits, bon, à part [euh] justement les pommes où on peut, il y a pas mal de recettes ou les poivres mais [euh] [euh] pour les légumes par exemple. Est-ce que vous arrivez au marché, au supermarché en disant je veux cuisiner ça ?

00:00:00
*Speaker 2:* Oui, c'est ça.

00:00:00
*Speaker 1:* C’est ça. C'est votre organisation.

00:00:00
*Speaker 2:* Je sais que je vais faire ça comme [euh] comme plat, donc il me faut [euh] des carottes, des oignons, enfin et je fais- je fais mes achats en fonction des- des menus que- que j'ai en tête.

00:00:00
*Speaker 1:* D'accord. Et donc plutôt la veille pour le lendemain ou deux trois jours d'avance ? Enfin, c'est [euh] ?

00:00:00
*Speaker 2:* Ça dépend pour [euh] un jour [euh]. Ça m'arrive même de faire pour [euh] pour le même- le surlendemain hein. Quand c'est des- quand c'est des plats en sauce qui- qui sont meilleurs finalement quand ils sont un peu réchauffés. Sinon je reste sur un- un jour. Un jour deux jours à l’avance.

00:00:00
*M. [Prénom du conjoint]:* Avec deux, c'est pas- c'est pas facile non plus de faire des trucs [euh] ou alors vous en mangez toute la semaine quoi. Ah là, ça me dérange pas de toute la semaine.

00:00:00
*Speaker 2:* Le- le fiston quand il fait tard.

00:00:00
*Speaker 1:* Et alors vous êtes plutôt [euh] [euh], vous avez des stratégies de sur les quantités, vous connaissez d'avance [euh] vos quantités convenables ?

00:00:00
*Speaker 2:* Bon, en fait, j'ai un peu de mal avec ça parce que je suis issue d'une famille nombreuse. On était cinq enfants. Mettez huit à la maison puisqu'il y avait aussi le grand-père. Et c'est vrai que quand j'étais jeune je faisais beaucoup de la cuisine avec ma mère. Et j'ai toujours eu l'habitude de cuisiner pour [euh] un nombre important de personnes. Et après on a eu quatre enfants. Donc on était six. J'ai toujours un petit peu de mal à faire que pour deux. J'ai toujours eu la part du *pauvre*.

00:00:00
*M. [Prénom du conjoint]:* Tout le temps, tout le temps.

00:00:00
*Speaker 2:* C'est- c'est vrai, c'est vrai. Mais bon, c'est pas gaspiller pour autant hein si ce qui n'a pas été mangé à midi [euh] sera remangé le soir ou le lendemain midi. Mais j'ai un peu de mal, j'ai un peu de mal à respecter les toasts.

00:00:00
*Speaker 1:* D'accord. Je- je vois un regard complice et attendri.

00:00:00
*M. [Prénom du conjoint]:* Bon des fois ça- ça fait des choses, ça me- ça me dérange pas parce que- ça me dérange pas d'en remanger. Mais des fois, je me retrouve avec un tout petit reste là. Vous me direz on aurait pu le finir tout de suite. Mais bon quand vous avez déjà la- l’assiette qui est pleine…

00:00:00
*Speaker 2:* Faut pas- faut pas se forcer non plus. Manger pour manger quoi. Ça n'a pas de sens.

00:00:00
*M. [Prénom du conjoint]:* Il ne faut pas non plus des quantités faramineuses hein.

00:00:00
*Speaker 2:* Gaspiller pour autant.

00:00:00
*Speaker 1:* Et alors est-ce que vous pourriez maintenant me faire une liste ? Alors vous pouvez la faire à deux hein, des [euh]- des fruits et des légumes que vous avez l'habitude de vous procurer, voilà. Commencez par les légumes par exemple.

00:00:00
*Speaker 2:* Des carottes souvent, des tomates très souvent aussi. Surtout là maintenant qu'elles sont quand même bonnes. Des oignons, de l'ail, en hiver ce sera des poireaux. Qu'est-ce que j'utilise [euh] pané aussi pané, du céleri, des pommes de terre. J'en oublie hein sûrement hein [euh]. Des courgettes aussi, poivron aussi, beaucoup. C'est plutôt, c'est un peu ça qui me viennent à l’esprit.

00:00:00
*M. [Prénom du conjoint]:* Betteraves rouges [euh].

00:00:00
*Speaker 2:* Betteraves rouges dans une moindre mesure parce que toi, t’aime pas trop donc. Qu’est-ce que j'oublie autrement ? Ça dépend hein, si c'est la saison, si c'est l'hiver, ce sera plutôt des- des légumes pour faire de la soupe.

00:00:00
*Speaker 1:* Hum hum.

00:00:00
*Speaker 2:* Donc c'est assez- assez varié, hein. Et puis autrement, ben ça sera plutôt, là maintenant, du melon, des tomates, concombres, [euh] des radis [euh] des- les- les légumes de- de saison, les fruits de saison, en fait, quoi.

00:00:00
*Speaker 1:* Si vous deviez [euh] [euh] chacun me donner [euh] pour chaque saison, votre légume préférés, ça serait quoi ?

00:00:00
*Speaker 2:* Pour chaque saison ?

00:00:00
*Speaker 1:* Au printemps.

00:00:00
*M. [Prénom du conjoint]:* Ben voilà, moi, déjà la- la tomate, je l'aime bien toutes les saisons, même si- même si, c'est vrai que…

00:00:00
*Speaker 2:* Non, l’hiver, c'est pas que des tomates.

00:00:00
*M. [Prénom du conjoint]:* C'est vrai il y a des saisons en hiver, elles sont malades, je trouve que la tomate ça passe partout. Après effectivement ça doit être fonction de…

00:00:00
*Speaker 2:* Des envies ?

00:00:00
*M. [Prénom du conjoint]:* Pas forcément pas, oui, des accompagnements de plats aussi quoi parce que et puis non j'ai pas moi- moi- je parle pas en tout cas de légumes [euh] de légumes [euh].

00:00:00
*Speaker 1:* Non moi non plus.

00:00:00
*M. [Prénom du conjoint]:* Très attachés.

00:00:00
*Speaker 2:* Moi j'aime bien tous les légumes. Tous les fruits. Il y a pas beaucoup de légumes ou de fruits que je n'aime pas. J'aime beaucoup la courgette par exemple. Et puis [euh] en été [euh] j'aime bien les carottes aussi, les céleris, le céleri aussi.

00:00:00
*M. [Prénom du conjoint]:* En fait, c'est des *igloo* en été des *igloo* qu'on peut manger cru quoi.

00:00:00
*Speaker 1:* Oui.

00:00:00
*Speaker 2:* C'est différent, la texture n'est pas la même [euh], puis, on recherche pas forcément les mêmes produits, en fonction des températures, de la température. On a moins envie maintenant de manger du potage, c'est clair.

00:00:00
*Speaker 1:* C'est clair.

00:00:00
*Speaker 2:* Des potages. On n'est pas attiré alors qu'en hiver [euh], bah, on fait bien un peu du potage pendant, cette année, on a dû faire de septembre jusqu'en avril hein, pratiquement un an. Donc [euh], vous voyez [euh].

00:00:00
*Speaker 1:* Du coup vous variez [euh], je vais prendre un exemple, la carotte. La carotte, c'est vrai qu'on en a- on en a quasiment toute l'année, on en a en été [euh]. Est-ce que vous diriez que vous l'avez préparé de manière différente entre l'hiver et l'été ?

00:00:00
*Speaker 2:* Ah oui, quand même la carotte, c'est davantage en hiver, automne, hiver sera plutôt en potage et puis la carotte après, ce sera plutôt [euh], râpé [euh].

00:00:00
*Speaker 1:* D'accord.

00:00:00
*Speaker 2:* Avec du citron et puis [euh], de l'huile d'olive quoi.

00:00:00
*Speaker 1:* D'accord. Oui donc il y a vraiment différence.

00:00:00
*Speaker 2:* Ah oui, oui, oui !

00:00:00
*Speaker 1:* Qui est liée finalement à la saison.

00:00:00
*Speaker 2:* Voilà !

00:00:00
*Speaker 1:* Au besoin de finalement d'avoir quelque chose de chaud l'hiver.

00:00:00
*Speaker 2:* Tout à fait. Ouais, c'est ça.

00:00:00
*Speaker 1:* C'est super intéressant. Et du coup [euh] toutes ces- ces [euh] quand- quand [euh] vous revenez des courses admettons hein, vous avez été au marché.

00:00:00
*Speaker 2:* Voilà, oui.

00:00:00
*Speaker 1:* Voilà. [Euh] qu'est-ce que vous faites ? [euh] [euh] Quels sont vos gestes en fait, de rangement ? Que ça soit pour les légumes ou pour les fruits. Voilà. Comment vous voulez ranger ? Est-ce qu'il y a des- des stratégies particulières ?

00:00:00
*Speaker 2:* Alors, je ferai- je ferai abstraction de la pandémie hein parce que pendant la pandémie, c'était- c'était un peu compliqué par rapport aux légumes et fruits qu’on laissait toujours [euh] dans le garage pendant, les laver après mais bon, on va faire abstraction de ça.

00:00:00
*M. [Prénom du conjoint]:* Même les laver, les rincer avant de les ranger, bon.

00:00:00
*Speaker 1:* D'accord.

00:00:00
*Speaker 2:* Personnellement on le fait plus. Parce que ça fait un an, on l'a fait, on en a un petit peu marre. Ben en- en définitive quand on arrive la première chose qu'on fait, c'est ranger les- les fruits, les légumes, ben les- les fruits comme ça je les mets à la corbeille. Mais si par exemple j'en ai acheté beaucoup beaucoup [euh] les pommes je vais pas les mettre toutes dans la corbeille en même temps. Je vais mettre [euh] peut-être [euh] un tiers ou la consommation pour un ou deux jours.

00:00:00
*Speaker 1:* Hum hum.

00:00:00
*Speaker 2:* Et le- les autres ils vont, je vais les stocker dans le- le frigidaire.

00:00:00
*Speaker 1:* D'accord.

00:00:00
*Speaker 2:* Et les légumes [euh] par contre [euh] ce qui est carottes [euh] carottes, oignon, tout ça, c'est mis dans la, une petite réserve dans le garage quoi.

00:00:00
*Speaker 1:* D'accord. D'accord.

00:00:00
*Speaker 2:* Les pommes de terre aussi.

00:00:00
*Speaker 1:* Stocker à température tempérée en fait.

00:00:00
*Speaker 2:* Oui, voilà !

00:00:00
*Speaker 1:* Et vous avez [euh] est-ce qu'il y a certains légumes par exemple, que vous stockez comme ça à température tempérée et d'autres où vous dites ah ben non ceux-là il faut pas. Est-ce que vous faites une différence en fait [euh] ?

00:00:00
*Speaker 2:* Oui, oui ! Il y a certains quand même que- que je préfère mettre dans le frigidaire comme [euh] les concombres ou en fait tout ce qui peut se gâter assez rapidement quoi.

00:00:00
*Speaker 1:* D'accord.

00:00:00
*Speaker 2:* Mais, c'est pas forcément bien, je ne sais pas.

00:00:00
*Speaker 1:* Non, non. Mais moi ce qui m'intéresse, c'est en fait, comment vous en fait, vous estimez [euh] puisque il y a- il y a plein de- de choses qui sont bâties sur [euh] sur [euh] l'expérience finalement [euh] des années de- de voilà de- de conservation et de confrontation avec la réalité.

00:00:00
*Speaker 2:* Oui, c’est un peu ça. C'est presque du bon sens après.

00:00:00
*Speaker 1:* Voilà. Et mais [euh] et- et du coup [euh] oui ça enfin ça nous intéresse de savoir par exemple si [euh] [euh] là vous avez parlé des concombres, je sais pas est-ce que les courgettes [euh] vous les mettez systématiquement frais ou … ?

00:00:00
*Speaker 2:* Les courgettes, je les mets plutôt dans le frigidaire aussi.

00:00:00
*Speaker 1:* D'accord. Okay ! Donc, finalement ce que vous mettez plutôt en arrière [euh] ça va être ce que vous avez dit, carottes, oignons, des choses peut-être.

00:00:00
*M. [Prénom du conjoint]:* Ouais, des pommes de terre.

00:00:00
*Speaker 1:* Pommes de terre.

00:00:00
*Speaker 2:* Des pommes de terre parce que autrement [euh] les poireaux, un exemple, mais bon en sachant pertinemment je veux pas mettre deux trois jours avant de les- de les utiliser donc.

00:00:00
*Speaker 1:* Ouais. Oui, c'est ça. Gardez en tête que il y a un temps d'utilisation.

00:00:00
*Speaker 2:* Tout à fait.

00:00:00
*Speaker 1:* Voilà. D'accord. Et qui [euh] qui s'explique aussi enfin avec le rythme de vos courses où vous avez un rythme de course qui est assez intense.

00:00:00
*Speaker 2:* On y va souvent parce que de toute façon on va acheter le pain tout jour donc [euh], et bon, comme on est à la retraite, on a le temps quoi.

00:00:00
*M. [Prénom du conjoint]:* Je trouve que ça serait un peu mal venu de- de faire un stock de fruits et légumes pour une semaine. De s'apercevoir qu'au bout de la semaine il y en a une partie qui n'est- qui n'est plus consommable parce que ben parce que c'est comme ça, alors qu'on peut aller effectivement acheter [euh], vous me direz, c'est peut-être pas très- c'est- c'est pas très écologique parce qu'on utilise sa voiture. De toute façon, évidemment on y va quand c’est bon.

00:00:00
*Speaker 2:* Autrement il faut acheter son pain pour la semaine et le congeler. C’est franchement pas terrible les pains congelés non plus quoi, il faut dire ce qui est.

00:00:00
*Speaker 1:* Oui et puis c'est lié à votre situation géographique aussi.

00:00:00
*Speaker 2:* Ben voilà, on est pas tout près- on est pas tout là. On est quand même à presque trois kilomètres hein du- du bout.

00:00:00
*M. [Prénom du conjoint]:* On est pas très loin non plus. Pour d’autres, c’est pire.

00:00:00
*Speaker 2:* Oui oui, c'est clair, mais bon, [euh] on a pas la possibilité de se dire ben on va aller à pied faire les courses [euh] tous les jours. Ça, c'est pas possible.

00:00:00
*Speaker 1:* Mais c'est- c'est assez classique dans nos- dans nos coins.

00:00:00
*Speaker 2:* Ben voilà hein. Quelque soit je pense le coin où on habite.

00:00:00
*M. [Prénom du conjoint]:* Des fois ici, il pleut hein. [Rire]

00:00:00
*Speaker 1:* Parfois ? Non, non, non. Mais non, vous êtes, très rarement.

00:00:00
*Speaker 2:* Des fois, il fait chaud- des fois, il fait chaud ici. [Rire]

00:00:00
*Speaker 1:* D'accord. Et globalement [euh] vous diriez que finalement vous avez une organisation qui vous satisfait dans le sens où est-ce que [euh] ben voilà le fait d'avoir [euh] un espace de stockage comme ça tempéré [euh] des réfrigérateurs [euh] voilà ou- ou est-ce que il y a des moments où vous dites, ah finalement peut-être que je pourrais m'organiser différemment, peut-être que ça serait mieux différemment ou est-ce que vous… ?

00:00:00
*Speaker 2:* Ça s'est fait comme ça petit à petit parce que c'est vrai que je me suis rendue compte que parfois en stockant à un endroit, c'était pas- c'était pas l'endroit approprié et que le- le- les légumes se gâtaient. Au bout de deux quatre jours et puis [euh] ben.

00:00:00
*M. [Prénom du conjoint]:* Ça, c’est arrivé il y a deux ans je crois, on a vu ça, il y avait des choux, il y a un monsieur qui- qui vend- qui composait des pommes de terre. Ah c'était très rare. En plus c'est lui qui les avait cultivés.

00:00:00
*Speaker 2:* Ah, c’était pas une bonne idée hein.

00:00:00
*M. [Prénom du conjoint]:* Donc [euh] c'est vrai que c'était, ça valait le coup, c'était très cher. Racheter deux sacs de 25 kilos je crois. Ben il y a quasiment un sac qui est parti à la poubelle.

00:00:00
*Speaker 2:* Elles ont toutes gelées en même temps.

00:00:00
*M. [Prénom du conjoint]:* Parce qu’en fait, on en mange pas tant que ça.

00:00:00
*Speaker 2:* Non nous, on est pas trop pomme de terre.

00:00:00
*M. [Prénom du conjoint]:* On en mange pas tant que ça. Non je t'ai dit bon 50 kilos quoi. Si tu vois, tu veux être encore de temps en temps.

00:00:00
*Speaker 2:* Oui oui.

00:00:00
*M. [Prénom du conjoint]:* J'ai dit bon à trois peut-être que ça va le faire quoi. Mais bon, finalement non, ça n’a pas fait quoi. Donc, finalement, donc il y a pratiquement tout un sac qui est parti [euh], qui était pas enfin que j’ai retrouvé pas consommable quoi.

00:00:00
*Speaker 1:* Oui.

00:00:00
*M. [Prénom du conjoint]:* Donc, en voulant entre guillemets en voulant faire plaisir au monsieur qui avait cultivé ses pommes de terre, en lui achetant des pommes de terre pas trop chères, là finalement on n'a rien gagné du tout.

00:00:00
*Speaker 2:* Et aussi, il faut avoir des- des conditions de conservation idéales qu'on pourra acheter quand on achète des- des produits en- en grande quantité.

00:00:00
*M. [Prénom du conjoint]:* Et puis il faut les consommer quoi, il faut les consommer.

00:00:00
*Speaker 2:* Il faut en manger tous les jours.

00:00:00
*Speaker 1:* Oui, c’est ça.

00:00:00
*Speaker 2:* Voilà.

00:00:00
*M. [Prénom du conjoint]:* C'est pour ça qu'on va un peu faire nos courses tous les jours, à la limite, on préfère acheter trois pommes et retourner en acheter trois, deux jours après, vu que acheter six, bon quoique six elles vont tenir le coup, mais bon là, j'exagère un peu quoi.

00:00:00
*Speaker 1:* Je vais passer maintenant à tout ce qui est un petit peu [euh] ben préparation parce que là on a enfin, voilà, on a balayé en fait, vos pratiques sur [euh] les achats ou est-ce que vous achetez, à quel rythme, comment vous conservez finalement hein, comment vous stockez, comment vous êtes organisés. [Euh] et alors maintenant ben on va passer à tout ce qui est préparation. On va dire ça comme ça. [Euh] je sais pas, on va prendre un- un peut-être un, on a parlé des- des pommes hein. Voilà, au tout début, donc a priori, c'est un- c'est un fruit que [euh] en tout cas [euh] vous madame consommez.

00:00:00
*Speaker 2:* Oh [Prénom du conjoint] aussi.

00:00:00
*Speaker 1:* Votre mari aussi, oui. D'ailleurs je me souviens quand ça a été épluché, coupé en petits morceaux. Alors justement, [Rire].

00:00:00
*Speaker 2:* Ah, vous voyez un peu hein.

00:00:00
*M. [Prénom du conjoint]:* Non, c'est vrai que j'aime pas éplucher les pommes ou peut-être des compotes.

00:00:00
*Speaker 1:* Eh ben alors justement, c'était la question que j'allais vous poser, est-ce que vous pouvez me donner toutes les formes sous laquelle vous consommez des pommes ? Toutes les recettes.

00:00:00
*Speaker 2:* Cru, cru, [euh] en compote.

00:00:00
*Speaker 1:* Hum hum.

00:00:00
*Speaker 2:* [Euh] faire des crumbles, des tartes aux pommes. [Euh] Qu’est-ce que je peux faire autrement ?

00:00:00
*M. [Prénom du conjoint]:* En salade de fruit.

00:00:00
*Speaker 2:* Ah salade de fruit. Si, je fais souvent des salades de fruits en été.

00:00:00
*Speaker 1:* D'accord.

00:00:00
*Speaker 2:* J'aime bien- j'aime bien jouer avec des couleurs [euh] une pomme [euh] avec de la peau verte [euh] rouge, mettre de la couleur dans la salade de fruits.

00:00:00
*Speaker 1:* D'accord. Et donc du coup, ça veut dire que [euh] les pommes ça, vous les consommés parfois avec la peau ?

00:00:00
*Speaker 2:* Oui.

00:00:00
*Speaker 1:* Est-ce qu’il vous arrive de les consommer sans ? Enfin, voilà.

00:00:00
*Speaker 2:* [Euh] quand je les mange comme ça, je les- je les épluche. Mais si par exemple je fais une salade de fruits pour que, il y ait de la couleur, je- je lave la pomme et je vais, je garde la- la peau.

00:00:00
*Speaker 1:* Et vous garder la peau.

00:00:00
*Speaker 2:* Oui.

00:00:00
*Speaker 1:* D'accord. Okay. Et par contre quand vous la manger [euh] en désert comme ça, vous l’éplucher.

00:00:00
*Speaker 2:* Oui.

00:00:00
*Speaker 1:* D'accord.

00:00:00
*Speaker 2:* C’est bizarre en fait.

00:00:00
*Speaker 1:* Vous enlevez aussi la partie centrale ?

00:00:00
*Speaker 2:* Oui.

00:00:00
*Speaker 1:* Oui ?

00:00:00
*Speaker 2:* Oui.

00:00:00
*Speaker 1:* D’accord. Donc en fait voilà, c'est les quartiers épluchés quand consomme comme ça et puis [euh] il y a- il y a des petits jeux avec la couleur.

00:00:00
*Speaker 2:* Oui.

00:00:00
*Speaker 1:* D'accord, super. [Euh] est-ce que vous avez en tête un- un légume que vous consommez très régulièrement et [euh] sur lequel il y aurait aussi cette diversité, enfin ces- ces aspects de préparation ? Quand on a parlé de la tomate tout à l'heure par exemple ?

00:00:00
*Speaker 2:* Oui, ben oui les tomates par exemple, oui.

00:00:00
*M. [Prénom du conjoint]:* Oui la tomate, c'est quand même, oui [euh].

00:00:00
*Speaker 2:* On fait- on fait pas mal de tomates, je fais souvent des tomates farcies par exemple.

00:00:00
*Speaker 1:* D'accord.

00:00:00
*Speaker 2:* [Euh] je fais aussi pas mal de préparation avec des courgettes, des poivrons, des tomates, des oignons, de l'ail. La- la comment ? La ratatouille par exemple.

00:00:00
*Speaker 1:* Oui.

00:00:00
*Speaker 2:* [Euh] qu'est-ce que je peux faire autrement [euh] ? J'utilise pas mal aux tomates hein. Quand on fait du- du poisson aussi [euh].

00:00:00
*Speaker 1:* Hum hum.

00:00:00
*Speaker 2:* J'aime bien- J'aime bien faire une [euh]- une petite préparation avec des oignons, de l'ail, des tomates. Parce que parfois le poisson, c'est sec autrement.

00:00:00
*Speaker 1:* Hum hum.

00:00:00
*Speaker 2:* Voilà. Essentiellement ou alors ben des- des blanquettes, des- du curry, les choses comme ça, donc [euh], il y a toujours aussi des, il y a toujours des carottes hein [euh.]

00:00:00
*M. [Prénom du conjoint]:* Non pas des carottes.

00:00:00
*Speaker 2:* Pas des carottes, des- des tomates. Des tomates quoi. Ouais. Ouais.

00:00:00
*Speaker 1:* D’accord. Et ça vous arrive de les manger [euh] cru aussi ?

00:00:00
*M. [Prénom du conjoint]:* Ah oui, oui.

00:00:00
*Speaker 2:* Oui. Oui.

00:00:00
*Speaker 1:* Et alors là, c'est, est-ce que, qu'est-ce qu'il y a comme type de préparation ? Est-ce que…

00:00:00
*Speaker 2:* Oh, c'est râpé en général.

00:00:00
*Speaker 1:* Ça, c'est pour les carottes j'imagine ?

00:00:00
*Speaker 2:* Non, oui pardon, les tomates.

00:00:00
*Speaker 1:* Et pour les tomates ?

00:00:00
*Speaker 2:* Les tomates. Comme ça.

00:00:00
*M. [Prénom du conjoint]:* Oui, comme ça, il y a même pas couper.

00:00:00
*Speaker 1:* Couper en fait.

00:00:00
*Speaker 2:* À la demi-crête éventuellement.

00:00:00
*Speaker 1:* D’accord.

00:00:00
*Speaker 2:* Quand elles sont bonnes, il y a pas besoin d'ajouter quoi que ce soit.

00:00:00
*Speaker 1:* D'accord. Oui donc, quasiment nature quoi.

00:00:00
*Speaker 2:* Ouais.

00:00:00
*Speaker 1:* Et est-ce que [euh] vous avez un légume [euh] ou un fruit [euh], que vous consommez sans rien faire en fait ? C'est-à-dire sans enlever un petit morceau, sans éplucher, sans qu'il y ait rien qui, qu'est-ce qui vous viendrait éventuellement à l'esprit ?

00:00:00
*Speaker 2:* Le seul…

00:00:00
*M. [Prénom du conjoint]:* Il y en a beaucoup.

00:00:00
*Speaker 2:* Oui, il suffit de le rincer [euh], comme fruit comme légumes peut-être le radis juste pour l'avoir lavé.

00:00:00
*Speaker 1:* Oui.

00:00:00
*M. [Prénom du conjoint]:* On était obligé de les rincer quoi.

00:00:00
*Speaker 2:* Oui, le rincer.

00:00:00
*M. [Prénom du conjoint]:* On est obligé d’enlever la racine.

00:00:00
*Speaker 2:* L’abricot, les- les cerises, les fraises. Qu’est-ce que je fais autrement ? Ah oui, le reste [euh].

00:00:00
*Speaker 1:* Et donc la plupart, il y a …

00:00:00
*Speaker 2:* Oui, il faut quand même pour la plupart, il faut enlever la peau.

00:00:00
*Speaker 1:* Hum hum.

00:00:00
*Speaker 2:* Les pépins pour [euh].

00:00:00
*Speaker 1:* Il y a une préparation.

00:00:00
*Speaker 2:* Un petit peu quoi. Un petit peu, voilà.

00:00:00
*Speaker 1:* Un petit peu. [Euh] alors là, je- je- j'arrive au- au enfin on en a- on a un petit peu abordé hein [euh] déjà précédemment, mais puisque vous m'avez expliqué pour les- les [euh] les confitures. Mais à la partie [euh] [euh] sur- sur un- un fruit ou un légume qui hum- qui est un petit peu abîmé.

00:00:00
*Speaker 2:* : Hein !

00:00:00
*Speaker 1:* Voilà en fait, ce que ce- que j'aimerais [euh] creuser avec vous c'est finalement [euh] [euh] comment et quand est-ce et sur quels critères en fait vous vous basez pour [euh] décider que vous jetez plutôt que de consommer. Quand [euh] les rares faux ça vous arrive.

00:00:00
*Speaker 2:* Ça n'arrive pas souvent par exemple pour les- les bananes vous voyez il y en a deux là qui sont quand même bien mûres.

00:00:00
*Speaker 1:* Oui.

00:00:00
*Speaker 2:* Mais comme mon mari aime bien les bananes au four. Je les utilise je les épluche je les coupe en deux. J'enlève les parties gâtées hein. Je mets du citron. Citron, un tout petit peu de beurre, un tout petit peu de sucre et je fais gratiner au four.

00:00:00
*Speaker 1:* D’accord.

00:00:00
*Speaker 2:* Pour les pommes, c'est pareil, quand elles sont un peu [euh] tâchées, je les coupe et je fais de la compote.

00:00:00
*M. [Prénom du conjoint]:* À l’époque elle estimait- estimait si on la garde, qu'on la garde pas, je pense que ça, si je reviens sur la banane, encore que comme ça, moi, ça me dérange pas trop de manger une banane comme ça.

00:00:00
*Speaker 1:* Hum.

00:00:00
*Speaker 2:* Ben là, il est- il est temps de les manger.

00:00:00
*M. [Prénom du conjoint]:* Même si elle est un peu [euh] je préfère, enfin, manger une banane comme ça que l'autre à côté, qui est toute jaune au clair.

00:00:00
*Speaker 1:* D’accord.

00:00:00
*M. [Prénom du conjoint]:* Bon ben alors, si il y a la moitié, si effectivement il y a la moitié de la banane qui est- est noire là, bon peut-être que je vais quand même couper [euh] les *coussins* de notre monde [euh] à l'époque les pommes pour moi, ben, c'est pareil hein. Je ne me vois pas non plus l'acheter, il faudrait qu'il y ait quasiment [euh] les trois quarts de la pomme qui soient esquintées quoi.

00:00:00
*Speaker 2:* Moi je- je jette rarement aussi. Même les- les abricots par exemple ou des fraises, on a un petit peu dans le jardin. Ben les abricots, si c'est un peu touché, je- je vais récupérer le reste et puis, je vais mettre avec un, je vais le manger avec un fromage blanc ou un yaourt.

00:00:00
*Speaker 1:* Quand vous [euh] vous enlevez les parties qui sont abîmées vous avez tendance à aller un peu large ou est-ce que finalement vous avez tendance à aller un peu près ?

00:00:00
*Speaker 2:* Non je vais large.

00:00:00
*Speaker 1:* Vous allez large.

00:00:00
*Speaker 2:* Je vais l'arche.

00:00:00
*Speaker 1:* D'accord.

00:00:00
*Speaker 2:* Parce que j'ai toujours peur que c'est le goût du- du produit, en fait.

00:00:00
*M. [Prénom du conjoint]:* En fait, on sait pas [euh], finalement, on sait pas pourquoi- on sait pas pourquoi le fruit est comme ça et c'est pas forcément [euh]- c'est pas forcément contraire à la santé.

00:00:00
*Speaker 1:* Non ?

00:00:00
*M. [Prénom du conjoint]:* À la limite, on pourrait le manger comme ça sans être malade quoi, c'est, oui, c'est presque un peu une [euh]…

00:00:00
*Speaker 2:* Je trouve qu’il y a une petite différence, je trouve hein.

00:00:00
*M. [Prénom du conjoint]:* Quand je parle des abricots pourris, c'est vrai que dans la confiture malheureusement je pense que quand on achète un pot de confiture…

00:00:00
*Speaker 2:* On sait pas hein.

00:00:00
*M. [Prénom du conjoint]:* Il y a dû avoir des fruits pourris dedans c'est certain quoi donc on ne sait pas pour autant s’ils sont…

00:00:00
*Speaker 2:* Ils ne savent pas les trillés comme on le fait, non, je pense à coter ils font- ils font des confitures.

00:00:00
*M. [Prénom du conjoint]:* Sinon, c'est pas, c'est moins appétissant quoi.

00:00:00
*Speaker 1:* Ouais.

00:00:00
*M. [Prénom du conjoint]:* C’est ça.

00:00:00
*Speaker 2:* Ouais, mais près, [eux] ils mettent des conservateurs aussi quand il faut les, quand ils font les confitures, il y a des conservateurs sur ce que nous on fait pas pourtant.

00:00:00
*M. [Prénom du conjoint]:* Pour un fruit que tu achètes qui va être un tout petit peu, un petit peu esquinté, à la limite on pourrait je pense que c'est plus une question de, oui, c'est ça quoi, c'est pas très appétissant, ça donne pas trop envie de le manger quoi.

00:00:00
*Speaker 1:* Et du coup vous avez des parce que là on a beaucoup parlé de- du visuel en fait, de- de- du fait de voir mais [euh] est-ce que vous utilisez d'autres sens, enfin [euh] est-ce que par exemple.

00:00:00
*Speaker 2:* Odeur aussi.

00:00:00
*Speaker 1:* Oui voilà, enfin tous ces éléments-là ça intervient dans votre jugement, ça intervient comment ?

00:00:00
*Speaker 2:* Oui quand même. Par exemple si vous voyez des petites moisissures sur un fruit [euh] il va passer à la poubelle tout de suite.

00:00:00
*M. [Prénom du conjoint]:* Ah oui pareil, pour revenir- pour venir aux étales des supermarchés comme je disais tout à l'heure. La plupart des pommes elles sont toutes rouges comme vous regardez dessus et puis, en général elles ont une belle forme, elles sont toutes- elles sont toutes rondes et on voit…

00:00:00
*Speaker 2:* Ils sont toutes calibrées quoi, en fait.

00:00:00
*M. [Prénom du conjoint]:* Ça nous arrive on voit des, quand il y a des ramasses, enfin on voit des trucs à la télé, ils éliminent- ils éliminent les- les fruits qui ont été qui n'ont pas une forme [euh].

00:00:00
*Speaker 2:* C’est un peu idiot.

00:00:00
*M. [Prénom du conjoint]:* Alors qu'il n’est pas forcément [euh].

00:00:00
*Speaker 2:* C'est complètement idiot.

00:00:00
*M. [Prénom du conjoint]:* Moins bon pour autant. Donc, c'est vrai qu'on est un petit peu aussi [euh]- on est un petit peu guidés par tout ça et c'est pas- c'est très bien quoi.

00:00:00
*Speaker 1:* Est-ce que ce que vous voulez dire c'est qu'il y a encore un peu l'idée [euh] par cette habitude, une sorte de perfection.

00:00:00
*Speaker 2:* Oui, c’est ça. C’est ça.

00:00:00
*Speaker 1:* Que du coup peut-être des choses qui sont finalement anodines [euh] on les écarte.

00:00:00
*Speaker 2:* Oui.

00:00:00
*Speaker 1:* C'est- c'est- c'est ça que vous voulez dire.

00:00:00
*Speaker 2:* Oui.

00:00:00
*M. [Prénom du conjoint]:* Oui.

00:00:00
*M. [Prénom du conjoint]:* Vous voulez des carottes, par exemple, les carottes elles sont toutes [euh], elles ont toutes la même [euh], ah, après si des fois vous cultivez des carottes dans votre jardin, vous allez retrouver des fruits qui vont avoir deux- deux tiges.

00:00:00
*Speaker 2:* Qui ont des drôles de forme hein.

00:00:00
*M. [Prénom du conjoint]:* Non, c'est moins facile à toucher c'est sûr.

00:00:00
*Speaker 2:* Qui sont originales hein.

00:00:00
*M. [Prénom du conjoint]:* C'est pas pour autant qu'elle est- qu'elle va pas être bonne quoi. Donc c'est- c'est là-dessus que…

00:00:00
*Speaker 2:* Même les tomates hein, bien souvent quand elles ont une drôle de forme, elles sont- elles sont bonnes, elles sont super bonnes.

00:00:00
*Speaker 1:* Ouais. Ouais. Parce que là finalement, il y a- il y a- il y a deux choses [euh] qui sont pas forcément exactement les mêmes. Il y a cet aspect de forme, cet aspect visuel enfin de ce qu'on voit. Et puis, après, il y a- il y a ce que vous disiez.

00:00:00
*Speaker 2:* Le goût.

00:00:00
*Speaker 1:* Enfin le, il y a le goût [euh].

00:00:00
*Speaker 2:* Oui le goût.

00:00:00
*Speaker 1:* Il y a quelque chose qui est vraiment [euh]- qui est vraiment altéré. Il peut y avoir d'autres [euh] ça peut se sentir.

00:00:00
*Speaker 2:* Voilà.

00:00:00
*Speaker 1:* [Euh] enfin et sentir et avoir du coup, un goût [euh] voilà. Donc c'est- c'est un petit peu- un petit peu différent. Ça vous est déjà arrivé d'avoir ce, de croquer dans un fruit comme ça, puis, de vous apercevoir après que- que il était [euh] altéré enfin [euh]…

00:00:00
*Speaker 2:* Ouais quand même.

00:00:00
*Speaker 1:* Des souvenirs comme ça.

00:00:00
*Speaker 2:* Oui parfois. Une pomme essentiellement, c'est tout mou quoi.

00:00:00
*Speaker 1:* Ah ouais ?

00:00:00
*Speaker 2:* C'est- c'est un drôle de goût.

00:00:00
*Speaker 1:* Voilà, c'est ça.

00:00:00
*Speaker 2:* C'est pas bon.

00:00:00
*Speaker 1:* C'est pas bon. [Rire]. Clairement. [Euh] C'étaient des questions que, et [euh] est-ce que [euh] alors deux questions en fait, [euh] est-ce que je- je vous écoutais parler là, de la banane donc, [euh] et- et cette différence finalement de- de l'appétence visiblement pour des bananes qui vont être [euh] peu mûres, et de celles qui vont être mûres. Il y a- il y a des différences comme ça [euh] [euh] au sein de votre [euh] de- de- de votre foyer, vous avez chacun des goûts différents ?

00:00:00
*M. [Prénom du conjoint]:* Moi je n'aime pas les bananes de, je préfère manger une banane comme ça- comme ça quoi, encore que cela soit à peu près, pour moi, les bananes mûres, c'est quasiment ça quoi.

00:00:00
*Speaker 1:* Ouais. C’est ça.

00:00:00
*M. [Prénom du conjoint]:* Et c’est bien possible parce que je vous dis j'en ai vu partir moi, de- de Martinique. [Euh] c'étaient des- des régimes qui étaient verts.

00:00:00
*Speaker 2:* Bâh oui, c’est comme sa mûrie après, dans les bateaux hein.

00:00:00
*M. [Prénom du conjoint]:* Et on les pique même pour que pendant la- à l'époque les trajets de bateaux mettait une dizaine de jours à faire le, à traverser. Et pendant les dix jours, les bananes murissaient quoi.

00:00:00
*Speaker 1:* Ouais.

00:00:00
*M. [Prénom du conjoint]:* C'est vrai que ça n'a aucun- cela, ça n’a rien à voir avec une banane que vous cueillez mûre et que vous mangez.

00:00:00
*Speaker 2:* Le- le- le problème, je trouve quand elles sont [euh] tachetées comme ça, c'est que, elles sont vraiment sucrées- sucrées.

00:00:00
*Speaker 1:* Hum !

00:00:00
*Speaker 2:* Moi je préfère quand elle est sont moins mûres.

00:00:00
*M. [Prénom du conjoint]:* J’aime bien quand elles sont sucrées hein.

00:00:00
*Speaker 2:* Oui, toi, t'aimes bien le sucre.

00:00:00
*Speaker 1:* Ah oui ! Oui oui, donc il y a- il y a une, et [euh] est-ce que [euh] alors pour aller un peu plus loin [euh] vous avez le sentiment que vous- vous avez des- des [euh] choses au sein de- de- de- de votre foyer, vous avez des- des réactions différentes. Est-ce que par exemple, il arriverait un lundi, Ah non mais attends ça, c'est, on va le jeter enfin, c'est plus bon et que l'autre [euh] [euh] dirait, non non mais attends, c'est récupérable là.

00:00:00
*M. [Prénom du conjoint]:* Oh, ça nous arrive, ça nous a déjà arrivé de dire, oui le truc n’est pas trop bon.

00:00:00
*Speaker 2:* Pas trop souvent. Non, on n’a pas trop de différence sens.

00:00:00
*M. [Prénom du conjoint]:* En général, oui en général quand on dit qu’on ne mange pas, c’est que nous, on a vraiment pas envie de- on n’en a vraiment pas envie de manger.

00:00:00
*Speaker 2:* J’avoue que je n’en demande pas, juste que le- le fruit ou le légume est franchement trop gâté pour être [euh] utilisé [euh] moi, je le mets dans la poubelle hein.

00:00:00
*Speaker 1:* Hum hum !

00:00:00
*Speaker 2:* J'ai pas trop l'état d'âme.

00:00:00
*Speaker 1:* Ouais. Pas d’état d’âme, oui.

00:00:00
*Speaker 2:* Non.

00:00:00
*M. [Prénom du conjoint]:* Ah oui, je pense que c'est, il y a au-delà de- au-delà de ne pas gaspiller.

00:00:00
*Speaker 2:* On ne va pas se rendre malade non plus à se forcer à manger des trucs.

00:00:00
*M. [Prénom du conjoint]:* Non mais t'es pas, c’est pas forcément être malades, ça, c'est pas nocif, mais c'est vrai que il y a plus non plus le plaisir de si on est un tout petit peu dégoûté, il y a plus non plus, même si le reste du fruit est bon ou de la- il y a plus le plaisir de le manger et voilà.

00:00:00
*Speaker 2:* Au pire, à consommer quelque chose qui sera pas bon, ça va peut-être aussi [euh] nous toucher l'idée que ben le fruit finalement il est- il est pas bon.

00:00:00
*Speaker 1:* Ah oui !

00:00:00
*Speaker 2:* Ça m’arrive de me dégoûtée complètement d'un fruit ou d'un produit tout court.

00:00:00
*M. [Prénom du conjoint]:* Même si on jetait un kilo toutes les semaines moi, je dis pas. Non. Non, c'est pas le cas, donc, c'est pas non plus le- en général on fait quand même- on fait attention à ça.

00:00:00
*Speaker 1:* Hum. C'est très intéressant ce que vous venez de dire.

00:00:00
*M. [Prénom du conjoint]:* Mais peut-être que quand- peut-être que quand on était plus jeune, c'était pas tout à fait comme ça, c'est toujours si je pense que, il y a une façon d'évoluer en mentalité.

00:00:00
*Speaker 2:* Ouais, puis, c'est différent. Quand on- est quand on a une famille [euh] c'est plus difficile de prévoir parfois les quantités pour une famille nombreuse que pour [euh] deux. [Euh] on sait à peu près ce qu'on mange, les quantités qu'on mange [euh], il faut faire les – il faut faire les pleins en fonction de ça.

00:00:00
*M. [Prénom du conjoint]:* Voilà, on en avait pas que ça, *vachement* paire à propos des pommes, on a un garçon, ben lui il prend des, il mangeait exclusivement que ça, c'étaient des pommes granny.

00:00:00
*Speaker 2:* Que ça ?

00:00:00
*Speaker 1:* D’accord.

00:00:00
*M. [Prénom du conjoint]:* Il y a pas une autre pomme.

00:00:00
*Speaker 2:* Ah non, les pommes granny avec des petits pois blancs en plus. Je sais pas pourquoi.

00:00:00
*M. [Prénom du conjoint]:* Déjà s'il y avait pas de petits pois blancs, c’est déjà- c’est déjà bien.

00:00:00
*Speaker 2:* Non, c’était pas bonne. Mais il mange que ça.

00:00:00
*M. [Prénom du conjoint]:* Il mange que des pommes granny. Alors, pourquoi ? Je ne sais pas.

00:00:00
*Speaker 2:* Mais bon.

00:00:00
*M. [Prénom du conjoint]:* Les autres sont bonnes aussi, la vedette, les golden et tout.

00:00:00
*Speaker 2:* Ben oui, ben oui. Quand même hein. Chacun a ses goûts hein.

00:00:00
*Speaker 1:* Oui oui.

00:00:00
*Speaker 2:* Chacun a ses goûts.

00:00:00
*Speaker 1:* C'est vrai que c'est- c'est peut-être plus facile d'être [euh], et du coup, on gaspille moins quand on est dans quelque chose de- de finalement plus simple en terme d'organisation.

00:00:00
*Speaker 2:* Ah je pense hein parce que c'est vrai que quand vous avez [euh] des enfants à la maison ils n'ont pas forcément les mêmes goûts.

00:00:00
*Speaker 1:* Hum hum.

00:00:00
*Speaker 2:* Et certains- certains il faut les forcer à- à manger à vouloir, à goûter certains plats. Je pense qu'on a pas la même approche du tout. Du tout du tout. On essaye de faire plaisir quand même à ses enfants là, parfois on fait des plats pour leur faire plaisir, mais que, on n'aurait pas fait si on n’était que tous les deux.

00:00:00
*Speaker 1:* C'est ça. Eh bien, c'est une parfaite transition sur [euh] sur [euh] la- la- la- on arrive presque à la fin de- de notre entretien. [Euh] sur le gaspillage alimentaire [euh] en fait, j'aimerais alors séparément l'un puis l'autre [euh] que [euh] vous me disiez quels sont les mots ou les expressions qui vous viennent spontanément à l'esprit quand je vous dis gaspillage alimentaire.

00:00:00
*Speaker 2:* Un gâchis, surtout quand on sait qu'il y a plein de gens qui- qui ne mangent pas leur faim sur cette terre. Et puis [euh] une irresponsabilité aussi.

00:00:00
*M. [Prénom du conjoint]:* Peut-être pas irresponsabilité mais…

00:00:00
*Speaker 2:* On devrait- on devrait tous faire des efforts pour que ça ne se produise pas.

00:00:00
*M. [Prénom du conjoint]:* Méconnaissance quoi. Les gens pensent que c'est…

00:00:00
*Speaker 2:* Enfin, le zéro gâchis ça ne va pas, ça, je ne pense pas que ça puisse exister.

00:00:00
*Speaker 1:* Hum hum.

00:00:00
*Speaker 2:* Mais il faudrait tendre [euh].

00:00:00
*M. [Prénom du conjoint]:* Mais je pense que c'est aussi un peu une question de, pas forcément que les questions, mais de maturité. Il y a des choses que- il y a des choses qu'on aimait pas ou qu'on aimait bien quand on était plus jeunes et que, on aime plus, enfin on aime moins maintenant ou qu'on aime- ou qu’on aime moins maintenant alors que, on ne bougeait pas quand on était plus petits quoi. Je sais pas il y a, après il faut se dire aussi que, oui quand on voit des gens qui triment pour [euh] pour dire ceci, pour dire cela ou…

00:00:00
*Speaker 2:* Il faut respecter je pense, le travail.

00:00:00
*M. [Prénom du conjoint]:* Peut-être- peut-être qu'il faut faire attention quand même quoi que tout le monde n'a pas forcément [euh]. Ah nous en plus, on a été avec autre association on a été au Burkina Faso ben je peux vous dire que quand vous avez- quand vous avez passé quelques jours au Burkina Faso vous avez vu une petite partie de la population.

00:00:00
*Speaker 1:* Ouais.

00:00:00
*M. [Prénom du conjoint]:* Ben quand vous revenez à la maison vous vous dites que vous gaspillez pas, enfin les- les premiers jours qui arrivent là même les enfants ils gaspillent *plus* beaucoup hein.

00:00:00
*Speaker 2:* Non.

00:00:00
*Speaker 1:* Non.

00:00:00
*M. [Prénom du conjoint]:* Après, évidemment [euh], ça revient, mais bon [euh], c'est vrai qu'on est, on se dit effectivement qu'on a quand même [euh]…

00:00:00
*Speaker 2:* Que comme vous avez mangé du riz tous les jours pendant trois semaines quand on reste [euh] c’est vraiment pas varié quoi comme nourriture.

00:00:00
*Speaker 1:* Ouais, comme- comme nourriture. Du coup sur la base de tout ce que vous venez de vous dire finalement si vous deviez [euh] [euh] choisir le- le mot ou l'expression la plus importante [euh] pour vous [euh] [euh] associé au gaspillage alimentaire qui est que vous m'avez exprimé, ça serait lequel finalement ?

00:00:00
*Speaker 2:* Moi, ce serait un gâchis.

00:00:00
*M. [Prénom du conjoint]:* Moi, je dirai que c’est un gâchis.

00:00:00
*Speaker 2:* Un gâchis. Un gâchis.

00:00:00
*Speaker 1:* Tous les sens du terme, c'est-à-dire que, effectivement on gâche quelque chose qui pourrait- qui pourrait servir. On gâche aussi une partie de- du travail que quelqu'un a fait ou que plusieurs personnes ont fait pour arriver à ça.

00:00:00
*Speaker 2:* Oui, c’est surtout ça, oui.

00:00:00
*M. [Prénom du conjoint]:* Et on gâche aussi je pense pour soi-même, même si on le sait pas, parce que finalement, c'est peut-être que, c’est une méconnaissance.

00:00:00
*Speaker 2:* Il faudrait, je pense, responsabiliser les gens et se dire ben que si on a acheté un produit, ben il faut faire en sorte de- de le consommer [euh] et ne pas le laisser [euh] mais bon.

00:00:00
*M. [Prénom du conjoint]:* Nos petits-enfants, voit ça en été. T'aimes bien ça ? Ah non, non, j'aime pas, j'aime pas, j'aime pas. T'en as déjà mangé ? Non.

00:00:00
*Speaker 1:* Hum.

00:00:00
*M. [Prénom du conjoint]:* Comment tu veux déjà savoir que tu m'aimes pas ?

00:00:00
*Speaker 2:* Et puis, finalement quand on leur fait le- le plat…

00:00:00
*M. [Prénom du conjoint]:* Voilà.

00:00:00
*Speaker 2:* Souvent ils me disent grand-mère, c’est super bon ce que tu as fait.

00:00:00
*M. [Prénom du conjoint]:* Comment tu peux savoir que tu n’aimes pas si tu ne l’as jamais mangé ?

00:00:00
*Speaker 2:* Je sais, mais oui, il suffit de juste le goûter. Je sais pas, il y a- il y a parfois aussi quand on est enfant ou quand on est jeune il y a des produits qui ben qui nous découpent, mais bon qui sont pas attirants. Faut dire ce qui est hein [euh] et ça me dit, on voit ça avec les- les- les- la nouvelle génération.

00:00:00
*M. [Prénom du conjoint]:* La nature la- la publicité est tellement axée sur certains produits qu’il y en a d'autres qui sont connus au rebut quoi. Et puis [euh] bon ben ça fait je sais pas moi, ça fait partie de, je sais pas, par exemple, manger de la soupe, ça fait un peu, ça fait ça fait un peu vieillot quoi, des choses comme ça.

00:00:00
*Speaker 2:* Et c'est ce que nous dit [euh], le, un de nos petits-enfants.

00:00:00
*Speaker 1:* Ouais.

00:00:00
*Speaker 2:* Qui a 13 ans. Ah, il dit j'adore aller chez vous alors, grand-mère avec ta soupe. [Rire]

00:00:00
*M. [Prénom du conjoint]:* Pourtant, il en mange hein.

00:00:00
*Speaker 2:* Pourtant, il la prend il me dit, mais bon la soupe que les *soirs* en hiver, oh non-pitié. Eh ben je dis ben tant pis, tu- tu bougeras ou autre chose après la soupe si tu veux, mais tu mangeras ta soupe.

00:00:00
*Speaker 1:* Oui oui, un petit peu de soupe quoi. [Euh] donc du coup, on a- on a ressorti le mot gâchis.

00:00:00
*Speaker 2:* Oui, pour moi, c’est ça.

00:00:00
*Speaker 1:* C'est vraiment intéressant la- la- la manière dont vous l'avez [euh] défini enfin voilà les et du coup maintenant si je vous demande de me faire [euh] une définition parce que là on a un mot en fait, alors une définition, une phrase chacun de votre façon de définir le gaspillage alimentaire.

00:00:00
*Speaker 2:* Qu’est-ce qu’on pourrait dire ? C'est en fait peut-être ne- acheter peut-être plus que ce qu’il l'aurait fallu qu'on achète pour notre propre consommation. Et sans peut-être [euh] anticiper les repas, ne pas avoir réfléchi suffisamment en amont avant les achats.

00:00:00
*Speaker 1:* D’accord.

00:00:00
*M. [Prénom du conjoint]:* Moi, c’est peut-être preuve- c'est peut-être faire preuve d'égoïsme parce que quand on dit, je vais acheter, je vais acheter, je vais acheter pour pas, guillemets, pour pas manquer. Sans s'en rendre compte que d'abord ces choses-là, ça a été faites par des gens qui ben qui- qui ont travaillé pour et puis, qu'il y en a d'autres qui les auront pas quoi. Je pense qu'il y a ouais, c'est- c'est pas très facile à donner une définition [euh].

00:00:00
*Speaker 1:* Non non, je sais. [Rire]. Je vous pose une question difficile. Je suis désolée. Mais vous deux, vous deux, vos phrases sont intéressantes.

00:00:00
*M. [Prénom du conjoint]:* Mais je pense que vous n'avez pas, je pense pas que si vous avez des- si vous avez différent candidat parmi vos- vos corbeilles, vous ne pouvez pas avoir les mêmes définitions selon les…

00:00:00
*Speaker 1:* C'est parce que j’en recherche.

00:00:00
*Speaker 2:* Je pense que, il y a 20 ou 30 ans, on n'aurait pas forcément porté ces réponses-là.

00:00:00
*Speaker 1:* C’est comme ça.

00:00:00
*Speaker 2:* Sinon, on évolue.

00:00:00
*Speaker 1:* C’est clair.

00:00:00
*Speaker 2:* Ouais, toute cette expérience aussi.

00:00:00
*Speaker 1:* Non mais jamais, je pense qu'on l'a jamais trop trop parce que déjà [euh] que déjà les acheter donc, on a jamais non plus trop trop gaspillé hein quoi quand- quand bien même, qu'on avait quatre enfants bon, fallait quand même bien acheter les choses, mais, oui je pense quand même que plus on vit enfin à mon avis plus on vieillit…

00:00:00
*Speaker 2:* On prend conscience de certaines choses.

00:00:00
*M. [Prénom du conjoint]:* On se rend compte de certaines choses que plus on voit bien aussi des gens [euh] bon ben oui on s'aperçoit que, il y a des incohérences quoi, des incohérences quoi.

00:00:00
*Speaker 2:* Et puis quand on œuvre dans le domaine social, on se rend compte qu'il y a des gens bah qui n'ont pas la chance de manger. Manger leur faim, on se dit, c'est- c'est pas possible de voir le gâchis partout.

00:00:00
*Speaker 1:* Et [euh] du coup quand [euh] les rares fois où ça vous arrive, vous jetez quelque chose, il y a un sentiment particulier ?

00:00:00
*Speaker 2:* Non moi, je m'en veux.

00:00:00
*M. [Prénom du conjoint]:* Oui oui.

00:00:00
*Speaker 2:* Je m'en veux parce que je me dis ben j'aurais dû voir.

00:00:00
*M. [Prénom du conjoint]:* Moi, j'ai mal au cœur hein.

00:00:00
*Speaker 2:* Oui. Je m'en veux moi. Je me dis [euh] c'est- c'est hommage quoi de ne pas avoir vu que ben il fallait le consommer peut-être hier ou avant-hier.

00:00:00
*Speaker 1:* C'est- c'est- c'est ce qu'on me dit [euh]

00:00:00
*Speaker 2:* C'est vrai qu'on sent veut. On s'en veut, mais bon [euh] parfois on peut pas faire autrement.

00:00:00
*Speaker 1:* Oui, voilà.

00:00:00
*Speaker 2:* Voilà, il faut bien se rendre à l'évidence.

00:00:00
*M. [Prénom du conjoint]:* On peut pas- on peut pas arriver au parfait hein, c'est pas malheureusement, c'est pas possible hein.

00:00:00
*Speaker 1:* C'est clair.

00:00:00
*M. [Prénom du conjoint]:* Il y a toujours des imperfections hein.

00:00:00
*Speaker 2:* Mais bon.

00:00:00
*Speaker 1:* [Euh] est-ce que vous avez sur cette partie-là, sur ces aspects-là, est-ce qu'il y a d'autres choses qui vous viennent à l'esprit ou … ?

00:00:00
*Speaker 2:* Non, je pense pas, non.

00:00:00
*Speaker 1:* Sur la partie gaspillage vraiment [euh] [euh] parce que après je voulais vous poser quelques petites questions sur l'utilisation de la poubelle connectée, mais finalement, c'est [euh] c'est en lien hein.

00:00:00
*Speaker 2:* Hum hum.

00:00:00
*Speaker 1:* Vous m'avez dit il me semble que vous aviez pas eu le sentiment de l'utiliser très fréquemment…

00:00:00
*M. [Prénom du conjoint]:* Ben non, vous avez pas eu le- vous n'avez pas le poids de…

00:00:00
*Speaker 1:* Non, j’ai, alors j'ai- j'ai…

00:00:00
*Speaker 2:* Il y a peut-être une hauteur comme ça.

00:00:00
*Speaker 1:* Ouais, j'ai pas- j'ai pas- j'ai pas regardé pour pas arriver avec des idées préconçues. Voilà.

00:00:00
*Speaker 2:* Moi, je me suis même dit, mais ils m'ont mis rien à poubelle.

00:00:00
*M. [Prénom du conjoint]:* On a utilisé qu'un seul sac hein.

00:00:00
*Speaker 2:* Non mais, une fois j'ai- j'ai mis une pomme gâtée [euh] je me suis dit elle est quand même pas gâtée au point de la mettre [euh] quand même entièrement dans la poubelle alors je l'ai coupée en quatre morceaux et je l’ai donné aux oiseaux. Moi, mon mari m'a dit qu’ils ne vont pas aimer ça les oiseaux, que, ils en ont mangé hein, vite fait.

00:00:00
*Speaker 1:* D'accord. Donc, du coup, vous l'avez pas du tout mis dans la- la poubelle [euh] connectée ? Elle est partie aux oiseaux.

00:00:00
*Speaker 2:* Non, non, non. Je l'ai mise [euh] aux oiseaux.

00:00:00
*Speaker 1:* D'accord. D'accord. D'accord.

00:00:00
*Speaker 2:* C’est la seule entorse.

00:00:00
*Speaker 1:* Et globalement vous allez [euh] alors [euh] là, c'est sur la période de la poubelle connectée, mais plus en général, vous avez quand même l'impression qu'il y a des fruits ou des légumes que vous jetez vous, plus particulièrement, plus fréquemment.

00:00:00
*Speaker 2:* Moi, je dis, je dirais comme je vous avais dit la dernière fois, bien souvent ce sont les fruits de saison. Speakeur 1 : Ouais. Speakeur 2 : [Euh] les pêches [euh] les pêches, les brugnons, même les fraises hein celles-ci on les consomme pas rapidement, ça se garde très très vite hein. Les abricots [euh] vous voyez, c’est difficile aussi de trouver des abricots qui soient consommables le jour J, le jour- le jour de l'achat, alors on les laisse et puis, finalement ils nourrissent tous en même temps et puis il faut manger des abricots très rapidement quoi, par exemple. C'est tous les fruits de saison et moi je pense que ça vient aussi en grande partie du fait que, ils ont déjà [euh] subi un voyage [euh] un peu plus l'Espagne ou…

00:00:00
*Speaker 1:* Ouais ; c’est ça.

00:00:00
*Speaker 2:* Et je pense que ça, ça n'arrange pas hein.

00:00:00
*M. [Prénom du conjoint]:* Ah oui enfin ça, t'as des fruits qui vont un peu ici hein, de toute façon.

00:00:00
*Speaker 2:* On se rend bien compte là mais, je disais à un de mes enfants, notre aîné est venu, m’a amené un bouquet de fleur pour la fête des mères. Ah, elle était super, mon mari, moi ça me fait mal au cœur au bout de, une semaine, il était à mettre à la poubelle. Il dit, je coupe des fleurs de mon jardin, mais elle dure beaucoup plus longtemps que les fleurs qu'on achète et qui coûte quand même.

00:00:00
*M. [Prénom du conjoint]:* Encore qu'on ait vu il y a pas longtemps là, il y a un producteur de bananes alors, je sais pas si c'est dans Finistère ou dans la, en Bretagne.

00:00:00
*Speaker 2:* Ah oui, on a vu un reportage.

00:00:00
*M. [Prénom du conjoint]:* Le service est installé, il a l’air de se tailler des sièges là, et il va produire ses…

00:00:00
*Speaker 2:* Ses bananes.

00:00:00
*M. [Prénom du conjoint]:* Il va recruter ses premières bananes.

00:00:00
*Speaker 2:* Ouais, c’est une, sûr, je le sais hein.

00:00:00
*M. [Prénom du conjoint]:* Oui, ah oui oui, c’est sûr hein. Oui, ça doit être dans le coin, pas très très loin d’ici.

00:00:00
*Speaker 1:* Ah d’accord, c’est- c’est [euh]- c’est très intéressant.

00:00:00
*M. [Prénom du conjoint]:* C’est en Bretagne hein. C'est pas…

00:00:00
*Speaker 1:* J'avais entendu parler d'un projet comme ça dans le Morbihan mais [euh].

00:00:00
*M. [Prénom du conjoint]:* Bah, c'est peut-être ça hein.

00:00:00
*Speaker 1:* C'est peut-être Morbihan, mais il faudrait que je me renseigne.

00:00:00
*Speaker 2:* C’est peut-être pas *Morbihan*, mais, je le connaissais pas de nom.

00:00:00
*M. [Prénom du conjoint]:* C'est peut-être ça, non non, moi, j'ai jamais été surpris parce que c’est quand même des bananes [euh] c’est quand-même, j'ai dit, c'est pas très [euh], même si on voit beaucoup de bananiers dans les jardins.

00:00:00
*Speaker 1:* Ouais.

00:00:00
*M. [Prénom du conjoint]:* Ils fournissent pas beaucoup de bonheur.

00:00:00
*Speaker 2:* C'est purement exotique là.

00:00:00
*Speaker 1:* Oui oui. Ah, il y a- des, je, il y a des régimes. Après je pense que c'est des problèmes pour arriver à maturité en fait.

00:00:00
*Speaker 2:* Ouais.

00:00:00
*Speaker 1:* Mais [euh].

00:00:00
*M. [Prénom du conjoint]:* Ah oui là le monsieur disait que ça faisait presque trois ans, trois ans qu'il tâtonnait, qu'il faisait des essais sur les fruits.

00:00:00
*Speaker 2:* Ça marchait pas.

00:00:00
*M. [Prénom du conjoint]:* Que certains, ça n'avaient pas- ça n'avaient pas marché et que là maintenant, effectivement ils, ça- ça avait l'air, il avait l'air d'avoir eu des fruits et donc il avait récolté ses premiers.

00:00:00
*Speaker 1:* D'accord.

00:00:00
*M. [Prénom du conjoint]:* Oui.

00:00:00
*Speaker 1:* De- de trouver la, l’espèce, le bon [euh].

00:00:00
*Speaker 2:* Oh, c'est pas évident hein non plus.

00:00:00
*Speaker 1:* Non mais c'était intéressant. [Euh] vous avez jamais hésité pour la poubelle connectée à vous dire [euh] je dois mettre dedans ou je dois pas mettre dedans, c'était clair ?

00:00:00
*Speaker 2:* Jamais.

00:00:00
*Speaker 1:* D'accord. Ok, très bien.

00:00:00
*Speaker 2:* Même si c'était [euh] ça je faisais… [Rire]

00:00:00
*Speaker 1:* Vous êtes parfait.

00:00:00
*Speaker 2:* Parfois, c'était ça hein.

00:00:00
*Speaker 1:* C'est très bien. Voilà, j'ai fini en fait de vous poser l'ensemble des questions que j’avais à vous poser. Alors [euh] ben merci déjà beaucoup d'y avoir répondu. Et puis [euh] je sais pas est-ce que vous avez des choses [euh] que vous souhaitez ajouter sur [euh] sur [euh] ce sujet ou est-ce que vous avez l'impression des- des points que, auxquels vous connaissez ?

00:00:00
*M. [Prénom du conjoint]:* Est-ce qu'on aura un retour de tout ça ?

00:00:00
*Speaker 1:* Alors.

00:00:00
*M. [Prénom du conjoint]:* Pas forcément de- de la préparation globale, de la préparation globale.

00:00:00
*Speaker 1:* Ouais.

00:00:00
*Speaker 2:* De ce qu'ils ressortent de l'ensemble des…

00:00:00
*Speaker 1:* Oui oui, oui oui. Alors, il y aura un retour, on est en train de réfléchir à la manière dont on va organiser ce retour [euh], en fait, on- on souhaiterait globalement [euh], organiser un petit événement [euh], autour de ça avec [euh], enfin les foyers qui ont participé, voilà. [Euh], ça sera probablement [euh], je dirais sur le premier semestre 2022 parce que là il faut enfin on va revenir vous voir à cet automne, après il faut le temps de traiter les informations et cetera [euh]. Mais l'idée, c'est effectivement non seulement de bah, de- de vous faire un retour sur les résultats de l'étude, mais aussi d'essayer de faire enfin voilà, de- de- de donner un petit peu de- de- de poids à cet événement parce qu'on est [euh], extrêmement [euh], [euh], contents et reconnaissant d'avoir [euh], [euh], autant de foyers qui participent et puis qui participent avec [euh], de l'entre. Voilà.

00:00:00
*Speaker 2:* Bon je pense que dès l'instant où ce porte volontaire c'est que on est- on est sensible à ça.

00:00:00
*M. [Prénom du conjoint]:* Et c’est pas, je me rappelle plus, c'est pas dans le cadre de la société IP ça.

00:00:00
*Speaker 2:* Non non non.

00:00:00
*Speaker 1:* *Non.

00:00:00
*M. [Prénom du conjoint]:* n'a rien à voir.

00:00:00
*Speaker 2:* C’est apparu, l'article est apparu dans la revue de la société.

00:00:00
*Speaker 1:* Oui. Mais c'est, ce n'est pas- ce n'est pas.

00:00:00
*Speaker 2:* C'est eux qui ont relayé l'information.

00:00:00
*Speaker 1:* Je vais du coup [euh] interrompre. Oui du coup maintenant le- le l'enregistrement puisque voilà.

**Household F45, Interview 2**

*Mme. 1:* Tac. [Alors], un petit matériel. [Toux] tac [silence 00:00:09-00:00:15] Alors, non. Je vais vous demander de prendre une de ces [euh] images, peu importe laquelle de la retourner, et de me dire spontanément quels sont les [euh] les premiers mots qui vous viennent [euh] à l'esprit.

*Mme. 2:* Carré potager. [silence 00:00:34-00:00:38] Vous me demandez ce que c'est, je pense.

*Mme. 1:* [rire]

*M. 1:* Je réponds aussi ?

*Mme. 1:* Enfin, une photo par photo plutôt. Les deux, vous pouvez répondre d'abord sur celui-là, et vous ferez celui-là après.

*Mme. 2:* Il faut donner le nom des légumes ?

*Mme. 1:* [rire] C'est ce qui vous vient [euh] par la tête.

*Mme. 2:* [Alors] [euh], je dirais qu'il y a des poireaux. Je crois que c'est des blettes. [silence 00:00:57-00:01:01] Il peut y avoir des radis aussi.

*Mme. 1:* Qu'est-ce que ça vous évoque le potager ? Pour vous ?

*Mme. 2:* [euh]

*M. 1:* Les beaux jours.

*Mme. 1:* [rire]

*Mme. 2:* Ouais, ouais.

*M. 1:* Et si.

*Mme. 2:* Et, les moments agréables ou si [euh] --

*M. 1:* Les moments de détente.

*Mme. 2:* Ouais. Et puis (euh) la satisfaction de récupérer aussi [euh] quelques- quelques légumes qu'on cuisinera ultérieurement.

*Mme. 1:* Ouais.

*Mme. 2:* Donc, je sais pas ce que c'est ça.

*Mme. 1:* Donc, vous avez un- un potager ici vous ou par contre--?

*Mme. 2:* Oui, oui, oui. On a un potager là, puis on a quelques carrés aussi sur le côté. Enfin, c'est pas très, très grand quand-même [hein]. Mais bon, on essaie quand-même de mettre [euh] une variété [euh]-- ouais. C'est plus marqué donc.

*M. 1:* Je bêche et puis [euh] c'est toi qui plantes.

*Mme. 2:* On n'a plus besoin de bêcher, maintenant [hein]. Depuis qu'on- qu'on met le carton et tout ça, il n'y a plus besoin de bécher [hein].

*Mme. 1:* Et [euh] qu'est-ce que vous pensez des produits que vous consommez et qui sont issus de- de votre potager ?

*Mme. 2:* [Ben], moi je les trouve meilleurs. Mais [bon] [euh], est-ce que c'est vraiment objectif- objectif ? Je sais pas. C'est peut-être le fait aussi de les avoir [euh] mis en terre, de regarder la croissance [euh] puisqu'y a- y a un côté un petit peu [euh]-- une relation un peu particulière quoi.

*M. 1:* A priori, ils ne sont pas-- à priori ils sont ça.

*Mme. 2:* Moi, je veux zéro [euh]-- je veux pas d'engrais [ah].

*M. 1:* Sauf que maintenant, notre terrain a été-- était polluant, mais à priori non.

*Mme. 2:* Si ça poussait bien, si ça pousse pas [bah] tant pis [rire]. Je mets pas de-- je mets pas d'engrais du tout.

*Mme. 1:* Ouais.

*Mme. 2:* Après. Parfois, le rendement n'est pas non plus extraordinaire. Mais [bon], on a quand même la satisfaction d'avoir [euh]-- d'avoir fait pousser quelque chose.

*Mme. 1:* [silence 00:03:00-00:03:04] Et du coup, est-ce que [euh] quand vous êtes dans le potager, vous, c'est un moment [euh] c'est, est-ce que c'est un endroit dans lequel vous vous sentez bien ? Ou-- [ehm] ?

*Mme. 2:* [Ah] oui, [ah] moi, j'aime bien, oui.

*Mme. 1:* Oui.

*Mme. 2:* Ça me détend.

*Mme. 1:* [Silence 00:03:16-00:03:22] Et est-ce que ça vous arrive de devoir jeter les fruits et légumes que vous aviez vous même récoltés ? Non.

*Mme. 2:* Non, je- je les donne autrement [euh] [toux] le dernier de nos enfants qui est pas très loin d'en profiter un petit peu aussi.

*M. 1:* Et il n'y en pas beaucoup, beaucoup non plus.

*Mme. 2:* Non, non, mais les betteraves rouges quand même on en a eu beaucoup. Et puis [bon] les betteraves rouges elles arrivent toutes [euh] toutes en même temps. Donc je- j'ai souvent [euh] j'en prends beauc- je- je récolte beaucoup, je les c-- je les cuis et puis [euh] je- je donne [euh] je donne --

*Mme. 1:* D'accord, vous n'avez pas le sentiment d'avoir [euh] des fois jeté des produits que vous aviez ?

*Mme. 2:* Oh non non, oh non non.

*Mme. 1:* [silence 00:04:00-00:04:04] Ok, [ben] si on fait une autre image maintenant.

*Mme. 2:* [bruit] [Alors] [bruit]

*M. 1:* Comme ça c'est rayon- rayon fruits et légumes d'un --

*Mme. 2:* D'un supermarché ou --

*M. 1:* D'un supermarché.

*Mme. 2:* Le temps est très... c'est difficile de reconnaître les légumes là [hein].

*Mme. 1:* Mm-hmm

*Mme. 2:* [silence 00:04:22-00:04:27] Tirer des mirabelles. Peut-être là.

*Mme. 1:* Il est plus gros et je ne sais même pas si c'est des mirabelles ?

*Mme. 2:* Hmm?

*M. 1:* C'est pas des mirabelles.

*Mme. 2:* Je ne sais pas. C'est est-ce que c'est des fruits et légumes ou, ou est-ce que c'est que des légumes ou que des fruits ? Parce que en général [euh], il y a- y a un rayon bien distinct pour les- les fruits et légumes. Moi, je pense que c'est plu- plutôt raisonner en termes-- ou que des légumes, ou que des fruits.

*Mme. 1:* Ça vous évoque quoi [euh] ce type de rayon ?

*Mme. 2:* [euh] [ben] je trouve que les- les couleurs sont variées dans tous les cas donc. Ce qui laisse peut-être supposer.

*M. 1:* Il a l'air bien achalandé, mais [bon] je sais pas, parce qu'il y a plusieurs fois les mêmes [euh]

*Mme. 2:* Je pense que c'est plutôt en- en sai- saison été je dirais moi.

*Mme. 1:* D'accord.

*Mme. 2:* Mais [bon], après, c'est vrai que ça peut être des fruits rouges comme ça peut être des pommes de terre [euh] rouge là aussi avec la peau. Disons que c'est pas-- c'est pas très parlant [hein].

*M. 1:* [Ben] et il faut reconnaître que les --

*Mme. 2:* On voit les couleurs, mais [euh] sans vraiment pouvoir [euh] dire à quoi correspondent les- les images.

*Mme. 1:* Mm-hmm. Oui, on est sur une la photo et prise de loin et de haut.

*Mme. 2:* Oui, oui.

*Mme. 1:* Est-ce que le supermarché c'est un lieu dans lequel vous vous rendez [euh] régulièrement ou pas ?

*M. 1:* Mm-hmm

*Mme. 2:* Si.

*Mme. 1:* Ouais.

*Mme. 2:* Ici on n'a pas-- on n'a pas franchement [euh] l'embarras du choix non plus.

*Mme. 1:* Ouais.

*Mme. 2:* Si autrement le marché [euh] le marché qui a lieu le vendredi quoi.

*M. 1:* Et celui là, c'est peut-être un ma-- un-- c'est peut-être un magasin bio.

*Mme. 2:* Ouais, mais ils sont pas très grand hein parce que je trouve que y a beaucoup d'espace partout.

*M. 1:* C'est pas qu'ils sont grands, c'est que il y a pas beaucoup de [euh]...

*Mme. 2:* De quoi?

*M. 1:* De client.

*Mme. 2:* Y a pas beaucoup de clients ?

*M. 1:* Ouais je trouve pas non [hein]

*Mme. 2:* Tout dépend de leur --

*M. 1:* Un, deux, trois, quatre, cinq, six.

*Mme. 2:* Tout dépend de l'heure aussi dans la journée hein. C'est pas-- c'est pas très révélateur non plus [hein], l'heure.

*Mme. 1:* Et vous pensez quoi des fruits et légumes que vous trouvez dans ce type de magasin ?

*M. 1:* Dans ce, là là ?

*Mme. 1:* Ouais.

*M. 1:* Ce qu'on voit sur la photo ?

*Mme. 1:* Ouais.

*Mme. 2:* Moi je pense que [euh] il peut satisfaire les- les demandes des clients, parce qu'il a l'air d'avoir quand-même une assez grande variété [hein].

*Mme. 1:* Vous, ça vous arrive d'en consommer ?

*M. 1:* Les fruits et légumes ?

*Mme. 1:* De qui sont issus de supermarchés ?

*Mme. 2:* Ah oui.

*M. 1:* Oui.

*Mme. 1:* [Ah] ouais ? Et vous en-pensez quoi ? Vous êtes vous êtes satisfaits aussi de ce que vous trouvez ? Ou [euh] ou pas ?

*Mme. 2:* [Ben] en sommes oui, parce que y a [enfin] nous, on va essentiellement au Leclerc, ici hein Leclerc de Ploudalmézeau. Et on peut quand même noter qu'il le travaille beaucoup avec des producteurs locaux [hein] pour les [euh]

*Mme. 1:* Donc là.

*Mme. 2:* Pour les courges et tout ça, ça vient de Milizac [hein]. Après, c'est pas du bio [hein].

*M. 1:* C'est l'exception [hein].

*Mme. 2:* [Ah] non. Les salades aussi. Les salades aussi, c'est [euh]

*M. 1:* [diaphonie 00:07:25-00:07:27]

*Mme. 2:* Mais [bon] [euh]

*M. 1:* ça vient même pas de France, c'est d'Espagne ...

*Mme. 2:* [Ah] voilà. De toute façon [euh]

*M. 1:* [silence 00:07:32-00:07:36] Mais nous on y va quand-même régulièrement, donc, on paie pas non plus de--

*Mme. 2:* Non.

*M. 1:* C'est vrai que ça à tendance à- à se gâter.

*Mme. 1:* Ouais. Il s'abîme assez vite [euh]

*M. 1:* Même des fois dans le rayon ils ne sont pas terribles- terribles [hein]

*Mme. 1:* Ouais.

*Mme. 2:* [Ben], parfois oui, il faut [inaudible 00:07:52-00:07:54] C'est pas facile parce que--

*Mme. 1:* Ouais. [silence 00:07:58-00:08:02] Vous gérez toutes les commandes parfois, vous savez. C'est régulier ou c'est--

*Mme. 2:* [diaphonie 00:08:05-00:08:07] on vient pratiquement tous les jours.

*M. 1:* Oui oui.

*Mme. 1:* [Ah] ouais ? Ouais, du coup [euh] effectivement, ça reste pas stocké [euh]

*Mme. 2:* [Ah] non, non, non puis en général, quand on achète des- des fruits ou des légumes on- on sait ce qu'on va faire comme- comme plat ou comme recette. Donc [euh] c'est pas quelque chose qui est dans l'attente d'être cuisiné non plus pour nos ...

*M. 1:* Ou alors [euh] si on a si on est en promo [euh], on peut les garder plus longtemps.

*Mme. 2:* Puis ça, puis ça dépend des légumes aussi, par exemple les courges et tout ça, ça peut, ça supporte de rester [euh] d'être resté stocké pendant --

*M. 1:* Des potimarons on avait pris plusieurs parce que ce n'était pas cher. Ben, ils étaient en- ils étaient en cours quoi ! Comme on fait de la soupe tout l'hiver, comme elle fait de la soupe tout l'hiver. Que j'ai pris ce petit peu. [Rire] Donc oui, on avait pris quelques-uns quoi.

*Mme. 1:* Ouais.

*Mme. 2:* [Ben] après [euh], comme les pommes aussi si on peut [euh] si on les- si on les met pas au bon endroit, ça peut être conservé quand-même [euh] quelques jours ou même une ou deux semaines. ça peut-- ça supporte quoi mais [bon] c'est vrai que moi je me méfie un petit peu [hein] les surtout en surtout en l'été, y avait les fruits de saison. On sait pertinemment que il ne faut pas en acheter en grande quantité parce que ça se gâte très très vite [hein].

*Mme. 1:* Ouais.

*Mme. 2:* En plus l'été, je trouve qu'il y a le plus de gaspillage.

*Mme. 1:* Vous avez remarqué que ...

*Mme. 2:* Ah oui notamment les-les abricots, les pêches ?

*Mme. 1:* Ouais.

*Mme. 2:* C'est vrai que si on les vends, on les utilise pas assez rapidement [euh]

*Mme. 1:* Ouais.

*Mme. 2:* Bien souvent, ça finit en compote [hein] ou--

*Mme. 1:* Ouais [rire]

*Mme. 2:* [Ben] voilà.

*M. 1:* Mais comme ils ont-- ils ont voyagé obligatoirement c'est un peu [diaphonie 00:09:50-00:09:52]

*Mme. 2:* Sujet des variations de température.

*M. 1:* Même s'il y a des précautions même s'il y des précau-. Or normalement, il y a [euh]

*Mme. 2:* Les transports et tout [hein]. C'est dans les camions réfrigérés seulement [hein] donc [euh]

*M. 1:* Je pense que c'est seulement bien que quand il est cuit sur l'arbre [hein].

*Mme. 2:* [Ah] oui, c'est clair.

*Mme. 1:* Sauf s'il a des gens dans le sud et qui la cueillent [euh]

*Mme. 2:* [Enfin] il n'a pas, on n'a pas non plus [euh] la possibilité d'aller cueillir des- des fruits ou ...

*M. 1:* Qui est seul endroit à Paris [euh], Le verger carambole à Milizac. Il propose deux fois par an ...

*Mme. 2:* Non une fois c'est tout.

*M. 1:* Une fois par an, [euh] un week-end là où on peut aller [euh] dans le verger cueillir ses pommes. Il y a plusieurs variétés de pommes.

*Mme. 2:* Des poires aussi je crois...

*M. 1:* Et vous mettez votre cageot, qu'est-ce que vous voulez [euh] dix pommes d'ici, dix pommes dedans.

*Mme. 2:* C'est ça l'avantage puisque vous pouvez- vous pouvez choisir différentes variétés et c'est ce qu'elle propose sur le marché aussi. [En fait] tous les- toutes les pommes sont au même prix.

*Mme. 1:* Ouias.

*Mme. 2:* Je trouve ça intéressant s'il y a des variétés qu'on ne connaît pas forcément, tiens on va prendre [euh] une ou deux on va voir si elle est bonne.

*Mme. 1:* Et après vous payez le cageot pour récupérer [euh]

*M. 1:* Le problème c'est qu'une fois on a- une fois on les appelle, on a dû les garder trop longtemps. Puis il y en a quand même pas mal qui ont été [euh]

*Mme. 2:* Il faut être sûr qu'il faut pas, il ne faut pas en prendre en trop trop grand nombre non plus parce que il faut avoir un lieu approprié pour les conserver. C'est ça le problème [hein]

*M. 1:* Puisqu'on dit que les pommes, il faut les conserver, on peut si on dit que les pommes se conservent longtemps. La conservation se fera dans un lieu relativement frais. Et que les- les ...

*Mme. 2:* Pas qu'elles se touchent.

*M. 1:* que les pommes ne se touchent pas.

*Mme. 1:* D'accord, et c'est pour cela qu'ils ont des petits [euh]

*Mme. 2:* Oui, voilà. Ouais, ouais.

*M. 1:* Apparemment si elle touche. Parce que s'il y en a une qui est gâtée et qu'elle est-- et qu'elle touche l'autre.

*Mme. 2:* Ouais

*Mme. 1:* Trois ou quatre.

*Mme. 2:* ça contamine tout [hein] ?

*M. 1:* On dirait qu'ils gardent des pommes très très fins. Je me rappelle [euh] un oncle, mais c'était c'était pas pareil. C'était un- c'était un pommier qu'il avait, ses parents avaient dans son jardin, dans leur jardin. Eux n'utilisaient pas tant que ça de pommes.

*Mme. 1:* Ouais.

*M. 1:* Et lui, il habitait à 300 kilomètres de là. Et tous les fins d'été, il venait avec les cageots. I- il vidait les pommiers et un jour je lui demandais mais qu'est-ce que tu fais, qu'est-ce que tu fais de toute ces pommes ? Bah je les mets dans ma cave, sur des cueillettes en bois ?

*Mme. 1:* Ouais.

*Mme. 2:* Mm

*Mme. 1:* Oui, c'est là qu'on m'avait dit. La seule chose laquelle faut faire très attention, c'est qu'elles se touchent pas. Et pourtant, autant jeter un coup d'œil. Il y en a qui disent qu'il y en a une qui est gâtée. Il faut une poubelle. Et il me dit qu'il est gardé [euh]

*Mme. 1:* [diaphonie 00:12:15-00:12:17]

*M. 1:* pres- presque l'hiver presque tout l'hiver.

*Mme. 2:* Parmi nos amis [hein] les-- De du -- ils font ça aussi [hein]

*Mme. 1:* Bon fin ça c'était il y a, c'était y a très longtemps et- et c'était un p- pommier qui n'était pas traité du tout quoi ? Qui poussait comme ça. Donc maintenant, je peux pas vous garantir que les po- les pommes, c'est maintenant [hein]

*Mme. 1:* Ouais. Vous savez que les pommes, ça fait partie des fruits qui sont, par rapport aux fruits d'été [euh]

*Mme. 2:* Ah oui, les fruits d'été je trouve- je trouve que c'est- c'est difficile [hein] de--

*Mme. 1:* De les garder.

*M. 1:* Eh [ben] dans les grandes surfaces hein, depuis qu'il y a la pandémie, il y a des panneaux qui font ici. En tout cas, il y a un panneau qui comme prenez le fruit ou le légume que vous avez touché. Moi, je veux bien. Mais comme à une fois, ça m'est arrivé de le prendre, de le retourner et [euh]

*Mme. 2:* [diaphonie 00:12:56:58]

*M. 1:* Je suis désolé, je ne vais pas là. S'il y avait une poubelle à côté, je le veux bien, je le mettrai à la poubelle.

*Mme. 1:* Ouais, il faudrait bien qu'il y a une composte à côté.

*M. 1:* Mais ce n'était pas le cas [hein]. Donc [euh] à la limite, je le remets du côté pas beau en me disant [bah] il va passer peut-être toucher. Il va pas peut-être passer tout à l'heure. Puisque normalement--

*Mme. 1:* [diaphonie 00:12:13-00:12:14]

*M. 1:* Ils vont passer de temps en temps, quand même c'est son travail, il a pu un petit peu.

*Mme. 1:* Comme mais bon. Oui justement, il faut y aller. C'est normalement quand chaque fois. Après, je ne sais pas s'ils jettent vraiment ce qui est vraiment abîmé ou s'ils cachent tout.

*Mme. 2:* [Bah] je ne sais pas [hein].

*M. 1:* Si! Ce matin, j'en ai vu les gens.

*Mme. 2:* [Bah] il vaut mieux, parce que c'est pas bon calcul de le laisser. Parce que ça [ah]. Un fruit gâté ça- que ça contamine combien d'autre.

*Mme. 1:* Ouais?

*Mme. 2:* Je sais pas. C'est pas bon calcul.

*Mme. 1:* [Hein]. Donc ça vous arrive de-- c'est là d'ailleurs où vous avez acheté vos plupart de fruits et légumes ? Ouais

*Mme. 2:* Et sur le marché aussi. Chez moi, j'achète les pommes, les kiwis. Pomme, kiwi, et même des poires aussi.

*Mme. 1:* Et [euh] donc le, quand vous êtes dans le supermarché, est-ce que c'est un endroit dans lequel vous vous sentez bien ou vous allez que-- uniquement par nécessité ou sûrement, dois-je vous ?

*Mme. 2:* J'aime bien.

*Mme. 1:* Ouais ?

*Mme. 2:* J'aime bien moi. J'aime bien les faire. [Bon] on reste pas non plus une heure dans le magasin tous les ans, à chaque fois qu'on y va ?

*M. 1:* De toute façon, ici, vue où on habite. Ne serait-ce que pour prendre notre pain, il faut qu'on aille jusqu'au bout.

*Mme. 1:* Ouais.

*M. 1:* Une fois qu'on était arrivé là, on n'est plus très loin. Donc j'ai stocké, stocker, stocker pour se dire je n'irai que deux fois par semaine [euh] au Leclerc alors que tous les jours, on va y prendre le pain parce que je prends pas de pain congelé. [fin] qu'on congèle pas notre pain plutôt. Je me dis [bah] l'effort, c'est pas beaucoup pour-

*Mme. 1:* [diaphonie 00:14:42-00:14:44]

*Mme. 2:* Ça permet de voir du monde.

*M. 1:* On travaille plus, donc on n'est pas non plus conditionné par des, par des horaires.

*Mme. 2:* Oui.

*M. 1:* Abracadabra ou quoi

*Mme. 2:* Après, c'est vrai que quand on travaillait, on procédait pas comme ça non plus [hein]

*Mme. 1:* Oui.

*Mme. 2:* Oui les ports mais pas disposés. Pas de 1 h tous les jours pour aller faire les courses non plus [hein].

*Mme. 1:* Oui, il faut même le temps.

*Mme. 2:* [Ah] oui, il faut avoir le temps. Ça- ça, je le conçois parfaitement.

*Mme. 1:* Et [euh] est-ce que ça vous arrive de jeter des fruits et légumes que vous aviez achetés en grande surface ou pas?

*Mme. 2:* [Bah] il faut vraiment que se soit très gâté [hein].

*Mme. 1:* S'ils sont très gâtés.

*Mme. 2:* En général, je regarde les fruits pour tout le pa- pas tous les jours, mais tous les tous les deux jours. Et si je m'aperçois qu'il y a un petit point [euh] [bruit] sur une pomme par e- [silence 00:15:32-00:15:53] moi je les fais au four.

*Mme. 1:* Ok

*M. 1:* [toux]

*Mme. 1:* Et si jamais vous êtes amené à jeter un fruit et légume que vous aviez acheté en- en supermarché. Qu'est-ce que vous ressentez ?

*Mme. 2:* Du gâchis. C'est du gâchis!

*Mme. 1:* Ouais. [silence 00:16:10-00:16:16]

*Mme. 2:* Mais [bon] ! Je peux pas savoir quel- quel circuit a- a effectué t- [ah] a effectué tous les fruits qu'on a dans notre assiette [hein].

*Mme. 1:* Ouais!

*Mme. 2:* Y a pas de très grands producteurs de fruits ici. À part des pommes, y a pas.

*Mme. 1:* Ouais. [inaudible 00:16:33-00:16:35]

*Mme. 2:* Et pas assez chaud ?

*Mme. 1:* Non [rire]

*Mme. 2:* Voilà. Et puis moi, j'ai envie de dire que quand on les-- quand on a une serre comme, moi je trouve que c'est pas très naturel une serre. Mais [bon] après, c'est mon sentiment. Il y en a qui trouvent ça très très bien, mais, moi je trouve que--

*M. 1:* Bien sûr, c'est peut être mieux que d'avoir des produits qui sont qui sont produits en plein air et qui sont traités ou semi traités ou à moitié traité ?

*Mme. 2:* Oui oui, oui d'accord, mais bon [euh]

*M. 1:* A condition, dans la serre on les tripote.

*Mme. 2:* C'est pas très naturel la serre je trouve.

*Mme. 1:* [Ah] [bah].

*Mme. 2:* Qu'on force un petit peu le fruit à mûrir rapidement.

*Mme. 1:* [Ah] [bah] oui oui, on lui crée des essais. C'est ne situation artificielle quoi.

*Mme. 2:* Oui voilà je-

*Mme. 1:* Mais effectivement, après on peut faire une serre et au moins de ne pas les traiter. Mais effectivement [euh]

*Mme. 2:* Oui, oui, oui. Mais [bon] ça, on le sait pas. On ne sait pas toujours, à moins d'aller essentiellement dans des magasins bio quoi.

*Mme. 1:* Ouais, c'est ça.

*Mme. 2:* Oui. [Bon] ici, il y en a, on en a pas. Donc il faut aller jusqu'à ça. Je crois bien que ce double projet, ça rend--

*Mme. 1:* Donc il y a ça aussi la proximité des magasins.

*Mme. 2:* [Ben] oui, oui. [silence 00:17:38-00:18:44] Par contre, je pense que les l'idée qu'un homme fait un peu son chemin, même si je lis dans le bulletin municipal d'aujourd'hui que moi, il y a un groupe qu'on connaît un petit peu, qui propose donc une réunion en vue de l'achat- de l'achat groupé des produits [euh] locaux. [Bon] après, m'interpellent un peu, parce qu'il parle de d'huile, d'huile, du sel.

*M. 1:* De miel.

*Mme. 2:* De miel ouais.

*M. 1:* Miel et du miel encore oui

*Mme. 2:* [inaudible 00:18:05-00:18:06]

*M. 1:* Des pois chiches, des lentilles.

*Mme. 2:* C'est pas, je ne pense pas que ce soit tellement produit [euh] dans un rayon de- de 30 kilomètre d'ici [hein].

*Mme. 1:* Ouais les miels viennent de [inaudible 00:18:17-00:18:22] et tout ça aussi y a du miel ?

*Mme. 2:* Oui, oui, oui.

*M. 1:* Oui.

*Mme. 2:* Oui mais ici, nous- nous en a- on a un apiculteur à pouvoir.

*Mme. 1:* Oui.

*M. 1:* Oui, y a des ruches qui sont un peu.

*Mme. 2:* Mais elles sont dissimulées.

*M. 1:* Un peu parfait hein.

*Mme. 2:* C'est vrai qu'il va dans les monts d'Arrée pour avoir une variété de miel à proposer aux clients quoi.

*Mme. 1:* [Bah] oui, oui, ou alors ils veulent puisque l'huile, c'est pareil, l'huile, l'huile. De quoi.

*Mme. 2:* [Bah] ? Du colza peut-être ? Alors je sais pas parce--

*M. 1:* Des lentilles et des poichiche. On cultive tout ça ici ?

*Mme. 1:* Non, non, je ne pense pas. Je sais même pas comment ça se cultive. J'avoue, je ne sais même pas trop.

*Mme. 2:* [inaudible 00:18:54-00:18:56]

*M. 1:* C'est des petites.

*Mme. 2:* Par ici [hein], non je pense pas que--

*M. 1:* Si c'est pas des graines, c'est des-- ça pousse en dehors des terres quoi. Et j'ai pas l'impression que ça, c'est bon du coup.

*Mme. 2:* ça peut se cultiver, je pense. Mais [euh] pour avoir les conditions idéales, les climatique idéales quoi.

*Mme. 1:* Ah [bah] ouais c'est vrai.

*Mme. 2:* Oh, c'est un peu compliqué [hein]. Et puis après, il faut tenir compte du climat aussi.

*Mme. 1:* Voilà, c'est ça. Alors si on prend une troisième illustration.

*Mme. 2:* Celle-là ? Rapporte !

*Mme. 1:* C'est quoi les premiers mots qui vous viennent à l'esprit ?

*Mme. 2:* Oh là là, là, je pense plutôt que c'est un- un magasin qui est un peu comme celui qu'on-- dans lequel on va quelquefois à Landerneau. Là, les primeurs. Parce que c'est- c'est présenté de de cette façon-là.

*M. 1:* Il a un beau tablier en tout cas.

*Mme. 1:* [rire]

*Mme. 2:* On va voir, on va peut-être, on va peut-être avoir le truc là-dessus. [Ben] on va voir.

*Mme. 1:* C'est un -

*Mme. 2:* Je pense que c'est plutôt- peut-être ou un magasin bio ou alors [euh] producteur de.

*M. 1:* Mon petit primeur.

*Mme. 2:* Ou alors des producteurs locaux qui fournissent le- le magasin.

*M. 1:* parce que

*Mme. 2:* On fait quand même la différence entre ça. Ces présentations-là, et puis ça

*Mme. 1:* Ouais.

*M. 1:* Les rayons font un peu plus [euh]

*Mme. 2:* Et ça ressemble plus aussi à la- la Coop bio dans laquelle on va quelquefois à Brest aussi, [hein] ? Par rapport à la présentation des produits.

*M. 1:* à moindre effort de mise en. Si je puisse-dire, à moindre effort de mise en valeur, même si ils sont bien mis en valeur quand même. Mais ça reste dans le cageot.

*Mme. 2:* Ouais.

*M. 1:* [Bah] oui, c'est comme ça.

*Mme. 2:* Je pense que le cageot est vide, [ben] ils prennent peut-être un cageot et puis le mettent à la place. Je le fais. Je pense que ça fait plus [euh] produits locaux, ça.

*Mme. 1:* Ouais.

*Mme. 2:* Après ouais. C'est pas ok mais--

*Mme. 1:* Il est à Brest.

*Mme. 2:* Il est à Brest ? Ah ouais, c'est appétissant. Ouais.

*M. 1:* Il s'appelle mon petit.

*Mme. 2:* Déprimé à celui-là.

*M. 1:* Mon petit primeur.

*Mme. 2:* Il est où à Brest ?

*Mme. 1:* Je sais même pas. Je- je cherchais une photo de primeur et puis et j'ai vu.

*Mme. 2:* Mais on voit que c'est bon. C'est quand même différent. De la présentation.

*M. 1:* Et ça, c'est où là ?

*Mme. 1:* ça où là ? Je ne sais pas. Je sais, je ne sais même pas si c'est en France, je suis aussi. Si je cherchais des des images de supermarchés, de primeurs et--.

*Mme. 2:* Et vous a vous a vu les photos que comme ça ou c'est vous qui avez [euh] qui avait un petit peu modifié puisqu'on- on a l'impression que c'est comme des- comme des surfaces, on ne voit pas. On n'a pas l'impression que c'est que des fruits, c'est- [fin] c'est tout petit quoi, petit, on a l'impression que c'est comme des- des petites perles.

*M. 1:* [diaphonie 00:21:38-00:21:41]

*Mme. 2:* C'est pris de trop haut quoi.

*Mme. 1:* Je crois qu'il y a des s- qui a des salades, des pommes ou des oranges. Je pense que c'est des gros [euh]

*Mme. 2:* Ouais.

*Mme. 1:* Voilà, ça buzze.

*Mme. 2:* Et les fruits rouges le sait. C'est peut-être des framboises ou des fraises.

*Mme. 1:* Ou je pensais plutôt des c'est plus gros encore.

*Mme. 2:* Parce que c'était nectarines. Des nectarines, pas au début, mais c'est plutôt c'est plutôt l'été. Là, sans doute non ?

*Mme. 1:* Je ne sais pas.

*Mme. 2:* Parce qu'il n'y a pas une une aussi grande variété de couleurs dans dans les fruits actuellement quand même.

*Mme. 1:* Ouais

*Mme. 2:* Si les litchi qui pouvaient donner une.

*Mme. 1:* Ouais

*Mme. 2:* Couleur rose comme ça. Mais [bon] là c'est, là c'est plus que rose ? C'est plutôt rouge.

*Mme. 1:* Ouais.

*M. 1:* [inaudible 00:21:21-00:21:22]

*Mme. 2:* [Hein] ?

*M. 1:* Mais sur celle là.

*Mme. 2:* Voilà, [bon]

*Mme. 1:* Est-ce que ça c'est un magasin dans lequel vous vous sentiriez bien ?

*Mme. 2:* Oh oui! Oh oui.

*M. 1:* Ouais.

*Mme. 2:* Après comme on n'en a pas. Si !

*Mme. 1:* Ouais. [silence 00:22:37-00:22:39] Vous n'en avez pas à proximité.

*Mme. 2:* Mais il y en aura peut-être un.

*M. 1:* Peut-être [hein], peut-être.

*Mme. 2:* Nous avons refait le hall. Apparemment, y a, on parle de maintenant, c'est-à-dire quelques magasins bio Ricoré.

*M. 1:* Non mais celui-là n'est pas forcément bio.

*Mme. 2:* Non, mais même sans être bio, si c'est production locale, c'est quand même que les fruits n'ont pas circulé pendant deux ou trois jours dans les camions réfrigérés. Ce n'est pas pareil [hein]

*Mme. 1:* Donc c'est un magasin dans lequel vous, vous pourriez y aller ? Mais en tout cas c'est pour un sens pas possible dans le sens ou il n'y a pas-- vous avez pas d'équivalent en tout cas de magasin qui s'apparente à ça ?

*Mme. 2:* Non, il n'y en a pas.

*Mme. 1:* Près de chez vous.

*Mme. 2:* Peut-être lundi 10? Mais moins précis et plus proche de sept à Saint-Renan ? Je pense bien, non ?

*Mme. 1:* Ouais. [silence 00:23:24-00:23:27] OK! [euh] si on prend la dernière image.

*Mme. 2:* Allez hop! [silence 00:23:32-00:23:35] Oh là, c'est un marché je pense. Cela était un homme idéal, non je pense. Ça me fait penser au hall Saint-Martin quand on y.

*Mme. 1:* [rire]

*Mme. 1:* Ouais. Sûrement que ce que-- Regarde, je vais poser sur les tables. Le Roi rit.

*M. 1:* En plus il y a des--

*Mme. 2:* Oui et il y a des- des stands et tout. C'est sûrement une nuage ou [euh]. Oui, oui, c'est pas- c'est pas en plein air non plus. A priori, c'est à l'intérieur.

*M. 1:* Mais c'est peut. Je croix se servir des parasols, des puits et de panneaux solaires.

*Mme. 2:* Ouais, un peu importe. Et ça.

*Mme. 1:* Et ça, qu'est-ce que ça vous évoque le marché ?

*Mme. 2:* Ah, pour moi, c'est c'est mm ça me fait penser au- ou alors au temps où j'allais sur le marché de Saint-Martin Ouais.

*Mme. 1:* [silence 00:24:36-00:24:39] Est-ce que c'est un moment ? Enfin, est ce que vous aimez bien ou c'était--

*Mme. 2:* Moi j'aimai bien oui, ouais. Tu regardes les muscles, tu vois pas des œufs comme des œufs conditionnés comme ça dans des- des marchés. Oui c'est un marché ça.

*Mme. 1:* Qu'est-ce que vous aimez bien dans le marché ?

*Mme. 2:* L'ambiance.

*Mme. 1:* Ouais. C'est-à-dire ?

*Mme. 2:* [Ben] je trouve que c'est convivial.

*Mme. 1:* Ouais.

*Mme. 2:* Et puis on a l'impression quand même qu'il y a beaucoup d'habitués. Donc les gens finissent par tisser des des liens. Pas d'amitié forcément y a- y assez fort quoi.

*M. 1:* Certains vendeurs aussi qui sont. Comment dirai-je- qui sont obligé de faire un peu la promotion de leur--

*Mme. 2:* Sont plus investis on va dire.

*M. 1:* C'est un peu. C'est plus vivant quoi ?

*Mme. 1:* Ouais.

*M. 1:* C'est pas comme quand vous arrivez ici. Ici, on se sert dans les rayons. Là [euh] peut-être que sous serre aussi. Je ne sais pas. Mais toujours utile que--

*Mme. 2:* [Bah] en général les marchés ont tous [euh]

*M. 1:* Y en a même qui haranguent les--

*Mme. 1:* [Ah] oui.

*Mme. 2:* Y a une autre ambiance où c'est pas pareil. Ça ressemble davantage aux- aux petits commerces que- qu'on a pu connaître quand on était jeune.

*Mme. 1:* Ouais.

*Mme. 2:* Et puis c'est peut-être ça aussi. On est peut-être un nostalgique de- de notre jeunesse. Et puis ça, ça ramène quand même à des -

*M. 1:* Non, parce--

*Mme. 2:* ça fait penser à des bons souvenirs.

*M. 1:* ça donne quand même du monde, que cela nous ramène nous rien qu'ici, on s'aperçoit le vendredi, c'est le, c'est le marché le vendredi matin. Et bien il y a beaucoup plus de voitures. Et beaucoup plus de voitures sur le bourg. Il y a beaucoup plus de voitures au centre Leclerc, donc les gens y viennent sans doute- sans doute parce que c'est le marché.

*Mme. 2:* Sur le plat aussi peut-être, ils doivent il faut la plupart doivent faire un tour au marché quoi.

*M. 1:* Dans Ville et celui de Saint-Renan. Le samedi, ça draine, ça donne les foules.

*Mme. 2:* C'est un peu étroit là.

*M. 1:* Oui, mais ça, il y a du monde.

*Mme. 1:* Et vous ? Où est-ce que vous faites du coup le marché ?

*Mme. 2:* Ici, on est à Ploudalmézeau. C'est sur la grande place là, en face du- du commandant de l'établissement du collège Saint-Joseph.

*Mme. 1:* Et du coup, vous vous rendez toutes les semaines ?

*Mme. 2:* On y va toutes les semaines.

*Mme. 1:* Ouais.

*M. 1:* Sauf aujourd'hui.

*Mme. 2:* Aujourd'hui, on n'a pas été. Comme il fallait qu'on le fasse vite.

*M. 1:* Donc c'est pas toutes les, c'est pas toutes les semaines, on--

*Mme. 2:* On va quasiment toutes les semaines.

*M. 1:* On y va régulièrement.

*Mme. 2:* On achète des fruits [euh] et parfois des -

*M. 1:* Mais il n'est pas très- il est pas très achalandé.

*Mme. 1:* Ouais.

*Mme. 2:* L'été, c'est un peu mieux.

*M. 1:* Et dès que, pendant qu'on y est.

*Mme. 2:* Non mais, ça marche bien quand-même.

*M. 1:* Je comprends, mais je comprends les les gens. Les marchands aiment comme il y a, comme Il y a vraiment un temps dégueulasse qui a été, qui a été annoncé. Il n'y a pas foule.

*Mme. 2:* Et puis, il faut être courageux pour tenir un stand quand ça souffle et qu'y a de la pluie [hein]

*Mme. 1:* Ouais. Vous, vous y allez quand même quand il pleut ?

*Mme. 2:* Oh oui, [ben] si on a prévu d'aller acheter des fruits et des lé- des fruits. Oui, on y va.

*M. 1:* Le défaut n'est pas long, a conclu. C'est arrivé qu'elle soit pas là.

*Mme. 2:* [Ben] en plus elle est- elle est absente pendant parfois elle ferme de--

*M. 1:* Fin septembre.

*Mme. 2:* Non, non. Même fin, l'été.

*M. 1:* Fin l'été oui.

*Mme. 2:* Pendant deux mois d'affilée, elle est pas là.

*M. 1:* Fin juillet à fin septembre.

*Mme. 2:* C'est qui elle?

*M. 1:* [Bah], c'est le verger de.

*Mme. 2:* De Ke- Kerry en bléo là.

*M. 1:* Oh oui Kerry en bléo. Et sec. Vous n'allez pas dire ça.

*Mme. 1:* Mais ci. Du coup, c'est la. Ouais.

*Mme. 2:* Non mais je pense que comme ils ont pas mal de. Ils ont pas mal de de bergers. Elle en profite aussi [euh], je crois, pour [euh]. Mais Iouri sont.

*M. 1:* Ils ont, ils ont du travail à faire.

*Mme. 2:* Voilà, il y a eu des plantations, peut-être, ou peut- être l'arrachage de de pommiers ou de poiriers qui sont arrivés. Un bout de course quoi.

*M. 1:* C'est peut-être plus, c'est plus la saison. C'est moins je trouvais très bizarre que pendant l'été, alors que 'en principe, il y a plus de monde quand même quoi. Enfin, il n'y a pas moins, et je veux dire, il n'y a pas moins de monde, même s'il y a des plus amusant qui parle. Très bizarre quand même qu'en été ne soit pas là du tout. Puis [bah] après ils ont nous a un peu expliqué. Oui que [bon] c'est vrai qu'il y a quand même--

*Mme. 2:* Elle va beaucoup en Turquie, au moins une bonne chose qu'elle achète des fruits secs, elle doit négocier ses prix peut-être je pense--

*M. 1:* Mais puis des fois il y a des travaux aussi de comme tu dis, de soit d'élagage, soit même, tant il.

*Mme. 2:* Y a du travail quand même.

*M. 1:* De faire des replantations de pommiers. Donc elle a dû prendre l'habitude que finalement tel quel, ça me fait peut-être pas un chiffre d'affaires aussi important là que les autres.

*Mme. 2:* Ouais, je suppose.

*Mme. 1:* Et l'ambiance dont vous parliez [euh] vous parlez du-- notamment du-du marché que vous aviez l'habitude de faire à Saint-Martin ?

*Mme. 2:* Non, mais c'est vieux [hein]

*Mme. 1:* Et vous ne trouvez pas cette ambiance là dans le marché de Ploudalmézeau ?

*Mme. 2:* Oh, si ! Il est sympa aussi [hein] ?

*M. 1:* Non mais y a quand-même beaucoup moins de--

*Mme. 2:* Y a moins de- de d'of- l'offre est à même moins importante.

*M. 1:* Il y a pas mal d'étals de fruits et légumes.

*Mme. 2:* Oh, il y a quand même un poissonnier.

*M. 1:* Mais oui. Oui, oui, oui, mais--

*Mme. 2:* [inaudible 00:29:42-00:29:44] aussi [hein] qui vend des moules [euh]

*M. 1:* Des fruits de mer et tout. Un peu de poisson.

*Mme. 2:* Un peu de poisson, c'est ponctuel quoi c'est pas- il est pas là toute l'année, non ?

*M. 1:* En plus de l'assumer. C'est logique puisque c'est quelque chose qui vient d'un côté. Même s'il n'y en a pas, il y en a pour.

*Mme. 2:* Le crêpier aussi de Plouguin qui vient dans la rue qui fait- qui propose des crêpes chaudes. Nous on n'achète pas de crêpes.

*Mme. 1:* On fait nous-même. OK. Est-ce que ça vous arrive de jeter des fruits et légumes que vous aviez acheté sur le marché ou pas ?

*Mme. 2:* Moi je mentirais en disant que on jette jamais jamais [hein]

*Mme. 1:* Ouais,

*Mme. 2:* Mais [bon] on évite.

*M. 1:* C'est rare encore le manant, qu'on jette en plus un peu depuis qu'on fait faudrait c'est encore pire que.

*Mme. 1:* Faire encore plus attention.

*Mme. 2:* Oui mais c'est vrai que ça sen- sen- sensibilise le fait d'avoir la poubelle, la- les petits enfants. Rigoler cet été me dit mais pourquoi vous faites ça avant ? Je dis alors je leur ai montré le livre. Vous voyez un petit, un abricot qui était gâté. Je dis on le mêle et ça fait peut-être 50 grammes. Mais si on fait ça multiplié par dix, multiplié par 20, un petit millier. Ça fait beaucoup.

*Mme. 1:* Et oui, ça peut monter. Alors oui. Au hasard que vous jetez des fruits et légumes du marché. Et [euh] qu'est-ce que vous ressentez si jamais vous êtes amené à le jeter ?

*Mme. 2:* Pareil, je me dis, paraît-il, la même chose qu'Achille. De toute façon on se dit on n'a pas bien gérés, [hein] ?

*Mme. 2:* [Ah] selon vous, y a pas une différence entre ces différents lieux. De votre ressenti au moment de jeter. Peu importe, il vient de votre potager, du marché ou de la grande surface. Votre sentiment est le même au long de l'été ?

*M. 1:* Moi-même, personnellement, je serais plus contrarié si c'est un magasin, un producteur local et un petit peu bio ou complètement bio. Je serais plus contrarié de jeter quelque chose qui vient de [fin] mort [hein] qui vient de ce magasin-là, que d'un magasin [euh] comme on va par exemple, Libre service Leclerc quoi. Parce que- ils se sont peut--

*Mme. 2:* Ils sont peut-être plus impliqués, il y a--

*M. 1:* Y a plus d'implication de leur part.

*Mme. 2:* Et de là je pense. En fait, on est plus impliqués parce que [ben] je pense que lui, il est convaincu le-

*M. 1:* Que sur le plan--

*Mme. 2:* De la qualité des produits qu'ils proposent, je ne vais pas dire que l [euh], mais [bon] il n'y a pas le même, il n'y a pas [euh] je pense qu'il ne ressente pas la même chose vis à vis des produits.

*M. 1:* Je vais vous donner une anecdote, mais c'est pareil, vous ne trouvez pas ?

*Mme. 1:* Non

*Mme. 2:* C'était enregistré.

*Mme. 1:* Ça reste entre.

*M. 1:* Nous. Vous a t il fait beaucoup de confitures ? En l'occurrence, le fait que je tire d'abricots. Donc elle est toujours à l'affut parce que vous le savez, le coup, ça coûte ou ça coûte plus ou moins cher. Et puis souvent, il y a des périodes om ils vendent des abricots pour de la confiture. Et justement, il y en avait- il avait mis dans le prospectus abricots à confiture, cagettes d'abricots à confiture. Un kilo sur votre produit. Ben tiens, tiens, on va leur racheter vous ? Sincèrement, il y avait une pile de cageots comme ça. Sur le premier, il y en avait une dizaine qui était gâté.

*Mme. 2:* J'ai déjà été- j'ai été dégouté.

*M. 1:* Sur le deuxième pareil, sur le troisième pareil. Alors c'est du-[bon]. Peut-être que c'est parce que ceux-là sont peut-être là depuis deux ou trois jours. On verra demain. On est revenu. On est allé voir le lendemain pareil. J'ai interpellé le chef de rayon.

*Mme. 1:* Oui.

*M. 1:* Je lui ai dit, vous avez quand même beaucoup de-- c'est quand même beaucoup--

*Mme. 2:* De fruit gâté.

*M. 1:* Fruits gâtés dans les rayons. Il m'a répondu : On s'en servira pour la confiture, ce n'est pas grave. Vous voyez ! [bon], ce n'est pas lui qui les produits [hein] c'est sûr !

*Mme. 2:* Il avait- il n'avait aucun remords quoi.

*M. 1:* Déjà pour le vendeur que c'est pas très top top parce qu'il ne m'a même pas discuté avant. Il m'a répondu ça texto. Pour ma part, il n'y a pas continue après. Voilà. Je me disais peut-être qu'il va me dire oui, effectivement, [bah] écoutez [euh] je vous fais la caisse. C'est pas ce que je recherchais, [hein.] Mais je vous fais la caisse à moitié prix ou-- Non, non, non. Non, non.

*Mme. 2:* Aucun état d'âme, aucun état d'âme. Et je pense que [euh] ce monsieur, je sais pas si c'est le patron ou si c'est qu'un employé, mais je pense qu'il est- il prétendrait pas de la même façon ?

*Mme. 1:* Oui.

*Mme. 2:* Parce qu'il connaît les gens à qui il achète, probablement dans les cas, les gens. Donc il y a quand même une relation qui s'est établie.

*M. 1:* Je pense que c'est la dame chez qui on achète les pommes sur le marché. Si jamais un jour on lui disait ou si, si, deux, mercredi, vendredi ou deux ou trois ven-, deux, deux, trois vendredi, dans deux ou trois mois, on lui disait qu'on a trouvé les fruits, ce ne sont pas conservés. Je pense effectivement que c'est la loi.

*Mme. 2:* Elle serait contrariée.

*M. 1:* Est-ce que ça va compter ?

*Mme. 2:* Oui.

*Mme. 2:* Je pense qu'il n'a pas la même approche.

*M. 1:* Du tout, c'est ça-- Ça, c'est quand même de leur production.

*Mme. 2:* Oui.

*Mme. 2:* Parce que je pense qu'il arrive. On a la fin d'un circuit, c'est que des vendeurs en fait.

*Mme. 1:* Oui.

*Mme. 2:* Il faut dire ce qu'il est [hein], c'est que des vendeurs, ils sont là pour vendre.

*Mme. 1:* Et encore. Les gens qui s'occupent des rayons. Ils sont juste missionnés pour remettre les fruits en place.

*Mme. 2:* Voilà, voilà, ils font un travail utile, ça- ça c'est sûr, ça c'est sûr. Mais je pense qu'il n'a pas la même application.

*Mme. 1:* Non, non.

*Mme. 2:* Il n'y a pas le même rapport. Le rapport avec les fruits et légumes est différent selon qu'on se place là, là, là ou là, c'est pas assez, pas pareil.

*Mme. 1:* Ouais. Et c'est bien possible que la personne a qui- qui dit vous avez parlé du cageot d'abricots. On lui dit de faire des cagettes d'abricots qui commençaient à être un peu fatigués.

*M. 1:* Mais si pour la confiture, il m'a répondu textuellement.

*Mme. 1:* Oui, pour la confiture, c'est.

*Mme. 2:* Pas moi, c'est grave comme ça. Moi, je sais que les fruits gâtés, moi je ne mets pas dans la confiture.

*Mme. 1:* Ouais.

*M. 1:* Non, je dis à la rigueur, il y en a.

*Mme. 2:* Qui ont des moisissures.

*M. 1:* Il y en a- il y en a eu. Effectivement, si tu vas, tu vas pas mourir. Mais je doute, ce n'est pas moi. D'un point de vue, c'est pas, ce n'est pas correct que bon.

*Mme. 1:* Oui.

*M. 1:* Personne ne m'a dit que les abricots un petit peu moisi, c'était. Cet éboulement, c'est un peu de repos. On vous dit que je suis sûre, donc c'est bon. Mais c'est vrai quoi. Donc ça me paraissait un peu juste. J'ai trouvé ça un peu- un peu dur.

*Mme. 2:* D'autant plus que peut être que il n'accepterait pas, lui, de manger de la confiture faite avec les abricots qu'il était en train de vendre.

*M. 1:* Après ça, c'est le jour et c'est sans doute le problème des grandes surfaces parce que là il y a du est tellement de flux.

*Mme. 2:* Il faut faire du chiffre.

*Mme. 1:* Bruxelles se préoccupe pas trop de.

*Mme. 2:* Non, c'est pas, c'est pas pareil [hein] C'est pas le même approche [hein]

*Mme. 1:* Oui. OK. [Alors] est-ce que vous vous mangez de la banane ?

*Mme. 2:* Oui [Ben] oui.

*Mme. 1:* Effectivement. [Alors] Afin qu'il soit un de vous deux qui commencez. Que ce soit vous n'avez pas la même, la même approche sur la banane [alors] ? Je me souviens avoir discuté, [alors] je le mélange. Il y a dix photos de banane et je vais vous demander de les ranger.

*Mme. 2:* Par ordre que ce soit mûr ou pas mur.

*Mme. 1:* Ou non, celles qui vous donnent le plus envie ? Celle qui vous donne le moins envie.

*Mme. 2:* Tout en y allant ? Pas du tout. Elle doit être toute molle à l'intérieur. Allez, tu vas m'aider [hein] ?

*M. 1:* Non, non, chacun son --

*Mme. 2:* Chacun son tour.

*Mme. 1:* Vous pouvez faire votre rangement, je vous le dirai après. Moi, je n'en ai pas fait comme ça. Mais c'est bon vos goûts à vous. Donc cela ne m'inspire pas du tout. Et de.

*Mme. 2:* Tout cela, je le sais là, il finirait au four [hein]. Cela dit, elle n'est pas non plus assez murs.

*M. 1:* C'est la même variété de bananes.

*Mme. 1:* C'est la même banane.

*Mme. 2:* Moi, je ne fais pas beaucoup de différence.

*M. 1:* C'est carrément la même banane.

*Mme. 1:* C'est la même banane ouais.

*Mme. 2:* Ça va être encore pire. Celle-là elle a l'air-- elle a l'air tout--

*M. 1:* En plusieurs étapes, de--

*Mme. 2:* Déformée.

*Mme. 1:* Oui.

*M. 1:* Plusieurs étapes de sa vie.

*Mme. 2:* En cela, elle commence juste là et il y a peu de jaune. Celle-là elle est- elle commence à être vraiment bizarre. Alors à moins d'une journée tranche. C'est comme ça. Je savais comment on serait peut s'y attacher. Dans les shorts. C'est classe ?

*M. 1:* Classe comment ?

*Mme. 2:* C'est comme ça, je. Celle que je préfère, celle que je préfère.

*M. 1:* Mais tu- tu devais en manger là. Tu mangerais celle-là !

*Mme. 2:* Non, elle est pas si mur.

*M. 1:* [Ah] [bah] [alors]

*Mme. 2:* C'est pour voir- c'est pour voir. Moi, celle-là me plaît bien.

*Mme. 1:* Ça serait celle-là la première ?

*Mme. 2:* La première. Et puis [euh] . Éventuellement je croix que ça.

*M. 1:* Non mais il est dans l'ordre [hein].

*Mme. 2:* Celle-là.

*Mme. 1:* Ouais; mettez dans l'ordre.

*Mme. 2:* De préférence ?

*Mme. 1:* C'est ça. Celle qui vous donne le plus envie d'être mangé.

*Mme. 2:* Il faut en mettre combien ?

*Mme. 2:* Vous les ranger ?

*M. 1:* Toute.

*Mme. 2:* Oui, d'accord. D'accord.

*M. 1:* Pose les deux [hein].

*Mme. 2:* Pour cela, je les mangerais.

*M. 1:* Mais laquelle ? Lesquels ?

*Mme. 2:* Celle-là que je préfère.

*M. 1:* Donc c'est celle-là. Après, c'est celle-là. Après, c'est celle-là.

*Mme. 2:* Ouais. Et après, c'est celle-là ?

*M. 1:* Oui.

*Mme. 2:* Et après, à vrai dire. Bonne note. Tout dépend comment on l'a mangé, si elle est cuisinée, éventuellement celle-là qu'on met.

*Mme. 1:* Je prends une photo de votre rangement.

*Mme. 2:* Si elle est- si elle est cuisinée comme elle avait du citron ; un peu. Mais [bon], c'est pas forcément. C'est pas forcément rationnel non plus.

*Mme. 1:* Tac.

*M. 1:* Tu vas comme ça.

*Mme. 2:* Mais celle-là, je l'ai mangé quoi.

*Mme. 1:* A partir de laquelle [euh] --

*Mme. 2:* Celle-là, ces trois-là, je ne mangerai pas.

*Mme. 1:* Donc là, là en jaune vous ne mangez plus ? Est-ce que vous la- carrés bleu, vous la mangez quand même ?

*Mme. 2:* Oui, oui.

*Mme. 1:* Et à partir de là, en jaune. Du coup, vous faites quoi de la banane ? Vous la jetez ?

*Mme. 2:* [Ben] je les ouvre, je regarde comment ils sont. Eventuellement, je les fais au four, mais si elles sont toutes molles, toute couleur jaune et orangé.

*Mme. 1:* Elles vont en poubelle ou en composteur.

*Mme. 2:* En composte.

*Mme. 1:* Et du coup, pourquoi c'est celle-ci votre préféré ?

*Mme. 2:* Parce que celle-là, elle est mûre, mais elle est juste à point, je trouve. Elle est bien.

*Mme. 1:* Ouais.

*Mme. 2:* Elle est bien celle-là. [Bon], elles ne sont pas, sont pas toutes mériteraient d'être peut être un tout petit peu plus mûres.

*Mme. 1:* Ouais, attendre encore un peu pour les deux.

*Mme. 2:* [En fait] [bon] après je ne vois pas. Je ne vois pas une grande différence. Par exemple, celle-ci, je pense qu'elle est plus mûre que n'est celle-là. Elle est, elle fait paraît plus jaune. Ceci un petit peu verte qu'elle même encore sur le côté.

*Mme. 1:* Et cela, vous pouvez la manger aussi ?

*Mme. 2:* [Ah] oui oui !

*Mme. 1:* Et quand elles sont vertes comme [inaudible 00:41:12-00:41:13]

*Mme. 2:* [Alors] je les laisse. Ce sont-- elles ont pas de goût [enfait] [hein]. Elles sont comme ça. Jusqu'à là, elles sont--. Tout est à la limite. Si on a été plus rationnel de mettre ceci avant cela.

*Mme. 1:* Là, ça va, vous êtes.

*Mme. 2:* Celle-là, je la mangerai pas- je la mangerai pas d'emblée, non.

*Mme. 1:* Celle-là, y a. Vous pouvez quand même la manger.

*M. 1:* Je l'aurais même mise à la fin parce que celle-là mangerai pas non plus. Moi, personnellement, je la mangerai pas non.

*Mme. 1:* Maintenant faites votre tour de rangement.

*Mme. 2:* [Bah] vas-y !

*M. 1:* Là, ça n'a pas varié beaucoup. Moi, j'aurais fait [euh]

*Mme. 2:* Tu préfères plus mûre que moi [hein]

*M. 1:* Comme ça, comme ça. Celle-là me paraît quand même bien.

*Mme. 2:* Mais celle-là elle est bien aussi, à l'intérieur de la banane est bien.

*M. 1:* Oui après si c'est que du visuel qu'il.

*Mme. 2:* Faut ne faut pas s'arrêter qu'au visuel.

*M. 1:* [Ah] bah ça dépend, mais tu peux savoir comment il est.

*Mme. 2:* Après, tout dépend si on la mange comme ça ou si on l'a --

*M. 1:* Je trouve que celle-là pour moi est un peu limite de ce que je touche. Et moi j'aime bien les bananes.

*Mme. 2:* Moi c'est ce que je te dis. Elle est un peu verte celle-là, tu la mangerai pas.

*M. 1:* Non, je la mangerai pas. J'attendrai qu'elle murisse. Et celle-là, je la mangerai au four.

*Mme. 2:* Voilà u four. C'est toi qui la mangerai au four. Moi, je ne vais pas dire que je vais les manger parce que je ne mange pas au four.

*Mme. 1:* [Ah] oui ? Et est-ce que vous faites d'autre préparation avec la banane ou pas ? Ou c'est juste le four qui va- c'est juste au four.

*Mme. 2:* Moi c'est juste au four avec du citron, un peu de- un peu de sucre. Et puis [euh].

*M. 1:* Et puis tu fais quoi d'autre ?

*Mme. 2:* Et puis un tout petit peu de beurre pour doré.

*Mme. 1:* Mais ça va être bon.

*Mme. 2:* Je trouve. Ça, c'est finalement les bananes cuisinées, c'est c'est assez écœurant. Oui mais bon, on aime ou on n'aime pas.

*Mme. 1:* C'est oui. C'est un goût

*Mme. 2:* Pas des tartes ou des non. Non, je trouve que c'est pas. C'est pas très rafraîchissant comme un peu pâteux quand c'est--

*M. 1:* Il y a des gens qui vont jouer celle-là ?

*Mme. 1:* Ouais, alors oui, celle-ci [euh], celle-ci oui.

*Mme. 2:* [Bah] elle est- elle n'est pas complètement gâtée celle-là [hein]. Y a des gens qui la mangent crue encore comme ça.

*M. 1:* Il la mangerait cru ou pas, pour pas gâter ou bien--.

*Mme. 2:* [diaphonie 00:43:23-00:43:25] quand même.

*M. 1:* J'aime bien.

*Mme. 1:* Y en a qui aiment. À partir de là, c'est rare où les personnes disent qui la cuisine mette dans des gâteaux ou-- des crêpes ou-.

*Mme. 2:* [Ah] oui, peut-être que dans des crêpes, finalement [euh] ça peut le faire aussi.

*Mme. 1:* Mais dans la patte quand même. Dans la patte à pancake.

*Mme. 2:* [Ah] oui.

*Mme. 1:* Moi j'aime bien la banane, mais c'est rare que je l'apprécie cru. J'attends qu'e- je les laisse arriver jusque-là où je me dis "[Ah], c'est trop tard" et je les mets --

*Mme. 2:* [Ah] même comme ça, vous la mangeriez pas ?

*Mme. 1:* [Ben] je prends, mais je saisi, je prends pas trop de plaisir en fait, à manger la banane crue. J'attends qu'elle soit comme ça pour en faire des gâteaux. Les faire dans des gâteaux.

*Mme. 2:* Oui, oui, mais après, c'est vrai que c'est une question d'habitude aussi [hein].

*M. 1:* Moi, j'aime bien la banane.

*Mme. 2:* Toi, t'aimes bien les bananes ?

*M. 1:* Et il y a des différences. Moi, j'ai vécu trois ans en Martinique quand j'étais plus petit et dans mon jardin, on avait des bananiers.

*Mme. 1:* Ça, c'était peut-être le même.

*M. 1:* Que vous mangez une banane. Et mon père m'a amené des fois au port. Mais il était en garde et charger et charger les bateaux parce que c'est un grand port. La Martinique produit de la banane, il chargeait les bateaux de bananes, les régimes sont ouverts. Ouais donc ils mûrissent. À l'époque, le bateau mettait huit, dix jours à faire la traversée. Il mûrisse un petit peu pendant la traversée.

*Mme. 1:* [ah] oui, c'est ça.

*M. 1:* ça n'a rien à voir.

*Mme. 1:* Du coup, et sinon, sur place, est-ce que des fois il y en a- Il y a des bananes qui restent à l'arbre et que vous pouvez décrocher une fois qu'elles sont déjà bien jaunes ? Oui, ça doit pas être le même --.

*Mme. 2:* Elles sont plus petites aussi les bananes.

*M. 1:* [Ah] [bah] non ! Puisque j'avais, y avait avait une variété de banane sur tout les bananes normales. Comme vous dites, on les cueillait sur l'arbre.

*Mme. 2:* Ça pas, ça n'a pas la même saveur, ça, c'est clair.

*Mme. 1:* Ah non. Elles ont eu le plus de temps de prendre encore.

*Mme. 2:* Et les bananes qu'on trouve en grande surface, elles ont été traitées aussi pendant le transport pour les obliger à mûrir. On l'a vu, je ne vois pas des reportages, on les pique la, pour les-, pour les obliger à murir.

*M. 1:* Pas forcément, on les mets dans les-- [bon] déjà elles sont conservées. Mais [bon], je sais pas si vous n'êtes pas dans des [bah] dans des-- dans des cases spéciales pour qu'elles mûrissent un petit peu plus.

*Mme. 2:* Le cage, j'avais vu, mais j'avais mis des petites affiches, ça c'est pas vieux et j'expliquais alors parfois- parfois les deux extrémités sont toutes- toutes vertes. Et quand et quand-- y parfois là c'est- là c'est- c'est tout marron, c'est tout vert. Par contre, j'ai plus, je m'en peux plus des explications qu'ils donnaient, mais en fait c'est- ça venait du traitement qu'avaient subi les bananes.

*Mme. 1:* Subi les bananes. Oui, je sais que c'est des indicateurs comme ça.

*Mme. 2:* Oui, mais alors parfois c'est ouvert. Les deux extrémités sont vertes et parfois c'est bien. C'est plutôt de pas aussi prononcé que ça, mais plutôt marron là.

*Mme. 1:* Ouais. Et est-ce que, selon vous, il y a un moment donné où la banane, elle peut être dangereuse pour l'organisme ou pas ?

*Mme. 2:* Dangereuse, je crois pas.

*Mme. 1:* Non ?

*Mme. 2:* Je crois pas.

*Mme. 1:* C'est pas des choses qui pas ---

*M. 1:* Moi j'ai pas l'imp-, Je ne pense pas qu'un fruit qu'on appelle pourrit. A condition qu'on le ramasse pas par terre. Et je ne pense pas qu'il soit nocif pour l'organisme. C'est plus petit, puis, entre guillemets, c'est plutôt sympa. Le téflon, c'est plutôt dégoûtant. [Bah] moi je sais pas. Est-ce que j'ai raison non ?

*Mme. 1:* [Alors] faudrait demander aux biologistes [alors] je-- justement ce qui m'intéresse c'est [enfin] est-ce que si vous décidez de jeter les fruits et légumes, est-ce que c'est parce que vous pensez qu'il peut être mauvais ou c'est juste qu'il est mauvais en bouche ? Et ça vous --

*M. 1:* [Ah] oui, je pense qu'il y a-- je pense qu'il y a un peu cette--

*Mme. 2:* Un peu ce dégoût quoi.

*M. 1:* Oui c'est p-, c'est le faite-- comment on peut appeler ça le fait-, on se dit que le fait qu'un fruit soit pourri ou a une pourri, il n'est pas bon au sens ou il n'a pas un bon goût. Des fois, il n'est pas bon non plus parce qu'il peut être justement [euh]-

*Mme. 2:* Y a peut-être une prolifération de microbes quoi peut-être.

*M. 1:* Peut-être, mais y a cette image-là, qui perdure peut-être depuis [euh] je sait pas depuis--

*Mme. 2:* Mais je pense que si on avait vraiment faim, vraiment, vraiment, on avait rien d'autre à manger, on mangerait bien celle-là.

*Mme. 1:* [Ben] oui.

*Mme. 2:* C'est clair [hein]. C'est parce qu'on est un peu gaté aussi [hein]. C'est bien ce qui est [hein]

*M. 1:* Mais il faudrait que vous demandiez à un biologiste si c'est préjudiciable à la santé, [Ah] Évidemment, si on mange un régime de bananes pourries, peut être que là, effectivement, oui.

*Mme. 1:* Sur la durée, on va peut-être- peut-être nos intestins s'habituer à ça.

*Mme. 2:* [Ben] oui.

*Mme. 1:* Mais je crois que l'un- l'autre labo y travaille, ils sont fous de restes. Ils ont une forêt qu'on regarde, mais il y a des fruits ou des légumes ou [euh]

*M. 1:* Qui sont nocifs.

*Mme. 1:* Oui, il y en a qui pour qu'il n'y a pas de problème puisque les peaux sont épaisses, font ça, ça ne passe pas sur le--, y en a ou par conséquent, apparemment, quand ils deviennent pourris, il y a quand même un.

*M. 1:* Risque de temps de.

*Mme. 1:* Micro-organisme qui devient encore [euh]. Mais [bon], je crois que ça reste minime. Donc si on en prend un ou deux comme ça, ça fait rien. Mais si on n'a qu'une alimentation que.

*Mme. 2:* [Ben] oui, je pense que ça peut quand même être dommageable.

*Mme. 1:* C'est vrai que je pense qu'il y a de l'acidité qui arrive, que je conseille sur la durée. Il pourrait y avoir.

*Mme. 2:* ça détraque un peu l'estomac quand même [hein].

*Mme. 1:* C'est possible, mais en petites quantités.

*Mme. 2:* Des trucs comme ça, je pense que gâtés.

*M. 1:* Non, mais il y a le fait que ça peut ne pas être bon. Parce que si tu es habitué au goût d'un fruit, à un fruit et à son goût, si tu n'as- si tu n'as que si tu n'as pas le ressenti de ce goût là en mangeant tel fruit, oui, tu vas dire qu'il n'est pas mou.

*Mme. 2:* Oui, c'est vraiment ça, mais on s'attend à quelque chose quoi.

*M. 1:* Autant. Qu'il est gâté ou qu'il est préjudiciable à la santé.

*Mme. 1:* Et sur les apports nutritifs. Est-ce que vous avez [euh]-- l'idée de ce que [inaudible 00:48:46-00:48:47] où la banane est à son optimum pour ces apports nutritifs.

*Mme. 2:* Plus elle est mûre et plus elle nutritive.

*Mme. 1:* Pour vous. Mais est-ce- ce qui m'intéresse c'est ce- est ce que vous vous êtes déjà posé la question ou qu'est-ce que vous pensez ? Est-ce que ça peut être ça ? Peut-être parce que ou selon vous, la banane reste la banane. Elle a le même apport.

*Mme. 2:* Non, je- je pense. Mais c'est plus l'avoir lu que

*Mme. 1:* Ouais.

*Mme. 2:* Que une banane bien mûre est plus [euh]

*M. 1:* Nutritive et que les bananes vertes.

*Mme. 1:* [Bah] ouais

*Mme. 2:* Une banane verte de toute façon, c'est difficile à manger.

*M. 1:* Ouais c'est un peu aussi lié au fait qu'on mûre maturité. Qui dit maturité en pleine force de l'âge, donc je ne sais pas. C'est peut être aussi des épithètes. C'est peut-être des idées reçues ou des--

*Mme. 1:* Ouais.

*M. 1:* C'est vrai que d'un point de vue purement pratique et comestible, personnellement, je trouve que c'est plus agréable de manger une banane comme ça, comme ça, comme ça ou comme ça qu'une banane verte quoi.

*Mme. 2:* C'est pas digeste de manger une banane verte quand même.

*Mme. 1:* Ouais, c'est un peu--

*M. 1:* C'est dur.

*Mme. 2:* Elles sont pas dures forcément, mais-- elle sont pas dur, elles sont-- elles sont fanes [en faite] pas de goût.

*Mme. 1:* Oui, mais les personnes qui les préfèrent, il y a des, ça qui rigole avec la banane et des personnes à partir d'ici. Ils arrêtent de la manger presque.

*Mme. 2:* C'est trop robut.

*Mme. 1:* Ouais.

*Mme. 2:* Ils mangeront ça.

*Mme. 1:* Plus facilement, même si ça va, elles préfèrent quand même attendre un peu.

*Mme. 2:* C'est effectivement plus mature.

*Mme. 1:* Ça va être ces trois-là et une fois que ça arrive là, c'est- ça commence à être trop sucré pour eux ou trop mou ou trop--.

*Mme. 2:* Oui, c'est vrai qu'il faut reconnaître que plus elle est- elle est mûre et plus elle est sucré. Et peut-être plus elle est calorique.

*Mme. 1:* [Ah] ouais. Je ne sais pas. Justement, est-ce que du coup--

*Mme. 2:* c'est vrai que ce serait intéressant de savoir ?

*Mme. 1:* Ouais.

*Mme. 2:* Je pense que c'est plus de calories. Si c'est--

*Mme. 1:* Plus sucré, mais un peu plus ultra, c'est moins digeste à ça. Souffle, c'est plus lourd quand même. Mais [bon] je sais pas.

*M. 1:* Oui, mais au départ, a priori. Au départ, quand on regarde une banane. Je ne sais pas, mais c'est vrai que cela peut paraître plus appétissante que celle-là ou que celle-là ou celle-là puisqu'elle n'a pas de taches, y en a du tout.

*Mme. 2:* Ouais enfin c'est en surface.

*M. 1:* Oui mais justement.

*Mme. 2:* C'est pas parce que la peau est comme ça que le--

*Mme. 1:* L'intérieur est comme ça.

*Mme. 2:* C'est juste l'enveloppe du fruit [hein]

*M. 1:* Oui mais si on ne peut pas éplucher les enveloppes et protéger les bananes avant de les acheter.

*Mme. 1:* Oui.

*Mme. 2:* Non mais [enfaite] tu vois comme un peu par rapport à la peau, c'est quand même assez révélateur. Mais celle-là, celle-là surtout. Elle est peu appétissante.

*Mme. 1:* Elle commence à-- voilà

*Mme. 2:* Elle est tout flasque. On a l'impression qu'on la prend elle--

*Mme. 1:* Ouais, ouais. C'est bien de composer quand même.

*Mme. 2:* Mais bon, ceci étant si on a vraiment faim les mangerait quand-même.

*Mme. 1:* Je pense qu'on est sur une île déserte si on n'avait que des bananes et que--

*Mme. 2:* Et voilà. Je pense que oui.

*Mme. 1:* On ne dirait pas. Non.

*Mme. 2:* Non, c'est clair.

*Mme. 1:* [Alors] dernier petit [euh] petit jeu. [Alors] Mehdi, vous avez un composteur ? D'accord, est-ce que des fois vous mettez des fruits et légumes dans une poubelle classique ou pas ? Ou tout finit dans le compost.

*Mme. 2:* Toi tu tri [hein], tu tri un peu. [diaphonie 00:51:39-00:51:42]

*M. 1:* C'est-à-dire--

*Mme. 2:* Est-ce que- est-ce que tous vos fruits et légumes vont dans le compost ou des fois, ça oui.

*M. 1:* Sauf s'il y en a. Sauf si par hasard--

*Mme. 2:* Tu mets pas les, tu mets pas les citrons.

*M. 1:* Par contre, [diaphonie 00:51:50-00:51:52]

*Mme. 2:* Parce qu'à ce qui parait c'est pas bien de mettre les citrons et les--

*Mme. 1:* Les agrumes pour la.

*Mme. 2:* Ma belle-fille, elle m'a dit que les épluchures de pommes de terre c'est pas bon non plus pour le compost.

*Mme. 1:* Ah bon ?

*Mme. 2:* Parce qu'on leur a dit, j'habite dans le Nord, alors que tout le permet.

*Mme. 1:* Aussi parce que les agrumes, je trouve que ça tue justement les verts. Il ne faut pas, il ne faut pas, c'est tout l'intérêt du compost.

*Mme. 2:* Fait.

*M. 1:* Les pour les plus d'agrumes, il disait qu'il fallait les découper, ne jamais mettre, mettre une demi peau d'orange, demi orange parce que ça met du temps. Alors au début, je l'ai coupé, puis après j'ai lu ailleurs.

*Mme. 2:* Ce qui paraissait pas peu.

*M. 1:* Elle a été pillée. On reçoit de temps en temps des petites brochures là-dessus. Oui, c'est ça une autre SCPI. Ils se sensibilisent.

*Mme. 2:* À eux, ils sont vachement de sensibilisation.

*M. 1:* Et donnons imiter qu'il valait mieux éviter de mettre des pierres d'agrumes. Maintenant, je mets plus.

*Mme. 1:* D'efforts pour arriver là. Est-ce que vous avez des poules ? Non, pas de poule.

*Mme. 2:* Pas de poules, parce que ça, quand on a des poules, il y a des rats.

*Mme. 1:* [Ah] oui ? Le Falcon peut spécial.

*Mme. 2:* Oui, oui aussi. Même si--

*M. 1:* On en a eu- on en a eu.

*Mme. 2:* Ouais ouais ouais ouais. Mais pas encore pas hésité. Ils allaient voir le poulla- le poullaillé à coté--

*Mme. 1:* Alors est ce que des fois avec des fruits et légumes, vous pouvez faire notamment des légumes ? Vous pouvez faire des poêlées, des juliennes ? Oui, des Ratatouille.

*Mme. 2:* La ratatouille, voilà toujours. Deux.

*Mme. 1:* Est-ce que ça vous arrive de faire des gâteaux à base de fruits ou pas ?

*Mme. 2:* Oui des fois. Des tartes aux pommes.

*Mme. 1:* Ouais. Est-ce que ça vous arrive de faire des salades de fruits ?

*Mme. 2:* Ah oui. Oui, toujours en été quoi.

*Mme. 1:* Oui, oui sont des gaspachos.

*Mme. 2:* Non,

*Mme. 1:* Des soupes froides.

*Mme. 2:* C'est un peu trop. J'aime bien, mais on achète.

*Mme. 1:* Oui, mais c'est pas très répandu en Bretagne je crois.

*Mme. 2:* Non. Mais pourtant, c'est pas dur à faire.

*Mme. 1:* Non, non, il faut une soupe chaude.

*Mme. 2:* Mais voilà, tout à fait, c'est bon.

*Mme. 2:* Est-ce que ça vous arrive de congeler vos produits frais ?

*Mme. 2:* Non, j'aime pas moi.

*Mme. 1:* Non

*M. 1:* Un adepte de la conjuration.

*Mme. 1:* La compote, est ce que ça vous arrive ? Oui

*Mme. 2:* Oui, très souvent.

*Mme. 1:* Souvent, des salades de l'humidité,

*Mme. 2:* Très souvent.

*Mme. 1:* Une soupe. De l'hiver ?

*Mme. 2:* De septembre à Mars.

*Mme. 1:* C'est la saison. Et est-ce que ça vous arrive de faire des- des jus ou des smoothies comme ça ?

*Mme. 2:* Non, non.

*Mme. 1:* OK, [alors] là, je vais vous donner des fruits et légumes. Je vais vous demander de les regarder et de dire un peu comment vous les voyez et me dire ce que vous auriez fait avec. Est-ce que vous les aurez cuisinés ? Si oui, comment ? Ou est-ce que vous les auriez jeté ?

*Mme. 2:* Moi j'aurai bien fait une tomate farcie.

*Mme. 1:* Une tomate farcie avec cette tomate.

*Mme. 2:* En tout cas elle est belle mais-- Elle doit être assez mûre mais c'est une tomate.

*Mme. 1:* Ok, on peut la mettre en cuisson. Cette banane.

*Mme. 2:* Woauh [euh]

*M. 1:* Tu peux le faire [hein] Tu peux faire la même.

*Mme. 2:* [bah] même tu vois quand je te fais des bananes, j'enlève [euh]

*M. 1:* Tu enlève ça.

*Mme. 2:* J'enlève des parties [euh] marron là.

*Mme. 1:* Ouais.

*M. 1:* J'aurais fait- j'aurais fait aussi au four avec du citron, de.

*Mme. 2:* L'huile, ok.

*M. 1:* Citron, un.

*Mme. 2:* Petit peu de sucre et puis un tout petit peu de beurre pour redorer. D'accord, vous parlez, mais dans les gâteaux. C'est Orange.

*M. 1:* Là, on ne sait pas s'il y a que la moitié qui guettait ou si la.

*Mme. 2:* Face de l'autre côté. Ouais, si c'est de ce côté-là, qu'est-ce que vous faites ?

*M. 1:* J'enlève, j'enlève toute la partie. Je coupe la moitié pratiquement.

*Mme. 2:* Ouais, mais.

*M. 1:* Après je vois comment est l'autre partie.

*Mme. 2:* D'accord.

*M. 1:* Si, si elle est encore consommable, on peut faire un jus, hein ?

*Mme. 2:* Oui.

*M. 1:* Moi, le jus, ça présente l'avantage dès que, dès la première gorgée, on se rend compte si c'est bon, si c'est.

*Mme. 2:* Ouais, voilà.

*M. 1:* Mais non, je ne peux pas grand-chose autrement. Ou éventuellement, si on fait une salade de fruits. Mais je dis non. Je ne crois pas que je prendrai même le risque. Parce que si elle a du goût et va donner un mauvais goût.

*Mme. 2:* Toute la salade, oui. Donc ce serait plutôt vous la jetterez plutôt donne ouais. Est-ce que j'ai du goût sans agrumes ? Les vers de poireaux ici, il y en a quelques-uns qui sont un peu abîmés.

*M. 1:* Oui, je suis un peu. J'ai un peu flétrie quand même.

*Mme. 2:* Oui.

*M. 1:* Bon je récupère ce qui est encore vert. Je les mets dans un potage.

*Mme. 2:* Est dans l'action. La soupe. On peut mettre l'air. Si c'est bon, c'est une tomate cerise ou booster ?

*M. 1:* Je la mets à la poubelle. Ouais, elle est trop.

*Mme. 2:* Con, mais non.

*M. 1:* Une fois. Bling bling était aussi une. Je ne sais pas. Commencer en.

*Mme. 2:* Dessous ? Oui.

*M. 1:* Ça dépend. Je coupe, ça me coupe, ça déjà, je coupe ça. Donc on récupère à peu près la moitié d'une demi poire.

*Mme. 2:* Ce qui fait beaucoup.

*M. 1:* Puisque beaucoup moins la chair de ce côté-là, oui. Éventuellement, si elle l'est encore, ça semble correct. Je veux mettre dans une popote, mais elle ne popote à manger dans la journée, à part à garder jusqu'au lendemain.

*Mme. 2:* Ouais, voilà, je prendrais pas de risque. Non, encore.

*M. 1:* Pour moi, ça irait au composteur.

*Mme. 2:* Donc est ce qu'on l'aimez plutôt compost ou plutôt compost ?

*M. 1:* Plutôt compost, plutôt compost. Mais je sais pas, mais m'inspire pas trop. Ben ça, c'est quoi une salade ?

*Mme. 2:* Ouais.

*M. 1:* Qu'est-ce qu'elle a cette salade ? Il y un peu flétrie.

*Mme. 2:* Je ne sais pas comment.

*M. 1:* Elle a pu, mais je pense qu'il y a quelque chose.

*Mme. 2:* Ouais.

*M. 1:* Me le reste également.

*Mme. 2:* Vous, [Prénom M. 1], je coupe ? Non, nous ne me dégoûterait pas.

*M. 1:* Celles-là sont bonnes.

*Mme. 2:* Son but ? Quand je demande des sandwichs, je monte. Moi, je cuisine une façon.

*Mme. 1:* Assez détendue de l'eau claire.

*M. 1:* Alors j'ai cuisiné.

*Mme. 2:* Tout.

*M. 1:* Nouveau. Elles ont une drôle de couleur à l'intérieur.

*Mme. 2:* Non compost post compost.

*M. 1:* Pour ne pas de risques non plus. Avec les ça, c'est une courgette. C'est un fait, l'humanité.

*Mme. 2:* Commence à être.

*M. 1:* Je pense qu'elle est un peu elle un peu malade.

*Mme. 2:* Oui, un peu comme ça. Ramollir quand compost, compost.

*M. 1:* Le rapide. On va dire.

*Mme. 2:* On. Pour. Moi je couperais, je mangerais le reste.

*M. 1:* Ouai mais bon après c'est à voir aussi en fonction. Il n'y a pas le visuel de la photo.

*Mme. 1:* Qui couvre la partie.

*M. 1:* Qui n'est pas bonne. Non je pense, je mangerais le parti.

*Mme. 2:* En crudités.

*M. 1:* Et toi.

*Mme. 2:* Le.

*M. 1:* Citron. Non, ce couperet met vraiment en coupant l'un, en laissant un large, quand même un peu de marche. Et je l'utiliserai. Moralement, tout le monde peut s'en étonner. Les bananes au four ?

*Mme. 2:* Ouais. Voilà le tout en l'automne.

*M. 1:* Mais après, c'est difficile.

*Mme. 2:* Ben oui, ça dépend. On sait c'est.

*M. 1:* Quoi un bon coup ?

*Mme. 2:* C'est une carte blanche à la veille contre la grippe.

*M. 1:* J'ai pas envie d'être.

*Mme. 2:* Malade et c'est là.

*Mme. 1:* Nous avons un scoop. C'est beau ça! Il épluche.

*M. 1:* Comment savoir une drôle de couleur éventuellement dans la soupe ?

*Mme. 1:* On va faire beaucoup quoi ?

*M. 1:* C'est bien vrai, oui, mais en fait je pense que tu récupères pas.

*Mme. 2:* Beaucoup finalement.

*M. 1:* À couper les deux bouts là. Mais je pense que la peau, elle est elle fait un peu flétrie. Mais c'est bien parce qu'elle a été conservée dans un sachet.

*Mme. 1:* En plastique et c'est pas parce qu'elle est, comme.

*M. 1:* Je pense, je pense.

*Mme. 1:* Je pense, c'est pas parce qu'elle est comme ça qu'elle n'est pas bonne.

*M. 1:* Après, c'est toujours pareil. On se dit on va l'utiliser. Et puis quand on commence à couper, on.

*Mme. 1:* N'est pas.

*M. 1:* La même. Elle est bien gâtée.

*Mme. 2:* Après.

*M. 1:* On apprécie en fonction. Non, on pourrait l'utiliser en soupe. Mais sous réserve.

*Mme. 2:* Tentatives.

*Mme. 1:* Ouais ouais.

*M. 1:* Le dernier ?

*Mme. 2:* Ça c'est quoi un poivron ?

*Mme. 2:* C'est un poivron, ouais.

*M. 1:* Moi je trouve qu'un poivron, c'est bon quand c'est croquant. Là, je ne pense pas qu'il soit très croquant.

*Mme. 2:* Alors il n'a plus--.

*M. 1:* [inaudible]. Non, je vais être honnête.

*Mme. 2:* J'aime prendre en photo le menu. Hop!

*M. 1:* On en mettrait beaucoup. Finalement, le compost.

*Mme. 2:* Ce sont sont des fruits et légumes qui ont été jetés.

*M. 1:* Donc oui, on voit plus bon.

*Mme. 2:* Vous en avez fait quelques uns ou.

*M. 1:* Bien de se dire qu'on ne va pas. [Inaudible] ils étaient jetés ?

*Mme. 1:* Oui.

*M. 1:* C'est vrai.

*Mme. 2:* Oui, c'est.

*M. 1:* Même la tomate, c'est.

**Household F46, Interview 1**

00:00:00
*Intervieweuse:* Je lance l'entretien. Alors du coup, dans un premier temps-

00:00:04
*Homme interviewé:* Ça t'embête si je [inaudible 00:00:05] ?

00:00:04
*Intervieweuse:* Oui, c'est comme vous voulez.

00:00:04
*Femme interviewée:* Oui, mais c'est bien qu'il y a de l'air aussi.

00:00:04
*Homme interviewé:* Oui, mais il y a de l'air là-bas.

00:00:10
*Intervieweuse:* Le temps qu'il passe, on peut fermer. Alors là, on va commencer par vous. Je vous propose un petit jeu de devinettes, c'est-à-dire, je vais vous demander de penser à un légume ou à un fruit que vous aimez tout particulièrement. Et vous allez penser à ce légume ou à ce fruit sans me révéler son identité. L'idée c'est que je devine uniquement grâce à votre description. Est-ce que vous avez un légume ou un fruit en tête ?

00:00:36
*Femme interviewée:* Oui.

00:00:37
*Intervieweuse:* Alors je vous écoute. Dites-moi tout ce qui vous vient par la tête sur ce légume.

00:00:41
*Femme interviewée:* Sans vous dire ce que c'est ?

00:00:42
*Intervieweuse:* Vous pouvez me dire si c'est un fruit ou un légume, si ça vous aide à décrire. Mais pas me dire son identité réelle.

00:00:51
*Femme interviewée:* Alors c'est un fruit botaniquement parlant. Mais c'est un légume gustativement parlant.

00:00:55
*Intervieweuse:* Ok.

00:00:57
*Femme interviewée:* C'est un fruit théoriquement de saison, c'est-à-dire qu'on trouve de préférence en été, mais qu'on trouve par ici toute l'année.

00:01:05
*Intervieweuse:* Ok.

00:01:05
*Femme interviewée:* [euh] Vous voyez déjà de quoi il s'agit ?

00:01:10
*Intervieweuse:* Oui, mais je vais pousser la question quand même. [rires]

00:01:13
*Femme interviewée:* Je continue ?

00:01:14
*Intervieweuse:* Oui.

00:01:15
*Femme interviewée:* Donc, c'est un légume, enfin qu'on achète en tant que légume, qu'on consomme plut- quand même plus quand c'est moins cher, c'est à dire depuis un mois, c'est à peu près, c'est entre de moins un euro, deux euros le kilo maximum. Et qu'on consomme principalement cuit.

00:01:38
*Intervieweuse:* D'accord.

00:01:39
*Femme interviewée:* Mais on le consomme aussi cru. Et bon par ici, on trouve essentiellement qu'une variété de ce fruit et légume. Mais je dois dire que quand on va chez notre fille, en région toulousaine, on trouve de ce légume-fruit avec des variétés différentes et poussées en pleine terre et en plein soleil et que le goût est quand même nettement différent. Que pourrais-je vous dire de plus ?

00:02:10
*Intervieweuse:* Qu'est-ce que vous aimez particulièrement dans ce légume qui est un fruit ? [rire]

00:02:15
*Femme interviewée:* [euh] Qu'il se prête déjà à être mangé soit cru, soit cuit.

00:02:24
*Intervieweuse:* Oui.

00:02:25
*Femme interviewée:* Sa conservation est assez, enfin n'est pas trop problématique, je dirais. Enfin pour en tout cas les variétés qu'on trouve ici.

00:02:36
*Intervieweuse:* Oui.

00:02:39
*Femme interviewée:* C'est, cuit, alors c'est notre goût qui fait ça sans doute, nous, on aime particulièrement ça cuit. On cuit au four avec de l'ail, du thym, un peu de chapelure, un peu d'huile d'olive-

00:02:56
*Homme interviewé:* Un qualificatif qui me vient à l'esprit, savoureux, non ?

00:03:00
*Femme interviewée:* Oui-

00:03:00
*Homme interviewé:* [rire]

00:03:00
*Femme interviewée:* Ben on aime ça, c'est ce qui chatouille le mieux les papilles.

00:03:05
*Intervieweuse:* [rire]

00:03:06
*Femme interviewée:* Après, quand c'est cru, bon suivant si c'est un ingrédient majoritaire ou accessoire, ça fait aussi, ça donne de la couleur, ça, ça... Ça se prête à être mélangé avec d'autres ingrédients, comme des pâtes cuites froide, par exemple. Du fromage, etc. Bon par ici, peut-être ailleurs, je sais pas trop, on commence à trouver depuis deux, trois ans des variétés dites anciennes. Alors je ne sais pas si elles sont anciennes ou si elles sont nouvellement-

00:03:44
*Homme interviewé:* Nouvellement créées [rires]

00:03:46
*Femme interviewée:* Nouvellement créées, mais ça permet de varier les textures et les goûts aussi.

00:03:49
*Intervieweuse:* Oui.

00:03:51
*Femme interviewée:* Donc il y a la variété verte qu'on consomme crue. Voilà [rires] que vous dire de plus ?

00:04:03
*Intervieweuse:* Ok. En général, vous le mangez plutôt quand, plutôt le matin, le midi, l'après-midi ?

00:04:08
*Femme interviewée:* Au repas, midi et soir, midi ou soir, midi et soir.

00:04:11
*Intervieweuse:* D'accord, aussi bien cru que cuit. [euh]Alors, est-ce que vous pouvez me raconter les différentes étapes, s'il y en a, avant que vous puissiez consommer. Vous avez votre fruit-légume devant vous, qu'est-ce que vous faites avant de pouvoir le mettre dans votre bouche ?

00:04:32
*Femme interviewée:* Alors quand c'est cru, je le coupe une demi-heure avant le repas, je sale un petit peu de façon à, au moment du repas, d'égouter pour que ce fruit-légume crache une partie de son eau en trop. Ce qui fait qu'après, c'est plus agréable de le manger. Quand c'est cuit, alors, j'ai changé de procédé. Avant je retirais les pépins.

00:05:02
*Intervieweuse:* D'accord.

00:05:05
*Femme interviewée:* Et je, j'en faisais des demi fruits et légumes, donc sans, en retirant une partie des pépins, quasiment tous les pépins et en mettant à la place ail, fines herbes, etc. Et maintenant, pour aller plus vite, parce que finalement, c'est aussi bon, je coupe en tranches.

00:05:22
*Intervieweuse:* D'accord.

00:05:23
*Femme interviewée:* Et puis assez rapidement, enfin, de façon assez grossière, je dirais, je retire une partie des pépins comme ça à la main.

00:05:32
*Intervieweuse:* D'accord.

00:05:33
*Femme interviewée:* Et puis je les mets dans un plat, toujours pareil avec ail, fines herbes, un peu de chapelure, un peu d'huile d'olive, voilà. Et je les lave quand même préalablement à toutes ces préparations-là.

00:05:48
*Intervieweuse:* D'accord, vous ne l'épluchez pas, vous retirez les pépins et vous lavez-

00:05:52
*Femme interviewée:* Je lave d'abord.

00:05:54
*Intervieweuse:* Et après vous coupez et vous enlevez les pépins quand c'est en tranches.

00:05:56
*Femme interviewée:* C'est un fruit-légume qui est quand même très riche en eau.

00:06:01
*Intervieweuse:* Oui.

00:06:02
*Femme interviewée:* Et quand c'est, enfin comme tous les fruits et légumes d'ailleurs, mais que ce soit cru ou cuit, quand on retire une partie des pépins, la préparation est moins aqueuse.

00:06:11
*Intervieweuse:* D'accord. En général, vous l'achetez où, souvent ?

00:06:19
*Femme interviewée:* Alors, soit en supermarché, soit, enfin on fréquente nous deux magasins qui vendent des fruits et légumes, supermarché, au Super U à Keredern. Et le marchand de fruits et légumes qui fait aussi charcuteries, fromages. Anciennement Mauricette Coulomb. Alors je ne sais plus comment ça s'appelle maintenant, qui est place Napoléon III.

00:06:35
*Homme interviewé:* Tercoec.

00:06:40
*Femme interviewée:* Tertoec.

00:06:40
*Homme interviewé:* Tercoec

00:06:40
*Femme interviewée:* Tercoec.

00:06:40
*Homme interviewé:* T-E-R-C-O-E-C.

00:06:42
*Femme interviewée:* Ah bon. Qui est Place Napoléon III entre la boucherie Bœuf Charolais et le restaurant à côté. Alors tout dépend, en général on fait les deux, ça va dépendre du prix, ça va dépendre de l'aspect.

00:06:57
*Intervieweuse:* D'accord.

00:06:57
*Femme interviewée:* Ah si, c'est chez, donc anciennement Mauricette Coulomb, il y a quand même des fruits dits défor- difformes.

00:07:04
*Intervieweuse:* D'accord.

00:07:05
*Femme interviewée:* Qui sont, enfin ces légumes difformes qui sont quand même moins chers et puis se prêtent tout à fait aux mêmes préparations.

00:07:15
*Intervieweuse:* Ok. Et du coup, alors vous m'avez dit, du coup il y a le goût savoureux, qu'il se conserve assez simplement.

00:07:23
*Femme interviewée:* Enfin il ne faut pas non plus-

00:07:25
*Homme interviewé:* Il ne faut pas non plus exagérer non plus [rires].

00:07:30
*Intervieweuse:* Il s'accommode facilement, parce qu'il peut être cru, cuit, il se mélange facilement avec d'autres ingrédients. Qu'est-ce que vous avez dit d'autre comme avantages ? Vous, voilà, ce que vous m'avez dit. Est-ce que vous lui trouvez d'autres, d'autres avantages, d'autres apports ou c'est exclusivement ce côté facile à préparer et bon à la fois ?

00:07:52
*Femme interviewée:* Alors ce n'est pas forcément le plus facile à préparer, parce que le temps de cuisson est quand même assez long.

00:07:55
*Intervieweuse:* D'accord.

00:07:56
*Femme interviewée:* Bon, nous ça ne nous pose pas de problème ici de le faire. Enfin même si ça prend, ça prend trois quarts d'heure de cuisson au four quand même, donc il faut penser à le faire.

00:08:06
*Intervieweuse:* Oui.

00:08:08
*Femme interviewée:* Une fois que c'est quoi, ça se conserve très bien au réfrigérateur aussi d'ailleurs. Souvent moi j'en cuits de plat à four en même temps, et puis on met une partie au réfrigérateur pour les jours suivants.

00:08:19
*Intervieweuse:* D'accord.

00:08:20
*Femme interviewée:* Qu'est-ce que vous m'avez demandé ? Qu'est-ce que...

00:08:23
*Intervieweuse:* Quels avantages vous lui trouvez ? Qu'est-ce qu'il vous apporte, quand vous le consommez, comment vous-

00:08:30
*Femme interviewée:* Ben j'imagine que ça fait un légume-fruit avec un apport en fibres intéressant, un apport calorique pas trop élevé, si je ne mets pas trop d'huile d'olive au moins.

00:08:38
*Intervieweuse:* [rire]

00:08:42
*Femme interviewée:* Et puis quand c'est cru, en fait, on peut très bien se passer, même si on met un petit peu d'huile de colza pour-

00:08:46
*Homme interviewé:* [inaudible 00:08:47]

00:08:47
*Femme interviewée:* Dans l'idée que c'est bien, on peut ne pas en mettre, on peut ne pas mettre d'huile.

00:08:54
*Homme interviewé:* À la limite nature.

00:08:55
*Femme interviewée:* Oui, nature, un peu de vinaigre balsamique, enfin bon. Voilà donc...

00:09:04
*Intervieweuse:* Et du coup, est-ce que vous lui trouvez à l'inverse certains inconvénients ou pas vraiment ?

00:09:10
*Femme interviewée:* En tant que tel ?

00:09:15
*Intervieweuse:* Il peut être parfait.

00:09:19
*Femme interviewée:* Oui, à l'exception alors de ces nouveaux fruits-légumes de couleurs différentes, en particulier le vert, qui est un peu difficile à choisir. Parce qu'en fait, on découvre parfois que ce légume-fruit est, malgré son bel aspect, pas mûr du tout.

00:09:40
*Intervieweuse:* D'accord.

00:09:42
*Femme interviewée:* C'est arrivé plusieurs fois, donc... Enfin ce n'est pas grave.

00:09:49
*Homme interviewé:* C'est la plus grosse perte qu'on ait eu.

00:09:52
*Intervieweuse:* Ah oui ?

00:09:55
*Homme interviewé:* Si on compare par rapport à il y a une grosse dizaine d'années, où il y a eu tout un passage où est elles étaient complètement insipides.

00:10:02
*Intervieweuse:* D'accord.

00:10:03
*Homme interviewé:* Farineuses. C'est plus rarement le cas là maintenant, c'est vraiment redevenu-

00:10:08
*Femme interviewée:* Ben disons qu'ici ce légume-fruit qu'on trouve par ici toute l'année, reste quand même, même si le prix est plus élevé en hiver, reste quand même à notre avis, à notre goût savoureux.

00:10:19
*Homme interviewé:* Il se ressemble.

00:10:21
*Femme interviewée:* On voit la différence quand on va chez une de nos filles en région toulousaine et chez chez l'autre à Lyon où ce légume-fruits ne se trouve pas sur les étals des supermarchés hors saison.

00:10:37
*Intervieweuse:* Oui. Ok, alors c'est le moment fatidique. Je dois deviner et je dirais : la tomate.

00:10:47
*Femme interviewée:* Oui, bien sûr. [rires]

00:10:54
*Intervieweuse:* D'accord, alors maintenant, il s'agit de discuter de votre organisation en terme d'achats alimentaires. Au sein de votre foyer, qui est amené à faire les courses alimentaires ? Les deux, une fois sur deux, comment vous-

00:11:09
*Femme interviewée:* Non, c'est-

00:11:09
*Homme interviewé:* C'est principalement toi.

00:11:11
*Femme interviewée:* Oui, parfois tu m'accompagnes, mais c'est surtout à but de pousser le caddie et porter les sacs. Ça fait la sortie.

00:11:17
*Homme interviewé:* Je fais les opérations annexes, mais c'est tout. Enfin-

00:11:21
*Femme interviewée:* Si, parfois, je peux te dire-

00:11:22
*Homme interviewé:* Oui, mais bon.

00:11:23
*Femme interviewée:* "Prends un kilo de ceci ou trois courgettes ou deux aubergines."

00:11:29
*Intervieweuse:* C'est plutôt vous qui gérez l'aspect-

00:11:33
*Homme interviewé:* Oui.

00:11:33
*Intervieweuse:* L'aspect alimentaire des courses.

00:11:33
*Femme interviewée:* Oui.

00:11:35
*Intervieweuse:* Ok, alors est-ce que vous pouvez me raconter votre, votre organisation, comment vous organisez vos courses ?

00:11:40
*Femme interviewée:* Sur les fruits et légumes ?

00:11:42
*Intervieweuse:* Essentiellement, après ça peut, forcément ça peut être influencé par le reste, mais voilà comment vous organisez vos achats ?

00:11:48
*Femme interviewée:* Alors, de façon générale, on fait les cou-, enfin ou je, ou on-

00:11:52
*Homme interviewé:* C'est souvent le cas.

00:11:56
*Femme interviewée:* C'est souvent le cas, on en achète quand même assez souvent. C'est-à-dire, on ne fait pas des stocks pour une semaine.

00:12:01
*Intervieweuse:* D'accord.

00:12:01
*Femme interviewée:* Donc souvent, on en prend le samedi, par exemple.

00:12:08
*Intervieweuse:* D'accord.

00:12:10
*Femme interviewée:* Puis après le mardi, je retourne en chercher.

00:12:13
*Intervieweuse:* Ok.

00:12:13
*Femme interviewée:* Et parfois le jeudi, ça arrive de devoir faire un complément. Enfin je dis jeudi, ça peut être mercredi, enfin après ça dépend, c'est pas fixé comme ça, mais. Parce qu'il y a beaucoup, enfin il y a quand même de nombreux fruits et légumes qui se conservent mal au réfrigérateur, enfin qui perdent du goût au réfrigérateur.

00:12:31
*Intervieweuse:* Oui.

00:12:35
*Femme interviewée:* On n'a pas des moyens de stockage non plus infinis. Donc comme on a le temps et l'occasion d'aller faire les courses plus souvent, on le fait.

00:12:42
*Homme interviewé:* Oui, la proximité aussi.

00:12:45
*Femme interviewée:* Et la proximité oui, voilà, c'est pas loin. Place Napoléon III et le Super U de Keredern. On a vu pendant le premier confinement, par exemple, là où on se forçait à faire des courses le moins souvent possible, que c'était quand même, on avait plus de pertes en fruits et légumes que d'habitude.

00:13:02
*Intervieweuse:* Oui.

00:13:03
*Femme interviewée:* Parce que prendre des fruits et légumes pour une semaine de consommation, c'est, enfin moi je trouve ça difficile à gérer. Alors après, qu'est-ce-, vous m'avez posé la question de l'approvisionnement ?

00:13:17
*Intervieweuse:* Oui, voilà comment vous organisez tout ça. Donc vous y allez, sur les fruits et légumes, vous y allez presque tout de même tous les trois ou quatre jours, voire-

00:13:25
*Femme interviewée:* À peu près, oui.

00:13:25
*Intervieweuse:* -plus si besoin. Et vous allez donc essentiellement au supermarché ou en marchant Tercoec. [euh] Vous y allez à pied ? C'est à côté de chez vous ?

00:13:38
*Femme interviewée:* Non, on y va en voiture parce que je ne peux pas, enfin je ne peux pas, je n'aime pas porter des sacs ou des sacs à dos. Donc c'est vrai que c'est très accessible en voiture, mais- À pied je vais dire, mais pour des raisons de portage après, non. C'est en voiture.

00:13:53
*Intervieweuse:* Et c'est, tous vos achats se font, toutes vos courses alimentaires se font là-bas ? Ou vous avez des fois, enfin là c'est les fruits et légumes et vous allez autre part pour-

00:14:02
*Femme interviewée:* Actuellement non.

00:14:03
*Intervieweuse:* Vous allez essentiellement-

00:14:04
*Femme interviewée:* À une époque, on y allait, on allait au marché de Kerinou.

00:14:08
*Homme interviewé:* On allait à la Biocoop aussi plus.

00:14:10
*Femme interviewée:* Alors la Biocoop, on n'a jamais vraiment acheté de fruits et légumes parce que c'est hyper cher.

00:14:14
*Intervieweuse:* Oui.

00:14:17
*Femme interviewée:* Le marché de Kerinou, on l'a fait un petit peu, mais moi, j'aime pas trop l'ambiance marché finalement.

00:14:25
*Intervieweuse:* D'accord.

00:14:28
*Femme interviewée:* Donc act-, enfin depuis déjà au moins et même avant, et depuis deux ans, c'est entre Super U et Tercoec.

00:14:36
*Intervieweuse:* Ok.

00:14:39
*Femme interviewée:* Alors la différence, parce que j'aime pas Super U parfois, c'est pour ça que, qu'on va souvent à Tercoec avant Super U, c'est qu'à Super U ils sortent, enfin visiblement les fruits et les légumes, de chambres réfrigérées.

00:14:50
*Intervieweuse:* D'accord.

00:14:54
*Femme interviewée:* Donc on n'a pas vraiment, on ne sait pas si le-

00:15:00
*Homme interviewé:* C'est difficile de-

00:15:01
*Femme interviewée:* Si c'est mûr.

00:15:01
*Intervieweuse:* Oui.

00:15:02
*Femme interviewée:* Alors qu'à Tercoec, bon, c'est en étal, en étal, je pense pas qu'ils aient de chambre réfrigérée, je pense pas.

00:15:10
*Intervieweuse:* C'est à température ambiante dans le magasin...

00:15:15
*Femme interviewée:* Alors c'est vrai que, par contre, il ne faut pas y aller le lundi à Tercoec, parce qu'ils se réapprovisionnent le mardi.

00:15:20
*Intervieweuse:* D'accord.

00:15:21
*Femme interviewée:* Donc je ne vais pas le lundi, puisque je sais que ce sont les fruits de, et légumes de la semaine précédente. Mais à partir du mardi, ils ont les nouveaux approvisionnements. À Super U, c'est pareil, on évite le lundi parce qu'ils sont livrés le mardi, pour tout d'ailleurs. Donc quand on va le lundi, on a que les restes du week-end.

00:15:40
*Intervieweuse:* Du coup vous y allez tous les mardis ?

00:15:44
*Femme interviewée:* Pas forcément, ça dépend, là je sais pas aujourd'hui, par exemple, est-ce qu'on va y aller ? Non.

00:15:47
*Intervieweuse:* D'accord.

00:15:48
*Femme interviewée:* Ah, peut-être pour des fruits, je ne sais plus. Non, on a du melon encore.

00:15:53
*Homme interviewé:* On a encore.

00:15:53
*Femme interviewée:* Alors après, quand on revient, non c'est l'approvisionnement.

00:16:01
*Intervieweuse:* Oui, après vous pouvez toujours continuer la-

00:16:05
*Femme interviewée:* Mais après, quand on vient avec des, quand on est que tous les deux ans, c'est assez facile de gérer je dirais.

00:16:13
*Intervieweuse:* Oui.

00:16:13
*Femme interviewée:* Parce que soit ça se conserve à température ambiante tout en surveillant, et soit, si je vois que ça va pas, les fruits et les lé- enfin les légumes essentiellement qui se mangent cuits, je les cuisine donc, enfin rapidement, quitte à les conserver au réfrigérateur après. Plutôt que d'attendre qu'ils ne soient plus bons et puis...

00:16:37
*Intervieweuse:* D'accord.

00:16:37
*Femme interviewée:* Bon je sais qu'il y a des légumes, par exemple, comme les courgettes qui vont rester plus de temps à température ambiante sans s'abimer, bon les aubergines on les met au réfrigérateur parce que je sais par expérience que ça...

00:16:52
*Homme interviewé:* Ça ne dure pas longtemps.

00:16:53
*Femme interviewée:* Que ça peut ne pas durer longtemps et surtout, on ne voit pas ce qui se passe sous la peau. Donc au réfrigérateur, c'est mieux. Après, qu'est-ce qu'on mange comme légumes ? Souvent, quand même je cuisine assez rapidement.

00:17:07
*Intervieweuse:* D'accord.

00:17:09
*Femme interviewée:* Après. Si on achète le samedi, par exemple, le dimanche soir, ce sera tout cuisiné, quitte à ce qu'on consomme-

00:17:16
*Intervieweuse:* D'accord.

00:17:17
*Femme interviewée:* -après, sur lundi, mardi, mercredi.

00:17:22
*Intervieweuse:* D'accord. Ça vous fait une consommation après si vous cuisinez par exemple tout le dimanche, enfin un plat, trois jours après, vous pouvez toujours le consommer ?

00:17:33
*Femme interviewée:* Oui. Là actuellement par exemple au réfrigérateur, on a un petit reste de courgettes cuisinées de dimanche. On est mardi, donc ça ira. Des tomates cuites dimanche.

00:17:43
*Intervieweuse:* D'accord.

00:17:44
*Femme interviewée:* Et on n'avait pas besoin d'en manger dimanche, mais je préférais les, cuire parce que je voyais que sinon, je voyais que ça n'allait pas tenir. Et qu'est-ce qu'on a comme légumes actuellement au réfrigérateur ? Je crois que c'est tout, on a un petit reste de tomates cerises à consommer ce soir, un petit reste de concombre.

00:17:59
*Homme interviewé:* Après ça dépend, c'est...

00:18:02
*Femme interviewée:* Ça dépend oui, de...

00:18:03
*Homme interviewé:* Un couscous par exemple...

00:18:07
*Femme interviewée:* Un couscous, on le met, moi je le fais rapidement aussi.

00:18:07
*Homme interviewé:* Oui oui, mais-

00:18:07
*Femme interviewée:* Après c'est un peu plus compliqué quand la famille est là, parce que évidemment il en faut plus.

00:18:14
*Intervieweuse:* Oui.

00:18:14
*Femme interviewée:* Donc [euh], enfin bon, c'est pas si souvent que ça. Donc là c'est pareil, soit je cuisine à l'avance et on sait qu'on va consommer dans les deux ou trois jours, soit je mets au congélateur.

00:18:28
*Intervieweuse:* D'accord.

00:18:31
*Femme interviewée:* Je cuisine et je mets au congélateur.

00:18:37
*Intervieweuse:* Quand ce, quand ça va être au-delà des deux, trois jours, vous congelez.

00:18:40
*Homme interviewé:* Oui.

00:18:40
*Femme interviewée:* Quitte à ressortir le lendemain d'ailleurs.

00:18:42
*Intervieweuse:* Ok. Et donc quand vous allez aux courses, si vous allez donc au supermarché ou à Tercoec, est-ce que vous savez ce que vous allez acheter avant d'y aller ou pas vraiment ?

00:18:57
*Homme interviewé:* Ça dépend.

00:19:00
*Femme interviewée:* Suivant la saison, on sait à peu près ce qu'on va trouver. En fin de compte, ça peut arriver effectivement qu'on trouve pas.

00:19:04
*Intervieweuse:* Oui.

00:19:05
*Femme interviewée:* Ou on peut décider, je ne sais pas, je pense à des bettes par exemple, on s'est dit : "On va en prendre." Et puis, quand on les voit, on se dit non.

00:19:11
*Homme interviewé:* Bof. [rire]

00:19:12
*Intervieweuse:* D'accord.

00:19:16
*Femme interviewée:* C'est...

00:19:16
*Homme interviewé:* Ça dépend des arrivages quoi.

00:19:19
*Femme interviewée:* Oui, après il y a des choses qu'on sait qu'on va trouver tout le temps, la plupart, enfin suivant la saison, on sait à peu près ce qu'on va trouver.

00:19:31
*Intervieweuse:* Oui

00:19:32
*Femme interviewée:* Oui, j'ai une idée dans la tête, dans la tête pour savoir ce qu'on va consommer. Maintenant, je peux changer d'avis aussi en fonction de ce qui est proposé dans le supermarché ou le primeurs.

00:19:45
*Intervieweuse:* Vous n'avez pas forcément de liste très précise. Vous savez à peu près ce que vous aimeriez trouver.

00:19:50
*Femme interviewée:* Oui.

00:19:51
*Intervieweuse:* Mais vous n'avez pas une liste avec vous qui-

00:19:54
*Femme interviewée:* Si.

00:19:54
*Intervieweuse:* Si ?

00:19:55
*Femme interviewée:* Si, on a une liste, enfin sur téléphone, mais bon c'est pareil.

00:19:57
*Homme interviewé:* Oui, enfin c'est un pense-bête.

00:19:59
*Femme interviewée:* Oui.

00:20:00
*Homme interviewé:* Qui peut être, on peut déroger.

00:20:05
*Intervieweuse:* D'accord. Donc dessus c'est écrit, c'est juste écrit par exemple "fruits et légumes" ou c'est quand même écrit, détaillé ?

00:20:11
*Femme interviewée:* Non, non, c'est détaillé.

00:20:12
*Homme interviewé:* [inaudible 00:20:11]

00:20:14
*Femme interviewée:* Mais pas forcément, par exemple, si je mets "légumes couscous", ben je ne vais pas détailler tout ce qu'il faut pour un couscous.

00:20:19
*Intervieweuse:* Oui.

00:20:21
*Femme interviewée:* On met rarement les fruits et légumes tout compte fait.

00:20:24
*Homme interviewé:* On quoi ?

00:20:24
*Femme interviewée:* Non, sur le pense-bête on ne met pas les fruits et légumes.

00:20:27
*Homme interviewé:* Non, en fait, non.

00:20:28
*Femme interviewée:* On met le reste, on met ce qu'on est censé oublier, ce qu'on pourrait oublier en faisant les courses. Donc en fait-

00:20:34
*Homme interviewé:* C'est vrai que les fruits et légumes, non.

00:20:35
*Femme interviewée:* Fruits et légumes, détails, tout ça.

00:20:39
*Homme interviewé:* C'est au feeling.

00:20:40
*Femme interviewée:* On ne met pas.

00:20:40
*Homme interviewé:* C'est au feeling, au visu en arrivant devant l'étal.

00:20:40
*Intervieweuse:* On ne met pas les fruits et légumes.

00:20:43
*Intervieweuse:* Donc c'est, vous savez que vous allez passer par l'étal fruits et légumes, puis vous voyez sur le moment, ou des fois vous pouvez quand même avoir une envie de recette et notez qu'il faut acheter les fruits et légumes qui correspondent.

00:20:52
*Homme interviewé:* Pour moi tout seul, il vaudrait mieux quand même.

00:20:54
*Femme interviewée:* Pardon ?

00:20:54
*Homme interviewé:* Pour moi, tout seul, il vaudrait mieux mettre sur le pense-bête. [rire] Mais oui c'est vrai, c'est un peu implicite quoi.

00:21:04
*Femme interviewée:* Oui, ben...

00:21:06
*Homme interviewé:* Que ce soit des poires, des pommes.

00:21:09
*Femme interviewée:* On voit quoi, on regarde, c'est...

00:21:14
*Intervieweuse:* Et comment vous gérez les quantités du coup ? Que, quand vous êtes face aux fruits et légumes, comment vous, voilà comment vous gérez le nombre de chaque produit que vous allez acheter, comment vous ?

00:21:25
*Femme interviewée:* Suivant à quoi ces légumes, c'est essentiellement, sont destinés, je peux en prendre plus si je sais que je peux les cuisiner et les conserver.

00:21:35
*Intervieweuse:* D'accord.

00:21:38
*Intervieweuse:* Qu'est-ce qu'il y aurait comme idée ? Alors les fruits, c'est un peu plus compliqué, surtout en ce moment avec les températures qu'il fait, la conservation est plus, est pas facile. Donc c'est vrai qu'on s'est retrouvé par exemple à acheter des poires l'autre jour et à devoir les consommer rapidement, parce qu'on voyait qu'elle mûrissaient trop vite, donc, on s'est dit qu'on allait devoir les jeter.

00:22:03
*Homme interviewé:* Avant qu'il fasse chaud, on a fait une petite crise fraise.

00:22:07
*Femme interviewée:* Oui, enfin on sait que la saison des fraises, ça c'était à consommer très rapidement.

00:22:09
*Intervieweuse:* Premier fruit un peu d'été.

00:22:13
*Femme interviewée:* Les quantités vous dites.

00:22:15
*Intervieweuse:* Oui, voilà, c'est, après c'est, après des fois il n'y a pas vraiment de stratégie. C'est, des fois c'est- Mais oui vous, comment vous, comment vous gérez, là vous achetez des poires justement, vous en achetez combien ? Comment vous faites ?

00:22:28
*Femme interviewée:* Si on achète les poires alors-

00:22:32
*Homme interviewé:* On va tabler qu'elles sont bonnes.

00:22:32
*Femme interviewée:* C'est toi surtout qui aime ça, donc on va-

00:22:35
*Homme interviewé:* Au moins une par jour.

00:22:37
*Femme interviewée:* On va en acheter six par exemple.

00:22:38
*Homme interviewé:* Maximum.

00:22:41
*Femme interviewée:* Et parfois on se retrouve à les manger en trois jours parce qu'on voit qu'elles mûrissent trop vite. Alors les pommes, alors c'est plus vraiment de saison, parce que maintenant, on trouve que les pommes qui ont été conservées, mais quand c'est la saison des pommes, ben là on peut en acheter plus. Enfin non, je vais rarement au-delà de sept, huit.

00:22:58
*Homme interviewé:* Maximum. C'est moins problématique la conservation des pommes.

00:23:04
*Femme interviewée:* Alors les tomates en ce moment, ben on peut en acheter deux kilos d'un coup, par exemple.

00:23:07
*Intervieweuse:* D'accord.

00:23:12
*Homme interviewé:* Voire plus si on prend deux variétés.

00:23:15
*Femme interviewée:* Dans ce cas-là, on prend des tomates de serre, des tomates grappes deux kilos. Et puis, si on prend des tomates pour manger crues, on en prend moins quand même.

00:23:23
*Intervieweuse:* D'accord.

00:23:23
*Femme interviewée:* Pour manger crues, on va en prendre pour deux repas maximum, c'est-à-dire, je ne sais pas, je ne pèse pas, mais peut-être deux tomates par personne si c'est des tomates vertes. Donc je sais pas, ça va, huit tomates vertes, non, je prends pas huit tomates vertes. Six tomates vertes je dirais. Ça dépend de la taille.

00:23:46
*Homme interviewé:* Ça dépend de la taille.

00:23:47
*Intervieweuse:* Oui.

00:23:49
*Femme interviewée:* Si c'est des bettes, de toute façon, c'est en botte. Donc après là, je cuisine et on en mange jusqu'à ce qu'il y en ait plus. Si c'est, j'ai pas de stratégie non au niveau des quantités, c'est plus complique de faire- Là par exemple, il y a des pêches plates que moi, j'aime bien. Mais là, c'est compliqué parce que souvent, on les trouve mûres. Et encore, non. Les dernières qu'on a achetées chez, chez Tertoec, elles étaient pas mûres. Donc je suis en train de mettre au les frigo, puis dehors, puis au frigo, pour voir si elles mûrissent sans pourrir.

00:24:26
*Intervieweuse:* Oui.

00:24:32
*Femme interviewée:* En fruit en ce moment, je trouve qu'il y a pas grand chose quand on, est encore, quand il n'y a pas encore les fruits d'été pas chers. Y a plus les fruits d'hiver. Et là-

00:24:40
*Homme interviewé:* On se rabat sur l'ananas. Puis là il y a le melon quand même.

00:24:45
*Femme interviewée:* Oui, les melons commencent, donc ça en ce moment, on mange, on prend du melon. Bon l'ananas c'est un fruit qu'on mange souvent, parce qu'on en trouve tout le temps, c'est jamais cher. C'est pareil, ça peut être, si on voit qu'il commence à être trop mûr, on le coupe et on le met au frigo, il se conserve.

00:25:01
*Intervieweuse:* Oui.

00:25:01
*Femme interviewée:* Le fruit qu'on ne mange plus jamais, c'est la cerise parce que c'est hyper cher.

00:25:08
*Intervieweuse:* D'accord.

00:25:13
*Femme interviewée:* Après bon vont venir maintenant les nectarines, les pêches plates vont être moins chères. Alors les abricots, on en a mangé, mais surtout pour faire des tartes, parce que ils étaient pas top à manger comme ça. La rhubarbe, on en a mangé. Je ne sais même pas combien de temps ça dure, on verra quand il n'y en aura plus. [rire] Les prunes, on en mange pas tellement.

00:25:33
*Homme interviewé:* C'est vraiment très rare les prunes.

00:25:42
*Femme interviewée:* Elles sont jamais terribles, soit elles sont farineuses, soit elles sont-

00:25:42
*Homme interviewé:* Souvent décevant.

00:25:42
*Intervieweuse:* D'accord.

00:25:45
*Femme interviewée:* Les bananes, pour nous, on n'en achète jamais. On en achète parfois-

00:25:49
*Homme interviewé:* C'est vraiment une exception.

00:25:50
*Femme interviewée:* -quand les enfants sont là.

00:25:52
*Intervieweuse:* Oui.

00:25:56
*Femme interviewée:* Après en légumes, en légumes, ben alors les aubergines, par exemple, curieusement, ça se trouve toute l'année pas cher.

00:26:03
*Intervieweuse:* Oui, d'accord.

00:26:08
*Femme interviewée:* Je ne sais pas comment ça se fait. Ben si, elle viennent d'Espagne ou du Maroc sans doute. Mais ça, on en mange toute l'année.

00:26:12
*Homme interviewé:* Courgettes.

00:26:13
*Femme interviewée:* Des courgettes.

00:26:13
*Homme interviewé:* Oui.

00:26:14
*Femme interviewée:* Des tomates, on en mange toute l'année. Alors, les carottes, on en mange pas tellement comme ça. On en achète peu, en couscous, à part ça, on en achète peu. Quand il y a un plat cuisiné, genre bœuf aux carottes évidemment. Carottes crues, râpées, on aime pas trop. Qu'est-ce qu'il y a dans légumes et fruits ?

00:26:41
*Homme interviewé:* Les choux, genre chou doux, etc.

00:26:45
*Femme interviewée:* Ah oui, les choux doux, qu'on trouve qu'à Super U d'ailleurs. C'est un chou, je sais pas si vous voyez ce que c'est, c'est un chou-

00:26:50
*Homme interviewé:* Très compact.

00:26:50
*Femme interviewée:* Très compact. Et qui se cuit très vite, contrairement au chou.

00:26:53
*Intervieweuse:* Il est quelle couleur ?

00:26:53
*Femme interviewée:* Il est vert clair.

00:27:00
*Homme interviewé:* Vert clair.

00:27:00
*Intervieweuse:* D'accord.

00:27:00
*Homme interviewé:* Vert jaune.

00:27:00
*Femme interviewée:* Chou doux qu'il s'appelle. C'est une variété ancienne qui a été remise, qui a été recultivée apparemment.

00:27:05
*Homme interviewé:* C'est une boule aplatie.

00:27:08
*Intervieweuse:* D'accord, j'ai acheté un chou l'autre jour que je ne connaissais pas non plus. Que je n'ai pas encore cuisiné. Mais c'est un rond vert, comme un navet presque.

00:27:08
*Femme interviewée:* Oui. Alors le chou doux, il est sous cellophane tout le temps, je ne sais pas pourquoi.

00:27:18
*Homme interviewé:* C'est un feuillu, enfin je veux dire-

00:27:24
*Femme interviewée:* C'est un chou quoi.

00:27:24
*Intervieweuse:* Oui, feuillu.

00:27:24
*Homme interviewé:* Oui, c'est un feuillu, c'est pas un, un, une racine quoi

00:27:29
*Femme interviewée:* Autrement quand c'est la saison aussi, on mange pas mal de butternut et de-

00:27:32
*Homme interviewé:* Oui.

00:27:34
*Femme interviewée:* Et de potimarron. [euh]

00:27:40
*Intervieweuse:* Oui.

00:27:40
*Femme interviewée:* Si, les carottes, on fait des soupes tous légumes quand c'est la saison aussi, enfin quand on a envie de manger de la soupe. Mais plus tellement en ce moment.

00:27:45
*Intervieweuse:* Oui.

00:27:49
*Homme interviewé:* D'autres types de choux. Brocolis non, mais-

00:27:51
*Femme interviewée:* Si on, j'ai acheté des brocolis l'autre jour. Brocolis de temps en temps, oui. Alors ça, c'est un truc que je n'achète jamais bio par exemple le brocoli.

00:27:59
*Intervieweuse:* D'accord.

00:28:01
*Femme interviewée:* Parce qu'on a eu une mauvaise surprise une fois, à mettre des brocolis bio à cuire avec tous les petits vers qui sortaient, donc c'est...

00:28:10
*Intervieweuse:* Ah !

00:28:10
*Femme interviewée:* Alors si j'ai du bio, souvent, je compare, je jette un œil sur les prix du bio. Quand c'est à peu près comparable, je peux prendre du bio, mais je n'en fais pas une religion.

00:28:21
*Intervieweuse:* D'accord, si le prix est trop important, la différence de prix, vous...

00:28:24
*Femme interviewée:* Non.

00:28:26
*Intervieweuse:* Si c'est un petit peu, vous prenez bio, mais si c'est trop important.

00:28:30
*Femme interviewée:* Alors, il y a des trucs, par exemple le gingembre, je le prends systématiquement bio, parce que la quantité consommée est faible.

00:28:39
*Intervieweuse:* Ok.

00:28:39
*Femme interviewée:* Et je ne veux pas de gingembre de Chine, par exemple, donc là je prends bio.

00:28:44
*Intervieweuse:* D'accord.

00:28:46
*Femme interviewée:* Bon, il y a des, qu'est-ce qu'on peut prendre bio encore ?

00:28:50
*Homme interviewé:* En légumes ?

00:28:51
*Femme interviewée:* Oui,

00:28:51
*Homme interviewé:* [euh]

00:28:53
*Femme interviewée:* Alors ça dépend si on est avec notre fille ou pas, parce qu'il y en a une de nos fille qui, quoi qu'elle s'en défende, aime bien le bio. Dans là quand on est, surtout, chez elle, surtout on va choisir du bio. Si, les carottes bio sont pas chères. Quand on prend des carottes. Souvent un sachet de carottes bio, c'est à peine plus cher que les carottes non bio.

00:29:13
*Intervieweuse:* Oui.

00:29:17
*Femme interviewée:* Les bananes bio aussi, systématiquement aussi, quand on en prend.

00:29:20
*Intervieweuse:* D'accord.

00:29:20
*Femme interviewée:* Les poivrons, vaudrait mieux choisir bio, semble-t-il. Mais là je dois dire que je vérifie pas toujours.

00:29:29
*Homme interviewé:* Les poivrons non, on en mange pas mal aussi.

00:29:29
*Femme interviewée:* Ben avec le couscous quoi-

00:29:29
*Homme interviewé:* Oui, mais, même-

00:29:35
*Femme interviewée:* À une époque, on en mangeait tout seul.

00:29:36
*Homme interviewé:* On en mangeait plus.

00:29:36
*Femme interviewée:* Plus maintenant. Bon que vous dire d'autre.

00:29:42
*Intervieweuse:* Eh bien, ma question c'est, l'idée c'est de dresser une liste de différents fruits et légumes que vous consommez. Donc, là, on a commencé à avoir une belle liste. Et si on s'intéresse à ceux que vous ne consommez pas. Et donc, la cerise vous m'avez dit, par exemple si on s'intéresse aux fruits, que c'est trop cher. En ce moment, il paraît que c'est encore plus-

00:30:00
*Homme interviewé:* C'est encore pire.

00:30:01
*Intervieweuse:* Oui, il parait.

00:30:02
*Femme interviewée:* 15 euros le kilo l'autre jour.

00:30:04
*Intervieweuse:* Voilà.

00:30:04
*Femme interviewée:* On ne comprend pas.

00:30:05
*Intervieweuse:* Effectivement, ça-

00:30:08
*Femme interviewée:* Les, les fraises étaient au même prix. Ceci dit, avec un kilo de fraises, on peut faire beaucoup plus de choses qu'avec un kilo de cerises.

00:30:15
*Intervieweuse:* Oui.

00:30:15
*Femme interviewée:* Puis bon, aussi c'est vrai, je ne prends que des fraises de [inaudible 00:30:18].

00:30:22
*Intervieweuse:* D'accord.

00:30:22
*Femme interviewée:* Jamais de fraises espagnoles ou marocaines, ça c'est sûr.

00:30:27
*Intervieweuse:* Ok, pourquoi vous préférez ?

00:30:30
*Femme interviewée:* Parce que, enfin, je, à tort ou à raison, je, je pense que les standards, l'utilisation des pesticides en France est plus réglementée qu'en Espagne et au, et surtout au Maroc.

00:30:39
*Intervieweuse:* D'accord.

00:30:42
*Femme interviewée:* C'est peut-être faux pour l'Espagne, parce que c'est quand même l'Europe.

00:30:44
*Homme interviewé:* Ouais, enfin.

00:30:44
*Femme interviewée:* Mais c'est sûr qu'en France, on, il y a quand même plus de contrôles de pesticides qu'en Espagne.

00:30:55
*Intervieweuse:* C'est possible, oui non, je ne sais pas, c'est possible. Donc, vous préférez prendre, quand vous pouvez prendre des choses qui viennent de France en espérant qu'il y ait eu moins de pesticides.

00:31:00
*Femme interviewée:* En espérant, je ne sais pas si c'est un vœux pieux.

00:31:05
*Homme interviewé:* L'espoir fait vivre. [rire]

00:31:09
*Intervieweuse:* Vous m'avez dit que la prune, les prunes vous, vous en achetez que très peu parce que vous avez été déçus de-

00:31:15
*Femme interviewée:* Souvent, souvent, c'est soit farineux, soit-

00:31:17
*Homme interviewé:* Insipide.

00:31:19
*Femme interviewée:* Soit pas assez mûr.

00:31:21
*Homme interviewé:* Soit les deux.

00:31:22
*Intervieweuse:* Oui.

00:31:22
*Femme interviewée:* Après ça dépend où l'on est aussi. Parce que ici, c'est le cas, quand on va chez nos filles en région toulousaine, on peut en trouver des très bonnes, comme les abricots, par exemple.

00:31:33
*Intervieweuse:* D'accord.

00:31:33
*Femme interviewée:* En région toulousaine, on peut trouver des très bons abricots, ici c'est, c'est plus risqué.

00:31:42
*Intervieweuse:* Oui. D'accord, donc c'est pas un problème de goût, enfin si, c'est un problème de goût, mais à la base vous aimez le fruit.

00:31:46
*Homme interviewé:* Ah oui.

00:31:46
*Intervieweuse:* C'est juste qu'ici, vu que chaque vous trouvez pas très bon.

00:31:49
*Homme interviewé:* Un bon abricot, une bonne prune, c'est bien.

00:31:52
*Femme interviewée:* Oui oui, en goût, alors, qu'est-ce qu'on n'aime pas en fruits et légumes ? Qu'est-ce qu'on n'aime pas ? Moi, je n'aime pas trop le fenouil. On en met un peu dans le couscous, mais j'aime pas trop. Je ne mangerais pas du fenouil en plat, par exemple.

00:32:06
*Intervieweuse:* D'accord.

00:32:08
*Femme interviewée:* Le céleri, j'aime pas. Toi t'aimes bien, j'aime pas. Qu'est-ce qu'on n'aime pas ?

00:32:15
*Homme interviewé:* Je pense aux épinards, mais là on aime, au contraire.

00:32:17
*Femme interviewée:* Les épinards on les prend uniquement surgelés.

00:32:19
*Intervieweuse:* D'accord.

00:32:20
*Femme interviewée:* On n'achète jamais d'épinards frais.

00:32:22
*Intervieweuse:* Pourquoi, vous... ?

00:32:23
*Femme interviewée:* Parce que c'est, on en trouve en surgelés pas cher, alors qu'en frais, il faut en prendre des quantités, laver. C'est plus facile de les prendre surgelés.

00:32:32
*Intervieweuse:* D'accord.

00:32:33
*Femme interviewée:* Pareil les haricots verts, sauf quand c'est vraiment la saison et qu'ils sont pas chers, on les prend en surgelé.

00:32:39
*Intervieweuse:* D'accord.

00:32:42
*Homme interviewé:* Qu'est-ce qu'on aime pas ?

00:32:45
*Femme interviewée:* Qu'est-ce qu'on aime pas ?

00:32:46
*Homme interviewé:* En légumes, fruits et légumes ?

00:32:52
*Intervieweuse:* Les bananes, vous avez dit que vous en achetez que rarement, c'est pourquoi, vous...

00:32:58
*Femme interviewée:* Parce que ça se conserve pas très bien d'une part, et puis on, c'est pas spécialement-

00:33:01
*Homme interviewé:* Y a meilleur.

00:33:02
*Femme interviewée:* Oui, c'est pas un fruit qu'on apprécie, enfin c'est pas le meilleur des fruits pour nous.

00:33:04
*Homme interviewé:* On termine celles que les petits enfants [rire].

00:33:09
*Femme interviewée:* Oui, c'est pas ce qu'on apprécie le plus, voilà.

00:33:11
*Intervieweuse:* D'accord, donc d'une au niveau du goût, c'est pas l'extase pour vous. Et en plus, ça se conserve pas non plus terrible terrible. Donc, si vous n'avez pas envie d'en manger tous les jours... Ok.

00:33:21
*Homme interviewé:* Après ça se mange.

00:33:28
*Femme interviewée:* Oui, enfin on n'en achète pas pour-

00:33:29
*Homme interviewé:* On ne court pas après.

00:33:32
*Intervieweuse:* Oui, et vous m'avez dit par contre que vous consommez de l'ananas.

00:33:35
*Femme interviewée:* Oui.

00:33:36
*Intervieweuse:* Est-ce que vous avez d'autres fruits exotiques que vous allez consommer ou c'est exclusivement l'ananas et de temps en temps un peu la banane ?

00:33:45
*Homme interviewé:* On essaye la mangue de temps en temps.

00:33:47
*Femme interviewée:* La mangue, c'est compliqué parce que moi je sais mal choisir les mangues. Et... Non, c'est rare, c'est rare parce que les expériences de mangues sont quand même très, très décevantes en général.

00:33:58
*Homme interviewé:* Les kakis.

00:34:00
*Femme interviewée:* Kakis, on en a déjà mangé des kakis ? Ah oui, j'aime beaucoup les kakis quand c'est la saison. Mais alors cette année, c'était aussi, j'en ai pas, j'en ai peu mangé parce qu'ils étaient, je ne sais pas comment dire, astringents quand on les met en bouche. Ils étaient très astringents, très désagréables.

00:34:14
*Intervieweuse:* D'accord.

00:34:15
*Femme interviewée:* Donc je ne sais pas si j'ai mal choisi ou si c'est cette année qu'ils étaient comme ça, mais l'hiver précédent, j'en ai pas mal mangé des kakis, ils étaient très bons. Bon les kiwis on en mange pas, peu, moi, j'en mangerais bien, mais bon ce n'est pas mon fruit préféré, donc-

00:34:32
*Intervieweuse:* D'accord, et vous n'aimez pas ?

00:34:35
*Homme interviewé:* Non, non, ce n'est pas ma tasse de thé, c'est en-dessous de la banane.

00:34:41
*Intervieweuse:* On fait un classement.

00:34:44
*Femme interviewée:* En fruits exotiques, qu'est-ce qu'on pourrait-

00:34:46
*Homme interviewé:* Le raisin jamais.

00:34:47
*Femme interviewée:* Non, on ne mange pas de raisin, c'est vrai. Toi tu n'aimes pas le raisin à cause des pépins.

00:34:50
*Homme interviewé:* Oui, ça m'agace.

00:34:50
*Femme interviewée:* Je ne cours pas après non plus. Non, on n'en mange pas, non.

00:34:50
*Intervieweuse:* D'accord.

00:34:55
*Homme interviewé:* Je pense à un autre truc, là. Fruits exotiques...

00:35:01
*Femme interviewée:* Le raisin n'est pas un fruit exotique.

00:35:07
*Intervieweuse:* Non.

00:35:07
*Femme interviewée:* Sauf quand il vient d'Afrique du Sud.

00:35:07
*Homme interviewé:* On est en Bretagne quand même. [rire]

00:35:08
*Femme interviewée:* Sauf quand il vient d'Afrique du Sud.

00:35:17
*Intervieweuse:* Qu'est-ce qu'il y a comme fruits. On a fait un bon petit tout. Et sur les légumes, est-ce qu'il y en a là que vous voyez que vous consommez pas. Par exemple le brocoli, vous avez dit que vous le consommiez qu'un peu. Qu'est-ce qui fait que vous le consommiez peu par rapport à l'aubergine que vous allez vouloir consommer toute l'année plus souvent ?

00:35:36
*Femme interviewée:* C'est une question surtout de préparation et de conservation.

00:35:40
*Intervieweuse:* D'accord.

00:35:44
*Femme interviewée:* Parce que ça se conserve pas bien du tout à la température ambiante. Ça file très vite. Et puis après, en terme de préparation, peut-être que c'est un manque de savoir faire de ma part, mais à part le manger comme ça ou éventuellement sur une tarte, je ne vois pas.

00:36:02
*Intervieweuse:* D'accord, d'une, c'est pas facile, voilà, on risque de le perdre assez vite. Et en plus, c'est pas, voilà, vous n'avez pas-

00:36:10
*Femme interviewée:* Je n'ai pas d'idée de comment le faire, comment le cuisiner pour qu'on puisse le conserver au réfrigérateur.

00:36:17
*Homme interviewé:* Pareil le poireau seul, on fait plus tellement.

00:36:24
*Intervieweuse:* Le poireau, oui, vous n'en consommez pas tant que ça ?

00:36:26
*Homme interviewé:* Pourtant-

00:36:26
*Femme interviewée:* Pourtant, je sais que tu adores.

00:36:27
*Homme interviewé:* Objectivement, je trouve le poireau très bon.

00:36:30
*Femme interviewée:* Oui, [*Homme interviewé]* aime beaucoup le poireau, mais je n'en fais pas.

00:36:34
*Homme interviewé:* C'est pas grave, on fait des tomates [rire].

00:36:41
*Intervieweuse:* Les tomates ! [euh] Ok, mais sinon oui, vous mangez quand même les courges, le chou-fleur ça vous arrive ou pas du tout ?

00:36:48
*Femme interviewée:* Oui oui.

00:36:49
*Homme interviewé:* Oui, oui, tout à fait. le chou-

00:36:53
*Femme interviewée:* Si si, le chou-fleur. Ce n'est pas régulier. C'est peut-être une fois par quinzaine, à peu près, un chou-fleur ou deux une fois par quinzaine. Ça dépend du prix là aussi, ça dépend comme il est.

00:37:02
*Intervieweuse:* Oui.

00:37:05
*Homme interviewé:* Des fois ils sont très feuillus.

00:37:05
*Femme interviewée:* Oui, c'est ça, il faut voir comment-

00:37:07
*Homme interviewé:* Il ne reste plus grand chose une fois enlevé le trognon.

00:37:10
*Intervieweuse:* Ok.

00:37:10
*Homme interviewé:* Le chou-

00:37:10
*Femme interviewée:* Ben le chou doux, ça c'est-

00:37:10
*Homme interviewé:* Non, enfin si, le chou doux a un peu détrôné-

00:37:18
*Femme interviewée:* Le chou-fleur, oui.

00:37:18
*Homme interviewé:* Le chou tout court. Sinon la choucroute, mais bon.

00:37:18
*Femme interviewée:* Ah oui la choucroute, enfin je ne sais pas si c'est un légume, enfin en saison on mange pas mal de choucroute. C'est pas nous qui la faisons évidemment, mais-

00:37:30
*Homme interviewé:* Si, ça peut être un légume puisque que-

00:37:30
*Intervieweuse:* Oui, vous l'achetez déjà-

00:37:35
*Femme interviewée:* Oui, on achète la choucroute nature, pas le dîner.

00:37:40
*Intervieweuse:* Ok, la carotte, vous n'en consommez que très peu, pourquoi ? C'est par, vous avez dit par exemple cru vous n'aimez pas trop ça.

00:37:52
*Femme interviewée:* Non, après on peut en faire des soupes de carottes. On a une de nos filles et sa fille qui adorent la soupe de carottes alors on en fait, mais. Bon, un peu sucrée à notre goût en fait.

00:38:03
*Homme interviewé:* Oui.

00:38:03
*Femme interviewée:* C'est un légume sucré quand même.

00:38:08
*Intervieweuse:* D'accord, oui, c'est pas l'extase non plus au niveau de-

00:38:09
*Femme interviewée:* Non. Du tout. Un bœuf aux carottes, c'est bon, mais enfin.

00:38:12
*Homme interviewé:* La tomate, il y a l'acidité qui est chouette.

00:38:16
*Intervieweuse:* Oui.

00:38:16
*Homme interviewé:* Dans les carottes, c'est un peu trop sucré.

00:38:21
*Femme interviewée:* Un peu trop sucré.

00:38:23
*Intervieweuse:* Vous préférez un peu d'acidité plutôt que quelque chose de-

00:38:26
*Femme interviewée:* Oui.

00:38:27
*Intervieweuse:* De très, pas trop doux, mais-

00:38:29
*Homme interviewé:* Moi, je suis assez attiré par l'acidité.

00:38:33
*Intervieweuse:* Oui. Et le citron, d'ailleurs. Ça vous arrive de consommer des citrons ?

00:38:35
*Homme interviewé:* Sauf le citron.

00:38:37
*Intervieweuse:* Ah pas le citron.

00:38:38
*Femme interviewée:* C'est rare, c'est très rare.

00:38:38
*Intervieweuse:* C'est trop acide là. [rire]

00:38:42
*Homme interviewé:* Et des agrumes tels que les mandarines-

00:38:44
*Femme interviewée:* Ah oui, les agrumes d'hiver, clémentines, moi, j'en mange en saison quand il y en a. Ça c'est mon fruit d'hiver, toi t'en manges jamais.

00:38:53
*Homme interviewé:* Très peu.

00:38:56
*Femme interviewée:* Par contre, je n'aime pas les oranges. On n'aime pas les oranges.

00:38:57
*Intervieweuse:* D'accord.

00:39:01
*Femme interviewée:* Alors clémentines, mandarines.

00:39:01
*Homme interviewé:* Le seuil d'acidité, c'est pas toujours pas toujours accepté.

00:39:04
*Intervieweuse:* Ça dépend finalement.

00:39:05
*Femme interviewée:* Après clémentines ou mandarines, je sais pas ce que je prends.

00:39:07
*Intervieweuse:* Oui. [rire]

00:39:08
*Femme interviewée:* Je ne sais jamais la différence.

00:39:12
*Intervieweuse:* Et l'orange, c'est en goût ? Qu'est-ce qui fait que l'orange, ça, voilà, vous... ?

00:39:16
*Femme interviewée:* La difficulté d'épluchage déjà, par rapport à une mandarine qui s'épluche en dix secondes.

00:39:21
*Intervieweuse:* Oui.

00:39:23
*Homme interviewé:* C'est plus fastidieux.

00:39:24
*Femme interviewée:* Après au niveau du goût, si c'est une bonne orange sucrée, on aime bien, mais c'est, voilà c'est pareil, je ne sais pas choisir les bonnes oranges.

00:39:34
*Homme interviewé:* Pamplemousse on n'en sert plus.

00:39:37
*Femme interviewée:* Non, on a trop peu à manger je trouve. [rire]

00:39:44
*Intervieweuse:* Ok. Alors là si on s'intéresse à vos lieux de stockage des fruits et légumes, quand vous revenez de vos courses, donc là vous m'avez dit, vous cuisinez direct le lendemain.

00:39:56
*Homme interviewé:* Oui, c'est assez rapide.

00:39:58
*Intervieweuse:* Vos légumes notamment. Et est-ce que vous pouvez me dire en terme de rangement, c'est quoi, qu'est-ce que vous faites de vos fruits, qu'est-ce que vous faites de vos légumes ?

00:40:06
*Femme interviewée:* Les fruits, normalement, restent dans des paniers à fruits dans la cuisine.

00:40:12
*Intervieweuse:* D'accord.

00:40:13
*Femme interviewée:* Après en surveillance, quitte à les mettre au réfrigérateur si ça mûrit trop vite et qu'on n'a pas-

00:40:21
*Homme interviewé:* Qu'on n'a pas le débit suffisant.

00:40:22
*Femme interviewée:* Qu'on a pas d'espoir de consommation le lendemain. Les légumes, le plus souvent vont entre les deux fenêtres.

00:40:30
*Intervieweuse:* Oui, je me souviens de cet endroit.

00:40:31
*Femme interviewée:* Parce que là c'est un espace- Alors en hiver, ça fait une sorte de réfrigérateur. En été c'est moins vrai.

00:40:40
*Homme interviewé:* C'est moins évident.

00:40:42
*Femme interviewée:* Et si on doit, s'il y a des légumes, surtout des légumes, les fruits, ça arrive pas, qu'on doit mettre en oeuvre plus tard. Alors tant que les températures sont pas trop élevées, mais bon, ici, c'est quand même rare qu'on arrive à-

00:40:56
*Homme interviewé:* À ces températures-là.

00:40:57
*Femme interviewée:* À ces températures-là, ça va au garage parce que c'est un endroit plus frais que le reste de la maison. Surtout en hiver, automne, début du printemps.

00:41:05
*Intervieweuse:* Ok.

00:41:09
*Femme interviewée:* Et après, on les prend au fur et à mesure du garage. Alors ce qui va au réfrigérateur, il y a peu de choses. Je vous disais les aubergines. Et quoi d'autre ? Les carottes, ça peut arriver, alors quand on prend un sachet de carottes. Je l'ouvre pour pas que ça macère et puis ça va, enfin pour pas-

00:41:27
*Homme interviewé:* Pour qu'il y ait pas trop d'humidité.

00:41:28
*Femme interviewée:* L'humidité, ça peut aller, ça va au réfrigérateur, mais pas systématiquement. Ça peut rester aussi dans le sac au garage. Quant aux fruits, ben ils sont dans le panier de la cuisine, dans les paniers de la cuisine. Le plus souvent.

00:41:49
*Intervieweuse:* D'accord. Je pense à un légume, le concombre, est-ce que vous en consommez ou pas du tout ?

00:41:52
*Femme interviewée:* Moi j'en consomme, oui.

00:41:53
*Intervieweuse:* D'accord. Vous n'aimez pas ?

00:41:56
*Homme interviewé:* J'ai du mal, j'ai du mal avec le concombre. Parfois, j'en mange.

00:42:00
*Femme interviewée:* Il digère mal.

00:42:01
*Homme interviewé:* J'en ai rapidement, oui, ma claque.

00:42:03
*Femme interviewée:* Hier par exemple, tu as mangé des tomates en salade, moi j'ai mangé du concombre.

00:42:09
*Intervieweuse:* D'accord, donc ça arrive. Et ce concombre, vous le mettez au frigo ou non ?

00:42:12
*Femme interviewée:* Oui.

00:42:14
*Intervieweuse:* Vous le mettez aussi au frigo. Ok et on n'a pas parlé d'oignons, d'ail et de pommes de terre, est-ce que vous en consommez ?

00:42:23
*Femme interviewée:* Oui.

00:42:23
*Intervieweuse:* Enfin échalotes, ce genre de-

00:42:26
*Femme interviewée:* Alors les pommes de terre sont stockées dans un bac sous l'évier.

00:42:29
*Intervieweuse:* Oui !

00:42:29
*Femme interviewée:* Je vous avais envoyé une photos d'ailleurs après, parce qu'on n'en avait pas parlé.

00:42:32
*Homme interviewé:* [inaudible 00:42:32]

00:42:32
*Intervieweuse:* Oui, d'accord.

00:42:35
*Femme interviewée:* Et là, alors, on prend toujours, quand on la, enfin, quand on, on ne prend que ça, et si il n'y a pas, on ne prend pas. C'est les pommes de terre bio de, qu'on trouve à Super U. C'est les seules dont on est sûr de la qualité. À notre goût en tout cas.

00:42:51
*Homme interviewé:* Oui, elles ont aussi bien une consistance qu'une cuisson-

00:42:55
*Femme interviewée:* Elles se conservent bien.

00:42:55
*Homme interviewé:* Elles se conservent bien, le goût est bien.

00:43:00
*Femme interviewée:* Le goût, la texture, quand elles sont cuites, on peut les réchauffer le lendemain et c'est comme si on avait des pommes de terre fraîchement cuites.

00:43:08
*Intervieweuse:* D'accord.

00:43:08
*Homme interviewé:* Elles sont pas farineuses.

00:43:09
*Femme interviewée:* Donc, c'est vrai que quand il n'y a pas ces pommes de terre-là, on ne prend pas pas de pommes de terre.

00:43:13
*Intervieweuse:* D'accord. C'est votre, c'est tout ou rien.

00:43:17
*Femme interviewée:* Alors l'ail, c'est, c'est l'ail surgelé. Je prends l'ail surgelé.

00:43:22
*Intervieweuse:* D'accord, qu'est-ce qui fait que vous préférez prendre surgelé sur l'ail ?

00:43:26
*Femme interviewée:* C'est plus facile à mettre en oeuvre. Il suffit de faire tomber quelques copeaux du sachet et puis-

00:43:36
*Homme interviewé:* Après l'oignon, c'est différent.

00:43:37
*Femme interviewée:* Alors l'oignon, fut un temps où on prenait beaucoup d'oignons surgelés. Maintenant, on n'en prend plus et j'en achète peu. Tout dépend des habitudes de consommation du moment.

00:43:52
*Intervieweuse:* D'accord.

00:43:54
*Femme interviewée:* Si on en achète, on les stocks avec les pommes de terre sous le bac de l'évier. Tout en surveillant parce que ça peut pourrir quand même.

00:44:06
*Intervieweuse:* Et est-ce que vous consommez ou pas de la salade ?

00:44:10
*Femme interviewée:* Rarement.

00:44:11
*Intervieweuse:* Rarement ?

00:44:12
*Femme interviewée:* Moi j'aime pas, [*Homme interviewé]* aime bien, mais c'est rare. Quand on achète une salade, en général, enfin ça peut arriver qu'il y ait la moitié qui parte au compost parce que moi, j'aime pas et toi t'as pas réussi à manger la salade en entier, la laitue en entier.

00:44:27
*Intervieweuse:* Oui.

00:44:29
*Homme interviewé:* Disons que je me fais bien, je me ferais bien une gloutonnerie de salade, mais en même temps, bon, ça fait beaucoup quoi.

00:44:37
*Femme interviewée:* Oui, ça peut arriver qu'on en prenne quand même quand on est pas que tous les deux. Quand les enfants sont là.

00:44:44
*Homme interviewé:* Oui, mais c'est souvent moi qui termine.

00:44:48
*Femme interviewée:* Oui, malgré tout ils ont pas une consommation effrénée de salade.

00:44:49
*Intervieweuse:* D'accord. Et quand vous en avez, vous la conservez où ?

00:44:55
*Femme interviewée:* On la lave tout de suite.

00:44:56
*Intervieweuse:* D'accord.

00:44:59
*Femme interviewée:* Et on conserve les feuilles dans un sac en plastique au frigo.

00:45:03
*Homme interviewé:* Entrouvert.

00:45:07
*Intervieweuse:* Donc, vous la lavez, vous-

00:45:09
*Femme interviewée:* On retire les feuilles, on lave, on passe avec un peu d'eau vinaigrée, histoire de, enfin on fait tremper dans un peu d'eau vinaigrée, enfin c'est toi qui fais d'habitude, alors comment tu fais ?

00:45:17
*Homme interviewé:* Ben oui, je mets l'eau vinaigrée. Si je suis impatient, je fais autre chose, comme ça ça se débrouille tout seul. Jje fais un deuxième rinçage et puis je regarde l'état de l'eau, en fait. Et puis, je mets en sachets-

00:45:33
*Femme interviewée:* Un essorage quand même, c'est ça?

00:45:34
*Homme interviewé:* Oui, oui, j'essors pas trop violemment parce que ça la brise quoi. Ça fait des blessures qui rosissent [inaudible 00:45:46]. Enfin qui rosissent de toute façon. L'aspect de salade est moins sympa après.

00:45:49
*Intervieweuse:* Ok.

00:45:52
*Homme interviewé:* L'une des raisons qui fait qu'on prend de le feuille de chêne rouge, souvent. C'est moi qui ai choisi, puisque t'es pas adepte de salade, mais plutôt feuille de chêne rouges, vu que, [euh] sa couleur planque que les défauts de [rire].

00:46:10
*Femme interviewée:* Et elles sont de façon générale plus petites que les laitues aussi.

00:46:11
*Intervieweuse:* D'accord.

00:46:11
*Femme interviewée:* Donc ça correspond mieux à notre consommation.

00:46:15
*Homme interviewé:* Je pense que j'aime mieux, tout compte fait, la chair de la, de cette feuille de chêne.

00:46:24
*Femme interviewée:* Alors ça, c'est quelque chose qu'on ne prendra jamais en sachet tout fait, par exemple.

00:46:27
*Femme interviewée:* D'accord, pourquoi du coup ?

00:46:27
*Homme interviewé:* On a appris à une époque, c'était-

00:46:29
*Femme interviewée:* Parce qu'on a lu, que c'était, déjà, pas top au niveau de ce qu'ils mettent pour la conservation, puis de toute façon il faudrait la relaver, la re-essorer, donc [pff], autant acheter la-

00:46:42
*Homme interviewé:* Non, ça apporte rien.

00:46:42
*Femme interviewée:* La salade nature et à la faire comme ça.

00:46:49
*Intervieweuse:* Ok. D'accord. Du coup, vous avez en terme de contenants de stockage, le frigo, donc ils sont tous au même endroit ?

00:47:03
*Femme interviewée:* Non.

00:47:03
*Intervieweuse:* Non, c'est un peu partout dans le frigo ?

00:47:04
*Femme interviewée:* Ça va dépendre de la place. Ça veut dire que je ne fais pas attention à, je ne mets pas spécialement dans le bac à légumes.

00:47:11
*Intervieweuse:* D'accord. Vous avez du coup à température ambiante les fruits, notamment.

00:47:19
*Femme interviewée:* Alors essentiellement les fruits, qui peuvent aussi être entre les deux fenêtres suivant la saison et leur degré de mûrissement.

00:47:27
*Intervieweuse:* D'accord.

00:47:28
*Femme interviewée:* Donc, souvent l'entre deux fenêtre là quand il fait pas trop chaud, ça fait un sas pour faire mûrir. Et puis, après on met dans-

00:47:34
*Homme interviewé:* C'est quand même une bonne partie de l'année globalement.

00:47:37
*Intervieweuse:* Oui.

00:47:38
*Femme interviewée:* On met après dans la cuisine, s'ils sont bons à consommer, quitte à remettre entre les deux fenêtres s'il faut les conserver un peu plus.

00:47:45
*Intervieweuse:* Donc à température ambiante, on va retrouver ce que vous allez consommer là bientôt-

00:47:51
*Femme interviewée:* Dans toute la journée ou les 48 heures.

00:47:53
*Intervieweuse:* D'accord. Finalement, vous avez presque un, deux, trois, quatre, presque cinq lieux de stockage : il y a le frigo, la température ambiante, l'entre deux fenêtre, le garage et sous l'évier.

00:48:09
*Homme interviewé:* Oui.

00:48:11
*Femme interviewée:* Oui.

00:48:12
*Intervieweuse:* Alors, et en terme de, comment vous, donc l'entre deux fenêtres, si vous trouvez ça, c'est pratique, notamment l'hiver, ça vient réfrigérer. Est-ce que vous êtes plutôt satisfaits de la manière dont vos fruits et légumes se conservent ou est-ce qu'il y en a ou vous dites : "Ben non, on n'arrive pas avec celui-là, on n'arrive jamais à les faire tenir." Qu'est-ce que, comment vous pensez vos lieux de stockage ?

00:48:34
*Femme interviewée:* Ben c'est-

00:48:38
*Homme interviewé:* C'est le recul qui nous fait dire que oui, c'est là que ça se passe le mieux, on a l'œil aussi sur les, quand c'est à la fenêtre. On a l'œil dessus.

00:48:47
*Femme interviewée:* Ce qu'on a jeté là dans la poubelle connectée, je ne sais pas si vous avez le-

00:48:52
*Homme interviewé:* Le bilan.

00:48:52
*Femme interviewée:* Le récapitulatif de ce qu'on a jeté.

00:48:54
*Homme interviewé:* Il n'y a pas eu grand chose.

00:48:54
*Intervieweuse:* On a les poids, oui..

00:48:54
*Femme interviewée:* On a jeté le sac hier quand même. Parce qu'on avait laissé le premier sac depuis le début. D'ailleurs on a été surpris-

00:49:02
*Intervieweuse:* C'était du jus, non ?

00:49:03
*Homme interviewé:* Oui, c'était un peu juteux oui.

00:49:04
*Intervieweuse:* Tout le monde a eu son petit jus. [rires]

00:49:07
*Femme interviewée:* Et le sac fuyait, donc-

00:49:09
*Intervieweuse:* Oui, c'est-

00:49:09
*Femme interviewée:* Mais donc je ne sais pas au poids, si, par rapport aux autres personnes que vous avez eu, si c'est beaucoup ou pas beaucoup, j'en sais rien. Mais j'ai pas l'impression qu'on jette beaucoup.

00:49:18
*Intervieweuse:* D'accord.

00:49:20
*Homme interviewé:* Non, on n'a pas jeté beaucoup.

00:49:22
*Femme interviewée:* J'ai l'impression qu'on arrive à se débrouiller assez bien entre, entre ces différents lieux de stockage, la surveillance, la mise en œuvre culinaire rapide pour, pour pas que ça se gâte.

00:49:36
*Intervieweuse:* D'accord. C'est vrai que c'est une articulation entre le fait que vous avez l'œil dessus aussi dans l'entre deux fenêtres. Il y a le fait que vous allez aussi régulièrement-

00:49:46
*Femme interviewée:* Oui, c'est ça.

00:49:47
*Intervieweuse:* Faire des achats.

00:49:47
*Homme interviewé:* Oui.

00:49:48
*Intervieweuse:* Donc ça vous demande pas d'acheter non plus des quantités [euh]. Et vous avez quand même pas mal de températures différentes, ce qui permet de- Du coup, vous avez sentiment de ne pas jeter beaucoup.

00:50:00
*Femme interviewée:* C'est le sentiment qu'on a.

00:50:01
*Intervieweuse:* Ok, alors en terme de nettoyage de ces lieux, est-ce que vous avez une organisation systématique ou c'est un peu quand... ?

00:50:12
*Femme interviewée:* Non. C'est quand on voit que c'est, que ça mérite. [rire] Donc c'est pas, c'est pas systématique. S'il y a un fruit qui a pourri dans un des paniers à fruits, par exemple, on va le laver, c'est sûr.

00:50:26
*Intervieweuse:* Oui.

00:50:28
*Femme interviewée:* Autrement non, on fait, le panier à fruits peut rester six mois sans qu'on le lave.

00:50:31
*Intervieweuse:* Oui.

00:50:32
*Femme interviewée:* Bon le frigo, c'est un peu plus souvent parce que, on ne le fait pas, on ne lave pas le frigo de façon-

00:50:39
*Homme interviewé:* De façon systématique.

00:50:41
*Femme interviewée:* Régulièrement quand même, mais je dis pas, je dirais pas de façon complètement hygiéniste ou hygiénique, parce qu'on se contente de passer quand même-

00:50:48
*Homme interviewé:* Alors moi, si je fais [inaudible 00:50:49].

00:50:50
*Femme interviewée:* Oui, mais c'est pas souvent.

00:50:51
*Homme interviewé:* Non.

00:50:51
*Femme interviewée:* Après, je passe souvent une éponge avec de l'eau un peu javélisée sur les différentes surfaces.

00:50:56
*Intervieweuse:* D'accord.

00:50:59
*Femme interviewée:* Et entre les deux fenêtres, alors là, c'est peut-être deux fois par an quoi.

00:51:03
*Intervieweuse:* D'accord.

00:51:04
*Homme interviewé:* En même temps, on a l'œil dessus, parce que des fois, il y a des traces, il y a un fruit ou une tomate qui a moisi. Bon, allez hop, là on vire tout et-

00:51:14
*Femme interviewée:* Oui.

00:51:15
*Homme interviewé:* On fait un nettoyage, surtout pour lutter contre les moisissures quoi, qui forcément s'implantent sinon. De toute façon, elles s'implantent, mais bon.

00:51:25
*Intervieweuse:* Donc l'entre deux fenêtres, c'est un peu comme la corbeille, si vraiment vous voyez qu'il y a quelques qui a coulé ou quoi.

00:51:34
*Homme interviewé:* Faut faire quelque chose.

00:51:37
*Intervieweuse:* Oui. Ok. Alors là, si on s'intéresse à vos pratiques de préparation et de consommation des fruits et légumes, vous m'avez déjà raconté pas mal de fois de petites recettes que vous, que vous avez l'habitude de faire. Si on prend un fruit que vous avez dit consommer régulièrement, par exemple l'ananas que vous trouvez toute l'année. Comment vous consommez l'ananas ? Le plus souvent, après de temps en temps, ou est-ce que vous faites autre chose avec ?

00:52:13
*Femme interviewée:* Non, c'est uniquement coupé cru.

00:52:15
*Intervieweuse:* D'accord.

00:52:16
*Femme interviewée:* Quand on l'achète, on le met entre les deux fenêtres, et puis après, suivant l'allure qu'il prend, je le coupe. Soit pour consommer tout de suite, soit, de toute façon, on ne va pas consommer un ananas, tous les, en un repas à deux, on ça fait au moins deux repas parce qu'on fait huit portions, oui, ça fait deux repas. Pas forcément un seul jour, ça peut être sur deux jours.

00:52:36
*Intervieweuse:* Ok.

00:52:36
*Femme interviewée:* Donc oui, non, je fais pas, j'ai essayé des recettes sucrées avec l'ananas cuit, mais non, c'est pas, c'est décevant.

00:52:42
*Intervieweuse:* C'est un peu dommage.

00:52:46
*Femme interviewée:* Oui, c'est dommage, un bon ananas, c'est très bon quand même.

00:52:49
*Intervieweuse:* Pourquoi vous trouvez ça dommage ?

00:52:50
*Femme interviewée:* Parce qu'un bon ananas cru, c'est très bon.

00:52:53
*Homme interviewé:* Ça dénature un peu, la chaire est moins ferme, le, il perd de son acidité,.

00:53:02
*Intervieweuse:* Oui.

00:53:03
*Homme interviewé:* Autrement l'abricot.

00:53:08
*Intervieweuse:* Finalement, quand il est cuit, vous perdez ce que vous avez un peu-

00:53:11
*Homme interviewé:* Ça ne le bonifie en rien.

00:53:13
*Femme interviewée:* Oui. Et puis, voilà ça nous fait quand même le fruit cru, sans apprêt, je dirais. Alors que s'il est cuisiné, ben il y aura forcément un peu de sucre ou y avoir de la crème, de l'œuf, de la farine. Enfin toute chose qui-

00:53:24
*Intervieweuse:* Oui.

00:53:26
*Femme interviewée:* Qui contribue-

00:53:26
*Homme interviewé:* Aux kilos.

00:53:29
*Femme interviewée:* À faire qu'on maîtrise pas trop ce qu'on mange quoi.

00:53:33
*Intervieweuse:* Oui. D'accord. Et donc l'abricot, vous m'avez dit que vous aviez fait une tarte l'autre jour, est-ce que c'est un fruit que vous allez plus cuisiner ? Ou non, c'est resté exceptionnel, comment vous mangez l'abricot ?

00:53:46
*Femme interviewée:* On peut faire des tartes, des clafoutis.

00:53:49
*Intervieweuse:* D'accord.

00:53:49
*Femme interviewée:* Ça, ça va dépendre s'ils sont [euh], quand on en achète, on va goûter un.

00:53:52
*Intervieweuse:* D'accord.

00:53:53
*Femme interviewée:* S'il est bon, on va les consommer comme ça et s'ils ne sont pas très bons, on va les convertir en tartes ou clafoutis.

00:54:03
*Intervieweuse:* Si c'est, si le goût vous plaît, vous, vous allez préférer manger cru.

00:54:08
*Femme interviewée:* Oui.

00:54:08
*Homme interviewé:* Ah oui.

00:54:08
*Intervieweuse:* Et si vous trouvez ça pas terrible, vous allez du coup le-

00:54:11
*Homme interviewé:* Le convertir.

00:54:13
*Femme interviewée:* Comme les fruits d'été aussi, par exemple nectarines, etc. Si elles arrivent pas à mûrir, par exemple, ça arrive que, ça arrive que ça n'arrive pas à mûrir et, ou alors que ça mûrit trop vite, ben je le passe au four avec un petit peu de sucre et ça, ça fait un gratin de fruits, un peu de sucre bon. J'utilise rarement du sucre d'ailleurs, je mets plutôt de la stévia. Ça c'est-

00:54:37
*Intervieweuse:* C'est quoi la-

00:54:41
*Femme interviewée:* La stévia ? C'est du sucre de-

00:54:43
*Homme interviewé:* C'est tiré d'une plante.

00:54:43
*Femme interviewée:* -de cactus, qui est, qui apporte pas de calories. Et qui est, sucre deux fois plus que le sucre de canne, donc [inaudible 00:54:55] voilà. Donc.

00:54:58
*Intervieweuse:* Ok. Donc les, finalement, les fruits, c'est plutôt crus. Mais quand ils vous plaisent pas trop, vous avez des-

00:55:08
*Femme interviewée:* C'est ça.

00:55:09
*Homme interviewé:* Oui, ou s'ils s'impatientent. Il faut faire quelque chose pour eux. [rire]

00:55:12
*Femme interviewée:* S'ils ne nous conviennent pas au goût ou s'ils s'impatientent comme tu dis, ben on convertit, on cuit.

00:55:17
*Intervieweuse:* D'accord. Ok. [euh] Est-ce que les pommes, pareil, est-ce que les pommes, vous les cuisinez ou c'est exclusivement cru ? Comment vous... ?

00:55:32
*Femme interviewée:* C'est essentiellement cru, mais ça c'est, moi j'en mange pas.

00:55:35
*Homme interviewé:* C'est surtout moi.

00:55:36
*Femme interviewée:* Alors ça, c'est toi qui en manges.

00:55:36
*Intervieweuse:* D'accord.

00:55:36
*Femme interviewée:* Après, on peut faire, moi j'aime bien cuit, alors ça arrive qu'on fasse une tarte aux pommes ou un gâteau aux pommes.

00:55:42
*Homme interviewé:* C'est bon aussi.

00:55:42
*Intervieweuse:* Ou un flanc aux pommes.

00:55:47
*Intervieweuse:* Oui, ok.

00:55:51
*Homme interviewé:* En même temps les pommes, enfin, on prend pas dans les- beaucoup de différentes variétés à consommer comme ça.

00:55:59
*Intervieweuse:* Oui.

00:55:59
*Femme interviewée:* À consommer comme ça, tu prends de la belle chaire essentiellement.

00:56:00
*Homme interviewé:* Oui, je prends de la belle chaire, parce que je n'arrive pas à trouver autre chose que ça.

00:56:04
*Femme interviewée:* [inaudible 00:56:04] et moi quand je veux les cuisiner, je vais prendre autre chose, je vais prendre des...

00:56:09
*Homme interviewé:* Des boscop.

00:56:10
*Femme interviewée:* Des boscop ou les faux Canada. C'est celles qui ont un petit goût acidulé à la cuisson, donc, voilà.

00:56:20
*Intervieweuse:* Vous allez pas prendre la même pomme selon la préparation que vous voulez faire avec.

00:56:23
*Femme interviewée:* Non.

00:56:23
*Intervieweuse:* L'utilisation. Et est-ce que la pomme, comment, comment vous la consommez donc crue ? Comment on fait, vous croquez dedans, vous la couper, vous l'épluchez ?

00:56:35
*Homme interviewé:* Non, je l'épluche.

00:56:35
*Intervieweuse:* Vous l'épluchez.

00:56:35
*Homme interviewé:* Je l'épluche avec un appareil magique.

00:56:37
*Femme interviewée:* Un éplucheur.

00:56:42
*Homme interviewé:* Superbe, ça prend trois secondes pour éplucher une pomme.

00:56:46
*Intervieweuse:* Oui, ok.

00:56:50
*Homme interviewé:* Non, je n'aime pas du tout la peau de la pomme qui se coince entre les dents, c'est absolument pénible. Ça me gâche tout le plaisir, donc [euh], et puis-

00:57:04
*Femme interviewée:* Depuis qu'on a découvert cet appareil-là, il y a cinq, six ans-

00:57:08
*Homme interviewé:* Oui, oui, c'est, je mange plus de pommes. Et je sais pas trop ce qu'il y a sur la peau, comme ça.

00:57:14
*Femme interviewée:* Comme les prend pas bio, de toute façon c'est bien de les éplucher.

00:57:17
*Intervieweuse:* Oui. Ok, si on passe aux légumes, si on prend un légume que vous avez dit consommer régulièrement, ben l'aubergine. Qu'est-ce que vous, comment vous la consommer ? Sous quelle forme vous la consommer l'aubergine ?

00:57:40
*Femme interviewée:* Sous forme cuite avec souvent de la sauce tomate.

00:57:44
*Intervieweuse:* D'accord.

00:57:45
*Femme interviewée:* Alors l'aubergine, je ne sais pas pourquoi, mais on l'épluche, moi je l'épluche, je coupe en cubes et je ne sais pas pourquoi, c'est un légume riche en eau, comme tous les légumes, mais qui ne cuit que si on ajoute un liquide.

00:58:00
*Intervieweuse:* Oui.

00:58:00
*Femme interviewée:* Alors soit de l'eau, soit de la tomate. Mais il faut du liquide pour qu'elles arrivent à cuire.

00:58:06
*Intervieweuse:* Oui.

00:58:07
*Homme interviewé:* Ça doit être un bon isolant parce que c'est très, elle est spongieuse.

00:58:12
*Femme interviewée:* Donc c'est essentiellement comme ça, à la poêle, dans une grande poêle avec de la sauce tomate. Et puis après assaisonné suivant ce qu'on a envie d'en faire quoi, soit un peu de crème, un peu de lait de coco. Du curry ou pas, enfin. Mais la base, ça va être ça, aubergine, coupée en dés, cuite avec de la sauce tomate.

00:58:29
*Intervieweuse:* Ok.

00:58:29
*Femme interviewée:* Pas de la tomate, fraîche pour le coup, là je prends de la sauce tomate, enfin pas de la sauce tomate, des tomates en conserve.

00:58:39
*Intervieweuse:* D'accord.

00:58:41
*Femme interviewée:* Éventuellement, après, je rajoute une sauce tomate arabiata, etc. Pour donner le goût.

00:58:47
*Intervieweuse:* Ok. Ça marche. La courgette, comment vous... ?

00:58:57
*Femme interviewée:* La courgette, je la cuis, j'épluche pas, je lave bien. Même si c'est pas bio, je, j'épluche pas.

00:59:03
*Intervieweuse:* D'accord.

00:59:05
*Femme interviewée:* Je lave bien, je coupe en rondelles, je cuis cinq minutes à l'autocuiseur.

00:59:10
*Intervieweuse:* Ok.

00:59:12
*Femme interviewée:* Et après à la poêle avec ben, je, toujours sans huile, je ne mets jamais d'huile pour les cuissons, sauf un petit peu sur la tomate.

00:59:22
*Intervieweuse:* D'accord.

00:59:22
*Femme interviewée:* Mais pas autrement. Alors là, c'est pareil. Soit, souvent, c'est de la tomate, de la sauce tomate qui va quand même donner-

00:59:30
*Homme interviewé:* Un peu de fines herbes.

00:59:31
*Femme interviewée:* Oui, du thym, bien sûr, ail, etc. Après c'est soit basilic, ce qu'on dans le congélateur, par exemple basilic. Puis là pareil, une sauce tomate cuisinée pour donner du goût, éventuellement après.

00:59:51
*Intervieweuse:* Donc finalement, vous vous aimez bien les légumes cuits, un peu poêlés ou cuits assez, avec une sauce et des herbes qui viennent.

01:00:02
*Homme interviewé:* Oui.

01:00:02
*Intervieweuse:* C'est plutôt comme ça que vous allez cuisiner vos-

01:00:04
*Femme interviewée:* Oui, alors après, ça peut être aussi, enfin que, c'est rare quand même, je pense au gratin. Parfois le brocoli, par exemple, ça arrive que l'on puisse le faire en, avec du fromage, alors pas forcément du, du comté, mais ça peut être des tranches de, de quoi, de fromage à raclette, ou- Et un peu de crème, au four quoi par exemple.

01:00:34
*Homme interviewé:* Les courgettes, on a fait des ventrées de soupe à la courgette, à une époque.

01:00:36
*Femme interviewée:* Oui, ça on en a trop mangé, moi je suis dégoûtée.

01:00:38
*Homme interviewé:* On en a trop mangé, mais c'est excellent quoi.

01:00:41
*Intervieweuse:* La soupe à la courgette ?

01:00:45
*Homme interviewé:* Courgette, sel, rien d'autre pratiquement.

01:00:45
*Femme interviewée:* On a fait ça, oui.

01:00:47
*Intervieweuse:* Et l'hiver, vous faites pas mal de soupes aussi ?

01:00:50
*Homme interviewé:* De potages, oui.

01:00:51
*Femme interviewée:* Oui, enfin moins cette année, parce que toi, tu disais que tu avais du mal à-

01:00:55
*Homme interviewé:* Oui, ça me gave un peu, mais bon peut-être que je me tasse et que j'en prenne un peu moins.

01:01:01
*Intervieweuse:* [rire]

01:01:04
*Homme interviewé:* Mais bon, mais, j'aime ça, c'est bien pourtant.

01:01:10
*Intervieweuse:* Ok et en termes d'épluchures, comment vous, des fois vous décidez que vous éplucher, des fois vous épluchez pas, c'est comment ? Qu'est-ce qui vient régir un peu ces choix-là ? S'il y a des, des fois il y a pas de choses qui viennent régir.

01:01:23
*Femme interviewée:* Ben il y a des choses qui sont nécessairement à éplucher. Pour moi en tout cas, l'aubergine, par exemple, s'épluche, les carottes-

01:01:31
*Homme interviewé:* Potiron, potimarron, etc. Ça s'épluche.

01:01:32
*Femme interviewée:* Après des légumes qui pourraient être épluchés et que je ne fais pas, c'est la courgette.

01:01:35
*Intervieweuse:* D'accord.

01:01:36
*Femme interviewée:* Parce que je trouve que la peau, [euh]-

01:01:39
*Homme interviewé:* Cuit bien.

01:01:41
*Femme interviewée:* Enfin comment dire. Enfin contribue à la préparation finale, sinon ça ferait un peu purée.

01:01:47
*Intervieweuse:* Ouais.

01:01:48
*Femme interviewée:* Alors qu'est-ce qu'on épluche pas ?

01:01:54
*Homme interviewé:* Les potimarrons pas systématiquement.

01:01:56
*Femme interviewée:* Oh quand même si, la peau donner un aspect-

01:01:58
*Homme interviewé:* Elle est un peu farineuse, oui.

01:01:58
*Femme interviewée:* La peau donne un aspect farineux que t'aimes pas trop. Du coup, on épluche le potimarron aussi. En fruits, quand on mange des poires, toi tu l'épluches, pas moi. Par contre je lave.

01:02:10
*Intervieweuse:* D'accord.

01:02:12
*Homme interviewé:* J'épluche grossièrement.

01:02:15
*Intervieweuse:* Oui.

01:02:15
*Femme interviewée:* De toute façon, tous les fruits, alors tous les fruits pas forcément, par exemple les pêches plates, je les ai pas lavées, elles ne sont pas bio, mais je ne les ai pas lavées.

01:02:23
*Intervieweuse:* Et pourquoi du coup, c'est... ?

01:02:25
*Femme interviewée:* Je ne sais pas. J'ai l'impression que si je les lave, enfin je pourrais les laver juste avant de consommer, mais c'est de la flemme plutôt. C'est plus ça.

01:02:37
*Intervieweuse:* En général, vous lavez ou épluchez, mais si des fois ça déroge la règle, c'est pas-

01:02:41
*Femme interviewée:* Oui, on se dit qu'à notre âge, on a mangé assez de pesticides sans, alors qu'on ne faisait pas gaffe, pour que ce soit pas un gros problème si on continue à pas faire trop attention maintenant.

01:03:00
*Intervieweuse:* Ok.

01:03:00
*Femme interviewée:* Par contre je conçois que pour les enfants qon fasse attention, pour les petits-enfants, c'est sûr que leurs parents font attention et nous aussi quand ils sont là.

01:03:05
*Homme interviewé:* Oui.

01:03:10
*Intervieweuse:* D'accord. Le poireau vous avez dit que vous le consommiez pas énormément, mais quand vous en consommez, qu'est-ce que, comment vous le préparez, le poireau ?

01:03:23
*Femme interviewée:* Alors, si, qu'est-ce qu'on fait avec du poireau ? Moi j'en mets un dans le couscous, mais c'est quand même minime, ça va être un poireau.

01:03:31
*Intervieweuse:* D'accord.

01:03:31
*Femme interviewée:* Qu'est-ce qu'on peut faire d'autre ?

01:03:32
*Homme interviewé:* On a fait des tartes au poireau, mais-

01:03:35
*Femme interviewée:* Des tartes aux poireaux ?

01:03:36
*Homme interviewé:* Oui, on en a fait quelques-unes.

01:03:38
*Femme interviewée:* Ah oui, c'est vrai, j'avais oublié. Oui, comme des tartes, on ne consomme pas très souvent quand même, pour des-

01:03:44
*Intervieweuse:* Oui, avec la pâte.

01:03:45
*Femme interviewée:* Oui, voilà. Donc, ça peut être, c'est pour ça qu'on en consomme assez peu, parce que toi, bon, toi je sais que tu aimerais bien manger des poireaux cuits à la vapeur-

01:03:55
*Homme interviewé:* Oui, en vinaigrette, froid.

01:03:56
*Femme interviewée:* Ou en vinaigrette. On peut en faire hein.

01:03:56
*Homme interviewé:* Comme les haricots verts d'ailleurs, très bon. Mais bon, on ne le fait pas, c'est des habitudes.

01:04:04
*Femme interviewée:* C'est moi qui pourrais-

01:04:05
*Homme interviewé:* Moi je ne suis pas du coin. Je suis solognose. Donc on faisait beaucoup de préparations froides, de légumes.

01:04:11
*Femme interviewée:* En vinaigrette.

01:04:14
*Homme interviewé:* De légumes cuits en vinaigrette. Souvenirs d'enfance [rire].

01:04:23
*Femme interviewée:* Non, c'est vrai qu'on en fait pas, parce que moi, je n'y pense pas, parce que je n'aime pas ça.

01:04:27
*Homme interviewé:* Ce n'est pas grave. Il y a plein d'autres choses. C'est pas...

01:04:32
*Intervieweuse:* Vous en mettez dans la soupe ou pas du tout des poireaux ?

01:04:34
*Femme interviewée:* Ça dépend des soupes, si c'est une soupe tous légumes, oui, si c'est une soupe-

01:04:39
*Homme interviewé:* Si c'est que une soupe au potiron-

01:04:42
*Femme interviewée:* Si c'est une soupe à la carotte-

01:04:43
*Homme interviewé:* Là non.

01:04:44
*Femme interviewée:* Moi je, à part la soupe tous légumes, on aime quand même mieux les soupes avec un seul légume.

01:04:50
*Intervieweuse:* D'accord.

01:04:53
*Femme interviewée:* Histoire de ne pas gâcher-

01:04:53
*Homme interviewé:* De ne pas noyer le goût de l'ingrédient quoi, de garder la spécificité du légume.

01:05:05
*Intervieweuse:* Oui, ok. Et quand, le poireau si vous le préparez, est-ce que, qu'est-ce que vous gardez ? Qu'est-ce que vous enlevez dans le poireau ? Est-ce que vous gardez tout ? Est-ce que non, vous enlevez-

01:05:14
*Femme interviewée:* On retire le minimum de feuilles vertes, un petit peu parce que c'est quand même nécessaire dans les bouts, après-

01:05:20
*Homme interviewé:* Un maximum, on garde le maximum de choses.

01:05:24
*Femme interviewée:* Et éventuellement, si c'est nécessaire, les premières feuilles, si elles sont, si ça mérite. Après par contre, on le coupe en deux-

01:05:34
*Homme interviewé:* Dans la longueur, on fait dans les deux sens. On le fend en deux et on le coupe en deux.

01:05:39
*Femme interviewée:* Et après on le lave par contre de façon très très soigneuse par contre, sous l'eau qui court encore, enfin l'eau qui coule. Parce que c'est quand même souvent-

01:05:50
*Homme interviewé:* Terreux.

01:05:51
*Femme interviewée:* -terreux.

01:05:52
*Intervieweuse:* Ok, ça marche, donc vous essayez aussi de ne pas enlever trop non plus de vert.

01:05:58
*Homme interviewé:* Non parce que le vert a vraiment un goût différent, enfin c'est, il participe, au goût du poireau.

01:06:07
*Intervieweuse:* Ok, alors là, si on discute sur le moment où il vous arrive de jeter un fruit ou un légume ou une partie seulement. Quand ça arrive, pourquoi décidez-vous de le jeter plutôt que de consommer ?

01:06:21
*Femme interviewée:* Ben quand c'est inconsommable, enfin c'est plus comestible. [sonnerie téléphone] Tu dois prendre ?

01:06:26
*Homme interviewé:* Oui.

01:06:26
*Femme interviewée:* Si c'est [Prénom], tu lui dis qu'on rappelle après.

01:06:30
*Intervieweuse:* Est qu'est-ce qui vous fait déterminer que c'est inconsommable et pas comestible ?

01:06:35
*Femme interviewée:* Quand c'est pourri, moisi, par exemple. Ça, c'est sûr que non. Pourri, moisi je jette, enfin je jette la partie pourrie, moisie.

01:06:49
*Intervieweuse:* Oui.

01:06:50
*Femme interviewée:* Quand c'est absolument pas mûr comme c'est arrivé parfois là sur deux tomates vertes, j'ai jeté aussi. Par contre, si la banane, si nous en reste, ça peut arriver quand ça brunit qu'on mange quand même. Parce que là, le goût d'une banane brunie, ce n'est pas pourri, moisi, mais ça pourrait, ça pourrait être jeté, mais là, on mange.

01:07:15
*Intervieweuse:* D'accord.

01:07:16
*Femme interviewée:* Après- Pourquoi on jette ? Pourri, moisi, voilà ça c'est sûr.

01:07:25
*Intervieweuse:* Et par contre, ça dépend des fruits et légumes. Il y a des fruits comme la banane où on peut accepter une maturité avancée.

01:07:34
*Femme interviewée:* Oui, alors par exemple l'ananas, c'est le cas aussi. Ça peut arriver quand vous coupez l'ananas qu'il y ait une partie-

01:07:38
*Homme interviewé:* Un peu marron.

01:07:39
*Femme interviewée:* Une extrémité du fruit un peu marron, mais tout à fait comestible.

01:07:44
*Intervieweuse:* D'accord.

01:07:44
*Femme interviewée:* Ça ne change pas le goût. Donc, ça on consomme. Faut vraiment que ce soit.

01:07:48
*Intervieweuse:* C'est le goût qui va venir déterminer si-

01:07:51
*Homme interviewé:* Oui, là vous parlez de la pourriture. Ça dépend en fait selon les fruits. Par exemple, une poire qui commence à pourrir, tout le fruit est, tout le fruit est envahi par le goût de pourriture.

01:08:03
*Intervieweuse:* Et c'est, est-ce que vous arriveriez à décrire ce goût ou pas de moisi ?

01:08:09
*Homme interviewé:* Ah ben oui, c'est le goût de moisi.

01:08:12
*Femme interviewée:* Vous n'avez jamais goûté de moisi ? [rire]

01:08:12
*Intervieweuse:* On essaye de trouver quel goût ça a, mais c'est très dur à décrire, le goût du moisi.

01:08:20
*Homme interviewé:* Ben oui, ça entre, ah. La pourriture en sous-bois, quand on perçoit parfois tous les déchets qui sont en décomposition à cause des champignons, ben c'est ça en fait.

01:08:41
*Femme interviewée:* Ce qui est acceptable quand on se promène en sous-bois, dans la bouche ça ne va plus quoi.

01:08:44
*Homme interviewé:* Oui, mais c'est reconnaissable. Et bon c'est comme les fromages, les fromages puent, mais c'est acceptable quoi. Mais si on rencontre cet odeur par ailleurs, alerte. Là, c'est l'inverse, dans un fruit, c'est inacceptable. Et vraiment, j'ai eu le cas l'autre jour d'une qui, ça a migré, ça avait migré dedans. Je me demande ce que j'ai fait de ça. On a dû le mettre [inaudible 01:09:08].

01:09:12
*Homme interviewé:* Sans doute.

01:09:12
*Homme interviewé:* On a dû le mettre dans le [inaudible 01:09:11].

01:09:12
*Femme interviewée:* À moins que ce soit avant.

01:09:15
*Homme interviewé:* [euh]

01:09:15
*Femme interviewée:* Après, ça arrive parfois les tomates, si je prends pas garde par exemple, qu'il y ait une partie qui soit- Alors ce n'est pas moisi, pourri là, c'est très mou, c'est comment dire, c'est très mou.

01:09:25
*Homme interviewé:* Juste la phase avant.

01:09:27
*Femme interviewée:* Donc là, je retire cette partie-là. Si c'est vraiment, ben si c'est pour être mangé cru, je vais la retirer, c'est sûr, si c'est pour être cuit, là, je juge en fonction.

01:09:38
*Homme interviewé:* Ça va être cuit.

01:09:41
*Intervieweuse:* Vous allez avoir tendance à être un peu plus exigeant sur-

01:09:44
*Femme interviewée:* Quand c'est cru.

01:09:49
*Homme interviewé:* En partant du principe que la cuisson va de toute façon-

01:09:52
*Femme interviewée:* Arrêter-

01:09:54
*Homme interviewé:* Bloquer les germes, détruire l'essentiel de ce qui est indésirable.

01:10:03
*Femme interviewée:* Alors là ce qu'on a jeté, par exemple, depuis qu'on a la poubelle connectée, j'ai pas fait après, mais début on notait.

01:10:24
*Homme interviewé:* La moisissure, selon le produit, c'est très variable, ce n'est pas les fruits et légumes là, mais si on prend le cas des fromages par exemple. Si on laisse trop longtemps, ça finit par envahir le fromage, mais au début, une moisissure étrangère au fromages, elle reste en surface. Clairement, ça modifie le goût du fromage.

01:10:49
*Intervieweuse:* Quand il-

01:10:51
*Homme interviewé:* Et de manière, enfin c'est pas normal, parce qu'on ne reconnait plus le fromage en question.

01:10:57
*Intervieweuse:* Oui.

01:10:58
*Homme interviewé:* Et donc là, ça ne va pas. On peut enlever une fine pellicule, plus ou moins fine d'ailleurs, selon la gravité de l'invasion. Mais ça met longtemps à progresser, parce que dans les pommes et les- enfin j'ai l'expérience des pommes et des poires, parce que je mange des pommes et des poires, c'est vraiment, surtout la poire, ça migre très vite, très vite, partout, dans l'ensemble du fruit.

01:11:31
*Femme interviewée:* Là par exemple-

01:11:32
*Homme interviewé:* Le goût et l'odeur.

01:11:33
*Intervieweuse:* Le goût et l'odeur.

01:11:34
*Femme interviewée:* J'ai fait jusqu'au trois juin. On a jeté une partie abîmée de côte de bettes qu'on voyait pas à l'achat, elle était marron.

01:11:43
*Intervieweuse:* D'accord, oui.

01:11:43
*Femme interviewée:* Donc pas envie de consommer, même cuit. On a jeté une partie de mandarine âbimée. Ça se voyait pas, ça se voyait pas du tout avec l'écorce, mais l'écorce retirée, c'était abîmé, c'était un peu pourri, mou, etc. Donc pas envie de consommer ça.

01:12:02
*Intervieweuse:* D'accord.

01:12:02
*Femme interviewée:* Un sixième de pomme pourrie. Là c'est pareil. On a jeté deux fois des tomates vertes pas assez mures, une fois une tomate verte trop mure. Trop mure, c'est à dire molle, pas envie de consommer cru ça. C'est mou, c'est une purée, pas envie.

01:12:16
*Homme interviewé:* Elle avait perdu sa fermeté.

01:12:20
*Femme interviewée:* On a jeté une pomme de terre dans le sac précédent. Je vous disais que les pommes de terre qu'on achetait étaient toujours bonnes, sauf dans le sac précédent. Il y avait quelques pommes de terre qui restaient dures à la cuisson.

01:12:31
*Intervieweuse:* D'accord.

01:12:32
*Femme interviewée:* Bon, c'était pas pourri, c'était pas- Mais la texture était trop désagréable.

01:12:37
*Intervieweuse:* D'accord. Et qu'est-ce qui fait, quand, quand la, donc vous dites quand, la tomate ou quand c'est trop mou, vous allez la jeter si c'est pour consommer cru, vous allez vouloir jeter cette partie molle que vous vouliez pas avoir crue. Il y a aussi eu les tomates qui n'étaient pas assez mures. Et là, du coup, qu'est-ce que c'est, est-ce que c'est toujours la texture qui va venir vous-

01:13:04
*Femme interviewée:* D'abord, on suppose qu'elle va pas être bonne-

01:13:06
*Homme interviewé:* Oui, le gôut va être-

01:13:06
*Femme interviewée:* -de goût.

01:13:06
*Intervieweuse:* Oui.

01:13:09
*Femme interviewée:* Et la texture va être désagréable aussi. Après, même si on la coupe, j'imagine-

01:13:14
*Homme interviewé:* Ça c'est dans la mesure où c'est à manger cru.

01:13:17
*Femme interviewée:* Oui.

01:13:18
*Homme interviewé:* La tomate verte-

01:13:18
*Femme interviewée:* La tomate verte, on l'a jamais, on a jamais essayé. Peut-être que ça se cuit, je sais pas.

01:13:22
*Homme interviewé:* Sûrement.

01:13:24
*Femme interviewée:* C'est vrai qu'on aurait pu la garder pour la mettre avec des tomates v-, des tomates rouges à cuire. Mais bon, le fait est qu'on a pas fait ça.

01:13:31
*Intervieweuse:* C'est vrai qu'on, c'est ce qu'on disait tout à l'heure, à être plus exigeant si c'est prévu pour que ça soit cru.

01:13:40
*Homme interviewé:* Si on avait réalisé qu'elles n'étaient pas mûres, elles auraient pu être intégrées à une cuisson.

01:13:45
*Femme interviewée:* Oui, mais je n'ai pas pensé.

01:13:47
*Homme interviewé:* Elle était découpée, bon. Tant pis.

01:13:54
*Intervieweuse:* D'accord. [auh] alors, là, si l'on s'intéresse au gaspillage alimentaire, de façon assez générale. Quand je vous dis : "gaspillage alimentaire", quels sont les mots qui vous viennent comme ça spontanément à l'esprit ?

01:14:11
*Femme interviewée:* Pour nous ou de façon générale ?

01:14:13
*Intervieweuse:* Ce qui vous vient, quand je dis "gaspillage alimentaire", qu'est-ce qui vous, qu'est-ce que ça vous évoque ?

01:14:18
*Femme interviewée:* Les achats qu'on peut faire en trop sans avoir réfléchi à ce qu'on allait consommer et qu'on se retrouve à, enfin pas forcément nous, mais, et qu'on se retrouve à devoir jeter. Après ce qu'on entend à la radio, les gens qui doivent jeter des- ah oui, des aliments avec des dates soi-disant de consommation limite alors que souvent, bon ça dépend ce qu'on achète, si, c'est de la viande, non je ne vais pas au-delà de la date limite de consommation-

01:14:53
*Homme interviewé:* Mais pour les laitages-

01:14:53
*Femme interviewée:* Les poissons non plus, mais les laitages, par exemple, on peut les garder plus longtemps que ce qui est indiqué sur la boîte. Donc, c'est ça, c'est des aliments qu'on peut jeter trop tôt, alors qu'on pourrait les consommer. Certains quoi je dis pas, pas tous, mais en particulier les laitages. Ou alors les produits secs genre pâtes, riz et tout ça, que si on se fie à la date limite de consommation, on pourrait jeter alors que ça pourrait être conservé. Après, gaspillage alimentaire, il y en a dans les cantines, dans les restaurants.

01:15:33
*Homme interviewé:* Moi je pense à quand on a les petits enfants, c'est-

01:15:36
*Femme interviewée:* Ah oui.

01:15:40
*Homme interviewé:* C'est une cata.

01:15:41
*Intervieweuse:* Ah oui ?

01:15:41
*Femme interviewée:* Ah oui, ça c'est vrai.

01:15:41
*Homme interviewé:* Ça on a aucune maitrise, et c'est-

01:15:41
*Femme interviewée:* On sert une assiette et aussi bien-

01:15:43
*Homme interviewé:* On a cent pour cent de gaspillage parfois.

01:15:45
*Femme interviewée:* Oui, ça peut aller à la poubelle directement.

01:15:46
*Homme interviewé:* Oui.

01:15:46
*Femme interviewée:* Oui, ça, c'est du gaspillage alimentaire qu'on pratique. Enfin, dont on est spectateurs en tout cas, ou acteurs, je ne sais pas. Ils peuvent dire :"Oui, ça, j'en veux, j'en veux." Et puis, quand c'est dans l'assiette ah ben non.

01:16:04
*Homme interviewé:* Pas toucher une seule bouchée.

01:16:06
*Femme interviewée:* Et là y a rien à faire. Enfin en tout cas nous on sait pas faire.

01:16:09
*Intervieweuse:* Donc oui, quand vous faites la cuisine pour les petits-enfants, ça arrive souvent que les restes partent.

01:16:14
*Femme interviewée:* Oui.

01:16:16
*Intervieweuse:* Qu'il y ait des restes d'assiette-

01:16:16
*Homme interviewé:* Moi je suis scié, vraiment. [rire]

01:16:19
*Femme interviewée:* Oui, mais qu'est-ce qu'on peut faire d'autre ?

01:16:21
*Homme interviewé:* Ben je sais pas.

01:16:22
*Femme interviewée:* Quand l'enfant dit : "Je veux pas."

01:16:23
*Homme interviewé:* Je sais pas, mais je me dis que dans les familles qui ont pas de fric, franchement ça doit les catastropher quoi, de voir, enfin de vivre une telle situation. C'est éprouvant.

01:16:36
*Femme interviewée:* Ouais, c'est vrai, bon on a un petit-fils, par exemple, qui a, qui était assez spécialiste de ça, qui l'est toujours d'ailleurs, bon croquer une pomme et puis hop, non, terminé quoi. Hop, un bout de banane et j'en veux plus.

01:16:46
*Intervieweuse:* Oui.

01:16:49
*Femme interviewée:* Parfois on peut arriver à manger le reste, mais quand c'est servi dans l'assiette quoi, c'est un mélange, enfin un mélange, de la viande d'un côté ou du poisson à côté, des légumes, féculents et qu'il a ratatouillé et pas mangé, ben ça va à la poubelle.

01:17:06
*Intervieweuse:* Ah oui oui oui.

01:17:08
*Homme interviewé:* On va pas finir ça, ça prend la direction du compost.

01:17:12
*Femme interviewée:* Comme on nous répète qu'il ne faut pas terminer les restes de ce que les enfants ont touché à cause des- On consommait, commençait à consommer, mais bon, avec tous les virus-

01:17:23
*Homme interviewé:* Oui, les germes, les germes-

01:17:24
*Femme interviewée:* Les germes qu'ils transmettent, si on veut pas être trop avec des rhumes et des rhinopharyngites ou des gastro ça -

01:17:37
*Intervieweuse:* Oui. Donc finalement, il y a, le gaspillage alimentaire, vous avez parlé d'achats sans trop réfléchir.

01:17:46
*Femme interviewée:* Ou alors, nous ce n'est pas notre cas, mais je vois le cas d'une de nos filles, par exemple, qui fait des courses peu souvent et d'ailleurs en drive depuis que, depuis l'histoire du coronavirus. Et quand on va chez elle, parfois, on retrouve des couches de légumes achetés depuis, dont elle n'a même plus notion quoi. Elle empile dans son frigo, alors tout va au frigo chez elle. Elle empile des légumes ou d'autres choses, parfois, [inaudible 01:18:17].

01:18:18
*Intervieweuse:* Oui.

01:18:18
*Homme interviewé:* Ça peut terminer en petit jus au fond. [rire]

01:18:22
*Femme interviewée:* Et ça c'est parce que, déjà elle a pas envie de faire des courses en magasin à cause des risques sanitaires. Et puis, comme ils travaillent tous les deux, je conçois aussi que la notion de-

01:18:33
*Homme interviewé:* Un peu débordés quoi.

01:18:35
*Femme interviewée:* Des courses, pas au quotidien, mais de courses pluri-hebdomadaires est compliquée. Donc elle a tendance à acheter une fois par semaine. Et puis, du coup, elle achète trop et voilà.

01:18:48
*Homme interviewé:* Du surstockage.

01:18:53
*Femme interviewée:* Et je pense que, à mon avis, le gaspillage alimentaire de façon globale, c'est quand même restaurants, cantines, restauration scolaire, restauration collective en général, plus d'ailleurs, plus scolaires que collectif. Parce qu'en entreprises, c'est pas le cas, c'est moins le cas. Et en restaurants quoi.

01:19:11
*Intervieweuse:* Oui. Pour vous, c'est ici que ça se passe ?

01:19:13
*Femme interviewée:* Oui, j'ai peut-être tord, mais dans mon-

01:19:17
*Femme interviewée:* Dans l'imaginaire qu'on a-

01:19:19
*Homme interviewé:* Oui. Après, il y a en amont, en amont de ça il y a des trucs dans la distribution.

01:19:26
*Femme interviewée:* Oui, c'est ça tous les fruits, sous prétexte qu'ils n'ont pas le bon calibre, qui vont être jetés. C'est affreux aussi quoi.

01:19:34
*Intervieweuse:* Oui.

01:19:34
*Femme interviewée:* Les melons que-

01:19:36
*Homme interviewé:* Entre la quantité produite et la quantité qui arrive-

01:19:41
*Femme interviewée:* Sur les étals.

01:19:41
*Homme interviewé:* Qui est vendue sur les étals.

01:19:43
*Femme interviewée:* Il y a un gaspillage alimentaire énorme là je pense.

01:19:48
*Homme interviewé:* Il y a sûrement un truc là.

01:19:48
*Intervieweuse:* Oui. Vous disiez tout à l'heure que vous, je ne sais plus où c'était-

01:19:49
*Femme interviewée:* Les tomates difformes ?

01:19:53
*Intervieweuse:* Oui, voilà.

01:19:54
*Femme interviewée:* Elles arrivent quand même sur l'étal, alors que-

01:19:56
*Intervieweuse:* Et vous, vous les consommez les-

01:19:58
*Femme interviewée:* Oui ! Ben je les choisis quand même, je prends pas n'importe lesquelles, mais il n'y a pas de problème, c'est pas parce qu'elles sont difformes qu'elles sont pas bonnes.

01:20:14
*Intervieweuse:* Oui.

01:20:14
*Femme interviewée:* Et c'est, je pense c'est vrai qu'au niveau de l'agriculture, il y a, enfin de la production, il doit y avoir, peut-être en raison de normes européennes trop, trop précises, trop sévères concernant les calibres.

01:20:25
*Homme interviewé:* Et même au niveau des producteurs, si jamais ils se retrouvent avec une production un peu pléthorique au moment où les autres aussi ont cette production, ils n'arrivent pas à écouler et ça pourrit sur place et ils ne trouvent pas d'acheteurs.

01:20:39
*Femme interviewée:* Par exemple, on connait pas très bien ses terres agricoles, mais on a un cousin qui est un agriculteur du côté de Briac, qui peut se faire, parce qu'il travaille aussi avec des coopératives, dont on va lui dire par exemple de semer des petits pois. Il va le faire. Puis moment de la récolte à la coopérative va dire : "Ben non, on a assez cette année, donc on ne vend pas vos petits pois." Et voilà, on a un champ de petits pois-

01:21:02
*Intervieweuse:* Qui ne va pas trouver forcément preneur.

01:21:04
*Intervieweuse:* Qui va passer au compost.

01:21:05
*Homme interviewé:* il va l'enfuir, il va l'enfuir en engrais vert.

01:21:09
*Femme interviewée:* Bon il va en prendre un peu pour sa famille.

01:21:11
*Homme interviewé:* Oui mais voilà.

01:21:12
*Intervieweuse:* Heureusement.

01:21:12
*Femme interviewée:* Mais sur un champ c'est-

01:21:12
*Homme interviewé:* Rien à voir avec-

01:21:14
*Intervieweuse:* Oui, le fait de les vendre.

01:21:16
*Femme interviewée:* Donc là aussi, c'est du gaspillage à la production, mais, là, c'est un cas qu'on connait, mais je pense que ça doit être multiplié par autant d'agriculteurs en France.

01:21:24
*Intervieweuse:* Oui. Et justement, vous avez parlé de, [euh], qu'est-ce que vous avez dit là ? Quand vous parliez de quand il y a les petits-enfants que vous devez jeter, vous avez dit que c'était éprouvant pour les familles qui n'ont pas d'argent-

01:21:44
*Homme interviewé:* Oui, j'imagine.

01:21:45
*Intervieweuse:* Quel lien vous faites avec l'argent avec le gaspillage alimentaire ?

01:21:49
*Homme interviewé:* Quel lien ?

01:21:52
*Intervieweuse:* Oui.

01:21:52
*Homme interviewé:* Imaginez une famille qui est vraiment dans la dèche économiquement parlant, qui se plie en quatre pour arriver à faire manger ses gamins et lesquels gamins ne mangent pas et puis bon.

01:22:04
*Femme interviewée:* Bon, moi je ne suis pas tout à fait de ton avis, parce que je pense que dans ces cas-là, on fait en sorte de ne faire que des aliments que les enfants vont manger.

01:22:11
*Homme interviewé:* J'entends bien, mais à ce moment-là, on bascule justement dans une sorte d'alimentation très-

01:22:17
*Femme interviewée:* Ah oui, bien sûr, c'est-

01:22:18
*Homme interviewé:* Très réduite.

01:22:19
*Femme interviewée:* Ça favorise pas la divers-

01:22:20
*Homme interviewé:* Perte de richesse de la diversité, de voilà.

01:22:24
*Femme interviewée:* Et je pense que les familles font ça, effectivement, c'est des pâtes, du jambon, purée jambon et on n'achète plus de légumes et de fruits, parce qu'on n'est pas sûr que les enfants vont manger. Donc-

01:22:34
*Homme interviewé:* C'est le corolaire.

01:22:36
*Femme interviewée:* C'est une perte de chance, de diversité et d'ouverture du goût pour les enfants, perte de qualité nutritive aussi pour eux quoi. Enfin, comment dire, pertes de nutriments, de vitamines, minéraux, etc.

01:22:51
*Homme interviewé:* Perte de chance au niveau de la santé.

01:22:56
*Femme interviewée:* Oui. Parce ce que bon les nôtres, nos petits-enfants, c'est vrai qu'on peut arriver à jeter des assiettes, maintenant après on va compenser en leur offrant ce qui, ce qu'ils aiment quoi. Sur-

01:23:07
*Homme interviewé:* Quitte à ce qu'ils ne mangent pas non d'ailleurs.

01:23:09
*Femme interviewée:* Mais ça peut être un goûté. Je vois [prénom d’un petits-enfants] par exemple, qui est capable de pas manger son assiette, maintenant il va manger des carottes crues, il va manger des framboises, des fraises. On se dit qu'au niveau des fibres, il-

01:23:23
*Intervieweuse:* Va trouver son compte.

01:23:24
*Femme interviewée:* Nutriments, etc, il va y trouver son compte. Alors que c'est pas au repas. Ou on a une autre petite fille, par exemple, qui refuse tout légumes et fruits crus.

01:23:34
*Intervieweuse:* Oui.

01:23:35
*Femme interviewée:* Donc, c'est comme ça, elle veut pas. Bon, on compense, ses parents compensent avec des compotes, des purées de légumes, pas forcément au repas, ça peut être des gourdes de compote à ce moment-là au goûter. Enfin moi, ils me laissent, dans nos familles, il y a peut-être une certaine conscience de, enfin conscience, de part notre éducation, enfin éducation.

01:24:00
*Intervieweuse:* Si, bien sûr, oui.

01:24:01
*Femme interviewée:* Nos connaissances entre guillemets, une certaine connaissance de l'équilibre alimentaire je dirais qui fait qu'on est à peu, plus ou moins capables de compenser l'alimentation des petits-enfants par autre chose. Mais c'est vrai que si les gens sont, et puis on n'est pas, on n'est pas étranglés financièrement parlant. Mais dans les familles peu favorisées, c'est sûr qu'on va pas se permettre d'acheter des framboises, des cassis ou des groseilles pour-

01:24:29
*Intervieweuse:* Qu'ils ne soient pas consommés.

01:24:31
*Femme interviewée:* Oui, et puis ça coûte relativement cher quand même. S'il y a un enfant, ça va, mais s'il y en a trois ou quatre, bon.

01:24:36
*Intervieweuse:* Oui.

01:24:39
*Femme interviewée:* Et puis une méconnaissance aussi, probablement pour beaucoup de, pour certains, je vais pas dire beaucoup, je n'en sais rien, de, des notions d'équilibre alimentaire quoi. Avec des slogans "cinq fruits et légumes par jour" qui sont à mon avis complètement idiots.

01:24:53
*Homme interviewé:* Cinq groseilles ou cinq pommes ou cinq ananas.

01:24:58
*Intervieweuse:* C'est pareil oui.

01:25:01
*Femme interviewée:* Non, les gens ont des injonctions comme ça, alimentaires, "cinq fruits et légumes par jour", [pff] c'est idiot quoi.

01:25:09
*Intervieweuse:* Oui, je ne sais pas si c'est une stratégie qui a beaucoup marché. Mais il, on l'a tellement entendu que, on sait qu'on est censé manger des fruits légumes au moins.

01:25:20
*Homme interviewé:* Voilà, ça a-

01:25:20
*Intervieweuse:* Tous les jours on est supposé en manger.

01:25:20
*Femme interviewée:* Ça veut dire, non quoi.

01:25:22
*Intervieweuse:* Non, ça veut rien dire.

01:25:23
*Femme interviewée:* Et tous les gens qui conf-, qui prennent la pomme de terre qui est un légume botaniquement parlant, mais qui n'a rien d'un légume au niveau nutritif. "Oh oui, j'ai mangé des pommes de terre, tout a va bien."

01:25:32
*Intervieweuse:* J'ai mangé des frites au McDonald's, j'ai mangé-

01:25:33
*Femme interviewée:* Donc j'ai mangé des pommes de terre, donc j'ai mangé des légumes. [rires]

01:25:38
*Intervieweuse:* Alors si on essayait de faire une définition, comme si on devait écrire dans Le Petit Robert la définition du gaspillage alimentaire, qu'est-ce que vous vous y mettriez ? Gaspillage alimentaire.

01:25:53
*Femme interviewée:* Une définition ?

01:25:54
*Intervieweuse:* Ouais, vous pouvez réfléchir chacun pour la vôtre.

01:25:57
*Femme interviewée:* Gaspillage alimentaire... Pratique qui consiste, je dis comme ça.

01:26:05
*Intervieweuse:* Oui oui, allez-y.

01:26:06
*Femme interviewée:* Une pratique qui consiste à jeter des, des aliments bruts ou transformés qui pourraient être consommés.

01:26:17
*Intervieweuse:* Ok.

01:26:18
*Femme interviewée:* Ou recyclés, recyclés dans des préparations cuites. Ou donnés. Ou vendus à des prix, à bas prix.

01:26:29
*Homme interviewé:* Bon ben, pas mieux.

01:26:29
*Intervieweuse:* Ça vous ça serait votre définition aussi.

01:26:35
*Homme interviewé:* Oui [inaudible 01:26:33], c'est-

01:26:36
*Intervieweuse:* [inaudible 01:26:36] Ok. Très bien. Et quand vous, ça vous arrive de jeter un aliment, vous l'avez déjà un peu signifié avec vos petit-enfants, mais qu'est-ce que vous ressentez vous ? Qu'est-ce que vous personnellement, voilà, qu'est-ce que ça vous fait quand vous jetez ?

01:26:58
*Femme interviewée:* Un sentiment d'impuissance quoi. D'impuissance et de, de, de...

01:27:05
*Homme interviewé:* Alors je trouve pas le terme, mais ça, enfin ça me déçoit.

01:27:09
*Intervieweuse:* Ouais.

01:27:09
*Femme interviewée:* Ça te désole quoi ?

01:27:10
*Homme interviewé:* Oui, de devoir jeter.

01:27:16
*Intervieweuse:* Et qu'est-ce qui va venir, pourquoi du coup ce sentiment d'impuissance et de désolation, de déception ?

01:27:27
*Femme interviewée:* Pour moi, déjà, parce que jeter, c'est pas bien, c'est pas naturel.

01:27:30
*Homme interviewé:* C'est quelque chose qui a été produit quoi.

01:27:34
*Femme interviewée:* Après déception, parce qu'on a présenté quelque chose aux petits-enfants et puis finalement, ils aiment pas, donc déjà bon. C'est vrai qu'on leur fait pas spécialement de la cuisine, donc je vais pas dire que, je ne me suis pas pliée en quatre que pour eux quoi, c'est aussi pour tout le monde. Mais en même temps, enfin, moi, c'est mon point de vue, je veux pas faire de l'alimentation un enjeu à table.

01:27:58
*Intervieweuse:* Oui.

01:28:05
*Intervieweuse:* Donc [euh].

01:28:05
*Homme interviewé:* Une bagarre.

01:28:05
*Femme interviewée:* On veut pas, on veut pas, c'est pas grave. Là, je vois entre nos deux filles, par exemple, c'est, alors peut-être que je ne suis plus sur votre question là, mais il y a une de nos filles avec son conjoint qui sont très, qui, peut-être moins maintenant, mais qui peuvent passer une demi-heure à convaincre leurs filles, de finir leur assiette.

01:28:22
*Intervieweuse:* Oui.

01:28:23
*Homme interviewé:* Son assiette.

01:28:23
*Femme interviewée:* Son assiette, oui, ou alors de manger quatre cuillères. [Pff] Quand ils sont avec nous, j'essaye de pas m'en mêler, enfin je ne m'en mêle pas, parce que c'est, ça m'énerve. C'est leur façon de faire, mais... Le repas devient un marchandage.

01:28:40
*Intervieweuse:* Oui.

01:28:41
*Femme interviewée:* Et notre autre fille, alors par contre, c'est tout à fait l'inverse. Parce qu'elle n'a pas le temps, elle n'a pas envie de passer de l'énergie à ça. Et moi, pour moi, c'est ça aussi, enfin quand nos filles étaient petites. Je veux pas que l'alimentation, je n'ai jamais voulu que l'alimentation soit un enjeu. Que le repas soit un enjeu en tout cas.

01:29:04
*Intervieweuse:* Oui.

01:29:04
*Femme interviewée:* Ne soit pas une scène de bagarre. On mange si on a faim, si on a envie.

01:29:07
*Intervieweuse:* Oui.

01:29:08
*Femme interviewée:* Si on n'a pas faim, si on n'a pas envie, c'est pas grave. De toute façon, si l'enfant grandit normalement et grossit normalement, ni trop ni trop peu, est actif, bouge, c'est que ça va.

01:29:22
*Intervieweuse:* Oui.

01:29:24
*Homme interviewé:* Ni une infinité de négociations : "Mange au moins, mange au moins cela."

01:29:31
*Femme interviewée:* Non. Alors, c'était quoi votre question ?

01:29:33
*Intervieweuse:* Alors-

01:29:33
*Femme interviewée:* Qu'est-ce que ça fait quand on jette ?

01:29:34
*Intervieweuse:* Oui. Pourquoi ce sentiment d'impuissance, désolation, déception de jeter ?

01:29:43
*Femme interviewée:* Moi, je ne suis pas trop, toi t'es-

01:29:45
*Homme interviewé:* Moi l'image que j'ai, c'est que ce que l'on jette là, ça a été produit par quelqu'un. Moi, j'ai vu mon père trimer à faire un, mes parents vivaient en autarcie sur le plan alimentaire. Puisque qu'ils avaient la chance en fait d'avoir de l'espace pour jardiner.

01:30:05
*Intervieweuse:* Ouais.

01:30:06
*Homme interviewé:* Mais ça représentait un temps considérable, enfin, le moindre poireau, la moindre carotte. Tout, tout, tout est travail, travail, travail. Et le gaspillage, je ne parle pas particulièrement du gaspillage que font les enfants en refusant de manger quelque chose, mais le gaspillage, c'est, c'est-

01:30:27
*Femme interviewée:* C'est le mépris des gens-

01:30:28
*Homme interviewé:* Rien qu'à y penser, c'est, mais c'est épuisant, c'est de l'irrespect pour ces gens-là quoi, qui ont-

01:30:34
*Femme interviewée:* Ces gens qui ont produit.

01:30:35
*Homme interviewé:* Oui, qui on produit puis-

01:30:36
*Femme interviewée:* Qui ont transformé.

01:30:38
*Homme interviewé:* Et puis qui voient leur, leur labeur qui tourne en eau de boudin. C'est vraiment très rattaché pour moi, c'est-

01:30:52
*Femme interviewée:* C'est vrai que pour moi, c'est moins ce cas-là. Moi après je-

01:30:56
*Intervieweuse:* Pas la même expérience de, de, ça n'évoque pas forcément, du coup, les mêmes souvenirs. Mais oui.

01:31:03
*Femme interviewée:* Après, quand c'est gaspillé, alors ça dépend ce qu'on en fait. Si c'est, si ça peut être mis au compost, je me dis, après tout-

01:31:10
*Homme interviewé:* Oui, c'est un moindre mal.

01:31:11
*Femme interviewée:* Ça enrichit le compost. [rire]

01:31:12
*Homme interviewé:* ça doit ai être mis en incinérateur, donc finalement déshydraté. Et il faut mettre du fuel pour, ou je sais pas quoi du, gaz. Pour arriver à déshydrater. C'est un peu dommage quoi.

01:31:26
*Intervieweuse:* Oui, ok, vous pratiquez le compostage.

01:31:28
*Homme interviewé:* Vous savez, ce n'est pas uniquement le gaspillage alimentaire. On consomme pas mal de yaourts ou de laitage, etc. qui sont operculés avec de l'aluminium. Ça y est, maintenant on le recycle à Brest, mais pendant des années on le jetait et ça allait à l'incinérateur. C'est écologiquement une catastrophe. C'est un non, un non-sens. Et pendant des années, ça m'a turlupiné.

01:31:56
*Intervieweuse:* Oui.

01:31:57
*Homme interviewé:* Il faut cinq fois plus d'énergie pour faire de l'aluminium à partir de la bauxite, qu'à partir de l'aluminium recyclé. Cinq fois plus !

01:32:08
*Intervieweuse:* D'accord.

01:32:11
*Femme interviewée:* Ça va, depuis un an à Brest, ils prennent les-

01:32:12
*Homme interviewé:* Je suis content. [rire]

01:32:20
*Intervieweuse:* Petite victoire. Ok.

01:32:20
*Homme interviewé:* C'est la notion de perte de rendement quoi.

01:32:23
*Femme interviewée:* Dans le gaspillage alimentaire, c'est aussi les contenants quoi.

01:32:27
*Intervieweuse:* Oui.

01:32:27
*Femme interviewée:* De façon générale, c'est, et même si on n'achète pas en vrac nous...

01:32:34
*Intervieweuse:* C'est tous les déchets aussi qu'il y a autour, pas forcément alimentaire, toute la-

01:32:38
*Femme interviewée:* Par exemple-

01:32:40
*Homme interviewé:* Ben déjà, il y a l'épluchage, mais bon, c'est inéluctable. C'est inhérent à la nature du produit.

01:32:47
*Femme interviewée:* En bio en supermarché, par exemple, c'est forcément, c'est toujours en barquette, plus souvent en barquette, avec du cellophane dessus. Bon, je trouve que d'un côté, on fait, on produit bio pour, et de l'autre côté, on met des, on met des barquettes polystyrène et cellophane, bon.

01:33:04
*Intervieweuse:* Il y a pas mal de gens qui se fâchent. Des gens qui sont habitués à consommer, enfin qui souhaitent consommer bio, ils sont fâchés parce que c'est, c'est bio avec plastique ou alors pas bio, sans plastique. Il faut faire un choix.

01:33:18
*Homme interviewé:* En même temps-

01:33:18
*Femme interviewée:* Ou alors à Kerbio, au marché bio, on les trouve effectivement en vrac. [sonnerie téléphone]

01:33:22
*Intervieweuse:* Oui.

01:33:24
*Femme interviewée:* Mais c'est, Kerbio c'est hyper cher en légumes fruits. Moi je ne prends pas.

01:33:30
*Intervieweuse:* Au marché, c'est au marché que vous dites ?

01:33:32
*Femme interviewée:* Non, à Kerbio.

01:33:32
*Intervieweuse:* C'est où ça ?

01:33:35
*Femme interviewée:* Kerbio, nous on va à Europe [inaudible 01:33:37]

01:33:40
*Intervieweuse:* Ah oui oui, il y a le prix qui vient. Alors bon, eh bien, chouette. Pour terminer sur la poubelle connectée.

01:33:47
*Femme interviewée:* Je vais fermer la porte, parce que- Dis-lui de rappeler.

01:34:00
*Intervieweuse:* De toute façon là c'est, on a presque terminé, puis vous m'avez déjà en fait déjà un peu parlé de la poubelle connectée. Vous m'avez dit, vous avez le sentiment de l'utiliser assez peu, voire très peu.

01:34:10
*Femme interviewée:* Le sentiment qu'on a, oui. Je sais pas après.

01:34:13
*Homme interviewé:* D'utiliser quoi ?

01:34:14
*Femme interviewée:* La poub-, d'avoir assez peu jeté.

01:34:16
*Homme interviewé:* Oui, il me semble. Près-

01:34:18
*Femme interviewée:* Maintenant il faut voir par rapport aux autres maisons, enfin familles que vous suivez, voir si c'est plus ou moins.

01:34:23
*Intervieweuse:* Oui, je pense qu'on pourra vous le dire, on peut pas vous dire tant qu'il y a l'étude, parce que sinon on ne va pas- Déjà que la poubelle fait que peut-être on y pense plus que si elle n'était peut-être pas là. On vous dira oui, je sais pas, vous je n'ai pas regardé si, on n'a pas encore comparé les personnes.

01:34:38
*Homme interviewé:* Oui, ça n'a pas d'importance, c'est l'impression qu'on a quoi.

01:34:40
*Intervieweuse:* Oui, voilà, c'est ça qui, ça ça m'intéresse.

01:34:42
*Femme interviewée:* Vous nous direz après.

01:34:42
*Intervieweuse:* Vous m'avez déjà cité qu'est-ce que vous avez jeté. Alors, est-ce que ça vous est arrivé de vous poser des questions du style "Est-ce que ça, c'est supposé aller dedans ou pas ?" Avoir un petit doute sur... ?

01:34:56
*Femme interviewée:* Non, vous avez bien expliqué oui. Maintenant ce qu'on va faire, par contre, c'est ne pas attendre un mois pour jeter le sac quoi.

01:35:03
*Intervieweuse:* [rires] Ça c'est arrivé, parce que, alors les entretiens sont, alors moi je les ai commencés, donc au début, personne n'avait pas encore, mais là, ceux que je fais depuis une semaine, c'est alors là, le sac. Tout le monde a eu son petit jus avec la poubelle. C'est bien, on a vu les sacs du coup sont, ne résistent pas aux liquides. Une fois que c'est liquide, ça transperce.

01:35:22
*Femme interviewée:* On dirait, oui.

01:35:24
*Intervieweuse:* Oui après ça va, elle se lave bien finalement.

01:35:28
*Homme interviewé:* C'est au niveau de la souillure du fond qu'il y a un défaut.

01:35:31
*Intervieweuse:* Oui. C'était les seuls qu'on a trouvé compostables de quatre litres. Mais ils tiennent pas l'eau.

01:35:41
*Femme interviewée:* Alors j'ai une question, je me posais justement parce qu'on n'a pas d'idée. Quand je mettais quelque chose dedans, je voyais des moisissures. Mais rien, rien ne se crée en l'occurrence, il n'y a pas eu de poids supplémentaire à cause de la fermentation.

01:35:54
*Intervieweuse:* Non, j'ai posé la question, justement au concepteur, moi je n'ai pas du tout de notions de physique, j'ai demandé est-ce qu'un liquide pèse plus qu'un solide. Et il m'a dit "non non non".

01:36:02
*Femme interviewée:* Non, non, ça non. Mais est-ce que les produits de fermentation peuvent augmenter le poids ?

01:36:06
*Intervieweuse:* Non. Ah, une fois qu'il y a des trucs qui se mettent dessus. Mais de toute façon, on a le poids au moment où-

01:36:10
*Femme interviewée:* Où on le met, d'accord.

01:36:12
*Intervieweuse:* Où on le met. On a les deux en fait, à chaque fois, la poubelle elle pèse le nouvel objet.

01:36:16
*Femme interviewée:* D'accord.

01:36:16
*Intervieweuse:* Et après, on a le poids total de la, ce qui, à chaque fois fait les deux, nouveau poids plus poids total. Donc, même si ça, je pense qu'on verra une incohérence au bout d'un moment. Que tous les petits poids n'auraient pas dû faire ce poids-là. Parce que- Je pense que c'est minime après du coup, si, si la fermentation grossit ou pas, ça doit être assez, les vers et tout, peut-être que ça pèse son poids.

01:36:44
*Femme interviewée:* Normalement rien ne se crée, donc ça doit être-

01:36:46
*Intervieweuse:* Oui, parce que du coup, oui oui oui, je pense que oui, ça doit se- Oui, on devrait faire une expérience.

01:36:53
*Homme interviewé:* Enfin, dans le domaine du végétal, le végétal capte une partie de la matière, sous la forme du carbone dans l'atmosphère.

01:37:05
*Femme interviewée:* Oui, et alors ?

01:37:07
*Homme interviewé:* Eh bien il crée de la matière à partir de l'air qui, qui entoure. Enfin, ça ne doit pas être dans des proportions terribles.

01:37:13
*Homme interviewé:* Oui, je pense que-

01:37:14
*Homme interviewé:* Avec les moisissures.

01:37:15
*Intervieweuse:* Oui, on va pas être à deux grammes près. Alors du coup, vous avez changé un sac, donc là vous commencez le deuxième. Est-ce que ça vous arrivait de l'oublier, la poubelle, ce qui peut arriver des fois de travailler ses fruits et légumes et de s'être dit : "Mince, ça aurait dû aller dedans." ?

01:37:27
*Homme interviewé:* Non.

01:37:27
*Intervieweuse:* Non, vous pensez pas ?

01:37:32
*Femme interviewée:* Non, je crois qu'on a mis à chaque fois que, qu'on a trouvé quelque chose à jeter.

01:37:40
*Intervieweuse:* Ok, chouette. Eh ben, merci beaucoup. J'ai posé toutes mes questions. Est-ce qu'il y a des choses sur lesquelles vous aimeriez revenir ? Peut-être des choses que j'ai pu oublier. Parler de votre consommation de fruits et légumes, ou pas ?

01:37:53
*Femme interviewée:* Je sais pas, globalement, je crois qu'on est assez consommateurs de fruits et légumes quand même.

01:38:00
*Intervieweuse:* Oui.

01:38:00
*Femme interviewée:* Au poids, je ne saurais pas dire, mais on en mange à chaque repas. Légumes for- à chaque repas, fruits à chaque repas.

01:38:10
*Intervieweuse:* Oui.

01:38:12
*Femme interviewée:* Donc...

01:38:12
*Intervieweuse:* Vous avez une bonne consommation de fruits et légumes.

01:38:17
*Homme interviewé:* Oui.

01:38:20
*Femme interviewée:* Me semble-t-il.

01:38:22
*Intervieweuse:* Ok, très bien, merci beaucoup, ça je vais couper. Alors, j'ai deux ou trois petites choses à voir avec vous.

**Household F46, Interview 2**

*Mme.1:* Hop. Alors, du coup pour commencer, là il y a quatre illustrations je vais vous demander d'en choisir une.

*Mme.2:* Mais alors [euh], on fait ch- [euh] chacun son tour ?

*Mme.1:* Ouais, je- ouais, on peut faire comme ça enfin vous-- un des- un des deux choisit une-une photo. Et puis [euh] vous me dites spontanément ce qui vous vient à l'esprit.

*Mme.2:* Et on essaye de pas parler en même temps.

*Mme.1:* Voilà. Puis on peut [euh] commencer, puis après [euh] chacun pourra s'exprimer [euh].

*Mme.2:* Alors ça c'est un [euh]- un carré de plantes aromatiques apparemment, non pas forcément désolée-non non, mais attends je- je vais te voir après--

*Mr.3:* Ouais ouais, je vois.

*Mme.2:* Un carré potager, on va dire alors. Un carré potager et là, montre quand même dedans, ça je sais pas c'est un jardin partagé probablement. [Euh] un mur au fond ou alors c'est peut-être l'Abbaye de Daoulas. [Euh] ça me fait penser à ça.

*Mme.1:* Et qu'est-ce que ça vous évoque [euh], le potager ?

*Mme.2:* Une pratique- une pratique, alors on va dire ancestrale [euh] un peu délaissée-- [enfin] délaissé [euh] proprement de-de nos jours. J'ai l'impression d'avoir un grand- un très grand jardin pour s'auto-suffire en- en potager. Donc [euh] ça-- pour moi, ça reste du-- en ville en tout cas ça reste du domaine du loisir. Pour [euh] le plaisir de- de cueillir un peu de- de légumes ou de fruits frais [euh]. Et puis, c'est pas pour la consommation quotidienne. Pour moi, c'est ça.

*Mme.1:* Ouais.

*Mme.2:* Après en campagne, j'imagine qu'il y a des gens qui ont des grands potagers et qui peuvent s'autosuffire, mais...

*Mme.1:* Ouais

*Mme.2:* En ville, non. Donc le plaisir de toucher la terre, de toucher les plantes.

*Mme.1:* Okay.

*Mr.3:* Je peux dire moi ?

*Mme.1:* Ouais.

*Mr.3:* Moi, j'y vois un potager enfin très organisé et visiblement c'est qu'un seul coin du potager, parce qu'il y a de la vigne au fond, il y a des choux-- il y a différents choux de je ne sais pas trop ce que c'est, mais bref, c'est vraiment de- du potager, des poireaux, ici je sais pas ce que c'est au premier plan. [Euh] mais bon, voilà. Donc c'est [euh] une recherche d'autosuffisance [euh] à moins que ce soit un jardin comment dire [euh] pédagogique. Mais [euh] sinon, ça- ça sent l'autosuffisance-- [enfin] la recherche de l'autosuffisance en- en plante à consommer.

*Mme.1:* Ouais.

*Mr.3:* [Euh] bon c'est à peu près tout. [Euh] effectivement, on pourrait penser que c'est une partie de-- du Jardin de l'Abbaye chez Daoulas, mais bon vu-mais bon--

*Mme.2:* À cause du mur [hein] ?

*Mr.3:* Oui oui mais [euh]- oui puis aussi la- la forme, enfin les petits buis, tout ça. Mais bon, ça c'est-c'est un aspect un peu secondaire [euh] je pense.

*Mme.1:* Et vous ça vous évoque quoi le- le potager de manière assez générale ? Que ce soit celui-ci et là qui est- ass- effectivement c'est bien organisé, ou un potager donc en particulier ou...

*Mr.3:* [Ah ben] le- l'auto-- la recherche d'autosuffisance, c'est ça qui-qui me- me sau-saute aux yeux et le-le [euh] choix de-de- des choix de consommations [quoi]--

*Mme.1:* Oui, ouais.

*Mr.3:* Mais c'est un savoir-faire parce que... Là [euh] déjà, il y a quelques plantes. On peut imaginer qu'en dehors de la photo [euh], [euh] c'est encore plus diversifié. Donc pour chaque plante, il y a un savoir-faire nécessaire [enfin]-- c'est pas le- c'est pas le truc d'amateur.

*Mme.1:* Ouais.

*Mr.3:* C'est typiquement ce qu'on saurait pas faire.

*Mme.1:* [Rire]

*Mr.3:* Ce que ton père aurait su faire.

*Mme.1:* Oui, est-ce que vous avez des expériences vécues avec le potager, que ce soit vous ou votre entourage ? Ou est-ce que vous aviez voilà déjà des-- été dans ce style de potager ou est-ce que vous avez déjà consommé des produits [euh] ?

*Mme.2:* Ce style de potager enfin, alors je parle pour moi, non.

*Mme.1:* Ouais.

*Mme.2:* Mais c'est vrai que mon père, [euh] quand il est-- il est devenu enfin retraité même, peut-être un peu avant.

*Mme.1:* Oui.

*Mme.2:* A beaucoup, beaucoup jardiné et cultivé dans un terrain qu'ils avaient à Quimper. Avec là l'idée et ils étaient-- effectivement-- [enfin] ils produisaient beaucoup [euh] alors, des tomates en été, des har- haricots verts [euh], des pommes, des kiwis, des--

*Mr.3:* Poireaux, [euh] un peu d'asperges--

*Mme.2:* Non pas tellement de poireaux--

*Mme.2:* Asperges, ça a été une année seulement.

*Mr.3:* Oui, pas une année, quelques années--

*Mme.2:* Oui.

*Mr.3:* Parce que--

*Mme.2:* Elles étaient plus pour l'expérimentation, les asperges. Mais par contre, c'est vrai qu'elles étaient là autosuffisantes pour le coup en-en pommes, en tomates-- en tomates parce qu'ils faisaient des coulis de tomates pour tout l'hiver, en pommes avec des compotes [euh] en bocaux. Alors qu'est-ce qu'ils y a ? Des kiwis. Ça a duré longtemps. Qu'est-ce qu'il y avait ? Pas de poirier. Là, c'était l'autosuffisance. [Enfin] pour mon père, c'était un retour à la terre parce qu'il était d'origine paysanne. Et puis après, il a jamais- il a jamais travaillé dans le milieu agricole, mais pour lui c'était-- je pense qu'il avait besoin de- de retrouver un peu sa- la terre [euh], le plaisir de faire pousser de-de récolter, même s'il disait que c'était probablement-- ça revenait probablement beaucoup plus cher de faire ça que d'acheter dans les magasins, puisque bon déjà, c'était à Quimper qu'il fallait- il fallait y aller.

*Mme.1:* Oui.

*Mme.2:* Il fallait acheter les plants, les graines. Et il avait bien conscience que c'était pour le plaisir et pas pour le- les économies.

*Mme.1:* Oui.

*Mme.2:* Après dans notre entourage-- [enfin] de notre entourage, je vois des jardins partagés de- de Kerédern par exemple. Je pense à ceux-là parce qu'on a discuté un petit peu avec certains-- certaines personnes qui ont une parcelle.

*Mme.1:* Oui.

*Mme.2:* Et pour eux, c'est le plaisir aussi de pouvoir être dehors parce qu'ils sont en appartement et puis de discuter avec les voisins, puis de récolter tantôt un potimarron, tantôt [euh] quelques tomates. Mais en aucun cas c'est--pour [euh]- pour eux en tout cas c'est pas pour [euh]- pour s'autosuffire. C'est pas possible.

*Mme.1:* Oui.

*Mr.3:* Oui c'est une occasion de rencontre.

*Mme.2:* Oui, c'est plus çà, oui.

*Mr.3:* Parce que, [euh] ils n'en tirent pas grand-chose et parfois même, ils se font cueillir la- le fruit de leur culture avant de- d'en profiter.

*Mme.2:* Mais c'est vrai que récemment, par exemple, il y a-- il y a en avril dernier, quand une de nos petites filles est venue se confiner ici avec [euh] a mère, on a semé des ra--moi j'ai semé des radis avec elle.

*Mme.1:* Ouais.

*Mme.2:* Pour le plaisir. Ça- ça n'a pas marché parce qu'il faisait froid. Mais bon, mon idée c'était qu'elle voit qu'on sème les graines et puis que ça fasse des petits plants; et puis qu'au bout du compte ça faisait radis. Mais comme le mois d'avril a été frais [euh], ça- ça n'a pas poussé jusqu'aux radis [rire].

*Mr.3:* Les radis se sont pas précipitées.

*Mme.2:* C'étaient- c'est en principe des radis de 21 jours, donc ils auraient dû [euh] produire.

*Mme.1:* Ouais.

*Mme.2:* Mais [ben] non.

*Mme.1:* Ça n'a pas marché.

*Mme.2:* Ça n'a pas marché.

*Mr.3:* Pour en revenir à l'expérience passée, mon père [euh] [enfin] a toujours [euh] travaillé le jardin. Il est-- on était à la campagne, on était sur une propriété qui n'était pas la nôtre, mais sur laquelle mon père [euh] travaillait.

*Mme.1:* Oui.

*Mr.3:* [Euh] qui était--c'était une grande propriété dont il était garde-chasse sur--il y avait 400 et quelques hectares, c'est- c'est énorme. Et autour de la maison, il y avait de [bah] quoi cultiver et tout ce qui était cultivable l'était. Donc un énorme jardin potager. On on-- ce- c'était pas carré comme ça, mais [enfin] c'est des--

*Mme.2:* C'est des-- ton père faisait des lignes.

*Mme.1:* Des- des lignes?

*Mr.3:* Des lignes ouais, mail il y a pas [enfin] ça se fait beaucoup quand même ça. Il y a une ligne de poireaux, une ligne de carotte, une ligne de ceci, une ligne de cela. [Enfin] mais tout, tout ce qu'on peut imaginer, qui est- et qui poussait en Sologne [quoi]. C'est- c'était là--

*Mme.2:* C'était en Sologne.

*Mr.3:* Oui en Sologne. Aucun fruitier parce que sur--

*Mme.1:* Sologne c'est ?

*Mr.3:* [Euh] en-dessous d'Orléans.

*Mme.1:* D'accord.

*Mr.3:* Entre Orléans et Vierzon.

*Mme.2:* Ce sont des terres, juste pour situer, ce sont des terres sablonneuses.

*Mme.1:* Ouais.

*Mme.2:* Donc assez ingrates pour [euh]--

*Mr.3:* Sablonneuses [euh] sous-sol argile, donc [euh] hyper sec. Il était capable d'être inondées en quelques heures.

*Mme.1:* Ouais.

*Mr.3:* Voilà. [Euh] c'est des terres ingrates. Donc tout ne poussait pas, mais il arrivait à- à tout faire pousser quand même. [Euh] sauf les-les fruitiers, ce n'était pas la peine de [euh]--, hormis ceux qui étaient en sauvages. Les pommiers, ça marchait, quelques cerisiers, mais les pêchers jamais rien, les poiriers rien. [Euh] mais sinon après tout, melons, courges de toutes sortes, pommes de terre [euh], poireaux.

*Mme.2:* Et vous vous étiez [euh] à- à cinq, vous étiez autosuffisants.

*Mr.3:* Là, on était totalement autosuffisant à ce niveau-là.

*Mme.2:* Toute l'année [quoi].

*Mr.3:* Oui.

*Mme.2:* Une famille de cinq.

*Mme.2:* Toute l'année et donc [euh], dès la transformation en- on conserve nécessairement. Parce que quand ça produit, ça produit énormément trop.

*Mme.1:* Ouais.

*Mr.3:* [Euh] beaucoup plus qu'on ne peut consommer [euh] même à cinq. Et puis [euh] et c'était associé à une basse-cour qui nous- nous alimentait en volaille.

*Mme.1:* Ouais.

*Mr.3:* [Enfin] la volaille, ce n'était pas uniquement ça, mais ça n'a rien à voir avec les légumes. Enfin ça complétait le- l'autosuffisance [euh] si vous voulez. Les œufs, le- la viande. Et il y avait l'élevage de faisans [euh] pour la chasse des- des locataires de- de la propriété. Et il y a toujours des accidents en volière, les- les faisans sont relativement sauvageons [quoi], et trouvent le moyen de se tuer.

*Mme.1:* Ouais, d'accord.

*Mr.3:* [Enfin] en paniquant. Ça leur arrive et [voilà] donc ça fait de-de la viande en plus [euh] forcément, un accidenté comme ça c'est-- [voilà].

*Mme.2:* Je crois que la seule chose c'est que vous vous achetiez-- enfin, d'après ce que tu disais, c'était le pain et les produits laitiers.

*Mme.1:* Ouais.

*Mr.3:* Oui. Et puis des viandes qu'on n'avait pas sur place. Mais bon, rarement.

*Mme.1:* Oui, oui la viande rouge [euh].

*Mr.3:* Je vous ai coupé la parole.

*Mme.1:* Non mais, non non. Le-- justement, l'autosuffisance pour vous ça-- qu'est-ce que ça- qu'est-ce que ça vous évoque ? Est-ce que-- est-ce que pour vous c'est une bonne chose que-- c'est quand même aujourd'hui dépassé, [enfin] comment vous voyez, [euh] ce- la- la manière de-de s'autosuffire par le potager.

*Mr.3:* Non, c'est pas-- c'est pas-c'est une belle chose, mais c'est un boulot monstre [quoi].

*Mme.1:* Ouais.

*Mr.3:* Moi, je- je vois mon père sans cesse, sans cesse, sans cesse [quoi].

*Mme.1:* Oui.

*Mr.3:* Quand il bossait pas pour la propriété, il bossait pour- pour son jardin. Mais en même temps, il faisait une surface considérable. Mais n'empêche que [euh] il faisait les surfaces qui était quand même [euh] sans doute associée à-- au besoin.

*Mme.1:* Oui.

*Mr.3:* [Hein] [euh] il faut bien que chaque jardinier doit avoir un peu ses petites lubies, tendances. Mais non, on avait les pommes de terre [euh] pour l'hiver--

*Mme.2:* [Euh] la-- votre question, c'est l'autosuffisance aujourd'hui ?

*Mme.1:* Ouais, [enfin] le fait de produire ses propres [euh]-- il y a l'aspect loisirs, il y a aussi ceux- ceux qui [euh] par exemple dans le milieu rural qui n'arrivent plus à s'auto suffire. Est-ce que pour vous-- est-ce que se sont des-- quelque chose qui-qui vous plairait ou est-ce que non c'est pas...

*Mme.2:* Qu'il nous plairait de faire ? Si on avait les moyens ? Si on a les moyens--

*Mme.1:* Ouais oui, là [enfin]--

*Mme.2:* Personnellement, pas du tout. Je déteste jardiner. [Ben] quoique ça m'est revenu un peu plus depuis-- j'ai tellement jardiné [euh] pour mes parents que j'ai- j'ai fait un gros sevrage de dix ans.

*Mme.1:* Ouais.

*Mme.2:* Ouais, mais, mais j'aime pas.

*Mme.1:* Ouais, c'est pas mon plaisir quand même.

*Mme.2:* Avoir les mains dans la terre, me pencher, c'est-- je trouve ça hyper fatigant [euh].

*Mr.3:* Ouais. Je voulais compléter par ça.

*Mme.2:* Quand je vois le-- l'état dans lequel je récupérais mon père le soir quand je travaillais à Quimper à l'époque [euh]...

*Mme.1:* Ouais.

*Mr.3:* C'est ingrat, c'est terrible.

*Mme.1:* Ouais.

*Mr.3:* Faut- faut pas rêver. [Enfin] ceux qui rêvent de- de faire leur jardin, c'est parce qu'ils l'ont pas encore fait.

*Mme.1:* C'est qu'ils savent pas encore ce que c'est.

*Mr.3:* Ou alors [euh] [enfin]--

*Mme.2:* Pour notre auto--pour l'autosuffisance en tout cas, mais oui--

*Mme.1:* Oui.

*Mme.2:* C'est ça un boulot, [euh] monstre, fatigant [euh].

*Mme.1:* Ouais.

*Mme.2:* Et puis pas forcément-- [enfin] je sais pas maintenant--

*Mme.2:* Pas rentable.

*Mme.2:* Comment- comment- comment les gens-- pas rentable forcément. Ça- ça dépend. Après, je sais pas comment les gens cultivent aujourd'hui, mais je sais que mon père à l'époque [enfin] là je parle des années [euh], il y a quoi 75 [euh], 90 [enfin] 2000 à peu près. C'était un gros coup de produits phytosanitaires [quoi] c'est--

*Mme.1:* Ouais.

*Mme.2:* C'est pour avoir des pommes non tavelées bon.

*Mme.1:* Ouais.

*Mme.2:* Et ces pommiers une deux fois par an, etc., etc. Donc- donc c'était pas- c'étaient pas des légumes bio. Loin de là. [Donc] [euh] si en plus on va avoir des légumes sans pesticides, ça doit être un travail en-- ou alors à moins d'opter pour la permaculture, quelqu'un--

*Mr.3:* Oui. Il faut pas rêver [hein], des- des attaques de-de parasites existent.

*Mme.2:* En permaculture Il paraît que tout ça s'équilibre [euh] [enfin] bon.

*Mr.3:* Ouais, ouais.

*Mme.1:* Quand il y a des techniques ouais.

*Mr.3:* Ouais.

*Mme.1:* Et concernant les produits [euh] que vous avez pu consommer, qui étaient issus de- de- du potager, qu'est-ce que vous en pensez ? Est-ce que vous les trouvez différents de ce que vous trouvez aujourd'hui ou pas du tout ? C'est- c'est-- un légume reste un légume, comment-- qu'est-ce que vous pensez de ces produits-là ?

*Mme.2:* Moi sincèrement, je peux pas dire [euh]-- c'est pas, même là quand on me parle parfois, on a des discussions avec un de nos gendres qui ne jure que par les- le produit local. Ils habitent tou-- région toulousaine [hein]. Donc [euh] le produit local, la framboise, la- la-- cueillie sur l'arbre serait incomparable. Elles sont incomparablement meilleures que- que les autres. Nous- moi- pour moi [euh] non hors mis les tomates, c'est vrai que les tomates d'été sont quand même-- même- même si elles sont produites, tous serres ici [euh] elles sont quand même meilleures en été qu'en-- que maintenant.

*Mr.3:* Il y a eu--

*Mme.2:* Après-- alors ça-ça dépend des régions. C'est vrai que je reconnais qu'une tomate achetée en région toulousaine, en été est bien-- ou en septembre-est bien meilleure qu'une tomate d'ici. Avec plus de soleil ça je-- Je- je sais plus quelle était votre question.

*Mme.1:* Oui, c'est est-ce que vos--

*Mme.2:* Ah oui. Ça- c'est- les goûts diffèrent.

*Mme.1:* Oui. Est-ce que vous vous avez [euh]--si vous avez remarqué des différences entre un- un produit qui était issu d'un potager ou [euh] les produits que vous consommez dans le-- dans d'autres commerces ?

*Mme.2:* Non.

*Mme.1:* Non.

*Mme.2:* Non.

*Mme.1:* Pour vous, c'est le même.

*Mme.2:* Pour moi non.

*Mr.3:* Je- je crois qu'il y a eu un passage très- très mauvais il y a quelques années où vraiment des variétés de tomates ont été introduites qui devaient être résistantes, sûrement [euh] poussées vite, etc. avec beaucoup d'eau dedans. Enfin, qui étaient devenues insipides, de farineuses, dégueulasses.

*Mme.1:* Ouais.

*Mr.3:* Mais je pense que c'était des choix de variétés qui étaient [euh] faciles à industrialiser.

*Mme.1:* Oui.

*Mr.3:* [Euh] sauf qu'il y a eu une sorte de rejet et un-un retour un peu en arrière. On revient à des variétés qui elles sont un peu plus goûtées. Je pense que c'est plutôt ça. C'est les choix [euh] qui sont faits sur les variétés plus que [enfin] oui la culture [euh] semi-industrielle ou la culture [euh] artisanale. Sauf si vraiment on y fuit des-des intrants.

*Mme.2:* Par- par exemple, aussi pour en avoir discuté avec un neveu, moi je--

*Mr.3:* Ça choque.

*Mme.2:* Ça me choque pas du tout le- la culture sous serre, avec [euh] [enfin] comment dire, sous serre et en- comment on appelle ça, en-- sans substrat [quoi]. Uniquement avec-- en hydroponie. C'est ça ?

*Mr.3:* Oui, hydroponie.

*Mme.2:* Uniquement avec de l'eau et des nutriments, parce qu'après tout le fruit, le légume, s'il a sa température, sa lumière, son eau, ses légumes, son--

*Mr.3:* Ses aliments.

*Mme.2:* Ses aliments [euh], je vois pas pourquoi ça ferait un fruit ou un légume différent que les nôtres poussés en pleine terre [euh]. C'est bien mené. Je vois pas pourquoi le goût serait différent.

*Mr.3:* Après, on peut se poser la question du bilan énergétique de-de tout ça. Parce que les apports énergétiques nécessaires, est-ce qu'il y en a ? [Euh] peut-être pas.

*Mme.2:* Là vous parliez- vous parliez du goût là. C'est que je veux qu'on réponde à cette question.

*Mr.3:* Ouais.

*Mr.3:* Mais [euh] en ce qui concerne le goût, normalement-- [enfin], on peut penser que ça devrait être la même chose. Si c'est bien-- si c'est bien étudié [quoi].

*Mme.1:* Oui, vous avez pas remarqué de- par rapport aux légumes que vous pouvez avoir du potager, une grande différence, avec ce que vous pouvez consommer aujourd'hui ?

*Mme.2:* Pour moi, le plaisir quand- quand mon père faisait ses- des framboisiers ou des fraisiers, c'était d'aller les cueillir sur l'arbre.

*Mme.1:* Ouais.

*Mme.2:* Manger une framboise [euh] chaude de soleil ou [euh]--

*Mr.3:* C'est normal qu'il y ait du goût ça.

*Mme.2:* Oui non, mais c'est vrai. C'est-- une fraise qu'on cueille ju-- [euh] encore chaude de soleil. C'est- c'est vrai que c'est incomparable par rapport à--

*Mr.3:* C'est normal, elle est à 25, 30 degrés.

*Mme.2:* Oui. C'est ça, mais c'est-- après si cette même fraise reste deux heures à la maison, ça n'est pas différente de- d'une autre [quoi].

*Mme.1:* Oui.

*Mme.1:* C'est plus [euh] la pratique de faire qui va faire qu'il y a un plaisir différent de le manger, que le goût en soi du fruit ou du légume. C'est dire que le fait de- le fait de cueillir. C'est là que le côté agréable peut être supérieur au fait de- d'acheter.

*Mme.2:* Oui.

*Mme.1:* Mais le goût en soi est sensiblement le même.

*Mme.2:* Oui. Je pense.

*Mr.3:* Alors, [euh] sachant que quand même, c'est-- quelqu'un qui jardine depuis longtemps, je pense que, à un moment donné, il a ses dadas et- et il constate pour sa consommation personnelle que telle variété [euh] [ben] elle est préférée dans le cadre de sa consommation [quoi].

*Mme.1:* Oui.

*Mr.3:* Et- et du coup il va choisir ça comme semence les- les fois d'après.

*Mr.3:* Ouais.

*Mr.3:* Bon, donc il- il fait des choix-- des choix gustatifs en fonction de ce qu'il a constaté précédemment. Mais... Et donc ça le conforte dans son-- dans sa démarche.

*Mme.2:* Maintenant je--

*Mr.3:* Il fait des vrais choix.

*Mme.2:* Au niveau du goût, je dirai aussi quand même que moi je vois-- je vois pas fon--[enfin] il ne semble pas qu'il y ait des différences fondamentales entre un- un fruit ou un légume qu'on cueille dans son potager ou celui qu'on achète en supermarché ou chez un marchand de légumes, à condition qu'on respecte les saisons aussi.

*Mme.1:* D'accord.

*Mme.2:* Parce que c'est vrai qu'acheter un [euh], je sais pas [euh] bon [ben] oui, une tomate hors saison par exemple, c'est- c'est-- effectivement c'est moins bon ou acheter quoi ? Qu'est-ce qui est insipide à certaines saisons ?

*Mr.3:* [Ben], les melons.

*Mme.2:* Oui, un melon trop tôt c'est insipide. Disons que-- oui par exemple.

*Mr.3:* Oui, faut prendre ce genre d'exemple. Ou même certaines fraises poussées [euh] hâtivement.

*Mme.2:* Oui.

*Mr.3:* Qui finalement, [euh] ça a l'air un peu trop--

*Mme.2:* Ah bien aussi c'est ça, à condition de choisir en supermarché ou en marchand de légumes. Les fruits ou légumes qui nous semblent, à tort ou à raison, j'en sais rien plus- plus proche de-de la saisonnalité. Bon...

*Mme.1:* Ouais.

*Mme.2:* Mais...

*Mr.3:* Mais [euh] j'insiste encore là dans ces approvisionnement-là, on ne maîtrise pas du tout les variétés et parmi ce qui est offert en- en supermarché-- [enfin], etc. ça peut être des variétés commerciales qui ont juste un intérêt à- [euh] pour le- pour le producteur.

*Mme.1:* Oui.

*Mr.3:* Ou pour les distributeurs [euh], le- le fruit résiste bien aux chocs, etc. ou il a une belle allure, naturellement il est super rouge, il est pas bon mais il est super rouge impeccable. [Enfin] [voilà], ça peut être choisi pour des- des critères qui sont pas des critères gustatifs.

*Mme.1:* Oui.

*Mr.3:* Et donc on va quand même tomber sur des variétés carottes.

*Mme.2:* Oui, mais on les achète une fois mais pas deux.

*Mr.3:* Et puis voilà, c'est ça. On- on recommence pas.

*Mme.1:* Oui.

*Mme.2:* Mais si on a un doute [euh], on prend-- par- par exemple, je pense aux pommes en ce moment.

*Mr.3:* Oui.

*Mme.2:* On trouve pas forcément les pommes-- des variétés qu'on aime. Si- si c'est une autre on va prendre une- une- une ou deux. Puis si ça nous convient, on continue. Si on dit non, c'est pas bon.

*Mme.1:* Ouais.

*Mr.3:* Faut chercher. Faut chercher [hein].

*Mme.1:* Oui. Selon vos expériences passées vous, vous adaptez vos choix de [euh] consommation.

*Mme.2:* Oui.

*Mr.3:* Ouais.

*Mme.1:* Okay, alors si on prend une autre photo maintenant. Même- même-le même exercice me dire spontanément ce qui est vraiment [euh]--

*Mr.3:* Elles m'attirent [inaudible 00:21:06].

*Mme.1:* Ouais, ouais.

*Mr.3:* [Ah] vendeur de primeurs.

*Mme.1:* Ouais.

*Mr.3:* [Euh] t'as le droit de démarrer, [hein] ?

*Mme.2:* Mais non vas-y puisque--

*Mr.3:* Non, non, je sais pas moi.

*Mme.2:* C'est moi qui démarré la première fois.

*Mme.1:* Qu'est-ce que ça vous évoque, ce type de lieux ?

*Mr.3:* Étal de primeurs, ça me fait [euh] un peu penser à celui chez qui on va [hein] régulièrement.

*Mme.2:* C'est ce que tu vois ?

*Mr.3:* Un peu, un peu, voilà. [Euh] qu'est-ce que j'y vois ?

*Mr.3:* Son- son truc est clean [hein]. [Euh], ça- c'est- c'est on dirait que ça vient d'être installé.

*Mr.3:* Pour l'instant j'ai pas trop grand-chose à--

*Mme.1:* Ça vous évoque pas plus que ça ?

*Mr.3:* Ah bon, vraiment c'est- c'est un étal de légumes [quoi].

*Mme.1:* Ouais.

*Mr.3:* Il y en a pas beaucoup de chaque [euh], il va falloir se précipiter un peu pour acheter ou alors il en a à l'arrière-- dans l'arrière-boutique. Et puis [voilà].

*Mme.1:* OK. On voit aussi vous avec.

*Mme.2:* Alors moi, ça me fait penser effectivement à un étal-- un étal d'un tout petit supermarché de ville. Parce qu'il y a-- trop bien présenté. Il y a pas beaucoup de légumes effectivement, il y a que des légumes et on dirait-- [Euh] donc pas beaucoup de choix. C'est-à-dire que si je veux choisir [euh]-- d'ailleurs, je sais même pas si j'ai le droit de choisir moi-même là, ou si c'est lui qui va choisir, ce que je déteste.

*Mme.1:* Ouais.

*Mme.2:* Parce que si c'est- si c'est moi qui choisis, [ben] je vais être un peu limité là, donc on va avoir [euh] cinq poivrons, trois aubergines [euh].

*Mr.3:* Mais il y a bien plus de courgettes quand même.

*Mme.2:* Oui. Alors contrairement à ce que tu disais, ça reste- [enfin] pour moi ça ressemble pas du tout à--

*Mr.3:* Non. J'ai dit un peu.

*Mme.2:* Oui [enfin], je que dis que pour moi ça ressemble pas du tout justement à notre--le magasin de primeurs où on va-- enfin le primeur fruits et légumes où on va. Parce que oui, effectivement, c'est en cageots, mais il y en a beaucoup plus [quoi].

*Mr.3:* Oui, c'est vrai.

*Mme.2:* Plus de variété [enfin] là- là je- on ne peut pas juger, mais en tout cas plus de quantité, donc [euh] moyen de trier, de choisir [euh], de tâter [euh]. Et personne à nous regarder choisir.

*Mme.1:* Donc c'est pas un magasin-là qui vous donne forcément [euh] vraiment envie d'aller [euh]...

*Mme.2:* Si on est dans une ville où il y a que ça, [ben] on irait-- [enfin] dans une grande ville où il y aurait qu'une petite supérette de quartier, avec ce genre de rayons de légumes, on s'en contenterait, mais ce serait [euh], comment-comment dire, à notre corps défendant.

*Mme.1:* D'accord. C'est pas une partie d'aller [euh]--

*Mme.1:* Non.

*Mme.1:* S'approvisionner dans ce magasin.

*Mme.2:* Non. Trop, trop bien léché, trop bien présenté et trop... On met en scène des- des légumes là.

*Mme.2:* Mouais.

*Mr.3:* Ouais, c'est vrai, oui oui je suis d'accord pour ça et même il est un peu en position de gardien devant son- son étal. Je vous attends, mais pas touche ! C'est vrai.

*Mme.1:* Vous avez pas forcément d'expérience vécue avec ce type de lieux [euh] ?

*Mme.2:* [Ben] si un petit peu, ça nous est arrivé, par exemple quand on séjourne chez une de nos filles. Alors non plus maintenant. Mais quand on séjournait, quand elle habitait, pas encore là où elle habite en- à Lyon.

*Mme.1:* Oui.

*Mme.2:* [Euh] c'est arrivé effectivement d'être confronté-- aussi c'était- non, alors c'est pas- c'était quand on est allé passer un Noël à Lyon. On était dans un appartement Airbnb où la seule supérette du coin, c'était ce genre-là.

*Mme.1:* Oui.

*Mme.2:* [Ben] c'était pas top.

*Mme.1:* Okay et les produits que vous aviez consommés, c'étaient pas...

*Mme.2:* Je m'en souviens plus.

*Mme.1:* Oui. Pas de- pas de choses marquantes là-dessus.

*Mme.2:* Non, on peut pas le dire non.

*Mr.3:* Il fallait manger.

*Mme.2:* Il fallait manger, oui, je pense pas que j'aurais quoi que ce soit à leur reprocher en l'occurrence à ces- ces légumes qu'on trouvait à ce moment-là, mais [euh] c'est parce qu'il y avait pas le choix que je- j'étais allée là.

*Mme.1:* Oui. OK, alors une autre photo maintenant.

*Mme.2:* Donc ça c'est un marché, un marché couvert dirait-on.

*Mme.1:* Qu'est-ce que ça vous évoque le marché ?

*Mme.2:* [Euh] alors moi j'aime pas aller au marché, parce qu'on peut pas se servir soi-même.

*Mme.1:* D'accord.

*Mme.2:* Et là, effectivement, le monsieur a l'air-- le vendeur à l'air-- à l'air d'être derrière ça- c'est lui qui va aller-- la dame va lui demander-- la cliente va lui dire qu'elle voudrait un chou, mais c'est pas elle qui va le choisir.

*Mme.1:* Ouais.

*Mme.2:* Etc., etc. Moi j'aime bien choisir moi-même, regarder, voir et-- j'essaye maintenant de- de ne toucher que ce que je suis à peu près sûre de prendre.

*Mme.1:* Oui.

*Mme.2:* Quand même. Mais je- c'est pour ça que j'ai jamais commandé, par exemple en drive ou [euh] ou le système de commande groupé, genre [Nom de l’association] qu'il peut y avoir un Kerédern ou à Bellevue, parce que je peux pas- je vois pas.

*Mme.1:* Ouais.

*Mme.2:* Je- je commande et puis je sais qu'après que- que c'était comme ça. Et puis parfois, c'est arrivé une fois ou deux et avec des déceptions, parce que bon, sur cet aliment-là, les poireaux si j'avais su... Donc le marché [euh] non, moi j'aime pas le marché. Puis il y a trop de monde, je- enfin bref j'aime pas- j'aime pas le marché.

*Mme.1:* Ouais.

*Mr.3:* Avec aussi l'aspect un peu bruyant.

*Mme.2:* Même si là il y a même pas grand monde.

*Mr.3:* Là il y a pas grand monde, mais souvent c'est- c'est le cas quand-même à certaines heures, en tout cas.

*Mme.2:* C'est là qu'on a l'impression de se faire avoir parfois.

*Mme.1:* Ouais, c'est-à-dire ?

*Mme.2:* [Bah] comme nous on choisit pas [euh], et on ne-- [enfin] j'ai tendance à pas trop dire ce que je vois. C'est arrivé que j'aille par exemple au marché bio à Kerinou et puis je vois qu'on met une pomme un peu pourrie ou tablée et puis [ben] je dis pas--

*Mme.1:* Ouais.

*Mme.2:* Je lai- laisse faire d'ailleurs alors que je devrais dire ah vous pourriez me changer celle-là ? [Ben] non, j'ose pas le dire. Donc, je me retrouve à acheter une pomme que je sais qu'elle va pas être consommable.

*Mr.3:* Certains doivent le dire, mais du coup [euh], c'est toi qui passe derrière qui te la fait placer la pomme.

*Mme.2:* Oui et puis- puis, comme j'ose pas le dire [ben] voilà.

*Mme.1:* Donc pareil pour vous le marché, c'est pas forcément un endroit [euh] dans lequel vous allez prendre plaisir à- à faire vos courses.

*Mme.2:* Non, déjà à cause du- du monde en général, j'ai- je déteste la foule. Donc [euh]--

*Mme.1:* Oui.

*Mme.2:* Je sais pas le marché. Non c'est pas...

*Mr.3:* Je me souviens de quelques expériences qu'on a faites à Lyon là, certains matins, le dimanche matin je crois ou un truc comme ça.

*Mme.2:* Samedi matin ?

*Mr.3:* Le samedi matin où on arrive sur un marché où il y a beaucoup de monde à vendre les mêmes choses.

*Mme.1:* Oui.

*Mr.3:* Bon des carottes, il y en a partout, des- des ceci, des cela, partout.

*Mme.2:* Il faut d'abord faire le tour puis voir les prix et puis [euh].

*Mr.3:* [Euh] et en vertu de quoi, à part si on est vraiment un habitué de ce marché et qu'on a acquis un savoir-faire [quoi]. Le bouche à oreille ou je ne sais trop quoi. Là, on est à la merci de n'importe quel [euh] commerçant qui va nous placer n'importe quoi.

*Mme.1:* Oui.

*Mme.2:* Par exemple au marché bio de Kerinou [euh], nous [euh], nous on fréquente pas beaucoup, mais on a une amie qui est- qui- qui fréquente-- [enfin] qui y va et elle m'avait dit : « Pour les pommes, tu vas avoir [Prénom de la marchande] ». Je savais que [Prénom de la marchande], c'était la marchande de bonnes pommes. N'empêche que c'est elle qui m'a placé une pomme tablée, [quoi]. Bon alors peut-être que ses pommes sont bonnes, mais comme elle me connaissait pas, j'ai l'impression qu'elle a- en a profité. Donc [euh]--

*Mr.3:* Alors ça, c'est un peu le- le risque qu'il y a [enfin] dans d'autres-- c'est assez connu et je pense que c'est absolument vrai. Dans le domaine de la viande par exemple, c'est classique [quoi].

*Mme.1:* Oui.

*Mr.3:* Vous êtes pas client fréquent chez un boucher, vous vous pointez, et- et il en profite pour vous écouler le--

*Mme.1:* La partie la moins [euh]--

*Mr.3:* Voilà.

*Mme.2:* [Ben] là, on pense à un boucher en particulier.

*Mr.3:* Oui mais, tout le temps- tout le temps, ça a été comme ça. Le client il est repéré, on lui a en glisse encore plus que le besoin. Et comme ça, voilà, ça, ça y est, c'est bon, c'est écoulé. Ouf !

*Mme.1:* Oui.

*Mr.3:* [Euh] dé-dégagez le morceau [euh] merdeux.

*Mme.1:* Oui.

*Mr.3:* [Euh] et donc [euh] en légumes, ça se fait pareil [quoi]. C'est- c'est bien évident que le commerçant connaît son étal et puis [euh] aussi connait- connait ses clients habituels.

*Mme.1:* Ouais.

*Mr.3:* Voilà.

*Mme.2:* Alors le marché-- en résumé, pour moi, le marché non, parce que trop de monde et on se sert pas soi-même et on peut- et puis c'est trop long. Parce que si on connaît pas bien ce marché, il faut faire le tour des stands et puis voir qui propose quoi, à quel prix, quel est le poids et qu'est-ce qu'on va choisir finalement ? Et puis on achète parce qu'on en a marre d'être là. Et puis finalement...

*Mme.1:* Ouais. Encore si on est habitué et qu'on sait quel commerçant [euh]--

*Mme.2:* Probablement dans ce cas-là, oui.

*Mr.3:* Oui.

*Mme.1:* Dans ce cas, ça pourrait le faire. Mais sinon, quand on connait pas, c'est plus galère qu'autre chose.

*Mme.1:* OK.

*Mr.3:* Alors ça y est on a fini de casser le marché ?

*Mme.1:* Ça y est le marché c'est fait.

*Mr.3:* Pour la dernière, ah [ben] ça, c'est un étal de super- supermarché. [Hum]...

*Mme.1:* Qu'est-ce que ça vous évoque ?

*Mr.3:* [Euh] il n'y a pas grand monde--

*Mme.1:* Sur la photo ?

*Mr.3:* Oui, c'est ça.

*Mr.3:* [Euh] je vois le même carrelage que là. Non, c'est pas ça. J'en sais rien. Bon enfin là, il y a des étals en apparence bien- bien chargés. C'est- c'est super ordonné. Ça- ça ressemble pas du tout à- à comment dirais-je un vendeur de primeurs [euh] où il y a beaucoup de vrac [euh] où c'est pas axé sur la présentation. Là il y a quand-même un-un effort de présentation. Je sais- je sais pas c'est une photo prise de loin, j'ai pas-- j'ai pas grand chose à en dire. Faudrait que je passe dans les allées.

*Mme.1:* Ouais.

*Mme.2:* Pour moi, ça se ressemble à- à l'étal qu'il pourrait y avoir au Super U de Kerédern où on va souvent par exemple. Donc l'avantage pour moi là, c'est qu'on peut regarder, se servir soi-même.

*Mme.1:* Ouais.

*Mr.3:* Ça- c'est un des pin- une des principales qualités en tout cas.

*Mme.1:* Oui.

*Mme.2:* Oui de la diversité, on peut regarder, se- se servir soi-même.

*Mme.1:* Donc c'est un lieu dans lequel vous- vous pouvez aller régulièrement acheter vos--

*Mme.2:* Oui.

*Mr.3:* Ah oui, oui.

*Mme.2:* Nous en fruits et légumes, on va soit dans le Super U de Keréden, soit chez Expilon place Napoléon III là chez un marchand de primeurs, donc [euh]...

*Mme.1:* Sur les produits que vous trouvez dans- dans le Super U, vous êtes-- ça vous satisfait ? Ou des fois non ? Comment--

*Mr.3:* Ça dépend [quoi].

*Mme.1:* Ouais.

*Mme.2:* En général, si quand même.

*Mr.3:* Oui, non mais c'est de-- des fois on sait que [euh] à telle une période, par exemple certaines choses faut pas les prendre.

*Mme.1:* Ouais.

*Mme.2:* Mais c'est pour ça que souvent quand on fait les courses, on va d'abord chez notre primeur ou alors ça- ça dépend.

*Mme.1:* Oui.

*Mme.2:* On sait par expérience que, par exemple, on va pas trouver de- de pommes Chanteclerc ou Belchard à Super-- non, au contraire à...

*Mr.3:* Oui, je sais plus où.

*Mme.2:* On va pas-- si on rentrait-- on...

*Mr.3:* Chez Terre Toc ils en ont ?

*Mme.2:* Ils en ont ? Alors je sais plus maintenant c'est à Super U qu'ils en ont ? On sait qu'il y a un des deux-- suis désolée, j'ai oublié maintenant--

*Mr.3:* Cette année, les Belchards on n'en voit pas beaucoup à Super U.

*Mme.2:* Oui donc ça chez- chez Terre Toc ils en ont. Donc on va aller les prendre là. Moi, je sais que jusqu'en octobre novembre, il y avait des tomates de- de pleine terre chez les primeurs.

*Mme.1:* D'accord.

*Mme.2:* Donc on allait plutôt les prendre là. Après maintenant à cette saison-ci... on fait les deux parce qu'on va souvent le vendredi ou le samedi à Super U et puis souvent le mardi, le mercredi, je complète à- chez- chez le primeur. Et c'était quoi la question ?

*Mme.1:* Là sur les produits, est-ce que vous êtes satisfaite--

*Mme.2:* Ah de-- en supermarché ?

*Mme.1:* Oui.

*Mme.2:* Oui. Parce qu'on ne choisit que ce que-- ah non. Oui et non. Parce que l'autre jour, j'ai pris une aubergine qu'on a jetée, par exemple, la poubelle connectée à dûe être contente là.

*Mme.2:* [Rire]

*Mr.3:* Oui et non.

*Mme.2:* Mais ça aurait pu arriver chez- c'est ma faute [hein] si je prends des aubergines en janvier [euh].

*Mr.3:* Oui et après le- le problème de- de saison [quoi], il y a une offre, mais...

*Mme.2:* Elle venait d'Espagne. Alors je suis toujours mitigée par rapport à ça. Je sais que l'Espagne utilise pas mal de pesticides en même temps c'est l'Europe parce qu'ils sont quand-même contraints par les mêmes normes, donc [euh] je suis pas partagée là. Puis cette saison on a-- quand on a fait le tour des carottes, des poireaux, des choux, des navets et qu'on a envie d'un peu d'autres choses [ben] moi, je craque pour des aubergines, des courgettes encore.

*Mme.1:* Oui, l'offre d'hiver au niveau des légumes est un peu limitée.

*Mr.3:* Oui, oui. Du coup, s'il y a une offre qui est un peu hors saison, [euh] on- on risque de se faire-- [enfin] on se fait tenter [euh] et parfois à tort [euh].

*Mr.3:* Et c'est vrai qu'un légume qui est hors saison, il va avoir des problèmes pour [euh]- pour pousser et le résultat, c'est que quand on coupe...

*Mme.2:* Moi j'imagine que quand ça vient d'Espagne, c'est aussi cultivé sous serres. Même en Espagne et que...

*Mr.3:* Oui, oui, il fait pas si chaud que ça en Espagne.

*Mme.1:* Oui. Et [euh] votre primeur, en quoi il est différent de celui-là ? Qu'est-ce qui fait que le--

*Mr.3:* Ah [bah] le souk, là c'est- c'est trop bien présenté [quoi] c'est pour la photo.

*Mme.1:* D'accord.

*Mme.2:* No-notre marchand de primeurs c'est-c'est- ce genre-là. Mais en moins bien présenté.

*Mr.3:* Oui en- en moins léché.

*Mme.1:* D'accord.

*Mme.2:* Et voilà donc c'est-c'est juste une--

*Mr.3:* En vérité, ils s'en foutent [quoi] de-de la présentation. C'est-à-dire c'est vraiment le- le produit--

*Mme.2:* Oh peut-être pas quand-même, parce que ils les mettent, ils essaient de faire des pyramides.

*Mr.3:* Oui, non d'accord, ils en-- ils empilent, etc., mais c'est aussi pour gagner de la- de la place verticalement. Mais c'est quand-même le produit qui est dans le- le cageot qui...

*Mme.1:* Oui.

*Mme.2:* Et la différence aussi, c'est que je peux demander à la vendeuse ce qu'elle pense de-- par exemple quand- quand vient la saison des melons

*Mme.2:* Oui.

*Mme.2:* Je lui demande est-ce qu'ils sont bons ? Elle me dit franchement oui ou non.

*Mme.1:* Oui, OK.

*Mme.2:* Elle me dit je vous- je vous dirai quand ils viendront bons. Quand c'était le début des kakis, par exemple, en octobre [euh], je lui ai demandé : est-ce qu'ils sont bons ? Elle m'a dit non, attendez, je vous dirais.

*Mme.1:* OK.

*Mme.2:* Donc, j'ai commencé à en acheter en novembre. Ils étaient bons effectivement. Donc je peux lui demander [euh] quand- quand on voit pas de l'extérieur, si le- le légume ou le fruit, peut être bon--

*Mme.1:* Ouais.

*Mme.2:* Je sais que je peux lui demander, elle me dira.

*Mme.1:* Ouais.

*Mme.2:* Et je sais aussi que si on a pris trois melons par exemple, et qu'il y en a- malgré tout il y en a un qui était immangeable, si je viens lui dire ça le- deux jours après, elle me dit [ben] prenez-en un et vous remplacez.

*Mme.1:* Ouais. Ce que vous avez pas au-- en grande surface du coup.

*Mme.2:* Ah [ben] non, ça en surface je vais pas ramener un melon [euh]. On pourrait [hein].

*Mr.3:* Oui mais enfin, en même temps [euh], c'est-- là c'est la responsabilité aussi du choix [quoi]. On- on regarde, on choisit [euh], faut- [enfin], je trouve exagéré--

*Mme.1:* Oui.

*Mr.3:* [Euh] de ra- ramener un produit qu'on a choisi nous-même [euh].

*Mme.2:* Par exemple, l'autre jour et récemment à Super U il y avait des- je voulais prendre des clémentines puis il y avait des- ce qu'on appelle des clémenvilles qui n'étaient pas très chères. Et donc le-- celui qui met-- [enfin] il y a plusieurs personnes maintenant qui s'occupent vraiment spécifiquement du rayon fruits et légumes, donc il était à côté de moi. Je lui demande : est-ce qu'elles sont sucrées ou acides ? Il me répond [euh] : elles sont moins acides que celles-là. Puis il me fait un geste. [Bah] celles-là, lesquelles ? Ça veut dire quoi ? Mais bon, j'ai pas insisté [quoi]. Je- j'ai bien senti que c'était pas...

*Mme.1:* Ouais.

*Mme.2:* Peut-être que j'aurais pu insister, mais je l'ai pas fait.

*Mr.3:* Il était dans son rayon, mais c'était pas son rayon.

*Mme.1:* Ouais.

*Mme.2:* Alors que-- si c'était dans le magasin de primeurs, je peux lui demander et elle me dira ou il me dira si je vais le matin, parce que le matin, ce sont les gars et l'après-midi la fille. Ils diront [euh]--

*Mme.1:* Oui.

*Mme.2:* D'ailleurs, ils le marquent sur leur [euh]- sur leurs petits panneaux quand-même, où ils indiquent le prix, ils mettent extra sucrée [euh]--

*Mr.3:* D'accord.

*Mme.2:* Ils mettent pas acide mais ils mettent un peu d'acidité. Mais souvent ils- ils donnent des indications de- de goût.

*Mr.3:* D'une certaine façon, ils connaissent leurs produits.

*Mme.2:* Ah oui, oui, ils connaissent bien leurs produits, oui.

*Mr.3:* Ils ont pas l'œil sur tout parce que- mais--

*Mme.2:* Mais ils en vendent [hein] des tonnes et des tonnes. Donc [euh] ils peuvent pas être maîtres de tout, non plus.

*Mme.1:* Ouais.

*Mr.3:* Ouais puis je pense que-- quand ils ont un approvisionnement qui arrive à goûter.

*Mme.2:* Ah ils goûtent tout le temps d'ailleurs [euh].

*Mr.3:* J'imagine-- tout ce qui est rente enfin ils vont pas se manger une aubergine mais--

*Mme.1:* Ouais.

*Mme.2:* Non, mais de tout ce qui peut se manger comme ça, ils sont approvisionnés le mardi donc c'est sur-- [enfin] on évite d'aller le lundi par exemple. Parce que le lundi on n'a que les restes de la semaine précédente.

*Mme.1:* Oui.

*Mme.2:* Et le mardi si on y va le matin, parce que là ce sont-- y a- y a trois hommes qui sont le matin, dont les deux patrons.

*Mme.1:* Ouais.

*Mme.2:* Alors ils passent leur temps à prendre des fruits, des légumes, à couper des tranches, proposer aux clients de goûter, donc [euh]--

*Mme.1:* Ouais.

*Mr.3:* Donc la relation est quand même différente par rapport au supermarché. Le- le responsable de- de l'étal là à part [euh]--

*Mme.2:* Mettre en rayon--

*Mr.3:* Une démarche personnelle, il y en a pas un qui- qui les goûte.

*Mme.1:* Oui.

*Mr.3:* Je me demande dans quelle mesure. S'il se fait prendre à goûter une mandarine il se ferait pas engueuler d'ailleurs.

*Mme.2:* Non en supermarché, on ne peut pas se permettre je pense de faire goûter.

*Mr.3:* Non, non, mais à lui de savoir.

*Mme.1:* Oui, les- les personnes travaillent dans les-- c'est plus des personnes qui vont être responsables de l'approvisionnement en rayon ou qui font du rangement--

*Mr.3:* Oui, oui c'est ça le classement et puis de l'entretien--

*Mme.1:* Plutôt que de connaître les--

*Mr.3:* Des présentoirs, quoi mais...

*Mme.2:* Et là un magasin de primeurs où on va, je sais que bon le mat-- j'y vais rarement le matin, parce qu'il y a trop de monde et je préfère [euh] y aller plutôt en début d'après-midi, quand il y a moins de monde. Mais en discutant avec un des deux patrons, il me racontait par exemple, je ne sais plus quand c'était que bon, il fait des commandes et puis il anticipe. Par-- il me disait par exemple que les tomates Savéols augmentent leurs prix quand le temps va être beau. Quand il va faire le temps enfin il- la météo va être belle, le prix des tomates augmente. Donc lui aussi regarde la météo.

*Mme.1:* D'accord.

*Mme.2:* Et du coup, il commande une semaine avant.

*Mme.1:* D'accord.

*Mme.2:* En se disant bon, là il fait- [enfin] bon, il joue là-dessus [quoi].

*Mr.3:* Ou il contractualise avant que ça n'augmente.

*Mme.2:* Oui.

*Mme.1:* On n'imagine pas qu'il y ait tout ce travail derrière.

*Mme.2:* Si c'est- c'est un jeu de-- Donc il achète des- il a acheté des tomates Savéols quand- enfin quand le temps était mauvais ou quand les probabilités de temps mauvais arrivaient.

*Mme.1:* Oui, pour éviter de subir [euh]--

*Mme.2:* Oui.

*Mme.1:* Le- l'augmentation de ce--

*Mme.1:* Et [euh] en termes de perte de produit, est-ce qu'il y a une différence selon vous entre le primeur ou le supermarché ? Est-ce que ou non vous perdez autant de produits [euh] comme fruits et légumes ?

*Mme.2:* Alors je pense-- à mon avis y a pas de différence.

*Mme.1:* Ouais.

*Mr.3:* On perd quand même assez peu de toute façon parce qu'on y va souvent.

*Mme.2:* Oui, moi c'est ar-- arrivé, l'aubergine que j'ai jeté entière [euh] il y a pas longtemps. Mais à part- à part ça [euh], c'est rare que-- j'ai- j'ai bon, vous me le direz peut-être un jour, mais j'ai pas l'impression qu'on jette tant que ça de fruits et légumes. Et là, on a jet- on jette beaucoup, typiquement, c'est quand les petits enfants sont là [quoi].

*Mme.1:* Ouais.

*Mme.2:* Parce que c'est pas consommé, on met des légumes pour décorer [quoi].

*Mme.1:* Ouais. Et quand vous jetez [euh] un produit, qui vient [euh] de chez le primeur ou du supermarché, [enfin] est-ce que vous avez une différence dans le- ce que vous ressentez ou c'est la même- même émotion ?

*Mme.2:* Si on jette un produit ?

*Mme.1:* Ouais. Est-ce que vous allez avoir une...

*Mme.2:* Non, pour moi, non.

*Mme.1:* Oui.

*Mme.2:* Après quand- quand ils sont ici, je peux réfléchir en me demandant [euh] d'où ça vient ça ? Et puis--

*Mme.1:* Oui.

*Mme.2:* Non, que ça vient du supermarché ou du primeur [euh], quand c'est pas bon, c'est pas bon [quoi].

*Mme.1:* Oui.

*Mme.2:* Sauf, je vous disais peut-être que si c'est un melon entier par exemple, là je suis preneur je peux retourner le lendemain pour dire en fait celui-là [euh], il était pas bon du tout.

*Mme.1:* Ouais.

*Mme.2:* Mais bon.

*Mr.3:* La- la démarche est- est différente selon aussi [euh] le fruit ou le légume qui est en cause. [Bah] un melon, c'est- c'est quand même un gros truc quoi.

*Mme.1:* Mm.

*Mr.3:* C'est tout le melon qui est- qui est mauvais.

*Mme.1:* Ouais.

*Mr.3:* Vous achetez des pommes, bon. Il y en a une qui est un peu esquintée, mais toutes les autres, finalement, ça va. Alors la perte est moindre le-- donc le comportement qu'on a par rapport à cette perte [euh] est pas le même.

*Mme.1:* Oui.

*Mr.3:* Et puis [euh] globalement, [bah] on espérait qu'elle serait meilleure que ça, mais bon, ça passe encore. [Euh] donc [euh] on va à chaque fois avoir un critère de-- un seuil de tolérance différent. [Euh] si c'est les pommes ou les poires qu'on mange [euh] ou un gros melon [euh]--

*Mme.2:* Oui, c'est ça. Ça dépend de la quantité qu'on jette. Effectivement, c'est plus en termes de quantité qu'on-- ou le pour-- enfin, si on doit jeter, [enfin], je -je prends le-le cas du melon parce que c'est le seul [euh] donc comme tu dis, le seul fruit [euh] qui peut être mauvais en- dans sa globalité. Donc ça, c'est [euh] oui un peu gros sur le- le cœur quand même.

*Mme.1:* Oui.

*Mme.2:* Et jeter aussi ce que les petits enfants n'ont pas mangé.

*Mme.1:* Ouais.

*Mme.2:* [Euh] toi plus que moi d'ailleurs.

*Mr.3:* Ouais. Parce que c'est- il y a du genre : j'en veux, j'en veux.

*Mme.1:* Ouais.

*Mr.3:* Là donc on en met un petit peu et puis [en fait] [euh] rien quoi, ils mangent rien. Et ça part tel que--[euh]

*Mme.1:* Ben oui.

*Mr.3:* Dans la poubelle.

*Mme.1:* Oui.

*Mr.3:* Et puis ça [ben] multiplié par trois, parce qu'il y en a un qui a pas bouffé comme ça. Alors l'autre, il ne voit pas du tout pourquoi il le ferait. Et le troisième qui a envie de l'imiter, parce qu'il commence à avoir trois ans donc il est temps d'imiter les autres.

*Mme.1:* Ouais.

*Mr.3:* Pareil, sauf quand vraiment une fringale terrible et qu'elle remarque pas que les autres mangent pas.

*Mme.2:* Ça dépend de la configuration de la table.

*Mr.3:* Voilà, ça dépend aussi de la configuration--

*Mme.2:* Quand elle est là et que je suis à côté [euh] elle mange [euh].

*Mme.1:* Ouais.

*Mme.2:* Il faut les séparer.

*Mme.1:* Ou manger en bandant les yeux, chacun à son assiette.

*Mme.2:* Mais on a essayé plein de configurations de table à Noël [euh].

*Mr.3:* Je trouve dommage la perte [quoi], je trouve dommage.

*Mme.1:* Ouais.

*Mr.3:* Ça- ça représente [euh]--, ou alors c'est mâchouiller. Puis complètement...

*Mme.1:* Oui.

*Mr.3:* Ce retour de table il est inexploitable [quoi].

*Mme.2:* Je me souviens que tu disais lors du premier entretien que c'étaient des expériences d'enfance quand-même qui t'avaient marqué à ce niveau-là. Ton père qui cultivait et pour qui chaque perte était-- voilà ça te reste.

*Mr.3:* Tu manges, tu manges pas, mais tu- tu- tu mets pas dans ton assiette [euh] ce que tu vas pas bouffer.

*Mme.2:* Oui oui alors que nous, avec nos filles par exemple [enfin] moi, j'ai eu une approche complètement différente, au contraire de pas- de- pas différente, je ne sais pas. Je ne vais pas comparer, mais pour moi je voulais pas faire de l'alimentation un enjeu.

*Mme.2:* Oui.

*Mme.2:* Donc tu manges ou tu manges pas--

*Mr.3:* C'est pas le cas-- c'était pas un enjeu. C'est simplement que si- si tu dis [euh] oui [euh], j'en prends--

*Mme.2:* Il fallait manger.

*Mr.3:* Tu bouffes.

*Mme.2:* Oui mais bon, c'était aussi ta génération. Moi aussi quand j'étais petite--

*Mr.3:* C'était alors l'après guerre aussi [euh], il y avait eu toute- toute une période de privations. C'était- ça a été terrible. Bon [euh] ce que tu mettais dans ton assiette et que tu mangeais pas, tu l'enlevais de la bouche de l'autre.

*Mme.1:* Oui.

*Mr.3:* Donc voilà.

*Mme.2:* Mais c'est ça, c'est une question de génération, parce que je- bon [euh], 30 ans plus tard [euh].

*Mr.3:* [Ben] oui, bien sûr oui.

*Mme.2:* Je- je sais que j'ai- j'ai volontairement-- je voulais pas que l'aliment-- l'alimentation, ce qu'on mange, soit un enjeu, c'est-c'est pas le bon mot. Que soit un problème à tel, quand on mange, on mange pas, c'est pas grave. Si l'enfant [euh]- grandi normalement, c'est ce qu'il faut [quoi].

*Mme.2:* Et je suis un peu [euh] avec une de nos petites filles dont les parents ont insisté jusqu'à finir son assiette, quatre cuillères on négocie, non trois, deux non. Oh ça, on essaye de pas s'en mêler mais [euh]...

*Mme.1:* Oui.

*Mme.2:* Ça...

*Mr.3:* C'est du marchandage permanent.

*Mme.1:* Oui. Pas de gain à table. Et après, le- dans ce que vous disiez avec [euh] votre papa, est-ce qu'il y avait pas aussi le fait que ce soit son-- le fait que c'est lui qui produit- qui travaille les produits, qui voit tout l'effort qu'on disait que c'était fatigant. Et que c'était un moment considérable d'effort qu'il mettait dans la production de voir du coup un produit qu'il a lui, transpiré pour le produire, finir à la poubelle.

*Mr.3:* Non, je pense que c'était en relation avec les privations de la--

*Mme.1:* Les privations de la guerre.

*Mr.3:* De la nourriture [euh]. De- t'avais un truc dans ton assiette [ben] écoute [euh]--

*Mme.1:* C'est une chance.

*Mr.3:* Tu passes à ton voisin si t'en veux pas--

*Mme.1:* Oui.

*Mr.3:* Ah okay. Parce que le voisin, lui, est en train de tirer la langue et de crever la dalle. Donc [euh] voilà, c'était vraiment lié à ça. Et c'était plus ma mère que- que mon père qui- enfin, la règle elle était établie [hein]. Mes deux frangins étaient plus âgés que moi, donc [euh], quand je suis arrivé, la règle, elle était déjà là [hein]--

*Mme.1:* Oui.

*Mr.3:* [Euh] et c'était pire avant.

*Mme.1:* Ouais.

*Mr.3:* Donc [euh] [euh] non. Je suis même pas sûr que la règle ait vraiment été dic- édictée clairement.

*Mme.2:* Là c'était plus aussi une question de caractère de tes parents.

*Mme.2:* Oui.

*Mme.1:* Enfin [euh], mon-mon père a vécu la même chose pendant la guerre et moi, j'ai jamais été élevée, entre guillemets, avec l'idée qu'il fallait terminer son assiette obligatoirement. On avait le droit de se tromper, on avait droit de goûter, de dire qu'on n'aimait pas, donc [euh]--

*Mr.3:* Oui mais, nous aussi. C'était juste que si on avait décidé que oui.

*Mme.1:* Oui.

*Mr.3:* Qu'on avait accepté une certaine quantité [ben] si finalement on n'y touchait pas [euh]-- ne pas finir, c'est une chose, mais si on n'y touchait pas, eh [ben] là, oui. Il y avait comme une sorte de- d'impératif. T'avais demandé, t'avais accepté [euh].

*Mr.3:* Mais on bouffait pas, mais la fois d'après, [euh] tu savais que... quoi faire.

*Mme.1:* Ouais. Oui, voilà. Il y avait quand même une-- on vous réprimandait si jamais vous aviez [euh] choisi de consommer ce que finalement, vous consommez pas. Après c'est des éducations finalement, qui sont un peu- elles sont différentes.

*Mr.3:* Mais- mais pour moi, c'était vraiment en relation avec l'importance qu'avait le- le fait de se procurer de la nourriture, et le-le bonheur de l'avoir dans son assiette [euh].

*Mme.1:* Oui. Qu'elle aurait pu servir à quelqu'un qui [euh], potentiellement ne mangeait pas à sa faim.

*Mr.3:* Oui et puis qu'il y avait eu trop d'années sombres précédemment.

*Mme.1:* Oui.

*Mr.3:* Où vraiment c'était difficile de se procurer des choses pour oublier qu'il y avait des razzias [quoi], de-de la part de- des Allemands qui venaient dans les fermes et qui disaient bon moi je- voilà je veux des choux des machins, des bidules, etc.

*Mme.1:* Oui.

*Mr.3:* Ils se servaient [quoi].

*Mme.2:* Il y a-- peut- être quelque chose d'anecdotique aussi mais qui-- Nos pères ont été prisonniers de guerre tous les deux-- Le tien a été libéré avant le mien, mais mon père n'a jamais ressenti la faim, c'est une sensation qu'il ne connaît pas, qu'il ne connaissait pas.

*Mme.1:* D'accord.

*Mme.2:* Il n'a jamais eu faim. Je dis pas-- il dit pas qu'il mangeait [euh] qu'il mangeait normalement, mais il ressentait pas la faim. Donc il a jamais ressenti cette- cette sensation. Et peut-être que du coup, c'était moins important aussi pour lui après de...

*Mme.1:* Il n'a jamais souffert physiquement de--

*Mr.3:* De la faim.

*Mme.1:* De la faim.

*Mme.2:* Non. Même--

*Mme.1:* Même s'il mangeait pas.

*Mr.3:* Il se rend pas compte que- quand il a faim.

*Mme.2:* Même plus tard-même plus tard, il mangeait par- parce qu'il savait que c'était l'heure et savait à peu près quoi manger pour et- et il s'arrêtait. Mais il ressentait ni la faim ni la satiété.

*Mme.1:* D'accord il aurait pu après faire des semaines sans manger [euh] son corps lui aurait pas [euh]--

*Mme.2:* Oh peut-être au bout de quelques semaines sans doute, donc [euh]--

*Mme.1:* Oui. Mais au bout de deux jours sans manger, son corps va pas lui dire hop hop hop.

*Mme.2:* Non.

*Mr.3:* Ce que, je pense pas- par- par rapport à ça, donc mon père était prisonnier, lui il est revenu, il était maigre comme un clou. Il faisait un- un mètre 85 un truc comme ça. Et il devait faire dans les 45 kilos quand il est rentré quoi.

*Mme.1:* D'accord.

*Mr.3:* [Euh] par contre, c'est ma mère-- enfin, les femmes quoi qui elles étaient restées sur place et- et étaient avec les autres femmes de la famille, les- les mères, leurs mères, etc. qui [euh] entretenaient tant bien que mal le- la famille et puis le-les animaux et tout ça. Elle se rendait compte aussi de la-- de la pénurie [quoi]--

*Mme.1:* Ouais.

*Mr.3:* Qui leur a été imposée chez eux. Comme elles étaient à la campagne, elles produisaient et elles arrivaient à produire tant bien que mal parce que les hommes étaient partis en guerre, mais produisaient tant bien que mal et arrivaient à se nourrir. Mais il y avait le prélèvement de- de l'armée allemande qui pouvait tomber à tout moment [quoi]. Ils avaient besoin de choux, ils prenaient les choux, ils avaient besoin de pommes de terre, ils venaient chercher des pommes de terre, etc. Ils se servaient, ils étaient chez eux. On pouvait rien dire. Donc il y avait un risque potentiel permanent.

*Mme.1:* C'est le fait de manquer de... d'être en danger.

*Mr.3:* Ouais, plus de mauvaises récoltes, plus le froid, le gel, etc. les maladies, etc.

*Mme.1:* Ouais.

*Mme.1:* Ce qui est de loin la crainte qui pesait sur le fait de ne pas pouvoir manger [euh].

*Mr.3:* Voilà la différence de comportements. Par-- ta -ta maman elle était en ville ?

*Mme.2:* Elle était plus jeune aussi.

*Mr.3:* Oui plus jeune et puis aussi [euh]--

*Mme.2:* Elle était avec ses parents encore.

*Mme.1:* Mm.

*Mme.2:* Surtout que mes parents n'étaient pas mariés.

*Mme.1:* Oui, ses parents qui s'inquiétaient plus qu'elle en ce moment-là.

*Mme.2:* Oui. Ma- ma- ma mère se souvient avoir eu faim, elle a eu faim.

*Mme.1:* Donc il y avait quand même une pénurie qui était aussi [euh]--

*Mme.2:* Après la guerre, le rêve, c'était d'aller manger s'ils habitaient de Landerneau, d'aller manger chacun six meringues à la crème fouettée [inaudible 00:54:10].

*Mme.1:* Rêve ultime.

*Mme.2:* Ouais.

*Mme.1:* OK. Alors [donc] petites activités là terminées. Alors est-ce que vous mangez des bananes ou pas ?

*Mme.2:* Non.

*Mr.3:* Peu.

*Mme.1:* Peu ? On ne peut pas faire le prochain.

*Mme.2:* On en mange quand on n'en n'achète pas avec les petits enfants, puis qu'il en reste.

*Mme.2:* Ouais.

*Mme.1:* Mais c'est pas vous qui avez les--

*Mme.2:* Non.

*Mme.1:* Les consommer.

*Mme.2:* Ça vous aide pas ?

*Mme.1:* On a un petit jeu de bananes, mais c'est pas grave. Vous êtes pas les seuls à pas en consommer.

*Mr.3:* Exemple on achète des bananes parce qu'il y a des petits enfants. Il y en a qui veut : « oui, oui, oui. ». Et puis il en grignote un coin, c'est éventuellement deux coin.

*Mme.2:* Oh non. Tu exagères là, on donne des- on donne des demi bananes.

*Mr.3:* Maintenant, oui, on a compris. Mais bon, oui, le reste des bananes va être mangé par un adulte.

*Mme.1:* Ouais.

*Mr.3:* Parce que sinon elle termine à la poubelle la banane.

*Mme.2:* Par goût non.

*Mme.1:* Mais [en fait], spontanément, on ne mange pas de bananes.

*Mme.1:* Vous, vous allez pas consommer une banane. Eh [ben] c'est pas grave. Faisons l'autre activité.

*Mr.3:* Sur des pommes.

*Mme.1:* On a que la banane. Alors là, il faut que je trie rapidement. Il va y avoir des fruits et légumes qui ont été jetés par des participants du projet et différentes choses qu'on peut faire avec les fruits et légumes. Je vais vous demander de garder à chaque fois [en fait], ce qui vous parle. Ce qu'on peut faire, c'est plutôt nourrir ses poules. Si vous avez pas de poules, ça on met de côté. On prend pas la carte. Si vous avez l'habitude de faire des salades de fruits ou consommer cru, les fruits on garde, de faire des gâteaux aux fruits on garde, des compotes, on garde.

*Mme.1:* Vous allez devoir enlevez en gros ce qui [euh] ce qui ne vous parle pas. Et après je vais vous demander de- de ranger les-les fruits et légumes. Là c'est une personne qui composte, par exemple, là qui congèle, qui font des poêlées.

*Mme.2:* Alors, avec des fruits qu'on ne consomme pas, ou les fruits gâtés ? J'ai pas compris ?

*Mme.1:* Des-- avec des--au fait tout ce qui-qui [euh] qui vous parle. Des préparations qui-qui vous pouvez faire vous, [en fait].

*Mme.2:* Ah, oui. D'accord. Pas- pas en terme de-de récupération de fruits [euh]...

*Mme.1:* Voilà, on va voir si vous vous récupérez ou justement ou si non vous préférez aussi jeter en gros. Ou si vous, vous allez en faire quelque chose. C'est ça. Est-ce que ça vous arrive de faire des salades de fruits ?

*Mme.2:* Oui rarement, mais ça arrive [euh] oui.

*Mr.3:* Ça peut arriver mais c'est rare oui.

*Mme.1:* Des [euh] gâteaux à base de fruits ?

*Mme.2:* Oui.

*Mr.3:* Mm-hmm.

*Mme.1:* Des soupes de légumes ?

*Mme.2:* Oui.

*Mme.1:* Ça, c'est des jus ou des smoothies ?

*Mme.2:* Non.

*Mme.1:* Non. Donc on enlève. Des compotes?

*Mme.2:* Rarement, mais ça peut arriver.

*Mme.2:* Est-ce que ça vous arrive de congeler des--

*Mme.2:* Oui.

*Mme.2:* Des légumes des fois ?

*Mr.3:* Oui.

*Mme.1:* De faire des poêlées ?

*Mr.3:* Oui.

*Mme.2:* Ça c'est une soupe froide, type gaspacho.

*Mme.2:* On en a fait, plus tellement en ce moment, mais ça peut arriver.

*Mme.1:* Ça peut arriver. Et ça, c'est des salades de crudités, [enfin] manger des crudités.

*Mme.2:* Oui.

*Mme.1:* Okay. Alors ça, c'est plutôt lié aux poubelles. Donc vous, est-ce que vous avez des poules ?

*Mme.1:* Non.

*Mr.3:* [Ben] non, pas de poule. Ça, oui par contre...

*Mme.1:* Est-ce que vous compostez ?

*Mr.3:* Oui, on composte.

*Mme.1:* Donc le compost et sinon, c'est une poubelle classique ?

*Mme.2:* Non, non, puisqu'on compost.

*Mr.3:* Non, non on composte.

*Mme.1:* Tous vos--les fruits et légumes on composte. Donc voilà. Et là, il y a--

*Mr.3:* La seule chose qu'on ne composte pas, c'est la partie [euh] feuillage des- des ananas.

*Mme.1:* Ouais, ça se-- ça se décompose pas ?

*Mme.2:* Enfin le- enfin le--

*Mme.1:* La grande tige verte-là ?

*Mme.2:* Oui.

*Mr.3:* Oui. On appelle des feuilles, mais ce sont pas des feuilles.

*Mme.2:* Oui.

*Mr.3:* L'ananas est toujours associé à quelques--

*Mme.2:* À son plumeau là.

*Mme.1:* Oui, oui.

*Mme.2:* Ça marche pas ça.

*Mme.1:* Ça se décompose pas ?

*Mme.2:* Non.

*Mr.3:* Ça met un temps...

*Mme.1:* Ah, oui. C'est curieux.

*Mr.3:* Faudrait le découper en morceaux et c'est tellement compliqué.

*Mme.1:* Oui, le broyer. Alors pour-- je vais vous demander de prendre à chaque fois le fruit- le fruit ou légume, de me le décrire un peu, et me dire du coup ce que vous vous en aurez fait.

*Mr.3:* On est bien déshydratées.

*Mme.1:* Alors donc ça se sont les carottes.

*Mr.3:* Les carottes trop attendues.

*Mme.2:* Elles sont trop attendues. Maintenant, je tenterais quand même de- de couper des bouts et d'éplucher pour voir si ça peut se mêler à une soupe ou une poêlée.

*Mme.1:* Ouais, plutôt cuites du coup ?

*Mme.2:* Ah, cuites du coup, oui, oui.

*Mr.3:* Oui. Ah, oui clairement.

*Mme.2:* Pas- pas pour manger cru là.

*Mme.1:* Qu'est-ce qui fait justement que vous préfériez la cuire que la manger crue ?

*Mme.2:* Bien parce qu'elle semble flétrie.

*Mme.1:* Ouais.

*Mme.2:* Donc [euh] je m'imagine que le goût ne va pas être bon cru.

*Mme.1:* D'accord.

*Mr.3:* S'il y a quelques germes ben, la cuisson--

*Mme.2:* Ouais.

*Mr.3:* Va régler le problème. Tandis que cru, c'est pas très appétissant quand même.

*Mme.2:* Ouais.

*Mr.3:* [Enfin] c'est- c'est déjà pas engageant comme ça.

*Mme.1:* Ouais.

*Mr.3:* On va pouvoir l'oublier si-- comme étant transformée.

*Mme.1:* D'accord. Donc plutôt en soupe ou poêlée ?

*Mme.2:* Oui.

*Mr.3:* Mm-hmm.

*Mme.1:* Donc là on peut faire ça, hop on la met dessus. Pour chaque, que vous allez pouvoir faire la--

*Mr.3:* Ça c'est un poivron bien flétri, [hein].

*Mme.2:* Ah là c'est pareil. Je tente de récupérer [euh] [enfin]--

*Mr.3:* Faut couper les morceaux qui... Quand même il est bien avancé [hein].

*Mme.2:* Ouais c'est même un peu sûr que j'essaie-- je tente quelque chose avec ça.

*Mme.1:* Ouais.

*Mme.2:* On va le couper en morceaux, puis on le met au compost.

*Mme.1:* Ouais.

*Mr.3:* C'est la salade qui a trop attendu. Ben moi je--

*Mme.2:* Oh compost. Ah bon ?

*Mr.3:* Ouais, mais moi je [euh] compost mais ça dépend du reste de la feuille. On voit pas le reste de la feuille là.

*Mme.1:* C'est cette feuille-là en gros.

*Mr.3:* Oui.

*Mme.1:* Cette feuille-là qu'est-ce que vous-- ce bout-là vous faites quoi ?

*Mr.3:* [Euh] là ?

*Mme.1:* Oui, vous l'enlevez ?

*Mr.3:* Si, c'est juste ce bout-là je l'enlève.

*Mme.1:* Mm.

*Mr.3:* Mais si est-- [euh] j'ai des grandes feuilles qui par ailleurs sont encore satisfaisantes, j'enlève la- la partie rosée.

*Mme.2:* Ah mais oui, bien sûr si le reste est bon, on- on se contente de retirer la partie rosée.

*Mme.1:* Cette partie-là vous la jetez ?

*Mme.2:* Oui.

*Mr.3:* Du coup--

*Mme.1:* Celle-là, vous voulez la jetez.

*Mr.3:* Okay.

*Mme.2:* C'est que là ce n'est pas bien cette tomate.

*Mr.3:* Ça doit être une té- une tomate qui a été choisie-- une variété qui a été choisie pour être bien--belle et bien ronde et.

*Mme.2:* On va- on va la cuire celle-là. On va la mettre en tarte.

*Mr.3:* Ou en potage.

*Mme.2:* Ou soupe, ou cuite.

*Mme.1:* OK mais pas crue.

*Mr.3:* La cuisson au four c'est-- ça améliore les- les mauvaises tomates.

*Mr.3:* Ou à la poêle oui.

*Mme.1:* Oui, on peut du coup mettre ça. Ça veut dire vous la mettez à cuire.

*Mme.2:* Un radis.

*Mme.1:* C'est un radis.

*Mme.2:* Bon là si on coupe le petit bout, il doit être bon non ?

*Mr.3:* Oui. Une moisissure qui est en train de s'installer, mais en coupant.

*Mme.2:* Où ça ?

*Mr.3:* Là- dans- sur- la- [enfin] moi je- je vois une moisissure.

*Mme.2:* Oui, [ben] si on coupe-- si on coupe plus on peut tenter de le manger.

*Mr.3:* Oui, oui. Si le-le reste est ferme [euh].

*Mme.1:* Ouais.

*Mme.2:* Oui, si le reste- est ferme.

*Mr.3:* Après si c'est- si en plus il est vide [euh].

*Mme.2:* Oui, il faut pas qu'il soit creux. Mais si en le-- si en le coupant, il est bi-bien-bien blanc, alors là on peut le manger.

*Mr.3:* Ça on ne fait jamais de potage de radis.

*Mme.2:* Non mais là, on ne va pas le mettre dans une salade non plus. On va le manger tel quel.

*Mme.1:* On peut le mettre là juste pour dire vous le mangez cru en croquant.

*Mr.3:* Poubelle.

*Mme.2:* Un citron.

*Mr.3:* C'est un citron.

*Mme.1:* Un citron qui est bien...

*Mr.3:* Là, le reste du citron est mauvais, là c'est- c'est carrément malade.

*Mme.2:* Ici c'est une tomate cerise en plus.

*Mme.1:* Oui.

*Mr.3:* Bien tomates cerises.

*Mme.1:* Ça c'est compost aussi.

*Mr.3:* Là, c'est pareil [hein]. Pour moi, c'est--- une poire--

*Mme.2:* C'est de la poire comme ça ? Ah non c'est pas bon ça.

*Mr.3:* Oui c'est une poire qui est pourrie à un endroit est contaminée intégralement.

*Mme.1:* D'accord.

*Mr.3:* [Euh] c'est quoi, c'est une patate douce ?

*Mme.1:* C'est une carotte aussi.

*Mr.3:* Ah !

*Mme.2:* [Euh] en moins mauvaise-- en plus mauvaise forme.

*Mr.3:* Oui là elle est vraiment dépassée je trouve.

*Mme.2:* Donc je pense--

*Mr.3:* Là je- je ferais la même chose. Non mais bon...

*Mme.1:* Ouais c'est du ver de poireaux ?

*Mme.2:* Et peut-être celui-là. Si on est en train de faire une soupe, on peut peut-être--

*Mr.3:* Oui, je suis d'accord.

*Mme.2:* Le mettre dedans, mais les deux premiers non.

*Mme.1:* Ouais.

*Mme.2:* Donc--

*Mr.3:* C'est à trier. Faut trier fortement. Les endives, celle-ci elle est- celle-ci on peut espérer enlever quelques feuilles, peut-être pour voir en dessous.

*Mme.2:* Non, t'as- t'as- t'as vu par transparence, là.

*Mr.3:* Oui.

*Mme.2:* Non tout est mauvais là-dessous, désolée.

*Mme.1:* Ça part au compost.

*Mr.3:* [Euh]...

*Mme.2:* Ça c'est de la courgette.

*Mr.3:* C'est un morceau de courgettes.

*Mme.1:* C'est un morceau de courgette oui.

*Mme.2:* Il faut voir comment c'est.

*Mr.3:* Si on coupe, là, est-ce que le reste est sain ? Question.

*Mme.2:* Alors si le reste est sain [euh], soupe ou poêlée.

*Mr.3:* Il manque les poules, mais je-- depuis quelques temps on a des- les cochons d'Inde qui viennent nous rendre visite.

*Mme.2:* Pas nous, ce sont ceux de la voisine.

*Mr.3:* Il les laisse sortir, viennent chez nous. Ça c'est typiquement le genre de truc que je leur mettrais. Quand même, on peut peut-être ressortir ça.

*Mme.1:* On peut mettre la poule.

*Mr.3:* Alors c'est vrai qu'il y a des choses qu'on peut remettre.

*Mme.2:* Quand tu les nourris trop aussi.

*Mr.3:* Oui oui. C'est un fait. Mais bon, de temps en temps, un petit peu [quoi] et la poule elle va choisir là-dedans, pour trouver ce qui lui convient.

*Mme.1:* C'est ça.

*Mme.1:* C'est- je suppose que c'est une orange non ?

*Mme.2:* Une orange moisie.

*Mr.3:* Bof.

*Mme.2:* Non.

*Mr.3:* Puis elle est bien avancée aussi.

*Mme.2:* Ah ouais, si je--

*Mr.3:* Pour les poules.

*Mme.2:* Je récupère le petit bout que je mange comme ça, puis le reste on jette.

*Mr.3:* Ouais, tu t'aventures. Et puis si c'est crado [euh]...

*Mme.2:* Non de toute autre façon ça je mangerais pas moi.

*Mr.3:* Mais entre les deux.

*Mme.1:* Ce qui est noir là vous--

*Mr.3:* Non. Je le mange pas.

*Mr.3:* Non, parce que là il est plus que noir quand même.

*Mme.2:* Il y en a qui aiment.

*Mme.2:* [Euh] Gérard il mange comme ça, il préfère comme ça.

*Mr.3:* [Euh], j'en suis pas sûr. Mais moi je jette.

*Mme.1:* Oui, pour vous, c'est trop--

*Mr.3:* Déjà que les bananes...

*Mme.1:* Oui, c'est pas votre-- Alors je vais prendre en photo votre rangement.

*Mme.1:* Vous avez sauvé quand même quelques-- Hop.

*Mr.3:* Vous avez tout là ?

*Mme.1:* Merci. Ouais. Super. Alors pour finir juste sur la poubelle connectée. Comment vous l'avez ressenti cette période ? Est-ce que vous avez le sentiment d'avoir plus jeté, moins jeté ? Comment--

*Mme.2:* Par rapport à avant l'été ?

*Mme.1:* Ouais, comment- comment s'est passée cette période ?

*Mr.3:* On a plus jeté, peut-être à cause des retours de table.

*Mme.2:* Oui parce que là vous l'aviez récupéré en juillet c'est ça ?

*Mme.1:* Ouais.

*Mme.2:* Et donc on n'avait pas eu encore les... On est-- En avril on l'avait ?

*Mme.1:* On vous l'a installé, non plutôt, en mai je crois.

*Mme.2:* Ça donc en fait de mai à juillet, on n'a pas eu les petits enfants.

*Mme.1:* Ouais.

*Mme.2:* Donc du coup, je pense qu'on a assez peu jeté.

*Mme.2:* D'accord.

*Mme.2:* Me semble-t-il. Et peut-être plus là, bon déjà avec les petits enfants d'une part au niveau de la variété des fruits et légumes [silence 01:04:59-01:05:05] peut-être plus me semble t-il.

*Mr.3:* Oui j'ai l'impression.

*Mme.1:* Oui, vous avez l'impression d'avoir plus jeté ?

*Mme.2:* Quelques pommes de terre. C'est arrivé.

*Mr.3:* Oui, il y a quelques mauvaises surprises sur certains lots de pommes de terre. On prend toujours la même variété, qu'on aime bien le goût. Et puis la chair, assez ferme, mais pas trop non plus.

*Mme.1:* Oui.

*Mr.3:* Qu'on porte bien à la cuisson. Et de temps en temps [euh]--

*Mme.2:* Alors [euh]...

*Mr.3:* C'est aléatoire [quoi] ça, c'est pas-- On n'a pas tellement jeté quand même malgré tout.

*Mme.2:* Je- je sais pas, parce qu'entre-- je- je sais pas, parce qu'entre-- c'est peut-être arrivé qu'on jette un melon. Je me souviens plus.

*Mme.1:* Ouais.

*Mme.2:* Oui, entre mai juillet et auquel cas ça fait du poids, alors que...

*Mme.1:* Oui.

*Mme.2:* Là, on a jeté une aubergine, ça c'est sûr, je m'en souviens, mais ça pèse moins lourd qu'un melon.

*Mme.1:* Ouais, oui, c'est vrai que dans le problème des poids aussi, des fois c'est...

*Mr.3:* De toute manière, vous allez avoir [euh] les-- le verdict.

*Mme.2:* Oui mais c'est du coup le total, vous savez parce qu'on...

*Mme.1:* Mais on n'a pas oui le détail de est-ce que c'est plein de petites choses ou est-ce que c'est une grosse chose, c'est ça qu'on n'a pas...

*Mme.2:* Sauf [euh] parce que vous savez à quel moment on a mis-- quand on a ouvert le couvercle et quel poids.

*Mr.3:* Oui, c'est daté.

*Mme.2:* Vous pouvez savoir si c'est une grosse chose.

*Mme.1:* Oui, oui, c'est vrai, on a le-- on a-- on a le détail de la--

*Mr.3:* De la pesée.

*Mme.1:* Oui. On a tous les soirs la pesée générale mais on a à chaque-- dès que quelque chose est mis, on a le poids de ce qui est mis.

*Mme.2:* C'est ça donc vous pouvez savoir si c'est probablement--

*Mme.1:* Un gros-- oui.

*Mme.2:* C'est probable que ce soit un gros-- un gros truc ou pas.

*Mme.1:* C'est vrai.

*Mr.3:* Vous allez pouvoir déterminer la période pendant laquelle il y a eu les petits enfants.

*Mme.1:* Oui, c'est [bah] ouais--

*Mme.2:* C'est fondamental pour vous je pense.

*Mme.1:* C'est vrai, ça- ça change normalement les-- Oui, ça change des pratiques quand on a-- quand il y a des enfants, c'est déjà prouvé dans les-- Justement, dans les études qui ont déjà été réalisées sur le gaspillage alimentaire, plus il y a d'enfants plus il y a de gaspillage. C'est [euh]-- les familles nombreuses forcément subissent un peu plus le gaspillage. Mais a contrario quand on divise par le-- c'est-- la- la poubelle va être plus lourde. Mais si on redivise par le nombre de personnes, il va finalement y avoir moins de gaspillage par personne--

*Mme.1:* [Ah] d'accord.

*Mme.2:* Que- mais ce serait une maison qui gaspille plus.

*Mme.2:* Oui, bien sûr. Le poids par personne.

*Mme.1:* Oui. Mais [voilà], on verra-- on verra ça. Alors là, du coup, la suite, c'est que [euh] nous on a encore des analyses, on a pris un peu de retard là [enfin], les entretiens auraient dû se finir en décembre. Mais [euh] vu que j'ai été covidée, [euh] ça a tout décalé. Il y a eu les vacances donc on a repris qu'en janvier. Je pense que d'ici mars avril, on devrait quand même à voir des choses, j'espère. Parce que nous, on a nos études de notre côté. Après, il y a l'autre labo qui fait leurs études de leur côté. Et l'idée, c'est de-- c'est de croiser les-- de croiser les données. Et donc, il y aura forcément une trace écrite. Il y aura des livrables, on a des- des cahiers des charges à remplir et tout ça. Donc ça, vous aurez-- vous aurez accès aussi à ces résultats-là. Et dans l'idée, on fait-- on va essayer de faire un événement où tous les-- tous les participants pourront venir et on présentera les résultats devant tout le monde. Si le Covid nous laisse tranquille.

*Mme.2:* Oui, c'est ça, oui.

*Mme.1:* Mais on pensait que ça pouvait être marrant si ceux qui sont volontaires [euh] puissent se rencontrer [euh], discuter de tout ça. Donc voilà, ça ce sera la suite. [Euh] une autre suite qu'on est en train de faire. Normalement je récupère les poubelles, mais on propose aux personnes mais que-- là c'est vraiment [euh], déjà que vous étiez déjà volontaires pour l'étude, donc un grand merci. Mais c'est ceux qu'ils veulent encore être volontaires. On se pose la question des différences de poids qui existent entre les fruits et légumes pourris et [euh] les-- le poids des épluchures. Parce que des études qui ont déjà été faites sur le gaspillage alimentaire vont sortir des chiffres. Sauf qu'on se pose la question de [euh] à partir de quoi, comment ils ont comptabilisé le- le poids. Puisqu'on est en train de se dire que, les déchets relatifs aux épluchures--

*Mme.2:* On n'a pas mis les épluchures.

*Mme.1:* [Ben] non exactement là [en fait], on propose aux personnes de garder la poubelle un mois supplémentaire.

*Mme.2:* D'accord.

*Mme.1:* Et cette fois-ci de la transformer en poubelle à compost en gros.

*Mme.2:* D'accord.

*Mr.3:* D'accord.

*Mme.1:* Ce qui nous permettrait de montrer que [ben] selon ce qu'on pèse, le résultat qu'on va annoncer dans nos recherches [euh] n'est pas le même entre les déchets--

*Mr.3:* Absolument.

*Mme.1:* Liés aux fruits et légumes des épluchures et le-- vraiment le fruit légume qu'on voulait manger là qu'on a perdu--

*Mme.1:* Eh [ben] qui a quand même un gros niveau. Donc ce qui veut dire que toutes les recherches ont été faites [bah] il faut bien regarder ce qui [euh]-- ce qui est pesé.

*Mme.2:* Pourquoi pas mais alors [euh] nous on je-- on va souvent un composteur [hein].

*Mr.3:* Oui !

*Mme.1:* C'est sûr. Donc là, c'est-- un mois, ça suffit largement. Je pense que fin février, je reviens--

*Mme.2:* Non je veux dire que même la poubelle là, parce que--

*Mme.1:* Oui, ah [ben] c'est clair que--

*Mr.3:* Il faut faire gaffe, parce qu'on y met aussi du- du papier, du carton, les- les coq-- les coquilles d'œufs--

*Mme.1:* Alors là par contre c'est ça. Il faudrait que-- il faudrait quand même que vous gardiez votre- votre petit compost habituel.

*Mme.2:* On a fait l'intermédiaire, mais...

*Mr.3:* Ça peut être deux, trois ou quatre fois par jour.

*Mme.1:* Ah oui, celui-là ?

*Mme.1:* Oui, en gros la poubelle--

*Mme.2:* Au moins deux fois par jour [quoi].

*Mme.2:* Ouais. [Ben] là ce serait le compost [euh] ce serait la-- [enfin] de--

*Mme.2:* Oui, [enfin] par exemple ça [quoi].

*Mme.1:* Ça exactement, ça, ça y va.

*Mr.3:* Oui.

*Mme.1:* Et par contre, pas le marc de café ou les coquilles d'œufs [euh]--

*Mr.3:* Oui bien sûr, ouais, ouais. Il faut l'avoir dans la tête.

*Mme.1:* J'ai une étiquette si vous voulez bien.

*Mme.2:* Mais dans ce cas [euh], on a besoin de sacs. Parce que là, il y a déjà--

*Mme.1:* Ouais, je peux vous laisser le--

*Mr.3:* On est en panne de sacs.

*Mme.1:* Et puis si vous en avez pas vous me dites, sur le reste je vous envoie--

*Mr.3:* Parce que là on va- on va faire du volume là. Je sais pas [enfin] quand on- on prépare des- je sais pas des-- quand on épluche des pommes [euh].

*Mme.1:* Ah, c'est ça.

*Mr.3:* Il y en a- il y en a énormément[quoi].

*Mme.1:* C'est pour ça qu'on--

*Mr.3:* Le trognon est le- la- la pelure [euh].

*Mme.1:* C'est ça qu'on a entrain de se dire.

*Mr.3:* Courges [euh]...

*Mme.1:* Et le poids est énorme en plus.

*Mr.3:* Le poireau il y a quand même [euh] a une partie de--
