# Supplementary material for: Participatory and multi-disciplinary science dataset and surveys for the assessment of the microbiological and behavioural factors influencing fresh fruits and vegetables' waste at home
Source: Data Brief. 2026 Jan 7;65:112434. doi: 10.1016/j.dib.2025.112434 (PMC12856149; doi:10.1016/j.dib.2025.112434)
Supplement: Supplementary file 1 [file mmc1.zip › Part1_FFV_waste_sampling_campaigns/Table4_Household_Survey/Table4a_Household_survey_questions.docx]

- Domestic hygiene practices
  - Concerning FFV storage at refrigerated temperature (fridge)

Ref 1. Do you use the refrigerator to store some of your FFV?

-Yes ◻

-No ◻

Ref 2. Do you store FFV in the crisper drawer?

-Yes ◻

-No ◻

Ref 3. Do you check the temperature of your refrigerator?

-Yes ◻

-No ◻

Ref 3bis. **if so**, what do you use?

-Thermometer built into the refrigerator ◻

-Additional thermometer ◻

-other (specify) ◻

Ref 4. How old is your refrigerator?

-Less than 2 years olds ◻

-Between 2 and 5 years olds ◻

-Between 6 and 10 years old ◻

-Between 11 and 14 years old ◻

-Over 15 years old ◻

-I don't know ◻

Ref . Are the walls of your refrigerator covered with an antimicrobial coating?

-Yes ◻

-No ◻

-I don't know ◻

Ref 5. Have you ever noticed condensation in your refrigerator?

-Yes ◻

-No ◻

-I don't know ◻

Ref 5bis. If so, where does this water condensation appear?

-On shelves and/or walls only ◻

-In the crisper drawer only ◻

-In both ◻

-I don't know ◻

Ref 5bis. How often do you observe condensation or liquid in the refrigerated storage area for your fruit and vegetables?

-Never ◻

-Sometimes (less than once a month) ◻

-Often (more than once a month) ◻

-Always ◻

Ref 7. Are there any food stains or marks in the refrigerated storage area for your FFV?

-Never ◻

-Sometimes (less than once a month) ◻

-Often (more than once a month) ◻

-Always ◻

Ref 8. Do you notice any visible mold in your refrigerator?

-Never ◻

-Sometimes (less than once a month) ◻

-Often (more than once a month) ◻

-Always ◻

Ref 9. When FFV are packaged and/or bagged, do you remove the packaging and/or bag (cardboard and/or plastic...) before placing them in your refrigerator?

-Never ◻

-Seldom ◻

-Often ◻

-Always ◻

Ref 10. Do you wrap partially eaten fruit and vegetables before putting them back in the fridge?

-Never ◻

-Seldom ◻

-Often ◻

-Always ◻

Ref 11bis. How often would you say you clean the crisper drawer (or the part where you store your FFV) in your refrigerator?

-Often (more than once a month) ◻

-Monthly (once a month) ◻

-Quarterly (every three months) ◻

-Bi-annually (once or twice a year) ◻

-Annually (once a year) ◻

-Less than once a year ◻

Ref 12. Regarding the frequency of this cleaning, it is :

- You clean at a more or less fixed frequency even if there is no visible filth ◻

- You clean at a more or less fixed frequency even if there is no visible filth, AND also when you notice filth ◻

- You only clean when you notice filth ◻

- You clean when you have time, irregularly and regardless of how filthy it is ◻

- Other (specify) ◻

refri-14. Ref 13. What type of product do you prefer to use to clean the crisper drawer in your refrigerator?

-Dish soap ◻

-Multi-purpose cleaner ◻

-Bleach ◻

-Wet wipes ◻

-White vinegar ◻

-Lemon juice ◻

-Baking soda ◻

-Water only ◻

-Other (specify) ◻

Ref 14. Do you rinse after applying the cleaning product?

-Yes ◻

-No ◻

Ref 15. What type of tool do you prefer to use to clean the crisper drawer in your refrigerator?

-Abrasive sponge (e.g.: green scrubbing side of the sponge) ◻

-Soft sponge ◻

-Washable wipe ◻

-Brush ◻

-Disposables ◻

-Other (specify) ◻

Ref 16. After cleaning, do you dry the cleaned area?

-Yes ◻

-No ◻

Ref 16bis. If so, what do you use?

-Open-air drying ◻

-Reusable (Cloth/Dishcloth) ◻

-Disposable (Disposable paper/Kitchen roll) ◻

-Other (specify) ◻

- - Concerning FFV storage at room temperature (kitchen/living room) or moderate temperature (pantry/cellar/shed/garage).

Unref 1. When FFV are packaged and/or bagged, do you remove the packaging and/or bag (cardboard and/or plastic...) before placing them in your unrefrigerated storage compartment (basket, hamper, crate...)?

-Never ◻

-Seldom ◻

-Often ◻

-Always ◻

Unref 2. What container do you mainly use to store your fruit and vegetables at room temperature or moderate temperatures? Only one answer possible.

-Fruit Basket ◻

-Hamper ◻

-Crate ◻

-Other (specify) ◻

Unref 3. How often would you say you clean this unrefrigerated FFV compartment?

-Often (more than once a month) ◻

-Monthly (once a month) ◻

-Quarterly (every three months) ◻

-Bi-annually (once or twice a year) ◻

-Annually (once a year) ◻

-Less than once a year ◻

Unref 4. Regarding the frequency of this cleaning, it is :

- You clean at a more or less fixed frequency even if there is no visible filth ◻

- You clean at a more or less fixed frequency even if there is no visible filth, AND also when you notice filth ◻

- You only clean when you notice filth ◻

- You clean when you have time, irregularly and regardless of how filthy it is ◻

- Other (specify) ◻

- Other (specify) ◻

Unref 5. What type of product do you prefer to use to clean the unrefrigerated FFV storage compartment?

-Dish soap ◻

-Multi-purpose cleaner ◻

-Bleach ◻

-Wet wipes ◻

-White vinegar ◻

-Lemon juice ◻

-Baking soda ◻

-Water only ◻

-Other (specify) ◻

Unref 6. Do you rinse after applying the cleaning product?

-Yes ◻

-No ◻

Unref 7. What type of tool do you prefer to use to clean the unrefrigerated FFV storage compartment?

-Abrasive sponge (e.g.: green scrubbing side of the sponge) ◻

-Soft sponge ◻

-Washable wipe ◻

-Brush ◻

-Disposables ◻

-Other (specify) ◻

Unref 8. After cleaning, do you dry the cleaned area?

-Yes ◻

-No ◻

Unref 8bis.If so, what do you use?

-Open-air drying ◻

-Reusable (Cloth/Dishcloth) ◻

-Disposable (Disposable paper/Kitchen roll) ◻

-Other (specify) ◻

- Origin of fruit and vegetables

Org 1. What percentage of your FFV do you estimate to come from :

- "Organic farming" or labelled “pesticide residue-free” %

-Conventional farming (using pesticides) %

- Place of purchase

Cons 1. What percentage of your FFV do you estimate to come from :

-large retailers (supermarkets, hypermarkets) %

-specialist shops (organic shops, greengrocers) %

-Farmer's Market %

-local association (Vegetables box schemes) %

-from your garden or that of a third party %

Cons 2. What percentage of your FFV do you estimate to be sourced :

- Locally %
- Nationally %
- Internationally %

Cons 3. How often do you buy FFV ?

-Often (more than twice a week) ◻

-Biweekly (twice a week) ◻

-Weekly (once a week) ◻

-Bimonthly (every other week) ◻

-Monthly (once a month) ◻

- Storage practices

Storage. Where do you usually store your whole fresh fruit and vegetables (uncut)? For each fruit or vegetable listed below, please tick the storage location you use most often (do not tick anything if you do not consume the food in question).

|  | Room/kitchen temperature | Cellar/basement/storage room/garage | Refrigerator | Doesn’t consume |
| --- | --- | --- | --- | --- |
| apricot |  |  |  |  |
| garlic,  onion, shallot |  |  |  |  |
| artichoke |  |  |  |  |
| asparagus |  |  |  |  |
| eggplant |  |  |  |  |
| avocado |  |  |  |  |
| banana |  |  |  |  |
| broccoli |  |  |  |  |
| carrot |  |  |  |  |
| cherry |  |  |  |  |
| mushrooms |  |  |  |  |
| cabbage  (red, white, green) |  |  |  |  |
| cauliflower |  |  |  |  |
| lemon |  |  |  |  |
| clementine,  mandarin, orange |  |  |  |  |
| cucumber |  |  |  |  |
| squash,  pumpkin, butternut squash... |  |  |  |  |
| courgette |  |  |  |  |
| spinach |  |  |  |  |
| strawberry |  |  |  |  |
| raspberry,  blueberry, redcurrant, ... |  |  |  |  |
| nuts  (walnuts, hazelnuts, almonds, etc.) |  |  |  |  |
| green bean |  |  |  |  |
| fresh aromatic herbs  Unpackaged (parsley, mint, basil, etc.) |  |  |  |  |
| kiwi |  |  |  |  |
| mango |  |  |  |  |
| melon  and watermelon |  |  |  |  |
| peach,  nectarine |  |  |  |  |
| peas, broad beans, white beans, red beans, etc. |  |  |  |  |
| pear |  |  |  |  |
| leek |  |  |  |  |
| pepper,  chilli pepper |  |  |  |  |
| apple |  |  |  |  |
| potato |  |  |  |  |
| plum |  |  |  |  |
| radish |  |  |  |  |
| table grape |  |  |  |  |
| salad,  chicory, ... |  |  |  |  |
| tomato |  |  |  |  |
